# Effects of timber harvesting on the genetic potential for carbon and nitrogen cycling in five North American forest ecozones

**DOI:** 10.1038/s41598-018-21197-0

**Published:** 2018-02-16

**Authors:** Erick Cardenas, Luis H. Orellana, Konstantinos T. Konstantinidis, William W. Mohn

**Affiliations:** 1 Department of Microbiology and Immunology, Life Sciences Institute, University of British Columbia, Vancouver, BC V6T 1Z3 Canada; 2 Georgia Institute of Technology, School of Civil and Environmental Engineering, Atlanta, GA 30332 USA

## Abstract

Forest ecosystems are critical to global biogeochemical cycles but under pressure from harvesting and climate change. We investigated the effects of organic matter (OM) removal during forest harvesting on the genetic potential of soil communities for biomass decomposition and nitrogen cycling in five ecozones across North America. We analyzed 107 samples, representing four treatments with varied levels of OM removal, at Long-Term Soil Productivity Study sites. Samples were collected more than ten years after harvesting and replanting and were analyzed via shotgun metagenomics. High-quality short reads totaling 1.2 Tbp were compared to the Carbohydrate Active Enzyme (CAZy) database and a custom database of nitrogen cycle genes. Gene profile variation was mostly explained by ecozone and soil layer. Eleven CAZy and nine nitrogen cycle gene families were associated with particular soil layers across all ecozones. Treatment effects on gene profiles were mainly due to harvesting, and only rarely to the extent of OM removal. Harvesting generally decreased the relative abundance of CAZy genes while increasing that of nitrogen cycle genes, although these effects varied among ecozones. Our results suggest that ecozone-specific nutrient availability modulates the sensitivity of the carbon and nitrogen cycles to harvesting with possible consequences for long-term forest sustainability.

## Introduction

Forests are natural resources with important economic, cultural, and ecological values^1^. Many of the ecosystem processes that occur in forests are mediated by soil microbes, key among them is nutrient cycling, in which plant-derived organic matter (OM) is degraded and nutrients are released for consumption by plants and other organisms. Globally, forests are under pressure by changes in land use, global climate, and biomass harvesting^2,3^. Management practices that affect the function of the soil microbial community can potentially influence the growth, survival and economic value of trees in managed forest stands.

Established in the late 1980s, the Long-Term Soil Productivity Study (LTSP) focuses on the long-term effects of OM removal during harvesting on forest productivity^4^. Ten years after harvesting and replanting, varying levels of OM removal had only marginal effects on tree productivity^5–7^. On the other hand, harvesting has substantial short- and long-term effects on soil microbial communities. A meta-analysis of forest perturbation studies found that harvesting reduces microbial biomass by 19% on average^8^, with stronger effects on fungal than on bacterial populations (27% vs. 14% reductions, respectively). Molecular ecology studies of LTSP sites in multiple ecozones showed that harvesting caused long-term changes in the overall soil microbial community structure^9,10^, and in hemicellulolytic populations^11^. We recently showed that harvesting reduced biomass degradation potential for more than a decade after harvesting at one LTSP site in the interior Douglas fir ecozone of British Columbia^12^. However, it remains unclear whether this effect on biomass degradation potential is generalizable across different ecozones. We hypothesized that OM removal during harvesting would consistently decrease the genetic potential for organic carbon decomposition, reducing the abundance and diversity of genes encoding this process.

The carbon and nitrogen cycles are closely linked. In forest soils, organic decomposition is accompanied by mineralization of organic nitrogen, and growth of plant biomass creates high demand for available nitrogen. Nitrogen cycling is critical in temperate coniferous forests where primary productivity is typically N-limited^13^. Previous studies of American and European forests^6,14,15^ have shown that harvesting can increase rates of nitrogen mineralization and nitrification. We hypothesized that OM removal during harvesting would increase the relative importance of these catabolic processes in soil communities, resulting in an increase abundance and diversity of genes involved in nitrogen cycling.

We tested the above hypotheses using the LTSP experimental design on sites in five ecozones across North America. Metagenomics allowed us to identify and quantify the microbial community genes and characterize the diversity and abundance of those related to the functions of interest for this study. Our analyses focused on genes encoding carbohydrate active enzymes (CAZy) as well as nitrogen cycling enzymes. We compared the diversity and relative abundance profiles (genetic potential) of biomass decomposition and nitrogen cycle genes among ecozones, soil layers, and OM removal treatments. We related environmental gradients to the metagenomic responses to OM removal during harvesting. This is the most extensive metagenomic analysis of forest soil communities to date and among the first to use a field experiment replicated among different ecozones.

## Methods

### Study sites

Soil samples were collected from five sites that are part of the Long-term Soil Productivity (LTSP) Study in North America^4,16^. The sites, which varied in weather, vegetation, and soil chemistry, represent five distinct ecozones across North America named for important commercial tree species in each: BS, black spruce; IDF, interior Douglas-fir; JP, jack pine; LP, loblolly pine; and PP, ponderosa pine (Supplementary Table S1). At each site, we sampled harvested treatment plots with three levels of OM removal (and strictly minimized soil compaction): stem-only harvesting (OM1), whole-tree harvesting (OM2) and whole-tree harvesting plus forest floor removal (OM3). We additionally sampled unharvested reference plots (OM0).

### Sampling

Sampling occurred 10 to 17 years after the OM removal treatments and replanting (Supplementary Table S1), as previously described^9,12^. Both organic and mineral soil layers were sampled; however, except for the LP ecozone, the organic layer had not yet re-developed in the OM3 treatments. The same samples were used for chemical analyses and DNA extraction, except for the IDF ecozone where samples for chemical analysis were taken the year prior to sampling for DNA extraction, as previously reported^12^.

### Soil chemistry

Chemical analyses (Supplementary Table S2) were done using standard methods, as previously described^9^. Briefly, total C and N were determined with a combustion elemental analyzer. Water content was determined gravimetrically. pH was measured in a 2:1 slurry (5 mL water : 2.5 g dry soil). For the LP and PP ecozones, measurements of total C and N were only available per plot (not per sample) and only for the mineral layer.

### DNA extraction and metagenome generation

Soil samples were processed for shotgun metagenome sequencing as previously reported^12^. That study of the IDF ecozone generated paired-end 75-bp metagenome reads, which were also used in the present study. For the remaining four ecozones, paired-end 150-bp metagenomics reads were generated at the Joint Genome Institute, Walnut Creek, CA, USA. Sequences were quality-filtered as previously reported^12^.

### Carbohydrate degradation and nitrogen cycle gene databases

To identify genes potentially involved in carbon and nitrogen cycling (genetic potential), we compared our high-quality short reads against the carbohydrate active enzymes (CAZy) database^17^ and a custom database for nitrogen cycle enzymes. A database was created from the CAZy website (as of May 6, 2014) by downloading the corresponding proteins from Genbank using custom scripts. The CAZy database includes four conventional enzymatic classes: Glycoside hydrolase (GH), glycosyl transferase (GT), polysaccharide lyase (PL), and carbohydrate esterases (CE), as wells as the recently added *auxiliary activities* (AA) class. CAZy proteins are further classified into families, based on manual curation and phylogenetic analysis (CAZy similarity thresholds are not public).

**Figure 1:**
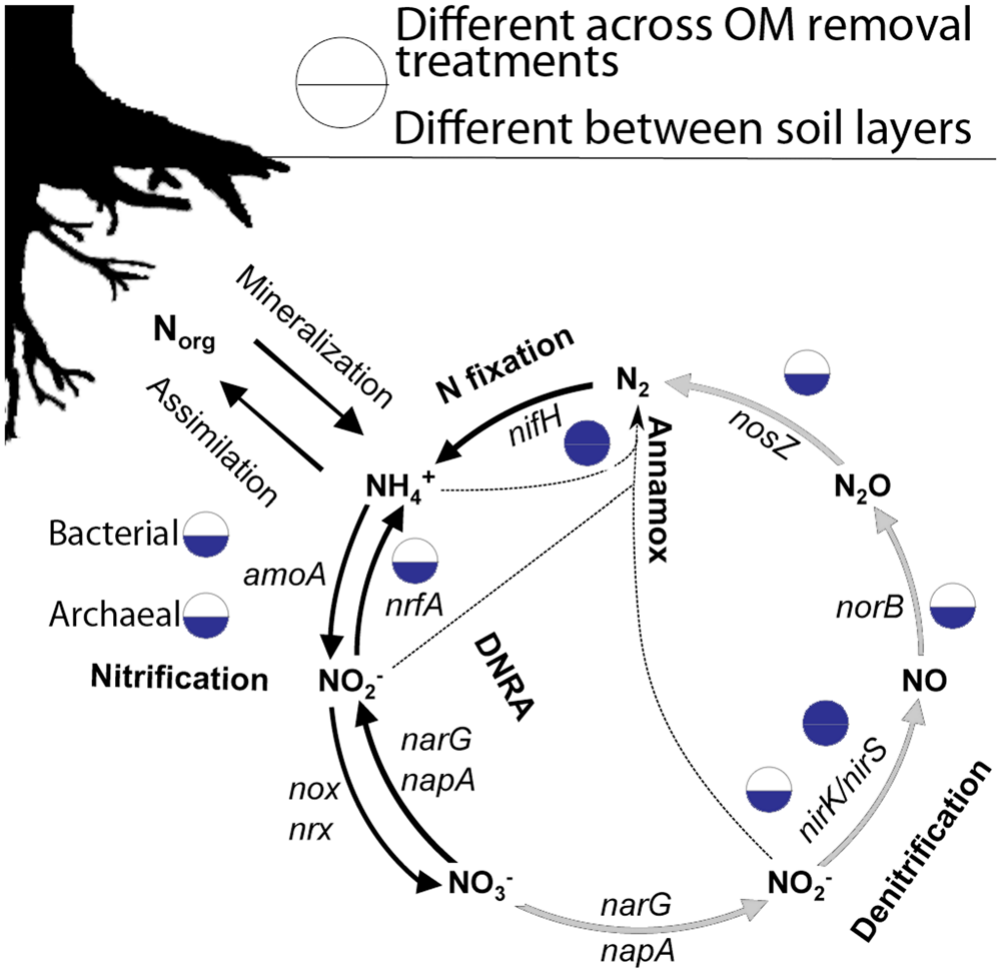
Model of nitrogen cycle in forest soils. DNRA: dissimilatory nitrate reduction to ammonia. Nitrite (NO_2_^−^) appears twice since it is an intermediate in denitrification, nitrification, and DNRA. The four reactions in denitrification are shown in grey arrows. Significant differences in abundance for genes targeted in our database are shown in blue (Anova p < 0.05, controlling for ecozone differences). Abundances for the IDF ecozone were not included in the calculation due to differences in the sequencing method for that ecozone.

The nitrogen cycle enzyme database included seven key enzymes of the nitrogen cycle (Fig. 1) representing nitrogen fixation (nifH), ammonia oxidation (bacterial and archaeal amoA), denitrification (nirS, nirK, norB, and nosZ), and dissimilatory nitrate reduction to ammonia (nrfA). We used a previously published database for the *nifH* gene^18^ and created custom databases for all the other nitrogen cycle genes. Protein sequences for amoA were retrieved from the FunGene database which uses Hidden Markov protein models^19^. On March 25, 2015, sequences were obtained and filtered according to their model fit. For filtering we used proteins with model coverage >75% and scores greater than 432.5 and 352 (for archaeal and bacterial versions, respectively). These minimum threshold scores were calculated using the score ratio between the lowest and highest sequences of the training set (i.e., the FunGene sequences) of archaeal amoA set. Custom databases for nirS, nirK, nosZ, norB, and nrfA were created by searching for proteins with related annotation in the NCBI’s Genbank and visually inspecting the alignments for the conservation of functional domains and motifs. The nitrogen cycle gene database was dereplicated by creating a group of sequences with a 95% identity threshold using cd-hit v4.6^20^. We classified nitrogen cycle protein into enzyme families using a ≥95% identity threshold. Thus, families of CAZy and nitrogen cycle enzymes are differently defined and not completely comparable.

Both forward and reverse reads as well as joined paired ends were compared against the databases using DIAMOND v0.5.3^21^. Hits were assigned to families according to the classification of the closest reference sequence, and counts were normalized to hits per million reads to account for differences in metagenome size. For both CAZy and nitrogen cycle genes, we used a 48.93 bits score threshold for a match for the similarity search. This score was found to be optimal in our previously reported analysis of CAZy families in the IDF site^12^. In addition, unlike E-values, bits scores are comparable among different databases.

### Data analysis

We used a 0.05 p-value as the significance threshold for all our statistical tests unless specified otherwise. Abundance information was analyzed as previously reported^12^. Significant differences in abundance among treatments were tested using ANOVA. Tests were done for each Ecozone-Layer combination. Post-hoc differences between treatment pairs were calculated using Tukey’s HSD test. The similarity of profiles was calculated using the Bray-Curtis dissimilarity index and visualized using principal coordinates (PCO) analysis. Constrained analysis of principal components (CAP) was performed with Bray-Curtis dissimilarities and environmental parameters (C, N, C/N ratio, water content, DNA recovery, and pH). For CAP, DNA recovery, N, and water content were removed as they were highly correlated with C which was considered a more important variable for modeling.

Linear regressions were calculated between the relative gene abundances (response) and each environmental parameter (predictor). Prior to regression, response variables with near zero variation were removed using the *NearZero* function of the caret R package. Since C and N measurements were calculated per plot for some ecozones, we averaged the abundances values of the triplicated samples for CAP and linear model estimations. For global analysis, we excluded one ecozone, IDF, because its metagenomes differed in read length from those of the other four ecozones. Variables with an adjusted R^2^ higher than 0.75, p-values < 0.05, and a false discovery rate <5% (q –value < 0.05) were considered significant for linear model calculations.

Gene families that predicted the classification of samples to soil layers or OM removal treatments were identified by Random Forest Analysis^22^ using 1000 trees. Prior to Random forest analysis, variables with no variation (SD = 0) were excluded. We selected the best predictors using Boruta, a feature selection algorithm^23^. Proteins from predictors families were taxonomically classified using the lowest common ancestor algorithm implemented in MEGAN v.5.10.1^24^.

Statistical analyses were done in the R environment v 3.1.2^25^ using the packages stats v3.1.2 (ANOVA, Tukey, Linear models, multiple test correction), Vegan v2.1 (PCO, CAP)^26^, randomForest v4.6-10 and Boruta v4.0.0, and caret v6.0-47. Detailed statistical results are shown in the Supplementary Table S3.

### Data availability

Raw sequence data for all ecozone is available at European Nucleotide Archive (http://www.ebi.ac.uk/ena) under study accession PRJEB8420, sample accessions ERS656878 - ERS656898 and ERS1420503 - ERS1420590). Raw sequencing data and annotations for PP, LP, JP, and JW are available at the JGI Genome portal (http://genome.jgi.doe.gov/, under JGI proposal ID 543).

## Results

After quality control, 7.79 × 10^9^ reads (1.17 Tb) were used for analysis. Around 81% of the reads passed our quality filter (Q20 over 70+ % read length), 68% of the paired reads were joined, and the final dataset had 70% of the initial bases. Our samples were dominated by bacterial sequences (>99.8%) according to taxonomic profiling using Metaphlan2^27^ (data not shown).

### Abundance of CAZy and nitrogen cycle genes

To evaluate the catabolic potential of the soil community, unassembled shotgun metagenomes were analyzed. We detected 252 CAZy and 1693 nitrogen cycle gene families. CAZy genes were more abundant than nitrogen cycle genes, averaging 14200 vs. 38.4 hits per million bases, respectively. Overall, CAZy genes had 12.8% higher relative abundances in the organic versus the mineral soil layer in unharvested reference treatments (OM0). This difference was driven by the GH, PL, and CE CAZy classes, while GT and AA classes were similarly distributed between layers. In contrast, nitrogen cycle genes had relative abundances 101% higher in the mineral than the organic soil layer in OM0. These general trends were also observed in the harvested treatments. Total CAZy gene abundances in OM0 varied among ecozones, 5–11% from the mean; while nitrogen cycle gene abundances were not significantly different among ecozones (data not shown).

**Figure 2:**
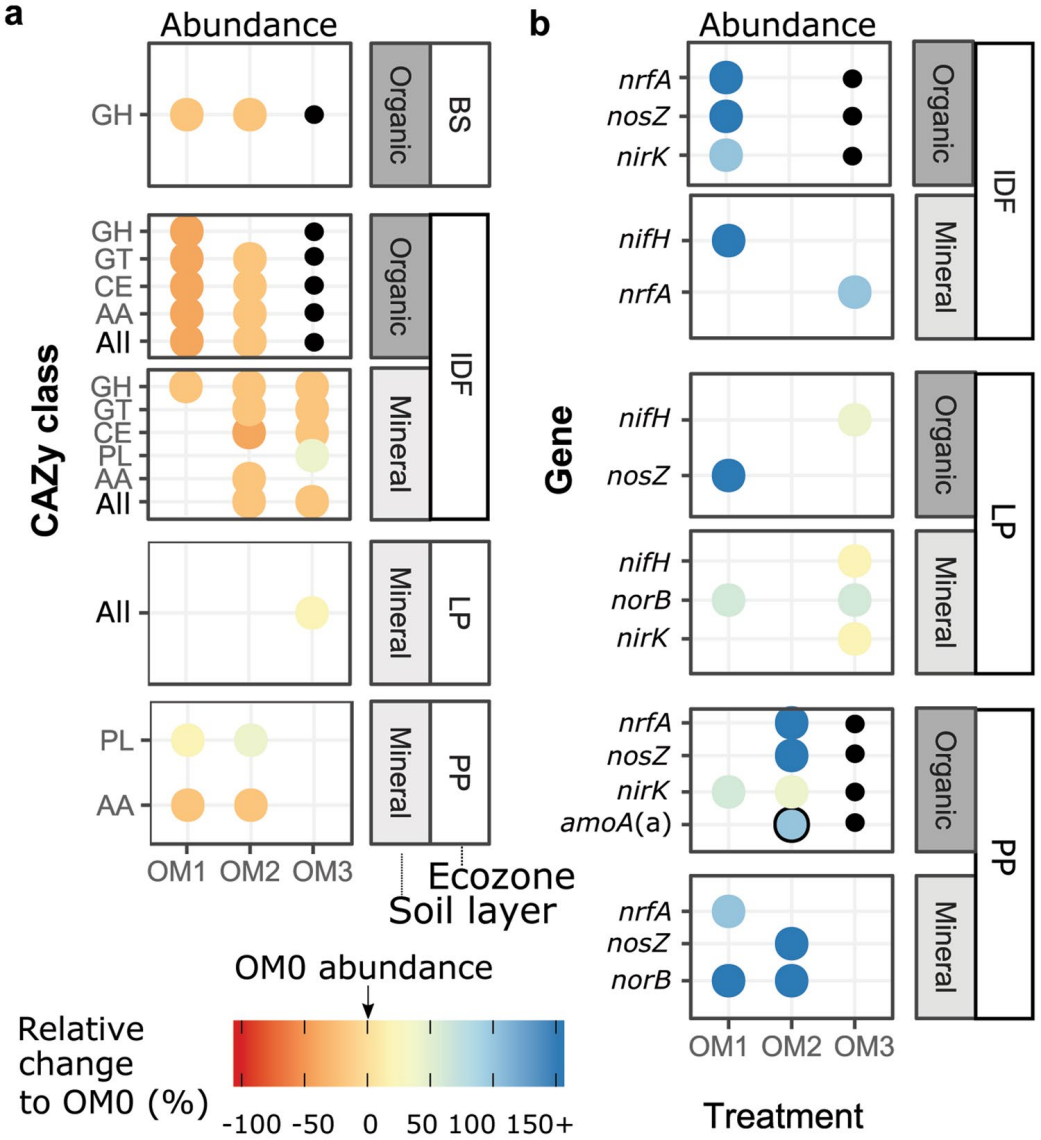
Effects of OM removal treatments on abundance of CAZy classes (**a**) and nitrogen cycle genes (**b**). Changes relative to the unharvested control (OM0) for that ecozone-layer are shown if significantly different (p < 0.05, ANOVA). For CAZy genes, “all” denotes the sum of abundances for the five classes. *amoA*(a) corresponds to the archaeal version of the *amoA* gene. Ecozones IDF, PP, BS, and JP lacked sufficient organic layer in the OM3 treatment for analysis and are marked with a black circle. When average values were zero for the OM0, the relative change was set to an arbitrary value of 100% (circled in black) as these relative changes cannot be mathematically defined.

More than ten years after harvesting and replanting, relative abundances of CAZy and nitrogen cycle genes remained significantly affected by the OM removal treatments, but these effects varied among both soil horizons and ecozones. In three of five ecozones (BS, IDF and PP), OM removal decreased the abundance of CAZy genes relative to OM0 (Fig. 2a). Individual CAZy classes responded similarly to harvesting, except the PL class was generally not affected, and increased in response to harvesting in the mineral layer in two ecozones.

In contrast to the CAZy genes, the nitrogen cycle genes typically increased in relative abundance after all OM removal treatments. Again, these effects varied greatly among ecozones, occurring in both soil layers of the IDF, LP and PP ecozones (Fig. 2b). Changes in relative abundances due to harvesting were greater for nitrogen cycle than CAZy genes. The most commonly affected functional genes types were related to denitrification (*nirK, norB, and nosZ*), dissimilatory nitrate reduction to ammonia (*nrfA*), and nitrogen fixation (*nifH*). Both *nirS* and bacterial *amoA* abundances were unaffected across all ecozones and layers.

Notably, the distribution of clade I and clade II *nosZ* types (also known as typical and atypical, respectively) varied across ecozones and layers. Clade I *nosZ* gene families were dominant in three ecozones (IDF, JP, and PP) where they comprised 63% of the *nosZ* sequences. On the other hand, clade II *nosZ* gene families were dominant in BS and LP ecozones (~69%). Both clade II, and to a lesser extent, clade I *nosZ* types increased with harvesting, but only in the IDF, LP and PP ecozones, mostly in the organic layer (data not shown).

### Alpha diversity of CAZy and nitrogen cycle genes

The richness of CAZy families was high for all samples, averaging 235 families per sample. The majority of the CAZy families (194) were encoded in all metagenomes, and so they are part of a core metagenome. Even greater numbers, 197 to 223 families, were part of the core metagenomes of individual ecozones. Within individual ecozones, 96 to 100% of CAZy gene families occurred in both soil layers. Nitrogen cycle gene families were much less conserved among ecozones, with only nine families, corresponding to three functional types (*nifH, nirK, and norB)*, found in all ecozones. These families were mainly associated with the *Alphaproteobacteria* (Table 1). Ecozone-specific core metagenomes contained more nitrogen cycle gene families (42 to 70), corresponding to as many as six functional gene types. No archaeal *amoA* gene family was part of the core metagenome in any ecozone. The proportion of nitrogen cycle gene families shared between soil layers varied but was markedly smaller for *nifH* (~38%) and *nirS* (~43%) families versus those of other nitrogen cycle gene types (64% to 84%; Supplementary Fig. 1).

**Table 1:** Ubiquitous nitrogen cycle gene families found in all forest soil samples. ^1^Average relative abundance across all ecozone normalized to hits per million reads.

Gene	Family	Abundance^1^	Phylum/Class	Family	Genus
*nifH*	Family320	9.61	*α-Proteobacteria*	*Bradyrhizobiaceae*	*Bosea*
*nirK*	Family0	4.53	*α-Proteobacteria*	*Bradyrhizobiaceae*	*Oligotropha*
*nirK*	Family16	0.34	*α-Proteobacteria*	*Hyphomicrobiaceae*	*Hyphomicrobium*
*nirK*	Family5	0.18	*γ-Proteobacteria*	*Xhantomonadaceae*	*Rhodanobacter*
*norB*	Family132	0.92	*Acidobacteria*	*Solibacteraceae*	*Solibacter*
*norB*	Family1	0.72	*δ-Proteobacteria*	*Polyangiaceae*	*Sorangium*
*norB*	Family6	0.45	*γ-Proteobacteria*	*Legionellaceae*	*Legionella*
*norB*	Family47	0.38	*δ-Proteobacteria*	*Anaeromyxobacteraceae*	*Anaeromyxobacter*
*norB*	Family33	0.35	*α-Proteobacteria*	*Bradyrhizobiaceae*	*Bradyrhizobium*

CAZy gene family relative abundance profiles were highly uneven, and consistently dominated by one or two families in each CAZy class. Dominant families were AA3 (51–65% of total AA abundance), CE4 (23–34%), GH13 (18–37%), GT4 (17–29%), and PL9 (22–28%). Similarly, nitrogen cycle gene family relative abundance profiles were strongly dominated by a few families. Strong patterns of dominance were found for families of five of the eight nitrogen cycle gene types studied (Table 2). These dominant families were associated with the *Bradyrhizobiaceae (Alphaproteobacteria; nifH, nirK*), *Gammaproteobacteria* (bacterial *amoA, nirS*), and *Deltaproteobacteria* (*nrfA*). For *nifH*, Family 320 was dominant in all ecozones and soil layers. Clade I *nosZ* gene families from the *Bradyrhizobiaceae* were dominant in mineral layers of all ecozones, while clade I and II *nosZ* gene families from other taxonomic groups were dominant in organic layers.

**Table 2:** Taxonomic classification of the dominant family of each nitrogen cycle gene type.

Gene	Most abundant family		Dominance									
	Name	Taxonomy (Phyla - Family)	BS		PP		JP		IDF		LP	
			Min	Org	Min	Org	Min	Org	Min	Org	Min	Org
*amoA* ^a^	Family148	*A – Archaea* ^c^	A	A	A	•	A	A	A	•	A	A
*amoA* ^b^	Family13	*GP - Chromatiaceae*	•	•	•	BP	•	•	BP	BP	•	•
*nifH*	Family320	*AP - Bradyrhizobiaceae*	•	•	•	•	•	•	•	•	•	•
*nirK*	Family0	*AP - Bradyrhizobiaceae*	•	•	•	•	•	•	AP	•	•	•
*nirS*	Family3	*GP - Alcanivoracaceae*	•	•	•	•	GP	•	GP	GP	•	•
*norB*	Family132	*AC - Solibacteraceae*	DP	BP	•	•	BP	BP	•	•	Ac	•
*nosZ*	Family57	*AP - Bradyrhizobiaceae*	•	BP^d^	•	BP	•	Fi^d^	AP	Ba^d^	AP	BP
*nrfA*	Family109	*DP - Pelobacteraceae*	DT	•	•	•	DT	DT	DP	•	DP	•

A dot (•) denotes where the overall most abundant family was dominant, otherwise the classification of the dominant family for that ecozone/layer is shown. Taxonomic classification is based on identity of closest relative in database. Phyla abbreviations: A, Archaea; AP, Alphaproteobacteria; BP, Betaproteobacteria; GP, Gammaproteobacteria; DP, Deltaproteobacteria; Ba, Bacteroidetes; DT, Deinococcus-Thermus; Fi, Firmicutes; Ac, Acidobacteria. ^a^Archaeal version of *amoA*, ^b^Bacterial version of *amoA*. ^c^Classification of archaeal *amoA* was only to phylum level. ^d^Clade II *nosZ* gene variant.

The richness within each CAZY gene family (unique hits in the database) was higher in the organic layer than in the mineral layer, except in the JP ecozone. Within-family richness was affected by harvesting in only the organic layer of one ecozone (IDF), where the richness of three classes declined an average by 19% in OM1 versus OM0 (Data not shown).

Between soil layers, alpha diversity was similar for nitrogen cycle genes, except bacterial *amoA* richness was 2.2- to 5-times higher in the mineral than in the organic layer in all but one ecozone (IDF). Alpha diversity of nitrogen cycle genes was increased by OM removal, most often differing between OM0 and other harvested treatments, in three ecozones in both soil horizons (Data not shown). The component of alpha diversity affected by OM removal varied among ecozones and soil layers, with richness more affected in the IDF and evenness more affected in the LP and PP.

### Beta Diversity of gene profiles

**Figure 3:**
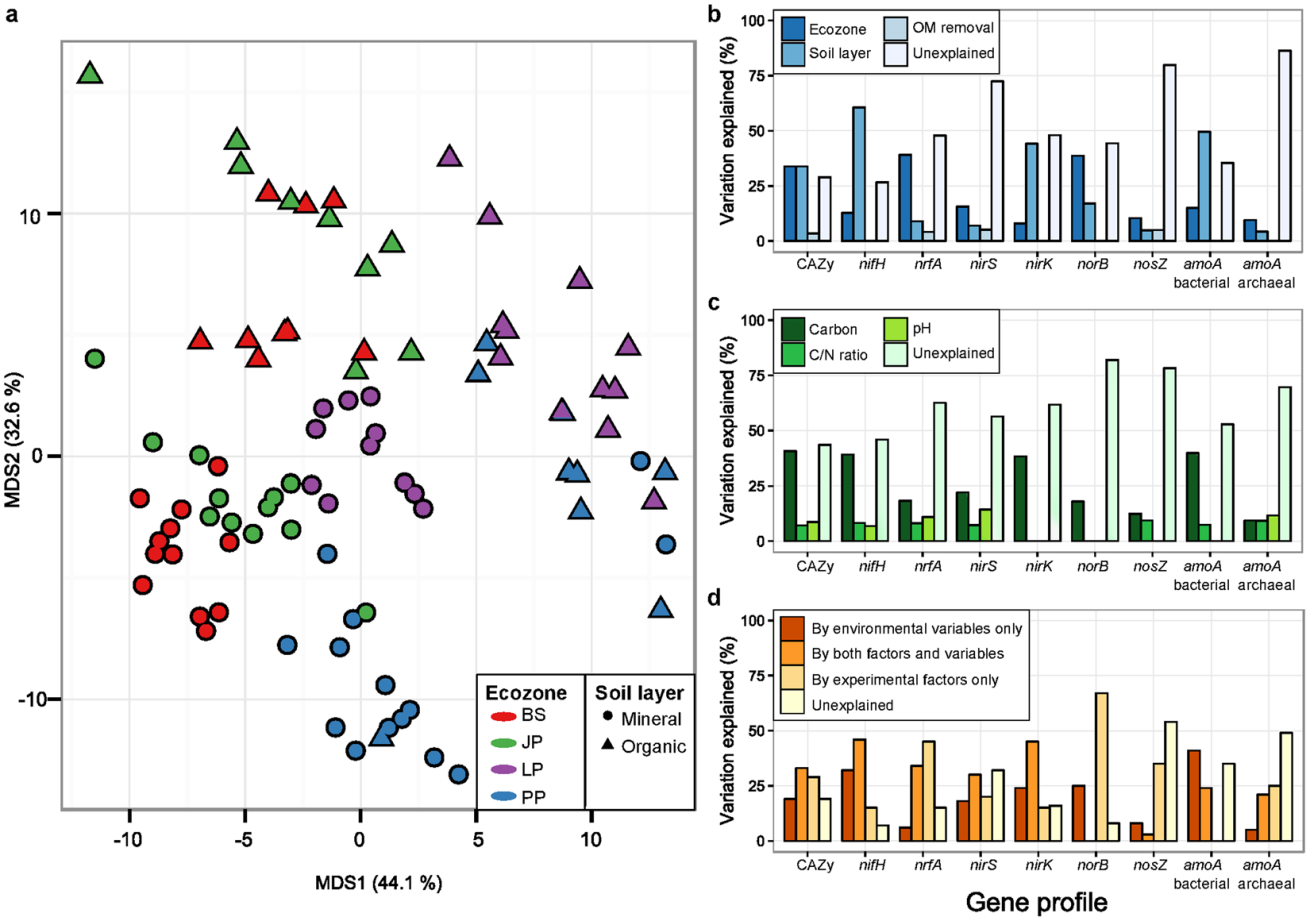
Effects of soil layer, ecozone and OM removal treatments on beta diversity of CAZy and nitrogen cycling gene families. Unconstrained principal coordinates analysis using Bray-Curtis distances of CAZy gene family profiles (**a**). Variation explained by each experimental factor based on PERMANOVA (**b**). Variation explained by environmental variables (C, C/N, and pH) based on constrained analysis of principal coordinates (**c**). Partition of variation between experimental factors and environmental variables (**d**). Significance of the fraction was tested using a permutational test for the redundant analysis results using 999 permutations over a Hellinger-transformed data. For B and C, all variation shown was statistically significant (p < 0.05). *p < 0.05, **p < 0.01, ***p < 0.001. Total nitrogen content was highly correlated with carbon content (Spearman R = 0.92); therefore variation attributed to carbon could alternatively be due to nitrogen.

Principal coordinates analysis, based on the similarity of CAZy gene family relative abundance profiles, grouped samples predominantly by soil layer and ecozone (Fig. 3a). PERMANOVA confirmed the importance of soil layer and ecozone in accounting for the majority of profile variability, while the variability explained by OM removal treatments was significant but relatively small (Fig. 3b). Our global model, which considers ecozones, layer, and OM removal treatments, showed differences between harvested versus unharvested treatments, but differences among the three harvested OM removal treatments were only found between OM1 and OM3 (Supplementary Table 4). Within most ecozones, the soil layer effect was large (~57% of variation), except for the PP ecozone where samples did not group by layer. The individual CAZy classes generally showed similar trends (Supplementary Fig. S2a). Within each ecozone, layer effects varied by CAZy class, with GH, PL, and AA classes most affected. OM removal effects on CAZy gene profiles were highly variable among ecozones and soil layers (Supplementary Fig. S2b).

The analysis of variation also showed that ecozone and soil layer had strong effects on nitrogen cycle gene family profiles (Fig. 3b). Three distinct patterns were found among these genes: *nrfA* and *norB* were highly influenced by ecozone (average, 38.9% of variability), *nifH, nirK*, and bacterial *amoA* were strongly influenced by the soil layer (average, 51.4%) and *nirS, nosZ* and archaeal *amoA* showed little influence of any of the explanatory factors with around 80% of variation remaining unexplained. The influence of soil layer on profiles of nitrogen cycle genes varied greatly among ecozones (Supplementary Fig. S2c). OM removal treatments had a substantial effect on the variability of nitrogen cycle gene profiles within individual ecozones, but this effect was again highly variable among the ecozones and soil layers.

When we analyzed the influence of environmental variables on the variation of CAZy and nitrogen cycle gene profiles we found strong influences of carbon content, pH, and the C/N ratio (Fig. 3c). The proportion of variation explained differed substantially among profiles of the various genes. Notably, the genes that were most heavily influenced by soil layer were also most heavily influenced by carbon content. The explanatory power of OM removal treatments partially overlapped with the explanatory power of the environmental variables, according to a variation partition analysis (Fig. 3d).

### Predictive gene families

Random Forests analysis found eleven CAZy and nine nitrogen cycle gene families to be significant predictors of soil layers across all five ecozones (Supplementary Fig. S3a). In contrast, no family was a significant predictor of OM removal treatments across all ecozones in either soil layer. The majority of CAZy gene families studied (81%) were found to be predictors of soil layer in at least one ecozone, in contrast to only 16.7% of the nitrogen cycle gene families. The number of layer predictors varied among ecozones and was notably lower in the PP ecozone, for both CAZy and nitrogen cycle genes, and in the IDF ecozone for nitrogen cycle genes (data not shown). For both CAZy and nitrogen cycle gene families, classification of samples to soil layers was very accurate (median error rate, 5.7%), classification to harvested versus unharvested plots was worse (median error rate, 42. 5%), and classification among OM removal treatments was generally very inaccurate (median error rate, 85.3%) (Supplementary Fig. S3b).

The CAZy gene families predictive of soil layer across all five ecozones (“universal” predictors) were mostly GH families associated with the organic layer and plant cell wall degradation, particularly cellulose and hemicellulose (Table 3). The nitrogen cycle “universal” predictors of layer were mostly *nifH* gene families, associated with the *Proteobacteria*, especially the *Alphaproteobacteria* class (Table 3). Most of these *nifH* families were more abundant in the organic layer, whereas other predictors from bacterial *amoA* and *nirK* families were more abundant in the mineral layer. The relative abundance of some of these predictors changed with OM removal (Table 3). Bacteria were the main contributors to these gene families, except for GH12 which also had a significant fungal contribution. The taxonomic affiliation of these “universal” predictors changed minimally across soil layers and OM removal treatments as shown for the GH12 family (Supplementary Fig. S4). Notably, the taxonomy of these predictors in the IDF ecozone tended to differ from those in the other four ecozones.

**Table 3:** Universal predictor of soil layer for five ecozones.

Gene family	Reaction/Substrate	Abundance	Layer preference	Abundance affected by harvesting	Taxonomic classification
CE6	PCW–Hemi	36.20	Organic	**↓** in IDF, PP	Bac (94%)
GH3	PCW–Hemi	522.10	Organic	**↓** in BS, IDF	Act (22%), Pro (21%), Aci (20%), Bac (17%)
GH10	PCW–Hemi	50.19	Organic	—	Act (21%), Aci (19%), Pro (12%), Bac (12%)
GH11	PCW–Hemi	14.67	Organic	**↓** in BS	Unk (52%), Act (27%)
GH12	PCW–Cell	8.72	Organic	**↓** in IDF	Pro (38%), Act (37%), Fungi (15%)
GH28	PCW–Pectin	170.71	Organic	**↓** in IDF	Aci (23%), Viri (46%), Bac (13%)
GH51	PCW–Hemi	100.11	Organic	—	Aci (37%), Bac (16%), Act (11%)
GH64	FCW–Gluc	19.52	Organic	**↓** in BS, IDF, PP	Act (46%), Aci (39%)
GH115	PCW–Hemi	19.17	Organic	**↓** in BS	Ver (32%), Bac (25%),
GH128	FCW–Gluc	7.24	Organic	**↓** in IDF, PP, ↑ in JP	Act (49%), Pro (26%)
PL22	PCW–Pectin	9.26	Organic	—	Aci (71%), Pro (11%)
*nifH*-Family320	N_2_→NH_4_^+^	9.60	Mineral	—	Pro-α (*Bradyrhizobiaceae*)
*nifH*- Family2432	N_2_→NH_4_^+^	0.11	Organic	**↑** in JP, LP	Pro-α (*Acetobacteraceae*)
*nifH*- Family1345	N_2_→NH_4_^+^	0.10	Organic	**↑** in JP, LP	Pro-α (*Acetobacteraceae*)
*nifH*- Family1099	N_2_→NH_4_^+^	0.12	Organic	—	Pro-α (*Sphingomonadaceae*)
*nifH*- Family1562	N_2_→NH_4_^+^	0.09	Organic	—	Pro-α (*Methylobacteriaceae*)
*nifH*- Family58	N_2_→NH_4_^+^	0.64	Organic	**↑** in JP, LP	Cya (*Prochlorococcaceae*)
*amoA*(Bac)- Family2	NH_4_^+^→NO_2_^−^	0.12	Mineral	—	Pro-β (*Nitrosospira*)
*amoA*(Bac)- Family13	NH_4_^+^→NO_2_^−^	0.33	Mineral	—	Pro-γ (*Nitrosococcus*)
*nirK*- Family25	NO_2_^−^→NO	0.38	Mineral	—	Pro-γ (*Aeromonadaceae*)

Classification of CAZy families is based on Lowest common ancestor algorithm implemented in MEGAN. Abundance was measured as hits per million reads. Substrates; PCW: Plant cell wall, FCW: Fungal cell wall, Hemi: Hemicellulose, Cell: Cellulose, Gluc: β-1,3-glucan. Taxonomy; Act: *Actinobacteria*, Aci: *Acidobacteria*, Bac: *Bacteroidetes*, Cya: *Cyanobacteria*, Pro: *Proteobacteria*, Ver: *Verrucomicrobia*, Unk: Unknown, Viri: *Viridiplantae*.

Within each ecozone, predictors of OM removal treatments were found in both soil layers. The number of such predictors varied among ecozones but showed similar patterns for both CAZy and nitrogen cycle genes, being the highest in the mineral layer in the PP ecozone and in the organic layer in the IDF ecozone (Data not shown).

### Gene abundances were correlated with environmental variables

**Figure 4:**
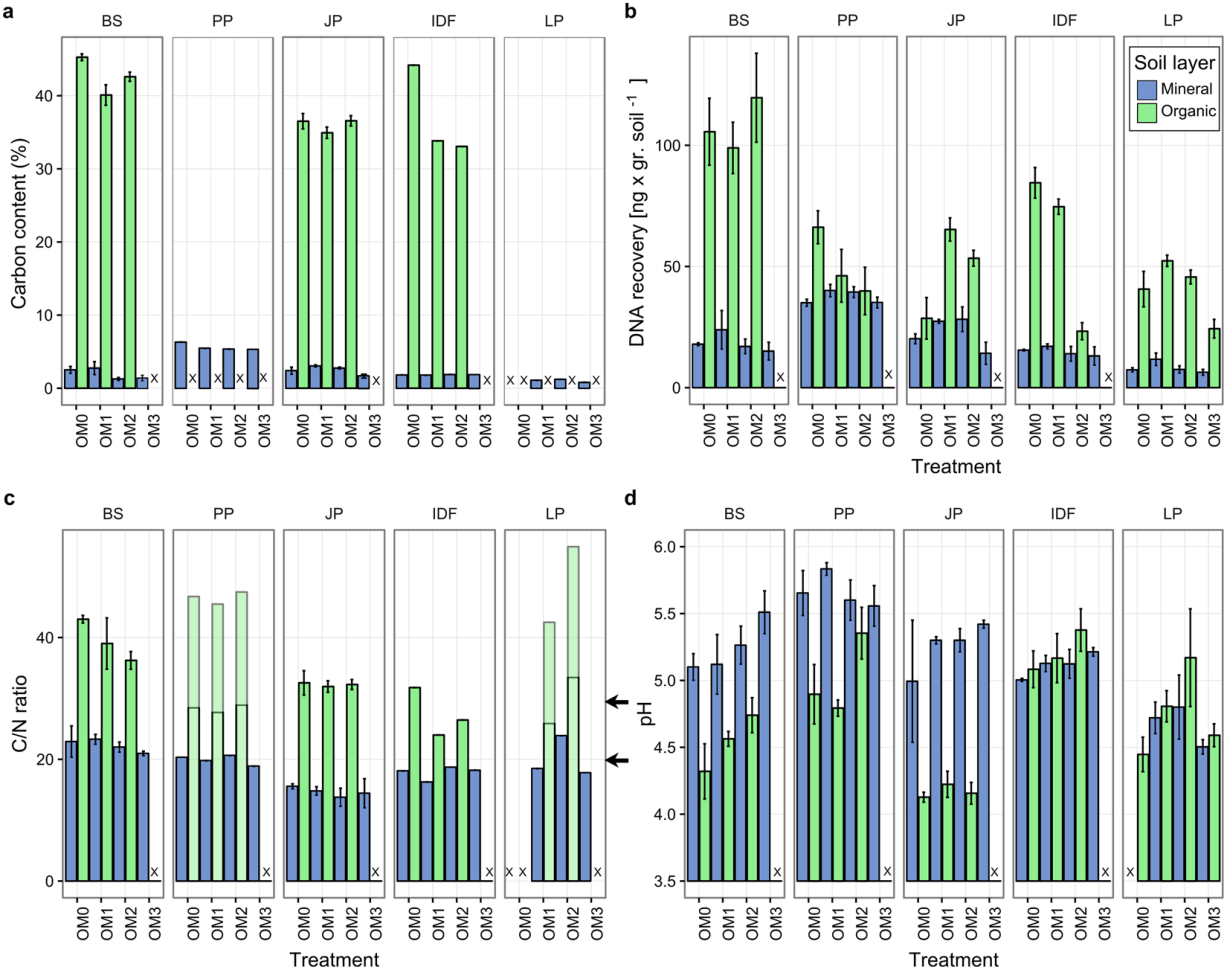
Changes in soil biogeochemistry due to OM removal treatments. X, no data available. Arrows indicate thresholds for nitrogen limitation (C/N ratios >30), and carbon limitation (C/N ratios <20). Error bars indicate standard error. C/N ratios for PP and LP organic layer samples were estimated using the C/N ratio for the corresponding mineral samples and the relationship between values of mineral and organic samples of the other ecozones, with estimated values shown as a range (upper portion of bars).

Soil composition varied across ecozones, soil layers, and OM removal treatments (Fig. 4, Supplementary Table S2). Organic layer samples had higher C, N, C/N ratios, and DNA (a proxy for microbial biomass) than mineral layer samples. The only exception was the PP ecozone, which had similar DNA in both layers in the OM1 and OM2 treatments. When comparing mineral and organic soil layers, pH levels were higher in the organic layers in the PP, IDF, and BS ecozones, similar between layers in the LP ecozone, and higher in the mineral layer in the JP ecozone. OM removal tended to reduce the microbial biomass, total carbon, total nitrogen, and the C/N ratio and increase the pH. These changes were mostly found in the organic soil layer. Soil DNA was highly correlated with C content (Pearson’s R = 0.82). Patterns of DNA concentration and CAZy and nitrogen cycle gene abundances were significantly correlated (Mantel statistic r = 0.5849 and 0.608 respectively, both p < 0.001).

**Figure 5:**
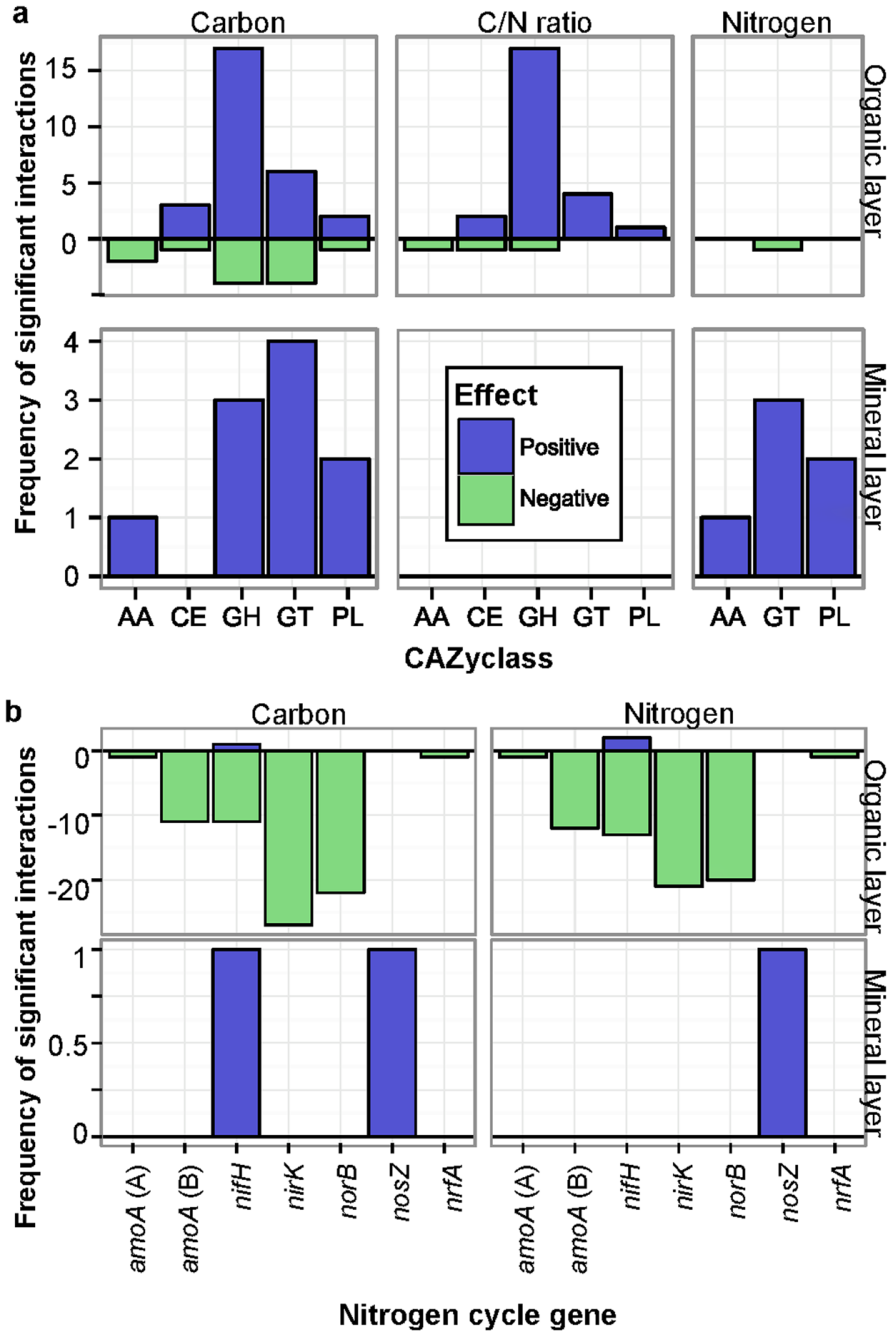
Significant relationships between environmental variables and CAZy (**a**) or nitrogen cycle gene families (**b**). Shown are the significant relationships found using linear models which had an adjusted R^2^ > 0.75 (p < 0.05, q < 0.05).

We related gene abundances to environmental variables using linear models for each soil layer separately and found strong correlations with environmental variables for 53 CAZy gene families and 86 nitrogen cycle gene families in at least one soil layer (Supplementary Figs S5,S6). Correlations involving CAZy gene families were predominately positive and occurred most frequently in the organic layer (Fig. 5a). In the organic layer, these correlations were mainly between carbon content or C/N ratio and GH or GT families. In the mineral layer, these correlations involved carbon or nitrogen content. In contrast, for nitrogen cycle gene families, the correlations were almost exclusively negative and occurring in the organic layer (Fig. 5b). The majority of these correlations were between carbon or nitrogen content and families of *nirK, norB, nifH*, or bacterial *amoA*.

## Discussion

Overall, harvesting per se, and not the level of OM removal associated with harvesting, had the greater impact on the soil metagenomic potential for organic decomposition and nitrogen cycling. Our global PERMANOVA models found significant differences in gene profiles between unharvested versus harvested treatments but rarely among the three harvested treatments (Supplementary Table S4). This pattern is consistent with our previous results pertaining to community composition and populations of hemicellulose and cellulose degraders^9,11,28,29^. Harvesting has a number of interrelated consequences, discussed below, many of which are directly or indirectly related to OM removal. Thus, in assessing the effects of the OM removal treatments, we mainly found what can be considered general harvesting effects, but we also note some effects of the extent of OM removal.

Forest harvesting can affect both aboveground and belowground systems. Aboveground impacts include changes in plant cover, niche availability, and microclimate, which can, in turn, affect the composition and activity of the soil communities^30^. Below ground, harvesting changes both the quantity and quality of organic inputs and directly and indirectly alters soil chemistry, including increases in pH and reductions the C/N ratio, and the soil carbon, nitrogen, and phosphorus pools^6,31,32^. Reductions in the microbial biomass have also been reported^8^. We found long-term (greater than 10-year) effects of harvesting on soil chemistry and microbial biomass. Harvesting also generally decreased the genetic potential for biomass decomposition and increased that for nitrogen-cycling. Changes in genetic potential were accompanied by shifts in the diversity of the corresponding genes. These changes in genetic potential were consistent with our main hypotheses. However, these effects of harvesting were not universal and were highly variable among ecozones and soil layers.

The ecozone-specific effects of harvesting on CAZy and nitrogen cycle genes were substantially explained in the context of ecozone-specific changes in soil chemistry. Total carbon was the main explanatory factor of CAZy gene occurrence. Accordingly, ecozones where harvesting most decreased CAZy gene abundances (IDF, PP, and LP) were also those with the strongest decreases in total carbon due to harvesting. Carbon was also the strongest explanatory variable of nitrogen cycle gene occurrence, but C/N and pH were also important factors for these genes. Notably, the C/N ratio is a determinant of the fate of soil organic N. At C/N ratios greater than 30, nitrogen becomes limiting and the mineralized nitrogen goes preferentially into microbial biomass, while at ratios less than 20, nitrogen is used for catabolism, resulting in loss of gaseous nitrogen species and leaching of nitrate^33^. We found low C/N ratios indicative of nitrogen loss in mineral layer samples, and harvesting tended to decrease the ratio. In ecozones with the highest C/N ratios (BS) and/or no significant changes in the ratio due to harvesting (JP), there was little impact of harvesting on nitrogen cycle gene abundance. In contrast, ecozones with lower initial C/N ratios (IDF, PP), or where harvesting reduced the ratio below 25 (IDF), showed greater increases in nitrogen cycle gene relative abundance, richness, and Shannon’s diversity (Fig. 2) as well as greater changes in the overall gene family abundance profile (Supplementary Fig. S2d). In particular, the increased abundance of genes involved in denitrification and DNRA associated with harvesting may be due to increased nitrate production by nitrogen-mineralizing populations. Increases in nitrification and denitrifrication genes, have also been observed in temperate forests with artificial N deposition, which also decreases the soil C/N ratio^34^.

Harvesting typically increases soil pH with consistent effects on nitrogen cycling. A meta-analysis found that clear-cut harvesting in boreal and temperate forests increases pH, a controlling factor in nitrogen fixation, nitrification, and denitrification (all reduced in acidic soils)^32^. Harvesting increased the pH in PP, BS, and LP ecozones, and although pH changes did not exactly match the changes in the occurrence of carbon and nitrogen cycle genes, they may have contributed to shifts in the community structure of nitrogen fixers, and nitrifiers. Thus, increased pH associated with harvesting may have increased available nitrate and further promoted populations responsible for denitrification and DNRA. Changes in pH can also lead to changes in the bacterial-fungal dynamic since bacterial populations are less abundant at lower pH conditions. This agrees with previous studies that have shown that fungal populations are both more active in biomass decomposition in the organic layer^35^ and more sensitive to changes due to harvesting^9,12^.

Additionally, it is possible that harvesting, through compaction, created conditions that further favored the anaerobic respiratory processes of denitrification and DNRA. Compaction of temperate forest soil increased the abundance of methanogens, methane production and its flux from soils^36^ as well as increased the nitrous oxide flux and reduced the soil microporosity^37^. Harvesting operations typically increase soil bulk density and reduce soil aeration; although, this is not always detrimental to soil productivity^38^. Although compaction was minimized in the plots we studied, we observed increased bulk densities after harvesting in the BS, and IDF ecozones, no changes in the JP ecozone, and have no such data for the LP and PP ecozones (Supplementary Table S2). We lack data for redox state, which might better indicate changes affecting respiration processes. Overall, our results suggest several mechanisms by which populations responsible for organic decomposition and nitrogen cycling were affected by harvesting, and further suggests that the increased sensitivity of those populations to harvesting in the BS and IDF ecozones is at least partially dependent on the relative availabilities of carbon and nitrogen as well as pH levels prior to harvesting.

Harvesting also opened new ecological niches in the soil as evidenced by increased abundance of PLs and some specific AA families. Contrary to the general trend where CAZy abundances decreased with harvesting and were positively correlated with carbon content, AA3, AA5, and PL13 increased in abundances with lower carbon content. PLs are a group of enzymes that use a lytic elimination mechanism and act on long polysaccharides such as pectins and alginates, and are only found in prokaryotes^39^. Harvesting increased the abundance of PLs in the IDF and PP ecozones, while other CAZy classes were negatively affected, suggesting changes in the types of organic matter available. Interestingly, some AA3 enzymes have been shown to generate H_2_O_2_ which can be used by lignin peroxidases^40^. Overall, by altering substrate availability, harvesting appears to select for populations that can use recalcitrant substrates not otherwise available.

In the rare cases where we found differences in gene profiles among the three OM removal treatments, they were generally between OM1 and OM3 (Supplementary Table S4). These results are consistent with previous reports that plant productivity was affected by only the most extreme harvesting regime (OM3), which dramatically changes the soil environment, increasing temperature, while reducing moisture, microbial biomass and cellulolytic populations^5,7^. A meta-analysis found that whole tree harvesting (OM2) is more likely than bole-only harvesting (OM1) to cause short-term losses of carbon and nitrogen^41^, but any such effects did not have a long-term impact on the soil community potential for decomposition and nitrogen cycling at the sights we studied.

Ecozone variability was the most important confounding factor in the evaluation of the long-term effects of OM removal on soil genetic potential. Ecozone variability was proposed to be mainly related to differences in climate, tree species and soil characteristics in a meta-analysis of tree biomass and foliar nutrition 10 years after harvesting and replanting at 27 LTSP Study sites^7^. These three main factors also affect soil microbial communities by controlling the soil nutritional status, controlling the quantity and quality of litter inputs, and by modulating key environmental conditions, such as moisture, temperature, and aeration. In our study, the variability of both CAZy and nitrogen cycle gene family profiles was explained by both the experimental factors (ecozone, soil layer, and OM removal treatments) as well as key environmental variables (Fig. 3, Supplementary Fig. S3). The overlapping explanatory power of experimental and environmental variables suggests that total carbon, total nitrogen, C/N ratio, and pH substantially drive the effects of the experimental variables on the metabolic potential of the soil community via selective pressure on decomposer and nitrogen-cycling populations. Soil chemistry, particularly pH, has been found to be a major determinant of soil bacterial diversity across North and South America^42^, highly correlated with bacterial abundances in Canadian agroforestry systems^43^, a driver of bacterial diversity under twelve North American forests^44^, and a driver of nitrogen cycle gene abundance over 107 sites across the Burgundy French region^45^.

Additional drivers of variation among ecozones in our study, which we did not measure, were likely the aboveground vegetation and soil nitrogen composition. Vegetation composition has been shown to be as important as soil chemistry in determining bacterial and fungal composition in forest soil^43,46^, because it influences the quantity and variety of carbon sources (as plant biomass) that enter the soil as carbon sources as well as the potential plant-microbe interactions. However, since the LTSP experiments in each ecozone had a different dominant tree species and associated vegetation, we cannot separate the ecozone and the vegetation effects. Different forms of nitrogen, such as nitrate and ammonia, as substrates for particular nitrogen-cycling populations, were found to be correlated with abundances of the corresponding genes^47^. These populations, in turn, modulate the fate of the nitrogen in the soil.

Forest soil is a highly stratified environment with particularly strong biochemical and biological differences between mineral and organic layers. The organic layer has higher carbon and nitrogen concentrations, higher microbial abundances, and higher denitrification and nitrogen fixation rates^48^. Accordingly, we observed large differences in soil chemistry and microbial biomass between layers (Fig. 4) as well as a strong influence of the soil layer on the distributions of decomposition and nitrogen cycle genes (Figs 2 and 3).

Despite our results that showed inconsistent effects of the OM removal treatments, we found a small set of gene families that were conserved in their distribution and seemed unaffected by OM removal. These core genes likely encode key functions in the degradation of plant biomass. These core genes were mainly bacterial; however, we did not sample the litter layer where fungal groups are reportedly more active^35^. The involvement of CAZymes in plant biomass decomposition is consistent with their skewed distribution toward the organic layer, and the predominance of plant cell wall degradation genes as soil layer predictors across ecozones. Additionally, CAZy genes showed more frequent and stronger correlations with C and the C/N ratio in the organic layer. These results are consistent with a meta-analysis of forest harvesting studies that showed that carbon storage in the organic layer was more sensitive to harvest impacts than in the mineral layer^31^. As the role of CAZy genes in decomposition would suggest, we found strong positive associations in the organic layer between CAZy GH and GT families and total carbon and the C/N ratio (Fig. 5, Supplementary Fig. S5). However, some AA families had negative associations with those two variables, which seems counterintuitive.

The distribution of nitrogen cycle genes suggests that they contribute proportionally more to catabolism in the mineral versus organic layer. However, this does not imply that more nitrogen cycling occurs in the mineral layer since it has lower biomass and lower total N than the organic layer. The C/N ratios imply that the organic layer is nitrogen-limited while the mineral layer is carbon-limited, likely making microbial catabolism of nitrogen compounds more favorable relative to heterotrophy in the mineral layer. This hypothesis is supported by the enrichment of nitrogen cycle genes in the mineral layer, both in terms of relative abundance and diversity (number of gene families) (Supplementary Fig. S1). The strongest case for specialization was from archaeal ammonia oxidizers, as the archaeal *amoA* gene was exclusively present or much more abundant in the mineral versus organic layer. Archaeal ammonia oxidizers are well adapted to low nitrogen concentrations, low pH, and low O_2_^49^ and can outcompete their bacterial counterpart in deep soil layers. However, total bacterial *amoA* abundances were higher than archaeal ones in 94% of the cases.

In addition, we found that two bacterial *amoA* families were predictors of mineral soils across all ecozones. These families were affiliated with *Nitrosospira* (*Betaproteobacteria*) and *Nitrosococcus* (*Gammaproteobacteria*), which are frequently found in low-pH forest soils, where they may benefit from urease activity^50^. Layer specialization was also found for nitrogen fixers, as five *nifH* families, four from *Alphaproteobacteria* and one from *Cyanobacteria* were consistently associated with the organic layer and family 320, the most abundant *nifH* family from a *Bradyrizobiaceae* member was consistently associated with the mineral layer. Nitrogen fixation is a key link between the nitrogen and carbon cycles, as fixed N may prime degradation of high-C/N litters^51^. These results suggest that populations carrying nitrogen cycle genes depend not only on nutrient availability (which is higher at the organic layer) but also on other drivers such as pH and C/N ratio.

Harvesting had long-term effects on the capacity of the forest soil community for carbon and nitrogen cycling, which were consistent with our initial hypotheses. These impacts were distinct in the organic and mineral soil layers and were highly variable among different ecozones in a manner dependent on key environmental variables. These alterations of the soil community were not associated with major effects on tree productivity. However, these alterations might presage later effects on productivity as the trees grow and have greater nutrient demands or effects on the resilience of the forest system to future perturbations. Our results suggest a mechanism by which harvesting can exacerbate nitrogen losses at sites predisposed to such losses, potentially lowering plant productivity and increasing greenhouse gas emissions.

## Electronic supplementary material

Supplementary materials
