# Supplementary material for: Effects of timber harvesting on the genetic potential for carbon and nitrogen cycling in five North American forest ecozones
Source: Sci Rep. 2018 Feb 16;8:3142. doi: 10.1038/s41598-018-21197-0 (PMC5816661; doi:10.1038/s41598-018-21197-0)
Supplement: Supplementary file 1 — Supplementary materials [file 41598_2018_21197_MOESM1_ESM.pdf]

Supplementary Materials

Effects of timber harvesting on the genetic potential for carbon and nitrogen cycling in five North American forest ecozones

Erick Cardenas[1], Luis H. Orellana[2], Konstantinos T. Konstantinidis[2], William W. Mohn[1*]

[1]Department of Microbiology and Immunology, Life Sciences Institute, University of British Columbia, Vancouver, BC, V6T 1Z3, Canada.

2. Georgia Institute of Technology, School of Civil and Environmental Engineering, Atlanta, GA, 30332, USA

*Correspondence to wmohn@mail.ubc.ca

Contents:

Supplementary figure S1. Presence of nitrogen cycle gene families in soil layers

Supplementary figure S2. Permutational multivariate analysis of variation of CAZy and nitrogen cycle gene family profiles.

Supplementary figure S3. Analysis of differentially distributed gene families

Supplementary figure S4. Taxonomic composition of sequences of the GH12 family across OM removal treatments.

Supplementary figure S5. Correlations between environmental variables and CAZy gene families.

Supplementary figure S6. Relationship between environmental gradients and nitrogen cycle genes

Supplementary Table 1. Site characteristics

Supplementary table 2. Biochemical characterization of soil samples

Supplementary table 3. Detailed statistical results

Supplementary table 4. Differences in gene family abundance profiles among harvesting treatments using PERMANOVA

Supplementary figure S1. Presence of nitrogen cycle gene families in soil layers

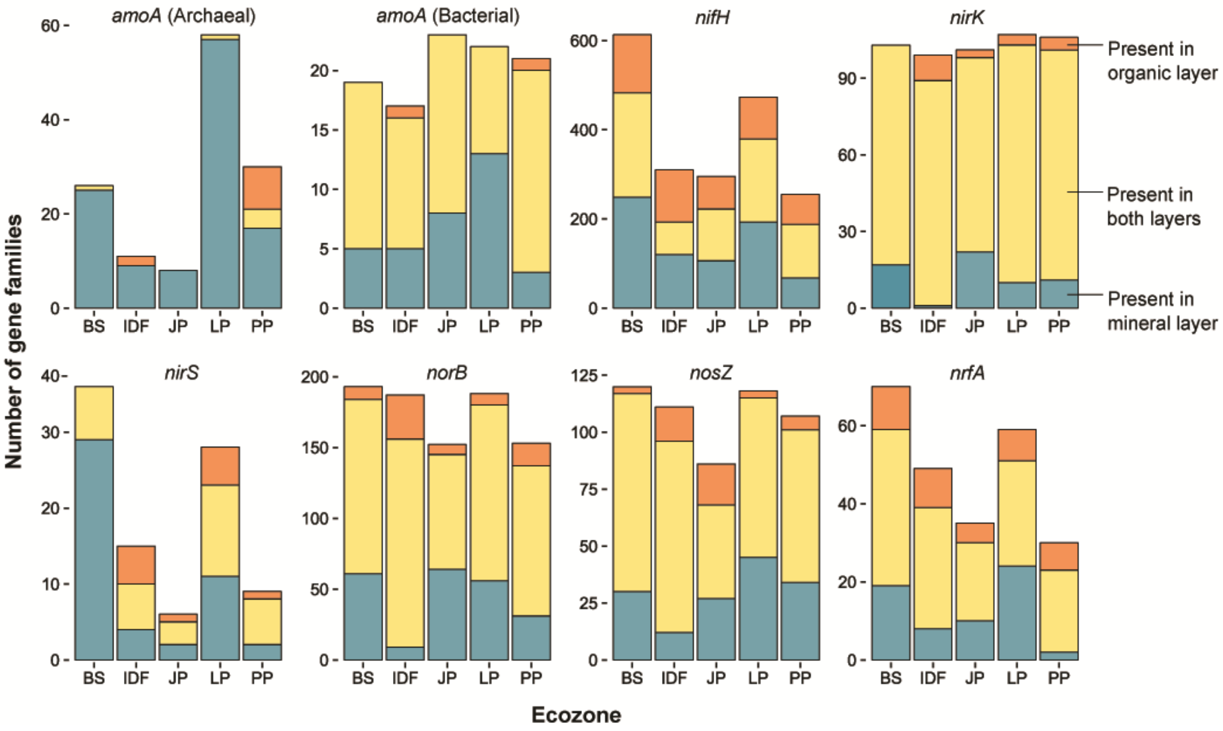

Supplementary figure S2. Permutational multivariate analysis of variation of CAZy and nitrogen cycle gene family profiles. Distribution of explained variation in Global models for CAZy genes (a) and nitrogen cycle genes (b). Ecozone specific models of CAZy (c) and nitrogen cycle genes (d). Global models did not include IDF samples since their sequences were shorter and ecozone and read length effects could not be separated. Only significant effects are shown ($p < 0.05$). Ecozone-specific models tested the harvesting effects for each layer separately, thus these effects are not additive.

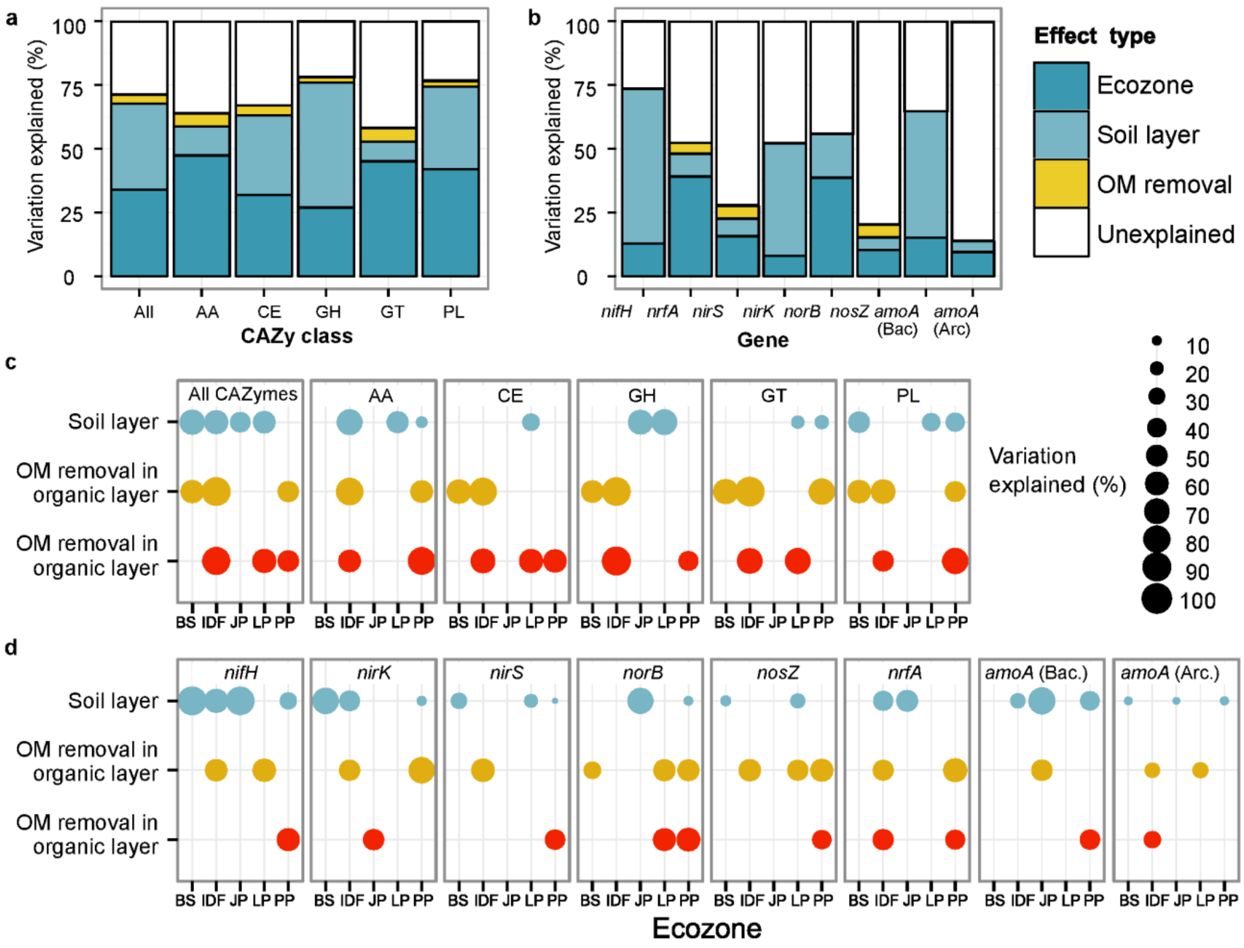

Supplementary figure S3. Analysis of differentially distributed gene families. a. Distribution of predictors of soil layer and OM removal treatments across ecozones. Predictors were identified by random forest analysis with Boruta feature selection. Ecozones: BS, yellow; PP, orange; JP, green; IDF, blue; LP, purple. b. Out of bag estimates of classification errors for CAZy and nitrogen cycle gene families. Estimates are means of ten Random Forests runs with 1000 trees each. Harvesting effects compares unharvested (OM0) versus harvested treatments (OM1, OM2, and OM3), whereas OM removal compares all OM removal treatments (OM0, OM1, OM2, and OM3) against one other.

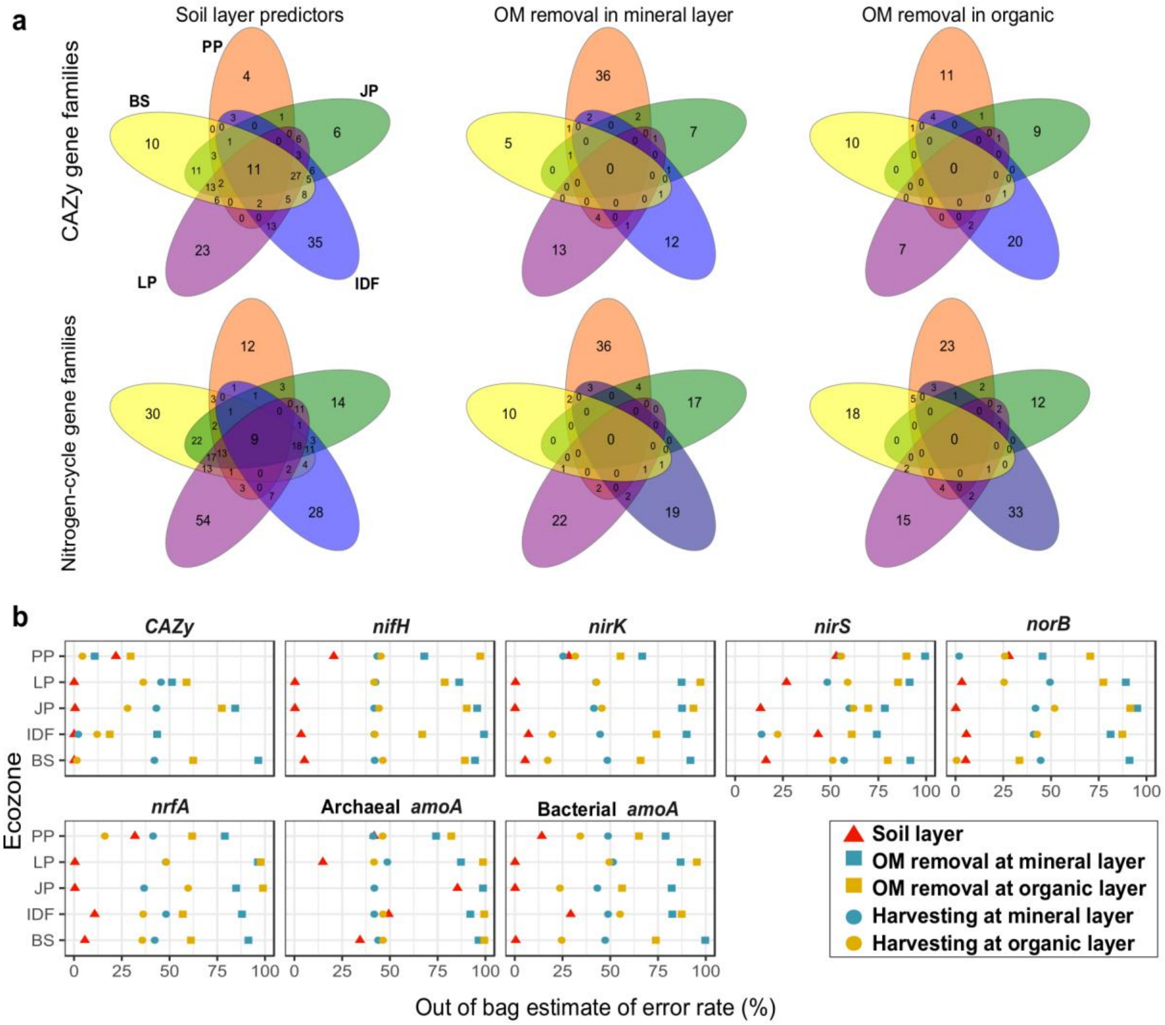

Supplementary figure S4. Taxonomic composition of sequences of the GH12 family across OM removal treatments. For each major taxonomic group (bottom part or circle), we show the contribution to the total GH12 abundance for each harvesting treatment (top part of circle). Values are averages of both soil layers. Data for the OM3 treatment was excluded since it was absent for organic layers of four ecozones. Taxonomic composition changes minimally between soil layers (data not shown).

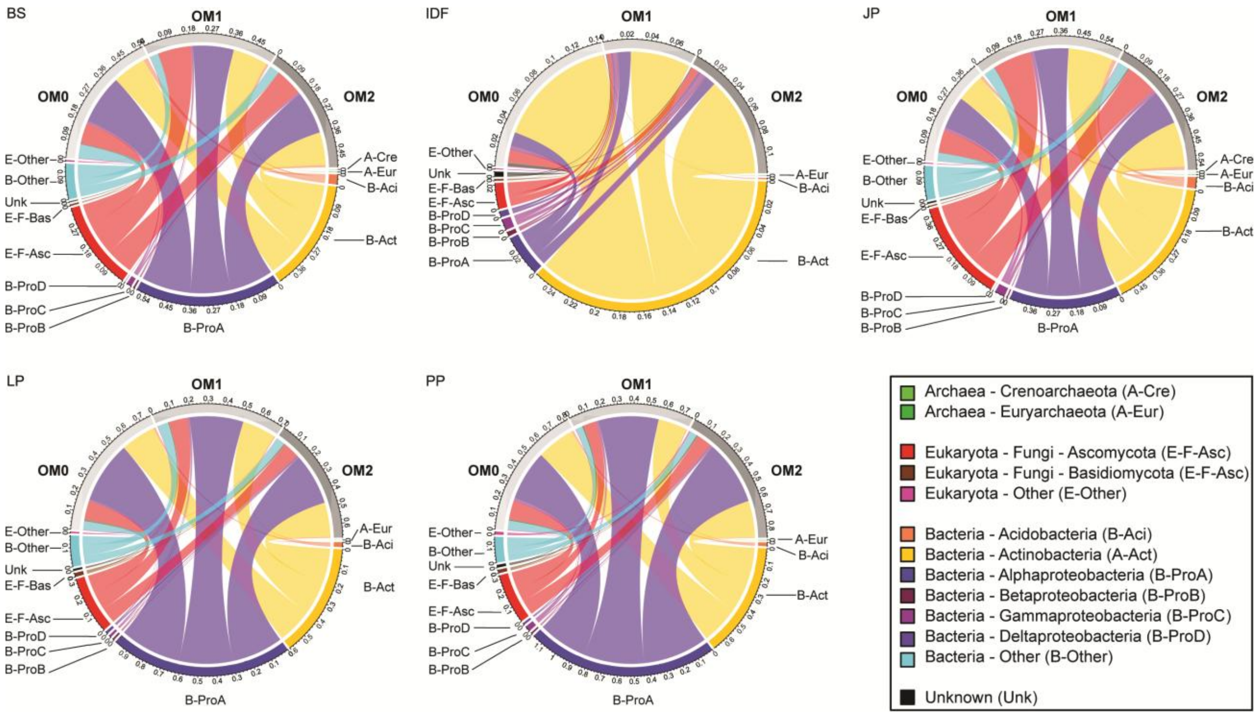

Supplementary figure S5. Correlations between environmental variables and CAZy gene families.

Families which were deemed significant (n = 69, p-value < 0.05, q-value < 0.05) and had an adjusted R^2 > 0.75 are shown. Not shown are interactions with pH (Positive correlation with GH98, negative with CE6).

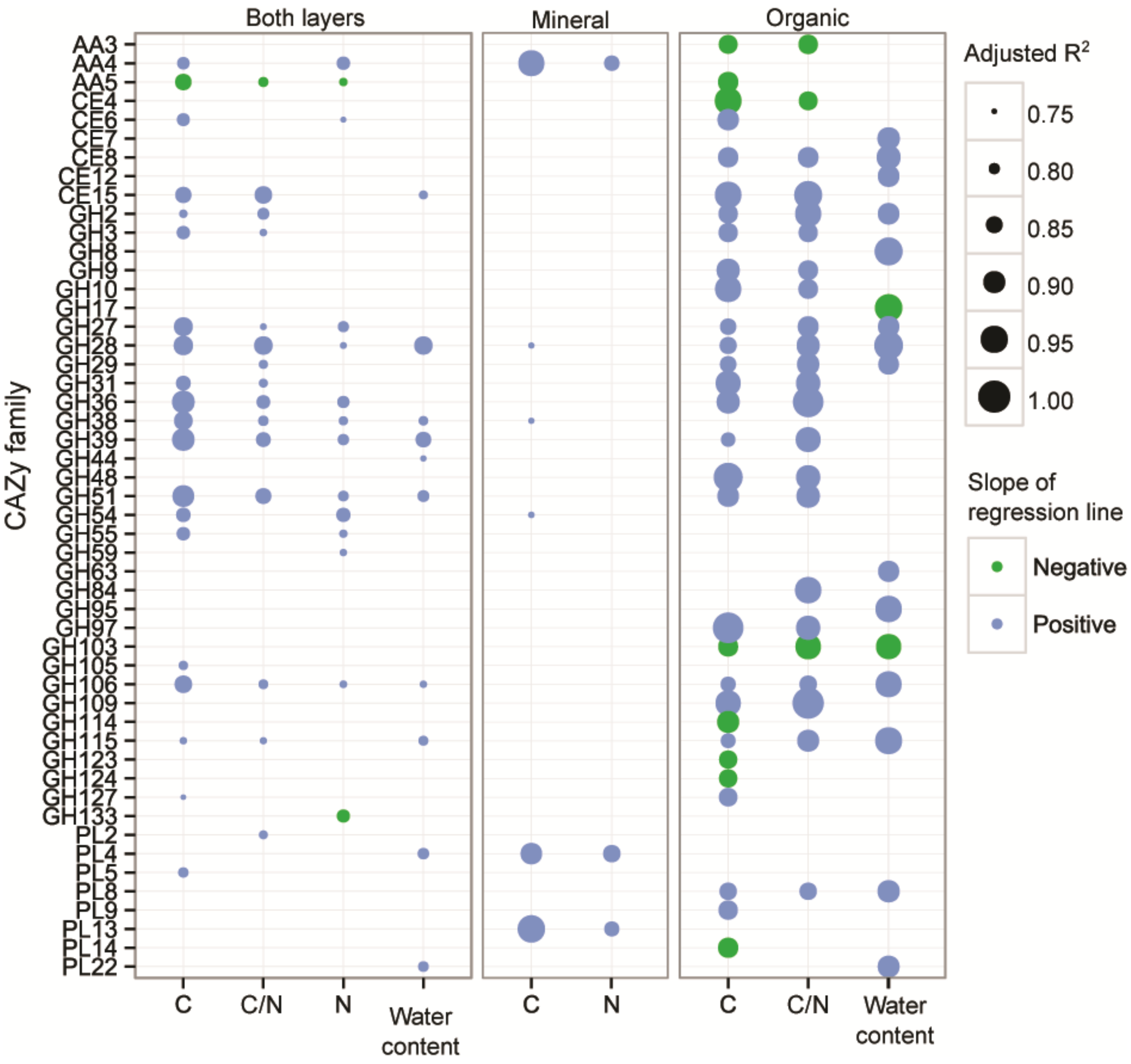

Supplementary figure S6. Relationship between environmental gradients and nitrogen cycle genes.

Nitrogen cycle gene families which were deemed significant (n = 87, p-value < 0.05, q-value < 0.05) and had an adjusted $R^2 > 0.75$ are shown. Not shown are positive correlations found in the mineral layer between *nosZ* family 110 and C and N, and *nifH* family 1562 and C.

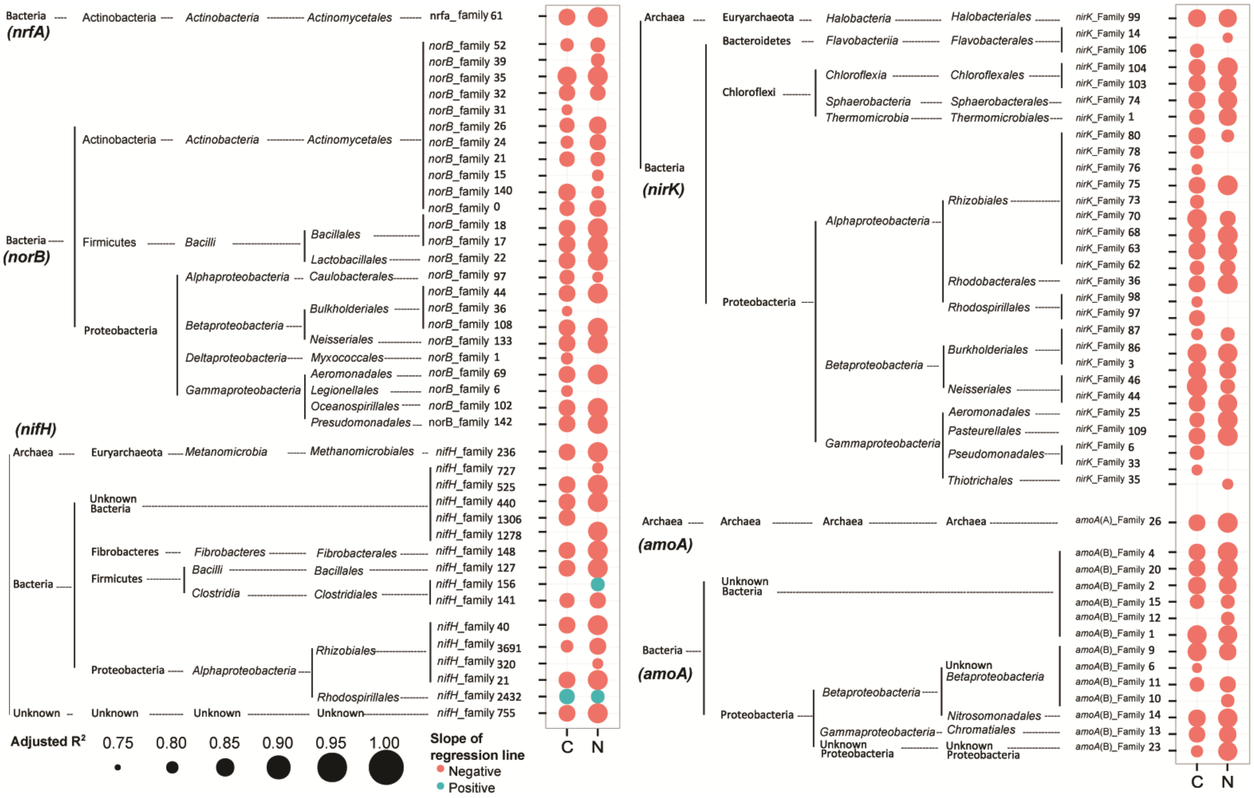

Supplementary Table 1. Site characteristics

Site Name (Site Code)	Ecozone (Ecozone code)	Region, Country	Latitude	Longitude	Elevation (m)
O'Connor Lake (OC)	Interior Douglas Fir (IDF)	British Columbia, Canada	50.88	-120.35	1075
Blodgett (BL)	Ponderosa Pine (PP)	California, USA	38.88	-120.64	1350
Kurth (TXA)	Loblolly Pine (LP)	Texas, USA	31.11	-95.15	88
Fensom (A8)	Black Spruce (BS)	Ontario, Canada	49.08	-89.38	450
Wells (JW)	Jack pine(JP)	Ontario, Canada	46.42	-83.37	228

Site Name (Site Code)	Soil Classification	Year of forest harvesting	Sample collection date	Tree planted after harvesting
O'Connor Lake (OC)	Brunisolic Gray Luvisol	2000	6/26/2010	Lodgepole pine
Blodgett (BL)	Mesic Ultic Haploxeralfs	1994	9/16/2011	Ponderosa pine
Kurth (TXA)	Aquic Glossudalfs	1997	3/12/2012	Lodgepole pine
Fensom (A8)	Orthic Dystric Brunisol	1995	7/4/2011	Black spruce
Wells (JW)	Orthic Humo-Ferric Podzol	1993-1994	7/7/2011	Jack pine

Site Name (Site Code)	Annual Mean Temp (°C)	Annual precipitation (cm)	Precipitation in the warmest quarter (mm)	Climatic Zone
O'Connor Lake (OC)	2.5	30 - 75	300	Dfb, Humid Continental warm summer
Blodgett (BL)	11.2	165.1	55	Csa, Mediterranean hot summer
Kurth (TXA)	19.0	109	253	Cfa, Humid subtropical
Fensom (A8)	1.8	61	266	Dfb, Humid Continental warm summer
Wells (JW)	4.4	87.4	248	Dfb, Humid Continental cool summer

Supplementary table 2

Biochemical characterization of soil samples

For the LP and PP ecozones, measurements of total C and N were only available per plot (not per sample).

Sample	Ecozone	Treatment	Replicate	Soil layer	Harvesting	Water content (%)	DNA (ng/uL)	Total C (%)	Total N (%)	pH	Bulk_density (g/mL)	C/N ratio
A8-OM0C0-M1	BS	OM0	1	Mineral	Unharvested	26	16.9	2.54	0.11	5.2	1.01	23.1
A8-OM0C0-M2	BS	OM0	2	Mineral	Unharvested	27.5	18	3.3	0.12	4.9	0.75	27.26
A8-OM0C0-M3	BS	OM0	3	Mineral	Unharvested	28.8	18.9	1.69	0.09	5.2	0.95	18.37
A8-OM0C0-O1	BS	OM0	1	Organic	Unharvested	73.3	113.3	45.78	1.1	4.08	0.15	41.77
A8-OM0C0-O2	BS	OM0	2	Organic	Unharvested	75.7	78.8	44.34	1.01	4.73	0.1	43.77
A8-OM0C0-O3	BS	OM0	3	Organic	Unharvested	73.2	124.7	45.67	1.05	4.15	0.13	43.49
A8-OM1C0-M1	BS	OM1	1	Mineral	Harvested	22.8	36.7	4.48	0.18	4.68	0.96	24.45
A8-OM1C0-M2	BS	OM1	2	Mineral	Harvested	24	25.6	2.11	0.1	5.28	1.12	21.73
A8-OM1C0-M3	BS	OM1	3	Mineral	Harvested	23.4	9.3	1.66	0.07	5.4	1.32	23.67
A8-OM1C0-O1	BS	OM1	1	Organic	Harvested	53.8	94.5	41.38	1.17	4.58	0.21	35.43
A8-OM1C0-O2	BS	OM1	2	Organic	Harvested	36.2	83.2	41.6	1.22	4.65	0.21	34.21
A8-OM1C0-O3	BS	OM1	3	Organic	Harvested	52.1	119.1	37.31	0.79	4.46	0.24	47.4
A8-OM2C0-M1	BS	OM2	1	Mineral	Harvested	19.5	21	1.33	0.06	4.98	1.31	20.81
A8-OM2C0-M2	BS	OM2	2	Mineral	Harvested	25.5	11	1.58	0.07	5.43	1.34	21.62
A8-OM2C0-M3	BS	OM2	3	Mineral	Harvested	21.1	19	0.9	0.04	5.38	1.7	23.58
A8-OM2C0-O1	BS	OM2	1	Organic	Harvested	45.8	98	42.26	1.08	4.95	0.21	39.05
A8-OM2C0-O2	BS	OM2	2	Organic	Harvested	70	156.1	43.82	1.28	4.77	0.25	34.21
A8-OM2C0-O3	BS	OM2	3	Organic	Harvested	52.5	104.8	41.71	1.18	4.5	0.28	35.47
A8-OM3C0-M1	BS	OM3	1	Mineral	Harvested	13.5	18.1	0.78	0.04	5.6	1.66	21.61
A8-OM3C0-M2	BS	OM3	2	Mineral	Harvested	18.1	19.3	2.05	0.1	5.2	1.58	20.92
A8-OM3C0-M3	BS	OM3	3	Mineral	Harvested	21.5	7.8	1.3	0.06	5.73	1.65	20.34
BL-OM0C0-M1	PP	OM0	1	Mineral	Unharvested	17.8	37.8	6.3	0.31	5.48	NA	20.32
BL-OM0C0-M2	PP	OM0	2	Mineral	Unharvested	17.5	34.4	6.3	0.31	5.49	NA	20.32
BL-OM0C0-M3	PP	OM0	3	Mineral	Unharvested	15.3	32.8	6.3	0.31	5.99	NA	20.32
BL-OM0C0-O1	PP	OM0	1	Organic	Unharvested	27	79.7	NA	NA	4.51	NA	NA
BL-OM0C0-O2	PP	OM0	2	Organic	Unharvested	25.8	58.1	NA	NA	5.28	NA	NA
BL-OM0C0-O3	PP	OM0	3	Organic	Unharvested	29.8	60.8	NA	NA	4.9	NA	NA
BL-OM1C0-M1	PP	OM1	1	Mineral	Harvested	22	36.2	5.47	0.28	5.74	NA	19.78

BL-OM1C0-M2	PP	OM1	2	Mineral	Harvested	21	39.2	5.47	0.28	5.87	NA	19.78
BL-OM1C0-M3	PP	OM1	3	Mineral	Harvested	23.7	44.8	5.47	0.28	5.89	NA	19.78
BL-OM1C0-O1	PP	OM1	1	Organic	Harvested	18.4	49.2	NA	NA	4.79	NA	NA
BL-OM1C0-O2	PP	OM1	2	Organic	Harvested	16.7	26	NA	NA	4.9	NA	NA
BL-OM1C0-O3	PP	OM1	3	Organic	Harvested	18.9	63.3	NA	NA	4.69	NA	NA
BL-OM2C0-M1	PP	OM2	1	Mineral	Harvested	21	38.9	5.35	0.26	5.78	NA	20.64
BL-OM2C0-M2	PP	OM2	2	Mineral	Harvested	20.5	35.9	5.35	0.26	5.3	NA	20.64
BL-OM2C0-M3	PP	OM2	3	Mineral	Harvested	22.3	43.5	5.35	0.26	5.72	NA	20.64
BL-OM2C0-O1	PP	OM2	1	Organic	Harvested	28.6	52.8	NA	NA	5.73	NA	NA
BL-OM2C0-O2	PP	OM2	2	Organic	Harvested	21.7	20.7	NA	NA	5.24	NA	NA
BL-OM2C0-O3	PP	OM2	3	Organic	Harvested	16.4	46.2	NA	NA	5.09	NA	NA
BL-OM3C0-M1	PP	OM3	1	Mineral	Harvested	21.7	36.7	5.27	0.28	5.42	NA	18.87
BL-OM3C0-M2	PP	OM3	2	Mineral	Harvested	22.2	37.9	5.27	0.28	5.86	NA	18.87
BL-OM3C0-M3	PP	OM3	3	Mineral	Harvested	21.5	30.8	5.27	0.28	5.39	NA	18.87
JW-OM0C0-M1	JP	OM0	1	Mineral	Unharvested	16	22.8	1.52	0.12	5.48	8.9	15.63
JW-OM0C0-M2	JP	OM0	2	Mineral	Unharvested	12	16.2	2.56	0.17	5.42	9.75	16.24
JW-OM0C0-M3	JP	OM0	3	Mineral	Unharvested	14	21.5	3.13	0.22	4.08	7.65	14.81
JW-OM0C0-O1	JP	OM0	1	Organic	Unharvested	31	44.7	38.13	1.06	4.1	8.9	36.55
JW-OM0C0-O2	JP	OM0	2	Organic	Unharvested	31.4	25.6	36.85	1.2	4.2	9.75	30.79
JW-OM0C0-O3	JP	OM0	3	Organic	Unharvested	40.4	15.5	34.55	1.14	4.08	7.85	30.33
JW-OM1C0-M1	JP	OM1	1	Mineral	Harvested	9.8	26.3	2.71	0.17	5.25	6.34	16
JW-OM1C0-M2	JP	OM1	2	Mineral	Harvested	12.2	28.9	3.13	0.22	5.34	7.65	14.81
JW-OM1C0-M3	JP	OM1	3	Mineral	Harvested	13.7	26.9	3.26	0.29	5.31	6.34	13.57
JW-OM1C0-O1	JP	OM1	1	Organic	Harvested	40	72.6	36.42	1.11	4.18	6.34	32.96
JW-OM1C0-O2	JP	OM1	2	Organic	Harvested	46.7	56.3	34.55	1.14	4.08	7.65	30.07
JW-OM1C0-O3	JP	OM1	3	Organic	Harvested	57	66.9	33.83	1.03	4.41	6.34	32.82
JW-OM2C0-M1	JP	OM2	1	Mineral	Harvested	14.7	36.5	2.56	0.17	5.42	9.75	16.24
JW-OM2C0-M2	JP	OM2	2	Mineral	Harvested	16.3	18.9	3.03	0.35	5.13	7.65	13.94
JW-OM2C0-M3	JP	OM2	3	Mineral	Harvested	12.9	29.2	2.66	0.48	5.35	9.73	11.11
JW-OM2C0-O1	JP	OM2	1	Organic	Harvested	46.7	56.2	36.85	1.2	4.2	9.75	30.79
JW-OM2C0-O2	JP	OM2	2	Organic	Harvested	60.4	46.9	37.62	1.12	4	7.65	33.6
JW-OM2C0-O3	JP	OM2	3	Organic	Harvested	47.5	57.1	35.24	1.09	4.27	9.73	32.47
JW-OM3C0-M1	JP	OM3	1	Mineral	Harvested	11	23.3	2.15	0.12	5.39	7.21	17.82
JW-OM3C0-M2	JP	OM3	2	Mineral	Harvested	13.3	9.2	1.52	0.12	5.48	8.9	15.63

JW-OM3C0-M3	JP	OM3	3	Mineral	Harvested	14.3	10	1.29	0.56	5.39	8.97	9.84
OC-OM0C0-M1	IDF	OM0	1	Mineral	Unharvested	12	15.8	1.81	0.1	4.98	NA	18.1
OC-OM0C0-M2	IDF	OM0	2	Mineral	Unharvested	13	15.8	1.81	0.1	5.02	NA	18.1
OC-OM0C0-M3	IDF	OM0	3	Mineral	Unharvested	19	14.8	1.81	0.1	5.01	NA	18.1
OC-OM0C0-O1	IDF	OM0	1	Organic	Unharvested	40	82.6	44.18	1.39	5.04	NA	31.78
OC-OM0C0-O2	IDF	OM0	2	Organic	Unharvested	46	74.7	44.18	1.39	5.34	NA	31.78
OC-OM0C0-O3	IDF	OM0	3	Organic	Unharvested	54	96.3	44.18	1.39	4.87	NA	31.78
OC-OM1C0-M1	IDF	OM1	1	Mineral	Harvested	20	17	1.79	0.11	5.16	1.17	16.27
OC-OM1C0-M2	IDF	OM1	2	Mineral	Harvested	20	15.3	1.79	0.11	5.21	1.17	16.27
OC-OM1C0-M3	IDF	OM1	3	Mineral	Harvested	22	18.8	1.79	0.11	5.01	1.17	16.27
OC-OM1C0-O1	IDF	OM1	1	Organic	Harvested	57	74.7	33.83	1.41	5.36	1.17	23.99
OC-OM1C0-O2	IDF	OM1	2	Organic	Harvested	57	69.2	33.83	1.41	4.8	1.17	23.99
OC-OM1C0-O3	IDF	OM1	3	Organic	Harvested	61	80.1	33.83	1.41	5.34	1.17	23.99
OC-OM2C0-M1	IDF	OM2	1	Mineral	Harvested	20	12.5	1.87	0.1	5.02	1.3	18.7
OC-OM2C0-M2	IDF	OM2	2	Mineral	Harvested	21	19.8	1.87	0.1	5.34	1.3	18.7
OC-OM2C0-M3	IDF	OM2	3	Mineral	Harvested	21	9.6	1.87	0.1	5.01	1.3	18.7
OC-OM2C0-O1	IDF	OM2	1	Organic	Harvested	60	20.3	33.06	1.25	5.07	1.3	26.45
OC-OM2C0-O2	IDF	OM2	2	Organic	Harvested	59	19.3	33.06	1.25	5.6	1.3	26.45
OC-OM2C0-O3	IDF	OM2	3	Organic	Harvested	60	30.3	33.06	1.25	5.46	1.3	26.45
OC-OM3C0-M1	IDF	OM3	1	Mineral	Harvested	23	20	1.82	0.1	5.16	1.35	18.2
OC-OM3C0-M2	IDF	OM3	2	Mineral	Harvested	21	7.1	1.82	0.1	5.21	1.35	18.2
OC-OM3C0-M3	IDF	OM3	3	Mineral	Harvested	16	12.2	1.82	0.1	5.27	1.35	18.2
TXA-OM0C0-M1	LP	OM0	1	Mineral	Unharvested	10.4	6	NA	NA	NA	NA	NA
TXA-OM0C0-M2	LP	OM0	2	Mineral	Unharvested	10.8	7	NA	NA	NA	NA	NA
TXA-OM0C0-M3	LP	OM0	3	Mineral	Unharvested	10.9	9	NA	NA	NA	NA	NA
TXA-OM0C0-O1	LP	OM0	1	Organic	Unharvested	18.9	31	NA	NA	4.69	NA	NA
TXA-OM0C0-O2	LP	OM0	2	Organic	Unharvested	33.8	55	NA	NA	4.4	NA	NA
TXA-OM0C0-O3	LP	OM0	3	Organic	Unharvested	26.3	36	NA	NA	4.25	NA	NA
TXA-OM1C0-M1	LP	OM1	1	Mineral	Harvested	12.2	12	1.09	0.05	4.95	1.27	18.48
TXA-OM1C0-M2	LP	OM1	2	Mineral	Harvested	13.9	16	1.09	0.05	4.65	1.27	18.48
TXA-OM1C0-M3	LP	OM1	3	Mineral	Harvested	13.8	7	1.09	0.05	4.56	1.27	18.48
TXA-OM1C0-O1	LP	OM1	1	Organic	Harvested	25.5	53	NA	NA	4.69	NA	NA
TXA-OM1C0-O2	LP	OM1	2	Organic	Harvested	27.8	56	NA	NA	4.69	NA	NA
TXA-OM1C0-O3	LP	OM1	3	Organic	Harvested	19	48	NA	NA	5.04	NA	NA

TXA-OM2C0-M1	LP	OM2	1	Mineral	Harvested	10.5	6	1.21	0.05	4.56	1.26	23.88
TXA-OM2C0-M2	LP	OM2	2	Mineral	Harvested	9.9	9	1.21	0.05	5.04	1.26	23.88
TXA-OM2C0-O1	LP	OM2	1	Organic	Harvested	15	49	NA	NA	4.49	NA	NA
TXA-OM2C0-O2	LP	OM2	2	Organic	Harvested	23.3	48	NA	NA	5.74	NA	NA
TXA-OM2C0-O3	LP	OM2	3	Organic	Harvested	13.5	40	NA	NA	5.28	NA	NA
TXA-OM3C0-M1	LP	OM3	1	Mineral	Harvested	8.9	8	0.79	0.04	4.58	1.32	17.79
TXA-OM3C0-M2	LP	OM3	2	Mineral	Harvested	8.6	7	0.79	0.04	4.53	1.32	17.79
TXA-OM3C0-M3	LP	OM3	3	Mineral	Harvested	9.5	4	0.79	0.04	4.4	1.32	17.79
TXA-OM3C0-O1	LP	OM3	1	Organic	Harvested	10.6	17	NA	NA	4.68	NA	NA
TXA-OM3C0-O2	LP	OM3	2	Organic	Harvested	9.8	26	NA	NA	4.67	NA	NA
TXA-OM3C0-O3	LP	OM3	3	Organic	Harvested	19	30	NA	NA	4.42	NA	NA

Supplementary table 4

Differences in gene family abundance profiles among harvesting treatments using PERMANOVA

* : p < 0.05, ** : p < 0.01 *** : p < 0.001.

Gene grou|	Ecozone	Contrast					
		OM0 vs. OM1	OM0 vs. OM2	OM0 vs. OM3	OM1 vs. OM2	OM1 vs. OM3	OM2 vs. OM3
CAZy	All	**	**	***		***	
CAZy	BS	**	**				
CAZy	IDF	***	**	**	**		
CAZy	JP						
CAZy	LP	**					**
CAZy	PP	***		**		**	
N cycle	All	**	**	***		***	
N cycle	BS	*					
N cycle	IDF	**	**	**	**		
N cycle	JP						
N cycle	LP	**		**		***	
N cycle	PP	**	**				

ANOVA tests for CAZy genes

Soil layer: M=mineral, O=Organic

Number of replicated for each groups being compared is included in parenthesis

Test	Statistical test	Ezozone	Layer	CAZy class	Group 1	Group 2	Group 3	Group 4	F value	p-value	Posthoc Tukey test result (p<0.05)
Differences in gene abundances	ANOVA	BS	M	All	OM0 (3)	OM1 (3)	OM2 (3)	OM3 (3)	0.425	0.741	NA
Differences in gene abundances	ANOVA	BS	O	All	OM0 (3)	OM1 (3)	OM2 (3)	NA	4.496	0.0641	NA
Differences in gene abundances	ANOVA	BS	M	AA	OM0 (3)	OM1 (3)	OM2 (3)	OM3 (3)	0.616	0.624	NA
Differences in gene abundances	ANOVA	BS	O	AA	OM0 (3)	OM1 (3)	OM2 (3)	NA	1.186	0.368	NA
Differences in gene abundances	ANOVA	BS	M	CE	OM0 (3)	OM1 (3)	OM2 (3)	OM3 (3)	0.317	0.813	NA
Differences in gene abundances	ANOVA	BS	O	CE	OM0 (3)	OM1 (3)	OM2 (3)	NA	4.862	0.0556	NA
Differences in gene abundances	ANOVA	BS	M	GH	OM0 (3)	OM1 (3)	OM2 (3)	OM3 (3)	0.094	0.961	NA
Differences in gene abundances	ANOVA	BS	O	GH	OM0 (3)	OM1 (3)	OM2 (3)	NA	13.61	0.00589	OM0>OM1, OM0>OM2
Differences in gene abundances	ANOVA	BS	M	GT	OM0 (3)	OM1 (3)	OM2 (3)	OM3 (3)	2.393	0.144	NA
Differences in gene abundances	ANOVA	BS	O	GT	OM0 (3)	OM1 (3)	OM2 (3)	NA	0.343	0.723	NA
Differences in gene abundances	ANOVA	BS	M	PL	OM0 (3)	OM1 (3)	OM2 (3)	OM3 (3)	0.796	0.53	NA
Differences in gene abundances	ANOVA	BS	O	PL	OM0 (3)	OM1 (3)	OM2 (3)	NA	2.01	0.215	NA
Differences in gene abundances	ANOVA	PP	M	All	OM0 (3)	OM1 (3)	OM2 (3)	OM3 (3)	2.057	0.184	NA
Differences in gene abundances	ANOVA	PP	O	All	OM0 (3)	OM1 (3)	OM2 (3)	NA	1.687	0.262	NA
Differences in gene abundances	ANOVA	PP	M	AA	OM0 (3)	OM1 (3)	OM2 (3)	OM3 (3)	18.01	0.000645	OM0>OM1, OM0>OM2, OM1<OM3
Differences in gene abundances	ANOVA	PP	O	AA	OM0 (3)	OM1 (3)	OM2 (3)	NA	3.978	0.0795	NA
Differences in gene abundances	ANOVA	PP	M	CE	OM0 (3)	OM1 (3)	OM2 (3)	OM3 (3)	0.103	0.956	NA
Differences in gene abundances	ANOVA	PP	O	CE	OM0 (3)	OM1 (3)	OM2 (3)	NA	0.669	0.546	NA
Differences in gene abundances	ANOVA	PP	M	GH	OM0 (3)	OM1 (3)	OM2 (3)	OM3 (3)	2.054	0.185	NA
Differences in gene abundances	ANOVA	PP	O	GH	OM0 (3)	OM1 (3)	OM2 (3)	NA	1.015	0.417	NA
Differences in gene abundances	ANOVA	PP	M	GT	OM0 (3)	OM1 (3)	OM2 (3)	OM3 (3)	0.848	0.505	NA
Differences in gene abundances	ANOVA	PP	O	GT	OM0 (3)	OM1 (3)	OM2 (3)	NA	0.485	0.638	NA
Differences in gene abundances	ANOVA	PP	M	PL	OM0 (3)	OM1 (3)	OM2 (3)	OM3 (3)	37.5	4.65E-05	OM0<OM1, OM0<OM2, OM1>OM3, OM2>OM3
Differences in gene abundances	ANOVA	PP	O	PL	OM0 (3)	OM1 (3)	OM2 (3)	NA	0.37	0.706	NA
Differences in gene abundances	ANOVA	JP	M	All	OM0 (3)	OM1 (3)	OM2 (3)	OM3 (3)	0.055	0.982	NA
Differences in gene abundances	ANOVA	JP	O	All	OM0 (3)	OM1 (3)	OM2 (3)	NA	1.069	0.401	NA
Differences in gene abundances	ANOVA	JP	M	AA	OM0 (3)	OM1 (3)	OM2 (3)	OM3 (3)	0.894	0.485	NA
Differences in gene abundances	ANOVA	JP	O	AA	OM0 (3)	OM1 (3)	OM2 (3)	NA	0.43	0.669	NA
Differences in gene abundances	ANOVA	JP	M	CE	OM0 (3)	OM1 (3)	OM2 (3)	OM3 (3)	0.182	0.906	NA
Differences in gene abundances	ANOVA	JP	O	CE	OM0 (3)	OM1 (3)	OM2 (3)	NA	0.569	0.594	NA
Differences in gene abundances	ANOVA	JP	M	GH	OM0 (3)	OM1 (3)	OM2 (3)	OM3 (3)	0.289	0.832	NA
Differences in gene abundances	ANOVA	JP	O	GH	OM0 (3)	OM1 (3)	OM2 (3)	NA	2.121	0.201	NA
Differences in gene abundances	ANOVA	JP	M	GT	OM0 (3)	OM1 (3)	OM2 (3)	OM3 (3)	0.07	0.974	NA
Differences in gene abundances	ANOVA	JP	O	GT	OM0 (3)	OM1 (3)	OM2 (3)	NA	0.652	0.554	NA
Differences in gene abundances	ANOVA	JP	M	PL	OM0 (3)	OM1 (3)	OM2 (3)	OM3 (3)	1.483	0.291	NA
Differences in gene abundances	ANOVA	JP	O	PL	OM0 (3)	OM1 (3)	OM2 (3)	NA	2.24	0.188	NA
Differences in gene abundances	ANOVA	IDF	M	All	OM0 (3)	OM1 (3)	OM2 (3)	OM3 (3)	16.25	0.000915	OM0>OM1, OM0>OM2, OM1>OM2
Differences in gene abundances	ANOVA	IDF	O	All	OM0 (3)	OM1 (3)	OM2 (3)	NA	31.9	0.000635	OM0>OM1, OM0>OM2, OM1<OM2
Differences in gene abundances	ANOVA	IDF	M	AA	OM0 (3)	OM1 (3)	OM2 (3)	OM3 (3)	5.447	0.0246	OM0>OM2

Differences in gene abundances	ANOVA	IDF	O	AA	OM0 (3)	OM1 (3)	OM2 (3)	NA	20.79	0.00201	OM0>OM1, OM0>OM2
Differences in gene abundances	ANOVA	IDF	M	CE	OM0 (3)	OM1 (3)	OM2 (3)	OM3 (3)	8.568	0.00703	OM0>OM2, OM0>OM3
Differences in gene abundances	ANOVA	IDF	O	CE	OM0 (3)	OM1 (3)	OM2 (3)	NA	26.09	0.0011	OM0>OM1, OM0>OM2, OM1<OM2
Differences in gene abundances	ANOVA	IDF	M	GH	OM0 (3)	OM1 (3)	OM2 (3)	OM3 (3)	24.15	0.000231	OM0>OM1, OM0>OM2, OM0>OM3, OM1>OM2
Differences in gene abundances	ANOVA	IDF	O	GH	OM0 (3)	OM1 (3)	OM2 (3)	NA	17.99	0.00292	OM0>OM1, OM1<OM2
Differences in gene abundances	ANOVA	IDF	M	GT	OM0 (3)	OM1 (3)	OM2 (3)	OM3 (3)	9.752	0.00476	OM0>OM2, OM0>OM3, OM1>OM2
Differences in gene abundances	ANOVA	IDF	O	GT	OM0 (3)	OM1 (3)	OM2 (3)	NA	59.27	0.00011	OM0>OM1, OM0>OM2, OM1<OM2
Differences in gene abundances	ANOVA	IDF	M	PL	OM0 (3)	OM1 (3)	OM2 (3)	OM3 (3)	5.387	0.0254	OM0<OM3
Differences in gene abundances	ANOVA	IDF	O	PL	OM0 (3)	OM1 (3)	OM2 (3)	NA	8.463	0.0179	OM1<OM2
Differences in gene abundances	ANOVA	LP	M	All	OM0 (3)	OM1 (3)	OM2 (3)	OM3 (2)	11.95	0.00384	OM0<OM3,OM1<OM3
Differences in gene abundances	ANOVA	LP	O	All	OM0 (3)	OM1 (3)	OM2 (3)	OM3 (3)	0.887	0.488	NA
Differences in gene abundances	ANOVA	LP	M	AA	OM0 (3)	OM1 (3)	OM2 (3)	OM3 (2)	1.375	0.327	NA
Differences in gene abundances	ANOVA	LP	O	AA	OM0 (3)	OM1 (3)	OM2 (3)	OM3 (3)	0.956	0.459	NA
Differences in gene abundances	ANOVA	LP	M	CE	OM0 (3)	OM1 (3)	OM2 (3)	OM3 (2)	8.097	0.0112	OM1<OM3
Differences in gene abundances	ANOVA	LP	O	CE	OM0 (3)	OM1 (3)	OM2 (3)	OM3 (3)	0.248	0.861	NA
Differences in gene abundances	ANOVA	LP	M	GH	OM0 (3)	OM1 (3)	OM2 (3)	OM3 (2)	5.457	0.03	OM1<OM3
Differences in gene abundances	ANOVA	LP	O	GH	OM0 (3)	OM1 (3)	OM2 (3)	OM3 (3)	1.247	0.355	NA
Differences in gene abundances	ANOVA	LP	M	GT	OM0 (3)	OM1 (3)	OM2 (3)	OM3 (2)	9.555	0.00718	OM1<OM3
Differences in gene abundances	ANOVA	LP	O	GT	OM0 (3)	OM1 (3)	OM2 (3)	OM3 (3)	0.785	0.535	NA
Differences in gene abundances	ANOVA	LP	M	PL	OM0 (3)	OM1 (3)	OM2 (3)	OM3 (2)	0.855	0.507	NA
Differences in gene abundances	ANOVA	LP	O	PL	OM0 (3)	OM1 (3)	OM2 (3)	OM3 (3)	1.004	0.44	NA
Differences in gene richness	ANOVA	BS	M	All	OM0 (3)	OM1 (3)	OM2 (3)	OM3 (3)	0.416	0.746	NA
Differences in gene richness	ANOVA	BS	O	All	OM0 (3)	OM1 (3)	OM2 (3)	NA	1.144	0.38	NA
Differences in gene richness	ANOVA	BS	M	AA	OM0 (3)	OM1 (3)	OM2 (3)	OM3 (3)	0.546	0.665	NA
Differences in gene richness	ANOVA	BS	O	AA	OM0 (3)	OM1 (3)	OM2 (3)	NA	4.592	0.0617	NA
Differences in gene richness	ANOVA	BS	M	CE	OM0 (3)	OM1 (3)	OM2 (3)	OM3 (3)	0.624	0.62	NA
Differences in gene richness	ANOVA	BS	O	CE	OM0 (3)	OM1 (3)	OM2 (3)	NA	0.908	0.453	NA
Differences in gene richness	ANOVA	BS	M	GH	OM0 (3)	OM1 (3)	OM2 (3)	OM3 (3)	0.343	0.795	NA
Differences in gene richness	ANOVA	BS	O	GH	OM0 (3)	OM1 (3)	OM2 (3)	NA	1.187	0.368	NA
Differences in gene richness	ANOVA	BS	M	GT	OM0 (3)	OM1 (3)	OM2 (3)	OM3 (3)	0.52	0.68	NA
Differences in gene richness	ANOVA	BS	O	GT	OM0 (3)	OM1 (3)	OM2 (3)	NA	1.071	0.4	NA
Differences in gene richness	ANOVA	BS	M	PL	OM0 (3)	OM1 (3)	OM2 (3)	OM3 (3)	0.161	0.92	NA
Differences in gene richness	ANOVA	BS	O	PL	OM0 (3)	OM1 (3)	OM2 (3)	NA	0.115	0.893	NA
Differences in gene richness	ANOVA	PP	M	All	OM0 (3)	OM1 (3)	OM2 (3)	OM3 (3)	0.528	0.675	NA
Differences in gene richness	ANOVA	PP	O	All	OM0 (3)	OM1 (3)	OM2 (3)	NA	3.004	0.125	NA
Differences in gene richness	ANOVA	PP	M	AA	OM0 (3)	OM1 (3)	OM2 (3)	OM3 (3)	0.842	0.508	NA
Differences in gene richness	ANOVA	PP	O	AA	OM0 (3)	OM1 (3)	OM2 (3)	NA	5.025	0.0523	NA
Differences in gene richness	ANOVA	PP	M	CE	OM0 (3)	OM1 (3)	OM2 (3)	OM3 (3)	0.403	0.755	NA
Differences in gene richness	ANOVA	PP	O	CE	OM0 (3)	OM1 (3)	OM2 (3)	NA	2.056	0.209	NA
Differences in gene richness	ANOVA	PP	M	GH	OM0 (3)	OM1 (3)	OM2 (3)	OM3 (3)	0.705	0.575	NA
Differences in gene richness	ANOVA	PP	O	GH	OM0 (3)	OM1 (3)	OM2 (3)	NA	3.73	0.0886	NA
Differences in gene richness	ANOVA	PP	M	GT	OM0 (3)	OM1 (3)	OM2 (3)	OM3 (3)	0.402	0.756	NA
Differences in gene richness	ANOVA	PP	O	GT	OM0 (3)	OM1 (3)	OM2 (3)	NA	2.298	0.182	NA
Differences in gene richness	ANOVA	PP	M	PL	OM0 (3)	OM1 (3)	OM2 (3)	OM3 (3)	1.916	0.205	NA
Differences in gene richness	ANOVA	PP	O	PL	OM0 (3)	OM1 (3)	OM2 (3)	NA	1.596	0.278	NA
Differences in gene richness	ANOVA	JP	M	All	OM0 (3)	OM1 (3)	OM2 (3)	OM3 (3)	1.05	0.422	NA

Differences in gene richness	ANOVA	JP	O	All	OM0 (3)	OM1 (3)	OM2 (3)	NA	0.89	0.459	NA
Differences in gene richness	ANOVA	JP	M	AA	OM0 (3)	OM1 (3)	OM2 (3)	OM3 (3)	0.913	0.477	NA
Differences in gene richness	ANOVA	JP	O	AA	OM0 (3)	OM1 (3)	OM2 (3)	NA	0.832	0.48	NA
Differences in gene richness	ANOVA	JP	M	CE	OM0 (3)	OM1 (3)	OM2 (3)	OM3 (3)	1.058	0.419	NA
Differences in gene richness	ANOVA	JP	O	CE	OM0 (3)	OM1 (3)	OM2 (3)	NA	0.98	0.428	NA
Differences in gene richness	ANOVA	JP	M	GH	OM0 (3)	OM1 (3)	OM2 (3)	OM3 (3)	0.981	0.449	NA
Differences in gene richness	ANOVA	JP	O	GH	OM0 (3)	OM1 (3)	OM2 (3)	NA	0.94	0.441	NA
Differences in gene richness	ANOVA	JP	M	GT	OM0 (3)	OM1 (3)	OM2 (3)	OM3 (3)	1.142	0.389	NA
Differences in gene richness	ANOVA	JP	O	GT	OM0 (3)	OM1 (3)	OM2 (3)	NA	0.806	0.49	NA
Differences in gene richness	ANOVA	JP	M	PL	OM0 (3)	OM1 (3)	OM2 (3)	OM3 (3)	1.028	0.43	NA
Differences in gene richness	ANOVA	JP	O	PL	OM0 (3)	OM1 (3)	OM2 (3)	NA	1.375	0.322	NA
Differences in gene richness	ANOVA	IDF	M	All	OM0 (3)	OM1 (3)	OM2 (3)	OM3 (3)	0.596	0.635	NA
Differences in gene richness	ANOVA	IDF	O	All	OM0 (3)	OM1 (3)	OM2 (3)	NA	5.048	0.0518	NA
Differences in gene richness	ANOVA	IDF	M	AA	OM0 (3)	OM1 (3)	OM2 (3)	OM3 (3)	0.136	0.936	NA
Differences in gene richness	ANOVA	IDF	O	AA	OM0 (3)	OM1 (3)	OM2 (3)	NA	5.358	0.0462	OM0>OM1
Differences in gene richness	ANOVA	IDF	M	CE	OM0 (3)	OM1 (3)	OM2 (3)	OM3 (3)	0.594	0.636	
Differences in gene richness	ANOVA	IDF	O	CE	OM0 (3)	OM1 (3)	OM2 (3)	NA	8.191	0.0193	OM0>OM1
Differences in gene richness	ANOVA	IDF	M	GH	OM0 (3)	OM1 (3)	OM2 (3)	OM3 (3)	0.382	0.769	
Differences in gene richness	ANOVA	IDF	O	GH	OM0 (3)	OM1 (3)	OM2 (3)	NA	3.696	0.0899	
Differences in gene richness	ANOVA	IDF	M	GT	OM0 (3)	OM1 (3)	OM2 (3)	OM3 (3)	1.331	0.331	
Differences in gene richness	ANOVA	IDF	O	GT	OM0 (3)	OM1 (3)	OM2 (3)	NA	7.028	0.0268	OM0>OM1
Differences in gene richness	ANOVA	IDF	M	PL	OM0 (3)	OM1 (3)	OM2 (3)	OM3 (3)	1.681	0.247	
Differences in gene richness	ANOVA	IDF	O	PL	OM0 (3)	OM1 (3)	OM2 (3)	NA	2.975	0.127	
Differences in gene richness	ANOVA	LP	M	All	OM0 (3)	OM1 (3)	OM2 (3)	OM3 (2)	5.921	0.0247	OM1>OM3
Differences in gene richness	ANOVA	LP	O	All	OM0 (3)	OM1 (3)	OM2 (3)	OM3 (3)	1.498	0.287	
Differences in gene richness	ANOVA	LP	M	AA	OM0 (3)	OM1 (3)	OM2 (3)	OM3 (2)	5.43	0.0303	OM1>OM3
Differences in gene richness	ANOVA	LP	O	AA	OM0 (3)	OM1 (3)	OM2 (3)	OM3 (3)	1.273	0.348	
Differences in gene richness	ANOVA	LP	M	CE	OM0 (3)	OM1 (3)	OM2 (3)	OM3 (2)	5.749	0.0265	OM1>OM3
Differences in gene richness	ANOVA	LP	O	CE	OM0 (3)	OM1 (3)	OM2 (3)	OM3 (3)	1.489	0.29	
Differences in gene richness	ANOVA	LP	M	GH	OM0 (3)	OM1 (3)	OM2 (3)	OM3 (2)	6.37	0.0207	OM1>OM3
Differences in gene richness	ANOVA	LP	O	GH	OM0 (3)	OM1 (3)	OM2 (3)	OM3 (3)	1.48	0.292	
Differences in gene richness	ANOVA	LP	M	GT	OM0 (3)	OM1 (3)	OM2 (3)	OM3 (2)	5.076	0.0354	OM1>OM3
Differences in gene richness	ANOVA	LP	O	GT	OM0 (3)	OM1 (3)	OM2 (3)	OM3 (3)	1.52	0.282	
Differences in gene richness	ANOVA	LP	M	PL	OM0 (3)	OM1 (3)	OM2 (3)	OM3 (2)	2.986	0.105	
Differences in gene richness	ANOVA	LP	O	PL	OM0 (3)	OM1 (3)	OM2 (3)	OM3 (3)	1.831	0.22	

ANOVA tests for nitrogen cycle genes

amoAA and amoAB stand for the bacterial and archaeal version of the amoA gene

Soil layer: M=mineral, O=Organic

Number of replicated for each groups being compared is included in parenthesis

Test	Statistical test	Ezozone	Layer	Gene	Group 1	Group2	Group3	Group4	F value	p-value	Posthoc Tukey test result (p<0.05)
Differences in gene abundances	ANOVA	BS	M	amoAA	OM0 (3)	OM1 (3)	OM2 (3)	OM3 (3)	0.155	0.923	NA
Differences in gene abundances	ANOVA	BS	O	amoAA	OM0 (3)	OM1 (3)	OM2 (3)	NA	1	0.422	NA
Differences in gene abundances	ANOVA	BS	M	amoAB	OM0 (3)	OM1 (3)	OM2 (3)	OM3 (3)	0.895	0.485	NA
Differences in gene abundances	ANOVA	BS	O	amoAB	OM0 (3)	OM1 (3)	OM2 (3)	NA	4.429	0.0658	NA
Differences in gene abundances	ANOVA	BS	M	nifH	OM0 (3)	OM1 (3)	OM2 (3)	OM3 (3)	0.291	0.831	NA
Differences in gene abundances	ANOVA	BS	O	nifH	OM0 (3)	OM1 (3)	OM2 (3)	NA	0.699	0.534	NA
Differences in gene abundances	ANOVA	BS	M	nirS	OM0 (3)	OM1 (3)	OM2 (3)	OM3 (3)	0.698	0.579	NA
Differences in gene abundances	ANOVA	BS	O	nirS	OM0 (3)	OM1 (3)	OM2 (3)	NA	2.71	0.145	NA
Differences in gene abundances	ANOVA	BS	M	nirK	OM0 (3)	OM1 (3)	OM2 (3)	OM3 (3)	1.303	0.339	NA
Differences in gene abundances	ANOVA	BS	O	nirK	OM0 (3)	OM1 (3)	OM2 (3)	NA	1.346	0.329	NA
Differences in gene abundances	ANOVA	BS	M	norB	OM0 (3)	OM1 (3)	OM2 (3)	OM3 (3)	0.549	0.663	NA
Differences in gene abundances	ANOVA	BS	O	norB	OM0 (3)	OM1 (3)	OM2 (3)	NA	1.919	0.227	NA
Differences in gene abundances	ANOVA	BS	M	nosZ	OM0 (3)	OM1 (3)	OM2 (3)	OM3 (3)	0.491	0.698	NA
Differences in gene abundances	ANOVA	BS	O	nosZ	OM0 (3)	OM1 (3)	OM2 (3)	NA	0.956	0.436	NA
Differences in gene abundances	ANOVA	BS	M	nrfA	OM0 (3)	OM1 (3)	OM2 (3)	OM3 (3)	0.7	0.578	NA
Differences in gene abundances	ANOVA	BS	O	nrfA	OM0 (3)	OM1 (3)	OM2 (3)	NA	1.014	0.417	NA
Differences in gene abundances	ANOVA	PP	M	amoAA	OM0 (3)	OM1 (3)	OM2 (3)	OM3 (3)	1.289	0.343	NA
Differences in gene abundances	ANOVA	PP	O	amoAA	OM0 (3)	OM1 (3)	OM2 (3)	NA	6.107	0.0357	OM0<OM2
Differences in gene abundances	ANOVA	PP	M	amoAB	OM0 (3)	OM1 (3)	OM2 (3)	OM3 (3)	1.459	0.297	NA
Differences in gene abundances	ANOVA	PP	O	amoAB	OM0 (3)	OM1 (3)	OM2 (3)	NA	1.685	0.263	NA
Differences in gene abundances	ANOVA	PP	M	nifH	OM0 (3)	OM1 (3)	OM2 (3)	OM3 (3)	2.759	0.112	NA
Differences in gene abundances	ANOVA	PP	O	nifH	OM0 (3)	OM1 (3)	OM2 (3)	NA	3.486	0.099	NA
Differences in gene abundances	ANOVA	PP	M	nirS	OM0 (3)	OM1 (3)	OM2 (3)	OM3 (3)	3.115	0.0883	NA
Differences in gene abundances	ANOVA	PP	O	nirS	OM0 (3)	OM1 (3)	OM2 (3)	NA	2.206	0.191	NA
Differences in gene abundances	ANOVA	PP	M	nirK	OM0 (3)	OM1 (3)	OM2 (3)	OM3 (3)	0.453	0.722	NA
Differences in gene abundances	ANOVA	PP	O	nirK	OM0 (3)	OM1 (3)	OM2 (3)	NA	8.679	0.017	OM0<OM1, OM0<OM2
Differences in gene abundances	ANOVA	PP	M	norB	OM0 (3)	OM1 (3)	OM2 (3)	OM3 (3)	11.7	0.00269	OM0<OM1, OM0<OM2, OM2>OM3
Differences in gene abundances	ANOVA	PP	O	norB	OM0 (3)	OM1 (3)	OM2 (3)	NA	4.405	0.066	NA
Differences in gene abundances	ANOVA	PP	M	nosZ	OM0 (3)	OM1 (3)	OM2 (3)	OM3 (3)	5.821	0.0207	OM0<OM2, OM1<OM2, OM2>OM3
Differences in gene abundances	ANOVA	PP	O	nosZ	OM0 (3)	OM1 (3)	OM2 (3)	NA	8.075	0.0199	OM0<OM2
Differences in gene abundances	ANOVA	PP	M	nrfA	OM0 (3)	OM1 (3)	OM2 (3)	OM3 (3)	4.804	0.0337	ns
Differences in gene abundances	ANOVA	PP	O	nrfA	OM0 (3)	OM1 (3)	OM2 (3)	NA	23.8	0.0014	OM0<OM2, OM1<OM2
Differences in gene abundances	ANOVA	JP	M	amoAA	OM0 (3)	OM1 (3)	OM2 (3)	OM3 (3)	1.939	0.202	NA
Differences in gene abundances	ANOVA	JP	O	amoAA	OM0 (3)	OM1 (3)	OM2 (3)	NA	NA	NA	NA
Differences in gene abundances	ANOVA	JP	M	amoAB	OM0 (3)	OM1 (3)	OM2 (3)	OM3 (3)	3.265	0.0803	NA
Differences in gene abundances	ANOVA	JP	O	amoAB	OM0 (3)	OM1 (3)	OM2 (3)	NA	5.018	0.0524	NA

Differences in gene abundances	ANOVA	JP	M	nifH	OM0 (3)	OM1 (3)	OM2 (3)	OM3 (3)	1.488	0.29	NA
Differences in gene abundances	ANOVA	JP	O	nifH	OM0 (3)	OM1 (3)	OM2 (3)	NA	0.652	0.554	NA
Differences in gene abundances	ANOVA	JP	M	nirS	OM0 (3)	OM1 (3)	OM2 (3)	OM3 (3)	1.995	0.193	NA
Differences in gene abundances	ANOVA	JP	O	nirS	OM0 (3)	OM1 (3)	OM2 (3)	NA	1.424	0.312	NA
Differences in gene abundances	ANOVA	JP	M	nirK	OM0 (3)	OM1 (3)	OM2 (3)	OM3 (3)	2.722	0.114	NA
Differences in gene abundances	ANOVA	JP	O	nirK	OM0 (3)	OM1 (3)	OM2 (3)	NA	0.822	0.484	NA
Differences in gene abundances	ANOVA	JP	M	norB	OM0 (3)	OM1 (3)	OM2 (3)	OM3 (3)	1.861	0.215	NA
Differences in gene abundances	ANOVA	JP	O	norB	OM0 (3)	OM1 (3)	OM2 (3)	NA	0.182	0.838	NA
Differences in gene abundances	ANOVA	JP	M	nosZ	OM0 (3)	OM1 (3)	OM2 (3)	OM3 (3)	1.483	0.291	NA
Differences in gene abundances	ANOVA	JP	O	nosZ	OM0 (3)	OM1 (3)	OM2 (3)	NA	3.337	0.106	NA
Differences in gene abundances	ANOVA	JP	M	nrfA	OM0 (3)	OM1 (3)	OM2 (3)	OM3 (3)	1.749	0.234	NA
Differences in gene abundances	ANOVA	JP	O	nrfA	OM0 (3)	OM1 (3)	OM2 (3)	NA	0.476	0.643	NA
Differences in gene abundances	ANOVA	IDF	M	amoAA	OM0 (3)	OM1 (3)	OM2 (3)	OM3 (3)	1.77	0.23	NA
Differences in gene abundances	ANOVA	IDF	O	amoAA	OM0 (3)	OM1 (3)	OM2 (3)	NA	1	0.422	NA
Differences in gene abundances	ANOVA	IDF	M	amoAB	OM0 (3)	OM1 (3)	OM2 (3)	OM3 (3)	1.15	0.386	NA
Differences in gene abundances	ANOVA	IDF	O	amoAB	OM0 (3)	OM1 (3)	OM2 (3)	NA	1.324	0.334	NA
Differences in gene abundances	ANOVA	IDF	M	nifH	OM0 (3)	OM1 (3)	OM2 (3)	OM3 (3)	24.07	0.000233	OM0<OM1, OM1>OM2, OM1>OM3
Differences in gene abundances	ANOVA	IDF	O	nifH	OM0 (3)	OM1 (3)	OM2 (3)	NA	0.93	0.445	NA
Differences in gene abundances	ANOVA	IDF	M	nirS	OM0 (3)	OM1 (3)	OM2 (3)	OM3 (3)	1.923	0.204	NA
Differences in gene abundances	ANOVA	IDF	O	nirS	OM0 (3)	OM1 (3)	OM2 (3)	NA	2.847	0.135	NA
Differences in gene abundances	ANOVA	IDF	M	nirK	OM0 (3)	OM1 (3)	OM2 (3)	OM3 (3)	4.487	0.0398	OM1>OM2
Differences in gene abundances	ANOVA	IDF	O	nirK	OM0 (3)	OM1 (3)	OM2 (3)	NA	8.327	0.0186	OM0<OM1
Differences in gene abundances	ANOVA	IDF	M	norB	OM0 (3)	OM1 (3)	OM2 (3)	OM3 (3)	1.577	0.269	NA
Differences in gene abundances	ANOVA	IDF	O	norB	OM0 (3)	OM1 (3)	OM2 (3)	NA	2.676	0.148	NA
Differences in gene abundances	ANOVA	IDF	M	nosZ	OM0 (3)	OM1 (3)	OM2 (3)	OM3 (3)	1.32	0.334	NA
Differences in gene abundances	ANOVA	IDF	O	nosZ	OM0 (3)	OM1 (3)	OM2 (3)	NA	13.05	0.00653	OM0<OM1, OM0<OM2
Differences in gene abundances	ANOVA	IDF	M	nrfA	OM0 (3)	OM1 (3)	OM2 (3)	OM3 (3)	6.865	0.0133	OM0<OM3, OM2<OM3
Differences in gene abundances	ANOVA	IDF	O	nrfA	OM0 (3)	OM1 (3)	OM2 (3)	NA	7.088	0.0263	OM0<OM1
Differences in gene abundances	ANOVA	LP	M	amoAA	OM0 (3)	OM1 (3)	OM2 (3)	OM3 (2)	0.631	0.618	NA
Differences in gene abundances	ANOVA	LP	O	amoAA	OM0 (3)	OM1 (3)	OM2 (3)	OM3 (3)	0.462	0.717	NA
Differences in gene abundances	ANOVA	LP	M	amoAB	OM0 (3)	OM1 (3)	OM2 (3)	OM3 (2)	0.711	0.575	NA
Differences in gene abundances	ANOVA	LP	O	amoAB	OM0 (3)	OM1 (3)	OM2 (3)	OM3 (3)	1.79	0.227	NA
Differences in gene abundances	ANOVA	LP	M	nifH	OM0 (3)	OM1 (3)	OM2 (3)	OM3 (2)	6.834	0.0173	OM0<OM3, OM1<OM3
Differences in gene abundances	ANOVA	LP	O	nifH	OM0 (3)	OM1 (3)	OM2 (3)	OM3 (3)	9.701	0.00484	OM0<OM3
Differences in gene abundances	ANOVA	LP	M	nirS	OM0 (3)	OM1 (3)	OM2 (3)	OM3 (2)	0.677	0.593	NA
Differences in gene abundances	ANOVA	LP	O	nirS	OM0 (3)	OM1 (3)	OM2 (3)	OM3 (3)	0.783	0.536	NA
Differences in gene abundances	ANOVA	LP	M	nirK	OM0 (3)	OM1 (3)	OM2 (3)	OM3 (2)	4.498	0.0464	OM0<OM3
Differences in gene abundances	ANOVA	LP	O	nirK	OM0 (3)	OM1 (3)	OM2 (3)	OM3 (3)	0.616	0.624	NA
Differences in gene abundances	ANOVA	LP	M	norB	OM0 (3)	OM1 (3)	OM2 (3)	OM3 (2)	7.451	0.0139	OM0<OM1, OM0<OM3
Differences in gene abundances	ANOVA	LP	O	norB	OM0 (3)	OM1 (3)	OM2 (3)	OM3 (3)	3.527	0.0684	NA
Differences in gene abundances	ANOVA	LP	M	nosZ	OM0 (3)	OM1 (3)	OM2 (3)	OM3 (2)	1.689	0.255	NA
Differences in gene abundances	ANOVA	LP	O	nosZ	OM0 (3)	OM1 (3)	OM2 (3)	OM3 (3)	4.384	0.042	OM0<OM1
Differences in gene abundances	ANOVA	LP	M	nrfA	OM0 (3)	OM1 (3)	OM2 (3)	OM3 (2)	1.807	0.234	NA
Differences in gene abundances	ANOVA	LP	O	nrfA	OM0 (3)	OM1 (3)	OM2 (3)	OM3 (3)	1.557	0.274	NA
Differences in gene richness	ANOVA	BS	M	amoAA	OM0 (3)	OM1 (3)	OM2 (3)	OM3 (3)	0.319	0.812	NA
Differences in gene richness	ANOVA	BS	O	amoAA	OM0 (3)	OM1 (3)	OM2 (3)	NA	1	0.422	NA

Differences in gene richness	ANOVA	BS	M	amoAB	OM0 (3)	OM1 (3)	OM2 (3)	OM3 (3)	0.18	0.907	NA
Differences in gene richness	ANOVA	BS	O	amoAB	OM0 (3)	OM1 (3)	OM2 (3)	NA	3.41	0.103	NA
Differences in gene richness	ANOVA	BS	M	nifH	OM0 (3)	OM1 (3)	OM2 (3)	OM3 (3)	7.061	0.0123	OM0<OM2, OM1<OM2
Differences in gene richness	ANOVA	BS	O	nifH	OM0 (3)	OM1 (3)	OM2 (3)	NA	1.153	0.377	NA
Differences in gene richness	ANOVA	BS	M	nirS	OM0 (3)	OM1 (3)	OM2 (3)	OM3 (3)	0.456	0.72	NA
Differences in gene richness	ANOVA	BS	O	nirS	OM0 (3)	OM1 (3)	OM2 (3)	NA	2.625	0.152	NA
Differences in gene richness	ANOVA	BS	M	nirK	OM0 (3)	OM1 (3)	OM2 (3)	OM3 (3)	0.985	0.447	NA
Differences in gene richness	ANOVA	BS	O	nirK	OM0 (3)	OM1 (3)	OM2 (3)	NA	1.405	0.316	NA
Differences in gene richness	ANOVA	BS	M	norB	OM0 (3)	OM1 (3)	OM2 (3)	OM3 (3)	1.791	0.227	NA
Differences in gene richness	ANOVA	BS	O	norB	OM0 (3)	OM1 (3)	OM2 (3)	NA	2.162	0.196	NA
Differences in gene richness	ANOVA	BS	M	nosZ	OM0 (3)	OM1 (3)	OM2 (3)	OM3 (3)	0.664	0.597	NA
Differences in gene richness	ANOVA	BS	O	nosZ	OM0 (3)	OM1 (3)	OM2 (3)	NA	1.228	0.357	NA
Differences in gene richness	ANOVA	BS	M	nrfA	OM0 (3)	OM1 (3)	OM2 (3)	OM3 (3)	1.281	0.345	NA
Differences in gene richness	ANOVA	BS	O	nrfA	OM0 (3)	OM1 (3)	OM2 (3)	NA	1.261	0.349	NA
Differences in gene richness	ANOVA	PP	M	amoAA	OM0 (3)	OM1 (3)	OM2 (3)	OM3 (3)	1.282	0.345	NA
Differences in gene richness	ANOVA	PP	O	amoAA	OM0 (3)	OM1 (3)	OM2 (3)	NA	13.9	0.00559	OM0<OM1, OM0<OM2, OM1<OM2
Differences in gene richness	ANOVA	PP	M	amoAB	OM0 (3)	OM1 (3)	OM2 (3)	OM3 (3)	0.669	0.595	NA
Differences in gene richness	ANOVA	PP	O	amoAB	OM0 (3)	OM1 (3)	OM2 (3)	NA	13.88	0.00562	OM0>OM1, OM0<OM2
Differences in gene richness	ANOVA	PP	M	nifH	OM0 (3)	OM1 (3)	OM2 (3)	OM3 (3)	1.456	0.298	NA
Differences in gene richness	ANOVA	PP	O	nifH	OM0 (3)	OM1 (3)	OM2 (3)	NA	4.258	0.0706	NA
Differences in gene richness	ANOVA	PP	M	nirS	OM0 (3)	OM1 (3)	OM2 (3)	OM3 (3)	0.98	0.449	NA
Differences in gene richness	ANOVA	PP	O	nirS	OM0 (3)	OM1 (3)	OM2 (3)	NA	1.444	0.308	NA
Differences in gene richness	ANOVA	PP	M	nirK	OM0 (3)	OM1 (3)	OM2 (3)	OM3 (3)	6.578	0.0149	OM0<OM1, OM0<OM2
Differences in gene richness	ANOVA	PP	O	nirK	OM0 (3)	OM1 (3)	OM2 (3)	NA	15.29	0.00441	OM0<OM2
Differences in gene richness	ANOVA	PP	M	norB	OM0 (3)	OM1 (3)	OM2 (3)	OM3 (3)	6.66	0.0144	OM0<OM1, OM0<OM2
Differences in gene richness	ANOVA	PP	O	norB	OM0 (3)	OM1 (3)	OM2 (3)	NA	6.869	0.0281	OM0<OM2
Differences in gene richness	ANOVA	PP	M	nosZ	OM0 (3)	OM1 (3)	OM2 (3)	OM3 (3)	1.517	0.283	NA
Differences in gene richness	ANOVA	PP	O	nosZ	OM0 (3)	OM1 (3)	OM2 (3)	NA	21.22	0.0019	OM0<OM1, OM0<OM2, OM1<OM2
Differences in gene richness	ANOVA	PP	M	nrfA	OM0 (3)	OM1 (3)	OM2 (3)	OM3 (3)	2.807	0.108	NA
Differences in gene richness	ANOVA	PP	O	nrfA	OM0 (3)	OM1 (3)	OM2 (3)	NA	9	0.0156	OM0<OM2
Differences in gene richness	ANOVA	JP	M	amoAA	OM0 (3)	OM1 (3)	OM2 (3)	OM3 (3)	0.922	0.473	NA
Differences in gene richness	ANOVA	JP	O	amoAA	OM0 (3)	OM1 (3)	OM2 (3)	NA	NA	NA	NA
Differences in gene richness	ANOVA	JP	M	amoAB	OM0 (3)	OM1 (3)	OM2 (3)	OM3 (3)	3.59	0.0658	NA
Differences in gene richness	ANOVA	JP	O	amoAB	OM0 (3)	OM1 (3)	OM2 (3)	NA	1.488	0.299	NA
Differences in gene richness	ANOVA	JP	M	nifH	OM0 (3)	OM1 (3)	OM2 (3)	OM3 (3)	0.682	0.587	NA
Differences in gene richness	ANOVA	JP	O	nifH	OM0 (3)	OM1 (3)	OM2 (3)	NA	0.671	0.546	NA
Differences in gene richness	ANOVA	JP	M	nirS	OM0 (3)	OM1 (3)	OM2 (3)	OM3 (3)	3.048	0.0922	NA
Differences in gene richness	ANOVA	JP	O	nirS	OM0 (3)	OM1 (3)	OM2 (3)	NA	2.6	0.154	NA
Differences in gene richness	ANOVA	JP	M	nirK	OM0 (3)	OM1 (3)	OM2 (3)	OM3 (3)	3.748	0.06	NA
Differences in gene richness	ANOVA	JP	O	nirK	OM0 (3)	OM1 (3)	OM2 (3)	NA	1.24	0.354	NA
Differences in gene richness	ANOVA	JP	M	norB	OM0 (3)	OM1 (3)	OM2 (3)	OM3 (3)	0.403	0.755	NA
Differences in gene richness	ANOVA	JP	O	norB	OM0 (3)	OM1 (3)	OM2 (3)	NA	1.297	0.34	NA
Differences in gene richness	ANOVA	JP	M	nosZ	OM0 (3)	OM1 (3)	OM2 (3)	OM3 (3)	1.39	0.314	NA
Differences in gene richness	ANOVA	JP	O	nosZ	OM0 (3)	OM1 (3)	OM2 (3)	NA	2.136	0.199	NA
Differences in gene richness	ANOVA	JP	M	nrfA	OM0 (3)	OM1 (3)	OM2 (3)	OM3 (3)	1.452	0.298	NA
Differences in gene richness	ANOVA	JP	O	nrfA	OM0 (3)	OM1 (3)	OM2 (3)	NA	0.198	0.826	NA

Differences in gene richness	ANOVA	IDF	M	amoAA	OM0 (3)	OM1 (3)	OM2 (3)	OM3 (3)	1.9	0.208	NA
Differences in gene richness	ANOVA	IDF	O	amoAA	OM0 (3)	OM1 (3)	OM2 (3)	NA	1	0.422	NA
Differences in gene richness	ANOVA	IDF	M	amoAB	OM0 (3)	OM1 (3)	OM2 (3)	OM3 (3)	0.205	0.89	NA
Differences in gene richness	ANOVA	IDF	O	amoAB	OM0 (3)	OM1 (3)	OM2 (3)	NA	2.049	0.21	NA
Differences in gene richness	ANOVA	IDF	M	nifH	OM0 (3)	OM1 (3)	OM2 (3)	OM3 (3)	27.848	1.38E-04	OM0<OM1, OM1>OM2, OM1>OM3
Differences in gene richness	ANOVA	IDF	O	nifH	OM0 (3)	OM1 (3)	OM2 (3)	NA	4.752	0.058	NA
Differences in gene richness	ANOVA	IDF	M	nirS	OM0 (3)	OM1 (3)	OM2 (3)	OM3 (3)	1.987	0.1946	NA
Differences in gene richness	ANOVA	IDF	O	nirS	OM0 (3)	OM1 (3)	OM2 (3)	NA	2.03	0.2121	NA
Differences in gene richness	ANOVA	IDF	M	nirK	OM0 (3)	OM1 (3)	OM2 (3)	OM3 (3)	4.275	0.0446	NA
Differences in gene richness	ANOVA	IDF	O	nirK	OM0 (3)	OM1 (3)	OM2 (3)	NA	7.088	0.0263	OM0<OM1
Differences in gene richness	ANOVA	IDF	M	norB	OM0 (3)	OM1 (3)	OM2 (3)	OM3 (3)	6.168	0.0178	OM0<OM1
Differences in gene richness	ANOVA	IDF	O	norB	OM0 (3)	OM1 (3)	OM2 (3)	NA	3.301	0.1079	NA
Differences in gene richness	ANOVA	IDF	M	nosZ	OM0 (3)	OM1 (3)	OM2 (3)	OM3 (3)	4.333	0.0432	OM0<OM1
Differences in gene richness	ANOVA	IDF	O	nosZ	OM0 (3)	OM1 (3)	OM2 (3)	NA	7.516	0.0232	OM0<OM1
Differences in gene richness	ANOVA	IDF	M	nrfA	OM0 (3)	OM1 (3)	OM2 (3)	OM3 (3)	0.858	0.501	NA
Differences in gene richness	ANOVA	IDF	O	nrfA	OM0 (3)	OM1 (3)	OM2 (3)	NA	9.213	0.0148	OM0<OM1
Differences in gene richness	ANOVA	LP	M	amoAA	OM0 (3)	OM1 (3)	OM2 (3)	OM3 (2)	1.065	0.4228	NA
Differences in gene richness	ANOVA	LP	O	amoAA	OM0 (3)	OM1 (3)	OM2 (3)	OM3 (3)	0.444	0.7278	NA
Differences in gene richness	ANOVA	LP	M	amoAB	OM0 (3)	OM1 (3)	OM2 (3)	OM3 (2)	3.966	0.0607	NA
Differences in gene richness	ANOVA	LP	O	amoAB	OM0 (3)	OM1 (3)	OM2 (3)	OM3 (3)	2.014	0.1905	NA
Differences in gene richness	ANOVA	LP	M	nifH	OM0 (3)	OM1 (3)	OM2 (3)	OM3 (2)	8.28	1.06E-02	OM1<OM3
Differences in gene richness	ANOVA	LP	O	nifH	OM0 (3)	OM1 (3)	OM2 (3)	OM3 (3)	6.702	0.0142	OM0<OM3,OM1<OM3, OM2<OM3
Differences in gene richness	ANOVA	LP	M	nirS	OM0 (3)	OM1 (3)	OM2 (3)	OM3 (2)	0.83	0.5181	NA
Differences in gene richness	ANOVA	LP	O	nirS	OM0 (3)	OM1 (3)	OM2 (3)	OM3 (3)	6.306	0.0168	OM0<OM3, OM2<OM3
Differences in gene richness	ANOVA	LP	M	nirK	OM0 (3)	OM1 (3)	OM2 (3)	OM3 (2)	1.942	0.2115	NA
Differences in gene richness	ANOVA	LP	O	nirK	OM0 (3)	OM1 (3)	OM2 (3)	OM3 (3)	1.67	0.2497	NA
Differences in gene richness	ANOVA	LP	M	norB	OM0 (3)	OM1 (3)	OM2 (3)	OM3 (2)	2.283	0.1661	NA
Differences in gene richness	ANOVA	LP	O	norB	OM0 (3)	OM1 (3)	OM2 (3)	OM3 (3)	5.128	0.0287	OM0<OM1
Differences in gene richness	ANOVA	LP	M	nosZ	OM0 (3)	OM1 (3)	OM2 (3)	OM3 (2)	0.588	0.6422	NA
Differences in gene richness	ANOVA	LP	O	nosZ	OM0 (3)	OM1 (3)	OM2 (3)	OM3 (3)	3.895	0.0551	NA
Differences in gene richness	ANOVA	LP	M	nrfA	OM0 (3)	OM1 (3)	OM2 (3)	OM3 (2)	1.401	0.3201	NA
Differences in gene richness	ANOVA	LP	O	nrfA	OM0 (3)	OM1 (3)	OM2 (3)	OM3 (3)	2.725	0.1142	NA
Differences in gene eveness	ANOVA	BS	M	amoAA	OM0 (3)	OM1 (3)	OM2 (3)	OM3 (3)	0.898	0.4834	NA
Differences in gene eveness	ANOVA	BS	O	amoAA	OM0 (3)	OM1 (3)	OM2 (3)	NA	NA	NA	NA
Differences in gene eveness	ANOVA	BS	M	amoAB	OM0 (3)	OM1 (3)	OM2 (3)	OM3 (3)	0.249	0.86	NA
Differences in gene eveness	ANOVA	BS	O	amoAB	OM0 (3)	OM1 (3)	OM2 (3)	NA	3.251	0.1106	NA
Differences in gene eveness	ANOVA	BS	M	nifH	OM0 (3)	OM1 (3)	OM2 (3)	OM3 (3)	4.605	0.0374	NA
Differences in gene eveness	ANOVA	BS	O	nifH	OM0 (3)	OM1 (3)	OM2 (3)	NA	0.645	0.5575	NA
Differences in gene eveness	ANOVA	BS	M	nirS	OM0 (3)	OM1 (3)	OM2 (3)	OM3 (3)	0.529	0.6746	NA
Differences in gene eveness	ANOVA	BS	O	nirS	OM0 (3)	OM1 (3)	OM2 (3)	NA	5.365	0.0461	OM0<OM1
Differences in gene eveness	ANOVA	BS	M	nirK	OM0 (3)	OM1 (3)	OM2 (3)	OM3 (3)	0.364	0.7808	NA
Differences in gene eveness	ANOVA	BS	O	nirK	OM0 (3)	OM1 (3)	OM2 (3)	NA	0.79	0.4958	NA
Differences in gene eveness	ANOVA	BS	M	norB	OM0 (3)	OM1 (3)	OM2 (3)	OM3 (3)	0.445	0.7274	NA
Differences in gene eveness	ANOVA	BS	O	norB	OM0 (3)	OM1 (3)	OM2 (3)	NA	2.89	0.1321	NA
Differences in gene eveness	ANOVA	BS	M	nosZ	OM0 (3)	OM1 (3)	OM2 (3)	OM3 (3)	0.238	0.8678	NA
Differences in gene eveness	ANOVA	BS	O	nosZ	OM0 (3)	OM1 (3)	OM2 (3)	NA	0.436	0.6654	NA

Differences in gene eveness	ANOVA	BS	M	nrfA	OM0 (3)	OM1 (3)	OM2 (3)	OM3 (3)	2.124	0.1754	NA
Differences in gene eveness	ANOVA	BS	O	nrfA	OM0 (3)	OM1 (3)	OM2 (3)	NA	0.734	0.5184	NA
Differences in gene eveness	ANOVA	PP	M	amoAA	OM0 (3)	OM1 (3)	OM2 (3)	OM3 (3)	30.4	5.03E-04	OM0<OM1, OM0<OM2, OM0<OM3
Differences in gene eveness	ANOVA	PP	O	amoAA	OM0 (3)	OM1 (3)	OM2 (3)	NA	6.552	0.0401	OM0<OM2
Differences in gene eveness	ANOVA	PP	M	amoAB	OM0 (3)	OM1 (3)	OM2 (3)	OM3 (3)	3.102	0.0891	NA
Differences in gene eveness	ANOVA	PP	O	amoAB	OM0 (3)	OM1 (3)	OM2 (3)	NA	1.388	0.3315	NA
Differences in gene eveness	ANOVA	PP	M	nifH	OM0 (3)	OM1 (3)	OM2 (3)	OM3 (3)	2.737	0.1133	NA
Differences in gene eveness	ANOVA	PP	O	nifH	OM0 (3)	OM1 (3)	OM2 (3)	NA	1.01	0.4188	NA
Differences in gene eveness	ANOVA	PP	M	nirS	OM0 (3)	OM1 (3)	OM2 (3)	OM3 (3)	0.433	0.735	NA
Differences in gene eveness	ANOVA	PP	O	nirS	OM0 (3)	OM1 (3)	OM2 (3)	NA	0.228	0.8042	NA
Differences in gene eveness	ANOVA	PP	M	nirK	OM0 (3)	OM1 (3)	OM2 (3)	OM3 (3)	6.294	0.0168	OM0<OM2
Differences in gene eveness	ANOVA	PP	O	nirK	OM0 (3)	OM1 (3)	OM2 (3)	NA	5.248	0.0481	OM1<OM2
Differences in gene eveness	ANOVA	PP	M	norB	OM0 (3)	OM1 (3)	OM2 (3)	OM3 (3)	1.907	0.2069	NA
Differences in gene eveness	ANOVA	PP	O	norB	OM0 (3)	OM1 (3)	OM2 (3)	NA	0.711	0.5284	NA
Differences in gene eveness	ANOVA	PP	M	nosZ	OM0 (3)	OM1 (3)	OM2 (3)	OM3 (3)	0.813	0.5218	NA
Differences in gene eveness	ANOVA	PP	O	nosZ	OM0 (3)	OM1 (3)	OM2 (3)	NA	5.266	0.0478	NA
Differences in gene eveness	ANOVA	PP	M	nrfA	OM0 (3)	OM1 (3)	OM2 (3)	OM3 (3)	1.724	0.239	NA
Differences in gene eveness	ANOVA	PP	O	nrfA	OM0 (3)	OM1 (3)	OM2 (3)	NA	4.172	0.0732	NA
Differences in gene eveness	ANOVA	JP	M	amoAA	OM0 (3)	OM1 (3)	OM2 (3)	OM3 (3)	2.13	0.215	NA
Differences in gene eveness	ANOVA	JP	O	amoAA	OM0 (3)	OM1 (3)	OM2 (3)	NA	NA	NA	NA
Differences in gene eveness	ANOVA	JP	M	amoAB	OM0 (3)	OM1 (3)	OM2 (3)	OM3 (3)	2.695	0.1166	NA
Differences in gene eveness	ANOVA	JP	O	amoAB	OM0 (3)	OM1 (3)	OM2 (3)	NA	4.423	0.066	NA
Differences in gene eveness	ANOVA	JP	M	nifH	OM0 (3)	OM1 (3)	OM2 (3)	OM3 (3)	1.891	0.2096	NA
Differences in gene eveness	ANOVA	JP	O	nifH	OM0 (3)	OM1 (3)	OM2 (3)	NA	6.342	0.0331	OM0<OM1
Differences in gene eveness	ANOVA	JP	M	nirS	OM0 (3)	OM1 (3)	OM2 (3)	OM3 (3)	2.34	0.1497	NA
Differences in gene eveness	ANOVA	JP	O	nirS	OM0 (3)	OM1 (3)	OM2 (3)	NA	0.767	0.5122	NA
Differences in gene eveness	ANOVA	JP	M	nirK	OM0 (3)	OM1 (3)	OM2 (3)	OM3 (3)	2.632	0.1217	NA
Differences in gene eveness	ANOVA	JP	O	nirK	OM0 (3)	OM1 (3)	OM2 (3)	NA	0.124	0.8852	NA
Differences in gene eveness	ANOVA	JP	M	norB	OM0 (3)	OM1 (3)	OM2 (3)	OM3 (3)	1.012	0.4363	NA
Differences in gene eveness	ANOVA	JP	O	norB	OM0 (3)	OM1 (3)	OM2 (3)	NA	0.272	0.7711	NA
Differences in gene eveness	ANOVA	JP	M	nosZ	OM0 (3)	OM1 (3)	OM2 (3)	OM3 (3)	1.166	0.3812	NA
Differences in gene eveness	ANOVA	JP	O	nosZ	OM0 (3)	OM1 (3)	OM2 (3)	NA	6.953	0.0274	OM0<OM1
Differences in gene eveness	ANOVA	JP	M	nrfA	OM0 (3)	OM1 (3)	OM2 (3)	OM3 (3)	3.862	0.0561	NA
Differences in gene eveness	ANOVA	JP	O	nrfA	OM0 (3)	OM1 (3)	OM2 (3)	NA	2.468	0.1652	NA
Differences in gene eveness	ANOVA	IDF	M	amoAA	OM0 (3)	OM1 (3)	OM2 (3)	OM3 (3)	3.393	0.083	NA
Differences in gene eveness	ANOVA	IDF	O	amoAA	OM0 (3)	OM1 (3)	OM2 (3)	NA	1	0.4219	NA
Differences in gene eveness	ANOVA	IDF	M	amoAB	OM0 (3)	OM1 (3)	OM2 (3)	OM3 (3)	3.039	0.0928	NA
Differences in gene eveness	ANOVA	IDF	O	amoAB	OM0 (3)	OM1 (3)	OM2 (3)	NA	0.805	0.49	NA
Differences in gene eveness	ANOVA	IDF	M	nifH	OM0 (3)	OM1 (3)	OM2 (3)	OM3 (3)	0.021	0.9956	NA
Differences in gene eveness	ANOVA	IDF	O	nifH	OM0 (3)	OM1 (3)	OM2 (3)	NA	26.136	1.09E-03	OM0<OM1, OM1>OM2
Differences in gene eveness	ANOVA	IDF	M	nirS	OM0 (3)	OM1 (3)	OM2 (3)	OM3 (3)	1.009	0.4286	NA
Differences in gene eveness	ANOVA	IDF	O	nirS	OM0 (3)	OM1 (3)	OM2 (3)	NA	0.169	0.8491	NA
Differences in gene eveness	ANOVA	IDF	M	nirK	OM0 (3)	OM1 (3)	OM2 (3)	OM3 (3)	0.21	0.8868	NA
Differences in gene eveness	ANOVA	IDF	O	nirK	OM0 (3)	OM1 (3)	OM2 (3)	NA	3.489	0.0988	NA
Differences in gene eveness	ANOVA	IDF	M	norB	OM0 (3)	OM1 (3)	OM2 (3)	OM3 (3)	0.661	0.599	NA
Differences in gene eveness	ANOVA	IDF	O	norB	OM0 (3)	OM1 (3)	OM2 (3)	NA	0.229	0.8017	NA

Differences in gene eveness	ANOVA	IDF	M	nosZ	OM0 (3)	OM1 (3)	OM2 (3)	OM3 (3)	1.313	0.3358	NA
Differences in gene eveness	ANOVA	IDF	O	nosZ	OM0 (3)	OM1 (3)	OM2 (3)	NA	2.517	0.1608	NA
Differences in gene eveness	ANOVA	IDF	M	nrfA	OM0 (3)	OM1 (3)	OM2 (3)	OM3 (3)	0.86	0.5	NA
Differences in gene eveness	ANOVA	IDF	O	nrfA	OM0 (3)	OM1 (3)	OM2 (3)	NA	0.979	0.4286	NA
Differences in gene eveness	ANOVA	LP	M	amoAA	OM0 (3)	OM1 (3)	OM2 (3)	OM3 (2)	1.799	0.2351	NA
Differences in gene eveness	ANOVA	LP	O	amoAA	OM0 (3)	OM1 (3)	OM2 (3)	OM3 (3)	0.7	0.5855	NA
Differences in gene eveness	ANOVA	LP	M	amoAB	OM0 (3)	OM1 (3)	OM2 (3)	OM3 (2)	0.361	0.7836	NA
Differences in gene eveness	ANOVA	LP	O	amoAB	OM0 (3)	OM1 (3)	OM2 (3)	OM3 (3)	0.383	0.7696	NA
Differences in gene eveness	ANOVA	LP	M	nifH	OM0 (3)	OM1 (3)	OM2 (3)	OM3 (2)	3.087	0.0992	NA
Differences in gene eveness	ANOVA	LP	O	nifH	OM0 (3)	OM1 (3)	OM2 (3)	OM3 (3)	2.527	0.131	NA
Differences in gene eveness	ANOVA	LP	M	nirS	OM0 (3)	OM1 (3)	OM2 (3)	OM3 (2)	0.776	0.5432	NA
Differences in gene eveness	ANOVA	LP	O	nirS	OM0 (3)	OM1 (3)	OM2 (3)	OM3 (3)	0.412	0.7486	NA
Differences in gene eveness	ANOVA	LP	M	nirK	OM0 (3)	OM1 (3)	OM2 (3)	OM3 (2)	0.344	0.7945	NA
Differences in gene eveness	ANOVA	LP	O	nirK	OM0 (3)	OM1 (3)	OM2 (3)	OM3 (3)	6.664	0.0144	OM2>OM3
Differences in gene eveness	ANOVA	LP	M	norB	OM0 (3)	OM1 (3)	OM2 (3)	OM3 (2)	1.859	0.2247	NA
Differences in gene eveness	ANOVA	LP	O	norB	OM0 (3)	OM1 (3)	OM2 (3)	OM3 (3)	2.332	0.1505	NA
Differences in gene eveness	ANOVA	LP	M	nosZ	OM0 (3)	OM1 (3)	OM2 (3)	OM3 (2)	7.961	0.0117	OM0>OM3, OM1>OM3, OM2>OM3
Differences in gene eveness	ANOVA	LP	O	nosZ	OM0 (3)	OM1 (3)	OM2 (3)	OM3 (3)	4.842	0.0331	OM0>OM3
Differences in gene eveness	ANOVA	LP	M	nrfA	OM0 (3)	OM1 (3)	OM2 (3)	OM3 (2)	0.402	0.7565	NA
Differences in gene eveness	ANOVA	LP	O	nrfA	OM0 (3)	OM1 (3)	OM2 (3)	OM3 (3)	4.198	0.0465	OM0>OM2
Differences in gene diversity (Shannon)	ANOVA	BS	M	amoAA	OM0 (3)	OM1 (3)	OM2 (3)	OM3 (3)	0.26	0.852	NA
Differences in gene diversity (Shannon)	ANOVA	BS	O	amoAA	OM0 (3)	OM1 (3)	OM2 (3)	NA	NA	NA	NA
Differences in gene diversity (Shannon)	ANOVA	BS	M	amoAB	OM0 (3)	OM1 (3)	OM2 (3)	OM3 (3)	0.172	0.9124	NA
Differences in gene diversity (Shannon)	ANOVA	BS	O	amoAB	OM0 (3)	OM1 (3)	OM2 (3)	NA	3.216	0.1124	NA
Differences in gene diversity (Shannon)	ANOVA	BS	M	nifH	OM0 (3)	OM1 (3)	OM2 (3)	OM3 (3)	5.642	0.0225	NA
Differences in gene diversity (Shannon)	ANOVA	BS	O	nifH	OM0 (3)	OM1 (3)	OM2 (3)	NA	0.937	0.4425	NA
Differences in gene diversity (Shannon)	ANOVA	BS	M	nirS	OM0 (3)	OM1 (3)	OM2 (3)	OM3 (3)	0.269	0.8459	NA
Differences in gene diversity (Shannon)	ANOVA	BS	O	nirS	OM0 (3)	OM1 (3)	OM2 (3)	NA	3.404	0.1028	NA
Differences in gene diversity (Shannon)	ANOVA	BS	M	nirK	OM0 (3)	OM1 (3)	OM2 (3)	OM3 (3)	0.479	0.7059	NA
Differences in gene diversity (Shannon)	ANOVA	BS	O	nirK	OM0 (3)	OM1 (3)	OM2 (3)	NA	0.744	0.5145	NA
Differences in gene diversity (Shannon)	ANOVA	BS	M	norB	OM0 (3)	OM1 (3)	OM2 (3)	OM3 (3)	0.679	0.5892	NA
Differences in gene diversity (Shannon)	ANOVA	BS	O	norB	OM0 (3)	OM1 (3)	OM2 (3)	NA	1.764	0.2498	NA
Differences in gene diversity (Shannon)	ANOVA	BS	M	nosZ	OM0 (3)	OM1 (3)	OM2 (3)	OM3 (3)	0.836	0.511	NA
Differences in gene diversity (Shannon)	ANOVA	BS	O	nosZ	OM0 (3)	OM1 (3)	OM2 (3)	NA	4.365	0.0676	NA
Differences in gene diversity (Shannon)	ANOVA	BS	M	nrfA	OM0 (3)	OM1 (3)	OM2 (3)	OM3 (3)	1.948	0.2005	NA
Differences in gene diversity (Shannon)	ANOVA	BS	O	nrfA	OM0 (3)	OM1 (3)	OM2 (3)	NA	3.081	0.1201	NA
Differences in gene diversity (Shannon)	ANOVA	PP	M	amoAA	OM0 (3)	OM1 (3)	OM2 (3)	OM3 (3)	1.32	0.3337	NA
Differences in gene diversity (Shannon)	ANOVA	PP	O	amoAA	OM0 (3)	OM1 (3)	OM2 (3)	NA	16.04	3.91E-03	OM0<OM2, OM1<OM2
Differences in gene diversity (Shannon)	ANOVA	PP	M	amoAB	OM0 (3)	OM1 (3)	OM2 (3)	OM3 (3)	0.542	0.6671	NA
Differences in gene diversity (Shannon)	ANOVA	PP	O	amoAB	OM0 (3)	OM1 (3)	OM2 (3)	NA	14.172	5.33E-03	OM0<OM1, OM0<OM2
Differences in gene diversity (Shannon)	ANOVA	PP	M	nifH	OM0 (3)	OM1 (3)	OM2 (3)	OM3 (3)	3.184	0.0845	NA
Differences in gene diversity (Shannon)	ANOVA	PP	O	nifH	OM0 (3)	OM1 (3)	OM2 (3)	NA	1.696	0.2608	NA
Differences in gene diversity (Shannon)	ANOVA	PP	M	nirS	OM0 (3)	OM1 (3)	OM2 (3)	OM3 (3)	2.317	0.1522	NA
Differences in gene diversity (Shannon)	ANOVA	PP	O	nirS	OM0 (3)	OM1 (3)	OM2 (3)	NA	1.372	0.3231	NA
Differences in gene diversity (Shannon)	ANOVA	PP	M	nirK	OM0 (3)	OM1 (3)	OM2 (3)	OM3 (3)	10.492	3.80E-03	OM0<OM1, OM0<OM2, OM0<OM3
Differences in gene diversity (Shannon)	ANOVA	PP	O	nirK	OM0 (3)	OM1 (3)	OM2 (3)	NA	9.503	0.0138	OM0<OM2, OM1<OM2

Differences in gene diversity (Shannon)	ANOVA	PP	M	norB	OM0 (3)	OM1 (3)	OM2 (3)	OM3 (3)	6.478	0.0156	OM0<OM1, OM0<OM2
Differences in gene diversity (Shannon)	ANOVA	PP	O	norB	OM0 (3)	OM1 (3)	OM2 (3)	NA	6.463	0.0319	OM0<OM2
Differences in gene diversity (Shannon)	ANOVA	PP	M	nosZ	OM0 (3)	OM1 (3)	OM2 (3)	OM3 (3)	1.027	0.4305	NA
Differences in gene diversity (Shannon)	ANOVA	PP	O	nosZ	OM0 (3)	OM1 (3)	OM2 (3)	NA	22.627	1.60E-03	OM0<OM1, OM0<OM2
Differences in gene diversity (Shannon)	ANOVA	PP	M	nrfA	OM0 (3)	OM1 (3)	OM2 (3)	OM3 (3)	2.295	0.1546	NA
Differences in gene diversity (Shannon)	ANOVA	PP	O	nrfA	OM0 (3)	OM1 (3)	OM2 (3)	NA	5.38	0.0459	OM0<OM2
Differences in gene diversity (Shannon)	ANOVA	JP	M	amoAA	OM0 (3)	OM1 (3)	OM2 (3)	OM3 (3)	0.692	0.582	NA
Differences in gene diversity (Shannon)	ANOVA	JP	O	amoAA	OM0 (3)	OM1 (3)	OM2 (3)	NA	NA	NA	NA
Differences in gene diversity (Shannon)	ANOVA	JP	M	amoAB	OM0 (3)	OM1 (3)	OM2 (3)	OM3 (3)	1.41	0.3092	NA
Differences in gene diversity (Shannon)	ANOVA	JP	O	amoAB	OM0 (3)	OM1 (3)	OM2 (3)	NA	1.149	0.378	NA
Differences in gene diversity (Shannon)	ANOVA	JP	M	nifH	OM0 (3)	OM1 (3)	OM2 (3)	OM3 (3)	2.631	0.1218	NA
Differences in gene diversity (Shannon)	ANOVA	JP	O	nifH	OM0 (3)	OM1 (3)	OM2 (3)	NA	12.176	7.73E-03	OM0<OM1, OM0<OM2
Differences in gene diversity (Shannon)	ANOVA	JP	M	nirS	OM0 (3)	OM1 (3)	OM2 (3)	OM3 (3)	1.801	0.2248	NA
Differences in gene diversity (Shannon)	ANOVA	JP	O	nirS	OM0 (3)	OM1 (3)	OM2 (3)	NA	2.371	0.1742	NA
Differences in gene diversity (Shannon)	ANOVA	JP	M	nirK	OM0 (3)	OM1 (3)	OM2 (3)	OM3 (3)	2.303	0.1537	NA
Differences in gene diversity (Shannon)	ANOVA	JP	O	nirK	OM0 (3)	OM1 (3)	OM2 (3)	NA	0.971	0.4311	NA
Differences in gene diversity (Shannon)	ANOVA	JP	M	norB	OM0 (3)	OM1 (3)	OM2 (3)	OM3 (3)	1.178	0.3771	NA
Differences in gene diversity (Shannon)	ANOVA	JP	O	norB	OM0 (3)	OM1 (3)	OM2 (3)	NA	4.527	0.0633	NA
Differences in gene diversity (Shannon)	ANOVA	JP	M	nosZ	OM0 (3)	OM1 (3)	OM2 (3)	OM3 (3)	0.68	0.5884	NA
Differences in gene diversity (Shannon)	ANOVA	JP	O	nosZ	OM0 (3)	OM1 (3)	OM2 (3)	NA	3.09	0.1196	NA
Differences in gene diversity (Shannon)	ANOVA	JP	M	nrfA	OM0 (3)	OM1 (3)	OM2 (3)	OM3 (3)	8.915	6.25E-03	OM0>OM3, OM1>OM3
Differences in gene diversity (Shannon)	ANOVA	JP	O	nrfA	OM0 (3)	OM1 (3)	OM2 (3)	NA	0.247	0.789	NA
Differences in gene diversity (Shannon)	ANOVA	IDF	M	amoAA	OM0 (3)	OM1 (3)	OM2 (3)	OM3 (3)	2.419	0.1413	NA
Differences in gene diversity (Shannon)	ANOVA	IDF	O	amoAA	OM0 (3)	OM1 (3)	OM2 (3)	NA	1	0.4219	NA
Differences in gene diversity (Shannon)	ANOVA	IDF	M	amoAB	OM0 (3)	OM1 (3)	OM2 (3)	OM3 (3)	0.798	0.5289	NA
Differences in gene diversity (Shannon)	ANOVA	IDF	O	amoAB	OM0 (3)	OM1 (3)	OM2 (3)	NA	1.846	0.2373	NA
Differences in gene diversity (Shannon)	ANOVA	IDF	M	nifH	OM0 (3)	OM1 (3)	OM2 (3)	OM3 (3)	6.61	0.0147	OM0<OM1, OM1>OM2, OM1>OM3
Differences in gene diversity (Shannon)	ANOVA	IDF	O	nifH	OM0 (3)	OM1 (3)	OM2 (3)	NA	17.112	3.32E-03	OM0<OM1, OM1>OM2
Differences in gene diversity (Shannon)	ANOVA	IDF	M	nirS	OM0 (3)	OM1 (3)	OM2 (3)	OM3 (3)	4.399	0.0417	OM0<OM2
Differences in gene diversity (Shannon)	ANOVA	IDF	O	nirS	OM0 (3)	OM1 (3)	OM2 (3)	NA	1.5	0.2963	NA
Differences in gene diversity (Shannon)	ANOVA	IDF	M	nirK	OM0 (3)	OM1 (3)	OM2 (3)	OM3 (3)	3.096	0.0894	NA
Differences in gene diversity (Shannon)	ANOVA	IDF	O	nirK	OM0 (3)	OM1 (3)	OM2 (3)	NA	2.865	0.1338	NA
Differences in gene diversity (Shannon)	ANOVA	IDF	M	norB	OM0 (3)	OM1 (3)	OM2 (3)	OM3 (3)	4.644	0.0366	OM0<OM1
Differences in gene diversity (Shannon)	ANOVA	IDF	O	norB	OM0 (3)	OM1 (3)	OM2 (3)	NA	1.551	0.2864	NA
Differences in gene diversity (Shannon)	ANOVA	IDF	M	nosZ	OM0 (3)	OM1 (3)	OM2 (3)	OM3 (3)	5.076	0.0295	OM0<OM1
Differences in gene diversity (Shannon)	ANOVA	IDF	O	nosZ	OM0 (3)	OM1 (3)	OM2 (3)	NA	4.78	0.0573	NA
Differences in gene diversity (Shannon)	ANOVA	IDF	M	nrfA	OM0 (3)	OM1 (3)	OM2 (3)	OM3 (3)	1.23	0.3605	NA
Differences in gene diversity (Shannon)	ANOVA	IDF	O	nrfA	OM0 (3)	OM1 (3)	OM2 (3)	NA	4.93	0.0541	NA
Differences in gene diversity (Shannon)	ANOVA	LP	M	amoAA	OM0 (3)	OM1 (3)	OM2 (3)	OM3 (2)	1.215	0.3727	NA
Differences in gene diversity (Shannon)	ANOVA	LP	O	amoAA	OM0 (3)	OM1 (3)	OM2 (3)	OM3 (3)	1	0.4411	NA
Differences in gene diversity (Shannon)	ANOVA	LP	M	amoAB	OM0 (3)	OM1 (3)	OM2 (3)	OM3 (2)	2.802	0.1182	NA
Differences in gene diversity (Shannon)	ANOVA	LP	O	amoAB	OM0 (3)	OM1 (3)	OM2 (3)	OM3 (3)	1.997	0.1931	NA
Differences in gene diversity (Shannon)	ANOVA	LP	M	nifH	OM0 (3)	OM1 (3)	OM2 (3)	OM3 (2)	5.088	0.0352	OM1<OM3
Differences in gene diversity (Shannon)	ANOVA	LP	O	nifH	OM0 (3)	OM1 (3)	OM2 (3)	OM3 (3)	20.605	4.04E-04	OM0<OM3, OM1<OM3, OM2<OM3
Differences in gene diversity (Shannon)	ANOVA	LP	M	nirS	OM0 (3)	OM1 (3)	OM2 (3)	OM3 (2)	0.358	0.7853	NA
Differences in gene diversity (Shannon)	ANOVA	LP	O	nirS	OM0 (3)	OM1 (3)	OM2 (3)	OM3 (3)	4.63	0.0369	NA

Differences in gene diversity (Shannon)	ANOVA	LP	M	nirK	OM0 (3)	OM1 (3)	OM2 (3)	OM3 (2)	0.267	0.8472	NA
Differences in gene diversity (Shannon)	ANOVA	LP	O	nirK	OM0 (3)	OM1 (3)	OM2 (3)	OM3 (3)	3.499	0.0695	NA
Differences in gene diversity (Shannon)	ANOVA	LP	M	norB	OM0 (3)	OM1 (3)	OM2 (3)	OM3 (2)	0.284	0.8355	NA
Differences in gene diversity (Shannon)	ANOVA	LP	O	norB	OM0 (3)	OM1 (3)	OM2 (3)	OM3 (3)	3.19	0.0842	NA
Differences in gene diversity (Shannon)	ANOVA	LP	M	nosZ	OM0 (3)	OM1 (3)	OM2 (3)	OM3 (2)	2.941	0.1084	NA
Differences in gene diversity (Shannon)	ANOVA	LP	O	nosZ	OM0 (3)	OM1 (3)	OM2 (3)	OM3 (3)	4.043	0.0507	NA
Differences in gene diversity (Shannon)	ANOVA	LP	M	nrfA	OM0 (3)	OM1 (3)	OM2 (3)	OM3 (2)	0.906	0.4848	NA
Differences in gene diversity (Shannon)	ANOVA	LP	O	nrfA	OM0 (3)	OM1 (3)	OM2 (3)	OM3 (3)	1.574	0.2699	NA

PERMANOVA tests

All using 999 permutations

Test	Layers	Ecozone	Genes or CAZy classes	Model formula	F-value term 1	F-value term 2	F-value term 3	R2 term 1	R2 term 2	R2 term 3	R2 residual	P-value term1	P-value term 2	P-value term 3
PERMANOVA	Both	All but IDF	All CAZy	~ Soil layer + OM removal + Ecozone	91.255	3.082	30.467	0.3381	0.03426	0.33865	0.28899	0.001	0.003	0.001
PERMANOVA	Both	All but IDF	AA	~ Soil layer + OM removal + Ecozone	24.482	3.752	34.116	0.11329	0.05209	0.47365	0.36097	0.001	0.003	0.001
PERMANOVA	Both	All but IDF	CE	~ Soil layer + OM removal + Ecozone	73.708	3.05	25.057	0.31229	0.03876	0.31848	0.33047	0.001	0.006	0.001
PERMANOVA	Both	All but IDF	GH	~ Soil layer + OM removal + Ecozone	174.829	2.762	32.026	0.48945	0.0232	0.26898	0.21837	0.001	0.019	0.001
PERMANOVA	Both	All but IDF	GT	~ Soil layer + OM removal + Ecozone	14.4318	3.3918	28.0261	0.07731	0.05451	0.45037	0.41781	0.001	0.003	0.001
PERMANOVA	Both	All but IDF	PL	~ Soil layer + OM removal + Ecozone	107.256	2.343	46.186	0.32419	0.02124	0.4188	0.23577	0.001	0.04	0.001
PERMANOVA	Both	BS	AA	~ Soil layer	7.634	-	-	0.28663	-	-	0.71337	1	-	-
PERMANOVA	Mineral	BS	AA	~ OM removal	0.51894	-	-	0.1629	-	-	0.8371	0.69	-	-
PERMANOVA	Organic	BS	AA	~ OM removal	1.1172	-	-	0.27135	-	-	0.72865	0.391	-	-
PERMANOVA	Both	PP	AA	~ Soil layer	3.1134	-	-	0.14079	-	-	0.85921	1	-	-
PERMANOVA	Mineral	PP	AA	~ OM removal	12.388	-	-	0.82287	-	-	0.17713	0.001	-	-
PERMANOVA	Organic	PP	AA	~ OM removal	3.5907	-	-	0.54481	-	-	0.45519	0.013	-	-
PERMANOVA	Both	JP	AA	~ Soil layer	16.39	-	-	0.46312	-	-	0.53688	1	-	-
PERMANOVA	Mineral	JP	AA	~ OM removal	0.978	-	-	0.26834	-	-	0.73166	0.48	-	-
PERMANOVA	Organic	JP	AA	~ OM removal	0.61434	-	-	0.16997	-	-	0.83003	0.694	-	-
PERMANOVA	Both	IDF	AA	~ Soil layer	48.85	-	-	0.71997	-	-	0.28003	1	-	-
PERMANOVA	Mineral	IDF	AA	~ OM removal	3.2178	-	-	0.54683	-	-	0.45317	0.045	-	-
PERMANOVA	Organic	IDF	AA	~ OM removal	13.487	-	-	0.81804	-	-	0.18196	0.014	-	-
PERMANOVA	Both	LP	AA	~ Soil layer	22.36	-	-	0.51568	-	-	0.48432	1	-	-
PERMANOVA	Mineral	LP	AA	~ OM removal	1.1558	-	-	0.33127	-	-	0.66873	0.391	-	-
PERMANOVA	Organic	LP	AA	~ OM removal	0.75637	-	-	0.22097	-	-	0.77903	0.642	-	-
PERMANOVA	Both	BS	CE	~ Soil layer	71.602	-	-	0.79029	-	-	0.20971	1	-	-
PERMANOVA	Mineral	BS	CE	~ OM removal	0.93886	-	-	0.26039	-	-	0.73961	0.465	-	-
PERMANOVA	Organic	BS	CE	~ OM removal	5.3543	-	-	0.6409	-	-	0.3591	0.004	-	-
PERMANOVA	Both	PP	CE	~ Soil layer	17.466	-	-	0.47897	-	-	0.52103	1	-	-
PERMANOVA	Mineral	PP	CE	~ OM removal	3.7178	-	-	0.58232	-	-	0.41768	0.013	-	-
PERMANOVA	Organic	PP	CE	~ OM removal	0.52147	-	-	0.14808	-	-	0.85192	0.82	-	-
PERMANOVA	Both	JP	CE	~ Soil layer	43.572	-	-	0.69635	-	-	0.30365	1	-	-
PERMANOVA	Mineral	JP	CE	~ OM removal	1.4502	-	-	0.35225	-	-	0.64775	0.269	-	-
PERMANOVA	Organic	JP	CE	~ OM removal	0.60099	-	-	0.16689	-	-	0.83311	0.634	-	-
PERMANOVA	Both	IDF	CE	~ Soil layer	38.475	-	-	0.66942	-	-	0.33058	1	-	-
PERMANOVA	Mineral	IDF	CE	~ OM removal	5.0337	-	-	0.6537	-	-	0.3463	0.009	-	-
PERMANOVA	Organic	IDF	CE	~ OM removal	14.275	-	-	0.82634	-	-	0.17366	0.006	-	-
PERMANOVA	Both	LP	CE	~ Soil layer	10.277	-	-	0.32858	-	-	0.67142	1	-	-

PERMANOVA	Mineral	LP	CE	~ OM removal	3.7602	-	-	0.61708	-	-	0.38292	0.009	-	-
PERMANOVA	Organic	LP	CE	~ OM removal	0.75227	-	-	0.22003	-	-	0.77997	0.632	-	-
PERMANOVA	Both	BS	GH	~ Soil layer	58.698	-	-	0.75546	-	-	0.24454	1	-	-
PERMANOVA	Mineral	BS	GH	~ OM removal	0.76248	-	-	0.22235	-	-	0.77765	0.665	-	-
PERMANOVA	Organic	BS	GH	~ OM removal	3.5663	-	-	0.54312	-	-	0.45688	0.004	-	-
PERMANOVA	Both	PP	GH	~ Soil layer	16.971	-	-	0.47179	-	-	0.52821	1	-	-
PERMANOVA	Mineral	PP	GH	~ OM removal	1.9668	-	-	0.42447	-	-	0.57553	0.046	-	-
PERMANOVA	Organic	PP	GH	~ OM removal	1.176	-	-	0.2816	-	-	0.7184	0.331	-	-
PERMANOVA	Both	JP	GH	~ Soil layer	36.9	-	-	0.66011	-	-	0.33989	1	-	-
PERMANOVA	Mineral	JP	GH	~ OM removal	0.4969	-	-	0.15707	-	-	0.84293	0.772	-	-
PERMANOVA	Organic	JP	GH	~ OM removal	1.9123	-	-	0.38928	-	-	0.61072	0.21	-	-
PERMANOVA	Both	IDF	GH	~ Soil layer	29.682	-	-	0.60971	-	-	0.39029	1	-	-
PERMANOVA	Mineral	IDF	GH	~ OM removal	22.317	-	-	0.89326	-	-	0.10674	0.001	-	-
PERMANOVA	Organic	IDF	GH	~ OM removal	21.346	-	-	0.87678	-	-	0.12322	0.003	-	-
PERMANOVA	Both	LP	GH	~ Soil layer	53.251	-	-	0.71718	-	-	0.28282	1	-	-
PERMANOVA	Mineral	LP	GH	~ OM removal	2.7235	-	-	0.53858	-	-	0.46142	0.067	-	-
PERMANOVA	Organic	LP	GH	~ OM removal	1.9016	-	-	0.41626	-	-	0.58374	0.069	-	-
PERMANOVA	Both	BS	GT	~ Soil layer	17.575	-	-	0.48051	-	-	0.51949	1	-	-
PERMANOVA	Mineral	BS	GT	~ OM removal	0.92285	-	-	0.2571	-	-	0.7429	0.511	-	-
PERMANOVA	Organic	BS	GT	~ OM removal	6.3639	-	-	0.67962	-	-	0.32038	0.027	-	-
PERMANOVA	Both	PP	GT	~ Soil layer	5.1092	-	-	0.21192	-	-	0.78808	1	-	-
PERMANOVA	Mineral	PP	GT	~ OM removal	3.039	-	-	0.53263	-	-	0.46737	0.051	-	-
PERMANOVA	Organic	PP	GT	~ OM removal	8.9286	-	-	0.7485	-	-	0.2515	0.005	-	-
PERMANOVA	Both	JP	GT	~ Soil layer	5.5901	-	-	0.22733	-	-	0.77267	1	-	-
PERMANOVA	Mineral	JP	GT	~ OM removal	0.66327	-	-	0.19918	-	-	0.80082	0.691	-	-
PERMANOVA	Organic	JP	GT	~ OM removal	0.69619	-	-	0.18835	-	-	0.81165	0.543	-	-
PERMANOVA	Both	IDF	GT	~ Soil layer	32.556	-	-	0.63147	-	-	0.36853	1	-	-
PERMANOVA	Mineral	IDF	GT	~ OM removal	6.2398	-	-	0.70059	-	-	0.29941	0.001	-	-
PERMANOVA	Organic	IDF	GT	~ OM removal	42.52	-	-	0.93409	-	-	0.06591	0.006	-	-
PERMANOVA	Both	LP	GT	~ Soil layer	4.7374	-	-	0.18407	-	-	0.81593	1	-	-
PERMANOVA	Mineral	LP	GT	~ OM removal	5.9717	-	-	0.71905	-	-	0.28095	0.001	-	-
PERMANOVA	Organic	LP	GT	~ OM removal	0.91966	-	-	0.25643	-	-	0.74357	0.494	-	-
PERMANOVA	Both	BS	PL	~ Soil layer	18.574	-	-	0.49433	-	-	0.50567	1	-	-
PERMANOVA	Mineral	BS	PL	~ OM removal	0.50893	-	-	0.16026	-	-	0.83974	0.912	-	-
PERMANOVA	Organic	BS	PL	~ OM removal	4.2597	-	-	0.58676	-	-	0.41324	0.034	-	-
PERMANOVA	Both	PP	PL	~ Soil layer	12.152	-	-	0.39009	-	-	0.60991	1	-	-
PERMANOVA	Mineral	PP	PL	~ OM removal	7.679	-	-	0.74224	-	-	0.25776	0.001	-	-
PERMANOVA	Organic	PP	PL	~ OM removal	2.5186	-	-	0.45638	-	-	0.54362	0.022	-	-
PERMANOVA	Both	JP	PL	~ Soil layer	37.297	-	-	0.66251	-	-	0.33749	1	-	-
PERMANOVA	Mineral	JP	PL	~ OM removal	1.9877	-	-	0.42706	-	-	0.57294	0.144	-	-
PERMANOVA	Organic	JP	PL	~ OM removal	1.8111	-	-	0.37644	-	-	0.62356	0.159	-	-

PERMANOVA	Both	IDF	PL	~ Soil layer	109.72	-	-	0.85239	-	-	0.14761	1	-	-
PERMANOVA	Mineral	IDF	PL	~ OM removal	2.4402	-	-	0.47783	-	-	0.52217	0.052	-	-
PERMANOVA	Organic	IDF	PL	~ OM removal	5.3808	-	-	0.64204	-	-	0.35796	0.013	-	-
PERMANOVA	Both	LP	PL	~ Soil layer	11.965	-	-	0.36296	-	-	0.63704	1	-	-
PERMANOVA	Mineral	LP	PL	~ OM removal	0.72761	-	-	0.23771	-	-	0.76229	0.543	-	-
PERMANOVA	Organic	LP	PL	~ OM removal	1.5355			0.36541	-	-	0.63459	0.223	-	-
PERMANOVA	Both	All but IDF	amoA(A)	~ Soil layer + Ecozone + OM removal	4.0478	3.0387	1.1225	0.04193	0.09444	0.03489	0.82874	0.001	0.001	0.209
PERMANOVA	Both	All but IDF	amoA(B)	~ Soil layer + Ecozone + OM removal	113.563	11.481	0.337	0.49587	0.15039	0.00442	0.34932	0.001	0.001	0.859
PERMANOVA	Both	All but IDF	nifH	~ Soil layer + Ecozone + OM removal	189.658	13.301	1.14	0.60597	0.12749	0.01093	0.25561	0.001	0.001	0.343
PERMANOVA	Both	All but IDF	nirK	~ Soil layer + Ecozone + OM removal	76.803	4.645	1.173	0.44075	0.07997	0.02019	0.45909	0.001	0.002	0.308
PERMANOVA	Both	All but IDF	nirS	~ Soil layer + Ecozone + OM removal	7.6385	5.7701	1.8582	0.06911	0.15662	0.05044	0.72383	0.001	0.001	0.056
PERMANOVA	Both	All but IDF	norB	~ Soil layer + Ecozone + OM removal	32.428	24.439	1.367	0.17081	0.38619	0.0216	0.4214	0.001	0.001	0.202
PERMANOVA	Both	All but IDF	nosZ	~ Soil layer + Ecozone + OM removal	4.8404	3.4523	1.6581	0.04832	0.10339	0.04966	0.79863	0.001	0.001	0.046
PERMANOVA	Both	All but IDF	nrfA	~ Soil layer + Ecozone + OM removal	15.0123	21.802	2.3255	0.08968	0.39073	0.04168	0.47791	0.001	0.001	0.025
PERMANOVA	Both	BS	amoA(A)	~ Soil layer	1.5555	-	-	0.07568	-	-	0.92432	1	-	-
PERMANOVA	Mineral	BS	amoA(A)	~ OM removal	1.0128	-	-	0.27525	-	-	0.72475	0.42	-	-
PERMANOVA	Organic	BS	amoA(A)	~ OM removal	1	-	-	0.25	-	-	0.75	1	-	-
PERMANOVA	Both	PP	amoA(A)	~ Soil layer	2.0439	-	-	0.0887	-	-	0.9113	1	-	-
PERMANOVA	Mineral	PP	amoA(A)	~ OM removal	2.4195	-	-	0.42057	-	-	0.57943	0.029	-	-
PERMANOVA	Organic	PP	amoA(A)	~ OM removal	4.8	-	-	0.61538	-	-	0.38462	0.009	-	-
PERMANOVA	Both	JP	amoA(A)	~ Soil layer	19.666	-	-	0.50861	-	-	0.49139	1	-	-
PERMANOVA	Mineral	JP	amoA(A)	~ OM removal	1.7021	-	-	0.3896	-	-	0.6104	0.068	-	-
PERMANOVA	Organic	JP	amoA(A)	~ OM removal	0.82468	-	-	0.21562	-	-	0.78438	0.662	-	-
PERMANOVA	Both	IDF	amoA(A)	~ Soil layer	14.358	-	-	0.43043	-	-	0.56957	1	-	-
PERMANOVA	Mineral	IDF	amoA(A)	~ OM removal	2.4275	-	-	0.47652	-	-	0.52348	0.007	-	-
PERMANOVA	Organic	IDF	amoA(A)	~ OM removal	2.6386	-	-	0.46795	-	-	0.53205	0.044	-	-
PERMANOVA	Both	LP	amoA(A)	~ Soil layer	29.58	-	-	0.58482	-	-	0.41518	1	-	-
PERMANOVA	Mineral	LP	amoA(A)	~ OM removal	1.5111	-	-	0.39305	-	-	0.60695	0.177	-	-
PERMANOVA	Organic	LP	amoA(A)	~ OM removal	1.3954	-	-	0.34351	-	-	0.65649	0.287	-	-
PERMANOVA	Both	BS	amoA(B)	~ Soil layer	41.207	-	-	0.68442	-	-	0.31558	1	-	-
PERMANOVA	Mineral	BS	amoA(B)	~ OM removal	1.0782	-	-	0.28791	-	-	0.71209	0.415	-	-
PERMANOVA	Organic	BS	amoA(B)	~ OM removal	1.5623	-	-	0.34243	-	-	0.65757	0.198	-	-
PERMANOVA	Both	PP	amoA(B)	~ Soil layer	14.325	-	-	0.40553	-	-	0.59447	1	-	-
PERMANOVA	Mineral	PP	amoA(B)	~ OM removal	2.6395	-	-	0.44192	-	-	0.55808	0.041	-	-
PERMANOVA	Organic	PP	amoA(B)	~ OM removal	1.3476	-	-	0.30997	-	-	0.69003	0.061	-	-
PERMANOVA	Both	JP	amoA(B)	~ Soil layer	72.571	-	-	0.79251	-	-	0.20749	1	-	-
PERMANOVA	Mineral	JP	amoA(B)	~ OM removal	2.2346	-	-	0.45592	-	-	0.54408	0.143	-	-
PERMANOVA	Organic	JP	amoA(B)	~ OM removal	2.8544	-	-	0.48756	-	-	0.51244	0.042	-	-
PERMANOVA	Both	IDF	amoA(B)	~ Soil layer	6.3276	-	-	0.24983	-	-	0.75017	1	-	-
PERMANOVA	Mineral	IDF	amoA(B)	~ OM removal	1.0136	-	-	0.27541	-	-	0.72459	0.426	-	-
PERMANOVA	Organic	IDF	amoA(B)	~ OM removal	1.0446	-	-	0.25826	-	-	0.74174	0.442	-	-

PERMANOVA	Both	LP	amoA(B)	~ Soil layer	114.29	-	-	0.84478	-	-	0.15522	1	-	-
PERMANOVA	Mineral	LP	amoA(B)	~ OM removal	0.91989	-	-	0.28276	-	-	0.71724	0.541	-	-
PERMANOVA	Organic	LP	amoA(B)	~ OM removal	0.91906	-	-	0.25631	-	-	0.74369	0.495	-	-
PERMANOVA	Both	BS	nifH	~ Soil layer	154.14	-	-	0.89026	-	-	0.10974	1	-	-
PERMANOVA	Mineral	BS	nifH	~ OM removal	0.82284	-	-	0.2358	-	-	0.7642	0.521	-	-
PERMANOVA	Organic	BS	nifH	~ OM removal	0.58465	-	-	0.1631	-	-	0.8369	0.826	-	-
PERMANOVA	Both	PP	nifH	~ Soil layer	9.6608	-	-	0.31509	-	-	0.68491	1	-	-
PERMANOVA	Mineral	PP	nifH	~ OM removal	4.4235	-	-	0.57027	-	-	0.42973	0.028	-	-
PERMANOVA	Organic	PP	nifH	~ OM removal	0.52218	-	-	0.14826	-	-	0.85174	0.653	-	-
PERMANOVA	Both	JP	nifH	~ Soil layer	126.24	-	-	0.86918	-	-	0.13082	1	-	-
PERMANOVA	Mineral	JP	nifH	~ OM removal	1.0161	-	-	0.2759	-	-	0.7241	0.494	-	-
PERMANOVA	Organic	JP	nifH	~ OM removal	0.38128	-	-	0.11276	-	-	0.88724	0.794	-	-
PERMANOVA	Both	IDF	nifH	~ Soil layer	31.657	-	-	0.62493	-	-	0.37507	1	-	-
PERMANOVA	Mineral	IDF	nifH	~ OM removal	1.2548	-	-	0.31998	-	-	0.68002	0.164	-	-
PERMANOVA	Organic	IDF	nifH	~ OM removal	3.4529	-	-	0.5351	-	-	0.4649	0.042	-	-
PERMANOVA	Both	LP	nifH	~ Soil layer	144.38	-	-	0.87302	-	-	0.12698	1	-	-
PERMANOVA	Mineral	LP	nifH	~ OM removal	1.9152	-	-	0.45079	-	-	0.54921	0.211	-	-
PERMANOVA	Organic	LP	nifH	~ OM removal	3.6926	-	-	0.58067	-	-	0.41933	0.01	-	-
PERMANOVA	Both	BS	nirS	~ Soil layer	7.4606	-	-	0.28195	-	-	0.71805	1	-	-
PERMANOVA	Mineral	BS	nirS	~ OM removal	0.51017	-	-	0.16059	-	-	0.83941	0.959	-	-
PERMANOVA	Organic	BS	nirS	~ OM removal	2.0716	-	-	0.40847	-	-	0.59153	0.14	-	-
PERMANOVA	Both	PP	nirS	~ Soil layer	0.73289	-	-	0.03372	-	-	0.96628	1	-	-
PERMANOVA	Mineral	PP	nirS	~ OM removal	2.6466	-	-	0.44258	-	-	0.55742	0.022	-	-
PERMANOVA	Organic	PP	nirS	~ OM removal	1.2162	-	-	0.28845	-	-	0.71155	0.423	-	-
PERMANOVA	Both	JP	nirS	~ Soil layer	7.7565	-	-	0.28989	-	-	0.71011	1	-	-
PERMANOVA	Mineral	JP	nirS	~ OM removal	1.2471	-	-	0.31864	-	-	0.68136	0.379	-	-
PERMANOVA	Organic	JP	nirS	~ OM removal	1.4619	-	-	0.32764	-	-	0.67236	0.306	-	-
PERMANOVA	Both	IDF	nirS	~ Soil layer	1.3255	-	-	0.06521	-	-	0.93479	1	-	-
PERMANOVA	Mineral	IDF	nirS	~ OM removal	2.0423	-	-	0.43371	-	-	0.56629	0.185	-	-
PERMANOVA	Organic	IDF	nirS	~ OM removal	4.3269	-	-	0.59055	-	-	0.40945	0.055	-	-
PERMANOVA	Both	LP	nirS	~ Soil layer	5.2347	-	-	0.19953	-	-	0.80047	1	-	-
PERMANOVA	Mineral	LP	nirS	~ OM removal	1.7245	-	-	0.42498	-	-	0.57502	0.153	-	-
PERMANOVA	Organic	LP	nirS	~ OM removal	0.70208	-	-	0.20841	-	-	0.79159	0.702	-	-
PERMANOVA	Both	BS	nirK	~ Soil layer	57.579	-	-	0.75189	-	-	0.24811	1	-	-
PERMANOVA	Mineral	BS	nirK	~ OM removal	0.65213	-	-	0.1965	-	-	0.8035	0.804	-	-
PERMANOVA	Organic	BS	nirK	~ OM removal	2.5444	-	-	0.45891	-	-	0.54109	0.116	-	-
PERMANOVA	Both	PP	nirK	~ Soil layer	2.3628	-	-	0.10113	-	-	0.89887	1	-	-
PERMANOVA	Mineral	PP	nirK	~ OM removal	1.8504	-	-	0.35697	-	-	0.64303	0.125	-	-
PERMANOVA	Organic	PP	nirK	~ OM removal	8.941	-	-	0.74877	-	-	0.25123	0.02	-	-
PERMANOVA	Both	JP	nirK	~ Soil layer	40.828	-	-	0.68242	-	-	0.31758	1	-	-
PERMANOVA	Mineral	JP	nirK	~ OM removal	2.4596	-	-	0.47981	-	-	0.52019	0.046	-	-

PERMANOVA	Organic	JP	nirK	~ OM removal	0.60586	-	-	0.16802	-	-	0.83198	0.598	-	-
PERMANOVA	Both	IDF	nirK	~ Soil layer	16.071	-	-	0.45825	-	-	0.54175	1	-	-
PERMANOVA	Mineral	IDF	nirK	~ OM removal	0.87461	-	-	0.24698	-	-	0.75302	0.564	-	-
PERMANOVA	Organic	IDF	nirK	~ OM removal	2.7542	-	-	0.47865	-	-	0.52135	0.009	-	-
PERMANOVA	Both	LP	nirK	~ Soil layer	50.599	-	-	0.7067	-	-	0.2933	1	-	-
PERMANOVA	Mineral	LP	nirK	~ OM removal	1.6212	-	-	0.40996	-	-	0.59004	0.207	-	-
PERMANOVA	Organic	LP	nirK	~ OM removal	2.2435	-	-	0.45691	-	-	0.54309	0.06	-	-
PERMANOVA	Both	BS	norB	~ Soil layer	15.784	-	-	0.45377	-	-	0.54623	1	-	-
PERMANOVA	Mineral	BS	norB	~ OM removal	0.63466	-	-	0.19224	-	-	0.80776	0.788	-	-
PERMANOVA	Organic	BS	norB	~ OM removal	1.4505	-	-	0.32592	-	-	0.67408	0.018	-	-
PERMANOVA	Both	PP	norB	~ Soil layer	2.1688	-	-	0.09361	-	-	0.90639	1	-	-
PERMANOVA	Mineral	PP	norB	~ OM removal	4.9839	-	-	0.59923	-	-	0.40077	0.001	-	-
PERMANOVA	Organic	PP	norB	~ OM removal	3.1742	-	-	0.51411	-	-	0.48589	0.015	-	-
PERMANOVA	Both	JP	norB	~ Soil layer	51.91	-	-	0.73205	-	-	0.26795	1	-	-
PERMANOVA	Mineral	JP	norB	~ OM removal	1.3752	-	-	0.34024	-	-	0.65976	0.183	-	-
PERMANOVA	Organic	JP	norB	~ OM removal	0.48349	-	-	0.1388	-	-	0.8612	0.86	-	-
PERMANOVA	Both	IDF	norB	~ Soil layer	9.9519	-	-	0.34374	-	-	0.65626	1	-	-
PERMANOVA	Mineral	IDF	norB	~ OM removal	1.0414	-	-	0.28085	-	-	0.71915	0.403	-	-
PERMANOVA	Organic	IDF	norB	~ OM removal	0.9188	-	-	0.23446	-	-	0.76554	0.466	-	-
PERMANOVA	Both	LP	norB	~ Soil layer	28.832	-	-	0.57858	-	-	0.42142	1	-	-
PERMANOVA	Mineral	LP	norB	~ OM removal	2.9733	-	-	0.5603	-	-	0.4397	0.069	-	-
PERMANOVA	Organic	LP	norB	~ OM removal	2.9437	-	-	0.52469	-	-	0.47531	0.035	-	-
PERMANOVA	Both	BS	nosZ	~ Soil layer	2.5983	-	-	0.1203	-	-	0.8797	1	-	-
PERMANOVA	Mineral	BS	nosZ	~ OM removal	0.79273	-	-	0.22915	-	-	0.77085	0.683	-	-
PERMANOVA	Organic	BS	nosZ	~ OM removal	0.95221	-	-	0.24093	-	-	0.75907	0.558	-	-
PERMANOVA	Both	PP	nosZ	~ Soil layer	1.9588	-	-	0.08532	-	-	0.91468	1	-	-
PERMANOVA	Mineral	PP	nosZ	~ OM removal	2.1786	-	-	0.39526	-	-	0.60474	0.01	-	-
PERMANOVA	Organic	PP	nosZ	~ OM removal	3.885	-	-	0.56427	-	-	0.43573	0.001	-	-
PERMANOVA	Both	JP	nosZ	~ Soil layer	12.171	-	-	0.39047	-	-	0.60953	1	-	-
PERMANOVA	Mineral	JP	nosZ	~ OM removal	1.0123	-	-	0.27516	-	-	0.72484	0.473	-	-
PERMANOVA	Organic	JP	nosZ	~ OM removal	1.1145	-	-	0.27088	-	-	0.72912	0.325	-	-
PERMANOVA	Both	IDF	nosZ	~ Soil layer	13.287	-	-	0.41153	-	-	0.58847	1	-	-
PERMANOVA	Mineral	IDF	nosZ	~ OM removal	1.3859	-	-	0.34198	-	-	0.65802	0.141	-	-
PERMANOVA	Organic	IDF	nosZ	~ OM removal	3.4354	-	-	0.53383	-	-	0.46617	0.007	-	-
PERMANOVA	Both	LP	nosZ	~ Soil layer	6.4609	-	-	0.23528	-	-	0.76472	1	-	-
PERMANOVA	Mineral	LP	nosZ	~ OM removal	1.7145	-	-	0.42355	-	-	0.57645	0.071	-	-
PERMANOVA	Organic	LP	nosZ	~ OM removal	2.379	-	-	0.4715	-	-	0.5285	0.001	-	-
PERMANOVA	Both	BS	nrfA	~ Soil layer	2.0657	-	-	0.09806	-	-	0.90194	1	-	-
PERMANOVA	Mineral	BS	nrfA	~ OM removal	1.0834	-	-	0.28891	-	-	0.71109	0.399	-	-
PERMANOVA	Organic	BS	nrfA	~ OM removal	1.0467	-	-	0.25866	-	-	0.74134	0.107	-	-
PERMANOVA	Both	PP	nrfA	~ Soil layer	2.0439	-	-	0.0887	-	-	0.9113	1	-	-

PERMANOVA	Mineral	PP	nrfA	~ OM removal	2.4195	-	-	0.42057	-	-	0.57943	0.034	-	-
PERMANOVA	Organic	PP	nrfA	~ OM removal	4.8	-	-	0.61538	-	-	0.38462	0.01	-	-
PERMANOVA	Both	JP	nrfA	~ Soil layer	19.666	-	-	0.50861	-	-	0.49139	1	-	-
PERMANOVA	Mineral	JP	nrfA	~ OM removal	1.7021	-	-	0.3896	-	-	0.6104	0.066	-	-
PERMANOVA	Organic	JP	nrfA	~ OM removal	0.82468	-	-	0.21562	-	-	0.78438	0.669	-	-
PERMANOVA	Both	IDF	nrfA	~ Soil layer	14.358	-	-	0.43043	-	-	0.56957	1	-	-
PERMANOVA	Mineral	IDF	nrfA	~ OM removal	2.4275	-	-	0.47652	-	-	0.52348	0.012	-	-
PERMANOVA	Organic	IDF	nrfA	~ OM removal	2.6386	-	-	0.46795	-	-	0.53205	0.057	-	-
PERMANOVA	Both	LP	nrfA	~ Soil layer	29.58	-	-	0.58482	-	-	0.41518	1	-	-
PERMANOVA	Mineral	LP	nrfA	~ OM removal	1.5111	-	-	0.39305	-	-	0.60695	0.199	-	-
PERMANOVA	Organic	LP	nrfA	~ OM removal	1.3954	-	-	0.34351	-	-	0.65649	0.271	-	-

Linear models

Explanatory variable	Response variable	Model intercept	Model slope	R2	Adjusted R2	P-value	Q-value	Layer tested
DNA	AA1	166.2439	10.0656	0.2797	0.2418	0.0137	0.0361949	Both
DNA	AA11	0.4336	0.0707	0.0642	0.0149	0.2678	0.4049304	Both
DNA	AA10	6.525	1.12	0.3791	0.3464	0.003	0.0106317	Both
DNA	AA2	1.5806	0.2809	0.2444	0.2047	0.0227	0.0522931	Both
DNA	AA3	414.0022	-23.8242	0.2159	0.1747	0.0338	0.0719258	Both
DNA	AA4	13.9824	5.3113	0.6192	0.5992	0.00E+00	0.0003881	Both
DNA	AA5	47.5523	-9.2653	0.4892	0.4623	0.0004	0.0025565	Both
DNA	AA8	0.7042	0.1464	0.2422	0.2024	0.0234	0.0534431	Both
DNA	AA7	55.8067	-2.9559	0.1023	0.055	0.1576	0.2586162	Both
DNA	AA6	28.9935	-4.0384	0.5941	0.5728	0.00E+00	0.0005818	Both
DNA	AA9	1.5682	0.3269	0.0815	0.0332	0.2096	0.3329309	Both
DNA	CE1	214.5689	-2.1863	0.0275	-0.0237	0.4725	0.5930061	Both
DNA	CE11	161.0901	0.9011	0.0063	-0.046	0.7329	0.8176444	Both
DNA	CE12	17.1999	3.7724	0.5477	0.5239	0.0001	0.0011543	Both
DNA	CE13	0.0445	0.016	0.189	0.1464	0.0489	0.0981402	Both
DNA	CE14	222.8524	7.5368	0.27	0.2315	0.0158	0.0399433	Both
DNA	CE15	41.9051	8.7621	0.6874	0.671	0.00E+00	9.48E-05	Both
DNA	CE2	2.7115	0.7492	0.3493	0.315	0.0048	0.0153993	Both
DNA	CE3	23.2608	-2.7048	0.0596	0.0101	0.2863	0.4252333	Both
DNA	CE4	354.0802	-36.4889	0.3324	0.2972	0.0062	0.018855	Both
DNA	CE16	1.0609	0.0544	0.0314	-0.0196	0.4423	0.5663811	Both
DNA	CE5	14.6374	-1.7906	0.0914	0.0436	0.1828	0.2941529	Both
DNA	CE7	11.1596	1.9636	0.541	0.5168	0.0001	0.0012141	Both
DNA	CE6	41.6877	7.2471	0.4564	0.4278	0.0008	0.004095	Both
DNA	CE8	22.9784	2.7215	0.3105	0.2742	0.0087	0.0242156	Both
DNA	CE9	93.7508	-0.8781	0.037	-0.0137	0.4035	0.5339961	Both
DNA	GH100	0.8338	0.3228	0.3037	0.267	0.0096	0.0265405	Both
DNA	GH10	54.9015	4.7129	0.4166	0.3859	0.0016	0.0069534	Both
DNA	GH102	40.7964	-3.235	0.4172	0.3865	0.0016	0.0069534	Both
DNA	GH101	0.7299	-0.1179	0.1068	0.0598	0.1482	0.2463953	Both
DNA	GH103	106.2158	-11.5133	0.3332	0.2981	0.0061	0.018855	Both
DNA	GH104	5.1644	-0.3704	0.1185	0.0721	0.1265	0.2160348	Both
DNA	GH105	29.0354	7.3822	0.4695	0.4416	0.0006	0.0034739	Both
DNA	GH106	49.5372	11.7711	0.6248	0.605	0.00E+00	0.0003671	Both
DNA	GH107	0.1563	-0.0363	0.0674	0.0183	0.2557	0.3913452	Both
DNA	GH108	6.355	-0.0321	0.0019	-0.0507	0.8526	0.9068363	Both
DNA	GH109	20.0561	1.1154	0.1952	0.1529	0.0449	0.0909216	Both
DNA	GH11	14.887	2.8643	0.5642	0.5413	1.00E-04	0.000949	Both
DNA	GH110	1.2431	0.2653	0.3263	0.2909	0.0068	0.0199405	Both
DNA	GH111	0.0414	-0.0058	0.051	0.001	0.325	0.4565644	Both
DNA	GH112	0.0997	0.0752	0.5555	0.5321	0.0001	0.001062	Both
DNA	GH113	8.2393	0.2109	0.0137	-0.0382	0.6136	0.7202681	Both
DNA	GH114	10.5907	-1.711	0.2571	0.2179	0.019	0.0462737	Both
DNA	GH115	18.1582	7.4469	0.7576	0.7448	0.00E+00	2.43E-05	Both
DNA	GH116	44.574	5.8979	0.3323	0.2971	0.0062	0.018855	Both
DNA	GH117	3.4772	0.0033	0.00E+00	-0.0526	0.9876	0.9876064	Both
DNA	GH118	0.0029	0.00E+00	0.00E+00	-0.0526	0.9872	0.9876064	Both
DNA	GH119	2.7623	-0.2683	0.054	0.0043	0.3105	0.4479725	Both
DNA	GH12	8.8559	1.854	0.4574	0.4288	0.0008	0.004095	Both
DNA	GH120	1.8739	0.0499	0.0029	-0.0496	0.8176	0.8808012	Both

DNA	GH121	1.5942	-0.2365	0.0359	-0.0148	0.4107	0.5339961	Both
DNA	GH123	1.8672	-0.5954	0.3538	0.3198	0.0045	0.0147025	Both
DNA	GH124	0.0511	-0.0236	0.3438	0.3093	0.0052	0.0163484	Both
DNA	GH125	11.4055	1.6419	0.2502	0.2107	0.0209	0.0495806	Both
DNA	GH126	0.0093	0.0008	0.0057	-0.0466	0.7444	0.8245156	Both
DNA	GH127	44.4763	7.6246	0.6063	0.5855	0.00E+00	0.0004748	Both
DNA	GH128	6.9808	1.5732	0.3877	0.3555	0.0026	0.0096553	Both
DNA	GH129	0.3906	0.0535	0.1222	0.076	0.1204	0.2106233	Both
DNA	GH13	1501.8611	-17.0414	0.0229	-0.0285	0.5122	0.6364771	Both
DNA	GH130	86.8053	9.1843	0.434	0.4042	0.0012	0.0055129	Both
DNA	GH131	0.195	0.0614	0.2475	0.2079	0.0217	0.0509988	Both
DNA	GH132	0.7589	0.14	0.0987	0.0512	0.1656	0.2691107	Both
DNA	GH133	72.2732	-9.3015	0.5677	0.5449	1.00E-04	0.0009171	Both
DNA	GH14	0.5412	0.0893	0.3271	0.2917	0.0067	0.0199266	Both
DNA	GH17	59.077	-4.5981	0.2076	0.1659	0.0379	0.0779739	Both
DNA	GH15	408.8678	4.4959	0.0132	-0.0388	0.6205	0.7210566	Both
DNA	GH18	245.204	13.9525	0.2357	0.1955	0.0257	0.0566841	Both
DNA	GH19	16.9586	-0.0599	0.0003	-0.0523	0.9416	0.9583187	Both
DNA	GH2	388.7654	55.5802	0.6764	0.6594	0.00E+00	0.0001192	Both
DNA	GH20	100.202	4.1078	0.1584	0.1141	0.074	0.1375713	Both
DNA	GH23	392.3336	-9.176	0.0778	0.0292	0.2209	0.3476303	Both
DNA	GH24	15.9645	0.2522	0.0216	-0.0299	0.5254	0.6464137	Both
DNA	GH25	19.8627	-0.2152	0.0026	-0.0499	0.8257	0.8857307	Both
DNA	GH26	22.0532	2.3014	0.2518	0.2124	0.0205	0.0493287	Both
DNA	GH27	80.433	18.0369	0.6122	0.5918	0.00E+00	0.0004346	Both
DNA	GH28	194.68	37.5255	0.7397	0.726	0.00E+00	2.91E-05	Both
DNA	GH29	112.4106	13.9748	0.5332	0.5086	0.0002	0.0013033	Both
DNA	GH3	587.4424	70.6498	0.5514	0.5278	0.0001	0.0011159	Both
DNA	GH30	51.6227	5.8121	0.3971	0.3654	0.0022	0.0087622	Both
DNA	GH31	139.0432	22.0342	0.5004	0.4741	0.0003	0.0022731	Both
DNA	GH33	59.3874	-3.7733	0.121	0.0747	0.1224	0.2106233	Both
DNA	GH36	91.5089	18.5041	0.6241	0.6043	0.00E+00	0.0003671	Both
DNA	GH32	31.3611	-1.3109	0.2248	0.184	0.0299	0.0647469	Both
DNA	GH37	39.0498	3.3724	0.1563	0.1119	0.076	0.1403383	Both
DNA	GH39	89.459	15.8127	0.7488	0.7356	0.00E+00	2.58E-05	Both
DNA	GH38	117.6702	24.4461	0.538	0.5137	0.0002	0.0012517	Both
DNA	GH4	144.275	1.5487	0.0046	-0.0478	0.7697	0.8459587	Both
DNA	GH42	45.2312	5.0392	0.5449	0.5209	0.0001	0.0011543	Both
DNA	GH43	113.62	19.3185	0.4057	0.3744	0.0019	0.0080903	Both
DNA	GH44	26.6648	5.7647	0.7977	0.787	0.00E+00	6.41E-06	Both
DNA	GH45	0.4552	0.1422	0.4954	0.4689	0.0004	0.0023531	Both
DNA	GH47	15.1347	0.2271	0.0081	-0.0441	0.6975	0.7921766	Both
DNA	GH46	2.1056	-0.2682	0.0508	0.0008	0.3261	0.4565644	Both
DNA	GH48	9.684	1.0552	0.3684	0.3352	0.0035	0.0118983	Both
DNA	GH49	0.1506	0.0066	0.0015	-0.051	0.8668	0.9172641	Both
DNA	GH5	170.4089	7.7496	0.1079	0.0609	0.1461	0.2460887	Both
DNA	GH50	7.8055	-0.2383	0.0144	-0.0375	0.6048	0.7160449	Both
DNA	GH51	110.7785	20.6894	0.7105	0.6952	0.00E+00	5.07E-05	Both
DNA	GH54	21.4612	8.2531	0.498	0.4716	0.0004	0.0023218	Both
DNA	GH53	57.7565	8.788	0.727	0.7127	0.00E+00	3.83E-05	Both
DNA	GH55	111.9622	21.3872	0.4325	0.4026	0.0012	0.0055547	Both
DNA	GH56	0.0046	0.0038	0.3475	0.3131	0.0049	0.0156401	Both
DNA	GH57	153.7528	-2.094	0.0188	-0.0328	0.5531	0.6706292	Both
DNA	GH58	0.039	-0.0014	0.003	-0.0494	0.8123	0.878776	Both
DNA	GH59	8.0215	2.6738	0.4428	0.4135	0.001	0.0049922	Both

DNA	GH6	18.8877	3.2493	0.3731	0.3401	0.0033	0.0113906	Both
DNA	GH62	3.2816	0.5501	0.1766	0.1333	0.0578	0.1142833	Both
DNA	GH63	15.2609	2.6887	0.456	0.4273	0.0008	0.004095	Both
DNA	GH64	20.0853	2.595	0.3811	0.3485	0.0029	0.0104357	Both
DNA	GH65	100.4831	-2.2598	0.0107	-0.0414	0.6559	0.7587056	Both
DNA	GH66	1.539	0.3108	0.1671	0.1233	0.0658	0.1260445	Both
DNA	GH68	0.282	0.0059	0.0082	-0.044	0.6957	0.7921766	Both
DNA	GH7	0.7235	0.2071	0.1584	0.1142	0.0739	0.1375713	Both
DNA	GH70	0.0383	0.0056	0.0362	-0.0146	0.409	0.5339961	Both
DNA	GH71	3.9384	0.7542	0.1776	0.1343	0.0571	0.1136777	Both
DNA	GH72	30.0493	1.0526	0.1072	0.0603	0.1473	0.2463953	Both
DNA	GH73	38.6432	-0.6746	0.0315	-0.0195	0.4414	0.5663811	Both
DNA	GH75	2.2066	0.0555	0.0089	-0.0433	0.6843	0.7878919	Both
DNA	GH74	13.9963	2.7925	0.6558	0.6377	0.00E+00	0.0001976	Both
DNA	GH76	14.814	2.4414	0.2755	0.2374	0.0146	0.0380834	Both
DNA	GH78	155.8729	16.0027	0.5026	0.4764	0.0003	0.0022374	Both
DNA	GH79	28.1345	2.5362	0.17	0.1263	0.0632	0.1229633	Both
DNA	GH8	19.2344	1.3428	0.0763	0.0277	0.2255	0.3515817	Both
DNA	GH80	0.002	-0.002	0.082	0.0337	0.2081	0.3327095	Both
DNA	GH81	5.7722	0.0523	0.0009	-0.0516	0.8953	0.9363172	Both
DNA	GH84	4.781	0.3475	0.1732	0.1296	0.0606	0.1188602	Both
DNA	GH85	0.6123	0.004	0.0002	-0.0524	0.9472	0.9586207	Both
DNA	GH86	1.587	-0.1728	0.1338	0.0882	0.103	0.184624	Both
DNA	GH87	68.8594	8.2746	0.5356	0.5112	0.0002	0.0012752	Both
DNA	GH88	24.4555	0.0498	0.0005	-0.0521	0.9248	0.9538692	Both
DNA	GH89	7.7554	1.7152	0.4008	0.3692	0.0021	0.0083703	Both
DNA	GH91	0.0888	-0.0168	0.055	0.0053	0.3062	0.4454504	Both
DNA	GH90	0.0034	1.00E-04	0.0002	-0.0524	0.9541	0.9617891	Both
DNA	GH9	54.2204	6.553	0.2512	0.2118	0.0206	0.0493287	Both
DNA	GH92	99.629	16.2132	0.2874	0.2498	0.0123	0.033042	Both
DNA	GH93	21.1746	1.3914	0.0158	-0.036	0.587	0.7039771	Both
DNA	GH94	30.4978	6.9733	0.5184	0.493	0.0002	0.0016727	Both
DNA	GH95	51.1365	6.4318	0.4153	0.3845	0.0016	0.0069826	Both
DNA	GH96	0.4341	-0.0875	0.0532	0.0034	0.3143	0.4485487	Both
DNA	GH97	31.9027	0.1137	0.0004	-0.0522	0.9344	0.9572623	Both
DNA	GH98	0.2928	-0.0636	0.3134	0.2773	0.0083	0.0234416	Both
DNA	GH99	12.1809	-0.9382	0.104	0.0569	0.1539	0.2540937	Both
DNA	GT1	152.7224	-3.7156	0.0157	-0.0362	0.589	0.7039771	Both
DNA	GT10	2.6221	0.4881	0.59	0.5685	0.00E+00	0.0005995	Both
DNA	GT12	0.0479	0.0034	0.0287	-0.0224	0.4629	0.5861856	Both
DNA	GT14	2.0818	0.1432	0.0775	0.029	0.2216	0.3476303	Both
DNA	GT11	5.172	0.643	0.3919	0.3599	0.0024	0.0092679	Both
DNA	GT13	0.1791	0.0739	0.3679	0.3346	0.0036	0.0118983	Both
DNA	GT15	2.0332	0.3505	0.1216	0.0754	0.1213	0.2106233	Both
DNA	GT16	0.0469	0.0274	0.5601	0.5369	1.00E-04	0.0009984	Both
DNA	GT17	1.0854	0.1005	0.2247	0.1839	0.0299	0.0647469	Both
DNA	GT18	0.0144	0.0087	0.4449	0.4157	0.001	0.0049035	Both
DNA	GT2	1818.8004	1.4259	0.0003	-0.0523	0.943	0.9583187	Both
DNA	GT19	83.4957	1.7491	0.1281	0.0822	0.1112	0.1964844	Both
DNA	GT20	149.4495	-3.2208	0.2694	0.2309	0.0159	0.0399433	Both
DNA	GT21	78.1092	8.8898	0.2355	0.1953	0.0257	0.0566841	Both
DNA	GT22	4.5977	0.5394	0.2179	0.1767	0.0329	0.0706013	Both
DNA	GT23	0.2539	0.104	0.5452	0.5213	0.0001	0.0011543	Both
DNA	GT24	2.8985	0.4781	0.2867	0.2491	0.0124	0.033042	Both
DNA	GT25	6.3037	0.8178	0.475	0.4474	0.0005	0.0032013	Both

DNA	GT26	137.4511	10.1858	0.7212	0.7066	0.00E+00	4.02E-05	Both
DNA	GT27	13.6554	1.1889	0.482	0.4547	0.0005	0.0028712	Both
DNA	GT28	177.389	4.2425	0.2083	0.1667	0.0375	0.0778592	Both
DNA	GT29	3.7879	-0.1275	0.0362	-0.0145	0.4087	0.5339961	Both
DNA	GT30	120.5539	3.2049	0.2127	0.1712	0.0354	0.0745874	Both
DNA	GT3	3.0716	0.8282	0.527	0.5021	0.0002	0.0014395	Both
DNA	GT33	1.6363	-0.1054	0.0429	-0.0074	0.3674	0.5011827	Both
DNA	GT34	0.9693	0.122	0.0693	0.0203	0.249	0.3834163	Both
DNA	GT32	12.037	-0.4933	0.0658	0.0166	0.2618	0.3982049	Both
DNA	GT31	1.5981	0.5666	0.3755	0.3427	0.0031	0.0111066	Both
DNA	GT35	354.1091	9.9201	0.0576	0.008	0.2948	0.4326528	Both
DNA	GT37	0.0077	0.0006	0.0035	-0.0489	0.7986	0.8677639	Both
DNA	GT39	21.0631	0.7823	0.0255	-0.0258	0.4893	0.6110212	Both
DNA	GT4	1835.3972	-36.4184	0.0505	0.0005	0.3274	0.4565644	Both
DNA	GT41	429.2439	28.0359	0.2725	0.2342	0.0152	0.0393819	Both
DNA	GT40	0.0014	0.0006	0.0532	0.0034	0.3145	0.4485487	Both
DNA	GT42	0.0596	0.0104	0.0548	0.0051	0.307	0.4454504	Both
DNA	GT43	0.0839	0.0392	0.3876	0.3554	0.0026	0.0096553	Both
DNA	GT47	70.9951	8.8831	0.2355	0.1953	0.0257	0.0566841	Both
DNA	GT45	0.2403	-0.0139	0.0493	-0.0007	0.3333	0.4622021	Both
DNA	GT44	0.0196	-0.0058	0.0984	0.0509	0.1662	0.2691107	Both
DNA	GT48	3.6235	1.0321	0.3246	0.289	0.007	0.0202388	Both
DNA	GT49	0.2071	0.0644	0.3288	0.2934	0.0066	0.0196603	Both
DNA	GT5	110.3216	-0.6914	0.0075	-0.0447	0.7085	0.8010523	Both
DNA	GT50	0.7517	0.2136	0.3213	0.2856	0.0074	0.0210318	Both
DNA	GT53	24.0821	-1.6573	0.0414	-0.0091	0.3766	0.5055465	Both
DNA	GT52	0	0	#VALUE!	#VALUE!	#VALUE!	NA	Both
DNA	GT51	587.598	-34.584	0.2585	0.2195	0.0186	0.045765	Both
DNA	GT54	0.0432	0.0263	0.3707	0.3376	0.0034	0.0116753	Both
DNA	GT57	1.512	0.3589	0.1981	0.1558	0.0432	0.088197	Both
DNA	GT56	0.434	0.0873	0.5753	0.5529	1.00E-04	0.0008074	Both
DNA	GT55	2.7859	-0.0269	0.0008	-0.0518	0.9031	0.940548	Both
DNA	GT58	0.5931	0.1463	0.2468	0.2071	0.022	0.0510532	Both
DNA	GT6	0.3486	0.0197	0.0177	-0.034	0.5659	0.6828407	Both
DNA	GT59	0.4033	0.0831	0.1473	0.1025	0.0858	0.1561028	Both
DNA	GT60	2.2724	0.8298	0.4947	0.4681	0.0004	0.0023531	Both
DNA	GT61	0.4009	0.107	0.4026	0.3712	0.002	0.0082459	Both
DNA	GT62	2.2504	0.1363	0.0731	0.0243	0.236	0.3655906	Both
DNA	GT64	0.8578	-0.0096	0.0023	-0.0502	0.8372	0.8942072	Both
DNA	GT65	0.0525	0.0205	0.3516	0.3174	0.0046	0.0150399	Both
DNA	GT66	19.0025	-0.5025	0.0302	-0.0208	0.4513	0.5749561	Both
DNA	GT69	0.8788	0.2119	0.1695	0.1258	0.0637	0.1229633	Both
DNA	GT68	0.033	0.0139	0.2662	0.2276	0.0167	0.041388	Both
DNA	GT7	5.6797	0.5054	0.3834	0.351	0.0028	0.0101884	Both
DNA	GT72	0.0007	-0.0002	0.0208	-0.0308	0.5331	0.6526762	Both
DNA	GT73	0.0676	0.0185	0.1209	0.0746	0.1225	0.2106233	Both
DNA	GT71	1.4697	0.1939	0.238	0.1978	0.0249	0.0562593	Both
DNA	GT70	0.7719	0.0215	0.0285	-0.0227	0.4647	0.5861856	Both
DNA	GT74	0.8743	-0.0361	0.0192	-0.0324	0.549	0.6688978	Both
DNA	GT75	0.9562	0.0415	0.0481	-0.0021	0.3397	0.4685362	Both
DNA	GT76	2.0735	-0.5953	0.4265	0.3964	0.0013	0.0060584	Both
DNA	GT77	0.8498	-0.2051	0.4374	0.4078	0.0011	0.0052839	Both
DNA	GT78	0.0021	-0.001	0.0601	0.0107	0.2841	0.4243889	Both
DNA	GT8	11.3981	0.0854	0.0015	-0.0511	0.8698	0.9172641	Both
DNA	GT80	5.8542	0.6275	0.4631	0.4349	0.0007	0.0038261	Both

DNA	GT82	0.4408	-0.0543	0.1175	0.0711	0.1282	0.2173387	Both
DNA	GT84	302.7752	27.8739	0.2089	0.1672	0.0373	0.0778592	Both
DNA	GT81	24.2095	-0.0671	0.0005	-0.0522	0.9273	0.9538692	Both
DNA	GT83	173.1688	2.8898	0.0467	-0.0035	0.3468	0.475699	Both
DNA	GT85	3.3791	-0.2606	0.042	-0.0084	0.373	0.5034114	Both
DNA	GT87	17.8526	1.703	0.1598	0.1156	0.0726	0.1375713	Both
DNA	GT88	0.0319	0.0067	0.0585	0.009	0.2906	0.4291283	Both
DNA	GT89	5.7995	-0.2884	0.0364	-0.0143	0.4072	0.5339961	Both
DNA	GT90	2.7262	0.6329	0.2921	0.2548	0.0114	0.031181	Both
DNA	GT9	169.2922	-3.2425	0.0356	-0.0152	0.4127	0.5339961	Both
DNA	GT91	0.0175	0.0019	0.0135	-0.0384	0.6156	0.7202681	Both
DNA	GT93	0.367	-0.0011	0.0007	-0.0519	0.9069	0.9406589	Both
DNA	GT94	0.8321	0.1643	0.3953	0.3635	0.0023	0.0088896	Both
DNA	GT92	0.1294	0.0553	0.4408	0.4113	0.001	0.0050782	Both
DNA	GT95	6.8457	-0.3135	0.1319	0.0862	0.1056	0.1880625	Both
DNA	PL1	17.2792	-0.4234	0.004	-0.0484	0.7843	0.8558918	Both
DNA	PL10	4.8889	0.788	0.1353	0.0898	0.1008	0.182082	Both
DNA	PL11	3.4173	0.1546	0.0085	-0.0437	0.6917	0.7921766	Both
DNA	PL12	23.1583	1.2841	0.1528	0.1082	0.0797	0.1461051	Both
DNA	PL13	0.0005	0.0001	0.0045	-0.0479	0.7718	0.8459587	Both
DNA	PL14	1.0529	-0.0393	0.0057	-0.0467	0.7457	0.8245156	Both
DNA	PL15	0.3252	-0.0417	0.04	-0.0105	0.3848	0.5136986	Both
DNA	PL17	1.5798	-0.1917	0.042	-0.0085	0.373	0.5034114	Both
DNA	PL18	0.003	-0.0018	0.1591	0.1148	0.0733	0.1375713	Both
DNA	PL16	0.5166	0.0094	0.0068	-0.0455	0.7224	0.8095153	Both
DNA	PL2	0.1859	0.089	0.6352	0.616	0.00E+00	0.0003189	Both
DNA	PL20	0.0885	-0.0023	0.001	-0.0515	0.8897	0.9343474	Both
DNA	PL21	0.5039	0.0233	0.0153	-0.0365	0.5926	0.7049673	Both
DNA	PL22	10.1314	2.1549	0.7999	0.7893	0.00E+00	6.41E-06	Both
DNA	PL23	0.0169	0.0018	0.0635	0.0142	0.2706	0.4067584	Both
DNA	PL3	0.2609	-0.0377	0.0134	-0.0385	0.617	0.7202681	Both
DNA	PL4	18.5959	6.6308	0.5934	0.572	0.00E+00	0.0005818	Both
DNA	PL5	2.4899	1.29	0.4029	0.3715	0.002	0.0082459	Both
DNA	PL6	1.9888	-0.1975	0.0217	-0.0298	0.5242	0.6464137	Both
DNA	PL7	3.6439	0.3635	0.0519	0.002	0.3206	0.4546438	Both
DNA	PL8	2.6452	0.5065	0.2714	0.233	0.0155	0.039592	Both
DNA	PL9	31.9165	0.7328	0.0069	-0.0454	0.7209	0.8095153	Both
Water_content	AA1	166.2439	11.8892	0.3902	0.3581	0.0025	0.0063828	Both
Water_content	AA11	0.4336	0.0798	0.0819	0.0335	0.2086	0.2958209	Both
Water_content	AA10	6.525	1.2338	0.46	0.4316	0.0007	0.002302	Both
Water_content	AA2	1.5806	0.3298	0.3369	0.302	0.0058	0.014007	Both
Water_content	AA3	414.0022	-31.8532	0.386	0.3537	0.0026	0.0067113	Both
Water_content	AA4	13.9824	5.4461	0.6511	0.6327	0.00E+00	6.54E-05	Both
Water_content	AA5	47.5523	-11.188	0.7133	0.6982	0.00E+00	1.54E-05	Both
Water_content	AA8	0.7042	0.1191	0.1603	0.1161	0.0721	0.1265979	Both
Water_content	AA7	55.8067	-3.8498	0.1734	0.1299	0.0604	0.1098308	Both
Water_content	AA6	28.9935	-4.2828	0.6682	0.6508	0.00E+00	4.76E-05	Both
Water_content	AA9	1.5682	0.3097	0.0732	0.0244	0.2357	0.3180674	Both
Water_content	CE1	214.5689	-3.2604	0.0612	0.0117	0.2798	0.3657834	Both
Water_content	CE11	161.0901	1.0246	0.0081	-0.0441	0.6979	0.7616212	Both
Water_content	CE12	17.1999	3.9811	0.61	0.5894	0.00E+00	0.0001533	Both
Water_content	CE13	0.0445	0.0212	0.3342	0.2991	0.0061	0.0143386	Both
Water_content	CE14	222.8524	7.6416	0.2775	0.2395	0.0141	0.0305965	Both
Water_content	CE15	41.9051	9.3977	0.7908	0.7798	0.00E+00	1.61E-06	Both
Water_content	CE2	2.7115	0.6569	0.2685	0.23	0.0161	0.0337091	Both

Water_content	CE3	23.2608	-4.3004	0.1506	0.1059	0.0822	0.1400066	Both
Water_content	CE4	354.0802	-47.8278	0.571	0.5484	1.00E-04	0.0003598	Both
Water_content	CE16	1.0609	0.0983	0.1026	0.0553	0.157	0.2331709	Both
Water_content	CE5	14.6374	-1.6961	0.082	0.0337	0.2082	0.2958209	Both
Water_content	CE7	11.1596	1.8638	0.4874	0.4604	0.0004	0.0015094	Both
Water_content	CE6	41.6877	8.6921	0.6565	0.6385	0.00E+00	6.09E-05	Both
Water_content	CE8	22.9784	2.7315	0.3128	0.2766	0.0084	0.0191462	Both
Water_content	CE9	93.7508	-0.6118	0.018	-0.0337	0.5624	0.6387472	Both
Water_content	GH100	0.8338	0.4048	0.4774	0.4499	0.0005	0.0017785	Both
Water_content	GH10	54.9015	4.7625	0.4254	0.3951	0.0014	0.0039536	Both
Water_content	GH102	40.7964	-3.7685	0.5661	0.5433	1.00E-04	0.0003918	Both
Water_content	GH101	0.7299	-0.1686	0.2182	0.177	0.0328	0.064273	Both
Water_content	GH103	106.2158	-14.4111	0.522	0.4969	0.0002	0.0008353	Both
Water_content	GH104	5.1644	-0.4528	0.1771	0.1337	0.0575	0.1061379	Both
Water_content	GH105	29.0354	8.832	0.672	0.6547	0.00E+00	4.54E-05	Both
Water_content	GH106	49.5372	13.1327	0.7777	0.7659	0.00E+00	2.65E-06	Both
Water_content	GH107	0.1563	-0.0529	0.1428	0.0977	0.0912	0.1537075	Both
Water_content	GH108	6.355	0.1004	0.0183	-0.0334	0.5593	0.6387472	Both
Water_content	GH109	20.0561	1.1211	0.1972	0.155	0.0437	0.0824782	Both
Water_content	GH11	14.887	3.1305	0.674	0.6568	0.00E+00	4.43E-05	Both
Water_content	GH110	1.2431	0.3309	0.5077	0.4818	0.0003	0.0010692	Both
Water_content	GH111	0.0414	-0.0071	0.0769	0.0284	0.2235	0.3116419	Both
Water_content	GH112	0.0997	0.0838	0.6907	0.6745	0.00E+00	2.96E-05	Both
Water_content	GH113	8.2393	-0.1521	0.0071	-0.0451	0.7162	0.7782081	Both
Water_content	GH114	10.5907	-2.3227	0.4737	0.446	0.0006	0.0018822	Both
Water_content	GH115	18.1582	7.651	0.7997	0.7892	0.00E+00	1.36E-06	Both
Water_content	GH116	44.574	8.0221	0.6147	0.5944	0.00E+00	0.0001441	Both
Water_content	GH117	3.4772	0.0387	0.0018	-0.0508	0.8571	0.8816749	Both
Water_content	GH118	0.0029	-0.0003	0.0127	-0.0393	0.6274	0.6967804	Both
Water_content	GH119	2.7623	-0.4342	0.1416	0.0964	0.0928	0.155235	Both
Water_content	GH12	8.8559	1.8129	0.4373	0.4077	0.0011	0.0033165	Both
Water_content	GH120	1.8739	0.1438	0.0238	-0.0276	0.5042	0.5958169	Both
Water_content	GH121	1.5942	-0.4131	0.1096	0.0627	0.1427	0.2158429	Both
Water_content	GH123	1.8672	-0.7451	0.5542	0.5307	0.0001	0.0004966	Both
Water_content	GH124	0.0511	-0.0274	0.4639	0.4357	0.0007	0.0021745	Both
Water_content	GH125	11.4055	2.3481	0.5117	0.486	0.0003	0.001001	Both
Water_content	GH126	0.0093	0.0012	0.0128	-0.0392	0.6257	0.6967804	Both
Water_content	GH127	44.4763	7.787	0.6324	0.613	0.00E+00	9.61E-05	Both
Water_content	GH128	6.9808	1.6306	0.4165	0.3858	0.0016	0.0045071	Both
Water_content	GH129	0.3906	0.0447	0.0852	0.0371	0.1992	0.287331	Both
Water_content	GH13	1501.8611	-21.4691	0.0364	-0.0143	0.4074	0.5036984	Both
Water_content	GH130	86.8053	9.802	0.4943	0.4677	0.0004	0.0013504	Both
Water_content	GH131	0.195	0.0655	0.2813	0.2435	0.0134	0.0294693	Both
Water_content	GH132	0.7589	0.1504	0.1139	0.0672	0.1346	0.2073384	Both
Water_content	GH133	72.2732	-9.6634	0.6127	0.5924	0.00E+00	0.0001461	Both
Water_content	GH14	0.5412	0.1063	0.464	0.4357	0.0007	0.0021745	Both
Water_content	GH17	59.077	-6.3132	0.3914	0.3594	0.0024	0.006322	Both
Water_content	GH15	408.8678	5.5934	0.0204	-0.0312	0.5372	0.6241962	Both
Water_content	GH18	245.204	15.0686	0.2749	0.2368	0.0147	0.0312354	Both
Water_content	GH19	16.9586	-0.2121	0.0036	-0.0488	0.7954	0.8388768	Both
Water_content	GH2	388.7654	58.348	0.7455	0.7321	0.00E+00	6.15E-06	Both
Water_content	GH20	100.202	5.9847	0.3362	0.3012	0.0059	0.0140331	Both
Water_content	GH23	392.3336	-13.1234	0.1591	0.1148	0.0733	0.1271207	Both
Water_content	GH24	15.9645	0.4057	0.0558	0.0061	0.3025	0.3893958	Both
Water_content	GH25	19.8627	-0.6435	0.0234	-0.028	0.508	0.5958169	Both

Water_content	GH26	22.0532	1.5603	0.1157	0.0692	0.1313	0.203433	Both
Water_content	GH27	80.433	20.0492	0.7565	0.7436	0.00E+00	4.49E-06	Both
Water_content	GH28	194.68	40.7685	0.873	0.8664	0.00E+00	1.47E-07	Both
Water_content	GH29	112.4106	15.8298	0.6841	0.6675	0.00E+00	3.42E-05	Both
Water_content	GH3	587.4424	80.9025	0.7231	0.7085	0.00E+00	1.19E-05	Both
Water_content	GH30	51.6227	6.7451	0.5348	0.5103	0.0002	0.0007066	Both
Water_content	GH31	139.0432	26.4658	0.722	0.7073	0.00E+00	1.19E-05	Both
Water_content	GH33	59.3874	-5.0258	0.2146	0.1733	0.0344	0.0670043	Both
Water_content	GH36	91.5089	20.4057	0.7589	0.7462	0.00E+00	4.33E-06	Both
Water_content	GH32	31.3611	-0.9958	0.1297	0.0839	0.1088	0.1750378	Both
Water_content	GH37	39.0498	5.2154	0.3739	0.341	0.0032	0.0080959	Both
Water_content	GH39	89.459	16.8327	0.8485	0.8405	0.00E+00	3.99E-07	Both
Water_content	GH38	117.6702	29.7432	0.7964	0.7857	0.00E+00	1.36E-06	Both
Water_content	GH4	144.275	-1.6644	0.0053	-0.047	0.753	0.8042401	Both
Water_content	GH42	45.2312	5.4774	0.6438	0.625	0.00E+00	7.60E-05	Both
Water_content	GH43	113.62	19.7974	0.4261	0.3959	0.0013	0.0039519	Both
Water_content	GH44	26.6648	5.6585	0.7686	0.7564	0.00E+00	3.34E-06	Both
Water_content	GH45	0.4552	0.1387	0.4714	0.4436	0.0006	0.0019409	Both
Water_content	GH47	15.1347	0.3353	0.0177	-0.034	0.5651	0.6389262	Both
Water_content	GH46	2.1056	-0.4148	0.1214	0.0752	0.1216	0.189608	Both
Water_content	GH48	9.684	0.9074	0.2725	0.2342	0.0152	0.032098	Both
Water_content	GH49	0.1506	-0.0236	0.0194	-0.0322	0.5467	0.6323122	Both
Water_content	GH5	170.4089	5.094	0.0466	-0.0036	0.3473	0.4368915	Both
Water_content	GH50	7.8055	-0.5278	0.0704	0.0215	0.2449	0.3269825	Both
Water_content	GH51	110.7785	22.1713	0.8159	0.8062	0.00E+00	1.22E-06	Both
Water_content	GH54	21.4612	9.4589	0.6542	0.6359	0.00E+00	6.31E-05	Both
Water_content	GH53	57.7565	8.8388	0.7355	0.7215	0.00E+00	8.48E-06	Both
Water_content	GH55	111.9622	26.2846	0.6533	0.635	0.00E+00	6.31E-05	Both
Water_content	GH56	0.0046	0.0045	0.5005	0.4742	0.0003	0.0012176	Both
Water_content	GH57	153.7528	-2.05	0.018	-0.0336	0.5615	0.6387472	Both
Water_content	GH58	0.039	-0.0037	0.0218	-0.0297	0.5232	0.6107512	Both
Water_content	GH59	8.0215	2.9115	0.525	0.5	0.0002	0.0007972	Both
Water_content	GH6	18.8877	3.0322	0.3249	0.2894	0.007	0.0162876	Both
Water_content	GH62	3.2816	0.3298	0.0635	0.0142	0.2705	0.3570953	Both
Water_content	GH63	15.2609	2.5582	0.4128	0.3819	0.0017	0.0047367	Both
Water_content	GH64	20.0853	2.7868	0.4395	0.41	0.0011	0.0032303	Both
Water_content	GH65	100.4831	-2.3914	0.0119	-0.0401	0.6372	0.704561	Both
Water_content	GH66	1.539	0.1712	0.0507	0.0007	0.3265	0.41596	Both
Water_content	GH68	0.282	0.0063	0.0093	-0.0429	0.6777	0.7427911	Both
Water_content	GH7	0.7235	0.1932	0.1379	0.0925	0.0975	0.1615674	Both
Water_content	GH70	0.0383	0.0018	0.0038	-0.0487	0.7916	0.8383591	Both
Water_content	GH71	3.9384	0.9444	0.2785	0.2405	0.0139	0.0304327	Both
Water_content	GH72	30.0493	0.9085	0.0799	0.0315	0.2145	0.3024057	Both
Water_content	GH73	38.6432	-1.0387	0.0747	0.026	0.2306	0.3179848	Both
Water_content	GH75	2.2066	0.0133	0.0005	-0.0521	0.9227	0.9338698	Both
Water_content	GH74	13.9963	2.1032	0.372	0.339	0.0033	0.0082685	Both
Water_content	GH76	14.814	2.4828	0.2849	0.2473	0.0127	0.0284589	Both
Water_content	GH78	155.8729	17.5967	0.6077	0.5871	0.00E+00	0.000159	Both
Water_content	GH79	28.1345	3.8279	0.3873	0.3551	0.0026	0.0066314	Both
Water_content	GH8	19.2344	1.2219	0.0632	0.0139	0.2717	0.3570953	Both
Water_content	GH80	0.002	-0.0019	0.0763	0.0277	0.2254	0.312509	Both
Water_content	GH81	5.7722	-0.1253	0.0054	-0.047	0.7521	0.8042401	Both
Water_content	GH84	4.781	0.3324	0.1584	0.1141	0.0739	0.1271207	Both
Water_content	GH85	0.6123	-0.0166	0.004	-0.0484	0.7851	0.8349997	Both
Water_content	GH86	1.587	-0.2432	0.2652	0.2265	0.0169	0.0350499	Both

Water_content	GH87	68.8594	9.1088	0.6491	0.6306	0.00E+00	6.73E-05	Both
Water_content	GH88	24.4555	-0.3793	0.0279	-0.0233	0.4696	0.5639829	Both
Water_content	GH89	7.7554	2.0571	0.5765	0.5542	1.00E-04	0.0003232	Both
Water_content	GH91	0.0888	-0.0298	0.1729	0.1294	0.0608	0.1098406	Both
Water_content	GH90	0.0034	-0.0007	0.0167	-0.0351	0.5767	0.6490635	Both
Water_content	GH9	54.2204	6.6387	0.2578	0.2188	0.0188	0.0383273	Both
Water_content	GH92	99.629	19.0893	0.3983	0.3667	0.0022	0.0057522	Both
Water_content	GH93	21.1746	0.1793	0.0003	-0.0524	0.9444	0.9481936	Both
Water_content	GH94	30.4978	7.4709	0.595	0.5737	0.00E+00	0.0002129	Both
Water_content	GH95	51.1365	6.523	0.4272	0.397	0.0013	0.0039223	Both
Water_content	GH96	0.4341	-0.1381	0.1327	0.0871	0.1044	0.1702034	Both
Water_content	GH97	31.9027	-0.4365	0.0054	-0.0469	0.7516	0.8042401	Both
Water_content	GH98	0.2928	-0.0829	0.5326	0.508	0.0002	0.000711	Both
Water_content	GH99	12.1809	-1.2786	0.1932	0.1508	0.0462	0.0864823	Both
Water_content	GT1	152.7224	-4.9245	0.0275	-0.0237	0.4726	0.5648519	Both
Water_content	GT10	2.6221	0.5707	0.8066	0.7964	0.00E+00	1.22E-06	Both
Water_content	GT12	0.0479	0.0037	0.0324	-0.0185	0.435	0.5352535	Both
Water_content	GT14	2.0818	0.1826	0.1261	0.0801	0.1142	0.1813582	Both
Water_content	GT11	5.172	0.7497	0.5328	0.5082	0.0002	0.000711	Both
Water_content	GT13	0.1791	0.0995	0.667	0.6494	0.00E+00	4.78E-05	Both
Water_content	GT15	2.0332	0.3895	0.1502	0.1055	0.0826	0.1400066	Both
Water_content	GT16	0.0469	0.0327	0.7968	0.7861	0.00E+00	1.36E-06	Both
Water_content	GT17	1.0854	0.1326	0.3917	0.3597	0.0024	0.006322	Both
Water_content	GT18	0.0144	0.0091	0.4855	0.4584	0.0004	0.0015431	Both
Water_content	GT2	1818.8004	-15.3124	0.0318	-0.0192	0.4393	0.5352535	Both
Water_content	GT19	83.4957	2.2232	0.207	0.1652	0.0383	0.0733061	Both
Water_content	GT20	149.4495	-3.5139	0.3206	0.2849	0.0075	0.0171621	Both
Water_content	GT21	78.1092	11.7622	0.4123	0.3814	0.0017	0.0047367	Both
Water_content	GT22	4.5977	0.4837	0.1752	0.1318	0.0589	0.1079994	Both
Water_content	GT23	0.2539	0.1265	0.8071	0.7969	0.00E+00	1.22E-06	Both
Water_content	GT24	2.8985	0.6036	0.4569	0.4283	0.0008	0.0023858	Both
Water_content	GT25	6.3037	0.9811	0.6837	0.6671	0.00E+00	3.42E-05	Both
Water_content	GT26	137.4511	10.2171	0.7257	0.7112	0.00E+00	1.15E-05	Both
Water_content	GT27	13.6554	1.4972	0.7644	0.752	0.00E+00	3.71E-06	Both
Water_content	GT28	177.389	3.3552	0.1303	0.0845	0.1079	0.1747219	Both
Water_content	GT29	3.7879	-0.27	0.1623	0.1182	0.0702	0.1240674	Both
Water_content	GT30	120.5539	3.5536	0.2615	0.2226	0.0178	0.0366731	Both
Water_content	GT3	3.0716	0.829	0.5279	0.5031	0.0002	0.0007619	Both
Water_content	GT33	1.6363	-0.1563	0.0944	0.0468	0.1754	0.255993	Both
Water_content	GT34	0.9693	0.1479	0.1019	0.0546	0.1585	0.2340014	Both
Water_content	GT32	12.037	-0.6424	0.1116	0.0648	0.1389	0.2126384	Both
Water_content	GT31	1.5981	0.6497	0.4938	0.4671	0.0004	0.0013504	Both
Water_content	GT35	354.1091	11.8921	0.0828	0.0345	0.2061	0.295542	Both
Water_content	GT37	0.0077	0.0005	0.0025	-0.05	0.8285	0.8628901	Both
Water_content	GT39	21.0631	0.3741	0.0058	-0.0465	0.7422	0.8029707	Both
Water_content	GT4	1835.3972	-66.6277	0.169	0.1253	0.0641	0.1141086	Both
Water_content	GT41	429.2439	32.0989	0.3572	0.3233	0.0042	0.0103925	Both
Water_content	GT40	0.0014	0.0007	0.0733	0.0246	0.2351	0.3180674	Both
Water_content	GT42	0.0596	0.0177	0.1586	0.1143	0.0737	0.1271207	Both
Water_content	GT43	0.0839	0.0529	0.7043	0.6888	0.00E+00	1.99E-05	Both
Water_content	GT47	70.9951	11.6286	0.4036	0.3722	0.002	0.005377	Both
Water_content	GT45	0.2403	-0.0233	0.1376	0.0922	0.0978	0.1615674	Both
Water_content	GT44	0.0196	-0.0077	0.1718	0.1283	0.0617	0.110634	Both
Water_content	GT48	3.6235	1.2279	0.4595	0.431	0.0007	0.002302	Both
Water_content	GT49	0.2071	0.0881	0.6141	0.5938	0.00E+00	0.0001441	Both

Water_content	GT5	110.3216	-0.4602	0.0033	-0.0491	0.8037	0.84051	Both
Water_content	GT50	0.7517	0.2392	0.403	0.3715	0.002	0.005377	Both
Water_content	GT53	24.0821	-1.4572	0.032	-0.019	0.4381	0.5352535	Both
Water_content	GT52	0	0	#VALUE!	#VALUE!	#VALUE!	NA	Both
Water_content	GT51	587.598	-44.3292	0.4247	0.3944	0.0014	0.0039538	Both
Water_content	GT54	0.0432	0.0352	0.6609	0.6431	0.00E+00	5.53E-05	Both
Water_content	GT57	1.512	0.4477	0.3082	0.2718	0.009	0.0203413	Both
Water_content	GT56	0.434	0.0916	0.6328	0.6135	0.00E+00	9.61E-05	Both
Water_content	GT55	2.7859	0.0947	0.01	-0.0422	0.667	0.7342333	Both
Water_content	GT58	0.5931	0.1678	0.3247	0.2891	0.007	0.0162876	Both
Water_content	GT6	0.3486	0.0491	0.11	0.0632	0.1418	0.2157622	Both
Water_content	GT59	0.4033	0.0998	0.2126	0.1711	0.0354	0.0683827	Both
Water_content	GT60	2.2724	1.0372	0.773	0.761	0.00E+00	2.99E-06	Both
Water_content	GT61	0.4009	0.1208	0.5138	0.4882	0.0003	0.0009738	Both
Water_content	GT62	2.2504	0.1224	0.0589	0.0094	0.2891	0.376037	Both
Water_content	GT64	0.8578	-0.0347	0.0297	-0.0214	0.4554	0.552235	Both
Water_content	GT65	0.0525	0.0275	0.6305	0.6111	0.00E+00	9.86E-05	Both
Water_content	GT66	19.0025	-0.807	0.0779	0.0293	0.2206	0.3093214	Both
Water_content	GT69	0.8788	0.2701	0.2752	0.2371	0.0146	0.0312354	Both
Water_content	GT68	0.033	0.0196	0.532	0.5074	0.0002	0.000711	Both
Water_content	GT7	5.6797	0.607	0.5533	0.5298	0.0001	0.0004974	Both
Water_content	GT72	0.0007	0.00E+00	0.0002	-0.0524	0.9541	0.954133	Both
Water_content	GT73	0.0676	0.0174	0.1072	0.0602	0.1473	0.2201348	Both
Water_content	GT71	1.4697	0.188	0.2237	0.1828	0.0304	0.0605041	Both
Water_content	GT70	0.7719	0.0297	0.0543	0.0045	0.3095	0.3963863	Both
Water_content	GT74	0.8743	-0.0101	0.0015	-0.051	0.8671	0.8847023	Both
Water_content	GT75	0.9562	0.0409	0.0467	-0.0035	0.3468	0.4368915	Both
Water_content	GT76	2.0735	-0.7262	0.6346	0.6154	0.00E+00	9.49E-05	Both
Water_content	GT77	0.8498	-0.2268	0.5346	0.5101	0.0002	0.0007066	Both
Water_content	GT78	0.0021	-0.0011	0.0726	0.0238	0.2377	0.3190102	Both
Water_content	GT8	11.3981	-0.0893	0.0016	-0.051	0.8637	0.8847023	Both
Water_content	GT80	5.8542	0.68	0.5439	0.5199	0.0001	0.0006002	Both
Water_content	GT82	0.4408	-0.0744	0.2205	0.1795	0.0317	0.0626853	Both
Water_content	GT84	302.7752	32.4362	0.2828	0.2451	0.0131	0.0290687	Both
Water_content	GT81	24.2095	-0.5335	0.0285	-0.0226	0.4645	0.5605191	Both
Water_content	GT83	173.1688	4.7752	0.1275	0.0816	0.1121	0.1791524	Both
Water_content	GT85	3.3791	-0.2034	0.0256	-0.0257	0.4887	0.5813145	Both
Water_content	GT87	17.8526	1.4922	0.1227	0.0765	0.1196	0.1876316	Both
Water_content	GT88	0.0319	0.0138	0.2478	0.2082	0.0217	0.0438488	Both
Water_content	GT89	5.7995	-0.3257	0.0464	-0.0037	0.3481	0.4368915	Both
Water_content	GT90	2.7262	0.7466	0.4065	0.3753	0.0019	0.0051754	Both
Water_content	GT9	169.2922	-5.2054	0.0917	0.0439	0.182	0.2640278	Both
Water_content	GT91	0.0175	0.0005	0.001	-0.0515	0.8897	0.9040739	Both
Water_content	GT93	0.367	-0.0008	0.0004	-0.0522	0.9334	0.9409382	Both
Water_content	GT94	0.8321	0.1528	0.3418	0.3072	0.0054	0.0131018	Both
Water_content	GT92	0.1294	0.0626	0.5656	0.5427	1.00E-04	0.0003918	Both
Water_content	GT95	6.8457	-0.3874	0.2014	0.1594	0.0413	0.0785083	Both
Water_content	PL1	17.2792	-1.3463	0.0409	-0.0096	0.3796	0.471649	Both
Water_content	PL10	4.8889	0.7837	0.1339	0.0883	0.1029	0.1687311	Both
Water_content	PL11	3.4173	-0.0982	0.0034	-0.049	0.8014	0.84051	Both
Water_content	PL12	23.1583	0.8271	0.0634	0.0141	0.2709	0.3570953	Both
Water_content	PL13	0.0005	0.0005	0.1075	0.0606	0.1467	0.2201348	Both
Water_content	PL14	1.0529	-0.1416	0.0736	0.0248	0.2342	0.3180674	Both
Water_content	PL15	0.3252	-0.0654	0.0984	0.0509	0.1662	0.244021	Both
Water_content	PL17	1.5798	-0.3279	0.1228	0.0767	0.1193	0.1876316	Both

Water_content	PL18	0.003	-0.002	0.183	0.1399	0.0531	0.0987074	Both
Water_content	PL16	0.5166	0.0055	0.0024	-0.0501	0.8339	0.8649627	Both
Water_content	PL2	0.1859	0.097	0.7543	0.7414	0.00E+00	4.62E-06	Both
Water_content	PL20	0.0885	-0.0089	0.0148	-0.037	0.5993	0.6715508	Both
Water_content	PL21	0.5039	0.0451	0.0576	0.008	0.2946	0.3812114	Both
Water_content	PL22	10.1314	2.1632	0.806	0.7958	0.00E+00	1.22E-06	Both
Water_content	PL23	0.0169	0.001	0.0183	-0.0334	0.5589	0.6387472	Both
Water_content	PL3	0.2609	-0.0686	0.0444	-0.0059	0.359	0.4483269	Both
Water_content	PL4	18.5959	7.7616	0.8131	0.8033	0.00E+00	1.22E-06	Both
Water_content	PL5	2.4899	1.6632	0.6698	0.6524	0.00E+00	4.69E-05	Both
Water_content	PL6	1.9888	-0.3634	0.0734	0.0247	0.2347	0.3180674	Both
Water_content	PL7	3.6439	0.2448	0.0235	-0.0279	0.5068	0.5958169	Both
Water_content	PL8	2.6452	0.4743	0.238	0.1979	0.0249	0.0499284	Both
Water_content	PL9	31.9165	-0.3707	0.0018	-0.0508	0.8568	0.8816749	Both
Bulk_density	AA1	167.5548	0.6346	0.0013	-0.0653	0.8907	0.9677864	Both
Bulk_density	AA11	0.3793	0.1495	0.365	0.3227	0.0102	0.1158478	Both
Bulk_density	AA10	6.2843	0.1377	0.0054	-0.0609	0.7785	0.9439118	Both
Bulk_density	AA2	1.4949	0.0591	0.0125	-0.0533	0.6687	0.8840434	Both
Bulk_density	AA3	400.4837	26.3138	0.3859	0.3449	0.0078	0.1148631	Both
Bulk_density	AA4	14.7999	0.164	0.0005	-0.0661	0.9316	0.9713642	Both
Bulk_density	AA5	44.5348	0.2522	0.0004	-0.0663	0.9404	0.9713642	Both
Bulk_density	AA8	0.642	0.0303	0.0157	-0.0499	0.6314	0.8840434	Both
Bulk_density	AA7	53.573	2.6839	0.1026	0.0427	0.2102	0.5994247	Both
Bulk_density	AA6	28.1736	1.2877	0.0605	-0.0021	0.3412	0.6871518	Both
Bulk_density	AA9	1.3374	0.202	0.1068	0.0473	0.2003	0.586269	Both
Bulk_density	CE1	212.1707	-4.1865	0.0973	0.0372	0.2228	0.61454	Both
Bulk_density	CE11	160.3592	-9.1476	0.5562	0.5266	0.0006	0.0369157	Both
Bulk_density	CE12	16.4953	-0.6393	0.0152	-0.0505	0.6376	0.8840434	Both
Bulk_density	CE13	0.0477	-0.0073	0.0356	-0.0287	0.4686	0.7788127	Both
Bulk_density	CE14	222.5899	-9.3226	0.3649	0.3226	0.0102	0.1158478	Both
Bulk_density	CE15	42.4449	-2.4511	0.0452	-0.0185	0.4128	0.7565776	Both
Bulk_density	CE2	2.4408	-0.2089	0.0406	-0.0234	0.438	0.7682185	Both
Bulk_density	CE3	18.6045	-0.4413	0.008	-0.0581	0.7324	0.9379539	Both
Bulk_density	CE4	333.7575	8.3713	0.0279	-0.0369	0.5217	0.8028494	Both
Bulk_density	CE16	1.0177	0.0392	0.0249	-0.0401	0.5454	0.821393	Both
Bulk_density	CE5	14.7314	1.8573	0.0819	0.0207	0.2654	0.6660923	Both
Bulk_density	CE7	10.712	-0.6174	0.0565	-0.0064	0.3582	0.6928601	Both
Bulk_density	CE6	44.0687	0.2068	0.0004	-0.0663	0.9404	0.9713642	Both
Bulk_density	CE8	22.297	-1.2976	0.0732	0.0114	0.2935	0.6821252	Both
Bulk_density	CE9	92.7064	-2.2077	0.2776	0.2295	0.0298	0.2264004	Both
Bulk_density	GH100	0.8315	0.0465	0.0054	-0.0609	0.7787	0.9439118	Both
Bulk_density	GH10	54.3526	-1.9845	0.0657	0.0034	0.3208	0.6871518	Both
Bulk_density	GH102	39.3955	-0.8641	0.0396	-0.0244	0.4438	0.7682185	Both
Bulk_density	GH101	0.5871	-0.0468	0.0571	-0.0057	0.3555	0.6928601	Both
Bulk_density	GH103	100.7469	5.0255	0.0877	0.0269	0.2484	0.6494465	Both
Bulk_density	GH104	4.8843	-0.3735	0.1474	0.0905	0.1282	0.4549731	Both
Bulk_density	GH105	29.5035	0.1666	0.0002	-0.0665	0.9573	0.9800102	Both
Bulk_density	GH106	50.7241	-1.6399	0.0101	-0.0559	0.7011	0.9079836	Both
Bulk_density	GH107	0.0989	-0.0261	0.1503	0.0937	0.1241	0.4515622	Both
Bulk_density	GH108	6.3301	0.09	0.0126	-0.0533	0.6683	0.8840434	Both
Bulk_density	GH109	19.6416	-1.0827	0.1719	0.1167	0.0979	0.3901366	Both
Bulk_density	GH11	14.8409	0.0856	0.0004	-0.0662	0.9375	0.9713642	Both
Bulk_density	GH110	1.2372	0.118	0.0564	-0.0065	0.3589	0.6928601	Both
Bulk_density	GH111	0.0337	0.0033	0.0289	-0.0359	0.5146	0.8028494	Both
Bulk_density	GH112	0.1033	-0.0351	0.1011	0.0412	0.2136	0.6023146	Both

Bulk_density	GH113	7.5467	-0.7001	0.4586	0.4226	0.0028	0.0879162	Both
Bulk_density	GH114	9.9748	0.3739	0.0131	-0.0527	0.6623	0.8840434	Both
Bulk_density	GH115	17.7348	-1.9708	0.0446	-0.0191	0.416	0.7565776	Both
Bulk_density	GH116	47.8964	-0.9537	0.0134	-0.0524	0.6582	0.8840434	Both
Bulk_density	GH117	3.3325	-0.2552	0.0793	0.0179	0.2735	0.6795807	Both
Bulk_density	GH118	0.0024	-0.0007	0.0663	0.0041	0.3183	0.6871518	Both
Bulk_density	GH119	2.343	-0.4632	0.4267	0.3885	0.0045	0.0879162	Both
Bulk_density	GH12	8.5827	-0.3237	0.0121	-0.0538	0.6745	0.8863248	Both
Bulk_density	GH120	2.0124	-0.156	0.0275	-0.0373	0.5246	0.8028494	Both
Bulk_density	GH121	1.0903	-0.1795	0.083	0.0219	0.2621	0.6660923	Both
Bulk_density	GH123	1.5611	-0.1622	0.0377	-0.0265	0.4555	0.7725231	Both
Bulk_density	GH124	0.0516	0.0019	0.002	-0.0645	0.8634	0.9558926	Both
Bulk_density	GH125	11.821	0.2468	0.0053	-0.0611	0.782	0.9439118	Both
Bulk_density	GH126	0.0107	-0.0005	0.002	-0.0645	0.8645	0.9558926	Both
Bulk_density	GH127	44.8967	-2.279	0.0451	-0.0185	0.413	0.7565776	Both
Bulk_density	GH128	6.9425	0.7679	0.0751	0.0134	0.2871	0.6821252	Both
Bulk_density	GH129	0.4094	-0.074	0.2211	0.1692	0.0568	0.2910962	Both
Bulk_density	GH13	1483.729	16.113	0.0215	-0.0438	0.5748	0.8339884	Both
Bulk_density	GH130	87.9204	-6.594	0.185	0.1307	0.0848	0.3734735	Both
Bulk_density	GH131	0.175	0.0275	0.0625	0.00E+00	0.333	0.6871518	Both
Bulk_density	GH132	0.7082	0.2413	0.3244	0.2794	0.017	0.1524156	Both
Bulk_density	GH133	73.0272	-3.5743	0.0685	0.0064	0.3101	0.6871518	Both
Bulk_density	GH14	0.5436	0.0269	0.0247	-0.0403	0.5465	0.821393	Both
Bulk_density	GH17	56.26	3.1888	0.1705	0.1152	0.0995	0.3901366	Both
Bulk_density	GH15	415.3637	-0.7882	0.0004	-0.0662	0.9391	0.9713642	Both
Bulk_density	GH18	239.796	-4.8636	0.0282	-0.0366	0.5193	0.8028494	Both
Bulk_density	GH19	15.8035	-0.5005	0.0351	-0.0292	0.4716	0.7788127	Both
Bulk_density	GH2	392.0372	-22.3872	0.0915	0.031	0.2379	0.6351695	Both
Bulk_density	GH20	99.0943	-2.787	0.0649	0.0025	0.3238	0.6871518	Both
Bulk_density	GH23	379.6866	-11.1855	0.2925	0.2454	0.025	0.202203	Both
Bulk_density	GH24	15.8398	-1.4592	0.5959	0.5689	0.0003	0.035534	Both
Bulk_density	GH25	18.5578	-0.6694	0.0446	-0.0191	0.4157	0.7565776	Both
Bulk_density	GH26	21.3385	-0.3036	0.0048	-0.0616	0.7918	0.9439118	Both
Bulk_density	GH27	82.4258	-1.4152	0.0032	-0.0633	0.8304	0.9540309	Both
Bulk_density	GH28	197.1089	-9.3691	0.0378	-0.0263	0.4545	0.7725231	Both
Bulk_density	GH29	116.8491	-6.4041	0.1186	0.0598	0.1759	0.5590026	Both
Bulk_density	GH3	597.3215	-11.7881	0.0131	-0.0526	0.6613	0.8840434	Both
Bulk_density	GH30	51.5232	1.5588	0.0236	-0.0415	0.556	0.8258305	Both
Bulk_density	GH31	145.8435	-3.5313	0.0132	-0.0526	0.6604	0.8840434	Both
Bulk_density	GH33	55.8107	-4.5547	0.3168	0.2712	0.0187	0.1615389	Both
Bulk_density	GH36	95.1663	-2.3154	0.0088	-0.0573	0.7204	0.9273328	Both
Bulk_density	GH32	31.7397	-0.2393	0.0078	-0.0584	0.7363	0.9380998	Both
Bulk_density	GH37	41.9856	0.114	0.0003	-0.0663	0.9455	0.9726448	Both
Bulk_density	GH39	92.7626	-2.791	0.0221	-0.0431	0.5693	0.8311279	Both
Bulk_density	GH38	122.3088	-0.817	0.0005	-0.0661	0.93	0.9713642	Both
Bulk_density	GH4	136.8651	4.3536	0.0644	0.002	0.3257	0.6871518	Both
Bulk_density	GH42	46.0601	-0.0901	0.0002	-0.0665	0.9617	0.9800102	Both
Bulk_density	GH43	110.7903	-3.2037	0.011	-0.0549	0.6882	0.8996698	Both
Bulk_density	GH44	26.6598	-2.4238	0.1136	0.0545	0.1859	0.5760857	Both
Bulk_density	GH45	0.4395	-0.0123	0.0042	-0.0622	0.8057	0.9439118	Both
Bulk_density	GH47	14.5476	0.5948	0.0755	0.0139	0.2858	0.6821252	Both
Bulk_density	GH46	1.6778	-0.0762	0.0101	-0.0559	0.7018	0.9079836	Both
Bulk_density	GH48	9.5178	-0.0847	0.002	-0.0645	0.8642	0.9558926	Both
Bulk_density	GH49	0.0735	0.0158	0.3266	0.2817	0.0165	0.1524156	Both
Bulk_density	GH5	162.8242	-3.3917	0.0356	-0.0287	0.4686	0.7788127	Both

Bulk_density	GH50	8.2131	-0.0552	0.0008	-0.0658	0.9158	0.9713642	Both
Bulk_density	GH51	114.0405	-4.7387	0.0327	-0.0318	0.4872	0.7854961	Both
Bulk_density	GH54	22.8537	1.5998	0.0161	-0.0495	0.6276	0.8840434	Both
Bulk_density	GH53	57.6369	-2.9875	0.0699	0.0079	0.3051	0.6871518	Both
Bulk_density	GH55	118.7382	3.8373	0.014	-0.0517	0.651	0.8840434	Both
Bulk_density	GH56	0.0054	-0.0003	0.0014	-0.0652	0.8859	0.9677864	Both
Bulk_density	GH57	155.1933	-10.0014	0.3945	0.3542	0.0069	0.1087774	Both
Bulk_density	GH58	0.0297	-0.0049	0.2391	0.1884	0.0464	0.2795735	Both
Bulk_density	GH59	8.4302	0.729	0.0278	-0.037	0.5222	0.8028494	Both
Bulk_density	GH6	18.0144	0.0355	0.00E+00	-0.0666	0.9797	0.9849794	Both
Bulk_density	GH62	2.9489	-0.4451	0.1514	0.0948	0.1227	0.4515622	Both
Bulk_density	GH63	14.7116	-0.2681	0.0042	-0.0621	0.804	0.9439118	Both
Bulk_density	GH64	20.2333	0.2433	0.0028	-0.0637	0.8394	0.9540309	Both
Bulk_density	GH65	100.6347	-1.6462	0.0049	-0.0615	0.7902	0.9439118	Both
Bulk_density	GH66	1.2336	-0.0912	0.0418	-0.0221	0.4314	0.767897	Both
Bulk_density	GH68	0.2762	0.0046	0.0046	-0.0617	0.7949	0.9439118	Both
Bulk_density	GH7	0.6125	0.0879	0.0667	0.0045	0.3169	0.6871518	Both
Bulk_density	GH70	0.0285	-0.003	0.0612	-0.0014	0.3386	0.6871518	Both
Bulk_density	GH71	3.7081	0.8313	0.2256	0.174	0.054	0.2880453	Both
Bulk_density	GH72	28.9583	-0.0532	0.0006	-0.0661	0.9283	0.9713642	Both
Bulk_density	GH73	37.355	-1.1752	0.1672	0.1117	0.1031	0.3982308	Both
Bulk_density	GH75	2.0654	-0.0306	0.004	-0.0624	0.8093	0.9439118	Both
Bulk_density	GH74	13.6378	-0.6708	0.0339	-0.0305	0.4793	0.7854961	Both
Bulk_density	GH76	14.6477	1.0947	0.0486	-0.0149	0.3954	0.747073	Both
Bulk_density	GH78	155.0604	-0.8136	0.0011	-0.0655	0.9003	0.9698454	Both
Bulk_density	GH79	30.0988	1.1713	0.0593	-0.0034	0.3463	0.6871518	Both
Bulk_density	GH8	18.1144	-1.8242	0.1711	0.1158	0.0988	0.3901366	Both
Bulk_density	GH80	0.0025	-0.0019	0.0599	-0.0028	0.3439	0.6871518	Both
Bulk_density	GH81	5.0743	-0.0604	0.006	-0.0603	0.7679	0.9439118	Both
Bulk_density	GH84	4.5296	-0.333	0.2342	0.1832	0.049	0.2795735	Both
Bulk_density	GH85	0.543	-0.0749	0.1094	0.05	0.1947	0.586269	Both
Bulk_density	GH86	1.3995	-0.0964	0.1211	0.0625	0.1711	0.5532673	Both
Bulk_density	GH87	68.918	0.7528	0.004	-0.0624	0.8096	0.9439118	Both
Bulk_density	GH88	23.6292	-0.9326	0.4	0.36	0.0064	0.1078697	Both
Bulk_density	GH89	7.8454	0.3398	0.0133	-0.0525	0.6593	0.8840434	Both
Bulk_density	GH91	0.063	-0.0021	0.0025	-0.064	0.8476	0.9558926	Both
Bulk_density	GH90	0.0012	1.00E-04	0.0016	-0.065	0.8792	0.9677864	Both
Bulk_density	GH9	53.6885	-3.4878	0.0622	-0.0003	0.3344	0.6871518	Both
Bulk_density	GH92	98.7333	1.2009	0.0013	-0.0652	0.8891	0.9677864	Both
Bulk_density	GH93	16.9461	0.491	0.014	-0.0517	0.651	0.8840434	Both
Bulk_density	GH94	32.6818	-3.1066	0.1072	0.0477	0.1996	0.586269	Both
Bulk_density	GH95	49.424	-2.2189	0.0485	-0.015	0.3959	0.747073	Both
Bulk_density	GH96	0.2919	-0.1136	0.2239	0.1721	0.0551	0.2880453	Both
Bulk_density	GH97	30.3207	-1.8464	0.1212	0.0626	0.1709	0.5532673	Both
Bulk_density	GH98	0.2638	0.001	1.00E-04	-0.0666	0.9712	0.9829016	Both
Bulk_density	GH99	11.0655	-0.5654	0.0992	0.0392	0.2181	0.6082062	Both
Bulk_density	GT1	145.9026	12.0105	0.1812	0.1266	0.0884	0.378262	Both
Bulk_density	GT10	2.645	-0.2104	0.0888	0.028	0.2454	0.6484564	Both
Bulk_density	GT12	0.049	-0.0049	0.0634	0.001	0.3295	0.6871518	Both
Bulk_density	GT14	2.0706	-0.1021	0.0363	-0.0279	0.4638	0.7788127	Both
Bulk_density	GT11	5.3339	-0.5727	0.2855	0.2379	0.0271	0.2127811	Both
Bulk_density	GT13	0.192	0.0036	0.0007	-0.0659	0.9171	0.9713642	Both
Bulk_density	GT15	1.9836	0.653	0.4155	0.3765	0.0052	0.0935723	Both
Bulk_density	GT16	0.0505	0.0018	0.0021	-0.0644	0.8606	0.9558926	Both
Bulk_density	GT17	1.0853	-0.0398	0.0326	-0.0319	0.4882	0.7854961	Both

Bulk_density	GT18	0.0162	0.0026	0.0397	-0.0243	0.4431	0.7682185	Both
Bulk_density	GT2	1796.3105	-50.2538	0.4336	0.3959	0.004	0.0879162	Both
Bulk_density	GT19	83.6067	-3.4872	0.4325	0.3947	0.0041	0.0879162	Both
Bulk_density	GT20	150.6898	1.6401	0.077	0.0154	0.281	0.6821252	Both
Bulk_density	GT21	84.8716	-1.6659	0.0183	-0.0471	0.6043	0.8717623	Both
Bulk_density	GT22	4.4116	0.422	0.1521	0.0955	0.1218	0.4515622	Both
Bulk_density	GT23	0.268	-0.0232	0.0231	-0.042	0.5605	0.8275435	Both
Bulk_density	GT24	2.9064	0.2452	0.0713	0.0094	0.3002	0.6871518	Both
Bulk_density	GT25	6.4045	-0.0697	0.003	-0.0635	0.8356	0.9540309	Both
Bulk_density	GT26	136.246	-1.0288	0.0064	-0.0598	0.7596	0.9439118	Both
Bulk_density	GT27	13.9644	-0.14	0.0064	-0.0598	0.7598	0.9439118	Both
Bulk_density	GT28	175.91	-7.1975	0.5624	0.5332	0.0005	0.0369157	Both
Bulk_density	GT29	3.6067	-0.1447	0.0649	0.0026	0.3237	0.6871518	Both
Bulk_density	GT30	120.3847	-5.0044	0.4253	0.387	0.0046	0.0879162	Both
Bulk_density	GT3	2.973	-0.0072	0.00E+00	-0.0666	0.9811	0.9849794	Both
Bulk_density	GT33	1.4241	0.0186	0.0056	-0.0607	0.7747	0.9439118	Both
Bulk_density	GT34	0.9234	0.2586	0.362	0.3195	0.0106	0.1158478	Both
Bulk_density	GT32	11.5004	1.02	0.4751	0.4401	0.0022	0.0879162	Both
Bulk_density	GT31	1.5165	0.2018	0.0472	-0.0163	0.4023	0.753581	Both
Bulk_density	GT35	365.0227	-25.4217	0.4459	0.409	0.0034	0.0879162	Both
Bulk_density	GT37	0.0087	0.005	0.209	0.1562	0.0651	0.3098875	Both
Bulk_density	GT39	19.8127	1.3137	0.1171	0.0582	0.1788	0.5610853	Both
Bulk_density	GT4	1772.3549	-49.2509	0.2591	0.2097	0.0369	0.257319	Both
Bulk_density	GT41	441.8492	-36.6359	0.5293	0.4979	0.0009	0.0468627	Both
Bulk_density	GT40	0.0018	-0.001	0.1206	0.062	0.1719	0.5532673	Both
Bulk_density	GT42	0.0597	0.0196	0.1792	0.1245	0.0904	0.378262	Both
Bulk_density	GT43	0.0945	-0.0075	0.0139	-0.0518	0.6523	0.8840434	Both
Bulk_density	GT47	70.4249	1.6021	0.0069	-0.0593	0.7515	0.9439118	Both
Bulk_density	GT45	0.2246	-0.0267	0.2343	0.1833	0.049	0.2795735	Both
Bulk_density	GT44	0.0183	-0.0011	0.0035	-0.0629	0.8219	0.9507186	Both
Bulk_density	GT48	3.6784	0.8288	0.189	0.1349	0.0812	0.3705776	Both
Bulk_density	GT49	0.2184	0.04	0.1118	0.0526	0.1896	0.5804287	Both
Bulk_density	GT5	109.5793	-4.9656	0.3262	0.2813	0.0166	0.1524156	Both
Bulk_density	GT50	0.74	0.1519	0.1601	0.1041	0.1116	0.4242782	Both
Bulk_density	GT53	24.3098	2.627	0.0855	0.0245	0.2547	0.6591105	Both
Bulk_density	GT52	0	0	#VALUE!	#VALUE!	#VALUE!	NA	Both
Bulk_density	GT51	564.8885	-14.9661	0.0776	0.0161	0.2791	0.6821252	Both
Bulk_density	GT54	0.0472	0.0073	0.024	-0.0411	0.553	0.8258305	Both
Bulk_density	GT57	1.4811	0.3751	0.2171	0.1649	0.0594	0.2983927	Both
Bulk_density	GT56	0.4345	-0.039	0.0926	0.0321	0.2351	0.6351695	Both
Bulk_density	GT55	2.7129	0.5483	0.3075	0.2613	0.0209	0.1746724	Both
Bulk_density	GT58	0.585	0.1452	0.2435	0.193	0.0441	0.2770206	Both
Bulk_density	GT6	0.39	-0.0696	0.2741	0.2257	0.031	0.2289562	Both
Bulk_density	GT59	0.381	0.1092	0.2645	0.2155	0.0347	0.2486723	Both
Bulk_density	GT60	2.4453	-0.0366	0.0009	-0.0657	0.911	0.9713642	Both
Bulk_density	GT61	0.4011	-0.0097	0.0028	-0.0637	0.84	0.9540309	Both
Bulk_density	GT62	2.1877	0.2282	0.2348	0.1838	0.0487	0.2795735	Both
Bulk_density	GT64	0.7811	-0.033	0.0918	0.0313	0.2371	0.6351695	Both
Bulk_density	GT65	0.0582	0.0022	0.0039	-0.0625	0.8123	0.9439118	Both
Bulk_density	GT66	18.0458	-1.1939	0.3708	0.3288	0.0095	0.1158478	Both
Bulk_density	GT69	0.8355	0.2246	0.2076	0.1548	0.066	0.3098875	Both
Bulk_density	GT68	0.0358	0.0001	0.00E+00	-0.0666	0.9874	0.9874139	Both
Bulk_density	GT7	5.8161	-0.3657	0.2068	0.1539	0.0667	0.3098875	Both
Bulk_density	GT72	0.0008	0.0005	0.0652	0.0028	0.3227	0.6871518	Both
Bulk_density	GT73	0.0593	0.0014	0.0012	-0.0654	0.8957	0.9690635	Both

Bulk_density	GT71	1.411	0.0485	0.0171	-0.0484	0.6171	0.8800865	Both
Bulk_density	GT70	0.7585	0.0281	0.059	-0.0038	0.3477	0.6871518	Both
Bulk_density	GT74	0.956	0.0097	0.0022	-0.0643	0.8583	0.9558926	Both
Bulk_density	GT75	0.9818	-0.0704	0.147	0.0902	0.1287	0.4549731	Both
Bulk_density	GT76	1.9311	0.2314	0.0608	-0.0018	0.3401	0.6871518	Both
Bulk_density	GT77	0.8405	0.055	0.027	-0.0378	0.5284	0.8037519	Both
Bulk_density	GT78	0.0021	0.0006	0.0178	-0.0476	0.6092	0.8737991	Both
Bulk_density	GT8	10.5639	0.0912	0.0066	-0.0597	0.757	0.9439118	Both
Bulk_density	GT80	5.8732	-0.3194	0.1033	0.0436	0.2083	0.5994247	Both
Bulk_density	GT82	0.3983	-0.047	0.1345	0.0768	0.1476	0.5008122	Both
Bulk_density	GT84	309.6313	-31.1232	0.2264	0.1748	0.0536	0.2880453	Both
Bulk_density	GT81	23.411	-0.4751	0.0327	-0.0318	0.4875	0.7854961	Both
Bulk_density	GT83	173.6571	-2.0297	0.0314	-0.0332	0.4964	0.7885575	Both
Bulk_density	GT85	3.4287	0.4568	0.1066	0.047	0.2009	0.586269	Both
Bulk_density	GT87	17.3516	0.912	0.0409	-0.023	0.4363	0.7682185	Both
Bulk_density	GT88	0.0377	0.005	0.0321	-0.0325	0.4917	0.7860553	Both
Bulk_density	GT89	5.6022	0.2766	0.0298	-0.0348	0.5073	0.8008178	Both
Bulk_density	GT90	2.659	0.3152	0.0741	0.0123	0.2906	0.6821252	Both
Bulk_density	GT9	163.8776	-9.3866	0.4292	0.3912	0.0043	0.0879162	Both
Bulk_density	GT91	0.0161	0.0071	0.3277	0.2829	0.0163	0.1524156	Both
Bulk_density	GT93	0.3622	0.0144	0.1348	0.0771	0.1472	0.5008122	Both
Bulk_density	GT94	0.8537	-0.0533	0.0437	-0.0201	0.4207	0.7597218	Both
Bulk_density	GT92	0.1335	0.0256	0.0822	0.021	0.2646	0.6660923	Both
Bulk_density	GT95	6.945	-0.5307	0.3682	0.326	0.0098	0.1158478	Both
Bulk_density	PL1	15.3109	-1.811	0.1394	0.082	0.14	0.487915	Both
Bulk_density	PL10	4.4356	-0.4399	0.0534	-0.0097	0.3723	0.7133975	Both
Bulk_density	PL11	3.0065	-0.7135	0.2446	0.1942	0.0436	0.2770206	Both
Bulk_density	PL12	22.4054	-2.3756	0.6214	0.5962	0.0002	0.035534	Both
Bulk_density	PL13	0.0006	0.0009	0.2241	0.1723	0.055	0.2880453	Both
Bulk_density	PL14	0.8333	0.1347	0.3585	0.3157	0.0111	0.116095	Both
Bulk_density	PL15	0.2413	-0.0719	0.3736	0.3319	0.0091	0.1158478	Both
Bulk_density	PL17	1.2088	-0.2393	0.2457	0.1954	0.043	0.2770206	Both
Bulk_density	PL18	0.0025	-0.0017	0.1275	0.0693	0.1595	0.5337087	Both
Bulk_density	PL16	0.5249	-0.0525	0.2523	0.2024	0.0399	0.2708511	Both
Bulk_density	PL2	0.2055	-0.0484	0.1774	0.1225	0.0923	0.3797028	Both
Bulk_density	PL20	0.0662	-0.0039	0.0055	-0.0608	0.7765	0.9439118	Both
Bulk_density	PL21	0.5529	-0.0738	0.1798	0.1251	0.0898	0.378262	Both
Bulk_density	PL22	9.9118	-0.5201	0.0389	-0.0252	0.4479	0.7700596	Both
Bulk_density	PL23	0.0169	-0.0016	0.0427	-0.0212	0.4264	0.7643983	Both
Bulk_density	PL3	0.1651	-0.0283	0.022	-0.0432	0.5695	0.8311279	Both
Bulk_density	PL4	19.1721	-0.6082	0.0041	-0.0623	0.8068	0.9439118	Both
Bulk_density	PL5	2.8189	0.238	0.0125	-0.0533	0.6692	0.8840434	Both
Bulk_density	PL6	1.5573	-0.4645	0.2121	0.1596	0.0628	0.3092239	Both
Bulk_density	PL7	3.1262	0.014	0.0001	-0.0665	0.9644	0.9800102	Both
Bulk_density	PL8	2.3987	-0.2362	0.0734	0.0116	0.293	0.6821252	Both
Bulk_density	PL9	28.9608	-2.7195	0.1861	0.1318	0.0838	0.3734735	Both
pH	AA1	166.2439	-5.6215	0.0872	0.0392	0.1936	0.292794	Both
pH	AA11	0.4336	-0.0348	0.0155	-0.0363	0.5903	0.6796712	Both
pH	AA10	6.525	-0.7387	0.1649	0.121	0.0677	0.1241256	Both
pH	AA2	1.5806	-0.1076	0.0359	-0.0149	0.4109	0.520915	Both
pH	AA3	414.0022	24.3592	0.2257	0.185	0.0295	0.0622558	Both
pH	AA4	13.9824	-4.3828	0.4216	0.3912	0.0014	0.0065155	Both
pH	AA5	47.5523	10.989	0.6882	0.6718	0.00E+00	6.94E-05	Both
pH	AA8	0.7042	-0.009	0.0009	-0.0517	0.8966	0.9455505	Both
pH	AA7	55.8067	1.7629	0.0364	-0.0143	0.4076	0.5193893	Both

pH	AA6	28.9935	1.9868	0.1438	0.0987	0.09	0.1579722	Both
pH	AA9	1.5682	-0.0213	0.0003	-0.0523	0.9361	0.9748181	Both
pH	CE1	214.5689	0.5422	0.0017	-0.0509	0.8595	0.9219635	Both
pH	CE11	161.0901	1.9131	0.0283	-0.0229	0.4663	0.5737616	Both
pH	CE12	17.1999	-1.7708	0.1207	0.0744	0.1228	0.2041888	Both
pH	CE13	0.0445	-0.021	0.3266	0.2912	0.0068	0.0205728	Both
pH	CE14	222.8524	-6.3968	0.1945	0.1521	0.0454	0.0896979	Both
pH	CE15	41.9051	-7.1518	0.458	0.4294	0.0008	0.0040296	Both
pH	CE2	2.7115	-0.1748	0.019	-0.0326	0.5511	0.6525052	Both
pH	CE3	23.2608	6.2384	0.3169	0.2809	0.0079	0.023016	Both
pH	CE4	354.0802	54.9481	0.7537	0.7407	0.00E+00	1.74E-05	Both
pH	CE16	1.0609	-0.033	0.0115	-0.0405	0.643	0.720472	Both
pH	CE5	14.6374	-1.5433	0.0679	0.0189	0.2539	0.3600583	Both
pH	CE7	11.1596	-0.7754	0.0843	0.0362	0.2015	0.301123	Both
pH	CE6	41.6877	-9.5433	0.7914	0.7804	0.00E+00	1.72E-05	Both
pH	CE8	22.9784	-1.9196	0.1545	0.11	0.078	0.1397725	Both
pH	CE9	93.7508	0.7722	0.0286	-0.0225	0.4634	0.5730308	Both
pH	GH100	0.8338	-0.1961	0.1121	0.0653	0.138	0.2248616	Both
pH	GH10	54.9015	-3.8904	0.2839	0.2462	0.0129	0.0326908	Both
pH	GH102	40.7964	3.6842	0.5411	0.5169	0.0001	0.0011721	Both
pH	GH101	0.7299	0.2228	0.381	0.3485	0.0029	0.0108214	Both
pH	GH103	106.2158	17.0246	0.7285	0.7142	0.00E+00	3.11E-05	Both
pH	GH104	5.1644	0.8936	0.6895	0.6731	0.00E+00	6.94E-05	Both
pH	GH105	29.0354	-7.9003	0.5377	0.5134	0.0002	0.0012204	Both
pH	GH106	49.5372	-10.9961	0.5452	0.5213	0.0001	0.0011461	Both
pH	GH107	0.1563	0.0807	0.3329	0.2977	0.0062	0.019633	Both
pH	GH108	6.355	-0.303	0.1661	0.1222	0.0667	0.123072	Both
pH	GH109	20.0561	-0.8808	0.1217	0.0755	0.1211	0.2026121	Both
pH	GH11	14.887	-2.4078	0.3987	0.3671	0.0021	0.0089573	Both
pH	GH110	1.2431	-0.2485	0.2864	0.2488	0.0124	0.0318239	Both
pH	GH111	0.0414	0.0066	0.0655	0.0163	0.263	0.3667051	Both
pH	GH112	0.0997	-0.0513	0.2589	0.2199	0.0185	0.0446513	Both
pH	GH113	8.2393	1.0865	0.3632	0.3297	0.0038	0.0131772	Both
pH	GH114	10.5907	1.6105	0.2277	0.1871	0.0287	0.061046	Both
pH	GH115	18.1582	-4.1931	0.2402	0.2002	0.0241	0.0536721	Both
pH	GH116	44.574	-7.9009	0.5963	0.575	0.00E+00	0.0004687	Both
pH	GH117	3.4772	-0.2127	0.053	0.0031	0.3156	0.4305061	Both
pH	GH118	0.0029	0.0005	0.0248	-0.0265	0.4954	0.5978329	Both
pH	GH119	2.7623	0.7945	0.4739	0.4463	0.0006	0.0032685	Both
pH	GH12	8.8559	-1.3804	0.2535	0.2143	0.02	0.0472692	Both
pH	GH120	1.8739	-0.6435	0.4773	0.4498	0.0005	0.0031388	Both
pH	GH121	1.5942	0.6147	0.2426	0.2027	0.0233	0.0536721	Both
pH	GH123	1.8672	0.8456	0.7137	0.6986	0.00E+00	4.05E-05	Both
pH	GH124	0.0511	0.0231	0.3284	0.293	0.0066	0.0202632	Both
pH	GH125	11.4055	-2.3256	0.5019	0.4757	0.0003	0.0022052	Both
pH	GH126	0.0093	-0.0051	0.2403	0.2004	0.0241	0.0536721	Both
pH	GH127	44.4763	-6.6833	0.4658	0.4377	0.0007	0.0037213	Both
pH	GH128	6.9808	-1.5315	0.3674	0.3341	0.0036	0.0124872	Both
pH	GH129	0.3906	-0.0687	0.2012	0.1592	0.0414	0.0824729	Both
pH	GH13	1501.8611	-17.0014	0.0228	-0.0286	0.5132	0.6134289	Both
pH	GH130	86.8053	-1.8416	0.0174	-0.0343	0.5681	0.663272	Both
pH	GH131	0.195	-0.0157	0.0162	-0.0355	0.582	0.6751327	Both
pH	GH132	0.7589	-0.0934	0.0439	-0.0064	0.3619	0.4780399	Both
pH	GH133	72.2732	5.2504	0.1809	0.1378	0.0546	0.105416	Both
pH	GH14	0.5412	-0.082	0.2762	0.2381	0.0144	0.0351452	Both

pH	GH17	59.077	7.8104	0.5991	0.578	0.00E+00	0.0004585	Both
pH	GH15	408.8678	-25.4747	0.4225	0.3921	0.0014	0.0065155	Both
pH	GH18	245.204	-10.9348	0.1448	0.0998	0.0888	0.157029	Both
pH	GH19	16.9586	0.4741	0.0181	-0.0336	0.5607	0.6607791	Both
pH	GH2	388.7654	-44.8291	0.4401	0.4106	0.001	0.0050428	Both
pH	GH20	100.202	-5.0377	0.2382	0.1981	0.0248	0.054594	Both
pH	GH23	392.3336	18.614	0.32	0.2842	0.0075	0.0224786	Both
pH	GH24	15.9645	-0.2864	0.0278	-0.0234	0.4699	0.5753835	Both
pH	GH25	19.8627	0.5564	0.0175	-0.0342	0.5677	0.663272	Both
pH	GH26	22.0532	-0.9947	0.047	-0.0031	0.345	0.4605706	Both
pH	GH27	80.433	-17.4074	0.5702	0.5476	1.00E-04	0.0007613	Both
pH	GH28	194.68	-28.7573	0.4344	0.4046	0.0012	0.0054742	Both
pH	GH29	112.4106	-15.5964	0.6641	0.6464	0.00E+00	0.0001227	Both
pH	GH3	587.4424	-75.5555	0.6307	0.6112	0.00E+00	0.0002701	Both
pH	GH30	51.6227	-6.183	0.4494	0.4204	0.0009	0.0044316	Both
pH	GH31	139.0432	-27.039	0.7536	0.7406	0.00E+00	1.74E-05	Both
pH	GH33	59.3874	7.5658	0.4863	0.4593	0.0004	0.0028424	Both
pH	GH36	91.5089	-18.7422	0.6402	0.6213	0.00E+00	0.0002232	Both
pH	GH32	31.3611	0.6382	0.0533	0.0034	0.3141	0.4305061	Both
pH	GH37	39.0498	-7.0414	0.6816	0.6648	0.00E+00	7.85E-05	Both
pH	GH39	89.459	-12.7645	0.4879	0.461	0.0004	0.0028282	Both
pH	GH38	117.6702	-25.8711	0.6025	0.5816	0.00E+00	0.0004585	Both
pH	GH4	144.275	4.4986	0.039	-0.0116	0.3909	0.5047768	Both
pH	GH42	45.2312	-4.5987	0.4538	0.425	0.0008	0.0042614	Both
pH	GH43	113.62	-14.8826	0.2408	0.2008	0.0239	0.0536721	Both
pH	GH44	26.6648	-2.0273	0.0987	0.0512	0.1656	0.2556583	Both
pH	GH45	0.4552	-0.0618	0.0934	0.0457	0.1779	0.2705663	Both
pH	GH47	15.1347	-0.3625	0.0207	-0.0308	0.5336	0.6347184	Both
pH	GH46	2.1056	0.4219	0.1256	0.0795	0.115	0.196374	Both
pH	GH48	9.684	-0.7876	0.2053	0.1634	0.0392	0.0798977	Both
pH	GH49	0.1506	0.0946	0.3118	0.2756	0.0085	0.0240067	Both
pH	GH5	170.4089	0.4138	0.0003	-0.0523	0.9399	0.9748181	Both
pH	GH50	7.8055	-0.5018	0.0637	0.0144	0.2697	0.3740286	Both
pH	GH51	110.7785	-18.2482	0.5527	0.5292	0.0001	0.0010447	Both
pH	GH54	21.4612	-9.2476	0.6252	0.6055	0.00E+00	0.000293	Both
pH	GH53	57.7565	-2.7084	0.0691	0.0201	0.2498	0.3562336	Both
pH	GH55	111.9622	-28.2216	0.7531	0.7401	0.00E+00	1.74E-05	Both
pH	GH56	0.0046	-0.0031	0.232	0.1916	0.027	0.0585851	Both
pH	GH57	153.7528	4.3224	0.0802	0.0318	0.2134	0.3169348	Both
pH	GH58	0.039	0.0139	0.3036	0.2669	0.0096	0.0262963	Both
pH	GH59	8.0215	-3.1127	0.6001	0.579	0.00E+00	0.0004585	Both
pH	GH6	18.8877	-2.0069	0.1423	0.0972	0.0918	0.160008	Both
pH	GH62	3.2816	-0.0435	0.0011	-0.0515	0.8864	0.9417223	Both
pH	GH63	15.2609	-1.6836	0.1788	0.1356	0.0562	0.1068219	Both
pH	GH64	20.0853	-2.9086	0.4788	0.4513	0.0005	0.0031388	Both
pH	GH65	100.4831	-8.6185	0.1552	0.1107	0.0772	0.1394878	Both
pH	GH66	1.539	0.3224	0.1798	0.1366	0.0554	0.1061891	Both
pH	GH68	0.282	0.0228	0.1236	0.0774	0.1182	0.2004142	Both
pH	GH7	0.7235	-0.0042	1.00E-04	-0.0526	0.9726	0.9883403	Both
pH	GH70	0.0383	0.0115	0.1515	0.1069	0.0812	0.1444625	Both
pH	GH71	3.9384	-0.4588	0.0657	0.0166	0.2619	0.3667051	Both
pH	GH72	30.0493	0.2878	0.008	-0.0442	0.6995	0.7700874	Both
pH	GH73	38.6432	1.2407	0.1066	0.0596	0.1486	0.2375518	Both
pH	GH75	2.2066	-0.0716	0.0148	-0.0371	0.5993	0.686924	Both
pH	GH74	13.9963	-0.7886	0.0523	0.0024	0.3187	0.4323842	Both

pH	GH76	14.814	-2.6904	0.3345	0.2995	0.006	0.0193702	Both
pH	GH78	155.8729	-14.0428	0.387	0.3548	0.0026	0.0101015	Both
pH	GH79	28.1345	-4.6377	0.5685	0.5458	1.00E-04	0.0007613	Both
pH	GH8	19.2344	-0.8306	0.0292	-0.0219	0.459	0.5703403	Both
pH	GH80	0.002	-0.0013	0.0346	-0.0162	0.4194	0.5289661	Both
pH	GH81	5.7722	0.8389	0.241	0.201	0.0238	0.0536721	Both
pH	GH84	4.781	0.012	0.0002	-0.0524	0.9508	0.9794589	Both
pH	GH85	0.6123	0.0243	0.0086	-0.0436	0.6895	0.7632179	Both
pH	GH86	1.587	0.3696	0.6123	0.5919	0.00E+00	0.0003859	Both
pH	GH87	68.8594	-6.2536	0.3059	0.2694	0.0093	0.0256528	Both
pH	GH88	24.4555	1.425	0.3932	0.3612	0.0023	0.0096646	Both
pH	GH89	7.7554	-1.9449	0.5153	0.4898	0.0002	0.0017806	Both
pH	GH91	0.0888	0.0391	0.2979	0.2609	0.0105	0.0279381	Both
pH	GH90	0.0034	0.0031	0.3298	0.2945	0.0065	0.0200629	Both
pH	GH9	54.2204	-7.5905	0.337	0.3022	0.0058	0.0188765	Both
pH	GH92	99.629	-19.609	0.4203	0.3898	0.0015	0.0065155	Both
pH	GH93	21.1746	3.2516	0.0864	0.0383	0.196	0.2946121	Both
pH	GH94	30.4978	-7.0171	0.5249	0.4999	0.0002	0.0015046	Both
pH	GH95	51.1365	-3.3772	0.1145	0.0679	0.1335	0.2190407	Both
pH	GH96	0.4341	0.1919	0.2563	0.2171	0.0192	0.0459054	Both
pH	GH97	31.9027	-0.008	0.00E+00	-0.0526	0.9954	0.9973103	Both
pH	GH98	0.2928	0.0997	0.7704	0.7583	0.00E+00	1.74E-05	Both
pH	GH99	12.1809	1.5641	0.2892	0.2517	0.0119	0.0308687	Both
pH	GT1	152.7224	-0.1704	0.00E+00	-0.0526	0.9803	0.989416	Both
pH	GT10	2.6221	-0.2007	0.0997	0.0523	0.1632	0.255569	Both
pH	GT12	0.0479	-0.004	0.0388	-0.0118	0.3922	0.5047768	Both
pH	GT14	2.0818	-0.2877	0.3131	0.277	0.0083	0.023813	Both
pH	GT11	5.172	-0.4332	0.1779	0.1346	0.0569	0.1070486	Both
pH	GT13	0.1791	-0.0867	0.5067	0.4807	0.0003	0.0020604	Both
pH	GT15	2.0332	-0.2674	0.0708	0.0219	0.2436	0.3534596	Both
pH	GT16	0.0469	-0.0204	0.311	0.2748	0.0086	0.0240249	Both
pH	GT17	1.0854	-0.0562	0.0704	0.0214	0.2452	0.3536948	Both
pH	GT18	0.0144	-0.0081	0.3807	0.3481	0.0029	0.0108214	Both
pH	GT2	1818.8004	48.3994	0.3177	0.2818	0.0078	0.0230026	Both
pH	GT19	83.4957	-0.555	0.0129	-0.0391	0.624	0.7087414	Both
pH	GT20	149.4495	-0.6707	0.0117	-0.0403	0.641	0.720472	Both
pH	GT21	78.1092	-13.5441	0.5467	0.5229	0.0001	0.0011461	Both
pH	GT22	4.5977	-0.2276	0.0388	-0.0118	0.3921	0.5047768	Both
pH	GT23	0.2539	-0.0877	0.3877	0.3554	0.0026	0.0101015	Both
pH	GT24	2.8985	-0.472	0.2795	0.2416	0.0137	0.034159	Both
pH	GT25	6.3037	-0.6302	0.2821	0.2443	0.0132	0.0332099	Both
pH	GT26	137.4511	-3.0797	0.0659	0.0168	0.2612	0.3667051	Both
pH	GT27	13.6554	-1.1842	0.4781	0.4507	0.0005	0.0031388	Both
pH	GT28	177.389	1.9211	0.0427	-0.0077	0.3687	0.4819736	Both
pH	GT29	3.7879	0.4002	0.3567	0.3228	0.0043	0.0144347	Both
pH	GT30	120.5539	0.0054	0.00E+00	-0.0526	0.9973	0.9973103	Both
pH	GT3	3.0716	-0.5219	0.2093	0.1677	0.0371	0.0762365	Both
pH	GT33	1.6363	0.3457	0.4619	0.4336	0.0007	0.003911	Both
pH	GT34	0.9693	-0.1019	0.0483	-0.0018	0.3383	0.4541482	Both
pH	GT32	12.037	1.177	0.3745	0.3416	0.0032	0.0114837	Both
pH	GT31	1.5981	-0.365	0.1558	0.1114	0.0766	0.1393047	Both
pH	GT35	354.1091	-27.7873	0.4518	0.423	0.0008	0.0043266	Both
pH	GT37	0.0077	-1.00E-04	0.00E+00	-0.0526	0.9815	0.989416	Both
pH	GT39	21.0631	-0.1764	0.0013	-0.0513	0.8768	0.9365408	Both
pH	GT4	1835.3972	123.2995	0.5788	0.5567	1.00E-04	0.0006506	Both

pH	GT41	429.2439	-29.3581	0.2988	0.2619	0.0104	0.0279367	Both
pH	GT40	0.0014	-0.0009	0.1022	0.0549	0.1578	0.2490736	Both
pH	GT42	0.0596	-0.0087	0.0385	-0.0121	0.3942	0.5047768	Both
pH	GT43	0.0839	-0.0373	0.3512	0.3171	0.0046	0.0155294	Both
pH	GT47	70.9951	-5.0104	0.0749	0.0262	0.2299	0.3374353	Both
pH	GT45	0.2403	0.0283	0.2024	0.1604	0.0407	0.0817474	Both
pH	GT44	0.0196	0.0013	0.0052	-0.0472	0.756	0.8250644	Both
pH	GT48	3.6235	-0.859	0.2249	0.1841	0.0299	0.0623699	Both
pH	GT49	0.2071	-0.0721	0.4119	0.3809	0.0017	0.0074072	Both
pH	GT5	110.3216	3.804	0.2278	0.1871	0.0287	0.061046	Both
pH	GT50	0.7517	-0.1588	0.1775	0.1342	0.0571	0.1070486	Both
pH	GT53	24.0821	-2.8441	0.1218	0.0756	0.121	0.2026121	Both
pH	GT52	0	0	#VALUE!	#VALUE!	#VALUE!	NA	Both
pH	GT51	587.598	58.3373	0.7356	0.7216	0.00E+00	2.82E-05	Both
pH	GT54	0.0432	-0.0274	0.4019	0.3704	0.002	0.0086364	Both
pH	GT57	1.512	-0.3472	0.1853	0.1425	0.0514	0.0999944	Both
pH	GT56	0.434	-0.0716	0.3867	0.3544	0.0026	0.0101015	Both
pH	GT55	2.7859	-0.2978	0.0984	0.051	0.166	0.2556583	Both
pH	GT58	0.5931	-0.1203	0.1668	0.1229	0.0661	0.122871	Both
pH	GT6	0.3486	-0.0407	0.0755	0.0268	0.228	0.3367056	Both
pH	GT59	0.4033	-0.07	0.1046	0.0575	0.1526	0.2424357	Both
pH	GT60	2.2724	-0.9885	0.7021	0.6864	0.00E+00	5.35E-05	Both
pH	GT61	0.4009	-0.0948	0.316	0.28	0.008	0.0230502	Both
pH	GT62	2.2504	-0.0546	0.0117	-0.0403	0.6407	0.720472	Both
pH	GT64	0.8578	0.0989	0.2411	0.2011	0.0238	0.0536721	Both
pH	GT65	0.0525	-0.0254	0.5412	0.5171	0.0001	0.0011721	Both
pH	GT66	19.0025	1.3699	0.2244	0.1836	0.0301	0.0623699	Both
pH	GT69	0.8788	-0.1927	0.1401	0.0949	0.0946	0.1637347	Both
pH	GT68	0.033	-0.0132	0.24	0.2	0.0242	0.0536721	Both
pH	GT7	5.6797	-0.5078	0.3871	0.3549	0.0026	0.0101015	Both
pH	GT72	0.0007	-1.00E-04	0.001	-0.0515	0.8892	0.9417223	Both
pH	GT73	0.0676	0.0007	0.0002	-0.0524	0.9521	0.9794589	Both
pH	GT71	1.4697	-0.142	0.1276	0.0817	0.1119	0.1923952	Both
pH	GT70	0.7719	0.0198	0.0241	-0.0272	0.5012	0.6019224	Both
pH	GT74	0.8743	-0.0159	0.0037	-0.0487	0.7927	0.8613042	Both
pH	GT75	0.9562	-0.0186	0.0097	-0.0425	0.6715	0.7491087	Both
pH	GT76	2.0735	0.6068	0.4432	0.4139	0.001	0.0048632	Both
pH	GT77	0.8498	0.2012	0.4208	0.3903	0.0015	0.0065155	Both
pH	GT78	0.0021	1.00E-04	0.0004	-0.0523	0.9355	0.9748181	Both
pH	GT8	11.3981	0.5912	0.0696	0.0207	0.2478	0.3553751	Both
pH	GT80	5.8542	-0.2877	0.0973	0.0498	0.1685	0.25795	Both
pH	GT82	0.4408	0.0975	0.3789	0.3462	0.003	0.0109758	Both
pH	GT84	302.7752	-2.8871	0.0022	-0.0503	0.8385	0.9033207	Both
pH	GT81	24.2095	0.5024	0.0253	-0.026	0.4913	0.5957345	Both
pH	GT83	173.1688	-1.6967	0.0161	-0.0357	0.5837	0.6751327	Both
pH	GT85	3.3791	-0.4245	0.1114	0.0646	0.1393	0.2255936	Both
pH	GT87	17.8526	-1.8532	0.1892	0.1465	0.0488	0.0956298	Both
pH	GT88	0.0319	-0.0211	0.5793	0.5571	1.00E-04	0.0006506	Both
pH	GT89	5.7995	-0.175	0.0134	-0.0385	0.6172	0.7041947	Both
pH	GT90	2.7262	-0.5271	0.2026	0.1606	0.0406	0.0817474	Both
pH	GT9	169.2922	12.5454	0.5329	0.5084	0.0002	0.0013095	Both
pH	GT91	0.0175	-0.0001	1.00E-04	-0.0525	0.9694	0.9883403	Both
pH	GT93	0.367	-0.0034	0.0071	-0.0452	0.7166	0.7854779	Both
pH	GT94	0.8321	-0.1597	0.3735	0.3405	0.0032	0.0114837	Both
pH	GT92	0.1294	-0.0454	0.2973	0.2603	0.0106	0.0279381	Both

pH	GT95	6.8457	0.1785	0.0428	-0.0076	0.3685	0.4819736	Both
pH	PL1	17.2792	1.6738	0.0632	0.0138	0.2718	0.3748805	Both
pH	PL10	4.8889	-0.4856	0.0514	0.0015	0.3231	0.4359555	Both
pH	PL11	3.4173	0.2994	0.0317	-0.0192	0.4397	0.5518275	Both
pH	PL12	23.1583	0.1943	0.0035	-0.0489	0.799	0.8643979	Both
pH	PL13	0.0005	-0.0008	0.2319	0.1915	0.0271	0.0585851	Both
pH	PL14	1.0529	0.3534	0.4587	0.4302	0.0007	0.0040296	Both
pH	PL15	0.3252	0.1276	0.3739	0.3409	0.0032	0.0114837	Both
pH	PL17	1.5798	0.4939	0.2787	0.2407	0.0139	0.0342231	Both
pH	PL18	0.003	-1.00E-04	0.0001	-0.0525	0.9589	0.9823933	Both
pH	PL16	0.5166	-0.0385	0.1145	0.0679	0.1335	0.2190407	Both
pH	PL2	0.1859	-0.0604	0.2926	0.2554	0.0113	0.0296351	Both
pH	PL20	0.0885	0.0067	0.0085	-0.0436	0.6902	0.7632179	Both
pH	PL21	0.5039	-0.108	0.3302	0.2949	0.0064	0.0200629	Both
pH	PL22	10.1314	-0.7596	0.0994	0.052	0.1639	0.255569	Both
pH	PL23	0.0169	-0.0013	0.0309	-0.0201	0.4459	0.5568135	Both
pH	PL3	0.2609	0.0871	0.0716	0.0228	0.2408	0.3514351	Both
pH	PL4	18.5959	-5.0184	0.3399	0.3052	0.0055	0.0182928	Both
pH	PL5	2.4899	-1.7233	0.719	0.7042	0.00E+00	3.80E-05	Both
pH	PL6	1.9888	0.4417	0.1085	0.0616	0.1448	0.2330089	Both
pH	PL7	3.6439	-0.0291	0.0003	-0.0523	0.9375	0.9748181	Both
pH	PL8	2.6452	-0.2066	0.0452	-0.0051	0.3551	0.4715519	Both
pH	PL9	31.9165	1.4633	0.0274	-0.0238	0.4733	0.5767212	Both
C	AA1	166.2439	11.684	0.3769	0.3441	0.0031	0.0074899	Both
C	AA11	0.4336	0.1077	0.1492	0.1044	0.0837	0.1338387	Both
C	AA10	6.525	1.2777	0.4934	0.4667	0.0004	0.0012237	Both
C	AA2	1.5806	0.2968	0.273	0.2347	0.0151	0.0311744	Both
C	AA3	414.0022	-26.6568	0.2703	0.2319	0.0157	0.0320337	Both
C	AA4	13.9824	6.1179	0.8216	0.8122	0.00E+00	2.39E-07	Both
C	AA5	47.5523	-12.2601	0.8566	0.8491	0.00E+00	5.24E-08	Both
C	AA8	0.7042	0.1294	0.1891	0.1465	0.0488	0.0844811	Both
C	AA7	55.8067	-2.984	0.1042	0.0571	0.1535	0.2214431	Both
C	AA6	28.9935	-3.9073	0.5562	0.5328	0.0001	0.000402	Both
C	AA9	1.5682	0.3198	0.078	0.0295	0.2201	0.2969594	Both
C	CE1	214.5689	-2.952	0.0501	0.0001	0.3292	0.4215962	Both
C	CE11	161.0901	-1.1027	0.0094	-0.0427	0.676	0.7377685	Both
C	CE12	17.1999	3.793	0.5537	0.5302	0.0001	0.0004182	Both
C	CE13	0.0445	0.0189	0.2661	0.2275	0.0167	0.0335006	Both
C	CE14	222.8524	7.4059	0.2607	0.2218	0.018	0.034814	Both
C	CE15	41.9051	9.7652	0.8538	0.8461	0.00E+00	5.67E-08	Both
C	CE2	2.7115	0.6477	0.261	0.2221	0.018	0.034814	Both
C	CE3	23.2608	-4.4976	0.1647	0.1208	0.0679	0.1129266	Both
C	CE4	354.0802	-50.3894	0.6338	0.6145	0.00E+00	7.80E-05	Both
C	CE16	1.0609	0.0859	0.0782	0.0297	0.2195	0.2969594	Both
C	CE5	14.6374	-0.7274	0.0151	-0.0368	0.5958	0.6646853	Both
C	CE7	11.1596	1.8539	0.4822	0.4549	0.0005	0.0014643	Both
C	CE6	41.6877	9.7466	0.8255	0.8163	0.00E+00	2.06E-07	Both
C	CE8	22.9784	2.8176	0.3328	0.2977	0.0062	0.0139799	Both
C	CE9	93.7508	-1.2999	0.0811	0.0327	0.2108	0.2891547	Both
C	GH100	0.8338	0.3543	0.3658	0.3324	0.0037	0.0087887	Both
C	GH10	54.9015	5.1436	0.4962	0.4697	0.0004	0.0011877	Both
C	GH102	40.7964	-4.1472	0.6856	0.669	0.00E+00	2.51E-05	Both
C	GH101	0.7299	-0.1885	0.2728	0.2345	0.0152	0.0311744	Both
C	GH103	106.2158	-15.0012	0.5656	0.5428	1.00E-04	0.0003355	Both
C	GH104	5.1644	-0.6671	0.3843	0.3519	0.0027	0.0066994	Both

C	GH105	29.0354	9.6036	0.7946	0.7837	0.00E+00	8.25E-07	Both
C	GH106	49.5372	13.8538	0.8654	0.8583	0.00E+00	3.68E-08	Both
C	GH107	0.1563	-0.062	0.1965	0.1542	0.0442	0.0786096	Both
C	GH108	6.355	0.1153	0.024	-0.0273	0.5022	0.5755382	Both
C	GH109	20.0561	1.2045	0.2277	0.1871	0.0287	0.0518497	Both
C	GH11	14.887	3.3126	0.7547	0.7417	0.00E+00	3.73E-06	Both
C	GH110	1.2431	0.3482	0.5621	0.5391	1.00E-04	0.0003577	Both
C	GH111	0.0414	-0.0075	0.0864	0.0383	0.196	0.2733585	Both
C	GH112	0.0997	0.0782	0.6001	0.579	0.00E+00	0.000168	Both
C	GH113	8.2393	-0.3408	0.0357	-0.015	0.4118	0.4945022	Both
C	GH114	10.5907	-2.2421	0.4414	0.412	0.001	0.0027303	Both
C	GH115	18.1582	7.5459	0.7779	0.7662	0.00E+00	1.57E-06	Both
C	GH116	44.574	7.8205	0.5842	0.5623	1.00E-04	0.0002373	Both
C	GH117	3.4772	0.0613	0.0044	-0.048	0.7753	0.8176663	Both
C	GH118	0.0029	-0.0004	0.0163	-0.0355	0.5814	0.6514774	Both
C	GH119	2.7623	-0.5653	0.2399	0.1999	0.0242	0.0440338	Both
C	GH12	8.8559	1.984	0.5238	0.4987	0.0002	0.0007374	Both
C	GH120	1.8739	0.2548	0.0748	0.0261	0.2303	0.3075396	Both
C	GH121	1.5942	-0.4514	0.1309	0.0851	0.1071	0.1659681	Both
C	GH123	1.8672	-0.8268	0.6823	0.6656	0.00E+00	2.70E-05	Both
C	GH124	0.0511	-0.0294	0.532	0.5074	0.0002	0.0006293	Both
C	GH125	11.4055	2.434	0.5498	0.5261	0.0001	0.000442	Both
C	GH126	0.0093	0.0025	0.0563	0.0067	0.3002	0.3883147	Both
C	GH127	44.4763	8.5529	0.7629	0.7504	0.00E+00	2.81E-06	Both
C	GH128	6.9808	1.9974	0.625	0.6053	0.00E+00	9.64E-05	Both
C	GH129	0.3906	0.0547	0.1277	0.0818	0.1117	0.1720371	Both
C	GH13	1501.8611	-8.4896	0.0057	-0.0466	0.7451	0.7924935	Both
C	GH130	86.8053	7.8572	0.3176	0.2817	0.0078	0.0171703	Both
C	GH131	0.195	0.0637	0.2663	0.2277	0.0166	0.0335006	Both
C	GH132	0.7589	0.1948	0.1909	0.1484	0.0476	0.0835922	Both
C	GH133	72.2732	-10.7104	0.7527	0.7397	0.00E+00	3.85E-06	Both
C	GH14	0.5412	0.1083	0.4813	0.454	0.0005	0.0014729	Both
C	GH17	59.077	-6.1424	0.3705	0.3374	0.0034	0.0082222	Both
C	GH15	408.8678	10.9509	0.0781	0.0295	0.22	0.2969594	Both
C	GH18	245.204	15.6619	0.297	0.26	0.0106	0.0228829	Both
C	GH19	16.9586	-0.2707	0.0059	-0.0464	0.7406	0.7909753	Both
C	GH2	388.7654	59.9275	0.7864	0.7752	0.00E+00	1.14E-06	Both
C	GH20	100.202	5.7347	0.3087	0.2723	0.0089	0.0194853	Both
C	GH23	392.3336	-15.5177	0.2224	0.1815	0.0309	0.0554192	Both
C	GH24	15.9645	0.1557	0.0082	-0.044	0.696	0.7523264	Both
C	GH25	19.8627	-0.5795	0.019	-0.0327	0.5516	0.6208224	Both
C	GH26	22.0532	1.9273	0.1766	0.1333	0.0579	0.0987901	Both
C	GH27	80.433	21.5944	0.8776	0.8711	0.00E+00	2.08E-08	Both
C	GH28	194.68	40.9838	0.8823	0.8761	0.00E+00	1.79E-08	Both
C	GH29	112.4106	16.5318	0.7461	0.7327	0.00E+00	4.57E-06	Both
C	GH3	587.4424	86.6034	0.8286	0.8196	0.00E+00	1.86E-07	Both
C	GH30	51.6227	7.6365	0.6855	0.669	0.00E+00	2.51E-05	Both
C	GH31	139.0432	28.5707	0.8414	0.833	0.00E+00	1.13E-07	Both
C	GH33	59.3874	-6.7402	0.386	0.3537	0.0026	0.0065774	Both
C	GH36	91.5089	22.365	0.9116	0.907	0.00E+00	2.79E-09	Both
C	GH32	31.3611	-1.4212	0.2642	0.2255	0.0171	0.0338545	Both
C	GH37	39.0498	5.8736	0.4742	0.4466	0.0006	0.0016438	Both
C	GH39	89.459	17.4304	0.9099	0.9051	0.00E+00	2.79E-09	Both
C	GH38	117.6702	31.176	0.875	0.8684	0.00E+00	2.12E-08	Both
C	GH4	144.275	-1.0531	0.0021	-0.0504	0.8423	0.8700138	Both

C	GH42	45.2312	5.8004	0.7219	0.7073	0.00E+00	9.82E-06	Both
C	GH43	113.62	21.2372	0.4903	0.4635	0.0004	0.0012714	Both
C	GH44	26.6648	5.2639	0.6651	0.6475	0.00E+00	3.97E-05	Both
C	GH45	0.4552	0.1381	0.4673	0.4392	0.0006	0.0017909	Both
C	GH47	15.1347	0.5002	0.0395	-0.0111	0.3881	0.4682932	Both
C	GH46	2.1056	-0.3992	0.1125	0.0657	0.1373	0.2038514	Both
C	GH48	9.684	1.0947	0.3966	0.3648	0.0022	0.0056857	Both
C	GH49	0.1506	-0.0234	0.0191	-0.0326	0.5506	0.6208224	Both
C	GH5	170.4089	5.5844	0.056	0.0063	0.3017	0.3883147	Both
C	GH50	7.8055	-0.2931	0.0217	-0.0298	0.5238	0.5948753	Both
C	GH51	110.7785	23.3322	0.9036	0.8985	0.00E+00	3.54E-09	Both
C	GH54	21.4612	10.7104	0.8387	0.8302	0.00E+00	1.21E-07	Both
C	GH53	57.7565	7.9607	0.5966	0.5754	0.00E+00	0.0001796	Both
C	GH55	111.9622	29.6181	0.8295	0.8205	0.00E+00	1.86E-07	Both
C	GH56	0.0046	0.0038	0.3475	0.3131	0.0049	0.0114357	Both
C	GH57	153.7528	-4.9678	0.106	0.0589	0.1498	0.21864	Both
C	GH58	0.039	-0.0061	0.0593	0.0098	0.2873	0.3736517	Both
C	GH59	8.0215	3.4811	0.7505	0.7374	0.00E+00	4.01E-06	Both
C	GH6	18.8877	3.4195	0.4132	0.3823	0.0017	0.0043735	Both
C	GH62	3.2816	0.3511	0.0719	0.0231	0.2398	0.3176847	Both
C	GH63	15.2609	2.8113	0.4985	0.4721	0.0003	0.0011495	Both
C	GH64	20.0853	3.1729	0.5697	0.5471	1.00E-04	0.0003158	Both
C	GH65	100.4831	0.1569	1.00E-04	-0.0526	0.9754	0.975387	Both
C	GH66	1.539	0.1199	0.0248	-0.0265	0.495	0.5699431	Both
C	GH68	0.282	-0.001	0.0002	-0.0524	0.9482	0.9674225	Both
C	GH7	0.7235	0.1903	0.1338	0.0882	0.103	0.1605458	Both
C	GH70	0.0383	-0.0003	0.0001	-0.0525	0.9623	0.9728197	Both
C	GH71	3.9384	1.0438	0.3402	0.3055	0.0055	0.0126977	Both
C	GH72	30.0493	0.7908	0.0605	0.0111	0.2824	0.369115	Both
C	GH73	38.6432	-1.1646	0.0939	0.0462	0.1766	0.2504638	Both
C	GH75	2.2066	0.0623	0.0112	-0.0408	0.6479	0.7101505	Both
C	GH74	13.9963	2.2906	0.4413	0.4119	0.001	0.0027303	Both
C	GH76	14.814	3.1854	0.469	0.441	0.0006	0.0017542	Both
C	GH78	155.8729	18.5247	0.6735	0.6563	0.00E+00	3.26E-05	Both
C	GH79	28.1345	4.306	0.4901	0.4633	0.0004	0.0012714	Both
C	GH8	19.2344	1.2098	0.0619	0.0126	0.2767	0.3635753	Both
C	GH80	0.002	-0.0015	0.0465	-0.0037	0.348	0.4389728	Both
C	GH81	5.7722	-0.2718	0.0253	-0.026	0.4912	0.5681048	Both
C	GH84	4.781	0.2748	0.1083	0.0613	0.1453	0.2145025	Both
C	GH85	0.6123	-0.0144	0.003	-0.0495	0.8133	0.8435129	Both
C	GH86	1.587	-0.2946	0.389	0.3569	0.0025	0.0063154	Both
C	GH87	68.8594	9.7533	0.7442	0.7307	0.00E+00	4.72E-06	Both
C	GH88	24.4555	-0.7126	0.0983	0.0509	0.1663	0.2385784	Both
C	GH89	7.7554	2.2939	0.7168	0.7019	0.00E+00	1.13E-05	Both
C	GH91	0.0888	-0.0312	0.1899	0.1472	0.0483	0.0842471	Both
C	GH90	0.0034	-0.0012	0.0461	-0.0041	0.3499	0.4390942	Both
C	GH9	54.2204	7.7461	0.351	0.3169	0.0047	0.010919	Both
C	GH92	99.629	21.6584	0.5128	0.4871	0.0003	0.0008989	Both
C	GH93	21.1746	0.1001	1.00E-04	-0.0525	0.9689	0.9728197	Both
C	GH94	30.4978	7.7202	0.6353	0.6162	0.00E+00	7.63E-05	Both
C	GH95	51.1365	6.5271	0.4277	0.3976	0.0013	0.0034362	Both
C	GH96	0.4341	-0.1558	0.1689	0.1252	0.0642	0.1074469	Both
C	GH97	31.9027	-0.1005	0.0003	-0.0523	0.942	0.9650226	Both
C	GH98	0.2928	-0.0922	0.6586	0.6406	0.00E+00	4.59E-05	Both
C	GH99	12.1809	-1.4632	0.253	0.2137	0.0201	0.0376624	Both

C	GT1	152.7224	-1.6543	0.0031	-0.0494	0.8105	0.8435129	Both
C	GT10	2.6221	0.4712	0.5499	0.5262	0.0001	0.000442	Both
C	GT12	0.0479	0.0035	0.0296	-0.0214	0.4556	0.5343851	Both
C	GT14	2.0818	0.2024	0.1551	0.1106	0.0774	0.1255773	Both
C	GT11	5.172	0.6413	0.3898	0.3577	0.0025	0.0062971	Both
C	GT13	0.1791	0.0987	0.6567	0.6386	0.00E+00	4.71E-05	Both
C	GT15	2.0332	0.5065	0.2541	0.2148	0.0198	0.0376624	Both
C	GT16	0.0469	0.0312	0.7273	0.7129	0.00E+00	8.43E-06	Both
C	GT17	1.0854	0.1213	0.3278	0.2924	0.0067	0.0149727	Both
C	GT18	0.0144	0.0106	0.6584	0.6404	0.00E+00	4.59E-05	Both
C	GT2	1818.8004	-29.0883	0.1148	0.0682	0.1331	0.1987897	Both
C	GT19	83.4957	1.3364	0.0748	0.0261	0.2303	0.3075396	Both
C	GT20	149.4495	-2.4898	0.161	0.1168	0.0714	0.117985	Both
C	GT21	78.1092	12.2269	0.4456	0.4164	0.0009	0.0025822	Both
C	GT22	4.5977	0.5934	0.2637	0.225	0.0173	0.0338545	Both
C	GT23	0.2539	0.1182	0.7041	0.6885	0.00E+00	1.52E-05	Both
C	GT24	2.8985	0.6435	0.5193	0.494	0.0002	0.0007974	Both
C	GT25	6.3037	1	0.7103	0.695	0.00E+00	1.32E-05	Both
C	GT26	137.4511	9.6873	0.6524	0.6341	0.00E+00	4.99E-05	Both
C	GT27	13.6554	1.4004	0.6687	0.6513	0.00E+00	3.66E-05	Both
C	GT28	177.389	1.5143	0.0265	-0.0247	0.4804	0.5582691	Both
C	GT29	3.7879	-0.3316	0.2449	0.2051	0.0226	0.0413517	Both
C	GT30	120.5539	1.9823	0.0814	0.033	0.2101	0.2891547	Both
C	GT3	3.0716	0.9138	0.6415	0.6226	0.00E+00	6.60E-05	Both
C	GT33	1.6363	-0.2106	0.1713	0.1277	0.0621	0.1046593	Both
C	GT34	0.9693	0.1924	0.1723	0.1287	0.0614	0.1040496	Both
C	GT32	12.037	-0.6614	0.1182	0.0718	0.1269	0.1907763	Both
C	GT31	1.5981	0.6591	0.5082	0.4823	0.0003	0.0009598	Both
C	GT35	354.1091	13.569	0.1077	0.0608	0.1463	0.2147666	Both
C	GT37	0.0077	0.0015	0.0224	-0.029	0.5169	0.5897457	Both
C	GT39	21.0631	0.8819	0.0324	-0.0185	0.4349	0.5167459	Both
C	GT4	1835.3972	-86.8507	0.2872	0.2497	0.0123	0.0258984	Both
C	GT41	429.2439	27.6096	0.2642	0.2255	0.0171	0.0338545	Both
C	GT40	0.0014	0.0006	0.0437	-0.0067	0.3634	0.4492781	Both
C	GT42	0.0596	0.0157	0.1255	0.0795	0.1151	0.1751396	Both
C	GT43	0.0839	0.0476	0.5714	0.5489	1.00E-04	0.0003089	Both
C	GT47	70.9951	9.9711	0.2967	0.2597	0.0107	0.0228829	Both
C	GT45	0.2403	-0.0276	0.193	0.1505	0.0463	0.0818951	Both
C	GT44	0.0196	-0.0064	0.1203	0.074	0.1235	0.1866688	Both
C	GT48	3.6235	1.3662	0.5688	0.5461	1.00E-04	0.0003175	Both
C	GT49	0.2071	0.0882	0.6156	0.5953	0.00E+00	0.0001206	Both
C	GT5	110.3216	-2.2924	0.0827	0.0344	0.2062	0.2858823	Both
C	GT50	0.7517	0.2688	0.5085	0.4827	0.0003	0.0009598	Both
C	GT53	24.0821	-0.081	1.00E-04	-0.0525	0.9659	0.9728197	Both
C	GT52	0	0	#VALUE!	#VALUE!	#VALUE!	NA	Both
C	GT51	587.598	-52.6905	0.6001	0.579	0.00E+00	0.000168	Both
C	GT54	0.0432	0.0339	0.6122	0.5918	0.00E+00	0.0001289	Both
C	GT57	1.512	0.5166	0.4102	0.3792	0.0018	0.0045549	Both
C	GT56	0.434	0.0945	0.6737	0.6565	0.00E+00	3.26E-05	Both
C	GT55	2.7859	0.1988	0.0438	-0.0065	0.3623	0.4492781	Both
C	GT58	0.5931	0.1969	0.4471	0.418	0.0009	0.0025377	Both
C	GT6	0.3486	0.0266	0.0322	-0.0187	0.4365	0.5167459	Both
C	GT59	0.4033	0.1177	0.2959	0.2589	0.0108	0.0229683	Both
C	GT60	2.2724	1.0958	0.8628	0.8555	0.00E+00	3.87E-08	Both
C	GT61	0.4009	0.1232	0.5345	0.51	0.0002	0.0006064	Both

C	GT62	2.2504	0.1857	0.1355	0.09	0.1006	0.1577762	Both
C	GT64	0.8578	-0.0445	0.0489	-0.0012	0.3354	0.4273575	Both
C	GT65	0.0525	0.0264	0.5826	0.5607	1.00E-04	0.0002421	Both
C	GT66	19.0025	-1.1249	0.1513	0.1066	0.0814	0.1309245	Both
C	GT69	0.8788	0.3075	0.3568	0.3229	0.0042	0.0100609	Both
C	GT68	0.033	0.0183	0.461	0.4326	0.0007	0.0019906	Both
C	GT7	5.6797	0.574	0.4946	0.468	0.0004	0.0012086	Both
C	GT72	0.0007	-1.00E-04	0.0038	-0.0487	0.7916	0.82785	Both
C	GT73	0.0676	0.0166	0.0971	0.0495	0.1692	0.2412828	Both
C	GT71	1.4697	0.2263	0.3242	0.2887	0.0071	0.0156673	Both
C	GT70	0.7719	0.0208	0.0267	-0.0245	0.4788	0.5582691	Both
C	GT74	0.8743	-0.0295	0.0129	-0.0391	0.6246	0.6906273	Both
C	GT75	0.9562	0.0342	0.0327	-0.0182	0.433	0.5167459	Both
C	GT76	2.0735	-0.7482	0.6738	0.6566	0.00E+00	3.26E-05	Both
C	GT77	0.8498	-0.2509	0.6544	0.6363	0.00E+00	4.81E-05	Both
C	GT78	0.0021	-0.0009	0.0412	-0.0093	0.3778	0.460327	Both
C	GT8	11.3981	-0.1415	0.004	-0.0484	0.7856	0.8250531	Both
C	GT80	5.8542	0.6339	0.4725	0.4447	0.0006	0.0016794	Both
C	GT82	0.4408	-0.0922	0.3385	0.3037	0.0057	0.012911	Both
C	GT84	302.7752	21.7407	0.1271	0.0811	0.1127	0.1725052	Both
C	GT81	24.2095	-0.364	0.0133	-0.0387	0.6192	0.6876508	Both
C	GT83	173.1688	3.5818	0.0717	0.0229	0.2405	0.3176847	Both
C	GT85	3.3791	-0.0145	0.0001	-0.0525	0.9608	0.9728197	Both
C	GT87	17.8526	2.1435	0.2531	0.2138	0.0201	0.0376624	Both
C	GT88	0.0319	0.0147	0.2787	0.2407	0.0139	0.0290774	Both
C	GT89	5.7995	-0.1434	0.009	-0.0432	0.6824	0.7415173	Both
C	GT90	2.7262	0.8038	0.4711	0.4433	0.0006	0.0017042	Both
C	GT9	169.2922	-8.6502	0.2534	0.2141	0.02	0.0376624	Both
C	GT91	0.0175	0.0029	0.0319	-0.0191	0.4388	0.5171343	Both
C	GT93	0.367	0.0045	0.012	-0.04	0.6362	0.7004249	Both
C	GT94	0.8321	0.1809	0.4792	0.4518	0.0005	0.0015149	Both
C	GT92	0.1294	0.0701	0.7087	0.6933	0.00E+00	1.35E-05	Both
C	GT95	6.8457	-0.4315	0.2498	0.2103	0.0211	0.0388686	Both
C	PL1	17.2792	-1.4008	0.0442	-0.0061	0.3601	0.4492781	Both
C	PL10	4.8889	0.8201	0.1466	0.1017	0.0867	0.136873	Both
C	PL11	3.4173	-0.1441	0.0074	-0.0449	0.7116	0.7632536	Both
C	PL12	23.1583	0.6539	0.0396	-0.0109	0.387	0.4682932	Both
C	PL13	0.0005	0.0007	0.1779	0.1346	0.0569	0.097745	Both
C	PL14	1.0529	-0.1564	0.0899	0.042	0.1867	0.2633117	Both
C	PL15	0.3252	-0.0828	0.1575	0.1131	0.0749	0.1228976	Both
C	PL17	1.5798	-0.3682	0.1549	0.1104	0.0775	0.1255773	Both
C	PL18	0.003	-0.0018	0.1474	0.1025	0.0858	0.1362967	Both
C	PL16	0.5166	0.0102	0.0081	-0.0441	0.6984	0.7523264	Both
C	PL2	0.1859	0.0903	0.6545	0.6364	0.00E+00	4.81E-05	Both
C	PL20	0.0885	-0.0054	0.0055	-0.0468	0.7486	0.7928296	Both
C	PL21	0.5039	0.0607	0.1042	0.0571	0.1535	0.2214431	Both
C	PL22	10.1314	2.0175	0.7011	0.6853	0.00E+00	1.63E-05	Both
C	PL23	0.0169	0.0015	0.0423	-0.0081	0.3709	0.4541153	Both
C	PL3	0.2609	-0.071	0.0475	-0.0026	0.3423	0.4339727	Both
C	PL4	18.5959	7.2587	0.7111	0.6959	0.00E+00	1.32E-05	Both
C	PL5	2.4899	1.8214	0.8032	0.7929	0.00E+00	5.77E-07	Both
C	PL6	1.9888	-0.3968	0.0875	0.0395	0.1928	0.2703933	Both
C	PL7	3.6439	0.331	0.043	-0.0073	0.3669	0.4514294	Both
C	PL8	2.6452	0.4859	0.2498	0.2103	0.0211	0.0388686	Both
C	PL9	31.9165	-0.1798	0.0004	-0.0522	0.9303	0.9569718	Both

N	AA1	166.2439	11.5233	0.3666	0.3332	0.0036	0.0088874	Both
N	AA11	0.4336	0.134	0.2308	0.1903	0.0275	0.0503894	Both
N	AA10	6.525	1.2698	0.4873	0.4603	0.0004	0.0015545	Both
N	AA2	1.5806	0.3022	0.283	0.2452	0.0131	0.027773	Both
N	AA3	414.0022	-20.5496	0.1606	0.1165	0.0718	0.1154704	Both
N	AA4	13.9824	6.1457	0.8291	0.8201	0.00E+00	9.12E-07	Both
N	AA5	47.5523	-11.7522	0.7871	0.7759	0.00E+00	2.08E-06	Both
N	AA8	0.7042	0.1402	0.2221	0.1812	0.031	0.0560346	Both
N	AA7	55.8067	-2.2318	0.0583	0.0087	0.2917	0.3895014	Both
N	AA6	28.9935	-3.6226	0.4781	0.4506	0.0005	0.001732	Both
N	AA9	1.5682	0.3561	0.0967	0.0492	0.17	0.2451686	Both
N	CE1	214.5689	-3.3992	0.0665	0.0173	0.2592	0.3535322	Both
N	CE11	161.0901	-2.5442	0.05	0.00E+00	0.3299	0.4176452	Both
N	CE12	17.1999	3.6517	0.5132	0.4876	0.0003	0.0010161	Both
N	CE13	0.0445	0.016	0.1895	0.1468	0.0486	0.0852518	Both
N	CE14	222.8524	5.938	0.1676	0.1238	0.0654	0.1081697	Both
N	CE15	41.9051	9.1457	0.7489	0.7357	0.00E+00	5.69E-06	Both
N	CE2	2.7115	0.6309	0.2477	0.2081	0.0217	0.0415756	Both
N	CE3	23.2608	-4.0861	0.136	0.0905	0.1	0.1559348	Both
N	CE4	354.0802	-45.8435	0.5246	0.4996	0.0002	0.0008167	Both
N	CE16	1.0609	0.0938	0.0932	0.0455	0.1783	0.2542499	Both
N	CE5	14.6374	-0.4889	0.0068	-0.0455	0.722	0.7913814	Both
N	CE7	11.1596	1.7678	0.4385	0.4089	0.0011	0.0031741	Both
N	CE6	41.6877	9.388	0.7659	0.7536	0.00E+00	3.48E-06	Both
N	CE8	22.9784	2.5865	0.2805	0.2426	0.0135	0.0283383	Both
N	CE9	93.7508	-1.6936	0.1377	0.0923	0.0977	0.15426	Both
N	GH100	0.8338	0.3319	0.321	0.2852	0.0074	0.0167657	Both
N	GH10	54.9015	4.7883	0.43	0.4	0.0012	0.003483	Both
N	GH102	40.7964	-4.0721	0.661	0.6432	0.00E+00	5.69E-05	Both
N	GH101	0.7299	-0.1788	0.2455	0.2058	0.0224	0.0422046	Both
N	GH103	106.2158	-13.1957	0.4377	0.4081	0.0011	0.003182	Both
N	GH104	5.1644	-0.6785	0.3975	0.3658	0.0022	0.0057079	Both
N	GH105	29.0354	9.353	0.7536	0.7407	0.00E+00	5.02E-06	Both
N	GH106	49.5372	13.1552	0.7803	0.7688	0.00E+00	2.36E-06	Both
N	GH107	0.1563	-0.0607	0.1882	0.1455	0.0494	0.0855591	Both
N	GH108	6.355	0.1174	0.0249	-0.0264	0.4941	0.5768252	Both
N	GH109	20.0561	1.0328	0.1674	0.1236	0.0655	0.1081697	Both
N	GH11	14.887	3.2395	0.7217	0.7071	0.00E+00	1.20E-05	Both
N	GH110	1.2431	0.3516	0.5732	0.5508	1.00E-04	0.0003292	Both
N	GH111	0.0414	-0.0068	0.0709	0.022	0.2434	0.3393911	Both
N	GH112	0.0997	0.0684	0.4597	0.4313	0.0007	0.0022916	Both
N	GH113	8.2393	-0.3645	0.0409	-0.0096	0.3794	0.4667656	Both
N	GH114	10.5907	-2.0865	0.3822	0.3497	0.0028	0.0071816	Both
N	GH115	18.1582	7.0748	0.6838	0.6672	0.00E+00	3.08E-05	Both
N	GH116	44.574	7.134	0.4861	0.4591	0.0004	0.0015564	Both
N	GH117	3.4772	0.0187	0.0004	-0.0522	0.9305	0.9572049	Both
N	GH118	0.0029	-0.0004	0.0164	-0.0354	0.58	0.6612482	Both
N	GH119	2.7623	-0.5904	0.2617	0.2229	0.0178	0.03595	Both
N	GH12	8.8559	1.9261	0.4937	0.467	0.0004	0.0014128	Both
N	GH120	1.8739	0.2114	0.0515	0.0016	0.3224	0.4107616	Both
N	GH121	1.5942	-0.4337	0.1208	0.0745	0.1227	0.185488	Both
N	GH123	1.8672	-0.8082	0.652	0.6337	0.00E+00	6.55E-05	Both
N	GH124	0.0511	-0.0279	0.4796	0.4523	0.0005	0.001704	Both
N	GH125	11.4055	2.323	0.5008	0.4745	0.0003	0.0012457	Both
N	GH126	0.0093	0.0025	0.0564	0.0068	0.2998	0.3898486	Both

N	GH127	44.4763	8.1038	0.6849	0.6683	0.00E+00	3.08E-05	Both
N	GH128	6.9808	2.1286	0.7098	0.6946	0.00E+00	1.71E-05	Both
N	GH129	0.3906	0.0408	0.0711	0.0222	0.2427	0.3393911	Both
N	GH13	1501.8611	-5.9757	0.0028	-0.0497	0.8191	0.8712127	Both
N	GH130	86.8053	6.8577	0.242	0.2021	0.0235	0.0440494	Both
N	GH131	0.195	0.0683	0.3059	0.2694	0.0093	0.0204942	Both
N	GH132	0.7589	0.2332	0.2737	0.2355	0.0149	0.0309932	Both
N	GH133	72.2732	-11.2316	0.8277	0.8187	0.00E+00	9.12E-07	Both
N	GH14	0.5412	0.1045	0.4485	0.4195	0.0009	0.0027301	Both
N	GH17	59.077	-5.0723	0.2527	0.2133	0.0202	0.0401754	Both
N	GH15	408.8678	10.1837	0.0675	0.0184	0.2553	0.3502283	Both
N	GH18	245.204	14.6194	0.2588	0.2198	0.0185	0.037199	Both
N	GH19	16.9586	-0.2615	0.0055	-0.0468	0.7491	0.8104616	Both
N	GH2	388.7654	54.8491	0.6588	0.6408	0.00E+00	5.88E-05	Both
N	GH20	100.202	4.93	0.2281	0.1875	0.0285	0.0519234	Both
N	GH23	392.3336	-15.8353	0.2316	0.1912	0.0272	0.0501936	Both
N	GH24	15.9645	-0.0826	0.0023	-0.0502	0.8361	0.8817405	Both
N	GH25	19.8627	-0.5872	0.0195	-0.0321	0.5462	0.6318196	Both
N	GH26	22.0532	1.9615	0.1829	0.1399	0.0531	0.09007	Both
N	GH27	80.433	20.7397	0.8095	0.7994	0.00E+00	1.20E-06	Both
N	GH28	194.68	38.3731	0.7735	0.7615	0.00E+00	2.72E-06	Both
N	GH29	112.4106	14.8503	0.6021	0.5811	0.00E+00	0.0002083	Both
N	GH3	587.4424	82.1782	0.7461	0.7327	0.00E+00	5.94E-06	Both
N	GH30	51.6227	7.7459	0.7053	0.6898	0.00E+00	1.85E-05	Both
N	GH31	139.0432	26.8862	0.7451	0.7317	0.00E+00	5.94E-06	Both
N	GH33	59.3874	-7.1517	0.4345	0.4048	0.0012	0.0032879	Both
N	GH36	91.5089	21.1974	0.8189	0.8094	0.00E+00	1.10E-06	Both
N	GH32	31.3611	-1.5383	0.3095	0.2732	0.0088	0.0195858	Both
N	GH37	39.0498	5.4861	0.4137	0.3829	0.0017	0.0044744	Both
N	GH39	89.459	16.4604	0.8114	0.8015	0.00E+00	1.20E-06	Both
N	GH38	117.6702	29.6802	0.793	0.7821	0.00E+00	1.99E-06	Both
N	GH4	144.275	0.0384	0.00E+00	-0.0526	0.9942	0.9981979	Both
N	GH42	45.2312	5.7056	0.6985	0.6826	0.00E+00	2.23E-05	Both
N	GH43	113.62	20.4947	0.4566	0.428	0.0008	0.0023971	Both
N	GH44	26.6648	4.838	0.5618	0.5388	1.00E-04	0.0004187	Both
N	GH45	0.4552	0.1353	0.4482	0.4191	0.0009	0.0027301	Both
N	GH47	15.1347	0.5775	0.0526	0.0027	0.3174	0.4094331	Both
N	GH46	2.1056	-0.3739	0.0986	0.0512	0.1656	0.2402461	Both
N	GH48	9.684	1.0724	0.3806	0.348	0.0029	0.0072645	Both
N	GH49	0.1506	-0.0126	0.0055	-0.0468	0.7485	0.8104616	Both
N	GH5	170.4089	5.6705	0.0577	0.0082	0.2941	0.3898486	Both
N	GH50	7.8055	-0.3424	0.0296	-0.0214	0.4555	0.5443853	Both
N	GH51	110.7785	22.0373	0.8061	0.7959	0.00E+00	1.22E-06	Both
N	GH54	21.4612	10.6972	0.8366	0.828	0.00E+00	9.12E-07	Both
N	GH53	57.7565	7.5664	0.539	0.5147	0.0002	0.000667	Both
N	GH55	111.9622	28.8158	0.7851	0.7738	0.00E+00	2.08E-06	Both
N	GH56	0.0046	0.0033	0.2708	0.2325	0.0156	0.0320562	Both
N	GH57	153.7528	-6.6307	0.1888	0.1461	0.049	0.0854371	Both
N	GH58	0.039	-0.0062	0.0611	0.0117	0.2801	0.3779248	Both
N	GH59	8.0215	3.5435	0.7777	0.766	0.00E+00	2.44E-06	Both
N	GH6	18.8877	3.4623	0.4236	0.3933	0.0014	0.0038523	Both
N	GH62	3.2816	0.3117	0.0567	0.0071	0.2985	0.3898486	Both
N	GH63	15.2609	2.7616	0.481	0.4537	0.0005	0.0016814	Both
N	GH64	20.0853	3.0964	0.5426	0.5185	0.0001	0.0006284	Both
N	GH65	100.4831	-0.127	0.00E+00	-0.0526	0.9801	0.9879454	Both

N	GH66	1.539	0.1265	0.0277	-0.0235	0.471	0.5575944	Both
N	GH68	0.282	0.0012	0.0003	-0.0523	0.9363	0.9592276	Both
N	GH7	0.7235	0.2112	0.1648	0.1208	0.0679	0.1099402	Both
N	GH70	0.0383	0.0003	0.0001	-0.0525	0.9632	0.9788293	Both
N	GH71	3.9384	1.154	0.4158	0.3851	0.0016	0.0043652	Both
N	GH72	30.0493	0.8476	0.0695	0.0206	0.2481	0.3440716	Both
N	GH73	38.6432	-1.1958	0.099	0.0516	0.1647	0.2402461	Both
N	GH75	2.2066	0.0684	0.0135	-0.0384	0.6157	0.6898673	Both
N	GH74	13.9963	2.2978	0.444	0.4147	0.001	0.0029081	Both
N	GH76	14.814	3.3166	0.5084	0.4825	0.0003	0.0010863	Both
N	GH78	155.8729	17.7587	0.6189	0.5989	0.00E+00	0.000143	Both
N	GH79	28.1345	4.1974	0.4657	0.4376	0.0007	0.0021078	Both
N	GH8	19.2344	0.9443	0.0377	-0.0129	0.3988	0.4832603	Both
N	GH80	0.002	-0.0019	0.0718	0.023	0.2401	0.3393269	Both
N	GH81	5.7722	-0.2321	0.0184	-0.0332	0.5572	0.6386421	Both
N	GH84	4.781	0.2236	0.0717	0.0228	0.2406	0.3393269	Both
N	GH85	0.6123	-0.0231	0.0078	-0.0444	0.7031	0.7740284	Both
N	GH86	1.587	-0.2849	0.3639	0.3304	0.0038	0.0091536	Both
N	GH87	68.8594	9.6574	0.7296	0.7154	0.00E+00	9.53E-06	Both
N	GH88	24.4555	-0.7767	0.1168	0.0703	0.1294	0.1933838	Both
N	GH89	7.7554	2.2812	0.709	0.6936	0.00E+00	1.71E-05	Both
N	GH91	0.0888	-0.0291	0.1649	0.1209	0.0678	0.1099402	Both
N	GH90	0.0034	-0.0011	0.0401	-0.0104	0.3842	0.4704481	Both
N	GH9	54.2204	7.076	0.2929	0.2557	0.0113	0.0244257	Both
N	GH92	99.629	21.0805	0.4858	0.4587	0.0004	0.0015564	Both
N	GH93	21.1746	0.5565	0.0025	-0.05	0.8286	0.8775295	Both
N	GH94	30.4978	7.075	0.5336	0.509	0.0002	0.0007348	Both
N	GH95	51.1365	6.0965	0.3731	0.3401	0.0033	0.0081181	Both
N	GH96	0.4341	-0.1614	0.1812	0.1381	0.0544	0.0915932	Both
N	GH97	31.9027	-0.2744	0.0021	-0.0504	0.8423	0.8822981	Both
N	GH98	0.2928	-0.0879	0.5988	0.5776	0.00E+00	0.0002207	Both
N	GH99	12.1809	-1.4593	0.2517	0.2123	0.0205	0.0401754	Both
N	GT1	152.7224	0.4637	0.0002	-0.0524	0.9464	0.9656832	Both
N	GT10	2.6221	0.4201	0.4369	0.4073	0.0011	0.0031871	Both
N	GT12	0.0479	0.0024	0.0144	-0.0375	0.6041	0.6830624	Both
N	GT14	2.0818	0.1742	0.1148	0.0683	0.1329	0.1962216	Both
N	GT11	5.172	0.5332	0.2695	0.231	0.0159	0.0324204	Both
N	GT13	0.1791	0.0928	0.5802	0.5581	1.00E-04	0.0003085	Both
N	GT15	2.0332	0.6045	0.3619	0.3283	0.0039	0.0093551	Both
N	GT16	0.0469	0.0305	0.6944	0.6783	0.00E+00	2.36E-05	Both
N	GT17	1.0854	0.115	0.2945	0.2574	0.011	0.0240554	Both
N	GT18	0.0144	0.0109	0.6969	0.6809	0.00E+00	2.26E-05	Both
N	GT2	1818.8004	-33.7887	0.1548	0.1104	0.0776	0.1232637	Both
N	GT19	83.4957	0.7105	0.0211	-0.0304	0.5294	0.6152378	Both
N	GT20	149.4495	-2.2063	0.1264	0.0804	0.1137	0.1740539	Both
N	GT21	78.1092	11.0866	0.3663	0.333	0.0036	0.0088874	Both
N	GT22	4.5977	0.6681	0.3343	0.2992	0.006	0.0137963	Both
N	GT23	0.2539	0.1081	0.5889	0.5672	0.00E+00	0.000274	Both
N	GT24	2.8985	0.6511	0.5317	0.5071	0.0002	0.0007473	Both
N	GT25	6.3037	0.9688	0.6667	0.6491	0.00E+00	4.98E-05	Both
N	GT26	137.4511	9.4939	0.6266	0.6069	0.00E+00	0.0001203	Both
N	GT27	13.6554	1.3014	0.5775	0.5553	1.00E-04	0.0003222	Both
N	GT28	177.389	0.5905	0.004	-0.0484	0.7844	0.8445377	Both
N	GT29	3.7879	-0.3365	0.2521	0.2127	0.0204	0.0401754	Both
N	GT30	120.5539	1.1329	0.0266	-0.0247	0.4802	0.5658264	Both

N	GT3	3.0716	0.9062	0.6309	0.6115	0.00E+00	0.0001101	Both
N	GT33	1.6363	-0.1865	0.1345	0.0889	0.102	0.1580818	Both
N	GT34	0.9693	0.2304	0.2471	0.2074	0.0219	0.0416115	Both
N	GT32	12.037	-0.4287	0.0497	-0.0003	0.3314	0.4176452	Both
N	GT31	1.5981	0.673	0.5298	0.505	0.0002	0.0007572	Both
N	GT35	354.1091	8.2328	0.0397	-0.0109	0.3868	0.4712758	Both
N	GT37	0.0077	0.0024	0.057	0.0074	0.2973	0.3898486	Both
N	GT39	21.0631	1.207	0.0607	0.0113	0.2817	0.3780639	Both
N	GT4	1835.3972	-86.1259	0.2824	0.2447	0.0132	0.027773	Both
N	GT41	429.2439	19.5815	0.1329	0.0873	0.1042	0.1604439	Both
N	GT40	0.0014	0.0002	0.0084	-0.0437	0.6919	0.7684368	Both
N	GT42	0.0596	0.0184	0.1713	0.1277	0.0622	0.1040217	Both
N	GT43	0.0839	0.0428	0.4608	0.4324	0.0007	0.0022748	Both
N	GT47	70.9951	9.1187	0.2482	0.2086	0.0215	0.0415756	Both
N	GT45	0.2403	-0.0294	0.2186	0.1775	0.0326	0.0583824	Both
N	GT44	0.0196	-0.0063	0.1169	0.0704	0.1293	0.1933838	Both
N	GT48	3.6235	1.4654	0.6544	0.6362	0.00E+00	6.47E-05	Both
N	GT49	0.2071	0.0908	0.6523	0.634	0.00E+00	6.55E-05	Both
N	GT5	110.3216	-2.9458	0.1366	0.0912	0.0992	0.155554	Both
N	GT50	0.7517	0.2853	0.5732	0.5508	1.00E-04	0.0003292	Both
N	GT53	24.0821	0.2464	0.0009	-0.0517	0.8965	0.9298205	Both
N	GT52	0	0	#VALUE!	#VALUE!	#VALUE!	NA	Both
N	GT51	587.598	-51.9342	0.583	0.561	1.00E-04	0.0002975	Both
N	GT54	0.0432	0.0331	0.5859	0.5641	1.00E-04	0.0002875	Both
N	GT57	1.512	0.5569	0.4767	0.4492	0.0005	0.0017532	Both
N	GT56	0.434	0.0873	0.5749	0.5525	1.00E-04	0.0003292	Both
N	GT55	2.7859	0.2907	0.0938	0.0461	0.177	0.2538546	Both
N	GT58	0.5931	0.2146	0.5312	0.5065	0.0002	0.0007473	Both
N	GT6	0.3486	0.0115	0.006	-0.0463	0.7378	0.80515	Both
N	GT59	0.4033	0.1337	0.3819	0.3493	0.0028	0.0071816	Both
N	GT60	2.2724	1.0466	0.787	0.7758	0.00E+00	2.08E-06	Both
N	GT61	0.4009	0.115	0.4656	0.4375	0.0007	0.0021078	Both
N	GT62	2.2504	0.2292	0.2064	0.1647	0.0385	0.0681059	Both
N	GT64	0.8578	-0.039	0.0375	-0.0132	0.4005	0.4832603	Both
N	GT65	0.0525	0.0251	0.5275	0.5027	0.0002	0.0007807	Both
N	GT66	19.0025	-1.2495	0.1867	0.1439	0.0505	0.0866161	Both
N	GT69	0.8788	0.3385	0.4325	0.4027	0.0012	0.0033692	Both
N	GT68	0.033	0.0168	0.3921	0.3601	0.0024	0.0061857	Both
N	GT7	5.6797	0.4845	0.3524	0.3184	0.0046	0.0107753	Both
N	GT72	0.0007	0.00E+00	0.00E+00	-0.0526	0.999	0.9990099	Both
N	GT73	0.0676	0.0181	0.1151	0.0685	0.1325	0.1962216	Both
N	GT71	1.4697	0.233	0.3438	0.3093	0.0052	0.0121143	Both
N	GT70	0.7719	0.0265	0.0431	-0.0072	0.3663	0.452971	Both
N	GT74	0.8743	-0.0359	0.0191	-0.0325	0.5503	0.6335946	Both
N	GT75	0.9562	0.022	0.0135	-0.0384	0.6153	0.6898673	Both
N	GT76	2.0735	-0.6904	0.5736	0.5512	1.00E-04	0.0003292	Both
N	GT77	0.8498	-0.2367	0.5827	0.5607	1.00E-04	0.0002975	Both
N	GT78	0.0021	-0.0007	0.0281	-0.0231	0.468	0.5567096	Both
N	GT8	11.3981	-0.0751	0.0011	-0.0514	0.8853	0.9220554	Both
N	GT80	5.8542	0.5921	0.4122	0.3813	0.0017	0.0044948	Both
N	GT82	0.4408	-0.0937	0.3501	0.3158	0.0047	0.0110847	Both
N	GT84	302.7752	15.9664	0.0685	0.0195	0.2517	0.347065	Both
N	GT81	24.2095	-0.2848	0.0081	-0.0441	0.6977	0.7714193	Both
N	GT83	173.1688	2.9729	0.0494	-0.0006	0.3328	0.4176452	Both
N	GT85	3.3791	0.0347	0.0007	-0.0518	0.9064	0.9362804	Both

N	GT87	17.8526	2.3008	0.2916	0.2544	0.0115	0.0246763	Both
N	GT88	0.0319	0.0138	0.2482	0.2086	0.0215	0.0415756	Both
N	GT89	5.7995	-0.0933	0.0038	-0.0486	0.7902	0.8445377	Both
N	GT90	2.7262	0.8368	0.5106	0.4849	0.0003	0.0010541	Both
N	GT9	169.2922	-9.5966	0.3118	0.2756	0.0085	0.0190755	Both
N	GT91	0.0175	0.0041	0.0651	0.0159	0.2643	0.3585911	Both
N	GT93	0.367	0.0066	0.0258	-0.0255	0.4868	0.5709836	Both
N	GT94	0.8321	0.1679	0.4128	0.3819	0.0017	0.0044948	Both
N	GT92	0.1294	0.0717	0.7411	0.7275	0.00E+00	6.56E-06	Both
N	GT95	6.8457	-0.504	0.3408	0.3061	0.0055	0.0125886	Both
N	PL1	17.2792	-1.5247	0.0524	0.0025	0.3182	0.4094331	Both
N	PL10	4.8889	0.7468	0.1216	0.0753	0.1214	0.1846359	Both
N	PL11	3.4173	-0.214	0.0162	-0.0356	0.5822	0.6612482	Both
N	PL12	23.1583	0.3609	0.0121	-0.0399	0.6354	0.7088617	Both
N	PL13	0.0005	0.0008	0.2349	0.1947	0.026	0.0482694	Both
N	PL14	1.0529	-0.1087	0.0434	-0.007	0.365	0.452971	Both
N	PL15	0.3252	-0.0852	0.1667	0.1229	0.0661	0.108461	Both
N	PL17	1.5798	-0.3692	0.1557	0.1113	0.0767	0.1226018	Both
N	PL18	0.003	-0.002	0.1863	0.1435	0.0507	0.0866161	Both
N	PL16	0.5166	0.0011	1.00E-04	-0.0525	0.9681	0.979795	Both
N	PL2	0.1859	0.0782	0.4906	0.4638	0.0004	0.0014781	Both
N	PL20	0.0885	-0.0045	0.0038	-0.0486	0.7907	0.8445377	Both
N	PL21	0.5039	0.0449	0.0571	0.0075	0.2968	0.3898486	Both
N	PL22	10.1314	1.9272	0.6397	0.6208	0.00E+00	8.93E-05	Both
N	PL23	0.0169	0.0013	0.0307	-0.0203	0.4474	0.5372683	Both
N	PL3	0.2609	-0.0681	0.0438	-0.0065	0.3627	0.452916	Both
N	PL4	18.5959	6.7172	0.609	0.5884	0.00E+00	0.0001796	Both
N	PL5	2.4899	1.7674	0.7563	0.7435	0.00E+00	4.81E-06	Both
N	PL6	1.9888	-0.4327	0.1041	0.057	0.1537	0.2256004	Both
N	PL7	3.6439	0.3641	0.0521	0.0022	0.3197	0.4094331	Both
N	PL8	2.6452	0.449	0.2132	0.1718	0.0351	0.0624578	Both
N	PL9	31.9165	-0.4051	0.0021	-0.0504	0.8436	0.8822981	Both
CN	AA1	166.2439	11.1243	0.3416	0.307	0.0054	0.013268	Both
CN	AA11	0.4336	0.0606	0.0472	-0.0029	0.344	0.438544	Both
CN	AA10	6.525	1.1925	0.4298	0.3997	0.0013	0.0036621	Both
CN	AA2	1.5806	0.2684	0.2231	0.1823	0.0306	0.0609466	Both
CN	AA3	414.0022	-34.3706	0.4494	0.4204	0.0009	0.002805	Both
CN	AA4	13.9824	5.5819	0.6839	0.6673	0.00E+00	3.28E-05	Both
CN	AA5	47.5523	-11.8594	0.8015	0.7911	0.00E+00	1.18E-06	Both
CN	AA8	0.7042	0.1042	0.1226	0.0765	0.1196	0.1900694	Both
CN	AA7	55.8067	-3.9988	0.1871	0.1443	0.0502	0.0886628	Both
CN	AA6	28.9935	-4.0837	0.6075	0.5869	0.00E+00	0.0001739	Both
CN	AA9	1.5682	0.2574	0.0505	0.0006	0.3273	0.4256256	Both
CN	CE1	214.5689	-2.0596	0.0244	-0.0269	0.4989	0.5797493	Both
CN	CE11	161.0901	1.9039	0.028	-0.0232	0.4685	0.5546958	Both
CN	CE12	17.1999	3.7529	0.542	0.5179	0.0001	0.0006135	Both
CN	CE13	0.0445	0.0206	0.3146	0.2785	0.0082	0.0189723	Both
CN	CE14	222.8524	9.3713	0.4174	0.3867	0.0016	0.0043906	Both
CN	CE15	41.9051	9.8322	0.8656	0.8585	0.00E+00	1.27E-07	Both
CN	CE2	2.7115	0.6228	0.2413	0.2014	0.0237	0.0488207	Both
CN	CE3	23.2608	-4.1173	0.138	0.0927	0.0973	0.1616646	Both
CN	CE4	354.0802	-51.1615	0.6534	0.6351	0.00E+00	6.47E-05	Both
CN	CE16	1.0609	0.0891	0.0842	0.036	0.2019	0.2863319	Both
CN	CE5	14.6374	-1.3425	0.0514	0.0015	0.3231	0.4252927	Both
CN	CE7	11.1596	1.8339	0.4719	0.4441	0.0006	0.0020473	Both

CN	CE6	41.6877	9.1191	0.7226	0.708	0.00E+00	1.12E-05	Both
CN	CE8	22.9784	3.0086	0.3795	0.3468	0.0029	0.0077838	Both
CN	CE9	93.7508	-0.484	0.0112	-0.0408	0.6473	0.7095306	Both
CN	GH100	0.8338	0.3384	0.3337	0.2987	0.0061	0.0148542	Both
CN	GH10	54.9015	5.2116	0.5094	0.4836	0.0003	0.0010787	Both
CN	GH102	40.7964	-3.5934	0.5147	0.4892	0.0003	0.0010014	Both
CN	GH101	0.7299	-0.1642	0.2069	0.1651	0.0383	0.0722758	Both
CN	GH103	106.2158	-15.4408	0.5993	0.5782	0.00E+00	0.000204	Both
CN	GH104	5.1644	-0.5062	0.2213	0.1803	0.0314	0.0620705	Both
CN	GH105	29.0354	9.1732	0.7249	0.7105	0.00E+00	1.08E-05	Both
CN	GH106	49.5372	13.3067	0.7984	0.7878	0.00E+00	1.24E-06	Both
CN	GH107	0.1563	-0.0501	0.1285	0.0827	0.1105	0.1789637	Both
CN	GH108	6.355	0.1323	0.0317	-0.0193	0.4401	0.5393469	Both
CN	GH109	20.0561	1.4464	0.3284	0.293	0.0066	0.0158309	Both
CN	GH11	14.887	3.1011	0.6614	0.6436	0.00E+00	5.62E-05	Both
CN	GH110	1.2431	0.309	0.4427	0.4133	0.001	0.0030894	Both
CN	GH111	0.0414	-0.0072	0.0789	0.0304	0.2175	0.3033363	Both
CN	GH112	0.0997	0.0829	0.6757	0.6586	0.00E+00	3.94E-05	Both
CN	GH113	8.2393	-0.155	0.0074	-0.0448	0.7109	0.7625282	Both
CN	GH114	10.5907	-2.4095	0.5098	0.484	0.0003	0.0010787	Both
CN	GH115	18.1582	7.5374	0.7761	0.7644	0.00E+00	2.12E-06	Both
CN	GH116	44.574	7.7619	0.5755	0.5531	1.00E-04	0.0003245	Both
CN	GH117	3.4772	0.1603	0.0301	-0.021	0.4522	0.5455936	Both
CN	GH118	0.0029	-0.0002	0.0057	-0.0467	0.7459	0.7967042	Both
CN	GH119	2.7623	-0.4011	0.1208	0.0745	0.1227	0.1930328	Both
CN	GH12	8.8559	1.8808	0.4707	0.4428	0.0006	0.0020473	Both
CN	GH120	1.8739	0.2768	0.0883	0.0403	0.1908	0.2736395	Both
CN	GH121	1.5942	-0.3792	0.0923	0.0446	0.1805	0.2631322	Both
CN	GH123	1.8672	-0.7409	0.5479	0.5241	0.0001	0.0005601	Both
CN	GH124	0.0511	-0.0297	0.544	0.52	0.0001	0.0005983	Both
CN	GH125	11.4055	2.3413	0.5087	0.4829	0.0003	0.0010787	Both
CN	GH126	0.0093	0.0025	0.0559	0.0062	0.3021	0.4033516	Both
CN	GH127	44.4763	8.4422	0.7433	0.7298	0.00E+00	6.35E-06	Both
CN	GH128	6.9808	1.5757	0.389	0.3568	0.0025	0.0068728	Both
CN	GH129	0.3906	0.0761	0.2472	0.2076	0.0218	0.0456635	Both
CN	GH13	1501.8611	-15.6368	0.0193	-0.0323	0.548	0.6223571	Both
CN	GH130	86.8053	9.3871	0.4534	0.4246	0.0008	0.0026425	Both
CN	GH131	0.195	0.055	0.1984	0.1562	0.043	0.0794074	Both
CN	GH132	0.7589	0.1134	0.0647	0.0155	0.2658	0.3646017	Both
CN	GH133	72.2732	-9.0289	0.5349	0.5104	0.0002	0.0007024	Both
CN	GH14	0.5412	0.095	0.3703	0.3372	0.0034	0.0087536	Both
CN	GH17	59.077	-6.8809	0.465	0.4368	0.0007	0.002217	Both
CN	GH15	408.8678	8.8581	0.0511	0.0011	0.3246	0.4252927	Both
CN	GH18	245.204	16.0157	0.3106	0.2743	0.0087	0.019797	Both
CN	GH19	16.9586	-0.0721	0.0004	-0.0522	0.9298	0.9372731	Both
CN	GH2	388.7654	61.0816	0.817	0.8074	0.00E+00	8.15E-07	Both
CN	GH20	100.202	6.7089	0.4224	0.392	0.0014	0.0040674	Both
CN	GH23	392.3336	-11.2952	0.1178	0.0714	0.1276	0.1990003	Both
CN	GH24	15.9645	0.6135	0.1276	0.0817	0.1119	0.1800724	Both
CN	GH25	19.8627	-0.4537	0.0116	-0.0404	0.6417	0.7066103	Both
CN	GH26	22.0532	1.5624	0.1161	0.0695	0.1307	0.2025543	Both
CN	GH27	80.433	20.2607	0.7725	0.7605	0.00E+00	2.33E-06	Both
CN	GH28	194.68	40.8016	0.8745	0.8678	0.00E+00	1.27E-07	Both
CN	GH29	112.4106	17.0402	0.7927	0.7818	0.00E+00	1.37E-06	Both
CN	GH3	587.4424	83.9569	0.7787	0.7671	0.00E+00	2.02E-06	Both

CN	GH30	51.6227	6.646	0.5192	0.4939	0.0002	0.0009276	Both
CN	GH31	139.0432	27.7062	0.7912	0.7802	0.00E+00	1.37E-06	Both
CN	GH33	59.3874	-4.8732	0.2018	0.1598	0.0411	0.0769512	Both
CN	GH36	91.5089	21.3608	0.8316	0.8227	0.00E+00	4.40E-07	Both
CN	GH32	31.3611	-1.1867	0.1842	0.1412	0.0522	0.0912918	Both
CN	GH37	39.0498	5.8559	0.4714	0.4436	0.0006	0.0020473	Both
CN	GH39	89.459	16.7618	0.8414	0.833	0.00E+00	3.10E-07	Both
CN	GH38	117.6702	29.8753	0.8035	0.7932	0.00E+00	1.18E-06	Both
CN	GH4	144.275	-3.6783	0.0261	-0.0252	0.4844	0.5655338	Both
CN	GH42	45.2312	5.1397	0.5668	0.544	1.00E-04	0.0003811	Both
CN	GH43	113.62	20.6141	0.462	0.4336	0.0007	0.002315	Both
CN	GH44	26.6648	5.5008	0.7263	0.7119	0.00E+00	1.07E-05	Both
CN	GH45	0.4552	0.1259	0.3881	0.3559	0.0026	0.006901	Both
CN	GH47	15.1347	0.379	0.0227	-0.0288	0.5149	0.5956162	Both
CN	GH46	2.1056	-0.3714	0.0973	0.0498	0.1686	0.2518837	Both
CN	GH48	9.684	0.9428	0.2942	0.257	0.0111	0.0246152	Both
CN	GH49	0.1506	-0.0331	0.038	-0.0126	0.3968	0.4942321	Both
CN	GH5	170.4089	5.3794	0.052	0.0021	0.3203	0.4252927	Both
CN	GH50	7.8055	-0.4447	0.05	0.00E+00	0.3298	0.4266649	Both
CN	GH51	110.7785	22.653	0.8517	0.8439	0.00E+00	2.16E-07	Both
CN	GH54	21.4612	9.4537	0.6534	0.6352	0.00E+00	6.47E-05	Both
CN	GH53	57.7565	8.2323	0.638	0.6189	0.00E+00	8.89E-05	Both
CN	GH55	111.9622	26.9157	0.685	0.6684	0.00E+00	3.28E-05	Both
CN	GH56	0.0046	0.0036	0.3182	0.2823	0.0077	0.0181334	Both
CN	GH57	153.7528	-1.7893	0.0138	-0.0382	0.6127	0.6804783	Both
CN	GH58	0.039	-0.0043	0.0285	-0.0226	0.4646	0.5526216	Both
CN	GH59	8.0215	2.9244	0.5297	0.5049	0.0002	0.0007715	Both
CN	GH6	18.8877	3.0008	0.3182	0.2824	0.0077	0.0181334	Both
CN	GH62	3.2816	0.3705	0.0801	0.0317	0.2138	0.2997575	Both
CN	GH63	15.2609	2.5522	0.4108	0.3798	0.0017	0.0048591	Both
CN	GH64	20.0853	2.7333	0.4228	0.3924	0.0014	0.0040674	Both
CN	GH65	100.4831	-0.5968	0.0007	-0.0518	0.9066	0.9212439	Both
CN	GH66	1.539	0.097	0.0163	-0.0355	0.5815	0.651607	Both
CN	GH68	0.282	-0.0032	0.0024	-0.0501	0.834	0.8614133	Both
CN	GH7	0.7235	0.1532	0.0867	0.0386	0.1952	0.2783599	Both
CN	GH70	0.0383	-0.0004	0.0002	-0.0525	0.9576	0.9575862	Both
CN	GH71	3.9384	0.785	0.1924	0.1499	0.0467	0.0837016	Both
CN	GH72	30.0493	0.7254	0.0509	0.001	0.3253	0.4252927	Both
CN	GH73	38.6432	-0.8104	0.0455	-0.0048	0.3533	0.4473837	Both
CN	GH75	2.2066	0.0534	0.0082	-0.044	0.6957	0.7592504	Both
CN	GH74	13.9963	1.9824	0.3305	0.2953	0.0064	0.0154619	Both
CN	GH76	14.814	2.5268	0.2951	0.258	0.0109	0.0244964	Both
CN	GH78	155.8729	17.5236	0.6027	0.5818	0.00E+00	0.0001918	Both
CN	GH79	28.1345	3.9038	0.4028	0.3714	0.002	0.0055072	Both
CN	GH8	19.2344	1.6231	0.1115	0.0647	0.1391	0.2141789	Both
CN	GH80	0.002	-0.0004	0.0033	-0.0492	0.8059	0.8463186	Both
CN	GH81	5.7722	-0.2507	0.0215	-0.03	0.5258	0.6054063	Both
CN	GH84	4.781	0.3852	0.2127	0.1713	0.0353	0.0682059	Both
CN	GH85	0.6123	0.0129	0.0024	-0.0501	0.832	0.8614133	Both
CN	GH86	1.587	-0.259	0.3006	0.2638	0.0101	0.0227593	Both
CN	GH87	68.8594	8.864	0.6147	0.5944	0.00E+00	0.0001486	Both
CN	GH88	24.4555	-0.3923	0.0298	-0.0213	0.4543	0.5455936	Both
CN	GH89	7.7554	2.0607	0.5785	0.5563	1.00E-04	0.0003086	Both
CN	GH91	0.0888	-0.0305	0.1813	0.1382	0.0543	0.0939943	Both
CN	GH90	0.0034	-0.0012	0.0452	-0.005	0.3547	0.4473837	Both

CN	GH9	54.2204	7.9013	0.3652	0.3318	0.0037	0.0094126	Both
CN	GH92	99.629	20.0527	0.4396	0.4101	0.0011	0.0032267	Both
CN	GH93	21.1746	-0.2142	0.0004	-0.0522	0.9336	0.9373429	Both
CN	GH94	30.4978	7.8025	0.649	0.6305	0.00E+00	7.12E-05	Both
CN	GH95	51.1365	6.669	0.4465	0.4174	0.0009	0.0029182	Both
CN	GH96	0.4341	-0.115	0.092	0.0442	0.1814	0.2631322	Both
CN	GH97	31.9027	0.3758	0.004	-0.0484	0.7853	0.8316367	Both
CN	GH98	0.2928	-0.0912	0.6443	0.6256	0.00E+00	7.68E-05	Both
CN	GH99	12.1809	-1.2475	0.184	0.141	0.0524	0.0912918	Both
CN	GT1	152.7224	-5.915	0.0397	-0.0109	0.3868	0.4854074	Both
CN	GT10	2.6221	0.5175	0.6632	0.6455	0.00E+00	5.50E-05	Both
CN	GT12	0.0479	0.0045	0.0489	-0.0012	0.3356	0.4319151	Both
CN	GT14	2.0818	0.2228	0.1878	0.1451	0.0497	0.0884547	Both
CN	GT11	5.172	0.7887	0.5897	0.5681	0.00E+00	0.0002518	Both
CN	GT13	0.1791	0.0963	0.6253	0.6055	0.00E+00	0.0001158	Both
CN	GT15	2.0332	0.2905	0.0836	0.0353	0.2038	0.2873536	Both
CN	GT16	0.0469	0.0291	0.6306	0.6112	0.00E+00	0.000103	Both
CN	GT17	1.0854	0.1303	0.3782	0.3455	0.003	0.0078613	Both
CN	GT18	0.0144	0.0088	0.4564	0.4278	0.0008	0.0025308	Both
CN	GT2	1818.8004	-16.6663	0.0377	-0.013	0.3992	0.4942321	Both
CN	GT19	83.4957	2.4987	0.2614	0.2226	0.0178	0.038261	Both
CN	GT20	149.4495	-2.8812	0.2156	0.1743	0.034	0.0661258	Both
CN	GT21	78.1092	12.1149	0.4374	0.4078	0.0011	0.003272	Both
CN	GT22	4.5977	0.3829	0.1098	0.0629	0.1423	0.2178173	Both
CN	GT23	0.2539	0.1233	0.7665	0.7542	0.00E+00	2.83E-06	Both
CN	GT24	2.8985	0.5473	0.3757	0.3428	0.0031	0.0081083	Both
CN	GT25	6.3037	1.0347	0.7604	0.7478	0.00E+00	3.44E-06	Both
CN	GT26	137.4511	9.1416	0.5809	0.5589	1.00E-04	0.0002974	Both
CN	GT27	13.6554	1.4267	0.6941	0.678	0.00E+00	2.77E-05	Both
CN	GT28	177.389	3.2549	0.1226	0.0765	0.1196	0.1900694	Both
CN	GT29	3.7879	-0.3268	0.2378	0.1977	0.0249	0.0504786	Both
CN	GT30	120.5539	3.3904	0.238	0.1979	0.0249	0.0504786	Both
CN	GT3	3.0716	0.8628	0.5719	0.5494	1.00E-04	0.0003456	Both
CN	GT33	1.6363	-0.195	0.147	0.1021	0.0863	0.1453342	Both
CN	GT34	0.9693	0.1097	0.0561	0.0064	0.3014	0.4033516	Both
CN	GT32	12.037	-0.898	0.218	0.1768	0.0329	0.0644477	Both
CN	GT31	1.5981	0.5705	0.3808	0.3482	0.0029	0.0076992	Both
CN	GT35	354.1091	18.915	0.2094	0.1677	0.037	0.0703841	Both
CN	GT37	0.0077	-0.0004	0.0015	-0.0511	0.8692	0.8868746	Both
CN	GT39	21.0631	0.3143	0.0041	-0.0483	0.7823	0.8316367	Both
CN	GT4	1835.3972	-72.5986	0.2007	0.1586	0.0417	0.0775324	Both
CN	GT41	429.2439	36.6286	0.4651	0.4369	0.0007	0.002217	Both
CN	GT40	0.0014	0.0011	0.1532	0.1086	0.0793	0.134533	Both
CN	GT42	0.0596	0.0114	0.0657	0.0165	0.262	0.3631379	Both
CN	GT43	0.0839	0.0506	0.6443	0.6256	0.00E+00	7.68E-05	Both
CN	GT47	70.9951	9.8426	0.2891	0.2517	0.0119	0.0262741	Both
CN	GT45	0.2403	-0.0196	0.0974	0.0499	0.1685	0.2518837	Both
CN	GT44	0.0196	-0.0074	0.16	0.1158	0.0724	0.1236381	Both
CN	GT48	3.6235	1.0727	0.3506	0.3165	0.0047	0.0117506	Both
CN	GT49	0.2071	0.0741	0.4343	0.4045	0.0012	0.003418	Both
CN	GT5	110.3216	-0.3988	0.0025	-0.05	0.8295	0.8614133	Both
CN	GT50	0.7517	0.2105	0.3119	0.2756	0.0085	0.0195944	Both
CN	GT53	24.0821	-1.0633	0.017	-0.0347	0.573	0.6478303	Both
CN	GT52	0	0	#VALUE!	#VALUE!	#VALUE!	NA	Both
CN	GT51	587.598	-45.0008	0.4377	0.4081	0.0011	0.003272	Both

CN	GT54	0.0432	0.0305	0.4974	0.471	0.0004	0.0013322	Both
CN	GT57	1.512	0.398	0.2436	0.2037	0.023	0.0476968	Both
CN	GT56	0.434	0.0988	0.7365	0.7226	0.00E+00	7.78E-06	Both
CN	GT55	2.7859	0.0381	0.0016	-0.0509	0.8629	0.8840542	Both
CN	GT58	0.5931	0.1472	0.2497	0.2102	0.0211	0.0444464	Both
CN	GT6	0.3486	0.0561	0.1433	0.0983	0.0906	0.1515339	Both
CN	GT59	0.4033	0.0787	0.1323	0.0866	0.105	0.1734525	Both
CN	GT60	2.2724	1.0568	0.8025	0.7921	0.00E+00	1.18E-06	Both
CN	GT61	0.4009	0.1164	0.477	0.4494	0.0005	0.0019231	Both
CN	GT62	2.2504	0.1096	0.0472	-0.003	0.3442	0.438544	Both
CN	GT64	0.8578	-0.0344	0.0292	-0.0219	0.459	0.5485923	Both
CN	GT65	0.0525	0.026	0.5646	0.5417	1.00E-04	0.0003932	Both
CN	GT66	19.0025	-0.7394	0.0654	0.0162	0.2633	0.3631379	Both
CN	GT69	0.8788	0.2365	0.2112	0.1696	0.0361	0.0691894	Both
CN	GT68	0.033	0.0188	0.488	0.461	0.0004	0.0015798	Both
CN	GT7	5.6797	0.6482	0.6308	0.6114	0.00E+00	0.000103	Both
CN	GT72	0.0007	-0.0002	0.0116	-0.0404	0.6419	0.7066103	Both
CN	GT73	0.0676	0.0162	0.0921	0.0443	0.1812	0.2631322	Both
CN	GT71	1.4697	0.1915	0.2321	0.1917	0.027	0.0541991	Both
CN	GT70	0.7719	0.0183	0.0206	-0.031	0.5349	0.6130261	Both
CN	GT74	0.8743	-0.0228	0.0077	-0.0445	0.705	0.7625282	Both
CN	GT75	0.9562	0.057	0.0907	0.0428	0.1846	0.2663526	Both
CN	GT76	2.0735	-0.8082	0.786	0.7747	0.00E+00	1.57E-06	Both
CN	GT77	0.8498	-0.2514	0.6571	0.639	0.00E+00	6.17E-05	Both
CN	GT78	0.0021	-0.0008	0.0376	-0.0131	0.3997	0.4942321	Both
CN	GT8	11.3981	-0.1958	0.0076	-0.0446	0.7064	0.7625282	Both
CN	GT80	5.8542	0.705	0.5844	0.5626	1.00E-04	0.0002794	Both
CN	GT82	0.4408	-0.0702	0.1962	0.1539	0.0443	0.0800236	Both
CN	GT84	302.7752	31.9704	0.2748	0.2366	0.0147	0.0318436	Both
CN	GT81	24.2095	-0.4446	0.0198	-0.0318	0.5431	0.6196486	Both
CN	GT83	173.1688	4.3518	0.1059	0.0588	0.15	0.2282265	Both
CN	GT85	3.3791	-0.1636	0.0165	-0.0352	0.5785	0.6511124	Both
CN	GT87	17.8526	1.5366	0.1301	0.0843	0.1083	0.1775915	Both
CN	GT88	0.0319	0.0141	0.2596	0.2206	0.0183	0.038947	Both
CN	GT89	5.7995	-0.2476	0.0268	-0.0244	0.4779	0.5604959	Both
CN	GT90	2.7262	0.6896	0.3468	0.3124	0.005	0.0123678	Both
CN	GT9	169.2922	-5.3836	0.0981	0.0507	0.1667	0.2518837	Both
CN	GT91	0.0175	0.0007	0.0017	-0.0508	0.8582	0.8828363	Both
CN	GT93	0.367	0.0035	0.0075	-0.0447	0.7081	0.7625282	Both
CN	GT94	0.8321	0.1799	0.4742	0.4465	0.0006	0.0019975	Both
CN	GT92	0.1294	0.0604	0.5265	0.5016	0.0002	0.0008105	Both
CN	GT95	6.8457	-0.2998	0.1206	0.0743	0.123	0.1930328	Both
CN	PL1	17.2792	-0.8387	0.0159	-0.0359	0.5865	0.6542973	Both
CN	PL10	4.8889	0.9521	0.1976	0.1554	0.0435	0.0796905	Both
CN	PL11	3.4173	0.0366	0.0005	-0.0521	0.9254	0.9365636	Both
CN	PL12	23.1583	1.1803	0.1291	0.0833	0.1097	0.1787518	Both
CN	PL13	0.0005	0.0005	0.0943	0.0466	0.1757	0.2594169	Both
CN	PL14	1.0529	-0.2122	0.1653	0.1214	0.0674	0.1158647	Both
CN	PL15	0.3252	-0.052	0.0622	0.0128	0.2757	0.376132	Both
CN	PL17	1.5798	-0.2897	0.0959	0.0483	0.172	0.2554486	Both
CN	PL18	0.003	-0.0011	0.0605	0.0111	0.2824	0.3811256	Both
CN	PL16	0.5166	0.0201	0.0312	-0.0198	0.4439	0.5408263	Both
CN	PL2	0.1859	0.0993	0.7906	0.7796	0.00E+00	1.37E-06	Both
CN	PL20	0.0885	-0.004	0.003	-0.0495	0.8142	0.851518	Both
CN	PL21	0.5039	0.0833	0.1963	0.154	0.0443	0.0800236	Both

CN	PL22	10.1314	2.0014	0.69	0.6737	0.00E+00	3.03E-05	Both
CN	PL23	0.0169	0.0018	0.0613	0.0119	0.2792	0.3788716	Both
CN	PL3	0.2609	-0.0535	0.027	-0.0242	0.4764	0.5604959	Both
CN	PL4	18.5959	7.0909	0.6786	0.6617	0.00E+00	3.72E-05	Both
CN	PL5	2.4899	1.6806	0.6839	0.6672	0.00E+00	3.28E-05	Both
CN	PL6	1.9888	-0.2349	0.0307	-0.0203	0.4477	0.542815	Both
CN	PL7	3.6439	0.2838	0.0316	-0.0193	0.4405	0.5393469	Both
CN	PL8	2.6452	0.5177	0.2835	0.2458	0.013	0.0282742	Both
CN	PL9	31.9165	0.5313	0.0036	-0.0488	0.7958	0.8392877	Both
Latitude	AA1	166.2439	11.7377	0.3804	0.3477	0.0029	0.0196983	Both
Latitude	AA11	0.4336	-0.0084	0.0009	-0.0517	0.8972	0.9336386	Both
Latitude	AA10	6.525	-0.1015	0.0031	-0.0494	0.8102	0.8841401	Both
Latitude	AA2	1.5806	0.0696	0.015	-0.0368	0.5968	0.7236283	Both
Latitude	AA3	414.0022	-26.5331	0.2678	0.2293	0.0163	0.0669677	Both
Latitude	AA4	13.9824	2.7424	0.1651	0.1211	0.0676	0.1759003	Both
Latitude	AA5	47.5523	-5.3486	0.163	0.119	0.0695	0.1761962	Both
Latitude	AA8	0.7042	0.0083	0.0008	-0.0518	0.9039	0.9336386	Both
Latitude	AA7	55.8067	-6.8309	0.5461	0.5222	0.0001	0.003064	Both
Latitude	AA6	28.9935	-3.7983	0.5256	0.5006	0.0002	0.0033608	Both
Latitude	AA9	1.5682	-0.049	0.0018	-0.0507	0.8539	0.897456	Both
Latitude	CE1	214.5689	-10.4532	0.6286	0.6091	0.00E+00	0.0011402	Both
Latitude	CE11	161.0901	-0.0787	0.00E+00	-0.0526	0.9763	0.9762783	Both
Latitude	CE12	17.1999	0.2944	0.0033	-0.0491	0.8036	0.8841401	Both
Latitude	CE13	0.0445	-0.0048	0.0171	-0.0347	0.5726	0.6977313	Both
Latitude	CE14	222.8524	-2.2554	0.0242	-0.0272	0.5009	0.6415222	Both
Latitude	CE15	41.9051	1.706	0.0261	-0.0252	0.4845	0.6320614	Both
Latitude	CE2	2.7115	-0.1984	0.0245	-0.0269	0.4982	0.6412474	Both
Latitude	CE3	23.2608	-7.0744	0.4075	0.3763	0.0018	0.0152717	Both
Latitude	CE4	354.0802	-24.0838	0.1448	0.0998	0.0888	0.2108876	Both
Latitude	CE16	1.0609	0.0319	0.0108	-0.0413	0.6539	0.7669736	Both
Latitude	CE5	14.6374	-3.569	0.3632	0.3296	0.0038	0.0240769	Both
Latitude	CE7	11.1596	-0.1198	0.002	-0.0505	0.8469	0.897456	Both
Latitude	CE6	41.6877	3.0377	0.0802	0.0318	0.2136	0.3884297	Both
Latitude	CE8	22.9784	-1.5029	0.0947	0.047	0.1748	0.3408952	Both
Latitude	CE9	93.7508	-2.429	0.2832	0.2455	0.013	0.0583363	Both
Latitude	GH100	0.8338	0.1589	0.0736	0.0249	0.2342	0.4139254	Both
Latitude	GH10	54.9015	-1.5621	0.0458	-0.0045	0.3518	0.5164786	Both
Latitude	GH102	40.7964	-2.6786	0.286	0.2484	0.0125	0.0570271	Both
Latitude	GH101	0.7299	-0.2304	0.4077	0.3765	0.0018	0.0152717	Both
Latitude	GH103	106.2158	-3.1866	0.0255	-0.0258	0.4891	0.6327996	Both
Latitude	GH104	5.1644	-0.021	0.0004	-0.0522	0.9331	0.9452916	Both
Latitude	GH105	29.0354	0.845	0.0062	-0.0462	0.7354	0.8428695	Both
Latitude	GH106	49.5372	2.1449	0.0207	-0.0308	0.5334	0.6691233	Both
Latitude	GH107	0.1563	-0.0899	0.4127	0.3818	0.0017	0.0151221	Both
Latitude	GH108	6.355	-0.2633	0.1254	0.0794	0.1153	0.2560406	Both
Latitude	GH109	20.0561	-1.0746	0.1812	0.1381	0.0543	0.1604546	Both
Latitude	GH11	14.887	0.4049	0.0113	-0.0408	0.6469	0.7627654	Both
Latitude	GH110	1.2431	0.0618	0.0177	-0.034	0.5652	0.6953977	Both
Latitude	GH111	0.0414	-0.0143	0.3107	0.2744	0.0087	0.0443408	Both
Latitude	GH112	0.0997	0.0148	0.0215	-0.03	0.5255	0.6628098	Both
Latitude	GH113	8.2393	-0.4764	0.0698	0.0209	0.2471	0.4306219	Both
Latitude	GH114	10.5907	-2.3778	0.4964	0.4699	0.0004	0.0043339	Both
Latitude	GH115	18.1582	1.5056	0.031	-0.02	0.4454	0.5978909	Both
Latitude	GH116	44.574	5.5923	0.2987	0.2618	0.0104	0.0509702	Both
Latitude	GH117	3.4772	-0.6532	0.4993	0.4729	0.0003	0.0043026	Both

Latitude	GH118	0.0029	-0.001	0.1037	0.0565	0.1546	0.3104595	Both
Latitude	GH119	2.7623	-0.5206	0.2035	0.1616	0.0401	0.1274951	Both
Latitude	GH12	8.8559	-0.4973	0.0329	-0.018	0.4313	0.5851691	Both
Latitude	GH120	1.8739	-0.485	0.2711	0.2328	0.0155	0.0649061	Both
Latitude	GH121	1.5942	-0.8556	0.47	0.4421	0.0006	0.006582	Both
Latitude	GH123	1.8672	-0.5044	0.2539	0.2147	0.0199	0.0769708	Both
Latitude	GH124	0.0511	-0.0016	0.0015	-0.051	0.8655	0.9051896	Both
Latitude	GH125	11.4055	0.6379	0.0378	-0.0129	0.3987	0.5530028	Both
Latitude	GH126	0.0093	-0.0034	0.1071	0.0601	0.1476	0.2986996	Both
Latitude	GH127	44.4763	0.2282	0.0005	-0.0521	0.9201	0.9426513	Both
Latitude	GH128	6.9808	0.0543	0.0005	-0.0521	0.9264	0.9452087	Both
Latitude	GH129	0.3906	-0.0342	0.05	0.00E+00	0.3299	0.4957668	Both
Latitude	GH13	1501.8611	-86.3561	0.5891	0.5675	0.00E+00	0.0018725	Both
Latitude	GH130	86.8053	10.2203	0.5374	0.5131	0.0002	0.003064	Both
Latitude	GH131	0.195	0.0269	0.0473	-0.0029	0.3438	0.5109851	Both
Latitude	GH132	0.7589	0.0286	0.0041	-0.0483	0.7825	0.8690573	Both
Latitude	GH133	72.2732	-4.1709	0.1141	0.0675	0.1342	0.2806187	Both
Latitude	GH14	0.5412	0.0132	0.0072	-0.045	0.7145	0.8240905	Both
Latitude	GH17	59.077	-1.1808	0.0137	-0.0382	0.6134	0.7402633	Both
Latitude	GH15	408.8678	-16.3075	0.1731	0.1296	0.0606	0.1672815	Both
Latitude	GH18	245.204	-10.83	0.142	0.0969	0.0922	0.2142674	Both
Latitude	GH19	16.9586	-2.433	0.4772	0.4497	0.0005	0.0060017	Both
Latitude	GH2	388.7654	6.6216	0.0096	-0.0425	0.6726	0.7852491	Both
Latitude	GH20	100.202	-2.3838	0.0533	0.0035	0.3138	0.4774038	Both
Latitude	GH23	392.3336	-19.8407	0.3636	0.3301	0.0038	0.0240769	Both
Latitude	GH24	15.9645	-0.6592	0.1473	0.1025	0.0858	0.2071226	Both
Latitude	GH25	19.8627	-3.3475	0.6332	0.6139	0.00E+00	0.0011402	Both
Latitude	GH26	22.0532	-1.6966	0.1368	0.0914	0.0988	0.2254956	Both
Latitude	GH27	80.433	3.7628	0.0266	-0.0246	0.4796	0.6302245	Both
Latitude	GH28	194.68	10.3195	0.0559	0.0062	0.302	0.4737281	Both
Latitude	GH29	112.4106	4.546	0.0564	0.0068	0.2998	0.4737281	Both
Latitude	GH3	587.4424	10.9435	0.0132	-0.0387	0.6196	0.7440538	Both
Latitude	GH30	51.6227	0.0751	1.00E-04	-0.0526	0.9721	0.9759624	Both
Latitude	GH31	139.0432	8.3639	0.0721	0.0233	0.2392	0.4198831	Both
Latitude	GH33	59.3874	-5.209	0.2305	0.19	0.0276	0.096102	Both
Latitude	GH36	91.5089	6.0849	0.0675	0.0184	0.2555	0.4361872	Both
Latitude	GH32	31.3611	0.2932	0.0112	-0.0408	0.6473	0.7627654	Both
Latitude	GH37	39.0498	4.2675	0.2503	0.2109	0.0209	0.0771003	Both
Latitude	GH39	89.459	8.7568	0.2296	0.1891	0.0279	0.096102	Both
Latitude	GH38	117.6702	10.2464	0.0945	0.0469	0.1752	0.3408952	Both
Latitude	GH4	144.275	-13.0311	0.3272	0.2918	0.0067	0.038456	Both
Latitude	GH42	45.2312	1.4472	0.0449	-0.0053	0.3563	0.5191596	Both
Latitude	GH43	113.62	-4.8795	0.0259	-0.0254	0.486	0.6320614	Both
Latitude	GH44	26.6648	2.918	0.2044	0.1625	0.0396	0.1274951	Both
Latitude	GH45	0.4552	0.0284	0.0197	-0.0319	0.544	0.6759663	Both
Latitude	GH47	15.1347	-1.1108	0.1946	0.1522	0.0453	0.1387741	Both
Latitude	GH46	2.1056	-0.9309	0.6114	0.5909	0.00E+00	0.0014217	Both
Latitude	GH48	9.684	-0.5181	0.0888	0.0409	0.1895	0.3522907	Both
Latitude	GH49	0.1506	-0.0573	0.1144	0.0678	0.1337	0.2806187	Both
Latitude	GH5	170.4089	-12.2898	0.2713	0.2329	0.0155	0.0649061	Both
Latitude	GH50	7.8055	-0.8275	0.1732	0.1297	0.0606	0.1672815	Both
Latitude	GH51	110.7785	6.4093	0.0682	0.0191	0.2529	0.4348016	Both
Latitude	GH54	21.4612	2.3946	0.0419	-0.0085	0.3733	0.5293493	Both
Latitude	GH53	57.7565	5.9037	0.3281	0.2928	0.0066	0.038456	Both
Latitude	GH55	111.9622	9.9488	0.0936	0.0459	0.1774	0.3425912	Both

Latitude	GH56	0.0046	0.0019	0.0921	0.0443	0.1812	0.3446104	Both
Latitude	GH57	153.7528	3.5808	0.0551	0.0053	0.3059	0.47498	Both
Latitude	GH58	0.039	-0.0103	0.1683	0.1245	0.0648	0.172803	Both
Latitude	GH59	8.0215	0.3706	0.0085	-0.0437	0.6909	0.8028731	Both
Latitude	GH6	18.8877	-1.1241	0.0447	-0.0056	0.3578	0.5191596	Both
Latitude	GH62	3.2816	-0.6925	0.28	0.2421	0.0136	0.0590555	Both
Latitude	GH63	15.2609	-0.6714	0.0284	-0.0227	0.465	0.6143057	Both
Latitude	GH64	20.0853	-0.4465	0.0113	-0.0408	0.6468	0.7627654	Both
Latitude	GH65	100.4831	-16.2701	0.553	0.5295	0.0001	0.003064	Both
Latitude	GH66	1.539	-0.1344	0.0313	-0.0197	0.4433	0.5978909	Both
Latitude	GH68	0.282	0.0179	0.0764	0.0278	0.2252	0.400866	Both
Latitude	GH7	0.7235	0.0273	0.0027	-0.0497	0.8215	0.8887246	Both
Latitude	GH70	0.0383	-0.0062	0.0442	-0.0061	0.3605	0.5200083	Both
Latitude	GH71	3.9384	0.3828	0.0457	-0.0045	0.3519	0.5164786	Both
Latitude	GH72	30.0493	-0.9368	0.0849	0.0368	0.1999	0.3662968	Both
Latitude	GH73	38.6432	-2.816	0.5491	0.5254	0.0001	0.003064	Both
Latitude	GH75	2.2066	-0.3735	0.4027	0.3712	0.002	0.0153083	Both
Latitude	GH74	13.9963	0.0434	0.0002	-0.0525	0.9568	0.9645027	Both
Latitude	GH76	14.814	-0.6922	0.0221	-0.0293	0.5197	0.6588062	Both
Latitude	GH78	155.8729	-0.6343	0.0008	-0.0518	0.9038	0.9336386	Both
Latitude	GH79	28.1345	3.493	0.3225	0.2869	0.0072	0.0395082	Both
Latitude	GH8	19.2344	-2.9507	0.3684	0.3352	0.0035	0.023299	Both
Latitude	GH80	0.002	-0.0041	0.3538	0.3198	0.0045	0.0272589	Both
Latitude	GH81	5.7722	-0.6919	0.1639	0.1199	0.0687	0.1759003	Both
Latitude	GH84	4.781	-0.1997	0.0572	0.0075	0.2966	0.4737281	Both
Latitude	GH85	0.6123	-0.1851	0.5002	0.4739	0.0003	0.0043026	Both
Latitude	GH86	1.587	-0.2332	0.2439	0.2041	0.0229	0.0820761	Both
Latitude	GH87	68.8594	3.9325	0.121	0.0747	0.1223	0.2647213	Both
Latitude	GH88	24.4555	-0.7939	0.122	0.0758	0.1206	0.2632866	Both
Latitude	GH89	7.7554	0.1953	0.0052	-0.0472	0.7561	0.8570591	Both
Latitude	GH91	0.0888	-0.0447	0.3901	0.358	0.0025	0.0174351	Both
Latitude	GH90	0.0034	-0.0013	0.0537	0.0039	0.3122	0.4774038	Both
Latitude	GH9	54.2204	-5.3685	0.1686	0.1248	0.0645	0.172803	Both
Latitude	GH92	99.629	-5.8116	0.0369	-0.0138	0.404	0.5572177	Both
Latitude	GH93	21.1746	-4.1109	0.138	0.0927	0.0973	0.22396	Both
Latitude	GH94	30.4978	2.2981	0.0563	0.0066	0.3004	0.4737281	Both
Latitude	GH95	51.1365	-1.7169	0.0296	-0.0215	0.4559	0.6086332	Both
Latitude	GH96	0.4341	-0.2702	0.5079	0.482	0.0003	0.0042591	Both
Latitude	GH97	31.9027	-4.8446	0.6654	0.6478	0.00E+00	0.0008264	Both
Latitude	GH98	0.2928	-0.0291	0.0655	0.0163	0.2629	0.4422512	Both
Latitude	GH99	12.1809	-2.1241	0.5333	0.5087	0.0002	0.003064	Both
Latitude	GT1	152.7224	-19.4042	0.4268	0.3967	0.0013	0.0127501	Both
Latitude	GT10	2.6221	0.3557	0.3133	0.2771	0.0083	0.0435493	Both
Latitude	GT12	0.0479	-0.0005	0.0006	-0.052	0.9132	0.9393542	Both
Latitude	GT14	2.0818	-0.2443	0.2259	0.1851	0.0295	0.0999141	Both
Latitude	GT11	5.172	0.5142	0.2507	0.2112	0.0208	0.0771003	Both
Latitude	GT13	0.1791	0.0237	0.0377	-0.0129	0.3988	0.5530028	Both
Latitude	GT15	2.0332	0.2269	0.051	0.001	0.325	0.4913956	Both
Latitude	GT16	0.0469	0.0171	0.2189	0.1778	0.0325	0.1071965	Both
Latitude	GT17	1.0854	0.0815	0.1478	0.1029	0.0853	0.2071226	Both
Latitude	GT18	0.0144	0.0043	0.1082	0.0612	0.1455	0.2968664	Both
Latitude	GT2	1818.8004	-18.6589	0.0472	-0.0029	0.344	0.5109851	Both
Latitude	GT19	83.4957	1.6578	0.1151	0.0685	0.1325	0.2806187	Both
Latitude	GT20	149.4495	-1.279	0.0425	-0.0079	0.3701	0.5293493	Both
Latitude	GT21	78.1092	10.7035	0.3414	0.3068	0.0054	0.0323205	Both

Latitude	GT22	4.5977	-0.0493	0.0018	-0.0507	0.8543	0.897456	Both
Latitude	GT23	0.2539	0.0493	0.1227	0.0765	0.1196	0.2632866	Both
Latitude	GT24	2.8985	0.1835	0.0422	-0.0082	0.3715	0.5293493	Both
Latitude	GT25	6.3037	0.6436	0.2942	0.2571	0.0111	0.0514709	Both
Latitude	GT26	137.4511	5.0718	0.1788	0.1356	0.0562	0.1607201	Both
Latitude	GT27	13.6554	0.7639	0.199	0.1568	0.0427	0.132265	Both
Latitude	GT28	177.389	1.4202	0.0233	-0.0281	0.5085	0.6478413	Both
Latitude	GT29	3.7879	-0.3711	0.3067	0.2702	0.0092	0.0461899	Both
Latitude	GT30	120.5539	2.5285	0.1324	0.0867	0.1049	0.2372809	Both
Latitude	GT3	3.0716	0.064	0.0032	-0.0493	0.809	0.8841401	Both
Latitude	GT33	1.6363	-0.1548	0.0926	0.0449	0.1798	0.3445692	Both
Latitude	GT34	0.9693	0.0221	0.0023	-0.0502	0.8374	0.897456	Both
Latitude	GT32	12.037	0.0951	0.0024	-0.0501	0.8314	0.8956516	Both
Latitude	GT31	1.5981	0.1571	0.0289	-0.0222	0.4615	0.612858	Both
Latitude	GT35	354.1091	-10.0584	0.0592	0.0097	0.2879	0.4631723	Both
Latitude	GT37	0.0077	0.0026	0.0667	0.0175	0.2585	0.4383681	Both
Latitude	GT39	21.0631	-2.0701	0.1786	0.1353	0.0563	0.1607201	Both
Latitude	GT4	1835.3972	-66.8979	0.1704	0.1267	0.0629	0.1716703	Both
Latitude	GT41	429.2439	13.3503	0.0618	0.0124	0.2773	0.4543737	Both
Latitude	GT40	0.0014	0.0001	0.0022	-0.0504	0.8415	0.897456	Both
Latitude	GT42	0.0596	0.0148	0.1107	0.0639	0.1406	0.2915625	Both
Latitude	GT43	0.0839	0.0296	0.2215	0.1806	0.0313	0.1046938	Both
Latitude	GT47	70.9951	6.5326	0.1274	0.0814	0.1123	0.2515808	Both
Latitude	GT45	0.2403	-0.0333	0.282	0.2442	0.0132	0.0583363	Both
Latitude	GT44	0.0196	-0.0121	0.423	0.3927	0.0014	0.0131212	Both
Latitude	GT48	3.6235	0.7106	0.1539	0.1094	0.0786	0.197261	Both
Latitude	GT49	0.2071	0.0437	0.1509	0.1062	0.0818	0.2033859	Both
Latitude	GT5	110.3216	2.0337	0.0651	0.0159	0.2643	0.4422512	Both
Latitude	GT50	0.7517	0.0931	0.061	0.0115	0.2806	0.4543737	Both
Latitude	GT53	24.0821	-4.6296	0.3227	0.287	0.0072	0.0395082	Both
Latitude	GT52	0	0	#VALUE!	#VALUE!	#VALUE!	NA	Both
Latitude	GT51	587.598	-27.5949	0.1646	0.1206	0.0681	0.1759003	Both
Latitude	GT54	0.0432	0.0122	0.0792	0.0307	0.2166	0.3910811	Both
Latitude	GT57	1.512	0.1463	0.0329	-0.018	0.4312	0.5851691	Both
Latitude	GT56	0.434	0.0123	0.0114	-0.0406	0.6449	0.7627654	Both
Latitude	GT55	2.7859	-0.2224	0.0549	0.0052	0.3066	0.47498	Both
Latitude	GT58	0.5931	0.0748	0.0645	0.0152	0.2667	0.4433678	Both
Latitude	GT6	0.3486	0.0805	0.2953	0.2582	0.0109	0.0514709	Both
Latitude	GT59	0.4033	0.0148	0.0047	-0.0477	0.7687	0.8613628	Both
Latitude	GT60	2.2724	0.2919	0.0612	0.0118	0.2795	0.4543737	Both
Latitude	GT61	0.4009	-0.009	0.0029	-0.0496	0.8182	0.8887246	Both
Latitude	GT62	2.2504	0.0285	0.0032	-0.0493	0.8081	0.8841401	Both
Latitude	GT64	0.8578	-0.0864	0.1839	0.1409	0.0524	0.1566112	Both
Latitude	GT65	0.0525	0.0091	0.0686	0.0195	0.2516	0.4348016	Both
Latitude	GT66	19.0025	-1.8431	0.4062	0.3749	0.0019	0.0152717	Both
Latitude	GT69	0.8788	0.0737	0.0205	-0.0311	0.5358	0.6691233	Both
Latitude	GT68	0.033	0.011	0.1675	0.1237	0.0654	0.172803	Both
Latitude	GT7	5.6797	0.2444	0.0897	0.0418	0.1873	0.3507507	Both
Latitude	GT72	0.0007	0.0004	0.0639	0.0146	0.2691	0.4442978	Both
Latitude	GT73	0.0676	0.0038	0.0051	-0.0472	0.758	0.8570591	Both
Latitude	GT71	1.4697	-0.0774	0.038	-0.0127	0.3973	0.5530028	Both
Latitude	GT70	0.7719	0.0539	0.1794	0.1362	0.0557	0.1607201	Both
Latitude	GT74	0.8743	0.1416	0.2962	0.2592	0.0107	0.0514709	Both
Latitude	GT75	0.9562	0.0881	0.2166	0.1754	0.0335	0.1091251	Both
Latitude	GT76	2.0735	-0.3805	0.1742	0.1307	0.0598	0.1672815	Both

Latitude	GT77	0.8498	-0.0262	0.0071	-0.0451	0.7157	0.8240905	Both
Latitude	GT78	0.0021	-0.0013	0.091	0.0431	0.1839	0.3470487	Both
Latitude	GT8	11.3981	-1.1284	0.2536	0.2144	0.0199	0.0769708	Both
Latitude	GT80	5.8542	0.4713	0.2612	0.2223	0.0179	0.0721019	Both
Latitude	GT82	0.4408	-0.0708	0.1997	0.1576	0.0423	0.132265	Both
Latitude	GT84	302.7752	43.7033	0.5135	0.4879	0.0003	0.0040438	Both
Latitude	GT81	24.2095	-2.0049	0.4024	0.3709	0.002	0.0153083	Both
Latitude	GT83	173.1688	5.1365	0.1475	0.1027	0.0856	0.2071226	Both
Latitude	GT85	3.3791	-0.6491	0.2604	0.2215	0.0181	0.0721019	Both
Latitude	GT87	17.8526	-1.6162	0.1439	0.0988	0.0899	0.2108876	Both
Latitude	GT88	0.0319	-0.0005	0.0004	-0.0522	0.934	0.9452916	Both
Latitude	GT89	5.7995	-1.0735	0.5047	0.4786	0.0003	0.0042909	Both
Latitude	GT90	2.7262	0.1608	0.0188	-0.0328	0.5529	0.6836785	Both
Latitude	GT9	169.2922	-3.2707	0.0362	-0.0145	0.4086	0.5604384	Both
Latitude	GT91	0.0175	0.0012	0.006	-0.0463	0.739	0.8431652	Both
Latitude	GT93	0.367	-0.0027	0.0044	-0.048	0.7757	0.8653283	Both
Latitude	GT94	0.8321	-0.0111	0.0018	-0.0507	0.8545	0.897456	Both
Latitude	GT92	0.1294	0.0316	0.1439	0.0989	0.0899	0.2108876	Both
Latitude	GT95	6.8457	-0.1753	0.0412	-0.0092	0.3773	0.5320669	Both
Latitude	PL1	17.2792	-4.8921	0.5395	0.5153	0.0001	0.003064	Both
Latitude	PL10	4.8889	-0.9323	0.1894	0.1468	0.0486	0.1469994	Both
Latitude	PL11	3.4173	-1.0603	0.3983	0.3666	0.0022	0.015926	Both
Latitude	PL12	23.1583	-1.0863	0.1093	0.0625	0.1432	0.294552	Both
Latitude	PL13	0.0005	0.0002	0.0174	-0.0343	0.5684	0.6959181	Both
Latitude	PL14	1.0529	-0.1452	0.0774	0.0289	0.222	0.3979739	Both
Latitude	PL15	0.3252	-0.1033	0.2452	0.2055	0.0225	0.0816973	Both
Latitude	PL17	1.5798	-0.6208	0.4403	0.4109	0.001	0.0104385	Both
Latitude	PL18	0.003	-0.0039	0.7329	0.7188	0.00E+00	0.0001865	Both
Latitude	PL16	0.5166	-0.0571	0.2518	0.2124	0.0205	0.0771003	Both
Latitude	PL2	0.1859	0.0541	0.235	0.1947	0.0259	0.0917072	Both
Latitude	PL20	0.0885	-0.0534	0.5361	0.5117	0.0002	0.003064	Both
Latitude	PL21	0.5039	0.0129	0.0047	-0.0477	0.7678	0.8613628	Both
Latitude	PL22	10.1314	0.7523	0.0975	0.05	0.1682	0.3324592	Both
Latitude	PL23	0.0169	-0.0022	0.0881	0.0401	0.1915	0.3533519	Both
Latitude	PL3	0.2609	-0.2031	0.3894	0.3573	0.0025	0.0174351	Both
Latitude	PL4	18.5959	2.7376	0.1012	0.0538	0.16	0.3187545	Both
Latitude	PL5	2.4899	0.4703	0.0536	0.0037	0.3128	0.4774038	Both
Latitude	PL6	1.9888	-1.0269	0.5863	0.5645	1.00E-04	0.0018725	Both
Latitude	PL7	3.6439	-0.8948	0.3145	0.2784	0.0082	0.0435493	Both
Latitude	PL8	2.6452	-0.3358	0.1193	0.0729	0.1252	0.2684955	Both
Latitude	PL9	31.9165	-6.0267	0.4647	0.4366	0.0007	0.0069598	Both
Longitude	AA1	166.2439	4.4887	0.0556	0.0059	0.3034	0.4943599	Both
Longitude	AA11	0.4336	-0.0718	0.0662	0.0171	0.2602	0.4442346	Both
Longitude	AA10	6.525	-0.4249	0.0546	0.0048	0.3081	0.4957621	Both
Longitude	AA2	1.5806	-0.1335	0.0552	0.0055	0.3053	0.4943599	Both
Longitude	AA3	414.0022	-24.5502	0.2293	0.1887	0.0281	0.0881347	Both
Longitude	AA4	13.9824	2.1291	0.0995	0.0521	0.1637	0.3260215	Both
Longitude	AA5	47.5523	-6.7124	0.2568	0.2177	0.0191	0.0655507	Both
Longitude	AA8	0.7042	-0.1057	0.1262	0.0802	0.114	0.2601178	Both
Longitude	AA7	55.8067	-4.7152	0.2602	0.2213	0.0182	0.063293	Both
Longitude	AA6	28.9935	-1.9584	0.1397	0.0944	0.0951	0.2272779	Both
Longitude	AA9	1.5682	-0.3959	0.1196	0.0732	0.1247	0.2745326	Both
Longitude	CE1	214.5689	-7.0646	0.2871	0.2496	0.0123	0.0447128	Both
Longitude	CE11	161.0901	-3.4133	0.09	0.0421	0.1865	0.3628208	Both
Longitude	CE12	17.1999	-1.3423	0.0693	0.0204	0.2488	0.4306591	Both

Longitude	CE13	0.0445	0.0042	0.0133	-0.0386	0.6183	0.8156531	Both
Longitude	CE14	222.8524	-2.6114	0.0324	-0.0185	0.4349	0.6272952	Both
Longitude	CE15	41.9051	0.9567	0.0082	-0.044	0.6964	0.8457975	Both
Longitude	CE2	2.7115	-0.5734	0.2046	0.1627	0.0395	0.1180854	Both
Longitude	CE3	23.2608	-10.0691	0.8256	0.8164	0.00E+00	7.72E-07	Both
Longitude	CE4	354.0802	-41.2565	0.4249	0.3946	0.0014	0.0061483	Both
Longitude	CE16	1.0609	-0.0692	0.0508	0.0008	0.3261	0.5115659	Both
Longitude	CE5	14.6374	-0.003	0.00E+00	-0.0526	0.9982	0.9982486	Both
Longitude	CE7	11.1596	-0.9492	0.1264	0.0804	0.1137	0.2601178	Both
Longitude	CE6	41.6877	5.182	0.2334	0.193	0.0265	0.0853935	Both
Longitude	CE8	22.9784	-1.7379	0.1266	0.0807	0.1134	0.2601178	Both
Longitude	CE9	93.7508	-2.8382	0.3867	0.3544	0.0026	0.0104273	Both
Longitude	GH100	0.8338	0.0353	0.0036	-0.0488	0.7953	0.8982623	Both
Longitude	GH10	54.9015	-1.585	0.0471	-0.003	0.3446	0.5210543	Both
Longitude	GH102	40.7964	-3.336	0.4436	0.4143	0.001	0.0047307	Both
Longitude	GH101	0.7299	-0.3181	0.777	0.7653	0.00E+00	3.27E-06	Both
Longitude	GH103	106.2158	-10.0702	0.2549	0.2157	0.0196	0.0664146	Both
Longitude	GH104	5.1644	-0.6249	0.3372	0.3023	0.0058	0.0219839	Both
Longitude	GH105	29.0354	1.1893	0.0122	-0.0398	0.6338	0.8180044	Both
Longitude	GH106	49.5372	2.4818	0.0278	-0.0234	0.4703	0.6558043	Both
Longitude	GH107	0.1563	-0.1291	0.8523	0.8446	0.00E+00	4.46E-07	Both
Longitude	GH108	6.355	-0.0742	0.01	-0.0422	0.667	0.8409533	Both
Longitude	GH109	20.0561	-1.1631	0.2123	0.1708	0.0355	0.1082314	Both
Longitude	GH11	14.887	0.0591	0.0002	-0.0524	0.9468	0.9621299	Both
Longitude	GH110	1.2431	0.0275	0.0035	-0.0489	0.7986	0.8982623	Both
Longitude	GH111	0.0414	-0.0164	0.4083	0.3771	0.0018	0.0076179	Both
Longitude	GH112	0.0997	0.0031	0.0009	-0.0516	0.8952	0.948048	Both
Longitude	GH113	8.2393	-1.5271	0.7176	0.7027	0.00E+00	2.28E-05	Both
Longitude	GH114	10.5907	-1.4777	0.1917	0.1492	0.0471	0.132888	Both
Longitude	GH115	18.1582	-0.8416	0.0097	-0.0424	0.6714	0.8409533	Both
Longitude	GH116	44.574	7.0702	0.4775	0.45	0.0005	0.0029197	Both
Longitude	GH117	3.4772	-0.4275	0.2139	0.1725	0.0348	0.1077728	Both
Longitude	GH118	0.0029	-0.0012	0.151	0.1063	0.0817	0.2050307	Both
Longitude	GH119	2.7623	-0.9797	0.7206	0.7059	0.00E+00	2.22E-05	Both
Longitude	GH12	8.8559	-0.6089	0.0493	-0.0007	0.3332	0.5163008	Both
Longitude	GH120	1.8739	0.1594	0.0293	-0.0218	0.4582	0.646162	Both
Longitude	GH121	1.5942	-1.1293	0.8188	0.8093	0.00E+00	8.87E-07	Both
Longitude	GH123	1.8672	-0.7039	0.4945	0.4679	0.0004	0.0022497	Both
Longitude	GH124	0.0511	0.0009	0.0006	-0.052	0.9192	0.9552197	Both
Longitude	GH125	11.4055	0.9749	0.0882	0.0402	0.1911	0.3689216	Both
Longitude	GH126	0.0093	0.0022	0.045	-0.0053	0.3561	0.5288797	Both
Longitude	GH127	44.4763	0.5342	0.003	-0.0495	0.8143	0.8982623	Both
Longitude	GH128	6.9808	0.143	0.0032	-0.0493	0.8075	0.8982623	Both
Longitude	GH129	0.3906	0.0165	0.0116	-0.0404	0.6425	0.8185914	Both
Longitude	GH13	1501.8611	-44.4815	0.1563	0.1119	0.0761	0.1966792	Both
Longitude	GH130	86.8053	2.428	0.0303	-0.0207	0.4502	0.638477	Both
Longitude	GH131	0.195	-0.0265	0.0461	-0.0041	0.3502	0.5231485	Both
Longitude	GH132	0.7589	-0.037	0.0069	-0.0454	0.7207	0.8614326	Both
Longitude	GH133	72.2732	-0.1487	0.0001	-0.0525	0.9587	0.9702736	Both
Longitude	GH14	0.5412	0.0141	0.0081	-0.0441	0.6975	0.8457975	Both
Longitude	GH17	59.077	-4.9555	0.2412	0.2012	0.0238	0.0785308	Both
Longitude	GH15	408.8678	10.0967	0.0664	0.0172	0.2596	0.4442346	Both
Longitude	GH18	245.204	-12.6656	0.1942	0.1518	0.0455	0.1298817	Both
Longitude	GH19	16.9586	-2.6605	0.5706	0.548	1.00E-04	0.000572	Both
Longitude	GH2	388.7654	3.6536	0.0029	-0.0496	0.816	0.8982623	Both

Longitude	GH20	100.202	-3.0379	0.0866	0.0385	0.1953	0.3742086	Both
Longitude	GH23	392.3336	-29.5828	0.8083	0.7982	0.00E+00	1.27E-06	Both
Longitude	GH24	15.9645	-0.6343	0.1364	0.091	0.0994	0.2331585	Both
Longitude	GH25	19.8627	-3.1261	0.5522	0.5287	0.0001	0.0007921	Both
Longitude	GH26	22.0532	-1.6154	0.1241	0.078	0.1174	0.2630342	Both
Longitude	GH27	80.433	4.4609	0.0374	-0.0132	0.4006	0.5846404	Both
Longitude	GH28	194.68	4.9731	0.013	-0.039	0.6228	0.8156531	Both
Longitude	GH29	112.4106	8.06	0.1773	0.1341	0.0573	0.1562643	Both
Longitude	GH3	587.4424	19.6171	0.0425	-0.0079	0.3699	0.5429104	Both
Longitude	GH30	51.6227	0.2861	0.001	-0.0516	0.8938	0.948048	Both
Longitude	GH31	139.0432	13.8883	0.1988	0.1566	0.0428	0.1248488	Both
Longitude	GH33	59.3874	-8.6305	0.6328	0.6135	0.00E+00	0.0001511	Both
Longitude	GH36	91.5089	7.7543	0.1096	0.0627	0.1427	0.3061124	Both
Longitude	GH32	31.3611	0.68	0.0605	0.011	0.2826	0.4704086	Both
Longitude	GH37	39.0498	6.2719	0.5408	0.5166	0.0001	0.0009626	Both
Longitude	GH39	89.459	7.3414	0.1614	0.1173	0.071	0.1857256	Both
Longitude	GH38	117.6702	10.691	0.1029	0.0557	0.1563	0.3214885	Both
Longitude	GH4	144.275	-14.9504	0.4307	0.4007	0.0012	0.0057982	Both
Longitude	GH42	45.2312	1.8937	0.077	0.0284	0.2234	0.4138198	Both
Longitude	GH43	113.62	-6.3237	0.0435	-0.0069	0.3644	0.5380481	Both
Longitude	GH44	26.6648	0.014	0.00E+00	-0.0526	0.9926	0.996543	Both
Longitude	GH45	0.4552	-0.0254	0.0159	-0.0359	0.5865	0.7830693	Both
Longitude	GH47	15.1347	-1.159	0.2118	0.1703	0.0358	0.1082314	Both
Longitude	GH46	2.1056	-0.9746	0.6702	0.6528	0.00E+00	6.84E-05	Both
Longitude	GH48	9.684	-0.3796	0.0477	-0.0024	0.3416	0.5196265	Both
Longitude	GH49	0.1506	-0.1517	0.8016	0.7912	0.00E+00	1.52E-06	Both
Longitude	GH5	170.4089	-16.779	0.5056	0.4796	0.0003	0.0018961	Both
Longitude	GH50	7.8055	0.6307	0.1006	0.0533	0.1612	0.3237479	Both
Longitude	GH51	110.7785	6.6083	0.0725	0.0237	0.2379	0.4235615	Both
Longitude	GH54	21.4612	3.5659	0.093	0.0452	0.179	0.3509343	Both
Longitude	GH53	57.7565	0.2147	0.0004	-0.0522	0.9286	0.9552197	Both
Longitude	GH55	111.9622	15.6493	0.2316	0.1911	0.0272	0.0864415	Both
Longitude	GH56	0.0046	0.002	0.096	0.0484	0.1717	0.3392573	Both
Longitude	GH57	153.7528	1.0521	0.0048	-0.0476	0.7665	0.8920639	Both
Longitude	GH58	0.039	-0.0203	0.6482	0.6297	0.00E+00	0.000111	Both
Longitude	GH59	8.0215	1.0624	0.0699	0.021	0.2468	0.4301466	Both
Longitude	GH6	18.8877	-1.7321	0.106	0.059	0.1498	0.3132514	Both
Longitude	GH62	3.2816	-0.8173	0.3899	0.3578	0.0025	0.0100343	Both
Longitude	GH63	15.2609	-1.1293	0.0804	0.032	0.2128	0.404606	Both
Longitude	GH64	20.0853	0.341	0.0066	-0.0457	0.7267	0.8644477	Both
Longitude	GH65	100.4831	-2.4033	0.0121	-0.0399	0.6355	0.8180044	Both
Longitude	GH66	1.539	-0.614	0.652	0.6336	0.00E+00	0.0001101	Both
Longitude	GH68	0.282	-0.0069	0.0112	-0.0409	0.6484	0.821971	Both
Longitude	GH7	0.7235	-0.1835	0.1244	0.0783	0.1168	0.2630342	Both
Longitude	GH70	0.0383	-0.0202	0.466	0.4379	0.0007	0.0033992	Both
Longitude	GH71	3.9384	-0.1946	0.0118	-0.0402	0.6389	0.8182422	Both
Longitude	GH72	30.0493	-2.1958	0.4667	0.4386	0.0006	0.0033992	Both
Longitude	GH73	38.6432	-3.1349	0.6806	0.6638	0.00E+00	5.54E-05	Both
Longitude	GH75	2.2066	-0.3295	0.3135	0.2773	0.0083	0.0306622	Both
Longitude	GH74	13.9963	-0.7421	0.0463	-0.0039	0.3488	0.5231485	Both
Longitude	GH76	14.814	-0.1194	0.0007	-0.0519	0.9121	0.9552197	Both
Longitude	GH78	155.8729	-1.4478	0.0041	-0.0483	0.7824	0.8982623	Both
Longitude	GH79	28.1345	4.5571	0.5489	0.5252	0.0001	0.0008279	Both
Longitude	GH8	19.2344	-2.9304	0.3633	0.3298	0.0038	0.0150044	Both
Longitude	GH80	0.002	-0.0002	0.0005	-0.0521	0.9248	0.9552197	Both

Longitude	GH81	5.7722	-1.4406	0.7105	0.6953	0.00E+00	2.70E-05	Both
Longitude	GH84	4.781	-0.5744	0.473	0.4453	0.0006	0.0031088	Both
Longitude	GH85	0.6123	-0.1761	0.453	0.4242	0.0008	0.0040697	Both
Longitude	GH86	1.587	-0.4139	0.768	0.7557	0.00E+00	4.36E-06	Both
Longitude	GH87	68.8594	1.056	0.0087	-0.0434	0.6872	0.841355	Both
Longitude	GH88	24.4555	-1.8785	0.6833	0.6666	0.00E+00	5.39E-05	Both
Longitude	GH89	7.7554	0.3049	0.0127	-0.0393	0.6272	0.8156531	Both
Longitude	GH91	0.0888	-0.0566	0.6238	0.604	0.00E+00	0.0001849	Both
Longitude	GH90	0.0034	-0.0044	0.6471	0.6285	0.00E+00	0.000111	Both
Longitude	GH9	54.2204	-2.4101	0.034	-0.0169	0.4238	0.6148248	Both
Longitude	GH92	99.629	-1.963	0.0042	-0.0482	0.7799	0.8982623	Both
Longitude	GH93	21.1746	-8.5241	0.5935	0.5721	0.00E+00	0.0003448	Both
Longitude	GH94	30.4978	3.9965	0.1703	0.1266	0.063	0.1683184	Both
Longitude	GH95	51.1365	-3.8287	0.1472	0.1023	0.086	0.2121648	Both
Longitude	GH96	0.4341	-0.3375	0.7928	0.7819	0.00E+00	2.01E-06	Both
Longitude	GH97	31.9027	-4.1357	0.4849	0.4578	0.0005	0.0026496	Both
Longitude	GH98	0.2928	-0.0605	0.2838	0.2461	0.0129	0.046289	Both
Longitude	GH99	12.1809	-2.5781	0.7856	0.7743	0.00E+00	2.49E-06	Both
Longitude	GT1	152.7224	-13.4034	0.2037	0.1617	0.04	0.1182091	Both
Longitude	GT10	2.6221	0.0595	0.0088	-0.0434	0.6864	0.841355	Both
Longitude	GT12	0.0479	0.001	0.0022	-0.0503	0.8383	0.9108612	Both
Longitude	GT14	2.0818	-0.0759	0.0218	-0.0297	0.5231	0.7214221	Both
Longitude	GT11	5.172	0.273	0.0706	0.0217	0.2442	0.4287127	Both
Longitude	GT13	0.1791	0.03	0.0607	0.0113	0.2816	0.4704086	Both
Longitude	GT15	2.0332	0.0841	0.007	-0.0453	0.7184	0.8614326	Both
Longitude	GT16	0.0469	0.0101	0.0763	0.0277	0.2253	0.4138198	Both
Longitude	GT17	1.0854	0.0039	0.0003	-0.0523	0.9369	0.9559887	Both
Longitude	GT18	0.0144	0.005	0.147	0.1021	0.0862	0.2121648	Both
Longitude	GT2	1818.8004	-56.312	0.4301	0.4001	0.0012	0.0057982	Both
Longitude	GT19	83.4957	-0.2512	0.0026	-0.0498	0.8248	0.9001609	Both
Longitude	GT20	149.4495	2.416	0.1516	0.1069	0.0811	0.2050307	Both
Longitude	GT21	78.1092	14.2513	0.6053	0.5845	0.00E+00	0.0002688	Both
Longitude	GT22	4.5977	-0.2621	0.0514	0.0015	0.3229	0.5115659	Both
Longitude	GT23	0.2539	0.0319	0.0513	0.0013	0.3236	0.5115659	Both
Longitude	GT24	2.8985	0.1007	0.0127	-0.0392	0.6263	0.8156531	Both
Longitude	GT25	6.3037	0.2864	0.0583	0.0087	0.2918	0.4786771	Both
Longitude	GT26	137.4511	-1.5441	0.0166	-0.0352	0.5781	0.7801197	Both
Longitude	GT27	13.6554	0.6987	0.1664	0.1226	0.0664	0.1753431	Both
Longitude	GT28	177.389	-3.995	0.1848	0.1418	0.0518	0.1444865	Both
Longitude	GT29	3.7879	-0.4342	0.4197	0.3892	0.0015	0.006584	Both
Longitude	GT30	120.5539	-0.951	0.0187	-0.0329	0.5542	0.7537953	Both
Longitude	GT3	3.0716	-0.1616	0.0201	-0.0315	0.5402	0.7409353	Both
Longitude	GT33	1.6363	-0.4239	0.6944	0.6783	0.00E+00	4.28E-05	Both
Longitude	GT34	0.9693	-0.0271	0.0034	-0.049	0.8011	0.8982623	Both
Longitude	GT32	12.037	-0.801	0.1734	0.1299	0.0604	0.1629988	Both
Longitude	GT31	1.5981	-0.0783	0.0072	-0.0451	0.7151	0.8614326	Both
Longitude	GT35	354.1091	14.0505	0.1155	0.069	0.1317	0.2874337	Both
Longitude	GT37	0.0077	0.0033	0.1073	0.0603	0.1472	0.3103951	Both
Longitude	GT39	21.0631	-2.3714	0.2343	0.194	0.0262	0.0853543	Both
Longitude	GT4	1835.3972	-140.9569	0.7565	0.7437	0.00E+00	6.36E-06	Both
Longitude	GT41	429.2439	18.8613	0.1233	0.0772	0.1186	0.2633324	Both
Longitude	GT40	0.0014	0.0005	0.0311	-0.0199	0.4444	0.6337198	Both
Longitude	GT42	0.0596	0.0061	0.0186	-0.0331	0.5556	0.7537953	Both
Longitude	GT43	0.0839	0.0234	0.1385	0.0931	0.0967	0.2289257	Both
Longitude	GT47	70.9951	0.3476	0.0004	-0.0523	0.9349	0.9559887	Both

Longitude	GT45	0.2403	-0.0409	0.4247	0.3944	0.0014	0.0061483	Both
Longitude	GT44	0.0196	-0.0046	0.0619	0.0125	0.277	0.469758	Both
Longitude	GT48	3.6235	0.4041	0.0498	-0.0003	0.3311	0.5161937	Both
Longitude	GT49	0.2071	0.0376	0.1121	0.0653	0.138	0.2986118	Both
Longitude	GT5	110.3216	-2.2335	0.0785	0.03	0.2186	0.4124573	Both
Longitude	GT50	0.7517	0.0276	0.0053	-0.047	0.7528	0.8870498	Both
Longitude	GT53	24.0821	0.2846	0.0012	-0.0513	0.8806	0.9409808	Both
Longitude	GT52	0	0	#VALUE!	#VALUE!	#VALUE!	NA	Both
Longitude	GT51	587.598	-51.295	0.5687	0.546	1.00E-04	0.0005801	Both
Longitude	GT54	0.0432	0.0117	0.0728	0.024	0.2369	0.4235615	Both
Longitude	GT57	1.512	0.0469	0.0034	-0.0491	0.8025	0.8982623	Both
Longitude	GT56	0.434	-0.0033	0.0008	-0.0517	0.9006	0.9498214	Both
Longitude	GT55	2.7859	-0.0581	0.0037	-0.0487	0.792	0.8982623	Both
Longitude	GT58	0.5931	0.0288	0.0096	-0.0426	0.6734	0.8409533	Both
Longitude	GT6	0.3486	0.0766	0.2671	0.2285	0.0164	0.05812	Both
Longitude	GT59	0.4033	-0.0152	0.005	-0.0474	0.7616	0.8920639	Both
Longitude	GT60	2.2724	0.3803	0.1039	0.0568	0.1541	0.3195809	Both
Longitude	GT61	0.4009	-0.001	0.00E+00	-0.0526	0.9789	0.9867679	Both
Longitude	GT62	2.2504	-0.0635	0.0159	-0.0359	0.5865	0.7830693	Both
Longitude	GT64	0.8578	-0.1673	0.6898	0.6734	0.00E+00	4.66E-05	Both
Longitude	GT65	0.0525	0.0131	0.1437	0.0987	0.0901	0.2174048	Both
Longitude	GT66	19.0025	-2.3688	0.671	0.6537	0.00E+00	6.84E-05	Both
Longitude	GT69	0.8788	-0.0214	0.0017	-0.0508	0.8578	0.9240156	Both
Longitude	GT68	0.033	0.0072	0.0725	0.0237	0.2377	0.4235615	Both
Longitude	GT7	5.6797	0.2253	0.0762	0.0276	0.2257	0.4138198	Both
Longitude	GT72	0.0007	0.0004	0.0707	0.0218	0.2439	0.4287127	Both
Longitude	GT73	0.0676	-0.0147	0.0762	0.0276	0.2259	0.4138198	Both
Longitude	GT71	1.4697	-0.1079	0.0737	0.025	0.2337	0.4235615	Both
Longitude	GT70	0.7719	-0.0121	0.0091	-0.0431	0.6814	0.841355	Both
Longitude	GT74	0.8743	0.1771	0.4636	0.4354	0.0007	0.0034797	Both
Longitude	GT75	0.9562	0.0465	0.0604	0.0109	0.283	0.4704086	Both
Longitude	GT76	2.0735	-0.2904	0.1015	0.0542	0.1593	0.3225524	Both
Longitude	GT77	0.8498	-0.0142	0.0021	-0.0504	0.8439	0.9130639	Both
Longitude	GT78	0.0021	1.00E-04	0.0005	-0.0521	0.9258	0.9552197	Both
Longitude	GT8	11.3981	-1.7428	0.605	0.5842	0.00E+00	0.0002688	Both
Longitude	GT80	5.8542	0.0503	0.003	-0.0495	0.8144	0.8982623	Both
Longitude	GT82	0.4408	-0.1022	0.4157	0.3849	0.0016	0.0068216	Both
Longitude	GT84	302.7752	13.3566	0.048	-0.0021	0.3402	0.5196265	Both
Longitude	GT81	24.2095	-1.8982	0.3607	0.327	0.004	0.0154188	Both
Longitude	GT83	173.1688	1.2808	0.0092	-0.043	0.6796	0.841355	Both
Longitude	GT85	3.3791	0.0872	0.0047	-0.0477	0.7677	0.8920639	Both
Longitude	GT87	17.8526	-0.939	0.0486	-0.0015	0.3371	0.5190424	Both
Longitude	GT88	0.0319	0.0123	0.1956	0.1532	0.0447	0.1289763	Both
Longitude	GT89	5.7995	-0.483	0.1022	0.0549	0.1578	0.3220311	Both
Longitude	GT90	2.7262	-0.0264	0.0005	-0.0521	0.9227	0.9552197	Both
Longitude	GT9	169.2922	-12.8711	0.561	0.5379	1.00E-04	0.0006711	Both
Longitude	GT91	0.0175	-0.001	0.0037	-0.0488	0.7944	0.8982623	Both
Longitude	GT93	0.367	-0.0068	0.0281	-0.0231	0.4677	0.655797	Both
Longitude	GT94	0.8321	0.0311	0.0142	-0.0377	0.6068	0.8058715	Both
Longitude	GT92	0.1294	0.0188	0.051	0.001	0.3251	0.5115659	Both
Longitude	GT95	6.8457	0.0446	0.0027	-0.0498	0.8239	0.9001609	Both
Longitude	PL1	17.2792	-4.8726	0.5352	0.5107	0.0002	0.0010559	Both
Longitude	PL10	4.8889	-1.0772	0.2529	0.2136	0.0201	0.0673987	Both
Longitude	PL11	3.4173	-1.0871	0.4186	0.388	0.0015	0.0065931	Both
Longitude	PL12	23.1583	-2.0626	0.3942	0.3623	0.0023	0.0094982	Both

Longitude	PL13	0.0005	0.0004	0.0589	0.0094	0.2892	0.4775666	Both
Longitude	PL14	1.0529	-0.4113	0.6213	0.6014	0.00E+00	0.0001904	Both
Longitude	PL15	0.3252	-0.192	0.8468	0.8387	0.00E+00	4.46E-07	Both
Longitude	PL17	1.5798	-0.8528	0.8309	0.8219	0.00E+00	7.66E-07	Both
Longitude	PL18	0.003	-0.0018	0.1556	0.1112	0.0768	0.1966792	Both
Longitude	PL16	0.5166	-0.004	0.0012	-0.0514	0.881	0.9409808	Both
Longitude	PL2	0.1859	0.0369	0.1089	0.062	0.144	0.3062217	Both
Longitude	PL20	0.0885	-0.0515	0.4991	0.4727	0.0003	0.0021062	Both
Longitude	PL21	0.5039	0.0799	0.1806	0.1375	0.0548	0.1512038	Both
Longitude	PL22	10.1314	-0.3646	0.0229	-0.0285	0.5126	0.7109086	Both
Longitude	PL23	0.0169	-0.0005	0.0056	-0.0467	0.7472	0.8846217	Both
Longitude	PL3	0.2609	-0.22	0.4571	0.4286	0.0008	0.0038475	Both
Longitude	PL4	18.5959	1.5239	0.0313	-0.0196	0.4427	0.6337198	Both
Longitude	PL5	2.4899	0.7751	0.1455	0.1005	0.088	0.2145072	Both
Longitude	PL6	1.9888	-1.0763	0.6441	0.6253	0.00E+00	0.000116	Both
Longitude	PL7	3.6439	-1.1059	0.4805	0.4531	0.0005	0.0028208	Both
Longitude	PL8	2.6452	-0.5584	0.3299	0.2946	0.0065	0.024229	Both
Longitude	PL9	31.9165	-7.1127	0.6473	0.6288	0.00E+00	0.000111	Both
Elevation	AA1	166.2439	0.3749	0.0004	-0.0522	0.9325	0.9636719	Both
Elevation	AA11	0.4336	0.0941	0.1139	0.0673	0.1346	0.321648	Both
Elevation	AA10	6.525	0.4738	0.0678	0.0188	0.2541	0.4869217	Both
Elevation	AA2	1.5806	0.1988	0.1224	0.0762	0.12	0.2896509	Both
Elevation	AA3	414.0022	18.4796	0.1299	0.0841	0.1085	0.2778527	Both
Elevation	AA4	13.9824	-1.1474	0.0289	-0.0222	0.4613	0.6468477	Both
Elevation	AA5	47.5523	5.3265	0.1617	0.1176	0.0708	0.2041654	Both
Elevation	AA8	0.7042	0.1336	0.2018	0.1598	0.0411	0.1356521	Both
Elevation	AA7	55.8067	2.5499	0.0761	0.0275	0.2262	0.4652843	Both
Elevation	AA6	28.9935	0.5766	0.0121	-0.0399	0.6349	0.79281	Both
Elevation	AA9	1.5682	0.4654	0.1652	0.1213	0.0675	0.1996393	Both
Elevation	CE1	214.5689	3.0037	0.0519	0.002	0.3206	0.5474115	Both
Elevation	CE11	161.0901	3.1927	0.0787	0.0302	0.218	0.4559085	Both
Elevation	CE12	17.1999	1.7101	0.1126	0.0658	0.1371	0.3245928	Both
Elevation	CE13	0.0445	-0.0077	0.0444	-0.0059	0.3591	0.5705085	Both
Elevation	CE14	222.8524	1.3022	0.0081	-0.0441	0.6988	0.8472975	Both
Elevation	CE15	41.9051	-0.4504	0.0018	-0.0507	0.8544	0.9340695	Both
Elevation	CE2	2.7115	0.5725	0.2039	0.162	0.0399	0.1334708	Both
Elevation	CE3	23.2608	8.4842	0.5861	0.5643	1.00E-04	0.0010121	Both
Elevation	CE4	354.0802	37.7545	0.3558	0.3219	0.0043	0.0283252	Both
Elevation	CE16	1.0609	0.1004	0.1069	0.0599	0.1479	0.3405929	Both
Elevation	CE5	14.6374	-1.5546	0.0689	0.0199	0.2503	0.4869217	Both
Elevation	CE7	11.1596	1.0341	0.15	0.1053	0.0828	0.2210466	Both
Elevation	CE6	41.6877	-4.6056	0.1843	0.1414	0.0521	0.1634899	Both
Elevation	CE8	22.9784	1.2535	0.0659	0.0167	0.2614	0.4907695	Both
Elevation	CE9	93.7508	2.0054	0.193	0.1506	0.0463	0.1508678	Both
Elevation	GH100	0.8338	0.0403	0.0047	-0.0476	0.7669	0.8946063	Both
Elevation	GH10	54.9015	1.008	0.0191	-0.0326	0.5507	0.7199166	Both
Elevation	GH102	40.7964	2.5632	0.2619	0.223	0.0177	0.0741351	Both
Elevation	GH101	0.7299	0.2619	0.5267	0.5017	0.0002	0.002241	Both
Elevation	GH103	106.2158	10.7245	0.2891	0.2517	0.0119	0.0555002	Both
Elevation	GH104	5.1644	0.6937	0.4155	0.3847	0.0016	0.012625	Both
Elevation	GH105	29.0354	-0.9355	0.0075	-0.0447	0.7082	0.8505343	Both
Elevation	GH106	49.5372	-1.9542	0.0172	-0.0345	0.5707	0.7346275	Both
Elevation	GH107	0.1563	0.1074	0.5898	0.5682	0.00E+00	0.0010043	Both
Elevation	GH108	6.355	-0.0326	0.0019	-0.0506	0.8505	0.9340695	Both
Elevation	GH109	20.0561	0.7785	0.0951	0.0475	0.1737	0.3859398	Both

Elevation	GH11	14.887	0.1575	0.0017	-0.0508	0.8589	0.9340695	Both
Elevation	GH110	1.2431	0.0087	0.0004	-0.0523	0.9356	0.9636719	Both
Elevation	GH111	0.0414	0.0127	0.2471	0.2075	0.0219	0.084438	Both
Elevation	GH112	0.0997	0.001	1.00E-04	-0.0525	0.9664	0.9761411	Both
Elevation	GH113	8.2393	1.5245	0.7152	0.7002	0.00E+00	7.85E-05	Both
Elevation	GH114	10.5907	0.6361	0.0355	-0.0152	0.4132	0.6122248	Both
Elevation	GH115	18.1582	1.6078	0.0353	-0.0155	0.4146	0.6122248	Both
Elevation	GH116	44.574	-5.7378	0.3145	0.2784	0.0082	0.0446344	Both
Elevation	GH117	3.4772	0.1728	0.035	-0.0158	0.4171	0.6122248	Both
Elevation	GH118	0.0029	0.0009	0.0849	0.0367	0.2	0.4327966	Both
Elevation	GH119	2.7623	0.8695	0.5676	0.5448	1.00E-04	0.001349	Both
Elevation	GH12	8.8559	0.4777	0.0304	-0.0207	0.45	0.6351064	Both
Elevation	GH120	1.8739	-0.4323	0.2154	0.1741	0.0341	0.1187282	Both
Elevation	GH121	1.5942	0.9116	0.5336	0.5091	0.0002	0.002027	Both
Elevation	GH123	1.8672	0.5742	0.329	0.2937	0.0066	0.0373815	Both
Elevation	GH124	0.0511	-0.0018	0.002	-0.0505	0.8479	0.9340695	Both
Elevation	GH125	11.4055	-0.814	0.0615	0.0121	0.2785	0.5064666	Both
Elevation	GH126	0.0093	-0.0042	0.1651	0.1211	0.0676	0.1996393	Both
Elevation	GH127	44.4763	-0.644	0.0043	-0.0481	0.777	0.8946063	Both
Elevation	GH128	6.9808	-0.0598	0.0006	-0.052	0.9189	0.9574423	Both
Elevation	GH129	0.3906	-0.0415	0.0736	0.0249	0.2342	0.4671474	Both
Elevation	GH13	1501.8611	12.6862	0.0127	-0.0392	0.6265	0.7862961	Both
Elevation	GH130	86.8053	1.5029	0.0116	-0.0404	0.6419	0.7949051	Both
Elevation	GH131	0.195	0.0472	0.146	0.1011	0.0874	0.2308438	Both
Elevation	GH132	0.7589	0.0796	0.0319	-0.019	0.4385	0.6330109	Both
Elevation	GH133	72.2732	-2.1962	0.0317	-0.0193	0.4404	0.6330109	Both
Elevation	GH14	0.5412	-0.0073	0.0022	-0.0503	0.8395	0.9323674	Both
Elevation	GH17	59.077	5.5348	0.3009	0.2641	0.01	0.0493763	Both
Elevation	GH15	408.8678	-19.6424	0.2512	0.2118	0.0206	0.082258	Both
Elevation	GH18	245.204	9.5248	0.1098	0.063	0.1422	0.3314838	Both
Elevation	GH19	16.9586	1.9495	0.3064	0.2699	0.0092	0.0463683	Both
Elevation	GH2	388.7654	-2.602	0.0015	-0.0511	0.8684	0.9395105	Both
Elevation	GH20	100.202	2.2286	0.0466	-0.0036	0.3472	0.5623951	Both
Elevation	GH23	392.3336	24.5286	0.5557	0.5323	0.0001	0.0015541	Both
Elevation	GH24	15.9645	0.3166	0.034	-0.0169	0.4238	0.6184611	Both
Elevation	GH25	19.8627	2.0474	0.2369	0.1967	0.0253	0.0932439	Both
Elevation	GH26	22.0532	1.1027	0.0578	0.0082	0.2938	0.5193257	Both
Elevation	GH27	80.433	-3.4436	0.0223	-0.0291	0.5181	0.6892736	Both
Elevation	GH28	194.68	-1.4491	0.0011	-0.0515	0.8864	0.9507524	Both
Elevation	GH29	112.4106	-7.8002	0.1661	0.1222	0.0667	0.1996393	Both
Elevation	GH3	587.4424	-18.3285	0.0371	-0.0136	0.4028	0.6122248	Both
Elevation	GH30	51.6227	-0.116	0.0002	-0.0525	0.9568	0.9761411	Both
Elevation	GH31	139.0432	-12.5712	0.1629	0.1188	0.0696	0.2032092	Both
Elevation	GH33	59.3874	7.3107	0.4541	0.4253	0.0008	0.0072656	Both
Elevation	GH36	91.5089	-6.3235	0.0729	0.0241	0.2366	0.4676355	Both
Elevation	GH32	31.3611	-0.6988	0.0639	0.0146	0.269	0.4928943	Both
Elevation	GH37	39.0498	-5.3698	0.3964	0.3646	0.0022	0.0164357	Both
Elevation	GH39	89.459	-4.6185	0.0639	0.0146	0.269	0.4928943	Both
Elevation	GH38	117.6702	-7.6366	0.0525	0.0026	0.3178	0.5470289	Both
Elevation	GH4	144.275	11.9254	0.274	0.2358	0.0149	0.0660751	Both
Elevation	GH42	45.2312	-1.5113	0.049	-0.001	0.3348	0.5602979	Both
Elevation	GH43	113.62	5.0743	0.028	-0.0232	0.4685	0.6533339	Both
Elevation	GH44	26.6648	1.2071	0.035	-0.0158	0.417	0.6122248	Both
Elevation	GH45	0.4552	0.0436	0.0466	-0.0035	0.3472	0.5623951	Both
Elevation	GH47	15.1347	0.8923	0.1255	0.0795	0.115	0.2856263	Both

Elevation	GH46	2.1056	0.7014	0.3471	0.3127	0.005	0.0310827	Both
Elevation	GH48	9.684	0.2081	0.0143	-0.0375	0.6052	0.767184	Both
Elevation	GH49	0.1506	0.1536	0.8221	0.8127	0.00E+00	3.73E-06	Both
Elevation	GH5	170.4089	13.7958	0.3418	0.3072	0.0054	0.0321401	Both
Elevation	GH50	7.8055	-1.1489	0.3338	0.2988	0.0061	0.0355337	Both
Elevation	GH51	110.7785	-4.9909	0.0413	-0.0091	0.3767	0.5909295	Both
Elevation	GH54	21.4612	-2.8545	0.0596	0.0101	0.2863	0.5098229	Both
Elevation	GH53	57.7565	2.3803	0.0533	0.0035	0.3138	0.54699	Both
Elevation	GH55	111.9622	-13.2688	0.1665	0.1226	0.0663	0.1996393	Both
Elevation	GH56	0.0046	-0.0014	0.0466	-0.0036	0.3473	0.5623951	Both
Elevation	GH57	153.7528	-0.4341	0.0008	-0.0518	0.9026	0.9574423	Both
Elevation	GH58	0.039	0.0186	0.5461	0.5222	0.0001	0.0016288	Both
Elevation	GH59	8.0215	-0.9884	0.0605	0.0111	0.2825	0.5098229	Both
Elevation	GH6	18.8877	1.5534	0.0853	0.0371	0.199	0.4327966	Both
Elevation	GH62	3.2816	0.6048	0.2136	0.1722	0.0349	0.1201228	Both
Elevation	GH63	15.2609	1.0231	0.066	0.0169	0.2608	0.4907695	Both
Elevation	GH64	20.0853	-0.5654	0.0181	-0.0336	0.5611	0.729657	Both
Elevation	GH65	100.4831	-5.0086	0.0524	0.0025	0.3182	0.5470289	Both
Elevation	GH66	1.539	0.6573	0.7472	0.7339	0.00E+00	5.47E-05	Both
Elevation	GH68	0.282	0.0173	0.071	0.0221	0.2428	0.4762014	Both
Elevation	GH7	0.7235	0.2396	0.2121	0.1707	0.0356	0.1208667	Both
Elevation	GH70	0.0383	0.0207	0.4893	0.4625	0.0004	0.0045459	Both
Elevation	GH71	3.9384	0.4875	0.0742	0.0255	0.2322	0.4671474	Both
Elevation	GH72	30.0493	2.1602	0.4516	0.4228	0.0008	0.007335	Both
Elevation	GH73	38.6432	2.2627	0.3546	0.3206	0.0044	0.0283252	Both
Elevation	GH75	2.2066	0.21	0.1273	0.0814	0.1124	0.2820539	Both
Elevation	GH74	13.9963	0.872	0.064	0.0147	0.2687	0.4928943	Both
Elevation	GH76	14.814	-0.0675	0.0002	-0.0524	0.9502	0.9735022	Both
Elevation	GH78	155.8729	1.4886	0.0043	-0.0481	0.7764	0.8946063	Both
Elevation	GH79	28.1345	-3.6092	0.3443	0.3098	0.0052	0.0316535	Both
Elevation	GH8	19.2344	1.9181	0.1557	0.1112	0.0767	0.2093195	Both
Elevation	GH80	0.002	-0.002	0.0797	0.0313	0.2149	0.4532308	Both
Elevation	GH81	5.7722	1.3713	0.6438	0.6251	0.00E+00	0.0003372	Both
Elevation	GH84	4.781	0.5579	0.4463	0.4171	0.0009	0.0078195	Both
Elevation	GH85	0.6123	0.1136	0.1884	0.1457	0.0493	0.1585441	Both
Elevation	GH86	1.587	0.3697	0.6127	0.5923	0.00E+00	0.0006878	Both
Elevation	GH87	68.8594	0.7666	0.0046	-0.0478	0.7703	0.8946063	Both
Elevation	GH88	24.4555	1.7692	0.6061	0.5853	0.00E+00	0.0007373	Both
Elevation	GH89	7.7554	-0.2257	0.0069	-0.0453	0.7196	0.8600475	Both
Elevation	GH91	0.0888	0.0453	0.3996	0.368	0.0021	0.0160516	Both
Elevation	GH90	0.0034	0.0046	0.7124	0.6973	0.00E+00	7.85E-05	Both
Elevation	GH9	54.2204	0.0558	0.00E+00	-0.0526	0.9854	0.9853627	Both
Elevation	GH92	99.629	-0.1995	0.00E+00	-0.0526	0.9774	0.9812732	Both
Elevation	GH93	21.1746	8.1832	0.547	0.5231	0.0001	0.0016288	Both
Elevation	GH94	30.4978	-3.8366	0.1569	0.1125	0.0755	0.2081481	Both
Elevation	GH95	51.1365	3.5865	0.1291	0.0833	0.1096	0.2779077	Both
Elevation	GH96	0.4341	0.2601	0.4707	0.4429	0.0006	0.0062214	Both
Elevation	GH97	31.9027	2.4251	0.1667	0.1229	0.0661	0.1996393	Both
Elevation	GH98	0.2928	0.0574	0.2552	0.216	0.0195	0.0802299	Both
Elevation	GH99	12.1809	1.9834	0.465	0.4368	0.0007	0.0063988	Both
Elevation	GT1	152.7224	7.6137	0.0657	0.0165	0.262	0.4907695	Both
Elevation	GT10	2.6221	0.084	0.0175	-0.0342	0.5679	0.7346275	Both
Elevation	GT12	0.0479	-0.0018	0.0078	-0.0444	0.7027	0.848	Both
Elevation	GT14	2.0818	-0.0338	0.0043	-0.0481	0.7769	0.8946063	Both
Elevation	GT11	5.172	-0.1231	0.0144	-0.0375	0.6048	0.767184	Both

Elevation	GT13	0.1791	-0.0235	0.0371	-0.0136	0.4028	0.6122248	Both
Elevation	GT15	2.0332	0.0666	0.0044	-0.048	0.7752	0.8946063	Both
Elevation	GT16	0.0469	-0.0035	0.0091	-0.0431	0.6808	0.8295152	Both
Elevation	GT17	1.0854	0.031	0.0213	-0.0302	0.5274	0.6967407	Both
Elevation	GT18	0.0144	-0.0036	0.074	0.0253	0.2329	0.4671474	Both
Elevation	GT2	1818.8004	53.4301	0.3872	0.3549	0.0026	0.0180905	Both
Elevation	GT19	83.4957	0.8011	0.0269	-0.0243	0.4777	0.6587854	Both
Elevation	GT20	149.4495	-3.3683	0.2946	0.2575	0.011	0.0521326	Both
Elevation	GT21	78.1092	-11.8524	0.4187	0.3881	0.0015	0.012327	Both
Elevation	GT22	4.5977	0.3266	0.0799	0.0314	0.2145	0.4532308	Both
Elevation	GT23	0.2539	-0.0151	0.0115	-0.0405	0.6429	0.7949051	Both
Elevation	GT24	2.8985	-0.0084	1.00E-04	-0.0525	0.9677	0.9761411	Both
Elevation	GT25	6.3037	-0.0331	0.0008	-0.0518	0.9044	0.9574423	Both
Elevation	GT26	137.4511	4.2385	0.1249	0.0788	0.1161	0.2856263	Both
Elevation	GT27	13.6554	-0.4643	0.0735	0.0248	0.2345	0.4671474	Both
Elevation	GT28	177.389	4.8432	0.2715	0.2332	0.0154	0.0667574	Both
Elevation	GT29	3.7879	0.3234	0.2328	0.1924	0.0267	0.0970074	Both
Elevation	GT30	120.5539	1.9245	0.0767	0.0281	0.2243	0.4652157	Both
Elevation	GT3	3.0716	0.2283	0.0401	-0.0105	0.3844	0.5992666	Both
Elevation	GT33	1.6363	0.4291	0.7115	0.6963	0.00E+00	7.85E-05	Both
Elevation	GT34	0.9693	0.0655	0.02	-0.0316	0.541	0.7109127	Both
Elevation	GT32	12.037	1.0758	0.3128	0.2767	0.0084	0.0447777	Both
Elevation	GT31	1.5981	0.189	0.0418	-0.0087	0.3741	0.5905891	Both
Elevation	GT35	354.1091	-23.5107	0.3235	0.2878	0.0071	0.0398155	Both
Elevation	GT37	0.0077	-0.0023	0.0499	-0.0001	0.3305	0.556821	Both
Elevation	GT39	21.0631	1.9477	0.1581	0.1138	0.0743	0.2081481	Both
Elevation	GT4	1835.3972	130.5034	0.6484	0.6299	0.00E+00	0.000334	Both
Elevation	GT41	429.2439	-18.8226	0.1228	0.0766	0.1194	0.2896509	Both
Elevation	GT40	0.0014	-0.0006	0.0461	-0.0041	0.3502	0.5634018	Both
Elevation	GT42	0.0596	0.0013	0.0009	-0.0517	0.8965	0.9574423	Both
Elevation	GT43	0.0839	-0.0141	0.05	0.00E+00	0.3297	0.556821	Both
Elevation	GT47	70.9951	2.8829	0.0248	-0.0265	0.4953	0.6757162	Both
Elevation	GT45	0.2403	0.0301	0.2293	0.1888	0.0281	0.0992384	Both
Elevation	GT44	0.0196	-0.0004	0.0006	-0.052	0.9184	0.9574423	Both
Elevation	GT48	3.6235	-0.0619	0.0012	-0.0514	0.8831	0.9507524	Both
Elevation	GT49	0.2071	-0.0199	0.0314	-0.0196	0.442	0.6330109	Both
Elevation	GT5	110.3216	3.1588	0.1571	0.1127	0.0753	0.2081481	Both
Elevation	GT50	0.7517	0.0262	0.0048	-0.0476	0.7649	0.8946063	Both
Elevation	GT53	24.0821	-2.3228	0.0812	0.0329	0.2105	0.4515203	Both
Elevation	GT52	0	0	#VALUE!	#VALUE!	#VALUE!	NA	Both
Elevation	GT51	587.598	45.9609	0.4566	0.428	0.0008	0.0071987	Both
Elevation	GT54	0.0432	-0.0071	0.0272	-0.024	0.4749	0.6586231	Both
Elevation	GT57	1.512	0.0479	0.0035	-0.0489	0.798	0.910183	Both
Elevation	GT56	0.434	0.0073	0.004	-0.0484	0.7841	0.8986822	Both
Elevation	GT55	2.7859	0.0088	1.00E-04	-0.0525	0.9684	0.9761411	Both
Elevation	GT58	0.5931	0.0148	0.0025	-0.05	0.8283	0.9239957	Both
Elevation	GT6	0.3486	-0.058	0.1532	0.1086	0.0794	0.2142344	Both
Elevation	GT59	0.4033	0.0351	0.0263	-0.025	0.4826	0.6619492	Both
Elevation	GT60	2.2724	-0.3079	0.0681	0.0191	0.2531	0.4869217	Both
Elevation	GT61	0.4009	-0.0031	0.0003	-0.0523	0.9368	0.9636719	Both
Elevation	GT62	2.2504	0.1094	0.0471	-0.0031	0.3449	0.5623951	Both
Elevation	GT64	0.8578	0.1534	0.5798	0.5577	1.00E-04	0.0010902	Both
Elevation	GT65	0.0525	-0.0108	0.0979	0.0505	0.1672	0.3746695	Both
Elevation	GT66	19.0025	1.8154	0.3941	0.3622	0.0023	0.0165919	Both
Elevation	GT69	0.8788	0.0805	0.0245	-0.0269	0.4982	0.675929	Both

Elevation	GT68	0.033	-0.0032	0.0145	-0.0374	0.6033	0.767184	Both
Elevation	GT7	5.6797	-0.1787	0.0479	-0.0022	0.3404	0.5623951	Both
Elevation	GT72	0.0007	-0.0003	0.0312	-0.0198	0.4439	0.6330109	Both
Elevation	GT73	0.0676	0.0194	0.133	0.0873	0.1041	0.2693826	Both
Elevation	GT71	1.4697	0.097	0.0596	0.0101	0.2864	0.5098229	Both
Elevation	GT70	0.7719	0.0422	0.1096	0.0628	0.1426	0.3314838	Both
Elevation	GT74	0.8743	-0.1419	0.2977	0.2607	0.0105	0.0507853	Both
Elevation	GT75	0.9562	-0.0193	0.0104	-0.0417	0.6599	0.8079653	Both
Elevation	GT76	2.0735	0.1774	0.0379	-0.0127	0.3978	0.6122248	Both
Elevation	GT77	0.8498	0.0073	0.0006	-0.052	0.9193	0.9574423	Both
Elevation	GT78	0.0021	-0.0007	0.0238	-0.0276	0.5047	0.6774572	Both
Elevation	GT8	11.3981	1.53	0.4663	0.4382	0.0006	0.0063988	Both
Elevation	GT80	5.8542	0.1423	0.0238	-0.0276	0.5042	0.6774572	Both
Elevation	GT82	0.4408	0.0829	0.2734	0.2352	0.015	0.0660751	Both
Elevation	GT84	302.7752	2.5059	0.0017	-0.0509	0.8596	0.9340695	Both
Elevation	GT81	24.2095	1.2594	0.1588	0.1145	0.0736	0.2081481	Both
Elevation	GT83	173.1688	0.7632	0.0033	-0.0492	0.8059	0.910183	Both
Elevation	GT85	3.3791	-0.3761	0.0874	0.0394	0.1931	0.4251541	Both
Elevation	GT87	17.8526	0.4443	0.0109	-0.0412	0.6528	0.8032205	Both
Elevation	GT88	0.0319	-0.0143	0.2641	0.2254	0.0172	0.0730333	Both
Elevation	GT89	5.7995	0.0815	0.0029	-0.0496	0.8163	0.9146657	Both
Elevation	GT90	2.7262	0.1389	0.0141	-0.0378	0.6086	0.767603	Both
Elevation	GT9	169.2922	12.8358	0.5579	0.5346	0.0001	0.0015541	Both
Elevation	GT91	0.0175	0.0024	0.0222	-0.0292	0.519	0.6892736	Both
Elevation	GT93	0.367	0.0079	0.0375	-0.0132	0.4004	0.6122248	Both
Elevation	GT94	0.8321	-0.0455	0.0303	-0.0207	0.4504	0.6351064	Both
Elevation	GT92	0.1294	-0.0047	0.0032	-0.0493	0.8086	0.910183	Both
Elevation	GT95	6.8457	-0.1838	0.0453	-0.0049	0.3541	0.5661828	Both
Elevation	PL1	17.2792	3.2765	0.242	0.2021	0.0235	0.0880417	Both
Elevation	PL10	4.8889	0.8003	0.1396	0.0943	0.0952	0.2489653	Both
Elevation	PL11	3.4173	0.7222	0.1848	0.1419	0.0518	0.1634899	Both
Elevation	PL12	23.1583	1.7351	0.279	0.241	0.0138	0.0631705	Both
Elevation	PL13	0.0005	-0.0003	0.0352	-0.0156	0.4153	0.6122248	Both
Elevation	PL14	1.0529	0.4288	0.6754	0.6583	0.00E+00	0.0001888	Both
Elevation	PL15	0.3252	0.1712	0.673	0.6558	0.00E+00	0.0001888	Both
Elevation	PL17	1.5798	0.6917	0.5466	0.5227	0.0001	0.0016288	Both
Elevation	PL18	0.003	0.0001	0.0006	-0.052	0.9181	0.9574423	Both
Elevation	PL16	0.5166	-0.027	0.0563	0.0066	0.3005	0.5275096	Both
Elevation	PL2	0.1859	-0.0212	0.0361	-0.0146	0.4092	0.6122248	Both
Elevation	PL20	0.0885	0.0351	0.232	0.1915	0.0271	0.0970074	Both
Elevation	PL21	0.5039	-0.0945	0.253	0.2137	0.0201	0.0814682	Both
Elevation	PL22	10.1314	0.7658	0.101	0.0537	0.1603	0.3625015	Both
Elevation	PL23	0.0169	-0.0005	0.0045	-0.0479	0.7727	0.8946063	Both
Elevation	PL3	0.2609	0.1607	0.2438	0.204	0.0229	0.0871782	Both
Elevation	PL4	18.5959	-0.492	0.0033	-0.0492	0.8056	0.910183	Both
Elevation	PL5	2.4899	-0.6614	0.1059	0.0589	0.15	0.3421736	Both
Elevation	PL6	1.9888	0.7436	0.3074	0.271	0.0091	0.0463683	Both
Elevation	PL7	3.6439	0.8877	0.3096	0.2732	0.0088	0.0460597	Both
Elevation	PL8	2.6452	0.4851	0.2489	0.2094	0.0213	0.0835715	Both
Elevation	PL9	31.9165	5.3097	0.3607	0.3271	0.004	0.0270555	Both
DNA	AA1	161.1286	7.1406	0.1537	0.0886	0.1484	0.3183036	Mineral
DNA	AA11	0.406	0.2297	0.5506	0.516	0.0015	0.035905	Mineral
DNA	AA10	6.1103	1.2519	0.4861	0.4465	0.0039	0.0368392	Mineral
DNA	AA2	1.4848	0.4203	0.5221	0.4853	0.0023	0.035905	Mineral
DNA	AA3	426.2452	15.2725	0.1048	0.036	0.2391	0.423337	Mineral

DNA	AA4	11.2554	2.6507	0.4088	0.3633	0.0103	0.062932	Mineral
DNA	AA5	53.5482	-1.2527	0.0248	-0.0502	0.5751	0.7187258	Mineral
DNA	AA8	0.6569	0.2206	0.4649	0.4238	0.0051	0.0429439	Mineral
DNA	AA7	57.2187	2.0965	0.0512	-0.0218	0.4176	0.5759519	Mineral
DNA	AA6	30.8574	-1.2772	0.0999	0.0307	0.251	0.4257141	Mineral
DNA	AA9	1.4632	0.7107	0.2879	0.2331	0.0392	0.1385484	Mineral
DNA	CE1	216.8049	-0.873	0.0036	-0.073	0.8313	0.8994337	Mineral
DNA	CE11	163.2288	-2.3195	0.0574	-0.0151	0.3896	0.5494411	Mineral
DNA	CE12	15.6874	3.0243	0.485	0.4454	0.0039	0.0368392	Mineral
DNA	CE13	0.0362	0.00E+00	0.00E+00	-0.0769	0.9964	0.9963937	Mineral
DNA	CE14	221.0157	0.0898	1.00E-04	-0.0769	0.9795	0.9845511	Mineral
DNA	CE15	37.8269	3.3385	0.2451	0.187	0.0606	0.1762166	Mineral
DNA	CE2	2.5005	0.7593	0.3205	0.2683	0.0278	0.1092647	Mineral
DNA	CE3	26.5046	4.3977	0.1457	0.08	0.1603	0.3363344	Mineral
DNA	CE4	378.0703	11.1945	0.0557	-0.0169	0.3971	0.5537359	Mineral
DNA	CE16	1.0559	0.1975	0.3226	0.2705	0.0272	0.1092647	Mineral
DNA	CE5	14.5299	-1.2538	0.0396	-0.0342	0.4769	0.6333288	Mineral
DNA	CE7	10.4421	1.4941	0.3698	0.3214	0.0162	0.0827619	Mineral
DNA	CE6	37.5291	1.3406	0.0318	-0.0427	0.5251	0.6794412	Mineral
DNA	CE8	22.1825	1.8666	0.1442	0.0784	0.1626	0.3363344	Mineral
DNA	CE9	94.9446	-0.2784	0.0043	-0.0723	0.8161	0.8872349	Mineral
DNA	GH100	0.6919	0.2542	0.5195	0.4825	0.0024	0.035905	Mineral
DNA	GH10	53.2214	2.4056	0.1335	0.0668	0.1805	0.3625202	Mineral
DNA	GH102	43.2037	-0.2668	0.0063	-0.0701	0.7782	0.8566715	Mineral
DNA	GH101	0.8491	0.0966	0.0715	0.0001	0.3351	0.5007233	Mineral
DNA	GH103	113.5215	4.4677	0.0815	0.0108	0.3024	0.4685988	Mineral
DNA	GH104	5.515	0.0962	0.0091	-0.0671	0.7347	0.8232448	Mineral
DNA	GH105	25.0043	3.8456	0.2371	0.1784	0.0657	0.1851551	Mineral
DNA	GH106	43.4576	4.2584	0.1954	0.1335	0.099	0.2388898	Mineral
DNA	GH107	0.1988	0.0444	0.0942	0.0245	0.2658	0.4267742	Mineral
DNA	GH108	6.4325	0.0682	0.0086	-0.0676	0.7421	0.8278952	Mineral
DNA	GH109	19.7839	0.5459	0.0455	-0.0279	0.4453	0.5977126	Mineral
DNA	GH11	13.636	1.9907	0.4621	0.4207	0.0053	0.0429439	Mineral
DNA	GH110	1.0855	0.2398	0.4455	0.4028	0.0066	0.0514089	Mineral
DNA	GH111	0.0484	0.0065	0.0588	-0.0136	0.3837	0.547754	Mineral
DNA	GH112	0.065	0.0148	0.1006	0.0314	0.2494	0.4257141	Mineral
DNA	GH113	8.5535	0.7546	0.1551	0.0901	0.1464	0.3168692	Mineral
DNA	GH114	11.4339	-0.3532	0.0143	-0.0615	0.6709	0.776055	Mineral
DNA	GH115	15.0153	3.9634	0.5329	0.4969	0.002	0.035905	Mineral
DNA	GH116	40.527	-1.1941	0.0235	-0.0516	0.5851	0.726973	Mineral
DNA	GH117	3.5852	0.0349	0.0012	-0.0757	0.9045	0.924937	Mineral
DNA	GH118	0.0033	0.0002	0.0023	-0.0744	0.8646	0.9138647	Mineral
DNA	GH119	3.1764	0.1881	0.0299	-0.0447	0.5377	0.6885797	Mineral
DNA	GH12	8.1909	1.3178	0.2822	0.227	0.0416	0.1434581	Mineral
DNA	GH120	1.8095	-0.3211	0.0953	0.0258	0.2628	0.4267742	Mineral
DNA	GH121	1.9359	0.4163	0.0974	0.028	0.2574	0.4267742	Mineral
DNA	GH123	2.2624	-0.0294	0.0015	-0.0753	0.8914	0.9169357	Mineral
DNA	GH124	0.0624	-0.0125	0.1212	0.0535	0.2036	0.3842608	Mineral
DNA	GH125	10.2003	0.7369	0.0932	0.0235	0.2684	0.4267742	Mineral
DNA	GH126	0.0079	-0.0027	0.073	0.0017	0.3301	0.4997563	Mineral
DNA	GH127	40.8673	2.4706	0.1302	0.0633	0.1865	0.366078	Mineral
DNA	GH128	6.089	1.3821	0.4414	0.3985	0.0069	0.0524595	Mineral
DNA	GH129	0.3723	-0.0502	0.1079	0.0393	0.232	0.4189087	Mineral
DNA	GH13	1511.7028	16.7782	0.0176	-0.058	0.6377	0.7479102	Mineral
DNA	GH130	83.298	2.7038	0.0479	-0.0253	0.4331	0.5876201	Mineral

DNA	GH131	0.1722	0.0988	0.5802	0.5479	0.001	0.0303526	Mineral
DNA	GH132	0.6932	0.3488	0.4951	0.4563	0.0034	0.0368392	Mineral
DNA	GH133	77.1918	-8.5365	0.802	0.7867	0.00E+00	0.0016069	Mineral
DNA	GH14	0.4871	0.0605	0.3328	0.2815	0.0244	0.1047653	Mineral
DNA	GH17	62.1908	3.4251	0.144	0.0781	0.1631	0.3363344	Mineral
DNA	GH15	404.3419	-9.3253	0.0502	-0.0229	0.4223	0.579195	Mineral
DNA	GH18	240.9138	12.7981	0.1908	0.1286	0.1035	0.2427538	Mineral
DNA	GH19	17.4844	1.3542	0.1164	0.0485	0.2133	0.3964971	Mineral
DNA	GH2	363.2682	14.3109	0.1135	0.0453	0.2196	0.4022889	Mineral
DNA	GH20	98.8613	2.8522	0.1042	0.0353	0.2406	0.423337	Mineral
DNA	GH23	402.7243	6.1116	0.034	-0.0403	0.5104	0.6638015	Mineral
DNA	GH24	16.0614	-0.4534	0.0663	-0.0055	0.3541	0.5228344	Mineral
DNA	GH25	20.5589	1.056	0.049	-0.0242	0.4281	0.5839409	Mineral
DNA	GH26	21.4128	2.0571	0.185	0.1223	0.1095	0.2522586	Mineral
DNA	GH27	70.6768	6.7292	0.2029	0.1416	0.092	0.2286931	Mineral
DNA	GH28	177.5732	15.0735	0.3215	0.2693	0.0275	0.1092647	Mineral
DNA	GH29	104.4902	-1.8135	0.0216	-0.0537	0.6011	0.7360395	Mineral
DNA	GH3	550.2428	21.0363	0.1006	0.0314	0.2493	0.4257141	Mineral
DNA	GH30	48.4301	4.5671	0.3565	0.307	0.0188	0.0889296	Mineral
DNA	GH31	125.157	1.1346	0.0033	-0.0734	0.84	0.9010498	Mineral
DNA	GH33	63.7208	0.4855	0.0026	-0.0741	0.8555	0.913787	Mineral
DNA	GH36	81.126	3.865	0.0891	0.019	0.2798	0.4417359	Mineral
DNA	GH32	31.8356	-1.2579	0.2656	0.2091	0.0493	0.1565952	Mineral
DNA	GH37	36.4506	-1.4285	0.0361	-0.0381	0.4979	0.6509126	Mineral
DNA	GH39	81.1725	3.7571	0.1634	0.0991	0.135	0.2999623	Mineral
DNA	GH38	103.1959	8.0023	0.1667	0.1026	0.1308	0.2931884	Mineral
DNA	GH4	145.5656	11.3883	0.1933	0.1313	0.101	0.2391382	Mineral
DNA	GH42	42.3015	1.9139	0.1359	0.0694	0.1764	0.3570666	Mineral
DNA	GH43	105.7244	13.6104	0.224	0.1643	0.0747	0.1988356	Mineral
DNA	GH44	24.2141	2.5454	0.4978	0.4592	0.0033	0.0368392	Mineral
DNA	GH45	0.4034	0.1099	0.3651	0.3163	0.017	0.0835206	Mineral
DNA	GH47	15.3148	1.5408	0.307	0.2537	0.0321	0.1184287	Mineral
DNA	GH46	2.3583	0.3032	0.0532	-0.0196	0.4082	0.5660198	Mineral
DNA	GH48	9.3733	0.7499	0.1993	0.1377	0.0953	0.2344926	Mineral
DNA	GH49	0.174	0.1126	0.3273	0.2755	0.0259	0.1081494	Mineral
DNA	GH5	169.7639	12.8965	0.2212	0.1613	0.0769	0.2009914	Mineral
DNA	GH50	7.8367	-1.2097	0.3155	0.2628	0.0293	0.1107208	Mineral
DNA	GH51	100.2913	5.8564	0.1551	0.0901	0.1463	0.3168692	Mineral
DNA	GH54	16.7488	3.9274	0.2443	0.1862	0.0611	0.1762166	Mineral
DNA	GH53	54.6684	5.7287	0.4918	0.4527	0.0036	0.0368392	Mineral
DNA	GH55	98.5985	5.3971	0.0613	-0.0109	0.3738	0.5395237	Mineral
DNA	GH56	0.003	0.0011	0.0875	0.0173	0.2846	0.4438929	Mineral
DNA	GH57	155.9399	-7.4335	0.2615	0.2047	0.0514	0.1591467	Mineral
DNA	GH58	0.0426	0.0093	0.104	0.035	0.2412	0.423337	Mineral
DNA	GH59	6.4703	1.3245	0.1975	0.1358	0.097	0.2362849	Mineral
DNA	GH6	17.654	3.1504	0.3447	0.2943	0.0214	0.095822	Mineral
DNA	GH62	3.2335	0.4414	0.1021	0.033	0.2457	0.4257141	Mineral
DNA	GH63	14.1513	1.9374	0.2721	0.2161	0.0461	0.1484569	Mineral
DNA	GH64	18.7715	1.1066	0.0939	0.0242	0.2666	0.4267742	Mineral
DNA	GH65	100.9432	-5.3302	0.0462	-0.0272	0.4417	0.5960642	Mineral
DNA	GH66	1.5117	0.5309	0.4012	0.3551	0.0112	0.0648982	Mineral
DNA	GH68	0.2737	0.0216	0.13	0.0631	0.1867	0.366078	Mineral
DNA	GH7	0.661	0.3874	0.4236	0.3793	0.0086	0.0552961	Mineral
DNA	GH70	0.0411	0.0175	0.2724	0.2164	0.046	0.1484569	Mineral
DNA	GH71	3.5602	1.4011	0.6301	0.6016	0.0004	0.0206414	Mineral

DNA	GH72	30.0334	2.3967	0.4199	0.3752	0.009	0.0564523	Mineral
DNA	GH73	39.5823	0.7873	0.0368	-0.0372	0.4931	0.6480191	Mineral
DNA	GH75	2.2715	0.2142	0.1082	0.0396	0.2313	0.4189087	Mineral
DNA	GH74	12.8907	1.5927	0.3857	0.3384	0.0135	0.0735543	Mineral
DNA	GH76	13.3927	2.046	0.2094	0.1486	0.0863	0.216696	Mineral
DNA	GH78	148.9518	10.017	0.3174	0.2649	0.0287	0.1107208	Mineral
DNA	GH79	25.909	-0.3772	0.005	-0.0715	0.8016	0.8785735	Mineral
DNA	GH8	19.2424	1.124	0.0441	-0.0294	0.4524	0.6040409	Mineral
DNA	GH80	0.0028	-0.0039	0.2257	0.1661	0.0735	0.1984817	Mineral
DNA	GH81	6.0259	1.1063	0.3466	0.2963	0.0209	0.0955872	Mineral
DNA	GH84	4.7774	0.4153	0.2272	0.1678	0.0724	0.1984817	Mineral
DNA	GH85	0.6451	0.0441	0.0217	-0.0536	0.6005	0.7360395	Mineral
DNA	GH86	1.7515	0.1169	0.0695	-0.0021	0.3426	0.5088244	Mineral
DNA	GH87	64.8133	5.7015	0.4625	0.4212	0.0053	0.0429439	Mineral
DNA	GH88	24.9678	0.8007	0.1236	0.0562	0.1987	0.3837826	Mineral
DNA	GH89	6.8897	1.2131	0.2909	0.2364	0.038	0.1361778	Mineral
DNA	GH91	0.106	0.0187	0.0612	-0.011	0.374	0.5395237	Mineral
DNA	GH90	0.0045	0.0031	0.2581	0.2011	0.0531	0.1607281	Mineral
DNA	GH9	51.5097	1.8546	0.02	-0.0554	0.615	0.7402697	Mineral
DNA	GH92	91.0695	9.9845	0.1362	0.0698	0.1758	0.3570666	Mineral
DNA	GH93	22.1339	7.5304	0.3397	0.2889	0.0226	0.0994476	Mineral
DNA	GH94	27.0425	-0.8083	0.0187	-0.0568	0.6268	0.7456787	Mineral
DNA	GH95	48.6912	4.6824	0.2583	0.2013	0.053	0.1607281	Mineral
DNA	GH96	0.5474	0.0557	0.02	-0.0554	0.6155	0.7402697	Mineral
DNA	GH97	32.6174	0.7242	0.0113	-0.0647	0.7057	0.8051857	Mineral
DNA	GH98	0.3397	0.0108	0.0179	-0.0577	0.6348	0.7479102	Mineral
DNA	GH99	13.1032	0.5698	0.0388	-0.0351	0.4815	0.6361205	Mineral
DNA	GT1	153.6974	9.9788	0.0964	0.0269	0.26	0.4267742	Mineral
DNA	GT10	2.4352	0.2511	0.353	0.3032	0.0195	0.0906939	Mineral
DNA	GT12	0.0482	-0.0028	0.0182	-0.0573	0.6315	0.7477067	Mineral
DNA	GT14	2.0647	0.0588	0.0116	-0.0644	0.7022	0.8048435	Mineral
DNA	GT11	4.9506	-0.0456	0.0033	-0.0734	0.839	0.9010498	Mineral
DNA	GT13	0.1413	0.0398	0.2161	0.1558	0.0808	0.2058474	Mineral
DNA	GT15	1.84	0.8102	0.5381	0.5025	0.0019	0.035905	Mineral
DNA	GT16	0.0336	0.0203	0.4278	0.3837	0.0082	0.0539605	Mineral
DNA	GT17	1.0318	0.0588	0.0874	0.0172	0.2847	0.4438929	Mineral
DNA	GT18	0.0082	0.0045	0.226	0.1665	0.0733	0.1984817	Mineral
DNA	GT2	1841.616	11.7971	0.0301	-0.0445	0.5365	0.6885797	Mineral
DNA	GT19	83.4375	-0.3759	0.0068	-0.0696	0.77	0.854192	Mineral
DNA	GT20	150.7734	-2.6869	0.2386	0.1801	0.0647	0.1844046	Mineral
DNA	GT21	72.0818	-4.4398	0.0738	0.0025	0.3275	0.4997563	Mineral
DNA	GT22	4.3521	0.8834	0.4701	0.4293	0.0048	0.0428387	Mineral
DNA	GT23	0.2031	0.0386	0.248	0.1901	0.0589	0.1738629	Mineral
DNA	GT24	2.657	0.5442	0.4039	0.3581	0.0109	0.0648982	Mineral
DNA	GT25	5.9103	0.4792	0.2325	0.1735	0.0687	0.1916261	Mineral
DNA	GT26	133.0274	8.1673	0.6977	0.6745	0.0001	0.0099463	Mineral
DNA	GT27	13.0687	0.47	0.1312	0.0644	0.1846	0.366078	Mineral
DNA	GT28	177.3669	1.4045	0.0294	-0.0453	0.5414	0.6887102	Mineral
DNA	GT29	3.9302	0.0275	0.0021	-0.0747	0.8722	0.9138647	Mineral
DNA	GT30	120.1726	0.2468	0.0016	-0.0752	0.8881	0.9169357	Mineral
DNA	GT3	2.7042	0.6622	0.4366	0.3933	0.0073	0.0539605	Mineral
DNA	GT33	1.8007	0.3225	0.3946	0.348	0.0122	0.0678127	Mineral
DNA	GT34	0.89	0.3306	0.4292	0.3852	0.008	0.0539605	Mineral
DNA	GT32	12.395	1.2168	0.3744	0.3262	0.0153	0.0802657	Mineral
DNA	GT31	1.3616	0.6794	0.5823	0.5502	0.0009	0.0303526	Mineral

DNA	GT35	348.7674	-27.774	0.4322	0.3885	0.0077	0.0539605	Mineral
DNA	GT37	0.0045	0.0024	0.2772	0.2216	0.0438	0.1445112	Mineral
DNA	GT39	20.8559	2.887	0.2639	0.2073	0.0501	0.157238	Mineral
DNA	GT4	1887.0784	46.4148	0.0937	0.024	0.2671	0.4267742	Mineral
DNA	GT41	420.3074	-20.4504	0.2775	0.222	0.0436	0.1445112	Mineral
DNA	GT40	0.0012	-0.0011	0.1886	0.1262	0.1057	0.2457407	Mineral
DNA	GT42	0.0617	0.037	0.4983	0.4597	0.0033	0.0368392	Mineral
DNA	GT43	0.0665	0.0182	0.1234	0.056	0.1991	0.3837826	Mineral
DNA	GT47	67.1447	7.1239	0.3024	0.2487	0.0337	0.1225855	Mineral
DNA	GT45	0.2617	-0.0018	0.0011	-0.0757	0.9065	0.924937	Mineral
DNA	GT44	0.0225	-0.0051	0.0582	-0.0142	0.3863	0.5478601	Mineral
DNA	GT48	3.0789	1.2972	0.5306	0.4945	0.0021	0.035905	Mineral
DNA	GT49	0.176	0.0639	0.4002	0.3541	0.0114	0.0648982	Mineral
DNA	GT5	111.8839	-0.3652	0.0021	-0.0746	0.8703	0.9138647	Mineral
DNA	GT50	0.6419	0.2691	0.5264	0.49	0.0022	0.035905	Mineral
DNA	GT53	23.5174	-1.2802	0.0217	-0.0535	0.6	0.7360395	Mineral
DNA	GT52	0	0	#VALUE!	#VALUE!	#VALUE!	NA	Mineral
DNA	GT51	615.404	6.4488	0.0154	-0.0604	0.6599	0.7668818	Mineral
DNA	GT54	0.0278	0.0169	0.3651	0.3163	0.017	0.0835206	Mineral
DNA	GT57	1.3329	0.5907	0.4969	0.4582	0.0033	0.0368392	Mineral
DNA	GT56	0.3995	0.0243	0.1063	0.0376	0.2356	0.4224586	Mineral
DNA	GT55	2.8539	0.5579	0.3148	0.2621	0.0296	0.1107208	Mineral
DNA	GT58	0.5128	0.211	0.5054	0.4674	0.003	0.0368392	Mineral
DNA	GT6	0.334	-0.0673	0.1697	0.1059	0.127	0.2872464	Mineral
DNA	GT59	0.3598	0.1684	0.5343	0.4985	0.002	0.035905	Mineral
DNA	GT60	1.8074	0.2698	0.1137	0.0456	0.219	0.4022889	Mineral
DNA	GT61	0.3572	0.0689	0.251	0.1933	0.0571	0.1707589	Mineral
DNA	GT62	2.1958	0.3722	0.4298	0.3859	0.008	0.0539605	Mineral
DNA	GT64	0.9005	0.1025	0.2156	0.1553	0.0812	0.2058474	Mineral
DNA	GT65	0.0411	0.0062	0.0726	0.0013	0.3313	0.4997563	Mineral
DNA	GT66	19.8555	0.3987	0.019	-0.0565	0.6245	0.7456787	Mineral
DNA	GT69	0.7751	0.3956	0.535	0.4992	0.0019	0.035905	Mineral
DNA	GT68	0.0223	0.0051	0.1228	0.0554	0.2003	0.3837826	Mineral
DNA	GT7	5.4876	-0.0146	0.0005	-0.0764	0.935	0.9463261	Mineral
DNA	GT72	0.0008	0.0002	0.0067	-0.0698	0.7725	0.854192	Mineral
DNA	GT73	0.0663	0.0308	0.2818	0.2265	0.0418	0.1434581	Mineral
DNA	GT71	1.4059	0.2413	0.3203	0.268	0.0279	0.1092647	Mineral
DNA	GT70	0.7826	0.0905	0.3798	0.3321	0.0144	0.0770356	Mineral
DNA	GT74	0.8924	-0.0877	0.0932	0.0234	0.2686	0.4267742	Mineral
DNA	GT75	0.9563	-0.031	0.0291	-0.0456	0.5433	0.6887102	Mineral
DNA	GT76	2.3528	-0.0754	0.0161	-0.0595	0.6519	0.7610441	Mineral
DNA	GT77	0.9469	-0.0614	0.0983	0.0289	0.2552	0.4267742	Mineral
DNA	GT78	0.0018	-0.0011	0.0638	-0.0082	0.3638	0.5309362	Mineral
DNA	GT8	11.7518	1.3536	0.2805	0.2252	0.0423	0.1434581	Mineral
DNA	GT80	5.6377	0.3222	0.1554	0.0905	0.1459	0.3168692	Mineral
DNA	GT82	0.4951	0.0067	0.002	-0.0748	0.8738	0.9138647	Mineral
DNA	GT84	296.2703	1.4059	0.0006	-0.0763	0.9324	0.9463261	Mineral
DNA	GT81	24.419	0.5342	0.0247	-0.0503	0.5756	0.7187258	Mineral
DNA	GT83	171.2538	0.6605	0.0023	-0.0744	0.8646	0.9138647	Mineral
DNA	GT85	3.2912	-0.1883	0.02	-0.0554	0.6153	0.7402697	Mineral
DNA	GT87	16.9111	1.7298	0.1485	0.083	0.156	0.3318319	Mineral
DNA	GT88	0.0259	-0.0009	0.0015	-0.0753	0.8911	0.9169357	Mineral
DNA	GT89	5.7924	-0.0114	0.00E+00	-0.0769	0.9806	0.9845511	Mineral
DNA	GT90	2.475	0.8483	0.4986	0.46	0.0033	0.0368392	Mineral
DNA	GT9	175.0926	1.5933	0.0106	-0.0655	0.7147	0.8116615	Mineral

DNA	GT91	0.0159	0.0073	0.1705	0.1067	0.1261	0.2872464	Mineral
DNA	GT93	0.368	0.0141	0.0989	0.0296	0.2536	0.4267742	Mineral
DNA	GT94	0.7572	0.0217	0.0094	-0.0668	0.7308	0.8232448	Mineral
DNA	GT92	0.0971	0.0521	0.5859	0.554	0.0009	0.0303526	Mineral
DNA	GT95	7.0408	-0.6577	0.6331	0.6049	0.0004	0.0206414	Mineral
DNA	PL1	18.5567	1.0492	0.0199	-0.0555	0.6164	0.7402697	Mineral
DNA	PL10	4.7494	0.8047	0.1219	0.0544	0.2021	0.3842608	Mineral
DNA	PL11	3.638	0.1159	0.0043	-0.0723	0.8165	0.8872349	Mineral
DNA	PL12	23.2607	0.3419	0.0131	-0.0628	0.6849	0.7886014	Mineral
DNA	PL13	0.0005	0.001	0.3318	0.2804	0.0246	0.1047653	Mineral
DNA	PL14	1.1325	0.3484	0.3638	0.3148	0.0173	0.0835206	Mineral
DNA	PL15	0.3857	0.0593	0.0738	0.0025	0.3274	0.4997563	Mineral
DNA	PL17	1.8343	0.2369	0.0566	-0.016	0.3934	0.5516534	Mineral
DNA	PL18	0.0042	-0.0019	0.1437	0.0778	0.1635	0.3363344	Mineral
DNA	PL16	0.52	-0.0426	0.1171	0.0492	0.2119	0.3964971	Mineral
DNA	PL2	0.1389	-0.0021	0.0018	-0.0749	0.8795	0.9159737	Mineral
DNA	PL20	0.0975	0.0215	0.065	-0.0069	0.3591	0.5271612	Mineral
DNA	PL21	0.4953	-0.0837	0.2188	0.1587	0.0787	0.2036744	Mineral
DNA	PL22	9.2334	1.3769	0.6925	0.6689	0.0001	0.0099463	Mineral
DNA	PL23	0.0157	-0.0013	0.0279	-0.0468	0.5516	0.6957086	Mineral
DNA	PL3	0.3266	0.0986	0.0723	0.0009	0.3325	0.4997563	Mineral
DNA	PL4	15.4469	2.998	0.4842	0.4446	0.004	0.0368392	Mineral
DNA	PL5	1.6555	0.3944	0.1008	0.0317	0.2488	0.4257141	Mineral
DNA	PL6	2.3184	0.1393	0.0092	-0.067	0.7339	0.8232448	Mineral
DNA	PL7	3.659	0.8669	0.2233	0.1636	0.0753	0.1988356	Mineral
DNA	PL8	2.5006	0.4363	0.194	0.132	0.1003	0.2391382	Mineral
DNA	PL9	33.1358	2.3829	0.0588	-0.0137	0.3841	0.547754	Mineral
Water_content	AA1	161.1286	9.1214	0.2508	0.1932	0.0572	0.1915375	Mineral
Water_content	AA11	0.406	0.1242	0.1609	0.0963	0.1384	0.3352856	Mineral
Water_content	AA10	6.1103	0.9547	0.2827	0.2276	0.0414	0.1643397	Mineral
Water_content	AA2	1.4848	0.3152	0.2936	0.2392	0.037	0.1546003	Mineral
Water_content	AA3	426.2452	-13.1808	0.0781	0.0072	0.3132	0.5541405	Mineral
Water_content	AA4	11.2554	3.5952	0.752	0.7329	0.00E+00	0.0023792	Mineral
Water_content	AA5	53.5482	-4.5783	0.3313	0.2799	0.0248	0.1374694	Mineral
Water_content	AA8	0.6569	0.1192	0.1356	0.0691	0.1769	0.3996689	Mineral
Water_content	AA7	57.2187	-1.3723	0.0219	-0.0533	0.5985	0.8048353	Mineral
Water_content	AA6	30.8574	-2.5447	0.3968	0.3503	0.0118	0.1003651	Mineral
Water_content	AA9	1.4632	0.3534	0.0712	-0.0003	0.3365	0.5789737	Mineral
Water_content	CE1	216.8049	-2.0174	0.0193	-0.0561	0.6212	0.8136383	Mineral
Water_content	CE11	163.2288	1.3833	0.0204	-0.0549	0.6114	0.8119153	Mineral
Water_content	CE12	15.6874	2.2972	0.2798	0.2244	0.0426	0.1643397	Mineral
Water_content	CE13	0.0362	0.004	0.0233	-0.0518	0.5868	0.8030023	Mineral
Water_content	CE14	221.0157	4.2334	0.1171	0.0492	0.2118	0.4424513	Mineral
Water_content	CE15	37.8269	4.4682	0.439	0.3958	0.0071	0.0714059	Mineral
Water_content	CE2	2.5005	0.4452	0.1102	0.0417	0.2268	0.4590684	Mineral
Water_content	CE3	26.5046	-0.839	0.0053	-0.0712	0.7965	0.9087083	Mineral
Water_content	CE4	378.0703	-16.4168	0.1198	0.0521	0.2063	0.4424513	Mineral
Water_content	CE16	1.0559	0.1955	0.3162	0.2636	0.0291	0.1407211	Mineral
Water_content	CE5	14.5299	-2.0147	0.1023	0.0333	0.2451	0.4806044	Mineral
Water_content	CE7	10.4421	1.0083	0.1684	0.1045	0.1287	0.3261932	Mineral
Water_content	CE6	37.5291	4.2536	0.3197	0.2674	0.028	0.1407211	Mineral
Water_content	CE8	22.1825	1.4889	0.0918	0.0219	0.2724	0.5194892	Mineral
Water_content	CE9	94.9446	-0.2138	0.0025	-0.0742	0.8583	0.927046	Mineral
Water_content	GH100	0.6919	0.1889	0.2867	0.2318	0.0397	0.1631898	Mineral
Water_content	GH10	53.2214	2.214	0.1131	0.0448	0.2204	0.4498238	Mineral

Water_content	GH102	43.2037	-1.2699	0.1432	0.0773	0.1642	0.3746711	Mineral
Water_content	GH101	0.8491	-0.058	0.0258	-0.0491	0.5674	0.8030023	Mineral
Water_content	GH103	113.5215	-3.4383	0.0483	-0.025	0.4315	0.7033043	Mineral
Water_content	GH104	5.515	0.0233	0.0005	-0.0763	0.9347	0.958887	Mineral
Water_content	GH105	25.0043	4.6733	0.3501	0.3002	0.0201	0.1238771	Mineral
Water_content	GH106	43.4576	5.6555	0.3447	0.2942	0.0214	0.1277692	Mineral
Water_content	GH107	0.1988	-0.012	0.0069	-0.0695	0.7688	0.8933677	Mineral
Water_content	GH108	6.4325	0.1604	0.0476	-0.0256	0.4346	0.703756	Mineral
Water_content	GH109	19.7839	0.4302	0.0283	-0.0465	0.5493	0.7975448	Mineral
Water_content	GH11	13.636	1.7647	0.3631	0.3141	0.0174	0.1164784	Mineral
Water_content	GH110	1.0855	0.183	0.2593	0.2023	0.0525	0.185503	Mineral
Water_content	GH111	0.0484	-0.0014	0.0028	-0.0739	0.8512	0.927046	Mineral
Water_content	GH112	0.065	0.0207	0.1973	0.1355	0.0972	0.2741365	Mineral
Water_content	GH113	8.5535	0.0943	0.0024	-0.0743	0.8618	0.927046	Mineral
Water_content	GH114	11.4339	-1.5059	0.2604	0.2035	0.052	0.185503	Mineral
Water_content	GH115	15.0153	3.4154	0.3957	0.3492	0.012	0.1003651	Mineral
Water_content	GH116	40.527	3.3458	0.1849	0.1222	0.1097	0.2990114	Mineral
Water_content	GH117	3.5852	0.0456	0.002	-0.0748	0.8756	0.927046	Mineral
Water_content	GH118	0.0033	-0.0004	0.0139	-0.062	0.676	0.8358636	Mineral
Water_content	GH119	3.1764	-0.1199	0.0121	-0.0638	0.6958	0.8484387	Mineral
Water_content	GH12	8.1909	0.9627	0.1506	0.0852	0.1529	0.3554056	Mineral
Water_content	GH120	1.8095	-0.0405	0.0015	-0.0753	0.8904	0.9311959	Mineral
Water_content	GH121	1.9359	-0.1195	0.008	-0.0683	0.7509	0.8814588	Mineral
Water_content	GH123	2.2624	-0.3223	0.1785	0.1153	0.1166	0.3049582	Mineral
Water_content	GH124	0.0624	-0.0161	0.201	0.1396	0.0937	0.2682873	Mineral
Water_content	GH125	10.2003	1.225	0.2576	0.2005	0.0534	0.185503	Mineral
Water_content	GH126	0.0079	-0.0005	0.0026	-0.0742	0.8576	0.927046	Mineral
Water_content	GH127	40.8673	3.2846	0.2301	0.1708	0.0704	0.2209079	Mineral
Water_content	GH128	6.089	1.0274	0.2439	0.1858	0.0613	0.1998949	Mineral
Water_content	GH129	0.3723	-0.0158	0.0107	-0.0654	0.7134	0.8567603	Mineral
Water_content	GH13	1511.7028	-10.1792	0.0065	-0.07	0.7757	0.8970703	Mineral
Water_content	GH130	83.298	7.304	0.3497	0.2997	0.0202	0.1238771	Mineral
Water_content	GH131	0.1722	0.0717	0.3057	0.2523	0.0325	0.1485262	Mineral
Water_content	GH132	0.6932	0.2055	0.172	0.1083	0.1243	0.3216611	Mineral
Water_content	GH133	77.1918	-5.8019	0.3705	0.322	0.016	0.1119253	Mineral
Water_content	GH14	0.4871	0.0363	0.1201	0.0524	0.2057	0.4424513	Mineral
Water_content	GH17	62.1908	-1.2229	0.0184	-0.0572	0.6302	0.8136383	Mineral
Water_content	GH15	404.3419	-1.5305	0.0014	-0.0755	0.8965	0.9337171	Mineral
Water_content	GH18	240.9138	8.626	0.0867	0.0164	0.2867	0.5253528	Mineral
Water_content	GH19	17.4844	0.3755	0.009	-0.0673	0.7373	0.8770639	Mineral
Water_content	GH2	363.2682	22.3678	0.2772	0.2216	0.0438	0.1643397	Mineral
Water_content	GH20	98.8613	3.631	0.1689	0.105	0.1281	0.3261932	Mineral
Water_content	GH23	402.7243	-2.3595	0.0051	-0.0715	0.8009	0.9095774	Mineral
Water_content	GH24	16.0614	0.0841	0.0023	-0.0745	0.8657	0.927046	Mineral
Water_content	GH25	20.5589	-0.3222	0.0046	-0.072	0.8111	0.9170321	Mineral
Water_content	GH26	21.4128	0.8201	0.0294	-0.0453	0.5412	0.7975448	Mineral
Water_content	GH27	70.6768	8.5201	0.3252	0.2733	0.0264	0.1382299	Mineral
Water_content	GH28	177.5732	20.0253	0.5675	0.5342	0.0012	0.0212465	Mineral
Water_content	GH29	104.4902	4.9402	0.1603	0.0958	0.1391	0.3352856	Mineral
Water_content	GH3	550.2428	37.014	0.3115	0.2585	0.0306	0.1449673	Mineral
Water_content	GH30	48.4301	4.1514	0.2945	0.2403	0.0366	0.1546003	Mineral
Water_content	GH31	125.157	10.4435	0.2755	0.2198	0.0445	0.1643397	Mineral
Water_content	GH33	63.7208	-1.8827	0.0398	-0.0341	0.476	0.7467141	Mineral
Water_content	GH36	81.126	7.5784	0.3426	0.292	0.0219	0.1277692	Mineral
Water_content	GH32	31.8356	-0.3795	0.0242	-0.0509	0.5801	0.8030023	Mineral

Water_content	GH37	36.4506	2.9746	0.1563	0.0914	0.1447	0.3425535	Mineral
Water_content	GH39	81.1725	7.272	0.6122	0.5824	0.0006	0.0141798	Mineral
Water_content	GH38	103.1959	13.6556	0.4854	0.4458	0.0039	0.0508641	Mineral
Water_content	GH4	145.5656	-1.7056	0.0043	-0.0723	0.8156	0.9180378	Mineral
Water_content	GH42	42.3015	2.9191	0.3161	0.2635	0.0292	0.1407211	Mineral
Water_content	GH43	105.7244	10.5551	0.1347	0.0682	0.1783	0.3996689	Mineral
Water_content	GH44	24.2141	3.0596	0.7192	0.6976	1.00E-04	0.0032562	Mineral
Water_content	GH45	0.4034	0.0819	0.2025	0.1412	0.0923	0.2682873	Mineral
Water_content	GH47	15.3148	0.7765	0.078	0.0071	0.3135	0.5541405	Mineral
Water_content	GH46	2.3583	-0.1939	0.0218	-0.0535	0.5997	0.8048353	Mineral
Water_content	GH48	9.3733	0.2458	0.0214	-0.0539	0.6028	0.8048353	Mineral
Water_content	GH49	0.174	0.0077	0.0015	-0.0753	0.8895	0.9311959	Mineral
Water_content	GH5	169.7639	4.2	0.0235	-0.0517	0.5858	0.8030023	Mineral
Water_content	GH50	7.8367	-1.1335	0.277	0.2214	0.0439	0.1643397	Mineral
Water_content	GH51	100.2913	10.2233	0.4727	0.4322	0.0046	0.0526823	Mineral
Water_content	GH54	16.7488	4.8742	0.3763	0.3284	0.015	0.1119253	Mineral
Water_content	GH53	54.6684	6.8121	0.6954	0.672	0.0001	0.0046683	Mineral
Water_content	GH55	98.5985	11.644	0.2852	0.2302	0.0403	0.1631898	Mineral
Water_content	GH56	0.003	0.0011	0.1004	0.0312	0.25	0.4863709	Mineral
Water_content	GH57	155.9399	-1.3077	0.0081	-0.0682	0.7498	0.8814588	Mineral
Water_content	GH58	0.0426	0.00E+00	0.00E+00	-0.0769	0.9963	0.9963372	Mineral
Water_content	GH59	6.4703	1.4336	0.2314	0.1723	0.0695	0.2207787	Mineral
Water_content	GH6	17.654	1.8183	0.1148	0.0467	0.2166	0.4456841	Mineral
Water_content	GH62	3.2335	0.0454	0.0011	-0.0758	0.9074	0.9411451	Mineral
Water_content	GH63	14.1513	1.0482	0.0796	0.0088	0.3082	0.5541405	Mineral
Water_content	GH64	18.7715	0.7809	0.0468	-0.0266	0.4389	0.7061698	Mineral
Water_content	GH65	100.9432	-4.7152	0.0362	-0.038	0.4973	0.764843	Mineral
Water_content	GH66	1.5117	0.0732	0.0076	-0.0687	0.7571	0.8838409	Mineral
Water_content	GH68	0.2737	0.018	0.0903	0.0203	0.2765	0.5194892	Mineral
Water_content	GH7	0.661	0.2338	0.1543	0.0893	0.1475	0.3459815	Mineral
Water_content	GH70	0.0411	0.0061	0.0326	-0.0418	0.5193	0.7852644	Mineral
Water_content	GH71	3.5602	0.8943	0.2567	0.1995	0.054	0.185503	Mineral
Water_content	GH72	30.0334	1.2051	0.1062	0.0374	0.236	0.470976	Mineral
Water_content	GH73	39.5823	-0.0778	0.0004	-0.0765	0.9465	0.9639129	Mineral
Water_content	GH75	2.2715	0.0717	0.0121	-0.0639	0.6963	0.8484387	Mineral
Water_content	GH74	12.8907	0.7673	0.0895	0.0195	0.2787	0.5194892	Mineral
Water_content	GH76	13.3927	1.1783	0.0695	-0.0021	0.3426	0.5810092	Mineral
Water_content	GH78	148.9518	8.2089	0.2132	0.1527	0.0832	0.2515184	Mineral
Water_content	GH79	25.909	1.8838	0.1256	0.0584	0.1949	0.4291259	Mineral
Water_content	GH8	19.2424	0.3939	0.0054	-0.0711	0.7943	0.9087083	Mineral
Water_content	GH80	0.0028	-0.0026	0.1039	0.035	0.2412	0.4767956	Mineral
Water_content	GH81	6.0259	0.2587	0.019	-0.0565	0.6246	0.8136383	Mineral
Water_content	GH84	4.7774	0.2836	0.106	0.0372	0.2364	0.470976	Mineral
Water_content	GH85	0.6451	-0.0148	0.0024	-0.0743	0.8616	0.927046	Mineral
Water_content	GH86	1.7515	-0.0566	0.0163	-0.0594	0.6505	0.8138708	Mineral
Water_content	GH87	64.8133	5.0445	0.3621	0.313	0.0176	0.1164784	Mineral
Water_content	GH88	24.9678	-0.0058	0.00E+00	-0.0769	0.9928	0.9963372	Mineral
Water_content	GH89	6.8897	1.2966	0.3324	0.281	0.0245	0.1374694	Mineral
Water_content	GH91	0.106	-0.0176	0.0542	-0.0186	0.4037	0.6666394	Mineral
Water_content	GH90	0.0045	-1.00E-04	0.0003	-0.0766	0.9546	0.9660976	Mineral
Water_content	GH9	51.5097	2.0013	0.0233	-0.0518	0.587	0.8030023	Mineral
Water_content	GH92	91.0695	8.0494	0.0885	0.0184	0.2815	0.5194892	Mineral
Water_content	GH93	22.1339	2.4493	0.0359	-0.0382	0.4986	0.764843	Mineral
Water_content	GH94	27.0425	2.6466	0.2006	0.1391	0.0941	0.2682873	Mineral
Water_content	GH95	48.6912	2.7807	0.0911	0.0212	0.2742	0.5194892	Mineral

Water_content	GH96	0.5474	-0.0766	0.0377	-0.0363	0.4879	0.7606458	Mineral
Water_content	GH97	32.6174	-0.7329	0.0116	-0.0644	0.7023	0.851299	Mineral
Water_content	GH98	0.3397	-0.0275	0.1164	0.0484	0.2133	0.4424513	Mineral
Water_content	GH99	13.1032	-0.4878	0.0285	-0.0463	0.5479	0.7975448	Mineral
Water_content	GT1	153.6974	-3.9543	0.0151	-0.0606	0.6622	0.8228364	Mineral
Water_content	GT10	2.4352	0.3742	0.7842	0.7676	0.00E+00	0.0022455	Mineral
Water_content	GT12	0.0482	0.0022	0.0114	-0.0647	0.7055	0.851299	Mineral
Water_content	GT14	2.0647	0.0998	0.0335	-0.0409	0.514	0.7819569	Mineral
Water_content	GT11	4.9506	0.4316	0.2958	0.2416	0.0361	0.1546003	Mineral
Water_content	GT13	0.1413	0.0571	0.4453	0.4026	0.0066	0.0687059	Mineral
Water_content	GT15	1.84	0.5258	0.2266	0.1671	0.0728	0.22574	Mineral
Water_content	GT16	0.0336	0.0272	0.7687	0.7509	0.00E+00	0.0022455	Mineral
Water_content	GT17	1.0318	0.0927	0.2176	0.1574	0.0796	0.2437215	Mineral
Water_content	GT18	0.0082	0.0054	0.3297	0.2781	0.0252	0.1374694	Mineral
Water_content	GT2	1841.616	2.9715	0.0019	-0.0749	0.8771	0.927046	Mineral
Water_content	GT19	83.4375	1.5791	0.1201	0.0524	0.2057	0.4424513	Mineral
Water_content	GT20	150.7734	-1.5212	0.0765	0.0054	0.3184	0.5587992	Mineral
Water_content	GT21	72.0818	4.3566	0.071	-0.0004	0.337	0.5789737	Mineral
Water_content	GT22	4.3521	0.4638	0.1296	0.0626	0.1875	0.4165626	Mineral
Water_content	GT23	0.2031	0.0585	0.5686	0.5354	0.0012	0.0212465	Mineral
Water_content	GT24	2.657	0.5212	0.3704	0.322	0.0161	0.1119253	Mineral
Water_content	GT25	5.9103	0.7601	0.5849	0.553	0.0009	0.0187281	Mineral
Water_content	GT26	133.0274	6.7663	0.4789	0.4388	0.0043	0.0508641	Mineral
Water_content	GT27	13.0687	0.9397	0.5243	0.4878	0.0023	0.0355908	Mineral
Water_content	GT28	177.3669	2.5783	0.099	0.0297	0.2534	0.4892544	Mineral
Water_content	GT29	3.9302	-0.2082	0.1183	0.0504	0.2095	0.4424513	Mineral
Water_content	GT30	120.1726	2.4999	0.1624	0.098	0.1363	0.3352856	Mineral
Water_content	GT3	2.7042	0.4856	0.2347	0.1759	0.0672	0.2162706	Mineral
Water_content	GT33	1.8007	0.0783	0.0232	-0.0519	0.5876	0.8030023	Mineral
Water_content	GT34	0.89	0.2016	0.1595	0.0949	0.1403	0.3352856	Mineral
Water_content	GT32	12.395	0.2655	0.0178	-0.0577	0.6352	0.8136383	Mineral
Water_content	GT31	1.3616	0.5268	0.3502	0.3002	0.0201	0.1238771	Mineral
Water_content	GT35	348.7674	-7.0113	0.0275	-0.0473	0.5544	0.7975448	Mineral
Water_content	GT37	0.0045	0.0021	0.2009	0.1394	0.0939	0.2682873	Mineral
Water_content	GT39	20.8559	0.7542	0.018	-0.0575	0.6335	0.8136383	Mineral
Water_content	GT4	1887.0784	-6.8095	0.002	-0.0748	0.8737	0.927046	Mineral
Water_content	GT41	420.3074	3.7923	0.0095	-0.0666	0.7291	0.8714079	Mineral
Water_content	GT40	0.0012	-0.0005	0.0358	-0.0384	0.4997	0.764843	Mineral
Water_content	GT42	0.0617	0.0424	0.6574	0.631	0.0002	0.0088024	Mineral
Water_content	GT43	0.0665	0.0409	0.6269	0.5982	0.0004	0.013667	Mineral
Water_content	GT47	67.1447	5.7301	0.1956	0.1338	0.0988	0.2754569	Mineral
Water_content	GT45	0.2617	-0.0089	0.0255	-0.0495	0.5699	0.8030023	Mineral
Water_content	GT44	0.0225	-0.0081	0.149	0.0835	0.1553	0.3575762	Mineral
Water_content	GT48	3.0789	1.1607	0.4248	0.3806	0.0085	0.0817532	Mineral
Water_content	GT49	0.176	0.0795	0.6196	0.5903	0.0005	0.0138474	Mineral
Water_content	GT5	111.8839	2.1256	0.0721	0.0007	0.3333	0.5789737	Mineral
Water_content	GT50	0.6419	0.1898	0.2618	0.2051	0.0512	0.185503	Mineral
Water_content	GT53	23.5174	-2.2664	0.0682	-0.0035	0.3473	0.5850757	Mineral
Water_content	GT52	0	0	#VALUE!	#VALUE!	#VALUE!	NA	Mineral
Water_content	GT51	615.404	-11.0269	0.0449	-0.0286	0.4484	0.7168514	Mineral
Water_content	GT54	0.0278	0.0208	0.5483	0.5136	0.0016	0.0266477	Mineral
Water_content	GT57	1.3329	0.472	0.3173	0.2648	0.0288	0.1407211	Mineral
Water_content	GT56	0.3995	0.041	0.3027	0.2491	0.0336	0.1505132	Mineral
Water_content	GT55	2.8539	0.452	0.2067	0.1456	0.0887	0.2649682	Mineral
Water_content	GT58	0.5128	0.1473	0.2465	0.1885	0.0598	0.1973984	Mineral

Water_content	GT6	0.334	0.0248	0.0231	-0.052	0.5887	0.8030023	Mineral
Water_content	GT59	0.3598	0.1157	0.2523	0.1948	0.0564	0.1912913	Mineral
Water_content	GT60	1.8074	0.5671	0.5025	0.4643	0.0031	0.0455862	Mineral
Water_content	GT61	0.3572	0.0593	0.1856	0.1229	0.109	0.2990114	Mineral
Water_content	GT62	2.1958	0.2299	0.1639	0.0996	0.1344	0.3339477	Mineral
Water_content	GT64	0.9005	0.0326	0.0218	-0.0534	0.5995	0.8048353	Mineral
Water_content	GT65	0.0411	0.0126	0.2967	0.2426	0.0358	0.1546003	Mineral
Water_content	GT66	19.8555	-0.2292	0.0063	-0.0702	0.7791	0.8970703	Mineral
Water_content	GT69	0.7751	0.3295	0.3712	0.3228	0.0159	0.1119253	Mineral
Water_content	GT68	0.0223	0.0093	0.4113	0.366	0.01	0.0927321	Mineral
Water_content	GT7	5.4876	0.3512	0.309	0.2558	0.0314	0.1460788	Mineral
Water_content	GT72	0.0008	0.0005	0.0704	-0.0011	0.3391	0.5789737	Mineral
Water_content	GT73	0.0663	0.0236	0.1649	0.1007	0.1331	0.3339477	Mineral
Water_content	GT71	1.4059	0.1272	0.0891	0.019	0.2799	0.5194892	Mineral
Water_content	GT70	0.7826	0.0901	0.3764	0.3284	0.015	0.1119253	Mineral
Water_content	GT74	0.8924	0.0143	0.0025	-0.0743	0.8603	0.927046	Mineral
Water_content	GT75	0.9563	0.0378	0.0433	-0.0303	0.4567	0.7255136	Mineral
Water_content	GT76	2.3528	-0.3739	0.3964	0.35	0.0119	0.1003651	Mineral
Water_content	GT77	0.9469	-0.0833	0.1811	0.1181	0.1138	0.3007209	Mineral
Water_content	GT78	0.0018	-0.0012	0.0806	0.0098	0.3053	0.5541405	Mineral
Water_content	GT8	11.7518	0.4088	0.0256	-0.0494	0.5691	0.8030023	Mineral
Water_content	GT80	5.6377	0.5509	0.4543	0.4124	0.0059	0.0639391	Mineral
Water_content	GT82	0.4951	-0.0191	0.0165	-0.0591	0.648	0.8138708	Mineral
Water_content	GT84	296.2703	25.1563	0.1838	0.1211	0.1108	0.2990114	Mineral
Water_content	GT81	24.419	-0.146	0.0018	-0.0749	0.879	0.927046	Mineral
Water_content	GT83	171.2538	2.7607	0.0406	-0.0332	0.4717	0.7445564	Mineral
Water_content	GT85	3.2912	-0.336	0.0636	-0.0084	0.3644	0.6056539	Mineral
Water_content	GT87	16.9111	0.401	0.008	-0.0683	0.7515	0.8814588	Mineral
Water_content	GT88	0.0259	0.006	0.0651	-0.0068	0.3589	0.6004879	Mineral
Water_content	GT89	5.7924	-0.384	0.0534	-0.0194	0.4072	0.6680369	Mineral
Water_content	GT90	2.475	0.7343	0.3736	0.3254	0.0155	0.1119253	Mineral
Water_content	GT9	175.0926	-0.7492	0.0023	-0.0744	0.8638	0.927046	Mineral
Water_content	GT91	0.0159	-0.0003	0.0003	-0.0766	0.9486	0.9639129	Mineral
Water_content	GT93	0.368	0.0059	0.0175	-0.0581	0.6386	0.8136383	Mineral
Water_content	GT94	0.7572	0.0378	0.0286	-0.0461	0.5465	0.7975448	Mineral
Water_content	GT92	0.0971	0.0472	0.4809	0.441	0.0041	0.0508641	Mineral
Water_content	GT95	7.0408	-0.3527	0.1821	0.1191	0.1127	0.3007209	Mineral
Water_content	PL1	18.5567	-0.9461	0.0162	-0.0595	0.6517	0.8138708	Mineral
Water_content	PL10	4.7494	0.4116	0.0319	-0.0426	0.5243	0.7879608	Mineral
Water_content	PL11	3.638	-0.2478	0.0196	-0.0558	0.6186	0.8136383	Mineral
Water_content	PL12	23.2607	0.1581	0.0028	-0.0739	0.8515	0.927046	Mineral
Water_content	PL13	0.0005	0.0015	0.7243	0.7031	1.00E-04	0.0032562	Mineral
Water_content	PL14	1.1325	0.014	0.0006	-0.0763	0.9316	0.958887	Mineral
Water_content	PL15	0.3857	0.0004	0.00E+00	-0.0769	0.9955	0.9963372	Mineral
Water_content	PL17	1.8343	-0.1156	0.0135	-0.0624	0.6805	0.8373396	Mineral
Water_content	PL18	0.0042	-0.0017	0.117	0.0491	0.2121	0.4424513	Mineral
Water_content	PL16	0.52	-0.0207	0.0275	-0.0473	0.5546	0.7975448	Mineral
Water_content	PL2	0.1389	0.0264	0.2785	0.223	0.0432	0.1643397	Mineral
Water_content	PL20	0.0975	-0.0042	0.0025	-0.0742	0.8599	0.927046	Mineral
Water_content	PL21	0.4953	0.0238	0.0177	-0.0579	0.6364	0.8136383	Mineral
Water_content	PL22	9.2334	1.158	0.4899	0.4506	0.0037	0.0508641	Mineral
Water_content	PL23	0.0157	-0.0023	0.0788	0.0079	0.311	0.5541405	Mineral
Water_content	PL3	0.3266	-0.0083	0.0005	-0.0764	0.936	0.958887	Mineral
Water_content	PL4	15.4469	3.3437	0.6023	0.5717	0.0007	0.0152871	Mineral
Water_content	PL5	1.6555	0.7107	0.3274	0.2756	0.0258	0.1379294	Mineral

Water_content	PL6	2.3184	-0.2401	0.0273	-0.0475	0.5561	0.7975448	Mineral
Water_content	PL7	3.659	0.2348	0.0164	-0.0593	0.6494	0.8138708	Mineral
Water_content	PL8	2.5006	0.1737	0.0308	-0.0438	0.5318	0.7945897	Mineral
Water_content	PL9	33.1358	-0.4533	0.0021	-0.0746	0.8704	0.927046	Mineral
Bulk_density	AA1	161.2944	5.8575	0.137	0.0411	0.2625	0.6043664	Mineral
Bulk_density	AA11	0.3121	0.1758	0.4645	0.405	0.0209	0.1786831	Mineral
Bulk_density	AA10	5.5876	0.6552	0.1498	0.0554	0.2395	0.5837369	Mineral
Bulk_density	AA2	1.3175	0.1814	0.1471	0.0523	0.2443	0.5885725	Mineral
Bulk_density	AA3	409.805	19.8768	0.3173	0.2415	0.0711	0.3088867	Mineral
Bulk_density	AA4	11.5271	2.2317	0.2127	0.1252	0.1534	0.441306	Mineral
Bulk_density	AA5	51.0651	-2.1392	0.0794	-0.0228	0.4011	0.6848007	Mineral
Bulk_density	AA8	0.5436	0.0965	0.2081	0.1201	0.1585	0.4463424	Mineral
Bulk_density	AA7	54.2802	0.769	0.0084	-0.1018	0.7891	0.8850052	Mineral
Bulk_density	AA6	30.2681	-0.4203	0.0108	-0.0991	0.7611	0.8733663	Mineral
Bulk_density	AA9	1.0684	0.3049	0.3349	0.261	0.0621	0.2999047	Mineral
Bulk_density	CE1	213.9117	-4.9357	0.0966	-0.0037	0.3521	0.6406076	Mineral
Bulk_density	CE11	162.8769	-7.4096	0.4685	0.4095	0.0202	0.1786831	Mineral
Bulk_density	CE12	14.0483	0.5767	0.0306	-0.0771	0.6069	0.8072496	Mineral
Bulk_density	CE13	0.0381	0.0003	1.00E-04	-0.111	0.9773	0.9891475	Mineral
Bulk_density	CE14	219.9422	-6.8384	0.2745	0.1939	0.098	0.3515726	Mineral
Bulk_density	CE15	37.1782	0.7605	0.0107	-0.0992	0.762	0.8733663	Mineral
Bulk_density	CE2	2.0054	0.0932	0.013	-0.0966	0.7381	0.8690088	Mineral
Bulk_density	CE3	20.488	-1.7144	0.1265	0.0295	0.283	0.6203405	Mineral
Bulk_density	CE4	355.3861	-3.2685	0.0148	-0.0946	0.7212	0.8620344	Mineral
Bulk_density	CE16	0.9872	0.0301	0.0118	-0.098	0.7506	0.8722799	Mineral
Bulk_density	CE5	14.636	0.4977	0.0047	-0.1059	0.8414	0.9084819	Mineral
Bulk_density	CE7	9.4895	0.0326	0.0004	-0.1107	0.9564	0.9789174	Mineral
Bulk_density	CE6	39.6967	2.9946	0.1552	0.0614	0.2305	0.5786104	Mineral
Bulk_density	CE8	20.8399	-0.2656	0.0036	-0.1071	0.861	0.9235006	Mineral
Bulk_density	CE9	93.7646	-2.3832	0.365	0.2944	0.049	0.27342	Mineral
Bulk_density	GH100	0.6368	0.2533	0.5669	0.5188	0.0075	0.1192712	Mineral
Bulk_density	GH10	51.7622	-0.2889	0.0019	-0.109	0.8987	0.9598874	Mineral
Bulk_density	GH102	41.914	-1.951	0.5945	0.5494	0.0055	0.1143398	Mineral
Bulk_density	GH101	0.6717	-0.1023	0.2937	0.2152	0.0851	0.323534	Mineral
Bulk_density	GH103	107.7262	1.2098	0.0107	-0.0992	0.7618	0.8733663	Mineral
Bulk_density	GH104	5.2096	-0.5351	0.3075	0.2306	0.0766	0.3088867	Mineral
Bulk_density	GH105	24.2619	2.0363	0.0524	-0.0529	0.4985	0.7492244	Mineral
Bulk_density	GH106	43.0812	2.6041	0.0546	-0.0505	0.4893	0.7443658	Mineral
Bulk_density	GH107	0.1255	-0.0458	0.443	0.3811	0.0254	0.1964169	Mineral
Bulk_density	GH108	6.4222	0.1081	0.017	-0.0922	0.7022	0.8596207	Mineral
Bulk_density	GH109	19.0443	-0.7938	0.0953	-0.0052	0.3557	0.6406076	Mineral
Bulk_density	GH11	13.1099	1.3537	0.1946	0.1051	0.1744	0.4811052	Mineral
Bulk_density	GH110	1.0191	0.1955	0.3006	0.2229	0.0808	0.3167162	Mineral
Bulk_density	GH111	0.039	0.0043	0.0403	-0.0663	0.5538	0.7837393	Mineral
Bulk_density	GH112	0.0579	-0.0064	0.02	-0.0889	0.6786	0.8558385	Mineral
Bulk_density	GH113	7.5974	-0.5552	0.3573	0.2859	0.0521	0.2842469	Mineral
Bulk_density	GH114	10.7887	-0.1938	0.0046	-0.106	0.8433	0.9084819	Mineral
Bulk_density	GH115	13.2181	1.0694	0.0507	-0.0548	0.5058	0.7556197	Mineral
Bulk_density	GH116	44.1901	1.2249	0.0551	-0.0498	0.487	0.7443658	Mineral
Bulk_density	GH117	3.401	-0.2873	0.0726	-0.0304	0.423	0.7043721	Mineral
Bulk_density	GH118	0.0026	-0.001	0.1221	0.0246	0.2922	0.6203405	Mineral
Bulk_density	GH119	2.6789	-0.5188	0.779	0.7544	0.0003	0.0805485	Mineral
Bulk_density	GH12	7.5269	0.4862	0.0375	-0.0694	0.5683	0.7855525	Mineral
Bulk_density	GH120	2.0002	-0.2381	0.0466	-0.0593	0.5236	0.7686295	Mineral
Bulk_density	GH121	1.2814	-0.2763	0.1536	0.0596	0.2332	0.5794269	Mineral

Bulk_density	GH123	1.9331	-0.4298	0.5804	0.5338	0.0064	0.1152352	Mineral
Bulk_density	GH124	0.0673	-0.0054	0.0217	-0.087	0.6657	0.848959	Mineral
Bulk_density	GH125	10.4041	0.4445	0.0304	-0.0773	0.6078	0.8072496	Mineral
Bulk_density	GH126	0.0096	-0.0023	0.0433	-0.063	0.5393	0.7837393	Mineral
Bulk_density	GH127	40.2048	0.3017	0.0016	-0.1094	0.9078	0.9613869	Mineral
Bulk_density	GH128	5.7054	1.1923	0.2704	0.1893	0.1011	0.3574002	Mineral
Bulk_density	GH129	0.3947	-0.042	0.0662	-0.0375	0.4449	0.7122208	Mineral
Bulk_density	GH13	1487.2592	10.5351	0.0066	-0.1038	0.813	0.9024246	Mineral
Bulk_density	GH130	83.7461	-2.9034	0.0399	-0.0667	0.5558	0.7837393	Mineral
Bulk_density	GH131	0.133	0.0543	0.3083	0.2314	0.0762	0.3088867	Mineral
Bulk_density	GH132	0.5908	0.3193	0.4774	0.4193	0.0186	0.1786831	Mineral
Bulk_density	GH133	80.1456	-6.5856	0.483	0.4256	0.0176	0.1786831	Mineral
Bulk_density	GH14	0.4711	0.0655	0.3449	0.2722	0.0575	0.2943183	Mineral
Bulk_density	GH17	58.9696	1.9813	0.1159	0.0176	0.3057	0.6247638	Mineral
Bulk_density	GH15	412.7352	1.1794	0.0007	-0.1103	0.9373	0.9765885	Mineral
Bulk_density	GH18	230.9959	-0.1402	0.00E+00	-0.1111	0.9881	0.9894726	Mineral
Bulk_density	GH19	15.8905	-0.7037	0.049	-0.0566	0.5128	0.7601422	Mineral
Bulk_density	GH2	359.0529	-0.5047	0.0001	-0.111	0.9748	0.9891475	Mineral
Bulk_density	GH20	96.662	-2.1856	0.0607	-0.0437	0.4654	0.7346471	Mineral
Bulk_density	GH23	386.9574	-16.3229	0.5824	0.5361	0.0063	0.1152352	Mineral
Bulk_density	GH24	15.904	-1.5399	0.5654	0.5172	0.0076	0.1192712	Mineral
Bulk_density	GH25	18.7953	-0.8518	0.0486	-0.0571	0.5148	0.7601422	Mineral
Bulk_density	GH26	20.0754	0.9741	0.053	-0.0522	0.4958	0.7492244	Mineral
Bulk_density	GH27	70.2089	4.9994	0.0837	-0.0181	0.3883	0.6788582	Mineral
Bulk_density	GH28	175.1063	6.1325	0.0407	-0.0658	0.5517	0.7837393	Mineral
Bulk_density	GH29	108.4695	-1.4874	0.0153	-0.0941	0.717	0.8610744	Mineral
Bulk_density	GH3	551.9835	12.118	0.0252	-0.0831	0.6411	0.838108	Mineral
Bulk_density	GH30	47.1153	3.1304	0.1411	0.0457	0.2549	0.5923394	Mineral
Bulk_density	GH31	130.617	2.7944	0.0189	-0.0901	0.687	0.8558385	Mineral
Bulk_density	GH33	59.7688	-5.221	0.6216	0.5795	0.0039	0.0988427	Mineral
Bulk_density	GH36	83.0027	5.0944	0.1189	0.021	0.299	0.6247638	Mineral
Bulk_density	GH32	32.5933	-0.5609	0.0844	-0.0173	0.386	0.6788582	Mineral
Bulk_density	GH37	40.0428	1.3136	0.078	-0.0244	0.4054	0.6875895	Mineral
Bulk_density	GH39	83.2648	4.5301	0.2115	0.1239	0.1547	0.441306	Mineral
Bulk_density	GH38	105.1014	6.8881	0.0947	-0.0059	0.3573	0.6406076	Mineral
Bulk_density	GH4	134.5833	7.0197	0.1352	0.0391	0.266	0.6069476	Mineral
Bulk_density	GH42	42.5172	1.775	0.0979	-0.0023	0.3488	0.6406076	Mineral
Bulk_density	GH43	98.4801	3.4709	0.0182	-0.0908	0.6922	0.8558385	Mineral
Bulk_density	GH44	23.3153	0.0927	0.0006	-0.1104	0.943	0.9765885	Mineral
Bulk_density	GH45	0.3604	0.0541	0.1709	0.0788	0.2063	0.5337467	Mineral
Bulk_density	GH47	14.473	0.811	0.1136	0.0151	0.3109	0.6247638	Mineral
Bulk_density	GH46	1.7888	-0.2542	0.0802	-0.0219	0.3986	0.6848007	Mineral
Bulk_density	GH48	9.0035	0.5746	0.1003	0.0003	0.3427	0.6406076	Mineral
Bulk_density	GH49	0.0634	0.0213	0.7092	0.6769	0.0011	0.0988427	Mineral
Bulk_density	GH5	157.8075	-0.3506	0.0003	-0.1108	0.9582	0.9789174	Mineral
Bulk_density	GH50	8.478	0.2966	0.0187	-0.0903	0.6884	0.8558385	Mineral
Bulk_density	GH51	101.5191	3.4252	0.0402	-0.0664	0.5543	0.7837393	Mineral
Bulk_density	GH54	17.1874	4.303	0.2155	0.1283	0.1503	0.441306	Mineral
Bulk_density	GH53	53.3606	1.0345	0.0137	-0.0959	0.7321	0.8667448	Mineral
Bulk_density	GH55	104.2111	12.06	0.2839	0.2043	0.0915	0.337817	Mineral
Bulk_density	GH56	0.0037	0.0026	0.4786	0.4206	0.0184	0.1786831	Mineral
Bulk_density	GH57	158.9616	-9.5885	0.4244	0.3604	0.0299	0.2028123	Mineral
Bulk_density	GH58	0.0297	-0.0074	0.4622	0.4025	0.0214	0.1786831	Mineral
Bulk_density	GH59	6.5378	1.6043	0.2115	0.1238	0.1547	0.441306	Mineral
Bulk_density	GH6	15.8559	1.2576	0.0715	-0.0316	0.4266	0.7043721	Mineral

Bulk_density	GH62	2.7017	-0.1584	0.0216	-0.0871	0.6663	0.848959	Mineral
Bulk_density	GH63	12.8988	0.7942	0.0574	-0.0473	0.4779	0.7443658	Mineral
Bulk_density	GH64	18.5226	1.4317	0.1251	0.0279	0.2858	0.6203405	Mineral
Bulk_density	GH65	101.3447	-4.2929	0.0232	-0.0853	0.6548	0.8472127	Mineral
Bulk_density	GH66	1.0298	0.0934	0.4286	0.3651	0.0288	0.2028123	Mineral
Bulk_density	GH68	0.2616	-0.0045	0.0054	-0.1051	0.8296	0.9024246	Mineral
Bulk_density	GH7	0.4667	0.1667	0.2948	0.2165	0.0843	0.323534	Mineral
Bulk_density	GH70	0.0269	0.0008	0.006	-0.1044	0.8208	0.9024246	Mineral
Bulk_density	GH71	3.0668	1.0923	0.5128	0.4586	0.0132	0.1655297	Mineral
Bulk_density	GH72	28.3414	0.3706	0.0254	-0.0829	0.6396	0.838108	Mineral
Bulk_density	GH73	37.9329	-1.6412	0.2347	0.1497	0.131	0.4162291	Mineral
Bulk_density	GH75	2.0769	0.0095	0.0003	-0.1108	0.9594	0.9789174	Mineral
Bulk_density	GH74	11.9347	0.6578	0.1071	0.0079	0.3259	0.6401777	Mineral
Bulk_density	GH76	12.6187	1.8852	0.1617	0.0686	0.2202	0.562523	Mineral
Bulk_density	GH78	145.1794	6.3934	0.1168	0.0186	0.3037	0.6247638	Mineral
Bulk_density	GH79	28.1354	1.9543	0.2563	0.1736	0.1121	0.3906932	Mineral
Bulk_density	GH8	17.5144	-1.5538	0.1058	0.0065	0.329	0.6401777	Mineral
Bulk_density	GH80	0.0038	-0.003	0.1023	0.0026	0.3376	0.6406076	Mineral
Bulk_density	GH81	5.0397	0.004	0.00E+00	-0.1111	0.9855	0.9894726	Mineral
Bulk_density	GH84	4.3876	-0.1737	0.0833	-0.0186	0.3895	0.6788582	Mineral
Bulk_density	GH85	0.5499	-0.0742	0.0721	-0.031	0.4246	0.7043721	Mineral
Bulk_density	GH86	1.5216	-0.183	0.7015	0.6683	0.0013	0.0988427	Mineral
Bulk_density	GH87	63.4325	4.2214	0.2876	0.2084	0.089	0.3335775	Mineral
Bulk_density	GH88	23.8771	-0.6324	0.2507	0.1674	0.1167	0.3913892	Mineral
Bulk_density	GH89	6.7139	0.9454	0.1436	0.0484	0.2505	0.5897071	Mineral
Bulk_density	GH91	0.0724	-0.0053	0.0162	-0.0931	0.709	0.8596207	Mineral
Bulk_density	GH90	0.0014	0.0008	0.0993	-0.0008	0.3453	0.6406076	Mineral
Bulk_density	GH9	49.702	-1.5604	0.0121	-0.0976	0.7471	0.8722227	Mineral
Bulk_density	GH92	86.5726	5.7071	0.0387	-0.0681	0.562	0.7844296	Mineral
Bulk_density	GH93	15.9477	0.8358	0.0386	-0.0682	0.5625	0.7844296	Mineral
Bulk_density	GH94	29.1615	-0.3995	0.0054	-0.1051	0.8295	0.9024246	Mineral
Bulk_density	GH95	45.1553	-0.2164	0.0008	-0.1102	0.9332	0.9765885	Mineral
Bulk_density	GH96	0.3687	-0.1496	0.3115	0.235	0.0744	0.3088867	Mineral
Bulk_density	GH97	30.4323	-2.0636	0.1016	0.0018	0.3393	0.6406076	Mineral
Bulk_density	GH98	0.3119	-0.0143	0.0373	-0.0697	0.5696	0.7855525	Mineral
Bulk_density	GH99	11.7148	-0.9772	0.3086	0.2318	0.076	0.3088867	Mineral
Bulk_density	GT1	143.5121	9.897	0.1039	0.0044	0.3336	0.6406076	Mineral
Bulk_density	GT10	2.4027	0.0203	0.0017	-0.1092	0.9036	0.9610368	Mineral
Bulk_density	GT12	0.05	-0.0026	0.0187	-0.0903	0.6882	0.8558385	Mineral
Bulk_density	GT14	2.0412	-0.0182	0.001	-0.11	0.9279	0.9765885	Mineral
Bulk_density	GT11	5.1203	-0.2781	0.1067	0.0074	0.3269	0.6401777	Mineral
Bulk_density	GT13	0.1475	0.0339	0.1224	0.0249	0.2915	0.6203405	Mineral
Bulk_density	GT15	1.6932	0.8913	0.6287	0.5875	0.0036	0.0988427	Mineral
Bulk_density	GT16	0.0343	0.015	0.1854	0.0948	0.1862	0.5068263	Mineral
Bulk_density	GT17	1.0121	-0.0385	0.0355	-0.0717	0.5793	0.7901743	Mineral
Bulk_density	GT18	0.0089	0.0044	0.2136	0.1262	0.1524	0.441306	Mineral
Bulk_density	GT2	1815.1555	-30.1914	0.3408	0.2675	0.0594	0.2980058	Mineral
Bulk_density	GT19	83.5879	-2.5663	0.2504	0.1671	0.1169	0.3913892	Mineral
Bulk_density	GT20	153.1716	1.3996	0.1739	0.0821	0.202	0.5281081	Mineral
Bulk_density	GT21	80.3411	2.9935	0.1245	0.0272	0.2872	0.6203405	Mineral
Bulk_density	GT22	3.9753	0.7056	0.3983	0.3314	0.0373	0.2218801	Mineral
Bulk_density	GT23	0.2063	0.0229	0.0677	-0.0359	0.4398	0.7122208	Mineral
Bulk_density	GT24	2.5814	0.4951	0.3232	0.248	0.068	0.3088867	Mineral
Bulk_density	GT25	5.9232	0.2047	0.0327	-0.0747	0.5945	0.7979793	Mineral
Bulk_density	GT26	129.5563	3.2251	0.1431	0.0478	0.2514	0.5897071	Mineral

Bulk_density	GT27	13.3328	0.46	0.1092	0.0103	0.3209	0.6391684	Mineral
Bulk_density	GT28	175.0731	-4.7908	0.3454	0.2727	0.0572	0.2943183	Mineral
Bulk_density	GT29	3.7019	-0.1247	0.0744	-0.0284	0.417	0.7024965	Mineral
Bulk_density	GT30	119.7723	-3.2495	0.2067	0.1186	0.16	0.4463424	Mineral
Bulk_density	GT3	2.4181	0.2936	0.1212	0.0236	0.2941	0.6203405	Mineral
Bulk_density	GT33	1.5325	0.0062	0.0007	-0.1104	0.9392	0.9765885	Mineral
Bulk_density	GT34	0.7902	0.3056	0.4665	0.4072	0.0205	0.1786831	Mineral
Bulk_density	GT32	11.6959	0.966	0.4248	0.3609	0.0298	0.2028123	Mineral
Bulk_density	GT31	1.1494	0.476	0.3464	0.2738	0.0568	0.2943183	Mineral
Bulk_density	GT35	363.6916	-20.6602	0.2765	0.1961	0.0967	0.3515726	Mineral
Bulk_density	GT37	0.0049	0.0031	0.4091	0.3435	0.0341	0.213797	Mineral
Bulk_density	GT39	18.8481	1.4742	0.127	0.03	0.2821	0.6203405	Mineral
Bulk_density	GT4	1808.4426	-54.9777	0.659	0.6211	0.0024	0.0988427	Mineral
Bulk_density	GT41	436.5386	-17.403	0.4156	0.3507	0.0322	0.2129532	Mineral
Bulk_density	GT40	0.0016	-0.0003	0.0094	-0.1007	0.7766	0.8817812	Mineral
Bulk_density	GT42	0.0626	0.0364	0.3995	0.3328	0.0369	0.2218801	Mineral
Bulk_density	GT43	0.0765	0.0154	0.0819	-0.0201	0.3937	0.681473	Mineral
Bulk_density	GT47	64.8633	7.5086	0.3961	0.329	0.038	0.2218801	Mineral
Bulk_density	GT45	0.2453	-0.0232	0.2267	0.1407	0.1388	0.4247979	Mineral
Bulk_density	GT44	0.0215	-0.0036	0.0246	-0.0838	0.6452	0.8390657	Mineral
Bulk_density	GT48	2.9657	1.352	0.4985	0.4428	0.0152	0.1786831	Mineral
Bulk_density	GT49	0.1823	0.0728	0.4123	0.347	0.0332	0.21349	Mineral
Bulk_density	GT5	111.3049	-5.2662	0.3255	0.2506	0.0668	0.3088867	Mineral
Bulk_density	GT50	0.5838	0.2578	0.5252	0.4725	0.0116	0.1622859	Mineral
Bulk_density	GT53	23.6639	1.0076	0.0099	-0.1001	0.7706	0.8791848	Mineral
Bulk_density	GT52	0	0	#VALUE!	#VALUE!	#VALUE!	NA	Mineral
Bulk_density	GT51	590.4187	-27.2965	0.6279	0.5866	0.0036	0.0988427	Mineral
Bulk_density	GT54	0.0285	0.0159	0.2456	0.1618	0.1211	0.3948133	Mineral
Bulk_density	GT57	1.22	0.5642	0.4715	0.4128	0.0196	0.1786831	Mineral
Bulk_density	GT56	0.3877	-0.0067	0.0063	-0.1041	0.8162	0.9024246	Mineral
Bulk_density	GT55	2.7657	0.5963	0.306	0.2289	0.0775	0.3088867	Mineral
Bulk_density	GT58	0.4711	0.2208	0.5959	0.551	0.0054	0.1143398	Mineral
Bulk_density	GT6	0.3926	-0.0723	0.2286	0.1429	0.1369	0.4242067	Mineral
Bulk_density	GT59	0.3096	0.1442	0.4432	0.3814	0.0253	0.1964169	Mineral
Bulk_density	GT60	1.9055	0.2168	0.0564	-0.0484	0.482	0.7443658	Mineral
Bulk_density	GT61	0.3416	0.053	0.123	0.0255	0.2904	0.6203405	Mineral
Bulk_density	GT62	2.0791	0.3055	0.3379	0.2644	0.0607	0.2987452	Mineral
Bulk_density	GT64	0.7976	-0.0295	0.0585	-0.0461	0.4736	0.7429596	Mineral
Bulk_density	GT65	0.0458	0.0107	0.1809	0.0899	0.1922	0.51319	Mineral
Bulk_density	GT66	18.6873	-1.2779	0.4411	0.379	0.0258	0.1964169	Mineral
Bulk_density	GT69	0.6705	0.2981	0.3702	0.3002	0.047	0.268074	Mineral
Bulk_density	GT68	0.0227	0.0025	0.033	-0.0745	0.5932	0.7979793	Mineral
Bulk_density	GT7	5.6284	-0.0754	0.0164	-0.0928	0.7071	0.8596207	Mineral
Bulk_density	GT72	0.0011	0.0006	0.0693	-0.0341	0.4341	0.7122208	Mineral
Bulk_density	GT73	0.053	0.0096	0.0619	-0.0423	0.4606	0.7316536	Mineral
Bulk_density	GT71	1.292	0.1435	0.1516	0.0573	0.2366	0.5821185	Mineral
Bulk_density	GT70	0.7657	0.0587	0.1752	0.0836	0.2001	0.5281081	Mineral
Bulk_density	GT74	1.0251	0.0488	0.0661	-0.0377	0.4455	0.7122208	Mineral
Bulk_density	GT75	0.996	-0.0189	0.0128	-0.0969	0.7409	0.8690088	Mineral
Bulk_density	GT76	2.2343	0.0577	0.0091	-0.101	0.7799	0.8817812	Mineral
Bulk_density	GT77	0.9679	-0.0009	0.00E+00	-0.1111	0.9895	0.9894726	Mineral
Bulk_density	GT78	0.0018	-0.0007	0.0225	-0.0861	0.6598	0.848959	Mineral
Bulk_density	GT8	10.5913	0.2549	0.0365	-0.0706	0.5739	0.7871182	Mineral
Bulk_density	GT80	5.5883	-0.081	0.0081	-0.1021	0.792	0.8850052	Mineral
Bulk_density	GT82	0.4491	-0.0593	0.2464	0.1627	0.1204	0.3948133	Mineral

Bulk_density	GT84	304.5007	-15.4962	0.055	-0.05	0.4876	0.7443658	Mineral
Bulk_density	GT81	23.2612	-0.9147	0.1134	0.0149	0.3111	0.6247638	Mineral
Bulk_density	GT83	171.3121	-1.423	0.0172	-0.092	0.7004	0.8596207	Mineral
Bulk_density	GT85	3.3357	0.2593	0.0281	-0.0799	0.6224	0.8221631	Mineral
Bulk_density	GT87	15.7945	1.4388	0.0961	-0.0043	0.3534	0.6406076	Mineral
Bulk_density	GT88	0.0327	0.0061	0.0668	-0.0369	0.4428	0.7122208	Mineral
Bulk_density	GT89	5.485	-0.0429	0.0005	-0.1105	0.9455	0.9765885	Mineral
Bulk_density	GT90	2.2799	0.6343	0.3079	0.231	0.0764	0.3088867	Mineral
Bulk_density	GT9	168.8338	-9.4071	0.5137	0.4597	0.0131	0.1655297	Mineral
Bulk_density	GT91	0.013	0.0071	0.3308	0.2565	0.0641	0.3037842	Mineral
Bulk_density	GT93	0.361	0.016	0.1324	0.0359	0.2714	0.6137014	Mineral
Bulk_density	GT94	0.7634	0.0412	0.0411	-0.0655	0.5501	0.7837393	Mineral
Bulk_density	GT92	0.0917	0.0506	0.4732	0.4146	0.0193	0.1786831	Mineral
Bulk_density	GT95	7.2653	-0.6126	0.6587	0.6208	0.0024	0.0988427	Mineral
Bulk_density	PL1	15.9793	-1.8489	0.1014	0.0016	0.3399	0.6406076	Mineral
Bulk_density	PL10	3.9981	-0.222	0.0138	-0.0958	0.7307	0.8667448	Mineral
Bulk_density	PL11	3.0834	-0.6085	0.1606	0.0674	0.2219	0.562523	Mineral
Bulk_density	PL12	22.1345	-1.7083	0.5515	0.5017	0.0088	0.1305776	Mineral
Bulk_density	PL13	0.0006	0.001	0.2208	0.1343	0.1447	0.4376198	Mineral
Bulk_density	PL14	0.8222	0.155	0.6597	0.6219	0.0024	0.0988427	Mineral
Bulk_density	PL15	0.278	-0.1037	0.6393	0.5992	0.0031	0.0988427	Mineral
Bulk_density	PL17	1.3535	-0.3512	0.4309	0.3676	0.0283	0.2028123	Mineral
Bulk_density	PL18	0.0039	-0.0027	0.2426	0.1584	0.1238	0.3982872	Mineral
Bulk_density	PL16	0.534	-0.0436	0.146	0.0511	0.2462	0.5885725	Mineral
Bulk_density	PL2	0.152	-0.0143	0.0864	-0.0151	0.3803	0.6769582	Mineral
Bulk_density	PL20	0.0663	-0.0082	0.0158	-0.0935	0.7124	0.8596207	Mineral
Bulk_density	PL21	0.5679	-0.0749	0.2509	0.1677	0.1165	0.3913892	Mineral
Bulk_density	PL22	8.5676	0.2826	0.041	-0.0655	0.5504	0.7837393	Mineral
Bulk_density	PL23	0.0152	-0.0008	0.008	-0.1022	0.7933	0.8850052	Mineral
Bulk_density	PL3	0.2025	-0.042	0.0332	-0.0742	0.5917	0.7979793	Mineral
Bulk_density	PL4	15.1922	2.7896	0.3201	0.2445	0.0697	0.3088867	Mineral
Bulk_density	PL5	1.8604	0.5977	0.1842	0.0935	0.1878	0.5068263	Mineral
Bulk_density	PL6	1.7714	-0.5709	0.2307	0.1452	0.1348	0.4230444	Mineral
Bulk_density	PL7	2.8643	0.0957	0.0054	-0.1051	0.8305	0.9024246	Mineral
Bulk_density	PL8	2.067	-0.093	0.0186	-0.0905	0.6896	0.8558385	Mineral
Bulk_density	PL9	29.0113	-2.3974	0.114	0.0156	0.3098	0.6247638	Mineral
pH	AA1	161.1286	0.4917	0.0007	-0.0761	0.9239	0.966266	Mineral
pH	AA11	0.406	0.0234	0.0057	-0.0708	0.7892	0.8996393	Mineral
pH	AA10	6.1103	-0.2333	0.0169	-0.0587	0.6445	0.8584218	Mineral
pH	AA2	1.4848	0.0246	0.0018	-0.075	0.8812	0.9516909	Mineral
pH	AA3	426.2452	12.3527	0.0686	-0.0031	0.3458	0.6651927	Mineral
pH	AA4	11.2554	-2.0478	0.2439	0.1858	0.0613	0.2145638	Mineral
pH	AA5	53.5482	5.8116	0.5338	0.4979	0.002	0.0275625	Mineral
pH	AA8	0.6569	0.0605	0.035	-0.0393	0.5046	0.7693795	Mineral
pH	AA7	57.2187	-0.2842	0.0009	-0.0759	0.9136	0.966266	Mineral
pH	AA6	30.8574	-0.1092	0.0007	-0.0761	0.9238	0.966266	Mineral
pH	AA9	1.4632	0.2094	0.025	-0.05	0.5737	0.8305125	Mineral
pH	CE1	216.8049	-3.6618	0.0637	-0.0084	0.3642	0.6722475	Mineral
pH	CE11	163.2288	0.7284	0.0057	-0.0708	0.7898	0.8996393	Mineral
pH	CE12	15.6874	0.3084	0.005	-0.0715	0.8014	0.9061389	Mineral
pH	CE13	0.0362	-0.0178	0.456	0.4141	0.0057	0.0533367	Mineral
pH	CE14	221.0157	-4.5193	0.1335	0.0668	0.1806	0.4120044	Mineral
pH	CE15	37.8269	-3.0411	0.2033	0.1421	0.0916	0.2726595	Mineral
pH	CE2	2.5005	0.1252	0.0087	-0.0675	0.7408	0.8853873	Mineral
pH	CE3	26.5046	5.5857	0.2351	0.1762	0.067	0.2212857	Mineral

pH	CE4	378.0703	38.1847	0.6481	0.621	0.0003	0.0110588	Mineral
pH	CE16	1.0559	-0.0004	0.00E+00	-0.0769	0.9964	0.9964393	Mineral
pH	CE5	14.5299	-2.9694	0.2223	0.1625	0.076	0.2446355	Mineral
pH	CE7	10.4421	0.2186	0.0079	-0.0684	0.7525	0.8909869	Mineral
pH	CE6	37.5291	-6.4206	0.7285	0.7076	1.00E-04	0.0032539	Mineral
pH	CE8	22.1825	-0.9035	0.0338	-0.0405	0.5119	0.7693795	Mineral
pH	CE9	94.9446	0.0426	0.0001	-0.0768	0.9716	0.9854265	Mineral
pH	GH100	0.6919	-0.0119	0.0011	-0.0757	0.9046	0.9620996	Mineral
pH	GH10	53.2214	-1.9769	0.0901	0.0202	0.2769	0.5515999	Mineral
pH	GH102	43.2037	1.87	0.3106	0.2576	0.0309	0.1292003	Mineral
pH	GH101	0.8491	0.1863	0.2661	0.2096	0.0491	0.1894101	Mineral
pH	GH103	113.5215	12.9492	0.6844	0.6601	0.0001	0.0071048	Mineral
pH	GH104	5.515	0.7378	0.5378	0.5023	0.0019	0.0274971	Mineral
pH	GH105	25.0043	-3.8576	0.2386	0.18	0.0647	0.2194497	Mineral
pH	GH106	43.4576	-5.5954	0.3374	0.2864	0.0232	0.1086981	Mineral
pH	GH107	0.1988	0.0683	0.2226	0.1628	0.0758	0.2446355	Mineral
pH	GH108	6.4325	-0.3327	0.2049	0.1437	0.0902	0.2726595	Mineral
pH	GH109	19.7839	-0.3756	0.0215	-0.0537	0.6017	0.8305125	Mineral
pH	GH11	13.636	-0.9689	0.1095	0.0409	0.2284	0.481793	Mineral
pH	GH110	1.0855	-0.0797	0.0492	-0.024	0.427	0.7193698	Mineral
pH	GH111	0.0484	0.0039	0.0207	-0.0546	0.6086	0.8305125	Mineral
pH	GH112	0.065	-0.0112	0.0581	-0.0143	0.3867	0.693184	Mineral
pH	GH113	8.5535	1.1858	0.3828	0.3353	0.0139	0.0813164	Mineral
pH	GH114	11.4339	0.2681	0.0083	-0.068	0.7475	0.8892075	Mineral
pH	GH115	15.0153	-0.2793	0.0026	-0.0741	0.8556	0.9450886	Mineral
pH	GH116	40.527	-5.3228	0.4679	0.4269	0.0049	0.0514539	Mineral
pH	GH117	3.5852	-0.2992	0.0843	0.0139	0.2937	0.5759356	Mineral
pH	GH118	0.0033	0.0004	0.0191	-0.0564	0.6235	0.8413696	Mineral
pH	GH119	3.1764	0.6934	0.4063	0.3606	0.0106	0.0764343	Mineral
pH	GH12	8.1909	-0.6221	0.0629	-0.0092	0.3673	0.6729576	Mineral
pH	GH120	1.8095	-0.7741	0.5541	0.5198	0.0015	0.0243971	Mineral
pH	GH121	1.9359	0.5333	0.1598	0.0952	0.1399	0.3647251	Mineral
pH	GH123	2.2624	0.5537	0.5267	0.4903	0.0022	0.028361	Mineral
pH	GH124	0.0624	0.0094	0.069	-0.0026	0.3443	0.6651927	Mineral
pH	GH125	10.2003	-1.3991	0.3361	0.285	0.0235	0.1086981	Mineral
pH	GH126	0.0079	-0.0052	0.2719	0.2159	0.0462	0.1841931	Mineral
pH	GH127	40.8673	-3.3851	0.2444	0.1862	0.0611	0.2145638	Mineral
pH	GH128	6.089	-0.7227	0.1207	0.0531	0.2045	0.4449129	Mineral
pH	GH129	0.3723	-0.0704	0.2122	0.1516	0.084	0.263029	Mineral
pH	GH13	1511.7028	-40.5531	0.1027	0.0336	0.2443	0.503742	Mineral
pH	GH130	83.298	2.6006	0.0443	-0.0292	0.4513	0.7452168	Mineral
pH	GH131	0.1722	0.0203	0.0244	-0.0506	0.5782	0.8305125	Mineral
pH	GH132	0.6932	-0.0236	0.0023	-0.0745	0.866	0.9450886	Mineral
pH	GH133	77.1918	-1.3595	0.0203	-0.055	0.6121	0.8305125	Mineral
pH	GH14	0.4871	-0.0334	0.1012	0.0321	0.2478	0.5057044	Mineral
pH	GH17	62.1908	6.446	0.5099	0.4722	0.0028	0.0333083	Mineral
pH	GH15	404.3419	-32.029	0.5918	0.5604	0.0008	0.0174451	Mineral
pH	GH18	240.9138	-4.6884	0.0256	-0.0493	0.5689	0.8302148	Mineral
pH	GH19	17.4844	0.2076	0.0027	-0.074	0.8531	0.9450886	Mineral
pH	GH2	363.2682	-21.4111	0.254	0.1966	0.0554	0.20167	Mineral
pH	GH20	98.8613	-2.306	0.0681	-0.0036	0.3474	0.6651927	Mineral
pH	GH23	402.7243	12.8673	0.1509	0.0855	0.1525	0.3753963	Mineral
pH	GH24	16.0614	-0.3563	0.041	-0.0328	0.4694	0.7566331	Mineral
pH	GH25	20.5589	0.1784	0.0014	-0.0754	0.8948	0.9556985	Mineral
pH	GH26	21.4128	-0.6469	0.0183	-0.0572	0.6308	0.8466675	Mineral

pH	GH27	70.6768	-8.9014	0.355	0.3054	0.0191	0.1024383	Mineral
pH	GH28	177.5732	-12.1185	0.2078	0.1469	0.0877	0.2683674	Mineral
pH	GH29	104.4902	-10.7817	0.7637	0.7455	0.00E+00	0.0018451	Mineral
pH	GH3	550.2428	-45.0157	0.4607	0.4192	0.0054	0.0521198	Mineral
pH	GH30	48.4301	-3.0401	0.1579	0.0932	0.1424	0.3647251	Mineral
pH	GH31	125.157	-17.3973	0.7646	0.7465	0.00E+00	0.0018451	Mineral
pH	GH33	63.7208	5.554	0.3463	0.296	0.021	0.1086981	Mineral
pH	GH36	81.126	-9.861	0.58	0.5477	0.001	0.0187206	Mineral
pH	GH32	31.8356	-0.3514	0.0207	-0.0546	0.6088	0.8305125	Mineral
pH	GH37	36.4506	-5.4493	0.5246	0.488	0.0023	0.028361	Mineral
pH	GH39	81.1725	-5.8535	0.3967	0.3503	0.0119	0.0783275	Mineral
pH	GH38	103.1959	-12.4762	0.4052	0.3594	0.0107	0.0764343	Mineral
pH	GH4	145.5656	4.7713	0.0339	-0.0404	0.511	0.7693795	Mineral
pH	GH42	42.3015	-3.0405	0.3429	0.2924	0.0218	0.1086981	Mineral
pH	GH43	105.7244	-6.3261	0.0484	-0.0248	0.4308	0.7208334	Mineral
pH	GH44	24.2141	0.4517	0.0157	-0.06	0.6566	0.8584218	Mineral
pH	GH45	0.4034	-0.0028	0.0002	-0.0767	0.957	0.9764972	Mineral
pH	GH47	15.3148	-0.2887	0.0108	-0.0653	0.7127	0.8764244	Mineral
pH	GH46	2.3583	0.2689	0.0419	-0.0318	0.4645	0.7566331	Mineral
pH	GH48	9.3733	-0.4	0.0567	-0.0159	0.3928	0.6942585	Mineral
pH	GH49	0.174	0.1245	0.3997	0.3535	0.0114	0.0776561	Mineral
pH	GH5	169.7639	2.6688	0.0095	-0.0667	0.7301	0.8852321	Mineral
pH	GH50	7.8367	-1.2566	0.3404	0.2897	0.0224	0.1086981	Mineral
pH	GH51	100.2913	-10.1209	0.4633	0.422	0.0052	0.0521198	Mineral
pH	GH54	16.7488	-4.9624	0.3901	0.3432	0.0128	0.0784134	Mineral
pH	GH53	54.6684	1.2091	0.0219	-0.0533	0.5986	0.8305125	Mineral
pH	GH55	98.5985	-17.0973	0.6149	0.5852	0.0005	0.0150442	Mineral
pH	GH56	0.003	-0.0014	0.1437	0.0779	0.1634	0.3820039	Mineral
pH	GH57	155.9399	1.0817	0.0055	-0.071	0.7921	0.8996393	Mineral
pH	GH58	0.0426	0.0161	0.3119	0.2589	0.0305	0.1292003	Mineral
pH	GH59	6.4703	-1.8619	0.3903	0.3434	0.0128	0.0784134	Mineral
pH	GH6	17.654	-0.563	0.011	-0.0651	0.7098	0.8764244	Mineral
pH	GH62	3.2335	0.0671	0.0024	-0.0744	0.8635	0.9450886	Mineral
pH	GH63	14.1513	-0.389	0.011	-0.0651	0.7103	0.8764244	Mineral
pH	GH64	18.7715	-1.8748	0.2696	0.2134	0.0473	0.1856278	Mineral
pH	GH65	100.9432	-13.6967	0.3051	0.2516	0.0328	0.134781	Mineral
pH	GH66	1.5117	0.5413	0.4171	0.3723	0.0093	0.0764343	Mineral
pH	GH68	0.2737	0.0249	0.1723	0.1086	0.1239	0.3344377	Mineral
pH	GH7	0.661	0.114	0.0367	-0.0374	0.4942	0.7656707	Mineral
pH	GH70	0.0411	0.0147	0.192	0.1299	0.1023	0.2917832	Mineral
pH	GH71	3.5602	0.2156	0.0149	-0.0609	0.6645	0.8597998	Mineral
pH	GH72	30.0334	0.7408	0.0401	-0.0337	0.4742	0.7566331	Mineral
pH	GH73	39.5823	0.5293	0.0167	-0.059	0.6467	0.8584218	Mineral
pH	GH75	2.2715	-0.1011	0.0241	-0.051	0.5806	0.8305125	Mineral
pH	GH74	12.8907	0.1013	0.0016	-0.0752	0.8888	0.9533877	Mineral
pH	GH76	13.3927	-1.5734	0.1238	0.0564	0.1983	0.4405821	Mineral
pH	GH78	148.9518	-6.371	0.1284	0.0614	0.1897	0.4288985	Mineral
pH	GH79	25.909	-3.0509	0.3295	0.2779	0.0252	0.1111657	Mineral
pH	GH8	19.2424	-0.4556	0.0072	-0.0691	0.7629	0.8948106	Mineral
pH	GH80	0.0028	-0.0031	0.148	0.0825	0.1567	0.3753963	Mineral
pH	GH81	6.0259	0.8634	0.2111	0.1504	0.0849	0.263029	Mineral
pH	GH84	4.7774	0.2046	0.0551	-0.0175	0.3996	0.7013409	Mineral
pH	GH85	0.6451	0.0028	1.00E-04	-0.0768	0.9736	0.9854265	Mineral
pH	GH86	1.7515	0.2966	0.4472	0.4047	0.0064	0.0574951	Mineral
pH	GH87	64.8133	-1.027	0.015	-0.0608	0.6636	0.8597998	Mineral

pH	GH88	24.9678	1.3163	0.3342	0.2829	0.024	0.1086981	Mineral
pH	GH89	6.8897	-1.0961	0.2375	0.1789	0.0654	0.2194497	Mineral
pH	GH91	0.106	0.0291	0.148	0.0824	0.1568	0.3753963	Mineral
pH	GH90	0.0045	0.0039	0.4133	0.3682	0.0097	0.0764343	Mineral
pH	GH9	51.5097	-5.6766	0.1876	0.1251	0.1068	0.2979708	Mineral
pH	GH92	91.0695	-11.4601	0.1794	0.1163	0.1156	0.3154333	Mineral
pH	GH93	22.1339	4.5226	0.1225	0.055	0.2009	0.4422557	Mineral
pH	GH94	27.0425	-5.1554	0.7613	0.7429	0.00E+00	0.0018451	Mineral
pH	GH95	48.6912	0.0543	0.00E+00	-0.0769	0.9834	0.9873015	Mineral
pH	GH96	0.5474	0.1591	0.1625	0.0981	0.1362	0.3599467	Mineral
pH	GH97	32.6174	-0.2819	0.0017	-0.0751	0.8834	0.9516909	Mineral
pH	GH98	0.3397	0.0646	0.641	0.6134	0.0003	0.0110588	Mineral
pH	GH99	13.1032	1.0024	0.1202	0.0525	0.2056	0.4449129	Mineral
pH	GT1	153.6974	-2.0972	0.0043	-0.0723	0.8173	0.9187257	Mineral
pH	GT10	2.4352	0.0351	0.0069	-0.0695	0.7687	0.897414	Mineral
pH	GT12	0.0482	-0.004	0.036	-0.0381	0.4981	0.7669679	Mineral
pH	GT14	2.0647	-0.317	0.3379	0.287	0.023	0.1086981	Mineral
pH	GT11	4.9506	-0.1806	0.0518	-0.0212	0.4147	0.7118663	Mineral
pH	GT13	0.1413	-0.0525	0.377	0.3291	0.0149	0.0849638	Mineral
pH	GT15	1.84	-0.1051	0.0091	-0.0672	0.7359	0.8853779	Mineral
pH	GT16	0.0336	-0.0119	0.1478	0.0823	0.157	0.3753963	Mineral
pH	GT17	1.0318	0.0158	0.0063	-0.0702	0.7789	0.8996393	Mineral
pH	GT18	0.0082	-0.0046	0.2436	0.1854	0.0615	0.2145638	Mineral
pH	GT2	1841.616	30.3574	0.1992	0.1376	0.0954	0.2751713	Mineral
pH	GT19	83.4375	0.0949	0.0004	-0.0765	0.9413	0.969851	Mineral
pH	GT20	150.7734	-3.6581	0.4423	0.3994	0.0068	0.0590269	Mineral
pH	GT21	72.0818	-10.3821	0.4033	0.3574	0.011	0.0764343	Mineral
pH	GT22	4.3521	-0.0369	0.0008	-0.076	0.9193	0.966266	Mineral
pH	GT23	0.2031	-0.0348	0.2013	0.1399	0.0934	0.2726595	Mineral
pH	GT24	2.657	-0.2783	0.1056	0.0368	0.2372	0.4962456	Mineral
pH	GT25	5.9103	-0.1601	0.026	-0.049	0.5662	0.8302148	Mineral
pH	GT26	133.0274	2.3549	0.058	-0.0145	0.3872	0.693184	Mineral
pH	GT27	13.0687	-0.7727	0.3545	0.3049	0.0192	0.1024383	Mineral
pH	GT28	177.3669	2.7479	0.1124	0.0442	0.2218	0.47582	Mineral
pH	GT29	3.9302	0.2308	0.1453	0.0796	0.1609	0.3810885	Mineral
pH	GT30	120.1726	0.9188	0.0219	-0.0533	0.5983	0.8305125	Mineral
pH	GT3	2.7042	-0.0644	0.0041	-0.0725	0.8199	0.9187257	Mineral
pH	GT33	1.8007	0.3276	0.407	0.3614	0.0105	0.0764343	Mineral
pH	GT34	0.89	-0.0384	0.0058	-0.0707	0.7874	0.8996393	Mineral
pH	GT32	12.395	1.1167	0.3153	0.2627	0.0294	0.1271693	Mineral
pH	GT31	1.3616	-0.0768	0.0074	-0.0689	0.7598	0.8948106	Mineral
pH	GT35	348.7674	-33.7566	0.6384	0.6106	0.0004	0.0110588	Mineral
pH	GT37	0.0045	-0.0008	0.0296	-0.0451	0.54	0.806736	Mineral
pH	GT39	20.8559	0.1386	0.0006	-0.0763	0.9305	0.9690615	Mineral
pH	GT4	1887.0784	94.8147	0.3912	0.3444	0.0126	0.0784134	Mineral
pH	GT41	420.3074	-23.458	0.3652	0.3163	0.017	0.0949891	Mineral
pH	GT40	0.0012	-0.0007	0.0912	0.0213	0.274	0.5515999	Mineral
pH	GT42	0.0617	-0.0134	0.0652	-0.0068	0.3585	0.6665809	Mineral
pH	GT43	0.0665	-0.0252	0.2372	0.1785	0.0656	0.2194497	Mineral
pH	GT47	67.1447	1.2262	0.009	-0.0673	0.7372	0.8853779	Mineral
pH	GT45	0.2617	0.008	0.0207	-0.0547	0.6094	0.8305125	Mineral
pH	GT44	0.0225	-0.0032	0.0225	-0.0527	0.5933	0.8305125	Mineral
pH	GT48	3.0789	-0.33	0.0343	-0.0399	0.5084	0.7693795	Mineral
pH	GT49	0.176	-0.044	0.1896	0.1272	0.1048	0.2954619	Mineral
pH	GT5	111.8839	2.789	0.1241	0.0567	0.1979	0.4405821	Mineral

pH	GT50	0.6419	-0.0412	0.0124	-0.0636	0.6932	0.8764244	Mineral
pH	GT53	23.5174	-4.3951	0.2563	0.1991	0.0542	0.1999064	Mineral
pH	GT52	0	0	#VALUE!	#VALUE!	#VALUE!	NA	Mineral
pH	GT51	615.404	38.8243	0.5565	0.5224	0.0014	0.0243971	Mineral
pH	GT54	0.0278	-0.0131	0.2182	0.158	0.0792	0.2516079	Mineral
pH	GT57	1.3329	-0.1648	0.0387	-0.0353	0.4823	0.7566331	Mineral
pH	GT56	0.3995	-0.029	0.1516	0.0864	0.1514	0.3753963	Mineral
pH	GT55	2.8539	-0.3857	0.1504	0.0851	0.1532	0.3753963	Mineral
pH	GT58	0.5128	-0.0352	0.0141	-0.0618	0.6739	0.8630415	Mineral
pH	GT6	0.334	-0.0319	0.0382	-0.0358	0.4853	0.7566331	Mineral
pH	GT59	0.3598	-0.0267	0.0135	-0.0624	0.6804	0.8669602	Mineral
pH	GT60	1.8074	-0.5898	0.5436	0.5085	0.0017	0.0267984	Mineral
pH	GT61	0.3572	-0.0547	0.1583	0.0936	0.1419	0.3647251	Mineral
pH	GT62	2.1958	0.0115	0.0004	-0.0765	0.9428	0.969851	Mineral
pH	GT64	0.9005	0.0809	0.1344	0.0678	0.179	0.4120044	Mineral
pH	GT65	0.0411	-0.0159	0.4715	0.4309	0.0047	0.0511763	Mineral
pH	GT66	19.8555	0.8531	0.0868	0.0165	0.2865	0.5661573	Mineral
pH	GT69	0.7751	-0.0682	0.0159	-0.0598	0.6544	0.8584218	Mineral
pH	GT68	0.0223	-0.0035	0.058	-0.0145	0.3873	0.693184	Mineral
pH	GT7	5.4876	-0.3652	0.3341	0.2829	0.024	0.1086981	Mineral
pH	GT72	0.0008	-0.0002	0.0105	-0.0656	0.7158	0.8764244	Mineral
pH	GT73	0.0663	0.0087	0.0224	-0.0528	0.5946	0.8305125	Mineral
pH	GT71	1.4059	-0.051	0.0143	-0.0615	0.6712	0.8630415	Mineral
pH	GT70	0.7826	0.0293	0.0399	-0.034	0.4754	0.7566331	Mineral
pH	GT74	0.8924	-0.0736	0.0658	-0.0061	0.3562	0.6665809	Mineral
pH	GT75	0.9563	-0.0147	0.0066	-0.0699	0.7742	0.8996393	Mineral
pH	GT76	2.3528	0.2309	0.1511	0.0858	0.1521	0.3753963	Mineral
pH	GT77	0.9469	0.059	0.0907	0.0207	0.2754	0.5515999	Mineral
pH	GT78	0.0018	-0.0006	0.0219	-0.0533	0.5987	0.8305125	Mineral
pH	GT8	11.7518	0.5754	0.0507	-0.0223	0.4197	0.7118663	Mineral
pH	GT80	5.6377	0.0056	0.00E+00	-0.0769	0.9808	0.9873015	Mineral
pH	GT82	0.4951	0.0561	0.1431	0.0772	0.1644	0.3820039	Mineral
pH	GT84	296.2703	11.4755	0.0383	-0.0357	0.4848	0.7566331	Mineral
pH	GT81	24.419	0.1534	0.002	-0.0747	0.873	0.9485978	Mineral
pH	GT83	171.2538	0.6556	0.0023	-0.0745	0.8656	0.9450886	Mineral
pH	GT85	3.2912	-0.6763	0.2578	0.2007	0.0533	0.1998446	Mineral
pH	GT87	16.9111	-1.1659	0.0675	-0.0043	0.3498	0.6651927	Mineral
pH	GT88	0.0259	-0.0182	0.6064	0.5761	0.0006	0.0156821	Mineral
pH	GT89	5.7924	-0.4246	0.0653	-0.0066	0.3579	0.6665809	Mineral
pH	GT90	2.475	-0.2458	0.0419	-0.0318	0.4645	0.7566331	Mineral
pH	GT9	175.0926	10.7031	0.4796	0.4396	0.0042	0.0480875	Mineral
pH	GT91	0.0159	0.0041	0.0535	-0.0194	0.407	0.7094816	Mineral
pH	GT93	0.368	-0.0008	0.0003	-0.0766	0.9508	0.9741082	Mineral
pH	GT94	0.7572	-0.1144	0.2619	0.2051	0.0512	0.1946376	Mineral
pH	GT92	0.0971	-0.0163	0.0575	-0.015	0.3894	0.693184	Mineral
pH	GT95	7.0408	-0.1366	0.0273	-0.0475	0.5561	0.8258692	Mineral
pH	PL1	18.5567	0.9397	0.0159	-0.0598	0.654	0.8584218	Mineral
pH	PL10	4.7494	-0.047	0.0004	-0.0765	0.9425	0.969851	Mineral
pH	PL11	3.638	0.1845	0.0109	-0.0652	0.7115	0.8764244	Mineral
pH	PL12	23.2607	0.6737	0.0508	-0.0222	0.4194	0.7118663	Mineral
pH	PL13	0.0005	-0.001	0.3332	0.2819	0.0243	0.1086981	Mineral
pH	PL14	1.1325	0.3676	0.405	0.3593	0.0107	0.0764343	Mineral
pH	PL15	0.3857	0.1174	0.2894	0.2347	0.0386	0.1562462	Mineral
pH	PL17	1.8343	0.4232	0.1805	0.1175	0.1144	0.3154333	Mineral
pH	PL18	0.0042	-0.0017	0.1107	0.0423	0.2257	0.4800404	Mineral

pH	PL16	0.52	-0.056	0.2019	0.1405	0.0929	0.2726595	Mineral
pH	PL2	0.1389	-0.016	0.1024	0.0334	0.2448	0.503742	Mineral
pH	PL20	0.0975	0.0041	0.0024	-0.0743	0.8625	0.9450886	Mineral
pH	PL21	0.4953	-0.1112	0.3859	0.3386	0.0134	0.080372	Mineral
pH	PL22	9.2334	0.3759	0.0516	-0.0213	0.4154	0.7118663	Mineral
pH	PL23	0.0157	-0.0008	0.0107	-0.0654	0.714	0.8764244	Mineral
pH	PL3	0.3266	0.0587	0.0256	-0.0493	0.5689	0.8302148	Mineral
pH	PL4	15.4469	-1.7592	0.1667	0.1026	0.1308	0.3492367	Mineral
pH	PL5	1.6555	-0.9535	0.5893	0.5577	0.0008	0.0174451	Mineral
pH	PL6	2.3184	0.3076	0.0448	-0.0286	0.4487	0.7452168	Mineral
pH	PL7	3.659	0.1835	0.01	-0.0661	0.7228	0.8807338	Mineral
pH	PL8	2.5006	0.1089	0.0121	-0.0639	0.6964	0.8764244	Mineral
pH	PL9	33.1358	1.924	0.0383	-0.0357	0.4845	0.7566331	Mineral
C	AA1	161.1286	7.8777	0.1871	0.1245	0.1074	0.2226855	Mineral
C	AA11	0.406	0.1915	0.3826	0.3351	0.014	0.0487006	Mineral
C	AA10	6.1103	1.2991	0.5234	0.4868	0.0023	0.012511	Mineral
C	AA2	1.4848	0.3658	0.3954	0.3488	0.012	0.0438094	Mineral
C	AA3	426.2452	0.4959	0.0001	-0.0768	0.9703	0.974218	Mineral
C	AA4	11.2554	4.0281	0.944	0.9396	0.00E+00	2.05E-07	Mineral
C	AA5	53.5482	-5.7153	0.5163	0.4791	0.0025	0.0127831	Mineral
C	AA8	0.6569	0.1557	0.2314	0.1722	0.0695	0.1615203	Mineral
C	AA7	57.2187	1.1045	0.0142	-0.0616	0.6723	0.8609931	Mineral
C	AA6	30.8574	-1.3862	0.1177	0.0499	0.2106	0.3796479	Mineral
C	AA9	1.4632	0.4251	0.103	0.034	0.2434	0.427299	Mineral
C	CE1	216.8049	1.1397	0.0062	-0.0703	0.7809	0.8868504	Mineral
C	CE11	163.2288	-1.6168	0.0279	-0.0469	0.5518	0.7694753	Mineral
C	CE12	15.6874	2.558	0.347	0.2967	0.0209	0.0638659	Mineral
C	CE13	0.0362	0.0119	0.2052	0.1441	0.0899	0.1979817	Mineral
C	CE14	221.0157	3.7174	0.0903	0.0203	0.2765	0.4595432	Mineral
C	CE15	37.8269	5.2888	0.615	0.5854	0.0005	0.0045013	Mineral
C	CE2	2.5005	0.5567	0.1723	0.1086	0.1239	0.2488852	Mineral
C	CE3	26.5046	-0.2857	0.0006	-0.0763	0.9301	0.9536392	Mineral
C	CE4	378.0703	-18.0612	0.145	0.0792	0.1615	0.3024196	Mineral
C	CE16	1.0559	0.1907	0.301	0.2473	0.0342	0.0986107	Mineral
C	CE5	14.5299	0.411	0.0043	-0.0723	0.8173	0.9157923	Mineral
C	CE7	10.4421	1.1665	0.2254	0.1659	0.0737	0.1697283	Mineral
C	CE6	37.5291	5.7807	0.5905	0.559	0.0008	0.0060302	Mineral
C	CE8	22.1825	2.1914	0.1988	0.1372	0.0957	0.2039512	Mineral
C	CE9	94.9446	-0.4112	0.0094	-0.0668	0.7308	0.8797199	Mineral
C	GH100	0.6919	0.2583	0.5364	0.5008	0.0019	0.0108459	Mineral
C	GH10	53.2214	3.4106	0.2683	0.212	0.0479	0.1227721	Mineral
C	GH102	43.2037	-1.5371	0.2099	0.1491	0.0859	0.1921526	Mineral
C	GH101	0.8491	-0.0664	0.0338	-0.0405	0.5117	0.7339578	Mineral
C	GH103	113.5215	-4.8205	0.0948	0.0252	0.2641	0.446831	Mineral
C	GH104	5.515	-0.3527	0.1229	0.0555	0.2001	0.3666209	Mineral
C	GH105	25.0043	6.3514	0.6467	0.6196	0.0003	0.0030256	Mineral
C	GH106	43.4576	7.9651	0.6836	0.6593	0.0001	0.0016846	Mineral
C	GH107	0.1988	-0.0134	0.0086	-0.0676	0.7418	0.8797199	Mineral
C	GH108	6.4325	0.2815	0.1467	0.081	0.1588	0.2997491	Mineral
C	GH109	19.7839	0.7919	0.0957	0.0262	0.2618	0.446831	Mineral
C	GH11	13.636	2.3717	0.6559	0.6294	0.0003	0.0026443	Mineral
C	GH110	1.0855	0.2697	0.5633	0.5297	0.0013	0.0075654	Mineral
C	GH111	0.0484	0.0011	0.0015	-0.0753	0.8902	0.9408317	Mineral
C	GH112	0.065	0.025	0.2874	0.2326	0.0394	0.1063449	Mineral
C	GH113	8.5535	-0.092	0.0023	-0.0744	0.865	0.9278743	Mineral

C	GH114	11.4339	-0.9806	0.1104	0.042	0.2263	0.4028311	Mineral
C	GH115	15.0153	3.7804	0.4848	0.4451	0.0039	0.0173248	Mineral
C	GH116	40.527	3.0292	0.1515	0.0863	0.1515	0.2925728	Mineral
C	GH117	3.5852	0.2098	0.0415	-0.0322	0.4665	0.6888452	Mineral
C	GH118	0.0033	-0.0003	0.0072	-0.0692	0.7636	0.8838137	Mineral
C	GH119	3.1764	-0.2434	0.0501	-0.023	0.4228	0.6354413	Mineral
C	GH12	8.1909	1.5404	0.3855	0.3383	0.0135	0.0477258	Mineral
C	GH120	1.8095	0.2079	0.04	-0.0339	0.475	0.6971912	Mineral
C	GH121	1.9359	-0.0642	0.0023	-0.0744	0.8648	0.9278743	Mineral
C	GH123	2.2624	-0.3964	0.27	0.2139	0.0471	0.1219077	Mineral
C	GH124	0.0624	-0.0182	0.2561	0.1989	0.0543	0.1375634	Mineral
C	GH125	10.2003	1.6764	0.4825	0.4427	0.0041	0.0175566	Mineral
C	GH126	0.0079	0.0013	0.0166	-0.0591	0.6476	0.8409035	Mineral
C	GH127	40.8673	4.5963	0.4505	0.4082	0.0062	0.0261304	Mineral
C	GH128	6.089	1.6473	0.6271	0.5984	0.0004	0.0038881	Mineral
C	GH129	0.3723	0.0093	0.0037	-0.073	0.8301	0.9171603	Mineral
C	GH13	1511.7028	28.629	0.0512	-0.0218	0.4176	0.6313742	Mineral
C	GH130	83.298	2.5359	0.0422	-0.0315	0.4629	0.6874871	Mineral
C	GH131	0.1722	0.0775	0.3571	0.3076	0.0186	0.0592437	Mineral
C	GH132	0.6932	0.3213	0.4202	0.3756	0.009	0.0356768	Mineral
C	GH133	77.1918	-7.1564	0.5636	0.5301	0.0013	0.0075654	Mineral
C	GH14	0.4871	0.0697	0.4419	0.399	0.0069	0.0282023	Mineral
C	GH17	62.1908	-1.2961	0.0206	-0.0547	0.6097	0.8054545	Mineral
C	GH15	404.3419	10.7962	0.0672	-0.0045	0.3507	0.5606505	Mineral
C	GH18	240.9138	14.1574	0.2335	0.1746	0.068	0.1610647	Mineral
C	GH19	17.4844	0.9319	0.0551	-0.0175	0.3995	0.619031	Mineral
C	GH2	363.2682	28.4787	0.4493	0.4069	0.0062	0.0261304	Mineral
C	GH20	98.8613	4.3827	0.2461	0.1881	0.06	0.1466847	Mineral
C	GH23	402.7243	-3.1066	0.0088	-0.0675	0.7396	0.8797199	Mineral
C	GH24	16.0614	-0.0503	0.0008	-0.076	0.9195	0.9498132	Mineral
C	GH25	20.5589	0.4049	0.0072	-0.0692	0.7637	0.8838137	Mineral
C	GH26	21.4128	1.885	0.1553	0.0904	0.146	0.2841595	Mineral
C	GH27	70.6768	12.4313	0.6923	0.6687	0.0001	0.0014979	Mineral
C	GH28	177.5732	23.3767	0.7733	0.7559	0.00E+00	0.0004367	Mineral
C	GH29	104.4902	6.5317	0.2803	0.2249	0.0424	0.1132271	Mineral
C	GH3	550.2428	51.5042	0.6031	0.5726	0.0007	0.0051868	Mineral
C	GH30	48.4301	6.0635	0.6283	0.5997	0.0004	0.0038881	Mineral
C	GH31	125.157	13.8645	0.4856	0.4461	0.0039	0.0173248	Mineral
C	GH33	63.7208	-3.6269	0.1477	0.0821	0.1573	0.2993705	Mineral
C	GH36	81.126	10.7816	0.6934	0.6698	0.0001	0.0014979	Mineral
C	GH32	31.8356	-0.8354	0.1171	0.0492	0.2118	0.3796479	Mineral
C	GH37	36.4506	3.0431	0.1636	0.0993	0.1348	0.266417	Mineral
C	GH39	81.1725	7.972	0.7358	0.7154	0.00E+00	0.0009053	Mineral
C	GH38	103.1959	17.2391	0.7736	0.7562	0.00E+00	0.0004367	Mineral
C	GH4	145.5656	4.1209	0.0253	-0.0497	0.5711	0.7845327	Mineral
C	GH42	42.3015	3.9807	0.5877	0.556	0.0009	0.0061326	Mineral
C	GH43	105.7244	15.6715	0.297	0.243	0.0356	0.1002402	Mineral
C	GH44	24.2141	2.3311	0.4175	0.3727	0.0093	0.0357405	Mineral
C	GH45	0.4034	0.1025	0.3176	0.2651	0.0287	0.0847195	Mineral
C	GH47	15.3148	1.4932	0.2883	0.2336	0.039	0.1063449	Mineral
C	GH46	2.3583	-0.015	0.0001	-0.0768	0.9678	0.974218	Mineral
C	GH48	9.3733	0.8088	0.2319	0.1728	0.0692	0.1615203	Mineral
C	GH49	0.174	0.0138	0.0049	-0.0716	0.8033	0.9058306	Mineral
C	GH5	169.7639	8.3514	0.0928	0.023	0.2697	0.4513517	Mineral
C	GH50	7.8367	-0.4915	0.0521	-0.0208	0.4133	0.6313742	Mineral

C	GH51	100.2913	12.6508	0.7239	0.7026	1.00E-04	0.0011043	Mineral
C	GH54	16.7488	6.998	0.7757	0.7585	0.00E+00	0.0004367	Mineral
C	GH53	54.6684	4.9353	0.365	0.3162	0.0171	0.0563497	Mineral
C	GH55	98.5985	17.2182	0.6236	0.5947	0.0005	0.0039992	Mineral
C	GH56	0.003	0.0016	0.2001	0.1386	0.0946	0.2039512	Mineral
C	GH57	155.9399	-6.4788	0.1987	0.137	0.0959	0.2039512	Mineral
C	GH58	0.0426	-0.0023	0.0062	-0.0702	0.7798	0.8868504	Mineral
C	GH59	6.4703	2.3832	0.6395	0.6117	0.0003	0.0033364	Mineral
C	GH6	17.654	2.9507	0.3024	0.2488	0.0337	0.098313	Mineral
C	GH62	3.2335	0.2521	0.0333	-0.0411	0.5152	0.7347136	Mineral
C	GH63	14.1513	1.9429	0.2736	0.2177	0.0454	0.1199776	Mineral
C	GH64	18.7715	2.107	0.3404	0.2897	0.0224	0.0677781	Mineral
C	GH65	100.9432	2.6817	0.0117	-0.0643	0.7013	0.8698254	Mineral
C	GH66	1.5117	0.0899	0.0115	-0.0645	0.7035	0.8698254	Mineral
C	GH68	0.2737	0.0094	0.0248	-0.0502	0.5751	0.7845327	Mineral
C	GH7	0.661	0.253	0.1807	0.1177	0.1142	0.2350302	Mineral
C	GH70	0.0411	0.0038	0.0127	-0.0633	0.6893	0.8698254	Mineral
C	GH71	3.5602	1.1499	0.4244	0.3801	0.0085	0.0344724	Mineral
C	GH72	30.0334	1.5453	0.1745	0.111	0.1213	0.2474516	Mineral
C	GH73	39.5823	0.2367	0.0033	-0.0733	0.8381	0.9186418	Mineral
C	GH75	2.2715	0.2132	0.1071	0.0384	0.2338	0.4132922	Mineral
C	GH74	12.8907	1.1874	0.2144	0.1539	0.0822	0.1858792	Mineral
C	GH76	13.3927	2.6765	0.3583	0.309	0.0184	0.0591872	Mineral
C	GH78	148.9518	12.9003	0.5265	0.4901	0.0022	0.0122679	Mineral
C	GH79	25.909	2.1925	0.1702	0.1063	0.1265	0.2519918	Mineral
C	GH8	19.2424	1.184	0.049	-0.0242	0.4281	0.6395417	Mineral
C	GH80	0.0028	-0.0014	0.0282	-0.0465	0.5493	0.7694753	Mineral
C	GH81	6.0259	0.353	0.0353	-0.0389	0.5026	0.7275282	Mineral
C	GH84	4.7774	0.2677	0.0944	0.0248	0.2653	0.446831	Mineral
C	GH85	0.6451	0.0303	0.0102	-0.0659	0.7197	0.8727639	Mineral
C	GH86	1.7515	-0.1151	0.0673	-0.0044	0.3504	0.5606505	Mineral
C	GH87	64.8133	6.0485	0.5206	0.4837	0.0024	0.012511	Mineral
C	GH88	24.9678	-0.2805	0.0152	-0.0606	0.6618	0.8518755	Mineral
C	GH89	6.8897	1.8867	0.7038	0.681	1.00E-04	0.0012926	Mineral
C	GH91	0.106	-0.0084	0.0124	-0.0636	0.6928	0.8698254	Mineral
C	GH90	0.0045	-0.0002	0.0015	-0.0753	0.8921	0.9408317	Mineral
C	GH9	51.5097	5.0354	0.1476	0.082	0.1574	0.2993705	Mineral
C	GH92	91.0695	16.2945	0.3628	0.3138	0.0175	0.0570403	Mineral
C	GH93	22.1339	2.9312	0.0515	-0.0215	0.4161	0.6313742	Mineral
C	GH94	27.0425	3.2281	0.2985	0.2445	0.0351	0.1001383	Mineral
C	GH95	48.6912	4.299	0.2178	0.1576	0.0795	0.1814116	Mineral
C	GH96	0.5474	-0.0604	0.0234	-0.0517	0.5859	0.7856963	Mineral
C	GH97	32.6174	0.5735	0.0071	-0.0693	0.7652	0.8838137	Mineral
C	GH98	0.3397	-0.0355	0.1931	0.131	0.1012	0.2135037	Mineral
C	GH99	13.1032	-0.318	0.0121	-0.0639	0.6965	0.8698254	Mineral
C	GT1	153.6974	7.4853	0.0542	-0.0185	0.4035	0.6213268	Mineral
C	GT10	2.4352	0.2568	0.3694	0.3209	0.0162	0.0550803	Mineral
C	GT12	0.0482	0.0019	0.0086	-0.0677	0.743	0.8797199	Mineral
C	GT14	2.0647	0.2494	0.2092	0.1483	0.0865	0.1921526	Mineral
C	GT11	4.9506	0.1989	0.0628	-0.0093	0.3675	0.5802198	Mineral
C	GT13	0.1413	0.075	0.7687	0.751	0.00E+00	0.0004483	Mineral
C	GT15	1.84	0.7793	0.4979	0.4593	0.0033	0.0153011	Mineral
C	GT16	0.0336	0.0279	0.8098	0.7951	0.00E+00	0.0002466	Mineral
C	GT17	1.0318	0.0713	0.1288	0.0617	0.189	0.3488811	Mineral
C	GT18	0.0082	0.0071	0.5763	0.5437	0.001	0.0069912	Mineral

C	GT2	1841.616	-10.7808	0.0251	-0.0499	0.5726	0.7845327	Mineral
C	GT19	83.4375	0.0913	0.0004	-0.0765	0.9435	0.9548719	Mineral
C	GT20	150.7734	-0.2145	0.0015	-0.0753	0.8902	0.9408317	Mineral
C	GT21	72.0818	4.0403	0.0611	-0.0111	0.3745	0.5875088	Mineral
C	GT22	4.3521	0.7817	0.3681	0.3195	0.0165	0.0551678	Mineral
C	GT23	0.2031	0.0651	0.7042	0.6815	1.00E-04	0.0012926	Mineral
C	GT24	2.657	0.6957	0.66	0.6338	0.0002	0.0025438	Mineral
C	GT25	5.9103	0.7036	0.5012	0.4628	0.0031	0.0148927	Mineral
C	GT26	133.0274	6.1101	0.3905	0.3436	0.0127	0.0456984	Mineral
C	GT27	13.0687	0.9785	0.5685	0.5353	0.0012	0.0075049	Mineral
C	GT28	177.3669	-0.2521	0.0009	-0.0759	0.9133	0.9472956	Mineral
C	GT29	3.9302	-0.1906	0.0991	0.0298	0.2532	0.4412718	Mineral
C	GT30	120.1726	0.2075	0.0011	-0.0757	0.9058	0.9449128	Mineral
C	GT3	2.7042	0.6311	0.3966	0.3502	0.0119	0.0438094	Mineral
C	GT33	1.8007	0.0328	0.0041	-0.0725	0.821	0.9158962	Mineral
C	GT34	0.89	0.3207	0.4039	0.358	0.0109	0.0414249	Mineral
C	GT32	12.395	0.338	0.0289	-0.0458	0.5448	0.7681942	Mineral
C	GT31	1.3616	0.6683	0.5635	0.5299	0.0013	0.0075654	Mineral
C	GT35	348.7674	-1.4289	0.0011	-0.0757	0.9048	0.9449128	Mineral
C	GT37	0.0045	0.0028	0.3791	0.3313	0.0145	0.0499955	Mineral
C	GT39	20.8559	2.1195	0.1423	0.0763	0.1658	0.3082157	Mineral
C	GT4	1887.0784	-27.3599	0.0326	-0.0418	0.5198	0.7370955	Mineral
C	GT41	420.3074	0.8475	0.0005	-0.0764	0.9384	0.9536392	Mineral
C	GT40	0.0012	-0.0005	0.035	-0.0392	0.5043	0.7275282	Mineral
C	GT42	0.0617	0.0442	0.7115	0.6893	1.00E-04	0.0012189	Mineral
C	GT43	0.0665	0.0391	0.5734	0.5406	0.0011	0.0071262	Mineral
C	GT47	67.1447	6.421	0.2457	0.1876	0.0603	0.1466847	Mineral
C	GT45	0.2617	-0.0039	0.0049	-0.0717	0.8048	0.9058306	Mineral
C	GT44	0.0225	-0.005	0.0566	-0.0159	0.3931	0.6128204	Mineral
C	GT48	3.0789	1.471	0.6824	0.658	0.0001	0.0016846	Mineral
C	GT49	0.176	0.0903	0.8007	0.7854	0.00E+00	0.000279	Mineral
C	GT5	111.8839	-1.2075	0.0233	-0.0519	0.5874	0.7856963	Mineral
C	GT50	0.6419	0.2677	0.5209	0.4841	0.0024	0.012511	Mineral
C	GT53	23.5174	1.1611	0.0179	-0.0577	0.6346	0.8315296	Mineral
C	GT52	0	0	#VALUE!	#VALUE!	#VALUE!	NA	Mineral
C	GT51	615.404	-20.8728	0.1609	0.0963	0.1384	0.2714642	Mineral
C	GT54	0.0278	0.0256	0.8329	0.8201	0.00E+00	0.0001309	Mineral
C	GT57	1.3329	0.6495	0.6007	0.57	0.0007	0.0052374	Mineral
C	GT56	0.3995	0.044	0.3489	0.2988	0.0204	0.0632863	Mineral
C	GT55	2.8539	0.7568	0.5793	0.547	0.001	0.0068368	Mineral
C	GT58	0.5128	0.2117	0.5089	0.4712	0.0028	0.0138999	Mineral
C	GT6	0.334	-0.0264	0.0262	-0.0487	0.5646	0.7829198	Mineral
C	GT59	0.3598	0.166	0.5192	0.4822	0.0024	0.012511	Mineral
C	GT60	1.8074	0.6795	0.7214	0.6999	1.00E-04	0.0011043	Mineral
C	GT61	0.3572	0.0968	0.495	0.4561	0.0034	0.0156396	Mineral
C	GT62	2.1958	0.3379	0.3542	0.3046	0.0193	0.0603969	Mineral
C	GT64	0.9005	0.0428	0.0375	-0.0365	0.489	0.7135525	Mineral
C	GT65	0.0411	0.0165	0.5074	0.4695	0.0029	0.0139267	Mineral
C	GT66	19.8555	-0.2608	0.0081	-0.0682	0.7495	0.8802298	Mineral
C	GT69	0.7751	0.4027	0.5544	0.5202	0.0015	0.0084694	Mineral
C	GT68	0.0223	0.0092	0.402	0.356	0.0111	0.041722	Mineral
C	GT7	5.4876	0.306	0.2346	0.1757	0.0673	0.1608848	Mineral
C	GT72	0.0008	0.0002	0.0163	-0.0593	0.6499	0.8409035	Mineral
C	GT73	0.0663	0.0254	0.1914	0.1292	0.1029	0.2152144	Mineral
C	GT71	1.4059	0.2316	0.2952	0.2409	0.0363	0.1002402	Mineral

C	GT70	0.7826	0.0727	0.2448	0.1867	0.0608	0.1466847	Mineral
C	GT74	0.8924	-0.0161	0.0031	-0.0736	0.8431	0.9200304	Mineral
C	GT75	0.9563	0.006	0.0011	-0.0758	0.9073	0.9449128	Mineral
C	GT76	2.3528	-0.3229	0.2957	0.2416	0.0361	0.1002402	Mineral
C	GT77	0.9469	-0.0983	0.2518	0.1942	0.0567	0.1422946	Mineral
C	GT78	0.0018	-0.0006	0.0177	-0.0578	0.6361	0.8315296	Mineral
C	GT8	11.7518	0.6906	0.073	0.0017	0.33	0.5344246	Mineral
C	GT80	5.6377	0.4047	0.2451	0.1871	0.0606	0.1466847	Mineral
C	GT82	0.4951	-0.0377	0.0648	-0.0072	0.36	0.5719487	Mineral
C	GT84	296.2703	1.3563	0.0005	-0.0763	0.9348	0.9536392	Mineral
C	GT81	24.419	0.2832	0.007	-0.0694	0.7676	0.8838137	Mineral
C	GT83	171.2538	1.39	0.0103	-0.0658	0.7191	0.8727639	Mineral
C	GT85	3.2912	0.1938	0.0212	-0.0541	0.6049	0.8033956	Mineral
C	GT87	16.9111	2.0034	0.1992	0.1376	0.0953	0.2039512	Mineral
C	GT88	0.0259	0.0122	0.2705	0.2143	0.0469	0.1219077	Mineral
C	GT89	5.7924	0.1681	0.0102	-0.0659	0.7198	0.8727639	Mineral
C	GT90	2.475	0.9399	0.612	0.5822	0.0006	0.0045893	Mineral
C	GT9	175.0926	-5.3226	0.1186	0.0508	0.2087	0.3796479	Mineral
C	GT91	0.0159	0.0019	0.012	-0.064	0.6977	0.8698254	Mineral
C	GT93	0.368	0.0126	0.0792	0.0083	0.3097	0.5047581	Mineral
C	GT94	0.7572	0.0931	0.1734	0.1098	0.1226	0.2482387	Mineral
C	GT92	0.0971	0.0594	0.7603	0.7419	0.00E+00	0.0005168	Mineral
C	GT95	7.0408	-0.466	0.3179	0.2655	0.0286	0.0847195	Mineral
C	PL1	18.5567	-0.1743	0.0005	-0.0763	0.934	0.9536392	Mineral
C	PL10	4.7494	0.7127	0.0956	0.0261	0.262	0.446831	Mineral
C	PL11	3.638	-0.0949	0.0029	-0.0738	0.8494	0.9203361	Mineral
C	PL12	23.2607	-0.0273	1.00E-04	-0.0768	0.9743	0.9742692	Mineral
C	PL13	0.0005	0.0017	0.9584	0.9552	0.00E+00	5.87E-08	Mineral
C	PL14	1.1325	0.0256	0.002	-0.0748	0.8756	0.9352313	Mineral
C	PL15	0.3857	-0.021	0.0092	-0.067	0.7333	0.8797199	Mineral
C	PL17	1.8343	-0.1012	0.0103	-0.0658	0.7187	0.8727639	Mineral
C	PL18	0.0042	-0.0008	0.0237	-0.0514	0.5835	0.7856963	Mineral
C	PL16	0.52	-0.0075	0.0036	-0.073	0.8307	0.9171603	Mineral
C	PL2	0.1389	0.0148	0.0873	0.0171	0.285	0.4701327	Mineral
C	PL20	0.0975	0.0098	0.0134	-0.0625	0.6808	0.8674254	Mineral
C	PL21	0.4953	0.0272	0.0231	-0.052	0.5885	0.7856963	Mineral
C	PL22	9.2334	1.0691	0.4175	0.3727	0.0093	0.0357405	Mineral
C	PL23	0.0157	-0.0006	0.0065	-0.07	0.7759	0.8868504	Mineral
C	PL3	0.3266	0.0218	0.0035	-0.0731	0.8331	0.9171603	Mineral
C	PL4	15.4469	4.0957	0.9037	0.8963	0.00E+00	4.69E-06	Mineral
C	PL5	1.6555	1.05	0.7145	0.6925	1.00E-04	0.0012128	Mineral
C	PL6	2.3184	-0.1303	0.0081	-0.0683	0.7505	0.8802298	Mineral
C	PL7	3.659	0.5658	0.0951	0.0255	0.2634	0.446831	Mineral
C	PL8	2.5006	0.2917	0.0868	0.0165	0.2866	0.4701327	Mineral
C	PL9	33.1358	0.5229	0.0028	-0.0739	0.8507	0.9203361	Mineral
N	AA1	161.1286	8.5256	0.2191	0.159	0.0785	0.1949722	Mineral
N	AA11	0.406	0.2141	0.4781	0.438	0.0043	0.0211999	Mineral
N	AA10	6.1103	1.296	0.5209	0.4841	0.0024	0.0132912	Mineral
N	AA2	1.4848	0.3817	0.4305	0.3867	0.0079	0.0319678	Mineral
N	AA3	426.2452	7.4683	0.0251	-0.0499	0.5731	0.7691847	Mineral
N	AA4	11.2554	3.8231	0.8503	0.8388	0.00E+00	9.60E-05	Mineral
N	AA5	53.5482	-4.8984	0.3792	0.3315	0.0145	0.0520576	Mineral
N	AA8	0.6569	0.1673	0.2673	0.2109	0.0484	0.1307341	Mineral
N	AA7	57.2187	1.506	0.0264	-0.0485	0.5629	0.7637489	Mineral
N	AA6	30.8574	-1.1605	0.0825	0.0119	0.2992	0.5109067	Mineral

N	AA9	1.4632	0.4676	0.1246	0.0573	0.1968	0.3742122	Mineral
N	CE1	216.8049	0.3077	0.0004	-0.0764	0.9402	0.9515913	Mineral
N	CE11	163.2288	-2.7606	0.0813	0.0107	0.3028	0.5135665	Mineral
N	CE12	15.6874	2.5012	0.3317	0.2803	0.0246	0.0798396	Mineral
N	CE13	0.0362	0.0099	0.1415	0.0755	0.1669	0.3405961	Mineral
N	CE14	221.0157	1.586	0.0164	-0.0592	0.6488	0.8167271	Mineral
N	CE15	37.8269	4.6299	0.4713	0.4307	0.0047	0.0218554	Mineral
N	CE2	2.5005	0.5518	0.1693	0.1054	0.1276	0.273762	Mineral
N	CE3	26.5046	0.2453	0.0005	-0.0764	0.94	0.9515913	Mineral
N	CE4	378.0703	-11.5775	0.0596	-0.0128	0.3807	0.596748	Mineral
N	CE16	1.0559	0.1798	0.2675	0.2111	0.0484	0.1307341	Mineral
N	CE5	14.5299	0.3919	0.0039	-0.0728	0.8256	0.9049381	Mineral
N	CE7	10.4421	1.11	0.2041	0.1429	0.0909	0.2203974	Mineral
N	CE6	37.5291	5.0343	0.4479	0.4054	0.0064	0.0279698	Mineral
N	CE8	22.1825	1.9181	0.1523	0.0871	0.1504	0.3167313	Mineral
N	CE9	94.9446	-0.6645	0.0246	-0.0505	0.5768	0.7698491	Mineral
N	GH100	0.6919	0.2846	0.6511	0.6242	0.0003	0.0045409	Mineral
N	GH10	53.2214	2.9714	0.2037	0.1424	0.0913	0.2203974	Mineral
N	GH102	43.2037	-1.4228	0.1798	0.1167	0.1152	0.2514111	Mineral
N	GH101	0.8491	-0.046	0.0162	-0.0594	0.6508	0.8167271	Mineral
N	GH103	113.5215	-2.5525	0.0266	-0.0483	0.5615	0.7637489	Mineral
N	GH104	5.515	-0.3365	0.1119	0.0436	0.2229	0.4145092	Mineral
N	GH105	25.0043	5.8455	0.5478	0.513	0.0016	0.0098212	Mineral
N	GH106	43.4576	7.231	0.5634	0.5299	0.0013	0.0081239	Mineral
N	GH107	0.1988	-0.0095	0.0044	-0.0722	0.8153	0.9010455	Mineral
N	GH108	6.4325	0.2636	0.1286	0.0616	0.1894	0.3628034	Mineral
N	GH109	19.7839	0.6097	0.0567	-0.0158	0.3926	0.6008324	Mineral
N	GH11	13.636	2.3422	0.6397	0.6119	0.0003	0.0045409	Mineral
N	GH110	1.0855	0.2786	0.6011	0.5704	0.0007	0.0053427	Mineral
N	GH111	0.0484	0.0024	0.0081	-0.0682	0.7497	0.867215	Mineral
N	GH112	0.065	0.02	0.1846	0.1219	0.1099	0.2442175	Mineral
N	GH113	8.5535	-0.0511	0.0007	-0.0762	0.9249	0.9515913	Mineral
N	GH114	11.4339	-0.8034	0.0741	0.0029	0.3263	0.5423829	Mineral
N	GH115	15.0153	3.6574	0.4537	0.4117	0.0059	0.0264601	Mineral
N	GH116	40.527	2.3099	0.0881	0.018	0.2827	0.4859416	Mineral
N	GH117	3.5852	0.1281	0.0155	-0.0603	0.6588	0.8186183	Mineral
N	GH118	0.0033	-0.0003	0.012	-0.0641	0.6981	0.8422123	Mineral
N	GH119	3.1764	-0.2361	0.0471	-0.0262	0.4373	0.6455927	Mineral
N	GH12	8.1909	1.4804	0.3561	0.3065	0.0189	0.0639731	Mineral
N	GH120	1.8095	0.0765	0.0054	-0.0711	0.7943	0.8978707	Mineral
N	GH121	1.9359	-0.0238	0.0003	-0.0766	0.9498	0.9574088	Mineral
N	GH123	2.2624	-0.367	0.2315	0.1724	0.0694	0.1760213	Mineral
N	GH124	0.0624	-0.0155	0.186	0.1234	0.1085	0.2432262	Mineral
N	GH125	10.2003	1.483	0.3776	0.3297	0.0148	0.0522931	Mineral
N	GH126	0.0079	0.0002	0.0005	-0.0764	0.9395	0.9515913	Mineral
N	GH127	40.8673	3.9879	0.3391	0.2883	0.0227	0.0760817	Mineral
N	GH128	6.089	1.6862	0.6571	0.6308	0.0002	0.0045409	Mineral
N	GH129	0.3723	-0.008	0.0027	-0.074	0.8533	0.9165627	Mineral
N	GH13	1511.7028	27.5745	0.0475	-0.0258	0.4354	0.6455927	Mineral
N	GH130	83.298	1.77	0.0205	-0.0548	0.6104	0.7847381	Mineral
N	GH131	0.1722	0.0821	0.401	0.3549	0.0113	0.0416036	Mineral
N	GH132	0.6932	0.3532	0.5078	0.4699	0.0029	0.0156602	Mineral
N	GH133	77.1918	-7.8569	0.6794	0.6547	0.0002	0.0035892	Mineral
N	GH14	0.4871	0.0728	0.4828	0.443	0.004	0.0206887	Mineral
N	GH17	62.1908	0.0906	0.0001	-0.0768	0.9717	0.9755541	Mineral

N	GH15	404.3419	7.2638	0.0304	-0.0441	0.534	0.7405553	Mineral
N	GH18	240.9138	13.1558	0.2017	0.1402	0.0931	0.2226023	Mineral
N	GH19	17.4844	0.8723	0.0483	-0.0249	0.4312	0.644242	Mineral
N	GH2	363.2682	24.1609	0.3234	0.2713	0.027	0.083615	Mineral
N	GH20	98.8613	3.4709	0.1543	0.0893	0.1475	0.3136714	Mineral
N	GH23	402.7243	-3.5958	0.0118	-0.0642	0.7002	0.8422123	Mineral
N	GH24	16.0614	-0.3139	0.0318	-0.0427	0.5248	0.7405553	Mineral
N	GH25	20.5589	0.364	0.0058	-0.0707	0.7871	0.8978707	Mineral
N	GH26	21.4128	1.8622	0.1516	0.0863	0.1514	0.3167313	Mineral
N	GH27	70.6768	11.58	0.6008	0.5701	0.0007	0.0053427	Mineral
N	GH28	177.5732	21.1477	0.6329	0.6046	0.0004	0.0045409	Mineral
N	GH29	104.4902	4.5146	0.1339	0.0673	0.1798	0.3499413	Mineral
N	GH3	550.2428	45.3738	0.4681	0.4272	0.0049	0.0223945	Mineral
N	GH30	48.4301	5.9529	0.6056	0.5753	0.0006	0.0053427	Mineral
N	GH31	125.157	11.4275	0.3299	0.2784	0.0251	0.0798396	Mineral
N	GH33	63.7208	-3.5407	0.1407	0.0746	0.1683	0.3405961	Mineral
N	GH36	81.126	9.77	0.5694	0.5363	0.0011	0.0077651	Mineral
N	GH32	31.8356	-0.928	0.1446	0.0788	0.1621	0.3335421	Mineral
N	GH37	36.4506	2.2624	0.0904	0.0205	0.2761	0.4779768	Mineral
N	GH39	81.1725	7.4125	0.6361	0.6081	0.0004	0.0045409	Mineral
N	GH38	103.1959	15.635	0.6364	0.6084	0.0004	0.0045409	Mineral
N	GH4	145.5656	6.4572	0.0622	-0.01	0.3702	0.5954641	Mineral
N	GH42	42.3015	3.7712	0.5275	0.4912	0.0022	0.0123602	Mineral
N	GH43	105.7244	14.6442	0.2594	0.2024	0.0525	0.1387073	Mineral
N	GH44	24.2141	2.1597	0.3583	0.309	0.0184	0.0632191	Mineral
N	GH45	0.4034	0.1026	0.3179	0.2655	0.0286	0.0853958	Mineral
N	GH47	15.3148	1.5169	0.2976	0.2435	0.0354	0.1022421	Mineral
N	GH46	2.3583	0.0087	0.00E+00	-0.0769	0.9814	0.9813849	Mineral
N	GH48	9.3733	0.8533	0.2581	0.201	0.0532	0.1390651	Mineral
N	GH49	0.174	0.0344	0.0306	-0.044	0.5329	0.7405553	Mineral
N	GH5	169.7639	8.7348	0.1015	0.0323	0.2472	0.4432486	Mineral
N	GH50	7.8367	-0.5101	0.0561	-0.0165	0.3954	0.6013325	Mineral
N	GH51	100.2913	11.2062	0.568	0.5347	0.0012	0.0077651	Mineral
N	GH54	16.7488	6.6685	0.7044	0.6817	1.00E-04	0.0022937	Mineral
N	GH53	54.6684	4.7226	0.3342	0.283	0.024	0.0791994	Mineral
N	GH55	98.5985	15.9602	0.5358	0.5001	0.0019	0.0114702	Mineral
N	GH56	0.003	0.0018	0.241	0.1826	0.0632	0.163461	Mineral
N	GH57	155.9399	-7.7282	0.2827	0.2275	0.0414	0.1166698	Mineral
N	GH58	0.0426	-0.0015	0.0029	-0.0738	0.8497	0.9165627	Mineral
N	GH59	6.4703	2.3076	0.5995	0.5687	0.0007	0.0053427	Mineral
N	GH6	17.654	2.9808	0.3086	0.2554	0.0316	0.0920922	Mineral
N	GH62	3.2335	0.2451	0.0315	-0.043	0.5271	0.7405553	Mineral
N	GH63	14.1513	1.9687	0.2809	0.2256	0.0421	0.1174844	Mineral
N	GH64	18.7715	2.0746	0.3301	0.2785	0.0251	0.0798396	Mineral
N	GH65	100.9432	0.8862	0.0013	-0.0755	0.8994	0.9400946	Mineral
N	GH66	1.5117	0.1758	0.044	-0.0296	0.4532	0.657511	Mineral
N	GH68	0.2737	0.0095	0.0252	-0.0498	0.5723	0.7691847	Mineral
N	GH7	0.661	0.2735	0.2111	0.1504	0.0849	0.2088946	Mineral
N	GH70	0.0411	0.0059	0.0307	-0.0439	0.5324	0.7405553	Mineral
N	GH71	3.5602	1.2894	0.5335	0.4977	0.002	0.0115824	Mineral
N	GH72	30.0334	1.6268	0.1934	0.1314	0.1009	0.2367264	Mineral
N	GH73	39.5823	0.1382	0.0011	-0.0757	0.9051	0.9400946	Mineral
N	GH75	2.2715	0.1997	0.094	0.0243	0.2664	0.464908	Mineral
N	GH74	12.8907	1.3182	0.2642	0.2076	0.05	0.1334626	Mineral
N	GH76	13.3927	2.7139	0.3684	0.3198	0.0164	0.0572425	Mineral

N	GH78	148.9518	12.6183	0.5037	0.4655	0.003	0.0162207	Mineral
N	GH79	25.909	1.9389	0.1331	0.0664	0.1812	0.3499413	Mineral
N	GH8	19.2424	0.8745	0.0267	-0.0482	0.5606	0.7637489	Mineral
N	GH80	0.0028	-0.002	0.0613	-0.0109	0.3737	0.5954641	Mineral
N	GH81	6.0259	0.4561	0.0589	-0.0135	0.3834	0.596748	Mineral
N	GH84	4.7774	0.2338	0.072	0.0006	0.3335	0.5470736	Mineral
N	GH85	0.6451	0.0209	0.0049	-0.0717	0.8052	0.9010455	Mineral
N	GH86	1.7515	-0.0948	0.0457	-0.0277	0.4443	0.6521743	Mineral
N	GH87	64.8133	6.3332	0.5707	0.5377	0.0011	0.0077651	Mineral
N	GH88	24.9678	-0.1861	0.0067	-0.0697	0.7721	0.8890291	Mineral
N	GH89	6.8897	1.8019	0.6419	0.6144	0.0003	0.0045409	Mineral
N	GH91	0.106	-0.0032	0.0018	-0.075	0.8797	0.9238328	Mineral
N	GH90	0.0045	0.0003	0.0022	-0.0746	0.8687	0.9226825	Mineral
N	GH9	51.5097	4.0159	0.0939	0.0242	0.2667	0.464908	Mineral
N	GH92	91.0695	15.3263	0.3209	0.2687	0.0277	0.0836962	Mineral
N	GH93	22.1339	3.4913	0.073	0.0017	0.33	0.5449386	Mineral
N	GH94	27.0425	2.2841	0.1494	0.084	0.1546	0.3207947	Mineral
N	GH95	48.6912	4.0694	0.1951	0.1332	0.0992	0.2350119	Mineral
N	GH96	0.5474	-0.0571	0.021	-0.0543	0.6066	0.7847381	Mineral
N	GH97	32.6174	0.3525	0.0027	-0.074	0.8545	0.9165627	Mineral
N	GH98	0.3397	-0.0295	0.1337	0.0671	0.1802	0.3499413	Mineral
N	GH99	13.1032	-0.2838	0.0096	-0.0666	0.7279	0.8589505	Mineral
N	GT1	153.6974	9.9168	0.0952	0.0256	0.2631	0.464908	Mineral
N	GT10	2.4352	0.237	0.3146	0.2618	0.0296	0.0874754	Mineral
N	GT12	0.0482	-0.0005	0.0005	-0.0764	0.9359	0.9515913	Mineral
N	GT14	2.0647	0.1998	0.1343	0.0677	0.1792	0.3499413	Mineral
N	GT11	4.9506	0.0967	0.0148	-0.0609	0.6654	0.8186462	Mineral
N	GT13	0.1413	0.0687	0.6451	0.6178	0.0003	0.0045409	Mineral
N	GT15	1.84	0.8725	0.6241	0.5952	0.0005	0.0047897	Mineral
N	GT16	0.0336	0.0268	0.7475	0.7281	0.00E+00	0.0008931	Mineral
N	GT17	1.0318	0.0644	0.1049	0.036	0.239	0.4399748	Mineral
N	GT18	0.0082	0.0066	0.4916	0.4525	0.0036	0.018756	Mineral
N	GT2	1841.616	-10.5851	0.0242	-0.0508	0.5797	0.7698491	Mineral
N	GT19	83.4375	-0.4454	0.0096	-0.0666	0.7289	0.8589505	Mineral
N	GT20	150.7734	-0.295	0.0029	-0.0738	0.8495	0.9165627	Mineral
N	GT21	72.0818	2.7969	0.0293	-0.0454	0.5421	0.7476037	Mineral
N	GT22	4.3521	0.8611	0.4466	0.404	0.0065	0.0279698	Mineral
N	GT23	0.2031	0.0605	0.6098	0.5798	0.0006	0.0053427	Mineral
N	GT24	2.657	0.697	0.6624	0.6364	0.0002	0.0045409	Mineral
N	GT25	5.9103	0.6366	0.4103	0.3649	0.0101	0.0378193	Mineral
N	GT26	133.0274	6.4374	0.4335	0.3899	0.0076	0.0313329	Mineral
N	GT27	13.0687	0.8926	0.473	0.4325	0.0046	0.0218554	Mineral
N	GT28	177.3669	-0.8361	0.0104	-0.0657	0.7175	0.8575682	Mineral
N	GT29	3.9302	-0.1618	0.0714	0.00E+00	0.3357	0.5471634	Mineral
N	GT30	120.1726	-0.4068	0.0043	-0.0723	0.8164	0.9010455	Mineral
N	GT3	2.7042	0.6292	0.3942	0.3476	0.0122	0.0444043	Mineral
N	GT33	1.8007	0.0771	0.0226	-0.0526	0.5932	0.7765958	Mineral
N	GT34	0.89	0.347	0.4727	0.4322	0.0046	0.0218554	Mineral
N	GT32	12.395	0.6425	0.1044	0.0355	0.2401	0.4399748	Mineral
N	GT31	1.3616	0.695	0.6095	0.5794	0.0006	0.0053427	Mineral
N	GT35	348.7674	-8.2885	0.0385	-0.0355	0.4834	0.693401	Mineral
N	GT37	0.0045	0.003	0.4196	0.375	0.009	0.0353864	Mineral
N	GT39	20.8559	2.3861	0.1803	0.1172	0.1147	0.2514111	Mineral
N	GT4	1887.0784	-21.3978	0.0199	-0.0555	0.6158	0.7847381	Mineral
N	GT41	420.3074	-5.1049	0.0173	-0.0583	0.6404	0.811782	Mineral

N	GT40	0.0012	-0.0006	0.0598	-0.0125	0.3797	0.596748	Mineral
N	GT42	0.0617	0.0453	0.7492	0.7299	0.00E+00	0.0008931	Mineral
N	GT43	0.0665	0.0339	0.4292	0.3853	0.008	0.0319748	Mineral
N	GT47	67.1447	6.9496	0.2878	0.233	0.0393	0.1119541	Mineral
N	GT45	0.2617	-0.0051	0.0084	-0.0679	0.7458	0.8666355	Mineral
N	GT44	0.0225	-0.0047	0.0503	-0.0227	0.4215	0.6334959	Mineral
N	GT48	3.0789	1.5469	0.7546	0.7358	0.00E+00	0.0008931	Mineral
N	GT49	0.176	0.0902	0.798	0.7824	0.00E+00	0.0003667	Mineral
N	GT5	111.8839	-1.915	0.0585	-0.0139	0.3852	0.596748	Mineral
N	GT50	0.6419	0.2885	0.605	0.5746	0.0006	0.0053427	Mineral
N	GT53	23.5174	1.0937	0.0159	-0.0598	0.6546	0.817455	Mineral
N	GT52	0	0	#VALUE!	#VALUE!	#VALUE!	NA	Mineral
N	GT51	615.404	-19.0323	0.1337	0.0671	0.1801	0.3499413	Mineral
N	GT54	0.0278	0.0246	0.7689	0.7511	0.00E+00	0.0007446	Mineral
N	GT57	1.3329	0.6741	0.6472	0.6201	0.0003	0.0045409	Mineral
N	GT56	0.3995	0.0364	0.238	0.1794	0.0651	0.1666522	Mineral
N	GT55	2.8539	0.7904	0.6318	0.6035	0.0004	0.0045409	Mineral
N	GT58	0.5128	0.2318	0.6099	0.5799	0.0006	0.0053427	Mineral
N	GT6	0.334	-0.0421	0.0665	-0.0053	0.3533	0.5721239	Mineral
N	GT59	0.3598	0.1781	0.5975	0.5665	0.0007	0.0053683	Mineral
N	GT60	1.8074	0.5947	0.5526	0.5181	0.0015	0.0093677	Mineral
N	GT61	0.3572	0.0951	0.478	0.4378	0.0043	0.0211999	Mineral
N	GT62	2.1958	0.3659	0.4153	0.3703	0.0095	0.0361538	Mineral
N	GT64	0.9005	0.0528	0.0573	-0.0152	0.3903	0.6008324	Mineral
N	GT65	0.0411	0.0154	0.4436	0.4008	0.0067	0.0285652	Mineral
N	GT66	19.8555	-0.3536	0.0149	-0.0609	0.6646	0.8186462	Mineral
N	GT69	0.7751	0.4142	0.5864	0.5546	0.0009	0.0062661	Mineral
N	GT68	0.0223	0.0083	0.3232	0.2712	0.027	0.083615	Mineral
N	GT7	5.4876	0.2137	0.1144	0.0463	0.2175	0.4073503	Mineral
N	GT72	0.0008	0.0004	0.0442	-0.0293	0.4519	0.657511	Mineral
N	GT73	0.0663	0.0254	0.1915	0.1293	0.1028	0.2388969	Mineral
N	GT71	1.4059	0.242	0.3222	0.27	0.0273	0.083615	Mineral
N	GT70	0.7826	0.0771	0.2756	0.2199	0.0445	0.1226755	Mineral
N	GT74	0.8924	-0.0209	0.0053	-0.0712	0.7963	0.8978707	Mineral
N	GT75	0.9563	-0.0044	0.0006	-0.0763	0.9311	0.9515913	Mineral
N	GT76	2.3528	-0.2459	0.1715	0.1077	0.1249	0.270329	Mineral
N	GT77	0.9469	-0.0845	0.1862	0.1236	0.1083	0.2432262	Mineral
N	GT78	0.0018	-0.0006	0.0224	-0.0527	0.594	0.7765958	Mineral
N	GT8	11.7518	0.7917	0.096	0.0264	0.2612	0.464908	Mineral
N	GT80	5.6377	0.3525	0.186	0.1233	0.1085	0.2432262	Mineral
N	GT82	0.4951	-0.0366	0.061	-0.0112	0.3748	0.5954641	Mineral
N	GT84	296.2703	-2.9152	0.0025	-0.0743	0.8604	0.9189945	Mineral
N	GT81	24.419	0.1892	0.0031	-0.0736	0.8436	0.9165627	Mineral
N	GT83	171.2538	1.3028	0.009	-0.0672	0.7362	0.8634471	Mineral
N	GT85	3.2912	0.2048	0.0236	-0.0515	0.5843	0.7719458	Mineral
N	GT87	16.9111	2.1172	0.2225	0.1627	0.0759	0.190423	Mineral
N	GT88	0.0259	0.0101	0.1877	0.1253	0.1067	0.2432262	Mineral
N	GT89	5.7924	0.165	0.0099	-0.0663	0.7248	0.8589505	Mineral
N	GT90	2.475	0.953	0.6292	0.6007	0.0004	0.0045549	Mineral
N	GT9	175.0926	-5.4123	0.1226	0.0551	0.2007	0.3787168	Mineral
N	GT91	0.0159	0.0036	0.0411	-0.0327	0.4687	0.6760725	Mineral
N	GT93	0.368	0.0144	0.1027	0.0337	0.2441	0.4407953	Mineral
N	GT94	0.7572	0.0832	0.1386	0.0724	0.1717	0.3447718	Mineral
N	GT92	0.0971	0.061	0.8035	0.7884	0.00E+00	0.0003667	Mineral
N	GT95	7.0408	-0.5476	0.4389	0.3957	0.0071	0.0297829	Mineral

N	PL1	18.5567	-0.2475	0.0011	-0.0757	0.9064	0.9400946	Mineral
N	PL10	4.7494	0.6298	0.0747	0.0035	0.3243	0.5423829	Mineral
N	PL11	3.638	-0.1418	0.0064	-0.07	0.7764	0.8898896	Mineral
N	PL12	23.2607	-0.2164	0.0052	-0.0713	0.7977	0.8978707	Mineral
N	PL13	0.0005	0.0016	0.8474	0.8357	0.00E+00	9.60E-05	Mineral
N	PL14	1.1325	0.1079	0.0349	-0.0393	0.505	0.7201836	Mineral
N	PL15	0.3857	-0.0236	0.0117	-0.0643	0.7013	0.8422123	Mineral
N	PL17	1.8343	-0.093	0.0087	-0.0675	0.7408	0.8648073	Mineral
N	PL18	0.0042	-0.0012	0.0556	-0.0171	0.3977	0.6013325	Mineral
N	PL16	0.52	-0.0184	0.0219	-0.0533	0.5986	0.7784967	Mineral
N	PL2	0.1389	0.007	0.0199	-0.0555	0.6159	0.7847381	Mineral
N	PL20	0.0975	0.01	0.0141	-0.0617	0.6732	0.8242018	Mineral
N	PL21	0.4953	-0.008	0.002	-0.0748	0.8749	0.9226825	Mineral
N	PL22	9.2334	1.0668	0.4158	0.3708	0.0095	0.0361538	Mineral
N	PL23	0.0157	-0.0005	0.0042	-0.0724	0.8185	0.9010455	Mineral
N	PL3	0.3266	0.0244	0.0044	-0.0722	0.8139	0.9010455	Mineral
N	PL4	15.4469	4.0132	0.8677	0.8575	0.00E+00	9.60E-05	Mineral
N	PL5	1.6555	0.965	0.6035	0.573	0.0007	0.0053427	Mineral
N	PL6	2.3184	-0.1606	0.0122	-0.0638	0.6949	0.8422123	Mineral
N	PL7	3.659	0.5889	0.103	0.034	0.2434	0.4407953	Mineral
N	PL8	2.5006	0.278	0.0788	0.0079	0.3109	0.5236722	Mineral
N	PL9	33.1358	0.4483	0.0021	-0.0747	0.8718	0.9226825	Mineral
CN	AA1	161.1286	5.1125	0.0788	0.0079	0.3109	0.5505925	Mineral
CN	AA11	0.406	0.08	0.0668	-0.005	0.3525	0.5898223	Mineral
CN	AA10	6.1103	0.9394	0.2737	0.2179	0.0454	0.2070137	Mineral
CN	AA2	1.4848	0.2469	0.1802	0.1171	0.1148	0.3141061	Mineral
CN	AA3	426.2452	-17.3844	0.1358	0.0693	0.1765	0.4015873	Mineral
CN	AA4	11.2554	3.0623	0.5456	0.5106	0.0017	0.0460048	Mineral
CN	AA5	53.5482	-5.3819	0.4578	0.4161	0.0056	0.0703502	Mineral
CN	AA8	0.6569	0.0837	0.0668	-0.0049	0.3521	0.5898223	Mineral
CN	AA7	57.2187	-0.9365	0.0102	-0.0659	0.7201	0.8547408	Mineral
CN	AA6	30.8574	-1.8173	0.2023	0.141	0.0925	0.2902102	Mineral
CN	AA9	1.4632	0.2439	0.0339	-0.0404	0.5111	0.7167284	Mineral
CN	CE1	216.8049	1.9745	0.0185	-0.057	0.6287	0.8208187	Mineral
CN	CE11	163.2288	3.6126	0.1393	0.0731	0.1706	0.3989225	Mineral
CN	CE12	15.6874	2.3012	0.2808	0.2255	0.0422	0.1997516	Mineral
CN	CE13	0.0362	0.0116	0.1943	0.1323	0.1001	0.2990452	Mineral
CN	CE14	221.0157	7.2988	0.3481	0.298	0.0206	0.1189824	Mineral
CN	CE15	37.8269	5.077	0.5668	0.5334	0.0012	0.0460048	Mineral
CN	CE2	2.5005	0.4497	0.1124	0.0442	0.2218	0.4453529	Mineral
CN	CE3	26.5046	0.0421	0.00E+00	-0.0769	0.9897	0.9974988	Mineral
CN	CE4	378.0703	-21.4059	0.2037	0.1424	0.0913	0.2901261	Mineral
CN	CE16	1.0559	0.1924	0.3064	0.253	0.0323	0.1689164	Mineral
CN	CE5	14.5299	-0.6858	0.0119	-0.0642	0.6993	0.8478877	Mineral
CN	CE7	10.4421	1.0305	0.1759	0.1125	0.1196	0.316071	Mineral
CN	CE6	37.5291	4.4322	0.3471	0.2969	0.0208	0.1189824	Mineral
CN	CE8	22.1825	2.2993	0.2189	0.1588	0.0786	0.2819643	Mineral
CN	CE9	94.9446	0.5336	0.0158	-0.0599	0.6548	0.821322	Mineral
CN	GH100	0.6919	0.127	0.1296	0.0626	0.1875	0.4015873	Mineral
CN	GH10	53.2214	3.1623	0.2307	0.1715	0.07	0.2661638	Mineral
CN	GH102	43.2037	-0.5763	0.0295	-0.0452	0.5405	0.7536564	Mineral
CN	GH101	0.8491	-0.0322	0.008	-0.0683	0.7516	0.8654241	Mineral
CN	GH103	113.5215	-5.6781	0.1316	0.0648	0.1839	0.4015873	Mineral
CN	GH104	5.515	-0.0245	0.0006	-0.0763	0.9314	0.9822904	Mineral
CN	GH105	25.0043	5.4709	0.4799	0.4398	0.0042	0.0670394	Mineral

CN	GH106	43.4576	6.3511	0.4347	0.3912	0.0075	0.0840696	Mineral
CN	GH107	0.1988	0.0042	0.0008	-0.076	0.9188	0.9822904	Mineral
CN	GH108	6.4325	0.263	0.128	0.0609	0.1905	0.4015873	Mineral
CN	GH109	19.7839	1.1133	0.1892	0.1269	0.1051	0.3030404	Mineral
CN	GH11	13.636	1.5958	0.2969	0.2428	0.0357	0.1787516	Mineral
CN	GH110	1.0855	0.174	0.2344	0.1755	0.0674	0.2661638	Mineral
CN	GH111	0.0484	-0.0007	0.0007	-0.0761	0.9241	0.9822904	Mineral
CN	GH112	0.065	0.0291	0.3907	0.3439	0.0127	0.0958718	Mineral
CN	GH113	8.5535	0.2336	0.0149	-0.0609	0.6651	0.821322	Mineral
CN	GH114	11.4339	-1.3437	0.2073	0.1463	0.0881	0.2877057	Mineral
CN	GH115	15.0153	3.1889	0.3449	0.2946	0.0213	0.1189824	Mineral
CN	GH116	40.527	2.8415	0.1333	0.0667	0.1808	0.4015873	Mineral
CN	GH117	3.5852	0.3696	0.1287	0.0617	0.1891	0.4015873	Mineral
CN	GH118	0.0033	0.0003	0.0082	-0.0681	0.7484	0.8654241	Mineral
CN	GH119	3.1764	0.0235	0.0005	-0.0764	0.939	0.9861777	Mineral
CN	GH12	8.1909	1.1	0.1966	0.1348	0.0978	0.2990452	Mineral
CN	GH120	1.8095	0.2939	0.0799	0.0091	0.3075	0.5505925	Mineral
CN	GH121	1.9359	0.0124	1.00E-04	-0.0768	0.9737	0.9974988	Mineral
CN	GH123	2.2624	-0.2215	0.0843	0.0139	0.2938	0.5383011	Mineral
CN	GH124	0.0624	-0.0186	0.268	0.2117	0.0481	0.2155813	Mineral
CN	GH125	10.2003	1.4254	0.3488	0.2987	0.0204	0.1189824	Mineral
CN	GH126	0.0079	0.0028	0.0798	0.009	0.3078	0.5505925	Mineral
CN	GH127	40.8673	4.2743	0.3896	0.3426	0.0129	0.0958718	Mineral
CN	GH128	6.089	0.8383	0.1624	0.098	0.1364	0.3492957	Mineral
CN	GH129	0.3723	0.0553	0.131	0.0641	0.185	0.4015873	Mineral
CN	GH13	1511.7028	3.6713	0.0008	-0.076	0.9183	0.9822904	Mineral
CN	GH130	83.298	5.8569	0.2249	0.1652	0.0741	0.2772169	Mineral
CN	GH131	0.1722	0.0505	0.1516	0.0863	0.1514	0.3687792	Mineral
CN	GH132	0.6932	0.1223	0.0609	-0.0114	0.3753	0.6078175	Mineral
CN	GH133	77.1918	-3.7749	0.1568	0.092	0.1439	0.3613135	Mineral
CN	GH14	0.4871	0.0262	0.0627	-0.0094	0.3682	0.6008217	Mineral
CN	GH17	62.1908	-3.0438	0.1137	0.0455	0.2191	0.4434433	Mineral
CN	GH15	404.3419	5.4674	0.0172	-0.0584	0.6409	0.821322	Mineral
CN	GH18	240.9138	11.9515	0.1664	0.1023	0.1312	0.3429451	Mineral
CN	GH19	17.4844	1.1561	0.0849	0.0145	0.2922	0.5383011	Mineral
CN	GH2	363.2682	27.52	0.4196	0.3749	0.009	0.0906362	Mineral
CN	GH20	98.8613	5.3954	0.3729	0.3247	0.0156	0.1087616	Mineral
CN	GH23	402.7243	4.4762	0.0183	-0.0573	0.6311	0.8208187	Mineral
CN	GH24	16.0614	0.8175	0.2156	0.1553	0.0812	0.283601	Mineral
CN	GH25	20.5589	0.4325	0.0082	-0.0681	0.7481	0.8654241	Mineral
CN	GH26	21.4128	0.9342	0.0382	-0.0358	0.4854	0.6962181	Mineral
CN	GH27	70.6768	8.7941	0.3465	0.2962	0.021	0.1189824	Mineral
CN	GH28	177.5732	20.756	0.6096	0.5796	0.0006	0.037086	Mineral
CN	GH29	104.4902	7.6545	0.3849	0.3376	0.0136	0.0974959	Mineral
CN	GH3	550.2428	43.6174	0.4325	0.3889	0.0077	0.0840696	Mineral
CN	GH30	48.4301	3.7332	0.2382	0.1796	0.065	0.2661638	Mineral
CN	GH31	125.157	12.5762	0.3996	0.3534	0.0115	0.0958718	Mineral
CN	GH33	63.7208	-0.7463	0.0063	-0.0702	0.7794	0.8890443	Mineral
CN	GH36	81.126	7.6717	0.3511	0.3012	0.0199	0.1189824	Mineral
CN	GH32	31.8356	-0.5137	0.0443	-0.0292	0.4515	0.6690447	Mineral
CN	GH37	36.4506	3.0556	0.1649	0.1007	0.1331	0.3443248	Mineral
CN	GH39	81.1725	5.7958	0.3889	0.3419	0.013	0.0958718	Mineral
CN	GH38	103.1959	13.4317	0.4696	0.4288	0.0048	0.0670394	Mineral
CN	GH4	145.5656	-4.3725	0.0285	-0.0462	0.5475	0.7592734	Mineral
CN	GH42	42.3015	2.5584	0.2428	0.1845	0.062	0.2638944	Mineral

CN	GH43	105.7244	13.0869	0.2071	0.1461	0.0883	0.2877057	Mineral
CN	GH44	24.2141	2.6905	0.5561	0.522	0.0014	0.0460048	Mineral
CN	GH45	0.4034	0.0709	0.152	0.0868	0.1508	0.3687792	Mineral
CN	GH47	15.3148	0.9273	0.1112	0.0428	0.2245	0.447191	Mineral
CN	GH46	2.3583	0.0028	0.00E+00	-0.0769	0.9939	0.9974988	Mineral
CN	GH48	9.3733	0.2609	0.0241	-0.0509	0.5805	0.7833351	Mineral
CN	GH49	0.174	-0.0193	0.0096	-0.0666	0.7287	0.8547408	Mineral
CN	GH5	169.7639	6.1433	0.0502	-0.0229	0.4222	0.6521574	Mineral
CN	GH50	7.8367	-0.8493	0.1555	0.0905	0.1458	0.3623568	Mineral
CN	GH51	100.2913	10.7419	0.5219	0.4851	0.0024	0.0536256	Mineral
CN	GH54	16.7488	4.3211	0.2958	0.2416	0.0361	0.1787516	Mineral
CN	GH53	54.6684	5.2103	0.4068	0.3612	0.0105	0.0958718	Mineral
CN	GH55	98.5985	10.5229	0.2329	0.1739	0.0684	0.2661638	Mineral
CN	GH56	0.003	0.00E+00	0.00E+00	-0.0769	0.9943	0.9974988	Mineral
CN	GH57	155.9399	-0.4605	0.001	-0.0758	0.9108	0.9822904	Mineral
CN	GH58	0.0426	0.0008	0.0007	-0.0761	0.9233	0.9822904	Mineral
CN	GH59	6.4703	1.2603	0.1788	0.1157	0.1163	0.3141061	Mineral
CN	GH6	17.654	1.7765	0.1096	0.0411	0.228	0.4506961	Mineral
CN	GH62	3.2335	0.2207	0.0255	-0.0495	0.5697	0.7771049	Mineral
CN	GH63	14.1513	1.1251	0.0917	0.0219	0.2725	0.5142414	Mineral
CN	GH64	18.7715	0.8681	0.0578	-0.0147	0.3881	0.6204661	Mineral
CN	GH65	100.9432	1.0422	0.0018	-0.075	0.8818	0.9622818	Mineral
CN	GH66	1.5117	-0.0467	0.0031	-0.0736	0.8438	0.9329782	Mineral
CN	GH68	0.2737	0.0121	0.0408	-0.033	0.4704	0.6824706	Mineral
CN	GH7	0.661	0.1539	0.0669	-0.0049	0.352	0.5898223	Mineral
CN	GH70	0.0411	0.002	0.0036	-0.073	0.8317	0.9300725	Mineral
CN	GH71	3.5602	0.4949	0.0786	0.0077	0.3115	0.5505925	Mineral
CN	GH72	30.0334	1.1381	0.0947	0.025	0.2646	0.5069115	Mineral
CN	GH73	39.5823	0.7927	0.0374	-0.0367	0.4901	0.6989597	Mineral
CN	GH75	2.2715	0.1514	0.054	-0.0187	0.4044	0.6424996	Mineral
CN	GH74	12.8907	0.5512	0.0462	-0.0272	0.4417	0.6679035	Mineral
CN	GH76	13.3927	1.1552	0.0668	-0.005	0.3525	0.5898223	Mineral
CN	GH78	148.9518	8.4059	0.2235	0.1638	0.0751	0.2772169	Mineral
CN	GH79	25.909	1.4183	0.0712	-0.0002	0.3363	0.5862392	Mineral
CN	GH8	19.2424	1.7526	0.1073	0.0386	0.2334	0.4573173	Mineral
CN	GH80	0.0028	0.0013	0.0263	-0.0486	0.5636	0.7730687	Mineral
CN	GH81	6.0259	0.2257	0.0144	-0.0614	0.6698	0.821322	Mineral
CN	GH84	4.7774	0.4019	0.2128	0.1522	0.0835	0.283601	Mineral
CN	GH85	0.6451	0.0749	0.0626	-0.0096	0.3686	0.6008217	Mineral
CN	GH86	1.7515	-0.0531	0.0143	-0.0615	0.6708	0.821322	Mineral
CN	GH87	64.8133	3.1395	0.1402	0.0741	0.1691	0.3989225	Mineral
CN	GH88	24.9678	0.1323	0.0034	-0.0733	0.837	0.9300725	Mineral
CN	GH89	6.8897	1.2559	0.3118	0.2589	0.0305	0.1628574	Mineral
CN	GH91	0.106	-0.0115	0.023	-0.0521	0.5891	0.7840529	Mineral
CN	GH90	0.0045	-0.0009	0.0247	-0.0503	0.5757	0.78113	Mineral
CN	GH9	51.5097	4.8681	0.1379	0.0716	0.1729	0.3989225	Mineral
CN	GH92	91.0695	11.403	0.1777	0.1144	0.1176	0.3141061	Mineral
CN	GH93	22.1339	1.6024	0.0154	-0.0604	0.6597	0.821322	Mineral
CN	GH94	27.0425	4.0284	0.4648	0.4236	0.0051	0.0676331	Mineral
CN	GH95	48.6912	3.9376	0.1827	0.1198	0.112	0.3124103	Mineral
CN	GH96	0.5474	0.0003	0.00E+00	-0.0769	0.9975	0.9974988	Mineral
CN	GH97	32.6174	1.2541	0.034	-0.0403	0.5107	0.7167284	Mineral
CN	GH98	0.3397	-0.0372	0.2126	0.1521	0.0836	0.283601	Mineral
CN	GH99	13.1032	-0.0566	0.0004	-0.0765	0.9448	0.9881541	Mineral
CN	GT1	153.6974	-3.2071	0.01	-0.0662	0.7235	0.8547408	Mineral

CN	GT10	2.4352	0.3023	0.5117	0.4741	0.0027	0.0568258	Mineral
CN	GT12	0.0482	0.0048	0.0521	-0.0208	0.413	0.6439022	Mineral
CN	GT14	2.0647	0.2405	0.1945	0.1325	0.0999	0.2990452	Mineral
CN	GT11	4.9506	0.4969	0.3921	0.3454	0.0125	0.0958718	Mineral
CN	GT13	0.1413	0.0607	0.5043	0.4661	0.003	0.0582126	Mineral
CN	GT15	1.84	0.253	0.0525	-0.0204	0.4116	0.6439022	Mineral
CN	GT16	0.0336	0.0214	0.4762	0.4359	0.0044	0.0670394	Mineral
CN	GT17	1.0318	0.1011	0.2586	0.2015	0.0529	0.2330581	Mineral
CN	GT18	0.0082	0.005	0.2781	0.2226	0.0433	0.2014875	Mineral
CN	GT2	1841.616	2.9509	0.0019	-0.0749	0.878	0.9622818	Mineral
CN	GT19	83.4375	2.1939	0.2319	0.1728	0.0691	0.2661638	Mineral
CN	GT20	150.7734	-1.1268	0.042	-0.0317	0.4639	0.680932	Mineral
CN	GT21	72.0818	3.64	0.0496	-0.0235	0.4251	0.6521574	Mineral
CN	GT22	4.3521	0.2621	0.0414	-0.0324	0.4671	0.6816875	Mineral
CN	GT23	0.2031	0.0616	0.6319	0.6036	0.0004	0.037086	Mineral
CN	GT24	2.657	0.3933	0.211	0.1503	0.085	0.2844818	Mineral
CN	GT25	5.9103	0.7943	0.6387	0.6109	0.0004	0.037086	Mineral
CN	GT26	133.0274	4.268	0.1905	0.1283	0.1038	0.3030404	Mineral
CN	GT27	13.0687	0.8954	0.4761	0.4358	0.0044	0.0670394	Mineral
CN	GT28	177.3669	2.8609	0.1219	0.0543	0.2022	0.4159665	Mineral
CN	GT29	3.9302	-0.2176	0.1291	0.0621	0.1884	0.4015873	Mineral
CN	GT30	120.1726	2.6999	0.1895	0.1271	0.1049	0.3030404	Mineral
CN	GT3	2.7042	0.4834	0.2326	0.1736	0.0686	0.2661638	Mineral
CN	GT33	1.8007	0.016	0.001	-0.0759	0.9121	0.9822904	Mineral
CN	GT34	0.89	0.1321	0.0686	-0.0031	0.3458	0.5898223	Mineral
CN	GT32	12.395	-0.2313	0.0135	-0.0624	0.6797	0.8281966	Mineral
CN	GT31	1.3616	0.3862	0.1881	0.1257	0.1062	0.3030404	Mineral
CN	GT35	348.7674	9.3865	0.0494	-0.0238	0.4261	0.6521574	Mineral
CN	GT37	0.0045	0.0009	0.0403	-0.0336	0.4733	0.6827189	Mineral
CN	GT39	20.8559	0.7216	0.0165	-0.0592	0.6483	0.821322	Mineral
CN	GT4	1887.0784	-8.0902	0.0028	-0.0739	0.8502	0.9359403	Mineral
CN	GT41	420.3074	13.8427	0.1272	0.06	0.192	0.4015873	Mineral
CN	GT40	0.0012	0.0002	0.0097	-0.0665	0.7273	0.8547408	Mineral
CN	GT42	0.0617	0.0284	0.2952	0.241	0.0363	0.1787516	Mineral
CN	GT43	0.0665	0.0385	0.5554	0.5212	0.0014	0.0460048	Mineral
CN	GT47	67.1447	3.2771	0.064	-0.008	0.363	0.5994579	Mineral
CN	GT45	0.2617	0.0084	0.0229	-0.0523	0.5904	0.7840529	Mineral
CN	GT44	0.0225	-0.0076	0.1307	0.0638	0.1856	0.4015873	Mineral
CN	GT48	3.0789	0.7527	0.1787	0.1155	0.1165	0.3141061	Mineral
CN	GT49	0.176	0.0545	0.2909	0.2364	0.038	0.1833089	Mineral
CN	GT5	111.8839	2.6937	0.1157	0.0477	0.2147	0.4381515	Mineral
CN	GT50	0.6419	0.117	0.0996	0.0303	0.2519	0.486417	Mineral
CN	GT53	23.5174	-0.6504	0.0056	-0.0709	0.7907	0.8940301	Mineral
CN	GT52	0	0	#VALUE!	#VALUE!	#VALUE!	NA	Mineral
CN	GT51	615.404	-6.8923	0.0175	-0.058	0.638	0.821322	Mineral
CN	GT54	0.0278	0.0166	0.3508	0.3008	0.02	0.1189824	Mineral
CN	GT57	1.3329	0.3337	0.1586	0.0939	0.1415	0.3588081	Mineral
CN	GT56	0.3995	0.0499	0.4473	0.4048	0.0064	0.0765285	Mineral
CN	GT55	2.8539	0.3477	0.1223	0.0548	0.2013	0.4159665	Mineral
CN	GT58	0.5128	0.0862	0.0844	0.014	0.2934	0.5383011	Mineral
CN	GT6	0.334	0.0432	0.07	-0.0015	0.3405	0.5894083	Mineral
CN	GT59	0.3598	0.0706	0.0939	0.0242	0.2667	0.5070852	Mineral
CN	GT60	1.8074	0.5923	0.5481	0.5133	0.0016	0.0460048	Mineral
CN	GT61	0.3572	0.0534	0.1507	0.0853	0.1528	0.3687792	Mineral
CN	GT62	2.1958	0.1583	0.0777	0.0067	0.3144	0.5519218	Mineral

CN	GT64	0.9005	0.0471	0.0455	-0.028	0.4454	0.6690447	Mineral
CN	GT65	0.0411	0.0147	0.404	0.3582	0.0109	0.0958718	Mineral
CN	GT66	19.8555	0.3105	0.0115	-0.0645	0.7036	0.849103	Mineral
CN	GT69	0.7751	0.2321	0.1841	0.1213	0.1105	0.3116911	Mineral
CN	GT68	0.0223	0.0095	0.4221	0.3776	0.0088	0.0906362	Mineral
CN	GT7	5.4876	0.3711	0.345	0.2946	0.0213	0.1189824	Mineral
CN	GT72	0.0008	0.00E+00	1.00E-04	-0.0768	0.9761	0.9974988	Mineral
CN	GT73	0.0663	0.0258	0.1979	0.1362	0.0966	0.2990452	Mineral
CN	GT71	1.4059	0.1207	0.0802	0.0094	0.3065	0.5505925	Mineral
CN	GT70	0.7826	0.0545	0.1377	0.0714	0.1732	0.3989225	Mineral
CN	GT74	0.8924	-0.0047	0.0003	-0.0766	0.9542	0.9937618	Mineral
CN	GT75	0.9563	0.0416	0.0522	-0.0207	0.4125	0.6439022	Mineral
CN	GT76	2.3528	-0.4666	0.6174	0.588	0.0005	0.037086	Mineral
CN	GT77	0.9469	-0.0922	0.2215	0.1616	0.0766	0.2787386	Mineral
CN	GT78	0.0018	0.0002	0.0034	-0.0733	0.8374	0.9300725	Mineral
CN	GT8	11.7518	0.3308	0.0168	-0.0589	0.6457	0.821322	Mineral
CN	GT80	5.6377	0.5612	0.4714	0.4307	0.0047	0.0670394	Mineral
CN	GT82	0.4951	0.0037	0.0006	-0.0762	0.9293	0.9822904	Mineral
CN	GT84	296.2703	21.4698	0.1339	0.0673	0.1798	0.4015873	Mineral
CN	GT81	24.419	0.2689	0.0063	-0.0702	0.7791	0.8890443	Mineral
CN	GT83	171.2538	2.2334	0.0265	-0.0483	0.5618	0.7730687	Mineral
CN	GT85	3.2912	-0.1037	0.0061	-0.0704	0.7828	0.8890443	Mineral
CN	GT87	16.9111	0.5707	0.0162	-0.0595	0.6516	0.821322	Mineral
CN	GT88	0.0259	0.0106	0.2044	0.1432	0.0906	0.2901261	Mineral
CN	GT89	5.7924	-0.0082	0.00E+00	-0.0769	0.9861	0.9974988	Mineral
CN	GT90	2.475	0.5938	0.2443	0.1862	0.0611	0.2638944	Mineral
CN	GT9	175.0926	0.0977	0.00E+00	-0.0769	0.9822	0.9974988	Mineral
CN	GT91	0.0159	-0.0026	0.0221	-0.0531	0.5969	0.7885125	Mineral
CN	GT93	0.368	0.0094	0.044	-0.0296	0.4531	0.6690447	Mineral
CN	GT94	0.7572	0.073	0.1066	0.0378	0.235	0.4573173	Mineral
CN	GT92	0.0971	0.0334	0.2409	0.1826	0.0632	0.2643013	Mineral
CN	GT95	7.0408	-0.2013	0.0593	-0.0131	0.3818	0.6143599	Mineral
CN	PL1	18.5567	0.7168	0.0093	-0.0669	0.7329	0.8555616	Mineral
CN	PL10	4.7494	0.8707	0.1427	0.0768	0.165	0.3944367	Mineral
CN	PL11	3.638	0.2679	0.0229	-0.0522	0.5901	0.7840529	Mineral
CN	PL12	23.2607	0.7602	0.0646	-0.0073	0.3605	0.5992002	Mineral
CN	PL13	0.0005	0.0013	0.5389	0.5035	0.0018	0.0460048	Mineral
CN	PL14	1.1325	-0.1263	0.0478	-0.0254	0.4336	0.6595811	Mineral
CN	PL15	0.3857	0.0406	0.0347	-0.0396	0.5065	0.7167284	Mineral
CN	PL17	1.8343	0.014	0.0002	-0.0767	0.9604	0.9961142	Mineral
CN	PL18	0.0042	0.0005	0.0102	-0.0659	0.7197	0.8547408	Mineral
CN	PL16	0.52	0.0089	0.0051	-0.0714	0.8006	0.901156	Mineral
CN	PL2	0.1389	0.0321	0.4142	0.3691	0.0096	0.0929698	Mineral
CN	PL20	0.0975	0.0109	0.0166	-0.0591	0.6473	0.821322	Mineral
CN	PL21	0.4953	0.0826	0.2133	0.1528	0.0831	0.283601	Mineral
CN	PL22	9.2334	0.9944	0.3612	0.3121	0.0178	0.1189824	Mineral
CN	PL23	0.0157	0.00E+00	0.00E+00	-0.0769	0.9915	0.9974988	Mineral
CN	PL3	0.3266	0.0451	0.0151	-0.0606	0.6623	0.821322	Mineral
CN	PL4	15.4469	2.7017	0.3932	0.3466	0.0123	0.0958718	Mineral
CN	PL5	1.6555	0.7199	0.3359	0.2848	0.0236	0.1285535	Mineral
CN	PL6	2.3184	0.1528	0.0111	-0.065	0.7091	0.8516212	Mineral
CN	PL7	3.659	0.3887	0.0449	-0.0286	0.4484	0.6690447	Mineral
CN	PL8	2.5006	0.298	0.0905	0.0206	0.2758	0.5166892	Mineral
CN	PL9	33.1358	1.3549	0.019	-0.0565	0.6243	0.820373	Mineral
Latitude	AA1	161.1286	10.2581	0.3172	0.2647	0.0288	0.1147416	Mineral

Latitude	AA11	0.406	-0.0261	0.0071	-0.0692	0.7648	0.8725499	Mineral
Latitude	AA10	6.1103	-0.5543	0.0953	0.0257	0.2629	0.4348822	Mineral
Latitude	AA2	1.4848	-0.0024	0.00E+00	-0.0769	0.9882	0.9989883	Mineral
Latitude	AA3	426.2452	-21.133	0.2007	0.1392	0.094	0.2359184	Mineral
Latitude	AA4	11.2554	0.984	0.0563	-0.0163	0.3943	0.5688508	Mineral
Latitude	AA5	53.5482	-1.4255	0.0321	-0.0423	0.5228	0.7033086	Mineral
Latitude	AA8	0.6569	-0.0354	0.012	-0.064	0.6979	0.8207299	Mineral
Latitude	AA7	57.2187	-7.2536	0.6124	0.5825	0.0006	0.0157195	Mineral
Latitude	AA6	30.8574	-3.0524	0.5708	0.5378	0.0011	0.0188007	Mineral
Latitude	AA9	1.4632	-0.1526	0.0133	-0.0626	0.6826	0.8168238	Mineral
Latitude	CE1	216.8049	-12.0477	0.6892	0.6653	0.0001	0.0080099	Mineral
Latitude	CE11	163.2288	0.7606	0.0062	-0.0703	0.7807	0.878751	Mineral
Latitude	CE12	15.6874	-1.1475	0.0698	-0.0017	0.3413	0.5129204	Mineral
Latitude	CE13	0.0362	-0.0151	0.3283	0.2767	0.0256	0.1051626	Mineral
Latitude	CE14	221.0157	-5.6276	0.207	0.146	0.0884	0.2241443	Mineral
Latitude	CE15	37.8269	-1.9647	0.0849	0.0145	0.2921	0.4640544	Mineral
Latitude	CE2	2.5005	-0.5038	0.1411	0.075	0.1677	0.3449272	Mineral
Latitude	CE3	26.5046	-6.3276	0.3016	0.2479	0.034	0.1291581	Mineral
Latitude	CE4	378.0703	-8.4184	0.0315	-0.043	0.5269	0.7033086	Mineral
Latitude	CE16	1.0559	0.0433	0.0155	-0.0602	0.6581	0.8137316	Mineral
Latitude	CE5	14.5299	-4.481	0.5063	0.4683	0.0029	0.0264886	Mineral
Latitude	CE7	10.4421	-0.9248	0.1417	0.0757	0.1667	0.3449272	Mineral
Latitude	CE6	37.5291	0.0103	0.00E+00	-0.0769	0.9961	0.9989883	Mineral
Latitude	CE8	22.1825	-2.921	0.3532	0.3035	0.0195	0.0872288	Mineral
Latitude	CE9	94.9446	-2.3114	0.2974	0.2434	0.0355	0.1329072	Mineral
Latitude	GH100	0.6919	0.0614	0.0303	-0.0443	0.5352	0.7033086	Mineral
Latitude	GH10	53.2214	-3.9204	0.3545	0.3049	0.0192	0.0872288	Mineral
Latitude	GH102	43.2037	-1.3439	0.1604	0.0959	0.139	0.317151	Mineral
Latitude	GH101	0.8491	-0.1952	0.2921	0.2376	0.0375	0.1329072	Mineral
Latitude	GH103	113.5215	3.1518	0.0405	-0.0333	0.4718	0.6578481	Mineral
Latitude	GH104	5.515	0.2705	0.0723	0.0009	0.3325	0.5088605	Mineral
Latitude	GH105	25.0043	-2.6788	0.115	0.047	0.2162	0.3962113	Mineral
Latitude	GH106	43.4576	-3.2201	0.1117	0.0434	0.2233	0.4004176	Mineral
Latitude	GH107	0.1988	-0.0807	0.3113	0.2584	0.0307	0.1202373	Mineral
Latitude	GH108	6.4325	-0.2694	0.1343	0.0677	0.1791	0.3597033	Mineral
Latitude	GH109	19.7839	-1.7891	0.4887	0.4493	0.0037	0.0323083	Mineral
Latitude	GH11	13.636	-0.7165	0.0599	-0.0125	0.3795	0.5538444	Mineral
Latitude	GH110	1.0855	-0.0586	0.0266	-0.0483	0.5615	0.7264973	Mineral
Latitude	GH111	0.0484	-0.0125	0.2153	0.1549	0.0815	0.2162974	Mineral
Latitude	GH112	0.065	-0.0181	0.1513	0.086	0.1519	0.3270322	Mineral
Latitude	GH113	8.5535	-0.4253	0.0492	-0.0239	0.4267	0.6053083	Mineral
Latitude	GH114	11.4339	-2.2877	0.6009	0.5702	0.0007	0.0172196	Mineral
Latitude	GH115	15.0153	-1.3583	0.0626	-0.0095	0.3685	0.5441277	Mineral
Latitude	GH116	40.527	3.4382	0.1952	0.1333	0.0992	0.2416612	Mineral
Latitude	GH117	3.5852	-0.7818	0.576	0.5433	0.001	0.0188007	Mineral
Latitude	GH118	0.0033	-0.001	0.0963	0.0268	0.2603	0.4348822	Mineral
Latitude	GH119	3.1764	-0.3507	0.1039	0.035	0.2412	0.4204833	Mineral
Latitude	GH12	8.1909	-1.3627	0.3017	0.248	0.0339	0.1291581	Mineral
Latitude	GH120	1.8095	-0.6959	0.4479	0.4054	0.0064	0.0469498	Mineral
Latitude	GH121	1.9359	-0.8288	0.386	0.3388	0.0134	0.0717159	Mineral
Latitude	GH123	2.2624	-0.2964	0.151	0.0857	0.1523	0.3270322	Mineral
Latitude	GH124	0.0624	0.009	0.0628	-0.0093	0.3676	0.5441277	Mineral
Latitude	GH125	10.2003	-0.2293	0.009	-0.0672	0.7363	0.8438337	Mineral
Latitude	GH126	0.0079	-0.0054	0.2875	0.2327	0.0394	0.1364454	Mineral
Latitude	GH127	40.8673	-3.3943	0.2457	0.1877	0.0603	0.1735182	Mineral

Latitude	GH128	6.089	-0.6953	0.1117	0.0434	0.2233	0.4004176	Mineral
Latitude	GH129	0.3723	-0.0713	0.2177	0.1575	0.0796	0.2162974	Mineral
Latitude	GH13	1511.7028	-103.0411	0.6628	0.6369	0.0002	0.0092209	Mineral
Latitude	GH130	83.298	9.353	0.5735	0.5407	0.0011	0.0188007	Mineral
Latitude	GH131	0.1722	0.0148	0.0131	-0.0629	0.6852	0.8168238	Mineral
Latitude	GH132	0.6932	-0.0093	0.0003	-0.0765	0.9473	0.9650796	Mineral
Latitude	GH133	77.1918	-1.0718	0.0126	-0.0633	0.6899	0.8168238	Mineral
Latitude	GH14	0.4871	-0.0334	0.1017	0.0326	0.2466	0.4239375	Mineral
Latitude	GH17	62.1908	1.6066	0.0317	-0.0428	0.5257	0.7033086	Mineral
Latitude	GH15	404.3419	-25.5153	0.3756	0.3275	0.0151	0.074513	Mineral
Latitude	GH18	240.9138	-19.0697	0.4237	0.3794	0.0086	0.0566994	Mineral
Latitude	GH19	17.4844	-2.7481	0.4795	0.4395	0.0042	0.0353112	Mineral
Latitude	GH2	363.2682	-18.5422	0.1905	0.1282	0.1039	0.2482819	Mineral
Latitude	GH20	98.8613	-4.672	0.2796	0.2242	0.0427	0.1421226	Mineral
Latitude	GH23	402.7243	-17.2439	0.2709	0.2149	0.0467	0.1500885	Mineral
Latitude	GH24	16.0614	-0.8906	0.2559	0.1987	0.0544	0.1671481	Mineral
Latitude	GH25	20.5589	-3.8239	0.642	0.6144	0.0003	0.0103515	Mineral
Latitude	GH26	21.4128	-2.9715	0.386	0.3388	0.0134	0.0717159	Mineral
Latitude	GH27	70.6768	-4.5923	0.0945	0.0248	0.2651	0.4348822	Mineral
Latitude	GH28	177.5732	-4.2426	0.0255	-0.0495	0.5699	0.7298607	Mineral
Latitude	GH29	104.4902	-2.1763	0.0311	-0.0434	0.5294	0.7033086	Mineral
Latitude	GH3	550.2428	-22.3331	0.1134	0.0452	0.2197	0.3996159	Mineral
Latitude	GH30	48.4301	-2.7737	0.1315	0.0647	0.1841	0.3645508	Mineral
Latitude	GH31	125.157	-2.3778	0.0143	-0.0615	0.6714	0.8168238	Mineral
Latitude	GH33	63.7208	-3.3381	0.1251	0.0578	0.1959	0.3742057	Mineral
Latitude	GH36	81.126	-2.3229	0.0322	-0.0423	0.5223	0.7033086	Mineral
Latitude	GH32	31.8356	0.8278	0.115	0.0469	0.2163	0.3962113	Mineral
Latitude	GH37	36.4506	3.1745	0.178	0.1148	0.1172	0.2749362	Mineral
Latitude	GH39	81.1725	3.0346	0.1066	0.0379	0.2349	0.4181933	Mineral
Latitude	GH38	103.1959	-0.3495	0.0003	-0.0766	0.9497	0.9650796	Mineral
Latitude	GH4	145.5656	-15.9604	0.3798	0.332	0.0144	0.0743021	Mineral
Latitude	GH42	42.3015	-0.9064	0.0305	-0.0441	0.5338	0.7033086	Mineral
Latitude	GH43	105.7244	-14.4712	0.2533	0.1958	0.0558	0.1688363	Mineral
Latitude	GH44	24.2141	1.1231	0.0969	0.0274	0.2587	0.4348822	Mineral
Latitude	GH45	0.4034	-0.0179	0.0097	-0.0664	0.7265	0.8364614	Mineral
Latitude	GH47	15.3148	-1.2813	0.2123	0.1517	0.0839	0.2170428	Mineral
Latitude	GH46	2.3583	-0.9884	0.5656	0.5321	0.0012	0.0191588	Mineral
Latitude	GH48	9.3733	-1.0492	0.3902	0.3433	0.0128	0.0713481	Mineral
Latitude	GH49	0.174	-0.0545	0.0766	0.0056	0.318	0.489616	Mineral
Latitude	GH5	169.7639	-17.4422	0.4046	0.3588	0.0108	0.0661382	Mineral
Latitude	GH50	7.8367	-1.1186	0.2698	0.2136	0.0472	0.1500885	Mineral
Latitude	GH51	100.2913	-2.2571	0.023	-0.0521	0.5892	0.7506727	Mineral
Latitude	GH54	16.7488	-1.1749	0.0219	-0.0534	0.5989	0.7592561	Mineral
Latitude	GH53	54.6684	4.3138	0.2789	0.2234	0.043	0.1421226	Mineral
Latitude	GH55	98.5985	0.5939	0.0007	-0.0761	0.9232	0.9629079	Mineral
Latitude	GH56	0.003	0.0008	0.0547	-0.018	0.4013	0.5756216	Mineral
Latitude	GH57	155.9399	5.7056	0.1541	0.089	0.1478	0.3270322	Mineral
Latitude	GH58	0.0426	-0.0106	0.1349	0.0684	0.178	0.3597033	Mineral
Latitude	GH59	6.4703	-0.9168	0.0946	0.025	0.2647	0.4348822	Mineral
Latitude	GH6	17.654	-2.712	0.2555	0.1982	0.0546	0.1671481	Mineral
Latitude	GH62	3.2335	-1.031	0.5568	0.5227	0.0014	0.0195219	Mineral
Latitude	GH63	14.1513	-2.0175	0.295	0.2408	0.0364	0.1329072	Mineral
Latitude	GH64	18.7715	-1.8705	0.2683	0.212	0.0479	0.1504213	Mineral
Latitude	GH65	100.9432	-20.7148	0.6978	0.6746	0.0001	0.0080099	Mineral
Latitude	GH66	1.5117	-0.2359	0.0792	0.0084	0.3096	0.4856508	Mineral

Latitude	GH68	0.2737	0.018	0.0898	0.0198	0.2779	0.4470728	Mineral
Latitude	GH7	0.661	-0.0204	0.0012	-0.0757	0.9033	0.9566977	Mineral
Latitude	GH70	0.0411	-0.0066	0.0381	-0.0359	0.4857	0.6698579	Mineral
Latitude	GH71	3.5602	0.2026	0.0132	-0.0627	0.6838	0.8168238	Mineral
Latitude	GH72	30.0334	-1.3001	0.1236	0.0561	0.1989	0.3749299	Mineral
Latitude	GH73	39.5823	-2.9167	0.5057	0.4677	0.003	0.0264886	Mineral
Latitude	GH75	2.2715	-0.4413	0.4589	0.4173	0.0055	0.0420441	Mineral
Latitude	GH74	12.8907	-1.1454	0.1995	0.1379	0.0952	0.2364658	Mineral
Latitude	GH76	13.3927	-2.1699	0.2355	0.1767	0.0667	0.1839505	Mineral
Latitude	GH78	148.9518	-7.9185	0.1984	0.1367	0.0962	0.236632	Mineral
Latitude	GH79	25.909	2.6293	0.2447	0.1866	0.0608	0.1735182	Mineral
Latitude	GH8	19.2424	-4.0674	0.5778	0.5453	0.001	0.0188007	Mineral
Latitude	GH80	0.0028	-0.0047	0.3397	0.2889	0.0226	0.0995261	Mineral
Latitude	GH81	6.0259	-0.7004	0.1389	0.0727	0.1712	0.3494501	Mineral
Latitude	GH84	4.7774	-0.3156	0.1313	0.0644	0.1845	0.3645508	Mineral
Latitude	GH85	0.6451	-0.2222	0.5505	0.516	0.0015	0.0203359	Mineral
Latitude	GH86	1.7515	-0.1592	0.1289	0.0619	0.1888	0.3645508	Mineral
Latitude	GH87	64.8133	1.2181	0.0211	-0.0542	0.6054	0.7635529	Mineral
Latitude	GH88	24.9678	-0.7082	0.0967	0.0272	0.2592	0.4348822	Mineral
Latitude	GH89	6.8897	-0.5502	0.0599	-0.0125	0.3795	0.5538444	Mineral
Latitude	GH91	0.106	-0.0436	0.3309	0.2794	0.0249	0.1051626	Mineral
Latitude	GH90	0.0045	-0.0008	0.0193	-0.0561	0.6211	0.7795369	Mineral
Latitude	GH9	51.5097	-10.0065	0.5828	0.5507	0.0009	0.0188007	Mineral
Latitude	GH92	91.0695	-15.6741	0.3357	0.2846	0.0236	0.1021648	Mineral
Latitude	GH93	22.1339	-4.5611	0.1246	0.0573	0.1968	0.3742057	Mineral
Latitude	GH94	27.0425	-0.6074	0.0106	-0.0655	0.7154	0.8313537	Mineral
Latitude	GH95	48.6912	-4.8647	0.2789	0.2234	0.043	0.1421226	Mineral
Latitude	GH96	0.5474	-0.2613	0.4384	0.3952	0.0072	0.0513432	Mineral
Latitude	GH97	32.6174	-5.8941	0.7506	0.7314	0.00E+00	0.0073938	Mineral
Latitude	GH98	0.3397	0.0051	0.004	-0.0726	0.8218	0.9087064	Mineral
Latitude	GH99	13.1032	-1.9788	0.4682	0.4272	0.0049	0.0384461	Mineral
Latitude	GT1	153.6974	-23.467	0.5332	0.4973	0.002	0.0223943	Mineral
Latitude	GT10	2.4352	0.2551	0.3644	0.3156	0.0172	0.0813201	Mineral
Latitude	GT12	0.0482	-0.001	0.0021	-0.0746	0.87	0.9331962	Mineral
Latitude	GT14	2.0647	-0.3539	0.4211	0.3766	0.0089	0.0570119	Mineral
Latitude	GT11	4.9506	0.3962	0.2492	0.1915	0.0582	0.1717374	Mineral
Latitude	GT13	0.1413	-0.0059	0.0047	-0.0719	0.8086	0.9020107	Mineral
Latitude	GT15	1.84	0.152	0.0189	-0.0565	0.6248	0.7802608	Mineral
Latitude	GT16	0.0336	0.0093	0.09	0.0199	0.2775	0.4470728	Mineral
Latitude	GT17	1.0318	0.0553	0.0775	0.0065	0.315	0.48811	Mineral
Latitude	GT18	0.0082	0.0003	0.0007	-0.0762	0.9245	0.9629079	Mineral
Latitude	GT2	1841.616	-11.1466	0.0269	-0.048	0.5595	0.7264973	Mineral
Latitude	GT19	83.4375	1.721	0.1427	0.0767	0.1651	0.3449272	Mineral
Latitude	GT20	150.7734	-0.3359	0.0037	-0.0729	0.8288	0.9096357	Mineral
Latitude	GT21	72.0818	8.0311	0.2414	0.183	0.0629	0.1754938	Mineral
Latitude	GT22	4.3521	-0.2844	0.0487	-0.0245	0.4293	0.6053083	Mineral
Latitude	GT23	0.2031	0.0125	0.0262	-0.0488	0.5648	0.7269458	Mineral
Latitude	GT24	2.657	0.0154	0.0003	-0.0766	0.9493	0.9650796	Mineral
Latitude	GT25	5.9103	0.4606	0.2148	0.1544	0.0819	0.2162974	Mineral
Latitude	GT26	133.0274	2.1287	0.0474	-0.0259	0.4357	0.6109521	Mineral
Latitude	GT27	13.0687	0.4057	0.0977	0.0283	0.2566	0.4348822	Mineral
Latitude	GT28	177.3669	0.9578	0.0137	-0.0622	0.6783	0.8168238	Mineral
Latitude	GT29	3.9302	-0.3796	0.393	0.3463	0.0124	0.0706336	Mineral
Latitude	GT30	120.1726	2.3522	0.1438	0.078	0.1633	0.3449272	Mineral
Latitude	GT3	2.7042	-0.2809	0.0786	0.0077	0.3116	0.4857369	Mineral

Latitude	GT33	1.8007	-0.0551	0.0115	-0.0645	0.7033	0.8210939	Mineral
Latitude	GT34	0.89	-0.0254	0.0025	-0.0742	0.8588	0.9273763	Mineral
Latitude	GT32	12.395	0.5326	0.0717	0.0003	0.3345	0.5088755	Mineral
Latitude	GT31	1.3616	-0.0189	0.0004	-0.0764	0.9402	0.9650796	Mineral
Latitude	GT35	348.7674	-20.7551	0.2413	0.183	0.0629	0.1754938	Mineral
Latitude	GT37	0.0045	0.0012	0.0707	-0.0008	0.3381	0.5111518	Mineral
Latitude	GT39	20.8559	-2.8114	0.2503	0.1926	0.0575	0.1717374	Mineral
Latitude	GT4	1887.0784	-45.8129	0.0913	0.0214	0.2736	0.4459724	Mineral
Latitude	GT41	420.3074	4.2201	0.0118	-0.0642	0.6997	0.8207299	Mineral
Latitude	GT40	0.0012	-0.0003	0.0127	-0.0632	0.6889	0.8168238	Mineral
Latitude	GT42	0.0617	0.0209	0.1595	0.0948	0.1403	0.3172004	Mineral
Latitude	GT43	0.0665	0.0201	0.1509	0.0856	0.1524	0.3270322	Mineral
Latitude	GT47	67.1447	4.5417	0.1229	0.0554	0.2002	0.3749299	Mineral
Latitude	GT45	0.2617	-0.0276	0.2458	0.1877	0.0602	0.1735182	Mineral
Latitude	GT44	0.0225	-0.0132	0.3932	0.3466	0.0123	0.0706336	Mineral
Latitude	GT48	3.0789	0.4608	0.067	-0.0048	0.3517	0.525476	Mineral
Latitude	GT49	0.176	0.0298	0.0874	0.0172	0.2848	0.4553321	Mineral
Latitude	GT5	111.8839	3.6571	0.2133	0.1528	0.083	0.2170428	Mineral
Latitude	GT50	0.6419	0.0243	0.0043	-0.0723	0.8163	0.9065449	Mineral
Latitude	GT53	23.5174	-6.1745	0.5058	0.4678	0.0029	0.0264886	Mineral
Latitude	GT52	0	0	#VALUE!	#VALUE!	#VALUE!	NA	Mineral
Latitude	GT51	615.404	-11.5347	0.0491	-0.024	0.4273	0.6053083	Mineral
Latitude	GT54	0.0278	0.0022	0.0064	-0.07	0.7767	0.8781862	Mineral
Latitude	GT57	1.3329	0.0415	0.0025	-0.0743	0.8609	0.9273763	Mineral
Latitude	GT56	0.3995	-0.0213	0.0818	0.0111	0.3015	0.4759475	Mineral
Latitude	GT55	2.8539	-0.1882	0.0358	-0.0383	0.4992	0.6847478	Mineral
Latitude	GT58	0.5128	0.0296	0.01	-0.0662	0.7233	0.8364614	Mineral
Latitude	GT6	0.334	0.0875	0.2867	0.2318	0.0397	0.1364454	Mineral
Latitude	GT59	0.3598	-0.014	0.0037	-0.073	0.8299	0.9096357	Mineral
Latitude	GT60	1.8074	-0.0616	0.0059	-0.0705	0.785	0.8795906	Mineral
Latitude	GT61	0.3572	-0.0597	0.188	0.1255	0.1064	0.2518988	Mineral
Latitude	GT62	2.1958	-0.0011	0.00E+00	-0.0769	0.9947	0.9989883	Mineral
Latitude	GT64	0.9005	-0.0794	0.1295	0.0625	0.1877	0.3645508	Mineral
Latitude	GT65	0.0411	0.0012	0.0026	-0.0741	0.857	0.9273763	Mineral
Latitude	GT66	19.8555	-1.7655	0.3718	0.3234	0.0158	0.0763157	Mineral
Latitude	GT69	0.7751	0.0155	0.0008	-0.076	0.9193	0.9629079	Mineral
Latitude	GT68	0.0223	0.0047	0.1028	0.0338	0.2439	0.4221128	Mineral
Latitude	GT7	5.4876	0.0839	0.0176	-0.0579	0.637	0.7915448	Mineral
Latitude	GT72	0.0008	0.0007	0.1415	0.0755	0.167	0.3449272	Mineral
Latitude	GT73	0.0663	0.0031	0.0029	-0.0738	0.8483	0.9257349	Mineral
Latitude	GT71	1.4059	-0.1672	0.1538	0.0887	0.1483	0.3270322	Mineral
Latitude	GT70	0.7826	0.0793	0.2919	0.2374	0.0376	0.1329072	Mineral
Latitude	GT74	0.8924	0.1979	0.4748	0.4344	0.0045	0.0363823	Mineral
Latitude	GT75	0.9563	0.1037	0.3249	0.2729	0.0265	0.1074143	Mineral
Latitude	GT76	2.3528	-0.2139	0.1297	0.0628	0.1873	0.3645508	Mineral
Latitude	GT77	0.9469	0.0632	0.1041	0.0352	0.2408	0.4204833	Mineral
Latitude	GT78	0.0018	-0.0017	0.163	0.0987	0.1355	0.3121311	Mineral
Latitude	GT8	11.7518	-1.186	0.2154	0.155	0.0814	0.2162974	Mineral
Latitude	GT80	5.6377	0.3733	0.2086	0.1477	0.087	0.222831	Mineral
Latitude	GT82	0.4951	-0.0478	0.1041	0.0352	0.2409	0.4204833	Mineral
Latitude	GT84	296.2703	47.2325	0.6481	0.621	0.0003	0.0103515	Mineral
Latitude	GT81	24.419	-2.4271	0.5109	0.4732	0.0027	0.0264886	Mineral
Latitude	GT83	171.2538	4.7714	0.1212	0.0536	0.2036	0.3784972	Mineral
Latitude	GT85	3.2912	-0.8722	0.4287	0.3848	0.0081	0.0547702	Mineral
Latitude	GT87	16.9111	-2.9416	0.4295	0.3856	0.008	0.0547702	Mineral

Latitude	GT88	0.0259	-0.0056	0.0566	-0.016	0.3933	0.5688508	Mineral
Latitude	GT89	5.7924	-1.3597	0.6699	0.6445	0.0002	0.0092209	Mineral
Latitude	GT90	2.475	-0.0214	0.0003	-0.0766	0.9497	0.9650796	Mineral
Latitude	GT9	175.0926	0.0055	0.00E+00	-0.0769	0.999	0.9989883	Mineral
Latitude	GT91	0.0159	0.0007	0.0015	-0.0753	0.8893	0.9457857	Mineral
Latitude	GT93	0.368	-0.0019	0.0018	-0.075	0.8819	0.9419366	Mineral
Latitude	GT94	0.7572	-0.0977	0.1909	0.1287	0.1034	0.2482819	Mineral
Latitude	GT92	0.0971	0.0118	0.0298	-0.0448	0.5381	0.703503	Mineral
Latitude	GT95	7.0408	-0.0931	0.0127	-0.0633	0.6893	0.8168238	Mineral
Latitude	PL1	18.5567	-5.4718	0.5402	0.5048	0.0018	0.0215021	Mineral
Latitude	PL10	4.7494	-1.4143	0.3766	0.3287	0.015	0.074513	Mineral
Latitude	PL11	3.638	-1.2775	0.5213	0.4845	0.0024	0.0247869	Mineral
Latitude	PL12	23.2607	-1.6225	0.2945	0.2402	0.0366	0.1329072	Mineral
Latitude	PL13	0.0005	0.0003	0.0303	-0.0443	0.535	0.7033086	Mineral
Latitude	PL14	1.1325	-0.1131	0.0383	-0.0357	0.4844	0.6698579	Mineral
Latitude	PL15	0.3857	-0.0851	0.152	0.0868	0.1508	0.3270322	Mineral
Latitude	PL17	1.8343	-0.5989	0.3615	0.3124	0.0177	0.0824843	Mineral
Latitude	PL18	0.0042	-0.0041	0.6931	0.6695	0.0001	0.0080099	Mineral
Latitude	PL16	0.52	-0.0782	0.3937	0.3471	0.0123	0.0706336	Mineral
Latitude	PL2	0.1389	0.0209	0.1757	0.1123	0.1198	0.2785273	Mineral
Latitude	PL20	0.0975	-0.0622	0.5425	0.5073	0.0017	0.0215021	Mineral
Latitude	PL21	0.4953	0.0038	0.0005	-0.0764	0.9398	0.9650796	Mineral
Latitude	PL22	9.2334	0.0537	0.0011	-0.0758	0.9086	0.958192	Mineral
Latitude	PL23	0.0157	-0.0042	0.2717	0.2157	0.0463	0.1500885	Mineral
Latitude	PL3	0.3266	-0.2103	0.3288	0.2772	0.0254	0.1051626	Mineral
Latitude	PL4	15.4469	0.3499	0.0066	-0.0698	0.7735	0.8781862	Mineral
Latitude	PL5	1.6555	-0.1411	0.0129	-0.063	0.687	0.8168238	Mineral
Latitude	PL6	2.3184	-1.0877	0.5606	0.5268	0.0013	0.0194794	Mineral
Latitude	PL7	3.659	-1.1737	0.4094	0.3639	0.0102	0.0640498	Mineral
Latitude	PL8	2.5006	-0.61	0.3793	0.3316	0.0145	0.0743021	Mineral
Latitude	PL9	33.1358	-7.1657	0.5313	0.4952	0.0021	0.0223943	Mineral
Longitude	AA1	161.1286	2.655	0.0212	-0.054	0.6042	0.7326246	Mineral
Longitude	AA11	0.406	-0.1236	0.1594	0.0947	0.1404	0.2649948	Mineral
Longitude	AA10	6.1103	-0.8123	0.2047	0.1435	0.0904	0.2003841	Mineral
Longitude	AA2	1.4848	-0.2363	0.165	0.1007	0.1331	0.2609053	Mineral
Longitude	AA3	426.2452	-25.6432	0.2955	0.2413	0.0362	0.1032805	Mineral
Longitude	AA4	11.2554	0.9084	0.048	-0.0252	0.4327	0.5998639	Mineral
Longitude	AA5	53.5482	-4.4613	0.3146	0.2618	0.0296	0.0874747	Mineral
Longitude	AA8	0.6569	-0.1684	0.2707	0.2146	0.0468	0.1223901	Mineral
Longitude	AA7	57.2187	-5.5694	0.361	0.3118	0.0178	0.0594135	Mineral
Longitude	AA6	30.8574	-1.4404	0.1271	0.06	0.1921	0.3464905	Mineral
Longitude	AA9	1.4632	-0.5982	0.204	0.1428	0.091	0.2003841	Mineral
Longitude	CE1	216.8049	-7.2218	0.2477	0.1898	0.0591	0.1468081	Mineral
Longitude	CE11	163.2288	-1.7257	0.0318	-0.0427	0.5249	0.6826986	Mineral
Longitude	CE12	15.6874	-2.6899	0.3837	0.3363	0.0138	0.0501869	Mineral
Longitude	CE13	0.0362	0.0006	0.0005	-0.0764	0.9359	0.9587773	Mineral
Longitude	CE14	221.0157	-3.73	0.0909	0.021	0.2748	0.4478136	Mineral
Longitude	CE15	37.8269	-1.2464	0.0342	-0.0401	0.5096	0.6716414	Mineral
Longitude	CE2	2.5005	-0.8473	0.3991	0.3529	0.0115	0.0431874	Mineral
Longitude	CE3	26.5046	-10.7199	0.8657	0.8554	0.00E+00	3.11E-05	Mineral
Longitude	CE4	378.0703	-37.3633	0.6205	0.5913	0.0005	0.0031442	Mineral
Longitude	CE16	1.0559	-0.0993	0.0816	0.0109	0.3021	0.4769535	Mineral
Longitude	CE5	14.5299	-0.4269	0.0046	-0.072	0.8103	0.8766749	Mineral
Longitude	CE7	10.4421	-1.6417	0.4465	0.404	0.0065	0.0261903	Mineral
Longitude	CE6	37.5291	3.87	0.2647	0.2081	0.0498	0.1287353	Mineral

Longitude	CE8	22.1825	-2.6209	0.2844	0.2293	0.0406	0.1121124	Mineral
Longitude	CE9	94.9446	-2.601	0.3766	0.3287	0.015	0.0518256	Mineral
Longitude	GH100	0.6919	-0.0387	0.0121	-0.0639	0.6968	0.7877729	Mineral
Longitude	GH10	53.2214	-2.955	0.2014	0.14	0.0933	0.2037219	Mineral
Longitude	GH102	43.2037	-2.5496	0.5774	0.5449	0.001	0.0055181	Mineral
Longitude	GH101	0.8491	-0.325	0.8099	0.7952	0.00E+00	0.0001024	Mineral
Longitude	GH103	113.5215	-8.3906	0.2873	0.2325	0.0394	0.110908	Mineral
Longitude	GH104	5.515	-0.5293	0.2768	0.2212	0.0439	0.1186134	Mineral
Longitude	GH105	25.0043	-1.3035	0.0272	-0.0476	0.5567	0.7092353	Mineral
Longitude	GH106	43.4576	-0.7332	0.0058	-0.0707	0.7875	0.8601623	Mineral
Longitude	GH107	0.1988	-0.1352	0.8726	0.8628	0.00E+00	3.11E-05	Mineral
Longitude	GH108	6.4325	-0.0471	0.0041	-0.0725	0.8206	0.8840242	Mineral
Longitude	GH109	19.7839	-1.582	0.3821	0.3346	0.014	0.0503732	Mineral
Longitude	GH11	13.636	-0.73	0.0621	-0.01	0.3703	0.5467623	Mineral
Longitude	GH110	1.0855	-0.084	0.0547	-0.0181	0.4017	0.5794407	Mineral
Longitude	GH111	0.0484	-0.0162	0.3584	0.3091	0.0184	0.059783	Mineral
Longitude	GH112	0.065	-0.0154	0.1088	0.0402	0.2299	0.3873644	Mineral
Longitude	GH113	8.5535	-1.6619	0.752	0.7329	0.00E+00	0.0004199	Mineral
Longitude	GH114	11.4339	-1.3815	0.2191	0.1591	0.0784	0.1806452	Mineral
Longitude	GH115	15.0153	-2.9062	0.2865	0.2316	0.0398	0.110908	Mineral
Longitude	GH116	40.527	6.4489	0.6868	0.6627	0.0001	0.0013812	Mineral
Longitude	GH117	3.5852	-0.4565	0.1964	0.1346	0.098	0.2103117	Mineral
Longitude	GH118	0.0033	-0.0013	0.161	0.0965	0.1382	0.2630057	Mineral
Longitude	GH119	3.1764	-0.9226	0.7192	0.6976	1.00E-04	0.0008562	Mineral
Longitude	GH12	8.1909	-1.1758	0.2246	0.165	0.0743	0.1726841	Mineral
Longitude	GH120	1.8095	0.1554	0.0223	-0.0529	0.5949	0.7279088	Mineral
Longitude	GH121	1.9359	-1.2011	0.8107	0.7961	0.00E+00	0.0001024	Mineral
Longitude	GH123	2.2624	-0.6309	0.6839	0.6596	0.0001	0.0013812	Mineral
Longitude	GH124	0.0624	0.0088	0.0593	-0.013	0.3816	0.5601639	Mineral
Longitude	GH125	10.2003	0.3479	0.0208	-0.0545	0.6082	0.7339769	Mineral
Longitude	GH126	0.0079	0.0015	0.0234	-0.0517	0.586	0.724555	Mineral
Longitude	GH127	40.8673	-1.5464	0.051	-0.022	0.4184	0.5932614	Mineral
Longitude	GH128	6.089	-0.5191	0.0623	-0.0099	0.3698	0.5467623	Mineral
Longitude	GH129	0.3723	0.0176	0.0132	-0.0627	0.6834	0.7813652	Mineral
Longitude	GH13	1511.7028	-52.5344	0.1723	0.1086	0.1239	0.2468603	Mineral
Longitude	GH130	83.298	1.5479	0.0157	-0.06	0.6563	0.7591056	Mineral
Longitude	GH131	0.1722	-0.0513	0.1561	0.0912	0.145	0.2715849	Mineral
Longitude	GH132	0.6932	-0.1097	0.0489	-0.0242	0.4281	0.5969753	Mineral
Longitude	GH133	77.1918	3.4759	0.133	0.0663	0.1814	0.3324274	Mineral
Longitude	GH14	0.4871	-0.02	0.0364	-0.0377	0.4957	0.6660926	Mineral
Longitude	GH17	62.1908	-4.4655	0.2447	0.1866	0.0609	0.1482902	Mineral
Longitude	GH15	404.3419	9.5838	0.053	-0.0199	0.4092	0.5835518	Mineral
Longitude	GH18	240.9138	-18.69	0.407	0.3614	0.0105	0.0399168	Mineral
Longitude	GH19	17.4844	-3.0461	0.5891	0.5575	0.0008	0.0046616	Mineral
Longitude	GH2	363.2682	-9.8623	0.0539	-0.0189	0.4051	0.5810713	Mineral
Longitude	GH20	98.8613	-4.6127	0.2726	0.2166	0.0459	0.1223901	Mineral
Longitude	GH23	402.7243	-30.1535	0.8285	0.8153	0.00E+00	6.51E-05	Mineral
Longitude	GH24	16.0614	-0.6389	0.1317	0.0649	0.1837	0.334084	Mineral
Longitude	GH25	20.5589	-3.5005	0.538	0.5024	0.0019	0.0093319	Mineral
Longitude	GH26	21.4128	-2.4134	0.2546	0.1973	0.0551	0.1410538	Mineral
Longitude	GH27	70.6768	-0.7136	0.0023	-0.0745	0.8658	0.9075583	Mineral
Longitude	GH28	177.5732	-3.7972	0.0204	-0.055	0.6116	0.7344611	Mineral
Longitude	GH29	104.4902	5.6029	0.2062	0.1452	0.089	0.1995286	Mineral
Longitude	GH3	550.2428	0.9943	0.0002	-0.0767	0.9577	0.9655406	Mineral
Longitude	GH30	48.4301	-2.0194	0.0697	-0.0019	0.3417	0.5230162	Mineral

Longitude	GH31	125.157	8.561	0.1852	0.1225	0.1094	0.2287696	Mineral
Longitude	GH33	63.7208	-7.57	0.6433	0.6158	0.0003	0.0024533	Mineral
Longitude	GH36	81.126	3.3263	0.066	-0.0058	0.3553	0.5405262	Mineral
Longitude	GH32	31.8356	1.2224	0.2508	0.1932	0.0572	0.1441206	Mineral
Longitude	GH37	36.4506	6.3088	0.7032	0.6803	1.00E-04	0.0011232	Mineral
Longitude	GH39	81.1725	4.4222	0.2264	0.1669	0.073	0.1712675	Mineral
Longitude	GH38	103.1959	3.9064	0.0397	-0.0341	0.4764	0.6471777	Mineral
Longitude	GH4	145.5656	-18.5009	0.5103	0.4726	0.0028	0.0128828	Mineral
Longitude	GH42	42.3015	0.4484	0.0075	-0.0689	0.7596	0.8398747	Mineral
Longitude	GH43	105.7244	-13.153	0.2092	0.1484	0.0865	0.1955144	Mineral
Longitude	GH44	24.2141	-1.2717	0.1243	0.0569	0.1975	0.3492263	Mineral
Longitude	GH45	0.4034	-0.0626	0.1184	0.0506	0.2091	0.3595168	Mineral
Longitude	GH47	15.3148	-1.3917	0.2505	0.1928	0.0574	0.1441206	Mineral
Longitude	GH46	2.3583	-1.0882	0.6855	0.6614	0.0001	0.0013812	Mineral
Longitude	GH48	9.3733	-0.6364	0.1435	0.0777	0.1637	0.3021622	Mineral
Longitude	GH49	0.174	-0.1791	0.8273	0.814	0.00E+00	6.51E-05	Mineral
Longitude	GH5	169.7639	-21.5753	0.619	0.5897	0.0005	0.0031453	Mineral
Longitude	GH50	7.8367	0.8954	0.1729	0.1092	0.1232	0.2468603	Mineral
Longitude	GH51	100.2913	2.0416	0.0189	-0.0566	0.6256	0.7412261	Mineral
Longitude	GH54	16.7488	1.1935	0.0226	-0.0526	0.5931	0.7279088	Mineral
Longitude	GH53	54.6684	-1.3022	0.0254	-0.0496	0.5704	0.7158278	Mineral
Longitude	GH55	98.5985	10.8125	0.2459	0.1879	0.0601	0.1479246	Mineral
Longitude	GH56	0.003	0.0017	0.2336	0.1746	0.068	0.1641239	Mineral
Longitude	GH57	155.9399	4.0269	0.0768	0.0057	0.3175	0.4888883	Mineral
Longitude	GH58	0.0426	-0.0234	0.6605	0.6344	0.0002	0.0019966	Mineral
Longitude	GH59	6.4703	0.226	0.0058	-0.0707	0.7882	0.8601623	Mineral
Longitude	GH6	17.654	-3.0467	0.3224	0.2703	0.0272	0.0814028	Mineral
Longitude	GH62	3.2335	-1.0199	0.5449	0.5099	0.0017	0.0085903	Mineral
Longitude	GH63	14.1513	-2.1574	0.3373	0.2864	0.0232	0.0727357	Mineral
Longitude	GH64	18.7715	-0.4401	0.0149	-0.0609	0.6652	0.7659032	Mineral
Longitude	GH65	100.9432	-3.1501	0.0161	-0.0595	0.6519	0.7591056	Mineral
Longitude	GH66	1.5117	-0.781	0.8683	0.8582	0.00E+00	3.11E-05	Mineral
Longitude	GH68	0.2737	-0.017	0.08	0.0093	0.3069	0.4784947	Mineral
Longitude	GH7	0.661	-0.2843	0.2282	0.1688	0.0717	0.1698316	Mineral
Longitude	GH70	0.0411	-0.0232	0.4766	0.4363	0.0044	0.0189911	Mineral
Longitude	GH71	3.5602	-0.5735	0.1056	0.0368	0.2374	0.3971759	Mineral
Longitude	GH72	30.0334	-2.8068	0.5758	0.5432	0.001	0.0055434	Mineral
Longitude	GH73	39.5823	-3.3156	0.6535	0.6268	0.0003	0.0022154	Mineral
Longitude	GH75	2.2715	-0.3719	0.326	0.2741	0.0262	0.0792923	Mineral
Longitude	GH74	12.8907	-1.5743	0.3768	0.3289	0.0149	0.0518256	Mineral
Longitude	GH76	13.3927	-1.2432	0.0773	0.0063	0.3157	0.4888883	Mineral
Longitude	GH78	148.9518	-6.1759	0.1207	0.053	0.2046	0.3568026	Mineral
Longitude	GH79	25.909	4.2203	0.6305	0.6021	0.0004	0.0026942	Mineral
Longitude	GH8	19.2424	-3.6084	0.4547	0.4127	0.0058	0.0239988	Mineral
Longitude	GH80	0.0028	0.0004	0.0027	-0.0741	0.8552	0.9057521	Mineral
Longitude	GH81	6.0259	-1.6613	0.7816	0.7648	0.00E+00	0.0002194	Mineral
Longitude	GH84	4.7774	-0.6967	0.6395	0.6118	0.0003	0.0024782	Mineral
Longitude	GH85	0.6451	-0.1961	0.4289	0.3849	0.0081	0.0316084	Mineral
Longitude	GH86	1.7515	-0.4139	0.8709	0.8609	0.00E+00	3.11E-05	Mineral
Longitude	GH87	64.8133	-1.3127	0.0245	-0.0505	0.5773	0.7209317	Mineral
Longitude	GH88	24.9678	-1.9257	0.7152	0.6933	1.00E-04	0.000894	Mineral
Longitude	GH89	6.8897	-0.2229	0.0098	-0.0663	0.7253	0.8090803	Mineral
Longitude	GH91	0.106	-0.0604	0.6368	0.6089	0.0004	0.0025316	Mineral
Longitude	GH90	0.0045	-0.0048	0.6307	0.6023	0.0004	0.0026942	Mineral
Longitude	GH9	51.5097	-4.6617	0.1265	0.0593	0.1933	0.3464905	Mineral

Longitude	GH92	91.0695	-8.6481	0.1022	0.0331	0.2455	0.4080155	Mineral
Longitude	GH93	22.1339	-10.3322	0.6396	0.6118	0.0003	0.0024782	Mineral
Longitude	GH94	27.0425	3.1431	0.283	0.2278	0.0412	0.1125271	Mineral
Longitude	GH95	48.6912	-6.3817	0.4799	0.4399	0.0042	0.0184932	Mineral
Longitude	GH96	0.5474	-0.3461	0.7693	0.7516	0.00E+00	0.0002938	Mineral
Longitude	GH97	32.6174	-4.6821	0.4737	0.4332	0.0046	0.0190981	Mineral
Longitude	GH98	0.3397	-0.0447	0.3066	0.2533	0.0322	0.0929665	Mineral
Longitude	GH99	13.1032	-2.6473	0.8379	0.8255	0.00E+00	6.13E-05	Mineral
Longitude	GT1	153.6974	-17.9709	0.3127	0.2598	0.0302	0.0881995	Mineral
Longitude	GT10	2.4352	-0.0145	0.0012	-0.0757	0.9036	0.9333499	Mineral
Longitude	GT12	0.0482	0.0021	0.0098	-0.0663	0.7251	0.8090803	Mineral
Longitude	GT14	2.0647	-0.095	0.0303	-0.0443	0.5348	0.6884424	Mineral
Longitude	GT11	4.9506	0.2748	0.1199	0.0522	0.2061	0.3568026	Mineral
Longitude	GT13	0.1413	0.0139	0.0263	-0.0486	0.5634	0.7141805	Mineral
Longitude	GT15	1.84	-0.0624	0.0032	-0.0735	0.8414	0.8948775	Mineral
Longitude	GT16	0.0336	0.0046	0.022	-0.0532	0.5974	0.7279088	Mineral
Longitude	GT17	1.0318	-0.0309	0.0242	-0.0509	0.5802	0.7209773	Mineral
Longitude	GT18	0.0082	0.0018	0.0359	-0.0382	0.4986	0.6660926	Mineral
Longitude	GT2	1841.616	-48.6134	0.5108	0.4732	0.0027	0.0128828	Mineral
Longitude	GT19	83.4375	0.0619	0.0002	-0.0767	0.9617	0.9655406	Mineral
Longitude	GT20	150.7734	3.9527	0.5164	0.4792	0.0025	0.0122633	Mineral
Longitude	GT21	72.0818	14.6603	0.8042	0.7892	0.00E+00	0.0001145	Mineral
Longitude	GT22	4.3521	-0.5251	0.1661	0.102	0.1316	0.260041	Mineral
Longitude	GT23	0.2031	0.0103	0.0178	-0.0578	0.6356	0.7489945	Mineral
Longitude	GT24	2.657	-0.0403	0.0022	-0.0745	0.8678	0.9075583	Mineral
Longitude	GT25	5.9103	0.1137	0.0131	-0.0628	0.6849	0.7813652	Mineral
Longitude	GT26	133.0274	-4.7004	0.2311	0.172	0.0697	0.1665726	Mineral
Longitude	GT27	13.0687	0.5439	0.1757	0.1123	0.1199	0.2427661	Mineral
Longitude	GT28	177.3669	-4.2633	0.2706	0.2145	0.0468	0.1223901	Mineral
Longitude	GT29	3.9302	-0.4382	0.5237	0.4871	0.0023	0.0112644	Mineral
Longitude	GT30	120.1726	-0.8505	0.0188	-0.0567	0.6261	0.7412261	Mineral
Longitude	GT3	2.7042	-0.4484	0.2002	0.1386	0.0945	0.2044515	Mineral
Longitude	GT33	1.8007	-0.4276	0.6933	0.6697	0.0001	0.0013046	Mineral
Longitude	GT34	0.89	-0.1118	0.0491	-0.024	0.4273	0.5969753	Mineral
Longitude	GT32	12.395	-0.8573	0.1858	0.1232	0.1087	0.2287696	Mineral
Longitude	GT31	1.3616	-0.2644	0.0882	0.0181	0.2824	0.4573391	Mineral
Longitude	GT35	348.7674	16.9493	0.161	0.0964	0.1383	0.2630057	Mineral
Longitude	GT37	0.0045	0.0013	0.0808	0.0101	0.3044	0.4774907	Mineral
Longitude	GT39	20.8559	-3.341	0.3535	0.3038	0.0194	0.0616605	Mineral
Longitude	GT4	1887.0784	-139.8579	0.8512	0.8397	0.00E+00	4.90E-05	Mineral
Longitude	GT41	420.3074	23.3006	0.3603	0.3111	0.018	0.0594135	Mineral
Longitude	GT40	0.0012	0.0006	0.0554	-0.0173	0.3986	0.5783207	Mineral
Longitude	GT42	0.0617	0.0099	0.0359	-0.0383	0.4989	0.6660926	Mineral
Longitude	GT43	0.0665	0.0209	0.1629	0.0985	0.1357	0.2619886	Mineral
Longitude	GT47	67.1447	-1.7864	0.019	-0.0564	0.6241	0.7412261	Mineral
Longitude	GT45	0.2617	-0.0341	0.3759	0.3279	0.0151	0.0518256	Mineral
Longitude	GT44	0.0225	-0.0039	0.0352	-0.039	0.5031	0.6680992	Mineral
Longitude	GT48	3.0789	0.1034	0.0034	-0.0733	0.8371	0.8941289	Mineral
Longitude	GT49	0.176	0.0254	0.0631	-0.0089	0.3663	0.5467623	Mineral
Longitude	GT5	111.8839	-1.2735	0.0259	-0.0491	0.5669	0.7150045	Mineral
Longitude	GT50	0.6419	-0.0467	0.0159	-0.0598	0.6546	0.7591056	Mineral
Longitude	GT53	23.5174	-0.5198	0.0036	-0.0731	0.8321	0.8925978	Mineral
Longitude	GT52	0	0	#VALUE!	#VALUE!	#VALUE!	NA	Mineral
Longitude	GT51	615.404	-45.5367	0.7656	0.7476	0.00E+00	0.0003064	Mineral
Longitude	GT54	0.0278	0.0036	0.0168	-0.0588	0.6451	0.7565892	Mineral

Longitude	GT57	1.3329	-0.083	0.0098	-0.0663	0.7253	0.8090803	Mineral
Longitude	GT56	0.3995	-0.0235	0.099	0.0297	0.2533	0.4155978	Mineral
Longitude	GT55	2.8539	-0.0704	0.005	-0.0715	0.8021	0.8715219	Mineral
Longitude	GT58	0.5128	-0.026	0.0077	-0.0686	0.7559	0.8395486	Mineral
Longitude	GT6	0.334	0.0941	0.3315	0.2801	0.0247	0.0765212	Mineral
Longitude	GT59	0.3598	-0.058	0.0635	-0.0086	0.3651	0.5467623	Mineral
Longitude	GT60	1.8074	0.1744	0.0475	-0.0257	0.435	0.5998639	Mineral
Longitude	GT61	0.3572	-0.0253	0.0339	-0.0404	0.5111	0.6716414	Mineral
Longitude	GT62	2.1958	-0.1339	0.0556	-0.017	0.3974	0.5783207	Mineral
Longitude	GT64	0.9005	-0.1809	0.6713	0.646	0.0002	0.0017287	Mineral
Longitude	GT65	0.0411	0.009	0.1501	0.0848	0.1536	0.2855596	Mineral
Longitude	GT66	19.8555	-2.3291	0.647	0.6198	0.0003	0.0024282	Mineral
Longitude	GT69	0.7751	-0.1172	0.0469	-0.0264	0.4381	0.6008235	Mineral
Longitude	GT68	0.0223	0.0016	0.0122	-0.0637	0.6947	0.7877729	Mineral
Longitude	GT7	5.4876	0.2163	0.1172	0.0493	0.2117	0.3614486	Mineral
Longitude	GT72	0.0008	0.0007	0.1242	0.0569	0.1976	0.3492263	Mineral
Longitude	GT73	0.0663	-0.0193	0.1107	0.0423	0.2255	0.3824636	Mineral
Longitude	GT71	1.4059	-0.18	0.1783	0.115	0.117	0.2424213	Mineral
Longitude	GT70	0.7826	-0.0062	0.0018	-0.075	0.882	0.9186051	Mineral
Longitude	GT74	0.8924	0.2485	0.7485	0.7292	0.00E+00	0.0004346	Mineral
Longitude	GT75	0.9563	0.0735	0.1633	0.0989	0.1352	0.2619886	Mineral
Longitude	GT76	2.3528	-0.2058	0.1201	0.0524	0.2058	0.3568026	Mineral
Longitude	GT77	0.9469	0.0436	0.0496	-0.0235	0.425	0.5969753	Mineral
Longitude	GT78	0.0018	-0.0003	0.006	-0.0704	0.7834	0.8601623	Mineral
Longitude	GT8	11.7518	-1.979	0.5997	0.5689	0.0007	0.0042883	Mineral
Longitude	GT80	5.6377	-0.0406	0.0025	-0.0743	0.8606	0.9075583	Mineral
Longitude	GT82	0.4951	-0.0886	0.3574	0.308	0.0186	0.059783	Mineral
Longitude	GT84	296.2703	17.2471	0.0864	0.0161	0.2876	0.4622057	Mineral
Longitude	GT81	24.419	-2.3368	0.4736	0.4331	0.0046	0.0190981	Mineral
Longitude	GT83	171.2538	0.5278	0.0015	-0.0753	0.8916	0.9247967	Mineral
Longitude	GT85	3.2912	-0.0196	0.0002	-0.0767	0.9584	0.9655406	Mineral
Longitude	GT87	16.9111	-1.9636	0.1914	0.1292	0.1029	0.218928	Mineral
Longitude	GT88	0.0259	0.0109	0.2159	0.1556	0.081	0.184836	Mineral
Longitude	GT89	5.7924	-0.6982	0.1766	0.1133	0.1188	0.2424213	Mineral
Longitude	GT90	2.475	-0.2142	0.0318	-0.0427	0.5249	0.6826986	Mineral
Longitude	GT9	175.0926	-11.4096	0.545	0.51	0.0017	0.0085903	Mineral
Longitude	GT91	0.0159	-0.003	0.0298	-0.0448	0.5382	0.6892048	Mineral
Longitude	GT93	0.368	-0.0089	0.0396	-0.0343	0.477	0.6471777	Mineral
Longitude	GT94	0.7572	0.0004	0.00E+00	-0.0769	0.995	0.9950199	Mineral
Longitude	GT92	0.0971	0.0011	0.0003	-0.0766	0.9535	0.9655406	Mineral
Longitude	GT95	7.0408	0.2422	0.0859	0.0156	0.2891	0.4622057	Mineral
Longitude	PL1	18.5567	-5.1932	0.4866	0.4471	0.0038	0.017211	Mineral
Longitude	PL10	4.7494	-1.4411	0.391	0.3442	0.0127	0.0467616	Mineral
Longitude	PL11	3.638	-1.1655	0.4339	0.3903	0.0076	0.0301854	Mineral
Longitude	PL12	23.2607	-2.3065	0.595	0.5639	0.0008	0.0044234	Mineral
Longitude	PL13	0.0005	0.0005	0.0643	-0.0077	0.3619	0.5467623	Mineral
Longitude	PL14	1.1325	-0.4806	0.6923	0.6686	0.0001	0.0013046	Mineral
Longitude	PL15	0.3857	-0.2003	0.8416	0.8294	0.00E+00	6.13E-05	Mineral
Longitude	PL17	1.8343	-0.9063	0.8278	0.8146	0.00E+00	6.51E-05	Mineral
Longitude	PL18	0.0042	-0.0014	0.0824	0.0118	0.2997	0.4761362	Mineral
Longitude	PL16	0.52	0.0033	0.0007	-0.0762	0.9257	0.9522823	Mineral
Longitude	PL2	0.1389	0.021	0.1771	0.1138	0.1183	0.2424213	Mineral
Longitude	PL20	0.0975	-0.0601	0.5072	0.4693	0.0029	0.0131985	Mineral
Longitude	PL21	0.4953	0.1027	0.3295	0.2779	0.0252	0.0772804	Mineral
Longitude	PL22	9.2334	-1.0078	0.371	0.3226	0.0159	0.0540754	Mineral

Longitude	PL23	0.0157	-0.0014	0.0305	-0.0441	0.5338	0.6884424	Mineral
Longitude	PL3	0.3266	-0.2346	0.4094	0.364	0.0102	0.0393978	Mineral
Longitude	PL4	15.4469	0.0648	0.0002	-0.0767	0.9576	0.9655406	Mineral
Longitude	PL5	1.6555	0.3915	0.0993	0.03	0.2526	0.4155978	Mineral
Longitude	PL6	2.3184	-1.1191	0.5934	0.5622	0.0008	0.0044398	Mineral
Longitude	PL7	3.659	-1.4162	0.596	0.5649	0.0007	0.0044234	Mineral
Longitude	PL8	2.5006	-0.7944	0.6432	0.6157	0.0003	0.0024533	Mineral
Longitude	PL9	33.1358	-7.9916	0.6608	0.6347	0.0002	0.0019966	Mineral
Elevation	AA1	161.1286	1.9191	0.0111	-0.065	0.7086	0.809612	Mineral
Elevation	AA11	0.406	0.1399	0.2042	0.143	0.0908	0.2349916	Mineral
Elevation	AA10	6.1103	0.7119	0.1572	0.0923	0.1435	0.3077803	Mineral
Elevation	AA2	1.4848	0.2784	0.2291	0.1698	0.0711	0.2005475	Mineral
Elevation	AA3	426.2452	21.0158	0.1985	0.1368	0.0961	0.240199	Mineral
Elevation	AA4	11.2554	-0.4618	0.0124	-0.0636	0.6927	0.807164	Mineral
Elevation	AA5	53.5482	4.3039	0.2928	0.2384	0.0373	0.1264002	Mineral
Elevation	AA8	0.6569	0.1817	0.3151	0.2624	0.0294	0.1090731	Mineral
Elevation	AA7	57.2187	3.136	0.1145	0.0463	0.2174	0.4012868	Mineral
Elevation	AA6	30.8574	0.2468	0.0037	-0.0729	0.8288	0.874051	Mineral
Elevation	AA9	1.4632	0.6301	0.2263	0.1668	0.0731	0.203766	Mineral
Elevation	CE1	216.8049	2.4715	0.029	-0.0457	0.544	0.682323	Mineral
Elevation	CE11	163.2288	1.8035	0.0347	-0.0395	0.5061	0.6650846	Mineral
Elevation	CE12	15.6874	2.5696	0.3501	0.3001	0.0201	0.0800274	Mineral
Elevation	CE13	0.0362	-0.0075	0.0812	0.0105	0.3033	0.4848418	Mineral
Elevation	CE14	221.0157	1.2504	0.0102	-0.0659	0.72	0.8099855	Mineral
Elevation	CE15	37.8269	0.563	0.007	-0.0694	0.7674	0.8374428	Mineral
Elevation	CE2	2.5005	0.7408	0.3051	0.2516	0.0328	0.117459	Mineral
Elevation	CE3	26.5046	9.2388	0.643	0.6156	0.0003	0.0052387	Mineral
Elevation	CE4	378.0703	38.5789	0.6615	0.6355	0.0002	0.0043675	Mineral
Elevation	CE16	1.0559	0.1333	0.1471	0.0815	0.1582	0.3281572	Mineral
Elevation	CE5	14.5299	-1.496	0.0564	-0.0162	0.394	0.5816685	Mineral
Elevation	CE7	10.4421	1.4514	0.349	0.2989	0.0204	0.0800274	Mineral
Elevation	CE6	37.5291	-4.2207	0.3148	0.2621	0.0295	0.1090731	Mineral
Elevation	CE8	22.1825	1.6433	0.1118	0.0435	0.2232	0.4045138	Mineral
Elevation	CE9	94.9446	1.7667	0.1738	0.1102	0.1222	0.2762153	Mineral
Elevation	GH100	0.6919	0.0868	0.0605	-0.0117	0.3767	0.5765549	Mineral
Elevation	GH10	53.2214	1.5745	0.0572	-0.0153	0.3907	0.580264	Mineral
Elevation	GH102	43.2037	2.1734	0.4196	0.375	0.009	0.0443906	Mineral
Elevation	GH101	0.8491	0.2757	0.583	0.5509	0.0009	0.0110502	Mineral
Elevation	GH103	113.5215	11.0633	0.4996	0.4611	0.0032	0.0218241	Mineral
Elevation	GH104	5.515	0.6889	0.4689	0.4281	0.0049	0.0304432	Mineral
Elevation	GH105	25.0043	0.3844	0.0024	-0.0744	0.8632	0.8953457	Mineral
Elevation	GH106	43.4576	-0.4782	0.0025	-0.0743	0.8605	0.8953457	Mineral
Elevation	GH107	0.1988	0.1146	0.6266	0.5979	0.0004	0.0062347	Mineral
Elevation	GH108	6.4325	-0.0637	0.0075	-0.0688	0.7588	0.8317276	Mineral
Elevation	GH109	19.7839	0.9384	0.1344	0.0678	0.1789	0.3567097	Mineral
Elevation	GH11	13.636	0.589	0.0405	-0.0334	0.4723	0.6477404	Mineral
Elevation	GH110	1.0855	0.0807	0.0505	-0.0226	0.4209	0.6092561	Mineral
Elevation	GH111	0.0484	0.013	0.2304	0.1712	0.0702	0.2001351	Mineral
Elevation	GH112	0.065	0.0087	0.0352	-0.039	0.5029	0.6644188	Mineral
Elevation	GH113	8.5535	1.6636	0.7535	0.7346	0.00E+00	0.0009771	Mineral
Elevation	GH114	11.4339	0.5357	0.0329	-0.0414	0.5174	0.6728352	Mineral
Elevation	GH115	15.0153	2.7462	0.2558	0.1986	0.0544	0.1606823	Mineral
Elevation	GH116	40.527	-5.7285	0.5419	0.5067	0.0018	0.0162934	Mineral
Elevation	GH117	3.5852	0.1492	0.021	-0.0543	0.6066	0.7319886	Mineral
Elevation	GH118	0.0033	0.0009	0.09	0.02	0.2773	0.4608961	Mineral

Elevation	GH119	3.1764	0.8583	0.6226	0.5936	0.0005	0.0062347	Mineral
Elevation	GH12	8.1909	0.7547	0.0925	0.0227	0.2703	0.4583844	Mineral
Elevation	GH120	1.8095	-0.5051	0.2359	0.1771	0.0664	0.1916652	Mineral
Elevation	GH121	1.9359	0.9763	0.5356	0.4999	0.0019	0.0163412	Mineral
Elevation	GH123	2.2624	0.5582	0.5354	0.4997	0.0019	0.0163412	Mineral
Elevation	GH124	0.0624	-0.006	0.0283	-0.0465	0.5491	0.682323	Mineral
Elevation	GH125	10.2003	-0.4748	0.0387	-0.0352	0.4822	0.6577411	Mineral
Elevation	GH126	0.0079	-0.0043	0.1837	0.1209	0.1109	0.2565817	Mineral
Elevation	GH127	40.8673	0.2376	0.0012	-0.0756	0.9023	0.9241853	Mineral
Elevation	GH128	6.089	0.3521	0.0286	-0.0461	0.5465	0.682323	Mineral
Elevation	GH129	0.3723	-0.0548	0.1285	0.0614	0.1896	0.3688278	Mineral
Elevation	GH13	1511.7028	14.0497	0.0123	-0.0637	0.6937	0.807164	Mineral
Elevation	GH130	83.298	2.2201	0.0323	-0.0421	0.5215	0.6728352	Mineral
Elevation	GH131	0.1722	0.0682	0.2763	0.2207	0.0442	0.1402948	Mineral
Elevation	GH132	0.6932	0.1412	0.0812	0.0105	0.3033	0.4848418	Mineral
Elevation	GH133	77.1918	-4.8314	0.2569	0.1997	0.0538	0.1606823	Mineral
Elevation	GH14	0.4871	0.0117	0.0124	-0.0636	0.6927	0.807164	Mineral
Elevation	GH17	62.1908	5.9444	0.4336	0.39	0.0076	0.0394071	Mineral
Elevation	GH15	404.3419	-22.2845	0.2865	0.2316	0.0398	0.1296704	Mineral
Elevation	GH18	240.9138	12.6177	0.1855	0.1228	0.109	0.2565817	Mineral
Elevation	GH19	17.4844	2.1831	0.3026	0.249	0.0336	0.118839	Mineral
Elevation	GH2	363.2682	2.7214	0.0041	-0.0725	0.8206	0.8727478	Mineral
Elevation	GH20	98.8613	2.9749	0.1134	0.0452	0.2197	0.4025869	Mineral
Elevation	GH23	402.7243	25.4939	0.5922	0.5608	0.0008	0.0099698	Mineral
Elevation	GH24	16.0614	0.2266	0.0166	-0.0591	0.6476	0.7776951	Mineral
Elevation	GH25	20.5589	2.2069	0.2138	0.1534	0.0826	0.220775	Mineral
Elevation	GH26	21.4128	1.4653	0.0939	0.0242	0.2667	0.4554493	Mineral
Elevation	GH27	70.6768	-0.9726	0.0042	-0.0724	0.8177	0.8727478	Mineral
Elevation	GH28	177.5732	2.6971	0.0103	-0.0658	0.719	0.8099855	Mineral
Elevation	GH29	104.4902	-7.4828	0.3679	0.3192	0.0165	0.0702912	Mineral
Elevation	GH3	550.2428	-10.5342	0.0252	-0.0498	0.5718	0.698394	Mineral
Elevation	GH30	48.4301	1.2341	0.026	-0.0489	0.5657	0.698394	Mineral
Elevation	GH31	125.157	-10.6825	0.2883	0.2336	0.039	0.128916	Mineral
Elevation	GH33	63.7208	6.7833	0.5165	0.4793	0.0025	0.0205449	Mineral
Elevation	GH36	81.126	-4.5467	0.1233	0.0559	0.1993	0.3848959	Mineral
Elevation	GH32	31.8356	-1.056	0.1872	0.1246	0.1073	0.2565817	Mineral
Elevation	GH37	36.4506	-5.6832	0.5706	0.5376	0.0011	0.0121551	Mineral
Elevation	GH39	81.1725	-3.4062	0.1343	0.0677	0.1791	0.3567097	Mineral
Elevation	GH38	103.1959	-4.2275	0.0465	-0.0268	0.4401	0.6184858	Mineral
Elevation	GH4	145.5656	14.3441	0.3067	0.2534	0.0322	0.1170846	Mineral
Elevation	GH42	42.3015	-0.8218	0.025	-0.0499	0.5732	0.698394	Mineral
Elevation	GH43	105.7244	8.6317	0.0901	0.0201	0.277	0.4608961	Mineral
Elevation	GH44	24.2141	1.9414	0.2896	0.2349	0.0385	0.1289065	Mineral
Elevation	GH45	0.4034	0.0662	0.1324	0.0657	0.1825	0.3605995	Mineral
Elevation	GH47	15.3148	1.0571	0.1445	0.0787	0.1622	0.3309464	Mineral
Elevation	GH46	2.3583	0.7773	0.3498	0.2998	0.0202	0.0800274	Mineral
Elevation	GH48	9.3733	0.2932	0.0305	-0.0441	0.5338	0.675304	Mineral
Elevation	GH49	0.174	0.1805	0.8408	0.8285	0.00E+00	0.000191	Mineral
Elevation	GH5	169.7639	16.6624	0.3692	0.3207	0.0163	0.0702912	Mineral
Elevation	GH50	7.8367	-1.5002	0.4852	0.4456	0.0039	0.0251778	Mineral
Elevation	GH51	100.2913	-3.1619	0.0452	-0.0282	0.4467	0.6194794	Mineral
Elevation	GH54	16.7488	-1.6206	0.0416	-0.0321	0.4659	0.6425554	Mineral
Elevation	GH53	54.6684	3.4614	0.1796	0.1164	0.1155	0.2635245	Mineral
Elevation	GH55	98.5985	-11.2993	0.2686	0.2123	0.0478	0.15003	Mineral
Elevation	GH56	0.003	-0.0014	0.1554	0.0905	0.1459	0.3077831	Mineral

Elevation	GH57	155.9399	-2.6382	0.0329	-0.0414	0.5174	0.6728352	Mineral
Elevation	GH58	0.0426	0.0213	0.5494	0.5148	0.0016	0.0151152	Mineral
Elevation	GH59	6.4703	-0.5685	0.0364	-0.0377	0.4958	0.6620033	Mineral
Elevation	GH6	17.654	2.3231	0.1874	0.1249	0.107	0.2565817	Mineral
Elevation	GH62	3.2335	0.6865	0.2469	0.1889	0.0595	0.1737804	Mineral
Elevation	GH63	14.1513	1.5943	0.1842	0.1215	0.1104	0.2565817	Mineral
Elevation	GH64	18.7715	-0.2485	0.0047	-0.0718	0.8075	0.8661158	Mineral
Elevation	GH65	100.9432	-5.9809	0.0582	-0.0143	0.3865	0.5782079	Mineral
Elevation	GH66	1.5117	0.7878	0.8834	0.8744	0.00E+00	4.94E-05	Mineral
Elevation	GH68	0.2737	0.0271	0.2044	0.1432	0.0907	0.2349916	Mineral
Elevation	GH7	0.661	0.3239	0.2962	0.242	0.036	0.1236608	Mineral
Elevation	GH70	0.0411	0.0235	0.488	0.4486	0.0038	0.0248817	Mineral
Elevation	GH71	3.5602	0.8109	0.2111	0.1504	0.0849	0.224386	Mineral
Elevation	GH72	30.0334	2.6299	0.5055	0.4675	0.003	0.0215918	Mineral
Elevation	GH73	39.5823	2.3529	0.3291	0.2775	0.0254	0.0964177	Mineral
Elevation	GH75	2.2715	0.2252	0.1195	0.0518	0.2069	0.3908666	Mineral
Elevation	GH74	12.8907	1.3169	0.2637	0.207	0.0503	0.1538238	Mineral
Elevation	GH76	13.3927	0.5574	0.0155	-0.0602	0.658	0.786452	Mineral
Elevation	GH78	148.9518	3.8418	0.0467	-0.0266	0.4392	0.6184858	Mineral
Elevation	GH79	25.909	-3.496	0.4326	0.389	0.0077	0.0394071	Mineral
Elevation	GH8	19.2424	2.1707	0.1645	0.1003	0.1336	0.2967157	Mineral
Elevation	GH80	0.0028	-0.0028	0.1177	0.0498	0.2106	0.394455	Mineral
Elevation	GH81	6.0259	1.5742	0.7018	0.6789	1.00E-04	0.0023155	Mineral
Elevation	GH84	4.7774	0.637	0.5346	0.4988	0.002	0.0163412	Mineral
Elevation	GH85	0.6451	0.1183	0.1561	0.0912	0.145	0.3077831	Mineral
Elevation	GH86	1.7515	0.3885	0.7671	0.7492	0.00E+00	0.0007943	Mineral
Elevation	GH87	64.8133	2.3051	0.0756	0.0045	0.3213	0.5103821	Mineral
Elevation	GH88	24.9678	1.8331	0.6481	0.621	0.0003	0.0052387	Mineral
Elevation	GH89	6.8897	0.0598	0.0007	-0.0762	0.9251	0.9400974	Mineral
Elevation	GH91	0.106	0.0488	0.4146	0.3696	0.0096	0.0462025	Mineral
Elevation	GH90	0.0045	0.0051	0.7221	0.7007	1.00E-04	0.0016878	Mineral
Elevation	GH9	51.5097	0.6879	0.0028	-0.074	0.8526	0.8917272	Mineral
Elevation	GH92	91.0695	3.1064	0.0132	-0.0627	0.6837	0.807164	Mineral
Elevation	GH93	22.1339	9.743	0.5687	0.5355	0.0012	0.0121551	Mineral
Elevation	GH94	27.0425	-3.8885	0.4331	0.3895	0.0076	0.0394071	Mineral
Elevation	GH95	48.6912	5.0431	0.2997	0.2458	0.0347	0.1208524	Mineral
Elevation	GH96	0.5474	0.2665	0.4561	0.4142	0.0057	0.0342518	Mineral
Elevation	GH97	32.6174	2.5409	0.1395	0.0733	0.1703	0.3446982	Mineral
Elevation	GH98	0.3397	0.0525	0.4233	0.3789	0.0086	0.0433348	Mineral
Elevation	GH99	13.1032	2.0592	0.507	0.4691	0.0029	0.0215918	Mineral
Elevation	GT1	153.6974	10.5268	0.1073	0.0386	0.2333	0.4178338	Mineral
Elevation	GT10	2.4352	0.1304	0.0953	0.0257	0.263	0.4520933	Mineral
Elevation	GT12	0.0482	-0.003	0.0211	-0.0542	0.6056	0.7319886	Mineral
Elevation	GT14	2.0647	-0.0528	0.0094	-0.0668	0.7315	0.8099855	Mineral
Elevation	GT11	4.9506	-0.1549	0.0381	-0.0359	0.4856	0.6588682	Mineral
Elevation	GT13	0.1413	-0.0165	0.0371	-0.037	0.4917	0.6620033	Mineral
Elevation	GT15	1.84	0.1962	0.0315	-0.043	0.5266	0.6743341	Mineral
Elevation	GT16	0.0336	-0.0002	0.00E+00	-0.0769	0.9802	0.9841638	Mineral
Elevation	GT17	1.0318	0.0568	0.0817	0.0111	0.3018	0.4848418	Mineral
Elevation	GT18	0.0082	-0.0016	0.0302	-0.0444	0.5354	0.675304	Mineral
Elevation	GT2	1841.616	48.2445	0.5031	0.4649	0.0031	0.0215918	Mineral
Elevation	GT19	83.4375	0.5335	0.0137	-0.0622	0.6777	0.8061731	Mineral
Elevation	GT20	150.7734	-4.5593	0.6871	0.6631	0.0001	0.0027933	Mineral
Elevation	GT21	72.0818	-12.8971	0.6224	0.5934	0.0005	0.0062347	Mineral
Elevation	GT22	4.3521	0.513	0.1585	0.0938	0.1416	0.3063947	Mineral

Elevation	GT23	0.2031	-0.0048	0.0039	-0.0728	0.8261	0.874051	Mineral
Elevation	GT24	2.657	0.0825	0.0093	-0.0669	0.7325	0.8099855	Mineral
Elevation	GT25	5.9103	0.087	0.0077	-0.0687	0.7564	0.8317276	Mineral
Elevation	GT26	133.0274	6.4773	0.4389	0.3957	0.0071	0.0394071	Mineral
Elevation	GT27	13.0687	-0.4105	0.1	0.0308	0.2508	0.4401321	Mineral
Elevation	GT28	177.3669	4.9704	0.3679	0.3192	0.0165	0.0702912	Mineral
Elevation	GT29	3.9302	0.3196	0.2786	0.2231	0.0431	0.1388484	Mineral
Elevation	GT30	120.1726	1.8003	0.0842	0.0138	0.294	0.4791406	Mineral
Elevation	GT3	2.7042	0.4012	0.1602	0.0956	0.1393	0.3039756	Mineral
Elevation	GT33	1.8007	0.4629	0.8125	0.798	0.00E+00	0.0003739	Mineral
Elevation	GT34	0.89	0.135	0.0716	0.0002	0.335	0.5222566	Mineral
Elevation	GT32	12.395	1.2782	0.4131	0.368	0.0098	0.0462025	Mineral
Elevation	GT31	1.3616	0.3206	0.1297	0.0627	0.1874	0.3674587	Mineral
Elevation	GT35	348.7674	-29.9429	0.5023	0.464	0.0031	0.0215918	Mineral
Elevation	GT37	0.0045	-0.0007	0.0256	-0.0493	0.5687	0.698394	Mineral
Elevation	GT39	20.8559	2.6331	0.2196	0.1595	0.0781	0.213096	Mineral
Elevation	GT4	1887.0784	134.9702	0.7927	0.7768	0.00E+00	0.0005436	Mineral
Elevation	GT41	420.3074	-25.741	0.4397	0.3966	0.007	0.0394071	Mineral
Elevation	GT40	0.0012	-0.0008	0.1055	0.0367	0.2375	0.419781	Mineral
Elevation	GT42	0.0617	0.0002	0.00E+00	-0.0769	0.9897	0.9897274	Mineral
Elevation	GT43	0.0665	-0.0139	0.0727	0.0014	0.3311	0.5222566	Mineral
Elevation	GT47	67.1447	4.5102	0.1212	0.0536	0.2035	0.3899346	Mineral
Elevation	GT45	0.2617	0.0248	0.199	0.1374	0.0956	0.240199	Mineral
Elevation	GT44	0.0225	-0.0016	0.0061	-0.0704	0.7827	0.8431225	Mineral
Elevation	GT48	3.0789	0.1722	0.0093	-0.0669	0.7318	0.8099855	Mineral
Elevation	GT49	0.176	-0.0112	0.0122	-0.0637	0.6946	0.807164	Mineral
Elevation	GT5	111.8839	2.7361	0.1194	0.0517	0.2071	0.3908666	Mineral
Elevation	GT50	0.6419	0.0801	0.0466	-0.0267	0.4395	0.6184858	Mineral
Elevation	GT53	23.5174	-2.1126	0.0592	-0.0132	0.3821	0.5778182	Mineral
Elevation	GT52	0	0	#VALUE!	#VALUE!	#VALUE!	NA	Mineral
Elevation	GT51	615.404	44.8865	0.7439	0.7242	0.00E+00	0.0011029	Mineral
Elevation	GT54	0.0278	-0.0022	0.0064	-0.0701	0.7775	0.8411835	Mineral
Elevation	GT57	1.3329	0.1476	0.031	-0.0435	0.53	0.6752381	Mineral
Elevation	GT56	0.3995	0.0164	0.0485	-0.0246	0.4301	0.6168567	Mineral
Elevation	GT55	2.8539	0.0333	0.0011	-0.0757	0.9058	0.9241853	Mineral
Elevation	GT58	0.5128	0.0567	0.0365	-0.0377	0.4955	0.6620033	Mineral
Elevation	GT6	0.334	-0.0722	0.1951	0.1332	0.0992	0.2442238	Mineral
Elevation	GT59	0.3598	0.0688	0.0891	0.019	0.2799	0.4622713	Mineral
Elevation	GT60	1.8074	-0.2147	0.0721	0.0007	0.3334	0.5222566	Mineral
Elevation	GT61	0.3572	0.0052	0.0014	-0.0754	0.8937	0.9193346	Mineral
Elevation	GT62	2.1958	0.1714	0.0911	0.0212	0.2743	0.4608961	Mineral
Elevation	GT64	0.9005	0.1674	0.5751	0.5424	0.001	0.0119726	Mineral
Elevation	GT65	0.0411	-0.0091	0.1542	0.0892	0.1476	0.3087395	Mineral
Elevation	GT66	19.8555	1.7694	0.3734	0.3252	0.0155	0.0695391	Mineral
Elevation	GT69	0.7751	0.1579	0.0852	0.0149	0.2911	0.4774811	Mineral
Elevation	GT68	0.0223	0.0003	0.0005	-0.0764	0.9355	0.9468054	Mineral
Elevation	GT7	5.4876	-0.2155	0.1163	0.0483	0.2136	0.3970702	Mineral
Elevation	GT72	0.0008	-0.0004	0.0463	-0.027	0.4411	0.6184858	Mineral
Elevation	GT73	0.0663	0.0239	0.1687	0.1048	0.1283	0.2875488	Mineral
Elevation	GT71	1.4059	0.1392	0.1067	0.038	0.2347	0.4178338	Mineral
Elevation	GT70	0.7826	0.0459	0.0978	0.0284	0.2564	0.4457619	Mineral
Elevation	GT74	0.8924	-0.19	0.4377	0.3945	0.0072	0.0394071	Mineral
Elevation	GT75	0.9563	-0.0388	0.0456	-0.0278	0.4448	0.6194794	Mineral
Elevation	GT76	2.3528	0.1431	0.0581	-0.0144	0.387	0.5782079	Mineral
Elevation	GT77	0.9469	-0.0208	0.0113	-0.0648	0.7066	0.809612	Mineral

Elevation	GT78	0.0018	-0.0004	0.0099	-0.0662	0.7239	0.8099855	Mineral
Elevation	GT8	11.7518	1.7292	0.4578	0.4161	0.0056	0.0342518	Mineral
Elevation	GT80	5.6377	0.2065	0.0638	-0.0082	0.3637	0.5600656	Mineral
Elevation	GT82	0.4951	0.0758	0.2616	0.2048	0.0513	0.1551798	Mineral
Elevation	GT84	296.2703	0.4961	1.00E-04	-0.0768	0.9761	0.9839823	Mineral
Elevation	GT81	24.419	1.5104	0.1978	0.1361	0.0967	0.240199	Mineral
Elevation	GT83	171.2538	1.433	0.0109	-0.0652	0.7108	0.809612	Mineral
Elevation	GT85	3.2912	-0.3533	0.0703	-0.0012	0.3394	0.5258572	Mineral
Elevation	GT87	16.9111	1.0051	0.0501	-0.0229	0.4224	0.6092561	Mineral
Elevation	GT88	0.0259	-0.0145	0.3835	0.3361	0.0138	0.0630455	Mineral
Elevation	GT89	5.7924	0.1798	0.0117	-0.0643	0.7009	0.809612	Mineral
Elevation	GT90	2.475	0.2716	0.0511	-0.0219	0.4178	0.6092561	Mineral
Elevation	GT9	175.0926	12.3766	0.6413	0.6137	0.0003	0.0052387	Mineral
Elevation	GT91	0.0159	0.0043	0.0594	-0.013	0.3815	0.5778182	Mineral
Elevation	GT93	0.368	0.0103	0.0526	-0.0203	0.411	0.6033012	Mineral
Elevation	GT94	0.7572	-0.0422	0.0357	-0.0385	0.5002	0.6642365	Mineral
Elevation	GT92	0.0971	0.0071	0.0108	-0.0653	0.7128	0.809612	Mineral
Elevation	GT95	7.0408	-0.3553	0.1848	0.1221	0.1098	0.2565817	Mineral
Elevation	PL1	18.5567	3.3288	0.1999	0.1384	0.0947	0.240199	Mineral
Elevation	PL10	4.7494	0.9865	0.1832	0.1204	0.1114	0.2565817	Mineral
Elevation	PL11	3.638	0.713	0.1624	0.098	0.1364	0.3002799	Mineral
Elevation	PL12	23.2607	1.7825	0.3554	0.3058	0.019	0.0794861	Mineral
Elevation	PL13	0.0005	-0.0003	0.0321	-0.0423	0.5227	0.6728352	Mineral
Elevation	PL14	1.1325	0.5057	0.7666	0.7487	0.00E+00	0.0007943	Mineral
Elevation	PL15	0.3857	0.1826	0.6997	0.6766	0.0001	0.0023155	Mineral
Elevation	PL17	1.8343	0.739	0.5504	0.5158	0.0015	0.0151152	Mineral
Elevation	PL18	0.0042	-0.0004	0.0067	-0.0697	0.7722	0.8390694	Mineral
Elevation	PL16	0.52	-0.0416	0.1114	0.0431	0.224	0.4045138	Mineral
Elevation	PL2	0.1389	-0.0156	0.0974	0.0279	0.2575	0.4457619	Mineral
Elevation	PL20	0.0975	0.0398	0.2228	0.163	0.0757	0.2086701	Mineral
Elevation	PL21	0.4953	-0.1199	0.4491	0.4067	0.0063	0.0365705	Mineral
Elevation	PL22	9.2334	1.1834	0.5116	0.474	0.0027	0.0213462	Mineral
Elevation	PL23	0.0157	-0.0003	0.0017	-0.0751	0.8847	0.9138002	Mineral
Elevation	PL3	0.3266	0.1696	0.2138	0.1533	0.0827	0.220775	Mineral
Elevation	PL4	15.4469	0.2446	0.0032	-0.0735	0.8407	0.8829558	Mineral
Elevation	PL5	1.6555	-0.4736	0.1454	0.0797	0.1608	0.3308472	Mineral
Elevation	PL6	2.3184	0.7479	0.265	0.2085	0.0496	0.1535656	Mineral
Elevation	PL7	3.659	1.0886	0.3521	0.3023	0.0197	0.0800274	Mineral
Elevation	PL8	2.5006	0.622	0.3943	0.3477	0.0122	0.0566678	Mineral
Elevation	PL9	33.1358	5.7098	0.3373	0.2863	0.0232	0.0895555	Mineral
DNA	AA1	179.0321	7.5154	0.2302	0.0378	0.3355	0.546006	Organic
DNA	AA11	0.5025	-0.0778	0.1743	-0.0321	0.4101	0.6154485	Organic
DNA	AA10	7.5616	0.9366	0.3651	0.2063	0.204	0.4235537	Organic
DNA	AA2	1.8202	0.1681	0.1142	-0.1072	0.5124	0.6714922	Organic
DNA	AA3	383.3947	-45.5841	0.7666	0.7082	0.0223	0.1911941	Organic
DNA	AA4	20.7999	4.8557	0.4209	0.2762	0.1634	0.3877621	Organic
DNA	AA5	32.5624	-7.4061	0.3725	0.2156	0.1982	0.4235537	Organic
DNA	AA8	0.8225	0.0948	0.2412	0.0514	0.3226	0.5397305	Organic
DNA	AA7	52.2765	-6.1799	0.4757	0.3446	0.1295	0.3706182	Organic
DNA	AA6	24.3337	-5.0203	0.9125	0.8907	0.003	0.1645202	Organic
DNA	AA9	1.8306	0.0226	0.0024	-0.2471	0.9272	0.9522022	Organic
DNA	CE1	208.9789	1.4257	0.0383	-0.2022	0.7104	0.8076638	Organic
DNA	CE11	155.7435	11.7019	0.6596	0.5745	0.0496	0.2330833	Organic
DNA	CE12	20.9813	3.8249	0.5397	0.4246	0.0963	0.2959452	Organic
DNA	CE13	0.0653	0.0213	0.1659	-0.0427	0.4229	0.6305199	Organic

DNA	CE14	227.4441	16.7148	0.7396	0.6745	0.028	0.1951435	Organic
DNA	CE15	52.1006	10.2841	0.7306	0.6633	0.0301	0.1951435	Organic
DNA	CE2	3.239	0.9108	0.8968	0.871	0.0041	0.1645202	Organic
DNA	CE3	15.1515	-1.4931	0.2453	0.0567	0.3178	0.5397305	Organic
DNA	CE4	294.1051	-42.0986	0.4814	0.3517	0.1263	0.3706182	Organic
DNA	CE16	1.0735	-0.0254	0.0168	-0.229	0.8066	0.8848186	Organic
DNA	CE5	14.9063	-4.6558	0.7444	0.6805	0.027	0.1951435	Organic
DNA	CE7	12.9532	2.288	0.8438	0.8048	0.0097	0.1645202	Organic
DNA	CE6	52.0839	7.2021	0.4312	0.2891	0.1566	0.3821938	Organic
DNA	CE8	24.9683	3.9949	0.7554	0.6943	0.0246	0.1951435	Organic
DNA	CE9	90.7663	1.4698	0.1195	-0.1006	0.5021	0.6614357	Organic
DNA	GH100	1.1884	0.291	0.1042	-0.1198	0.5327	0.6880822	Organic
DNA	GH10	59.1017	6.6637	0.7133	0.6417	0.0344	0.1989281	Organic
DNA	GH102	34.7782	-1.7166	0.3693	0.2117	0.2006	0.4235537	Organic
DNA	GH101	0.4319	-0.0537	0.4368	0.296	0.153	0.377849	Organic
DNA	GH103	87.9513	-14.9609	0.6489	0.5612	0.053	0.2445316	Organic
DNA	GH104	4.2879	-0.0644	0.0082	-0.2398	0.8648	0.9241796	Organic
DNA	GH105	39.1132	5.8252	0.2851	0.1063	0.2752	0.4930265	Organic
DNA	GH106	64.736	12.0633	0.6084	0.5105	0.0673	0.2730144	Organic
DNA	GH107	0.05	-0.0106	0.1742	-0.0323	0.4103	0.6154485	Organic
DNA	GH108	6.1612	0.0949	0.0142	-0.2323	0.8221	0.8897523	Organic
DNA	GH109	20.7365	2.005	0.6312	0.539	0.059	0.2449114	Organic
DNA	GH11	18.0145	2.8392	0.455	0.3188	0.1416	0.3756058	Organic
DNA	GH110	1.6372	0.0786	0.0257	-0.2178	0.7614	0.8502043	Organic
DNA	GH111	0.0241	-0.0007	0.0063	-0.2422	0.8816	0.9301575	Organic
DNA	GH112	0.1865	0.1013	0.4669	0.3336	0.1346	0.3756058	Organic
DNA	GH113	7.4538	0.9949	0.5896	0.487	0.0746	0.2768264	Organic
DNA	GH114	8.4827	-2.1182	0.3274	0.1593	0.2354	0.447392	Organic
DNA	GH115	26.0154	8.8016	0.7272	0.6589	0.0309	0.1951435	Organic
DNA	GH116	54.6915	5.7146	0.4228	0.2786	0.1621	0.3877621	Organic
DNA	GH117	3.207	0.3303	0.3361	0.1701	0.2279	0.4409912	Organic
DNA	GH118	0.0019	0.0011	0.1285	-0.0893	0.4853	0.6531223	Organic
DNA	GH119	1.7272	0.3694	0.6369	0.5461	0.057	0.2449114	Organic
DNA	GH12	10.5185	2.2821	0.6364	0.5454	0.0572	0.2449114	Organic
DNA	GH120	2.0347	0.2235	0.1251	-0.0936	0.4915	0.658023	Organic
DNA	GH121	0.74	0.0301	0.0431	-0.1961	0.6931	0.7989767	Organic
DNA	GH123	0.8792	-0.4886	0.3234	0.1543	0.2389	0.4506573	Organic
DNA	GH124	0.0228	-0.0235	0.3649	0.2061	0.2041	0.4235537	Organic
DNA	GH125	14.4186	0.3972	0.0137	-0.2329	0.8254	0.8897523	Organic
DNA	GH126	0.0126	0.0004	0.0014	-0.2482	0.943	0.9584159	Organic
DNA	GH127	53.4987	9.1349	0.7225	0.6531	0.0321	0.1951435	Organic
DNA	GH128	9.2104	0.6413	0.0812	-0.1485	0.5842	0.7272745	Organic
DNA	GH129	0.4364	0.1462	0.8619	0.8274	0.0075	0.1645202	Organic
DNA	GH13	1477.2569	-34.2225	0.2456	0.057	0.3175	0.5397305	Organic
DNA	GH130	95.5733	13.9703	0.8824	0.853	0.0054	0.1645202	Organic
DNA	GH131	0.252	0.0248	0.0729	-0.1589	0.6049	0.7347168	Organic
DNA	GH132	0.9233	-0.0969	0.153	-0.0588	0.4433	0.6339946	Organic
DNA	GH133	59.9767	-4.6218	0.2112	0.0139	0.3592	0.580185	Organic
DNA	GH14	0.6765	0.0407	0.0461	-0.1924	0.683	0.7947258	Organic
DNA	GH17	51.2924	-6.7781	0.5943	0.4929	0.0727	0.2768264	Organic
DNA	GH15	420.1827	7.0621	0.0464	-0.192	0.682	0.7947258	Organic
DNA	GH18	255.9295	16.9664	0.407	0.2588	0.1729	0.4012513	Organic
DNA	GH19	15.6439	0.3383	0.0438	-0.1953	0.6908	0.7989767	Organic
DNA	GH2	452.5084	72.468	0.8223	0.7779	0.0126	0.1645202	Organic
DNA	GH20	103.5535	5.5469	0.163	-0.0462	0.4273	0.6332942	Organic

DNA	GH23	366.3567	0.6724	0.0036	-0.2455	0.9096	0.9476957	Organic
DNA	GH24	15.7222	1.4741	0.719	0.6488	0.0329	0.1951435	Organic
DNA	GH25	18.1223	0.6844	0.2435	0.0544	0.3199	0.5397305	Organic
DNA	GH26	23.6541	3.0644	0.5951	0.4938	0.0724	0.2768264	Organic
DNA	GH27	104.8235	16.875	0.5683	0.4603	0.0834	0.2823084	Organic
DNA	GH28	237.447	44.5284	0.7734	0.7168	0.0209	0.1860921	Organic
DNA	GH29	132.2116	18.0685	0.8584	0.823	0.0079	0.1645202	Organic
DNA	GH3	680.4412	74.0663	0.586	0.4825	0.076	0.2768264	Organic
DNA	GH30	59.6043	3.174	0.1452	-0.0686	0.4562	0.6339946	Organic
DNA	GH31	173.7587	20.4433	0.5589	0.4486	0.0875	0.2823084	Organic
DNA	GH33	48.5541	2.2527	0.2097	0.0121	0.3612	0.580185	Organic
DNA	GH36	117.4663	18.7642	0.5934	0.4918	0.0731	0.2768264	Organic
DNA	GH32	30.1748	-1.3133	0.1495	-0.0631	0.4489	0.6339946	Organic
DNA	GH37	45.5476	2.9964	0.1459	-0.0677	0.455	0.6339946	Organic
DNA	GH39	110.1752	17.5401	0.8252	0.7814	0.0122	0.1645202	Organic
DNA	GH38	153.8558	19.7893	0.3353	0.1692	0.2285	0.4409912	Organic
DNA	GH4	141.0485	-1.1271	0.007	-0.2412	0.8744	0.9301575	Organic
DNA	GH42	52.5554	4.0224	0.7776	0.722	0.0201	0.1860921	Organic
DNA	GH43	133.3589	20.7484	0.6065	0.5082	0.068	0.2730144	Organic
DNA	GH44	32.7913	7.2148	0.7753	0.7191	0.0206	0.1860921	Organic
DNA	GH45	0.5846	0.1648	0.6394	0.5493	0.0562	0.2449114	Organic
DNA	GH47	14.6845	-0.0817	0.002	-0.2475	0.9333	0.9524724	Organic
DNA	GH46	1.4741	-0.2556	0.4014	0.2517	0.1768	0.4012513	Organic
DNA	GH48	10.4606	1.5192	0.727	0.6587	0.031	0.1951435	Organic
DNA	GH49	0.0919	-0.0027	0.0137	-0.2329	0.8253	0.8897523	Organic
DNA	GH5	172.0215	9.9876	0.8494	0.8117	0.009	0.1645202	Organic
DNA	GH50	7.7274	0.4434	0.0698	-0.1628	0.613	0.737332	Organic
DNA	GH51	136.9965	23.2768	0.8519	0.8148	0.0087	0.1645202	Organic
DNA	GH54	33.242	6.1709	0.2776	0.097	0.2828	0.5029602	Organic
DNA	GH53	65.4769	11.4796	0.9555	0.9443	0.0008	0.1645202	Organic
DNA	GH55	145.3713	16.4456	0.2641	0.0802	0.297	0.5211764	Organic
DNA	GH56	0.0085	0.0054	0.2863	0.1078	0.274	0.4930265	Organic
DNA	GH57	148.2848	7.3038	0.1842	-0.0197	0.3957	0.6075103	Organic
DNA	GH58	0.0299	-0.0005	0.0025	-0.2469	0.925	0.9522022	Organic
DNA	GH59	11.8995	1.8739	0.2431	0.0538	0.3204	0.5397305	Organic
DNA	GH6	21.9717	3.0405	0.5571	0.4464	0.0883	0.2823084	Organic
DNA	GH62	3.402	1.1173	0.8418	0.8023	0.0099	0.1645202	Organic
DNA	GH63	18.035	2.8147	0.6682	0.5852	0.047	0.2266657	Organic
DNA	GH64	23.3696	2.5984	0.4207	0.2759	0.1635	0.3877621	Organic
DNA	GH65	99.333	-0.6517	0.0022	-0.2472	0.9293	0.9522022	Organic
DNA	GH66	1.6072	0.3841	0.4364	0.2954	0.1533	0.377849	Organic
DNA	GH68	0.3029	-0.0283	0.1329	-0.0839	0.4775	0.6461175	Organic
DNA	GH7	0.8798	0.0823	0.1361	-0.0798	0.4717	0.6417854	Organic
DNA	GH70	0.0313	0.0107	0.4391	0.2988	0.1515	0.377849	Organic
DNA	GH71	4.8837	-0.1803	0.0126	-0.2343	0.8325	0.893523	Organic
DNA	GH72	30.0891	1.0234	0.3471	0.1838	0.2186	0.4353555	Organic
DNA	GH73	36.2954	0.3217	0.0742	-0.1573	0.6015	0.7342245	Organic
DNA	GH75	2.0444	0.1903	0.2363	0.0453	0.3283	0.5437427	Organic
DNA	GH74	16.7603	3.4442	0.7272	0.659	0.0309	0.1951435	Organic
DNA	GH76	18.3675	0.9606	0.0986	-0.1267	0.5444	0.6880822	Organic
DNA	GH78	173.1756	17.0544	0.4474	0.3092	0.1463	0.3756058	Organic
DNA	GH79	33.6982	0.7621	0.0287	-0.2141	0.7482	0.8430287	Organic
DNA	GH8	19.2144	3.0941	0.6669	0.5837	0.0473	0.2266657	Organic
DNA	GH80	0	0	#VALUE!	#VALUE!	#VALUE!	NA	Organic
DNA	GH81	5.1377	0.0111	0.0001	-0.2499	0.9843	0.9882801	Organic

DNA	GH84	4.79	0.6732	0.6816	0.602	0.043	0.220809	Organic
DNA	GH85	0.5304	0.0799	0.5601	0.4501	0.087	0.2823084	Organic
DNA	GH86	1.1757	-0.0899	0.1416	-0.0731	0.4623	0.6359363	Organic
DNA	GH87	78.9746	6.8423	0.3283	0.1603	0.2346	0.447392	Organic
DNA	GH88	23.1746	1.1116	0.3651	0.2064	0.204	0.4235537	Organic
DNA	GH89	9.9199	1.3029	0.2316	0.0396	0.3338	0.546006	Organic
DNA	GH91	0.0458	-0.0113	0.0951	-0.1312	0.5522	0.6944126	Organic
DNA	GH90	0.0009	0.0008	0.1439	-0.0701	0.4583	0.6339946	Organic
DNA	GH9	60.9971	9.3568	0.6972	0.6216	0.0386	0.2044209	Organic
DNA	GH92	121.0279	12.3975	0.1828	-0.0214	0.3977	0.6075103	Organic
DNA	GH93	18.7765	0.7078	0.0395	-0.2007	0.7059	0.8076638	Organic
DNA	GH94	39.1359	10.5451	0.731	0.6637	0.03	0.1951435	Organic
DNA	GH95	57.25	7.3708	0.5543	0.4429	0.0896	0.2823084	Organic
DNA	GH96	0.1511	0.0547	0.7034	0.6293	0.0369	0.1998862	Organic
DNA	GH97	30.1161	2.0285	0.6701	0.5877	0.0464	0.2266657	Organic
DNA	GH98	0.1755	-0.0492	0.2351	0.0438	0.3297	0.5437427	Organic
DNA	GH99	9.8752	-0.3379	0.0765	-0.1544	0.5958	0.7307917	Organic
DNA	GT1	150.285	-16.811	0.451	0.3137	0.1441	0.3756058	Organic
DNA	GT10	3.0892	0.6469	0.5587	0.4484	0.0876	0.2823084	Organic
DNA	GT12	0.0472	0.0139	0.4367	0.2959	0.153	0.377849	Organic
DNA	GT14	2.1246	0.3251	0.4774	0.3467	0.1285	0.3706182	Organic
DNA	GT11	5.7255	1.2626	0.8208	0.7761	0.0128	0.1645202	Organic
DNA	GT13	0.2735	0.0656	0.1801	-0.0249	0.4017	0.6098779	Organic
DNA	GT15	2.516	-0.28	0.338	0.1725	0.2262	0.4409912	Organic
DNA	GT16	0.0801	0.0218	0.5791	0.4739	0.0789	0.2768264	Organic
DNA	GT17	1.2195	0.0777	0.1555	-0.0556	0.4391	0.6339946	Organic
DNA	GT18	0.0298	0.0024	0.1517	-0.0603	0.4453	0.6339946	Organic
DNA	GT2	1761.7613	68.0457	0.4181	0.2727	0.1652	0.3881401	Organic
DNA	GT19	83.6411	5.4224	0.7868	0.7335	0.0184	0.1835037	Organic
DNA	GT20	146.1397	-3.0681	0.1848	-0.019	0.3949	0.6075103	Organic
DNA	GT21	93.1775	11.1665	0.5871	0.4838	0.0756	0.2768264	Organic
DNA	GT22	5.2116	0.056	0.0521	-0.1849	0.6636	0.7868076	Organic
DNA	GT23	0.3811	0.115	0.3739	0.2174	0.1971	0.4235537	Organic
DNA	GT24	3.5024	0.1815	0.063	-0.1713	0.6315	0.7559428	Organic
DNA	GT25	7.2869	0.7715	0.4795	0.3493	0.1274	0.3706182	Organic
DNA	GT26	148.5105	9.223	0.8319	0.7899	0.0113	0.1645202	Organic
DNA	GT27	15.1224	1.2718	0.4758	0.3448	0.1294	0.3706182	Organic
DNA	GT28	177.4443	11.5062	0.8405	0.8007	0.0101	0.1645202	Organic
DNA	GT29	3.4324	0.0527	0.005	-0.2438	0.8943	0.9389232	Organic
DNA	GT30	121.5074	8.2223	0.8055	0.7569	0.0152	0.1749816	Organic
DNA	GT3	3.9902	0.7265	0.5404	0.4255	0.0959	0.2959452	Organic
DNA	GT33	1.2253	-0.0675	0.3535	0.1919	0.2132	0.4351843	Organic
DNA	GT34	1.1675	-0.1793	0.4008	0.251	0.1773	0.4012513	Organic
DNA	GT32	11.1421	-1.3948	0.8183	0.7729	0.0132	0.1645202	Organic
DNA	GT31	2.1893	0.3466	0.196	-0.005	0.3793	0.5940331	Organic
DNA	GT35	367.4633	36.983	0.889	0.8613	0.0048	0.1645202	Organic
DNA	GT37	0.0155	-0.0105	0.4514	0.3142	0.1439	0.3756058	Organic
DNA	GT39	21.5812	-0.8445	0.1	-0.125	0.5414	0.6880822	Organic
DNA	GT4	1706.1942	17.1305	0.0232	-0.221	0.7734	0.855947	Organic
DNA	GT41	451.5852	73.5836	0.8353	0.7942	0.0108	0.1645202	Organic
DNA	GT40	0.0022	0.0019	0.2941	0.1177	0.2662	0.4874415	Organic
DNA	GT42	0.0544	0.0057	0.1909	-0.0114	0.3863	0.6012158	Organic
DNA	GT43	0.1274	0.0459	0.4048	0.256	0.1744	0.4012513	Organic
DNA	GT47	80.6211	8.0671	0.0911	-0.1362	0.5611	0.7020476	Organic
DNA	GT45	0.1867	0.0291	0.3685	0.2106	0.2013	0.4235537	Organic

DNA	GT44	0.0123	-0.0037	0.2872	0.109	0.2731	0.4930265	Organic
DNA	GT48	4.9851	0.2093	0.0387	-0.2016	0.7088	0.8076638	Organic
DNA	GT49	0.2847	0.0377	0.1196	-0.1005	0.5019	0.6614357	Organic
DNA	GT5	106.4158	3.3064	0.2065	0.0081	0.3653	0.5830371	Organic
DNA	GT50	1.0262	0.0523	0.0485	-0.1893	0.6749	0.7947258	Organic
DNA	GT53	25.4939	-5.7148	0.6366	0.5457	0.0572	0.2449114	Organic
DNA	GT52	0	0	#VALUE!	#VALUE!	#VALUE!	NA	Organic
DNA	GT51	518.0831	-19.7896	0.1396	-0.0755	0.4656	0.6370167	Organic
DNA	GT54	0.0816	0.0148	0.0778	-0.1527	0.5923	0.7307917	Organic
DNA	GT57	1.9599	-0.0163	0.0009	-0.2489	0.9554	0.967031	Organic
DNA	GT56	0.5203	0.1302	0.68	0.5999	0.0435	0.220809	Organic
DNA	GT55	2.6159	-0.3499	0.1556	-0.0556	0.4391	0.6339946	Organic
DNA	GT58	0.7939	-0.0042	0.0005	-0.2493	0.9652	0.9730355	Organic
DNA	GT6	0.3853	0.0709	0.4663	0.3329	0.1349	0.3756058	Organic
DNA	GT59	0.5119	-0.0381	0.077	-0.1538	0.5945	0.7307917	Organic
DNA	GT60	3.4349	0.7587	0.3826	0.2282	0.1905	0.4235537	Organic
DNA	GT61	0.5101	0.1206	0.358	0.1975	0.2096	0.4313833	Organic
DNA	GT62	2.3868	-0.0922	0.1016	-0.1231	0.5382	0.6880822	Organic
DNA	GT64	0.751	0.0198	0.0584	-0.177	0.6446	0.7679197	Organic
DNA	GT65	0.0811	0.0194	0.196	-0.005	0.3793	0.5940331	Organic
DNA	GT66	16.8699	0.9028	0.3491	0.1864	0.2169	0.4353555	Organic
DNA	GT69	1.138	-0.044	0.0151	-0.2311	0.8165	0.8897523	Organic
DNA	GT68	0.0598	0.0026	0.0063	-0.2421	0.8809	0.9301575	Organic
DNA	GT7	6.1602	0.9147	0.7221	0.6527	0.0322	0.1951435	Organic
DNA	GT72	0.0003	-0.0003	0.2408	0.051	0.323	0.5397305	Organic
DNA	GT73	0.071	0.0245	0.3199	0.1499	0.2421	0.453167	Organic
DNA	GT71	1.6293	0.1664	0.3448	0.181	0.2204	0.4355951	Organic
DNA	GT70	0.7452	0.0201	0.1216	-0.098	0.4982	0.6614357	Organic
DNA	GT74	0.8293	0.0266	0.0195	-0.2256	0.7919	0.8725381	Organic
DNA	GT75	0.956	0.1527	0.4614	0.3268	0.1378	0.3756058	Organic
DNA	GT76	1.3751	-0.8243	0.4479	0.3099	0.146	0.3756058	Organic
DNA	GT77	0.6069	-0.25	0.3507	0.1883	0.2156	0.4353555	Organic
DNA	GT78	0.0028	-0.0031	0.4542	0.3178	0.1421	0.3756058	Organic
DNA	GT8	10.5137	0.2187	0.0995	-0.1256	0.5426	0.6880822	Organic
DNA	GT80	6.3954	0.9091	0.7957	0.7446	0.0169	0.1749816	Organic
DNA	GT82	0.3053	0.0061	0.0047	-0.2441	0.8974	0.9389232	Organic
DNA	GT84	319.0374	61.8093	0.7966	0.7458	0.0167	0.1749816	Organic
DNA	GT81	23.6857	0.0092	0.00E+00	-0.25	0.9949	0.9948745	Organic
DNA	GT83	177.9561	1.9276	0.0246	-0.2192	0.7665	0.852035	Organic
DNA	GT85	3.5991	-0.9074	0.5789	0.4737	0.0789	0.2768264	Organic
DNA	GT87	20.2064	0.5668	0.0466	-0.1918	0.6812	0.7947258	Organic
DNA	GT88	0.0469	0.0016	0.0022	-0.2472	0.9292	0.9522022	Organic
DNA	GT89	5.8171	-0.8838	0.5559	0.4448	0.0889	0.2823084	Organic
DNA	GT90	3.3542	0.3508	0.1574	-0.0532	0.4361	0.6339946	Organic
DNA	GT9	154.7911	7.6476	0.3673	0.2091	0.2022	0.4235537	Organic
DNA	GT91	0.0217	-0.0061	0.3011	0.1264	0.2595	0.4787068	Organic
DNA	GT93	0.3645	-0.0125	0.1507	-0.0617	0.447	0.6339946	Organic
DNA	GT94	1.0193	0.2339	0.7359	0.6699	0.0289	0.1951435	Organic
DNA	GT92	0.2101	0.0163	0.07	-0.1625	0.6125	0.737332	Organic
DNA	GT95	6.3578	0.2642	0.1044	-0.1195	0.5322	0.6880822	Organic
DNA	PL1	14.0854	1.9495	0.7438	0.6798	0.0271	0.1951435	Organic
DNA	PL10	5.2376	1.2204	0.4544	0.318	0.142	0.3756058	Organic
DNA	PL11	2.8656	1.0816	0.5807	0.4759	0.0782	0.2768264	Organic
DNA	PL12	22.902	3.9103	0.8485	0.8106	0.0091	0.1645202	Organic
DNA	PL13	0.0005	-0.0006	0.2408	0.051	0.323	0.5397305	Organic

DNA	PL14	0.8538	-0.1658	0.3111	0.1389	0.2501	0.4646825	Organic
DNA	PL15	0.1739	0.0185	0.1526	-0.0593	0.4439	0.6339946	Organic
DNA	PL17	0.9434	0.04	0.0373	-0.2033	0.7137	0.8078169	Organic
DNA	PL18	0	0	#VALUE!	#VALUE!	#VALUE!	NA	Organic
DNA	PL16	0.5082	0.0767	0.7091	0.6364	0.0354	0.1997507	Organic
DNA	PL2	0.3036	0.1178	0.7066	0.6332	0.0361	0.1997507	Organic
DNA	PL20	0.0658	0.0034	0.027	-0.2163	0.7559	0.8478653	Organic
DNA	PL21	0.5253	0.1159	0.2639	0.0799	0.2972	0.5211764	Organic
DNA	PL22	12.3762	2.3825	0.8005	0.7506	0.016	0.1749816	Organic
DNA	PL23	0.02	0.0027	0.4764	0.3455	0.1291	0.3706182	Organic
DNA	PL3	0.0966	0.0131	0.1035	-0.1206	0.5341	0.6880822	Organic
DNA	PL4	26.4684	7.1647	0.366	0.2074	0.2033	0.4235537	Organic
DNA	PL5	4.576	0.8393	0.1441	-0.0699	0.458	0.6339946	Organic
DNA	PL6	1.1647	0.352	0.8588	0.8235	0.0079	0.1645202	Organic
DNA	PL7	3.6063	0.3856	0.1964	-0.0046	0.3788	0.5940331	Organic
DNA	PL8	3.0069	0.6768	0.5623	0.4529	0.086	0.2823084	Organic
DNA	PL9	28.8683	4.0812	0.6317	0.5396	0.0588	0.2449114	Organic
Water_content	AA1	179.0321	9.5073	0.3684	0.2106	0.2013	0.3387206	Organic
Water_content	AA11	0.5025	0.0009	0.00E+00	-0.25	0.9928	0.9999084	Organic
Water_content	AA10	7.5616	1.4082	0.8253	0.7816	0.0122	0.084321	Organic
Water_content	AA2	1.8202	0.3483	0.4904	0.3631	0.1213	0.2495337	Organic
Water_content	AA3	383.3947	-46.6818	0.804	0.7549	0.0155	0.0896438	Organic
Water_content	AA4	20.7999	3.7409	0.2498	0.0623	0.3127	0.4590333	Organic
Water_content	AA5	32.5624	-10.1471	0.6993	0.6241	0.038	0.1376941	Organic
Water_content	AA8	0.8225	0.071	0.135	-0.0812	0.4736	0.6016863	Organic
Water_content	AA7	52.2765	-6.203	0.4793	0.3491	0.1275	0.2580461	Organic
Water_content	AA6	24.3337	-4.3831	0.6956	0.6195	0.039	0.1376941	Organic
Water_content	AA9	1.8306	0.1908	0.1687	-0.0391	0.4185	0.5542815	Organic
Water_content	CE1	208.9789	-0.1791	0.0006	-0.2492	0.9631	0.9788708	Organic
Water_content	CE11	155.7435	9.5978	0.4437	0.3047	0.1486	0.2803081	Organic
Water_content	CE12	20.9813	4.9902	0.9187	0.8984	0.0025	0.0470102	Organic
Water_content	CE13	0.0653	0.0372	0.5082	0.3853	0.1118	0.2359327	Organic
Water_content	CE14	227.4441	13.8007	0.5042	0.3803	0.1139	0.2363485	Organic
Water_content	CE15	52.1006	11.2241	0.8703	0.8379	0.0066	0.071499	Organic
Water_content	CE2	3.239	0.8747	0.8272	0.784	0.0119	0.084321	Organic
Water_content	CE3	15.1515	-1.6894	0.3141	0.1426	0.2473	0.3999229	Organic
Water_content	CE4	294.1051	-53.9174	0.7896	0.737	0.0179	0.0912754	Organic
Water_content	CE16	1.0735	0.0494	0.0635	-0.1706	0.63	0.7196016	Organic
Water_content	CE5	14.9063	-3.478	0.4154	0.2693	0.1671	0.3014883	Organic
Water_content	CE7	12.9532	2.3954	0.9249	0.9061	0.0022	0.0470102	Organic
Water_content	CE6	52.0839	8.7713	0.6396	0.5495	0.0561	0.1668075	Organic
Water_content	CE8	24.9683	4.4473	0.9362	0.9203	0.0016	0.0470102	Organic
Water_content	CE9	90.7663	2.6216	0.3803	0.2254	0.1922	0.3301281	Organic
Water_content	GH100	1.1884	0.6145	0.4643	0.3304	0.1361	0.2705319	Organic
Water_content	GH10	59.1017	7.1996	0.8327	0.7909	0.0111	0.084321	Organic
Water_content	GH102	34.7782	-2.3544	0.6948	0.6185	0.0393	0.1376941	Organic
Water_content	GH101	0.4319	-0.0607	0.5564	0.4455	0.0886	0.2102034	Organic
Water_content	GH103	87.9513	-18.0699	0.9467	0.9333	0.0011	0.0386357	Organic
Water_content	GH104	4.2879	-0.3005	0.1777	-0.0279	0.4052	0.5425704	Organic
Water_content	GH105	39.1132	9.1449	0.7026	0.6282	0.0372	0.1376941	Organic
Water_content	GH106	64.736	15.0903	0.952	0.94	0.0009	0.0363917	Organic
Water_content	GH107	0.05	-0.0085	0.113	-0.1088	0.5148	0.6269647	Organic
Water_content	GH108	6.1612	0.4135	0.2695	0.0869	0.2913	0.4476729	Organic
Water_content	GH109	20.7365	2.2818	0.8175	0.7719	0.0133	0.0851366	Organic
Water_content	GH11	18.0145	3.7526	0.7948	0.7436	0.017	0.0912754	Organic

Water_content	GH110	1.6372	0.3069	0.3922	0.2403	0.1834	0.3215782	Organic
Water_content	GH111	0.0241	0.0031	0.1136	-0.108	0.5135	0.6269647	Organic
Water_content	GH112	0.1865	0.1332	0.807	0.7588	0.015	0.0896438	Organic
Water_content	GH113	7.4538	0.5104	0.1552	-0.056	0.4397	0.5643378	Organic
Water_content	GH114	8.4827	-2.826	0.5827	0.4784	0.0774	0.1945808	Organic
Water_content	GH115	26.0154	10.1099	0.9594	0.9492	0.0006	0.0363917	Organic
Water_content	GH116	54.6915	8.1278	0.8554	0.8192	0.0083	0.0753438	Organic
Water_content	GH117	3.207	0.4735	0.6904	0.613	0.0405	0.1399672	Organic
Water_content	GH118	0.0019	0.0007	0.0599	-0.1751	0.6402	0.7279463	Organic
Water_content	GH119	1.7272	0.2012	0.189	-0.0138	0.389	0.535114	Organic
Water_content	GH12	10.5185	2.4779	0.7502	0.6878	0.0257	0.1140805	Organic
Water_content	GH120	2.0347	0.3168	0.2514	0.0642	0.3109	0.4590333	Organic
Water_content	GH121	0.74	0.0044	0.0009	-0.2488	0.9542	0.9737633	Organic
Water_content	GH123	0.8792	-0.6669	0.6024	0.503	0.0695	0.1822774	Organic
Water_content	GH124	0.0228	-0.0306	0.6165	0.5206	0.0643	0.1766626	Organic
Water_content	GH125	14.4186	1.9584	0.3323	0.1654	0.2311	0.3785665	Organic
Water_content	GH126	0.0126	-0.0002	0.0002	-0.2498	0.9796	0.9915031	Organic
Water_content	GH127	53.4987	9.0287	0.7058	0.6323	0.0363	0.1376941	Organic
Water_content	GH128	9.2104	0.9077	0.1627	-0.0467	0.4278	0.5560669	Organic
Water_content	GH129	0.4364	0.1093	0.482	0.3525	0.1259	0.2570453	Organic
Water_content	GH13	1477.2569	-23.7236	0.118	-0.1025	0.5049	0.6243062	Organic
Water_content	GH130	95.5733	10.7873	0.5261	0.4077	0.1028	0.227201	Organic
Water_content	GH131	0.252	0.0424	0.2131	0.0164	0.3567	0.5075759	Organic
Water_content	GH132	0.9233	-0.0098	0.0016	-0.248	0.9407	0.9638811	Organic
Water_content	GH133	59.9767	-7.148	0.5051	0.3813	0.1135	0.2363485	Organic
Water_content	GH14	0.6765	0.117	0.3816	0.227	0.1912	0.3301281	Organic
Water_content	GH17	51.2924	-8.6357	0.9647	0.9559	0.0005	0.0363917	Organic
Water_content	GH15	420.1827	4.7244	0.0208	-0.2241	0.7854	0.8365474	Organic
Water_content	GH18	255.9295	24.3328	0.8372	0.7965	0.0105	0.084321	Organic
Water_content	GH19	15.6439	0.6968	0.1857	-0.0179	0.3936	0.5355976	Organic
Water_content	GH2	452.5084	76.5519	0.9176	0.897	0.0026	0.0470102	Organic
Water_content	GH20	103.5535	10.7311	0.6101	0.5126	0.0666	0.178348	Organic
Water_content	GH23	366.3567	-3.6033	0.1045	-0.1194	0.532	0.6368842	Organic
Water_content	GH24	15.7222	1.6545	0.9058	0.8823	0.0034	0.0503325	Organic
Water_content	GH25	18.1223	0.8082	0.3396	0.1745	0.2249	0.3732602	Organic
Water_content	GH26	23.6541	1.8908	0.2265	0.0332	0.34	0.489306	Organic
Water_content	GH27	104.8235	21.4277	0.9163	0.8953	0.0027	0.0470102	Organic
Water_content	GH28	237.447	49.9722	0.9741	0.9676	0.0003	0.0363917	Organic
Water_content	GH29	132.2116	18.6094	0.9106	0.8882	0.0031	0.0481244	Organic
Water_content	GH3	680.4412	90.8602	0.8819	0.8524	0.0055	0.0650731	Organic
Water_content	GH30	59.6043	5.7435	0.4753	0.3442	0.1297	0.2604385	Organic
Water_content	GH31	173.7587	25.6545	0.8801	0.8502	0.0056	0.0650731	Organic
Water_content	GH33	48.5541	1.3108	0.071	-0.1613	0.6098	0.7094584	Organic
Water_content	GH36	117.4663	21.6497	0.7899	0.7374	0.0179	0.0912754	Organic
Water_content	GH32	30.1748	-1.163	0.1172	-0.1034	0.5065	0.6243062	Organic
Water_content	GH37	45.5476	4.284	0.2981	0.1227	0.2624	0.4160959	Organic
Water_content	GH39	110.1752	17.4677	0.8184	0.7729	0.0132	0.0851366	Organic
Water_content	GH38	153.8558	30.3623	0.7894	0.7367	0.018	0.0912754	Organic
Water_content	GH4	141.0485	1.4106	0.011	-0.2362	0.843	0.889442	Organic
Water_content	GH42	52.5554	4.0407	0.7847	0.7309	0.0188	0.0936569	Organic
Water_content	GH43	133.3589	24.6379	0.8553	0.8191	0.0083	0.0753438	Organic
Water_content	GH44	32.7913	6.2595	0.5836	0.4795	0.077	0.1945808	Organic
Water_content	GH45	0.5846	0.1748	0.7192	0.649	0.0329	0.1376941	Organic
Water_content	GH47	14.6845	0.75	0.1666	-0.0418	0.4218	0.5557191	Organic
Water_content	GH46	1.4741	-0.2295	0.3234	0.1542	0.239	0.3888834	Organic

Water_content	GH48	10.4606	1.6535	0.8611	0.8264	0.0076	0.0753438	Organic
Water_content	GH49	0.0919	-0.0055	0.0574	-0.1783	0.6476	0.7329236	Organic
Water_content	GH5	172.0215	9.4298	0.7572	0.6965	0.0242	0.111567	Organic
Water_content	GH50	7.7274	-0.1318	0.0062	-0.2423	0.8824	0.919509	Organic
Water_content	GH51	136.9965	23.6976	0.8829	0.8537	0.0054	0.0650731	Organic
Water_content	GH54	33.242	8.5044	0.5273	0.4091	0.1022	0.227201	Organic
Water_content	GH53	65.4769	9.6887	0.6806	0.6007	0.0433	0.1442253	Organic
Water_content	GH55	145.3713	25.291	0.6247	0.5308	0.0613	0.1715645	Organic
Water_content	GH56	0.0085	0.0083	0.6829	0.6037	0.0426	0.1442253	Organic
Water_content	GH57	148.2848	2.8547	0.0281	-0.2148	0.7507	0.8134057	Organic
Water_content	GH58	0.0299	0.0007	0.0062	-0.2423	0.8826	0.919509	Organic
Water_content	GH59	11.8995	2.3356	0.3776	0.2219	0.1943	0.3313914	Organic
Water_content	GH6	21.9717	3.4004	0.6968	0.6211	0.0387	0.1376941	Organic
Water_content	GH62	3.402	0.9038	0.5509	0.4387	0.0911	0.2139882	Organic
Water_content	GH63	18.035	3.2927	0.9144	0.893	0.0028	0.0470102	Organic
Water_content	GH64	23.3696	3.7011	0.8535	0.8169	0.0085	0.0753438	Organic
Water_content	GH65	99.333	0.0008	0.00E+00	-0.25	0.9999	0.9999084	Organic
Water_content	GH66	1.6072	0.3912	0.4528	0.316	0.143	0.2760452	Organic
Water_content	GH68	0.3029	-0.0346	0.1985	-0.0019	0.376	0.5200764	Organic
Water_content	GH7	0.8798	0.1113	0.2492	0.0615	0.3134	0.4590333	Organic
Water_content	GH70	0.0313	0.0082	0.2607	0.0759	0.3007	0.45652	Organic
Water_content	GH71	4.8837	0.6226	0.15	-0.0625	0.4481	0.572248	Organic
Water_content	GH72	30.0891	1.4127	0.6613	0.5767	0.0491	0.1566362	Organic
Water_content	GH73	36.2954	-0.0027	0.00E+00	-0.25	0.9966	0.9999084	Organic
Water_content	GH75	2.0444	0.1877	0.2299	0.0373	0.336	0.4875968	Organic
Water_content	GH74	16.7603	2.1187	0.2752	0.094	0.2853	0.444928	Organic
Water_content	GH76	18.3675	1.6861	0.3039	0.1299	0.2568	0.4099635	Organic
Water_content	GH78	173.1756	23.9021	0.8788	0.8485	0.0057	0.0650731	Organic
Water_content	GH79	33.6982	2.3786	0.2798	0.0998	0.2806	0.4421443	Organic
Water_content	GH8	19.2144	3.7277	0.968	0.96	0.0004	0.0363917	Organic
Water_content	GH80	0	0	#VALUE!	#VALUE!	#VALUE!	NA	Organic
Water_content	GH81	5.1377	0.1479	0.0195	-0.2256	0.7918	0.8389586	Organic
Water_content	GH84	4.79	0.7455	0.8357	0.7946	0.0107	0.084321	Organic
Water_content	GH85	0.5304	0.0852	0.637	0.5463	0.057	0.1668075	Organic
Water_content	GH86	1.1757	-0.1534	0.4116	0.2645	0.1697	0.3039726	Organic
Water_content	GH87	78.9746	9.5317	0.637	0.5463	0.057	0.1668075	Organic
Water_content	GH88	23.1746	0.5988	0.1059	-0.1176	0.529	0.6368842	Organic
Water_content	GH89	9.9199	2.1113	0.6083	0.5103	0.0673	0.178348	Organic
Water_content	GH91	0.0458	-0.0147	0.1621	-0.0474	0.4288	0.5560669	Organic
Water_content	GH90	0.0009	0.0015	0.4623	0.3279	0.1372	0.2705319	Organic
Water_content	GH9	60.9971	10.1806	0.8254	0.7818	0.0122	0.084321	Organic
Water_content	GH92	121.0279	23.2088	0.6408	0.551	0.0557	0.1668075	Organic
Water_content	GH93	18.7765	0.7695	0.0467	-0.1917	0.681	0.756996	Organic
Water_content	GH94	39.1359	9.3958	0.5803	0.4754	0.0784	0.195119	Organic
Water_content	GH95	57.25	9.676	0.9553	0.9441	0.0008	0.0363917	Organic
Water_content	GH96	0.1511	0.0491	0.5667	0.4583	0.0841	0.2033806	Organic
Water_content	GH97	30.1161	2.1721	0.7684	0.7105	0.0219	0.1029106	Organic
Water_content	GH98	0.1755	-0.075	0.5456	0.432	0.0935	0.2176274	Organic
Water_content	GH99	9.8752	-0.3256	0.071	-0.1612	0.6097	0.7094584	Organic
Water_content	GT1	150.285	-7.9544	0.101	-0.1238	0.5394	0.6426398	Organic
Water_content	GT10	3.0892	0.7644	0.7802	0.7252	0.0196	0.0958987	Organic
Water_content	GT12	0.0472	0.0108	0.2637	0.0796	0.2974	0.4543678	Organic
Water_content	GT14	2.1246	0.4314	0.8406	0.8008	0.0101	0.084321	Organic
Water_content	GT11	5.7255	1.1776	0.7141	0.6427	0.0341	0.1376941	Organic
Water_content	GT13	0.2735	0.1254	0.657	0.5712	0.0504	0.1570012	Organic

Water_content	GT15	2.516	-0.1016	0.0445	-0.1944	0.6883	0.76169	Organic
Water_content	GT16	0.0801	0.0261	0.8295	0.7869	0.0116	0.084321	Organic
Water_content	GT17	1.2195	0.1316	0.4461	0.3076	0.1471	0.2803081	Organic
Water_content	GT18	0.0298	0.0012	0.0367	-0.2041	0.7162	0.7856365	Organic
Water_content	GT2	1761.7613	27.6324	0.069	-0.1638	0.6152	0.7094584	Organic
Water_content	GT19	83.6411	5.3033	0.7526	0.6908	0.0252	0.1138962	Organic
Water_content	GT20	146.1397	-5.1551	0.5218	0.4022	0.1049	0.227201	Organic
Water_content	GT21	93.1775	12.3376	0.7167	0.6458	0.0335	0.1376941	Organic
Water_content	GT22	5.2116	0.1175	0.2291	0.0364	0.3368	0.4875968	Organic
Water_content	GT23	0.3811	0.1685	0.8039	0.7549	0.0155	0.0896438	Organic
Water_content	GT24	3.5024	0.4688	0.42	0.275	0.164	0.2980692	Organic
Water_content	GT25	7.2869	0.8815	0.626	0.5325	0.0609	0.1715645	Organic
Water_content	GT26	148.5105	9.5677	0.8953	0.8691	0.0043	0.0590327	Organic
Water_content	GT27	15.1224	1.7036	0.8538	0.8172	0.0084	0.0753438	Organic
Water_content	GT28	177.4443	7.9755	0.4038	0.2548	0.1751	0.311409	Organic
Water_content	GT29	3.4324	-0.1172	0.0246	-0.2192	0.7665	0.8262407	Organic
Water_content	GT30	121.5074	7.4359	0.6588	0.5735	0.0499	0.1570012	Organic
Water_content	GT3	3.9902	0.8333	0.711	0.6388	0.0349	0.1376941	Organic
Water_content	GT33	1.2253	-0.0403	0.1264	-0.092	0.4892	0.6151892	Organic
Water_content	GT34	1.1675	-0.0634	0.05	-0.1874	0.67	0.7481557	Organic
Water_content	GT32	11.1421	-1.2715	0.68	0.6	0.0434	0.1442253	Organic
Water_content	GT31	2.1893	0.6402	0.6688	0.586	0.0468	0.1512609	Organic
Water_content	GT35	367.4633	31.2621	0.6353	0.5441	0.0576	0.1668075	Organic
Water_content	GT37	0.0155	-0.0129	0.6713	0.5891	0.046	0.1507694	Organic
Water_content	GT39	21.5812	-0.5276	0.039	-0.2012	0.7075	0.7794915	Organic
Water_content	GT4	1706.1942	-29.5315	0.0689	-0.1639	0.6154	0.7094584	Organic
Water_content	GT41	451.5852	73.0063	0.8223	0.7779	0.0126	0.084954	Organic
Water_content	GT40	0.0022	0.0021	0.3741	0.2177	0.1969	0.3335688	Organic
Water_content	GT42	0.0544	0.0099	0.5638	0.4548	0.0853	0.2043448	Organic
Water_content	GT43	0.1274	0.0616	0.7292	0.6615	0.0304	0.1307227	Organic
Water_content	GT47	80.6211	18.0642	0.4566	0.3208	0.1407	0.2736277	Organic
Water_content	GT45	0.1867	0.0113	0.0554	-0.1808	0.6535	0.7363029	Organic
Water_content	GT44	0.0123	-0.0046	0.4364	0.2955	0.1532	0.2805307	Organic
Water_content	GT48	4.9851	0.615	0.3341	0.1676	0.2296	0.3785518	Organic
Water_content	GT49	0.2847	0.0792	0.5295	0.4119	0.1011	0.227201	Organic
Water_content	GT5	106.4158	1.2468	0.0294	-0.2133	0.7455	0.8134057	Organic
Water_content	GT50	1.0262	0.1569	0.4373	0.2966	0.1527	0.2805307	Organic
Water_content	GT53	25.4939	-3.999	0.3117	0.1397	0.2495	0.4008715	Organic
Water_content	GT52	0	0	#VALUE!	#VALUE!	#VALUE!	NA	Organic
Water_content	GT51	518.0831	-35.0744	0.4386	0.2982	0.1519	0.2805307	Organic
Water_content	GT54	0.0816	0.036	0.4611	0.3264	0.138	0.2705319	Organic
Water_content	GT57	1.9599	0.2285	0.1745	-0.0319	0.4099	0.5458015	Organic
Water_content	GT56	0.5203	0.1324	0.7025	0.6282	0.0372	0.1376941	Organic
Water_content	GT55	2.6159	-0.0501	0.0032	-0.246	0.9154	0.945788	Organic
Water_content	GT58	0.7939	0.0779	0.1862	-0.0172	0.3929	0.5355976	Organic
Water_content	GT6	0.3853	0.0815	0.6157	0.5196	0.0646	0.1766626	Organic
Water_content	GT59	0.5119	0.023	0.028	-0.215	0.7513	0.8134057	Organic
Water_content	GT60	3.4349	1.0797	0.7747	0.7184	0.0207	0.0990077	Organic
Water_content	GT61	0.5101	0.1744	0.7482	0.6852	0.0261	0.1140805	Organic
Water_content	GT62	2.3868	-0.0996	0.1186	-0.1018	0.5039	0.6243062	Organic
Water_content	GT64	0.751	-0.007	0.0073	-0.2408	0.872	0.9161143	Organic
Water_content	GT65	0.0811	0.0353	0.6504	0.5631	0.0525	0.1615138	Organic
Water_content	GT66	16.8699	0.6943	0.2064	0.0081	0.3654	0.5110839	Organic
Water_content	GT69	1.138	0.0913	0.0652	-0.1685	0.6254	0.717623	Organic
Water_content	GT68	0.0598	0.0152	0.2114	0.0143	0.3589	0.5077215	Organic

Water_content	GT7	6.1602	0.903	0.7038	0.6297	0.0368	0.1376941	Organic
Water_content	GT72	0.0003	-0.0002	0.0702	-0.1622	0.6118	0.7094584	Organic
Water_content	GT73	0.071	0.0226	0.2728	0.091	0.2878	0.4450752	Organic
Water_content	GT71	1.6293	0.2376	0.7031	0.6289	0.037	0.1376941	Organic
Water_content	GT70	0.7452	0.0168	0.0848	-0.144	0.5755	0.6824165	Organic
Water_content	GT74	0.8293	0.012	0.004	-0.245	0.9055	0.9394986	Organic
Water_content	GT75	0.956	0.0895	0.1584	-0.0519	0.4345	0.5605258	Organic
Water_content	GT76	1.3751	-0.9606	0.6083	0.5104	0.0673	0.178348	Organic
Water_content	GT77	0.6069	-0.3097	0.5384	0.423	0.0969	0.2233792	Organic
Water_content	GT78	0.0028	-0.0033	0.5218	0.4023	0.1049	0.227201	Organic
Water_content	GT8	10.5137	0.4356	0.3947	0.2433	0.1816	0.3207663	Organic
Water_content	GT80	6.3954	0.7866	0.5957	0.4946	0.0722	0.1872068	Organic
Water_content	GT82	0.3053	-0.0301	0.1123	-0.1096	0.5162	0.6269647	Organic
Water_content	GT84	319.0374	53.3294	0.593	0.4913	0.0732	0.1879513	Organic
Water_content	GT81	23.6857	-0.8699	0.1049	-0.1189	0.5312	0.6368842	Organic
Water_content	GT83	177.9561	5.603	0.2082	0.0102	0.3631	0.5108092	Organic
Water_content	GT85	3.5991	-0.5648	0.2243	0.0304	0.3427	0.4904455	Organic
Water_content	GT87	20.2064	1.1182	0.1813	-0.0233	0.3998	0.5410848	Organic
Water_content	GT88	0.0469	0.0174	0.2569	0.0711	0.3048	0.4572422	Organic
Water_content	GT89	5.8171	-0.6212	0.2746	0.0933	0.2859	0.444928	Organic
Water_content	GT90	3.3542	0.6321	0.511	0.3888	0.1104	0.2348561	Organic
Water_content	GT9	154.7911	4.5675	0.131	-0.0862	0.4808	0.6076562	Organic
Water_content	GT91	0.0217	-0.0039	0.1219	-0.0976	0.4976	0.6226078	Organic
Water_content	GT93	0.3645	-0.0073	0.0518	-0.1852	0.6644	0.7452217	Organic
Water_content	GT94	1.0193	0.1992	0.5338	0.4172	0.0991	0.2263852	Organic
Water_content	GT92	0.2101	0.0313	0.2575	0.0719	0.3041	0.4572422	Organic
Water_content	GT95	6.3578	-0.1262	0.0238	-0.2202	0.7704	0.8268319	Organic
Water_content	PL1	14.0854	1.4048	0.3863	0.2328	0.1878	0.3269886	Organic
Water_content	PL10	5.2376	1.6294	0.81	0.7625	0.0145	0.0896438	Organic
Water_content	PL11	2.8656	0.8282	0.3405	0.1756	0.2241	0.3732602	Organic
Water_content	PL12	22.902	3.037	0.5118	0.3897	0.11	0.2348561	Organic
Water_content	PL13	0.0005	-0.0003	0.0702	-0.1622	0.6118	0.7094584	Organic
Water_content	PL14	0.8538	-0.2152	0.5238	0.4048	0.1039	0.227201	Organic
Water_content	PL15	0.1739	-0.0025	0.0027	-0.2466	0.922	0.948629	Organic
Water_content	PL17	0.9434	-0.0297	0.0206	-0.2242	0.7862	0.8365474	Organic
Water_content	PL18	0	0	#VALUE!	#VALUE!	#VALUE!	NA	Organic
Water_content	PL16	0.5082	0.0608	0.4455	0.3069	0.1475	0.2803081	Organic
Water_content	PL2	0.3036	0.1249	0.7949	0.7436	0.017	0.0912754	Organic
Water_content	PL20	0.0658	0.0088	0.1776	-0.028	0.4053	0.5425704	Organic
Water_content	PL21	0.5253	0.0916	0.1647	-0.0441	0.4247	0.5560669	Organic
Water_content	PL22	12.3762	2.5543	0.9201	0.9001	0.0025	0.0470102	Organic
Water_content	PL23	0.02	0.002	0.2543	0.0679	0.3077	0.4587586	Organic
Water_content	PL3	0.0966	0.0181	0.199	-0.0012	0.3752	0.5200764	Organic
Water_content	PL4	26.4684	10.5723	0.7968	0.746	0.0167	0.0912754	Organic
Water_content	PL5	4.576	1.6786	0.5763	0.4704	0.08	0.1973137	Organic
Water_content	PL6	1.1647	0.301	0.628	0.535	0.0601	0.1715645	Organic
Water_content	PL7	3.6063	0.5785	0.4419	0.3024	0.1497	0.2803089	Organic
Water_content	PL8	3.0069	0.8671	0.9231	0.9038	0.0023	0.0470102	Organic
Water_content	PL9	28.8683	3.8902	0.574	0.4674	0.081	0.1977726	Organic
Bulk_density	AA1	179.0321	-8.8429	0.3188	0.1484	0.2431	0.5272595	Organic
Bulk_density	AA11	0.5025	0.1112	0.3563	0.1954	0.211	0.4979652	Organic
Bulk_density	AA10	7.5616	-0.8052	0.2698	0.0873	0.2909	0.5841348	Organic
Bulk_density	AA2	1.8202	-0.163	0.1075	-0.1156	0.5259	0.7525096	Organic
Bulk_density	AA3	383.3947	40.9753	0.6194	0.5243	0.0632	0.3805859	Organic
Bulk_density	AA4	20.7999	-3.7165	0.2466	0.0582	0.3164	0.6154265	Organic

Bulk_density	AA5	32.5624	5.1981	0.1835	-0.0206	0.3967	0.6696148	Organic
Bulk_density	AA8	0.8225	-0.0904	0.219	0.0238	0.3493	0.6291049	Organic
Bulk_density	AA7	52.2765	6.2145	0.481	0.3513	0.1265	0.4510213	Organic
Bulk_density	AA6	24.3337	4.5452	0.748	0.685	0.0262	0.3805859	Organic
Bulk_density	AA9	1.8306	0.0125	0.0007	-0.2491	0.9596	0.9674042	Organic
Bulk_density	CE1	208.9789	-3.1139	0.1825	-0.0219	0.3982	0.6696148	Organic
Bulk_density	CE11	155.7435	-12.5548	0.7593	0.6991	0.0238	0.3805859	Organic
Bulk_density	CE12	20.9813	-3.0743	0.3487	0.1859	0.2172	0.4979652	Organic
Bulk_density	CE13	0.0653	-0.0221	0.1785	-0.0269	0.404	0.6696148	Organic
Bulk_density	CE14	227.4441	-14.7536	0.5762	0.4703	0.0801	0.3966602	Organic
Bulk_density	CE15	52.1006	-8.7795	0.5325	0.4156	0.0997	0.4510213	Organic
Bulk_density	CE2	3.239	-0.7929	0.6797	0.5997	0.0435	0.3805859	Organic
Bulk_density	CE3	15.1515	1.8939	0.3948	0.2435	0.1816	0.480953	Organic
Bulk_density	CE4	294.1051	31.6383	0.2719	0.0899	0.2887	0.5841348	Organic
Bulk_density	CE16	1.0735	0.0536	0.0749	-0.1564	0.5998	0.7749438	Organic
Bulk_density	CE5	14.9063	4.2693	0.6259	0.5324	0.0609	0.3805859	Organic
Bulk_density	CE7	12.9532	-1.9342	0.603	0.5038	0.0693	0.3805859	Organic
Bulk_density	CE6	52.0839	-5.0207	0.2096	0.012	0.3613	0.6425655	Organic
Bulk_density	CE8	24.9683	-3.3474	0.5304	0.413	0.1007	0.4510213	Organic
Bulk_density	CE9	90.7663	-1.9825	0.2175	0.0219	0.3512	0.6291049	Organic
Bulk_density	GH100	1.1884	-0.3185	0.1248	-0.094	0.4922	0.7338563	Organic
Bulk_density	GH10	59.1017	-5.3668	0.4627	0.3284	0.137	0.4510213	Organic
Bulk_density	GH102	34.7782	1.2173	0.1857	-0.0179	0.3936	0.6696148	Organic
Bulk_density	GH101	0.4319	0.0526	0.4188	0.2735	0.1648	0.4510213	Organic
Bulk_density	GH103	87.9513	12.8668	0.48	0.35	0.1271	0.4510213	Organic
Bulk_density	GH104	4.2879	-0.0963	0.0183	-0.2272	0.7986	0.8840328	Organic
Bulk_density	GH105	39.1132	-3.6842	0.114	-0.1075	0.5127	0.7466036	Organic
Bulk_density	GH106	64.736	-9.944	0.4134	0.2668	0.1685	0.4510213	Organic
Bulk_density	GH107	0.05	0.0076	0.0901	-0.1374	0.5633	0.7730188	Organic
Bulk_density	GH108	6.1612	0.0831	0.0109	-0.2364	0.844	0.8981453	Organic
Bulk_density	GH109	20.7365	-1.7577	0.4851	0.3564	0.1242	0.4510213	Organic
Bulk_density	GH11	18.0145	-2.2543	0.2869	0.1086	0.2734	0.5626995	Organic
Bulk_density	GH110	1.6372	-0.0303	0.0038	-0.2452	0.9072	0.9309841	Organic
Bulk_density	GH111	0.0241	0.0026	0.0827	-0.1466	0.5805	0.7730188	Organic
Bulk_density	GH112	0.1865	-0.0927	0.3906	0.2382	0.1846	0.482106	Organic
Bulk_density	GH113	7.4538	-1.0019	0.5979	0.4974	0.0713	0.3805859	Organic
Bulk_density	GH114	8.4827	1.4688	0.1574	-0.0532	0.4361	0.7005231	Organic
Bulk_density	GH115	26.0154	-7.8557	0.5793	0.4741	0.0788	0.3966602	Organic
Bulk_density	GH116	54.6915	-5.1953	0.3495	0.1869	0.2165	0.4979652	Organic
Bulk_density	GH117	3.207	-0.2179	0.1462	-0.0672	0.4544	0.7180524	Organic
Bulk_density	GH118	0.0019	-0.0003	0.0118	-0.2352	0.8375	0.8950552	Organic
Bulk_density	GH119	1.7272	-0.3677	0.6312	0.5389	0.059	0.3805859	Organic
Bulk_density	GH12	10.5185	-1.8487	0.4176	0.272	0.1656	0.4510213	Organic
Bulk_density	GH120	2.0347	-0.0386	0.0037	-0.2453	0.9086	0.9309841	Organic
Bulk_density	GH121	0.74	-0.0096	0.0043	-0.2446	0.9013	0.9309841	Organic
Bulk_density	GH123	0.8792	0.3178	0.1368	-0.079	0.4704	0.7230726	Organic
Bulk_density	GH124	0.0228	0.0165	0.1791	-0.0262	0.4031	0.6696148	Organic
Bulk_density	GH125	14.4186	-0.2671	0.0062	-0.2423	0.8823	0.9269639	Organic
Bulk_density	GH126	0.0126	0.0023	0.0398	-0.2003	0.7048	0.8317004	Organic
Bulk_density	GH127	53.4987	-7.4647	0.4825	0.3531	0.1257	0.4510213	Organic
Bulk_density	GH128	9.2104	-0.0396	0.0003	-0.2496	0.9736	0.9775353	Organic
Bulk_density	GH129	0.4364	-0.1365	0.7518	0.6897	0.0253	0.3805859	Organic
Bulk_density	GH13	1477.2569	27.457	0.1581	-0.0524	0.435	0.7005231	Organic
Bulk_density	GH130	95.5733	-13.9655	0.8818	0.8523	0.0055	0.3805859	Organic
Bulk_density	GH131	0.252	-0.0203	0.0488	-0.189	0.6741	0.80693	Organic

Bulk_density	GH132	0.9233	0.1196	0.2331	0.0413	0.3321	0.6217676	Organic
Bulk_density	GH133	59.9767	2.0942	0.0434	-0.1958	0.6922	0.8207477	Organic
Bulk_density	GH14	0.6765	-0.0449	0.0562	-0.1797	0.651	0.7945733	Organic
Bulk_density	GH17	51.2924	5.9072	0.4514	0.3143	0.1438	0.4510213	Organic
Bulk_density	GH15	420.1827	-4.5031	0.0189	-0.2264	0.7953	0.8840328	Organic
Bulk_density	GH18	255.9295	-14.4571	0.2955	0.1194	0.2649	0.5578596	Organic
Bulk_density	GH19	15.6439	-0.2035	0.0158	-0.2302	0.8122	0.8840328	Organic
Bulk_density	GH2	452.5084	-65.6448	0.6747	0.5934	0.045	0.3805859	Organic
Bulk_density	GH20	103.5535	-4.4252	0.1037	-0.1203	0.5336	0.7591827	Organic
Bulk_density	GH23	366.3567	-2.763	0.0614	-0.1732	0.6358	0.7945733	Organic
Bulk_density	GH24	15.7222	-1.4642	0.7094	0.6368	0.0353	0.3805859	Organic
Bulk_density	GH25	18.1223	-0.401	0.0836	-0.1455	0.5784	0.7730188	Organic
Bulk_density	GH26	23.6541	-2.6281	0.4377	0.2971	0.1524	0.4510213	Organic
Bulk_density	GH27	104.8235	-13.9591	0.3888	0.2361	0.1859	0.482106	Organic
Bulk_density	GH28	237.447	-39.1515	0.5979	0.4974	0.0713	0.3805859	Organic
Bulk_density	GH29	132.2116	-16.4081	0.7079	0.6349	0.0357	0.3805859	Organic
Bulk_density	GH3	680.4412	-59.1662	0.3739	0.2174	0.1971	0.4966114	Organic
Bulk_density	GH30	59.6043	-1.5351	0.034	-0.2076	0.7267	0.8493449	Organic
Bulk_density	GH31	173.7587	-16.5957	0.3683	0.2104	0.2014	0.4966114	Organic
Bulk_density	GH33	48.5541	-3.3222	0.456	0.32	0.141	0.4510213	Organic
Bulk_density	GH36	117.4663	-16.5805	0.4633	0.3292	0.1367	0.4510213	Organic
Bulk_density	GH32	30.1748	0.3945	0.0135	-0.2331	0.8265	0.8904843	Organic
Bulk_density	GH37	45.5476	-2.142	0.0745	-0.1568	0.6007	0.7749438	Organic
Bulk_density	GH39	110.1752	-16.5571	0.7353	0.6691	0.029	0.3805859	Organic
Bulk_density	GH38	153.8558	-16.1765	0.2241	0.0301	0.343	0.6233845	Organic
Bulk_density	GH4	141.0485	0.0606	0.00E+00	-0.25	0.9932	0.993235	Organic
Bulk_density	GH42	52.5554	-3.7036	0.6592	0.574	0.0497	0.3805859	Organic
Bulk_density	GH43	133.3589	-16.3703	0.3776	0.222	0.1943	0.4966114	Organic
Bulk_density	GH44	32.7913	-7.3156	0.7971	0.7464	0.0166	0.3805859	Organic
Bulk_density	GH45	0.5846	-0.1347	0.4272	0.284	0.1592	0.4510213	Organic
Bulk_density	GH47	14.6845	0.2877	0.0245	-0.2194	0.7671	0.8721881	Organic
Bulk_density	GH46	1.4741	0.2258	0.3131	0.1414	0.2483	0.5298826	Organic
Bulk_density	GH48	10.4606	-1.2563	0.4971	0.3714	0.1177	0.4510213	Organic
Bulk_density	GH49	0.0919	0.0067	0.0855	-0.1431	0.5739	0.7730188	Organic
Bulk_density	GH5	172.0215	-9.4967	0.768	0.7099	0.022	0.3805859	Organic
Bulk_density	GH50	7.7274	-0.6001	0.1278	-0.0902	0.4865	0.7298012	Organic
Bulk_density	GH51	136.9965	-20.5445	0.6636	0.5795	0.0484	0.3805859	Organic
Bulk_density	GH54	33.242	-3.5422	0.0915	-0.1357	0.5602	0.7730188	Organic
Bulk_density	GH53	65.4769	-10.5553	0.8078	0.7598	0.0148	0.3805859	Organic
Bulk_density	GH55	145.3713	-11.5002	0.1292	-0.0886	0.4841	0.7298012	Organic
Bulk_density	GH56	0.0085	-0.0053	0.2773	0.0966	0.2831	0.5778704	Organic
Bulk_density	GH57	148.2848	-11.1248	0.4275	0.2843	0.159	0.4510213	Organic
Bulk_density	GH58	0.0299	-0.0014	0.0209	-0.2239	0.7846	0.8837457	Organic
Bulk_density	GH59	11.8995	-0.9233	0.059	-0.1762	0.6428	0.7945733	Organic
Bulk_density	GH6	21.9717	-2.2982	0.3183	0.1479	0.2435	0.5272595	Organic
Bulk_density	GH62	3.402	-0.9992	0.6734	0.5917	0.0454	0.3805859	Organic
Bulk_density	GH63	18.035	-2.3172	0.4528	0.316	0.143	0.4510213	Organic
Bulk_density	GH64	23.3696	-1.9463	0.236	0.0451	0.3286	0.6198176	Organic
Bulk_density	GH65	99.333	2.6518	0.0369	-0.2039	0.7155	0.8403527	Organic
Bulk_density	GH66	1.6072	-0.4351	0.5599	0.4499	0.0871	0.4090723	Organic
Bulk_density	GH68	0.3029	0.0179	0.0533	-0.1834	0.66	0.7998476	Organic
Bulk_density	GH7	0.8798	-0.0549	0.0605	-0.1743	0.6383	0.7945733	Organic
Bulk_density	GH70	0.0313	-0.0098	0.3702	0.2127	0.2	0.4966114	Organic
Bulk_density	GH71	4.8837	0.3891	0.0586	-0.1768	0.6441	0.7945733	Organic
Bulk_density	GH72	30.0891	-0.8491	0.2389	0.0487	0.3252	0.6198176	Organic

Bulk_density	GH73	36.2954	-0.4399	0.1387	-0.0766	0.4672	0.7230726	Organic
Bulk_density	GH75	2.0444	-0.0981	0.0628	-0.1716	0.6321	0.7945733	Organic
Bulk_density	GH74	16.7603	-3.1824	0.6208	0.526	0.0627	0.3805859	Organic
Bulk_density	GH76	18.3675	-0.4016	0.0172	-0.2285	0.8042	0.8840328	Organic
Bulk_density	GH78	173.1756	-14.2495	0.3123	0.1404	0.249	0.5298826	Organic
Bulk_density	GH79	33.6982	-0.2949	0.0043	-0.2446	0.9018	0.9309841	Organic
Bulk_density	GH8	19.2144	-2.5441	0.4509	0.3136	0.1442	0.4510213	Organic
Bulk_density	GH80	0	0	#VALUE!	#VALUE!	#VALUE!	NA	Organic
Bulk_density	GH81	5.1377	-0.1776	0.0282	-0.2148	0.7507	0.8574354	Organic
Bulk_density	GH84	4.79	-0.6504	0.6362	0.5452	0.0573	0.3805859	Organic
Bulk_density	GH85	0.5304	-0.0827	0.5999	0.4999	0.0705	0.3805859	Organic
Bulk_density	GH86	1.1757	0.0548	0.0526	-0.1843	0.6621	0.7998476	Organic
Bulk_density	GH87	78.9746	-5.7079	0.2284	0.0355	0.3377	0.6218842	Organic
Bulk_density	GH88	23.1746	-1.4838	0.6505	0.5631	0.0525	0.3805859	Organic
Bulk_density	GH89	9.9199	-0.7935	0.0859	-0.1426	0.5729	0.7730188	Organic
Bulk_density	GH91	0.0458	0.0045	0.015	-0.2312	0.8172	0.884697	Organic
Bulk_density	GH90	0.0009	-0.001	0.2002	0.0003	0.3736	0.6551346	Organic
Bulk_density	GH9	60.9971	-7.6139	0.4617	0.3271	0.1376	0.4510213	Organic
Bulk_density	GH92	121.0279	-7.928	0.0748	-0.1565	0.6001	0.7749438	Organic
Bulk_density	GH93	18.7765	-0.1774	0.0025	-0.2469	0.9254	0.9404629	Organic
Bulk_density	GH94	39.1359	-8.4077	0.4647	0.3308	0.1359	0.4510213	Organic
Bulk_density	GH95	57.25	-6.3829	0.4157	0.2696	0.1669	0.4510213	Organic
Bulk_density	GH96	0.1511	-0.0545	0.6981	0.6226	0.0384	0.3805859	Organic
Bulk_density	GH97	30.1161	-1.6587	0.4481	0.3101	0.1459	0.4510213	Organic
Bulk_density	GH98	0.1755	0.0336	0.1096	-0.113	0.5215	0.7525096	Organic
Bulk_density	GH99	9.8752	0.1531	0.0157	-0.2304	0.813	0.8840328	Organic
Bulk_density	GT1	150.285	16.3444	0.4263	0.2829	0.1598	0.4510213	Organic
Bulk_density	GT10	3.0892	-0.6479	0.5606	0.4507	0.0868	0.4090723	Organic
Bulk_density	GT12	0.0472	-0.0088	0.1771	-0.0287	0.4061	0.6696148	Organic
Bulk_density	GT14	2.1246	-0.255	0.2938	0.1172	0.2666	0.5578596	Organic
Bulk_density	GT11	5.7255	-1.1478	0.6785	0.5981	0.0439	0.3805859	Organic
Bulk_density	GT13	0.2735	-0.0526	0.1157	-0.1054	0.5095	0.7463039	Organic
Bulk_density	GT15	2.516	0.283	0.3453	0.1817	0.22	0.4979652	Organic
Bulk_density	GT16	0.0801	-0.0221	0.5968	0.496	0.0717	0.3805859	Organic
Bulk_density	GT17	1.2195	-0.057	0.0839	-0.1452	0.5777	0.7730188	Organic
Bulk_density	GT18	0.0298	-0.0011	0.0303	-0.2121	0.7415	0.8547761	Organic
Bulk_density	GT2	1761.7613	-85.5275	0.6606	0.5757	0.0493	0.3805859	Organic
Bulk_density	GT19	83.6411	-5.3587	0.7684	0.7105	0.0219	0.3805859	Organic
Bulk_density	GT20	146.1397	2.5756	0.1302	-0.0872	0.4822	0.7298012	Organic
Bulk_density	GT21	93.1775	-10.1878	0.4887	0.3608	0.1222	0.4510213	Organic
Bulk_density	GT22	5.2116	-0.0681	0.0768	-0.154	0.5949	0.7749438	Organic
Bulk_density	GT23	0.3811	-0.1107	0.3465	0.1831	0.219	0.4979652	Organic
Bulk_density	GT24	3.5024	-0.1905	0.0693	-0.1633	0.6142	0.7802348	Organic
Bulk_density	GT25	7.2869	-0.6011	0.291	0.1138	0.2693	0.5588124	Organic
Bulk_density	GT26	148.5105	-9.1438	0.8177	0.7721	0.0133	0.3805859	Organic
Bulk_density	GT27	15.1224	-1.2371	0.4502	0.3127	0.1446	0.4510213	Organic
Bulk_density	GT28	177.4443	-12.0135	0.9163	0.8954	0.0027	0.3805859	Organic
Bulk_density	GT29	3.4324	-0.1779	0.0568	-0.1791	0.6494	0.7945733	Organic
Bulk_density	GT30	121.5074	-8.4917	0.8592	0.8239	0.0078	0.3805859	Organic
Bulk_density	GT3	3.9902	-0.5868	0.3526	0.1908	0.214	0.4979652	Organic
Bulk_density	GT33	1.2253	0.0573	0.2554	0.0692	0.3065	0.6057652	Organic
Bulk_density	GT34	1.1675	0.1873	0.4376	0.297	0.1525	0.4510213	Organic
Bulk_density	GT32	11.1421	1.2415	0.6483	0.5604	0.0532	0.3805859	Organic
Bulk_density	GT31	2.1893	-0.2846	0.1322	-0.0847	0.4786	0.7298012	Organic
Bulk_density	GT35	367.4633	-36.0313	0.8439	0.8048	0.0097	0.3805859	Organic

Bulk_density	GT37	0.0155	0.008	0.2628	0.0785	0.2984	0.5943551	Organic
Bulk_density	GT39	21.5812	1.0369	0.1508	-0.0615	0.4468	0.713148	Organic
Bulk_density	GT4	1706.1942	-39.3665	0.1224	-0.0971	0.4967	0.7362041	Organic
Bulk_density	GT41	451.5852	-72.8044	0.8177	0.7722	0.0133	0.3805859	Organic
Bulk_density	GT40	0.0022	-0.0024	0.4852	0.3565	0.1241	0.4510213	Organic
Bulk_density	GT42	0.0544	-0.0056	0.1849	-0.0189	0.3948	0.6696148	Organic
Bulk_density	GT43	0.1274	-0.0487	0.4551	0.3188	0.1416	0.4510213	Organic
Bulk_density	GT47	80.6211	-8.7981	0.1083	-0.1146	0.5241	0.7525096	Organic
Bulk_density	GT45	0.1867	-0.0319	0.4445	0.3057	0.1481	0.4510213	Organic
Bulk_density	GT44	0.0123	0.0033	0.2281	0.0351	0.3381	0.6218842	Organic
Bulk_density	GT48	4.9851	-0.0562	0.0028	-0.2465	0.9209	0.9397419	Organic
Bulk_density	GT49	0.2847	-0.0155	0.0203	-0.2247	0.7879	0.8837457	Organic
Bulk_density	GT5	106.4158	-4.7129	0.4196	0.2744	0.1643	0.4510213	Organic
Bulk_density	GT50	1.0262	-0.0306	0.0166	-0.2292	0.8076	0.8840328	Organic
Bulk_density	GT53	25.4939	5.4617	0.5814	0.4768	0.0779	0.3966602	Organic
Bulk_density	GT52	0	0	#VALUE!	#VALUE!	#VALUE!	NA	Organic
Bulk_density	GT51	518.0831	7.6665	0.021	-0.2238	0.7844	0.8837457	Organic
Bulk_density	GT54	0.0816	-0.009	0.0291	-0.2136	0.7466	0.8566906	Organic
Bulk_density	GT57	1.9599	0.0624	0.013	-0.2337	0.8297	0.8904843	Organic
Bulk_density	GT56	0.5203	-0.1033	0.4276	0.2844	0.159	0.4510213	Organic
Bulk_density	GT55	2.6159	0.5327	0.3605	0.2006	0.2076	0.4979652	Organic
Bulk_density	GT58	0.7939	0.0182	0.0102	-0.2373	0.8491	0.8997155	Organic
Bulk_density	GT6	0.3853	-0.0718	0.4788	0.3485	0.1277	0.4510213	Organic
Bulk_density	GT59	0.5119	0.052	0.1438	-0.0703	0.4585	0.7180524	Organic
Bulk_density	GT60	3.4349	-0.5388	0.1929	-0.0088	0.3835	0.6678099	Organic
Bulk_density	GT61	0.5101	-0.123	0.372	0.215	0.1986	0.4966114	Organic
Bulk_density	GT62	2.3868	0.1076	0.1384	-0.077	0.4677	0.7230726	Organic
Bulk_density	GT64	0.751	-0.0398	0.2368	0.0459	0.3277	0.6198176	Organic
Bulk_density	GT65	0.0811	-0.0133	0.092	-0.135	0.559	0.7730188	Organic
Bulk_density	GT66	16.8699	-1.0799	0.4994	0.3743	0.1164	0.4510213	Organic
Bulk_density	GT69	1.138	0.1015	0.0806	-0.1493	0.5857	0.7749438	Organic
Bulk_density	GT68	0.0598	-0.0058	0.0308	-0.2115	0.7393	0.8547761	Organic
Bulk_density	GT7	6.1602	-0.9089	0.7131	0.6414	0.0344	0.3805859	Organic
Bulk_density	GT72	0.0003	0.0004	0.3238	0.1547	0.2386	0.525761	Organic
Bulk_density	GT73	0.071	-0.013	0.0902	-0.1372	0.5629	0.7730188	Organic
Bulk_density	GT71	1.6293	-0.121	0.1822	-0.0222	0.3986	0.6696148	Organic
Bulk_density	GT70	0.7452	-0.0182	0.1004	-0.1245	0.5406	0.7648737	Organic
Bulk_density	GT74	0.8293	-0.0433	0.0517	-0.1854	0.6649	0.7998476	Organic
Bulk_density	GT75	0.956	-0.1602	0.5078	0.3847	0.112	0.4510213	Organic
Bulk_density	GT76	1.3751	0.5865	0.2268	0.0335	0.3397	0.6218842	Organic
Bulk_density	GT77	0.6069	0.1717	0.1654	-0.0432	0.4235	0.6937932	Organic
Bulk_density	GT78	0.0028	0.0027	0.3515	0.1893	0.2149	0.4979652	Organic
Bulk_density	GT8	10.5137	-0.1649	0.0565	-0.1793	0.65	0.7945733	Organic
Bulk_density	GT80	6.3954	-0.7871	0.5965	0.4957	0.0718	0.3805859	Organic
Bulk_density	GT82	0.3053	-0.024	0.0713	-0.1608	0.6089	0.7787097	Organic
Bulk_density	GT84	319.0374	-60.7707	0.7701	0.7126	0.0216	0.3805859	Organic
Bulk_density	GT81	23.6857	0.1781	0.0044	-0.2445	0.9007	0.9309841	Organic
Bulk_density	GT83	177.9561	-3.5869	0.0853	-0.1434	0.5743	0.7730188	Organic
Bulk_density	GT85	3.5991	0.8141	0.4661	0.3326	0.1351	0.4510213	Organic
Bulk_density	GT87	20.2064	-0.0977	0.0014	-0.2483	0.9442	0.9557473	Organic
Bulk_density	GT88	0.0469	0.0028	0.0066	-0.2417	0.8781	0.926495	Organic
Bulk_density	GT89	5.8171	0.813	0.4703	0.3379	0.1326	0.4510213	Organic
Bulk_density	GT90	3.3542	-0.2356	0.071	-0.1613	0.6098	0.7787097	Organic
Bulk_density	GT9	154.7911	-9.5553	0.5734	0.4668	0.0812	0.3966602	Organic
Bulk_density	GT91	0.0217	0.0073	0.4231	0.2788	0.1619	0.4510213	Organic

Bulk_density	GT93	0.3645	0.013	0.1634	-0.0457	0.4267	0.6943992	Organic
Bulk_density	GT94	1.0193	-0.2278	0.6982	0.6227	0.0383	0.3805859	Organic
Bulk_density	GT92	0.2101	-0.0193	0.098	-0.1275	0.5458	0.7678011	Organic
Bulk_density	GT95	6.3578	-0.4012	0.2406	0.0508	0.3232	0.6198176	Organic
Bulk_density	PL1	14.0854	-1.8311	0.6562	0.5703	0.0507	0.3805859	Organic
Bulk_density	PL10	5.2376	-0.909	0.2521	0.0651	0.3102	0.6081065	Organic
Bulk_density	PL11	2.8656	-0.947	0.4452	0.3065	0.1477	0.4510213	Organic
Bulk_density	PL12	22.902	-3.7552	0.7825	0.7281	0.0192	0.3805859	Organic
Bulk_density	PL13	0.0005	0.0007	0.3238	0.1547	0.2386	0.525761	Organic
Bulk_density	PL14	0.8538	0.1133	0.1451	-0.0686	0.4562	0.7180524	Organic
Bulk_density	PL15	0.1739	-0.0213	0.2032	0.004	0.3696	0.6527367	Organic
Bulk_density	PL17	0.9434	-0.0575	0.0772	-0.1535	0.594	0.7749438	Organic
Bulk_density	PL18	0	0	#VALUE!	#VALUE!	#VALUE!	NA	Organic
Bulk_density	PL16	0.5082	-0.0709	0.6057	0.5071	0.0683	0.3805859	Organic
Bulk_density	PL2	0.3036	-0.1174	0.7022	0.6277	0.0373	0.3805859	Organic
Bulk_density	PL20	0.0658	0.0027	0.017	-0.2288	0.8058	0.8840328	Organic
Bulk_density	PL21	0.5253	-0.0773	0.1174	-0.1033	0.5062	0.7458688	Organic
Bulk_density	PL22	12.3762	-2.0968	0.62	0.525	0.063	0.3805859	Organic
Bulk_density	PL23	0.02	-0.0034	0.7465	0.6832	0.0265	0.3805859	Organic
Bulk_density	PL3	0.0966	-0.0031	0.0057	-0.2428	0.8866	0.9275811	Organic
Bulk_density	PL4	26.4684	-6.8633	0.3358	0.1698	0.2281	0.5116052	Organic
Bulk_density	PL5	4.576	-0.4746	0.0461	-0.1924	0.683	0.8137236	Organic
Bulk_density	PL6	1.1647	-0.3041	0.641	0.5513	0.0557	0.3805859	Organic
Bulk_density	PL7	3.6063	-0.1584	0.0331	-0.2086	0.73	0.8493449	Organic
Bulk_density	PL8	3.0069	-0.5457	0.3656	0.207	0.2036	0.4969673	Organic
Bulk_density	PL9	28.8683	-3.5172	0.4692	0.3364	0.1332	0.4510213	Organic
pH	AA1	179.0321	-7.9179	0.2556	0.0694	0.3063	0.52241	Organic
pH	AA11	0.5025	-0.1209	0.4216	0.277	0.1629	0.397233	Organic
pH	AA10	7.5616	-1.1543	0.5544	0.443	0.0895	0.3184039	Organic
pH	AA2	1.8202	-0.2017	0.1644	-0.0444	0.4251	0.6013628	Organic
pH	AA3	383.3947	30.273	0.3381	0.1726	0.2261	0.4611034	Organic
pH	AA4	20.7999	-4.0064	0.2866	0.1082	0.2737	0.4939061	Organic
pH	AA5	32.5624	11.2465	0.859	0.8237	0.0078	0.1396008	Organic
pH	AA8	0.8225	-0.0358	0.0345	-0.2069	0.7248	0.7985464	Organic
pH	AA7	52.2765	3.5544	0.1574	-0.0533	0.4362	0.6067569	Organic
pH	AA6	24.3337	2.4115	0.2105	0.0132	0.36	0.5662584	Organic
pH	AA9	1.8306	-0.2962	0.4066	0.2582	0.1732	0.397233	Organic
pH	CE1	208.9789	4.4726	0.3765	0.2206	0.1951	0.4207249	Organic
pH	CE11	155.7435	-1.2791	0.0079	-0.2401	0.8672	0.9034704	Organic
pH	CE12	20.9813	-3.2878	0.3988	0.2485	0.1787	0.403498	Organic
pH	CE13	0.0653	-0.0143	0.0752	-0.156	0.599	0.7068659	Organic
pH	CE14	227.4441	-9.4519	0.2365	0.0456	0.328	0.5445323	Organic
pH	CE15	52.1006	-8.7477	0.5286	0.4108	0.1016	0.3300081	Organic
pH	CE2	3.239	-0.3629	0.1424	-0.072	0.4608	0.6235859	Organic
pH	CE3	15.1515	0.5689	0.0356	-0.2055	0.7203	0.7971009	Organic
pH	CE4	294.1051	52.7318	0.7553	0.6941	0.0246	0.1944584	Organic
pH	CE16	1.0735	-0.1291	0.4336	0.292	0.155	0.397233	Organic
pH	CE5	14.9063	1.7479	0.1049	-0.1189	0.5311	0.6547176	Organic
pH	CE7	12.9532	-1.4706	0.3486	0.1857	0.2173	0.4471275	Organic
pH	CE6	52.0839	-9.7796	0.7951	0.7439	0.017	0.1594416	Organic
pH	CE8	24.9683	-3.2032	0.4857	0.3571	0.1239	0.3586619	Organic
pH	CE9	90.7663	-1.0291	0.0586	-0.1768	0.644	0.7389554	Organic
pH	GH100	1.1884	-0.3295	0.1336	-0.083	0.4762	0.6278955	Organic
pH	GH10	59.1017	-5.8368	0.5473	0.4341	0.0928	0.3194665	Organic
pH	GH102	34.7782	2.5283	0.8012	0.7515	0.0159	0.1594416	Organic

pH	GH101	0.4319	0.0448	0.304	0.13	0.2568	0.4807135	Organic
pH	GH103	87.9513	13.6727	0.542	0.4275	0.0952	0.320351	Organic
pH	GH104	4.2879	0.6759	0.8987	0.8734	0.004	0.1396008	Organic
pH	GH105	39.1132	-10.0123	0.8422	0.8027	0.0099	0.1396008	Organic
pH	GH106	64.736	-11.5763	0.5603	0.4503	0.0869	0.3152509	Organic
pH	GH107	0.05	0.0164	0.4211	0.2764	0.1632	0.397233	Organic
pH	GH108	6.1612	-0.7207	0.8189	0.7736	0.0131	0.1556835	Organic
pH	GH109	20.7365	-1.9214	0.5797	0.4746	0.0786	0.3005144	Organic
pH	GH11	18.0145	-3.5482	0.7106	0.6383	0.035	0.2181528	Organic
pH	GH110	1.6372	-0.3182	0.4215	0.2768	0.163	0.397233	Organic
pH	GH111	0.0241	-0.0068	0.5458	0.4322	0.0934	0.3194665	Organic
pH	GH112	0.1865	-0.0724	0.2384	0.048	0.3258	0.5445257	Organic
pH	GH113	7.4538	0.4256	0.1079	-0.1151	0.525	0.6547176	Organic
pH	GH114	8.4827	3.4141	0.8505	0.8132	0.0088	0.1396008	Organic
pH	GH115	26.0154	-6.5731	0.4056	0.2569	0.1739	0.397233	Organic
pH	GH116	54.6915	-5.8524	0.4435	0.3044	0.1488	0.397233	Organic
pH	GH117	3.207	-0.5201	0.8331	0.7914	0.0111	0.1396008	Organic
pH	GH118	0.0019	-0.0004	0.0202	-0.2247	0.7881	0.8350976	Organic
pH	GH119	1.7272	0.1194	0.0665	-0.1669	0.6217	0.7234374	Organic
pH	GH12	10.5185	-2.0394	0.5082	0.3853	0.1118	0.3370171	Organic
pH	GH120	2.0347	-0.4994	0.6248	0.531	0.0613	0.2630127	Organic
pH	GH121	0.74	0.0227	0.0246	-0.2193	0.7667	0.815847	Organic
pH	GH123	0.8792	0.8109	0.8908	0.8635	0.0046	0.1396008	Organic
pH	GH124	0.0228	0.036	0.8538	0.8172	0.0084	0.1396008	Organic
pH	GH125	14.4186	-2.1408	0.3971	0.2464	0.1799	0.403498	Organic
pH	GH126	0.0126	-0.0035	0.0908	-0.1365	0.5616	0.6788208	Organic
pH	GH127	53.4987	-7.3969	0.4737	0.3422	0.1306	0.369537	Organic
pH	GH128	9.2104	-1.6048	0.5084	0.3855	0.1117	0.3370171	Organic
pH	GH129	0.4364	-0.0453	0.0827	-0.1466	0.5805	0.6949666	Organic
pH	GH13	1477.2569	0.6248	1.00E-04	-0.2499	0.9864	0.9864273	Organic
pH	GH130	95.5733	-2.685	0.0326	-0.2093	0.7321	0.8026501	Organic
pH	GH131	0.252	-0.0439	0.2282	0.0353	0.3379	0.5535328	Organic
pH	GH132	0.9233	-0.1134	0.2095	0.0119	0.3613	0.5662584	Organic
pH	GH133	59.9767	9.3984	0.8731	0.8414	0.0063	0.1396008	Organic
pH	GH14	0.6765	-0.0775	0.1672	-0.041	0.4208	0.598804	Organic
pH	GH17	51.2924	5.7328	0.4251	0.2814	0.1606	0.397233	Organic
pH	GH15	420.1827	-8.6016	0.0688	-0.164	0.6156	0.7196529	Organic
pH	GH18	255.9295	-20.4284	0.5901	0.4876	0.0744	0.2987474	Organic
pH	GH19	15.6439	-0.3777	0.0546	-0.1818	0.656	0.7492767	Organic
pH	GH2	452.5084	-48.4953	0.3682	0.2103	0.2015	0.4251707	Organic
pH	GH20	103.5535	-11.3434	0.6817	0.6021	0.0429	0.2328985	Organic
pH	GH23	366.3567	9.7181	0.76	0.7	0.0236	0.1944584	Organic
pH	GH24	15.7222	-0.6516	0.1405	-0.0744	0.4641	0.6245882	Organic
pH	GH25	18.1223	-0.5516	0.1582	-0.0523	0.4349	0.6067569	Organic
pH	GH26	23.6541	-0.3202	0.0065	-0.2419	0.8794	0.9047864	Organic
pH	GH27	104.8235	-17.718	0.6265	0.5331	0.0607	0.2630127	Organic
pH	GH28	237.447	-32.8732	0.4215	0.2769	0.163	0.397233	Organic
pH	GH29	132.2116	-11.0351	0.3202	0.1502	0.2418	0.4778612	Organic
pH	GH3	680.4412	-77.9789	0.6496	0.5619	0.0528	0.2410279	Organic
pH	GH30	59.6043	-7.6379	0.8406	0.8007	0.0101	0.1396008	Organic
pH	GH31	173.7587	-22.1874	0.6583	0.5729	0.05	0.2397993	Organic
pH	GH33	48.5541	2.6377	0.2875	0.1094	0.2728	0.4939061	Organic
pH	GH36	117.4663	-18.7471	0.5923	0.4904	0.0735	0.2987474	Organic
pH	GH32	30.1748	2.1179	0.3888	0.236	0.1859	0.4130092	Organic
pH	GH37	45.5476	-6.949	0.7845	0.7306	0.0189	0.1676535	Organic

pH	GH39	110.1752	-10.7096	0.3076	0.1345	0.2534	0.4807135	Organic
pH	GH38	153.8558	-28.536	0.6973	0.6216	0.0386	0.2320054	Organic
pH	GH4	141.0485	2.1882	0.0266	-0.2168	0.7577	0.8158169	Organic
pH	GH42	52.5554	-1.4538	0.1016	-0.123	0.5381	0.6600604	Organic
pH	GH43	133.3589	-20.3859	0.5855	0.4819	0.0762	0.2999517	Organic
pH	GH44	32.7913	-1.3935	0.0289	-0.2138	0.7474	0.8094712	Organic
pH	GH45	0.5846	-0.0803	0.152	-0.06	0.4448	0.6077144	Organic
pH	GH47	14.6845	-1.4907	0.6581	0.5727	0.0501	0.2397993	Organic
pH	GH46	1.4741	0.2208	0.2994	0.1243	0.2611	0.4816358	Organic
pH	GH48	10.4606	-1.2909	0.5249	0.4061	0.1034	0.3300782	Organic
pH	GH49	0.0919	-0.008	0.1201	-0.0999	0.501	0.6418542	Organic
pH	GH5	172.0215	-3.2336	0.089	-0.1387	0.5657	0.6804855	Organic
pH	GH50	7.7274	1.1399	0.4613	0.3267	0.1379	0.3771972	Organic
pH	GH51	136.9965	-15.4055	0.3731	0.2164	0.1977	0.4207249	Organic
pH	GH54	33.242	-10.4905	0.8023	0.7529	0.0157	0.1594416	Organic
pH	GH53	65.4769	-4.2423	0.1305	-0.0869	0.4817	0.6289414	Organic
pH	GH55	145.3713	-30.2898	0.896	0.87	0.0042	0.1396008	Organic
pH	GH56	0.0085	-0.0044	0.1867	-0.0167	0.3923	0.5919583	Organic
pH	GH57	148.2848	8.4276	0.2453	0.0566	0.3178	0.5347098	Organic
pH	GH58	0.0299	0.0043	0.2079	0.0099	0.3634	0.5662584	Organic
pH	GH59	11.8995	-3.123	0.675	0.5938	0.0449	0.2328985	Organic
pH	GH6	21.9717	-2.9397	0.5208	0.401	0.1054	0.3322836	Organic
pH	GH62	3.402	-0.2068	0.0288	-0.2139	0.7477	0.8094712	Organic
pH	GH63	18.035	-2.4243	0.4957	0.3696	0.1184	0.3510572	Organic
pH	GH64	23.3696	-3.04	0.5758	0.4698	0.0802	0.3005144	Organic
pH	GH65	99.333	-2.2276	0.026	-0.2175	0.7601	0.8158169	Organic
pH	GH66	1.6072	0.0393	0.0046	-0.2443	0.8987	0.9208807	Organic
pH	GH68	0.3029	0.0638	0.6769	0.5962	0.0443	0.2328985	Organic
pH	GH7	0.8798	-0.1078	0.2337	0.0422	0.3313	0.5463396	Organic
pH	GH70	0.0313	0.0005	0.0008	-0.249	0.9567	0.964476	Organic
pH	GH71	4.8837	-1.2829	0.6367	0.5459	0.0571	0.2539254	Organic
pH	GH72	30.0891	-0.7175	0.1706	-0.0367	0.4156	0.5982352	Organic
pH	GH73	36.2954	0.6598	0.3121	0.1401	0.2492	0.4801365	Organic
pH	GH75	2.0444	-0.2788	0.5072	0.384	0.1123	0.3370171	Organic
pH	GH74	16.7603	0.227	0.0032	-0.246	0.9158	0.9269345	Organic
pH	GH76	18.3675	-2.5032	0.6699	0.5873	0.0464	0.2360196	Organic
pH	GH78	173.1756	-20.0572	0.6188	0.5235	0.0634	0.2676905	Organic
pH	GH79	33.6982	-4.1934	0.8697	0.8371	0.0067	0.1396008	Organic
pH	GH8	19.2144	-2.5025	0.4363	0.2954	0.1533	0.397233	Organic
pH	GH80	0	0	#VALUE!	#VALUE!	#VALUE!	NA	Organic
pH	GH81	5.1377	0.4338	0.168	-0.04	0.4196	0.598804	Organic
pH	GH84	4.79	-0.5057	0.3845	0.2306	0.1891	0.4130092	Organic
pH	GH85	0.5304	-0.0191	0.0319	-0.2101	0.735	0.8026501	Organic
pH	GH86	1.1757	0.2315	0.9373	0.9217	0.0015	0.1396008	Organic
pH	GH87	78.9746	-10.4535	0.7662	0.7077	0.0224	0.1919059	Organic
pH	GH88	23.1746	0.8586	0.2178	0.0222	0.3508	0.5635732	Organic
pH	GH89	9.9199	-2.5207	0.867	0.8337	0.007	0.1396008	Organic
pH	GH91	0.0458	0.0315	0.7383	0.6728	0.0283	0.1976227	Organic
pH	GH90	0.0009	-0.0006	0.0641	-0.1699	0.6284	0.7244214	Organic
pH	GH9	60.9971	-8.38	0.5593	0.4491	0.0874	0.3152509	Organic
pH	GH92	121.0279	-24.8937	0.7372	0.6715	0.0286	0.1976227	Organic
pH	GH93	18.7765	-1.524	0.183	-0.0212	0.3974	0.5934019	Organic
pH	GH94	39.1359	-4.5103	0.1337	-0.0828	0.4759	0.6278955	Organic
pH	GH95	57.25	-6.3928	0.417	0.2712	0.166	0.397233	Organic
pH	GH96	0.1511	0.0037	0.0032	-0.2459	0.9147	0.9269345	Organic

pH	GH97	30.1161	-1.8779	0.5743	0.4679	0.0809	0.3005144	Organic
pH	GH98	0.1755	0.0965	0.9031	0.8789	0.0036	0.1396008	Organic
pH	GH99	9.8752	0.8563	0.4911	0.3639	0.1209	0.3541058	Organic
pH	GT1	150.285	1.5161	0.0037	-0.2454	0.9093	0.9269345	Organic
pH	GT10	3.0892	-0.316	0.1333	-0.0833	0.4766	0.6278955	Organic
pH	GT12	0.0472	-0.0082	0.1511	-0.0611	0.4462	0.6077144	Organic
pH	GT14	2.1246	-0.3475	0.5453	0.4317	0.0937	0.3194665	Organic
pH	GT11	5.7255	-0.6344	0.2072	0.0091	0.3643	0.5662584	Organic
pH	GT13	0.2735	-0.1053	0.4638	0.3297	0.1364	0.3771972	Organic
pH	GT15	2.516	-0.2016	0.1752	-0.031	0.4088	0.5957707	Organic
pH	GT16	0.0801	-0.0099	0.1192	-0.101	0.5027	0.6418542	Organic
pH	GT17	1.2195	-0.1005	0.2602	0.0753	0.3012	0.522207	Organic
pH	GT18	0.0298	-0.0008	0.0152	-0.231	0.8158	0.8571442	Organic
pH	GT2	1761.7613	49.4226	0.2206	0.0257	0.3473	0.5625868	Organic
pH	GT19	83.6411	-2.5566	0.1749	-0.0314	0.4092	0.5957707	Organic
pH	GT20	146.1397	2.3459	0.108	-0.1149	0.5247	0.6547176	Organic
pH	GT21	93.1775	-9.8607	0.4578	0.3222	0.14	0.3788304	Organic
pH	GT22	5.2116	-0.02	0.0066	-0.2417	0.8782	0.9047864	Organic
pH	GT23	0.3811	-0.1111	0.3492	0.1866	0.2167	0.4471275	Organic
pH	GT24	3.5024	-0.4618	0.4075	0.2594	0.1725	0.397233	Organic
pH	GT25	7.2869	-0.9479	0.7238	0.6548	0.0317	0.2026415	Organic
pH	GT26	148.5105	-4.2103	0.1734	-0.0333	0.4115	0.5957707	Organic
pH	GT27	15.1224	-1.02	0.306	0.1325	0.2548	0.4807135	Organic
pH	GT28	177.4443	1.0553	0.0071	-0.2412	0.8742	0.9047864	Organic
pH	GT29	3.4324	0.6364	0.7263	0.6579	0.0311	0.2026415	Organic
pH	GT30	121.5074	-1.1452	0.0156	-0.2305	0.8135	0.8571442	Organic
pH	GT3	3.9902	-0.8225	0.6926	0.6158	0.0398	0.2320054	Organic
pH	GT33	1.2253	0.0375	0.1091	-0.1136	0.5226	0.6547176	Organic
pH	GT34	1.1675	-0.0603	0.0453	-0.1933	0.6855	0.7688288	Organic
pH	GT32	11.1421	0.8664	0.3157	0.1446	0.2459	0.4783001	Organic
pH	GT31	2.1893	-0.5614	0.5143	0.3928	0.1087	0.3370171	Organic
pH	GT35	367.4633	-11.1972	0.0815	-0.1481	0.5834	0.6950795	Organic
pH	GT37	0.0155	0.0136	0.7473	0.6842	0.0263	0.1944584	Organic
pH	GT39	21.5812	-0.4217	0.0249	-0.2188	0.7651	0.815847	Organic
pH	GT4	1706.1942	100.6656	0.8001	0.7501	0.0161	0.1594416	Organic
pH	GT41	451.5852	-33.9015	0.1773	-0.0284	0.4057	0.5957707	Organic
pH	GT40	0.0022	-0.0007	0.0471	-0.1911	0.6796	0.7688288	Organic
pH	GT42	0.0544	-0.0096	0.5306	0.4132	0.1006	0.3300081	Organic
pH	GT43	0.1274	-0.0338	0.2201	0.0251	0.3479	0.5625868	Organic
pH	GT47	80.6211	-11.8743	0.1973	-0.0034	0.3775	0.576708	Organic
pH	GT45	0.1867	0.0263	0.3006	0.1258	0.26	0.4816358	Organic
pH	GT44	0.0123	0.0042	0.3735	0.2169	0.1974	0.4207249	Organic
pH	GT48	4.9851	-0.9477	0.7932	0.7415	0.0173	0.1594416	Organic
pH	GT49	0.2847	-0.0881	0.6543	0.5678	0.0513	0.2410279	Organic
pH	GT5	106.4158	3.6056	0.2456	0.057	0.3175	0.5347098	Organic
pH	GT50	1.0262	-0.1969	0.6885	0.6107	0.041	0.2320054	Organic
pH	GT53	25.4939	1.5534	0.047	-0.1912	0.6798	0.7688288	Organic
pH	GT52	0	0	#VALUE!	#VALUE!	#VALUE!	NA	Organic
pH	GT51	518.0831	52.1885	0.971	0.9637	0.0003	0.0795034	Organic
pH	GT54	0.0816	-0.0305	0.3323	0.1653	0.2311	0.4636639	Organic
pH	GT57	1.9599	-0.4461	0.6654	0.5817	0.0478	0.2381548	Organic
pH	GT56	0.5203	-0.1147	0.5276	0.4096	0.1021	0.3300081	Organic
pH	GT55	2.6159	-0.5144	0.3361	0.1702	0.2278	0.4611034	Organic
pH	GT58	0.7939	-0.1486	0.6778	0.5972	0.0441	0.2328985	Organic
pH	GT6	0.3853	-0.0403	0.1509	-0.0614	0.4466	0.6077144	Organic

pH	GT59	0.5119	-0.0811	0.3491	0.1863	0.2169	0.4471275	Organic
pH	GT60	3.4349	-1.0976	0.8005	0.7507	0.016	0.1594416	Organic
pH	GT61	0.5101	-0.1136	0.3173	0.1467	0.2444	0.4783001	Organic
pH	GT62	2.3868	-0.0776	0.072	-0.16	0.6071	0.7131099	Organic
pH	GT64	0.751	0.0625	0.5834	0.4792	0.0771	0.2999517	Organic
pH	GT65	0.0811	-0.0283	0.4174	0.2718	0.1657	0.397233	Organic
pH	GT66	16.8699	0.653	0.1826	-0.0217	0.398	0.5934019	Organic
pH	GT69	1.138	-0.297	0.6898	0.6122	0.0406	0.2320054	Organic
pH	GT68	0.0598	-0.0102	0.0953	-0.1309	0.5517	0.6733434	Organic
pH	GT7	6.1602	-0.5614	0.272	0.09	0.2886	0.5132765	Organic
pH	GT72	0.0003	-0.0003	0.2041	0.0052	0.3684	0.5662584	Organic
pH	GT73	0.071	-0.0161	0.1376	-0.078	0.4691	0.6278955	Organic
pH	GT71	1.6293	-0.263	0.8614	0.8268	0.0076	0.1396008	Organic
pH	GT70	0.7452	-0.0321	0.3105	0.1381	0.2507	0.4801365	Organic
pH	GT74	0.8293	0.0683	0.1287	-0.0891	0.485	0.6289414	Organic
pH	GT75	0.956	-0.0431	0.0367	-0.2042	0.7163	0.7962369	Organic
pH	GT76	1.3751	1.0639	0.7462	0.6827	0.0266	0.1944584	Organic
pH	GT77	0.6069	0.3851	0.8322	0.7902	0.0112	0.1396008	Organic
pH	GT78	0.0028	0.0031	0.4626	0.3282	0.1371	0.3771972	Organic
pH	GT8	10.5137	-0.2458	0.1257	-0.0929	0.4905	0.6328466	Organic
pH	GT80	6.3954	-0.5172	0.2576	0.072	0.3041	0.522207	Organic
pH	GT82	0.3053	0.0836	0.8677	0.8347	0.0069	0.1396008	Organic
pH	GT84	319.0374	-21.2286	0.094	-0.1325	0.5546	0.6736224	Organic
pH	GT81	23.6857	1.0668	0.1577	-0.0528	0.4356	0.6067569	Organic
pH	GT83	177.9561	-2.4447	0.0396	-0.2005	0.7053	0.7875755	Organic
pH	GT85	3.5991	0.3025	0.0644	-0.1696	0.6276	0.7244214	Organic
pH	GT87	20.2064	-1.6327	0.3866	0.2333	0.1875	0.4130092	Organic
pH	GT88	0.0469	-0.0223	0.4232	0.279	0.1618	0.397233	Organic
pH	GT89	5.8171	0.4258	0.129	-0.0887	0.4844	0.6289414	Organic
pH	GT90	3.3542	-0.7657	0.7499	0.6874	0.0257	0.1944584	Organic
pH	GT9	154.7911	5.2888	0.1757	-0.0304	0.4081	0.5957707	Organic
pH	GT91	0.0217	-0.006	0.2899	0.1124	0.2704	0.4939061	Organic
pH	GT93	0.3645	-0.017	0.2815	0.1019	0.2788	0.4995135	Organic
pH	GT94	1.0193	-0.1246	0.209	0.0113	0.362	0.5662584	Organic
pH	GT92	0.2101	-0.0403	0.4276	0.2845	0.1589	0.397233	Organic
pH	GT95	6.3578	0.47	0.3304	0.163	0.2328	0.4636639	Organic
pH	PL1	14.0854	0.2192	0.0094	-0.2382	0.855	0.8945098	Organic
pH	PL10	5.2376	-1.5466	0.7299	0.6623	0.0303	0.2026415	Organic
pH	PL11	2.8656	0.0219	0.0002	-0.2497	0.9769	0.9807958	Organic
pH	PL12	22.902	-1.3876	0.1068	-0.1165	0.5272	0.6547176	Organic
pH	PL13	0.0005	-0.0006	0.2041	0.0052	0.3684	0.5662584	Organic
pH	PL14	0.8538	0.2723	0.8388	0.7985	0.0103	0.1396008	Organic
pH	PL15	0.1739	0.0241	0.2589	0.0736	0.3027	0.522207	Organic
pH	PL17	0.9434	0.1066	0.2651	0.0814	0.2959	0.5188292	Organic
pH	PL18	0	0	#VALUE!	#VALUE!	#VALUE!	NA	Organic
pH	PL16	0.5082	-0.0295	0.1052	-0.1185	0.5306	0.6547176	Organic
pH	PL2	0.3036	-0.0546	0.1518	-0.0602	0.4451	0.6077144	Organic
pH	PL20	0.0658	-0.0145	0.4808	0.3509	0.1266	0.362396	Organic
pH	PL21	0.5253	-0.1445	0.4105	0.2631	0.1705	0.397233	Organic
pH	PL22	12.3762	-1.1756	0.1949	-0.0064	0.3808	0.5781607	Organic
pH	PL23	0.02	0.0011	0.0779	-0.1526	0.5922	0.7022307	Organic
pH	PL3	0.0966	-0.0133	0.1068	-0.1165	0.5273	0.6547176	Organic
pH	PL4	26.4684	-6.1302	0.2679	0.0849	0.2929	0.5173281	Organic
pH	PL5	4.576	-2.0353	0.8472	0.809	0.0092	0.1396008	Organic
pH	PL6	1.1647	-0.0814	0.0459	-0.1926	0.6836	0.7688288	Organic

pH	PL7	3.6063	-0.7006	0.6483	0.5604	0.0532	0.2410279	Organic
pH	PL8	3.0069	-0.7052	0.6105	0.5132	0.0665	0.2758921	Organic
pH	PL9	28.8683	-3.2738	0.4065	0.2581	0.1732	0.397233	Organic
C	AA1	179.0321	10.4356	0.4439	0.3049	0.1485	0.2768739	Organic
C	AA11	0.5025	0.0162	0.0075	-0.2406	0.8701	0.9183984	Organic
C	AA10	7.5616	1.2301	0.6297	0.5371	0.0595	0.1611724	Organic
C	AA2	1.8202	0.1896	0.1453	-0.0683	0.4558	0.598051	Organic
C	AA3	383.3947	-49.3139	0.8972	0.8715	0.0041	0.0386307	Organic
C	AA4	20.7999	5.3572	0.5124	0.3904	0.1097	0.2404138	Organic
C	AA5	32.5624	-11.5692	0.909	0.8863	0.0032	0.0386307	Organic
C	AA8	0.8225	0.0592	0.0939	-0.1326	0.5548	0.6705529	Organic
C	AA7	52.2765	-6.9209	0.5966	0.4958	0.0718	0.1849473	Organic
C	AA6	24.3337	-4.6079	0.7688	0.7109	0.0218	0.0921568	Organic
C	AA9	1.8306	0.1474	0.1006	-0.1243	0.5402	0.6561563	Organic
C	CE1	208.9789	-2.4845	0.1162	-0.1048	0.5085	0.6362964	Organic
C	CE11	155.7435	9.1166	0.4003	0.2504	0.1776	0.3135694	Organic
C	CE12	20.9813	4.1694	0.6413	0.5517	0.0556	0.1536979	Organic
C	CE13	0.0653	0.0181	0.1207	-0.0991	0.4997	0.6348809	Organic
C	CE14	227.4441	16.0154	0.679	0.5988	0.0437	0.1378206	Organic
C	CE15	52.1006	11.7641	0.956	0.9451	0.0007	0.0291397	Organic
C	CE2	3.239	0.7765	0.652	0.5649	0.052	0.150684	Organic
C	CE3	15.1515	-1.5228	0.2552	0.069	0.3067	0.4656262	Organic
C	CE4	294.1051	-59.3905	0.9581	0.9476	0.0007	0.0291397	Organic
C	CE16	1.0735	0.0435	0.0493	-0.1883	0.6723	0.7679327	Organic
C	CE5	14.9063	-4.2939	0.6331	0.5414	0.0583	0.1596412	Organic
C	CE7	12.9532	2.2824	0.8397	0.7996	0.0102	0.0604891	Organic
C	CE6	52.0839	10.5007	0.9167	0.8959	0.0027	0.0386307	Organic
C	CE8	24.9683	4.3787	0.9075	0.8844	0.0033	0.0386307	Organic
C	CE9	90.7663	1.4222	0.1119	-0.1101	0.5169	0.6399745	Organic
C	GH100	1.1884	0.3566	0.1564	-0.0545	0.4377	0.5875476	Organic
C	GH10	59.1017	7.7048	0.9536	0.942	0.0008	0.0291397	Organic
C	GH102	34.7782	-2.5218	0.7971	0.7463	0.0166	0.0796082	Organic
C	GH101	0.4319	-0.0681	0.7019	0.6273	0.0373	0.1298924	Organic
C	GH103	87.9513	-17.6748	0.9057	0.8822	0.0034	0.0386307	Organic
C	GH104	4.2879	-0.4365	0.3748	0.2185	0.1964	0.3304563	Organic
C	GH105	39.1132	9.6849	0.788	0.735	0.0182	0.0839809	Organic
C	GH106	64.736	14.4229	0.8697	0.8371	0.0067	0.0448664	Organic
C	GH107	0.05	-0.0179	0.5005	0.3756	0.1159	0.2424527	Organic
C	GH108	6.1612	0.4644	0.34	0.175	0.2245	0.3702178	Organic
C	GH109	20.7365	2.4559	0.947	0.9338	0.0011	0.0293969	Organic
C	GH11	18.0145	3.7983	0.8143	0.7679	0.0138	0.0748235	Organic
C	GH110	1.6372	0.2295	0.2193	0.0241	0.349	0.5065066	Organic
C	GH111	0.0241	0.003	0.1055	-0.1182	0.53	0.6469163	Organic
C	GH112	0.1865	0.1099	0.5497	0.4372	0.0916	0.2152701	Organic
C	GH113	7.4538	0.3549	0.075	-0.1562	0.5994	0.7040679	Organic
C	GH114	8.4827	-3.5547	0.922	0.9025	0.0023	0.0386307	Organic
C	GH115	26.0154	9.6088	0.8667	0.8333	0.007	0.045597	Organic
C	GH116	54.6915	7.1573	0.6633	0.5791	0.0485	0.1453735	Organic
C	GH117	3.207	0.5073	0.7927	0.7408	0.0174	0.0816704	Organic
C	GH118	0.0019	0.0007	0.063	-0.1712	0.6313	0.7345362	Organic
C	GH119	1.7272	0.1628	0.1238	-0.0953	0.4941	0.634727	Organic
C	GH12	10.5185	2.5349	0.7851	0.7314	0.0187	0.0846458	Organic
C	GH120	2.0347	0.4211	0.4443	0.3053	0.1483	0.2768739	Organic
C	GH121	0.74	-0.0129	0.0079	-0.2401	0.8672	0.9183984	Organic
C	GH123	0.8792	-0.8117	0.8925	0.8656	0.0045	0.0386307	Organic

C	GH124	0.0228	-0.0368	0.893	0.8663	0.0045	0.0386307	Organic
C	GH125	14.4186	1.3817	0.1654	-0.0432	0.4236	0.5731916	Organic
C	GH126	0.0126	0.0015	0.0162	-0.2297	0.8099	0.8845359	Organic
C	GH127	53.4987	10.1631	0.8943	0.8679	0.0043	0.0386307	Organic
C	GH128	9.2104	1.228	0.2977	0.1221	0.2628	0.4195176	Organic
C	GH129	0.4364	0.1214	0.5941	0.4926	0.0728	0.1849473	Organic
C	GH13	1477.2569	-28.2784	0.1677	-0.0404	0.4201	0.5715473	Organic
C	GH130	95.5733	10.9284	0.54	0.425	0.0961	0.2200273	Organic
C	GH131	0.252	0.0306	0.1108	-0.1115	0.5192	0.6399745	Organic
C	GH132	0.9233	-0.0164	0.0044	-0.2445	0.901	0.9370374	Organic
C	GH133	59.9767	-8.4531	0.7063	0.6329	0.0362	0.129833	Organic
C	GH14	0.6765	0.0662	0.1222	-0.0973	0.4971	0.634727	Organic
C	GH17	51.2924	-7.6371	0.7545	0.6931	0.0248	0.1010427	Organic
C	GH15	420.1827	5.9273	0.0327	-0.2092	0.7319	0.8208644	Organic
C	GH18	255.9295	21.9967	0.6842	0.6052	0.0422	0.1378206	Organic
C	GH19	15.6439	0.1897	0.0138	-0.2328	0.8249	0.8968955	Organic
C	GH2	452.5084	75.8562	0.901	0.8762	0.0038	0.0386307	Organic
C	GH20	103.5535	10.5091	0.5851	0.4814	0.0764	0.1890788	Organic
C	GH23	366.3567	-6.2111	0.3105	0.1381	0.2507	0.402745	Organic
C	GH24	15.7222	1.2513	0.5182	0.3977	0.1067	0.239121	Organic
C	GH25	18.1223	0.5923	0.1824	-0.022	0.3984	0.5510714	Organic
C	GH26	23.6541	1.8921	0.2269	0.0336	0.3396	0.5037171	Organic
C	GH27	104.8235	20.9783	0.8782	0.8478	0.0058	0.0424965	Organic
C	GH28	237.447	47.5986	0.8837	0.8547	0.0053	0.0410742	Organic
C	GH29	132.2116	18.2856	0.8792	0.849	0.0057	0.0424965	Organic
C	GH3	680.4412	91.7722	0.8997	0.8746	0.0039	0.0386307	Organic
C	GH30	59.6043	6.204	0.5546	0.4432	0.0894	0.212098	Organic
C	GH31	173.7587	26.5757	0.9445	0.9306	0.0012	0.0293969	Organic
C	GH33	48.5541	-0.1306	0.0007	-0.2491	0.9602	0.9640642	Organic
C	GH36	117.4663	23.4881	0.9298	0.9122	0.0019	0.0386307	Organic
C	GH32	30.1748	-2.2397	0.4348	0.2935	0.1543	0.2783221	Organic
C	GH37	45.5476	6.5165	0.6898	0.6123	0.0406	0.134882	Organic
C	GH39	110.1752	17.9437	0.8636	0.8295	0.0073	0.045597	Organic
C	GH38	153.8558	29.3236	0.7363	0.6704	0.0288	0.1155866	Organic
C	GH4	141.0485	-4.019	0.0896	-0.138	0.5644	0.6724747	Organic
C	GH42	52.5554	3.01	0.4354	0.2943	0.1539	0.2783221	Organic
C	GH43	133.3589	24.2896	0.8312	0.7891	0.0113	0.0656885	Organic
C	GH44	32.7913	5.811	0.5029	0.3787	0.1146	0.241743	Organic
C	GH45	0.5846	0.1548	0.5638	0.4548	0.0854	0.2063365	Organic
C	GH47	14.6845	0.7705	0.1758	-0.0302	0.4079	0.5611439	Organic
C	GH46	1.4741	-0.3379	0.701	0.6263	0.0376	0.1298924	Organic
C	GH48	10.4606	1.7597	0.9753	0.9691	0.0002	0.028765	Organic
C	GH49	0.0919	0.0032	0.0199	-0.2251	0.7897	0.8700517	Organic
C	GH5	172.0215	7.4157	0.4683	0.3353	0.1338	0.2622739	Organic
C	GH50	7.7274	-0.3208	0.0365	-0.2043	0.7168	0.8075638	Organic
C	GH51	136.9965	24.1589	0.9176	0.897	0.0026	0.0386307	Organic
C	GH54	33.242	9.7878	0.6984	0.6231	0.0383	0.1305093	Organic
C	GH53	65.4769	9.8763	0.7072	0.634	0.0359	0.129833	Organic
C	GH55	145.3713	28.607	0.7992	0.749	0.0163	0.0795608	Organic
C	GH56	0.0085	0.0053	0.2774	0.0967	0.283	0.4404919	Organic
C	GH57	148.2848	0.4621	0.0007	-0.2491	0.9593	0.9640642	Organic
C	GH58	0.0299	-0.0033	0.1223	-0.0971	0.4967	0.634727	Organic
C	GH59	11.8995	2.9096	0.5859	0.4824	0.0761	0.1890788	Organic
C	GH6	21.9717	3.4498	0.7172	0.6465	0.0334	0.1258917	Organic
C	GH62	3.402	0.8476	0.4845	0.3556	0.1245	0.2500438	Organic

C	GH63	18.035	3.078	0.799	0.7488	0.0163	0.0795608	Organic
C	GH64	23.3696	3.2822	0.6713	0.5891	0.046	0.1432358	Organic
C	GH65	99.333	-1.0445	0.0057	-0.2428	0.8868	0.9297832	Organic
C	GH66	1.6072	0.2261	0.1512	-0.061	0.4461	0.5908275	Organic
C	GH68	0.3029	-0.0628	0.6554	0.5692	0.0509	0.150684	Organic
C	GH7	0.8798	0.0872	0.1528	-0.059	0.4435	0.5904974	Organic
C	GH70	0.0313	0.0075	0.2185	0.0232	0.3499	0.5065066	Organic
C	GH71	4.8837	0.6597	0.1684	-0.0395	0.419	0.5715473	Organic
C	GH72	30.0891	0.8888	0.2618	0.0772	0.2995	0.4603561	Organic
C	GH73	36.2954	-0.35	0.0878	-0.1402	0.5685	0.6740461	Organic
C	GH75	2.0444	0.2783	0.5056	0.382	0.1132	0.241743	Organic
C	GH74	16.7603	2.0543	0.2587	0.0734	0.3029	0.4626382	Organic
C	GH76	18.3675	1.8865	0.3804	0.2256	0.1921	0.3299256	Organic
C	GH78	173.1756	22.1966	0.7578	0.6973	0.0241	0.099827	Organic
C	GH79	33.6982	3.1452	0.4892	0.3615	0.1219	0.2488459	Organic
C	GH8	19.2144	3.3535	0.7834	0.7293	0.019	0.0846458	Organic
C	GH80	0	0	#VALUE!	#VALUE!	#VALUE!	NA	Organic
C	GH81	5.1377	-0.3586	0.1148	-0.1065	0.5112	0.6363878	Organic
C	GH84	4.79	0.7349	0.8122	0.7653	0.0142	0.0749652	Organic
C	GH85	0.5304	0.0655	0.3758	0.2197	0.1957	0.3304563	Organic
C	GH86	1.1757	-0.2043	0.73	0.6625	0.0303	0.1163755	Organic
C	GH87	78.9746	10.7097	0.8042	0.7552	0.0154	0.0795608	Organic
C	GH88	23.1746	0.1139	0.0038	-0.2452	0.9072	0.937355	Organic
C	GH89	9.9199	2.2554	0.6941	0.6177	0.0394	0.1326932	Organic
C	GH91	0.0458	-0.0286	0.6109	0.5136	0.0663	0.1757087	Organic
C	GH90	0.0009	0.0008	0.1285	-0.0893	0.4853	0.6293328	Organic
C	GH9	60.9971	10.8052	0.9298	0.9123	0.0019	0.0386307	Organic
C	GH92	121.0279	21.6071	0.5554	0.4443	0.0891	0.212098	Organic
C	GH93	18.7765	0.9674	0.0737	-0.1578	0.6027	0.7045273	Organic
C	GH94	39.1359	9.5516	0.5997	0.4996	0.0706	0.1849473	Organic
C	GH95	57.25	8.1657	0.6803	0.6004	0.0433	0.1378206	Organic
C	GH96	0.1511	0.0299	0.2104	0.013	0.3602	0.5154507	Organic
C	GH97	30.1161	2.4644	0.9891	0.9864	0.00E+00	0.0111662	Organic
C	GH98	0.1755	-0.0891	0.7698	0.7123	0.0216	0.0921568	Organic
C	GH99	9.8752	-0.8507	0.4847	0.3558	0.1244	0.2500438	Organic
C	GT1	150.285	-13.4393	0.2882	0.1103	0.2721	0.4315001	Organic
C	GT10	3.0892	0.6179	0.5097	0.3872	0.111	0.2404138	Organic
C	GT12	0.0472	0.0136	0.4161	0.2702	0.1666	0.2963116	Organic
C	GT14	2.1246	0.3843	0.6671	0.5838	0.0473	0.1441501	Organic
C	GT11	5.7255	1.2472	0.801	0.7513	0.016	0.0795608	Organic
C	GT13	0.2735	0.1041	0.4526	0.3158	0.1431	0.2726627	Organic
C	GT15	2.516	-0.0726	0.0227	-0.2216	0.7757	0.858409	Organic
C	GT16	0.0801	0.0194	0.4576	0.3219	0.1401	0.2704451	Organic
C	GT17	1.2195	0.1219	0.3831	0.2289	0.1901	0.3287156	Organic
C	GT18	0.0298	0.0015	0.0608	-0.174	0.6375	0.7383495	Organic
C	GT2	1761.7613	13.6588	0.0168	-0.2289	0.8064	0.8845359	Organic
C	GT19	83.6411	5.2293	0.7318	0.6647	0.0298	0.1163755	Organic
C	GT20	146.1397	-3.2497	0.2073	0.0092	0.3642	0.5181798	Organic
C	GT21	93.1775	13.8497	0.9031	0.8789	0.0036	0.0386307	Organic
C	GT22	5.2116	0.0159	0.0042	-0.2448	0.9032	0.9370374	Organic
C	GT23	0.3811	0.1327	0.4986	0.3733	0.1168	0.2424575	Organic
C	GT24	3.5024	0.3825	0.2796	0.0995	0.2808	0.4397423	Organic
C	GT25	7.2869	1.0828	0.9444	0.9305	0.0012	0.0293969	Organic
C	GT26	148.5105	8.338	0.6799	0.5999	0.0435	0.1378206	Organic
C	GT27	15.1224	1.3368	0.5257	0.4071	0.103	0.2332087	Organic

C	GT28	177.4443	6.7065	0.2856	0.1069	0.2747	0.4329732	Organic
C	GT29	3.4324	-0.3628	0.2361	0.0452	0.3285	0.4927116	Organic
C	GT30	121.5074	6.0861	0.4413	0.3016	0.1501	0.2768739	Organic
C	GT3	3.9902	0.9653	0.9542	0.9427	0.0008	0.0291397	Organic
C	GT33	1.2253	-0.0763	0.452	0.3151	0.1434	0.2726627	Organic
C	GT34	1.1675	-0.0972	0.1179	-0.1027	0.5053	0.6354152	Organic
C	GT32	11.1421	-1.4401	0.8723	0.8404	0.0064	0.0442213	Organic
C	GT31	2.1893	0.518	0.4378	0.2973	0.1523	0.2783221	Organic
C	GT35	367.4633	31.6253	0.6501	0.5626	0.0526	0.150684	Organic
C	GT37	0.0155	-0.0146	0.8637	0.8296	0.0073	0.045597	Organic
C	GT39	21.5812	-0.6226	0.0544	-0.182	0.6566	0.7534117	Organic
C	GT4	1706.1942	-53.5774	0.2266	0.0333	0.3399	0.5037171	Organic
C	GT41	451.5852	68.7629	0.7295	0.6618	0.0304	0.1163755	Organic
C	GT40	0.0022	0.0015	0.2027	0.0033	0.3703	0.5209948	Organic
C	GT42	0.0544	0.0097	0.5404	0.4256	0.0959	0.2200273	Organic
C	GT43	0.1274	0.0498	0.4759	0.3448	0.1294	0.255686	Organic
C	GT47	80.6211	12.094	0.2047	0.0059	0.3677	0.520173	Organic
C	GT45	0.1867	0.002	0.0018	-0.2478	0.9367	0.9566934	Organic
C	GT44	0.0123	-0.0043	0.385	0.2312	0.1887	0.328617	Organic
C	GT48	4.9851	0.6535	0.3771	0.2214	0.1946	0.3304563	Organic
C	GT49	0.2847	0.0709	0.424	0.2799	0.1613	0.2890326	Organic
C	GT5	106.4158	0.3834	0.0028	-0.2465	0.921	0.9476688	Organic
C	GT50	1.0262	0.1479	0.3886	0.2357	0.1861	0.3263037	Organic
C	GT53	25.4939	-5.1228	0.5115	0.3894	0.1101	0.2404138	Organic
C	GT52	0	0	#VALUE!	#VALUE!	#VALUE!	NA	Organic
C	GT51	518.0831	-44.6546	0.7109	0.6386	0.035	0.129833	Organic
C	GT54	0.0816	0.0231	0.1898	-0.0127	0.3878	0.539501	Organic
C	GT57	1.9599	0.2448	0.2003	0.0004	0.3735	0.5224442	Organic
C	GT56	0.5203	0.151	0.914	0.8925	0.0029	0.0386307	Organic
C	GT55	2.6159	0.0347	0.0015	-0.2481	0.9413	0.9566934	Organic
C	GT58	0.7939	0.0852	0.2225	0.0281	0.3449	0.5052148	Organic
C	GT6	0.3853	0.0763	0.5396	0.4245	0.0963	0.2200273	Organic
C	GT59	0.5119	0.014	0.0104	-0.237	0.8473	0.9164904	Organic
C	GT60	3.4349	1.1483	0.8763	0.8454	0.006	0.042625	Organic
C	GT61	0.5101	0.1431	0.5042	0.3803	0.1139	0.241743	Organic
C	GT62	2.3868	-0.0215	0.0055	-0.2431	0.8887	0.9297832	Organic
C	GT64	0.751	-0.0295	0.13	-0.0875	0.4826	0.6291122	Organic
C	GT65	0.0811	0.0263	0.3624	0.2031	0.206	0.3420392	Organic
C	GT66	16.8699	0.1323	0.0075	-0.2406	0.8704	0.9183984	Organic
C	GT69	1.138	0.1362	0.145	-0.0687	0.4563	0.598051	Organic
C	GT68	0.0598	0.0067	0.041	-0.1987	0.7003	0.7925941	Organic
C	GT7	6.1602	0.9502	0.7793	0.7241	0.0198	0.086543	Organic
C	GT72	0.0003	1.00E-04	0.0084	-0.2395	0.8631	0.9183984	Organic
C	GT73	0.071	0.0241	0.311	0.1388	0.2502	0.402745	Organic
C	GT71	1.6293	0.2664	0.8837	0.8547	0.0053	0.0410742	Organic
C	GT70	0.7452	0.0326	0.3203	0.1504	0.2417	0.393336	Organic
C	GT74	0.8293	-0.0052	0.0008	-0.2491	0.9588	0.9640642	Organic
C	GT75	0.956	0.1365	0.3685	0.2107	0.2013	0.3363422	Organic
C	GT76	1.3751	-1.2037	0.9551	0.9439	0.0008	0.0291397	Organic
C	GT77	0.6069	-0.4006	0.9007	0.8758	0.0038	0.0386307	Organic
C	GT78	0.0028	-0.0043	0.8519	0.8149	0.0087	0.052626	Organic
C	GT8	10.5137	0.2017	0.0846	-0.1442	0.576	0.6797271	Organic
C	GT80	6.3954	0.9237	0.8215	0.7769	0.0127	0.0705009	Organic
C	GT82	0.3053	-0.0469	0.2738	0.0922	0.2868	0.4435458	Organic
C	GT84	319.0374	55.8939	0.6514	0.5643	0.0522	0.150684	Organic

C	GT81	23.6857	-0.8764	0.1065	-0.1169	0.5279	0.6469163	Organic
C	GT83	177.9561	2.9494	0.0577	-0.1779	0.6467	0.7454731	Organic
C	GT85	3.5991	-0.8261	0.4799	0.3498	0.1271	0.2532225	Organic
C	GT87	20.2064	1.036	0.1557	-0.0554	0.4389	0.5875476	Organic
C	GT88	0.0469	0.0118	0.1188	-0.1015	0.5035	0.6354152	Organic
C	GT89	5.8171	-0.9062	0.5844	0.4805	0.0767	0.1890788	Organic
C	GT90	3.3542	0.6354	0.5165	0.3957	0.1076	0.239121	Organic
C	GT9	154.7911	2.1285	0.0285	-0.2144	0.7494	0.8367464	Organic
C	GT91	0.0217	0.0004	0.0016	-0.248	0.94	0.9566934	Organic
C	GT93	0.3645	0.0048	0.0227	-0.2216	0.7755	0.858409	Organic
C	GT94	1.0193	0.2289	0.705	0.6313	0.0365	0.129833	Organic
C	GT92	0.2101	0.0354	0.3299	0.1624	0.2332	0.3820188	Organic
C	GT95	6.3578	-0.0819	0.01	-0.2375	0.8502	0.9164904	Organic
C	PL1	14.0854	1.0699	0.224	0.03	0.3431	0.5052148	Organic
C	PL10	5.2376	1.6462	0.8268	0.7836	0.012	0.0677026	Organic
C	PL11	2.8656	0.7006	0.2436	0.0546	0.3197	0.4824975	Organic
C	PL12	22.902	3.3622	0.6273	0.5341	0.0604	0.1616931	Organic
C	PL13	0.0005	0.0001	0.0084	-0.2395	0.8631	0.9183984	Organic
C	PL14	0.8538	-0.2833	0.9082	0.8853	0.0033	0.0386307	Organic
C	PL15	0.1739	0.0005	0.0001	-0.2498	0.9833	0.9832833	Organic
C	PL17	0.9434	-0.0628	0.092	-0.135	0.559	0.6724488	Organic
C	PL18	0	0	#VALUE!	#VALUE!	#VALUE!	NA	Organic
C	PL16	0.5082	0.073	0.6413	0.5517	0.0555	0.1536979	Organic
C	PL2	0.3036	0.1123	0.6421	0.5527	0.0553	0.1536979	Organic
C	PL20	0.0658	0.0097	0.216	0.02	0.353	0.5081334	Organic
C	PL21	0.5253	0.1704	0.5706	0.4633	0.0824	0.2012068	Organic
C	PL22	12.3762	2.174	0.6665	0.5831	0.0475	0.1441501	Organic
C	PL23	0.02	0.0008	0.0453	-0.1934	0.6855	0.7794373	Organic
C	PL3	0.0966	0.0122	0.0906	-0.1368	0.5621	0.6724747	Organic
C	PL4	26.4684	7.8693	0.4415	0.3018	0.15	0.2768739	Organic
C	PL5	4.576	1.7062	0.5954	0.4942	0.0723	0.1849473	Organic
C	PL6	1.1647	0.2578	0.4607	0.3258	0.1382	0.2689289	Organic
C	PL7	3.6063	0.6117	0.4942	0.3677	0.1192	0.2453652	Organic
C	PL8	3.0069	0.8493	0.8855	0.8569	0.0051	0.0410742	Organic
C	PL9	28.8683	4.8731	0.9006	0.8758	0.0038	0.0386307	Organic
N	AA1	179.0321	7.8263	0.2497	0.0621	0.3129	0.4930689	Organic
N	AA11	0.5025	0.0406	0.0475	-0.1906	0.6783	0.7855796	Organic
N	AA10	7.5616	1.0248	0.437	0.2963	0.1528	0.3280446	Organic
N	AA2	1.8202	0.1005	0.0408	-0.199	0.701	0.7970467	Organic
N	AA3	383.3947	-44.0924	0.7172	0.6465	0.0334	0.1661582	Organic
N	AA4	20.7999	5.7776	0.5959	0.4949	0.0721	0.2361256	Organic
N	AA5	32.5624	-11.3958	0.882	0.8525	0.0054	0.1043096	Organic
N	AA8	0.8225	0.05	0.0671	-0.1661	0.62	0.7351734	Organic
N	AA7	52.2765	-5.7963	0.4185	0.2731	0.165	0.3372548	Organic
N	AA6	24.3337	-4.1836	0.6337	0.5421	0.0582	0.2011082	Organic
N	AA9	1.8306	0.1297	0.0779	-0.1526	0.5922	0.7336446	Organic
N	CE1	208.9789	-3.9059	0.2871	0.1089	0.2732	0.459711	Organic
N	CE11	155.7435	6.6329	0.2119	0.0149	0.3583	0.5413697	Organic
N	CE12	20.9813	3.613	0.4816	0.352	0.1262	0.3032791	Organic
N	CE13	0.0653	0.0074	0.02	-0.225	0.7893	0.8582623	Organic
N	CE14	227.4441	15.3114	0.6206	0.5258	0.0628	0.2111927	Organic
N	CE15	52.1006	10.807	0.8068	0.7585	0.015	0.1553182	Organic
N	CE2	3.239	0.6807	0.501	0.3762	0.1156	0.2967458	Organic
N	CE3	15.1515	-0.8816	0.0855	-0.1431	0.5738	0.7162023	Organic
N	CE4	294.1051	-57.2367	0.8898	0.8623	0.0047	0.1043096	Organic

N	CE16	1.0735	0.053	0.073	-0.1588	0.6047	0.7351734	Organic
N	CE5	14.9063	-4.0602	0.5661	0.4576	0.0844	0.255282	Organic
N	CE7	12.9532	2.0655	0.6877	0.6096	0.0412	0.1745182	Organic
N	CE6	52.0839	10.6486	0.9427	0.9284	0.0013	0.0780677	Organic
N	CE8	24.9683	3.9886	0.753	0.6913	0.0251	0.1558156	Organic
N	CE9	90.7663	0.3651	0.0074	-0.2408	0.8715	0.9156091	Organic
N	GH100	1.1884	0.1742	0.0373	-0.2034	0.7139	0.8079862	Organic
N	GH10	59.1017	7.3477	0.8673	0.8341	0.0069	0.1230699	Organic
N	GH102	34.7782	-2.4554	0.7557	0.6946	0.0245	0.1558156	Organic
N	GH101	0.4319	-0.0582	0.5125	0.3906	0.1096	0.284289	Organic
N	GH103	87.9513	-15.6466	0.7098	0.6372	0.0353	0.1688537	Organic
N	GH104	4.2879	-0.5115	0.5147	0.3933	0.1085	0.284289	Organic
N	GH105	39.1132	9.4907	0.7567	0.6959	0.0243	0.1558156	Organic
N	GH106	64.736	12.9261	0.6985	0.6232	0.0382	0.1733225	Organic
N	GH107	0.05	-0.02	0.6232	0.5289	0.0619	0.2109913	Organic
N	GH108	6.1612	0.515	0.4181	0.2727	0.1652	0.3372548	Organic
N	GH109	20.7365	2.2281	0.7795	0.7244	0.0198	0.1558156	Organic
N	GH11	18.0145	3.511	0.6958	0.6197	0.039	0.1733225	Organic
N	GH110	1.6372	0.1882	0.1474	-0.0657	0.4523	0.614337	Organic
N	GH111	0.0241	0.0037	0.1606	-0.0492	0.431	0.602519	Organic
N	GH112	0.1865	0.0862	0.3379	0.1723	0.2263	0.4143421	Organic
N	GH113	7.4538	0.2402	0.0344	-0.2071	0.7251	0.8170189	Organic
N	GH114	8.4827	-3.5779	0.9341	0.9176	0.0017	0.0829529	Organic
N	GH115	26.0154	8.2681	0.6417	0.5521	0.0554	0.1956414	Organic
N	GH116	54.6915	5.7889	0.4339	0.2924	0.1548	0.3295032	Organic
N	GH117	3.207	0.5015	0.7747	0.7183	0.0207	0.1558156	Organic
N	GH118	0.0019	0.0012	0.1485	-0.0643	0.4505	0.614337	Organic
N	GH119	1.7272	0.1234	0.0711	-0.1611	0.6095	0.7351734	Organic
N	GH12	10.5185	2.3958	0.7014	0.6267	0.0375	0.1733225	Organic
N	GH120	2.0347	0.5077	0.6457	0.5571	0.0541	0.1956414	Organic
N	GH121	0.74	-0.0004	0.00E+00	-0.25	0.9957	0.9978793	Organic
N	GH123	0.8792	-0.8264	0.9251	0.9064	0.0022	0.0877269	Organic
N	GH124	0.0228	-0.037	0.8996	0.8744	0.0039	0.1043096	Organic
N	GH125	14.4186	0.99	0.0849	-0.1439	0.5753	0.7162023	Organic
N	GH126	0.0126	0.0037	0.1064	-0.117	0.5281	0.6743919	Organic
N	GH127	53.4987	9.858	0.8414	0.8018	0.01	0.1511912	Organic
N	GH128	9.2104	1.5379	0.4669	0.3336	0.1346	0.3087327	Organic
N	GH129	0.4364	0.1107	0.4942	0.3678	0.1192	0.2972876	Organic
N	GH13	1477.2569	-28.8614	0.1747	-0.0316	0.4096	0.5801673	Organic
N	GH130	95.5733	8.9964	0.3659	0.2074	0.2033	0.3867476	Organic
N	GH131	0.252	0.0246	0.0717	-0.1603	0.6078	0.7351734	Organic
N	GH132	0.9233	0.0004	0.00E+00	-0.25	0.9979	0.9978793	Organic
N	GH133	59.9767	-9.0545	0.8104	0.763	0.0144	0.1553182	Organic
N	GH14	0.6765	0.0316	0.0279	-0.2151	0.7518	0.8283551	Organic
N	GH17	51.2924	-6.4332	0.5354	0.4192	0.0983	0.2632409	Organic
N	GH15	420.1827	7.4795	0.052	-0.185	0.6638	0.7724101	Organic
N	GH18	255.9295	18.7697	0.4982	0.3727	0.1171	0.2972876	Organic
N	GH19	15.6439	0.0804	0.0025	-0.2469	0.9255	0.9598673	Organic
N	GH2	452.5084	66.1483	0.6851	0.6064	0.042	0.1745182	Organic
N	GH20	103.5535	9.1345	0.4421	0.3026	0.1496	0.3268464	Organic
N	GH23	366.3567	-7.2675	0.425	0.2813	0.1606	0.3345532	Organic
N	GH24	15.7222	0.8853	0.2593	0.0742	0.3021	0.4791994	Organic
N	GH25	18.1223	0.6127	0.1952	-0.006	0.3804	0.5600597	Organic
N	GH26	23.6541	1.8923	0.2269	0.0336	0.3395	0.5317146	Organic
N	GH27	104.8235	18.8889	0.712	0.64	0.0347	0.1688537	Organic

N	GH28	237.447	41.5729	0.6742	0.5927	0.0452	0.1757024	Organic
N	GH29	132.2116	15.9061	0.6653	0.5816	0.0479	0.1805458	Organic
N	GH3	680.4412	85.2519	0.7764	0.7205	0.0204	0.1558156	Organic
N	GH30	59.6043	6.3363	0.5785	0.4731	0.0791	0.2525515	Organic
N	GH31	173.7587	24.5081	0.8032	0.754	0.0156	0.1553182	Organic
N	GH33	48.5541	-1.3428	0.0745	-0.1569	0.6007	0.7351734	Organic
N	GH36	117.4663	21.2424	0.7605	0.7006	0.0235	0.1558156	Organic
N	GH32	30.1748	-2.8341	0.6963	0.6203	0.0389	0.1733225	Organic
N	GH37	45.5476	6.6455	0.7174	0.6468	0.0333	0.1661582	Organic
N	GH39	110.1752	15.4551	0.6406	0.5508	0.0558	0.1956414	Organic
N	GH38	153.8558	25.8368	0.5716	0.4645	0.082	0.2546028	Organic
N	GH4	141.0485	-5.8288	0.1884	-0.0145	0.3898	0.5642508	Organic
N	GH42	52.5554	2.3412	0.2634	0.0793	0.2977	0.4791994	Organic
N	GH43	133.3589	22.7163	0.7271	0.6588	0.031	0.1627813	Organic
N	GH44	32.7913	4.4885	0.3001	0.1251	0.2605	0.4504574	Organic
N	GH45	0.5846	0.139	0.4546	0.3182	0.1419	0.3126875	Organic
N	GH47	14.6845	0.7673	0.1743	-0.0321	0.4101	0.5801673	Organic
N	GH46	1.4741	-0.3317	0.6758	0.5948	0.0447	0.1757024	Organic
N	GH48	10.4606	1.649	0.8565	0.8206	0.0081	0.1349093	Organic
N	GH49	0.0919	0.0086	0.141	-0.0737	0.4632	0.614337	Organic
N	GH5	172.0215	5.7566	0.2822	0.1027	0.2781	0.459711	Organic
N	GH50	7.7274	-0.435	0.0672	-0.166	0.6199	0.7351734	Organic
N	GH51	136.9965	21.7254	0.7421	0.6776	0.0275	0.1593411	Organic
N	GH54	33.242	10.2433	0.765	0.7062	0.0226	0.1558156	Organic
N	GH53	65.4769	8.7924	0.5605	0.4506	0.0868	0.255282	Organic
N	GH55	145.3713	27.7494	0.752	0.69	0.0253	0.1558156	Organic
N	GH56	0.0085	0.0033	0.1075	-0.1157	0.5259	0.6743919	Organic
N	GH57	148.2848	-3.0925	0.033	-0.2087	0.7304	0.8192171	Organic
N	GH58	0.0299	-0.0054	0.3276	0.1595	0.2352	0.4274528	Organic
N	GH59	11.8995	3.2379	0.7256	0.657	0.0313	0.1627813	Organic
N	GH6	21.9717	3.3578	0.6795	0.5993	0.0436	0.1750984	Organic
N	GH62	3.402	0.7541	0.3835	0.2294	0.1898	0.3711055	Organic
N	GH63	18.035	2.78	0.6518	0.5648	0.0521	0.1936215	Organic
N	GH64	23.3696	2.9665	0.5483	0.4354	0.0923	0.255282	Organic
N	GH65	99.333	0.3214	0.0005	-0.2493	0.9651	0.9848745	Organic
N	GH66	1.6072	0.0887	0.0233	-0.2209	0.773	0.8479256	Organic
N	GH68	0.3029	-0.0679	0.7646	0.7058	0.0227	0.1558156	Organic
N	GH7	0.8798	0.0836	0.1405	-0.0743	0.464	0.614337	Organic
N	GH70	0.0313	0.0064	0.1588	-0.0515	0.434	0.602519	Organic
N	GH71	4.8837	0.6768	0.1772	-0.0285	0.4058	0.5801673	Organic
N	GH72	30.0891	0.6645	0.1463	-0.0671	0.4542	0.614337	Organic
N	GH73	36.2954	-0.4691	0.1578	-0.0528	0.4356	0.602519	Organic
N	GH75	2.0444	0.3242	0.6858	0.6073	0.0418	0.1745182	Organic
N	GH74	16.7603	1.8577	0.2115	0.0144	0.3587	0.5413697	Organic
N	GH76	18.3675	2.0029	0.4289	0.2861	0.1581	0.33364	Organic
N	GH78	173.1756	19.3338	0.575	0.4687	0.0806	0.2540183	Organic
N	GH79	33.6982	3.1176	0.4807	0.3508	0.1267	0.3032791	Organic
N	GH8	19.2144	2.9729	0.6157	0.5196	0.0646	0.2143432	Organic
N	GH80	0	0	#VALUE!	#VALUE!	#VALUE!	NA	Organic
N	GH81	5.1377	-0.5827	0.3032	0.129	0.2576	0.4504574	Organic
N	GH84	4.79	0.6099	0.5594	0.4493	0.0873	0.255282	Organic
N	GH85	0.5304	0.0438	0.1685	-0.0394	0.4189	0.5892741	Organic
N	GH86	1.1757	-0.2071	0.7503	0.6879	0.0257	0.1558156	Organic
N	GH87	78.9746	9.8829	0.6848	0.606	0.0421	0.1745182	Organic
N	GH88	23.1746	-0.3177	0.0298	-0.2127	0.7436	0.8265361	Organic

N	GH89	9.9199	2.2325	0.6801	0.6001	0.0434	0.1750984	Organic
N	GH91	0.0458	-0.0327	0.7986	0.7482	0.0164	0.1558156	Organic
N	GH90	0.0009	0.0003	0.0159	-0.2301	0.8116	0.8648047	Organic
N	GH9	60.9971	10.2349	0.8343	0.7928	0.0109	0.1511912	Organic
N	GH92	121.0279	19.9944	0.4756	0.3445	0.1295	0.3072088	Organic
N	GH93	18.7765	1.2646	0.126	-0.0925	0.4898	0.6385949	Organic
N	GH94	39.1359	9.3095	0.5697	0.4621	0.0828	0.2546028	Organic
N	GH95	57.25	6.774	0.4682	0.3352	0.1338	0.3087327	Organic
N	GH96	0.1511	0.0179	0.0755	-0.1556	0.5981	0.7351734	Organic
N	GH97	30.1161	2.3618	0.9085	0.8856	0.0032	0.1009418	Organic
N	GH98	0.1755	-0.088	0.7508	0.6885	0.0255	0.1558156	Organic
N	GH99	9.8752	-0.9994	0.6689	0.5862	0.0467	0.1790132	Organic
N	GT1	150.285	-13.2827	0.2815	0.1019	0.2788	0.459711	Organic
N	GT10	3.0892	0.4457	0.2652	0.0815	0.2958	0.4791994	Organic
N	GT12	0.0472	0.0156	0.5496	0.437	0.0917	0.255282	Organic
N	GT14	2.1246	0.3452	0.5382	0.4227	0.097	0.2625123	Organic
N	GT11	5.7255	1.1163	0.6417	0.5521	0.0554	0.1956414	Organic
N	GT13	0.2735	0.0848	0.3009	0.1261	0.2597	0.4504574	Organic
N	GT15	2.516	-0.0484	0.0101	-0.2374	0.8497	0.8965135	Organic
N	GT16	0.0801	0.0131	0.2089	0.0111	0.3622	0.5433271	Organic
N	GT17	1.2195	0.1075	0.2978	0.1223	0.2626	0.4510319	Organic
N	GT18	0.0298	0.0024	0.1442	-0.0698	0.4578	0.614337	Organic
N	GT2	1761.7613	-4.9826	0.0022	-0.2472	0.929	0.9598673	Organic
N	GT19	83.6411	4.2614	0.4859	0.3574	0.1237	0.302039	Organic
N	GT20	146.1397	-2.2552	0.0999	-0.1252	0.5418	0.6882692	Organic
N	GT21	93.1775	12.4118	0.7253	0.6566	0.0314	0.1627813	Organic
N	GT22	5.2116	-0.0302	0.0152	-0.231	0.8162	0.8648047	Organic
N	GT23	0.3811	0.0978	0.2709	0.0886	0.2898	0.4746816	Organic
N	GT24	3.5024	0.2699	0.1392	-0.076	0.4663	0.614337	Organic
N	GT25	7.2869	1.0687	0.92	0.9	0.0025	0.0877269	Organic
N	GT26	148.5105	6.4097	0.4018	0.2522	0.1765	0.3544906	Organic
N	GT27	15.1224	0.981	0.2831	0.1038	0.2772	0.459711	Organic
N	GT28	177.4443	4.7365	0.1424	-0.072	0.4608	0.614337	Organic
N	GT29	3.4324	-0.4663	0.3899	0.2374	0.1851	0.3686927	Organic
N	GT30	121.5074	4.2885	0.2191	0.0239	0.3491	0.5366283	Organic
N	GT3	3.9902	0.9281	0.882	0.8524	0.0054	0.1043096	Organic
N	GT33	1.2253	-0.0817	0.5185	0.3981	0.1066	0.2823133	Organic
N	GT34	1.1675	-0.0866	0.0935	-0.1331	0.5556	0.6987415	Organic
N	GT32	11.1421	-1.3406	0.7559	0.6949	0.0245	0.1558156	Organic
N	GT31	2.1893	0.4184	0.2857	0.1071	0.2746	0.459711	Organic
N	GT35	367.4633	26.5654	0.4587	0.3234	0.1394	0.312201	Organic
N	GT37	0.0155	-0.0142	0.8207	0.7759	0.0129	0.1553182	Organic
N	GT39	21.5812	-0.3818	0.0204	-0.2244	0.787	0.8582623	Organic
N	GT4	1706.1942	-66.2268	0.3463	0.1828	0.2192	0.4073257	Organic
N	GT41	451.5852	54.9536	0.4659	0.3324	0.1351	0.3087327	Organic
N	GT40	0.0022	0.0007	0.0422	-0.1972	0.6961	0.7970467	Organic
N	GT42	0.0544	0.0078	0.3489	0.1862	0.217	0.4062498	Organic
N	GT43	0.1274	0.0341	0.223	0.0288	0.3443	0.5324226	Organic
N	GT47	80.6211	7.0021	0.0686	-0.1642	0.6161	0.7351734	Organic
N	GT45	0.1867	-0.0032	0.0046	-0.2443	0.8985	0.9361346	Organic
N	GT44	0.0123	-0.0037	0.2902	0.1127	0.2702	0.459711	Organic
N	GT48	4.9851	0.6485	0.3714	0.2143	0.199	0.3841573	Organic
N	GT49	0.2847	0.0696	0.4089	0.2611	0.1716	0.347326	Organic
N	GT5	106.4158	-0.963	0.0175	-0.2281	0.8026	0.8648047	Organic
N	GT50	1.0262	0.1304	0.302	0.1275	0.2586	0.4504574	Organic

N	GT53	25.4939	-4.7406	0.4381	0.2976	0.1522	0.3280446	Organic
N	GT52	0	0	#VALUE!	#VALUE!	#VALUE!	NA	Organic
N	GT51	518.0831	-47.7914	0.8142	0.7678	0.0138	0.1553182	Organic
N	GT54	0.0816	0.0181	0.1164	-0.1044	0.508	0.6554113	Organic
N	GT57	1.9599	0.2335	0.1823	-0.0221	0.3984	0.5734374	Organic
N	GT56	0.5203	0.1484	0.8831	0.8539	0.0053	0.1043096	Organic
N	GT55	2.6159	0.1827	0.0424	-0.197	0.6954	0.7970467	Organic
N	GT58	0.7939	0.0802	0.1973	-0.0034	0.3775	0.5595478	Organic
N	GT6	0.3853	0.0587	0.3201	0.1502	0.2419	0.4364139	Organic
N	GT59	0.5119	0.0175	0.0163	-0.2296	0.8095	0.8648047	Organic
N	GT60	3.4349	1.1044	0.8105	0.7631	0.0144	0.1553182	Organic
N	GT61	0.5101	0.1029	0.2608	0.076	0.3006	0.4791994	Organic
N	GT62	2.3868	0.0228	0.0062	-0.2422	0.8818	0.9225588	Organic
N	GT64	0.751	-0.0448	0.3005	0.1257	0.26	0.4504574	Organic
N	GT65	0.0811	0.0223	0.2609	0.0762	0.3004	0.4791994	Organic
N	GT66	16.8699	-0.2086	0.0186	-0.2267	0.7965	0.8623294	Organic
N	GT69	1.138	0.1655	0.2141	0.0177	0.3554	0.5413697	Organic
N	GT68	0.0598	1.00E-04	0.00E+00	-0.25	0.997	0.9978793	Organic
N	GT7	6.1602	0.794	0.5441	0.4302	0.0942	0.2577919	Organic
N	GT72	0.0003	0.0002	0.0573	-0.1784	0.6478	0.760874	Organic
N	GT73	0.071	0.0282	0.4241	0.2802	0.1612	0.3345532	Organic
N	GT71	1.6293	0.2571	0.8232	0.779	0.0125	0.1553182	Organic
N	GT70	0.7452	0.0337	0.3422	0.1778	0.2226	0.4106093	Organic
N	GT74	0.8293	-0.0237	0.0155	-0.2306	0.8141	0.8648047	Organic
N	GT75	0.956	0.1235	0.3016	0.127	0.2591	0.4504574	Organic
N	GT76	1.3751	-1.2258	0.9905	0.9881	0.00E+00	0.0084881	Organic
N	GT77	0.6069	-0.4111	0.9485	0.9356	0.001	0.0780677	Organic
N	GT78	0.0028	-0.004	0.76	0.7	0.0236	0.1558156	Organic
N	GT8	10.5137	0.1201	0.03	-0.2125	0.7427	0.8265361	Organic
N	GT80	6.3954	0.8858	0.7554	0.6943	0.0246	0.1558156	Organic
N	GT82	0.3053	-0.0558	0.3865	0.2331	0.1876	0.3707405	Organic
N	GT84	319.0374	47.0339	0.4613	0.3266	0.1379	0.3121212	Organic
N	GT81	23.6857	-0.5466	0.0414	-0.1982	0.699	0.7970467	Organic
N	GT83	177.9561	0.208	0.0003	-0.2496	0.9746	0.9905038	Organic
N	GT85	3.5991	-0.8332	0.4882	0.3602	0.1225	0.3020363	Organic
N	GT87	20.2064	1.1559	0.1938	-0.0078	0.3824	0.5600597	Organic
N	GT88	0.0469	0.0106	0.0948	-0.1315	0.5528	0.6986821	Organic
N	GT89	5.8171	-0.8882	0.5614	0.4518	0.0864	0.255282	Organic
N	GT90	3.3542	0.5977	0.457	0.3213	0.1404	0.312201	Organic
N	GT9	154.7911	-0.4351	0.0012	-0.2485	0.9483	0.9717146	Organic
N	GT91	0.0217	0.0026	0.0544	-0.182	0.6565	0.7674522	Organic
N	GT93	0.3645	0.0084	0.0682	-0.1647	0.6171	0.7351734	Organic
N	GT94	1.0193	0.2021	0.5496	0.437	0.0917	0.255282	Organic
N	GT92	0.2101	0.028	0.2066	0.0082	0.3652	0.5445341	Organic
N	GT95	6.3578	-0.1388	0.0288	-0.214	0.7478	0.8275774	Organic
N	PL1	14.0854	0.8457	0.14	-0.075	0.465	0.614337	Organic
N	PL10	5.2376	1.5593	0.7419	0.6773	0.0275	0.1593411	Organic
N	PL11	2.8656	0.6183	0.1898	-0.0128	0.3879	0.5642508	Organic
N	PL12	22.902	2.9834	0.4939	0.3673	0.1194	0.2972876	Organic
N	PL13	0.0005	0.0003	0.0573	-0.1784	0.6478	0.760874	Organic
N	PL14	0.8538	-0.2894	0.9474	0.9343	0.0011	0.0780677	Organic
N	PL15	0.1739	-0.0003	0.00E+00	-0.2499	0.9895	0.9978793	Organic
N	PL17	0.9434	-0.0711	0.118	-0.1024	0.5049	0.6548178	Organic
N	PL18	0	0	#VALUE!	#VALUE!	#VALUE!	NA	Organic
N	PL16	0.5082	0.0676	0.5504	0.438	0.0913	0.255282	Organic

N	PL2	0.3036	0.0866	0.3823	0.2278	0.1908	0.3711055	Organic
N	PL20	0.0658	0.0125	0.3546	0.1932	0.2124	0.4006316	Organic
N	PL21	0.5253	0.1937	0.7369	0.6711	0.0286	0.1620936	Organic
N	PL22	12.3762	1.8303	0.4724	0.3405	0.1314	0.3085631	Organic
N	PL23	0.02	-0.0002	0.0016	-0.248	0.9393	0.9664878	Organic
N	PL3	0.0966	0.0149	0.1349	-0.0814	0.4739	0.6210708	Organic
N	PL4	26.4684	5.6078	0.2242	0.0302	0.3428	0.5324226	Organic
N	PL5	4.576	1.6385	0.5491	0.4364	0.0919	0.255282	Organic
N	PL6	1.1647	0.2297	0.3657	0.2071	0.2035	0.3867476	Organic
N	PL7	3.6063	0.6674	0.5883	0.4854	0.0751	0.2428261	Organic
N	PL8	3.0069	0.7722	0.7322	0.6652	0.0297	0.1627813	Organic
N	PL9	28.8683	4.6914	0.8347	0.7934	0.0109	0.1511912	Organic
CN	AA1	179.0321	12.2973	0.6164	0.5205	0.0643	0.1633655	Organic
CN	AA11	0.5025	0.0021	0.0001	-0.2498	0.9833	0.9872894	Organic
CN	AA10	7.5616	1.3274	0.7332	0.6665	0.0295	0.1034513	Organic
CN	AA2	1.8202	0.2442	0.2411	0.0514	0.3226	0.4782073	Organic
CN	AA3	383.3947	-49.4466	0.902	0.8775	0.0037	0.0421659	Organic
CN	AA4	20.7999	4.9345	0.4347	0.2934	0.1543	0.288922	Organic
CN	AA5	32.5624	-11.1453	0.8436	0.8045	0.0097	0.0654915	Organic
CN	AA8	0.8225	0.072	0.1391	-0.0761	0.4665	0.5896579	Organic
CN	AA7	52.2765	-7.1765	0.6415	0.5519	0.0555	0.1485932	Organic
CN	AA6	24.3337	-4.6421	0.7802	0.7253	0.0196	0.0788558	Organic
CN	AA9	1.8306	0.1757	0.143	-0.0712	0.4598	0.5871181	Organic
CN	CE1	208.9789	-1.1076	0.0231	-0.2211	0.7738	0.8414201	Organic
CN	CE11	155.7435	10.0288	0.4845	0.3556	0.1245	0.2501011	Organic
CN	CE12	20.9813	4.2234	0.658	0.5726	0.0501	0.1388903	Organic
CN	CE13	0.0653	0.0245	0.2193	0.0242	0.3489	0.496368	Organic
CN	CE14	227.4441	15.9137	0.6704	0.588	0.0463	0.1355737	Organic
CN	CE15	52.1006	11.8359	0.9678	0.9597	0.0004	0.0327196	Organic
CN	CE2	3.239	0.7661	0.6346	0.5433	0.0578	0.1523839	Organic
CN	CE3	15.1515	-1.8984	0.3966	0.2458	0.1802	0.3160011	Organic
CN	CE4	294.1051	-57.4721	0.8972	0.8715	0.0041	0.0426283	Organic
CN	CE16	1.0735	0.0422	0.0464	-0.192	0.6818	0.7932804	Organic
CN	CE5	14.9063	-4.1372	0.5878	0.4847	0.0753	0.1786003	Organic
CN	CE7	12.9532	2.2651	0.827	0.7838	0.0119	0.0654915	Organic
CN	CE6	52.0839	9.9059	0.8158	0.7698	0.0136	0.0677013	Organic
CN	CE8	24.9683	4.3845	0.9099	0.8874	0.0031	0.0421659	Organic
CN	CE9	90.7663	2.1784	0.2626	0.0782	0.2986	0.4618495	Organic
CN	GH100	1.1884	0.4624	0.263	0.0787	0.2982	0.4618495	Organic
CN	GH10	59.1017	7.4969	0.9029	0.8786	0.0037	0.0421659	Organic
CN	GH102	34.7782	-2.4813	0.7717	0.7146	0.0213	0.0840524	Organic
CN	GH101	0.4319	-0.0709	0.7603	0.7003	0.0236	0.0902267	Organic
CN	GH103	87.9513	-18.0898	0.9488	0.9359	0.001	0.0334192	Organic
CN	GH104	4.2879	-0.3781	0.2812	0.1015	0.2791	0.4454935	Organic
CN	GH105	39.1132	9.2562	0.7198	0.6497	0.0327	0.1131979	Organic
CN	GH106	64.736	14.5596	0.8863	0.8578	0.005	0.0471306	Organic
CN	GH107	0.05	-0.0164	0.4177	0.2721	0.1655	0.3030483	Organic
CN	GH108	6.1612	0.413	0.2689	0.0861	0.2919	0.4600775	Organic
CN	GH109	20.7365	2.5159	0.9938	0.9923	0.00E+00	0.0035421	Organic
CN	GH11	18.0145	3.8733	0.8468	0.8085	0.0093	0.0654915	Organic
CN	GH110	1.6372	0.2422	0.2442	0.0552	0.3191	0.4757921	Organic
CN	GH111	0.0241	0.0024	0.0707	-0.1616	0.6104	0.7307591	Organic
CN	GH112	0.1865	0.116	0.6119	0.5148	0.066	0.1659525	Organic
CN	GH113	7.4538	0.3604	0.0774	-0.1533	0.5935	0.7173887	Organic
CN	GH114	8.4827	-3.365	0.8262	0.7828	0.012	0.0654915	Organic

CN	GH115	26.0154	9.9024	0.9204	0.9005	0.0024	0.0379807	Organic
CN	GH116	54.6915	7.6939	0.7665	0.7081	0.0223	0.0867109	Organic
CN	GH117	3.207	0.4892	0.7371	0.6713	0.0286	0.1017645	Organic
CN	GH118	0.0019	0.0003	0.0088	-0.239	0.8598	0.9033245	Organic
CN	GH119	1.7272	0.1613	0.1215	-0.0982	0.4984	0.6158169	Organic
CN	GH12	10.5185	2.5358	0.7857	0.7321	0.0186	0.0786072	Organic
CN	GH120	2.0347	0.3326	0.2772	0.0965	0.2832	0.4491911	Organic
CN	GH121	0.74	-0.0234	0.0261	-0.2174	0.7598	0.8359988	Organic
CN	GH123	0.8792	-0.7638	0.7903	0.7379	0.0178	0.0786072	Organic
CN	GH124	0.0228	-0.0354	0.8233	0.7791	0.0125	0.0654915	Organic
CN	GH125	14.4186	1.6592	0.2385	0.0481	0.3257	0.4798327	Organic
CN	GH126	0.0126	-0.0002	0.0003	-0.2496	0.9749	0.9849884	Organic
CN	GH127	53.4987	9.8645	0.8425	0.8032	0.0098	0.0654915	Organic
CN	GH128	9.2104	0.9648	0.1837	-0.0203	0.3964	0.5393959	Organic
CN	GH129	0.4364	0.1214	0.595	0.4938	0.0724	0.1734297	Organic
CN	GH13	1477.2569	-21.0293	0.0927	-0.1341	0.5573	0.6769224	Organic
CN	GH130	95.5733	11.4802	0.5959	0.4949	0.0721	0.1734297	Organic
CN	GH131	0.252	0.0383	0.1736	-0.033	0.4111	0.5504029	Organic
CN	GH132	0.9233	-0.0151	0.0037	-0.2453	0.9085	0.9439687	Organic
CN	GH133	59.9767	-7.5567	0.5645	0.4556	0.0851	0.1891406	Organic
CN	GH14	0.6765	0.0875	0.2134	0.0167	0.3564	0.5013567	Organic
CN	GH17	51.2924	-7.9465	0.8169	0.7711	0.0134	0.0677013	Organic
CN	GH15	420.1827	6.3099	0.037	-0.2037	0.715	0.8055482	Organic
CN	GH18	255.9295	23.5061	0.7813	0.7266	0.0194	0.0788558	Organic
CN	GH19	15.6439	0.2751	0.0289	-0.2138	0.7473	0.8306972	Organic
CN	GH2	452.5084	78.0142	0.953	0.9412	0.0008	0.0334192	Organic
CN	GH20	103.5535	11.0794	0.6504	0.5629	0.0526	0.1422809	Organic
CN	GH23	366.3567	-5.0965	0.209	0.0113	0.362	0.5063795	Organic
CN	GH24	15.7222	1.4321	0.6786	0.5983	0.0438	0.1305186	Organic
CN	GH25	18.1223	0.5288	0.1454	-0.0683	0.4558	0.584985	Organic
CN	GH26	23.6541	1.7484	0.1937	-0.0079	0.3824	0.5287064	Organic
CN	GH27	104.8235	21.3908	0.9131	0.8914	0.0029	0.0421659	Organic
CN	GH28	237.447	48.8194	0.9297	0.9121	0.0019	0.0338045	Organic
CN	GH29	132.2116	18.7408	0.9235	0.9044	0.0023	0.0374096	Organic
CN	GH3	680.4412	91.746	0.8992	0.874	0.0039	0.0426283	Organic
CN	GH30	59.6043	5.9696	0.5135	0.3918	0.1091	0.2283084	Organic
CN	GH31	173.7587	26.4939	0.9387	0.9233	0.0014	0.0334192	Organic
CN	GH33	48.5541	0.6306	0.0164	-0.2295	0.8088	0.8755778	Organic
CN	GH36	117.4663	24.2141	0.9882	0.9852	1.00E-04	0.0065782	Organic
CN	GH32	30.1748	-1.621	0.2278	0.0347	0.3385	0.4871553	Organic
CN	GH37	45.5476	6.3652	0.6582	0.5727	0.0501	0.1388903	Organic
CN	GH39	110.1752	18.7594	0.9439	0.9298	0.0012	0.0334192	Organic
CN	GH38	153.8558	30.6186	0.8028	0.7535	0.0157	0.0750237	Organic
CN	GH4	141.0485	-2.1807	0.0264	-0.217	0.7585	0.8359988	Organic
CN	GH42	52.5554	3.2918	0.5208	0.401	0.1054	0.2224862	Organic
CN	GH43	133.3589	24.1927	0.8246	0.7808	0.0123	0.0654915	Organic
CN	GH44	32.7913	6.1727	0.5675	0.4594	0.0838	0.1878962	Organic
CN	GH45	0.5846	0.1456	0.4991	0.3739	0.1166	0.2399227	Organic
CN	GH47	14.6845	0.8038	0.1913	-0.0108	0.3857	0.5287064	Organic
CN	GH46	1.4741	-0.3212	0.6337	0.5422	0.0581	0.1523839	Organic
CN	GH48	10.4606	1.7255	0.9378	0.9223	0.0015	0.0334192	Organic
CN	GH49	0.0919	-0.0001	0.00E+00	-0.25	0.9928	0.9927673	Organic
CN	GH5	172.0215	8.2052	0.5733	0.4666	0.0813	0.185757	Organic
CN	GH50	7.7274	-0.3289	0.0384	-0.202	0.7098	0.8051852	Organic
CN	GH51	136.9965	24.3547	0.9326	0.9157	0.0017	0.0334192	Organic

CN	GH54	33.242	9.0997	0.6037	0.5046	0.0691	0.170271	Organic
CN	GH53	65.4769	9.9274	0.7146	0.6432	0.034	0.1161155	Organic
CN	GH55	145.3713	28.3647	0.7857	0.7321	0.0186	0.0786072	Organic
CN	GH56	0.0085	0.0065	0.4139	0.2673	0.1681	0.3033888	Organic
CN	GH57	148.2848	2.7215	0.0256	-0.218	0.7621	0.8359988	Organic
CN	GH58	0.0299	-0.0018	0.0353	-0.2058	0.7214	0.8091511	Organic
CN	GH59	11.8995	2.5581	0.4529	0.3161	0.1429	0.2737408	Organic
CN	GH6	21.9717	3.3663	0.6829	0.6037	0.0426	0.1305186	Organic
CN	GH62	3.402	0.8076	0.4399	0.2998	0.151	0.2849131	Organic
CN	GH63	18.035	3.1238	0.823	0.7787	0.0125	0.0654915	Organic
CN	GH64	23.3696	3.2986	0.678	0.5975	0.044	0.1305186	Organic
CN	GH65	99.333	-1.6274	0.0139	-0.2326	0.824	0.8769325	Organic
CN	GH66	1.6072	0.2885	0.2462	0.0577	0.3168	0.4752068	Organic
CN	GH68	0.3029	-0.0577	0.553	0.4412	0.0902	0.1969406	Organic
CN	GH7	0.8798	0.0933	0.1749	-0.0314	0.4093	0.5504029	Organic
CN	GH70	0.0313	0.0067	0.1754	-0.0307	0.4085	0.5504029	Organic
CN	GH71	4.8837	0.6327	0.1549	-0.0564	0.4402	0.5727944	Organic
CN	GH72	30.0891	0.9823	0.3198	0.1497	0.2422	0.3916118	Organic
CN	GH73	36.2954	-0.2316	0.0385	-0.2019	0.7096	0.8051852	Organic
CN	GH75	2.0444	0.2363	0.3643	0.2054	0.2046	0.346522	Organic
CN	GH74	16.7603	1.9143	0.2246	0.0308	0.3423	0.4898418	Organic
CN	GH76	18.3675	1.8198	0.354	0.1925	0.2128	0.3580596	Organic
CN	GH78	173.1756	23.2227	0.8295	0.7869	0.0116	0.0654915	Organic
CN	GH79	33.6982	3.1648	0.4953	0.3692	0.1186	0.2420909	Organic
CN	GH8	19.2144	3.3699	0.7911	0.7389	0.0177	0.0786072	Organic
CN	GH80	0	0	#VALUE!	#VALUE!	#VALUE!	NA	Organic
CN	GH81	5.1377	-0.1857	0.0308	-0.2115	0.7395	0.8256883	Organic
CN	GH84	4.79	0.7968	0.9548	0.9435	0.0008	0.0334192	Organic
CN	GH85	0.5304	0.0731	0.469	0.3362	0.1334	0.2594409	Organic
CN	GH86	1.1757	-0.1981	0.6863	0.6079	0.0416	0.1305186	Organic
CN	GH87	78.9746	11.0962	0.8633	0.8291	0.0074	0.0654196	Organic
CN	GH88	23.1746	0.425	0.0534	-0.1833	0.6597	0.7711571	Organic
CN	GH89	9.9199	2.1955	0.6577	0.5722	0.0502	0.1388903	Organic
CN	GH91	0.0458	-0.0249	0.4611	0.3264	0.138	0.2663711	Organic
CN	GH90	0.0009	0.0011	0.2504	0.0631	0.312	0.4715961	Organic
CN	GH9	60.9971	10.6543	0.904	0.88	0.0036	0.0421659	Organic
CN	GH92	121.0279	21.8939	0.5702	0.4628	0.0826	0.1869541	Organic
CN	GH93	18.7765	0.8606	0.0584	-0.177	0.6446	0.7646214	Organic
CN	GH94	39.1359	8.5653	0.4823	0.3528	0.1258	0.2505593	Organic
CN	GH95	57.25	8.6146	0.7572	0.6965	0.0242	0.091282	Organic
CN	GH96	0.1511	0.0337	0.2666	0.0832	0.2943	0.460939	Organic
CN	GH97	30.1161	2.3994	0.9377	0.9221	0.0015	0.0334192	Organic
CN	GH98	0.1755	-0.088	0.7515	0.6893	0.0254	0.091673	Organic
CN	GH99	9.8752	-0.7163	0.3437	0.1796	0.2214	0.360301	Organic
CN	GT1	150.285	-12.5164	0.25	0.0625	0.3125	0.4715961	Organic
CN	GT10	3.0892	0.6838	0.6243	0.5303	0.0615	0.1590798	Organic
CN	GT12	0.0472	0.0106	0.2528	0.066	0.3094	0.4715961	Organic
CN	GT14	2.1246	0.3938	0.7005	0.6256	0.0377	0.1268892	Organic
CN	GT11	5.7255	1.2356	0.7862	0.7328	0.0185	0.0786072	Organic
CN	GT13	0.2735	0.1104	0.5091	0.3864	0.1113	0.231011	Organic
CN	GT15	2.516	-0.0556	0.0133	-0.2333	0.8276	0.8769325	Organic
CN	GT16	0.0801	0.0223	0.6052	0.5065	0.0685	0.170271	Organic
CN	GT17	1.2195	0.1157	0.3452	0.1815	0.2201	0.360301	Organic
CN	GT18	0.0298	0.0007	0.0136	-0.233	0.8261	0.8769325	Organic
CN	GT2	1761.7613	25.4078	0.0583	-0.1771	0.6449	0.7646214	Organic

CN	GT19	83.6411	5.5604	0.8274	0.7842	0.0119	0.0654915	Organic
CN	GT20	146.1397	-3.4387	0.2322	0.0402	0.3332	0.4851399	Organic
CN	GT21	93.1775	14.1109	0.9375	0.9218	0.0015	0.0334192	Organic
CN	GT22	5.2116	0.0513	0.0437	-0.1954	0.691	0.800274	Organic
CN	GT23	0.3811	0.1521	0.6543	0.5679	0.0513	0.1403679	Organic
CN	GT24	3.5024	0.4635	0.4105	0.2631	0.1704	0.3053258	Organic
CN	GT25	7.2869	1.0513	0.8903	0.8629	0.0047	0.0467015	Organic
CN	GT26	148.5105	9.1887	0.8257	0.7822	0.0121	0.0654915	Organic
CN	GT27	15.1224	1.5351	0.6932	0.6165	0.0397	0.1283814	Organic
CN	GT28	177.4443	7.4422	0.3516	0.1896	0.2148	0.3589099	Organic
CN	GT29	3.4324	-0.3062	0.1682	-0.0398	0.4193	0.5583658	Organic
CN	GT30	121.5074	6.7525	0.5433	0.4291	0.0946	0.2048623	Organic
CN	GT3	3.9902	0.9544	0.9327	0.9159	0.0017	0.0334192	Organic
CN	GT33	1.2253	-0.0669	0.348	0.185	0.2178	0.360301	Organic
CN	GT34	1.1675	-0.0913	0.1039	-0.1201	0.5332	0.6507654	Organic
CN	GT32	11.1421	-1.4303	0.8605	0.8256	0.0077	0.0654915	Organic
CN	GT31	2.1893	0.5712	0.5325	0.4156	0.0997	0.2125428	Organic
CN	GT35	367.4633	32.7185	0.6958	0.6198	0.039	0.1283709	Organic
CN	GT37	0.0155	-0.0144	0.8421	0.8026	0.0099	0.0654915	Organic
CN	GT39	21.5812	-0.6215	0.0542	-0.1823	0.6572	0.7711571	Organic
CN	GT4	1706.1942	-43.122	0.1468	-0.0665	0.4534	0.5849421	Organic
CN	GT41	451.5852	74.176	0.8489	0.8111	0.009	0.0654915	Organic
CN	GT40	0.0022	0.0022	0.4158	0.2698	0.1668	0.3031542	Organic
CN	GT42	0.0544	0.0108	0.6805	0.6006	0.0433	0.1305186	Organic
CN	GT43	0.1274	0.0586	0.6587	0.5733	0.0499	0.1388903	Organic
CN	GT47	80.6211	15.0148	0.3155	0.1444	0.2461	0.3953189	Organic
CN	GT45	0.1867	0.0032	0.0044	-0.2445	0.9008	0.9424115	Organic
CN	GT44	0.0123	-0.0048	0.4871	0.3589	0.1231	0.2491962	Organic
CN	GT48	4.9851	0.6622	0.3873	0.2341	0.187	0.3234725	Organic
CN	GT49	0.2847	0.0673	0.3822	0.2277	0.1908	0.3276852	Organic
CN	GT5	106.4158	1.1246	0.0239	-0.2201	0.77	0.8409188	Organic
CN	GT50	1.0262	0.1576	0.4412	0.3015	0.1502	0.2849131	Organic
CN	GT53	25.4939	-4.9538	0.4783	0.3479	0.128	0.252574	Organic
CN	GT52	0	0	#VALUE!	#VALUE!	#VALUE!	NA	Organic
CN	GT51	518.0831	-40.3377	0.5801	0.4751	0.0785	0.1825949	Organic
CN	GT54	0.0816	0.0254	0.2301	0.0376	0.3356	0.4858943	Organic
CN	GT57	1.9599	0.2636	0.2323	0.0403	0.3331	0.4851399	Organic
CN	GT56	0.5203	0.1448	0.8408	0.801	0.0101	0.0654915	Organic
CN	GT55	2.6159	-0.0534	0.0036	-0.2455	0.9098	0.9439687	Organic
CN	GT58	0.7939	0.0913	0.256	0.07	0.3058	0.47005	Organic
CN	GT6	0.3853	0.0819	0.6228	0.5285	0.062	0.1590798	Organic
CN	GT59	0.5119	0.0159	0.0135	-0.2331	0.8266	0.8769325	Organic
CN	GT60	3.4349	1.1124	0.8223	0.7779	0.0126	0.0654915	Organic
CN	GT61	0.5101	0.166	0.6784	0.598	0.0439	0.1305186	Organic
CN	GT62	2.3868	-0.0315	0.0119	-0.2352	0.8372	0.8833588	Organic
CN	GT64	0.751	-0.0161	0.0387	-0.2016	0.7087	0.8051852	Organic
CN	GT65	0.0811	0.0272	0.3872	0.234	0.1871	0.3234725	Organic
CN	GT66	16.8699	0.2978	0.038	-0.2025	0.7114	0.8051852	Organic
CN	GT69	1.138	0.1243	0.1208	-0.099	0.4996	0.6158169	Organic
CN	GT68	0.0598	0.0119	0.129	-0.0888	0.4845	0.6061799	Organic
CN	GT7	6.1602	1.024	0.9051	0.8814	0.0035	0.0421659	Organic
CN	GT72	0.0003	0.00E+00	0.0002	-0.2497	0.9771	0.9849884	Organic
CN	GT73	0.071	0.0175	0.1639	-0.0452	0.4259	0.5641558	Organic
CN	GT71	1.6293	0.2618	0.8535	0.8169	0.0085	0.0654915	Organic
CN	GT70	0.7452	0.0338	0.3452	0.1815	0.2201	0.360301	Organic

CN	GT74	0.8293	-0.0042	0.0005	-0.2494	0.9667	0.9849884	Organic
CN	GT75	0.956	0.1429	0.4037	0.2546	0.1752	0.3094246	Organic
CN	GT76	1.3751	-1.1215	0.8291	0.7864	0.0116	0.0654915	Organic
CN	GT77	0.6069	-0.3772	0.7986	0.7483	0.0164	0.0754526	Organic
CN	GT78	0.0028	-0.0042	0.8228	0.7784	0.0126	0.0654915	Organic
CN	GT8	10.5137	0.247	0.1269	-0.0914	0.4883	0.6079004	Organic
CN	GT80	6.3954	0.8856	0.7551	0.6939	0.0246	0.0914992	Organic
CN	GT82	0.3053	-0.0403	0.2018	0.0023	0.3715	0.5167313	Organic
CN	GT84	319.0374	57.7356	0.6951	0.6188	0.0392	0.1283709	Organic
CN	GT81	23.6857	-1.0722	0.1593	-0.0508	0.433	0.570517	Organic
CN	GT83	177.9561	4.8406	0.1554	-0.0558	0.4394	0.5727944	Organic
CN	GT85	3.5991	-0.7618	0.408	0.26	0.1722	0.3061922	Organic
CN	GT87	20.2064	0.9821	0.1399	-0.0752	0.4652	0.5896579	Organic
CN	GT88	0.0469	0.0124	0.1311	-0.0861	0.4806	0.6044044	Organic
CN	GT89	5.8171	-0.8648	0.5321	0.4152	0.0999	0.2125428	Organic
CN	GT90	3.3542	0.6603	0.5578	0.4472	0.088	0.1939449	Organic
CN	GT9	154.7911	3.3582	0.0708	-0.1615	0.6102	0.7307591	Organic
CN	GT91	0.0217	-0.0006	0.0031	-0.2461	0.9161	0.9465172	Organic
CN	GT93	0.3645	0.004	0.0158	-0.2303	0.8126	0.8759484	Organic
CN	GT94	1.0193	0.241	0.7812	0.7265	0.0195	0.0788558	Organic
CN	GT92	0.2101	0.0426	0.4769	0.3461	0.1288	0.252574	Organic
CN	GT95	6.3578	-0.0342	0.0017	-0.2478	0.9374	0.9644775	Organic
CN	PL1	14.0854	1.0471	0.2146	0.0182	0.3549	0.5013567	Organic
CN	PL10	5.2376	1.6313	0.812	0.765	0.0142	0.0692837	Organic
CN	PL11	2.8656	0.62	0.1908	-0.0115	0.3864	0.5287064	Organic
CN	PL12	22.902	3.4681	0.6674	0.5842	0.0472	0.136658	Organic
CN	PL13	0.0005	0.00E+00	0.0002	-0.2497	0.9771	0.9849884	Organic
CN	PL14	0.8538	-0.2658	0.7995	0.7493	0.0162	0.0754526	Organic
CN	PL15	0.1739	-0.001	0.0004	-0.2495	0.9686	0.9849884	Organic
CN	PL17	0.9434	-0.048	0.0539	-0.1827	0.6582	0.7711571	Organic
CN	PL18	0	0	#VALUE!	#VALUE!	#VALUE!	NA	Organic
CN	PL16	0.5082	0.0704	0.5972	0.4965	0.0716	0.1734297	Organic
CN	PL2	0.3036	0.1216	0.7524	0.6905	0.0252	0.091673	Organic
CN	PL20	0.0658	0.0068	0.1064	-0.117	0.5281	0.6477435	Organic
CN	PL21	0.5253	0.1481	0.431	0.2887	0.1567	0.2912201	Organic
CN	PL22	12.3762	2.2025	0.6841	0.6051	0.0423	0.1305186	Organic
CN	PL23	0.02	0.0015	0.1539	-0.0576	0.4417	0.5727944	Organic
CN	PL3	0.0966	0.008	0.0387	-0.2017	0.7089	0.8051852	Organic
CN	PL4	26.4684	8.9672	0.5732	0.4666	0.0813	0.185757	Organic
CN	PL5	4.576	1.6914	0.5851	0.4814	0.0764	0.1794292	Organic
CN	PL6	1.1647	0.2477	0.4253	0.2817	0.1604	0.2959059	Organic
CN	PL7	3.6063	0.5364	0.38	0.2251	0.1924	0.3281865	Organic
CN	PL8	3.0069	0.8493	0.8856	0.857	0.0051	0.0471306	Organic
CN	PL9	28.8683	4.7123	0.8421	0.8027	0.0099	0.0654915	Organic
Latitude	AA1	179.0321	10.0164	0.409	0.2612	0.1715	0.4721471	Organic
Latitude	AA11	0.5025	-0.122	0.4289	0.2861	0.1581	0.4721471	Organic
Latitude	AA10	7.5616	0.7389	0.2272	0.034	0.3392	0.6697938	Organic
Latitude	AA2	1.8202	0.1345	0.0732	-0.1585	0.6041	0.7943762	Organic
Latitude	AA3	383.3947	-42.0652	0.6528	0.566	0.0518	0.4030018	Organic
Latitude	AA4	20.7999	3.209	0.1838	-0.0202	0.3963	0.6948393	Organic
Latitude	AA5	32.5624	-4.898	0.1629	-0.0463	0.4274	0.7142742	Organic
Latitude	AA8	0.8225	0.0642	0.1106	-0.1118	0.5196	0.7240116	Organic
Latitude	AA7	52.2765	-7.0641	0.6215	0.5269	0.0624	0.4030018	Organic
Latitude	AA6	24.3337	-4.3562	0.6871	0.6088	0.0414	0.4030018	Organic
Latitude	AA9	1.8306	-0.0589	0.0161	-0.2299	0.811	0.9119387	Organic

Latitude	CE1	208.9789	2.7014	0.1373	-0.0783	0.4696	0.7240116	Organic
Latitude	CE11	155.7435	13.5493	0.8843	0.8554	0.0052	0.2319836	Organic
Latitude	CE12	20.9813	2.7462	0.2782	0.0978	0.2822	0.6005285	Organic
Latitude	CE13	0.0653	0.0193	0.1373	-0.0783	0.4696	0.7240116	Organic
Latitude	CE14	227.4441	13.756	0.5009	0.3762	0.1156	0.4331128	Organic
Latitude	CE15	52.1006	8.418	0.4895	0.3619	0.1218	0.4331128	Organic
Latitude	CE2	3.239	0.7476	0.6043	0.5054	0.0688	0.4030018	Organic
Latitude	CE3	15.1515	-2.2193	0.5421	0.4276	0.0952	0.4205944	Organic
Latitude	CE4	294.1051	-30.6796	0.2557	0.0696	0.3062	0.6300936	Organic
Latitude	CE16	1.0735	-0.0746	0.1448	-0.069	0.4567	0.7240116	Organic
Latitude	CE5	14.9063	-4.3887	0.6614	0.5768	0.049	0.4030018	Organic
Latitude	CE7	12.9532	1.8061	0.5258	0.4073	0.1029	0.4256531	Organic
Latitude	CE6	52.0839	4.6054	0.1763	-0.0296	0.4071	0.7033579	Organic
Latitude	CE8	24.9683	3.134	0.4649	0.3311	0.1357	0.4506549	Organic
Latitude	CE9	90.7663	2.0263	0.2272	0.034	0.3392	0.6697938	Organic
Latitude	GH100	1.1884	0.3183	0.1246	-0.0943	0.4926	0.7240116	Organic
Latitude	GH10	59.1017	5.0448	0.4088	0.2611	0.1716	0.4721471	Organic
Latitude	GH102	34.7782	-1.037	0.1348	-0.0815	0.4741	0.7240116	Organic
Latitude	GH101	0.4319	-0.0595	0.535	0.4188	0.0985	0.4205944	Organic
Latitude	GH103	87.9513	-12.4753	0.4512	0.314	0.144	0.4655238	Organic
Latitude	GH104	4.2879	0.1348	0.0357	-0.2053	0.7198	0.8730702	Organic
Latitude	GH105	39.1132	3.3263	0.093	-0.1338	0.5569	0.7527722	Organic
Latitude	GH106	64.736	9.418	0.3708	0.2135	0.1995	0.5064389	Organic
Latitude	GH107	0.05	-0.0079	0.0963	-0.1296	0.5494	0.7516059	Organic
Latitude	GH108	6.1612	-0.1371	0.0296	-0.213	0.7444	0.8787113	Organic
Latitude	GH109	20.7365	1.7302	0.4701	0.3376	0.1327	0.4506549	Organic
Latitude	GH11	18.0145	2.0137	0.2289	0.0361	0.3371	0.6697938	Organic
Latitude	GH110	1.6372	0.0213	0.0019	-0.2476	0.9349	0.9593766	Organic
Latitude	GH111	0.0241	-0.0035	0.1457	-0.0679	0.4553	0.7240116	Organic
Latitude	GH112	0.1865	0.095	0.4108	0.2635	0.1702	0.4721471	Organic
Latitude	GH113	7.4538	0.9628	0.5523	0.4403	0.0905	0.4177614	Organic
Latitude	GH114	8.4827	-1.4972	0.1636	-0.0455	0.4264	0.7142742	Organic
Latitude	GH115	26.0154	7.7721	0.567	0.4587	0.084	0.4177614	Organic
Latitude	GH116	54.6915	5.2129	0.3519	0.1898	0.2146	0.5213816	Organic
Latitude	GH117	3.207	0.183	0.1032	-0.121	0.5347	0.7397347	Organic
Latitude	GH118	0.0019	0.00E+00	1.00E-04	-0.2499	0.9864	0.9904092	Organic
Latitude	GH119	1.7272	0.3604	0.6061	0.5077	0.0681	0.4030018	Organic
Latitude	GH12	10.5185	1.6086	0.3162	0.1452	0.2454	0.5492287	Organic
Latitude	GH120	2.0347	-0.02	0.001	-0.2487	0.9525	0.9680058	Organic
Latitude	GH121	0.74	-0.0141	0.0094	-0.2382	0.8549	0.9175565	Organic
Latitude	GH123	0.8792	-0.2984	0.1206	-0.0992	0.5	0.7240116	Organic
Latitude	GH124	0.0228	-0.0153	0.1535	-0.0581	0.4424	0.7240116	Organic
Latitude	GH125	14.4186	0.16	0.0022	-0.2472	0.9294	0.9593766	Organic
Latitude	GH126	0.0126	-0.0038	0.1119	-0.1101	0.517	0.7240116	Organic
Latitude	GH127	53.4987	6.9491	0.4181	0.2727	0.1652	0.4721471	Organic
Latitude	GH128	9.2104	-0.2323	0.0107	-0.2367	0.8457	0.9155648	Organic
Latitude	GH129	0.4364	0.1331	0.7148	0.6435	0.034	0.4030018	Organic
Latitude	GH13	1477.2569	-32.0449	0.2154	0.0192	0.3539	0.6697938	Organic
Latitude	GH130	95.5733	14.2092	0.9129	0.8911	0.0029	0.2319836	Organic
Latitude	GH131	0.252	0.0081	0.0078	-0.2402	0.8677	0.9203926	Organic
Latitude	GH132	0.9233	-0.1441	0.3382	0.1727	0.2261	0.5360808	Organic
Latitude	GH133	59.9767	-1.5022	0.0223	-0.2221	0.7776	0.8935626	Organic
Latitude	GH14	0.6765	0.0442	0.0545	-0.1819	0.6563	0.833813	Organic
Latitude	GH17	51.2924	-5.7028	0.4207	0.2759	0.1635	0.4721471	Organic
Latitude	GH15	420.1827	-0.1698	0.00E+00	-0.25	0.9922	0.9922322	Organic

Latitude	GH18	255.9295	13.1454	0.2443	0.0554	0.3189	0.6509271	Organic
Latitude	GH19	15.6439	-0.0547	0.0011	-0.2486	0.9493	0.9680058	Organic
Latitude	GH2	452.5084	64.8273	0.658	0.5725	0.0501	0.4030018	Organic
Latitude	GH20	103.5535	4.5728	0.1108	-0.1115	0.5192	0.7240116	Organic
Latitude	GH23	366.3567	2.2405	0.0404	-0.1995	0.7026	0.8617823	Organic
Latitude	GH24	15.7222	1.3965	0.6454	0.5567	0.0542	0.4030018	Organic
Latitude	GH25	18.1223	0.1748	0.0159	-0.2302	0.812	0.9119387	Organic
Latitude	GH26	23.6541	2.219	0.312	0.14	0.2492	0.5492287	Organic
Latitude	GH27	104.8235	13.0954	0.3422	0.1778	0.2226	0.5329659	Organic
Latitude	GH28	237.447	37.7264	0.5552	0.444	0.0892	0.4177614	Organic
Latitude	GH29	132.2116	16.2094	0.6909	0.6136	0.0403	0.4030018	Organic
Latitude	GH3	680.4412	54.2734	0.3147	0.1433	0.2468	0.5492287	Organic
Latitude	GH30	59.6043	0.862	0.0107	-0.2366	0.8454	0.9155648	Organic
Latitude	GH31	173.7587	16.1906	0.3505	0.1882	0.2157	0.5213816	Organic
Latitude	GH33	48.5541	3.5582	0.5231	0.4039	0.1043	0.4256531	Organic
Latitude	GH36	117.4663	16.3937	0.4529	0.3162	0.1429	0.4655238	Organic
Latitude	GH32	30.1748	-0.3054	0.0081	-0.2399	0.8655	0.9203926	Organic
Latitude	GH37	45.5476	2.3718	0.0914	-0.1358	0.5604	0.7527722	Organic
Latitude	GH39	110.1752	16.6197	0.7408	0.676	0.0277	0.4030018	Organic
Latitude	GH38	153.8558	15.4149	0.2035	0.0043	0.3693	0.6697938	Organic
Latitude	GH4	141.0485	-1.6316	0.0148	-0.2315	0.8186	0.9119387	Organic
Latitude	GH42	52.5554	3.2205	0.4985	0.3731	0.1169	0.4331128	Organic
Latitude	GH43	133.3589	14.3807	0.2914	0.1142	0.269	0.5823417	Organic
Latitude	GH44	32.7913	7.6884	0.8804	0.8505	0.0056	0.2319836	Organic
Latitude	GH45	0.5846	0.1316	0.4075	0.2593	0.1726	0.4721471	Organic
Latitude	GH47	14.6845	-0.3726	0.0411	-0.1986	0.7	0.8617823	Organic
Latitude	GH46	1.4741	-0.2581	0.4092	0.2615	0.1714	0.4721471	Organic
Latitude	GH48	10.4606	1.2186	0.4678	0.3347	0.1341	0.4506549	Organic
Latitude	GH49	0.0919	-0.0078	0.1149	-0.1064	0.5111	0.7240116	Organic
Latitude	GH5	172.0215	8.4802	0.6123	0.5154	0.0658	0.4030018	Organic
Latitude	GH50	7.7274	0.7019	0.1749	-0.0314	0.4092	0.7033579	Organic
Latitude	GH51	136.9965	20.0071	0.6293	0.5367	0.0597	0.4030018	Organic
Latitude	GH54	33.242	2.5849	0.0487	-0.1891	0.6743	0.8407703	Organic
Latitude	GH53	65.4769	10.2688	0.7645	0.7057	0.0227	0.4030018	Organic
Latitude	GH55	145.3713	10.6197	0.1101	-0.1123	0.5205	0.7240116	Organic
Latitude	GH56	0.0085	0.0047	0.22	0.025	0.3481	0.6697938	Organic
Latitude	GH57	148.2848	12.6543	0.5531	0.4413	0.0901	0.4177614	Organic
Latitude	GH58	0.0299	0.0015	0.0252	-0.2185	0.7638	0.884587	Organic
Latitude	GH59	11.8995	0.5602	0.0217	-0.2228	0.7805	0.8935626	Organic
Latitude	GH6	21.9717	1.8886	0.215	0.0187	0.3544	0.6697938	Organic
Latitude	GH62	3.402	0.98	0.6477	0.5596	0.0534	0.4030018	Organic
Latitude	GH63	18.035	2.058	0.3572	0.1965	0.2102	0.5183279	Organic
Latitude	GH64	23.3696	1.6652	0.1728	-0.034	0.4124	0.7033579	Organic
Latitude	GH65	99.333	-5.0412	0.1333	-0.0834	0.4767	0.7240116	Organic
Latitude	GH66	1.6072	0.4563	0.6157	0.5197	0.0645	0.4030018	Organic
Latitude	GH68	0.3029	-0.0216	0.0777	-0.1529	0.5928	0.7851339	Organic
Latitude	GH7	0.8798	0.0196	0.0077	-0.2404	0.8686	0.9203926	Organic
Latitude	GH70	0.0313	0.0106	0.4351	0.2938	0.1541	0.4721471	Organic
Latitude	GH71	4.8837	-0.3928	0.0597	-0.1754	0.6408	0.8267181	Organic
Latitude	GH72	30.0891	0.6453	0.138	-0.0775	0.4684	0.7240116	Organic
Latitude	GH73	36.2954	0.294	0.062	-0.1726	0.6344	0.822686	Organic
Latitude	GH75	2.0444	0.0648	0.0274	-0.2157	0.7539	0.884587	Organic
Latitude	GH74	16.7603	3.0683	0.5771	0.4714	0.0797	0.4177614	Organic
Latitude	GH76	18.3675	0.067	0.0005	-0.2494	0.9671	0.9789372	Organic
Latitude	GH78	173.1756	13.2295	0.2692	0.0865	0.2916	0.6100665	Organic

Latitude	GH79	33.6982	0.3792	0.0071	-0.2411	0.8738	0.9212573	Organic
Latitude	GH8	19.2144	2.3429	0.3824	0.228	0.1907	0.5048941	Organic
Latitude	GH80	0	0	#VALUE!	#VALUE!	#VALUE!	NA	Organic
Latitude	GH81	5.1377	0.125	0.0139	-0.2326	0.8237	0.9119387	Organic
Latitude	GH84	4.79	0.6482	0.6319	0.5399	0.0588	0.4030018	Organic
Latitude	GH85	0.5304	0.0873	0.6687	0.5859	0.0468	0.4030018	Organic
Latitude	GH86	1.1757	-0.0563	0.0555	-0.1806	0.6531	0.833813	Organic
Latitude	GH87	78.9746	5.583	0.2185	0.0232	0.3498	0.6697938	Organic
Latitude	GH88	23.1746	1.4646	0.6337	0.5421	0.0581	0.4030018	Organic
Latitude	GH89	9.9199	0.6232	0.053	-0.1838	0.6608	0.8352079	Organic
Latitude	GH91	0.0458	-0.0054	0.0214	-0.2233	0.7823	0.8935626	Organic
Latitude	GH90	0.0009	0.001	0.2	0	0.3739	0.6697938	Organic
Latitude	GH9	60.9971	7.0484	0.3957	0.2446	0.1809	0.4843953	Organic
Latitude	GH92	121.0279	6.2138	0.0459	-0.1926	0.6834	0.8466526	Organic
Latitude	GH93	18.7765	-0.3582	0.0101	-0.2374	0.8497	0.9158897	Organic
Latitude	GH94	39.1359	8.0588	0.4269	0.2836	0.1594	0.4721471	Organic
Latitude	GH95	57.25	5.9332	0.3592	0.199	0.2087	0.5183279	Organic
Latitude	GH96	0.1511	0.052	0.6367	0.5459	0.0571	0.4030018	Organic
Latitude	GH97	30.1161	1.6198	0.4273	0.2842	0.1591	0.4721471	Organic
Latitude	GH98	0.1755	-0.0305	0.0905	-0.1368	0.5623	0.7527722	Organic
Latitude	GH99	9.8752	-0.2212	0.0328	-0.209	0.7314	0.8755398	Organic
Latitude	GT1	150.285	-17.515	0.4896	0.3619	0.1217	0.4331128	Organic
Latitude	GT10	3.0892	0.6737	0.606	0.5075	0.0682	0.4030018	Organic
Latitude	GT12	0.0472	0.0074	0.1243	-0.0946	0.493	0.7240116	Organic
Latitude	GT14	2.1246	0.2133	0.2055	0.0069	0.3666	0.6697938	Organic
Latitude	GT11	5.7255	1.1805	0.7176	0.6471	0.0333	0.4030018	Organic
Latitude	GT13	0.2735	0.0522	0.1137	-0.1078	0.5133	0.7240116	Organic
Latitude	GT15	2.516	-0.2934	0.3714	0.2142	0.1991	0.5064389	Organic
Latitude	GT16	0.0801	0.0221	0.5945	0.4932	0.0726	0.4030018	Organic
Latitude	GT17	1.2195	0.0685	0.121	-0.0987	0.4992	0.7240116	Organic
Latitude	GT18	0.0298	0.0003	0.0018	-0.2477	0.9363	0.9593766	Organic
Latitude	GT2	1761.7613	87.4469	0.6906	0.6132	0.0404	0.4030018	Organic
Latitude	GT19	83.6411	5.5741	0.8314	0.7893	0.0113	0.3131067	Organic
Latitude	GT20	146.1397	-2.5266	0.1253	-0.0933	0.4911	0.7240116	Organic
Latitude	GT21	93.1775	10.7926	0.5484	0.4355	0.0922	0.4177614	Organic
Latitude	GT22	5.2116	0.0431	0.0308	-0.2115	0.7393	0.8787113	Organic
Latitude	GT23	0.3811	0.1053	0.3138	0.1422	0.2476	0.5492287	Organic
Latitude	GT24	3.5024	0.1946	0.0724	-0.1595	0.6062	0.7943762	Organic
Latitude	GT25	7.2869	0.5838	0.2746	0.0932	0.2859	0.6033942	Organic
Latitude	GT26	148.5105	9.0827	0.8068	0.7585	0.015	0.3738301	Organic
Latitude	GT27	15.1224	1.1659	0.3999	0.2498	0.1779	0.4815078	Organic
Latitude	GT28	177.4443	11.9853	0.912	0.89	0.003	0.2319836	Organic
Latitude	GT29	3.4324	0.191	0.0654	-0.1682	0.6247	0.8143886	Organic
Latitude	GT30	121.5074	8.7073	0.9033	0.8791	0.0036	0.2319836	Organic
Latitude	GT3	3.9902	0.5643	0.3261	0.1576	0.2366	0.5492287	Organic
Latitude	GT33	1.2253	-0.0639	0.3175	0.1469	0.2442	0.5492287	Organic
Latitude	GT34	1.1675	-0.2024	0.511	0.3887	0.1104	0.4331128	Organic
Latitude	GT32	11.1421	-1.2269	0.6331	0.5414	0.0583	0.4030018	Organic
Latitude	GT31	2.1893	0.2458	0.0986	-0.1267	0.5444	0.7489486	Organic
Latitude	GT35	367.4633	36.8697	0.8836	0.8545	0.0053	0.2319836	Organic
Latitude	GT37	0.0155	-0.0072	0.2127	0.0159	0.3573	0.6697938	Organic
Latitude	GT39	21.5812	-1.4139	0.2804	0.1004	0.28	0.6005285	Organic
Latitude	GT4	1706.1942	38.3778	0.1163	-0.1046	0.5083	0.7240116	Organic
Latitude	GT41	451.5852	74.091	0.8469	0.8086	0.0093	0.2888557	Organic
Latitude	GT40	0.0022	0.0024	0.4951	0.3689	0.1187	0.4331128	Organic

Latitude	GT42	0.0544	0.006	0.2109	0.0137	0.3595	0.6697938	Organic
Latitude	GT43	0.1274	0.0503	0.4861	0.3576	0.1236	0.4336236	Organic
Latitude	GT47	80.6211	9.3689	0.1228	-0.0965	0.4958	0.7240116	Organic
Latitude	GT45	0.1867	0.0313	0.4278	0.2847	0.1588	0.4721471	Organic
Latitude	GT44	0.0123	-0.0026	0.1413	-0.0733	0.4626	0.7240116	Organic
Latitude	GT48	4.9851	-0.012	0.0001	-0.2498	0.983	0.9904092	Organic
Latitude	GT49	0.2847	0.0069	0.004	-0.245	0.905	0.9389516	Organic
Latitude	GT5	106.4158	5.3878	0.5483	0.4354	0.0923	0.4177614	Organic
Latitude	GT50	1.0262	0.0269	0.0128	-0.234	0.8309	0.9140595	Organic
Latitude	GT53	25.4939	-5.855	0.6682	0.5853	0.0469	0.4030018	Organic
Latitude	GT52	0	0	#VALUE!	#VALUE!	#VALUE!	NA	Organic
Latitude	GT51	518.0831	-6.2418	0.0139	-0.2326	0.824	0.9119387	Organic
Latitude	GT54	0.0816	0.0044	0.0068	-0.2415	0.8769	0.9212573	Organic
Latitude	GT57	1.9599	-0.0761	0.0194	-0.2258	0.7926	0.9011832	Organic
Latitude	GT56	0.5203	0.0959	0.3691	0.2114	0.2008	0.5064389	Organic
Latitude	GT55	2.6159	-0.6115	0.475	0.3438	0.1299	0.4490967	Organic
Latitude	GT58	0.7939	-0.0196	0.0118	-0.2353	0.8378	0.9149151	Organic
Latitude	GT6	0.3853	0.0797	0.5889	0.4862	0.0749	0.4051878	Organic
Latitude	GT59	0.5119	-0.0628	0.2094	0.0117	0.3615	0.6697938	Organic
Latitude	GT60	3.4349	0.5103	0.173	-0.0337	0.412	0.7033579	Organic
Latitude	GT61	0.5101	0.1242	0.3798	0.2247	0.1926	0.5048941	Organic
Latitude	GT62	2.3868	-0.1243	0.1848	-0.0189	0.3948	0.6948393	Organic
Latitude	GT64	0.751	0.0385	0.2213	0.0266	0.3465	0.6697938	Organic
Latitude	GT65	0.0811	0.0099	0.0516	-0.1856	0.6653	0.8366295	Organic
Latitude	GT66	16.8699	1.0732	0.4933	0.3666	0.1197	0.4331128	Organic
Latitude	GT69	1.138	-0.1241	0.1204	-0.0995	0.5005	0.7240116	Organic
Latitude	GT68	0.0598	0.0061	0.0335	-0.2081	0.7284	0.8755398	Organic
Latitude	GT7	6.1602	0.9323	0.7503	0.6879	0.0257	0.4030018	Organic
Latitude	GT72	0.0003	-0.0003	0.2	0.00E+00	0.3739	0.6697938	Organic
Latitude	GT73	0.071	0.0095	0.0484	-0.1895	0.6753	0.8407703	Organic
Latitude	GT71	1.6293	0.1157	0.1668	-0.0415	0.4214	0.7137853	Organic
Latitude	GT70	0.7452	0.0174	0.0914	-0.1357	0.5603	0.7527722	Organic
Latitude	GT74	0.8293	0.0668	0.1231	-0.0962	0.4954	0.7240116	Organic
Latitude	GT75	0.956	0.1702	0.5732	0.4665	0.0813	0.4177614	Organic
Latitude	GT76	1.3751	-0.575	0.218	0.0224	0.3506	0.6697938	Organic
Latitude	GT77	0.6069	-0.1648	0.1524	-0.0595	0.4441	0.7240116	Organic
Latitude	GT78	0.0028	-0.003	0.4327	0.2909	0.1556	0.4721471	Organic
Latitude	GT8	10.5137	0.0825	0.0142	-0.2323	0.8223	0.9119387	Organic
Latitude	GT80	6.3954	0.7855	0.594	0.4925	0.0728	0.4030018	Organic
Latitude	GT82	0.3053	0.0259	0.0834	-0.1458	0.5789	0.7708648	Organic
Latitude	GT84	319.0374	63.8532	0.8502	0.8127	0.0089	0.2888557	Organic
Latitude	GT81	23.6857	-0.5031	0.0351	-0.2061	0.7223	0.8730702	Organic
Latitude	GT83	177.9561	4.413	0.1291	-0.0886	0.4842	0.7240116	Organic
Latitude	GT85	3.5991	-0.8379	0.4936	0.367	0.1195	0.4331128	Organic
Latitude	GT87	20.2064	-0.293	0.0125	-0.2344	0.8333	0.9140595	Organic
Latitude	GT88	0.0469	-0.0055	0.0259	-0.2176	0.7605	0.884587	Organic
Latitude	GT89	5.8171	-0.8651	0.5326	0.4157	0.0997	0.4205944	Organic
Latitude	GT90	3.3542	0.1717	0.0377	-0.2029	0.7123	0.8694859	Organic
Latitude	GT9	154.7911	10.3291	0.6701	0.5876	0.0464	0.4030018	Organic
Latitude	GT91	0.0217	-0.0068	0.3684	0.2105	0.2014	0.5064389	Organic
Latitude	GT93	0.3645	-0.0107	0.1112	-0.111	0.5184	0.7240116	Organic
Latitude	GT94	1.0193	0.228	0.6994	0.6242	0.038	0.4030018	Organic
Latitude	GT92	0.2101	0.0209	0.1151	-0.1061	0.5105	0.7240116	Organic
Latitude	GT95	6.3578	0.4416	0.2916	0.1145	0.2688	0.5823417	Organic
Latitude	PL1	14.0854	1.7536	0.6019	0.5023	0.0698	0.4030018	Organic

Latitude	PL10	5.2376	0.7966	0.1936	-0.008	0.3826	0.6804843	Organic
Latitude	PL11	2.8656	0.9415	0.4401	0.3001	0.1509	0.4721471	Organic
Latitude	PL12	22.902	3.6971	0.7584	0.698	0.0239	0.4030018	Organic
Latitude	PL13	0.0005	-0.0006	0.2	0.00E+00	0.3739	0.6697938	Organic
Latitude	PL14	0.8538	-0.1166	0.1537	-0.0579	0.4421	0.7240116	Organic
Latitude	PL15	0.1739	0.0243	0.263	0.0788	0.2982	0.6186818	Organic
Latitude	PL17	0.9434	0.0334	0.026	-0.2175	0.7603	0.884587	Organic
Latitude	PL18	0	0	#VALUE!	#VALUE!	#VALUE!	NA	Organic
Latitude	PL16	0.5082	0.0751	0.6788	0.5985	0.0438	0.4030018	Organic
Latitude	PL2	0.3036	0.1224	0.7632	0.704	0.0229	0.4030018	Organic
Latitude	PL20	0.0658	-0.0049	0.0558	-0.1803	0.6523	0.833813	Organic
Latitude	PL21	0.5253	0.0753	0.1116	-0.1106	0.5176	0.7240116	Organic
Latitude	PL22	12.3762	2.0058	0.5674	0.4592	0.0838	0.4177614	Organic
Latitude	PL23	0.02	0.0033	0.6975	0.6218	0.0385	0.4030018	Organic
Latitude	PL3	0.0966	-0.0026	0.0042	-0.2447	0.9028	0.9389516	Organic
Latitude	PL4	26.4684	6.63	0.3134	0.1417	0.248	0.5492287	Organic
Latitude	PL5	4.576	0.3802	0.0296	-0.213	0.7446	0.8787113	Organic
Latitude	PL6	1.1647	0.2777	0.5343	0.4178	0.0989	0.4205944	Organic
Latitude	PL7	3.6063	0.0627	0.0052	-0.2435	0.8921	0.933332	Organic
Latitude	PL8	3.0069	0.5209	0.3332	0.1665	0.2303	0.5410616	Organic
Latitude	PL9	28.8683	3.6671	0.51	0.3875	0.1109	0.4331128	Organic
Longitude	AA1	179.0321	-10.0164	0.409	0.2612	0.1715	0.4721471	Organic
Longitude	AA11	0.5025	0.122	0.4289	0.2861	0.1581	0.4721471	Organic
Longitude	AA10	7.5616	-0.7389	0.2272	0.034	0.3392	0.6697938	Organic
Longitude	AA2	1.8202	-0.1345	0.0732	-0.1585	0.6041	0.7943762	Organic
Longitude	AA3	383.3947	42.0652	0.6528	0.566	0.0518	0.4030018	Organic
Longitude	AA4	20.7999	-3.209	0.1838	-0.0202	0.3963	0.6948393	Organic
Longitude	AA5	32.5624	4.898	0.1629	-0.0463	0.4274	0.7142742	Organic
Longitude	AA8	0.8225	-0.0642	0.1106	-0.1118	0.5196	0.7240116	Organic
Longitude	AA7	52.2765	7.0641	0.6215	0.5269	0.0624	0.4030018	Organic
Longitude	AA6	24.3337	4.3562	0.6871	0.6088	0.0414	0.4030018	Organic
Longitude	AA9	1.8306	0.0589	0.0161	-0.2299	0.811	0.9119387	Organic
Longitude	CE1	208.9789	-2.7014	0.1373	-0.0783	0.4696	0.7240116	Organic
Longitude	CE11	155.7435	-13.5493	0.8843	0.8554	0.0052	0.2319836	Organic
Longitude	CE12	20.9813	-2.7462	0.2782	0.0978	0.2822	0.6005285	Organic
Longitude	CE13	0.0653	-0.0193	0.1373	-0.0783	0.4696	0.7240116	Organic
Longitude	CE14	227.4441	-13.756	0.5009	0.3762	0.1156	0.4331128	Organic
Longitude	CE15	52.1006	-8.418	0.4895	0.3619	0.1218	0.4331128	Organic
Longitude	CE2	3.239	-0.7476	0.6043	0.5054	0.0688	0.4030018	Organic
Longitude	CE3	15.1515	2.2193	0.5421	0.4276	0.0952	0.4205944	Organic
Longitude	CE4	294.1051	30.6796	0.2557	0.0696	0.3062	0.6300936	Organic
Longitude	CE16	1.0735	0.0746	0.1448	-0.069	0.4567	0.7240116	Organic
Longitude	CE5	14.9063	4.3887	0.6614	0.5768	0.049	0.4030018	Organic
Longitude	CE7	12.9532	-1.8061	0.5258	0.4073	0.1029	0.4256531	Organic
Longitude	CE6	52.0839	-4.6054	0.1763	-0.0296	0.4071	0.7033579	Organic
Longitude	CE8	24.9683	-3.134	0.4649	0.3311	0.1357	0.4506549	Organic
Longitude	CE9	90.7663	-2.0263	0.2272	0.034	0.3392	0.6697938	Organic
Longitude	GH100	1.1884	-0.3183	0.1246	-0.0943	0.4926	0.7240116	Organic
Longitude	GH10	59.1017	-5.0448	0.4088	0.2611	0.1716	0.4721471	Organic
Longitude	GH102	34.7782	1.037	0.1348	-0.0815	0.4741	0.7240116	Organic
Longitude	GH101	0.4319	0.0595	0.535	0.4188	0.0985	0.4205944	Organic
Longitude	GH103	87.9513	12.4753	0.4512	0.314	0.144	0.4655238	Organic
Longitude	GH104	4.2879	-0.1348	0.0357	-0.2053	0.7198	0.8730702	Organic
Longitude	GH105	39.1132	-3.3263	0.093	-0.1338	0.5569	0.7527722	Organic
Longitude	GH106	64.736	-9.418	0.3708	0.2135	0.1995	0.5064389	Organic

Longitude	GH107	0.05	0.0079	0.0963	-0.1296	0.5494	0.7516059	Organic
Longitude	GH108	6.1612	0.1371	0.0296	-0.213	0.7444	0.8787113	Organic
Longitude	GH109	20.7365	-1.7302	0.4701	0.3376	0.1327	0.4506549	Organic
Longitude	GH11	18.0145	-2.0137	0.2289	0.0361	0.3371	0.6697938	Organic
Longitude	GH110	1.6372	-0.0213	0.0019	-0.2476	0.9349	0.9593766	Organic
Longitude	GH111	0.0241	0.0035	0.1457	-0.0679	0.4553	0.7240116	Organic
Longitude	GH112	0.1865	-0.095	0.4108	0.2635	0.1702	0.4721471	Organic
Longitude	GH113	7.4538	-0.9628	0.5523	0.4403	0.0905	0.4177614	Organic
Longitude	GH114	8.4827	1.4972	0.1636	-0.0455	0.4264	0.7142742	Organic
Longitude	GH115	26.0154	-7.7721	0.567	0.4587	0.084	0.4177614	Organic
Longitude	GH116	54.6915	-5.2129	0.3519	0.1898	0.2146	0.5213816	Organic
Longitude	GH117	3.207	-0.183	0.1032	-0.121	0.5347	0.7397347	Organic
Longitude	GH118	0.0019	0.00E+00	1.00E-04	-0.2499	0.9864	0.9904092	Organic
Longitude	GH119	1.7272	-0.3604	0.6061	0.5077	0.0681	0.4030018	Organic
Longitude	GH12	10.5185	-1.6086	0.3162	0.1452	0.2454	0.5492287	Organic
Longitude	GH120	2.0347	0.02	0.001	-0.2487	0.9525	0.9680058	Organic
Longitude	GH121	0.74	0.0141	0.0094	-0.2382	0.8549	0.9175565	Organic
Longitude	GH123	0.8792	0.2984	0.1206	-0.0992	0.5	0.7240116	Organic
Longitude	GH124	0.0228	0.0153	0.1535	-0.0581	0.4424	0.7240116	Organic
Longitude	GH125	14.4186	-0.16	0.0022	-0.2472	0.9294	0.9593766	Organic
Longitude	GH126	0.0126	0.0038	0.1119	-0.1101	0.517	0.7240116	Organic
Longitude	GH127	53.4987	-6.9491	0.4181	0.2727	0.1652	0.4721471	Organic
Longitude	GH128	9.2104	0.2323	0.0107	-0.2367	0.8457	0.9155648	Organic
Longitude	GH129	0.4364	-0.1331	0.7148	0.6435	0.034	0.4030018	Organic
Longitude	GH13	1477.2569	32.0449	0.2154	0.0192	0.3539	0.6697938	Organic
Longitude	GH130	95.5733	-14.2092	0.9129	0.8911	0.0029	0.2319836	Organic
Longitude	GH131	0.252	-0.0081	0.0078	-0.2402	0.8677	0.9203926	Organic
Longitude	GH132	0.9233	0.1441	0.3382	0.1727	0.2261	0.5360808	Organic
Longitude	GH133	59.9767	1.5022	0.0223	-0.2221	0.7776	0.8935626	Organic
Longitude	GH14	0.6765	-0.0442	0.0545	-0.1819	0.6563	0.833813	Organic
Longitude	GH17	51.2924	5.7028	0.4207	0.2759	0.1635	0.4721471	Organic
Longitude	GH15	420.1827	0.1698	0.00E+00	-0.25	0.9922	0.9922322	Organic
Longitude	GH18	255.9295	-13.1454	0.2443	0.0554	0.3189	0.6509271	Organic
Longitude	GH19	15.6439	0.0547	0.0011	-0.2486	0.9493	0.9680058	Organic
Longitude	GH2	452.5084	-64.8273	0.658	0.5725	0.0501	0.4030018	Organic
Longitude	GH20	103.5535	-4.5728	0.1108	-0.1115	0.5192	0.7240116	Organic
Longitude	GH23	366.3567	-2.2405	0.0404	-0.1995	0.7026	0.8617823	Organic
Longitude	GH24	15.7222	-1.3965	0.6454	0.5567	0.0542	0.4030018	Organic
Longitude	GH25	18.1223	-0.1748	0.0159	-0.2302	0.812	0.9119387	Organic
Longitude	GH26	23.6541	-2.219	0.312	0.14	0.2492	0.5492287	Organic
Longitude	GH27	104.8235	-13.0954	0.3422	0.1778	0.2226	0.5329659	Organic
Longitude	GH28	237.447	-37.7264	0.5552	0.444	0.0892	0.4177614	Organic
Longitude	GH29	132.2116	-16.2094	0.6909	0.6136	0.0403	0.4030018	Organic
Longitude	GH3	680.4412	-54.2734	0.3147	0.1433	0.2468	0.5492287	Organic
Longitude	GH30	59.6043	-0.862	0.0107	-0.2366	0.8454	0.9155648	Organic
Longitude	GH31	173.7587	-16.1906	0.3505	0.1882	0.2157	0.5213816	Organic
Longitude	GH33	48.5541	-3.5582	0.5231	0.4039	0.1043	0.4256531	Organic
Longitude	GH36	117.4663	-16.3937	0.4529	0.3162	0.1429	0.4655238	Organic
Longitude	GH32	30.1748	0.3054	0.0081	-0.2399	0.8655	0.9203926	Organic
Longitude	GH37	45.5476	-2.3718	0.0914	-0.1358	0.5604	0.7527722	Organic
Longitude	GH39	110.1752	-16.6197	0.7408	0.676	0.0277	0.4030018	Organic
Longitude	GH38	153.8558	-15.4149	0.2035	0.0043	0.3693	0.6697938	Organic
Longitude	GH4	141.0485	1.6316	0.0148	-0.2315	0.8186	0.9119387	Organic
Longitude	GH42	52.5554	-3.2205	0.4985	0.3731	0.1169	0.4331128	Organic
Longitude	GH43	133.3589	-14.3807	0.2914	0.1142	0.269	0.5823417	Organic

Longitude	GH44	32.7913	-7.6884	0.8804	0.8505	0.0056	0.2319836	Organic
Longitude	GH45	0.5846	-0.1316	0.4075	0.2593	0.1726	0.4721471	Organic
Longitude	GH47	14.6845	0.3726	0.0411	-0.1986	0.7	0.8617823	Organic
Longitude	GH46	1.4741	0.2581	0.4092	0.2615	0.1714	0.4721471	Organic
Longitude	GH48	10.4606	-1.2186	0.4678	0.3347	0.1341	0.4506549	Organic
Longitude	GH49	0.0919	0.0078	0.1149	-0.1064	0.5111	0.7240116	Organic
Longitude	GH5	172.0215	-8.4802	0.6123	0.5154	0.0658	0.4030018	Organic
Longitude	GH50	7.7274	-0.7019	0.1749	-0.0314	0.4092	0.7033579	Organic
Longitude	GH51	136.9965	-20.0071	0.6293	0.5367	0.0597	0.4030018	Organic
Longitude	GH54	33.242	-2.5849	0.0487	-0.1891	0.6743	0.8407703	Organic
Longitude	GH53	65.4769	-10.2688	0.7645	0.7057	0.0227	0.4030018	Organic
Longitude	GH55	145.3713	-10.6197	0.1101	-0.1123	0.5205	0.7240116	Organic
Longitude	GH56	0.0085	-0.0047	0.22	0.025	0.3481	0.6697938	Organic
Longitude	GH57	148.2848	-12.6543	0.5531	0.4413	0.0901	0.4177614	Organic
Longitude	GH58	0.0299	-0.0015	0.0252	-0.2185	0.7638	0.884587	Organic
Longitude	GH59	11.8995	-0.5602	0.0217	-0.2228	0.7805	0.8935626	Organic
Longitude	GH6	21.9717	-1.8886	0.215	0.0187	0.3544	0.6697938	Organic
Longitude	GH62	3.402	-0.98	0.6477	0.5596	0.0534	0.4030018	Organic
Longitude	GH63	18.035	-2.058	0.3572	0.1965	0.2102	0.5183279	Organic
Longitude	GH64	23.3696	-1.6652	0.1728	-0.034	0.4124	0.7033579	Organic
Longitude	GH65	99.333	5.0412	0.1333	-0.0834	0.4767	0.7240116	Organic
Longitude	GH66	1.6072	-0.4563	0.6157	0.5197	0.0645	0.4030018	Organic
Longitude	GH68	0.3029	0.0216	0.0777	-0.1529	0.5928	0.7851339	Organic
Longitude	GH7	0.8798	-0.0196	0.0077	-0.2404	0.8686	0.9203926	Organic
Longitude	GH70	0.0313	-0.0106	0.4351	0.2938	0.1541	0.4721471	Organic
Longitude	GH71	4.8837	0.3928	0.0597	-0.1754	0.6408	0.8267181	Organic
Longitude	GH72	30.0891	-0.6453	0.138	-0.0775	0.4684	0.7240116	Organic
Longitude	GH73	36.2954	-0.294	0.062	-0.1726	0.6344	0.822686	Organic
Longitude	GH75	2.0444	-0.0648	0.0274	-0.2157	0.7539	0.884587	Organic
Longitude	GH74	16.7603	-3.0683	0.5771	0.4714	0.0797	0.4177614	Organic
Longitude	GH76	18.3675	-0.067	0.0005	-0.2494	0.9671	0.9789372	Organic
Longitude	GH78	173.1756	-13.2295	0.2692	0.0865	0.2916	0.6100665	Organic
Longitude	GH79	33.6982	-0.3792	0.0071	-0.2411	0.8738	0.9212573	Organic
Longitude	GH8	19.2144	-2.3429	0.3824	0.228	0.1907	0.5048941	Organic
Longitude	GH80	0	0	#VALUE!	#VALUE!	#VALUE!	NA	Organic
Longitude	GH81	5.1377	-0.125	0.0139	-0.2326	0.8237	0.9119387	Organic
Longitude	GH84	4.79	-0.6482	0.6319	0.5399	0.0588	0.4030018	Organic
Longitude	GH85	0.5304	-0.0873	0.6687	0.5859	0.0468	0.4030018	Organic
Longitude	GH86	1.1757	0.0563	0.0555	-0.1806	0.6531	0.833813	Organic
Longitude	GH87	78.9746	-5.583	0.2185	0.0232	0.3498	0.6697938	Organic
Longitude	GH88	23.1746	-1.4646	0.6337	0.5421	0.0581	0.4030018	Organic
Longitude	GH89	9.9199	-0.6232	0.053	-0.1838	0.6608	0.8352079	Organic
Longitude	GH91	0.0458	0.0054	0.0214	-0.2233	0.7823	0.8935626	Organic
Longitude	GH90	0.0009	-0.001	0.2	0	0.3739	0.6697938	Organic
Longitude	GH9	60.9971	-7.0484	0.3957	0.2446	0.1809	0.4843953	Organic
Longitude	GH92	121.0279	-6.2138	0.0459	-0.1926	0.6834	0.8466526	Organic
Longitude	GH93	18.7765	0.3582	0.0101	-0.2374	0.8497	0.9158897	Organic
Longitude	GH94	39.1359	-8.0588	0.4269	0.2836	0.1594	0.4721471	Organic
Longitude	GH95	57.25	-5.9332	0.3592	0.199	0.2087	0.5183279	Organic
Longitude	GH96	0.1511	-0.052	0.6367	0.5459	0.0571	0.4030018	Organic
Longitude	GH97	30.1161	-1.6198	0.4273	0.2842	0.1591	0.4721471	Organic
Longitude	GH98	0.1755	0.0305	0.0905	-0.1368	0.5623	0.7527722	Organic
Longitude	GH99	9.8752	0.2212	0.0328	-0.209	0.7314	0.8755398	Organic
Longitude	GT1	150.285	17.515	0.4896	0.3619	0.1217	0.4331128	Organic
Longitude	GT10	3.0892	-0.6737	0.606	0.5075	0.0682	0.4030018	Organic

Longitude	GT12	0.0472	-0.0074	0.1243	-0.0946	0.493	0.7240116	Organic
Longitude	GT14	2.1246	-0.2133	0.2055	0.0069	0.3666	0.6697938	Organic
Longitude	GT11	5.7255	-1.1805	0.7176	0.6471	0.0333	0.4030018	Organic
Longitude	GT13	0.2735	-0.0522	0.1137	-0.1078	0.5133	0.7240116	Organic
Longitude	GT15	2.516	0.2934	0.3714	0.2142	0.1991	0.5064389	Organic
Longitude	GT16	0.0801	-0.0221	0.5945	0.4932	0.0726	0.4030018	Organic
Longitude	GT17	1.2195	-0.0685	0.121	-0.0987	0.4992	0.7240116	Organic
Longitude	GT18	0.0298	-0.0003	0.0018	-0.2477	0.9363	0.9593766	Organic
Longitude	GT2	1761.7613	-87.4469	0.6906	0.6132	0.0404	0.4030018	Organic
Longitude	GT19	83.6411	-5.5741	0.8314	0.7893	0.0113	0.3131067	Organic
Longitude	GT20	146.1397	2.5266	0.1253	-0.0933	0.4911	0.7240116	Organic
Longitude	GT21	93.1775	-10.7926	0.5484	0.4355	0.0922	0.4177614	Organic
Longitude	GT22	5.2116	-0.0431	0.0308	-0.2115	0.7393	0.8787113	Organic
Longitude	GT23	0.3811	-0.1053	0.3138	0.1422	0.2476	0.5492287	Organic
Longitude	GT24	3.5024	-0.1946	0.0724	-0.1595	0.6062	0.7943762	Organic
Longitude	GT25	7.2869	-0.5838	0.2746	0.0932	0.2859	0.6033942	Organic
Longitude	GT26	148.5105	-9.0827	0.8068	0.7585	0.015	0.3738301	Organic
Longitude	GT27	15.1224	-1.1659	0.3999	0.2498	0.1779	0.4815078	Organic
Longitude	GT28	177.4443	-11.9853	0.912	0.89	0.003	0.2319836	Organic
Longitude	GT29	3.4324	-0.191	0.0654	-0.1682	0.6247	0.8143886	Organic
Longitude	GT30	121.5074	-8.7073	0.9033	0.8791	0.0036	0.2319836	Organic
Longitude	GT3	3.9902	-0.5643	0.3261	0.1576	0.2366	0.5492287	Organic
Longitude	GT33	1.2253	0.0639	0.3175	0.1469	0.2442	0.5492287	Organic
Longitude	GT34	1.1675	0.2024	0.511	0.3887	0.1104	0.4331128	Organic
Longitude	GT32	11.1421	1.2269	0.6331	0.5414	0.0583	0.4030018	Organic
Longitude	GT31	2.1893	-0.2458	0.0986	-0.1267	0.5444	0.7489486	Organic
Longitude	GT35	367.4633	-36.8697	0.8836	0.8545	0.0053	0.2319836	Organic
Longitude	GT37	0.0155	0.0072	0.2127	0.0159	0.3573	0.6697938	Organic
Longitude	GT39	21.5812	1.4139	0.2804	0.1004	0.28	0.6005285	Organic
Longitude	GT4	1706.1942	-38.3778	0.1163	-0.1046	0.5083	0.7240116	Organic
Longitude	GT41	451.5852	-74.091	0.8469	0.8086	0.0093	0.2888557	Organic
Longitude	GT40	0.0022	-0.0024	0.4951	0.3689	0.1187	0.4331128	Organic
Longitude	GT42	0.0544	-0.006	0.2109	0.0137	0.3595	0.6697938	Organic
Longitude	GT43	0.1274	-0.0503	0.4861	0.3576	0.1236	0.4336236	Organic
Longitude	GT47	80.6211	-9.3689	0.1228	-0.0965	0.4958	0.7240116	Organic
Longitude	GT45	0.1867	-0.0313	0.4278	0.2847	0.1588	0.4721471	Organic
Longitude	GT44	0.0123	0.0026	0.1413	-0.0733	0.4626	0.7240116	Organic
Longitude	GT48	4.9851	0.012	0.0001	-0.2498	0.983	0.9904092	Organic
Longitude	GT49	0.2847	-0.0069	0.004	-0.245	0.905	0.9389516	Organic
Longitude	GT5	106.4158	-5.3878	0.5483	0.4354	0.0923	0.4177614	Organic
Longitude	GT50	1.0262	-0.0269	0.0128	-0.234	0.8309	0.9140595	Organic
Longitude	GT53	25.4939	5.855	0.6682	0.5853	0.0469	0.4030018	Organic
Longitude	GT52	0	0	#VALUE!	#VALUE!	#VALUE!	NA	Organic
Longitude	GT51	518.0831	6.2418	0.0139	-0.2326	0.824	0.9119387	Organic
Longitude	GT54	0.0816	-0.0044	0.0068	-0.2415	0.8769	0.9212573	Organic
Longitude	GT57	1.9599	0.0761	0.0194	-0.2258	0.7926	0.9011832	Organic
Longitude	GT56	0.5203	-0.0959	0.3691	0.2114	0.2008	0.5064389	Organic
Longitude	GT55	2.6159	0.6115	0.475	0.3438	0.1299	0.4490967	Organic
Longitude	GT58	0.7939	0.0196	0.0118	-0.2353	0.8378	0.9149151	Organic
Longitude	GT6	0.3853	-0.0797	0.5889	0.4862	0.0749	0.4051878	Organic
Longitude	GT59	0.5119	0.0628	0.2094	0.0117	0.3615	0.6697938	Organic
Longitude	GT60	3.4349	-0.5103	0.173	-0.0337	0.412	0.7033579	Organic
Longitude	GT61	0.5101	-0.1242	0.3798	0.2247	0.1926	0.5048941	Organic
Longitude	GT62	2.3868	0.1243	0.1848	-0.0189	0.3948	0.6948393	Organic
Longitude	GT64	0.751	-0.0385	0.2213	0.0266	0.3465	0.6697938	Organic

Longitude	GT65	0.0811	-0.0099	0.0516	-0.1856	0.6653	0.8366295	Organic
Longitude	GT66	16.8699	-1.0732	0.4933	0.3666	0.1197	0.4331128	Organic
Longitude	GT69	1.138	0.1241	0.1204	-0.0995	0.5005	0.7240116	Organic
Longitude	GT68	0.0598	-0.0061	0.0335	-0.2081	0.7284	0.8755398	Organic
Longitude	GT7	6.1602	-0.9323	0.7503	0.6879	0.0257	0.4030018	Organic
Longitude	GT72	0.0003	0.0003	0.2	0.00E+00	0.3739	0.6697938	Organic
Longitude	GT73	0.071	-0.0095	0.0484	-0.1895	0.6753	0.8407703	Organic
Longitude	GT71	1.6293	-0.1157	0.1668	-0.0415	0.4214	0.7137853	Organic
Longitude	GT70	0.7452	-0.0174	0.0914	-0.1357	0.5603	0.7527722	Organic
Longitude	GT74	0.8293	-0.0668	0.1231	-0.0962	0.4954	0.7240116	Organic
Longitude	GT75	0.956	-0.1702	0.5732	0.4665	0.0813	0.4177614	Organic
Longitude	GT76	1.3751	0.575	0.218	0.0224	0.3506	0.6697938	Organic
Longitude	GT77	0.6069	0.1648	0.1524	-0.0595	0.4441	0.7240116	Organic
Longitude	GT78	0.0028	0.003	0.4327	0.2909	0.1556	0.4721471	Organic
Longitude	GT8	10.5137	-0.0825	0.0142	-0.2323	0.8223	0.9119387	Organic
Longitude	GT80	6.3954	-0.7855	0.594	0.4925	0.0728	0.4030018	Organic
Longitude	GT82	0.3053	-0.0259	0.0834	-0.1458	0.5789	0.7708648	Organic
Longitude	GT84	319.0374	-63.8532	0.8502	0.8127	0.0089	0.2888557	Organic
Longitude	GT81	23.6857	0.5031	0.0351	-0.2061	0.7223	0.8730702	Organic
Longitude	GT83	177.9561	-4.413	0.1291	-0.0886	0.4842	0.7240116	Organic
Longitude	GT85	3.5991	0.8379	0.4936	0.367	0.1195	0.4331128	Organic
Longitude	GT87	20.2064	0.293	0.0125	-0.2344	0.8333	0.9140595	Organic
Longitude	GT88	0.0469	0.0055	0.0259	-0.2176	0.7605	0.884587	Organic
Longitude	GT89	5.8171	0.8651	0.5326	0.4157	0.0997	0.4205944	Organic
Longitude	GT90	3.3542	-0.1717	0.0377	-0.2029	0.7123	0.8694859	Organic
Longitude	GT9	154.7911	-10.3291	0.6701	0.5876	0.0464	0.4030018	Organic
Longitude	GT91	0.0217	0.0068	0.3684	0.2105	0.2014	0.5064389	Organic
Longitude	GT93	0.3645	0.0107	0.1112	-0.111	0.5184	0.7240116	Organic
Longitude	GT94	1.0193	-0.228	0.6994	0.6242	0.038	0.4030018	Organic
Longitude	GT92	0.2101	-0.0209	0.1151	-0.1061	0.5105	0.7240116	Organic
Longitude	GT95	6.3578	-0.4416	0.2916	0.1145	0.2688	0.5823417	Organic
Longitude	PL1	14.0854	-1.7536	0.6019	0.5023	0.0698	0.4030018	Organic
Longitude	PL10	5.2376	-0.7966	0.1936	-0.008	0.3826	0.6804843	Organic
Longitude	PL11	2.8656	-0.9415	0.4401	0.3001	0.1509	0.4721471	Organic
Longitude	PL12	22.902	-3.6971	0.7584	0.698	0.0239	0.4030018	Organic
Longitude	PL13	0.0005	0.0006	0.2	0.00E+00	0.3739	0.6697938	Organic
Longitude	PL14	0.8538	0.1166	0.1537	-0.0579	0.4421	0.7240116	Organic
Longitude	PL15	0.1739	-0.0243	0.263	0.0788	0.2982	0.6186818	Organic
Longitude	PL17	0.9434	-0.0334	0.026	-0.2175	0.7603	0.884587	Organic
Longitude	PL18	0	0	#VALUE!	#VALUE!	#VALUE!	NA	Organic
Longitude	PL16	0.5082	-0.0751	0.6788	0.5985	0.0438	0.4030018	Organic
Longitude	PL2	0.3036	-0.1224	0.7632	0.704	0.0229	0.4030018	Organic
Longitude	PL20	0.0658	0.0049	0.0558	-0.1803	0.6523	0.833813	Organic
Longitude	PL21	0.5253	-0.0753	0.1116	-0.1106	0.5176	0.7240116	Organic
Longitude	PL22	12.3762	-2.0058	0.5674	0.4592	0.0838	0.4177614	Organic
Longitude	PL23	0.02	-0.0033	0.6975	0.6218	0.0385	0.4030018	Organic
Longitude	PL3	0.0966	0.0026	0.0042	-0.2447	0.9028	0.9389516	Organic
Longitude	PL4	26.4684	-6.63	0.3134	0.1417	0.248	0.5492287	Organic
Longitude	PL5	4.576	-0.3802	0.0296	-0.213	0.7446	0.8787113	Organic
Longitude	PL6	1.1647	-0.2777	0.5343	0.4178	0.0989	0.4205944	Organic
Longitude	PL7	3.6063	-0.0627	0.0052	-0.2435	0.8921	0.933332	Organic
Longitude	PL8	3.0069	-0.5209	0.3332	0.1665	0.2303	0.5410616	Organic
Longitude	PL9	28.8683	-3.6671	0.51	0.3875	0.1109	0.4331128	Organic
Elevation	AA1	179.0321	10.0164	0.409	0.2612	0.1715	0.4721471	Organic
Elevation	AA11	0.5025	-0.122	0.4289	0.2861	0.1581	0.4721471	Organic

Elevation	AA10	7.5616	0.7389	0.2272	0.034	0.3392	0.6697938	Organic
Elevation	AA2	1.8202	0.1345	0.0732	-0.1585	0.6041	0.7943762	Organic
Elevation	AA3	383.3947	-42.0652	0.6528	0.566	0.0518	0.4030018	Organic
Elevation	AA4	20.7999	3.209	0.1838	-0.0202	0.3963	0.6948393	Organic
Elevation	AA5	32.5624	-4.898	0.1629	-0.0463	0.4274	0.7142742	Organic
Elevation	AA8	0.8225	0.0642	0.1106	-0.1118	0.5196	0.7240116	Organic
Elevation	AA7	52.2765	-7.0641	0.6215	0.5269	0.0624	0.4030018	Organic
Elevation	AA6	24.3337	-4.3562	0.6871	0.6088	0.0414	0.4030018	Organic
Elevation	AA9	1.8306	-0.0589	0.0161	-0.2299	0.811	0.9119387	Organic
Elevation	CE1	208.9789	2.7014	0.1373	-0.0783	0.4696	0.7240116	Organic
Elevation	CE11	155.7435	13.5493	0.8843	0.8554	0.0052	0.2319836	Organic
Elevation	CE12	20.9813	2.7462	0.2782	0.0978	0.2822	0.6005285	Organic
Elevation	CE13	0.0653	0.0193	0.1373	-0.0783	0.4696	0.7240116	Organic
Elevation	CE14	227.4441	13.756	0.5009	0.3762	0.1156	0.4331128	Organic
Elevation	CE15	52.1006	8.418	0.4895	0.3619	0.1218	0.4331128	Organic
Elevation	CE2	3.239	0.7476	0.6043	0.5054	0.0688	0.4030018	Organic
Elevation	CE3	15.1515	-2.2193	0.5421	0.4276	0.0952	0.4205944	Organic
Elevation	CE4	294.1051	-30.6796	0.2557	0.0696	0.3062	0.6300936	Organic
Elevation	CE16	1.0735	-0.0746	0.1448	-0.069	0.4567	0.7240116	Organic
Elevation	CE5	14.9063	-4.3887	0.6614	0.5768	0.049	0.4030018	Organic
Elevation	CE7	12.9532	1.8061	0.5258	0.4073	0.1029	0.4256531	Organic
Elevation	CE6	52.0839	4.6054	0.1763	-0.0296	0.4071	0.7033579	Organic
Elevation	CE8	24.9683	3.134	0.4649	0.3311	0.1357	0.4506549	Organic
Elevation	CE9	90.7663	2.0263	0.2272	0.034	0.3392	0.6697938	Organic
Elevation	GH100	1.1884	0.3183	0.1246	-0.0943	0.4926	0.7240116	Organic
Elevation	GH10	59.1017	5.0448	0.4088	0.2611	0.1716	0.4721471	Organic
Elevation	GH102	34.7782	-1.037	0.1348	-0.0815	0.4741	0.7240116	Organic
Elevation	GH101	0.4319	-0.0595	0.535	0.4188	0.0985	0.4205944	Organic
Elevation	GH103	87.9513	-12.4753	0.4512	0.314	0.144	0.4655238	Organic
Elevation	GH104	4.2879	0.1348	0.0357	-0.2053	0.7198	0.8730702	Organic
Elevation	GH105	39.1132	3.3263	0.093	-0.1338	0.5569	0.7527722	Organic
Elevation	GH106	64.736	9.418	0.3708	0.2135	0.1995	0.5064389	Organic
Elevation	GH107	0.05	-0.0079	0.0963	-0.1296	0.5494	0.7516059	Organic
Elevation	GH108	6.1612	-0.1371	0.0296	-0.213	0.7444	0.8787113	Organic
Elevation	GH109	20.7365	1.7302	0.4701	0.3376	0.1327	0.4506549	Organic
Elevation	GH11	18.0145	2.0137	0.2289	0.0361	0.3371	0.6697938	Organic
Elevation	GH110	1.6372	0.0213	0.0019	-0.2476	0.9349	0.9593766	Organic
Elevation	GH111	0.0241	-0.0035	0.1457	-0.0679	0.4553	0.7240116	Organic
Elevation	GH112	0.1865	0.095	0.4108	0.2635	0.1702	0.4721471	Organic
Elevation	GH113	7.4538	0.9628	0.5523	0.4403	0.0905	0.4177614	Organic
Elevation	GH114	8.4827	-1.4972	0.1636	-0.0455	0.4264	0.7142742	Organic
Elevation	GH115	26.0154	7.7721	0.567	0.4587	0.084	0.4177614	Organic
Elevation	GH116	54.6915	5.2129	0.3519	0.1898	0.2146	0.5213816	Organic
Elevation	GH117	3.207	0.183	0.1032	-0.121	0.5347	0.7397347	Organic
Elevation	GH118	0.0019	0.00E+00	1.00E-04	-0.2499	0.9864	0.9904092	Organic
Elevation	GH119	1.7272	0.3604	0.6061	0.5077	0.0681	0.4030018	Organic
Elevation	GH12	10.5185	1.6086	0.3162	0.1452	0.2454	0.5492287	Organic
Elevation	GH120	2.0347	-0.02	0.001	-0.2487	0.9525	0.9680058	Organic
Elevation	GH121	0.74	-0.0141	0.0094	-0.2382	0.8549	0.9175565	Organic
Elevation	GH123	0.8792	-0.2984	0.1206	-0.0992	0.5	0.7240116	Organic
Elevation	GH124	0.0228	-0.0153	0.1535	-0.0581	0.4424	0.7240116	Organic
Elevation	GH125	14.4186	0.16	0.0022	-0.2472	0.9294	0.9593766	Organic
Elevation	GH126	0.0126	-0.0038	0.1119	-0.1101	0.517	0.7240116	Organic
Elevation	GH127	53.4987	6.9491	0.4181	0.2727	0.1652	0.4721471	Organic
Elevation	GH128	9.2104	-0.2323	0.0107	-0.2367	0.8457	0.9155648	Organic

Elevation	GH129	0.4364	0.1331	0.7148	0.6435	0.034	0.4030018	Organic
Elevation	GH13	1477.2569	-32.0449	0.2154	0.0192	0.3539	0.6697938	Organic
Elevation	GH130	95.5733	14.2092	0.9129	0.8911	0.0029	0.2319836	Organic
Elevation	GH131	0.252	0.0081	0.0078	-0.2402	0.8677	0.9203926	Organic
Elevation	GH132	0.9233	-0.1441	0.3382	0.1727	0.2261	0.5360808	Organic
Elevation	GH133	59.9767	-1.5022	0.0223	-0.2221	0.7776	0.8935626	Organic
Elevation	GH14	0.6765	0.0442	0.0545	-0.1819	0.6563	0.833813	Organic
Elevation	GH17	51.2924	-5.7028	0.4207	0.2759	0.1635	0.4721471	Organic
Elevation	GH15	420.1827	-0.1698	0.00E+00	-0.25	0.9922	0.9922322	Organic
Elevation	GH18	255.9295	13.1454	0.2443	0.0554	0.3189	0.6509271	Organic
Elevation	GH19	15.6439	-0.0547	0.0011	-0.2486	0.9493	0.9680058	Organic
Elevation	GH2	452.5084	64.8273	0.658	0.5725	0.0501	0.4030018	Organic
Elevation	GH20	103.5535	4.5728	0.1108	-0.1115	0.5192	0.7240116	Organic
Elevation	GH23	366.3567	2.2405	0.0404	-0.1995	0.7026	0.8617823	Organic
Elevation	GH24	15.7222	1.3965	0.6454	0.5567	0.0542	0.4030018	Organic
Elevation	GH25	18.1223	0.1748	0.0159	-0.2302	0.812	0.9119387	Organic
Elevation	GH26	23.6541	2.219	0.312	0.14	0.2492	0.5492287	Organic
Elevation	GH27	104.8235	13.0954	0.3422	0.1778	0.2226	0.5329659	Organic
Elevation	GH28	237.447	37.7264	0.5552	0.444	0.0892	0.4177614	Organic
Elevation	GH29	132.2116	16.2094	0.6909	0.6136	0.0403	0.4030018	Organic
Elevation	GH3	680.4412	54.2734	0.3147	0.1433	0.2468	0.5492287	Organic
Elevation	GH30	59.6043	0.862	0.0107	-0.2366	0.8454	0.9155648	Organic
Elevation	GH31	173.7587	16.1906	0.3505	0.1882	0.2157	0.5213816	Organic
Elevation	GH33	48.5541	3.5582	0.5231	0.4039	0.1043	0.4256531	Organic
Elevation	GH36	117.4663	16.3937	0.4529	0.3162	0.1429	0.4655238	Organic
Elevation	GH32	30.1748	-0.3054	0.0081	-0.2399	0.8655	0.9203926	Organic
Elevation	GH37	45.5476	2.3718	0.0914	-0.1358	0.5604	0.7527722	Organic
Elevation	GH39	110.1752	16.6197	0.7408	0.676	0.0277	0.4030018	Organic
Elevation	GH38	153.8558	15.4149	0.2035	0.0043	0.3693	0.6697938	Organic
Elevation	GH4	141.0485	-1.6316	0.0148	-0.2315	0.8186	0.9119387	Organic
Elevation	GH42	52.5554	3.2205	0.4985	0.3731	0.1169	0.4331128	Organic
Elevation	GH43	133.3589	14.3807	0.2914	0.1142	0.269	0.5823417	Organic
Elevation	GH44	32.7913	7.6884	0.8804	0.8505	0.0056	0.2319836	Organic
Elevation	GH45	0.5846	0.1316	0.4075	0.2593	0.1726	0.4721471	Organic
Elevation	GH47	14.6845	-0.3726	0.0411	-0.1986	0.7	0.8617823	Organic
Elevation	GH46	1.4741	-0.2581	0.4092	0.2615	0.1714	0.4721471	Organic
Elevation	GH48	10.4606	1.2186	0.4678	0.3347	0.1341	0.4506549	Organic
Elevation	GH49	0.0919	-0.0078	0.1149	-0.1064	0.5111	0.7240116	Organic
Elevation	GH5	172.0215	8.4802	0.6123	0.5154	0.0658	0.4030018	Organic
Elevation	GH50	7.7274	0.7019	0.1749	-0.0314	0.4092	0.7033579	Organic
Elevation	GH51	136.9965	20.0071	0.6293	0.5367	0.0597	0.4030018	Organic
Elevation	GH54	33.242	2.5849	0.0487	-0.1891	0.6743	0.8407703	Organic
Elevation	GH53	65.4769	10.2688	0.7645	0.7057	0.0227	0.4030018	Organic
Elevation	GH55	145.3713	10.6197	0.1101	-0.1123	0.5205	0.7240116	Organic
Elevation	GH56	0.0085	0.0047	0.22	0.025	0.3481	0.6697938	Organic
Elevation	GH57	148.2848	12.6543	0.5531	0.4413	0.0901	0.4177614	Organic
Elevation	GH58	0.0299	0.0015	0.0252	-0.2185	0.7638	0.884587	Organic
Elevation	GH59	11.8995	0.5602	0.0217	-0.2228	0.7805	0.8935626	Organic
Elevation	GH6	21.9717	1.8886	0.215	0.0187	0.3544	0.6697938	Organic
Elevation	GH62	3.402	0.98	0.6477	0.5596	0.0534	0.4030018	Organic
Elevation	GH63	18.035	2.058	0.3572	0.1965	0.2102	0.5183279	Organic
Elevation	GH64	23.3696	1.6652	0.1728	-0.034	0.4124	0.7033579	Organic
Elevation	GH65	99.333	-5.0412	0.1333	-0.0834	0.4767	0.7240116	Organic
Elevation	GH66	1.6072	0.4563	0.6157	0.5197	0.0645	0.4030018	Organic
Elevation	GH68	0.3029	-0.0216	0.0777	-0.1529	0.5928	0.7851339	Organic

Elevation	GH7	0.8798	0.0196	0.0077	-0.2404	0.8686	0.9203926	Organic
Elevation	GH70	0.0313	0.0106	0.4351	0.2938	0.1541	0.4721471	Organic
Elevation	GH71	4.8837	-0.3928	0.0597	-0.1754	0.6408	0.8267181	Organic
Elevation	GH72	30.0891	0.6453	0.138	-0.0775	0.4684	0.7240116	Organic
Elevation	GH73	36.2954	0.294	0.062	-0.1726	0.6344	0.822686	Organic
Elevation	GH75	2.0444	0.0648	0.0274	-0.2157	0.7539	0.884587	Organic
Elevation	GH74	16.7603	3.0683	0.5771	0.4714	0.0797	0.4177614	Organic
Elevation	GH76	18.3675	0.067	0.0005	-0.2494	0.9671	0.9789372	Organic
Elevation	GH78	173.1756	13.2295	0.2692	0.0865	0.2916	0.6100665	Organic
Elevation	GH79	33.6982	0.3792	0.0071	-0.2411	0.8738	0.9212573	Organic
Elevation	GH8	19.2144	2.3429	0.3824	0.228	0.1907	0.5048941	Organic
Elevation	GH80	0	0	#VALUE!	#VALUE!	#VALUE!	NA	Organic
Elevation	GH81	5.1377	0.125	0.0139	-0.2326	0.8237	0.9119387	Organic
Elevation	GH84	4.79	0.6482	0.6319	0.5399	0.0588	0.4030018	Organic
Elevation	GH85	0.5304	0.0873	0.6687	0.5859	0.0468	0.4030018	Organic
Elevation	GH86	1.1757	-0.0563	0.0555	-0.1806	0.6531	0.833813	Organic
Elevation	GH87	78.9746	5.583	0.2185	0.0232	0.3498	0.6697938	Organic
Elevation	GH88	23.1746	1.4646	0.6337	0.5421	0.0581	0.4030018	Organic
Elevation	GH89	9.9199	0.6232	0.053	-0.1838	0.6608	0.8352079	Organic
Elevation	GH91	0.0458	-0.0054	0.0214	-0.2233	0.7823	0.8935626	Organic
Elevation	GH90	0.0009	0.001	0.2	0	0.3739	0.6697938	Organic
Elevation	GH9	60.9971	7.0484	0.3957	0.2446	0.1809	0.4843953	Organic
Elevation	GH92	121.0279	6.2138	0.0459	-0.1926	0.6834	0.8466526	Organic
Elevation	GH93	18.7765	-0.3582	0.0101	-0.2374	0.8497	0.9158897	Organic
Elevation	GH94	39.1359	8.0588	0.4269	0.2836	0.1594	0.4721471	Organic
Elevation	GH95	57.25	5.9332	0.3592	0.199	0.2087	0.5183279	Organic
Elevation	GH96	0.1511	0.052	0.6367	0.5459	0.0571	0.4030018	Organic
Elevation	GH97	30.1161	1.6198	0.4273	0.2842	0.1591	0.4721471	Organic
Elevation	GH98	0.1755	-0.0305	0.0905	-0.1368	0.5623	0.7527722	Organic
Elevation	GH99	9.8752	-0.2212	0.0328	-0.209	0.7314	0.8755398	Organic
Elevation	GT1	150.285	-17.515	0.4896	0.3619	0.1217	0.4331128	Organic
Elevation	GT10	3.0892	0.6737	0.606	0.5075	0.0682	0.4030018	Organic
Elevation	GT12	0.0472	0.0074	0.1243	-0.0946	0.493	0.7240116	Organic
Elevation	GT14	2.1246	0.2133	0.2055	0.0069	0.3666	0.6697938	Organic
Elevation	GT11	5.7255	1.1805	0.7176	0.6471	0.0333	0.4030018	Organic
Elevation	GT13	0.2735	0.0522	0.1137	-0.1078	0.5133	0.7240116	Organic
Elevation	GT15	2.516	-0.2934	0.3714	0.2142	0.1991	0.5064389	Organic
Elevation	GT16	0.0801	0.0221	0.5945	0.4932	0.0726	0.4030018	Organic
Elevation	GT17	1.2195	0.0685	0.121	-0.0987	0.4992	0.7240116	Organic
Elevation	GT18	0.0298	0.0003	0.0018	-0.2477	0.9363	0.9593766	Organic
Elevation	GT2	1761.7613	87.4469	0.6906	0.6132	0.0404	0.4030018	Organic
Elevation	GT19	83.6411	5.5741	0.8314	0.7893	0.0113	0.3131067	Organic
Elevation	GT20	146.1397	-2.5266	0.1253	-0.0933	0.4911	0.7240116	Organic
Elevation	GT21	93.1775	10.7926	0.5484	0.4355	0.0922	0.4177614	Organic
Elevation	GT22	5.2116	0.0431	0.0308	-0.2115	0.7393	0.8787113	Organic
Elevation	GT23	0.3811	0.1053	0.3138	0.1422	0.2476	0.5492287	Organic
Elevation	GT24	3.5024	0.1946	0.0724	-0.1595	0.6062	0.7943762	Organic
Elevation	GT25	7.2869	0.5838	0.2746	0.0932	0.2859	0.6033942	Organic
Elevation	GT26	148.5105	9.0827	0.8068	0.7585	0.015	0.3738301	Organic
Elevation	GT27	15.1224	1.1659	0.3999	0.2498	0.1779	0.4815078	Organic
Elevation	GT28	177.4443	11.9853	0.912	0.89	0.003	0.2319836	Organic
Elevation	GT29	3.4324	0.191	0.0654	-0.1682	0.6247	0.8143886	Organic
Elevation	GT30	121.5074	8.7073	0.9033	0.8791	0.0036	0.2319836	Organic
Elevation	GT3	3.9902	0.5643	0.3261	0.1576	0.2366	0.5492287	Organic
Elevation	GT33	1.2253	-0.0639	0.3175	0.1469	0.2442	0.5492287	Organic

Elevation	GT34	1.1675	-0.2024	0.511	0.3887	0.1104	0.4331128	Organic
Elevation	GT32	11.1421	-1.2269	0.6331	0.5414	0.0583	0.4030018	Organic
Elevation	GT31	2.1893	0.2458	0.0986	-0.1267	0.5444	0.7489486	Organic
Elevation	GT35	367.4633	36.8697	0.8836	0.8545	0.0053	0.2319836	Organic
Elevation	GT37	0.0155	-0.0072	0.2127	0.0159	0.3573	0.6697938	Organic
Elevation	GT39	21.5812	-1.4139	0.2804	0.1004	0.28	0.6005285	Organic
Elevation	GT4	1706.1942	38.3778	0.1163	-0.1046	0.5083	0.7240116	Organic
Elevation	GT41	451.5852	74.091	0.8469	0.8086	0.0093	0.2888557	Organic
Elevation	GT40	0.0022	0.0024	0.4951	0.3689	0.1187	0.4331128	Organic
Elevation	GT42	0.0544	0.006	0.2109	0.0137	0.3595	0.6697938	Organic
Elevation	GT43	0.1274	0.0503	0.4861	0.3576	0.1236	0.4336236	Organic
Elevation	GT47	80.6211	9.3689	0.1228	-0.0965	0.4958	0.7240116	Organic
Elevation	GT45	0.1867	0.0313	0.4278	0.2847	0.1588	0.4721471	Organic
Elevation	GT44	0.0123	-0.0026	0.1413	-0.0733	0.4626	0.7240116	Organic
Elevation	GT48	4.9851	-0.012	0.0001	-0.2498	0.983	0.9904092	Organic
Elevation	GT49	0.2847	0.0069	0.004	-0.245	0.905	0.9389516	Organic
Elevation	GT5	106.4158	5.3878	0.5483	0.4354	0.0923	0.4177614	Organic
Elevation	GT50	1.0262	0.0269	0.0128	-0.234	0.8309	0.9140595	Organic
Elevation	GT53	25.4939	-5.855	0.6682	0.5853	0.0469	0.4030018	Organic
Elevation	GT52	0	0	#VALUE!	#VALUE!	#VALUE!	NA	Organic
Elevation	GT51	518.0831	-6.2418	0.0139	-0.2326	0.824	0.9119387	Organic
Elevation	GT54	0.0816	0.0044	0.0068	-0.2415	0.8769	0.9212573	Organic
Elevation	GT57	1.9599	-0.0761	0.0194	-0.2258	0.7926	0.9011832	Organic
Elevation	GT56	0.5203	0.0959	0.3691	0.2114	0.2008	0.5064389	Organic
Elevation	GT55	2.6159	-0.6115	0.475	0.3438	0.1299	0.4490967	Organic
Elevation	GT58	0.7939	-0.0196	0.0118	-0.2353	0.8378	0.9149151	Organic
Elevation	GT6	0.3853	0.0797	0.5889	0.4862	0.0749	0.4051878	Organic
Elevation	GT59	0.5119	-0.0628	0.2094	0.0117	0.3615	0.6697938	Organic
Elevation	GT60	3.4349	0.5103	0.173	-0.0337	0.412	0.7033579	Organic
Elevation	GT61	0.5101	0.1242	0.3798	0.2247	0.1926	0.5048941	Organic
Elevation	GT62	2.3868	-0.1243	0.1848	-0.0189	0.3948	0.6948393	Organic
Elevation	GT64	0.751	0.0385	0.2213	0.0266	0.3465	0.6697938	Organic
Elevation	GT65	0.0811	0.0099	0.0516	-0.1856	0.6653	0.8366295	Organic
Elevation	GT66	16.8699	1.0732	0.4933	0.3666	0.1197	0.4331128	Organic
Elevation	GT69	1.138	-0.1241	0.1204	-0.0995	0.5005	0.7240116	Organic
Elevation	GT68	0.0598	0.0061	0.0335	-0.2081	0.7284	0.8755398	Organic
Elevation	GT7	6.1602	0.9323	0.7503	0.6879	0.0257	0.4030018	Organic
Elevation	GT72	0.0003	-0.0003	0.2	0.00E+00	0.3739	0.6697938	Organic
Elevation	GT73	0.071	0.0095	0.0484	-0.1895	0.6753	0.8407703	Organic
Elevation	GT71	1.6293	0.1157	0.1668	-0.0415	0.4214	0.7137853	Organic
Elevation	GT70	0.7452	0.0174	0.0914	-0.1357	0.5603	0.7527722	Organic
Elevation	GT74	0.8293	0.0668	0.1231	-0.0962	0.4954	0.7240116	Organic
Elevation	GT75	0.956	0.1702	0.5732	0.4665	0.0813	0.4177614	Organic
Elevation	GT76	1.3751	-0.575	0.218	0.0224	0.3506	0.6697938	Organic
Elevation	GT77	0.6069	-0.1648	0.1524	-0.0595	0.4441	0.7240116	Organic
Elevation	GT78	0.0028	-0.003	0.4327	0.2909	0.1556	0.4721471	Organic
Elevation	GT8	10.5137	0.0825	0.0142	-0.2323	0.8223	0.9119387	Organic
Elevation	GT80	6.3954	0.7855	0.594	0.4925	0.0728	0.4030018	Organic
Elevation	GT82	0.3053	0.0259	0.0834	-0.1458	0.5789	0.7708648	Organic
Elevation	GT84	319.0374	63.8532	0.8502	0.8127	0.0089	0.2888557	Organic
Elevation	GT81	23.6857	-0.5031	0.0351	-0.2061	0.7223	0.8730702	Organic
Elevation	GT83	177.9561	4.413	0.1291	-0.0886	0.4842	0.7240116	Organic
Elevation	GT85	3.5991	-0.8379	0.4936	0.367	0.1195	0.4331128	Organic
Elevation	GT87	20.2064	-0.293	0.0125	-0.2344	0.8333	0.9140595	Organic
Elevation	GT88	0.0469	-0.0055	0.0259	-0.2176	0.7605	0.884587	Organic

Elevation	GT89	5.8171	-0.8651	0.5326	0.4157	0.0997	0.4205944	Organic
Elevation	GT90	3.3542	0.1717	0.0377	-0.2029	0.7123	0.8694859	Organic
Elevation	GT9	154.7911	10.3291	0.6701	0.5876	0.0464	0.4030018	Organic
Elevation	GT91	0.0217	-0.0068	0.3684	0.2105	0.2014	0.5064389	Organic
Elevation	GT93	0.3645	-0.0107	0.1112	-0.111	0.5184	0.7240116	Organic
Elevation	GT94	1.0193	0.228	0.6994	0.6242	0.038	0.4030018	Organic
Elevation	GT92	0.2101	0.0209	0.1151	-0.1061	0.5105	0.7240116	Organic
Elevation	GT95	6.3578	0.4416	0.2916	0.1145	0.2688	0.5823417	Organic
Elevation	PL1	14.0854	1.7536	0.6019	0.5023	0.0698	0.4030018	Organic
Elevation	PL10	5.2376	0.7966	0.1936	-0.008	0.3826	0.6804843	Organic
Elevation	PL11	2.8656	0.9415	0.4401	0.3001	0.1509	0.4721471	Organic
Elevation	PL12	22.902	3.6971	0.7584	0.698	0.0239	0.4030018	Organic
Elevation	PL13	0.0005	-0.0006	0.2	0.00E+00	0.3739	0.6697938	Organic
Elevation	PL14	0.8538	-0.1166	0.1537	-0.0579	0.4421	0.7240116	Organic
Elevation	PL15	0.1739	0.0243	0.263	0.0788	0.2982	0.6186818	Organic
Elevation	PL17	0.9434	0.0334	0.026	-0.2175	0.7603	0.884587	Organic
Elevation	PL18	0	0	#VALUE!	#VALUE!	#VALUE!	NA	Organic
Elevation	PL16	0.5082	0.0751	0.6788	0.5985	0.0438	0.4030018	Organic
Elevation	PL2	0.3036	0.1224	0.7632	0.704	0.0229	0.4030018	Organic
Elevation	PL20	0.0658	-0.0049	0.0558	-0.1803	0.6523	0.833813	Organic
Elevation	PL21	0.5253	0.0753	0.1116	-0.1106	0.5176	0.7240116	Organic
Elevation	PL22	12.3762	2.0058	0.5674	0.4592	0.0838	0.4177614	Organic
Elevation	PL23	0.02	0.0033	0.6975	0.6218	0.0385	0.4030018	Organic
Elevation	PL3	0.0966	-0.0026	0.0042	-0.2447	0.9028	0.9389516	Organic
Elevation	PL4	26.4684	6.63	0.3134	0.1417	0.248	0.5492287	Organic
Elevation	PL5	4.576	0.3802	0.0296	-0.213	0.7446	0.8787113	Organic
Elevation	PL6	1.1647	0.2777	0.5343	0.4178	0.0989	0.4205944	Organic
Elevation	PL7	3.6063	0.0627	0.0052	-0.2435	0.8921	0.933332	Organic
Elevation	PL8	3.0069	0.5209	0.3332	0.1665	0.2303	0.5410616	Organic
Elevation	PL9	28.8683	3.6671	0.51	0.3875	0.1109	0.4331128	Organic
DNA	amoA_A_Cluster26	0.004	-0.0036	0.2224	0.1815	0.0309	0.1739295	Both
DNA	amoA_A_Cluster45	0.0051	-0.004	0.2156	0.1743	0.034	0.1773182	Both
DNA	amoA_B_Cluster0	0.1099	-0.0317	0.3914	0.3594	0.0024	0.075132	Both
DNA	amoA_B_Cluster1	0.0172	-0.0065	0.1446	0.0996	0.0891	0.2649811	Both
DNA	amoA_B_Cluster10	0.0422	-0.0196	0.4073	0.3761	0.0019	0.0682806	Both
DNA	amoA_B_Cluster11	0.0377	-0.0135	0.1752	0.1317	0.059	0.2248984	Both
DNA	amoA_B_Cluster12	0.0807	-0.0225	0.2808	0.2429	0.0135	0.1432166	Both
DNA	amoA_B_Cluster13	0.5271	-0.2178	0.289	0.2516	0.012	0.1383265	Both
DNA	amoA_B_Cluster14	0.0275	-0.0142	0.239	0.199	0.0245	0.1739295	Both
DNA	amoA_B_Cluster15	0.0318	-0.0122	0.1918	0.1493	0.0471	0.2041775	Both
DNA	amoA_B_Cluster2	0.1721	-0.0762	0.3189	0.283	0.0077	0.1173335	Both
DNA	amoA_B_Cluster20	0.0058	-0.0022	0.0578	0.0082	0.294	0.4777812	Both
DNA	amoA_B_Cluster23	0.0507	-0.0155	0.1956	0.1532	0.0447	0.1995936	Both
DNA	amoA_B_Cluster3	0.0182	-0.0047	0.0572	0.0076	0.2964	0.4804991	Both
DNA	amoA_B_Cluster4	0.0125	-0.0056	0.1452	0.1002	0.0883	0.2649811	Both
DNA	amoA_B_Cluster5	0.0928	-0.0272	0.3216	0.2859	0.0073	0.1173335	Both
DNA	amoA_B_Cluster6	0.0233	-0.007	0.1711	0.1275	0.0623	0.2248984	Both
DNA	amoA_B_Cluster7	0.0179	-0.0037	0.0362	-0.0145	0.4088	0.568969	Both
DNA	amoA_B_Cluster8	0.0207	-0.0059	0.0648	0.0156	0.2654	0.4499566	Both
DNA	amoA_B_Cluster9	0.0549	-0.0184	0.1275	0.0816	0.1121	0.288473	Both
DNA	nifh_Cluster0	0.0332	0.0051	0.1029	0.0556	0.1563	0.3294129	Both
DNA	nifh_Cluster10	0.005	0.0042	0.1654	0.1215	0.0673	0.2307465	Both
DNA	nifh_Cluster100	0.0019	0.0004	0.0133	-0.0387	0.619	0.7365087	Both
DNA	nifh_Cluster103	0.0383	-0.0125	0.2334	0.1931	0.0265	0.1739295	Both
DNA	nifh_Cluster1034	0.0262	-0.0105	0.1204	0.0741	0.1234	0.3060007	Both

DNA	nifh_Cluster105	0.013	-0.0056	0.1309	0.0852	0.107	0.2821784	Both
DNA	nifh_Cluster108	0.0083	-0.0021	0.0594	0.0099	0.287	0.4716106	Both
DNA	nifh_Cluster1099	0.066	0.0387	0.3476	0.3133	0.0049	0.0956583	Both
DNA	nifh_Cluster11	0.0021	-0.0006	0.0381	-0.0125	0.3965	0.560972	Both
DNA	nifh_Cluster1112	0.0029	-0.0011	0.0472	-0.0029	0.3441	0.5218882	Both
DNA	nifh_Cluster112	0.0112	0.0028	0.057	0.0073	0.2974	0.4807995	Both
DNA	nifh_Cluster113	0.0327	-0.0039	0.0367	-0.014	0.4054	0.568969	Both
DNA	nifh_Cluster114	0.0332	0.0007	0.0027	-0.0498	0.8229	0.8857525	Both
DNA	nifh_Cluster1141	0.0251	-0.0099	0.175	0.1316	0.0591	0.2248984	Both
DNA	nifh_Cluster115	0.0095	0.0013	0.0307	-0.0204	0.4477	0.6039013	Both
DNA	nifh_Cluster116	0.0775	-0.0107	0.0509	0.0009	0.3255	0.5116244	Both
DNA	nifh_Cluster1163	0.0044	-0.0011	0.05	0.00E+00	0.3298	0.5147852	Both
DNA	nifh_Cluster1179	0.0031	-0.001	0.0346	-0.0162	0.4196	0.575754	Both
DNA	nifh_Cluster1182	0.0204	0.0005	0.0025	-0.0501	0.8312	0.8857525	Both
DNA	nifh_Cluster1183	0.0066	0.0035	0.3297	0.2944	0.0065	0.1093053	Both
DNA	nifh_Cluster1197	0.0115	-0.0012	0.0133	-0.0387	0.6192	0.7365087	Both
DNA	nifh_Cluster1199	0.0046	-0.0006	0.0147	-0.0372	0.6012	0.7209375	Both
DNA	nifh_Cluster120	0.0149	-0.0087	0.0946	0.0469	0.1751	0.3550164	Both
DNA	nifh_Cluster1203	0.0155	-0.0076	0.1501	0.1054	0.0827	0.2577293	Both
DNA	nifh_Cluster1206	0.0043	-0.0013	0.0553	0.0056	0.3047	0.491248	Both
DNA	nifh_Cluster1207	0.0074	-0.0023	0.059	0.0095	0.2886	0.4716106	Both
DNA	nifh_Cluster1209	0.1506	-0.0024	0.0019	-0.0506	0.8517	0.9005863	Both
DNA	nifh_Cluster121	0.0095	-0.0061	0.1267	0.0807	0.1133	0.288473	Both
DNA	nifh_Cluster1211	0.0056	-0.0006	0.0093	-0.0428	0.6767	0.7767833	Both
DNA	nifh_Cluster1213	0.0026	-0.0007	0.0362	-0.0145	0.4089	0.568969	Both
DNA	nifh_Cluster122	0.0029	-0.0024	0.227	0.1863	0.029	0.1739295	Both
DNA	nifh_Cluster123	0.0095	0.0027	0.0261	-0.0251	0.4839	0.6288631	Both
DNA	nifh_Cluster1236	0.0234	-0.0153	0.1712	0.1276	0.0622	0.2248984	Both
DNA	nifh_Cluster1238	0.0028	-0.0009	0.041	-0.0095	0.3787	0.5430503	Both
DNA	nifh_Cluster124	0.0091	0.0006	0.0043	-0.0481	0.7777	0.8592002	Both
DNA	nifh_Cluster1261	0.002	-0.0007	0.0337	-0.0171	0.4255	0.5797596	Both
DNA	nifh_Cluster1262	0.0065	-0.0023	0.0717	0.0228	0.2407	0.4316457	Both
DNA	nifh_Cluster1266	0.0094	0.0044	0.1942	0.1518	0.0455	0.1995936	Both
DNA	nifh_Cluster1267	0.0562	0.032	0.2739	0.2356	0.0149	0.1491401	Both
DNA	nifh_Cluster127	0.0026	-0.002	0.1497	0.1049	0.0832	0.2577293	Both
DNA	nifh_Cluster1278	0.1602	-0.069	0.2768	0.2387	0.0143	0.1454166	Both
DNA	nifh_Cluster128	0.0036	-0.0008	0.0231	-0.0284	0.5111	0.6541736	Both
DNA	nifh_Cluster1289	0.0039	0.0005	0.0103	-0.0418	0.6621	0.76649	Both
DNA	nifh_Cluster129	0.0077	0.00E+00	0.00E+00	-0.0526	0.9863	0.9951643	Both
DNA	nifh_Cluster1292	0.0086	-0.0023	0.0531	0.0033	0.3148	0.5019136	Both
DNA	nifh_Cluster130	0.0059	-0.0019	0.0263	-0.025	0.4826	0.6288631	Both
DNA	nifh_Cluster1305	0.0048	0.0012	0.0518	0.0019	0.321	0.507764	Both
DNA	nifh_Cluster1306	0.005	-0.0021	0.1181	0.0717	0.1272	0.3083641	Both
DNA	nifh_Cluster131	0.0051	-0.003	0.1152	0.0686	0.1323	0.3101587	Both
DNA	nifh_Cluster1319	0.0036	-0.0007	0.0157	-0.0361	0.5882	0.7112892	Both
DNA	nifh_Cluster132	0.0064	0.0011	0.0142	-0.0376	0.6065	0.7258114	Both
DNA	nifh_Cluster1320	0.0025	-0.0004	0.0057	-0.0467	0.7458	0.8381634	Both
DNA	nifh_Cluster1323	0.0023	0.0007	0.0624	0.0131	0.2747	0.4578801	Both
DNA	nifh_Cluster1324	0.03	0.0081	0.0886	0.0406	0.19	0.3737855	Both
DNA	nifh_Cluster1336	0.0153	0.0012	0.0215	-0.0299	0.5254	0.6609657	Both
DNA	nifh_Cluster1340	0.0363	-0.0047	0.0086	-0.0436	0.6895	0.7867879	Both
DNA	nifh_Cluster1345	0.0483	0.0297	0.3191	0.2833	0.0076	0.1173335	Both
DNA	nifh_Cluster1370	0.0021	-0.0004	0.016	-0.0358	0.585	0.7101868	Both
DNA	nifh_Cluster1375	0.0018	-0.0004	0.0179	-0.0337	0.5626	0.6901421	Both
DNA	nifh_Cluster139	0.0041	0.0018	0.0824	0.0341	0.2071	0.392904	Both

DNA	nifh_Cluster140	0.0043	-0.0015	0.0808	0.0324	0.2118	0.3968292	Both
DNA	nifh_Cluster141	0.0677	-0.0318	0.496	0.4695	0.0004	0.0196047	Both
DNA	nifh_Cluster148	0.0046	-0.0012	0.0202	-0.0314	0.5387	0.6719267	Both
DNA	nifh_Cluster1480	0.001	0.001	0.2175	0.1764	0.0331	0.1772493	Both
DNA	nifh_Cluster152	0.0599	-0.0011	0.001	-0.0516	0.8927	0.9338979	Both
DNA	nifh_Cluster156	0.0659	0.0328	0.4387	0.4092	0.0011	0.0485811	Both
DNA	nifh_Cluster1562	0.0543	0.049	0.5493	0.5255	0.0001	0.0159293	Both
DNA	nifh_Cluster158	0.0144	-0.0058	0.1169	0.0704	0.1292	0.3084324	Both
DNA	nifh_Cluster16	0.0019	-0.0006	0.0238	-0.0275	0.504	0.6478715	Both
DNA	nifh_Cluster166	0.0172	0.0052	0.0283	-0.0228	0.466	0.6164083	Both
DNA	nifh_Cluster205	0.0122	0.0024	0.0394	-0.0111	0.3881	0.5517608	Both
DNA	nifh_Cluster21	0.0021	-0.0013	0.1319	0.0862	0.1056	0.2807408	Both
DNA	nifh_Cluster222	0.006	-0.0009	0.006	-0.0463	0.7389	0.8351087	Both
DNA	nifh_Cluster225	0.0189	-0.0006	0.0025	-0.05	0.8299	0.8857525	Both
DNA	nifh_Cluster230	0.0042	-0.0019	0.1419	0.0967	0.0924	0.2658115	Both
DNA	nifh_Cluster231	0.0022	0.0012	0.1084	0.0615	0.1449	0.3132027	Both
DNA	nifh_Cluster236	0.0027	-0.0016	0.1743	0.1308	0.0597	0.2248984	Both
DNA	nifh_Cluster237	0.0058	-0.0023	0.1285	0.0826	0.1106	0.2861779	Both
DNA	nifh_Cluster243	0.0333	0.0085	0.0674	0.0183	0.2559	0.4414059	Both
DNA	nifh_Cluster2432	0.0436	0.0268	0.3747	0.3418	0.0032	0.0827207	Both
DNA	nifh_Cluster2433	0.0032	0.0018	0.0919	0.0441	0.1816	0.3656187	Both
DNA	nifh_Cluster246	0.0057	-0.0022	0.0737	0.0249	0.2339	0.422995	Both
DNA	nifh_Cluster25	0.0295	0.0165	0.516	0.4906	0.0002	0.0160348	Both
DNA	nifh_Cluster256	0.0029	-0.0002	0.0023	-0.0503	0.8382	0.8910742	Both
DNA	nifh_Cluster264	0.0029	0.0013	0.1236	0.0775	0.1181	0.2976598	Both
DNA	nifh_Cluster265	0.0736	0.0091	0.0637	0.0145	0.2695	0.4537459	Both
DNA	nifh_Cluster267	0.0109	0.0049	0.1468	0.1019	0.0865	0.2643381	Both
DNA	nifh_Cluster268	0.0111	0.0019	0.0461	-0.0041	0.3499	0.5280328	Both
DNA	nifh_Cluster269	0.023	-0.0085	0.1051	0.058	0.1517	0.3219818	Both
DNA	nifh_Cluster272	0.0041	0.0052	0.5358	0.5114	0.0002	0.0159293	Both
DNA	nifh_Cluster274	0.0032	0.0049	0.2227	0.1818	0.0308	0.1739295	Both
DNA	nifh_Cluster275	0.0036	-0.0018	0.1577	0.1134	0.0746	0.2475966	Both
DNA	nifh_Cluster278	0.0077	0.0045	0.2568	0.2177	0.0191	0.1739295	Both
DNA	nifh_Cluster279	0.006	0.0004	0.0068	-0.0455	0.7225	0.8181616	Both
DNA	nifh_Cluster28	0.002	0.0004	0.0172	-0.0345	0.5705	0.6954683	Both
DNA	nifh_Cluster280	0.0031	-0.0009	0.019	-0.0326	0.5512	0.6820943	Both
DNA	nifh_Cluster282	0.0052	0.0006	0.0105	-0.0416	0.6583	0.7645463	Both
DNA	nifh_Cluster287	0.0022	-0.0007	0.043	-0.0074	0.3672	0.5363013	Both
DNA	nifh_Cluster290	0.011	0.0015	0.0189	-0.0327	0.5524	0.6820943	Both
DNA	nifh_Cluster297	0.0029	-0.0025	0.1245	0.0784	0.1167	0.2954394	Both
DNA	nifh_Cluster299	0.0026	0.001	0.0453	-0.0049	0.3541	0.5299245	Both
DNA	nifh_Cluster303	0.002	0.0005	0.0177	-0.034	0.5649	0.6914184	Both
DNA	nifh_Cluster304	0.0024	-0.0002	0.0038	-0.0486	0.7903	0.8666895	Both
DNA	nifh_Cluster313	0.0103	-0.0042	0.0578	0.0082	0.2938	0.4777812	Both
DNA	nifh_Cluster314	0.0022	-0.0007	0.0335	-0.0173	0.4268	0.5802601	Both
DNA	nifh_Cluster317	0.0071	-0.0035	0.1842	0.1412	0.0522	0.2139662	Both
DNA	nifh_Cluster32	0.0033	-0.0007	0.028	-0.0232	0.4685	0.6184184	Both
DNA	nifh_Cluster320	12.7419	-2.3235	0.5477	0.5239	0.0001	0.0159293	Both
DNA	nifh_Cluster334	0.0063	-0.0033	0.0714	0.0226	0.2415	0.4317378	Both
DNA	nifh_Cluster335	0.0075	0.0056	0.1142	0.0675	0.1341	0.3101587	Both
DNA	nifh_Cluster338	0.0123	-0.0036	0.1022	0.0549	0.1578	0.3312607	Both
DNA	nifh_Cluster340	0.007	0.0091	0.2414	0.2015	0.0237	0.1739295	Both
DNA	nifh_Cluster341	0.0023	0.0005	0.013	-0.039	0.6232	0.7397796	Both
DNA	nifh_Cluster35	0.0078	-0.0013	0.0308	-0.0202	0.4466	0.6039013	Both
DNA	nifh_Cluster351	0.0025	-0.0017	0.1203	0.074	0.1234	0.3060007	Both

DNA	nifh_Cluster3574	0.1093	-0.0222	0.1339	0.0883	0.1028	0.279396	Both
DNA	nifh_Cluster3691	0.0174	-0.0056	0.0611	0.0117	0.2799	0.4638595	Both
DNA	nifh_Cluster37	0.0329	-0.0078	0.0438	-0.0065	0.3624	0.5345994	Both
DNA	nifh_Cluster374	0.0021	-0.0002	0.0024	-0.0501	0.8317	0.8857525	Both
DNA	nifh_Cluster38	0.0009	0.0002	0.0084	-0.0438	0.6923	0.7869536	Both
DNA	nifh_Cluster382	0.0016	-0.0014	0.0986	0.0512	0.1656	0.3415803	Both
DNA	nifh_Cluster384	0.0019	-0.0007	0.0433	-0.0071	0.3655	0.5363013	Both
DNA	nifh_Cluster386	0.0035	-0.0005	0.0059	-0.0464	0.7411	0.836004	Both
DNA	nifh_Cluster389	0.0031	0.0004	0.0058	-0.0466	0.7434	0.8370141	Both
DNA	nifh_Cluster394	0.002	-0.0014	0.0824	0.0342	0.2069	0.392904	Both
DNA	nifh_Cluster40	0.0012	-0.0008	0.1166	0.0701	0.1299	0.3084324	Both
DNA	nifh_Cluster43	0.0108	0.00E+00	0.00E+00	-0.0526	0.9852	0.9951643	Both
DNA	nifh_Cluster434	0.0035	-0.0017	0.0836	0.0354	0.2035	0.3898921	Both
DNA	nifh_Cluster440	0.0133	-0.0057	0.1106	0.0638	0.1408	0.3122895	Both
DNA	nifh_Cluster462	0.0034	-0.0018	0.0879	0.0399	0.1919	0.3748879	Both
DNA	nifh_Cluster470	0.0152	0.0012	0.0085	-0.0437	0.6908	0.7867879	Both
DNA	nifh_Cluster49	0.0213	-0.0006	0.0004	-0.0522	0.934	0.9601089	Both
DNA	nifh_Cluster499	0.0026	-0.0006	0.0224	-0.029	0.517	0.6558655	Both
DNA	nifh_Cluster5	0.0256	-0.0007	0.0028	-0.0497	0.8198	0.8842243	Both
DNA	nifh_Cluster506	0.0048	0.00E+00	0.00E+00	-0.0526	0.9956	0.9984133	Both
DNA	nifh_Cluster51	0.0028	-0.0003	0.004	-0.0485	0.7863	0.8638984	Both
DNA	nifh_Cluster52	0.0336	-0.0111	0.0644	0.0152	0.267	0.4512985	Both
DNA	nifh_Cluster525	0.0033	-0.0022	0.0768	0.0283	0.2238	0.4138545	Both
DNA	nifh_Cluster531	0.0258	0.0203	0.5371	0.5127	0.0002	0.0159293	Both
DNA	nifh_Cluster539	0.0048	-0.0008	0.0054	-0.047	0.752	0.8435283	Both
DNA	nifh_Cluster578	0.0481	-0.0122	0.1087	0.0618	0.1444	0.3132027	Both
DNA	nifh_Cluster58	0.3542	0.2136	0.3672	0.3339	0.0036	0.0827207	Both
DNA	nifh_Cluster60	0.0089	-0.0038	0.1106	0.0638	0.1408	0.3122895	Both
DNA	nifh_Cluster61	0.0025	0.0006	0.0306	-0.0204	0.4483	0.6039013	Both
DNA	nifh_Cluster65	0.009	0.0017	0.0188	-0.0328	0.5529	0.6820943	Both
DNA	nifh_Cluster686	0.0009	-0.0004	0.0312	-0.0198	0.4436	0.6016567	Both
DNA	nifh_Cluster69	0.009	-0.0026	0.0424	-0.008	0.3704	0.5382162	Both
DNA	nifh_Cluster70	0.0111	-0.006	0.0818	0.0335	0.2087	0.3946468	Both
DNA	nifh_Cluster717	0.003	0.0021	0.318	0.2821	0.0078	0.1173335	Both
DNA	nifh_Cluster725	0.0058	0.003	0.1964	0.1541	0.0442	0.1995936	Both
DNA	nifh_Cluster727	0.0039	-0.0012	0.044	-0.0063	0.3614	0.5345994	Both
DNA	nifh_Cluster73	0.0106	-0.0043	0.0955	0.0479	0.1728	0.3515669	Both
DNA	nifh_Cluster74	0.0049	0.0009	0.0102	-0.0419	0.6639	0.76649	Both
DNA	nifh_Cluster748	0.0081	-0.0024	0.1148	0.0682	0.133	0.3101587	Both
DNA	nifh_Cluster749	0.0071	0.0033	0.2218	0.1809	0.0312	0.1739295	Both
DNA	nifh_Cluster755	0.0102	-0.0033	0.0675	0.0184	0.2556	0.4414059	Both
DNA	nifh_Cluster76	0.0029	-0.0006	0.0223	-0.0291	0.518	0.6558655	Both
DNA	nifh_Cluster79	0.0022	0.0002	0.0034	-0.049	0.8017	0.8726701	Both
DNA	nifh_Cluster81	0.0055	-0.0006	0.0033	-0.0492	0.8058	0.8732855	Both
DNA	nifh_Cluster82	0.0011	-0.0008	0.0981	0.0506	0.1669	0.3431235	Both
DNA	nifh_Cluster83	0.0069	0.0044	0.2013	0.1592	0.0414	0.192189	Both
DNA	nifh_Cluster86	0.0135	-0.0032	0.0609	0.0115	0.2808	0.4641491	Both
DNA	nifh_Cluster868	0.0099	-0.0006	0.0049	-0.0475	0.7631	0.8478727	Both
DNA	nifh_Cluster87	0.0024	-0.0008	0.0351	-0.0157	0.4159	0.5733655	Both
DNA	nifh_Cluster88	0.0039	-0.0008	0.015	-0.0369	0.5973	0.7177006	Both
DNA	nifh_Cluster9	0.0078	0.0005	0.0033	-0.0492	0.8058	0.8732855	Both
DNA	nifh_Cluster92	0.0051	-0.0026	0.0718	0.023	0.2402	0.4316457	Both
DNA	nifh_Cluster93	0.0036	0.0009	0.03	-0.021	0.4524	0.6066515	Both
DNA	nifh_Cluster94	0.0047	0.00E+00	0.00E+00	-0.0526	0.9964	0.9984133	Both
DNA	nifh_Cluster95	0.0121	-0.003	0.0277	-0.0235	0.4712	0.6205115	Both

DNA	nifh_Cluster96	0.0123	0.0012	0.0096	-0.0426	0.673	0.7740448	Both
DNA	nifh_Cluster97	0.018	0.012	0.3428	0.3083	0.0053	0.0975584	Both
DNA	nifh_Cluster98	0.0059	0.0015	0.0625	0.0131	0.2745	0.4578801	Both
DNA	nifh_Cluster99	0.0033	0.00E+00	0.00E+00	-0.0526	0.9791	0.9942629	Both
DNA	nirk_Cluster0	5.9009	-1.2425	0.6884	0.672	0.00E+00	0.0019466	Both
DNA	nirk_Cluster1	0.1708	-0.0867	0.4018	0.3703	0.002	0.0705568	Both
DNA	nirk_Cluster10	0.5713	-0.0934	0.1336	0.088	0.1032	0.279396	Both
DNA	nirk_Cluster101	0.0031	-0.0009	0.034	-0.0169	0.424	0.5797596	Both
DNA	nirk_Cluster102	0.0426	-0.0187	0.2501	0.2106	0.021	0.1739295	Both
DNA	nirk_Cluster103	0.0754	-0.032	0.3063	0.2698	0.0093	0.1269241	Both
DNA	nirk_Cluster104	0.0023	-0.0009	0.0473	-0.0028	0.3435	0.5218882	Both
DNA	nirk_Cluster105	0.0946	-0.0447	0.3921	0.3601	0.0024	0.075132	Both
DNA	nirk_Cluster106	0.0592	-0.0278	0.2224	0.1815	0.0309	0.1739295	Both
DNA	nirk_Cluster107	0.0891	-0.0391	0.2972	0.2602	0.0106	0.135128	Both
DNA	nirk_Cluster108	0.0151	-0.0025	0.0251	-0.0262	0.493	0.6375931	Both
DNA	nirk_Cluster109	0.0115	-0.0062	0.2312	0.1908	0.0273	0.1739295	Both
DNA	nirk_Cluster11	0.1401	-0.0117	0.1123	0.0656	0.1375	0.3101587	Both
DNA	nirk_Cluster12	0.094	-0.0284	0.3832	0.3508	0.0028	0.0816864	Both
DNA	nirk_Cluster13	0.0408	-0.0026	0.0267	-0.0246	0.4795	0.627243	Both
DNA	nirk_Cluster14	0.0946	-0.0215	0.2098	0.1682	0.0368	0.1811591	Both
DNA	nirk_Cluster15	0.3358	-0.1471	0.3464	0.312	0.005	0.0956583	Both
DNA	nirk_Cluster16	0.4727	-0.2032	0.3064	0.2699	0.0092	0.1269241	Both
DNA	nirk_Cluster17	0.0998	-0.0037	0.0099	-0.0422	0.6683	0.7700786	Both
DNA	nirk_Cluster18	0.1296	-0.0314	0.1128	0.0661	0.1366	0.3101587	Both
DNA	nirk_Cluster19	0.2776	-0.0332	0.1567	0.1123	0.0756	0.2475966	Both
DNA	nirk_Cluster2	0.2032	-0.003	0.0019	-0.0506	0.8508	0.9005863	Both
DNA	nirk_Cluster20	0.1738	-0.0011	0.0001	-0.0525	0.958	0.9762253	Both
DNA	nirk_Cluster21	0.1532	-0.0211	0.1553	0.1109	0.0771	0.2490791	Both
DNA	nirk_Cluster22	0.1291	-0.0326	0.281	0.2432	0.0134	0.1432166	Both
DNA	nirk_Cluster23	0.0673	-0.0074	0.1175	0.071	0.1282	0.3083641	Both
DNA	nirk_Cluster24	0.0654	-0.0154	0.1796	0.1364	0.0556	0.2185169	Both
DNA	nirk_Cluster25	0.4782	-0.1489	0.4302	0.4003	0.0012	0.052418	Both
DNA	nirk_Cluster26	0.105	-0.0162	0.1828	0.1398	0.0532	0.214957	Both
DNA	nirk_Cluster27	0.1727	-0.0552	0.3365	0.3016	0.0058	0.1013565	Both
DNA	nirk_Cluster28	0.0685	-0.012	0.1031	0.0559	0.1558	0.3294091	Both
DNA	nirk_Cluster29	0.1526	-0.0018	0.0009	-0.0517	0.8979	0.9365593	Both
DNA	nirk_Cluster3	0.3023	-0.0636	0.2802	0.2423	0.0136	0.1432166	Both
DNA	nirk_Cluster30	0.0556	-0.0255	0.3461	0.3117	0.005	0.0956583	Both
DNA	nirk_Cluster31	0.1501	0.0193	0.2071	0.1654	0.0382	0.1845289	Both
DNA	nirk_Cluster32	0.1254	-0.067	0.38	0.3474	0.0029	0.0820044	Both
DNA	nirk_Cluster33	0.0638	-0.0326	0.3123	0.2761	0.0085	0.1247705	Both
DNA	nirk_Cluster34	0.0416	-0.0053	0.0366	-0.0141	0.4062	0.568969	Both
DNA	nirk_Cluster35	0.1007	-0.0478	0.2662	0.2276	0.0167	0.1615546	Both
DNA	nirk_Cluster36	0.0017	-0.001	0.1004	0.0531	0.1616	0.3357972	Both
DNA	nirk_Cluster39	0.0035	0.0002	0.0006	-0.052	0.9135	0.9472258	Both
DNA	nirk_Cluster4	0.1323	-0.0285	0.1855	0.1427	0.0512	0.2124081	Both
DNA	nirk_Cluster40	0.0455	-0.0161	0.3629	0.3293	0.0039	0.0842277	Both
DNA	nirk_Cluster41	0.1562	-0.0424	0.4134	0.3825	0.0017	0.0656043	Both
DNA	nirk_Cluster44	0.0669	-0.0279	0.2389	0.1988	0.0246	0.1739295	Both
DNA	nirk_Cluster46	0.0154	-0.0079	0.1885	0.1458	0.0492	0.2120444	Both
DNA	nirk_Cluster48	0.003	-0.0009	0.0479	-0.0022	0.3403	0.5218882	Both
DNA	nirk_Cluster49	0.0057	-0.0024	0.0711	0.0222	0.2428	0.4317664	Both
DNA	nirk_Cluster5	0.1932	-0.0331	0.2262	0.1855	0.0293	0.1739295	Both
DNA	nirk_Cluster50	0.0016	0.00E+00	0.00E+00	-0.0526	0.9865	0.9951643	Both
DNA	nirk_Cluster52	0.0452	-0.0176	0.3368	0.3019	0.0058	0.1013565	Both

DNA	nirk_Cluster53	0.0038	-0.0011	0.0284	-0.0227	0.4652	0.6164083	Both
DNA	nirk_Cluster54	0.002	-0.0005	0.0208	-0.0308	0.533	0.6676426	Both
DNA	nirk_Cluster55	0.0023	-0.0007	0.0187	-0.033	0.5549	0.6820943	Both
DNA	nirk_Cluster56	0.0009	-0.0004	0.0414	-0.009	0.3762	0.5413925	Both
DNA	nirk_Cluster57	0.0016	-0.0003	0.0051	-0.0473	0.7591	0.8466002	Both
DNA	nirk_Cluster58	0.0088	-0.001	0.0106	-0.0415	0.6572	0.7645463	Both
DNA	nirk_Cluster59	0.0058	-0.0007	0.0111	-0.041	0.65	0.7624705	Both
DNA	nirk_Cluster6	0.1558	-0.0264	0.1435	0.0984	0.0903	0.2649811	Both
DNA	nirk_Cluster60	0.0006	0.0004	0.0692	0.0203	0.2491	0.4348934	Both
DNA	nirk_Cluster62	0.1417	-0.0554	0.2144	0.173	0.0345	0.1775533	Both
DNA	nirk_Cluster63	0.1284	-0.0589	0.4552	0.4266	0.0008	0.0390074	Both
DNA	nirk_Cluster64	0.0044	0.0003	0.0032	-0.0492	0.8067	0.8732855	Both
DNA	nirk_Cluster65	0.0464	-0.0118	0.1681	0.1243	0.0649	0.2282116	Both
DNA	nirk_Cluster66	0.0068	-0.0045	0.3091	0.2728	0.0089	0.1269241	Both
DNA	nirk_Cluster67	0.0174	-0.0041	0.0409	-0.0096	0.3792	0.5430503	Both
DNA	nirk_Cluster68	0.0125	-0.0063	0.2417	0.2017	0.0236	0.1739295	Both
DNA	nirk_Cluster69	0.0305	-0.007	0.1087	0.0618	0.1444	0.3132027	Both
DNA	nirk_Cluster7	0.0804	-0.0205	0.1978	0.1556	0.0434	0.1995936	Both
DNA	nirk_Cluster70	0.0721	-0.03	0.1308	0.0851	0.1071	0.2821784	Both
DNA	nirk_Cluster71	0.0053	-0.0022	0.1152	0.0687	0.1322	0.3101587	Both
DNA	nirk_Cluster72	0.0239	-0.0074	0.1871	0.1443	0.0502	0.2124081	Both
DNA	nirk_Cluster73	0.1444	-0.0852	0.5791	0.5569	1.00E-04	0.0159293	Both
DNA	nirk_Cluster74	0.6398	-0.3134	0.3664	0.333	0.0036	0.0827207	Both
DNA	nirk_Cluster75	0.0147	-0.0097	0.2332	0.1929	0.0266	0.1739295	Both
DNA	nirk_Cluster76	0.1055	-0.0501	0.5213	0.4961	0.0002	0.0160348	Both
DNA	nirk_Cluster77	0.0081	-0.0008	0.0021	-0.0504	0.842	0.8934815	Both
DNA	nirk_Cluster78	0.2407	-0.1225	0.5078	0.4819	0.0003	0.0170439	Both
DNA	nirk_Cluster79	0.0069	-0.0014	0.0127	-0.0393	0.627	0.7428229	Both
DNA	nirk_Cluster8	0.0982	-0.0315	0.2194	0.1783	0.0322	0.1759636	Both
DNA	nirk_Cluster80	0.2665	-0.1521	0.522	0.4968	0.0002	0.0160348	Both
DNA	nirk_Cluster82	0.0015	-0.0006	0.025	-0.0263	0.4939	0.6375931	Both
DNA	nirk_Cluster83	0.004	-0.0001	0.0007	-0.0519	0.9083	0.9434788	Both
DNA	nirk_Cluster84	0.0207	-0.0033	0.0187	-0.033	0.5547	0.6820943	Both
DNA	nirk_Cluster85	0.004	-0.0015	0.0785	0.03	0.2186	0.4068991	Both
DNA	nirk_Cluster86	0.0286	-0.0136	0.2469	0.2073	0.0219	0.1739295	Both
DNA	nirk_Cluster87	0.0115	-0.0059	0.2405	0.2005	0.024	0.1739295	Both
DNA	nirk_Cluster88	0.0032	-0.0009	0.0511	0.0012	0.3243	0.5116244	Both
DNA	nirk_Cluster89	0.0065	-0.0044	0.2381	0.198	0.0248	0.1739295	Both
DNA	nirk_Cluster9	0.0517	-0.01	0.0655	0.0163	0.263	0.4471326	Both
DNA	nirk_Cluster90	0.0067	-0.0039	0.1509	0.1062	0.0819	0.2569036	Both
DNA	nirk_Cluster91	0.0374	-0.0138	0.2415	0.2016	0.0237	0.1739295	Both
DNA	nirk_Cluster92	0.0072	-0.0027	0.075	0.0263	0.2296	0.4207781	Both
DNA	nirk_Cluster93	0.0308	-0.0052	0.0505	0.0006	0.3273	0.5121901	Both
DNA	nirk_Cluster94	0.0175	-0.0056	0.112	0.0653	0.1381	0.3101587	Both
DNA	nirk_Cluster95	0.0473	-0.0172	0.173	0.1294	0.0608	0.2248984	Both
DNA	nirk_Cluster96	0.0472	-0.0201	0.2957	0.2586	0.0108	0.135128	Both
DNA	nirk_Cluster97	0.0306	-0.0094	0.1576	0.1133	0.0748	0.2475966	Both
DNA	nirk_Cluster98	0.0181	-0.0078	0.1328	0.0872	0.1043	0.2797103	Both
DNA	nirk_Cluster99	0.006	-0.0029	0.1291	0.0833	0.1097	0.2861779	Both
DNA	nirs_Cluster0	0.1409	-0.0266	0.1523	0.1077	0.0803	0.2566347	Both
DNA	nirs_Cluster1	0.004	-0.0015	0.0421	-0.0083	0.3721	0.5393578	Both
DNA	nirs_Cluster14	0.0107	-0.0062	0.0735	0.0248	0.2344	0.422995	Both
DNA	nirs_Cluster2	0.0139	-0.0024	0.011	-0.0411	0.6517	0.7628939	Both
DNA	nirs_Cluster26	0.0005	-0.0003	0.0456	-0.0047	0.3529	0.5299245	Both
DNA	nirs_Cluster28	0.0071	-0.0057	0.135	0.0895	0.1013	0.2793382	Both

DNA	nirs_Cluster3	0.1715	-0.0299	0.2649	0.2262	0.017	0.1615546	Both
DNA	nirs_Cluster36	0.0013	-0.0001	0.0005	-0.0521	0.9234	0.95414	Both
DNA	nirs_Cluster37	0.0066	-0.0049	0.0634	0.0141	0.2708	0.4539028	Both
DNA	nirs_Cluster4	0.0002	-0.0002	0.027	-0.0243	0.477	0.6253643	Both
DNA	nirs_Cluster50	0.002	-1.00E-04	0.0005	-0.0521	0.9209	0.953246	Both
DNA	nirs_Cluster9	0.0269	-0.0249	0.1768	0.1334	0.0577	0.224078	Both
DNA	norb_Cluster0	0.0775	-0.0279	0.2117	0.1703	0.0358	0.1811591	Both
DNA	norb_Cluster1	1.1042	-0.4564	0.2047	0.1629	0.0395	0.1874873	Both
DNA	norb_Cluster101	0.0026	-0.0021	0.1582	0.1139	0.0742	0.2475966	Both
DNA	norb_Cluster102	0.0038	-0.0026	0.2163	0.175	0.0336	0.1772493	Both
DNA	norb_Cluster103	0.0018	-0.0013	0.1143	0.0677	0.1339	0.3101587	Both
DNA	norb_Cluster104	0.0023	-0.0005	0.0092	-0.043	0.6798	0.7788454	Both
DNA	norb_Cluster106	0.125	-0.0328	0.1179	0.0715	0.1275	0.3083641	Both
DNA	norb_Cluster107	0.0133	-0.0058	0.151	0.1063	0.0817	0.2569036	Both
DNA	norb_Cluster108	0.0052	-0.0027	0.1955	0.1532	0.0448	0.1995936	Both
DNA	norb_Cluster109	0.0227	-0.0089	0.1687	0.1249	0.0644	0.2282116	Both
DNA	norb_Cluster11	0.0126	-0.0072	0.2415	0.2016	0.0237	0.1739295	Both
DNA	norb_Cluster110	0.0099	-0.0053	0.194	0.1516	0.0457	0.1995936	Both
DNA	norb_Cluster112	0.0171	-0.0051	0.109	0.0621	0.1439	0.3132027	Both
DNA	norb_Cluster113	0.0134	-0.0021	0.0176	-0.0341	0.5668	0.6923643	Both
DNA	norb_Cluster114	0.0025	-0.0018	0.0813	0.033	0.2101	0.3960744	Both
DNA	norb_Cluster115	0.0137	0.0266	0.2931	0.2559	0.0113	0.135128	Both
DNA	norb_Cluster116	0.0072	-0.0002	0.0003	-0.0523	0.9401	0.9646674	Both
DNA	norb_Cluster117	0.0027	-0.0009	0.0358	-0.0149	0.4113	0.5696228	Both
DNA	norb_Cluster118	0.0037	-0.0022	0.1813	0.1383	0.0543	0.2163135	Both
DNA	norb_Cluster119	0.019	-0.0118	0.2307	0.1902	0.0275	0.1739295	Both
DNA	norb_Cluster12	0.0047	-0.0027	0.1344	0.0888	0.1022	0.279396	Both
DNA	norb_Cluster121	0.0017	-0.0013	0.0903	0.0424	0.1856	0.3712693	Both
DNA	norb_Cluster122	0.0144	-0.0061	0.166	0.1221	0.0668	0.2307465	Both
DNA	norb_Cluster123	0.0053	-0.0026	0.1138	0.0672	0.1348	0.3101587	Both
DNA	norb_Cluster124	0.682	-0.1468	0.0704	0.0215	0.2451	0.4329795	Both
DNA	norb_Cluster125	0.004	-0.0021	0.1486	0.1038	0.0844	0.2593111	Both
DNA	norb_Cluster126	0.0082	-0.0048	0.2322	0.1918	0.027	0.1739295	Both
DNA	norb_Cluster127	0.1735	-0.0671	0.1945	0.1521	0.0454	0.1995936	Both
DNA	norb_Cluster128	0.0115	-0.004	0.1342	0.0887	0.1023	0.279396	Both
DNA	norb_Cluster13	0.0045	-0.003	0.1433	0.0982	0.0906	0.2649811	Both
DNA	norb_Cluster131	0.0184	-0.0029	0.026	-0.0253	0.4854	0.6293798	Both
DNA	norb_Cluster132	1.0357	-0.3913	0.3009	0.2641	0.01	0.1315262	Both
DNA	norb_Cluster133	0.0018	-0.0007	0.0432	-0.0071	0.3659	0.5363013	Both
DNA	norb_Cluster135	0.0567	-0.0204	0.1709	0.1272	0.0625	0.2248984	Both
DNA	norb_Cluster136	0.0213	-0.0065	0.1098	0.063	0.1423	0.313169	Both
DNA	norb_Cluster139	0.0328	-0.0115	0.1704	0.1268	0.0629	0.2249345	Both
DNA	norb_Cluster14	0.0026	-0.0021	0.2327	0.1923	0.0268	0.1739295	Both
DNA	norb_Cluster140	0.0437	-0.0231	0.3671	0.3338	0.0036	0.0827207	Both
DNA	norb_Cluster142	0.0015	-0.0014	0.1521	0.1075	0.0805	0.2566347	Both
DNA	norb_Cluster144	0.0098	-0.0012	0.0119	-0.0401	0.638	0.7523539	Both
DNA	norb_Cluster146	0.0348	-0.0096	0.1164	0.0699	0.1302	0.3084324	Both
DNA	norb_Cluster15	0.0469	-0.016	0.1358	0.0903	0.1002	0.2776796	Both
DNA	norb_Cluster150	0.0157	-0.0071	0.2803	0.2424	0.0136	0.1432166	Both
DNA	norb_Cluster155	0.1631	-0.0294	0.0543	0.0046	0.3092	0.4957794	Both
DNA	norb_Cluster16	0.0131	-0.0082	0.2354	0.1952	0.0258	0.1739295	Both
DNA	norb_Cluster164	0.0199	-0.0136	0.3536	0.3196	0.0045	0.0941457	Both
DNA	norb_Cluster165	0.0076	-0.0031	0.0756	0.027	0.2277	0.4185175	Both
DNA	norb_Cluster166	0.0039	-0.0012	0.0389	-0.0117	0.3915	0.5552675	Both
DNA	norb_Cluster169	0.0238	-0.0154	0.2945	0.2574	0.011	0.135128	Both

DNA	norb_Cluster17	0.0028	-0.002	0.2142	0.1729	0.0346	0.1775533	Both
DNA	norb_Cluster170	0.0243	-0.0088	0.0808	0.0324	0.2119	0.3968292	Both
DNA	norb_Cluster171	0.0069	-0.004	0.1179	0.0714	0.1276	0.3083641	Both
DNA	norb_Cluster172	0.014	-0.009	0.2235	0.1826	0.0304	0.1739295	Both
DNA	norb_Cluster175	0.0458	-0.0282	0.232	0.1916	0.027	0.1739295	Both
DNA	norb_Cluster176	0.0298	-0.0201	0.279	0.2411	0.0138	0.1432166	Both
DNA	norb_Cluster177	0.0282	-0.0133	0.1374	0.092	0.0981	0.2751719	Both
DNA	norb_Cluster179	0.0022	-0.0006	0.0188	-0.0328	0.5531	0.6820943	Both
DNA	norb_Cluster18	0.0037	-0.0024	0.2483	0.2087	0.0215	0.1739295	Both
DNA	norb_Cluster180	0.0109	-0.0036	0.1266	0.0806	0.1134	0.288473	Both
DNA	norb_Cluster181	0.0008	0.0013	0.221	0.18	0.0315	0.1739295	Both
DNA	norb_Cluster182	0.0013	-0.0009	0.1051	0.058	0.1516	0.3219818	Both
DNA	norb_Cluster184	0.0146	-0.0057	0.1232	0.0771	0.1187	0.2980421	Both
DNA	norb_Cluster186	0.0011	0.0002	0.0042	-0.0482	0.7791	0.8592002	Both
DNA	norb_Cluster19	0.0049	-0.0013	0.054	0.0042	0.3106	0.4966671	Both
DNA	norb_Cluster192	0.0173	-0.0109	0.2651	0.2264	0.0169	0.1615546	Both
DNA	norb_Cluster193	0.0021	0.0002	0.0018	-0.0507	0.8546	0.9019672	Both
DNA	norb_Cluster197	0.0303	-0.016	0.0419	-0.0086	0.3737	0.5401259	Both
DNA	norb_Cluster2	0.0031	0.0004	0.0047	-0.0477	0.7682	0.8518979	Both
DNA	norb_Cluster20	0.0587	-0.0201	0.1777	0.1344	0.057	0.2227531	Both
DNA	norb_Cluster208	0.0011	-0.0009	0.1148	0.0682	0.1331	0.3101587	Both
DNA	norb_Cluster21	0.0481	-0.0221	0.238	0.1979	0.0249	0.1739295	Both
DNA	norb_Cluster211	0.0104	-0.0056	0.055	0.0053	0.306	0.4919831	Both
DNA	norb_Cluster212	0.0264	0.0072	0.0228	-0.0287	0.5138	0.6547337	Both
DNA	norb_Cluster214	0.0119	-0.0066	0.0956	0.048	0.1725	0.3515669	Both
DNA	norb_Cluster217	0.0149	-0.0056	0.0485	-0.0016	0.3376	0.5213532	Both
DNA	norb_Cluster219	0.0083	-0.0047	0.0774	0.0289	0.2219	0.4116946	Both
DNA	norb_Cluster22	0.0025	-0.001	0.0398	-0.0108	0.3862	0.5503198	Both
DNA	norb_Cluster220	0.0177	-0.0129	0.1119	0.0652	0.1383	0.3101587	Both
DNA	norb_Cluster221	0.0177	-0.0124	0.0898	0.0419	0.1868	0.3723775	Both
DNA	norb_Cluster222	0.0074	-0.0053	0.0699	0.021	0.2467	0.4332254	Both
DNA	norb_Cluster224	0.0194	-0.0075	0.0476	-0.0026	0.3422	0.5218882	Both
DNA	norb_Cluster225	0.0097	0.0034	0.0431	-0.0072	0.3664	0.5363013	Both
DNA	norb_Cluster226	0.0043	-0.0004	0.0037	-0.0487	0.7922	0.8671279	Both
DNA	norb_Cluster228	0.0031	0.0057	0.2443	0.2045	0.0228	0.1739295	Both
DNA	norb_Cluster229	0.0034	-0.0004	0.0045	-0.0479	0.7731	0.8557749	Both
DNA	norb_Cluster230	0.0014	-0.0009	0.0879	0.0399	0.1918	0.3748879	Both
DNA	norb_Cluster231	0.0044	-0.0019	0.0452	-0.005	0.3548	0.5299245	Both
DNA	norb_Cluster232	0.0031	-0.0012	0.0438	-0.0065	0.3623	0.5345994	Both
DNA	norb_Cluster234	0.0121	-0.0094	0.2437	0.2039	0.023	0.1739295	Both
DNA	norb_Cluster235	0.0009	-0.0005	0.0854	0.0373	0.1986	0.3840957	Both
DNA	norb_Cluster238	0.0099	-0.0063	0.1267	0.0807	0.1133	0.288473	Both
DNA	norb_Cluster239	0.0081	-0.006	0.1415	0.0963	0.0928	0.2658115	Both
DNA	norb_Cluster24	0.2637	-0.1056	0.2425	0.2026	0.0234	0.1739295	Both
DNA	norb_Cluster244	0.0011	-0.0005	0.0425	-0.0078	0.3697	0.5382162	Both
DNA	norb_Cluster247	0.0048	-0.001	0.0365	-0.0142	0.407	0.568969	Both
DNA	norb_Cluster25	0.1114	-0.0433	0.1866	0.1438	0.0505	0.2124081	Both
DNA	norb_Cluster250	0.0115	-0.0036	0.0699	0.0209	0.2468	0.4332254	Both
DNA	norb_Cluster251	0.0052	-0.0019	0.0665	0.0173	0.2592	0.4446188	Both
DNA	norb_Cluster26	0.0931	-0.0503	0.3015	0.2648	0.0099	0.1315262	Both
DNA	norb_Cluster28	0.0302	-0.005	0.0286	-0.0225	0.4636	0.6160992	Both
DNA	norb_Cluster29	0.222	-0.0517	0.2066	0.1648	0.0385	0.1845289	Both
DNA	norb_Cluster31	0.0531	-0.0234	0.2293	0.1888	0.0281	0.1739295	Both
DNA	norb_Cluster32	0.1193	-0.059	0.222	0.181	0.0311	0.1739295	Both
DNA	norb_Cluster33	0.4946	0.0951	0.1385	0.0932	0.0966	0.2728327	Both

DNA	norb_Cluster35	0.0628	-0.0302	0.285	0.2473	0.0127	0.1432166	Both
DNA	norb_Cluster36	1.1345	-0.1194	0.0214	-0.0301	0.5265	0.6609657	Both
DNA	norb_Cluster37	0.7473	-0.0548	0.0162	-0.0356	0.5827	0.7088606	Both
DNA	norb_Cluster38	0.0116	-0.0035	0.119	0.0727	0.1256	0.307471	Both
DNA	norb_Cluster39	0.0426	-0.018	0.2097	0.1681	0.0368	0.1811591	Both
DNA	norb_Cluster40	0.186	-0.071	0.1965	0.1542	0.0441	0.1995936	Both
DNA	norb_Cluster42	0.2864	-0.0303	0.0236	-0.0278	0.5064	0.6495414	Both
DNA	norb_Cluster44	0.0182	-0.0109	0.2255	0.1847	0.0296	0.1739295	Both
DNA	norb_Cluster46	0.2556	0.0215	0.0102	-0.0419	0.6634	0.76649	Both
DNA	norb_Cluster47	0.555	-0.2049	0.2209	0.1799	0.0315	0.1739295	Both
DNA	norb_Cluster5	0.01	-0.0029	0.1132	0.0665	0.1359	0.3101587	Both
DNA	norb_Cluster51	0.0092	-0.0069	0.2114	0.1699	0.036	0.1811591	Both
DNA	norb_Cluster52	0.2391	-0.0981	0.2167	0.1754	0.0335	0.1772493	Both
DNA	norb_Cluster56	0.014	-0.0064	0.209	0.1674	0.0372	0.1813072	Both
DNA	norb_Cluster57	0.0034	-0.0002	0.0034	-0.049	0.801	0.8726701	Both
DNA	norb_Cluster59	0.0236	-0.01	0.0895	0.0416	0.1877	0.3728404	Both
DNA	norb_Cluster6	0.6862	-0.1064	0.0494	-0.0007	0.3331	0.5171724	Both
DNA	norb_Cluster60	0.3199	-0.045	0.0157	-0.0361	0.5883	0.7112892	Both
DNA	norb_Cluster61	0.482	-0.1148	0.076	0.0274	0.2264	0.4174084	Both
DNA	norb_Cluster62	0.0024	0.0001	0.0015	-0.051	0.867	0.910164	Both
DNA	norb_Cluster64	0.0326	-0.0106	0.1612	0.1171	0.0712	0.2414155	Both
DNA	norb_Cluster66	0.0012	-0.0007	0.0678	0.0188	0.2541	0.4413322	Both
DNA	norb_Cluster67	0.0218	-0.0091	0.1717	0.1281	0.0618	0.2248984	Both
DNA	norb_Cluster68	0.0034	-1.00E-04	0.0004	-0.0522	0.9341	0.9601089	Both
DNA	norb_Cluster69	0.0028	-0.0015	0.1103	0.0635	0.1414	0.3123632	Both
DNA	norb_Cluster7	0.0709	-0.0205	0.1192	0.0728	0.1254	0.307471	Both
DNA	norb_Cluster70	0.1051	-0.0302	0.1289	0.0831	0.1099	0.2861779	Both
DNA	norb_Cluster71	0.1295	-0.0306	0.1055	0.0585	0.1508	0.3219818	Both
DNA	norb_Cluster74	0.0327	-0.0095	0.132	0.0863	0.1055	0.2807408	Both
DNA	norb_Cluster75	0.1291	-0.0412	0.2415	0.2016	0.0237	0.1739295	Both
DNA	norb_Cluster76	0.1105	-0.0196	0.0187	-0.033	0.5548	0.6820943	Both
DNA	norb_Cluster77	0.0475	-0.0032	0.0118	-0.0402	0.6389	0.7523539	Both
DNA	norb_Cluster8	0.018	-0.0042	0.052	0.0021	0.3201	0.5076229	Both
DNA	norb_Cluster80	0.0015	-0.0013	0.1565	0.1122	0.0758	0.2475966	Both
DNA	norb_Cluster81	0.0012	0.00E+00	0.0002	-0.0525	0.9565	0.9762253	Both
DNA	norb_Cluster83	0.0078	-0.0068	0.1739	0.1305	0.06	0.2248984	Both
DNA	norb_Cluster84	0.0082	-0.0023	0.0613	0.0119	0.2793	0.4638595	Both
DNA	norb_Cluster85	0.0023	-0.0007	0.037	-0.0137	0.4038	0.5686299	Both
DNA	norb_Cluster86	0.031	-0.0189	0.1055	0.0585	0.1508	0.3219818	Both
DNA	norb_Cluster88	0.0016	-0.0003	0.0105	-0.0415	0.658	0.7645463	Both
DNA	norb_Cluster90	0.0009	-0.0007	0.0486	-0.0015	0.3368	0.5213532	Both
DNA	norb_Cluster91	0.6481	-0.2492	0.1761	0.1327	0.0583	0.2246536	Both
DNA	norb_Cluster92	0.0015	-0.0007	0.0299	-0.0212	0.4535	0.6067559	Both
DNA	norb_Cluster93	0.0022	-0.0006	0.0156	-0.0362	0.5898	0.7116369	Both
DNA	norb_Cluster94	0.0017	0.0001	0.0014	-0.0512	0.8733	0.9151341	Both
DNA	norb_Cluster96	0.0007	0.0005	0.0706	0.0217	0.2442	0.4327324	Both
DNA	norb_Cluster97	0.0119	-0.0041	0.0662	0.0171	0.2601	0.4448735	Both
DNA	norb_Cluster98	0.0132	-0.0077	0.1576	0.1133	0.0748	0.2475966	Both
DNA	nosz_Cluster0	0.0045	-0.0015	0.0697	0.0208	0.2475	0.4332254	Both
DNA	nosz_Cluster10	0.0136	0.0051	0.0866	0.0386	0.1953	0.3789959	Both
DNA	nosz_Cluster102	0.0044	-0.0012	0.066	0.0169	0.2609	0.4448735	Both
DNA	nosz_Cluster103	0.0455	-0.0108	0.1019	0.0546	0.1584	0.3314155	Both
DNA	nosz_Cluster105	0.0016	-0.0002	0.0031	-0.0493	0.8094	0.8746741	Both
DNA	nosz_Cluster106	0.0073	-0.0041	0.2107	0.1691	0.0364	0.1811591	Both
DNA	nosz_Cluster107	0.0002	-0.0002	0.027	-0.0243	0.477	0.6253643	Both

DNA	nosz_Cluster108	0.004	-0.0002	0.0015	-0.051	0.8663	0.910164	Both
DNA	nosz_Cluster109	0.0375	-0.0071	0.0967	0.0492	0.17	0.348299	Both
DNA	nosz_Cluster11	0.0129	0.0108	0.1858	0.1429	0.0511	0.2124081	Both
DNA	nosz_Cluster110	0.0003	0.0001	0.0109	-0.0412	0.653	0.7629601	Both
DNA	nosz_Cluster111	0.0061	-0.0028	0.1418	0.0966	0.0925	0.2658115	Both
DNA	nosz_Cluster116	0.0067	0.0007	0.0072	-0.045	0.7143	0.8104066	Both
DNA	nosz_Cluster118	0.0045	-0.0031	0.1517	0.1071	0.0809	0.2566422	Both
DNA	nosz_Cluster119	0.0013	-0.0013	0.1879	0.1451	0.0496	0.2122685	Both
DNA	nosz_Cluster120	0.0071	-0.0029	0.133	0.0873	0.1041	0.2797103	Both
DNA	nosz_Cluster123	0.0097	-0.0072	0.2919	0.2547	0.0115	0.135128	Both
DNA	nosz_Cluster128	0.0047	0.0002	0.0008	-0.0518	0.9023	0.9389117	Both
DNA	nosz_Cluster129	0.0494	-0.0029	0.0153	-0.0365	0.5927	0.7136067	Both
DNA	nosz_Cluster13	0.0115	0.0009	0.0042	-0.0482	0.7808	0.8594445	Both
DNA	nosz_Cluster130	0.089	-0.0488	0.0636	0.0144	0.2699	0.4537459	Both
DNA	nosz_Cluster132	0.0037	-0.0008	0.0404	-0.0101	0.3822	0.5459362	Both
DNA	nosz_Cluster14	0.024	0.0282	0.223	0.1821	0.0307	0.1739295	Both
DNA	nosz_Cluster16	0.0076	0.0018	0.0205	-0.0311	0.5359	0.6699347	Both
DNA	nosz_Cluster17	0.0364	-0.0106	0.1173	0.0708	0.1286	0.3083641	Both
DNA	nosz_Cluster18	0.0161	0.00E+00	0.00E+00	-0.0526	0.9984	0.9984133	Both
DNA	nosz_Cluster20	0.0091	-0.0053	0.1456	0.1006	0.0879	0.2649811	Both
DNA	nosz_Cluster21	0.0083	-0.0066	0.2452	0.2054	0.0225	0.1739295	Both
DNA	nosz_Cluster22	0.0267	-0.0078	0.0356	-0.0152	0.4129	0.5705819	Both
DNA	nosz_Cluster23	0.0141	-0.0097	0.2488	0.2092	0.0214	0.1739295	Both
DNA	nosz_Cluster24	0.0013	-0.0005	0.0261	-0.0251	0.4839	0.6288631	Both
DNA	nosz_Cluster25	0.0029	0.0019	0.1057	0.0586	0.1505	0.3219818	Both
DNA	nosz_Cluster26	0.0054	-0.0023	0.0743	0.0256	0.232	0.4212652	Both
DNA	nosz_Cluster27	0.0053	-0.0023	0.109	0.0621	0.1439	0.3132027	Both
DNA	nosz_Cluster28	0.0303	-0.0116	0.1432	0.0981	0.0907	0.2649811	Both
DNA	nosz_Cluster30	0.0276	-0.0058	0.0417	-0.0087	0.3744	0.5401259	Both
DNA	nosz_Cluster32	0.0137	-0.0003	0.0004	-0.0522	0.9299	0.9591116	Both
DNA	nosz_Cluster33	0.018	0.0031	0.0138	-0.0381	0.6124	0.7314495	Both
DNA	nosz_Cluster34	0.0377	-0.0005	1.00E-04	-0.0525	0.9661	0.9827661	Both
DNA	nosz_Cluster35	0.0075	-0.0027	0.0825	0.0342	0.2068	0.392904	Both
DNA	nosz_Cluster36	0.0179	-0.0031	0.0228	-0.0286	0.5133	0.6547337	Both
DNA	nosz_Cluster37	0.006	-0.0017	0.0442	-0.0061	0.3603	0.5345994	Both
DNA	nosz_Cluster38	0.0128	-0.0017	0.009	-0.0431	0.6823	0.7801437	Both
DNA	nosz_Cluster39	0.0537	-0.0141	0.071	0.0221	0.243	0.4317664	Both
DNA	nosz_Cluster40	0.0218	-0.0018	0.0051	-0.0473	0.759	0.8466002	Both
DNA	nosz_Cluster41	0.0203	-0.0072	0.0846	0.0364	0.2008	0.3872513	Both
DNA	nosz_Cluster42	0.0279	-0.0006	0.0003	-0.0523	0.9426	0.965487	Both
DNA	nosz_Cluster43	0.0007	-0.0006	0.0746	0.0258	0.2311	0.4212652	Both
DNA	nosz_Cluster44	0.0057	-0.0031	0.1709	0.1273	0.0625	0.2248984	Both
DNA	nosz_Cluster45	0.0045	-0.0019	0.0886	0.0406	0.1901	0.3737855	Both
DNA	nosz_Cluster46	0.007	-0.0011	0.0221	-0.0293	0.5199	0.656779	Both
DNA	nosz_Cluster47	0.0008	-0.0006	0.0995	0.0521	0.1637	0.3387999	Both
DNA	nosz_Cluster48	0.0023	-0.001	0.0475	-0.0026	0.3426	0.5218882	Both
DNA	nosz_Cluster49	0.0014	0.001	0.1423	0.0972	0.0918	0.2658115	Both
DNA	nosz_Cluster5	0.0045	0.0023	0.0665	0.0174	0.2591	0.4446188	Both
DNA	nosz_Cluster51	0.0021	-0.0013	0.1122	0.0655	0.1377	0.3101587	Both
DNA	nosz_Cluster53	0.0009	-0.0004	0.0216	-0.0299	0.5247	0.6609657	Both
DNA	nosz_Cluster54	0.0026	-0.0019	0.1372	0.0917	0.0984	0.2751719	Both
DNA	nosz_Cluster57	0.1757	-0.0674	0.1408	0.0956	0.0937	0.2669733	Both
DNA	nosz_Cluster58	0.0043	-0.0027	0.1494	0.1047	0.0834	0.2577293	Both
DNA	nosz_Cluster59	0.0009	0.0002	0.0049	-0.0475	0.7629	0.8478727	Both
DNA	nosz_Cluster6	0.0032	0.0003	0.0035	-0.049	0.8003	0.8726701	Both

DNA	nosz_Cluster60	0.0079	-0.0023	0.0473	-0.0029	0.3437	0.5218882	Both
DNA	nosz_Cluster61	0.0178	-0.0042	0.1199	0.0736	0.1242	0.3065259	Both
DNA	nosz_Cluster62	0.0413	-0.0128	0.092	0.0442	0.1814	0.3656187	Both
DNA	nosz_Cluster64	0.0016	-0.0002	0.0016	-0.0509	0.8621	0.908249	Both
DNA	nosz_Cluster65	0.0065	-0.002	0.0474	-0.0027	0.3429	0.5218882	Both
DNA	nosz_Cluster66	0.0324	-0.0168	0.168	0.1242	0.065	0.2282116	Both
DNA	nosz_Cluster67	0.0521	-0.0209	0.1116	0.0648	0.1389	0.3103363	Both
DNA	nosz_Cluster69	0.0061	-0.0037	0.2249	0.1841	0.0299	0.1739295	Both
DNA	nosz_Cluster70	0.0479	-0.0312	0.1121	0.0654	0.138	0.3101587	Both
DNA	nosz_Cluster71	0.0083	-0.0022	0.0291	-0.022	0.4601	0.6127187	Both
DNA	nosz_Cluster72	0.0506	-0.0215	0.1438	0.0987	0.09	0.2649811	Both
DNA	nosz_Cluster73	0.003	-0.0002	0.0026	-0.0499	0.827	0.8857525	Both
DNA	nosz_Cluster74	0.0017	-0.0006	0.0373	-0.0133	0.4013	0.5664516	Both
DNA	nosz_Cluster75	0.0967	-0.0679	0.0743	0.0255	0.2321	0.4212652	Both
DNA	nosz_Cluster77	0.0002	-1.00E-04	0.0052	-0.0471	0.7557	0.8460278	Both
DNA	nosz_Cluster78	0.0044	-0.0026	0.2279	0.1873	0.0286	0.1739295	Both
DNA	nosz_Cluster79	0.0333	-0.0073	0.0599	0.0104	0.285	0.4696148	Both
DNA	nosz_Cluster8	0.0099	0.00E+00	0.00E+00	-0.0526	0.9983	0.9984133	Both
DNA	nosz_Cluster80	0.0045	0.0024	0.0869	0.0388	0.1946	0.3789743	Both
DNA	nosz_Cluster81	0.0079	-0.0031	0.0466	-0.0036	0.3474	0.5254938	Both
DNA	nosz_Cluster82	0.0016	0.00E+00	0.00E+00	-0.0526	0.989	0.9957481	Both
DNA	nosz_Cluster83	0.0015	-0.0009	0.0591	0.0096	0.2882	0.4716106	Both
DNA	nosz_Cluster85	0.0022	0.0001	0.0009	-0.0517	0.8985	0.9365593	Both
DNA	nosz_Cluster86	0.0027	-0.0011	0.0525	0.0026	0.3178	0.505428	Both
DNA	nosz_Cluster87	0.003	0.00E+00	0.00E+00	-0.0526	0.9867	0.9951643	Both
DNA	nosz_Cluster88	0.0218	-0.007	0.224	0.1832	0.0302	0.1739295	Both
DNA	nosz_Cluster89	0.0466	-0.0228	0.1852	0.1423	0.0515	0.2124081	Both
DNA	nosz_Cluster9	0.003	-0.0006	0.0124	-0.0395	0.6302	0.7451725	Both
DNA	nosz_Cluster90	0.0116	-0.0029	0.089	0.041	0.189	0.3737855	Both
DNA	nosz_Cluster91	0.0189	-0.0046	0.0359	-0.0148	0.4107	0.5696228	Both
DNA	nosz_Cluster93	0.0393	-0.018	0.2164	0.1751	0.0336	0.1772493	Both
DNA	nosz_Cluster95	0.007	-0.0033	0.1552	0.1107	0.0773	0.2490791	Both
DNA	nosz_Cluster96	0.0066	0.0007	0.0115	-0.0405	0.6436	0.7564311	Both
DNA	nosz_Cluster97	0.0019	-0.0011	0.0904	0.0425	0.1854	0.3712693	Both
DNA	nosz_Cluster98	0.0025	-0.0001	0.0002	-0.0524	0.9534	0.9748473	Both
DNA	nosz_Cluster99	0.0055	0.0003	0.0025	-0.05	0.8303	0.8857525	Both
DNA	nrfa_Cluster10	0.0804	-0.0223	0.0495	-0.0005	0.3322	0.5171724	Both
DNA	nrfa_Cluster100	0.0963	-0.0601	0.1803	0.1371	0.0551	0.2180404	Both
DNA	nrfa_Cluster104	0.1143	-0.0556	0.2466	0.207	0.022	0.1739295	Both
DNA	nrfa_Cluster105	0.0062	0.002	0.0346	-0.0162	0.4192	0.575754	Both
DNA	nrfa_Cluster109	0.2294	-0.0577	0.0794	0.0309	0.216	0.4032818	Both
DNA	nrfa_Cluster116	0.2592	-0.114	0.3712	0.3381	0.0034	0.0827207	Both
DNA	nrfa_Cluster118	0.173	-0.1199	0.1816	0.1386	0.054	0.2163135	Both
DNA	nrfa_Cluster125	0.0809	-0.0571	0.1671	0.1233	0.0658	0.2295719	Both
DNA	nrfa_Cluster154	0.0658	-0.0332	0.1287	0.0829	0.1102	0.2861779	Both
DNA	nrfa_Cluster164	0.0045	0.0013	0.0291	-0.022	0.46	0.6127187	Both
DNA	nrfa_Cluster172	0.0026	-0.0021	0.1444	0.0994	0.0892	0.2649811	Both
DNA	nrfa_Cluster175	0.0044	-0.0023	0.0678	0.0187	0.2543	0.4413322	Both
DNA	nrfa_Cluster20	0.035	-0.0156	0.1227	0.0765	0.1195	0.2987902	Both
DNA	nrfa_Cluster26	0.1078	-0.0754	0.2042	0.1623	0.0397	0.1874873	Both
DNA	nrfa_Cluster29	0.0048	-0.0012	0.0224	-0.029	0.5172	0.6558655	Both
DNA	nrfa_Cluster34	0.0487	-0.0269	0.1834	0.1404	0.0528	0.214724	Both
DNA	nrfa_Cluster38	0.0031	-0.0009	0.0248	-0.0265	0.4956	0.638428	Both
DNA	nrfa_Cluster61	0.0036	-0.0023	0.1361	0.0906	0.0998	0.2776796	Both
DNA	nrfa_Cluster62	0.0452	-0.0303	0.1564	0.112	0.076	0.2475966	Both

DNA	nrfa_Cluster66	0.1082	-0.0337	0.1441	0.099	0.0897	0.2649811	Both
DNA	nrfa_Cluster69	0.003	-0.001	0.0455	-0.0048	0.3534	0.5299245	Both
DNA	nrfa_Cluster71	0.027	-0.0084	0.0438	-0.0065	0.3624	0.5345994	Both
DNA	nrfa_Cluster72	0.0732	-0.0446	0.2031	0.1611	0.0403	0.1889259	Both
DNA	nrfa_Cluster73	0.0038	-0.0004	0.0026	-0.0499	0.8272	0.8857525	Both
DNA	nrfa_Cluster74	0.1331	-0.0712	0.1655	0.1216	0.0672	0.2307465	Both
DNA	nrfa_Cluster76	0.0755	-0.0311	0.0839	0.0356	0.2029	0.3898921	Both
DNA	nrfa_Cluster79	0.0229	0.0261	0.1715	0.1279	0.062	0.2248984	Both
DNA	nrfa_Cluster87	0.017	-0.0055	0.0304	-0.0207	0.4499	0.6046331	Both
DNA	nrfa_Cluster89	0.0018	-0.0015	0.1391	0.0938	0.0959	0.2720437	Both
DNA	nrfa_Cluster92	0.0043	0.0013	0.0338	-0.017	0.4247	0.5797596	Both
DNA	nrfa_Cluster93	0.0428	-0.0252	0.1007	0.0533	0.1611	0.3357972	Both
DNA	nrfa_Cluster94	0.0002	-0.0002	0.0508	0.0008	0.3261	0.5116244	Both
DNA	nrfa_Cluster97	0.0168	-0.0099	0.2473	0.2077	0.0218	0.1739295	Both
DNA	nrfa_Cluster99	0.014	-0.0074	0.1642	0.1202	0.0684	0.2333298	Both
Water_content	amoA_A_Cluster26	0.004	-0.0034	0.2058	0.164	0.0389	0.1927317	Both
Water_content	amoA_A_Cluster45	0.0051	-0.0034	0.158	0.1137	0.0744	0.2566347	Both
Water_content	amoA_B_Cluster0	0.1099	-0.0326	0.4139	0.3831	0.0017	0.030356	Both
Water_content	amoA_B_Cluster1	0.0172	-0.0082	0.2307	0.1902	0.0276	0.1625826	Both
Water_content	amoA_B_Cluster10	0.0422	-0.0241	0.6149	0.5947	0.00E+00	0.0016958	Both
Water_content	amoA_B_Cluster11	0.0377	-0.0177	0.3023	0.2656	0.0098	0.0981828	Both
Water_content	amoA_B_Cluster12	0.0807	-0.0302	0.5057	0.4797	0.0003	0.0100103	Both
Water_content	amoA_B_Cluster13	0.5271	-0.2799	0.4774	0.4499	0.0005	0.0121511	Both
Water_content	amoA_B_Cluster14	0.0275	-0.0172	0.3544	0.3204	0.0044	0.0642982	Both
Water_content	amoA_B_Cluster15	0.0318	-0.017	0.372	0.339	0.0033	0.0503144	Both
Water_content	amoA_B_Cluster2	0.1721	-0.0983	0.5311	0.5064	0.0002	0.0070392	Both
Water_content	amoA_B_Cluster20	0.0058	-0.0023	0.0612	0.0118	0.2796	0.4972929	Both
Water_content	amoA_B_Cluster23	0.0507	-0.0205	0.3407	0.306	0.0055	0.0686845	Both
Water_content	amoA_B_Cluster3	0.0182	-0.0095	0.2321	0.1917	0.027	0.1609128	Both
Water_content	amoA_B_Cluster4	0.0125	-0.0082	0.3058	0.2693	0.0093	0.0964757	Both
Water_content	amoA_B_Cluster5	0.0928	-0.0322	0.4498	0.4208	0.0009	0.0178394	Both
Water_content	amoA_B_Cluster6	0.0233	-0.0081	0.2283	0.1877	0.0285	0.1637915	Both
Water_content	amoA_B_Cluster7	0.0179	-0.0047	0.0582	0.0086	0.2923	0.5027201	Both
Water_content	amoA_B_Cluster8	0.0207	-0.0092	0.159	0.1147	0.0734	0.2546797	Both
Water_content	amoA_B_Cluster9	0.0549	-0.0255	0.2442	0.2044	0.0228	0.1510123	Both
Water_content	nifh_Cluster0	0.0332	0.0071	0.1997	0.1576	0.0423	0.2043556	Both
Water_content	nifh_Cluster10	0.005	0.0026	0.0631	0.0138	0.272	0.4952758	Both
Water_content	nifh_Cluster100	0.0019	0.0003	0.0064	-0.0459	0.7307	0.8306238	Both
Water_content	nifh_Cluster103	0.0383	-0.0154	0.3517	0.3176	0.0046	0.0642982	Both
Water_content	nifh_Cluster1034	0.0262	-0.0106	0.1206	0.0743	0.123	0.3298473	Both
Water_content	nifh_Cluster105	0.013	-0.0047	0.0903	0.0424	0.1857	0.4049055	Both
Water_content	nifh_Cluster108	0.0083	-0.0024	0.0763	0.0277	0.2254	0.449237	Both
Water_content	nifh_Cluster1099	0.066	0.0427	0.4235	0.3932	0.0014	0.0275134	Both
Water_content	nifh_Cluster11	0.0021	-0.0004	0.0165	-0.0352	0.5787	0.7203726	Both
Water_content	nifh_Cluster1112	0.0029	-0.0013	0.0767	0.0281	0.2243	0.44863	Both
Water_content	nifh_Cluster112	0.0112	0.0058	0.2423	0.2024	0.0234	0.1525671	Both
Water_content	nifh_Cluster113	0.0327	-0.0026	0.0167	-0.0351	0.577	0.7196886	Both
Water_content	nifh_Cluster114	0.0332	0.0019	0.0182	-0.0335	0.5596	0.7093883	Both
Water_content	nifh_Cluster1141	0.0251	-0.0084	0.127	0.0811	0.1128	0.3094693	Both
Water_content	nifh_Cluster115	0.0095	0.0003	0.0014	-0.0511	0.8699	0.9099558	Both
Water_content	nifh_Cluster116	0.0775	-0.0176	0.1372	0.0918	0.0984	0.2854894	Both
Water_content	nifh_Cluster1163	0.0044	-0.0012	0.0599	0.0104	0.2849	0.4972929	Both
Water_content	nifh_Cluster1179	0.0031	-0.0009	0.0254	-0.0258	0.4897	0.6610748	Both
Water_content	nifh_Cluster1182	0.0204	-0.0004	0.0021	-0.0504	0.8436	0.8997038	Both
Water_content	nifh_Cluster1183	0.0066	0.0032	0.2838	0.2461	0.0129	0.1139491	Both

Water_content	nifh_Cluster1197	0.0115	-0.0025	0.0608	0.0113	0.2814	0.4972929	Both
Water_content	nifh_Cluster1199	0.0046	-0.0002	0.002	-0.0506	0.8487	0.9022016	Both
Water_content	nifh_Cluster120	0.0149	-0.0045	0.0253	-0.026	0.491	0.6610748	Both
Water_content	nifh_Cluster1203	0.0155	-0.01	0.2546	0.2153	0.0197	0.1398137	Both
Water_content	nifh_Cluster1206	0.0043	-0.002	0.1382	0.0928	0.097	0.2834609	Both
Water_content	nifh_Cluster1207	0.0074	-0.0023	0.0605	0.011	0.2826	0.4972929	Both
Water_content	nifh_Cluster1209	0.1506	-0.0153	0.0757	0.0271	0.2273	0.4515929	Both
Water_content	nifh_Cluster121	0.0095	-0.0048	0.0775	0.0289	0.2218	0.44863	Both
Water_content	nifh_Cluster1211	0.0056	-0.0014	0.0516	0.0016	0.3223	0.5314216	Both
Water_content	nifh_Cluster1213	0.0026	-0.0006	0.0266	-0.0246	0.4798	0.6610748	Both
Water_content	nifh_Cluster122	0.0029	-0.0018	0.1165	0.07	0.1299	0.3318614	Both
Water_content	nifh_Cluster123	0.0095	0.0017	0.0106	-0.0415	0.6568	0.7750062	Both
Water_content	nifh_Cluster1236	0.0234	-0.0144	0.1518	0.1072	0.0808	0.2655966	Both
Water_content	nifh_Cluster1238	0.0028	-0.0008	0.0262	-0.0251	0.4835	0.6610748	Both
Water_content	nifh_Cluster124	0.0091	-0.0004	0.0017	-0.0509	0.8605	0.902735	Both
Water_content	nifh_Cluster1261	0.002	-0.0006	0.0231	-0.0283	0.5106	0.6711959	Both
Water_content	nifh_Cluster1262	0.0065	-0.0014	0.0245	-0.0268	0.498	0.6638719	Both
Water_content	nifh_Cluster1266	0.0094	0.0049	0.2356	0.1954	0.0257	0.1579631	Both
Water_content	nifh_Cluster1267	0.0562	0.0425	0.4841	0.4569	0.0005	0.011338	Both
Water_content	nifh_Cluster127	0.0026	-0.0011	0.045	-0.0053	0.3559	0.5602953	Both
Water_content	nifh_Cluster1278	0.1602	-0.0649	0.2448	0.205	0.0226	0.1510123	Both
Water_content	nifh_Cluster128	0.0036	-0.0007	0.0177	-0.034	0.5656	0.7115404	Both
Water_content	nifh_Cluster1289	0.0039	-0.0003	0.0039	-0.0485	0.7875	0.8604005	Both
Water_content	nifh_Cluster129	0.0077	0.00E+00	0.00E+00	-0.0526	0.9974	0.9974423	Both
Water_content	nifh_Cluster1292	0.0086	-0.0028	0.0772	0.0286	0.2227	0.44863	Both
Water_content	nifh_Cluster130	0.0059	-0.0017	0.0205	-0.0311	0.5362	0.6868316	Both
Water_content	nifh_Cluster1305	0.0048	0.001	0.036	-0.0148	0.4102	0.5969009	Both
Water_content	nifh_Cluster1306	0.005	-0.0019	0.0934	0.0456	0.178	0.3963175	Both
Water_content	nifh_Cluster131	0.0051	-0.0018	0.0423	-0.0081	0.3712	0.5717661	Both
Water_content	nifh_Cluster1319	0.0036	-0.0014	0.0592	0.0097	0.288	0.4996695	Both
Water_content	nifh_Cluster132	0.0064	0.0014	0.0223	-0.0292	0.5185	0.6738587	Both
Water_content	nifh_Cluster1320	0.0025	-0.0002	0.0017	-0.0508	0.8578	0.902735	Both
Water_content	nifh_Cluster1323	0.0023	-0.0002	0.004	-0.0484	0.7841	0.859904	Both
Water_content	nifh_Cluster1324	0.03	0.0097	0.1263	0.0803	0.1139	0.3110503	Both
Water_content	nifh_Cluster1336	0.0153	0.0008	0.0094	-0.0427	0.6752	0.7920188	Both
Water_content	nifh_Cluster1340	0.0363	-0.0133	0.0692	0.0202	0.2491	0.464486	Both
Water_content	nifh_Cluster1345	0.0483	0.0329	0.3913	0.3592	0.0024	0.0392816	Both
Water_content	nifh_Cluster1370	0.0021	-0.0003	0.0096	-0.0425	0.672	0.7898378	Both
Water_content	nifh_Cluster1375	0.0018	0.00E+00	1.00E-04	-0.0526	0.9699	0.9848331	Both
Water_content	nifh_Cluster139	0.0041	0.0014	0.0488	-0.0013	0.3361	0.5447637	Both
Water_content	nifh_Cluster140	0.0043	-0.0015	0.0805	0.0321	0.2127	0.4398974	Both
Water_content	nifh_Cluster141	0.0677	-0.0347	0.591	0.5695	0.00E+00	0.0025021	Both
Water_content	nifh_Cluster148	0.0046	-0.0022	0.0712	0.0223	0.2424	0.4621257	Both
Water_content	nifh_Cluster1480	0.001	0.0014	0.4527	0.4238	0.0008	0.0175299	Both
Water_content	nifh_Cluster152	0.0599	0.0032	0.0088	-0.0433	0.6856	0.7952112	Both
Water_content	nifh_Cluster156	0.0659	0.0408	0.6796	0.6627	0.00E+00	0.0005101	Both
Water_content	nifh_Cluster1562	0.0543	0.0523	0.6261	0.6064	0.00E+00	0.0016377	Both
Water_content	nifh_Cluster158	0.0144	-0.0069	0.1622	0.1181	0.0703	0.2533016	Both
Water_content	nifh_Cluster16	0.0019	-0.0008	0.036	-0.0147	0.4101	0.5969009	Both
Water_content	nifh_Cluster166	0.0172	0.0037	0.0145	-0.0374	0.603	0.7312193	Both
Water_content	nifh_Cluster205	0.0122	0.0008	0.0042	-0.0482	0.7804	0.8587231	Both
Water_content	nifh_Cluster21	0.0021	-0.0015	0.1713	0.1277	0.0621	0.2450608	Both
Water_content	nifh_Cluster222	0.006	-0.0005	0.0023	-0.0503	0.8381	0.8974192	Both
Water_content	nifh_Cluster225	0.0189	-0.0017	0.0178	-0.0339	0.5643	0.711449	Both
Water_content	nifh_Cluster230	0.0042	-0.0017	0.1095	0.0626	0.1429	0.3527661	Both

Water_content	nifh_Cluster231	0.0022	0.0007	0.0388	-0.0118	0.3919	0.5839256	Both
Water_content	nifh_Cluster236	0.0027	-0.0017	0.1965	0.1542	0.0442	0.2085212	Both
Water_content	nifh_Cluster237	0.0058	-0.002	0.0969	0.0494	0.1695	0.3840619	Both
Water_content	nifh_Cluster243	0.0333	0.0106	0.1034	0.0563	0.1551	0.370484	Both
Water_content	nifh_Cluster2432	0.0436	0.0302	0.4763	0.4487	0.0005	0.0121511	Both
Water_content	nifh_Cluster2433	0.0032	0.0016	0.0737	0.025	0.2338	0.4573135	Both
Water_content	nifh_Cluster246	0.0057	-0.0013	0.0229	-0.0285	0.5123	0.6711959	Both
Water_content	nifh_Cluster25	0.0295	0.0168	0.5357	0.5113	0.0002	0.006843	Both
Water_content	nifh_Cluster256	0.0029	-0.0004	0.0093	-0.0429	0.6781	0.7938458	Both
Water_content	nifh_Cluster264	0.0029	0.0013	0.1156	0.0691	0.1315	0.3344101	Both
Water_content	nifh_Cluster265	0.0736	0.0183	0.2589	0.2199	0.0185	0.1366219	Both
Water_content	nifh_Cluster267	0.0109	0.0059	0.2128	0.1713	0.0353	0.179609	Both
Water_content	nifh_Cluster268	0.0111	0.0017	0.0389	-0.0117	0.3913	0.5839256	Both
Water_content	nifh_Cluster269	0.023	-0.0087	0.1102	0.0634	0.1414	0.3519934	Both
Water_content	nifh_Cluster272	0.0041	0.0059	0.7105	0.6952	0.00E+00	0.0004136	Both
Water_content	nifh_Cluster274	0.0032	0.0032	0.096	0.0484	0.1717	0.3865619	Both
Water_content	nifh_Cluster275	0.0036	-0.0021	0.2242	0.1834	0.0301	0.1710426	Both
Water_content	nifh_Cluster278	0.0077	0.0049	0.3031	0.2665	0.0097	0.0981828	Both
Water_content	nifh_Cluster279	0.006	0.0016	0.09	0.0421	0.1865	0.4049055	Both
Water_content	nifh_Cluster28	0.002	1.00E-04	0.0004	-0.0522	0.9345	0.955581	Both
Water_content	nifh_Cluster280	0.0031	-0.0015	0.0615	0.0121	0.2785	0.4972929	Both
Water_content	nifh_Cluster282	0.0052	0.0003	0.0034	-0.0491	0.8018	0.8696087	Both
Water_content	nifh_Cluster287	0.0022	-0.0005	0.0238	-0.0275	0.504	0.6679035	Both
Water_content	nifh_Cluster290	0.011	0.001	0.0074	-0.0448	0.7104	0.8145976	Both
Water_content	nifh_Cluster297	0.0029	-0.0021	0.0923	0.0445	0.1806	0.400554	Both
Water_content	nifh_Cluster299	0.0026	0.0008	0.0288	-0.0224	0.4624	0.6464595	Both
Water_content	nifh_Cluster303	0.002	0.0012	0.1185	0.0721	0.1264	0.3318614	Both
Water_content	nifh_Cluster304	0.0024	-0.0004	0.0143	-0.0376	0.606	0.7319793	Both
Water_content	nifh_Cluster313	0.0103	-0.0023	0.0167	-0.035	0.5763	0.7196886	Both
Water_content	nifh_Cluster314	0.0022	-0.0003	0.0056	-0.0468	0.7475	0.8415161	Both
Water_content	nifh_Cluster317	0.0071	-0.0035	0.1842	0.1412	0.0522	0.2257305	Both
Water_content	nifh_Cluster32	0.0033	-0.0005	0.0154	-0.0364	0.5923	0.72801	Both
Water_content	nifh_Cluster320	12.7419	-2.2192	0.4996	0.4733	0.0003	0.0100954	Both
Water_content	nifh_Cluster334	0.0063	-0.0012	0.0089	-0.0433	0.684	0.7952112	Both
Water_content	nifh_Cluster335	0.0075	0.0038	0.0545	0.0047	0.3085	0.5215395	Both
Water_content	nifh_Cluster338	0.0123	-0.0043	0.142	0.0969	0.0922	0.2732962	Both
Water_content	nifh_Cluster340	0.007	0.0053	0.0823	0.034	0.2073	0.4351769	Both
Water_content	nifh_Cluster341	0.0023	0.0006	0.0198	-0.0318	0.5433	0.6938867	Both
Water_content	nifh_Cluster35	0.0078	-0.0017	0.0571	0.0075	0.2968	0.5075165	Both
Water_content	nifh_Cluster351	0.0025	-0.0008	0.0245	-0.0269	0.4985	0.6638719	Both
Water_content	nifh_Cluster3574	0.1093	-0.0248	0.1676	0.1238	0.0654	0.2504196	Both
Water_content	nifh_Cluster3691	0.0174	-0.0089	0.1563	0.1119	0.0761	0.2584663	Both
Water_content	nifh_Cluster37	0.0329	-0.0065	0.0303	-0.0207	0.4505	0.6386786	Both
Water_content	nifh_Cluster374	0.0021	-0.0009	0.0511	0.0012	0.3244	0.5316137	Both
Water_content	nifh_Cluster38	0.0009	0.0001	0.005	-0.0474	0.7616	0.8462698	Both
Water_content	nifh_Cluster382	0.0016	-0.0012	0.0704	0.0215	0.2449	0.4631062	Both
Water_content	nifh_Cluster384	0.0019	-0.0005	0.0263	-0.0249	0.4823	0.6610748	Both
Water_content	nifh_Cluster386	0.0035	0.0001	0.0004	-0.0522	0.9288	0.9513644	Both
Water_content	nifh_Cluster389	0.0031	0.0011	0.0377	-0.013	0.3993	0.5889397	Both
Water_content	nifh_Cluster394	0.002	-0.0007	0.0183	-0.0333	0.5586	0.7093883	Both
Water_content	nifh_Cluster40	0.0012	-0.0007	0.1035	0.0563	0.155	0.370484	Both
Water_content	nifh_Cluster43	0.0108	0.0018	0.0452	-0.005	0.3547	0.5602953	Both
Water_content	nifh_Cluster434	0.0035	-0.0021	0.1179	0.0714	0.1276	0.3318614	Both
Water_content	nifh_Cluster440	0.0133	-0.0057	0.1071	0.0601	0.1475	0.3596728	Both
Water_content	nifh_Cluster462	0.0034	-0.0009	0.0254	-0.0259	0.4902	0.6610748	Both

Water_content	nifh_Cluster470	0.0152	0.0004	0.0007	-0.0519	0.9082	0.935184	Both
Water_content	nifh_Cluster49	0.0213	-0.0015	0.0021	-0.0504	0.843	0.8997038	Both
Water_content	nifh_Cluster499	0.0026	-0.0001	0.0008	-0.0518	0.9034	0.9318713	Both
Water_content	nifh_Cluster5	0.0256	-0.0007	0.0031	-0.0494	0.8111	0.8749038	Both
Water_content	nifh_Cluster506	0.0048	0.00E+00	0.00E+00	-0.0526	0.9834	0.9896808	Both
Water_content	nifh_Cluster51	0.0028	-0.0004	0.0046	-0.0478	0.7694	0.8516841	Both
Water_content	nifh_Cluster52	0.0336	-0.0066	0.0228	-0.0286	0.5131	0.6711959	Both
Water_content	nifh_Cluster525	0.0033	-0.002	0.0598	0.0103	0.2855	0.4972929	Both
Water_content	nifh_Cluster531	0.0258	0.0238	0.7351	0.7212	0.00E+00	0.0004041	Both
Water_content	nifh_Cluster539	0.0048	-1.00E-04	1.00E-04	-0.0526	0.9715	0.9848331	Both
Water_content	nifh_Cluster578	0.0481	-0.011	0.0894	0.0414	0.188	0.4049055	Both
Water_content	nifh_Cluster58	0.3542	0.2489	0.4983	0.4719	0.0003	0.0100954	Both
Water_content	nifh_Cluster60	0.0089	-0.0031	0.0768	0.0282	0.2239	0.44863	Both
Water_content	nifh_Cluster61	0.0025	0.0005	0.0219	-0.0296	0.5219	0.6752675	Both
Water_content	nifh_Cluster65	0.009	0.0011	0.008	-0.0443	0.7006	0.8072754	Both
Water_content	nifh_Cluster686	0.0009	-0.0003	0.0175	-0.0342	0.5677	0.7126013	Both
Water_content	nifh_Cluster69	0.009	-0.0033	0.0711	0.0222	0.2428	0.4621257	Both
Water_content	nifh_Cluster70	0.0111	-0.0042	0.0404	-0.0102	0.3826	0.5800219	Both
Water_content	nifh_Cluster717	0.003	0.0017	0.1905	0.1479	0.0479	0.2191055	Both
Water_content	nifh_Cluster725	0.0058	0.0031	0.2199	0.1789	0.032	0.1764033	Both
Water_content	nifh_Cluster727	0.0039	-0.0017	0.0896	0.0417	0.1874	0.4049055	Both
Water_content	nifh_Cluster73	0.0106	-0.0032	0.0515	0.0016	0.3226	0.5314216	Both
Water_content	nifh_Cluster74	0.0049	0.0004	0.0018	-0.0508	0.8563	0.902735	Both
Water_content	nifh_Cluster748	0.0081	-0.0016	0.0535	0.0037	0.3129	0.5251559	Both
Water_content	nifh_Cluster749	0.0071	0.0024	0.117	0.0705	0.1291	0.3318614	Both
Water_content	nifh_Cluster755	0.0102	-0.0052	0.1617	0.1176	0.0707	0.2533016	Both
Water_content	nifh_Cluster76	0.0029	-0.0009	0.0466	-0.0036	0.3475	0.5586921	Both
Water_content	nifh_Cluster79	0.0022	-0.0001	0.0019	-0.0506	0.8504	0.9023537	Both
Water_content	nifh_Cluster81	0.0055	-0.0008	0.0061	-0.0462	0.7356	0.8346509	Both
Water_content	nifh_Cluster82	0.0011	-0.0006	0.0671	0.018	0.2567	0.4733126	Both
Water_content	nifh_Cluster83	0.0069	0.0019	0.038	-0.0126	0.3971	0.5871259	Both
Water_content	nifh_Cluster86	0.0135	-0.0055	0.1804	0.1373	0.055	0.2310813	Both
Water_content	nifh_Cluster868	0.0099	-0.0003	0.001	-0.0516	0.8937	0.9234821	Both
Water_content	nifh_Cluster87	0.0024	-0.0005	0.0113	-0.0408	0.6471	0.7697132	Both
Water_content	nifh_Cluster88	0.0039	-0.0014	0.0449	-0.0054	0.3564	0.5602953	Both
Water_content	nifh_Cluster9	0.0078	-0.0005	0.0029	-0.0495	0.8155	0.8779784	Both
Water_content	nifh_Cluster92	0.0051	-0.0016	0.0269	-0.0244	0.4778	0.6602002	Both
Water_content	nifh_Cluster93	0.0036	0.0007	0.0167	-0.0351	0.5768	0.7196886	Both
Water_content	nifh_Cluster94	0.0047	-0.0005	0.0031	-0.0493	0.8092	0.8744187	Both
Water_content	nifh_Cluster95	0.0121	-0.0019	0.0106	-0.0414	0.6566	0.7750062	Both
Water_content	nifh_Cluster96	0.0123	0.00E+00	0.00E+00	-0.0526	0.9948	0.9965042	Both
Water_content	nifh_Cluster97	0.018	0.0145	0.4969	0.4704	0.0004	0.0100954	Both
Water_content	nifh_Cluster98	0.0059	0.0008	0.0159	-0.0359	0.5856	0.7242967	Both
Water_content	nifh_Cluster99	0.0033	-0.0002	0.0012	-0.0513	0.8796	0.912079	Both
Water_content	nirk_Cluster0	5.9009	-1.2402	0.6859	0.6694	0.00E+00	0.0005101	Both
Water_content	nirk_Cluster1	0.1708	-0.1021	0.5571	0.5338	0.0001	0.0046322	Both
Water_content	nirk_Cluster10	0.5713	-0.1332	0.2717	0.2334	0.0154	0.1278409	Both
Water_content	nirk_Cluster101	0.0031	-0.001	0.0383	-0.0123	0.3951	0.5856516	Both
Water_content	nirk_Cluster102	0.0426	-0.0181	0.2343	0.194	0.0262	0.1593042	Both
Water_content	nirk_Cluster103	0.0754	-0.0333	0.3326	0.2975	0.0062	0.0732226	Both
Water_content	nirk_Cluster104	0.0023	-0.0012	0.0873	0.0393	0.1935	0.4106746	Both
Water_content	nirk_Cluster105	0.0946	-0.0421	0.3491	0.3149	0.0048	0.0642982	Both
Water_content	nirk_Cluster106	0.0592	-0.0239	0.1648	0.1209	0.0678	0.2517324	Both
Water_content	nirk_Cluster107	0.0891	-0.04	0.3103	0.274	0.0087	0.0918118	Both
Water_content	nirk_Cluster108	0.0151	-0.0021	0.0183	-0.0334	0.5587	0.7093883	Both

Water_content	nirk_Cluster109	0.0115	-0.006	0.2162	0.175	0.0337	0.1781842	Both
Water_content	nirk_Cluster11	0.1401	-0.0101	0.0832	0.035	0.2047	0.431419	Both
Water_content	nirk_Cluster12	0.094	-0.0271	0.3499	0.3156	0.0047	0.0642982	Both
Water_content	nirk_Cluster13	0.0408	-0.0023	0.0219	-0.0295	0.5217	0.6752675	Both
Water_content	nirk_Cluster14	0.0946	-0.0266	0.3223	0.2866	0.0073	0.0824885	Both
Water_content	nirk_Cluster15	0.3358	-0.1331	0.2837	0.2461	0.0129	0.1139491	Both
Water_content	nirk_Cluster16	0.4727	-0.1782	0.2357	0.1955	0.0257	0.1579631	Both
Water_content	nirk_Cluster17	0.0998	-0.0074	0.0388	-0.0118	0.3923	0.5839256	Both
Water_content	nirk_Cluster18	0.1296	-0.0328	0.1229	0.0767	0.1193	0.3220835	Both
Water_content	nirk_Cluster19	0.2776	-0.0287	0.1168	0.0703	0.1294	0.3318614	Both
Water_content	nirk_Cluster2	0.2032	0.0011	0.0003	-0.0524	0.9457	0.9636863	Both
Water_content	nirk_Cluster20	0.1738	-0.0208	0.0523	0.0025	0.3185	0.5307819	Both
Water_content	nirk_Cluster21	0.1532	-0.0273	0.2599	0.2209	0.0182	0.1366219	Both
Water_content	nirk_Cluster22	0.1291	-0.0311	0.2557	0.2166	0.0194	0.1392285	Both
Water_content	nirk_Cluster23	0.0673	-0.0085	0.1542	0.1097	0.0783	0.2609639	Both
Water_content	nirk_Cluster24	0.0654	-0.0209	0.3329	0.2978	0.0062	0.0732226	Both
Water_content	nirk_Cluster25	0.4782	-0.1846	0.6609	0.6431	0.00E+00	0.0007362	Both
Water_content	nirk_Cluster26	0.105	-0.0176	0.215	0.1736	0.0343	0.1789148	Both
Water_content	nirk_Cluster27	0.1727	-0.0532	0.3122	0.276	0.0085	0.0907794	Both
Water_content	nirk_Cluster28	0.0685	-0.0138	0.1366	0.0911	0.0992	0.2855084	Both
Water_content	nirk_Cluster29	0.1526	-0.0079	0.0182	-0.0335	0.5603	0.7093883	Both
Water_content	nirk_Cluster3	0.3023	-0.0628	0.2733	0.2351	0.015	0.1266987	Both
Water_content	nirk_Cluster30	0.0556	-0.0224	0.2662	0.2276	0.0167	0.1345915	Both
Water_content	nirk_Cluster31	0.1501	0.0189	0.1975	0.1553	0.0436	0.2072322	Both
Water_content	nirk_Cluster32	0.1254	-0.059	0.294	0.2569	0.0111	0.1073757	Both
Water_content	nirk_Cluster33	0.0638	-0.0297	0.2579	0.2189	0.0188	0.1366219	Both
Water_content	nirk_Cluster34	0.0416	-0.0068	0.0603	0.0108	0.2833	0.4972929	Both
Water_content	nirk_Cluster35	0.1007	-0.0494	0.2836	0.2459	0.0129	0.1139491	Both
Water_content	nirk_Cluster36	0.0017	-0.0009	0.08	0.0316	0.214	0.4398974	Both
Water_content	nirk_Cluster39	0.0035	-0.0002	0.0005	-0.0521	0.9244	0.9485387	Both
Water_content	nirk_Cluster4	0.1323	-0.026	0.1554	0.1109	0.077	0.2592409	Both
Water_content	nirk_Cluster40	0.0455	-0.0167	0.3921	0.3602	0.0024	0.0392816	Both
Water_content	nirk_Cluster41	0.1562	-0.0469	0.5051	0.479	0.0003	0.0100103	Both
Water_content	nirk_Cluster44	0.0669	-0.0322	0.3188	0.2829	0.0077	0.0847889	Both
Water_content	nirk_Cluster46	0.0154	-0.0074	0.1653	0.1213	0.0674	0.2517324	Both
Water_content	nirk_Cluster48	0.003	-0.0015	0.1169	0.0704	0.1293	0.3318614	Both
Water_content	nirk_Cluster49	0.0057	-0.0033	0.1427	0.0976	0.0913	0.2732962	Both
Water_content	nirk_Cluster5	0.1932	-0.0332	0.228	0.1874	0.0286	0.1637915	Both
Water_content	nirk_Cluster50	0.0016	0.00E+00	0.0003	-0.0524	0.9443	0.9636863	Both
Water_content	nirk_Cluster52	0.0452	-0.0177	0.3419	0.3072	0.0054	0.0686845	Both
Water_content	nirk_Cluster53	0.0038	-0.0014	0.0506	0.0007	0.3267	0.5333791	Both
Water_content	nirk_Cluster54	0.002	-0.0009	0.0737	0.025	0.2338	0.4573135	Both
Water_content	nirk_Cluster55	0.0023	-0.0012	0.055	0.0052	0.3064	0.5194034	Both
Water_content	nirk_Cluster56	0.0009	-0.0004	0.0333	-0.0176	0.4288	0.6186109	Both
Water_content	nirk_Cluster57	0.0016	0.00E+00	1.00E-04	-0.0526	0.9733	0.984959	Both
Water_content	nirk_Cluster58	0.0088	-0.0037	0.1369	0.0915	0.0987	0.2854894	Both
Water_content	nirk_Cluster59	0.0058	-0.0016	0.0534	0.0035	0.3137	0.5251559	Both
Water_content	nirk_Cluster6	0.1558	-0.0372	0.2861	0.2485	0.0125	0.1139491	Both
Water_content	nirk_Cluster60	0.0006	0.0003	0.0246	-0.0267	0.497	0.6638719	Both
Water_content	nirk_Cluster62	0.1417	-0.0774	0.4185	0.3879	0.0015	0.0290506	Both
Water_content	nirk_Cluster63	0.1284	-0.0732	0.7025	0.6869	0.00E+00	0.0004136	Both
Water_content	nirk_Cluster64	0.0044	-0.0002	0.0021	-0.0505	0.8448	0.8997038	Both
Water_content	nirk_Cluster65	0.0464	-0.0156	0.2949	0.2578	0.011	0.1073757	Both
Water_content	nirk_Cluster66	0.0068	-0.005	0.3903	0.3582	0.0025	0.0392816	Both
Water_content	nirk_Cluster67	0.0174	-0.0065	0.1023	0.0551	0.1575	0.3731647	Both

Water_content	nirk_Cluster68	0.0125	-0.0081	0.396	0.3642	0.0022	0.0388906	Both
Water_content	nirk_Cluster69	0.0305	-0.0124	0.342	0.3073	0.0054	0.0686845	Both
Water_content	nirk_Cluster7	0.0804	-0.0249	0.2909	0.2535	0.0116	0.1107129	Both
Water_content	nirk_Cluster70	0.0721	-0.0436	0.2758	0.2377	0.0145	0.1239139	Both
Water_content	nirk_Cluster71	0.0053	-0.0018	0.0732	0.0244	0.2356	0.4573135	Both
Water_content	nirk_Cluster72	0.0239	-0.0116	0.4597	0.4313	0.0007	0.0159714	Both
Water_content	nirk_Cluster73	0.1444	-0.0873	0.608	0.5874	0.00E+00	0.001817	Both
Water_content	nirk_Cluster74	0.6398	-0.3758	0.5266	0.5017	0.0002	0.0072555	Both
Water_content	nirk_Cluster75	0.0147	-0.0114	0.3229	0.2873	0.0072	0.0824885	Both
Water_content	nirk_Cluster76	0.1055	-0.0546	0.6188	0.5987	0.00E+00	0.0016958	Both
Water_content	nirk_Cluster77	0.0081	-0.0029	0.0279	-0.0232	0.469	0.6541858	Both
Water_content	nirk_Cluster78	0.2407	-0.1204	0.4905	0.4637	0.0004	0.0109283	Both
Water_content	nirk_Cluster79	0.0069	-0.0027	0.0492	-0.0009	0.334	0.5429311	Both
Water_content	nirk_Cluster8	0.0982	-0.03	0.1989	0.1567	0.0427	0.2049696	Both
Water_content	nirk_Cluster80	0.2665	-0.1584	0.5666	0.5438	1.00E-04	0.0040507	Both
Water_content	nirk_Cluster82	0.0015	-0.0012	0.1001	0.0528	0.1622	0.3783689	Both
Water_content	nirk_Cluster83	0.004	-0.0011	0.0459	-0.0043	0.3508	0.5602953	Both
Water_content	nirk_Cluster84	0.0207	-0.0017	0.005	-0.0473	0.7603	0.8462698	Both
Water_content	nirk_Cluster85	0.004	-0.0023	0.1745	0.1311	0.0595	0.2397392	Both
Water_content	nirk_Cluster86	0.0286	-0.0176	0.4124	0.3814	0.0017	0.030356	Both
Water_content	nirk_Cluster87	0.0115	-0.0075	0.3801	0.3475	0.0029	0.0452393	Both
Water_content	nirk_Cluster88	0.0032	-0.0008	0.0395	-0.011	0.3875	0.5832852	Both
Water_content	nirk_Cluster89	0.0065	-0.0027	0.091	0.0432	0.1838	0.4031167	Both
Water_content	nirk_Cluster9	0.0517	-0.0097	0.0618	0.0124	0.2774	0.4972929	Both
Water_content	nirk_Cluster90	0.0067	-0.0028	0.0795	0.0311	0.2156	0.4400816	Both
Water_content	nirk_Cluster91	0.0374	-0.0148	0.2784	0.2404	0.014	0.1212127	Both
Water_content	nirk_Cluster92	0.0072	-0.0037	0.1443	0.0993	0.0894	0.2732533	Both
Water_content	nirk_Cluster93	0.0308	-0.0059	0.0646	0.0154	0.2662	0.4893376	Both
Water_content	nirk_Cluster94	0.0175	-0.0065	0.1511	0.1065	0.0816	0.2655966	Both
Water_content	nirk_Cluster95	0.0473	-0.0214	0.265	0.2263	0.0169	0.1350614	Both
Water_content	nirk_Cluster96	0.0472	-0.0182	0.2411	0.2012	0.0238	0.1525671	Both
Water_content	nirk_Cluster97	0.0306	-0.0078	0.108	0.061	0.1459	0.3586568	Both
Water_content	nirk_Cluster98	0.0181	-0.0061	0.0816	0.0332	0.2095	0.4366681	Both
Water_content	nirk_Cluster99	0.006	-0.0029	0.1314	0.0857	0.1063	0.301982	Both
Water_content	nirs_Cluster0	0.1409	-0.0326	0.2283	0.1877	0.0285	0.1637915	Both
Water_content	nirs_Cluster1	0.004	-0.0011	0.0254	-0.0259	0.4901	0.6610748	Both
Water_content	nirs_Cluster14	0.0107	-0.0039	0.0294	-0.0217	0.4573	0.6439924	Both
Water_content	nirs_Cluster2	0.0139	-0.0048	0.0438	-0.0065	0.3625	0.5653887	Both
Water_content	nirs_Cluster26	0.0005	-1.00E-04	0.0018	-0.0508	0.8563	0.902735	Both
Water_content	nirs_Cluster28	0.0071	-0.0041	0.0712	0.0223	0.2423	0.4621257	Both
Water_content	nirs_Cluster3	0.1715	-0.029	0.2486	0.209	0.0214	0.1469995	Both
Water_content	nirs_Cluster36	0.0013	-0.0004	0.0074	-0.0449	0.711	0.8145976	Both
Water_content	nirs_Cluster37	0.0066	-0.0032	0.0269	-0.0243	0.4776	0.6602002	Both
Water_content	nirs_Cluster4	0.0002	-0.0001	0.0145	-0.0374	0.6036	0.7312193	Both
Water_content	nirs_Cluster50	0.002	0.0003	0.0059	-0.0464	0.7401	0.8380867	Both
Water_content	nirs_Cluster9	0.0269	-0.023	0.151	0.1063	0.0817	0.2655966	Both
Water_content	norb_Cluster0	0.0775	-0.025	0.1706	0.127	0.0627	0.2450608	Both
Water_content	norb_Cluster1	1.1042	-0.3868	0.147	0.1021	0.0862	0.2723188	Both
Water_content	norb_Cluster101	0.0026	-0.0023	0.185	0.1421	0.0516	0.2257305	Both
Water_content	norb_Cluster102	0.0038	-0.0018	0.1071	0.0601	0.1475	0.3596728	Both
Water_content	norb_Cluster103	0.0018	-0.001	0.0694	0.0204	0.2485	0.464486	Both
Water_content	norb_Cluster104	0.0023	0.00E+00	0.00E+00	-0.0526	0.9863	0.9896808	Both
Water_content	norb_Cluster106	0.125	-0.0376	0.1551	0.1106	0.0773	0.2592409	Both
Water_content	norb_Cluster107	0.0133	-0.0049	0.1049	0.0578	0.152	0.3660809	Both
Water_content	norb_Cluster108	0.0052	-0.0024	0.1565	0.1121	0.0759	0.2584663	Both

Water_content	norb_Cluster109	0.0227	-0.0068	0.0968	0.0492	0.1699	0.3840619	Both
Water_content	norb_Cluster11	0.0126	-0.0072	0.2458	0.2061	0.0223	0.1510123	Both
Water_content	norb_Cluster110	0.0099	-0.0054	0.2	0.1579	0.0421	0.2043556	Both
Water_content	norb_Cluster112	0.0171	-0.0062	0.1647	0.1208	0.0679	0.2517324	Both
Water_content	norb_Cluster113	0.0134	-0.0034	0.0453	-0.005	0.3545	0.5602953	Both
Water_content	norb_Cluster114	0.0025	-0.001	0.0251	-0.0262	0.493	0.6610748	Both
Water_content	norb_Cluster115	0.0137	0.0169	0.1177	0.0713	0.1278	0.3318614	Both
Water_content	norb_Cluster116	0.0072	-0.0007	0.0045	-0.0479	0.773	0.8524368	Both
Water_content	norb_Cluster117	0.0027	-0.0008	0.033	-0.0179	0.431	0.6201674	Both
Water_content	norb_Cluster118	0.0037	-0.0015	0.0916	0.0438	0.1824	0.4015657	Both
Water_content	norb_Cluster119	0.019	-0.0113	0.2108	0.1693	0.0363	0.1829596	Both
Water_content	norb_Cluster12	0.0047	-0.0016	0.0452	-0.005	0.3547	0.5602953	Both
Water_content	norb_Cluster121	0.0017	-0.0011	0.0701	0.0211	0.2462	0.4639911	Both
Water_content	norb_Cluster122	0.0144	-0.0044	0.0876	0.0396	0.1926	0.41018	Both
Water_content	norb_Cluster123	0.0053	-0.0024	0.099	0.0515	0.1649	0.3830133	Both
Water_content	norb_Cluster124	0.682	-0.1254	0.0513	0.0014	0.3234	0.5314216	Both
Water_content	norb_Cluster125	0.004	-0.002	0.1426	0.0975	0.0914	0.2732962	Both
Water_content	norb_Cluster126	0.0082	-0.0043	0.1869	0.1441	0.0503	0.2257305	Both
Water_content	norb_Cluster127	0.1735	-0.055	0.1308	0.0851	0.1071	0.301982	Both
Water_content	norb_Cluster128	0.0115	-0.0035	0.1013	0.054	0.1597	0.3766694	Both
Water_content	norb_Cluster13	0.0045	-0.0029	0.1306	0.0848	0.1075	0.301982	Both
Water_content	norb_Cluster131	0.0184	-0.0037	0.0409	-0.0096	0.3795	0.5775699	Both
Water_content	norb_Cluster132	1.0357	-0.3707	0.2701	0.2317	0.0157	0.1290215	Both
Water_content	norb_Cluster133	0.0018	-0.0008	0.061	0.0115	0.2806	0.4972929	Both
Water_content	norb_Cluster135	0.0567	-0.0179	0.1307	0.0849	0.1074	0.301982	Both
Water_content	norb_Cluster136	0.0213	-0.0053	0.0732	0.0244	0.2356	0.4573135	Both
Water_content	norb_Cluster139	0.0328	-0.0119	0.1819	0.1388	0.0539	0.228584	Both
Water_content	norb_Cluster14	0.0026	-0.0022	0.2521	0.2128	0.0204	0.1413545	Both
Water_content	norb_Cluster140	0.0437	-0.0215	0.318	0.2821	0.0078	0.0847889	Both
Water_content	norb_Cluster142	0.0015	-0.0014	0.1486	0.1037	0.0844	0.2722238	Both
Water_content	norb_Cluster144	0.0098	-0.0022	0.0424	-0.008	0.3705	0.5717661	Both
Water_content	norb_Cluster146	0.0348	-0.0103	0.1333	0.0877	0.1037	0.2969157	Both
Water_content	norb_Cluster15	0.0469	-0.0129	0.0884	0.0404	0.1906	0.4075072	Both
Water_content	norb_Cluster150	0.0157	-0.0068	0.2587	0.2196	0.0186	0.1366219	Both
Water_content	norb_Cluster155	0.1631	-0.0308	0.0597	0.0102	0.2857	0.4972929	Both
Water_content	norb_Cluster16	0.0131	-0.0086	0.2598	0.2209	0.0183	0.1366219	Both
Water_content	norb_Cluster164	0.0199	-0.0122	0.2841	0.2464	0.0128	0.1139491	Both
Water_content	norb_Cluster165	0.0076	-0.0025	0.0483	-0.0018	0.3384	0.5469339	Both
Water_content	norb_Cluster166	0.0039	-0.0007	0.0145	-0.0373	0.6028	0.7312193	Both
Water_content	norb_Cluster169	0.0238	-0.0138	0.2363	0.1962	0.0254	0.1579631	Both
Water_content	norb_Cluster17	0.0028	-0.002	0.2133	0.1719	0.0351	0.179609	Both
Water_content	norb_Cluster170	0.0243	-0.0066	0.0448	-0.0055	0.3571	0.5602953	Both
Water_content	norb_Cluster171	0.0069	-0.0037	0.0976	0.0501	0.168	0.3840619	Both
Water_content	norb_Cluster172	0.014	-0.0065	0.1177	0.0712	0.1279	0.3318614	Both
Water_content	norb_Cluster175	0.0458	-0.0223	0.1453	0.1004	0.0882	0.2723188	Both
Water_content	norb_Cluster176	0.0298	-0.0173	0.2074	0.1656	0.038	0.1902127	Both
Water_content	norb_Cluster177	0.0282	-0.0105	0.0858	0.0376	0.1976	0.4179308	Both
Water_content	norb_Cluster179	0.0022	-0.0005	0.0142	-0.0377	0.6067	0.7319793	Both
Water_content	norb_Cluster18	0.0037	-0.0029	0.3529	0.3188	0.0045	0.0642982	Both
Water_content	norb_Cluster180	0.0109	-0.0032	0.0983	0.0508	0.1664	0.3834083	Both
Water_content	norb_Cluster181	0.0008	0.0009	0.1023	0.0551	0.1575	0.3731647	Both
Water_content	norb_Cluster182	0.0013	-0.0009	0.1137	0.067	0.1351	0.340543	Both
Water_content	norb_Cluster184	0.0146	-0.0056	0.1181	0.0717	0.1272	0.3318614	Both
Water_content	norb_Cluster186	0.0011	-1.00E-04	0.0006	-0.052	0.918	0.9435691	Both
Water_content	norb_Cluster19	0.0049	-0.0022	0.1649	0.121	0.0677	0.2517324	Both

Water_content	norb_Cluster192	0.0173	-0.009	0.18	0.1369	0.0552	0.2310813	Both
Water_content	norb_Cluster193	0.0021	0.00E+00	0.00E+00	-0.0526	0.9828	0.9896808	Both
Water_content	norb_Cluster197	0.0303	-0.0047	0.0037	-0.0488	0.7947	0.8634681	Both
Water_content	norb_Cluster2	0.0031	0.0002	0.0013	-0.0513	0.8788	0.912079	Both
Water_content	norb_Cluster20	0.0587	-0.018	0.1424	0.0973	0.0917	0.2732962	Both
Water_content	norb_Cluster208	0.0011	-0.0008	0.0735	0.0247	0.2346	0.4573135	Both
Water_content	norb_Cluster21	0.0481	-0.0194	0.1834	0.1404	0.0528	0.2257305	Both
Water_content	norb_Cluster211	0.0104	-0.0018	0.0055	-0.0468	0.7491	0.8415161	Both
Water_content	norb_Cluster212	0.0264	0.0034	0.0051	-0.0473	0.7594	0.8462698	Both
Water_content	norb_Cluster214	0.0119	-0.0037	0.0298	-0.0213	0.4542	0.6411471	Both
Water_content	norb_Cluster217	0.0149	-0.0032	0.0163	-0.0355	0.5811	0.7218435	Both
Water_content	norb_Cluster219	0.0083	-0.0034	0.0401	-0.0104	0.3841	0.5800219	Both
Water_content	norb_Cluster22	0.0025	-0.001	0.0401	-0.0105	0.3844	0.5800219	Both
Water_content	norb_Cluster220	0.0177	-0.0066	0.0293	-0.0218	0.4585	0.6441148	Both
Water_content	norb_Cluster221	0.0177	-0.0055	0.0178	-0.0339	0.564	0.711449	Both
Water_content	norb_Cluster222	0.0074	-0.0019	0.0091	-0.0431	0.6812	0.7942343	Both
Water_content	norb_Cluster224	0.0194	-0.0054	0.0252	-0.0261	0.4922	0.6610748	Both
Water_content	norb_Cluster225	0.0097	0.0018	0.0127	-0.0393	0.6267	0.7514936	Both
Water_content	norb_Cluster226	0.0043	-0.0009	0.0156	-0.0363	0.5902	0.726951	Both
Water_content	norb_Cluster228	0.0031	0.0034	0.0897	0.0418	0.1872	0.4049055	Both
Water_content	norb_Cluster229	0.0034	-0.0009	0.0264	-0.0248	0.4815	0.6610748	Both
Water_content	norb_Cluster230	0.0014	-0.0009	0.0783	0.0298	0.2192	0.4458972	Both
Water_content	norb_Cluster231	0.0044	-0.002	0.0533	0.0034	0.3142	0.5251559	Both
Water_content	norb_Cluster232	0.0031	-0.0015	0.0638	0.0145	0.2692	0.4917974	Both
Water_content	norb_Cluster234	0.0121	-0.0082	0.185	0.1421	0.0517	0.2257305	Both
Water_content	norb_Cluster235	0.0009	-0.0004	0.0387	-0.0119	0.3929	0.5839256	Both
Water_content	norb_Cluster238	0.0099	-0.0067	0.1422	0.097	0.092	0.2732962	Both
Water_content	norb_Cluster239	0.0081	-0.006	0.1403	0.0951	0.0943	0.278138	Both
Water_content	norb_Cluster24	0.2637	-0.0939	0.1915	0.1489	0.0473	0.2180344	Both
Water_content	norb_Cluster244	0.0011	-0.0007	0.0916	0.0438	0.1822	0.4015657	Both
Water_content	norb_Cluster247	0.0048	-0.0015	0.0767	0.0281	0.2243	0.44863	Both
Water_content	norb_Cluster25	0.1114	-0.0351	0.1227	0.0765	0.1196	0.3220835	Both
Water_content	norb_Cluster250	0.0115	-0.0037	0.0721	0.0233	0.2393	0.4613267	Both
Water_content	norb_Cluster251	0.0052	-0.0024	0.1061	0.059	0.1497	0.3634212	Both
Water_content	norb_Cluster26	0.0931	-0.0428	0.2174	0.1763	0.0331	0.1781842	Both
Water_content	norb_Cluster28	0.0302	-0.0058	0.0387	-0.0119	0.3929	0.5839256	Both
Water_content	norb_Cluster29	0.222	-0.049	0.1857	0.1428	0.0512	0.2257305	Both
Water_content	norb_Cluster31	0.0531	-0.0205	0.1745	0.1311	0.0595	0.2397392	Both
Water_content	norb_Cluster32	0.1193	-0.0479	0.1465	0.1015	0.0869	0.2723188	Both
Water_content	norb_Cluster33	0.4946	0.1064	0.1737	0.1302	0.0602	0.2399394	Both
Water_content	norb_Cluster35	0.0628	-0.0228	0.1629	0.1189	0.0696	0.2533016	Both
Water_content	norb_Cluster36	1.1345	-0.1294	0.0252	-0.0261	0.4919	0.6610748	Both
Water_content	norb_Cluster37	0.7473	-0.0447	0.0108	-0.0413	0.6544	0.7750062	Both
Water_content	norb_Cluster38	0.0116	-0.0036	0.1247	0.0786	0.1164	0.3163585	Both
Water_content	norb_Cluster39	0.0426	-0.014	0.1282	0.0823	0.111	0.3060068	Both
Water_content	norb_Cluster40	0.186	-0.0604	0.1423	0.0971	0.0919	0.2732962	Both
Water_content	norb_Cluster42	0.2864	-0.0133	0.0045	-0.0479	0.7717	0.8524368	Both
Water_content	norb_Cluster44	0.0182	-0.0112	0.2413	0.2014	0.0237	0.1525671	Both
Water_content	norb_Cluster46	0.2556	0.0232	0.0119	-0.0402	0.6385	0.7629953	Both
Water_content	norb_Cluster47	0.555	-0.1749	0.161	0.1168	0.0715	0.2539536	Both
Water_content	norb_Cluster5	0.01	-0.0029	0.11	0.0631	0.142	0.3519934	Both
Water_content	norb_Cluster51	0.0092	-0.0054	0.1294	0.0836	0.1092	0.3034987	Both
Water_content	norb_Cluster52	0.2391	-0.0801	0.1447	0.0996	0.089	0.2732533	Both
Water_content	norb_Cluster56	0.014	-0.0057	0.167	0.1231	0.0659	0.2507889	Both
Water_content	norb_Cluster57	0.0034	-0.0006	0.0211	-0.0304	0.5298	0.6810656	Both

Water_content	norb_Cluster59	0.0236	-0.0121	0.1298	0.0841	0.1086	0.3034987	Both
Water_content	norb_Cluster6	0.6862	-0.1019	0.0452	-0.005	0.3546	0.5602953	Both
Water_content	norb_Cluster60	0.3199	-0.0391	0.0118	-0.0402	0.6388	0.7629953	Both
Water_content	norb_Cluster61	0.482	-0.1308	0.0985	0.0511	0.1658	0.3834083	Both
Water_content	norb_Cluster62	0.0024	0.0005	0.0161	-0.0356	0.5831	0.7227386	Both
Water_content	norb_Cluster64	0.0326	-0.0102	0.1508	0.1061	0.0819	0.2655966	Both
Water_content	norb_Cluster66	0.0012	-0.0004	0.0215	-0.03	0.5261	0.6792272	Both
Water_content	norb_Cluster67	0.0218	-0.0091	0.1742	0.1308	0.0597	0.2397392	Both
Water_content	norb_Cluster68	0.0034	-0.0003	0.0048	-0.0476	0.7663	0.8498645	Both
Water_content	norb_Cluster69	0.0028	-0.0012	0.0675	0.0185	0.2552	0.4720807	Both
Water_content	norb_Cluster7	0.0709	-0.0143	0.0584	0.0088	0.2913	0.5026012	Both
Water_content	norb_Cluster70	0.1051	-0.0323	0.1475	0.1026	0.0857	0.2723188	Both
Water_content	norb_Cluster71	0.1295	-0.0338	0.1292	0.0833	0.1096	0.3034987	Both
Water_content	norb_Cluster74	0.0327	-0.0107	0.1684	0.1247	0.0646	0.2491699	Both
Water_content	norb_Cluster75	0.1291	-0.0427	0.2594	0.2204	0.0184	0.1366219	Both
Water_content	norb_Cluster76	0.1105	-0.0106	0.0055	-0.0469	0.7501	0.8415161	Both
Water_content	norb_Cluster77	0.0475	-0.0036	0.0145	-0.0374	0.6031	0.7312193	Both
Water_content	norb_Cluster8	0.018	-0.0045	0.06	0.0105	0.2845	0.4972929	Both
Water_content	norb_Cluster80	0.0015	-0.0009	0.0749	0.0262	0.23	0.4553513	Both
Water_content	norb_Cluster81	0.0012	-0.0001	0.0032	-0.0493	0.8076	0.8742898	Both
Water_content	norb_Cluster83	0.0078	-0.0068	0.1758	0.1325	0.0585	0.2395292	Both
Water_content	norb_Cluster84	0.0082	-0.002	0.0441	-0.0062	0.3608	0.5646152	Both
Water_content	norb_Cluster85	0.0023	-0.0007	0.0302	-0.0209	0.4514	0.6386786	Both
Water_content	norb_Cluster86	0.031	-0.0104	0.0318	-0.0192	0.4394	0.626872	Both
Water_content	norb_Cluster88	0.0016	-0.0004	0.0147	-0.0371	0.6003	0.7312193	Both
Water_content	norb_Cluster90	0.0009	-0.0008	0.0559	0.0063	0.302	0.5149288	Both
Water_content	norb_Cluster91	0.6481	-0.2283	0.1478	0.1029	0.0853	0.2723188	Both
Water_content	norb_Cluster92	0.0015	-0.0003	0.0041	-0.0483	0.7816	0.8587231	Both
Water_content	norb_Cluster93	0.0022	-0.0008	0.0237	-0.0276	0.5049	0.6679035	Both
Water_content	norb_Cluster94	0.0017	-0.0006	0.0363	-0.0144	0.4081	0.5969009	Both
Water_content	norb_Cluster96	0.0007	0.0001	0.007	-0.0452	0.7179	0.8208304	Both
Water_content	norb_Cluster97	0.0119	-0.005	0.098	0.0505	0.1671	0.3837043	Both
Water_content	norb_Cluster98	0.0132	-0.0052	0.0717	0.0228	0.2407	0.4621257	Both
Water_content	nosz_Cluster0	0.0045	-0.0014	0.0599	0.0105	0.2848	0.4972929	Both
Water_content	nosz_Cluster10	0.0136	0.0047	0.0745	0.0258	0.2314	0.4565464	Both
Water_content	nosz_Cluster102	0.0044	-0.0022	0.2324	0.192	0.0269	0.1609128	Both
Water_content	nosz_Cluster103	0.0455	-0.0129	0.1459	0.1009	0.0875	0.2723188	Both
Water_content	nosz_Cluster105	0.0016	-0.0004	0.0211	-0.0304	0.5298	0.6810656	Both
Water_content	nosz_Cluster106	0.0073	-0.004	0.1931	0.1507	0.0462	0.2163989	Both
Water_content	nosz_Cluster107	0.0002	-0.0001	0.0145	-0.0374	0.6036	0.7312193	Both
Water_content	nosz_Cluster108	0.004	0.0002	0.0012	-0.0514	0.8833	0.9142952	Both
Water_content	nosz_Cluster109	0.0375	-0.0107	0.2203	0.1793	0.0318	0.1764033	Both
Water_content	nosz_Cluster11	0.0129	0.0053	0.0456	-0.0047	0.3529	0.5602953	Both
Water_content	nosz_Cluster110	0.0003	0.0004	0.0973	0.0498	0.1687	0.3840619	Both
Water_content	nosz_Cluster111	0.0061	-0.0029	0.1616	0.1175	0.0708	0.2533016	Both
Water_content	nosz_Cluster116	0.0067	0.0003	0.0016	-0.0509	0.8614	0.902735	Both
Water_content	nosz_Cluster118	0.0045	-0.0019	0.0606	0.0111	0.2822	0.4972929	Both
Water_content	nosz_Cluster119	0.0013	-0.0012	0.1711	0.1274	0.0624	0.2450608	Both
Water_content	nosz_Cluster120	0.0071	-0.0023	0.0819	0.0336	0.2086	0.4363468	Both
Water_content	nosz_Cluster123	0.0097	-0.0068	0.2534	0.2141	0.02	0.1404559	Both
Water_content	nosz_Cluster128	0.0047	0.0003	0.0014	-0.0512	0.8734	0.910053	Both
Water_content	nosz_Cluster129	0.0494	0.0001	0.00E+00	-0.0526	0.9833	0.9896808	Both
Water_content	nosz_Cluster13	0.0115	-0.002	0.0224	-0.029	0.5173	0.6736957	Both
Water_content	nosz_Cluster130	0.089	-0.035	0.0327	-0.0182	0.4327	0.6211441	Both
Water_content	nosz_Cluster132	0.0037	-0.0013	0.1117	0.065	0.1386	0.3464724	Both

Water_content	nosz_Cluster14	0.024	0.0178	0.0885	0.0406	0.1902	0.4075072	Both
Water_content	nosz_Cluster16	0.0076	0.002	0.0259	-0.0254	0.4858	0.6610748	Both
Water_content	nosz_Cluster17	0.0364	-0.0119	0.1457	0.1008	0.0877	0.2723188	Both
Water_content	nosz_Cluster18	0.0161	-0.0014	0.0038	-0.0487	0.7918	0.8619396	Both
Water_content	nosz_Cluster20	0.0091	-0.0032	0.0552	0.0055	0.3051	0.5187631	Both
Water_content	nosz_Cluster21	0.0083	-0.0053	0.1591	0.1148	0.0733	0.2546797	Both
Water_content	nosz_Cluster22	0.0267	-0.0039	0.0092	-0.043	0.6799	0.7942343	Both
Water_content	nosz_Cluster23	0.0141	-0.0076	0.1537	0.1091	0.0788	0.2613073	Both
Water_content	nosz_Cluster24	0.0013	-0.0005	0.0289	-0.0222	0.4613	0.6464595	Both
Water_content	nosz_Cluster25	0.0029	0.001	0.0269	-0.0243	0.4773	0.6602002	Both
Water_content	nosz_Cluster26	0.0054	-0.0017	0.0408	-0.0097	0.3798	0.5775699	Both
Water_content	nosz_Cluster27	0.0053	-0.0022	0.0951	0.0475	0.1737	0.3896676	Both
Water_content	nosz_Cluster28	0.0303	-0.01	0.1051	0.058	0.1516	0.3660809	Both
Water_content	nosz_Cluster30	0.0276	-0.0058	0.0411	-0.0093	0.378	0.5775699	Both
Water_content	nosz_Cluster32	0.0137	-0.0014	0.0087	-0.0435	0.6874	0.7952112	Both
Water_content	nosz_Cluster33	0.018	0.0019	0.005	-0.0474	0.7613	0.8462698	Both
Water_content	nosz_Cluster34	0.0377	0.0005	1.00E-04	-0.0526	0.9701	0.9848331	Both
Water_content	nosz_Cluster35	0.0075	-0.0022	0.0514	0.0015	0.323	0.5314216	Both
Water_content	nosz_Cluster36	0.0179	-0.0035	0.0275	-0.0237	0.4723	0.6571933	Both
Water_content	nosz_Cluster37	0.006	-0.0017	0.0432	-0.0072	0.3662	0.5685118	Both
Water_content	nosz_Cluster38	0.0128	-0.0017	0.0087	-0.0434	0.6868	0.7952112	Both
Water_content	nosz_Cluster39	0.0537	-0.0149	0.0796	0.0312	0.2153	0.4400816	Both
Water_content	nosz_Cluster40	0.0218	-0.0021	0.0067	-0.0455	0.7234	0.8239401	Both
Water_content	nosz_Cluster41	0.0203	-0.007	0.0801	0.0317	0.2137	0.4398974	Both
Water_content	nosz_Cluster42	0.0279	-0.0017	0.0026	-0.0499	0.8267	0.888454	Both
Water_content	nosz_Cluster43	0.0007	-0.0005	0.0414	-0.009	0.3763	0.5775699	Both
Water_content	nosz_Cluster44	0.0057	-0.0034	0.2048	0.1629	0.0394	0.1937691	Both
Water_content	nosz_Cluster45	0.0045	-0.0009	0.0226	-0.0288	0.5151	0.6723971	Both
Water_content	nosz_Cluster46	0.007	-0.0011	0.0236	-0.0278	0.5062	0.6681844	Both
Water_content	nosz_Cluster47	0.0008	-0.0004	0.0437	-0.0066	0.3632	0.5653887	Both
Water_content	nosz_Cluster48	0.0023	-0.0009	0.0409	-0.0096	0.3792	0.5775699	Both
Water_content	nosz_Cluster49	0.0014	0.0011	0.1595	0.1153	0.0729	0.2546797	Both
Water_content	nosz_Cluster5	0.0045	0.0017	0.0357	-0.015	0.4118	0.5969009	Both
Water_content	nosz_Cluster51	0.0021	-0.0007	0.0316	-0.0194	0.4409	0.626872	Both
Water_content	nosz_Cluster53	0.0009	-0.0005	0.0325	-0.0185	0.4344	0.6221144	Both
Water_content	nosz_Cluster54	0.0026	-0.0011	0.0474	-0.0028	0.3433	0.553359	Both
Water_content	nosz_Cluster57	0.1757	-0.0569	0.1006	0.0532	0.1613	0.3776052	Both
Water_content	nosz_Cluster58	0.0043	-0.0027	0.1467	0.1018	0.0866	0.2723188	Both
Water_content	nosz_Cluster59	0.0009	0.00E+00	0.00E+00	-0.0526	0.9851	0.9896808	Both
Water_content	nosz_Cluster6	0.0032	0.0002	0.0013	-0.0512	0.8746	0.910053	Both
Water_content	nosz_Cluster60	0.0079	-0.0016	0.0229	-0.0285	0.5122	0.6711959	Both
Water_content	nosz_Cluster61	0.0178	-0.0056	0.2142	0.1728	0.0346	0.1793029	Both
Water_content	nosz_Cluster62	0.0413	-0.0101	0.0574	0.0077	0.2957	0.5071956	Both
Water_content	nosz_Cluster64	0.0016	-0.0006	0.013	-0.0389	0.6222	0.7476865	Both
Water_content	nosz_Cluster65	0.0065	-0.0011	0.0158	-0.036	0.587	0.7245296	Both
Water_content	nosz_Cluster66	0.0324	-0.0137	0.1129	0.0663	0.1364	0.3423586	Both
Water_content	nosz_Cluster67	0.0521	-0.0156	0.0619	0.0125	0.277	0.4972929	Both
Water_content	nosz_Cluster69	0.0061	-0.0027	0.1199	0.0736	0.1241	0.3312124	Both
Water_content	nosz_Cluster70	0.0479	-0.018	0.0374	-0.0133	0.4009	0.5899138	Both
Water_content	nosz_Cluster71	0.0083	-0.0008	0.0039	-0.0485	0.7873	0.8604005	Both
Water_content	nosz_Cluster72	0.0506	-0.0174	0.0941	0.0464	0.1762	0.3938305	Both
Water_content	nosz_Cluster73	0.003	-0.0004	0.0069	-0.0454	0.7208	0.8225244	Both
Water_content	nosz_Cluster74	0.0017	-0.0009	0.081	0.0326	0.2111	0.438544	Both
Water_content	nosz_Cluster75	0.0967	-0.0472	0.0358	-0.0149	0.4111	0.5969009	Both
Water_content	nosz_Cluster77	0.0002	-0.0001	0.0254	-0.0259	0.49	0.6610748	Both

Water_content	nosz_Cluster78	0.0044	-0.0019	0.1152	0.0686	0.1323	0.3351103	Both
Water_content	nosz_Cluster79	0.0333	-0.0072	0.059	0.0095	0.2888	0.4996695	Both
Water_content	nosz_Cluster8	0.0099	-0.001	0.0108	-0.0413	0.6542	0.7750062	Both
Water_content	nosz_Cluster80	0.0045	0.0016	0.0369	-0.0138	0.4042	0.5932188	Both
Water_content	nosz_Cluster81	0.0079	-0.0021	0.0204	-0.0311	0.5367	0.6868316	Both
Water_content	nosz_Cluster82	0.0016	-0.0006	0.0099	-0.0422	0.6681	0.7868322	Both
Water_content	nosz_Cluster83	0.0015	-0.0007	0.0351	-0.0157	0.4164	0.6021528	Both
Water_content	nosz_Cluster85	0.0022	-0.0002	0.0024	-0.0501	0.833	0.8935774	Both
Water_content	nosz_Cluster86	0.0027	-0.001	0.0428	-0.0075	0.368	0.5698867	Both
Water_content	nosz_Cluster87	0.003	-0.0005	0.0149	-0.0369	0.598	0.7312193	Both
Water_content	nosz_Cluster88	0.0218	-0.0069	0.2179	0.1767	0.0329	0.1781842	Both
Water_content	nosz_Cluster89	0.0466	-0.0181	0.117	0.0706	0.129	0.3318614	Both
Water_content	nosz_Cluster9	0.003	-0.0002	0.0014	-0.0512	0.872	0.910053	Both
Water_content	nosz_Cluster90	0.0116	-0.0036	0.1392	0.0939	0.0957	0.2809867	Both
Water_content	nosz_Cluster91	0.0189	-0.0017	0.0051	-0.0472	0.7578	0.8462698	Both
Water_content	nosz_Cluster93	0.0393	-0.0182	0.2199	0.1789	0.032	0.1764033	Both
Water_content	nosz_Cluster95	0.007	-0.0022	0.0722	0.0234	0.2387	0.4613267	Both
Water_content	nosz_Cluster96	0.0066	0.0011	0.0316	-0.0193	0.4404	0.626872	Both
Water_content	nosz_Cluster97	0.0019	-0.0009	0.0675	0.0185	0.2552	0.4720807	Both
Water_content	nosz_Cluster98	0.0025	-0.0008	0.0077	-0.0446	0.7057	0.8116165	Both
Water_content	nosz_Cluster99	0.0055	0.0002	0.0018	-0.0507	0.8535	0.902735	Both
Water_content	nrfa_Cluster10	0.0804	-0.0228	0.0514	0.0014	0.3231	0.5314216	Both
Water_content	nrfa_Cluster100	0.0963	-0.0611	0.1864	0.1436	0.0506	0.2257305	Both
Water_content	nrfa_Cluster104	0.1143	-0.0647	0.3335	0.2985	0.0061	0.0732226	Both
Water_content	nrfa_Cluster105	0.0062	0.0007	0.0038	-0.0487	0.7918	0.8619396	Both
Water_content	nrfa_Cluster109	0.2294	-0.0886	0.1872	0.1444	0.0501	0.2257305	Both
Water_content	nrfa_Cluster116	0.2592	-0.1307	0.4875	0.4606	0.0004	0.0110663	Both
Water_content	nrfa_Cluster118	0.173	-0.1206	0.1837	0.1407	0.0526	0.2257305	Both
Water_content	nrfa_Cluster125	0.0809	-0.0557	0.1594	0.1152	0.073	0.2546797	Both
Water_content	nrfa_Cluster154	0.0658	-0.0366	0.1562	0.1118	0.0762	0.2584663	Both
Water_content	nrfa_Cluster164	0.0045	0.0006	0.0056	-0.0467	0.7463	0.8415161	Both
Water_content	nrfa_Cluster172	0.0026	-0.0014	0.0708	0.0219	0.2436	0.4621646	Both
Water_content	nrfa_Cluster175	0.0044	-0.0022	0.0617	0.0123	0.2778	0.4972929	Both
Water_content	nrfa_Cluster20	0.035	-0.0187	0.1763	0.1329	0.0581	0.2395292	Both
Water_content	nrfa_Cluster26	0.1078	-0.073	0.1914	0.1489	0.0473	0.2180344	Both
Water_content	nrfa_Cluster29	0.0048	-0.0008	0.0116	-0.0404	0.6419	0.7650635	Both
Water_content	nrfa_Cluster34	0.0487	-0.0292	0.2159	0.1746	0.0338	0.1781842	Both
Water_content	nrfa_Cluster38	0.0031	-0.0013	0.0505	0.0006	0.3273	0.5333791	Both
Water_content	nrfa_Cluster61	0.0036	-0.0025	0.1639	0.1199	0.0687	0.2517324	Both
Water_content	nrfa_Cluster62	0.0452	-0.031	0.1641	0.1201	0.0685	0.2517324	Both
Water_content	nrfa_Cluster66	0.1082	-0.0453	0.261	0.2221	0.0179	0.1366219	Both
Water_content	nrfa_Cluster69	0.003	-0.0002	0.0017	-0.0509	0.8606	0.902735	Both
Water_content	nrfa_Cluster71	0.027	-0.0105	0.0691	0.0201	0.2496	0.464486	Both
Water_content	nrfa_Cluster72	0.0732	-0.0461	0.2171	0.1759	0.0333	0.1781842	Both
Water_content	nrfa_Cluster73	0.0038	-0.0007	0.0084	-0.0438	0.6923	0.799286	Both
Water_content	nrfa_Cluster74	0.1331	-0.0735	0.1761	0.1327	0.0583	0.2395292	Both
Water_content	nrfa_Cluster76	0.0755	-0.0341	0.101	0.0537	0.1602	0.3766694	Both
Water_content	nrfa_Cluster79	0.0229	0.0166	0.0697	0.0208	0.2474	0.464486	Both
Water_content	nrfa_Cluster87	0.017	-0.0036	0.0131	-0.0389	0.6217	0.7476865	Both
Water_content	nrfa_Cluster89	0.0018	-0.001	0.0643	0.015	0.2675	0.490199	Both
Water_content	nrfa_Cluster92	0.0043	0.0005	0.0055	-0.0469	0.7502	0.8415161	Both
Water_content	nrfa_Cluster93	0.0428	-0.0123	0.0241	-0.0272	0.5014	0.6662889	Both
Water_content	nrfa_Cluster94	0.0002	-0.0002	0.0535	0.0037	0.313	0.5251559	Both
Water_content	nrfa_Cluster97	0.0168	-0.0098	0.2391	0.1991	0.0245	0.1552976	Both
Water_content	nrfa_Cluster99	0.014	-0.0075	0.1701	0.1265	0.0631	0.2450608	Both

Bulk_density	amoA_A_Cluster26	0.005	-0.0028	0.1134	0.0543	0.1862	0.4042816	Both
Bulk_density	amoA_A_Cluster45	0.0058	-0.0043	0.2192	0.1672	0.058	0.2760172	Both
Bulk_density	amoA_B_Cluster0	0.1114	0.0004	0.00E+00	-0.0666	0.9792	0.9862087	Both
Bulk_density	amoA_B_Cluster1	0.0117	-0.0003	0.0005	-0.0661	0.9297	0.9507227	Both
Bulk_density	amoA_B_Cluster10	0.0389	-0.0004	0.0002	-0.0665	0.9587	0.973608	Both
Bulk_density	amoA_B_Cluster11	0.0278	-0.0006	0.0007	-0.0659	0.9205	0.9461712	Both
Bulk_density	amoA_B_Cluster12	0.0775	0.006	0.0194	-0.046	0.5942	0.7230533	Both
Bulk_density	amoA_B_Cluster13	0.515	0.1898	0.1825	0.128	0.0872	0.3147075	Both
Bulk_density	amoA_B_Cluster14	0.0289	0.0145	0.2141	0.1617	0.0614	0.2798971	Both
Bulk_density	amoA_B_Cluster15	0.0258	0.0063	0.0692	0.0071	0.3078	0.4933885	Both
Bulk_density	amoA_B_Cluster2	0.1576	0.0573	0.1602	0.1042	0.1114	0.3504205	Both
Bulk_density	amoA_B_Cluster20	0.0039	-0.0024	0.2031	0.15	0.0694	0.2852687	Both
Bulk_density	amoA_B_Cluster23	0.0402	0.0018	0.0048	-0.0616	0.7922	0.8721954	Both
Bulk_density	amoA_B_Cluster3	0.0114	0.0051	0.1485	0.0918	0.1266	0.3621716	Both
Bulk_density	amoA_B_Cluster4	0.0102	0.0048	0.1143	0.0553	0.1843	0.4042816	Both
Bulk_density	amoA_B_Cluster5	0.0907	0.0045	0.0076	-0.0586	0.7393	0.8326069	Both
Bulk_density	amoA_B_Cluster6	0.0194	0.0013	0.0093	-0.0567	0.7127	0.8135383	Both
Bulk_density	amoA_B_Cluster7	0.0123	-0.0064	0.1761	0.1212	0.0935	0.3244257	Both
Bulk_density	amoA_B_Cluster8	0.0117	0.0003	0.0015	-0.0651	0.8834	0.9308514	Both
Bulk_density	amoA_B_Cluster9	0.04	0.0133	0.1127	0.0536	0.1876	0.4042816	Both
Bulk_density	nifh_Cluster0	0.0325	-0.0053	0.0991	0.0391	0.2184	0.4230744	Both
Bulk_density	nifh_Cluster10	0.0057	-0.0023	0.0431	-0.0207	0.4241	0.6002706	Both
Bulk_density	nifh_Cluster100	0.0024	-0.0014	0.116	0.057	0.1811	0.4042816	Both
Bulk_density	nifh_Cluster103	0.0353	0.0064	0.065	0.0026	0.3234	0.5099602	Both
Bulk_density	nifh_Cluster1034	0.0286	-0.0002	0.00E+00	-0.0666	0.9795	0.9862087	Both
Bulk_density	nifh_Cluster105	0.0155	-0.0033	0.0404	-0.0236	0.4393	0.6124636	Both
Bulk_density	nifh_Cluster108	0.0093	-0.0009	0.009	-0.0571	0.7173	0.8156488	Both
Bulk_density	nifh_Cluster1099	0.067	-0.0068	0.0132	-0.0525	0.66	0.7713483	Both
Bulk_density	nifh_Cluster11	0.0025	-0.002	0.36	0.3173	0.0109	0.2693572	Both
Bulk_density	nifh_Cluster1112	0.0022	0.0006	0.0266	-0.0383	0.5317	0.6848541	Both
Bulk_density	nifh_Cluster112	0.0123	0.0024	0.0341	-0.0303	0.4778	0.6411124	Both
Bulk_density	nifh_Cluster113	0.0329	-0.0157	0.5066	0.4737	0.0014	0.2693572	Both
Bulk_density	nifh_Cluster114	0.0343	0.0012	0.007	-0.0592	0.7497	0.8378966	Both
Bulk_density	nifh_Cluster1141	0.0262	0.0005	0.0004	-0.0662	0.9375	0.9561963	Both
Bulk_density	nifh_Cluster115	0.0093	-0.0016	0.053	-0.0101	0.374	0.5493803	Both
Bulk_density	nifh_Cluster116	0.0626	-0.0069	0.0336	-0.0308	0.4812	0.6426656	Both
Bulk_density	nifh_Cluster1163	0.0036	-0.0001	0.0009	-0.0657	0.9089	0.9454083	Both
Bulk_density	nifh_Cluster1179	0.0038	-0.0003	0.0035	-0.0629	0.8211	0.8920581	Both
Bulk_density	nifh_Cluster1182	0.0199	0.0004	0.0026	-0.0639	0.8465	0.9081499	Both
Bulk_density	nifh_Cluster1183	0.0067	0.0004	0.0037	-0.0627	0.8167	0.8908444	Both
Bulk_density	nifh_Cluster1197	0.0109	-0.0018	0.0407	-0.0232	0.4373	0.6124636	Both
Bulk_density	nifh_Cluster1199	0.0041	0.0002	0.0022	-0.0644	0.8596	0.9174394	Both
Bulk_density	nifh_Cluster120	0.0183	-0.0126	0.169	0.1136	0.1011	0.336449	Both
Bulk_density	nifh_Cluster1203	0.011	-0.0037	0.0414	-0.0226	0.4337	0.6097043	Both
Bulk_density	nifh_Cluster1206	0.0024	-0.0006	0.0237	-0.0414	0.5554	0.6937193	Both
Bulk_density	nifh_Cluster1207	0.0081	0.0004	0.0016	-0.065	0.879	0.9308514	Both
Bulk_density	nifh_Cluster1209	0.1305	0.0044	0.0142	-0.0515	0.6489	0.7662355	Both
Bulk_density	nifh_Cluster121	0.0118	-0.0082	0.1974	0.1439	0.074	0.2896155	Both
Bulk_density	nifh_Cluster1211	0.0034	-0.0022	0.3005	0.2538	0.0227	0.2693572	Both
Bulk_density	nifh_Cluster1213	0.0025	-0.0018	0.2611	0.2119	0.036	0.2693572	Both
Bulk_density	nifh_Cluster122	0.0036	-0.0027	0.2394	0.1887	0.0462	0.2693572	Both
Bulk_density	nifh_Cluster123	0.0118	-0.0094	0.2685	0.2198	0.0331	0.2693572	Both
Bulk_density	nifh_Cluster1236	0.0249	-0.0132	0.1065	0.047	0.201	0.4169345	Both
Bulk_density	nifh_Cluster1238	0.0034	-0.0014	0.0799	0.0186	0.2715	0.4629597	Both
Bulk_density	nifh_Cluster124	0.0088	-0.0043	0.2994	0.2527	0.023	0.2693572	Both

Bulk_density	nifh_Cluster1261	0.002	-0.0004	0.009	-0.0571	0.7172	0.8156488	Both
Bulk_density	nifh_Cluster1262	0.0078	0.0032	0.1226	0.0641	0.1682	0.3995418	Both
Bulk_density	nifh_Cluster1266	0.0116	-0.0043	0.1874	0.1332	0.0826	0.3061064	Both
Bulk_density	nifh_Cluster1267	0.0603	0.0105	0.0303	-0.0344	0.5041	0.6627532	Both
Bulk_density	nifh_Cluster127	0.0032	-0.0018	0.1075	0.048	0.1988	0.4151813	Both
Bulk_density	nifh_Cluster1278	0.1379	-0.0457	0.121	0.0624	0.1713	0.4036396	Both
Bulk_density	nifh_Cluster128	0.0044	-0.0029	0.3036	0.2572	0.0219	0.2693572	Both
Bulk_density	nifh_Cluster1289	0.0042	0.0006	0.0142	-0.0516	0.6492	0.7662355	Both
Bulk_density	nifh_Cluster129	0.0096	-0.0078	0.3412	0.2972	0.0138	0.2693572	Both
Bulk_density	nifh_Cluster1292	0.0077	-0.0046	0.2267	0.1752	0.0533	0.2760172	Both
Bulk_density	nifh_Cluster130	0.0073	-0.0049	0.1442	0.0871	0.1328	0.3621716	Both
Bulk_density	nifh_Cluster1305	0.0048	-0.003	0.3842	0.3431	0.008	0.2693572	Both
Bulk_density	nifh_Cluster1306	0.0047	0.00E+00	0.00E+00	-0.0667	0.9892	0.9909185	Both
Bulk_density	nifh_Cluster131	0.0063	-0.0048	0.2549	0.2052	0.0387	0.2693572	Both
Bulk_density	nifh_Cluster1319	0.0037	0.001	0.0264	-0.0385	0.5333	0.6848541	Both
Bulk_density	nifh_Cluster132	0.0079	-0.0055	0.3053	0.259	0.0214	0.2693572	Both
Bulk_density	nifh_Cluster1320	0.002	-0.001	0.0906	0.03	0.2404	0.43146	Both
Bulk_density	nifh_Cluster1323	0.0017	-0.0002	0.0043	-0.062	0.8019	0.8795022	Both
Bulk_density	nifh_Cluster1324	0.0356	-0.0065	0.0563	-0.0066	0.3592	0.5411534	Both
Bulk_density	nifh_Cluster1336	0.0143	-0.0033	0.2998	0.2532	0.0229	0.2693572	Both
Bulk_density	nifh_Cluster1340	0.0138	0.0064	0.2076	0.1548	0.066	0.2814384	Both
Bulk_density	nifh_Cluster1345	0.0503	0.0039	0.0058	-0.0605	0.7721	0.8548796	Both
Bulk_density	nifh_Cluster1370	0.0024	-0.0005	0.0158	-0.0498	0.6307	0.7581792	Both
Bulk_density	nifh_Cluster1375	0.0017	0.0007	0.0618	-0.0007	0.336	0.5193196	Both
Bulk_density	nifh_Cluster139	0.0033	-0.0027	0.2755	0.2272	0.0305	0.2693572	Both
Bulk_density	nifh_Cluster140	0.0043	-0.0013	0.061	-0.0016	0.3392	0.5216661	Both
Bulk_density	nifh_Cluster141	0.0651	0.0044	0.0083	-0.0578	0.7281	0.8250603	Both
Bulk_density	nifh_Cluster148	0.0027	0.0022	0.1721	0.117	0.0977	0.3307876	Both
Bulk_density	nifh_Cluster1480	0.001	0.0003	0.0156	-0.05	0.6325	0.7587467	Both
Bulk_density	nifh_Cluster152	0.0668	-0.0071	0.0456	-0.0181	0.4107	0.5871025	Both
Bulk_density	nifh_Cluster156	0.0756	0.0023	0.0021	-0.0644	0.8614	0.9174394	Both
Bulk_density	nifh_Cluster1562	0.061	0.0054	0.0061	-0.0602	0.7667	0.8523979	Both
Bulk_density	nifh_Cluster158	0.0124	-0.002	0.0146	-0.0511	0.644	0.7656569	Both
Bulk_density	nifh_Cluster16	0.0024	-0.0014	0.1034	0.0436	0.2082	0.4170625	Both
Bulk_density	nifh_Cluster166	0.0213	-0.0176	0.2822	0.2344	0.0282	0.2693572	Both
Bulk_density	nifh_Cluster205	0.0126	-0.0045	0.1372	0.0797	0.1433	0.3677743	Both
Bulk_density	nifh_Cluster21	0.0021	0.0008	0.0434	-0.0203	0.4221	0.5996212	Both
Bulk_density	nifh_Cluster222	0.0075	-0.0058	0.2393	0.1886	0.0463	0.2693572	Both
Bulk_density	nifh_Cluster225	0.0179	-0.0025	0.0485	-0.015	0.3959	0.5728766	Both
Bulk_density	nifh_Cluster230	0.0047	-0.0021	0.1598	0.1038	0.1118	0.3504205	Both
Bulk_density	nifh_Cluster231	0.0022	-0.0019	0.2551	0.2054	0.0387	0.2693572	Both
Bulk_density	nifh_Cluster236	0.0028	-0.0004	0.0102	-0.0558	0.7004	0.8055532	Both
Bulk_density	nifh_Cluster237	0.0069	-0.0021	0.0984	0.0382	0.2203	0.4236938	Both
Bulk_density	nifh_Cluster243	0.0386	-0.002	0.0035	-0.063	0.8224	0.8920581	Both
Bulk_density	nifh_Cluster2432	0.0452	-0.0012	0.0008	-0.0658	0.9154	0.9454083	Both
Bulk_density	nifh_Cluster2433	0.0027	-0.0019	0.1533	0.0969	0.1201	0.3541145	Both
Bulk_density	nifh_Cluster246	0.0065	-0.0034	0.1498	0.0931	0.1249	0.3616366	Both
Bulk_density	nifh_Cluster25	0.0333	-0.008	0.1177	0.0589	0.1776	0.4042816	Both
Bulk_density	nifh_Cluster256	0.0031	-0.0008	0.0357	-0.0285	0.4674	0.6314423	Both
Bulk_density	nifh_Cluster264	0.0036	-0.0025	0.4379	0.4005	0.0038	0.2693572	Both
Bulk_density	nifh_Cluster265	0.0751	-0.0111	0.0793	0.0179	0.2735	0.4629597	Both
Bulk_density	nifh_Cluster267	0.0114	0.00E+00	0.00E+00	-0.0667	0.9949	0.9949125	Both
Bulk_density	nifh_Cluster268	0.0118	-0.0025	0.0793	0.0179	0.2735	0.4629597	Both
Bulk_density	nifh_Cluster269	0.0215	-0.0076	0.0699	0.0079	0.305	0.4933885	Both
Bulk_density	nifh_Cluster272	0.0048	-0.0015	0.0373	-0.0269	0.4579	0.6214753	Both

Bulk_density	nifh_Cluster274	0.0039	-0.0037	0.1028	0.043	0.2095	0.4175485	Both
Bulk_density	nifh_Cluster275	0.0035	0.0001	0.0008	-0.0659	0.9161	0.9454083	Both
Bulk_density	nifh_Cluster278	0.0081	-0.0006	0.0036	-0.0629	0.82	0.8920581	Both
Bulk_density	nifh_Cluster279	0.0063	-0.0014	0.0607	-0.0019	0.3405	0.5217902	Both
Bulk_density	nifh_Cluster28	0.002	-0.001	0.1404	0.0831	0.1384	0.3621716	Both
Bulk_density	nifh_Cluster280	0.0017	0.0015	0.2508	0.2009	0.0406	0.2693572	Both
Bulk_density	nifh_Cluster282	0.0051	-0.0021	0.1323	0.0745	0.1512	0.3757667	Both
Bulk_density	nifh_Cluster287	0.0022	1.00E-04	0.0007	-0.0659	0.9184	0.945688	Both
Bulk_density	nifh_Cluster290	0.0125	-0.0038	0.1148	0.0558	0.1835	0.4042816	Both
Bulk_density	nifh_Cluster297	0.0035	-0.0026	0.1155	0.0565	0.182	0.4042816	Both
Bulk_density	nifh_Cluster299	0.0032	-0.0017	0.1156	0.0566	0.1818	0.4042816	Both
Bulk_density	nifh_Cluster303	0.0025	-0.0013	0.1083	0.0489	0.197	0.4144751	Both
Bulk_density	nifh_Cluster304	0.0029	-0.0023	0.3782	0.3367	0.0086	0.2693572	Both
Bulk_density	nifh_Cluster313	0.0127	-0.01	0.2805	0.2325	0.0288	0.2693572	Both
Bulk_density	nifh_Cluster314	0.0027	-0.0019	0.2397	0.189	0.0461	0.2693572	Both
Bulk_density	nifh_Cluster317	0.0081	-0.0017	0.0387	-0.0254	0.4492	0.6153271	Both
Bulk_density	nifh_Cluster32	0.0041	-0.0004	0.0104	-0.0556	0.6971	0.8035466	Both
Bulk_density	nifh_Cluster320	12.9915	-1.1042	0.1043	0.0446	0.2061	0.4170625	Both
Bulk_density	nifh_Cluster334	0.0077	-0.006	0.2014	0.1482	0.0708	0.2852687	Both
Bulk_density	nifh_Cluster335	0.0092	-0.0073	0.164	0.1083	0.1068	0.3494845	Both
Bulk_density	nifh_Cluster338	0.0103	0.0014	0.0176	-0.0478	0.6112	0.7377516	Both
Bulk_density	nifh_Cluster340	0.0075	-0.0066	0.1037	0.044	0.2074	0.4170625	Both
Bulk_density	nifh_Cluster341	0.0029	-0.0007	0.0263	-0.0386	0.5337	0.6848541	Both
Bulk_density	nifh_Cluster35	0.006	0.0009	0.0211	-0.0442	0.5784	0.7159401	Both
Bulk_density	nifh_Cluster351	0.0031	-0.0024	0.1982	0.1447	0.0733	0.2896155	Both
Bulk_density	nifh_Cluster3574	0.1107	0.0493	0.5402	0.5095	0.0008	0.2693572	Both
Bulk_density	nifh_Cluster3691	0.01	-0.0015	0.0197	-0.0457	0.5915	0.7230533	Both
Bulk_density	nifh_Cluster37	0.0368	-0.0198	0.2471	0.1969	0.0423	0.2693572	Both
Bulk_density	nifh_Cluster374	0.0003	0.0004	0.105	0.0454	0.2044	0.4170625	Both
Bulk_density	nifh_Cluster38	0.0011	-0.0007	0.0951	0.0348	0.2284	0.4264211	Both
Bulk_density	nifh_Cluster382	0.002	-0.0014	0.0822	0.021	0.2645	0.4582307	Both
Bulk_density	nifh_Cluster384	0.0024	-0.0019	0.2907	0.2434	0.0255	0.2693572	Both
Bulk_density	nifh_Cluster386	0.0043	-0.0034	0.2315	0.1802	0.0506	0.2756752	Both
Bulk_density	nifh_Cluster389	0.0038	-0.0032	0.278	0.2299	0.0296	0.2693572	Both
Bulk_density	nifh_Cluster394	0.0024	-0.0018	0.1072	0.0477	0.1996	0.4154311	Both
Bulk_density	nifh_Cluster40	0.0015	-0.0002	0.006	-0.0603	0.7685	0.8523979	Both
Bulk_density	nifh_Cluster43	0.0114	-0.0019	0.0503	-0.013	0.3868	0.5660077	Both
Bulk_density	nifh_Cluster434	0.0027	-0.0014	0.0797	0.0184	0.2722	0.4629597	Both
Bulk_density	nifh_Cluster440	0.0103	-0.0018	0.0134	-0.0524	0.6582	0.770719	Both
Bulk_density	nifh_Cluster462	0.0035	-0.0024	0.1455	0.0886	0.1308	0.3621716	Both
Bulk_density	nifh_Cluster470	0.0115	-0.0028	0.1316	0.0737	0.1525	0.3773225	Both
Bulk_density	nifh_Cluster49	0.0261	-0.0207	0.3327	0.2882	0.0153	0.2693572	Both
Bulk_density	nifh_Cluster499	0.0032	-0.0019	0.2126	0.1601	0.0625	0.2804082	Both
Bulk_density	nifh_Cluster5	0.0226	0.0043	0.1421	0.0849	0.1358	0.3621716	Both
Bulk_density	nifh_Cluster506	0.0039	-0.0004	0.008	-0.0581	0.7324	0.8280338	Both
Bulk_density	nifh_Cluster51	0.0034	-0.0019	0.1116	0.0523	0.1901	0.4042816	Both
Bulk_density	nifh_Cluster52	0.0415	-0.0259	0.3261	0.2812	0.0166	0.2693572	Both
Bulk_density	nifh_Cluster525	0.0041	0.0017	0.0363	-0.0279	0.4637	0.6278293	Both
Bulk_density	nifh_Cluster531	0.0316	0.005	0.0327	-0.0318	0.4876	0.6483533	Both
Bulk_density	nifh_Cluster539	0.006	-0.0047	0.1551	0.0988	0.1178	0.3534988	Both
Bulk_density	nifh_Cluster578	0.0384	-0.011	0.1384	0.0809	0.1415	0.3671086	Both
Bulk_density	nifh_Cluster58	0.3791	0.0308	0.0071	-0.0591	0.7474	0.8371505	Both
Bulk_density	nifh_Cluster60	0.0104	-0.0068	0.326	0.2811	0.0167	0.2693572	Both
Bulk_density	nifh_Cluster61	0.0031	0.0006	0.0239	-0.0411	0.5533	0.6937193	Both
Bulk_density	nifh_Cluster65	0.0106	-0.0073	0.3222	0.277	0.0175	0.2693572	Both

Bulk_density	nifh_Cluster686	0.0011	-1.00E-04	0.0005	-0.0661	0.9294	0.9507227	Both
Bulk_density	nifh_Cluster69	0.0112	-0.007	0.2874	0.2399	0.0265	0.2693572	Both
Bulk_density	nifh_Cluster70	0.0137	-0.0102	0.2025	0.1494	0.0699	0.2852687	Both
Bulk_density	nifh_Cluster717	0.003	-1.00E-04	0.0008	-0.0658	0.9149	0.9454083	Both
Bulk_density	nifh_Cluster725	0.0067	-0.0012	0.0303	-0.0343	0.504	0.6627532	Both
Bulk_density	nifh_Cluster727	0.0034	0.0008	0.0201	-0.0452	0.5872	0.7219973	Both
Bulk_density	nifh_Cluster73	0.0129	-0.0046	0.1014	0.0415	0.2128	0.4175485	Both
Bulk_density	nifh_Cluster74	0.0061	-0.0042	0.2024	0.1492	0.07	0.2852687	Both
Bulk_density	nifh_Cluster748	0.0077	-0.0006	0.0094	-0.0566	0.7107	0.812844	Both
Bulk_density	nifh_Cluster749	0.0075	0.0003	0.0016	-0.065	0.8806	0.9308514	Both
Bulk_density	nifh_Cluster755	0.0061	0.0045	0.1991	0.1457	0.0726	0.2889718	Both
Bulk_density	nifh_Cluster76	0.0027	0.00E+00	0.00E+00	-0.0666	0.9819	0.9869467	Both
Bulk_density	nifh_Cluster79	0.0028	-0.0009	0.0643	0.002	0.3259	0.5099602	Both
Bulk_density	nifh_Cluster81	0.0068	-0.0013	0.0144	-0.0514	0.6469	0.7662355	Both
Bulk_density	nifh_Cluster82	0.0013	1.00E-04	0.0006	-0.066	0.9233	0.9474716	Both
Bulk_density	nifh_Cluster83	0.0058	-0.0032	0.1116	0.0524	0.1899	0.4042816	Both
Bulk_density	nifh_Cluster86	0.0088	0.0003	0.0013	-0.0653	0.8922	0.9368951	Both
Bulk_density	nifh_Cluster868	0.01	0.0013	0.021	-0.0442	0.5786	0.7159401	Both
Bulk_density	nifh_Cluster87	0.003	-0.0024	0.2493	0.1993	0.0413	0.2693572	Both
Bulk_density	nifh_Cluster88	0.0011	-0.0008	0.1586	0.1025	0.1133	0.3513073	Both
Bulk_density	nifh_Cluster9	0.0075	-0.0014	0.0194	-0.0459	0.5936	0.7230533	Both
Bulk_density	nifh_Cluster92	0.0063	-0.0048	0.2148	0.1625	0.0609	0.2798971	Both
Bulk_density	nifh_Cluster93	0.0038	-0.0026	0.2335	0.1824	0.0494	0.2745122	Both
Bulk_density	nifh_Cluster94	0.0058	-0.0044	0.2433	0.1928	0.0442	0.2693572	Both
Bulk_density	nifh_Cluster95	0.0141	-0.0052	0.0698	0.0078	0.3054	0.4933885	Both
Bulk_density	nifh_Cluster96	0.0091	0.001	0.0227	-0.0424	0.5635	0.7016526	Both
Bulk_density	nifh_Cluster97	0.0198	-0.0045	0.0402	-0.0238	0.4402	0.6124636	Both
Bulk_density	nifh_Cluster98	0.0045	-0.0003	0.003	-0.0634	0.8339	0.9022541	Both
Bulk_density	nifh_Cluster99	0.0041	-0.0032	0.299	0.2522	0.0231	0.2693572	Both
Bulk_density	nirk_Cluster0	5.9922	0.283	0.0294	-0.0353	0.5103	0.6684904	Both
Bulk_density	nirk_Cluster1	0.1803	0.0425	0.0796	0.0183	0.2725	0.4629597	Both
Bulk_density	nirk_Cluster10	0.4707	-0.0341	0.0477	-0.0158	0.3995	0.5767873	Both
Bulk_density	nirk_Cluster101	0.0032	-0.0019	0.1422	0.085	0.1357	0.3621716	Both
Bulk_density	nirk_Cluster102	0.0427	-0.015	0.1366	0.0791	0.1442	0.3677743	Both
Bulk_density	nirk_Cluster103	0.0743	0.0073	0.0139	-0.0519	0.6525	0.7671165	Both
Bulk_density	nirk_Cluster104	0.0018	0.0004	0.0197	-0.0457	0.5912	0.7230533	Both
Bulk_density	nirk_Cluster105	0.0962	-0.0217	0.0757	0.0141	0.285	0.4742178	Both
Bulk_density	nirk_Cluster106	0.0635	-0.0107	0.0275	-0.0373	0.5244	0.680733	Both
Bulk_density	nirk_Cluster107	0.0873	-0.0168	0.0462	-0.0174	0.4076	0.5841021	Both
Bulk_density	nirk_Cluster108	0.0143	-0.0087	0.3251	0.2802	0.0168	0.2693572	Both
Bulk_density	nirk_Cluster109	0.0105	-0.0043	0.1139	0.0548	0.1852	0.4042816	Both
Bulk_density	nirk_Cluster11	0.1378	-0.0124	0.108	0.0485	0.1978	0.4145846	Both
Bulk_density	nirk_Cluster12	0.1022	-0.0083	0.0314	-0.0332	0.4964	0.655523	Both
Bulk_density	nirk_Cluster13	0.0426	-0.0014	0.0075	-0.0587	0.7408	0.8326932	Both
Bulk_density	nirk_Cluster14	0.0856	0.0005	0.0001	-0.0665	0.9675	0.9790895	Both
Bulk_density	nirk_Cluster15	0.3548	-0.0516	0.0356	-0.0287	0.4685	0.6314541	Both
Bulk_density	nirk_Cluster16	0.5104	-0.0395	0.01	-0.056	0.703	0.8055622	Both
Bulk_density	nirk_Cluster17	0.0992	-0.0244	0.3704	0.3284	0.0095	0.2693572	Both
Bulk_density	nirk_Cluster18	0.1463	-0.0557	0.3347	0.2903	0.015	0.2693572	Both
Bulk_density	nirk_Cluster19	0.2903	-0.0184	0.047	-0.0165	0.4031	0.5791243	Both
Bulk_density	nirk_Cluster2	0.1952	0.0213	0.1035	0.0437	0.208	0.4170625	Both
Bulk_density	nirk_Cluster20	0.1625	-0.0482	0.2944	0.2474	0.0244	0.2693572	Both
Bulk_density	nirk_Cluster21	0.1486	-0.0209	0.1436	0.0865	0.1336	0.3621716	Both
Bulk_density	nirk_Cluster22	0.1311	-0.0075	0.0119	-0.0539	0.6762	0.7875627	Both
Bulk_density	nirk_Cluster23	0.0664	-0.0104	0.25	0.2	0.041	0.2693572	Both

Bulk_density	nirk_Cluster24	0.0575	0.001	0.0008	-0.0659	0.9165	0.9454083	Both
Bulk_density	nirk_Cluster25	0.4466	0.0249	0.0108	-0.0552	0.6918	0.8035466	Both
Bulk_density	nirk_Cluster26	0.1065	-0.0055	0.0179	-0.0476	0.6088	0.7377516	Both
Bulk_density	nirk_Cluster27	0.1621	-0.0304	0.096	0.0357	0.2263	0.4244829	Both
Bulk_density	nirk_Cluster28	0.0629	-0.0178	0.2316	0.1804	0.0505	0.2756752	Both
Bulk_density	nirk_Cluster29	0.1316	-0.0105	0.0695	0.0074	0.3068	0.4933885	Both
Bulk_density	nirk_Cluster3	0.2793	0.0195	0.026	-0.0389	0.5364	0.6868571	Both
Bulk_density	nirk_Cluster30	0.0571	-0.0086	0.0316	-0.0329	0.4946	0.6546561	Both
Bulk_density	nirk_Cluster31	0.1527	0.0053	0.0152	-0.0505	0.6375	0.7616377	Both
Bulk_density	nirk_Cluster32	0.1402	-0.0383	0.1099	0.0505	0.1937	0.4089403	Both
Bulk_density	nirk_Cluster33	0.0685	-0.0184	0.0828	0.0217	0.2627	0.4582307	Both
Bulk_density	nirk_Cluster34	0.0409	-0.017	0.3072	0.261	0.021	0.2693572	Both
Bulk_density	nirk_Cluster35	0.0936	-0.0279	0.0775	0.016	0.2794	0.4684904	Both
Bulk_density	nirk_Cluster36	0.0016	-0.0006	0.0352	-0.0292	0.4711	0.6335602	Both
Bulk_density	nirk_Cluster39	0.0014	-0.001	0.0625	0.00E+00	0.3331	0.5163112	Both
Bulk_density	nirk_Cluster4	0.1285	-0.0367	0.264	0.2149	0.0349	0.2693572	Both
Bulk_density	nirk_Cluster40	0.0442	-0.0077	0.0695	0.0075	0.3065	0.4933885	Both
Bulk_density	nirk_Cluster41	0.1558	-0.0117	0.0267	-0.0382	0.5309	0.6848541	Both
Bulk_density	nirk_Cluster44	0.0647	0.0207	0.1102	0.0509	0.193	0.4088887	Both
Bulk_density	nirk_Cluster46	0.0142	-0.0032	0.0274	-0.0375	0.5259	0.680733	Both
Bulk_density	nirk_Cluster48	0.003	0.0008	0.0417	-0.0222	0.4319	0.6085717	Both
Bulk_density	nirk_Cluster49	0.007	0.0013	0.0186	-0.0469	0.6021	0.7312567	Both
Bulk_density	nirk_Cluster5	0.1918	-0.0242	0.1024	0.0426	0.2105	0.4175485	Both
Bulk_density	nirk_Cluster50	0.0017	-0.0005	0.0393	-0.0247	0.4456	0.6124636	Both
Bulk_density	nirk_Cluster52	0.0446	-0.0067	0.0471	-0.0164	0.4026	0.5791243	Both
Bulk_density	nirk_Cluster53	0.0042	-0.0021	0.0927	0.0322	0.2347	0.4266312	Both
Bulk_density	nirk_Cluster54	0.0019	-0.0011	0.1189	0.0602	0.1753	0.4042816	Both
Bulk_density	nirk_Cluster55	0.0025	-0.0009	0.0247	-0.0403	0.5467	0.6924983	Both
Bulk_density	nirk_Cluster56	0.0011	0.0007	0.0826	0.0214	0.2634	0.4582307	Both
Bulk_density	nirk_Cluster57	0.0019	-0.001	0.0546	-0.0084	0.3668	0.5441998	Both
Bulk_density	nirk_Cluster58	0.0069	0.0042	0.2206	0.1687	0.0571	0.2760172	Both
Bulk_density	nirk_Cluster59	0.0043	-0.0013	0.0392	-0.0248	0.4461	0.6124636	Both
Bulk_density	nirk_Cluster6	0.1383	0.0199	0.0967	0.0365	0.2244	0.4236938	Both
Bulk_density	nirk_Cluster60	0.0006	-0.0005	0.0949	0.0345	0.2291	0.4264211	Both
Bulk_density	nirk_Cluster62	0.1325	0.0557	0.1821	0.1275	0.0876	0.3147075	Both
Bulk_density	nirk_Cluster63	0.1159	0.0234	0.0643	0.0019	0.3262	0.5099602	Both
Bulk_density	nirk_Cluster64	0.004	-0.0016	0.1189	0.0602	0.1752	0.4042816	Both
Bulk_density	nirk_Cluster65	0.044	-0.0098	0.0972	0.037	0.2232	0.4236938	Both
Bulk_density	nirk_Cluster66	0.0073	-0.0013	0.0238	-0.0413	0.5542	0.6937193	Both
Bulk_density	nirk_Cluster67	0.0095	-0.0011	0.0136	-0.0522	0.6562	0.7699062	Both
Bulk_density	nirk_Cluster68	0.0109	0.0028	0.0564	-0.0065	0.3586	0.5411534	Both
Bulk_density	nirk_Cluster69	0.0268	-0.0016	0.006	-0.0603	0.7681	0.8523979	Both
Bulk_density	nirk_Cluster7	0.0688	-0.0162	0.1708	0.1155	0.0992	0.332347	Both
Bulk_density	nirk_Cluster70	0.0711	0.051	0.3035	0.257	0.0219	0.2693572	Both
Bulk_density	nirk_Cluster71	0.005	-0.003	0.1957	0.1421	0.0754	0.2896155	Both
Bulk_density	nirk_Cluster72	0.0228	0.0016	0.0083	-0.0578	0.7284	0.8250603	Both
Bulk_density	nirk_Cluster73	0.1407	-0.0292	0.0551	-0.0079	0.3644	0.5441998	Both
Bulk_density	nirk_Cluster74	0.6374	0.1909	0.1116	0.0523	0.1901	0.4042816	Both
Bulk_density	nirk_Cluster75	0.0134	-0.0032	0.0206	-0.0447	0.5828	0.7181821	Both
Bulk_density	nirk_Cluster76	0.1	-0.0048	0.0041	-0.0623	0.8071	0.8819396	Both
Bulk_density	nirk_Cluster77	0.0012	-0.0009	0.1003	0.0403	0.2155	0.42024	Both
Bulk_density	nirk_Cluster78	0.2303	-0.0671	0.1284	0.0703	0.1578	0.3824281	Both
Bulk_density	nirk_Cluster79	0.0024	-0.0002	0.0021	-0.0644	0.8603	0.9174394	Both
Bulk_density	nirk_Cluster8	0.096	-0.0385	0.2748	0.2264	0.0308	0.2693572	Both
Bulk_density	nirk_Cluster80	0.2672	-0.0731	0.0968	0.0365	0.2243	0.4236938	Both

Bulk_density	nirk_Cluster82	0.0016	0.0011	0.0696	0.0075	0.3063	0.4933885	Both
Bulk_density	nirk_Cluster83	0.003	-0.001	0.0836	0.0225	0.2604	0.4582307	Both
Bulk_density	nirk_Cluster84	0.0226	-0.0101	0.1548	0.0984	0.1182	0.3534988	Both
Bulk_density	nirk_Cluster85	0.0022	-0.0008	0.0373	-0.0269	0.4577	0.6214753	Both
Bulk_density	nirk_Cluster86	0.0258	0.0112	0.1467	0.0898	0.1291	0.3621716	Both
Bulk_density	nirk_Cluster87	0.012	0.0029	0.0575	-0.0054	0.354	0.537434	Both
Bulk_density	nirk_Cluster88	0.003	-0.0002	0.0045	-0.0618	0.7974	0.8762547	Both
Bulk_density	nirk_Cluster89	0.0075	-0.0038	0.1621	0.1062	0.1091	0.3504205	Both
Bulk_density	nirk_Cluster9	0.0518	-0.0205	0.292	0.2448	0.0251	0.2693572	Both
Bulk_density	nirk_Cluster90	0.0078	0.0004	0.0016	-0.065	0.8803	0.9308514	Both
Bulk_density	nirk_Cluster91	0.0391	-0.0075	0.0636	0.0012	0.3288	0.5109534	Both
Bulk_density	nirk_Cluster92	0.0077	0.0034	0.1117	0.0525	0.1898	0.4042816	Both
Bulk_density	nirk_Cluster93	0.0289	-0.0006	0.0009	-0.0658	0.9114	0.9454083	Both
Bulk_density	nirk_Cluster94	0.0155	-0.0058	0.1707	0.1154	0.0993	0.332347	Both
Bulk_density	nirk_Cluster95	0.0503	0.0032	0.0049	-0.0614	0.7894	0.8706614	Both
Bulk_density	nirk_Cluster96	0.0481	-0.0098	0.0565	-0.0064	0.3583	0.5411534	Both
Bulk_density	nirk_Cluster97	0.0354	0.002	0.0071	-0.0591	0.7476	0.8371505	Both
Bulk_density	nirk_Cluster98	0.016	-0.005	0.0491	-0.0143	0.3929	0.5706918	Both
Bulk_density	nirk_Cluster99	0.0049	0.00E+00	0.00E+00	-0.0666	0.9859	0.989255	Both
Bulk_density	nirs_Cluster0	0.1283	-0.0012	0.0003	-0.0663	0.9449	0.9612656	Both
Bulk_density	nirs_Cluster1	0.0035	0.0001	0.0004	-0.0662	0.9383	0.9561963	Both
Bulk_density	nirs_Cluster14	0.0132	-0.0096	0.1505	0.0939	0.1239	0.3611472	Both
Bulk_density	nirs_Cluster2	0.0058	-0.0012	0.0209	-0.0444	0.5798	0.7159401	Both
Bulk_density	nirs_Cluster26	0.0006	-0.0005	0.0773	0.0158	0.28	0.4684904	Both
Bulk_density	nirs_Cluster28	0.0088	-0.0062	0.1345	0.0768	0.1476	0.3714225	Both
Bulk_density	nirs_Cluster3	0.1589	-0.0149	0.0802	0.0189	0.2706	0.4629597	Both
Bulk_density	nirs_Cluster36	0.0003	-0.0002	0.0253	-0.0397	0.542	0.6880218	Both
Bulk_density	nirs_Cluster37	0.0081	-0.0056	0.0673	0.0051	0.3148	0.5010716	Both
Bulk_density	nirs_Cluster4	0.0003	-0.0002	0.0253	-0.0397	0.542	0.6880218	Both
Bulk_density	nirs_Cluster50	0.0021	-0.0005	0.0145	-0.0512	0.6448	0.7656569	Both
Bulk_density	nirs_Cluster9	0.0332	-0.0248	0.1469	0.09	0.1289	0.3621716	Both
Bulk_density	norb_Cluster0	0.0915	0.0086	0.0212	-0.0441	0.5771	0.7159401	Both
Bulk_density	norb_Cluster1	1.3029	-0.0121	0.0001	-0.0665	0.9641	0.9773825	Both
Bulk_density	norb_Cluster101	0.0028	-0.0014	0.0542	-0.0089	0.3685	0.5444711	Both
Bulk_density	norb_Cluster102	0.0047	-0.0006	0.0105	-0.0555	0.6959	0.8035466	Both
Bulk_density	norb_Cluster103	0.0022	-0.0014	0.1182	0.0594	0.1767	0.4042816	Both
Bulk_density	norb_Cluster104	0.0025	-0.0014	0.0786	0.0172	0.2756	0.4650973	Both
Bulk_density	norb_Cluster106	0.1329	-0.0566	0.3146	0.2689	0.0192	0.2693572	Both
Bulk_density	norb_Cluster107	0.0126	-0.0058	0.1591	0.103	0.1128	0.3513073	Both
Bulk_density	norb_Cluster108	0.0045	-0.0023	0.1555	0.0993	0.1172	0.3534988	Both
Bulk_density	norb_Cluster109	0.022	-0.0109	0.2394	0.1887	0.0462	0.2693572	Both
Bulk_density	norb_Cluster11	0.0138	-0.0044	0.0823	0.0211	0.2643	0.4582307	Both
Bulk_density	norb_Cluster110	0.0084	-0.0042	0.117	0.0581	0.1791	0.4042816	Both
Bulk_density	norb_Cluster112	0.0169	-0.0086	0.2789	0.2309	0.0293	0.2693572	Both
Bulk_density	norb_Cluster113	0.0147	-0.0088	0.2578	0.2083	0.0375	0.2693572	Both
Bulk_density	norb_Cluster114	0.0031	-0.0021	0.0878	0.027	0.2481	0.4406251	Both
Bulk_density	norb_Cluster115	0.0165	-0.0139	0.0646	0.0022	0.325	0.5099602	Both
Bulk_density	norb_Cluster116	0.0087	-0.0034	0.0912	0.0306	0.2389	0.4303015	Both
Bulk_density	norb_Cluster117	0.0027	-0.0021	0.1928	0.139	0.0778	0.2919554	Both
Bulk_density	norb_Cluster118	0.0037	-0.0007	0.0236	-0.0415	0.5559	0.6937193	Both
Bulk_density	norb_Cluster119	0.018	-0.0133	0.2851	0.2374	0.0273	0.2693572	Both
Bulk_density	norb_Cluster12	0.0051	-0.0003	0.0015	-0.065	0.8812	0.9308514	Both
Bulk_density	norb_Cluster121	0.002	-0.0011	0.0502	-0.0131	0.3873	0.5660077	Both
Bulk_density	norb_Cluster122	0.0152	-0.0057	0.1327	0.0749	0.1506	0.3757667	Both
Bulk_density	norb_Cluster123	0.0053	-0.0038	0.2192	0.1671	0.0581	0.2760172	Both

Bulk_density	norb_Cluster124	0.8103	-0.1296	0.0579	-0.0049	0.352	0.5371597	Both
Bulk_density	norb_Cluster125	0.0043	-0.0032	0.3213	0.2761	0.0177	0.2693572	Both
Bulk_density	norb_Cluster126	0.0086	-0.0055	0.2543	0.2046	0.039	0.2693572	Both
Bulk_density	norb_Cluster127	0.1991	-0.0546	0.1217	0.0632	0.1699	0.4017931	Both
Bulk_density	norb_Cluster128	0.0133	-0.0028	0.0606	-0.0021	0.3411	0.5217902	Both
Bulk_density	norb_Cluster13	0.0056	-0.0023	0.0715	0.0096	0.2994	0.4912263	Both
Bulk_density	norb_Cluster131	0.0202	-0.0092	0.2247	0.173	0.0545	0.2760172	Both
Bulk_density	norb_Cluster132	1.0685	-0.3396	0.1939	0.1402	0.0769	0.2903707	Both
Bulk_density	norb_Cluster133	0.0012	-0.0004	0.0292	-0.0355	0.5119	0.6684904	Both
Bulk_density	norb_Cluster135	0.0622	-0.0198	0.1428	0.0857	0.1347	0.3621716	Both
Bulk_density	norb_Cluster136	0.0222	-0.0067	0.1119	0.0527	0.1895	0.4042816	Both
Bulk_density	norb_Cluster139	0.0329	-0.0119	0.1676	0.1122	0.1027	0.3397168	Both
Bulk_density	norb_Cluster14	0.0033	-0.0011	0.0541	-0.0089	0.3688	0.5444711	Both
Bulk_density	norb_Cluster140	0.0504	-0.0003	1.00E-04	-0.0666	0.9743	0.9843484	Both
Bulk_density	norb_Cluster142	0.0019	-0.0005	0.0195	-0.0459	0.5934	0.7230533	Both
Bulk_density	norb_Cluster144	0.0086	-0.0038	0.1455	0.0886	0.1308	0.3621716	Both
Bulk_density	norb_Cluster146	0.0358	-0.0062	0.0429	-0.0209	0.425	0.6002706	Both
Bulk_density	norb_Cluster15	0.0551	-0.0102	0.0533	-0.0098	0.3726	0.5486086	Both
Bulk_density	norb_Cluster150	0.015	-0.0008	0.003	-0.0635	0.8359	0.9022541	Both
Bulk_density	norb_Cluster155	0.1908	-0.0246	0.0392	-0.0248	0.446	0.6124636	Both
Bulk_density	norb_Cluster16	0.0154	-0.0063	0.124	0.0656	0.1657	0.3951218	Both
Bulk_density	norb_Cluster164	0.0246	-0.0074	0.1047	0.045	0.2053	0.4170625	Both
Bulk_density	norb_Cluster165	0.0094	-0.0055	0.2099	0.1573	0.0644	0.2809929	Both
Bulk_density	norb_Cluster166	0.0049	-0.0035	0.3045	0.2581	0.0216	0.2693572	Both
Bulk_density	norb_Cluster169	0.0287	-0.0127	0.1856	0.1314	0.0842	0.3100763	Both
Bulk_density	norb_Cluster17	0.0032	-0.0002	0.0028	-0.0637	0.8394	0.9022541	Both
Bulk_density	norb_Cluster170	0.03	-0.0151	0.2218	0.1699	0.0564	0.2760172	Both
Bulk_density	norb_Cluster171	0.008	-0.0063	0.2468	0.1966	0.0425	0.2693572	Both
Bulk_density	norb_Cluster172	0.0173	-0.0094	0.2263	0.1747	0.0536	0.2760172	Both
Bulk_density	norb_Cluster175	0.0561	-0.0274	0.2039	0.1509	0.0688	0.2852687	Both
Bulk_density	norb_Cluster176	0.0364	-0.0202	0.2617	0.2125	0.0358	0.2693572	Both
Bulk_density	norb_Cluster177	0.0337	-0.0217	0.3301	0.2854	0.0159	0.2693572	Both
Bulk_density	norb_Cluster179	0.0012	-0.0009	0.1116	0.0524	0.19	0.4042816	Both
Bulk_density	norb_Cluster18	0.0039	-0.0003	0.0028	-0.0636	0.8391	0.9022541	Both
Bulk_density	norb_Cluster180	0.0095	-0.0032	0.0961	0.0358	0.2259	0.4244829	Both
Bulk_density	norb_Cluster181	0.001	-0.0009	0.0943	0.0339	0.2307	0.4264211	Both
Bulk_density	norb_Cluster182	0.0016	-0.0003	0.0119	-0.054	0.6766	0.7875627	Both
Bulk_density	norb_Cluster184	0.0171	-0.0077	0.2009	0.1476	0.0712	0.2852687	Both
Bulk_density	norb_Cluster186	0.0013	-0.0001	0.0022	-0.0643	0.859	0.9174394	Both
Bulk_density	norb_Cluster19	0.0053	-0.0013	0.0547	-0.0084	0.3665	0.5441998	Both
Bulk_density	norb_Cluster192	0.0214	-0.0116	0.2898	0.2425	0.0258	0.2693572	Both
Bulk_density	norb_Cluster193	0.0026	-0.0021	0.2689	0.2202	0.033	0.2693572	Both
Bulk_density	norb_Cluster197	0.037	-0.0268	0.0974	0.0373	0.2226	0.4236938	Both
Bulk_density	norb_Cluster2	0.0039	-0.0026	0.2145	0.1621	0.0612	0.2798971	Both
Bulk_density	norb_Cluster20	0.0684	-0.0079	0.0274	-0.0375	0.5256	0.680733	Both
Bulk_density	norb_Cluster208	0.0013	-0.001	0.0994	0.0394	0.2177	0.4230744	Both
Bulk_density	norb_Cluster21	0.0557	-0.0034	0.0053	-0.061	0.7815	0.8636216	Both
Bulk_density	norb_Cluster211	0.0116	-0.0092	0.1203	0.0616	0.1726	0.4042816	Both
Bulk_density	norb_Cluster212	0.0326	-0.0258	0.2501	0.2001	0.0409	0.2693572	Both
Bulk_density	norb_Cluster214	0.0129	-0.0089	0.1404	0.0831	0.1383	0.3621716	Both
Bulk_density	norb_Cluster217	0.0177	-0.0126	0.2099	0.1572	0.0644	0.2809929	Both
Bulk_density	norb_Cluster219	0.0102	-0.0075	0.1667	0.1111	0.1038	0.3415184	Both
Bulk_density	norb_Cluster22	0.0021	0.0007	0.0238	-0.0413	0.5545	0.6937193	Both
Bulk_density	norb_Cluster220	0.0217	-0.0156	0.1376	0.0801	0.1427	0.3677743	Both
Bulk_density	norb_Cluster221	0.0209	-0.0154	0.1152	0.0563	0.1825	0.4042816	Both

Bulk_density	norb_Cluster222	0.0092	-0.0071	0.1035	0.0437	0.208	0.4170625	Both
Bulk_density	norb_Cluster224	0.024	-0.0186	0.2555	0.2058	0.0385	0.2693572	Both
Bulk_density	norb_Cluster225	0.0107	-0.0087	0.2441	0.1937	0.0438	0.2693572	Both
Bulk_density	norb_Cluster226	0.0047	-0.0038	0.2298	0.1784	0.0515	0.2756752	Both
Bulk_density	norb_Cluster228	0.0039	-0.0038	0.0877	0.0269	0.2484	0.4406251	Both
Bulk_density	norb_Cluster229	0.0037	-0.0017	0.0898	0.0292	0.2425	0.4328333	Both
Bulk_density	norb_Cluster230	0.0013	-0.0009	0.0929	0.0324	0.2342	0.4266312	Both
Bulk_density	norb_Cluster231	0.0033	-0.0012	0.049	-0.0144	0.3934	0.5706918	Both
Bulk_density	norb_Cluster232	0.0025	-0.0008	0.0376	-0.0265	0.4558	0.6214753	Both
Bulk_density	norb_Cluster234	0.0137	-0.008	0.1473	0.0904	0.1284	0.3621716	Both
Bulk_density	norb_Cluster235	0.0009	-0.0007	0.1187	0.0599	0.1758	0.4042816	Both
Bulk_density	norb_Cluster238	0.0088	-0.0059	0.0939	0.0335	0.2315	0.4264211	Both
Bulk_density	norb_Cluster239	0.0082	-0.0044	0.069	0.007	0.3083	0.4933885	Both
Bulk_density	norb_Cluster24	0.3043	-0.0085	0.0015	-0.0651	0.8824	0.9308514	Both
Bulk_density	norb_Cluster244	0.0006	-0.0005	0.0703	0.0083	0.3038	0.4933885	Both
Bulk_density	norb_Cluster247	0.003	-0.0019	0.2659	0.2169	0.0341	0.2693572	Both
Bulk_density	norb_Cluster25	0.1329	-0.0153	0.0237	-0.0414	0.5551	0.6937193	Both
Bulk_density	norb_Cluster250	0.0111	-0.0077	0.2677	0.2189	0.0334	0.2693572	Both
Bulk_density	norb_Cluster251	0.005	-0.0027	0.1265	0.0683	0.1611	0.3889884	Both
Bulk_density	norb_Cluster26	0.1112	-0.0028	0.0009	-0.0657	0.9078	0.9454083	Both
Bulk_density	norb_Cluster28	0.0332	-0.0187	0.4332	0.3954	0.0041	0.2693572	Both
Bulk_density	norb_Cluster29	0.2468	-0.0396	0.1287	0.0706	0.1573	0.3824281	Both
Bulk_density	norb_Cluster31	0.06	0.0042	0.0065	-0.0597	0.758	0.8456075	Both
Bulk_density	norb_Cluster32	0.1423	0.0043	0.0011	-0.0654	0.8975	0.9406085	Both
Bulk_density	norb_Cluster33	0.5733	0.0691	0.1042	0.0445	0.2064	0.4170625	Both
Bulk_density	norb_Cluster35	0.0738	-0.0057	0.0101	-0.0559	0.7016	0.8055532	Both
Bulk_density	norb_Cluster36	1.3547	0.3902	0.272	0.2235	0.0318	0.2693572	Both
Bulk_density	norb_Cluster37	0.8807	0.1985	0.2997	0.253	0.0229	0.2693572	Both
Bulk_density	norb_Cluster38	0.0126	-0.0038	0.1241	0.0657	0.1656	0.3951218	Both
Bulk_density	norb_Cluster39	0.0482	-0.0107	0.0687	0.0066	0.3094	0.4939235	Both
Bulk_density	norb_Cluster40	0.2112	-0.04	0.0576	-0.0052	0.3536	0.537434	Both
Bulk_density	norb_Cluster42	0.3451	0.0441	0.0661	0.0038	0.3192	0.5067217	Both
Bulk_density	norb_Cluster44	0.0159	-0.0091	0.1604	0.1044	0.1112	0.3504205	Both
Bulk_density	norb_Cluster46	0.2865	-0.0789	0.1286	0.0705	0.1576	0.3824281	Both
Bulk_density	norb_Cluster47	0.6437	-0.0579	0.0174	-0.0481	0.6136	0.7391407	Both
Bulk_density	norb_Cluster5	0.0109	0.002	0.0447	-0.019	0.4153	0.5922895	Both
Bulk_density	norb_Cluster51	0.0106	-0.0061	0.1436	0.0865	0.1336	0.3621716	Both
Bulk_density	norb_Cluster52	0.2765	-0.0222	0.0104	-0.0556	0.6971	0.8035466	Both
Bulk_density	norb_Cluster56	0.0146	-0.0032	0.056	-0.0069	0.3605	0.541724	Both
Bulk_density	norb_Cluster57	0.0037	-0.0017	0.1538	0.0974	0.1195	0.3541145	Both
Bulk_density	norb_Cluster59	0.0168	-0.009	0.0924	0.0319	0.2356	0.4268143	Both
Bulk_density	norb_Cluster6	0.8172	0.2224	0.2588	0.2094	0.037	0.2693572	Both
Bulk_density	norb_Cluster60	0.3932	-0.0725	0.0401	-0.0239	0.4411	0.6124636	Both
Bulk_density	norb_Cluster61	0.5458	-0.248	0.3217	0.2764	0.0176	0.2693572	Both
Bulk_density	norb_Cluster62	0.0023	1.00E-04	0.0008	-0.0658	0.9126	0.9454083	Both
Bulk_density	norb_Cluster64	0.033	-0.0117	0.1805	0.1259	0.0891	0.3161665	Both
Bulk_density	norb_Cluster66	0.0012	-0.0009	0.0937	0.0333	0.2321	0.4264211	Both
Bulk_density	norb_Cluster67	0.0188	-0.0097	0.2294	0.178	0.0518	0.2756752	Both
Bulk_density	norb_Cluster68	0.0039	-0.0022	0.2852	0.2375	0.0272	0.2693572	Both
Bulk_density	norb_Cluster69	0.0029	-0.0001	0.001	-0.0656	0.9036	0.9453386	Both
Bulk_density	norb_Cluster7	0.083	-0.0185	0.0973	0.0371	0.223	0.4236938	Both
Bulk_density	norb_Cluster70	0.109	-0.0303	0.1144	0.0554	0.1841	0.4042816	Both
Bulk_density	norb_Cluster71	0.1378	-0.055	0.3094	0.2633	0.0204	0.2693572	Both
Bulk_density	norb_Cluster74	0.034	-0.0139	0.2445	0.1941	0.0436	0.2693572	Both
Bulk_density	norb_Cluster75	0.1358	-0.0383	0.1809	0.1263	0.0887	0.3161665	Both

Bulk_density	norb_Cluster76	0.1348	-0.04	0.0718	0.0099	0.2984	0.490958	Both
Bulk_density	norb_Cluster77	0.0492	-0.0117	0.1599	0.1039	0.1118	0.3504205	Both
Bulk_density	norb_Cluster8	0.0204	-0.0096	0.2512	0.2013	0.0404	0.2693572	Both
Bulk_density	norb_Cluster80	0.0019	-0.0014	0.1613	0.1053	0.1101	0.3504205	Both
Bulk_density	norb_Cluster81	0.0014	-0.001	0.1435	0.0865	0.1337	0.3621716	Both
Bulk_density	norb_Cluster83	0.0089	-0.006	0.1121	0.0529	0.1891	0.4042816	Both
Bulk_density	norb_Cluster84	0.0081	-0.0032	0.102	0.0421	0.2115	0.4175485	Both
Bulk_density	norb_Cluster85	0.0028	-0.0011	0.0752	0.0135	0.287	0.4760953	Both
Bulk_density	norb_Cluster86	0.0383	-0.0263	0.175	0.12	0.0947	0.3244257	Both
Bulk_density	norb_Cluster88	0.002	-0.0016	0.2071	0.1543	0.0664	0.2814384	Both
Bulk_density	norb_Cluster90	0.0009	-0.0007	0.0339	-0.0305	0.4793	0.6416533	Both
Bulk_density	norb_Cluster91	0.7588	-0.3445	0.32	0.2747	0.0179	0.2693572	Both
Bulk_density	norb_Cluster92	0.0012	-0.001	0.0616	-0.001	0.3368	0.5193196	Both
Bulk_density	norb_Cluster93	0.0028	-0.0011	0.0392	-0.0248	0.4461	0.6124636	Both
Bulk_density	norb_Cluster94	0.0011	-0.0003	0.015	-0.0506	0.6393	0.7622673	Both
Bulk_density	norb_Cluster96	0.0006	-0.0006	0.1016	0.0417	0.2123	0.4175485	Both
Bulk_density	norb_Cluster97	0.0079	-0.0024	0.0642	0.0018	0.3264	0.5099602	Both
Bulk_density	norb_Cluster98	0.0161	-0.0093	0.2013	0.148	0.0709	0.2852687	Both
Bulk_density	nosz_Cluster0	0.004	-0.0022	0.1408	0.0835	0.1378	0.3621716	Both
Bulk_density	nosz_Cluster10	0.0158	-0.0099	0.2855	0.2378	0.0271	0.2693572	Both
Bulk_density	nosz_Cluster102	0.0033	-0.0006	0.0283	-0.0365	0.5186	0.6758398	Both
Bulk_density	nosz_Cluster103	0.0504	-0.0041	0.0155	-0.0501	0.6338	0.7587467	Both
Bulk_density	nosz_Cluster105	0.0015	-0.0007	0.0804	0.0191	0.2699	0.4629597	Both
Bulk_density	nosz_Cluster106	0.0085	-0.0019	0.0396	-0.0244	0.4439	0.6124636	Both
Bulk_density	nosz_Cluster107	0.0003	-0.0002	0.0253	-0.0397	0.542	0.6880218	Both
Bulk_density	nosz_Cluster108	0.0048	-0.0031	0.2425	0.192	0.0446	0.2693572	Both
Bulk_density	nosz_Cluster109	0.0379	-0.0013	0.0028	-0.0636	0.8392	0.9022541	Both
Bulk_density	nosz_Cluster11	0.0127	-0.0087	0.1018	0.0419	0.2119	0.4175485	Both
Bulk_density	nosz_Cluster110	0.0003	0.0005	0.1175	0.0587	0.178	0.4042816	Both
Bulk_density	nosz_Cluster111	0.0066	-0.0033	0.18	0.1253	0.0897	0.3163017	Both
Bulk_density	nosz_Cluster116	0.0066	-0.0048	0.3041	0.2577	0.0218	0.2693572	Both
Bulk_density	nosz_Cluster118	0.005	-0.0038	0.2023	0.1491	0.0701	0.2852687	Both
Bulk_density	nosz_Cluster119	0.0016	-0.0012	0.1452	0.0882	0.1313	0.3621716	Both
Bulk_density	nosz_Cluster120	0.007	-0.0021	0.0904	0.0297	0.241	0.43146	Both
Bulk_density	nosz_Cluster123	0.0104	-0.0069	0.221	0.1691	0.0569	0.2760172	Both
Bulk_density	nosz_Cluster128	0.0058	-0.0041	0.2719	0.2234	0.0318	0.2693572	Both
Bulk_density	nosz_Cluster129	0.0565	-0.0099	0.2535	0.2037	0.0394	0.2693572	Both
Bulk_density	nosz_Cluster13	0.011	0.0008	0.0042	-0.0622	0.8055	0.8818982	Both
Bulk_density	nosz_Cluster130	0.1081	-0.0613	0.0839	0.0229	0.2593	0.4582307	Both
Bulk_density	nosz_Cluster132	0.0039	-0.0003	0.0077	-0.0584	0.7374	0.832052	Both
Bulk_density	nosz_Cluster14	0.0271	-0.0249	0.1416	0.0844	0.1366	0.3621716	Both
Bulk_density	nosz_Cluster16	0.0083	-0.0063	0.2206	0.1687	0.0571	0.2760172	Both
Bulk_density	nosz_Cluster17	0.04	-0.0195	0.3515	0.3083	0.0121	0.2693572	Both
Bulk_density	nosz_Cluster18	0.0154	-0.0126	0.2687	0.22	0.033	0.2693572	Both
Bulk_density	nosz_Cluster20	0.0106	-0.0072	0.2367	0.1858	0.0477	0.2725772	Both
Bulk_density	nosz_Cluster21	0.0097	-0.0066	0.2115	0.1589	0.0632	0.2804082	Both
Bulk_density	nosz_Cluster22	0.0304	-0.0211	0.2184	0.1663	0.0586	0.2760172	Both
Bulk_density	nosz_Cluster23	0.0158	-0.0116	0.2955	0.2485	0.0241	0.2693572	Both
Bulk_density	nosz_Cluster24	0.0009	-0.0006	0.0498	-0.0136	0.3893	0.567585	Both
Bulk_density	nosz_Cluster25	0.0028	-0.0024	0.1475	0.0907	0.128	0.3621716	Both
Bulk_density	nosz_Cluster26	0.0062	-0.0047	0.2669	0.218	0.0337	0.2693572	Both
Bulk_density	nosz_Cluster27	0.0049	-0.0036	0.2136	0.1612	0.0618	0.2798971	Both
Bulk_density	nosz_Cluster28	0.0325	-0.0131	0.1495	0.0928	0.1253	0.3616366	Both
Bulk_density	nosz_Cluster30	0.0275	-0.0192	0.4389	0.4015	0.0038	0.2693572	Both
Bulk_density	nosz_Cluster32	0.0129	-0.0072	0.2346	0.1835	0.0488	0.2743643	Both

Bulk_density	nosz_Cluster33	0.0208	-0.0157	0.2922	0.2451	0.0251	0.2693572	Both
Bulk_density	nosz_Cluster34	0.0461	-0.0302	0.2933	0.2461	0.0248	0.2693572	Both
Bulk_density	nosz_Cluster35	0.0076	-0.0056	0.3337	0.2893	0.0152	0.2693572	Both
Bulk_density	nosz_Cluster36	0.0203	-0.012	0.2864	0.2388	0.0269	0.2693572	Both
Bulk_density	nosz_Cluster37	0.0056	-0.0043	0.2386	0.1878	0.0466	0.2693572	Both
Bulk_density	nosz_Cluster38	0.0129	-0.0097	0.269	0.2203	0.0329	0.2693572	Both
Bulk_density	nosz_Cluster39	0.0527	-0.033	0.3647	0.3224	0.0102	0.2693572	Both
Bulk_density	nosz_Cluster40	0.02	-0.0153	0.389	0.3482	0.0075	0.2693572	Both
Bulk_density	nosz_Cluster41	0.019	-0.0111	0.2256	0.1739	0.054	0.2760172	Both
Bulk_density	nosz_Cluster42	0.0311	-0.0224	0.3979	0.3577	0.0066	0.2693572	Both
Bulk_density	nosz_Cluster43	0.0009	-0.0007	0.0711	0.0092	0.3007	0.4920133	Both
Bulk_density	nosz_Cluster44	0.006	0.0026	0.097	0.0368	0.2237	0.4236938	Both
Bulk_density	nosz_Cluster45	0.0048	-0.0027	0.1836	0.1292	0.0861	0.3131771	Both
Bulk_density	nosz_Cluster46	0.007	-0.0034	0.1952	0.1415	0.0758	0.2896155	Both
Bulk_density	nosz_Cluster47	0.001	-0.0006	0.0743	0.0126	0.2899	0.4789538	Both
Bulk_density	nosz_Cluster48	0.0026	-0.002	0.1725	0.1173	0.0974	0.3307876	Both
Bulk_density	nosz_Cluster49	0.0013	-0.0012	0.2489	0.1988	0.0415	0.2693572	Both
Bulk_density	nosz_Cluster5	0.0053	-0.0046	0.2189	0.1668	0.0583	0.2760172	Both
Bulk_density	nosz_Cluster51	0.0024	-0.0015	0.1369	0.0793	0.1438	0.3677743	Both
Bulk_density	nosz_Cluster53	0.0006	-0.0004	0.0253	-0.0397	0.542	0.6880218	Both
Bulk_density	nosz_Cluster54	0.0033	-0.0018	0.1052	0.0455	0.2041	0.4170625	Both
Bulk_density	nosz_Cluster57	0.1909	-0.0501	0.0647	0.0023	0.3246	0.5099602	Both
Bulk_density	nosz_Cluster58	0.0053	-0.003	0.155	0.0987	0.1179	0.3534988	Both
Bulk_density	nosz_Cluster59	0.0011	-0.0009	0.0782	0.0167	0.2772	0.4664239	Both
Bulk_density	nosz_Cluster6	0.0035	-0.0029	0.2543	0.2046	0.039	0.2693572	Both
Bulk_density	nosz_Cluster60	0.007	-0.0048	0.3381	0.2939	0.0144	0.2693572	Both
Bulk_density	nosz_Cluster61	0.0192	0.0023	0.0333	-0.0312	0.4836	0.6444672	Both
Bulk_density	nosz_Cluster62	0.0454	-0.013	0.0827	0.0215	0.2631	0.4582307	Both
Bulk_density	nosz_Cluster64	0.0006	0.0002	0.0139	-0.0519	0.6524	0.7671165	Both
Bulk_density	nosz_Cluster65	0.0065	-0.0045	0.2344	0.1834	0.0489	0.2743643	Both
Bulk_density	nosz_Cluster66	0.0365	-0.0016	0.0013	-0.0653	0.8924	0.9368951	Both
Bulk_density	nosz_Cluster67	0.0567	-0.016	0.0548	-0.0082	0.366	0.5441998	Both
Bulk_density	nosz_Cluster69	0.0075	-0.0042	0.272	0.2234	0.0318	0.2693572	Both
Bulk_density	nosz_Cluster70	0.0551	-0.0406	0.1563	0.1	0.1163	0.3534988	Both
Bulk_density	nosz_Cluster71	0.0092	-0.0073	0.2781	0.23	0.0296	0.2693572	Both
Bulk_density	nosz_Cluster72	0.0566	-0.0197	0.104	0.0443	0.2068	0.4170625	Both
Bulk_density	nosz_Cluster73	0.0032	-0.0008	0.0237	-0.0414	0.555	0.6937193	Both
Bulk_density	nosz_Cluster74	0.0021	-0.0009	0.0798	0.0185	0.2718	0.4629597	Both
Bulk_density	nosz_Cluster75	0.1172	-0.0789	0.0827	0.0215	0.2631	0.4582307	Both
Bulk_density	nosz_Cluster77	0.0002	0.0003	0.1175	0.0587	0.178	0.4042816	Both
Bulk_density	nosz_Cluster78	0.0052	-0.0012	0.04	-0.024	0.4417	0.6124636	Both
Bulk_density	nosz_Cluster79	0.0319	-0.0125	0.2086	0.1558	0.0654	0.2810476	Both
Bulk_density	nosz_Cluster8	0.0105	-0.0064	0.3815	0.3402	0.0082	0.2693572	Both
Bulk_density	nosz_Cluster80	0.0053	-0.0039	0.1844	0.13	0.0854	0.3124691	Both
Bulk_density	nosz_Cluster81	0.0088	-0.0057	0.1305	0.0725	0.1543	0.3803333	Both
Bulk_density	nosz_Cluster82	0	0	#VALUE!	#VALUE!	#VALUE!	NA	Both
Bulk_density	nosz_Cluster83	0.0018	-0.0004	0.0106	-0.0554	0.6944	0.8035466	Both
Bulk_density	nosz_Cluster85	0.0012	-0.0009	0.1117	0.0525	0.1898	0.4042816	Both
Bulk_density	nosz_Cluster86	0.0017	-0.0013	0.0934	0.033	0.2329	0.4264211	Both
Bulk_density	nosz_Cluster87	0.0027	-0.0019	0.2204	0.1684	0.0573	0.2760172	Both
Bulk_density	nosz_Cluster88	0.0243	-0.0045	0.1012	0.0413	0.2134	0.4175485	Both
Bulk_density	nosz_Cluster89	0.0514	-0.0076	0.0177	-0.0478	0.6112	0.7377516	Both
Bulk_density	nosz_Cluster9	0.0031	-0.002	0.1348	0.0771	0.1472	0.3714225	Both
Bulk_density	nosz_Cluster90	0.0116	-0.0023	0.0547	-0.0083	0.3661	0.5441998	Both
Bulk_density	nosz_Cluster91	0.0205	-0.0117	0.1947	0.141	0.0762	0.2896155	Both

Bulk_density	nosz_Cluster93	0.0418	-0.0159	0.1397	0.0824	0.1394	0.3632721	Both
Bulk_density	nosz_Cluster95	0.0078	-0.0026	0.0933	0.0329	0.2331	0.4264211	Both
Bulk_density	nosz_Cluster96	0.0067	-0.0024	0.1332	0.0754	0.1498	0.3754879	Both
Bulk_density	nosz_Cluster97	0.0019	-0.0007	0.0374	-0.0268	0.4571	0.6214753	Both
Bulk_density	nosz_Cluster98	0.0003	-0.0003	0.0434	-0.0204	0.4225	0.5996212	Both
Bulk_density	nosz_Cluster99	0.0056	-0.0038	0.4183	0.3795	0.005	0.2693572	Both
Bulk_density	nrfa_Cluster10	0.0857	-0.0567	0.2613	0.2121	0.036	0.2693572	Both
Bulk_density	nrfa_Cluster100	0.1085	-0.0613	0.156	0.0997	0.1166	0.3534988	Both
Bulk_density	nrfa_Cluster104	0.1073	-0.0358	0.0921	0.0316	0.2362	0.4268143	Both
Bulk_density	nrfa_Cluster105	0.0069	-0.0057	0.2457	0.1955	0.043	0.2693572	Both
Bulk_density	nrfa_Cluster109	0.2016	-0.0946	0.1956	0.1419	0.0755	0.2896155	Both
Bulk_density	nrfa_Cluster116	0.2396	-0.0614	0.0936	0.0331	0.2325	0.4264211	Both
Bulk_density	nrfa_Cluster118	0.1975	-0.1209	0.1532	0.0968	0.1202	0.3541145	Both
Bulk_density	nrfa_Cluster125	0.0925	-0.0597	0.1516	0.095	0.1224	0.358714	Both
Bulk_density	nrfa_Cluster154	0.072	-0.0469	0.2114	0.1588	0.0633	0.2804082	Both
Bulk_density	nrfa_Cluster164	0.0051	-0.0038	0.2238	0.172	0.0551	0.2760172	Both
Bulk_density	nrfa_Cluster172	0.0032	-0.0024	0.163	0.1072	0.1081	0.3496786	Both
Bulk_density	nrfa_Cluster175	0.0055	-0.0041	0.1786	0.1238	0.091	0.3191125	Both
Bulk_density	nrfa_Cluster20	0.0306	-0.0174	0.145	0.088	0.1315	0.3621716	Both
Bulk_density	nrfa_Cluster26	0.1243	-0.0764	0.1759	0.1209	0.0938	0.3244257	Both
Bulk_density	nrfa_Cluster29	0.0059	-0.0014	0.0292	-0.0355	0.5118	0.6684904	Both
Bulk_density	nrfa_Cluster34	0.0441	-0.0237	0.1248	0.0665	0.1642	0.3947201	Both
Bulk_density	nrfa_Cluster38	0.0039	-0.0025	0.1579	0.1017	0.1143	0.3524168	Both
Bulk_density	nrfa_Cluster61	0.0031	-0.0017	0.0985	0.0384	0.2199	0.4236938	Both
Bulk_density	nrfa_Cluster62	0.0518	-0.0317	0.1417	0.0845	0.1364	0.3621716	Both
Bulk_density	nrfa_Cluster66	0.1051	-0.0407	0.1751	0.1201	0.0946	0.3244257	Both
Bulk_density	nrfa_Cluster69	0.0037	-0.0023	0.1967	0.1432	0.0745	0.2896155	Both
Bulk_density	nrfa_Cluster71	0.0292	-0.0203	0.2085	0.1558	0.0654	0.2810476	Both
Bulk_density	nrfa_Cluster72	0.0764	-0.0445	0.1635	0.1077	0.1074	0.3496535	Both
Bulk_density	nrfa_Cluster73	0.0047	-0.0038	0.245	0.1947	0.0434	0.2693572	Both
Bulk_density	nrfa_Cluster74	0.1504	-0.0839	0.1922	0.1383	0.0784	0.2921942	Both
Bulk_density	nrfa_Cluster76	0.0877	-0.0604	0.2722	0.2237	0.0317	0.2693572	Both
Bulk_density	nrfa_Cluster79	0.0283	-0.0254	0.1348	0.0771	0.1472	0.3714225	Both
Bulk_density	nrfa_Cluster87	0.021	-0.0159	0.2154	0.1631	0.0606	0.2798971	Both
Bulk_density	nrfa_Cluster89	0.0023	-0.0011	0.0637	0.0012	0.3286	0.5109534	Both
Bulk_density	nrfa_Cluster92	0.0053	-0.0037	0.2291	0.1777	0.052	0.2756752	Both
Bulk_density	nrfa_Cluster93	0.0528	-0.0308	0.1299	0.0718	0.1553	0.381254	Both
Bulk_density	nrfa_Cluster94	0.0003	-0.0002	0.0324	-0.0321	0.4892	0.6490264	Both
Bulk_density	nrfa_Cluster97	0.0186	-0.0059	0.0742	0.0124	0.2903	0.4789538	Both
Bulk_density	nrfa_Cluster99	0.0154	-0.0055	0.076	0.0144	0.2843	0.4742178	Both
pH	amoA_A_Cluster26	0.004	-0.0017	0.0532	0.0034	0.3145	0.6821347	Both
pH	amoA_A_Cluster45	0.0051	-0.0006	0.0045	-0.0478	0.7714	0.9213413	Both
pH	amoA_B_Cluster0	0.1099	0.0318	0.3923	0.3603	0.0024	0.0373186	Both
pH	amoA_B_Cluster1	0.0172	0.0128	0.5641	0.5411	1.00E-04	0.0036781	Both
pH	amoA_B_Cluster10	0.0422	0.0138	0.2004	0.1583	0.0419	0.2226046	Both
pH	amoA_B_Cluster11	0.0377	0.0265	0.6817	0.6649	0.00E+00	0.000299	Both
pH	amoA_B_Cluster12	0.0807	0.0304	0.5109	0.4852	0.0003	0.0094161	Both
pH	amoA_B_Cluster13	0.5271	0.2269	0.3137	0.2776	0.0083	0.0704813	Both
pH	amoA_B_Cluster14	0.0275	0.0115	0.1576	0.1133	0.0747	0.3050575	Both
pH	amoA_B_Cluster15	0.0318	0.0182	0.4293	0.3993	0.0013	0.0248666	Both
pH	amoA_B_Cluster2	0.1721	0.0872	0.4176	0.3869	0.0016	0.0277409	Both
pH	amoA_B_Cluster20	0.0058	0.0047	0.2672	0.2287	0.0164	0.1182783	Both
pH	amoA_B_Cluster23	0.0507	0.0295	0.7103	0.6951	0.00E+00	0.0001598	Both
pH	amoA_B_Cluster3	0.0182	0.0149	0.5676	0.5448	1.00E-04	0.0036603	Both
pH	amoA_B_Cluster4	0.0125	0.0059	0.1594	0.1152	0.0729	0.3009663	Both

pH	amoA_B_Cluster5	0.0928	0.0336	0.4908	0.464	0.0004	0.0118661	Both
pH	amoA_B_Cluster6	0.0233	0.0102	0.3608	0.3272	0.004	0.0467399	Both
pH	amoA_B_Cluster7	0.0179	0.0101	0.2663	0.2277	0.0166	0.1182783	Both
pH	amoA_B_Cluster8	0.0207	0.0154	0.4449	0.4157	0.001	0.0209326	Both
pH	amoA_B_Cluster9	0.0549	0.0383	0.5505	0.5268	0.0001	0.0043509	Both
pH	nifh_Cluster0	0.0332	-0.0023	0.0209	-0.0306	0.5318	0.8024797	Both
pH	nifh_Cluster10	0.005	0.0003	0.0008	-0.0518	0.9055	0.9698319	Both
pH	nifh_Cluster100	0.0019	-0.0003	0.0071	-0.0452	0.7167	0.8968256	Both
pH	nifh_Cluster103	0.0383	0.0081	0.097	0.0495	0.1693	0.4872797	Both
pH	nifh_Cluster1034	0.0262	0.0116	0.1462	0.1013	0.0871	0.3337936	Both
pH	nifh_Cluster105	0.013	0.0035	0.0499	-0.0001	0.3304	0.6962818	Both
pH	nifh_Cluster108	0.0083	-0.0007	0.0057	-0.0467	0.746	0.9001951	Both
pH	nifh_Cluster1099	0.066	-0.035	0.2853	0.2476	0.0126	0.0980567	Both
pH	nifh_Cluster11	0.0021	0.0003	0.0082	-0.044	0.6966	0.8890351	Both
pH	nifh_Cluster1112	0.0029	0.0016	0.1137	0.0671	0.135	0.4258208	Both
pH	nifh_Cluster112	0.0112	-0.0058	0.2437	0.2039	0.0229	0.1470761	Both
pH	nifh_Cluster113	0.0327	0.0053	0.0665	0.0174	0.2589	0.606194	Both
pH	nifh_Cluster114	0.0332	-0.0003	0.0003	-0.0523	0.9369	0.9870745	Both
pH	nifh_Cluster1141	0.0251	0.0105	0.1975	0.1553	0.0435	0.2233132	Both
pH	nifh_Cluster115	0.0095	-0.0003	0.0017	-0.0508	0.8593	0.951334	Both
pH	nifh_Cluster116	0.0775	0.0428	0.8149	0.8051	0.00E+00	1.28E-05	Both
pH	nifh_Cluster1163	0.0044	0.0024	0.2558	0.2166	0.0193	0.1300438	Both
pH	nifh_Cluster1179	0.0031	0.001	0.0332	-0.0177	0.4295	0.7702922	Both
pH	nifh_Cluster1182	0.0204	-0.0006	0.0039	-0.0485	0.7871	0.9306036	Both
pH	nifh_Cluster1183	0.0066	-0.0018	0.0909	0.043	0.1842	0.5102398	Both
pH	nifh_Cluster1197	0.0115	0.0018	0.0315	-0.0194	0.4413	0.773964	Both
pH	nifh_Cluster1199	0.0046	0.0016	0.089	0.0411	0.1889	0.5183206	Both
pH	nifh_Cluster120	0.0149	0.006	0.045	-0.0053	0.3561	0.720084	Both
pH	nifh_Cluster1203	0.0155	0.0056	0.0791	0.0307	0.2168	0.5658563	Both
pH	nifh_Cluster1206	0.0043	0.0034	0.3918	0.3598	0.0024	0.0373186	Both
pH	nifh_Cluster1207	0.0074	0.0026	0.0725	0.0237	0.2377	0.589368	Both
pH	nifh_Cluster1209	0.1506	0.0252	0.2059	0.1641	0.0388	0.2100181	Both
pH	nifh_Cluster121	0.0095	-0.0008	0.0021	-0.0504	0.8428	0.9481017	Both
pH	nifh_Cluster1211	0.0056	0.0038	0.3744	0.3415	0.0032	0.0429105	Both
pH	nifh_Cluster1213	0.0026	0.0003	0.0083	-0.0439	0.6948	0.8890351	Both
pH	nifh_Cluster122	0.0029	0.00E+00	1.00E-04	-0.0526	0.9756	0.9894061	Both
pH	nifh_Cluster123	0.0095	-0.0002	0.0001	-0.0525	0.9623	0.9894061	Both
pH	nifh_Cluster1236	0.0234	-0.0084	0.0522	0.0023	0.3193	0.6849676	Both
pH	nifh_Cluster1238	0.0028	-0.0006	0.015	-0.0368	0.5968	0.8323762	Both
pH	nifh_Cluster124	0.0091	0.0011	0.0149	-0.0369	0.598	0.8323762	Both
pH	nifh_Cluster1261	0.002	0.00E+00	0.00E+00	-0.0526	0.9783	0.9894061	Both
pH	nifh_Cluster1262	0.0065	-0.0003	0.001	-0.0516	0.8944	0.9647029	Both
pH	nifh_Cluster1266	0.0094	-0.006	0.3539	0.3199	0.0044	0.0484538	Both
pH	nifh_Cluster1267	0.0562	-0.0406	0.4426	0.4132	0.001	0.0210422	Both
pH	nifh_Cluster127	0.0026	0.0005	0.0098	-0.0423	0.6689	0.8791893	Both
pH	nifh_Cluster1278	0.1602	0.0917	0.4886	0.4617	0.0004	0.0118661	Both
pH	nifh_Cluster128	0.0036	0.0006	0.0127	-0.0392	0.6261	0.8551259	Both
pH	nifh_Cluster1289	0.0039	0.00E+00	1.00E-04	-0.0525	0.9697	0.9894061	Both
pH	nifh_Cluster129	0.0077	0.0004	0.0013	-0.0513	0.8776	0.9606908	Both
pH	nifh_Cluster1292	0.0086	0.0039	0.153	0.1084	0.0796	0.3150331	Both
pH	nifh_Cluster130	0.0059	0.0011	0.0079	-0.0443	0.7008	0.8890351	Both
pH	nifh_Cluster1305	0.0048	-0.0005	0.0083	-0.0439	0.695	0.8890351	Both
pH	nifh_Cluster1306	0.005	0.0011	0.0328	-0.0181	0.4319	0.7706898	Both
pH	nifh_Cluster131	0.0051	0.0006	0.0041	-0.0483	0.7828	0.9291854	Both
pH	nifh_Cluster1319	0.0036	0.0021	0.1235	0.0774	0.1182	0.3940624	Both

pH	nifh_Cluster132	0.0064	-1.00E-04	0.00E+00	-0.0526	0.9793	0.9894061	Both
pH	nifh_Cluster1320	0.0025	0.0006	0.0173	-0.0344	0.5693	0.8293081	Both
pH	nifh_Cluster1323	0.0023	0.0011	0.1343	0.0887	0.1023	0.3680838	Both
pH	nifh_Cluster1324	0.03	-0.0211	0.6022	0.5812	0.00E+00	0.0019092	Both
pH	nifh_Cluster1336	0.0153	0.0016	0.0378	-0.0129	0.3985	0.7487412	Both
pH	nifh_Cluster1340	0.0363	0.0335	0.4381	0.4086	0.0011	0.021999	Both
pH	nifh_Cluster1345	0.0483	-0.0286	0.2954	0.2583	0.0109	0.0868068	Both
pH	nifh_Cluster1370	0.0021	0.001	0.0767	0.0281	0.2242	0.5727488	Both
pH	nifh_Cluster1375	0.0018	-0.0012	0.2199	0.1789	0.032	0.1848549	Both
pH	nifh_Cluster139	0.0041	0.001	0.0261	-0.0251	0.4839	0.788532	Both
pH	nifh_Cluster140	0.0043	0.0009	0.0313	-0.0197	0.4429	0.773964	Both
pH	nifh_Cluster141	0.0677	0.0255	0.319	0.2832	0.0076	0.0682074	Both
pH	nifh_Cluster148	0.0046	0.0039	0.2156	0.1744	0.0339	0.1907224	Both
pH	nifh_Cluster1480	0.001	-0.0009	0.1981	0.1559	0.0432	0.2233132	Both
pH	nifh_Cluster152	0.0599	0.001	0.0008	-0.0518	0.9015	0.9698319	Both
pH	nifh_Cluster156	0.0659	-0.0425	0.7379	0.7241	0.00E+00	0.0001218	Both
pH	nifh_Cluster1562	0.0543	-0.043	0.4237	0.3933	0.0014	0.0265677	Both
pH	nifh_Cluster158	0.0144	0.0021	0.0145	-0.0373	0.6028	0.8329362	Both
pH	nifh_Cluster16	0.0019	-1.00E-04	0.0005	-0.0521	0.9223	0.978434	Both
pH	nifh_Cluster166	0.0172	0.0016	0.0026	-0.0499	0.8271	0.9457093	Both
pH	nifh_Cluster205	0.0122	0.0015	0.0148	-0.037	0.599	0.8323762	Both
pH	nifh_Cluster21	0.0021	0.0007	0.0426	-0.0078	0.3692	0.720084	Both
pH	nifh_Cluster222	0.006	0.0017	0.0252	-0.0261	0.4918	0.788532	Both
pH	nifh_Cluster225	0.0189	0.0035	0.0775	0.029	0.2216	0.5696876	Both
pH	nifh_Cluster230	0.0042	-0.0001	0.0007	-0.0519	0.9072	0.9698319	Both
pH	nifh_Cluster231	0.0022	0.0006	0.0253	-0.026	0.4914	0.788532	Both
pH	nifh_Cluster236	0.0027	0.0009	0.054	0.0042	0.3107	0.6787161	Both
pH	nifh_Cluster237	0.0058	-0.0008	0.0156	-0.0362	0.5894	0.8323762	Both
pH	nifh_Cluster243	0.0333	-0.0257	0.6132	0.5929	0.00E+00	0.0017704	Both
pH	nifh_Cluster2432	0.0436	-0.027	0.3796	0.3469	0.0029	0.041329	Both
pH	nifh_Cluster2433	0.0032	0.0005	0.0078	-0.0444	0.7031	0.8890351	Both
pH	nifh_Cluster246	0.0057	0.001	0.0155	-0.0363	0.5902	0.8323762	Both
pH	nifh_Cluster25	0.0295	-0.0131	0.3276	0.2923	0.0067	0.064748	Both
pH	nifh_Cluster256	0.0029	-0.001	0.064	0.0148	0.2684	0.6209334	Both
pH	nifh_Cluster264	0.0029	-0.0005	0.0178	-0.0339	0.5642	0.8293081	Both
pH	nifh_Cluster265	0.0736	-0.0165	0.2115	0.17	0.0359	0.1984029	Both
pH	nifh_Cluster267	0.0109	-0.0065	0.2635	0.2248	0.0173	0.1211536	Both
pH	nifh_Cluster268	0.0111	-0.0006	0.0046	-0.0478	0.7693	0.9213413	Both
pH	nifh_Cluster269	0.023	-0.0002	1.00E-04	-0.0526	0.9704	0.9894061	Both
pH	nifh_Cluster272	0.0041	-0.0042	0.3636	0.3301	0.0038	0.0467399	Both
pH	nifh_Cluster274	0.0032	-0.0008	0.0066	-0.0457	0.7271	0.8999091	Both
pH	nifh_Cluster275	0.0036	0.0004	0.0065	-0.0458	0.7281	0.8999091	Both
pH	nifh_Cluster278	0.0077	-0.003	0.1164	0.0699	0.1301	0.4148751	Both
pH	nifh_Cluster279	0.006	-0.0008	0.0225	-0.0289	0.5162	0.7988743	Both
pH	nifh_Cluster28	0.002	-0.0006	0.0425	-0.0079	0.3702	0.720084	Both
pH	nifh_Cluster280	0.0031	0.0026	0.1814	0.1383	0.0542	0.246085	Both
pH	nifh_Cluster282	0.0052	0.0014	0.0635	0.0142	0.2705	0.623309	Both
pH	nifh_Cluster287	0.0022	0.0006	0.0248	-0.0265	0.4952	0.7918379	Both
pH	nifh_Cluster290	0.011	-0.0053	0.2302	0.1896	0.0278	0.1670722	Both
pH	nifh_Cluster297	0.0029	-0.0006	0.007	-0.0453	0.7182	0.8968256	Both
pH	nifh_Cluster299	0.0026	-0.0004	0.0059	-0.0465	0.7417	0.9001951	Both
pH	nifh_Cluster303	0.002	-0.0018	0.2571	0.218	0.019	0.1300438	Both
pH	nifh_Cluster304	0.0024	-0.0003	0.0089	-0.0433	0.6849	0.8861113	Both
pH	nifh_Cluster313	0.0103	0.0006	0.001	-0.0516	0.8928	0.9647029	Both
pH	nifh_Cluster314	0.0022	1.00E-04	0.0003	-0.0523	0.9387	0.9872607	Both

pH	nifh_Cluster317	0.0071	1.00E-04	0.00E+00	-0.0526	0.9781	0.9894061	Both
pH	nifh_Cluster32	0.0033	-0.0007	0.029	-0.0221	0.4603	0.7780893	Both
pH	nifh_Cluster320	12.7419	1.2816	0.1666	0.1228	0.0662	0.2850726	Both
pH	nifh_Cluster334	0.0063	0.0025	0.0418	-0.0086	0.3739	0.7210131	Both
pH	nifh_Cluster335	0.0075	0.0006	0.0014	-0.0512	0.8733	0.9594568	Both
pH	nifh_Cluster338	0.0123	0.0034	0.0884	0.0404	0.1906	0.5205424	Both
pH	nifh_Cluster340	0.007	-0.0002	1.00E-04	-0.0525	0.9678	0.9894061	Both
pH	nifh_Cluster341	0.0023	-0.0024	0.3566	0.3227	0.0043	0.0483533	Both
pH	nifh_Cluster35	0.0078	0.0033	0.209	0.1673	0.0372	0.2033126	Both
pH	nifh_Cluster351	0.0025	0.0009	0.0313	-0.0197	0.4428	0.773964	Both
pH	nifh_Cluster3574	0.1093	0.0229	0.1429	0.0978	0.0911	0.344651	Both
pH	nifh_Cluster3691	0.0174	0.0134	0.3518	0.3177	0.0046	0.0484538	Both
pH	nifh_Cluster37	0.0329	0.002	0.0029	-0.0495	0.8155	0.9431753	Both
pH	nifh_Cluster374	0.0021	0.0025	0.3872	0.355	0.0026	0.0382566	Both
pH	nifh_Cluster38	0.0009	-0.0005	0.0544	0.0047	0.3088	0.677216	Both
pH	nifh_Cluster382	0.0016	0.0002	0.0027	-0.0498	0.8236	0.9457093	Both
pH	nifh_Cluster384	0.0019	-0.0004	0.0156	-0.0362	0.5897	0.8323762	Both
pH	nifh_Cluster386	0.0035	0.0013	0.0402	-0.0103	0.3834	0.7297501	Both
pH	nifh_Cluster389	0.0031	-1.00E-04	0.0002	-0.0524	0.9545	0.9886888	Both
pH	nifh_Cluster394	0.002	0.0007	0.0194	-0.0323	0.5476	0.8136292	Both
pH	nifh_Cluster40	0.0012	0.0004	0.0303	-0.0207	0.4504	0.7764447	Both
pH	nifh_Cluster43	0.0108	-0.0031	0.1324	0.0867	0.105	0.3708651	Both
pH	nifh_Cluster434	0.0035	0.0013	0.0461	-0.0041	0.3499	0.720084	Both
pH	nifh_Cluster440	0.0133	0.0111	0.4104	0.3794	0.0018	0.0295958	Both
pH	nifh_Cluster462	0.0034	0.0022	0.1404	0.0951	0.0942	0.3518955	Both
pH	nifh_Cluster470	0.0152	0.0037	0.0754	0.0267	0.2285	0.5786003	Both
pH	nifh_Cluster49	0.0213	-0.0042	0.0154	-0.0365	0.5925	0.8323762	Both
pH	nifh_Cluster499	0.0026	0.00E+00	1.00E-04	-0.0526	0.9702	0.9894061	Both
pH	nifh_Cluster5	0.0256	0.0021	0.0273	-0.0239	0.4744	0.7864352	Both
pH	nifh_Cluster506	0.0048	0.0006	0.0099	-0.0422	0.6684	0.8791893	Both
pH	nifh_Cluster51	0.0028	0.0008	0.0224	-0.0291	0.5175	0.7988743	Both
pH	nifh_Cluster52	0.0336	-0.0012	0.0007	-0.0518	0.9062	0.9698319	Both
pH	nifh_Cluster525	0.0033	0.0022	0.0727	0.0239	0.2373	0.589368	Both
pH	nifh_Cluster531	0.0258	-0.0207	0.5562	0.5328	0.0001	0.0040928	Both
pH	nifh_Cluster539	0.0048	0.0017	0.023	-0.0284	0.5114	0.7988743	Both
pH	nifh_Cluster578	0.0481	0.0232	0.3952	0.3633	0.0023	0.0372398	Both
pH	nifh_Cluster58	0.3542	-0.2198	0.3887	0.3565	0.0025	0.0382566	Both
pH	nifh_Cluster60	0.0089	-0.0009	0.0062	-0.0461	0.7349	0.9001951	Both
pH	nifh_Cluster61	0.0025	-0.0005	0.0164	-0.0354	0.58	0.8323762	Both
pH	nifh_Cluster65	0.009	0.0012	0.0102	-0.0419	0.6628	0.8791893	Both
pH	nifh_Cluster686	0.0009	0.0003	0.0151	-0.0367	0.5953	0.8323762	Both
pH	nifh_Cluster69	0.009	-0.0021	0.0273	-0.0239	0.4745	0.7864352	Both
pH	nifh_Cluster70	0.0111	0.0033	0.0241	-0.0272	0.5014	0.7930841	Both
pH	nifh_Cluster717	0.003	-0.0011	0.0762	0.0276	0.2258	0.5741719	Both
pH	nifh_Cluster725	0.0058	-0.0043	0.412	0.381	0.0017	0.0295958	Both
pH	nifh_Cluster727	0.0039	0.0018	0.0958	0.0482	0.1722	0.4933162	Both
pH	nifh_Cluster73	0.0106	0.0011	0.0068	-0.0454	0.7218	0.8985037	Both
pH	nifh_Cluster74	0.0049	0.0003	0.001	-0.0516	0.8909	0.9645039	Both
pH	nifh_Cluster748	0.0081	0.0018	0.0642	0.015	0.2676	0.6209334	Both
pH	nifh_Cluster749	0.0071	-0.0002	0.0011	-0.0515	0.8872	0.9621953	Both
pH	nifh_Cluster755	0.0102	0.0091	0.4948	0.4682	0.0004	0.0116347	Both
pH	nifh_Cluster76	0.0029	-1.00E-04	0.0002	-0.0525	0.9562	0.9886888	Both
pH	nifh_Cluster79	0.0022	0.0002	0.0043	-0.0482	0.7787	0.9263187	Both
pH	nifh_Cluster81	0.0055	0.0017	0.0298	-0.0213	0.4544	0.7764447	Both
pH	nifh_Cluster82	0.0011	-0.0004	0.0306	-0.0204	0.4482	0.7764447	Both

pH	nifh_Cluster83	0.0069	0.0017	0.0298	-0.0213	0.4542	0.7764447	Both
pH	nifh_Cluster86	0.0135	0.008	0.3851	0.3528	0.0027	0.0386379	Both
pH	nifh_Cluster868	0.0099	-0.0012	0.021	-0.0305	0.5309	0.8024797	Both
pH	nifh_Cluster87	0.0024	1.00E-04	0.0003	-0.0523	0.9428	0.9879862	Both
pH	nifh_Cluster88	0.0039	0.0041	0.3611	0.3275	0.004	0.0467399	Both
pH	nifh_Cluster9	0.0078	-0.0009	0.0101	-0.042	0.6641	0.8791893	Both
pH	nifh_Cluster92	0.0051	0.0009	0.0087	-0.0434	0.6869	0.8867816	Both
pH	nifh_Cluster93	0.0036	0.0006	0.0132	-0.0387	0.6194	0.8479099	Both
pH	nifh_Cluster94	0.0047	0.0001	0.0002	-0.0524	0.9521	0.9886888	Both
pH	nifh_Cluster95	0.0121	0.0033	0.0341	-0.0167	0.4227	0.7696476	Both
pH	nifh_Cluster96	0.0123	0.0041	0.1177	0.0712	0.1279	0.4102515	Both
pH	nifh_Cluster97	0.018	-0.0068	0.1113	0.0645	0.1394	0.4332645	Both
pH	nifh_Cluster98	0.0059	0.0017	0.0735	0.0247	0.2347	0.5867482	Both
pH	nifh_Cluster99	0.0033	-1.00E-04	0.0002	-0.0525	0.9568	0.9886888	Both
pH	nirk_Cluster0	5.9009	0.7287	0.2368	0.1966	0.0253	0.1587359	Both
pH	nirk_Cluster1	0.1708	0.0636	0.2161	0.1748	0.0337	0.1907224	Both
pH	nirk_Cluster10	0.5713	0.1985	0.6033	0.5824	0.00E+00	0.0019092	Both
pH	nirk_Cluster101	0.0031	-0.0008	0.0254	-0.0259	0.4899	0.788532	Both
pH	nirk_Cluster102	0.0426	0.016	0.183	0.14	0.053	0.2435155	Both
pH	nirk_Cluster103	0.0754	0.0354	0.3752	0.3423	0.0032	0.0429105	Both
pH	nirk_Cluster104	0.0023	0.0016	0.1484	0.1036	0.0846	0.3282342	Both
pH	nirk_Cluster105	0.0946	0.0337	0.2232	0.1823	0.0306	0.1804626	Both
pH	nirk_Cluster106	0.0592	0.0255	0.187	0.1443	0.0502	0.2408726	Both
pH	nirk_Cluster107	0.0891	0.034	0.2243	0.1835	0.0301	0.1793166	Both
pH	nirk_Cluster108	0.0151	0.0041	0.0676	0.0185	0.255	0.6038577	Both
pH	nirk_Cluster109	0.0115	0.0065	0.2556	0.2164	0.0194	0.1300438	Both
pH	nirk_Cluster11	0.1401	0.012	0.1191	0.0727	0.1255	0.4090249	Both
pH	nirk_Cluster12	0.094	0.0065	0.0201	-0.0314	0.5396	0.8080331	Both
pH	nirk_Cluster13	0.0408	0.0001	1.00E-04	-0.0526	0.9734	0.9894061	Both
pH	nirk_Cluster14	0.0946	0.0396	0.7124	0.6973	0.00E+00	0.0001598	Both
pH	nirk_Cluster15	0.3358	0.1289	0.2663	0.2277	0.0166	0.1182783	Both
pH	nirk_Cluster16	0.4727	0.1616	0.1939	0.1514	0.0458	0.2301907	Both
pH	nirk_Cluster17	0.0998	0.0018	0.0024	-0.0501	0.8325	0.9462588	Both
pH	nirk_Cluster18	0.1296	0.0139	0.0222	-0.0293	0.5196	0.7988743	Both
pH	nirk_Cluster19	0.2776	0.0214	0.0653	0.0161	0.2637	0.6149235	Both
pH	nirk_Cluster2	0.2032	0.0238	0.1191	0.0727	0.1255	0.4090249	Both
pH	nirk_Cluster20	0.1738	0.036	0.1564	0.112	0.076	0.3050575	Both
pH	nirk_Cluster21	0.1532	0.0266	0.2465	0.2068	0.0221	0.1430231	Both
pH	nirk_Cluster22	0.1291	0.03	0.2372	0.197	0.0251	0.1587359	Both
pH	nirk_Cluster23	0.0673	0.0091	0.1786	0.1354	0.0563	0.2515921	Both
pH	nirk_Cluster24	0.0654	0.0322	0.7898	0.7787	0.00E+00	2.17E-05	Both
pH	nirk_Cluster25	0.4782	0.1281	0.3184	0.2825	0.0077	0.0682074	Both
pH	nirk_Cluster26	0.105	0.0124	0.1076	0.0606	0.1466	0.4481866	Both
pH	nirk_Cluster27	0.1727	0.0562	0.3483	0.314	0.0049	0.0502983	Both
pH	nirk_Cluster28	0.0685	0.0256	0.4669	0.4389	0.0006	0.0157099	Both
pH	nirk_Cluster29	0.1526	0.0315	0.2856	0.248	0.0126	0.0980567	Both
pH	nirk_Cluster3	0.3023	0.0918	0.5839	0.562	1.00E-04	0.0027181	Both
pH	nirk_Cluster30	0.0556	0.024	0.3065	0.27	0.0092	0.0766472	Both
pH	nirk_Cluster31	0.1501	-0.0091	0.0455	-0.0047	0.3531	0.720084	Both
pH	nirk_Cluster32	0.1254	0.0339	0.0972	0.0496	0.169	0.4872797	Both
pH	nirk_Cluster33	0.0638	0.0227	0.1515	0.1068	0.0812	0.3172445	Both
pH	nirk_Cluster34	0.0416	0.01	0.1294	0.0836	0.1092	0.3744819	Both
pH	nirk_Cluster35	0.1007	0.0267	0.0832	0.0349	0.2048	0.5393988	Both
pH	nirk_Cluster36	0.0017	0.0012	0.1329	0.0873	0.1042	0.3708651	Both
pH	nirk_Cluster39	0.0035	0.0027	0.0985	0.0511	0.1659	0.485744	Both

pH	nirk_Cluster4	0.1323	0.0329	0.2475	0.2079	0.0218	0.142614	Both
pH	nirk_Cluster40	0.0455	0.0159	0.3529	0.3189	0.0045	0.0484538	Both
pH	nirk_Cluster41	0.1562	0.0427	0.4178	0.3871	0.0015	0.0277409	Both
pH	nirk_Cluster44	0.0669	0.0385	0.4562	0.4275	0.0008	0.0183172	Both
pH	nirk_Cluster46	0.0154	0.0088	0.2311	0.1906	0.0274	0.1666317	Both
pH	nirk_Cluster48	0.003	0.0009	0.0456	-0.0047	0.3529	0.720084	Both
pH	nirk_Cluster49	0.0057	0.0004	0.0017	-0.0508	0.8585	0.951334	Both
pH	nirk_Cluster5	0.1932	0.029	0.1747	0.1312	0.0594	0.2635295	Both
pH	nirk_Cluster50	0.0016	-0.0001	0.0028	-0.0497	0.8212	0.9457093	Both
pH	nirk_Cluster52	0.0452	0.0166	0.3021	0.2653	0.0099	0.079666	Both
pH	nirk_Cluster53	0.0038	0.00E+00	0.00E+00	-0.0526	0.9846	0.991335	Both
pH	nirk_Cluster54	0.002	0.0005	0.0252	-0.0261	0.4918	0.788532	Both
pH	nirk_Cluster55	0.0023	-0.0002	0.0017	-0.0509	0.861	0.951334	Both
pH	nirk_Cluster56	0.0009	-0.0003	0.0216	-0.0299	0.5246	0.7997678	Both
pH	nirk_Cluster57	0.0016	-0.0007	0.0373	-0.0133	0.4014	0.7517816	Both
pH	nirk_Cluster58	0.0088	0.006	0.3605	0.3269	0.004	0.0467399	Both
pH	nirk_Cluster59	0.0058	0.0047	0.4543	0.4256	0.0008	0.0183172	Both
pH	nirk_Cluster6	0.1558	0.058	0.6951	0.679	0.00E+00	0.0002252	Both
pH	nirk_Cluster60	0.0006	0.0003	0.0424	-0.008	0.3708	0.720084	Both
pH	nirk_Cluster62	0.1417	0.0669	0.313	0.2769	0.0084	0.0704813	Both
pH	nirk_Cluster63	0.1284	0.06	0.4726	0.4448	0.0006	0.0147415	Both
pH	nirk_Cluster64	0.0044	0.0009	0.0312	-0.0197	0.4434	0.773964	Both
pH	nirk_Cluster65	0.0464	0.0125	0.1901	0.1475	0.0482	0.2328708	Both
pH	nirk_Cluster66	0.0068	0.001	0.0167	-0.0351	0.5772	0.8323762	Both
pH	nirk_Cluster67	0.0174	0.0144	0.5063	0.4803	0.0003	0.0097612	Both
pH	nirk_Cluster68	0.0125	0.0067	0.277	0.2389	0.0143	0.1078455	Both
pH	nirk_Cluster69	0.0305	0.0102	0.2313	0.1909	0.0273	0.1666317	Both
pH	nirk_Cluster7	0.0804	0.0319	0.4791	0.4517	0.0005	0.0136092	Both
pH	nirk_Cluster70	0.0721	0.0363	0.1918	0.1493	0.0471	0.2303769	Both
pH	nirk_Cluster71	0.0053	0.002	0.0903	0.0425	0.1855	0.5115258	Both
pH	nirk_Cluster72	0.0239	0.0094	0.3028	0.2661	0.0097	0.079666	Both
pH	nirk_Cluster73	0.1444	0.0364	0.1061	0.059	0.1497	0.4540422	Both
pH	nirk_Cluster74	0.6398	0.3073	0.3522	0.3181	0.0046	0.0484538	Both
pH	nirk_Cluster75	0.0147	0.0016	0.0063	-0.046	0.7327	0.9001951	Both
pH	nirk_Cluster76	0.1055	0.0418	0.363	0.3295	0.0038	0.0467399	Both
pH	nirk_Cluster77	0.0081	0.0098	0.3159	0.2799	0.008	0.0695072	Both
pH	nirk_Cluster78	0.2407	0.0989	0.3307	0.2955	0.0064	0.0627918	Both
pH	nirk_Cluster79	0.0069	0.0073	0.3599	0.3262	0.004	0.0467399	Both
pH	nirk_Cluster8	0.0982	0.0231	0.1184	0.072	0.1267	0.409936	Both
pH	nirk_Cluster80	0.2665	0.0563	0.0716	0.0227	0.2409	0.5897383	Both
pH	nirk_Cluster82	0.0015	0.0008	0.0416	-0.0089	0.3753	0.7212441	Both
pH	nirk_Cluster83	0.004	0.0021	0.1839	0.141	0.0524	0.2435155	Both
pH	nirk_Cluster84	0.0207	0.0039	0.0269	-0.0244	0.4778	0.7879127	Both
pH	nirk_Cluster85	0.004	0.002	0.1296	0.0838	0.1089	0.3744819	Both
pH	nirk_Cluster86	0.0286	0.0124	0.2038	0.1619	0.0399	0.2141416	Both
pH	nirk_Cluster87	0.0115	0.0029	0.0582	0.0086	0.2923	0.6583688	Both
pH	nirk_Cluster88	0.0032	0.0011	0.0679	0.0189	0.2538	0.6038577	Both
pH	nirk_Cluster89	0.0065	0.0005	0.0035	-0.0489	0.7987	0.9424372	Both
pH	nirk_Cluster9	0.0517	0.0127	0.1059	0.0588	0.1501	0.4540422	Both
pH	nirk_Cluster90	0.0067	0.0029	0.0851	0.037	0.1994	0.5323225	Both
pH	nirk_Cluster91	0.0374	0.0132	0.2213	0.1803	0.0314	0.1834223	Both
pH	nirk_Cluster92	0.0072	0.0035	0.1249	0.0788	0.1161	0.3913888	Both
pH	nirk_Cluster93	0.0308	0.0016	0.0046	-0.0478	0.7704	0.9213413	Both
pH	nirk_Cluster94	0.0175	0.0068	0.1653	0.1214	0.0674	0.2880322	Both
pH	nirk_Cluster95	0.0473	0.0235	0.322	0.2863	0.0073	0.0682074	Both

pH	nirk_Cluster96	0.0472	0.0216	0.3421	0.3075	0.0054	0.0544413	Both
pH	nirk_Cluster97	0.0306	0.0065	0.0738	0.025	0.2337	0.5866636	Both
pH	nirk_Cluster98	0.0181	0.0121	0.3183	0.2825	0.0077	0.0682074	Both
pH	nirk_Cluster99	0.006	0.0036	0.1968	0.1545	0.044	0.2235919	Both
pH	nirs_Cluster0	0.1409	0.0577	0.7173	0.7024	0.00E+00	0.0001598	Both
pH	nirs_Cluster1	0.004	0.0017	0.0551	0.0054	0.3057	0.6755411	Both
pH	nirs_Cluster14	0.0107	0.0047	0.0434	-0.007	0.365	0.720084	Both
pH	nirs_Cluster2	0.0139	0.0133	0.3367	0.3018	0.0058	0.0581843	Both
pH	nirs_Cluster26	0.0005	0.0002	0.0147	-0.0371	0.6004	0.8323762	Both
pH	nirs_Cluster28	0.0071	0.0023	0.0212	-0.0303	0.5284	0.8024797	Both
pH	nirs_Cluster3	0.1715	0.0332	0.3253	0.2898	0.0069	0.0660334	Both
pH	nirs_Cluster36	0.0013	0.002	0.1641	0.1201	0.0685	0.2887862	Both
pH	nirs_Cluster37	0.0066	0.0048	0.0608	0.0114	0.2811	0.6406203	Both
pH	nirs_Cluster4	0.0002	0.0002	0.0508	0.0008	0.3261	0.6910437	Both
pH	nirs_Cluster50	0.002	-0.0001	0.0014	-0.0511	0.8714	0.9594568	Both
pH	nirs_Cluster9	0.0269	-0.013	0.0481	-0.002	0.3395	0.7083557	Both
pH	norb_Cluster0	0.0775	0.0149	0.0605	0.0111	0.2823	0.6406203	Both
pH	norb_Cluster1	1.1042	0.3702	0.1347	0.0891	0.1018	0.3680838	Both
pH	norb_Cluster101	0.0026	-0.001	0.0334	-0.0175	0.428	0.7702922	Both
pH	norb_Cluster102	0.0038	0.0007	0.0175	-0.0342	0.5672	0.8293081	Both
pH	norb_Cluster103	0.0018	0.0004	0.0099	-0.0422	0.6681	0.8791893	Both
pH	norb_Cluster104	0.0023	0.001	0.0425	-0.0079	0.3699	0.720084	Both
pH	norb_Cluster106	0.125	0.0158	0.0275	-0.0237	0.4729	0.7864352	Both
pH	norb_Cluster107	0.0133	0.0024	0.0253	-0.026	0.4914	0.788532	Both
pH	norb_Cluster108	0.0052	0.003	0.2359	0.1957	0.0256	0.1590899	Both
pH	norb_Cluster109	0.0227	0.0003	0.0002	-0.0524	0.952	0.9886888	Both
pH	norb_Cluster11	0.0126	-0.0011	0.0055	-0.0468	0.7483	0.9010271	Both
pH	norb_Cluster110	0.0099	0.0027	0.048	-0.0021	0.3398	0.7083557	Both
pH	norb_Cluster112	0.0171	0.0019	0.0156	-0.0362	0.5895	0.8323762	Both
pH	norb_Cluster113	0.0134	0.0004	0.0005	-0.0521	0.9207	0.978434	Both
pH	norb_Cluster114	0.0025	0.0015	0.0538	0.004	0.3117	0.6787161	Both
pH	norb_Cluster115	0.0137	-0.004	0.0066	-0.0456	0.7254	0.8999091	Both
pH	norb_Cluster116	0.0072	0.0025	0.0546	0.0049	0.3079	0.677216	Both
pH	norb_Cluster117	0.0027	0.0006	0.0192	-0.0324	0.5489	0.8136292	Both
pH	norb_Cluster118	0.0037	0.0009	0.0294	-0.0217	0.4572	0.7772924	Both
pH	norb_Cluster119	0.019	0.0028	0.0135	-0.0384	0.6163	0.8476224	Both
pH	norb_Cluster12	0.0047	0.0014	0.0333	-0.0175	0.4282	0.7702922	Both
pH	norb_Cluster121	0.0017	-0.0005	0.0155	-0.0363	0.5905	0.8323762	Both
pH	norb_Cluster122	0.0144	0.0017	0.0124	-0.0396	0.6315	0.8584772	Both
pH	norb_Cluster123	0.0053	0.0014	0.0333	-0.0176	0.4288	0.7702922	Both
pH	norb_Cluster124	0.682	0.1304	0.0555	0.0058	0.3039	0.6741522	Both
pH	norb_Cluster125	0.004	0.00E+00	0.00E+00	-0.0526	0.9879	0.9929606	Both
pH	norb_Cluster126	0.0082	0.0005	0.0023	-0.0502	0.8364	0.9462588	Both
pH	norb_Cluster127	0.1735	0.044	0.0838	0.0356	0.2032	0.5393988	Both
pH	norb_Cluster128	0.0115	0.0032	0.0871	0.0391	0.1939	0.5248653	Both
pH	norb_Cluster13	0.0045	-0.0008	0.0098	-0.0423	0.6693	0.8791893	Both
pH	norb_Cluster131	0.0184	-0.0027	0.0217	-0.0298	0.5243	0.7997678	Both
pH	norb_Cluster132	1.0357	0.3054	0.1833	0.1403	0.0528	0.2435155	Both
pH	norb_Cluster133	0.0018	0.0017	0.2736	0.2353	0.015	0.111834	Both
pH	norb_Cluster135	0.0567	0.0177	0.128	0.0821	0.1113	0.3775049	Both
pH	norb_Cluster136	0.0213	0.0051	0.0671	0.018	0.2569	0.6038577	Both
pH	norb_Cluster139	0.0328	0.0119	0.1827	0.1397	0.0532	0.2435155	Both
pH	norb_Cluster14	0.0026	-0.0004	0.0093	-0.0428	0.6774	0.8811452	Both
pH	norb_Cluster140	0.0437	0.0113	0.088	0.0399	0.1917	0.5213431	Both
pH	norb_Cluster142	0.0015	-0.0002	0.0029	-0.0496	0.8168	0.9431753	Both

pH	norb_Cluster144	0.0098	0.0043	0.1648	0.1208	0.0679	0.2880322	Both
pH	norb_Cluster146	0.0348	0.015	0.2836	0.2459	0.0129	0.0992017	Both
pH	norb_Cluster15	0.0469	0.0132	0.092	0.0442	0.1814	0.5056429	Both
pH	norb_Cluster150	0.0157	0.0082	0.3726	0.3396	0.0033	0.0431919	Both
pH	norb_Cluster155	0.1631	0.0368	0.0852	0.0371	0.1991	0.5323225	Both
pH	norb_Cluster16	0.0131	-0.0022	0.0162	-0.0356	0.5822	0.8323762	Both
pH	norb_Cluster164	0.0199	0.0009	0.0017	-0.0508	0.8594	0.951334	Both
pH	norb_Cluster165	0.0076	0.0011	0.0089	-0.0432	0.6839	0.8861113	Both
pH	norb_Cluster166	0.0039	-0.0003	0.0032	-0.0493	0.808	0.9431753	Both
pH	norb_Cluster169	0.0238	0.0012	0.0019	-0.0507	0.8531	0.951334	Both
pH	norb_Cluster17	0.0028	-1.00E-04	0.0004	-0.0522	0.9317	0.9833744	Both
pH	norb_Cluster170	0.0243	0.0017	0.0031	-0.0494	0.8111	0.9431753	Both
pH	norb_Cluster171	0.0069	-0.0026	0.0506	0.0007	0.3268	0.6910437	Both
pH	norb_Cluster172	0.014	0.0011	0.0034	-0.0491	0.8031	0.9431753	Both
pH	norb_Cluster175	0.0458	0.0032	0.003	-0.0494	0.8126	0.9431753	Both
pH	norb_Cluster176	0.0298	-0.002	0.0029	-0.0496	0.8167	0.9431753	Both
pH	norb_Cluster177	0.0282	0.0031	0.0076	-0.0446	0.7063	0.8890351	Both
pH	norb_Cluster179	0.0022	0.0019	0.1835	0.1405	0.0527	0.2435155	Both
pH	norb_Cluster18	0.0037	0.0003	0.005	-0.0473	0.76	0.9131822	Both
pH	norb_Cluster180	0.0109	0.0031	0.0919	0.0441	0.1815	0.5056429	Both
pH	norb_Cluster181	0.0008	-0.0001	0.0023	-0.0502	0.8354	0.9462588	Both
pH	norb_Cluster182	0.0013	-0.0001	0.0021	-0.0504	0.8436	0.9481017	Both
pH	norb_Cluster184	0.0146	0.0018	0.0126	-0.0393	0.6278	0.8554233	Both
pH	norb_Cluster186	0.0011	0.0004	0.0239	-0.0275	0.5039	0.7948443	Both
pH	norb_Cluster19	0.0049	0.0008	0.0206	-0.031	0.535	0.8031505	Both
pH	norb_Cluster192	0.0173	0.0005	0.0007	-0.0519	0.9117	0.9727119	Both
pH	norb_Cluster193	0.0021	-0.0003	0.008	-0.0443	0.7006	0.8890351	Both
pH	norb_Cluster197	0.0303	0.0072	0.0084	-0.0438	0.6922	0.8890351	Both
pH	norb_Cluster2	0.0031	-0.0013	0.0607	0.0113	0.2817	0.6406203	Both
pH	norb_Cluster20	0.0587	0.0152	0.1016	0.0543	0.1591	0.4739799	Both
pH	norb_Cluster208	0.0011	-0.0002	0.0059	-0.0465	0.7416	0.9001951	Both
pH	norb_Cluster21	0.0481	0.0187	0.1701	0.1264	0.0632	0.2767029	Both
pH	norb_Cluster211	0.0104	0.0047	0.0392	-0.0114	0.3898	0.7395056	Both
pH	norb_Cluster212	0.0264	-0.0023	0.0024	-0.0501	0.8335	0.9462588	Both
pH	norb_Cluster214	0.0119	0.0045	0.0443	-0.006	0.3599	0.720084	Both
pH	norb_Cluster217	0.0149	0.0014	0.0029	-0.0496	0.8169	0.9431753	Both
pH	norb_Cluster219	0.0083	0.0023	0.0193	-0.0323	0.5484	0.8136292	Both
pH	norb_Cluster22	0.0025	0.0021	0.1628	0.1187	0.0697	0.2909463	Both
pH	norb_Cluster220	0.0177	0.0058	0.0228	-0.0286	0.5132	0.7988743	Both
pH	norb_Cluster221	0.0177	0.0026	0.004	-0.0484	0.7843	0.9291854	Both
pH	norb_Cluster222	0.0074	0.0021	0.0108	-0.0413	0.6543	0.8753536	Both
pH	norb_Cluster224	0.0194	0.0009	0.0007	-0.0519	0.9074	0.9698319	Both
pH	norb_Cluster225	0.0097	0.0013	0.0063	-0.046	0.7316	0.9001951	Both
pH	norb_Cluster226	0.0043	-0.0004	0.003	-0.0495	0.8145	0.9431753	Both
pH	norb_Cluster228	0.0031	-0.0018	0.0244	-0.027	0.4992	0.7930841	Both
pH	norb_Cluster229	0.0034	-1.00E-04	0.0002	-0.0524	0.9508	0.9886888	Both
pH	norb_Cluster230	0.0014	0.00E+00	1.00E-04	-0.0525	0.9674	0.9894061	Both
pH	norb_Cluster231	0.0044	0.0033	0.1407	0.0955	0.0938	0.3518955	Both
pH	norb_Cluster232	0.0031	0.0027	0.2193	0.1782	0.0323	0.1848549	Both
pH	norb_Cluster234	0.0121	-0.0025	0.0176	-0.0341	0.5661	0.8293081	Both
pH	norb_Cluster235	0.0009	0.0002	0.011	-0.0411	0.6513	0.8753536	Both
pH	norb_Cluster238	0.0099	-0.0009	0.0026	-0.0499	0.8256	0.9457093	Both
pH	norb_Cluster239	0.0081	-0.0023	0.021	-0.0306	0.5313	0.8024797	Both
pH	norb_Cluster24	0.2637	0.0795	0.1374	0.092	0.0981	0.3618488	Both
pH	norb_Cluster244	0.0011	0.0004	0.0221	-0.0293	0.5199	0.7988743	Both

pH	norb_Cluster247	0.0048	0.0031	0.3181	0.2822	0.0077	0.0682074	Both
pH	norb_Cluster25	0.1114	0.0304	0.0919	0.0441	0.1817	0.5056429	Both
pH	norb_Cluster250	0.0115	0.0023	0.0287	-0.0224	0.4627	0.7799015	Both
pH	norb_Cluster251	0.0052	0.00E+00	0.00E+00	-0.0526	0.9991	0.9990919	Both
pH	norb_Cluster26	0.0931	0.0321	0.1226	0.0764	0.1197	0.3967927	Both
pH	norb_Cluster28	0.0302	0.0026	0.0076	-0.0446	0.7067	0.8890351	Both
pH	norb_Cluster29	0.222	0.0197	0.0299	-0.0211	0.4534	0.7764447	Both
pH	norb_Cluster31	0.0531	0.0225	0.2114	0.1699	0.036	0.1984029	Both
pH	norb_Cluster32	0.1193	0.0453	0.1313	0.0856	0.1064	0.373786	Both
pH	norb_Cluster33	0.4946	-0.0809	0.1002	0.0529	0.162	0.4803185	Both
pH	norb_Cluster35	0.0628	0.0163	0.0834	0.0352	0.2041	0.5393988	Both
pH	norb_Cluster36	1.1345	0.0837	0.0105	-0.0415	0.6579	0.8781598	Both
pH	norb_Cluster37	0.7473	0.0005	0.00E+00	-0.0526	0.9963	0.9979747	Both
pH	norb_Cluster38	0.0116	0.0041	0.1625	0.1184	0.07	0.2909463	Both
pH	norb_Cluster39	0.0426	0.0103	0.0687	0.0196	0.2512	0.6038577	Both
pH	norb_Cluster40	0.186	0.0592	0.1364	0.091	0.0994	0.3642602	Both
pH	norb_Cluster42	0.2864	0.0112	0.0032	-0.0492	0.807	0.9431753	Both
pH	norb_Cluster44	0.0182	0.0052	0.0524	0.0025	0.3182	0.6849676	Both
pH	norb_Cluster46	0.2556	0.038	0.0318	-0.0191	0.439	0.773964	Both
pH	norb_Cluster47	0.555	0.1508	0.1196	0.0732	0.1247	0.4090249	Both
pH	norb_Cluster5	0.01	0.0024	0.0774	0.0288	0.2221	0.5696876	Both
pH	norb_Cluster51	0.0092	-0.0015	0.0097	-0.0424	0.6706	0.8791893	Both
pH	norb_Cluster52	0.2391	0.0773	0.1345	0.0889	0.102	0.3680838	Both
pH	norb_Cluster56	0.014	0.0044	0.1018	0.0545	0.1587	0.4739799	Both
pH	norb_Cluster57	0.0034	0.0009	0.0446	-0.0057	0.3582	0.720084	Both
pH	norb_Cluster59	0.0236	0.0088	0.0684	0.0194	0.2522	0.6038577	Both
pH	norb_Cluster6	0.6862	0.051	0.0114	-0.0407	0.6457	0.8725829	Both
pH	norb_Cluster60	0.3199	0.0948	0.0696	0.0206	0.2479	0.6038577	Both
pH	norb_Cluster61	0.482	-0.0199	0.0023	-0.0502	0.8372	0.9462588	Both
pH	norb_Cluster62	0.0024	-0.0003	0.0081	-0.0441	0.6977	0.8890351	Both
pH	norb_Cluster64	0.0326	0.0091	0.1181	0.0717	0.1271	0.409936	Both
pH	norb_Cluster66	0.0012	0.0009	0.1144	0.0678	0.1338	0.4243161	Both
pH	norb_Cluster67	0.0218	0.009	0.1682	0.1245	0.0648	0.2810465	Both
pH	norb_Cluster68	0.0034	-0.0005	0.0193	-0.0323	0.5481	0.8136292	Both
pH	norb_Cluster69	0.0028	0.002	0.1984	0.1562	0.043	0.2233132	Both
pH	norb_Cluster7	0.0709	0.0154	0.0672	0.0181	0.2566	0.6038577	Both
pH	norb_Cluster70	0.1051	0.0346	0.1699	0.1262	0.0633	0.2767029	Both
pH	norb_Cluster71	0.1295	0.0178	0.0358	-0.015	0.4114	0.7585588	Both
pH	norb_Cluster74	0.0327	0.0082	0.0985	0.0511	0.1658	0.485744	Both
pH	norb_Cluster75	0.1291	0.0263	0.0981	0.0506	0.1669	0.485744	Both
pH	norb_Cluster76	0.1105	0.0373	0.0679	0.0189	0.2538	0.6038577	Both
pH	norb_Cluster77	0.0475	0.0099	0.1127	0.066	0.1368	0.4294357	Both
pH	norb_Cluster8	0.018	0.0042	0.0525	0.0026	0.318	0.6849676	Both
pH	norb_Cluster80	0.0015	-0.0004	0.0177	-0.034	0.5655	0.8293081	Both
pH	norb_Cluster81	0.0012	0.00E+00	0.00E+00	-0.0526	0.9947	0.9979747	Both
pH	norb_Cluster83	0.0078	-0.0027	0.0266	-0.0246	0.4799	0.788532	Both
pH	norb_Cluster84	0.0082	0.002	0.0456	-0.0046	0.3525	0.720084	Both
pH	norb_Cluster85	0.0023	0.0008	0.0426	-0.0078	0.3695	0.720084	Both
pH	norb_Cluster86	0.031	0.01	0.0297	-0.0214	0.4553	0.7764447	Both
pH	norb_Cluster88	0.0016	0.0001	0.0012	-0.0513	0.88	0.9615067	Both
pH	norb_Cluster90	0.0009	-0.0003	0.0059	-0.0464	0.7409	0.9001951	Both
pH	norb_Cluster91	0.6481	-0.0129	0.0005	-0.0521	0.9254	0.978434	Both
pH	norb_Cluster92	0.0015	0.001	0.056	0.0063	0.3017	0.6732025	Both
pH	norb_Cluster93	0.0022	0.001	0.0454	-0.0048	0.3537	0.720084	Both
pH	norb_Cluster94	0.0017	0.0008	0.072	0.0231	0.2396	0.5897383	Both

pH	norb_Cluster96	0.0007	-0.0001	0.0057	-0.0466	0.7441	0.9001951	Both
pH	norb_Cluster97	0.0119	0.0082	0.2697	0.2313	0.0158	0.1167691	Both
pH	norb_Cluster98	0.0132	0.0037	0.0367	-0.014	0.4057	0.7550295	Both
pH	nosz_Cluster0	0.0045	0.0018	0.098	0.0505	0.1671	0.485744	Both
pH	nosz_Cluster10	0.0136	-0.0014	0.0065	-0.0458	0.7291	0.8999091	Both
pH	nosz_Cluster102	0.0044	0.0018	0.1564	0.112	0.076	0.3050575	Both
pH	nosz_Cluster103	0.0455	-0.0064	0.036	-0.0147	0.41	0.7583601	Both
pH	nosz_Cluster105	0.0016	0.0008	0.0781	0.0295	0.22	0.5692041	Both
pH	nosz_Cluster106	0.0073	-0.0015	0.0283	-0.0229	0.4663	0.783827	Both
pH	nosz_Cluster107	0.0002	0.0002	0.0508	0.0008	0.3261	0.6910437	Both
pH	nosz_Cluster108	0.004	-0.0005	0.0077	-0.0445	0.7045	0.8890351	Both
pH	nosz_Cluster109	0.0375	0.0048	0.0433	-0.0071	0.3655	0.720084	Both
pH	nosz_Cluster11	0.0129	0.0024	0.0093	-0.0429	0.678	0.8811452	Both
pH	nosz_Cluster110	0.0003	-0.0004	0.1244	0.0783	0.1169	0.3917851	Both
pH	nosz_Cluster111	0.0061	-0.0006	0.007	-0.0453	0.719	0.8968256	Both
pH	nosz_Cluster116	0.0067	0.001	0.0162	-0.0356	0.5828	0.8323762	Both
pH	nosz_Cluster118	0.0045	0.0019	0.0581	0.0086	0.2924	0.6583688	Both
pH	nosz_Cluster119	0.0013	-0.0006	0.0423	-0.0081	0.371	0.720084	Both
pH	nosz_Cluster120	0.0071	-0.0014	0.0324	-0.0185	0.435	0.7706898	Both
pH	nosz_Cluster123	0.0097	-0.0008	0.0034	-0.0491	0.8025	0.9431753	Both
pH	nosz_Cluster128	0.0047	0.0004	0.0025	-0.05	0.8309	0.9462588	Both
pH	nosz_Cluster129	0.0494	-0.0078	0.1103	0.0635	0.1413	0.4365894	Both
pH	nosz_Cluster13	0.0115	0.0067	0.2546	0.2154	0.0197	0.1303853	Both
pH	nosz_Cluster130	0.089	0.0314	0.0263	-0.025	0.4828	0.788532	Both
pH	nosz_Cluster132	0.0037	0.00E+00	1.00E-04	-0.0526	0.97	0.9894061	Both
pH	nosz_Cluster14	0.024	-0.0004	0.00E+00	-0.0526	0.9778	0.9894061	Both
pH	nosz_Cluster16	0.0076	0.0016	0.0155	-0.0364	0.5914	0.8323762	Both
pH	nosz_Cluster17	0.0364	0.0032	0.0108	-0.0412	0.6535	0.8753536	Both
pH	nosz_Cluster18	0.0161	0.0047	0.0429	-0.0074	0.3675	0.720084	Both
pH	nosz_Cluster20	0.0091	0.0011	0.0059	-0.0464	0.7397	0.9001951	Both
pH	nosz_Cluster21	0.0083	-1.00E-04	0.00E+00	-0.0526	0.9843	0.991335	Both
pH	nosz_Cluster22	0.0267	0.0107	0.0674	0.0183	0.2557	0.6038577	Both
pH	nosz_Cluster23	0.0141	0.0006	0.0011	-0.0515	0.8866	0.9621953	Both
pH	nosz_Cluster24	0.0013	0.0016	0.2629	0.2242	0.0175	0.1211536	Both
pH	nosz_Cluster25	0.0029	0.0011	0.0336	-0.0173	0.4266	0.7702922	Both
pH	nosz_Cluster26	0.0054	0.0015	0.0297	-0.0213	0.4549	0.7764447	Both
pH	nosz_Cluster27	0.0053	0.0018	0.0609	0.0114	0.281	0.6406203	Both
pH	nosz_Cluster28	0.0303	0.0088	0.0816	0.0333	0.2093	0.5488322	Both
pH	nosz_Cluster30	0.0276	0.008	0.0784	0.0299	0.219	0.5691642	Both
pH	nosz_Cluster32	0.0137	0.0046	0.092	0.0442	0.1814	0.5056429	Both
pH	nosz_Cluster33	0.018	0.0028	0.0109	-0.0411	0.6523	0.8753536	Both
pH	nosz_Cluster34	0.0377	-0.0022	0.0018	-0.0507	0.8552	0.951334	Both
pH	nosz_Cluster35	0.0075	0.0028	0.0864	0.0383	0.1959	0.5278443	Both
pH	nosz_Cluster36	0.0179	0.0044	0.0439	-0.0064	0.3618	0.720084	Both
pH	nosz_Cluster37	0.006	0.0031	0.1443	0.0993	0.0894	0.3402427	Both
pH	nosz_Cluster38	0.0128	-0.0008	0.002	-0.0505	0.8479	0.9492878	Both
pH	nosz_Cluster39	0.0537	0.0141	0.0717	0.0229	0.2404	0.5897383	Both
pH	nosz_Cluster40	0.0218	0.0079	0.0923	0.0445	0.1806	0.5056429	Both
pH	nosz_Cluster41	0.0203	0.0089	0.1302	0.0844	0.1081	0.3744819	Both
pH	nosz_Cluster42	0.0279	0.004	0.0147	-0.0372	0.601	0.8323762	Both
pH	nosz_Cluster43	0.0007	-0.0004	0.0245	-0.0269	0.4983	0.7930841	Both
pH	nosz_Cluster44	0.0057	0.0009	0.0151	-0.0367	0.5952	0.8323762	Both
pH	nosz_Cluster45	0.0045	0.0011	0.0292	-0.0219	0.4587	0.7777434	Both
pH	nosz_Cluster46	0.007	0.0022	0.0941	0.0464	0.1762	0.5022107	Both
pH	nosz_Cluster47	0.0008	0.0002	0.0113	-0.0407	0.6463	0.8725829	Both

pH	nosz_Cluster48	0.0023	-0.0002	0.0017	-0.0509	0.8602	0.951334	Both
pH	nosz_Cluster49	0.0014	0.0001	0.0026	-0.0499	0.8271	0.9457093	Both
pH	nosz_Cluster5	0.0045	0.0002	0.0003	-0.0523	0.9412	0.9879862	Both
pH	nosz_Cluster51	0.0021	0.0007	0.0347	-0.0161	0.419	0.7693843	Both
pH	nosz_Cluster53	0.0009	0.001	0.1326	0.0869	0.1047	0.3708651	Both
pH	nosz_Cluster54	0.0026	-0.0008	0.0243	-0.0271	0.5001	0.7930841	Both
pH	nosz_Cluster57	0.1757	0.0802	0.1997	0.1575	0.0423	0.2227413	Both
pH	nosz_Cluster58	0.0043	-0.0007	0.0102	-0.0419	0.663	0.8791893	Both
pH	nosz_Cluster59	0.0009	-0.0004	0.0219	-0.0295	0.5217	0.7995151	Both
pH	nosz_Cluster6	0.0032	0.0011	0.0409	-0.0096	0.3795	0.7259115	Both
pH	nosz_Cluster60	0.0079	0.0019	0.0324	-0.0185	0.4347	0.7706898	Both
pH	nosz_Cluster61	0.0178	0.0004	0.0012	-0.0514	0.8833	0.96158	Both
pH	nosz_Cluster62	0.0413	0.0158	0.1393	0.094	0.0957	0.3550399	Both
pH	nosz_Cluster64	0.0016	0.0012	0.0509	0.001	0.3253	0.6910437	Both
pH	nosz_Cluster65	0.0065	0.0013	0.0225	-0.0289	0.516	0.7988743	Both
pH	nosz_Cluster66	0.0324	0.0161	0.1554	0.111	0.077	0.3069968	Both
pH	nosz_Cluster67	0.0521	0.0248	0.157	0.1126	0.0754	0.3050575	Both
pH	nosz_Cluster69	0.0061	0.0001	0.0002	-0.0524	0.9502	0.9886888	Both
pH	nosz_Cluster70	0.0479	0.0182	0.0382	-0.0124	0.3957	0.7458918	Both
pH	nosz_Cluster71	0.0083	0.0021	0.0277	-0.0235	0.4712	0.7864352	Both
pH	nosz_Cluster72	0.0506	0.0217	0.1465	0.1016	0.0868	0.3337936	Both
pH	nosz_Cluster73	0.003	-0.0006	0.0136	-0.0383	0.6142	0.8466396	Both
pH	nosz_Cluster74	0.0017	-0.0007	0.0567	0.007	0.2986	0.6699443	Both
pH	nosz_Cluster75	0.0967	0.0522	0.0439	-0.0064	0.3621	0.720084	Both
pH	nosz_Cluster77	0.0002	0.0001	0.0184	-0.0333	0.5577	0.824681	Both
pH	nosz_Cluster78	0.0044	-0.0007	0.0166	-0.0352	0.578	0.8323762	Both
pH	nosz_Cluster79	0.0333	0.0098	0.1084	0.0615	0.145	0.4455453	Both
pH	nosz_Cluster8	0.0099	0.0021	0.0489	-0.0012	0.3356	0.7046344	Both
pH	nosz_Cluster80	0.0045	-0.0009	0.0114	-0.0406	0.6453	0.8725829	Both
pH	nosz_Cluster81	0.0079	0.0039	0.0741	0.0254	0.2325	0.5861806	Both
pH	nosz_Cluster82	0.0016	0.0026	0.1915	0.149	0.0472	0.2303769	Both
pH	nosz_Cluster83	0.0015	-0.0015	0.185	0.1421	0.0517	0.2435155	Both
pH	nosz_Cluster85	0.0022	0.0021	0.1934	0.151	0.046	0.2301907	Both
pH	nosz_Cluster86	0.0027	0.0022	0.1925	0.15	0.0466	0.2303769	Both
pH	nosz_Cluster87	0.003	0.0004	0.0081	-0.0441	0.6975	0.8890351	Both
pH	nosz_Cluster88	0.0218	-0.0004	0.0006	-0.052	0.9158	0.9753277	Both
pH	nosz_Cluster89	0.0466	0.0206	0.1523	0.1077	0.0802	0.3156172	Both
pH	nosz_Cluster9	0.003	-0.0001	0.0005	-0.0521	0.9246	0.978434	Both
pH	nosz_Cluster90	0.0116	0.0032	0.1112	0.0645	0.1395	0.4332645	Both
pH	nosz_Cluster91	0.0189	0.0028	0.0133	-0.0386	0.6186	0.8479099	Both
pH	nosz_Cluster93	0.0393	-0.0017	0.002	-0.0505	0.8467	0.9492878	Both
pH	nosz_Cluster95	0.007	0.0011	0.0163	-0.0355	0.5813	0.8323762	Both
pH	nosz_Cluster96	0.0066	-0.0023	0.1304	0.0846	0.1077	0.3744819	Both
pH	nosz_Cluster97	0.0019	0.0006	0.0268	-0.0244	0.4781	0.7879127	Both
pH	nosz_Cluster98	0.0025	0.0037	0.1798	0.1366	0.0554	0.24963	Both
pH	nosz_Cluster99	0.0055	0.0002	0.0012	-0.0514	0.8831	0.96158	Both
pH	nrfa_Cluster10	0.0804	0.0196	0.0383	-0.0124	0.3955	0.7458918	Both
pH	nrfa_Cluster100	0.0963	-0.0256	0.0327	-0.0183	0.4331	0.7706898	Both
pH	nrfa_Cluster104	0.1143	0.0208	0.0344	-0.0165	0.4212	0.7693843	Both
pH	nrfa_Cluster105	0.0062	0.0001	0.0002	-0.0524	0.9525	0.9886888	Both
pH	nrfa_Cluster109	0.2294	0.0533	0.0678	0.0187	0.2544	0.6038577	Both
pH	nrfa_Cluster116	0.2592	0.0672	0.1289	0.0831	0.11	0.3750175	Both
pH	nrfa_Cluster118	0.173	-0.0534	0.0361	-0.0146	0.4094	0.7583601	Both
pH	nrfa_Cluster125	0.0809	-0.0296	0.045	-0.0053	0.3561	0.720084	Both
pH	nrfa_Cluster154	0.0658	-0.0142	0.0235	-0.0279	0.5072	0.7957977	Both

pH	nrfa_Cluster164	0.0045	0.0003	0.0013	-0.0513	0.8772	0.9606908	Both
pH	nrfa_Cluster172	0.0026	0.0005	0.0094	-0.0428	0.6762	0.8811452	Both
pH	nrfa_Cluster175	0.0044	-0.0029	0.1039	0.0567	0.1542	0.4640683	Both
pH	nrfa_Cluster20	0.035	0.0056	0.0159	-0.0359	0.5865	0.8323762	Both
pH	nrfa_Cluster26	0.1078	-0.0279	0.028	-0.0232	0.4684	0.7851329	Both
pH	nrfa_Cluster29	0.0048	0.0014	0.0297	-0.0214	0.4553	0.7764447	Both
pH	nrfa_Cluster34	0.0487	0.0053	0.0072	-0.0451	0.7148	0.8968256	Both
pH	nrfa_Cluster38	0.0031	-0.0014	0.0558	0.0062	0.3024	0.6732025	Both
pH	nrfa_Cluster61	0.0036	0.0003	0.0021	-0.0504	0.8422	0.9481017	Both
pH	nrfa_Cluster62	0.0452	-0.0155	0.0408	-0.0097	0.3802	0.7259115	Both
pH	nrfa_Cluster66	0.1082	0.017	0.0369	-0.0138	0.4043	0.7549083	Both
pH	nrfa_Cluster69	0.003	0.0004	0.0057	-0.0466	0.7452	0.9001951	Both
pH	nrfa_Cluster71	0.027	-0.0064	0.0259	-0.0253	0.4855	0.788532	Both
pH	nrfa_Cluster72	0.0732	-0.0074	0.0056	-0.0467	0.7461	0.9001951	Both
pH	nrfa_Cluster73	0.0038	-0.0009	0.0174	-0.0343	0.5681	0.8293081	Both
pH	nrfa_Cluster74	0.1331	-0.028	0.0256	-0.0257	0.4883	0.788532	Both
pH	nrfa_Cluster76	0.0755	-0.0199	0.0345	-0.0163	0.42	0.7693843	Both
pH	nrfa_Cluster79	0.0229	-0.0042	0.0044	-0.048	0.7749	0.9235774	Both
pH	nrfa_Cluster87	0.017	0.0046	0.0206	-0.0309	0.5348	0.8031505	Both
pH	nrfa_Cluster89	0.0018	-0.0006	0.0236	-0.0277	0.5057	0.7956899	Both
pH	nrfa_Cluster92	0.0043	-0.0007	0.0095	-0.0427	0.6747	0.8811452	Both
pH	nrfa_Cluster93	0.0428	0.0171	0.0466	-0.0035	0.3471	0.720084	Both
pH	nrfa_Cluster94	0.0002	-0.0002	0.0419	-0.0086	0.3736	0.7210131	Both
pH	nrfa_Cluster97	0.0168	-0.0007	0.0014	-0.0512	0.8718	0.9594568	Both
pH	nrfa_Cluster99	0.014	-0.0027	0.0226	-0.0288	0.5152	0.7988743	Both
C	amoA_A_Cluster26	0.004	-0.003	0.1528	0.1082	0.0798	0.203954	Both
C	amoA_A_Cluster45	0.0051	-0.0037	0.1821	0.139	0.0537	0.1686003	Both
C	amoA_B_Cluster0	0.1099	-0.0382	0.5675	0.5447	1.00E-04	0.0021665	Both
C	amoA_B_Cluster1	0.0172	-0.0097	0.3197	0.2839	0.0076	0.0524733	Both
C	amoA_B_Cluster10	0.0422	-0.0243	0.6262	0.6065	0.00E+00	0.0008155	Both
C	amoA_B_Cluster11	0.0377	-0.021	0.4268	0.3966	0.0013	0.016247	Both
C	amoA_B_Cluster12	0.0807	-0.0319	0.5631	0.5401	1.00E-04	0.002289	Both
C	amoA_B_Cluster13	0.5271	-0.283	0.4881	0.4611	0.0004	0.0078415	Both
C	amoA_B_Cluster14	0.0275	-0.0171	0.3482	0.3139	0.0049	0.0398535	Both
C	amoA_B_Cluster15	0.0318	-0.018	0.4186	0.3879	0.0015	0.0180052	Both
C	amoA_B_Cluster2	0.1721	-0.0999	0.5485	0.5248	0.0001	0.0029056	Both
C	amoA_B_Cluster20	0.0058	-0.0035	0.152	0.1074	0.0806	0.2047181	Both
C	amoA_B_Cluster23	0.0507	-0.0242	0.4782	0.4507	0.0005	0.0082083	Both
C	amoA_B_Cluster3	0.0182	-0.0099	0.2509	0.2114	0.0207	0.0955612	Both
C	amoA_B_Cluster4	0.0125	-0.0078	0.2795	0.2416	0.0137	0.0736999	Both
C	amoA_B_Cluster5	0.0928	-0.0348	0.5248	0.4998	0.0002	0.0046337	Both
C	amoA_B_Cluster6	0.0233	-0.0096	0.3186	0.2828	0.0077	0.0524733	Both
C	amoA_B_Cluster7	0.0179	-0.0077	0.1545	0.11	0.078	0.2018145	Both
C	amoA_B_Cluster8	0.0207	-0.0104	0.2026	0.1606	0.0406	0.142862	Both
C	amoA_B_Cluster9	0.0549	-0.03	0.3367	0.3018	0.0058	0.04465	Both
C	nifh_Cluster0	0.0332	0.005	0.1013	0.054	0.1598	0.3021059	Both
C	nifh_Cluster10	0.005	0.0026	0.0614	0.012	0.2788	0.407168	Both
C	nifh_Cluster100	0.0019	0.00E+00	1.00E-04	-0.0526	0.9736	0.9814204	Both
C	nifh_Cluster103	0.0383	-0.014	0.2924	0.2552	0.0114	0.0664301	Both
C	nifh_Cluster1034	0.0262	-0.0143	0.221	0.18	0.0315	0.1223694	Both
C	nifh_Cluster105	0.013	-0.0063	0.1635	0.1195	0.069	0.1914538	Both
C	nifh_Cluster108	0.0083	-0.0017	0.0375	-0.0132	0.4003	0.5259729	Both
C	nifh_Cluster1099	0.066	0.045	0.47	0.4421	0.0006	0.0088895	Both
C	nifh_Cluster11	0.0021	-0.0008	0.0639	0.0146	0.2691	0.4033763	Both
C	nifh_Cluster1112	0.0029	-0.0013	0.0713	0.0224	0.242	0.3737624	Both

C	nifh_Cluster112	0.0112	0.006	0.2571	0.218	0.019	0.0910585	Both
C	nifh_Cluster113	0.0327	-0.0068	0.1099	0.063	0.1422	0.2815285	Both
C	nifh_Cluster114	0.0332	0.0023	0.0274	-0.0238	0.4731	0.5790547	Both
C	nifh_Cluster1141	0.0251	-0.0119	0.2524	0.2131	0.0203	0.0950009	Both
C	nifh_Cluster115	0.0095	0.0004	0.0031	-0.0493	0.8094	0.8666619	Both
C	nifh_Cluster116	0.0775	-0.0252	0.2824	0.2446	0.0132	0.0733389	Both
C	nifh_Cluster1163	0.0044	-0.0015	0.094	0.0464	0.1764	0.3166001	Both
C	nifh_Cluster1179	0.0031	-0.0015	0.0775	0.029	0.2216	0.3533829	Both
C	nifh_Cluster1182	0.0204	0.0003	0.0008	-0.0518	0.9011	0.9294116	Both
C	nifh_Cluster1183	0.0066	0.0036	0.3546	0.3207	0.0044	0.0375835	Both
C	nifh_Cluster1197	0.0115	-0.0025	0.0614	0.012	0.2787	0.407168	Both
C	nifh_Cluster1199	0.0046	-0.0006	0.0112	-0.0409	0.6485	0.7302111	Both
C	nifh_Cluster120	0.0149	-0.0092	0.1059	0.0589	0.1499	0.2900645	Both
C	nifh_Cluster1203	0.0155	-0.0094	0.229	0.1884	0.0282	0.1136085	Both
C	nifh_Cluster1206	0.0043	-0.0025	0.2017	0.1597	0.0411	0.1435125	Both
C	nifh_Cluster1207	0.0074	-0.0033	0.1198	0.0735	0.1242	0.2717916	Both
C	nifh_Cluster1209	0.1506	-0.0138	0.062	0.0127	0.2762	0.407168	Both
C	nifh_Cluster121	0.0095	-0.0052	0.0922	0.0444	0.1808	0.3166001	Both
C	nifh_Cluster1211	0.0056	-0.002	0.1046	0.0575	0.1527	0.292492	Both
C	nifh_Cluster1213	0.0026	-0.0008	0.0559	0.0062	0.302	0.4287248	Both
C	nifh_Cluster122	0.0029	-0.0022	0.1774	0.1341	0.0572	0.1746134	Both
C	nifh_Cluster123	0.0095	0.0007	0.0015	-0.051	0.8669	0.906884	Both
C	nifh_Cluster1236	0.0234	-0.0115	0.0977	0.0502	0.1678	0.308643	Both
C	nifh_Cluster1238	0.0028	-0.0008	0.0269	-0.0243	0.4772	0.5817259	Both
C	nifh_Cluster124	0.0091	-0.0009	0.0095	-0.0426	0.6741	0.7490315	Both
C	nifh_Cluster1261	0.002	-0.0006	0.0293	-0.0217	0.4578	0.5686978	Both
C	nifh_Cluster1262	0.0065	-0.0015	0.0318	-0.0192	0.4395	0.5561105	Both
C	nifh_Cluster1266	0.0094	0.0047	0.225	0.1842	0.0298	0.1189289	Both
C	nifh_Cluster1267	0.0562	0.0466	0.5821	0.5601	1.00E-04	0.0016995	Both
C	nifh_Cluster127	0.0026	-0.0018	0.1262	0.0802	0.1141	0.2580088	Both
C	nifh_Cluster1278	0.1602	-0.0905	0.4756	0.448	0.0005	0.0082083	Both
C	nifh_Cluster128	0.0036	-0.0012	0.0554	0.0057	0.3045	0.4287248	Both
C	nifh_Cluster1289	0.0039	0.00E+00	1.00E-04	-0.0525	0.9668	0.9767592	Both
C	nifh_Cluster129	0.0077	-0.0014	0.0122	-0.0398	0.6335	0.714603	Both
C	nifh_Cluster1292	0.0086	-0.0034	0.1178	0.0713	0.1277	0.2721499	Both
C	nifh_Cluster130	0.0059	-0.0023	0.038	-0.0126	0.3969	0.5239382	Both
C	nifh_Cluster1305	0.0048	0.0007	0.0196	-0.032	0.5452	0.636948	Both
C	nifh_Cluster1306	0.005	-0.002	0.106	0.0589	0.1499	0.2900645	Both
C	nifh_Cluster131	0.0051	-0.003	0.1132	0.0666	0.1358	0.2775273	Both
C	nifh_Cluster1319	0.0036	-0.0017	0.0853	0.0371	0.199	0.3334469	Both
C	nifh_Cluster132	0.0064	1.00E-04	0.0001	-0.0525	0.9643	0.9758719	Both
C	nifh_Cluster1320	0.0025	-0.0004	0.0078	-0.0444	0.7031	0.7797743	Both
C	nifh_Cluster1323	0.0023	-1.00E-04	0.0005	-0.0521	0.9201	0.9441277	Both
C	nifh_Cluster1324	0.03	0.0131	0.2309	0.1904	0.0275	0.1125489	Both
C	nifh_Cluster1336	0.0153	-0.0001	0.0002	-0.0525	0.9555	0.9719289	Both
C	nifh_Cluster1340	0.0363	-0.0142	0.0786	0.0301	0.2182	0.350843	Both
C	nifh_Cluster1345	0.0483	0.0366	0.484	0.4569	0.0005	0.0080157	Both
C	nifh_Cluster1370	0.0021	-0.0006	0.0287	-0.0225	0.4631	0.5728206	Both
C	nifh_Cluster1375	0.0018	0.0005	0.0373	-0.0134	0.4018	0.5268623	Both
C	nifh_Cluster139	0.0041	0.001	0.0249	-0.0265	0.495	0.5971837	Both
C	nifh_Cluster140	0.0043	-0.0017	0.1109	0.0641	0.1403	0.2795648	Both
C	nifh_Cluster141	0.0677	-0.0364	0.6483	0.6298	0.00E+00	0.0004847	Both
C	nifh_Cluster148	0.0046	-0.0026	0.0986	0.0512	0.1657	0.3083235	Both
C	nifh_Cluster1480	0.001	0.0012	0.3172	0.2813	0.0078	0.0524733	Both
C	nifh_Cluster152	0.0599	-0.0012	0.0012	-0.0514	0.8813	0.9156884	Both

C	nifh_Cluster156	0.0659	0.0454	0.8402	0.8318	0.00E+00	3.14E-06	Both
C	nifh_Cluster1562	0.0543	0.0591	0.8003	0.7898	0.00E+00	8.85E-06	Both
C	nifh_Cluster158	0.0144	-0.007	0.1695	0.1258	0.0637	0.1851407	Both
C	nifh_Cluster16	0.0019	-0.0007	0.0339	-0.017	0.4244	0.5419068	Both
C	nifh_Cluster166	0.0172	0.0013	0.0017	-0.0509	0.8607	0.9051724	Both
C	nifh_Cluster205	0.0122	0.0003	0.0006	-0.052	0.9137	0.939155	Both
C	nifh_Cluster21	0.0021	-0.0014	0.1512	0.1065	0.0815	0.2047181	Both
C	nifh_Cluster222	0.006	-0.0016	0.0218	-0.0297	0.5231	0.6209393	Both
C	nifh_Cluster225	0.0189	-0.0026	0.0423	-0.0081	0.3712	0.4977989	Both
C	nifh_Cluster230	0.0042	-0.0017	0.1069	0.0599	0.1479	0.2890397	Both
C	nifh_Cluster231	0.0022	0.0006	0.024	-0.0273	0.5021	0.6033427	Both
C	nifh_Cluster236	0.0027	-0.0018	0.2322	0.1918	0.027	0.1112023	Both
C	nifh_Cluster237	0.0058	-0.0021	0.1038	0.0566	0.1544	0.2947169	Both
C	nifh_Cluster243	0.0333	0.0163	0.2468	0.2071	0.022	0.0989577	Both
C	nifh_Cluster2432	0.0436	0.0336	0.588	0.5663	1.00E-04	0.0016432	Both
C	nifh_Cluster2433	0.0032	0.0011	0.0316	-0.0193	0.4404	0.5561105	Both
C	nifh_Cluster246	0.0057	-0.0026	0.0974	0.0499	0.1684	0.308643	Both
C	nifh_Cluster25	0.0295	0.0171	0.554	0.5305	0.0001	0.0026851	Both
C	nifh_Cluster256	0.0029	0.0003	0.0048	-0.0476	0.7661	0.8324185	Both
C	nifh_Cluster264	0.0029	0.0008	0.048	-0.0021	0.3401	0.4655788	Both
C	nifh_Cluster265	0.0736	0.0138	0.1472	0.1023	0.086	0.212286	Both
C	nifh_Cluster267	0.0109	0.0067	0.2804	0.2426	0.0136	0.073376	Both
C	nifh_Cluster268	0.0111	0.0015	0.0295	-0.0215	0.4563	0.5686978	Both
C	nifh_Cluster269	0.023	-0.0079	0.0922	0.0445	0.1807	0.3166001	Both
C	nifh_Cluster272	0.0041	0.0058	0.6779	0.6609	0.00E+00	0.0002984	Both
C	nifh_Cluster274	0.0032	0.0032	0.0941	0.0464	0.1763	0.3166001	Both
C	nifh_Cluster275	0.0036	-0.0018	0.1621	0.118	0.0704	0.1914538	Both
C	nifh_Cluster278	0.0077	0.0051	0.3255	0.29	0.0069	0.0523014	Both
C	nifh_Cluster279	0.006	0.0007	0.0186	-0.0331	0.5558	0.6431223	Both
C	nifh_Cluster28	0.002	0.0004	0.0223	-0.0292	0.5185	0.6180563	Both
C	nifh_Cluster280	0.0031	-0.0011	0.0294	-0.0216	0.4571	0.5686978	Both
C	nifh_Cluster282	0.0052	-0.0003	0.004	-0.0484	0.7856	0.8489263	Both
C	nifh_Cluster287	0.0022	-0.0007	0.0385	-0.0121	0.3941	0.5213125	Both
C	nifh_Cluster290	0.011	0.0024	0.0475	-0.0026	0.3426	0.4679636	Both
C	nifh_Cluster297	0.0029	-0.0021	0.0912	0.0433	0.1835	0.3183098	Both
C	nifh_Cluster299	0.0026	0.0007	0.0203	-0.0312	0.5374	0.633867	Both
C	nifh_Cluster303	0.002	0.0008	0.0532	0.0033	0.3147	0.4399661	Both
C	nifh_Cluster304	0.0024	-0.0004	0.0095	-0.0426	0.6738	0.7490315	Both
C	nifh_Cluster313	0.0103	-0.0047	0.0723	0.0235	0.2385	0.3699188	Both
C	nifh_Cluster314	0.0022	-0.0007	0.0351	-0.0157	0.4162	0.538528	Both
C	nifh_Cluster317	0.0071	-0.0032	0.1585	0.1143	0.0738	0.1970812	Both
C	nifh_Cluster32	0.0033	-0.0006	0.0219	-0.0296	0.5219	0.6208416	Both
C	nifh_Cluster320	12.7419	-2.6067	0.6893	0.673	0.00E+00	0.000252	Both
C	nifh_Cluster334	0.0063	-0.0037	0.091	0.0431	0.184	0.3183098	Both
C	nifh_Cluster335	0.0075	0.0029	0.0301	-0.021	0.4524	0.5666466	Both
C	nifh_Cluster338	0.0123	-0.0046	0.1642	0.1202	0.0684	0.1912117	Both
C	nifh_Cluster340	0.007	0.0055	0.0883	0.0403	0.1908	0.3252903	Both
C	nifh_Cluster341	0.0023	0.0011	0.0755	0.0268	0.2281	0.3569966	Both
C	nifh_Cluster35	0.0078	-0.0019	0.0682	0.0192	0.2527	0.3856725	Both
C	nifh_Cluster351	0.0025	-0.0017	0.1272	0.0812	0.1126	0.2574211	Both
C	nifh_Cluster3574	0.1093	-0.0225	0.1379	0.0925	0.0975	0.2319245	Both
C	nifh_Cluster3691	0.0174	-0.0092	0.1659	0.122	0.0669	0.1905281	Both
C	nifh_Cluster37	0.0329	-0.009	0.0592	0.0097	0.2879	0.4184343	Both
C	nifh_Cluster374	0.0021	-0.001	0.0636	0.0143	0.27	0.4033763	Both
C	nifh_Cluster38	0.0009	0.0002	0.0136	-0.0383	0.6148	0.6961832	Both

C	nifh_Cluster382	0.0016	-0.0012	0.0749	0.0262	0.2301	0.3591718	Both
C	nifh_Cluster384	0.0019	-0.0007	0.0489	-0.0012	0.3356	0.4614584	Both
C	nifh_Cluster386	0.0035	-0.0011	0.0269	-0.0243	0.4772	0.5817259	Both
C	nifh_Cluster389	0.0031	1.00E-04	0.0002	-0.0524	0.9531	0.971215	Both
C	nifh_Cluster394	0.002	-0.0014	0.0805	0.0321	0.2126	0.3474526	Both
C	nifh_Cluster40	0.0012	-0.0009	0.1425	0.0974	0.0915	0.2231629	Both
C	nifh_Cluster43	0.0108	0.001	0.0135	-0.0385	0.6164	0.6967533	Both
C	nifh_Cluster434	0.0035	-0.0021	0.124	0.0779	0.1174	0.263425	Both
C	nifh_Cluster440	0.0133	-0.0081	0.22	0.179	0.0319	0.122396	Both
C	nifh_Cluster462	0.0034	-0.0022	0.142	0.0968	0.0922	0.2239532	Both
C	nifh_Cluster470	0.0152	-0.0009	0.0048	-0.0476	0.7654	0.8324185	Both
C	nifh_Cluster49	0.0213	-0.0025	0.0056	-0.0468	0.7482	0.817489	Both
C	nifh_Cluster499	0.0026	-0.0006	0.0227	-0.0287	0.5141	0.6140267	Both
C	nifh_Cluster5	0.0256	-1.00E-04	0.00E+00	-0.0526	0.9764	0.9814204	Both
C	nifh_Cluster506	0.0048	-0.0002	0.0017	-0.0508	0.8572	0.9046906	Both
C	nifh_Cluster51	0.0028	-0.0006	0.0146	-0.0372	0.6015	0.6837662	Both
C	nifh_Cluster52	0.0336	-0.0108	0.0608	0.0114	0.2811	0.4094899	Both
C	nifh_Cluster525	0.0033	-0.0023	0.0825	0.0343	0.2066	0.3424502	Both
C	nifh_Cluster531	0.0258	0.0252	0.825	0.8158	0.00E+00	3.73E-06	Both
C	nifh_Cluster539	0.0048	-0.0014	0.017	-0.0347	0.5727	0.6598082	Both
C	nifh_Cluster578	0.0481	-0.0179	0.2343	0.1941	0.0262	0.1087306	Both
C	nifh_Cluster58	0.3542	0.2747	0.6071	0.5865	0.00E+00	0.0011608	Both
C	nifh_Cluster60	0.0089	-0.0034	0.089	0.0411	0.1889	0.3240348	Both
C	nifh_Cluster61	0.0025	0.0006	0.0297	-0.0214	0.4552	0.5686978	Both
C	nifh_Cluster65	0.009	-1.00E-04	0.00E+00	-0.0526	0.9759	0.9814204	Both
C	nifh_Cluster686	0.0009	-0.0004	0.0424	-0.008	0.3704	0.4977989	Both
C	nifh_Cluster69	0.009	-0.0025	0.0417	-0.0087	0.3743	0.5007716	Both
C	nifh_Cluster70	0.0111	-0.006	0.0831	0.0349	0.205	0.3406422	Both
C	nifh_Cluster717	0.003	0.0017	0.2025	0.1605	0.0407	0.142862	Both
C	nifh_Cluster725	0.0058	0.0043	0.4164	0.3856	0.0016	0.0183342	Both
C	nifh_Cluster727	0.0039	-0.0017	0.0932	0.0455	0.1783	0.3166001	Both
C	nifh_Cluster73	0.0106	-0.0048	0.1171	0.0706	0.1289	0.2735581	Both
C	nifh_Cluster74	0.0049	-0.0002	0.0003	-0.0523	0.9394	0.9615099	Both
C	nifh_Cluster748	0.0081	-0.0027	0.1474	0.1025	0.0858	0.212286	Both
C	nifh_Cluster749	0.0071	0.0025	0.1278	0.0819	0.1117	0.2563185	Both
C	nifh_Cluster755	0.0102	-0.0057	0.1965	0.1542	0.0441	0.1470069	Both
C	nifh_Cluster76	0.0029	-0.0006	0.0186	-0.033	0.5551	0.6431223	Both
C	nifh_Cluster79	0.0022	-0.0003	0.0061	-0.0463	0.7375	0.8087548	Both
C	nifh_Cluster81	0.0055	-0.0012	0.0159	-0.0359	0.5857	0.6696739	Both
C	nifh_Cluster82	0.0011	-0.0005	0.0338	-0.0171	0.4253	0.5419068	Both
C	nifh_Cluster83	0.0069	0.0019	0.0355	-0.0153	0.4136	0.5376991	Both
C	nifh_Cluster86	0.0135	-0.0057	0.1962	0.1539	0.0444	0.1470069	Both
C	nifh_Cluster868	0.0099	0.00E+00	0.00E+00	-0.0526	0.9876	0.9893019	Both
C	nifh_Cluster87	0.0024	-0.001	0.0558	0.0061	0.3026	0.4287248	Both
C	nifh_Cluster88	0.0039	-0.0021	0.0932	0.0455	0.1784	0.3166001	Both
C	nifh_Cluster9	0.0078	0.00E+00	0.00E+00	-0.0526	0.9894	0.9894285	Both
C	nifh_Cluster92	0.0051	-0.003	0.0926	0.0449	0.1798	0.3166001	Both
C	nifh_Cluster93	0.0036	0.0001	0.0008	-0.0518	0.9032	0.9299926	Both
C	nifh_Cluster94	0.0047	-0.0007	0.0064	-0.0459	0.7301	0.8034453	Both
C	nifh_Cluster95	0.0121	-0.0043	0.0567	0.0071	0.2985	0.4287248	Both
C	nifh_Cluster96	0.0123	-0.0001	0.0001	-0.0525	0.9614	0.975596	Both
C	nifh_Cluster97	0.018	0.0114	0.3098	0.2735	0.0088	0.0569059	Both
C	nifh_Cluster98	0.0059	0.0006	0.0101	-0.042	0.6651	0.7432219	Both
C	nifh_Cluster99	0.0033	-0.0004	0.0052	-0.0471	0.7554	0.8238591	Both
C	nirk_Cluster0	5.9009	-1.2853	0.7367	0.7228	0.00E+00	6.36E-05	Both

C	nirk_Cluster1	0.1708	-0.1031	0.5678	0.545	1.00E-04	0.0021665	Both
C	nirk_Cluster10	0.5713	-0.1565	0.3753	0.3424	0.0032	0.0286453	Both
C	nirk_Cluster101	0.0031	-0.0009	0.0342	-0.0166	0.4223	0.5419068	Both
C	nirk_Cluster102	0.0426	-0.022	0.349	0.3147	0.0048	0.0398535	Both
C	nirk_Cluster103	0.0754	-0.0403	0.4867	0.4597	0.0004	0.0078415	Both
C	nirk_Cluster104	0.0023	-0.0014	0.1124	0.0657	0.1374	0.2775273	Both
C	nirk_Cluster105	0.0946	-0.0493	0.4769	0.4494	0.0005	0.0082083	Both
C	nirk_Cluster106	0.0592	-0.0309	0.275	0.2368	0.0147	0.0773024	Both
C	nirk_Cluster107	0.0891	-0.0447	0.3875	0.3552	0.0026	0.0247097	Both
C	nirk_Cluster108	0.0151	-0.0047	0.0901	0.0422	0.1862	0.3211677	Both
C	nirk_Cluster109	0.0115	-0.0075	0.3428	0.3082	0.0053	0.0416396	Both
C	nirk_Cluster11	0.1401	-0.0163	0.2186	0.1775	0.0326	0.124009	Both
C	nirk_Cluster12	0.094	-0.0279	0.371	0.3379	0.0034	0.0293276	Both
C	nirk_Cluster13	0.0408	-0.0024	0.0232	-0.0282	0.5098	0.610077	Both
C	nirk_Cluster14	0.0946	-0.0339	0.5222	0.4971	0.0002	0.0047057	Both
C	nirk_Cluster15	0.3358	-0.1755	0.4932	0.4666	0.0004	0.0073483	Both
C	nirk_Cluster16	0.4727	-0.2347	0.4089	0.3778	0.0018	0.0202209	Both
C	nirk_Cluster17	0.0998	-0.0083	0.0492	-0.0009	0.334	0.4614584	Both
C	nirk_Cluster18	0.1296	-0.0391	0.1749	0.1315	0.0592	0.1763266	Both
C	nirk_Cluster19	0.2776	-0.0389	0.2144	0.1731	0.0345	0.1276302	Both
C	nirk_Cluster2	0.2032	-0.0055	0.0064	-0.0459	0.7313	0.8034453	Both
C	nirk_Cluster20	0.1738	-0.0269	0.0876	0.0395	0.1928	0.3268682	Both
C	nirk_Cluster21	0.1532	-0.0336	0.3943	0.3624	0.0023	0.0243033	Both
C	nirk_Cluster22	0.1291	-0.0391	0.4027	0.3712	0.002	0.0214824	Both
C	nirk_Cluster23	0.0673	-0.0117	0.2963	0.2593	0.0107	0.0646055	Both
C	nirk_Cluster24	0.0654	-0.0256	0.4995	0.4731	0.0003	0.0067168	Both
C	nirk_Cluster25	0.4782	-0.1837	0.6548	0.6366	0.00E+00	0.0004386	Both
C	nirk_Cluster26	0.105	-0.0162	0.1835	0.1406	0.0527	0.16616	Both
C	nirk_Cluster27	0.1727	-0.0677	0.506	0.48	0.0003	0.0060935	Both
C	nirk_Cluster28	0.0685	-0.0211	0.3162	0.2802	0.008	0.0524733	Both
C	nirk_Cluster29	0.1526	-0.0149	0.0638	0.0146	0.2691	0.4033763	Both
C	nirk_Cluster3	0.3023	-0.0794	0.4375	0.4079	0.0011	0.0146617	Both
C	nirk_Cluster30	0.0556	-0.03	0.4783	0.4508	0.0005	0.0082083	Both
C	nirk_Cluster31	0.1501	0.0203	0.2289	0.1883	0.0283	0.1136085	Both
C	nirk_Cluster32	0.1254	-0.072	0.4384	0.4088	0.0011	0.0146617	Both
C	nirk_Cluster33	0.0638	-0.0361	0.3832	0.3507	0.0028	0.0259615	Both
C	nirk_Cluster34	0.0416	-0.0096	0.1178	0.0713	0.1278	0.2721499	Both
C	nirk_Cluster35	0.1007	-0.0523	0.3189	0.2831	0.0076	0.0524733	Both
C	nirk_Cluster36	0.0017	-0.0011	0.1207	0.0744	0.1229	0.2717916	Both
C	nirk_Cluster39	0.0035	-0.0011	0.0149	-0.0369	0.5978	0.6809284	Both
C	nirk_Cluster4	0.1323	-0.0389	0.3472	0.3129	0.0049	0.0399402	Both
C	nirk_Cluster40	0.0455	-0.0205	0.5896	0.568	0.00E+00	0.0016432	Both
C	nirk_Cluster41	0.1562	-0.0538	0.6642	0.6465	0.00E+00	0.0003658	Both
C	nirk_Cluster44	0.0669	-0.0355	0.3871	0.3549	0.0026	0.0247097	Both
C	nirk_Cluster46	0.0154	-0.009	0.2412	0.2013	0.0238	0.1023601	Both
C	nirk_Cluster48	0.003	-0.0013	0.0886	0.0407	0.19	0.3248945	Both
C	nirk_Cluster49	0.0057	-0.0028	0.1014	0.0541	0.1595	0.3021059	Both
C	nirk_Cluster5	0.1932	-0.0391	0.3159	0.2799	0.008	0.0524733	Both
C	nirk_Cluster50	0.0016	0.0002	0.0058	-0.0465	0.7423	0.8125349	Both
C	nirk_Cluster52	0.0452	-0.0209	0.4789	0.4515	0.0005	0.0082083	Both
C	nirk_Cluster53	0.0038	-0.0014	0.0442	-0.0061	0.3602	0.4851667	Both
C	nirk_Cluster54	0.002	-0.0006	0.0342	-0.0167	0.4225	0.5419068	Both
C	nirk_Cluster55	0.0023	-0.0008	0.0285	-0.0227	0.4646	0.5734746	Both
C	nirk_Cluster56	0.0009	-0.0001	0.0037	-0.0488	0.7943	0.8567612	Both
C	nirk_Cluster57	0.0016	-0.0002	0.0027	-0.0498	0.8226	0.8729214	Both

C	nirk_Cluster58	0.0088	-0.0034	0.1132	0.0665	0.1359	0.2775273	Both
C	nirk_Cluster59	0.0058	-0.0022	0.1017	0.0544	0.1588	0.3021059	Both
C	nirk_Cluster6	0.1558	-0.0429	0.3806	0.348	0.0029	0.0266429	Both
C	nirk_Cluster60	0.0006	0.0001	0.0073	-0.045	0.7134	0.7897467	Both
C	nirk_Cluster62	0.1417	-0.0783	0.4287	0.3986	0.0013	0.016247	Both
C	nirk_Cluster63	0.1284	-0.0751	0.7406	0.727	0.00E+00	6.36E-05	Both
C	nirk_Cluster64	0.0044	-0.0003	0.0034	-0.049	0.8015	0.8607168	Both
C	nirk_Cluster65	0.0464	-0.0175	0.3727	0.3397	0.0033	0.0293276	Both
C	nirk_Cluster66	0.0068	-0.0047	0.3366	0.3017	0.0058	0.04465	Both
C	nirk_Cluster67	0.0174	-0.0085	0.1764	0.1331	0.058	0.1746134	Both
C	nirk_Cluster68	0.0125	-0.008	0.3921	0.3601	0.0024	0.0243134	Both
C	nirk_Cluster69	0.0305	-0.0114	0.291	0.2537	0.0116	0.0671708	Both
C	nirk_Cluster7	0.0804	-0.0301	0.4254	0.3951	0.0014	0.0163151	Both
C	nirk_Cluster70	0.0721	-0.0417	0.2532	0.2139	0.0201	0.0946822	Both
C	nirk_Cluster71	0.0053	-0.0028	0.1807	0.1376	0.0547	0.1708878	Both
C	nirk_Cluster72	0.0239	-0.011	0.4084	0.3773	0.0018	0.0202209	Both
C	nirk_Cluster73	0.1444	-0.0918	0.6724	0.6552	0.00E+00	0.0003163	Both
C	nirk_Cluster74	0.6398	-0.3961	0.5852	0.5633	1.00E-04	0.0016653	Both
C	nirk_Cluster75	0.0147	-0.0098	0.2398	0.1998	0.0242	0.103151	Both
C	nirk_Cluster76	0.1055	-0.061	0.7735	0.7616	0.00E+00	2.23E-05	Both
C	nirk_Cluster77	0.0081	-0.0041	0.0561	0.0064	0.3014	0.4287248	Both
C	nirk_Cluster78	0.2407	-0.1425	0.6872	0.6708	0.00E+00	0.000252	Both
C	nirk_Cluster79	0.0069	-0.0038	0.0972	0.0497	0.1689	0.308643	Both
C	nirk_Cluster8	0.0982	-0.0382	0.3232	0.2876	0.0072	0.0524733	Both
C	nirk_Cluster80	0.2665	-0.165	0.6142	0.5939	0.00E+00	0.0010355	Both
C	nirk_Cluster82	0.0015	-0.001	0.0628	0.0134	0.2733	0.4051947	Both
C	nirk_Cluster83	0.004	-0.0011	0.0466	-0.0036	0.3475	0.4713244	Both
C	nirk_Cluster84	0.0207	-0.0039	0.0264	-0.0249	0.4819	0.5862459	Both
C	nirk_Cluster85	0.004	-0.002	0.1308	0.0851	0.1072	0.2499037	Both
C	nirk_Cluster86	0.0286	-0.0174	0.4055	0.3742	0.0019	0.0208689	Both
C	nirk_Cluster87	0.0115	-0.0069	0.3243	0.2888	0.007	0.0524733	Both
C	nirk_Cluster88	0.0032	-0.0012	0.0824	0.0341	0.2072	0.3424502	Both
C	nirk_Cluster89	0.0065	-0.0038	0.1771	0.1338	0.0574	0.1746134	Both
C	nirk_Cluster9	0.0517	-0.0159	0.1652	0.1213	0.0675	0.1905281	Both
C	nirk_Cluster90	0.0067	-0.0039	0.1553	0.1109	0.0771	0.2018145	Both
C	nirk_Cluster91	0.0374	-0.0184	0.4272	0.397	0.0013	0.016247	Both
C	nirk_Cluster92	0.0072	-0.0041	0.1715	0.1279	0.062	0.1819671	Both
C	nirk_Cluster93	0.0308	-0.0071	0.0929	0.0451	0.1792	0.3166001	Both
C	nirk_Cluster94	0.0175	-0.0086	0.2603	0.2214	0.0181	0.0890895	Both
C	nirk_Cluster95	0.0473	-0.0245	0.3494	0.3151	0.0048	0.0398535	Both
C	nirk_Cluster96	0.0472	-0.0247	0.4461	0.417	0.0009	0.0131603	Both
C	nirk_Cluster97	0.0306	-0.0097	0.1672	0.1234	0.0657	0.1892207	Both
C	nirk_Cluster98	0.0181	-0.01	0.2155	0.1742	0.034	0.1276302	Both
C	nirk_Cluster99	0.006	-0.0035	0.1851	0.1422	0.0516	0.163854	Both
C	nirs_Cluster0	0.1409	-0.0415	0.3717	0.3386	0.0033	0.0293276	Both
C	nirs_Cluster1	0.004	-0.0017	0.0557	0.006	0.303	0.4287248	Both
C	nirs_Cluster14	0.0107	-0.0064	0.0797	0.0313	0.215	0.3484683	Both
C	nirs_Cluster2	0.0139	-0.0065	0.0807	0.0323	0.212	0.3474037	Both
C	nirs_Cluster26	0.0005	-0.0003	0.0491	-0.001	0.3345	0.4614584	Both
C	nirs_Cluster28	0.0071	-0.0052	0.1135	0.0668	0.1354	0.2775273	Both
C	nirs_Cluster3	0.1715	-0.0362	0.3872	0.3549	0.0026	0.0247097	Both
C	nirs_Cluster36	0.0013	-0.0007	0.0186	-0.0331	0.5559	0.6431223	Both
C	nirs_Cluster37	0.0066	-0.0048	0.0616	0.0122	0.2781	0.407168	Both
C	nirs_Cluster4	0.0002	-0.0002	0.0256	-0.0257	0.4883	0.5903184	Both
C	nirs_Cluster50	0.002	0.00E+00	0.0001	-0.0525	0.9624	0.975596	Both

C	nirs_Cluster9	0.0269	-0.0202	0.1158	0.0692	0.1313	0.2755658	Both
C	norb_Cluster0	0.0775	-0.03	0.2455	0.2058	0.0224	0.099187	Both
C	norb_Cluster1	1.1042	-0.5222	0.2679	0.2294	0.0162	0.0826451	Both
C	norb_Cluster101	0.0026	-0.0019	0.1206	0.0743	0.123	0.2717916	Both
C	norb_Cluster102	0.0038	-0.0023	0.1746	0.1311	0.0595	0.1763266	Both
C	norb_Cluster103	0.0018	-0.0013	0.1155	0.069	0.1317	0.2755658	Both
C	norb_Cluster104	0.0023	-0.0008	0.0305	-0.0205	0.4487	0.5644579	Both
C	norb_Cluster106	0.125	-0.0451	0.2227	0.1818	0.0308	0.1205815	Both
C	norb_Cluster107	0.0133	-0.0067	0.2003	0.1582	0.0419	0.1440231	Both
C	norb_Cluster108	0.0052	-0.0034	0.3009	0.2641	0.01	0.0622988	Both
C	norb_Cluster109	0.0227	-0.0073	0.113	0.0663	0.1363	0.2775273	Both
C	norb_Cluster11	0.0126	-0.0068	0.2144	0.1731	0.0345	0.1276302	Both
C	norb_Cluster110	0.0099	-0.0054	0.2012	0.1592	0.0414	0.1435874	Both
C	norb_Cluster112	0.0171	-0.0072	0.218	0.1768	0.0329	0.1242793	Both
C	norb_Cluster113	0.0134	-0.0038	0.0559	0.0062	0.3024	0.4287248	Both
C	norb_Cluster114	0.0025	-0.0018	0.0803	0.0318	0.2133	0.3476168	Both
C	norb_Cluster115	0.0137	0.0178	0.1311	0.0854	0.1067	0.249812	Both
C	norb_Cluster116	0.0072	-0.0018	0.0281	-0.0231	0.468	0.5740045	Both
C	norb_Cluster117	0.0027	-0.0012	0.0636	0.0143	0.2701	0.4033763	Both
C	norb_Cluster118	0.0037	-0.0018	0.1314	0.0857	0.1063	0.249812	Both
C	norb_Cluster119	0.019	-0.0122	0.2469	0.2072	0.0219	0.0989577	Both
C	norb_Cluster12	0.0047	-0.0019	0.0615	0.0121	0.2786	0.407168	Both
C	norb_Cluster121	0.0017	-0.001	0.0572	0.0076	0.2964	0.4275349	Both
C	norb_Cluster122	0.0144	-0.0063	0.1775	0.1342	0.0572	0.1746134	Both
C	norb_Cluster123	0.0053	-0.0028	0.1341	0.0885	0.1026	0.2431163	Both
C	norb_Cluster124	0.682	-0.1987	0.1289	0.083	0.11	0.2545496	Both
C	norb_Cluster125	0.004	-0.0021	0.1593	0.1151	0.0731	0.1968545	Both
C	norb_Cluster126	0.0082	-0.0048	0.2287	0.1881	0.0283	0.1136085	Both
C	norb_Cluster127	0.1735	-0.079	0.2697	0.2312	0.0158	0.0813114	Both
C	norb_Cluster128	0.0115	-0.0043	0.1549	0.1104	0.0775	0.2018145	Both
C	norb_Cluster13	0.0045	-0.0027	0.118	0.0715	0.1274	0.2721499	Both
C	norb_Cluster131	0.0184	-0.0032	0.0316	-0.0194	0.4411	0.5561105	Both
C	norb_Cluster132	1.0357	-0.4817	0.4561	0.4275	0.0008	0.0112408	Both
C	norb_Cluster133	0.0018	-0.0011	0.1065	0.0595	0.1488	0.2896574	Both
C	norb_Cluster135	0.0567	-0.0262	0.2805	0.2426	0.0135	0.073376	Both
C	norb_Cluster136	0.0213	-0.0083	0.1795	0.1363	0.0557	0.1719199	Both
C	norb_Cluster139	0.0328	-0.0144	0.2658	0.2272	0.0167	0.0844597	Both
C	norb_Cluster14	0.0026	-0.0019	0.197	0.1547	0.0439	0.1470069	Both
C	norb_Cluster140	0.0437	-0.0238	0.3874	0.3552	0.0026	0.0247097	Both
C	norb_Cluster142	0.0015	-0.0011	0.0989	0.0515	0.165	0.3081604	Both
C	norb_Cluster144	0.0098	-0.0033	0.0937	0.046	0.1772	0.3166001	Both
C	norb_Cluster146	0.0348	-0.0151	0.2902	0.2528	0.0117	0.067301	Both
C	norb_Cluster15	0.0469	-0.0198	0.2068	0.165	0.0384	0.1374498	Both
C	norb_Cluster150	0.0157	-0.0088	0.4318	0.4019	0.0012	0.0158746	Both
C	norb_Cluster155	0.1631	-0.046	0.1334	0.0878	0.1035	0.2442267	Both
C	norb_Cluster16	0.0131	-0.0074	0.1897	0.147	0.0485	0.1578445	Both
C	norb_Cluster164	0.0199	-0.013	0.3227	0.287	0.0072	0.0524733	Both
C	norb_Cluster165	0.0076	-0.0035	0.0936	0.0459	0.1774	0.3166001	Both
C	norb_Cluster166	0.0039	-0.0012	0.0376	-0.0131	0.3998	0.5259729	Both
C	norb_Cluster169	0.0238	-0.0151	0.2815	0.2437	0.0133	0.073376	Both
C	norb_Cluster17	0.0028	-0.002	0.209	0.1674	0.0372	0.1345768	Both
C	norb_Cluster170	0.0243	-0.0104	0.112	0.0653	0.1381	0.2781028	Both
C	norb_Cluster171	0.0069	-0.0036	0.0942	0.0465	0.176	0.3166001	Both
C	norb_Cluster172	0.014	-0.0087	0.2111	0.1696	0.0361	0.1324216	Both
C	norb_Cluster175	0.0458	-0.0274	0.2204	0.1793	0.0318	0.122396	Both

C	norb_Cluster176	0.0298	-0.0187	0.2412	0.2013	0.0238	0.1023601	Both
C	norb_Cluster177	0.0282	-0.0146	0.1653	0.1213	0.0674	0.1905281	Both
C	norb_Cluster179	0.0022	-0.0012	0.077	0.0284	0.2232	0.3540646	Both
C	norb_Cluster18	0.0037	-0.0025	0.2513	0.2119	0.0206	0.0955612	Both
C	norb_Cluster180	0.0109	-0.0043	0.175	0.1316	0.0591	0.1763266	Both
C	norb_Cluster181	0.0008	0.0008	0.0916	0.0438	0.1824	0.3175024	Both
C	norb_Cluster182	0.0013	-0.0009	0.1091	0.0622	0.1436	0.2832086	Both
C	norb_Cluster184	0.0146	-0.0069	0.1796	0.1364	0.0556	0.1719199	Both
C	norb_Cluster186	0.0011	-1.00E-04	0.0011	-0.0515	0.889	0.9185574	Both
C	norb_Cluster19	0.0049	-0.002	0.1301	0.0844	0.1082	0.2512298	Both
C	norb_Cluster192	0.0173	-0.0105	0.2457	0.206	0.0223	0.099187	Both
C	norb_Cluster193	0.0021	-0.0002	0.0017	-0.0508	0.8593	0.9051724	Both
C	norb_Cluster197	0.0303	-0.0169	0.0466	-0.0036	0.3473	0.4713244	Both
C	norb_Cluster2	0.0031	0.00E+00	0.00E+00	-0.0526	0.9801	0.9834507	Both
C	norb_Cluster20	0.0587	-0.0242	0.2582	0.2192	0.0187	0.0910585	Both
C	norb_Cluster208	0.0011	-0.0008	0.0819	0.0336	0.2084	0.3434795	Both
C	norb_Cluster21	0.0481	-0.0251	0.3066	0.2701	0.0092	0.058734	Both
C	norb_Cluster211	0.0104	-0.0063	0.0698	0.0208	0.2473	0.3789917	Both
C	norb_Cluster212	0.0264	0.0019	0.0016	-0.0509	0.8633	0.9051724	Both
C	norb_Cluster214	0.0119	-0.0073	0.115	0.0684	0.1326	0.2755658	Both
C	norb_Cluster217	0.0149	-0.0063	0.0622	0.0129	0.2755	0.407168	Both
C	norb_Cluster219	0.0083	-0.0047	0.0778	0.0292	0.2209	0.3531643	Both
C	norb_Cluster22	0.0025	-0.0015	0.0869	0.0389	0.1944	0.3277415	Both
C	norb_Cluster220	0.0177	-0.0125	0.1046	0.0575	0.1527	0.292492	Both
C	norb_Cluster221	0.0177	-0.0121	0.0865	0.0384	0.1957	0.329031	Both
C	norb_Cluster222	0.0074	-0.0052	0.0668	0.0177	0.258	0.390277	Both
C	norb_Cluster224	0.0194	-0.0081	0.0554	0.0057	0.3043	0.4287248	Both
C	norb_Cluster225	0.0097	0.0009	0.003	-0.0495	0.8142	0.8686391	Both
C	norb_Cluster226	0.0043	-0.001	0.0188	-0.0328	0.5532	0.6431223	Both
C	norb_Cluster228	0.0031	0.0039	0.1151	0.0685	0.1325	0.2755658	Both
C	norb_Cluster229	0.0034	-0.0009	0.0321	-0.0189	0.4373	0.5548965	Both
C	norb_Cluster230	0.0014	-0.0009	0.0925	0.0448	0.18	0.3166001	Both
C	norb_Cluster231	0.0044	-0.0027	0.0974	0.0499	0.1685	0.308643	Both
C	norb_Cluster232	0.0031	-0.0019	0.1125	0.0657	0.1373	0.2775273	Both
C	norb_Cluster234	0.0121	-0.0084	0.1932	0.1507	0.0462	0.1523098	Both
C	norb_Cluster235	0.0009	-0.0006	0.101	0.0536	0.1605	0.3024531	Both
C	norb_Cluster238	0.0099	-0.0059	0.1111	0.0643	0.1398	0.2795189	Both
C	norb_Cluster239	0.0081	-0.0048	0.0919	0.0441	0.1817	0.3171991	Both
C	norb_Cluster24	0.2637	-0.1214	0.3206	0.2848	0.0075	0.0524733	Both
C	norb_Cluster244	0.0011	-0.0007	0.0763	0.0277	0.2255	0.3550269	Both
C	norb_Cluster247	0.0048	-0.0022	0.1664	0.1225	0.0664	0.1902681	Both
C	norb_Cluster25	0.1114	-0.0489	0.2382	0.1981	0.0248	0.1044898	Both
C	norb_Cluster250	0.0115	-0.0048	0.1255	0.0795	0.1151	0.2592577	Both
C	norb_Cluster251	0.0052	-0.0021	0.0849	0.0367	0.2	0.3334469	Both
C	norb_Cluster26	0.0931	-0.054	0.3463	0.3119	0.005	0.0399448	Both
C	norb_Cluster28	0.0302	-0.0087	0.0871	0.0391	0.194	0.3277415	Both
C	norb_Cluster29	0.222	-0.0617	0.2943	0.2572	0.0111	0.065219	Both
C	norb_Cluster31	0.0531	-0.0271	0.3055	0.269	0.0094	0.058734	Both
C	norb_Cluster32	0.1193	-0.0635	0.2575	0.2184	0.0189	0.0910585	Both
C	norb_Cluster33	0.4946	0.1073	0.1764	0.1331	0.058	0.1746134	Both
C	norb_Cluster35	0.0628	-0.0313	0.306	0.2694	0.0093	0.058734	Both
C	norb_Cluster36	1.1345	-0.1366	0.0281	-0.0231	0.4677	0.5740045	Both
C	norb_Cluster37	0.7473	-0.0423	0.0096	-0.0425	0.6724	0.7490315	Both
C	norb_Cluster38	0.0116	-0.0049	0.2344	0.1941	0.0262	0.1087306	Both
C	norb_Cluster39	0.0426	-0.0185	0.2233	0.1824	0.0305	0.1205815	Both

C	norb_Cluster40	0.186	-0.0876	0.2988	0.2619	0.0103	0.063564	Both
C	norb_Cluster42	0.2864	-0.0256	0.0169	-0.0349	0.5747	0.6598082	Both
C	norb_Cluster44	0.0182	-0.012	0.2763	0.2382	0.0144	0.0765584	Both
C	norb_Cluster46	0.2556	-0.0069	0.0011	-0.0515	0.8888	0.9185574	Both
C	norb_Cluster47	0.555	-0.2367	0.2947	0.2575	0.011	0.065219	Both
C	norb_Cluster5	0.01	-0.0033	0.1413	0.0961	0.0931	0.2241772	Both
C	norb_Cluster51	0.0092	-0.0058	0.1527	0.1081	0.0799	0.203954	Both
C	norb_Cluster52	0.2391	-0.1127	0.2859	0.2484	0.0125	0.0709418	Both
C	norb_Cluster56	0.014	-0.0076	0.2979	0.261	0.0105	0.0637694	Both
C	norb_Cluster57	0.0034	-0.0009	0.0487	-0.0013	0.3363	0.4614584	Both
C	norb_Cluster59	0.0236	-0.0131	0.1512	0.1065	0.0815	0.2047181	Both
C	norb_Cluster6	0.6862	-0.1132	0.0559	0.0062	0.3023	0.4287248	Both
C	norb_Cluster60	0.3199	-0.0869	0.0585	0.0089	0.2909	0.421646	Both
C	norb_Cluster61	0.482	-0.1372	0.1085	0.0616	0.1448	0.2837378	Both
C	norb_Cluster62	0.0024	0.0001	0.0016	-0.051	0.8637	0.9051724	Both
C	norb_Cluster64	0.0326	-0.0148	0.3159	0.2799	0.008	0.0524733	Both
C	norb_Cluster66	0.0012	-0.0008	0.0881	0.0401	0.1914	0.3254614	Both
C	norb_Cluster67	0.0218	-0.0117	0.2828	0.245	0.0131	0.0733389	Both
C	norb_Cluster68	0.0034	-0.0003	0.0066	-0.0456	0.7256	0.8002204	Both
C	norb_Cluster69	0.0028	-0.0018	0.1644	0.1205	0.0682	0.1912117	Both
C	norb_Cluster7	0.0709	-0.0239	0.1627	0.1187	0.0698	0.1914538	Both
C	norb_Cluster70	0.1051	-0.0436	0.2697	0.2312	0.0158	0.0813114	Both
C	norb_Cluster71	0.1295	-0.0431	0.2098	0.1682	0.0368	0.1339931	Both
C	norb_Cluster74	0.0327	-0.0134	0.2612	0.2223	0.0179	0.0887419	Both
C	norb_Cluster75	0.1291	-0.0525	0.3923	0.3603	0.0024	0.0243134	Both
C	norb_Cluster76	0.1105	-0.0332	0.0538	0.004	0.3119	0.437693	Both
C	norb_Cluster77	0.0475	-0.0084	0.0801	0.0317	0.2139	0.3476168	Both
C	norb_Cluster8	0.018	-0.0062	0.1155	0.0689	0.1317	0.2755658	Both
C	norb_Cluster80	0.0015	-0.0011	0.1098	0.063	0.1422	0.2815285	Both
C	norb_Cluster81	0.0012	-0.0001	0.004	-0.0484	0.7855	0.8489263	Both
C	norb_Cluster83	0.0078	-0.0057	0.1226	0.0764	0.1197	0.2675435	Both
C	norb_Cluster84	0.0082	-0.0029	0.0933	0.0456	0.1781	0.3166001	Both
C	norb_Cluster85	0.0023	-0.0009	0.0572	0.0076	0.2964	0.4275349	Both
C	norb_Cluster86	0.031	-0.0184	0.1001	0.0527	0.1624	0.3051016	Both
C	norb_Cluster88	0.0016	-0.0005	0.0239	-0.0275	0.5034	0.6036926	Both
C	norb_Cluster90	0.0009	-0.0006	0.0351	-0.0157	0.416	0.538528	Both
C	norb_Cluster91	0.6481	-0.2554	0.185	0.1421	0.0517	0.163854	Both
C	norb_Cluster92	0.0015	-0.0009	0.0488	-0.0013	0.336	0.4614584	Both
C	norb_Cluster93	0.0022	-0.0009	0.0336	-0.0173	0.4264	0.5421423	Both
C	norb_Cluster94	0.0017	-0.0003	0.0138	-0.0381	0.6124	0.6948089	Both
C	norb_Cluster96	0.0007	0.0002	0.0155	-0.0363	0.5908	0.6741929	Both
C	norb_Cluster97	0.0119	-0.0065	0.17	0.1263	0.0632	0.1847178	Both
C	norb_Cluster98	0.0132	-0.0079	0.1629	0.1188	0.0696	0.1914538	Both
C	nosz_Cluster0	0.0045	-0.0016	0.0791	0.0306	0.2169	0.3505864	Both
C	nosz_Cluster10	0.0136	0.0029	0.0287	-0.0224	0.463	0.5728206	Both
C	nosz_Cluster102	0.0044	-0.002	0.1997	0.1576	0.0423	0.1440231	Both
C	nosz_Cluster103	0.0455	-0.0095	0.0784	0.0299	0.2189	0.3508793	Both
C	nosz_Cluster105	0.0016	-0.0004	0.0245	-0.0268	0.4979	0.5994749	Both
C	nosz_Cluster106	0.0073	-0.0034	0.1406	0.0954	0.094	0.2253597	Both
C	nosz_Cluster107	0.0002	-0.0002	0.0256	-0.0257	0.4883	0.5903184	Both
C	nosz_Cluster108	0.004	-0.0002	0.0011	-0.0515	0.8883	0.9185574	Both
C	nosz_Cluster109	0.0375	-0.01	0.1893	0.1466	0.0487	0.1578445	Both
C	nosz_Cluster11	0.0129	0.0051	0.0412	-0.0093	0.3776	0.5039997	Both
C	nosz_Cluster110	0.0003	0.0004	0.0977	0.0502	0.1677	0.308643	Both
C	nosz_Cluster111	0.0061	-0.0026	0.1263	0.0803	0.1138	0.2580088	Both

C	nosz_Cluster116	0.0067	-0.0004	0.0029	-0.0496	0.8159	0.8689706	Both
C	nosz_Cluster118	0.0045	-0.0031	0.1547	0.1102	0.0777	0.2018145	Both
C	nosz_Cluster119	0.0013	-0.001	0.1179	0.0715	0.1275	0.2721499	Both
C	nosz_Cluster120	0.0071	-0.002	0.0671	0.018	0.2568	0.3895247	Both
C	nosz_Cluster123	0.0097	-0.0068	0.256	0.2168	0.0193	0.0917313	Both
C	nosz_Cluster128	0.0047	-0.0006	0.0067	-0.0456	0.7243	0.8002204	Both
C	nosz_Cluster129	0.0494	-0.0014	0.0034	-0.0491	0.8024	0.8607168	Both
C	nosz_Cluster13	0.0115	-0.0025	0.0361	-0.0146	0.4093	0.5342331	Both
C	nosz_Cluster130	0.089	-0.0456	0.0555	0.0057	0.3041	0.4287248	Both
C	nosz_Cluster132	0.0037	-0.001	0.0674	0.0183	0.2559	0.3891325	Both
C	nosz_Cluster14	0.024	0.0168	0.0788	0.0304	0.2176	0.3507553	Both
C	nosz_Cluster16	0.0076	0.0002	0.0002	-0.0524	0.9462	0.9658178	Both
C	nosz_Cluster17	0.0364	-0.0125	0.1626	0.1185	0.0699	0.1914538	Both
C	nosz_Cluster18	0.0161	-0.0033	0.0206	-0.0309	0.5345	0.6319462	Both
C	nosz_Cluster20	0.0091	-0.0052	0.1415	0.0963	0.0929	0.2241772	Both
C	nosz_Cluster21	0.0083	-0.0059	0.1993	0.1572	0.0425	0.1440231	Both
C	nosz_Cluster22	0.0267	-0.011	0.0708	0.0219	0.2435	0.3751636	Both
C	nosz_Cluster23	0.0141	-0.0092	0.2225	0.1816	0.0309	0.1205815	Both
C	nosz_Cluster24	0.0013	-0.0008	0.0722	0.0234	0.2389	0.3699188	Both
C	nosz_Cluster25	0.0029	0.0008	0.0167	-0.0351	0.5769	0.6608919	Both
C	nosz_Cluster26	0.0054	-0.0025	0.089	0.0411	0.1889	0.3240348	Both
C	nosz_Cluster27	0.0053	-0.0028	0.1547	0.1102	0.0778	0.2018145	Both
C	nosz_Cluster28	0.0303	-0.0139	0.2049	0.163	0.0394	0.1399365	Both
C	nosz_Cluster30	0.0276	-0.0102	0.1283	0.0825	0.1108	0.255394	Both
C	nosz_Cluster32	0.0137	-0.003	0.0392	-0.0114	0.3896	0.5165322	Both
C	nosz_Cluster33	0.018	-0.001	0.0013	-0.0512	0.8749	0.9120105	Both
C	nosz_Cluster34	0.0377	-0.0033	0.004	-0.0484	0.7856	0.8489263	Both
C	nosz_Cluster35	0.0075	-0.0037	0.1512	0.1065	0.0815	0.2047181	Both
C	nosz_Cluster36	0.0179	-0.0056	0.0729	0.0241	0.2365	0.3681325	Both
C	nosz_Cluster37	0.006	-0.0026	0.0992	0.0518	0.1643	0.3077103	Both
C	nosz_Cluster38	0.0128	-0.0025	0.0199	-0.0316	0.5414	0.6350466	Both
C	nosz_Cluster39	0.0537	-0.0212	0.1609	0.1168	0.0715	0.1934826	Both
C	nosz_Cluster40	0.0218	-0.0065	0.0628	0.0135	0.2732	0.4051947	Both
C	nosz_Cluster41	0.0203	-0.0099	0.1622	0.1181	0.0703	0.1914538	Both
C	nosz_Cluster42	0.0279	-0.0045	0.019	-0.0326	0.551	0.642528	Both
C	nosz_Cluster43	0.0007	-0.0005	0.0495	-0.0006	0.3325	0.4614584	Both
C	nosz_Cluster44	0.0057	-0.0026	0.114	0.0674	0.1344	0.2775273	Both
C	nosz_Cluster45	0.0045	-0.0017	0.0706	0.0217	0.2442	0.3752696	Both
C	nosz_Cluster46	0.007	-0.002	0.0761	0.0275	0.2262	0.3550269	Both
C	nosz_Cluster47	0.0008	-0.0006	0.0972	0.0496	0.169	0.308643	Both
C	nosz_Cluster48	0.0023	-0.001	0.0498	-0.0002	0.3308	0.4603685	Both
C	nosz_Cluster49	0.0014	0.0005	0.0396	-0.011	0.3872	0.5145672	Both
C	nosz_Cluster5	0.0045	0.0009	0.0102	-0.0419	0.6636	0.7429673	Both
C	nosz_Cluster51	0.0021	-0.0013	0.1116	0.0648	0.1389	0.2786775	Both
C	nosz_Cluster53	0.0009	-0.0005	0.0354	-0.0153	0.4138	0.5376991	Both
C	nosz_Cluster54	0.0026	-0.0014	0.0761	0.0274	0.2263	0.3550269	Both
C	nosz_Cluster57	0.1757	-0.0919	0.262	0.2232	0.0177	0.0884367	Both
C	nosz_Cluster58	0.0043	-0.0025	0.1261	0.0801	0.1141	0.2580088	Both
C	nosz_Cluster59	0.0009	1.00E-04	0.0003	-0.0523	0.9403	0.9615099	Both
C	nosz_Cluster6	0.0032	-0.0003	0.0035	-0.0489	0.798	0.8591385	Both
C	nosz_Cluster60	0.0079	-0.0031	0.0849	0.0367	0.2001	0.3334469	Both
C	nosz_Cluster61	0.0178	-0.0039	0.105	0.0579	0.1518	0.292492	Both
C	nosz_Cluster62	0.0413	-0.0175	0.1718	0.1283	0.0617	0.1819671	Both
C	nosz_Cluster64	0.0016	-0.0007	0.0196	-0.032	0.5449	0.636948	Both
C	nosz_Cluster65	0.0065	-0.0025	0.0764	0.0278	0.2251	0.3550269	Both

C	nosz_Cluster66	0.0324	-0.0202	0.2435	0.2037	0.023	0.1009186	Both
C	nosz_Cluster67	0.0521	-0.0275	0.1918	0.1493	0.0471	0.1542322	Both
C	nosz_Cluster69	0.0061	-0.0035	0.1962	0.1539	0.0443	0.1470069	Both
C	nosz_Cluster70	0.0479	-0.0313	0.1129	0.0662	0.1364	0.2775273	Both
C	nosz_Cluster71	0.0083	-0.0029	0.0527	0.0028	0.317	0.4421137	Both
C	nosz_Cluster72	0.0506	-0.028	0.2433	0.2034	0.0231	0.1009186	Both
C	nosz_Cluster73	0.003	-0.0003	0.0028	-0.0497	0.8202	0.8719619	Both
C	nosz_Cluster74	0.0017	-0.0006	0.034	-0.0169	0.4239	0.5419068	Both
C	nosz_Cluster75	0.0967	-0.0651	0.0682	0.0191	0.253	0.3856725	Both
C	nosz_Cluster77	0.0002	-0.0001	0.0201	-0.0315	0.5403	0.6350297	Both
C	nosz_Cluster78	0.0044	-0.0019	0.1198	0.0734	0.1244	0.2717916	Both
C	nosz_Cluster79	0.0333	-0.0118	0.1561	0.1117	0.0763	0.2018145	Both
C	nosz_Cluster8	0.0099	-0.0019	0.0409	-0.0096	0.3795	0.5054486	Both
C	nosz_Cluster80	0.0045	0.0014	0.0303	-0.0207	0.4501	0.5650753	Both
C	nosz_Cluster81	0.0079	-0.0041	0.081	0.0327	0.2111	0.346913	Both
C	nosz_Cluster82	0.0016	-0.0008	0.0169	-0.0349	0.5748	0.6598082	Both
C	nosz_Cluster83	0.0015	-0.0002	0.003	-0.0495	0.8131	0.8686391	Both
C	nosz_Cluster85	0.0022	-0.0006	0.0174	-0.0343	0.5688	0.6567811	Both
C	nosz_Cluster86	0.0027	-0.0016	0.1089	0.062	0.144	0.2832086	Both
C	nosz_Cluster87	0.003	-0.0004	0.0104	-0.0417	0.6597	0.7399222	Both
C	nosz_Cluster88	0.0218	-0.0058	0.1533	0.1087	0.0792	0.203954	Both
C	nosz_Cluster89	0.0466	-0.0275	0.2707	0.2323	0.0156	0.0813114	Both
C	nosz_Cluster9	0.003	-0.0008	0.0209	-0.0306	0.5318	0.6300736	Both
C	nosz_Cluster90	0.0116	-0.0044	0.2142	0.1729	0.0346	0.1276302	Both
C	nosz_Cluster91	0.0189	-0.0051	0.0443	-0.006	0.3598	0.4851667	Both
C	nosz_Cluster93	0.0393	-0.0173	0.2	0.1579	0.0421	0.1440231	Both
C	nosz_Cluster95	0.007	-0.0032	0.1491	0.1044	0.0838	0.2085516	Both
C	nosz_Cluster96	0.0066	0.0012	0.0344	-0.0164	0.4207	0.5419019	Both
C	nosz_Cluster97	0.0019	-0.0012	0.119	0.0726	0.1257	0.2721499	Both
C	nosz_Cluster98	0.0025	-0.0012	0.0203	-0.0313	0.5382	0.633867	Both
C	nosz_Cluster99	0.0055	-0.0003	0.002	-0.0505	0.8467	0.8952367	Both
C	nrfa_Cluster10	0.0804	-0.0309	0.0948	0.0472	0.1744	0.3166001	Both
C	nrfa_Cluster100	0.0963	-0.0537	0.1442	0.0991	0.0896	0.2192606	Both
C	nrfa_Cluster104	0.1143	-0.0634	0.3206	0.2849	0.0074	0.0524733	Both
C	nrfa_Cluster105	0.0062	0.0004	0.0014	-0.0511	0.8704	0.9089555	Both
C	nrfa_Cluster109	0.2294	-0.0888	0.1881	0.1454	0.0495	0.159515	Both
C	nrfa_Cluster116	0.2592	-0.1335	0.5089	0.483	0.0003	0.005962	Both
C	nrfa_Cluster118	0.173	-0.1048	0.1388	0.0935	0.0962	0.2298258	Both
C	nrfa_Cluster125	0.0809	-0.0479	0.1178	0.0713	0.1277	0.2721499	Both
C	nrfa_Cluster154	0.0658	-0.0321	0.1203	0.0739	0.1235	0.2717916	Both
C	nrfa_Cluster164	0.0045	0.0003	0.0012	-0.0514	0.8815	0.9156884	Both
C	nrfa_Cluster172	0.0026	-0.0019	0.1185	0.0721	0.1266	0.2721499	Both
C	nrfa_Cluster175	0.0044	-0.0017	0.0348	-0.016	0.418	0.5396857	Both
C	nrfa_Cluster20	0.035	-0.0182	0.1671	0.1233	0.0657	0.1892207	Both
C	nrfa_Cluster26	0.1078	-0.0664	0.1586	0.1144	0.0737	0.1970812	Both
C	nrfa_Cluster29	0.0048	-0.002	0.0628	0.0134	0.2733	0.4051947	Both
C	nrfa_Cluster34	0.0487	-0.0285	0.2066	0.1649	0.0384	0.1374498	Both
C	nrfa_Cluster38	0.0031	-0.0009	0.0261	-0.0252	0.4843	0.5879141	Both
C	nrfa_Cluster61	0.0036	-0.0024	0.1497	0.1049	0.0832	0.2079868	Both
C	nrfa_Cluster62	0.0452	-0.0267	0.1213	0.075	0.1218	0.2712779	Both
C	nrfa_Cluster66	0.1082	-0.0443	0.249	0.2095	0.0213	0.0973797	Both
C	nrfa_Cluster69	0.003	-0.0009	0.0365	-0.0142	0.4066	0.5319784	Both
C	nrfa_Cluster71	0.027	-0.0085	0.0452	-0.005	0.3546	0.4798511	Both
C	nrfa_Cluster72	0.0732	-0.0426	0.1857	0.1429	0.0511	0.163854	Both
C	nrfa_Cluster73	0.0038	-0.0007	0.0107	-0.0414	0.6554	0.7365116	Both

C	nrfa_Cluster74	0.1331	-0.0667	0.1452	0.1002	0.0883	0.2171829	Both
C	nrfa_Cluster76	0.0755	-0.0298	0.0773	0.0287	0.2224	0.3537468	Both
C	nrfa_Cluster79	0.0229	0.0162	0.0658	0.0166	0.2618	0.3950045	Both
C	nrfa_Cluster87	0.017	-0.0069	0.0471	-0.0031	0.3447	0.4697358	Both
C	nrfa_Cluster89	0.0018	-0.0009	0.0537	0.0039	0.3123	0.437693	Both
C	nrfa_Cluster92	0.0043	0.0004	0.0024	-0.0501	0.8331	0.8824609	Both
C	nrfa_Cluster93	0.0428	-0.027	0.1161	0.0696	0.1307	0.2755658	Both
C	nrfa_Cluster94	0.0002	-0.0002	0.0282	-0.0229	0.4668	0.5740045	Both
C	nrfa_Cluster97	0.0168	-0.0098	0.2396	0.1996	0.0243	0.103151	Both
C	nrfa_Cluster99	0.014	-0.0071	0.155	0.1105	0.0775	0.2018145	Both
N	amoA_A_Cluster26	0.004	-0.0035	0.21	0.1684	0.0367	0.1085624	Both
N	amoA_A_Cluster45	0.0051	-0.0044	0.2633	0.2246	0.0174	0.0731475	Both
N	amoA_B_Cluster0	0.1099	-0.0379	0.5575	0.5342	0.0001	0.0031571	Both
N	amoA_B_Cluster1	0.0172	-0.0089	0.2678	0.2293	0.0163	0.0722025	Both
N	amoA_B_Cluster10	0.0422	-0.0238	0.5977	0.5765	0.00E+00	0.0015632	Both
N	amoA_B_Cluster11	0.0377	-0.0195	0.3699	0.3367	0.0034	0.0294593	Both
N	amoA_B_Cluster12	0.0807	-0.0296	0.4848	0.4576	0.0005	0.0081394	Both
N	amoA_B_Cluster13	0.5271	-0.2478	0.3742	0.3413	0.0032	0.0291453	Both
N	amoA_B_Cluster14	0.0275	-0.0151	0.2721	0.2338	0.0153	0.0699669	Both
N	amoA_B_Cluster15	0.0318	-0.0159	0.3256	0.2901	0.0069	0.0421282	Both
N	amoA_B_Cluster2	0.1721	-0.0888	0.4335	0.4037	0.0012	0.0150685	Both
N	amoA_B_Cluster20	0.0058	-0.0037	0.1674	0.1236	0.0655	0.1533953	Both
N	amoA_B_Cluster23	0.0507	-0.0224	0.4095	0.3784	0.0018	0.019474	Both
N	amoA_B_Cluster3	0.0182	-0.0084	0.1808	0.1377	0.0546	0.1423311	Both
N	amoA_B_Cluster4	0.0125	-0.007	0.2225	0.1816	0.0309	0.099485	Both
N	amoA_B_Cluster5	0.0928	-0.0323	0.4538	0.4251	0.0008	0.0126036	Both
N	amoA_B_Cluster6	0.0233	-0.009	0.2776	0.2396	0.0141	0.0661116	Both
N	amoA_B_Cluster7	0.0179	-0.0078	0.1587	0.1145	0.0736	0.1639541	Both
N	amoA_B_Cluster8	0.0207	-0.0092	0.1593	0.1151	0.0731	0.163893	Both
N	amoA_B_Cluster9	0.0549	-0.0262	0.2577	0.2186	0.0188	0.0771314	Both
N	nifh_Cluster0	0.0332	0.004	0.0642	0.015	0.2677	0.3779065	Both
N	nifh_Cluster10	0.005	0.0026	0.0615	0.0121	0.2783	0.3873117	Both
N	nifh_Cluster100	0.0019	-0.0002	0.0017	-0.0508	0.8583	0.8883673	Both
N	nifh_Cluster103	0.0383	-0.0129	0.2481	0.2085	0.0216	0.0780373	Both
N	nifh_Cluster1034	0.0262	-0.0143	0.2218	0.1809	0.0312	0.099485	Both
N	nifh_Cluster105	0.013	-0.0071	0.2115	0.1701	0.0359	0.1081123	Both
N	nifh_Cluster108	0.0083	-0.0018	0.0429	-0.0075	0.3679	0.4719126	Both
N	nifh_Cluster1099	0.066	0.0426	0.4216	0.3911	0.0014	0.0166043	Both
N	nifh_Cluster11	0.0021	-0.0011	0.1194	0.073	0.125	0.2248317	Both
N	nifh_Cluster1112	0.0029	-0.0012	0.0623	0.013	0.2751	0.3855537	Both
N	nifh_Cluster112	0.0112	0.0061	0.2655	0.2268	0.0168	0.0724573	Both
N	nifh_Cluster113	0.0327	-0.009	0.195	0.1526	0.0451	0.1237339	Both
N	nifh_Cluster114	0.0332	0.0028	0.0414	-0.009	0.3762	0.4763195	Both
N	nifh_Cluster1141	0.0251	-0.0119	0.252	0.2126	0.0204	0.0777753	Both
N	nifh_Cluster115	0.0095	1.00E-04	0.00E+00	-0.0526	0.9773	0.9839992	Both
N	nifh_Cluster116	0.0775	-0.0238	0.2523	0.2129	0.0203	0.0777753	Both
N	nifh_Cluster1163	0.0044	-0.0014	0.0867	0.0387	0.195	0.3004101	Both
N	nifh_Cluster1179	0.0031	-0.0014	0.0653	0.0161	0.2636	0.3730233	Both
N	nifh_Cluster1182	0.0204	0.0006	0.0045	-0.0479	0.7722	0.8223867	Both
N	nifh_Cluster1183	0.0066	0.0038	0.3909	0.3589	0.0024	0.0247217	Both
N	nifh_Cluster1197	0.0115	-0.0026	0.0654	0.0162	0.2631	0.3730233	Both
N	nifh_Cluster1199	0.0046	-0.0003	0.0035	-0.0489	0.7977	0.8464775	Both
N	nifh_Cluster120	0.0149	-0.0114	0.1606	0.1164	0.0718	0.1629222	Both
N	nifh_Cluster1203	0.0155	-0.0097	0.2429	0.2031	0.0232	0.0829592	Both
N	nifh_Cluster1206	0.0043	-0.0023	0.1817	0.1386	0.054	0.1423311	Both

N	nifh_Cluster1207	0.0074	-0.0033	0.1173	0.0708	0.1286	0.2265521	Both
N	nifh_Cluster1209	0.1506	-0.0121	0.0473	-0.0028	0.3436	0.4484401	Both
N	nifh_Cluster121	0.0095	-0.0067	0.1516	0.1069	0.0811	0.1720885	Both
N	nifh_Cluster1211	0.0056	-0.002	0.1067	0.0597	0.1483	0.2514749	Both
N	nifh_Cluster1213	0.0026	-0.0012	0.1138	0.0672	0.1347	0.2330685	Both
N	nifh_Cluster122	0.0029	-0.0027	0.2668	0.2283	0.0165	0.0724573	Both
N	nifh_Cluster123	0.0095	-0.0005	0.0008	-0.0518	0.9017	0.9220277	Both
N	nifh_Cluster1236	0.0234	-0.014	0.1437	0.0987	0.0901	0.1826699	Both
N	nifh_Cluster1238	0.0028	-0.001	0.0479	-0.0022	0.3405	0.4468897	Both
N	nifh_Cluster124	0.0091	-0.0014	0.0244	-0.027	0.4991	0.5924809	Both
N	nifh_Cluster1261	0.002	-0.0007	0.0355	-0.0152	0.4133	0.506975	Both
N	nifh_Cluster1262	0.0065	-0.0014	0.025	-0.0263	0.4937	0.5872845	Both
N	nifh_Cluster1266	0.0094	0.0035	0.1201	0.0738	0.1238	0.2234622	Both
N	nifh_Cluster1267	0.0562	0.0466	0.5815	0.5595	1.00E-04	0.0020291	Both
N	nifh_Cluster127	0.0026	-0.0021	0.1676	0.1238	0.0653	0.1533953	Both
N	nifh_Cluster1278	0.1602	-0.0942	0.516	0.4906	0.0002	0.0055497	Both
N	nifh_Cluster128	0.0036	-0.0016	0.1016	0.0543	0.159	0.2608257	Both
N	nifh_Cluster1289	0.0039	0.0002	0.0011	-0.0515	0.8862	0.9139271	Both
N	nifh_Cluster129	0.0077	-0.0024	0.0367	-0.014	0.4056	0.5005999	Both
N	nifh_Cluster1292	0.0086	-0.0039	0.1502	0.1055	0.0826	0.1733464	Both
N	nifh_Cluster130	0.0059	-0.0032	0.0714	0.0226	0.2415	0.3491944	Both
N	nifh_Cluster1305	0.0048	0.0002	0.0009	-0.0516	0.8947	0.9189327	Both
N	nifh_Cluster1306	0.005	-0.0018	0.0901	0.0422	0.1862	0.2913336	Both
N	nifh_Cluster131	0.0051	-0.0038	0.1794	0.1362	0.0557	0.1435878	Both
N	nifh_Cluster1319	0.0036	-0.0016	0.0757	0.0271	0.2274	0.3358138	Both
N	nifh_Cluster132	0.0064	-0.0008	0.008	-0.0442	0.6994	0.7613823	Both
N	nifh_Cluster1320	0.0025	-0.0005	0.0095	-0.0426	0.6744	0.7395591	Both
N	nifh_Cluster1323	0.0023	1.00E-04	0.0003	-0.0523	0.9398	0.9498525	Both
N	nifh_Cluster1324	0.03	0.0114	0.1754	0.132	0.0588	0.1454837	Both
N	nifh_Cluster1336	0.0153	-0.0005	0.0034	-0.0491	0.8024	0.8499749	Both
N	nifh_Cluster1340	0.0363	-0.0108	0.0452	-0.0051	0.3549	0.4591917	Both
N	nifh_Cluster1345	0.0483	0.0372	0.5004	0.4741	0.0003	0.0065917	Both
N	nifh_Cluster1370	0.0021	-0.0006	0.0329	-0.018	0.4315	0.5253934	Both
N	nifh_Cluster1375	0.0018	0.0006	0.0535	0.0037	0.3131	0.4203294	Both
N	nifh_Cluster139	0.0041	0.0005	0.0058	-0.0465	0.7432	0.7962258	Both
N	nifh_Cluster140	0.0043	-0.0018	0.1244	0.0783	0.1169	0.2162296	Both
N	nifh_Cluster141	0.0677	-0.0347	0.5908	0.5692	0.00E+00	0.0017301	Both
N	nifh_Cluster148	0.0046	-0.0023	0.0728	0.0241	0.2367	0.3448358	Both
N	nifh_Cluster1480	0.001	0.0011	0.2975	0.2605	0.0105	0.0545445	Both
N	nifh_Cluster152	0.0599	-0.0026	0.0058	-0.0466	0.7436	0.7962258	Both
N	nifh_Cluster156	0.0659	0.044	0.7885	0.7774	0.00E+00	1.54E-05	Both
N	nifh_Cluster1562	0.0543	0.0597	0.8175	0.8079	0.00E+00	5.60E-06	Both
N	nifh_Cluster158	0.0144	-0.0074	0.1887	0.146	0.0491	0.1322086	Both
N	nifh_Cluster16	0.0019	-0.001	0.059	0.0095	0.2887	0.3989189	Both
N	nifh_Cluster166	0.0172	-0.0009	0.0009	-0.0517	0.8956	0.9189327	Both
N	nifh_Cluster205	0.0122	-1.00E-04	0.00E+00	-0.0526	0.9773	0.9839992	Both
N	nifh_Cluster21	0.0021	-0.0013	0.1287	0.0829	0.1102	0.2101965	Both
N	nifh_Cluster222	0.006	-0.0025	0.05	0.00E+00	0.3301	0.4376093	Both
N	nifh_Cluster225	0.0189	-0.0024	0.0356	-0.0151	0.4125	0.506975	Both
N	nifh_Cluster230	0.0042	-0.002	0.1518	0.1071	0.0808	0.1720885	Both
N	nifh_Cluster231	0.0022	0.0004	0.0111	-0.0409	0.6488	0.7264096	Both
N	nifh_Cluster236	0.0027	-0.0019	0.2578	0.2187	0.0188	0.0771314	Both
N	nifh_Cluster237	0.0058	-0.0025	0.1471	0.1022	0.0861	0.1769758	Both
N	nifh_Cluster243	0.0333	0.0155	0.2217	0.1808	0.0312	0.099485	Both
N	nifh_Cluster2432	0.0436	0.0327	0.5572	0.5339	0.0001	0.0031571	Both

N	nifh_Cluster2433	0.0032	0.0007	0.0128	-0.0392	0.6255	0.7120944	Both
N	nifh_Cluster246	0.0057	-0.0032	0.1512	0.1065	0.0815	0.1723235	Both
N	nifh_Cluster25	0.0295	0.0151	0.434	0.4042	0.0012	0.0150685	Both
N	nifh_Cluster256	0.0029	0.0002	0.0027	-0.0498	0.8237	0.861724	Both
N	nifh_Cluster264	0.0029	0.0004	0.0096	-0.0426	0.673	0.7395426	Both
N	nifh_Cluster265	0.0736	0.0098	0.075	0.0264	0.2295	0.3375817	Both
N	nifh_Cluster267	0.0109	0.0067	0.2763	0.2382	0.0144	0.0669044	Both
N	nifh_Cluster268	0.0111	0.0012	0.0205	-0.031	0.5358	0.6309604	Both
N	nifh_Cluster269	0.023	-0.0089	0.1158	0.0693	0.1311	0.2288737	Both
N	nifh_Cluster272	0.0041	0.0052	0.5422	0.5181	0.0001	0.0037902	Both
N	nifh_Cluster274	0.0032	0.0029	0.0778	0.0293	0.2208	0.3306443	Both
N	nifh_Cluster275	0.0036	-0.0019	0.1685	0.1247	0.0646	0.1524487	Both
N	nifh_Cluster278	0.0077	0.0053	0.3496	0.3154	0.0048	0.035988	Both
N	nifh_Cluster279	0.006	0.0006	0.011	-0.0411	0.6514	0.7278709	Both
N	nifh_Cluster28	0.002	0.0003	0.01	-0.0421	0.6663	0.7389029	Both
N	nifh_Cluster280	0.0031	-0.0006	0.0108	-0.0413	0.6542	0.7295817	Both
N	nifh_Cluster282	0.0052	-0.0006	0.0135	-0.0385	0.6166	0.706247	Both
N	nifh_Cluster287	0.0022	-0.0007	0.0383	-0.0123	0.3951	0.4938877	Both
N	nifh_Cluster290	0.011	0.0016	0.021	-0.0305	0.531	0.6265728	Both
N	nifh_Cluster297	0.0029	-0.0026	0.1362	0.0908	0.0997	0.1953425	Both
N	nifh_Cluster299	0.0026	0.0005	0.0099	-0.0422	0.6684	0.7395426	Both
N	nifh_Cluster303	0.002	0.0004	0.0128	-0.0392	0.6253	0.7120944	Both
N	nifh_Cluster304	0.0024	-0.0007	0.0394	-0.0112	0.3886	0.4877571	Both
N	nifh_Cluster313	0.0103	-0.0063	0.1283	0.0824	0.1109	0.2102707	Both
N	nifh_Cluster314	0.0022	-0.001	0.0736	0.0248	0.2342	0.3420503	Both
N	nifh_Cluster317	0.0071	-0.0036	0.1944	0.152	0.0455	0.1241576	Both
N	nifh_Cluster32	0.0033	-0.0007	0.0265	-0.0247	0.4804	0.5761482	Both
N	nifh_Cluster320	12.7419	-2.7551	0.7701	0.758	0.00E+00	2.06E-05	Both
N	nifh_Cluster334	0.0063	-0.0046	0.14	0.0948	0.0947	0.1908336	Both
N	nifh_Cluster335	0.0075	0.0021	0.0159	-0.0359	0.5858	0.6830295	Both
N	nifh_Cluster338	0.0123	-0.0043	0.1469	0.102	0.0864	0.1769758	Both
N	nifh_Cluster340	0.007	0.0051	0.0769	0.0283	0.2236	0.3330875	Both
N	nifh_Cluster341	0.0023	0.0009	0.0568	0.0072	0.2981	0.4061302	Both
N	nifh_Cluster35	0.0078	-0.0014	0.0369	-0.0138	0.4042	0.5005989	Both
N	nifh_Cluster351	0.0025	-0.0021	0.1857	0.1428	0.0511	0.1369237	Both
N	nifh_Cluster3574	0.1093	-0.014	0.0535	0.0036	0.3132	0.4203294	Both
N	nifh_Cluster3691	0.0174	-0.0083	0.1353	0.0898	0.1009	0.1971558	Both
N	nifh_Cluster37	0.0329	-0.0118	0.1014	0.0541	0.1595	0.2608257	Both
N	nifh_Cluster374	0.0021	-0.0008	0.0422	-0.0082	0.3719	0.4748885	Both
N	nifh_Cluster38	0.0009	0.0001	0.003	-0.0494	0.8126	0.8530346	Both
N	nifh_Cluster382	0.0016	-0.0015	0.1122	0.0655	0.1377	0.2354562	Both
N	nifh_Cluster384	0.0019	-0.001	0.0966	0.0491	0.1702	0.2697022	Both
N	nifh_Cluster386	0.0035	-0.0015	0.055	0.0052	0.3063	0.4153915	Both
N	nifh_Cluster389	0.0031	-0.0005	0.008	-0.0442	0.6994	0.7613823	Both
N	nifh_Cluster394	0.002	-0.0017	0.1201	0.0738	0.1239	0.2234622	Both
N	nifh_Cluster40	0.0012	-0.0009	0.1523	0.1076	0.0803	0.1720885	Both
N	nifh_Cluster43	0.0108	0.0004	0.0023	-0.0502	0.8376	0.8715497	Both
N	nifh_Cluster434	0.0035	-0.0023	0.1446	0.0996	0.0891	0.1811826	Both
N	nifh_Cluster440	0.0133	-0.0079	0.2075	0.1658	0.038	0.1109548	Both
N	nifh_Cluster462	0.0034	-0.0025	0.185	0.1421	0.0517	0.1373046	Both
N	nifh_Cluster470	0.0152	-0.0012	0.0073	-0.0449	0.7123	0.7725272	Both
N	nifh_Cluster49	0.0213	-0.0056	0.0276	-0.0236	0.4719	0.5670413	Both
N	nifh_Cluster499	0.0026	-0.0009	0.0486	-0.0015	0.337	0.4438016	Both
N	nifh_Cluster5	0.0256	0.0014	0.0114	-0.0406	0.6449	0.7234228	Both
N	nifh_Cluster506	0.0048	-0.0003	0.0032	-0.0493	0.8076	0.8511321	Both

N	nifh_Cluster51	0.0028	-0.001	0.0368	-0.0139	0.4047	0.5005989	Both
N	nifh_Cluster52	0.0336	-0.015	0.1165	0.07	0.1299	0.228155	Both
N	nifh_Cluster525	0.0033	-0.002	0.0599	0.0104	0.2849	0.3955568	Both
N	nifh_Cluster531	0.0258	0.0253	0.8314	0.8225	0.00E+00	5.24E-06	Both
N	nifh_Cluster539	0.0048	-0.0022	0.0395	-0.0111	0.3878	0.4877571	Both
N	nifh_Cluster578	0.0481	-0.0181	0.2399	0.1999	0.0242	0.0845481	Both
N	nifh_Cluster58	0.3542	0.2763	0.6142	0.5939	0.00E+00	0.0011958	Both
N	nifh_Cluster60	0.0089	-0.0044	0.153	0.1085	0.0795	0.1720885	Both
N	nifh_Cluster61	0.0025	0.0008	0.0424	-0.008	0.3704	0.4740607	Both
N	nifh_Cluster65	0.009	-0.0009	0.0054	-0.047	0.7519	0.8021847	Both
N	nifh_Cluster686	0.0009	-0.0004	0.0467	-0.0035	0.3468	0.451634	Both
N	nifh_Cluster69	0.009	-0.0036	0.0836	0.0353	0.2038	0.3114515	Both
N	nifh_Cluster70	0.0111	-0.0078	0.1371	0.0916	0.0985	0.1944292	Both
N	nifh_Cluster717	0.003	0.0017	0.1929	0.1504	0.0464	0.1255179	Both
N	nifh_Cluster725	0.0058	0.0041	0.3763	0.3434	0.0031	0.028617	Both
N	nifh_Cluster727	0.0039	-0.0014	0.0618	0.0125	0.2771	0.3864745	Both
N	nifh_Cluster73	0.0106	-0.0058	0.1755	0.1321	0.0587	0.1454837	Both
N	nifh_Cluster74	0.0049	-0.0007	0.0059	-0.0464	0.7412	0.7962258	Both
N	nifh_Cluster748	0.0081	-0.0028	0.1525	0.1079	0.0801	0.1720885	Both
N	nifh_Cluster749	0.0071	0.0025	0.1304	0.0847	0.1077	0.2070181	Both
N	nifh_Cluster755	0.0102	-0.0047	0.1308	0.0851	0.1071	0.2066638	Both
N	nifh_Cluster76	0.0029	-0.0005	0.0125	-0.0395	0.6301	0.7122085	Both
N	nifh_Cluster79	0.0022	-0.0003	0.0085	-0.0437	0.6913	0.7552866	Both
N	nifh_Cluster81	0.0055	-0.0012	0.015	-0.0368	0.5966	0.691512	Both
N	nifh_Cluster82	0.0011	-0.0005	0.0465	-0.0036	0.3476	0.4517237	Both
N	nifh_Cluster83	0.0069	0.0018	0.0328	-0.0181	0.4319	0.5253934	Both
N	nifh_Cluster86	0.0135	-0.0053	0.1652	0.1212	0.0675	0.155633	Both
N	nifh_Cluster868	0.0099	0.0004	0.0028	-0.0497	0.8208	0.8602136	Both
N	nifh_Cluster87	0.0024	-0.0014	0.0998	0.0524	0.1631	0.2610501	Both
N	nifh_Cluster88	0.0039	-0.0019	0.0803	0.0319	0.2132	0.3225137	Both
N	nifh_Cluster9	0.0078	-0.0002	0.0004	-0.0522	0.9306	0.9450025	Both
N	nifh_Cluster92	0.0051	-0.0037	0.1462	0.1012	0.0872	0.177995	Both
N	nifh_Cluster93	0.0036	-0.0001	0.0005	-0.0521	0.9256	0.9415493	Both
N	nifh_Cluster94	0.0047	-0.0013	0.0231	-0.0284	0.5112	0.6056231	Both
N	nifh_Cluster95	0.0121	-0.0053	0.0868	0.0387	0.1949	0.3004101	Both
N	nifh_Cluster96	0.0123	0.0007	0.0032	-0.0493	0.8079	0.8511321	Both
N	nifh_Cluster97	0.018	0.0094	0.2116	0.1701	0.0359	0.1081123	Both
N	nifh_Cluster98	0.0059	0.0007	0.0125	-0.0395	0.6294	0.7122085	Both
N	nifh_Cluster99	0.0033	-0.0009	0.0259	-0.0254	0.4861	0.5817132	Both
N	nirk_Cluster0	5.9009	-1.2183	0.6618	0.644	0.00E+00	0.0004305	Both
N	nirk_Cluster1	0.1708	-0.0954	0.4869	0.4598	0.0004	0.0080608	Both
N	nirk_Cluster10	0.5713	-0.1479	0.3352	0.3002	0.006	0.0410521	Both
N	nirk_Cluster101	0.0031	-0.0012	0.057	0.0074	0.2973	0.4061302	Both
N	nirk_Cluster102	0.0426	-0.024	0.4121	0.3812	0.0017	0.0189842	Both
N	nirk_Cluster103	0.0754	-0.0385	0.4438	0.4145	0.001	0.0140674	Both
N	nirk_Cluster104	0.0023	-0.0013	0.0916	0.0438	0.1824	0.2869715	Both
N	nirk_Cluster105	0.0946	-0.052	0.5313	0.5066	0.0002	0.0045302	Both
N	nirk_Cluster106	0.0592	-0.0321	0.2979	0.2609	0.0105	0.0545445	Both
N	nirk_Cluster107	0.0891	-0.047	0.4292	0.3992	0.0013	0.01559	Both
N	nirk_Cluster108	0.0151	-0.0058	0.1372	0.0918	0.0983	0.1944292	Both
N	nirk_Cluster109	0.0115	-0.008	0.3886	0.3564	0.0025	0.0249399	Both
N	nirk_Cluster11	0.1401	-0.0174	0.2501	0.2106	0.021	0.0777753	Both
N	nirk_Cluster12	0.094	-0.0302	0.4337	0.4039	0.0012	0.0150685	Both
N	nirk_Cluster13	0.0408	-0.0021	0.0185	-0.0332	0.5566	0.6541697	Both
N	nirk_Cluster14	0.0946	-0.0323	0.4741	0.4464	0.0006	0.0094115	Both

N	nirk_Cluster15	0.3358	-0.1797	0.5174	0.492	0.0002	0.0055497	Both
N	nirk_Cluster16	0.4727	-0.239	0.4241	0.3938	0.0014	0.0163454	Both
N	nirk_Cluster17	0.0998	-0.0119	0.0997	0.0523	0.1633	0.2610501	Both
N	nirk_Cluster18	0.1296	-0.0474	0.257	0.2179	0.019	0.0772624	Both
N	nirk_Cluster19	0.2776	-0.0399	0.2265	0.1858	0.0292	0.09731	Both
N	nirk_Cluster2	0.2032	0.0003	0.00E+00	-0.0526	0.9844	0.986023	Both
N	nirk_Cluster20	0.1738	-0.0308	0.1145	0.0679	0.1335	0.2316291	Both
N	nirk_Cluster21	0.1532	-0.0363	0.4582	0.4297	0.0008	0.0119888	Both
N	nirk_Cluster22	0.1291	-0.0403	0.4276	0.3975	0.0013	0.0156838	Both
N	nirk_Cluster23	0.0673	-0.0134	0.3851	0.3527	0.0027	0.0259743	Both
N	nirk_Cluster24	0.0654	-0.0238	0.4291	0.3991	0.0013	0.01559	Both
N	nirk_Cluster25	0.4782	-0.1761	0.6014	0.5804	0.00E+00	0.0015282	Both
N	nirk_Cluster26	0.105	-0.0161	0.1807	0.1375	0.0548	0.1423311	Both
N	nirk_Cluster27	0.1727	-0.0693	0.5297	0.505	0.0002	0.0045302	Both
N	nirk_Cluster28	0.0685	-0.0228	0.3708	0.3377	0.0034	0.0294593	Both
N	nirk_Cluster29	0.1526	-0.0142	0.0585	0.009	0.2906	0.400579	Both
N	nirk_Cluster3	0.3023	-0.0726	0.3658	0.3325	0.0037	0.0301223	Both
N	nirk_Cluster30	0.0556	-0.0308	0.5063	0.4803	0.0003	0.0060673	Both
N	nirk_Cluster31	0.1501	0.0201	0.2229	0.182	0.0307	0.099485	Both
N	nirk_Cluster32	0.1254	-0.0774	0.5064	0.4804	0.0003	0.0060673	Both
N	nirk_Cluster33	0.0638	-0.0385	0.4346	0.4049	0.0012	0.0150685	Both
N	nirk_Cluster34	0.0416	-0.0117	0.1773	0.134	0.0573	0.1454837	Both
N	nirk_Cluster35	0.1007	-0.0557	0.3607	0.3271	0.004	0.032238	Both
N	nirk_Cluster36	0.0017	-0.0012	0.1308	0.0851	0.1072	0.2066638	Both
N	nirk_Cluster39	0.0035	-0.001	0.0127	-0.0392	0.6264	0.7120944	Both
N	nirk_Cluster4	0.1323	-0.0429	0.421	0.3905	0.0015	0.0166043	Both
N	nirk_Cluster40	0.0455	-0.0215	0.6485	0.63	0.00E+00	0.0005698	Both
N	nirk_Cluster41	0.1562	-0.0537	0.6624	0.6446	0.00E+00	0.0004305	Both
N	nirk_Cluster44	0.0669	-0.0311	0.2971	0.2601	0.0106	0.0545445	Both
N	nirk_Cluster46	0.0154	-0.0092	0.2514	0.212	0.0206	0.0777753	Both
N	nirk_Cluster48	0.003	-0.001	0.0538	0.004	0.3117	0.4203294	Both
N	nirk_Cluster49	0.0057	-0.0026	0.086	0.0379	0.197	0.3018762	Both
N	nirk_Cluster5	0.1932	-0.0414	0.3553	0.3213	0.0044	0.0337768	Both
N	nirk_Cluster50	0.0016	0.0001	0.002	-0.0505	0.8456	0.8783463	Both
N	nirk_Cluster52	0.0452	-0.0216	0.5092	0.4833	0.0003	0.0060673	Both
N	nirk_Cluster53	0.0038	-0.0016	0.0634	0.0141	0.2708	0.3812922	Both
N	nirk_Cluster54	0.002	-0.0008	0.0534	0.0036	0.3135	0.4203294	Both
N	nirk_Cluster55	0.0023	-0.0009	0.0337	-0.0171	0.4255	0.5197599	Both
N	nirk_Cluster56	0.0009	0.00E+00	0.00E+00	-0.0526	0.9816	0.9848943	Both
N	nirk_Cluster57	0.0016	-0.0004	0.0136	-0.0383	0.6144	0.7052428	Both
N	nirk_Cluster58	0.0088	-0.0023	0.0528	0.0029	0.3165	0.4234557	Both
N	nirk_Cluster59	0.0058	-0.0022	0.0989	0.0514	0.1651	0.2625273	Both
N	nirk_Cluster6	0.1558	-0.0357	0.2636	0.2249	0.0173	0.0731475	Both
N	nirk_Cluster60	0.0006	0.0001	0.0042	-0.0482	0.781	0.8302087	Both
N	nirk_Cluster62	0.1417	-0.0684	0.327	0.2916	0.0068	0.0421282	Both
N	nirk_Cluster63	0.1284	-0.0695	0.6344	0.6152	0.00E+00	0.0007664	Both
N	nirk_Cluster64	0.0044	-0.0004	0.0066	-0.0456	0.7254	0.7824686	Both
N	nirk_Cluster65	0.0464	-0.0178	0.3831	0.3506	0.0028	0.0264098	Both
N	nirk_Cluster66	0.0068	-0.005	0.3901	0.358	0.0025	0.0247217	Both
N	nirk_Cluster67	0.0174	-0.0075	0.1378	0.0924	0.0976	0.1938696	Both
N	nirk_Cluster68	0.0125	-0.0074	0.3308	0.2955	0.0064	0.0421282	Both
N	nirk_Cluster69	0.0305	-0.011	0.2664	0.2278	0.0166	0.0724573	Both
N	nirk_Cluster7	0.0804	-0.031	0.4526	0.4238	0.0008	0.0126036	Both
N	nirk_Cluster70	0.0721	-0.0332	0.1598	0.1156	0.0726	0.1634719	Both
N	nirk_Cluster71	0.0053	-0.0033	0.2527	0.2134	0.0202	0.0777753	Both

N	nirk_Cluster72	0.0239	-0.0101	0.348	0.3137	0.0049	0.0363025	Both
N	nirk_Cluster73	0.1444	-0.0964	0.7423	0.7288	0.00E+00	4.42E-05	Both
N	nirk_Cluster74	0.6398	-0.3574	0.4762	0.4486	0.0005	0.0093082	Both
N	nirk_Cluster75	0.0147	-0.01	0.249	0.2095	0.0213	0.0780373	Both
N	nirk_Cluster76	0.1055	-0.0604	0.7595	0.7469	0.00E+00	2.65E-05	Both
N	nirk_Cluster77	0.0081	-0.0036	0.0432	-0.0071	0.3657	0.4710953	Both
N	nirk_Cluster78	0.2407	-0.1509	0.7701	0.758	0.00E+00	2.06E-05	Both
N	nirk_Cluster79	0.0069	-0.0034	0.079	0.0305	0.2171	0.3276523	Both
N	nirk_Cluster8	0.0982	-0.0424	0.3973	0.3656	0.0022	0.0230936	Both
N	nirk_Cluster80	0.2665	-0.1753	0.6941	0.678	0.00E+00	0.0002036	Both
N	nirk_Cluster82	0.0015	-0.0008	0.0392	-0.0113	0.3895	0.4879149	Both
N	nirk_Cluster83	0.004	-0.0011	0.0445	-0.0058	0.3588	0.4631693	Both
N	nirk_Cluster84	0.0207	-0.0053	0.0503	0.0003	0.3286	0.4375814	Both
N	nirk_Cluster85	0.004	-0.0019	0.1267	0.0808	0.1132	0.2125138	Both
N	nirk_Cluster86	0.0286	-0.0157	0.3295	0.2942	0.0065	0.0421282	Both
N	nirk_Cluster87	0.0115	-0.0066	0.2967	0.2597	0.0107	0.0545445	Both
N	nirk_Cluster88	0.0032	-0.0013	0.1002	0.0528	0.1622	0.2608257	Both
N	nirk_Cluster89	0.0065	-0.0045	0.2504	0.2109	0.0209	0.0777753	Both
N	nirk_Cluster9	0.0517	-0.0182	0.217	0.1758	0.0333	0.1050713	Both
N	nirk_Cluster90	0.0067	-0.004	0.162	0.1179	0.0705	0.1612114	Both
N	nirk_Cluster91	0.0374	-0.0188	0.4452	0.416	0.001	0.0140601	Both
N	nirk_Cluster92	0.0072	-0.0034	0.1213	0.0751	0.1218	0.2216939	Both
N	nirk_Cluster93	0.0308	-0.0076	0.1071	0.0601	0.1476	0.2508809	Both
N	nirk_Cluster94	0.0175	-0.0092	0.3005	0.2637	0.0101	0.0540754	Both
N	nirk_Cluster95	0.0473	-0.0237	0.327	0.2916	0.0068	0.0421282	Both
N	nirk_Cluster96	0.0472	-0.0259	0.4885	0.4616	0.0004	0.0080543	Both
N	nirk_Cluster97	0.0306	-0.0094	0.1576	0.1132	0.0748	0.1646995	Both
N	nirk_Cluster98	0.0181	-0.0101	0.2221	0.1812	0.031	0.099485	Both
N	nirk_Cluster99	0.006	-0.0034	0.178	0.1347	0.0568	0.1454837	Both
N	nirs_Cluster0	0.1409	-0.0389	0.3254	0.2899	0.0069	0.0421282	Both
N	nirs_Cluster1	0.004	-0.0017	0.0552	0.0055	0.3052	0.4149056	Both
N	nirs_Cluster14	0.0107	-0.0081	0.1261	0.0801	0.1142	0.2125138	Both
N	nirs_Cluster2	0.0139	-0.0057	0.0627	0.0134	0.2734	0.3841154	Both
N	nirs_Cluster26	0.0005	-0.0004	0.0723	0.0235	0.2385	0.3457989	Both
N	nirs_Cluster28	0.0071	-0.0064	0.1696	0.1259	0.0636	0.1517364	Both
N	nirs_Cluster3	0.1715	-0.0364	0.3904	0.3584	0.0025	0.0247217	Both
N	nirs_Cluster36	0.0013	-0.0006	0.0148	-0.0371	0.5994	0.6934179	Both
N	nirs_Cluster37	0.0066	-0.0059	0.0914	0.0435	0.183	0.2870982	Both
N	nirs_Cluster4	0.0002	-0.0002	0.0375	-0.0131	0.4	0.4968344	Both
N	nirs_Cluster50	0.002	0.00E+00	0.00E+00	-0.0526	0.98	0.9848943	Both
N	nirs_Cluster9	0.0269	-0.0249	0.176	0.1326	0.0583	0.1454837	Both
N	norb_Cluster0	0.0775	-0.0295	0.2377	0.1976	0.025	0.0856057	Both
N	norb_Cluster1	1.1042	-0.5189	0.2646	0.2259	0.017	0.0728629	Both
N	norb_Cluster101	0.0026	-0.0022	0.1655	0.1216	0.0672	0.155633	Both
N	norb_Cluster102	0.0038	-0.0026	0.2159	0.1746	0.0338	0.1056696	Both
N	norb_Cluster103	0.0018	-0.0015	0.1609	0.1167	0.0715	0.1629008	Both
N	norb_Cluster104	0.0023	-0.0009	0.0376	-0.013	0.3994	0.4968344	Both
N	norb_Cluster106	0.125	-0.0532	0.3101	0.2738	0.0087	0.0491232	Both
N	norb_Cluster107	0.0133	-0.0075	0.2511	0.2117	0.0207	0.0777753	Both
N	norb_Cluster108	0.0052	-0.0036	0.341	0.3063	0.0054	0.0387038	Both
N	norb_Cluster109	0.0227	-0.0091	0.1743	0.1309	0.0597	0.1460563	Both
N	norb_Cluster11	0.0126	-0.0074	0.2566	0.2174	0.0191	0.0772624	Both
N	norb_Cluster110	0.0099	-0.0059	0.2411	0.2012	0.0238	0.083968	Both
N	norb_Cluster112	0.0171	-0.0084	0.2992	0.2623	0.0103	0.0545445	Both
N	norb_Cluster113	0.0134	-0.0051	0.1031	0.0559	0.1558	0.2574264	Both

N	norb_Cluster114	0.0025	-0.0021	0.1132	0.0665	0.1359	0.2332723	Both
N	norb_Cluster115	0.0137	0.0173	0.1238	0.0777	0.1178	0.2172386	Both
N	norb_Cluster116	0.0072	-0.0021	0.0416	-0.0088	0.3749	0.4757224	Both
N	norb_Cluster117	0.0027	-0.0015	0.1013	0.054	0.1596	0.2608257	Both
N	norb_Cluster118	0.0037	-0.0019	0.1368	0.0914	0.0989	0.1944604	Both
N	norb_Cluster119	0.019	-0.0141	0.3294	0.2941	0.0065	0.0421282	Both
N	norb_Cluster12	0.0047	-0.0018	0.0582	0.0087	0.2919	0.400579	Both
N	norb_Cluster121	0.0017	-0.0012	0.0826	0.0343	0.2065	0.314081	Both
N	norb_Cluster122	0.0144	-0.0074	0.2424	0.2025	0.0234	0.0830744	Both
N	norb_Cluster123	0.0053	-0.0033	0.1951	0.1527	0.045	0.1237339	Both
N	norb_Cluster124	0.682	-0.2163	0.1527	0.1081	0.0799	0.1720885	Both
N	norb_Cluster125	0.004	-0.0027	0.2458	0.2061	0.0223	0.0801773	Both
N	norb_Cluster126	0.0082	-0.0056	0.3125	0.2763	0.0084	0.0478316	Both
N	norb_Cluster127	0.1735	-0.0869	0.3262	0.2907	0.0068	0.0421282	Both
N	norb_Cluster128	0.0115	-0.0045	0.1714	0.1278	0.062	0.1488134	Both
N	norb_Cluster13	0.0045	-0.0031	0.153	0.1085	0.0795	0.1720885	Both
N	norb_Cluster131	0.0184	-0.0044	0.0597	0.0102	0.2858	0.3957974	Both
N	norb_Cluster132	1.0357	-0.5285	0.5489	0.5251	0.0001	0.0035034	Both
N	norb_Cluster133	0.0018	-0.001	0.0965	0.0489	0.1705	0.2697022	Both
N	norb_Cluster135	0.0567	-0.029	0.3439	0.3093	0.0052	0.0374632	Both
N	norb_Cluster136	0.0213	-0.009	0.2124	0.1709	0.0355	0.1080172	Both
N	norb_Cluster139	0.0328	-0.0156	0.3147	0.2786	0.0082	0.0467003	Both
N	norb_Cluster14	0.0026	-0.0021	0.2408	0.2008	0.0239	0.083968	Both
N	norb_Cluster140	0.0437	-0.0231	0.3659	0.3325	0.0037	0.0301223	Both
N	norb_Cluster142	0.0015	-0.0012	0.1173	0.0709	0.1285	0.2265521	Both
N	norb_Cluster144	0.0098	-0.0036	0.1131	0.0665	0.136	0.2332723	Both
N	norb_Cluster146	0.0348	-0.0156	0.3074	0.2709	0.0091	0.0492601	Both
N	norb_Cluster15	0.0469	-0.0217	0.2489	0.2093	0.0213	0.0780373	Both
N	norb_Cluster150	0.0157	-0.0089	0.4388	0.4093	0.0011	0.0150112	Both
N	norb_Cluster155	0.1631	-0.0493	0.1528	0.1082	0.0797	0.1720885	Both
N	norb_Cluster16	0.0131	-0.0086	0.2584	0.2194	0.0186	0.0771314	Both
N	norb_Cluster164	0.0199	-0.0144	0.3986	0.367	0.0021	0.023002	Both
N	norb_Cluster165	0.0076	-0.0044	0.1492	0.1044	0.0838	0.1739911	Both
N	norb_Cluster166	0.0039	-0.0017	0.0779	0.0294	0.2205	0.3306443	Both
N	norb_Cluster169	0.0238	-0.0176	0.3821	0.3496	0.0028	0.0264106	Both
N	norb_Cluster17	0.0028	-0.002	0.2235	0.1827	0.0304	0.099485	Both
N	norb_Cluster170	0.0243	-0.013	0.1749	0.1315	0.0592	0.1454837	Both
N	norb_Cluster171	0.0069	-0.0046	0.154	0.1094	0.0785	0.1720885	Both
N	norb_Cluster172	0.014	-0.0102	0.2907	0.2533	0.0117	0.0578592	Both
N	norb_Cluster175	0.0458	-0.0325	0.3084	0.272	0.009	0.0492272	Both
N	norb_Cluster176	0.0298	-0.022	0.3357	0.3008	0.0059	0.0410521	Both
N	norb_Cluster177	0.0282	-0.018	0.2527	0.2134	0.0202	0.0777753	Both
N	norb_Cluster179	0.0022	-0.0012	0.077	0.0284	0.2232	0.3330875	Both
N	norb_Cluster18	0.0037	-0.0025	0.262	0.2231	0.0177	0.0740508	Both
N	norb_Cluster180	0.0109	-0.0046	0.2022	0.1603	0.0408	0.1157596	Both
N	norb_Cluster181	0.0008	0.0007	0.0765	0.0279	0.2248	0.3341207	Both
N	norb_Cluster182	0.0013	-0.0009	0.115	0.0684	0.1326	0.2307476	Both
N	norb_Cluster184	0.0146	-0.0079	0.2378	0.1976	0.0249	0.0856057	Both
N	norb_Cluster186	0.0011	-0.0001	0.0025	-0.05	0.8286	0.8652879	Both
N	norb_Cluster19	0.0049	-0.0022	0.1577	0.1134	0.0746	0.1646995	Both
N	norb_Cluster192	0.0173	-0.0125	0.3475	0.3131	0.0049	0.0363025	Both
N	norb_Cluster193	0.0021	-0.0004	0.0134	-0.0386	0.6177	0.706247	Both
N	norb_Cluster197	0.0303	-0.0207	0.0701	0.0212	0.2459	0.3530604	Both
N	norb_Cluster2	0.0031	-0.0005	0.0079	-0.0444	0.7023	0.7630848	Both
N	norb_Cluster20	0.0587	-0.025	0.2738	0.2356	0.0149	0.0687657	Both

N	norb_Cluster208	0.0011	-0.001	0.1256	0.0795	0.115	0.2134325	Both
N	norb_Cluster21	0.0481	-0.0255	0.3169	0.281	0.0079	0.0456037	Both
N	norb_Cluster211	0.0104	-0.0075	0.0992	0.0518	0.1644	0.2621788	Both
N	norb_Cluster212	0.0264	-0.0014	0.0009	-0.0517	0.8983	0.9201115	Both
N	norb_Cluster214	0.0119	-0.0085	0.158	0.1137	0.0744	0.1646995	Both
N	norb_Cluster217	0.0149	-0.0082	0.1042	0.057	0.1536	0.2545776	Both
N	norb_Cluster219	0.0083	-0.006	0.1263	0.0804	0.1138	0.2125138	Both
N	norb_Cluster22	0.0025	-0.0013	0.0684	0.0194	0.252	0.3600549	Both
N	norb_Cluster220	0.0177	-0.015	0.1507	0.106	0.082	0.1728806	Both
N	norb_Cluster221	0.0177	-0.0144	0.1215	0.0753	0.1214	0.2216939	Both
N	norb_Cluster222	0.0074	-0.0062	0.0959	0.0483	0.172	0.2713785	Both
N	norb_Cluster224	0.0194	-0.011	0.1024	0.0551	0.1573	0.2593066	Both
N	norb_Cluster225	0.0097	-1.00E-04	0.00E+00	-0.0526	0.9886	0.9885886	Both
N	norb_Cluster226	0.0043	-0.0015	0.0418	-0.0086	0.3739	0.4757224	Both
N	norb_Cluster228	0.0031	0.0036	0.1007	0.0533	0.1611	0.2608257	Both
N	norb_Cluster229	0.0034	-0.0011	0.0395	-0.011	0.3876	0.4877571	Both
N	norb_Cluster230	0.0014	-0.0011	0.1227	0.0765	0.1196	0.21979	Both
N	norb_Cluster231	0.0044	-0.003	0.1135	0.0668	0.1354	0.2332723	Both
N	norb_Cluster232	0.0031	-0.002	0.1174	0.071	0.1283	0.2265521	Both
N	norb_Cluster234	0.0121	-0.0099	0.2689	0.2304	0.016	0.0720287	Both
N	norb_Cluster235	0.0009	-0.0007	0.1385	0.0932	0.0967	0.1926648	Both
N	norb_Cluster238	0.0099	-0.0068	0.147	0.1021	0.0862	0.1769758	Both
N	norb_Cluster239	0.0081	-0.0057	0.1286	0.0827	0.1104	0.2101965	Both
N	norb_Cluster24	0.2637	-0.1224	0.3258	0.2903	0.0069	0.0421282	Both
N	norb_Cluster244	0.0011	-0.0007	0.0871	0.0391	0.1939	0.3004101	Both
N	norb_Cluster247	0.0048	-0.0023	0.1728	0.1292	0.0609	0.1473639	Both
N	norb_Cluster25	0.1114	-0.0506	0.2544	0.2151	0.0197	0.0777753	Both
N	norb_Cluster250	0.0115	-0.0057	0.176	0.1326	0.0584	0.1454837	Both
N	norb_Cluster251	0.0052	-0.0026	0.1281	0.0822	0.1112	0.2102707	Both
N	norb_Cluster26	0.0931	-0.0549	0.3583	0.3245	0.0041	0.0330855	Both
N	norb_Cluster28	0.0302	-0.0114	0.1496	0.1049	0.0832	0.1739911	Both
N	norb_Cluster29	0.222	-0.0692	0.3699	0.3368	0.0034	0.0294593	Both
N	norb_Cluster31	0.0531	-0.0262	0.287	0.2495	0.0123	0.0600267	Both
N	norb_Cluster32	0.1193	-0.0626	0.2503	0.2108	0.0209	0.0777753	Both
N	norb_Cluster33	0.4946	0.1153	0.2037	0.1618	0.04	0.1151761	Both
N	norb_Cluster35	0.0628	-0.0327	0.3344	0.2994	0.006	0.0410521	Both
N	norb_Cluster36	1.1345	-0.0798	0.0096	-0.0425	0.6729	0.7395426	Both
N	norb_Cluster37	0.7473	-0.0151	0.0012	-0.0513	0.8803	0.9096015	Both
N	norb_Cluster38	0.0116	-0.0054	0.2864	0.2488	0.0124	0.0601057	Both
N	norb_Cluster39	0.0426	-0.0202	0.266	0.2273	0.0167	0.0724573	Both
N	norb_Cluster40	0.186	-0.0926	0.3342	0.2991	0.0061	0.0410521	Both
N	norb_Cluster42	0.2864	-0.0194	0.0096	-0.0425	0.6723	0.7395426	Both
N	norb_Cluster44	0.0182	-0.0135	0.3454	0.3109	0.0051	0.0370492	Both
N	norb_Cluster46	0.2556	-0.0157	0.0054	-0.0469	0.7513	0.8021847	Both
N	norb_Cluster47	0.555	-0.246	0.3183	0.2824	0.0077	0.0451123	Both
N	norb_Cluster5	0.01	-0.0032	0.1335	0.0879	0.1034	0.2013621	Both
N	norb_Cluster51	0.0092	-0.0068	0.2098	0.1682	0.0368	0.1085624	Both
N	norb_Cluster52	0.2391	-0.1169	0.3081	0.2716	0.009	0.0492272	Both
N	norb_Cluster56	0.014	-0.0083	0.3536	0.3196	0.0045	0.0342356	Both
N	norb_Cluster57	0.0034	-0.0011	0.0656	0.0164	0.2624	0.3730233	Both
N	norb_Cluster59	0.0236	-0.014	0.1728	0.1293	0.0609	0.1473639	Both
N	norb_Cluster6	0.6862	-0.0843	0.0309	-0.0201	0.4456	0.5398183	Both
N	norb_Cluster60	0.3199	-0.095	0.07	0.021	0.2466	0.3531183	Both
N	norb_Cluster61	0.482	-0.1736	0.1738	0.1303	0.0601	0.1465565	Both
N	norb_Cluster62	0.0024	0.0002	0.0024	-0.0501	0.834	0.8693309	Both

N	norb_Cluster64	0.0326	-0.016	0.3676	0.3343	0.0036	0.0301019	Both
N	norb_Cluster66	0.0012	-0.0009	0.1217	0.0754	0.1212	0.2216939	Both
N	norb_Cluster67	0.0218	-0.0126	0.3331	0.298	0.0062	0.0412472	Both
N	norb_Cluster68	0.0034	-0.0007	0.0305	-0.0206	0.4492	0.5430442	Both
N	norb_Cluster69	0.0028	-0.0018	0.1587	0.1145	0.0736	0.1639541	Both
N	norb_Cluster7	0.0709	-0.0274	0.2141	0.1728	0.0347	0.1064779	Both
N	norb_Cluster70	0.1051	-0.0475	0.3192	0.2834	0.0076	0.0449321	Both
N	norb_Cluster71	0.1295	-0.05	0.2829	0.2452	0.0131	0.0627084	Both
N	norb_Cluster74	0.0327	-0.0149	0.3223	0.2866	0.0073	0.0437508	Both
N	norb_Cluster75	0.1291	-0.0575	0.4704	0.4426	0.0006	0.009805	Both
N	norb_Cluster76	0.1105	-0.0379	0.0702	0.0213	0.2456	0.3530604	Both
N	norb_Cluster77	0.0475	-0.0094	0.1001	0.0528	0.1622	0.2608257	Both
N	norb_Cluster8	0.018	-0.0076	0.1724	0.1289	0.0612	0.1474521	Both
N	norb_Cluster80	0.0015	-0.0013	0.1652	0.1213	0.0675	0.155633	Both
N	norb_Cluster81	0.0012	-0.0003	0.0123	-0.0397	0.6319	0.7128674	Both
N	norb_Cluster83	0.0078	-0.0068	0.1764	0.133	0.058	0.1454837	Both
N	norb_Cluster84	0.0082	-0.0032	0.1159	0.0694	0.131	0.2288737	Both
N	norb_Cluster85	0.0023	-0.001	0.0724	0.0236	0.2381	0.3457989	Both
N	norb_Cluster86	0.031	-0.0227	0.1516	0.1069	0.0811	0.1720885	Both
N	norb_Cluster88	0.0016	-0.0007	0.0512	0.0012	0.3242	0.4327805	Both
N	norb_Cluster90	0.0009	-0.0007	0.0497	-0.0003	0.3312	0.4381189	Both
N	norb_Cluster91	0.6481	-0.3086	0.2701	0.2317	0.0157	0.0714693	Both
N	norb_Cluster92	0.0015	-0.001	0.0569	0.0072	0.2979	0.4061302	Both
N	norb_Cluster93	0.0022	-0.0011	0.0461	-0.0041	0.3502	0.454047	Both
N	norb_Cluster94	0.0017	-0.0003	0.0118	-0.0402	0.6386	0.7190743	Both
N	norb_Cluster96	0.0007	0.0002	0.0096	-0.0426	0.6731	0.7395426	Both
N	norb_Cluster97	0.0119	-0.0065	0.1686	0.1248	0.0645	0.1524487	Both
N	norb_Cluster98	0.0132	-0.0095	0.2352	0.1949	0.0259	0.0877402	Both
N	nosz_Cluster0	0.0045	-0.0018	0.1052	0.0581	0.1514	0.2529945	Both
N	nosz_Cluster10	0.0136	0.0014	0.007	-0.0453	0.719	0.7783234	Both
N	nosz_Cluster102	0.0044	-0.002	0.1982	0.156	0.0431	0.1214472	Both
N	nosz_Cluster103	0.0455	-0.011	0.1053	0.0582	0.1512	0.2529945	Both
N	nosz_Cluster105	0.0016	-0.0005	0.0303	-0.0207	0.4501	0.5431231	Both
N	nosz_Cluster106	0.0073	-0.0037	0.1694	0.1257	0.0638	0.1517364	Both
N	nosz_Cluster107	0.0002	-0.0002	0.0375	-0.0131	0.4	0.4968344	Both
N	nosz_Cluster108	0.004	-0.0006	0.0104	-0.0417	0.66	0.7347269	Both
N	nosz_Cluster109	0.0375	-0.0102	0.198	0.1558	0.0432	0.1214472	Both
N	nosz_Cluster11	0.0129	0.0047	0.0357	-0.015	0.4119	0.506975	Both
N	nosz_Cluster110	0.0003	0.0004	0.1292	0.0834	0.1095	0.2096815	Both
N	nosz_Cluster111	0.0061	-0.0032	0.1929	0.1504	0.0464	0.1255179	Both
N	nosz_Cluster116	0.0067	-0.0009	0.0125	-0.0394	0.629	0.7122085	Both
N	nosz_Cluster118	0.0045	-0.0036	0.2144	0.1731	0.0345	0.1064779	Both
N	nosz_Cluster119	0.0013	-0.0012	0.1798	0.1366	0.0554	0.1434644	Both
N	nosz_Cluster120	0.0071	-0.0026	0.1061	0.0591	0.1496	0.2528318	Both
N	nosz_Cluster123	0.0097	-0.008	0.3561	0.3223	0.0043	0.0337583	Both
N	nosz_Cluster128	0.0047	-0.0012	0.0255	-0.0258	0.4892	0.5831264	Both
N	nosz_Cluster129	0.0494	-0.004	0.0289	-0.0222	0.461	0.5551331	Both
N	nosz_Cluster13	0.0115	-0.002	0.0226	-0.0288	0.5152	0.6091378	Both
N	nosz_Cluster130	0.089	-0.0571	0.0869	0.0389	0.1945	0.3004101	Both
N	nosz_Cluster132	0.0037	-0.0011	0.0749	0.0262	0.23	0.3375817	Both
N	nosz_Cluster14	0.024	0.0148	0.0619	0.0125	0.2769	0.3864745	Both
N	nosz_Cluster16	0.0076	-0.0004	0.0011	-0.0515	0.8876	0.9139271	Both
N	nosz_Cluster17	0.0364	-0.0152	0.2388	0.1987	0.0246	0.0853289	Both
N	nosz_Cluster18	0.0161	-0.0046	0.0417	-0.0088	0.3748	0.4757224	Both
N	nosz_Cluster20	0.0091	-0.0063	0.2059	0.1641	0.0388	0.1128266	Both

N	nosz_Cluster21	0.0083	-0.007	0.2804	0.2426	0.0136	0.0644967	Both
N	nosz_Cluster22	0.0267	-0.0138	0.1118	0.065	0.1386	0.236285	Both
N	nosz_Cluster23	0.0141	-0.011	0.3199	0.2841	0.0075	0.0448836	Both
N	nosz_Cluster24	0.0013	-0.0009	0.0831	0.0349	0.205	0.3124672	Both
N	nosz_Cluster25	0.0029	0.0006	0.009	-0.0431	0.6823	0.7468516	Both
N	nosz_Cluster26	0.0054	-0.0033	0.1494	0.1046	0.0835	0.1739911	Both
N	nosz_Cluster27	0.0053	-0.0032	0.2046	0.1627	0.0395	0.1143304	Both
N	nosz_Cluster28	0.0303	-0.0155	0.2535	0.2142	0.02	0.0777753	Both
N	nosz_Cluster30	0.0276	-0.0126	0.1967	0.1544	0.044	0.1225878	Both
N	nosz_Cluster32	0.0137	-0.0037	0.0584	0.0088	0.2913	0.400579	Both
N	nosz_Cluster33	0.018	-0.0029	0.0116	-0.0404	0.6422	0.7217551	Both
N	nosz_Cluster34	0.0377	-0.0071	0.0182	-0.0335	0.5601	0.6570063	Both
N	nosz_Cluster35	0.0075	-0.0044	0.2183	0.1772	0.0327	0.1037727	Both
N	nosz_Cluster36	0.0179	-0.0072	0.1184	0.072	0.1267	0.2265521	Both
N	nosz_Cluster37	0.006	-0.0031	0.1396	0.0943	0.0953	0.1912092	Both
N	nosz_Cluster38	0.0128	-0.0041	0.0541	0.0043	0.3102	0.4198329	Both
N	nosz_Cluster39	0.0537	-0.0252	0.2284	0.1878	0.0284	0.0952852	Both
N	nosz_Cluster40	0.0218	-0.0082	0.1006	0.0533	0.1613	0.2608257	Both
N	nosz_Cluster41	0.0203	-0.0114	0.2131	0.1717	0.0352	0.1074634	Both
N	nosz_Cluster42	0.0279	-0.0072	0.0477	-0.0024	0.3416	0.4468897	Both
N	nosz_Cluster43	0.0007	-0.0006	0.0739	0.0252	0.2331	0.3413004	Both
N	nosz_Cluster44	0.0057	-0.0025	0.1048	0.0577	0.1523	0.2538855	Both
N	nosz_Cluster45	0.0045	-0.002	0.1058	0.0587	0.1503	0.2529945	Both
N	nosz_Cluster46	0.007	-0.0023	0.1045	0.0574	0.1528	0.2539388	Both
N	nosz_Cluster47	0.0008	-0.0007	0.1265	0.0805	0.1136	0.2125138	Both
N	nosz_Cluster48	0.0023	-0.0013	0.0889	0.041	0.1892	0.2953557	Both
N	nosz_Cluster49	0.0014	0.0003	0.0137	-0.0382	0.6131	0.7050751	Both
N	nosz_Cluster5	0.0045	0.0004	0.002	-0.0505	0.8473	0.8785786	Both
N	nosz_Cluster51	0.0021	-0.0015	0.1601	0.1159	0.0723	0.163428	Both
N	nosz_Cluster53	0.0009	-0.0005	0.0394	-0.0111	0.3883	0.4877571	Both
N	nosz_Cluster54	0.0026	-0.0018	0.1176	0.0712	0.128	0.2265521	Both
N	nosz_Cluster57	0.1757	-0.0977	0.2964	0.2593	0.0107	0.0545445	Both
N	nosz_Cluster58	0.0043	-0.0031	0.1855	0.1426	0.0513	0.1369237	Both
N	nosz_Cluster59	0.0009	-1.00E-04	0.0005	-0.0521	0.9206	0.9380777	Both
N	nosz_Cluster6	0.0032	-0.0007	0.0169	-0.0348	0.5742	0.6722058	Both
N	nosz_Cluster60	0.0079	-0.0038	0.127	0.0811	0.1128	0.2125138	Both
N	nosz_Cluster61	0.0178	-0.0038	0.1005	0.0531	0.1615	0.2608257	Both
N	nosz_Cluster62	0.0413	-0.019	0.2029	0.1609	0.0405	0.1157596	Both
N	nosz_Cluster64	0.0016	-0.0006	0.0145	-0.0373	0.6027	0.6958713	Both
N	nosz_Cluster65	0.0065	-0.0031	0.1174	0.071	0.1283	0.2265521	Both
N	nosz_Cluster66	0.0324	-0.0204	0.2481	0.2085	0.0216	0.0780373	Both
N	nosz_Cluster67	0.0521	-0.0297	0.2242	0.1834	0.0302	0.0993929	Both
N	nosz_Cluster69	0.0061	-0.0042	0.2794	0.2414	0.0138	0.0649761	Both
N	nosz_Cluster70	0.0479	-0.0375	0.1622	0.1181	0.0703	0.1612114	Both
N	nosz_Cluster71	0.0083	-0.0041	0.1056	0.0586	0.1505	0.2529945	Both
N	nosz_Cluster72	0.0506	-0.0307	0.2918	0.2545	0.0115	0.0573772	Both
N	nosz_Cluster73	0.003	-0.0003	0.0032	-0.0492	0.8066	0.8511321	Both
N	nosz_Cluster74	0.0017	-0.0007	0.05	0.00E+00	0.3299	0.4376093	Both
N	nosz_Cluster75	0.0967	-0.0789	0.1002	0.0529	0.1621	0.2608257	Both
N	nosz_Cluster77	0.0002	0.00E+00	0.0031	-0.0493	0.8099	0.8517243	Both
N	nosz_Cluster78	0.0044	-0.0021	0.14	0.0947	0.0948	0.1908336	Both
N	nosz_Cluster79	0.0333	-0.0132	0.1967	0.1544	0.044	0.1225878	Both
N	nosz_Cluster8	0.0099	-0.0026	0.071	0.0222	0.2428	0.3503069	Both
N	nosz_Cluster80	0.0045	0.001	0.0156	-0.0362	0.5897	0.6853642	Both
N	nosz_Cluster81	0.0079	-0.0049	0.1179	0.0715	0.1275	0.2265521	Both

N	nosz_Cluster82	0.0016	-0.0006	0.0102	-0.0419	0.6627	0.7362904	Both
N	nosz_Cluster83	0.0015	-0.0003	0.0067	-0.0456	0.7245	0.7824686	Both
N	nosz_Cluster85	0.0022	-0.0006	0.0167	-0.035	0.5762	0.6731999	Both
N	nosz_Cluster86	0.0027	-0.0017	0.1211	0.0749	0.1221	0.2216939	Both
N	nosz_Cluster87	0.003	-0.0007	0.0257	-0.0255	0.4873	0.581973	Both
N	nosz_Cluster88	0.0218	-0.0065	0.1955	0.1531	0.0448	0.1237339	Both
N	nosz_Cluster89	0.0466	-0.0284	0.2892	0.2518	0.0119	0.0586176	Both
N	nosz_Cluster9	0.003	-0.001	0.0352	-0.0156	0.4156	0.508703	Both
N	nosz_Cluster90	0.0116	-0.0047	0.2369	0.1968	0.0252	0.0860741	Both
N	nosz_Cluster91	0.0189	-0.0067	0.0756	0.027	0.2277	0.3358138	Both
N	nosz_Cluster93	0.0393	-0.0201	0.2685	0.23	0.0161	0.0720287	Both
N	nosz_Cluster95	0.007	-0.0038	0.2105	0.169	0.0364	0.1085624	Both
N	nosz_Cluster96	0.0066	0.0005	0.0066	-0.0457	0.7272	0.782955	Both
N	nosz_Cluster97	0.0019	-0.0013	0.1309	0.0852	0.107	0.2066638	Both
N	nosz_Cluster98	0.0025	-0.001	0.0143	-0.0376	0.6059	0.6982589	Both
N	nosz_Cluster99	0.0055	-0.0007	0.0156	-0.0363	0.5901	0.6853642	Both
N	nrfa_Cluster10	0.0804	-0.0387	0.1484	0.1036	0.0846	0.1750531	Both
N	nrfa_Cluster100	0.0963	-0.0647	0.2092	0.1676	0.0371	0.1089036	Both
N	nrfa_Cluster104	0.1143	-0.0683	0.3722	0.3392	0.0033	0.0294593	Both
N	nrfa_Cluster105	0.0062	-0.0003	0.0005	-0.0521	0.9198	0.9380777	Both
N	nrfa_Cluster109	0.2294	-0.0972	0.2255	0.1847	0.0296	0.0981682	Both
N	nrfa_Cluster116	0.2592	-0.1385	0.548	0.5242	0.0001	0.0035034	Both
N	nrfa_Cluster118	0.173	-0.1266	0.2024	0.1604	0.0407	0.1157596	Both
N	nrfa_Cluster125	0.0809	-0.0587	0.1766	0.1333	0.0579	0.1454837	Both
N	nrfa_Cluster154	0.0658	-0.0394	0.1812	0.1381	0.0544	0.1423311	Both
N	nrfa_Cluster164	0.0045	-0.0001	0.0003	-0.0523	0.9402	0.9498525	Both
N	nrfa_Cluster172	0.0026	-0.0023	0.1749	0.1315	0.0592	0.1454837	Both
N	nrfa_Cluster175	0.0044	-0.0025	0.0779	0.0294	0.2204	0.3306443	Both
N	nrfa_Cluster20	0.035	-0.0205	0.2101	0.1685	0.0367	0.1085624	Both
N	nrfa_Cluster26	0.1078	-0.0801	0.2306	0.1901	0.0276	0.0930401	Both
N	nrfa_Cluster29	0.0048	-0.0022	0.0757	0.0271	0.2274	0.3358138	Both
N	nrfa_Cluster34	0.0487	-0.0316	0.2541	0.2148	0.0198	0.0777753	Both
N	nrfa_Cluster38	0.0031	-0.0013	0.0496	-0.0004	0.3319	0.4381189	Both
N	nrfa_Cluster61	0.0036	-0.0027	0.1808	0.1377	0.0546	0.1423311	Both
N	nrfa_Cluster62	0.0452	-0.0323	0.1774	0.1341	0.0572	0.1454837	Both
N	nrfa_Cluster66	0.1082	-0.048	0.2935	0.2564	0.0112	0.0564014	Both
N	nrfa_Cluster69	0.003	-0.0012	0.0671	0.018	0.257	0.366203	Both
N	nrfa_Cluster71	0.027	-0.0115	0.0819	0.0336	0.2086	0.3163339	Both
N	nrfa_Cluster72	0.0732	-0.0496	0.2509	0.2115	0.0207	0.0777753	Both
N	nrfa_Cluster73	0.0038	-0.0013	0.0323	-0.0186	0.4357	0.5289356	Both
N	nrfa_Cluster74	0.1331	-0.0811	0.2147	0.1733	0.0344	0.1064779	Both
N	nrfa_Cluster76	0.0755	-0.04	0.1389	0.0935	0.0962	0.1923704	Both
N	nrfa_Cluster79	0.0229	0.0138	0.0478	-0.0023	0.3411	0.4468897	Both
N	nrfa_Cluster87	0.017	-0.0094	0.0879	0.0399	0.1918	0.2986005	Both
N	nrfa_Cluster89	0.0018	-0.0011	0.0862	0.0381	0.1965	0.3018762	Both
N	nrfa_Cluster92	0.0043	-0.0001	0.0004	-0.0522	0.9337	0.9464848	Both
N	nrfa_Cluster93	0.0428	-0.0324	0.1665	0.1227	0.0663	0.1545532	Both
N	nrfa_Cluster94	0.0002	-0.0002	0.0431	-0.0073	0.3666	0.4712564	Both
N	nrfa_Cluster97	0.0168	-0.0111	0.3093	0.2729	0.0088	0.0492272	Both
N	nrfa_Cluster99	0.014	-0.0084	0.2158	0.1746	0.0339	0.1056696	Both
CN	amoA_A_Cluster26	0.004	-0.002	0.072	0.0232	0.2394	0.5331685	Both
CN	amoA_A_Cluster45	0.0051	-0.0025	0.084	0.0358	0.2026	0.5201002	Both
CN	amoA_B_Cluster0	0.1099	-0.0335	0.4374	0.4078	0.0011	0.0174628	Both
CN	amoA_B_Cluster1	0.0172	-0.0089	0.2677	0.2292	0.0163	0.1262175	Both
CN	amoA_B_Cluster10	0.0422	-0.0243	0.6262	0.6065	0.00E+00	0.0009138	Both

CN	amoA_B_Cluster11	0.0377	-0.0194	0.3628	0.3292	0.0039	0.0446769	Both
CN	amoA_B_Cluster12	0.0807	-0.0324	0.5818	0.5598	1.00E-04	0.002016	Both
CN	amoA_B_Cluster13	0.5271	-0.3334	0.6773	0.6604	0.00E+00	0.0003031	Both
CN	amoA_B_Cluster14	0.0275	-0.0197	0.4629	0.4347	0.0007	0.0126945	Both
CN	amoA_B_Cluster15	0.0318	-0.0206	0.5517	0.5281	0.0001	0.0037701	Both
CN	amoA_B_Cluster2	0.1721	-0.1129	0.701	0.6853	0.00E+00	0.0001908	Both
CN	amoA_B_Cluster20	0.0058	-0.0025	0.0749	0.0263	0.2298	0.5214893	Both
CN	amoA_B_Cluster23	0.0507	-0.0244	0.4836	0.4565	0.0005	0.00998	Both
CN	amoA_B_Cluster3	0.0182	-0.0114	0.3326	0.2975	0.0062	0.0665023	Both
CN	amoA_B_Cluster4	0.0125	-0.0092	0.3857	0.3534	0.0027	0.0340882	Both
CN	amoA_B_Cluster5	0.0928	-0.0345	0.5154	0.4899	0.0002	0.006651	Both
CN	amoA_B_Cluster6	0.0233	-0.0094	0.3055	0.269	0.0094	0.0909126	Both
CN	amoA_B_Cluster7	0.0179	-0.0059	0.0896	0.0417	0.1874	0.5055226	Both
CN	amoA_B_Cluster8	0.0207	-0.0109	0.2231	0.1822	0.0306	0.1796732	Both
CN	amoA_B_Cluster9	0.0549	-0.0326	0.3994	0.3678	0.0021	0.0290314	Both
CN	nifh_Cluster0	0.0332	0.0077	0.2343	0.194	0.0262	0.1635862	Both
CN	nifh_Cluster10	0.005	0.0019	0.034	-0.0169	0.4238	0.7129848	Both
CN	nifh_Cluster100	0.0019	0.0005	0.0151	-0.0367	0.5953	0.7907503	Both
CN	nifh_Cluster103	0.0383	-0.0166	0.4086	0.3775	0.0018	0.0266972	Both
CN	nifh_Cluster1034	0.0262	-0.0122	0.1624	0.1183	0.0701	0.3131529	Both
CN	nifh_Cluster105	0.013	-0.0042	0.0717	0.0229	0.2405	0.5333455	Both
CN	nifh_Cluster108	0.0083	-0.0018	0.0417	-0.0087	0.3744	0.6632753	Both
CN	nifh_Cluster1099	0.066	0.0452	0.4743	0.4467	0.0006	0.0109325	Both
CN	nifh_Cluster11	0.0021	-0.0002	0.0058	-0.0465	0.7428	0.841796	Both
CN	nifh_Cluster1112	0.0029	-0.0012	0.0633	0.014	0.2713	0.5663201	Both
CN	nifh_Cluster112	0.0112	0.0054	0.2105	0.169	0.0364	0.1989639	Both
CN	nifh_Cluster113	0.0327	-0.0014	0.0048	-0.0476	0.7665	0.8484465	Both
CN	nifh_Cluster114	0.0332	0.0017	0.0148	-0.037	0.599	0.7907503	Both
CN	nifh_Cluster1141	0.0251	-0.0097	0.1685	0.1248	0.0645	0.2951275	Both
CN	nifh_Cluster115	0.0095	0.0006	0.0057	-0.0466	0.744	0.841796	Both
CN	nifh_Cluster116	0.0775	-0.0226	0.2269	0.1862	0.029	0.1730459	Both
CN	nifh_Cluster1163	0.0044	-0.0013	0.072	0.0232	0.2395	0.5331685	Both
CN	nifh_Cluster1179	0.0031	-0.0014	0.0636	0.0143	0.2703	0.5663201	Both
CN	nifh_Cluster1182	0.0204	-1.00E-04	1.00E-04	-0.0525	0.9661	0.9750586	Both
CN	nifh_Cluster1183	0.0066	0.0032	0.2849	0.2472	0.0127	0.1040892	Both
CN	nifh_Cluster1197	0.0115	-0.0024	0.0536	0.0038	0.3127	0.6109736	Both
CN	nifh_Cluster1199	0.0046	-0.0005	0.0071	-0.0451	0.7157	0.838774	Both
CN	nifh_Cluster120	0.0149	-0.0031	0.0123	-0.0397	0.6322	0.8070139	Both
CN	nifh_Cluster1203	0.0155	-0.0073	0.1366	0.0911	0.0992	0.356893	Both
CN	nifh_Cluster1206	0.0043	-0.0024	0.1931	0.1506	0.0463	0.2421031	Both
CN	nifh_Cluster1207	0.0074	-0.0032	0.112	0.0653	0.1381	0.4427947	Both
CN	nifh_Cluster1209	0.1506	-0.0183	0.1082	0.0613	0.1453	0.4509092	Both
CN	nifh_Cluster121	0.0095	-0.0021	0.0154	-0.0364	0.5918	0.7907503	Both
CN	nifh_Cluster1211	0.0056	-0.0014	0.0525	0.0026	0.3178	0.6174487	Both
CN	nifh_Cluster1213	0.0026	-1.00E-04	0.0008	-0.0518	0.9022	0.9404111	Both
CN	nifh_Cluster122	0.0029	-0.0011	0.0459	-0.0043	0.3509	0.6449279	Both
CN	nifh_Cluster123	0.0095	0.0022	0.0162	-0.0355	0.5819	0.7907503	Both
CN	nifh_Cluster1236	0.0234	-0.0081	0.0485	-0.0016	0.3377	0.6344534	Both
CN	nifh_Cluster1238	0.0028	0.00E+00	1.00E-04	-0.0525	0.9679	0.9750586	Both
CN	nifh_Cluster124	0.0091	0.00E+00	0.00E+00	-0.0526	0.994	0.9939749	Both
CN	nifh_Cluster1261	0.002	-0.0006	0.0213	-0.0302	0.5277	0.7727341	Both
CN	nifh_Cluster1262	0.0065	-0.0019	0.0471	-0.003	0.3446	0.6393425	Both
CN	nifh_Cluster1266	0.0094	0.0053	0.2827	0.2449	0.0131	0.1060556	Both
CN	nifh_Cluster1267	0.0562	0.0424	0.4827	0.4555	0.0005	0.00998	Both
CN	nifh_Cluster127	0.0026	-0.0008	0.0252	-0.0261	0.4917	0.7476501	Both

CN	nifh_Cluster1278	0.1602	-0.0674	0.2638	0.2251	0.0172	0.1296235	Both
CN	nifh_Cluster128	0.0036	-0.0005	0.0108	-0.0412	0.6536	0.8101524	Both
CN	nifh_Cluster1289	0.0039	-0.0006	0.015	-0.0369	0.5972	0.7907503	Both
CN	nifh_Cluster129	0.0077	0.0011	0.0071	-0.0451	0.7162	0.838774	Both
CN	nifh_Cluster1292	0.0086	-0.0015	0.0219	-0.0296	0.5225	0.7687411	Both
CN	nifh_Cluster130	0.0059	-0.0011	0.0086	-0.0436	0.6891	0.8331915	Both
CN	nifh_Cluster1305	0.0048	0.0013	0.0652	0.016	0.2641	0.5624248	Both
CN	nifh_Cluster1306	0.005	-0.0016	0.0734	0.0246	0.235	0.5291731	Both
CN	nifh_Cluster131	0.0051	-0.0014	0.0244	-0.0269	0.4988	0.7479042	Both
CN	nifh_Cluster1319	0.0036	-0.0021	0.1237	0.0776	0.1179	0.3929886	Both
CN	nifh_Cluster132	0.0064	0.0012	0.016	-0.0358	0.5845	0.7907503	Both
CN	nifh_Cluster1320	0.0025	-1.00E-04	0.0002	-0.0525	0.956	0.9750586	Both
CN	nifh_Cluster1323	0.0023	-0.0004	0.0173	-0.0344	0.5698	0.7907503	Both
CN	nifh_Cluster1324	0.03	0.0139	0.2596	0.2207	0.0183	0.134952	Both
CN	nifh_Cluster1336	0.0153	0.0007	0.0068	-0.0455	0.722	0.838774	Both
CN	nifh_Cluster1340	0.0363	-0.017	0.1127	0.066	0.1368	0.4411184	Both
CN	nifh_Cluster1345	0.0483	0.0324	0.3804	0.3478	0.0029	0.0356674	Both
CN	nifh_Cluster1370	0.0021	-0.0004	0.0118	-0.0402	0.6392	0.8095113	Both
CN	nifh_Cluster1375	0.0018	0.0005	0.0339	-0.0169	0.4242	0.7129848	Both
CN	nifh_Cluster139	0.0041	0.0017	0.0763	0.0277	0.2255	0.5214893	Both
CN	nifh_Cluster140	0.0043	-0.001	0.0379	-0.0127	0.3976	0.6879379	Both
CN	nifh_Cluster141	0.0677	-0.035	0.5995	0.5784	0.00E+00	0.0014342	Both
CN	nifh_Cluster148	0.0046	-0.003	0.1306	0.0848	0.1075	0.3738039	Both
CN	nifh_Cluster1480	0.001	0.0011	0.2893	0.2519	0.0119	0.0995937	Both
CN	nifh_Cluster152	0.0599	0.0028	0.0068	-0.0455	0.7228	0.838774	Both
CN	nifh_Cluster156	0.0659	0.0425	0.736	0.7221	0.00E+00	0.0001032	Both
CN	nifh_Cluster1562	0.0543	0.053	0.6423	0.6234	0.00E+00	0.0007444	Both
CN	nifh_Cluster158	0.0144	-0.0066	0.1482	0.1033	0.0849	0.3399182	Both
CN	nifh_Cluster16	0.0019	-0.0006	0.0208	-0.0307	0.5328	0.7730399	Both
CN	nifh_Cluster166	0.0172	0.0052	0.0281	-0.0231	0.4679	0.7361457	Both
CN	nifh_Cluster205	0.0122	0.0006	0.0023	-0.0502	0.8358	0.8998923	Both
CN	nifh_Cluster21	0.0021	-0.0017	0.2368	0.1967	0.0253	0.1603469	Both
CN	nifh_Cluster222	0.006	0.0002	0.0004	-0.0523	0.935	0.9628657	Both
CN	nifh_Cluster225	0.0189	-0.0019	0.023	-0.0285	0.512	0.7595595	Both
CN	nifh_Cluster230	0.0042	-0.0006	0.0136	-0.0383	0.615	0.7989048	Both
CN	nifh_Cluster231	0.0022	0.001	0.0627	0.0134	0.2736	0.5663201	Both
CN	nifh_Cluster236	0.0027	-0.0017	0.2111	0.1696	0.0361	0.1989639	Both
CN	nifh_Cluster237	0.0058	-0.001	0.0253	-0.026	0.4912	0.7476501	Both
CN	nifh_Cluster243	0.0333	0.0157	0.2274	0.1868	0.0288	0.1730459	Both
CN	nifh_Cluster2432	0.0436	0.0324	0.5484	0.5246	0.0001	0.0038375	Both
CN	nifh_Cluster2433	0.0032	0.0014	0.058	0.0084	0.2929	0.5864614	Both
CN	nifh_Cluster246	0.0057	-0.0008	0.0099	-0.0422	0.6675	0.8196489	Both
CN	nifh_Cluster25	0.0295	0.0178	0.5985	0.5774	0.00E+00	0.0014342	Both
CN	nifh_Cluster256	0.0029	0.0002	0.0022	-0.0504	0.8414	0.9035006	Both
CN	nifh_Cluster264	0.0029	0.0015	0.159	0.1147	0.0734	0.3200022	Both
CN	nifh_Cluster265	0.0736	0.0197	0.302	0.2653	0.0099	0.0909126	Both
CN	nifh_Cluster267	0.0109	0.0063	0.2455	0.2058	0.0224	0.1483336	Both
CN	nifh_Cluster268	0.0111	0.0021	0.057	0.0074	0.2972	0.5864614	Both
CN	nifh_Cluster269	0.023	-0.0032	0.0147	-0.0371	0.6002	0.7907503	Both
CN	nifh_Cluster272	0.0041	0.0059	0.7003	0.6845	0.00E+00	0.0001908	Both
CN	nifh_Cluster274	0.0032	0.0029	0.0807	0.0323	0.212	0.520961	Both
CN	nifh_Cluster275	0.0036	-0.0017	0.1447	0.0997	0.089	0.3412453	Both
CN	nifh_Cluster278	0.0077	0.005	0.3145	0.2784	0.0082	0.0832002	Both
CN	nifh_Cluster279	0.006	0.0012	0.0509	0.0009	0.3255	0.6234669	Both
CN	nifh_Cluster28	0.002	0.0008	0.066	0.0168	0.261	0.5600046	Both

CN	nifh_Cluster280	0.0031	-0.0015	0.0588	0.0092	0.2897	0.5864614	Both
CN	nifh_Cluster282	0.0052	0.00E+00	1.00E-04	-0.0526	0.9725	0.9774936	Both
CN	nifh_Cluster287	0.0022	-0.0006	0.034	-0.0169	0.4237	0.7129848	Both
CN	nifh_Cluster290	0.011	0.003	0.0752	0.0265	0.2289	0.5214893	Both
CN	nifh_Cluster297	0.0029	-0.0015	0.0482	-0.0018	0.3387	0.6344534	Both
CN	nifh_Cluster299	0.0026	0.0007	0.0244	-0.027	0.499	0.7479042	Both
CN	nifh_Cluster303	0.002	0.0014	0.1443	0.0993	0.0894	0.3412453	Both
CN	nifh_Cluster304	0.0024	0.0003	0.0092	-0.0429	0.6791	0.8236502	Both
CN	nifh_Cluster313	0.0103	-0.001	0.0031	-0.0493	0.8099	0.8800088	Both
CN	nifh_Cluster314	0.0022	0.0004	0.0092	-0.043	0.6799	0.8236502	Both
CN	nifh_Cluster317	0.0071	-0.0023	0.0832	0.035	0.2048	0.5207008	Both
CN	nifh_Cluster32	0.0033	-0.0006	0.0196	-0.032	0.5448	0.7782218	Both
CN	nifh_Cluster320	12.7419	-2.0613	0.4311	0.4011	0.0012	0.0185446	Both
CN	nifh_Cluster334	0.0063	-0.0009	0.0055	-0.0468	0.7483	0.8422858	Both
CN	nifh_Cluster335	0.0075	0.0036	0.0474	-0.0027	0.343	0.6383773	Both
CN	nifh_Cluster338	0.0123	-0.0045	0.1561	0.1117	0.0763	0.3201541	Both
CN	nifh_Cluster340	0.007	0.0047	0.0653	0.0161	0.2634	0.5624248	Both
CN	nifh_Cluster341	0.0023	0.0012	0.0946	0.0469	0.1751	0.4990167	Both
CN	nifh_Cluster35	0.0078	-0.0021	0.0899	0.042	0.1867	0.5055226	Both
CN	nifh_Cluster351	0.0025	-0.0006	0.0157	-0.0361	0.5884	0.7907503	Both
CN	nifh_Cluster3574	0.1093	-0.0352	0.3379	0.303	0.0057	0.0624544	Both
CN	nifh_Cluster3691	0.0174	-0.0086	0.146	0.1011	0.0874	0.3403133	Both
CN	nifh_Cluster37	0.0329	-0.0039	0.011	-0.0411	0.6512	0.8096861	Both
CN	nifh_Cluster374	0.0021	-0.0012	0.08	0.0316	0.2142	0.520961	Both
CN	nifh_Cluster38	0.0009	0.0004	0.0385	-0.0121	0.3937	0.6831732	Both
CN	nifh_Cluster382	0.0016	-0.0008	0.0349	-0.0159	0.4177	0.7107665	Both
CN	nifh_Cluster384	0.0019	-0.0001	0.0013	-0.0512	0.8756	0.925849	Both
CN	nifh_Cluster386	0.0035	0.0002	0.001	-0.0516	0.8922	0.9333792	Both
CN	nifh_Cluster389	0.0031	0.0012	0.043	-0.0074	0.3672	0.6585375	Both
CN	nifh_Cluster394	0.002	-0.0006	0.0129	-0.039	0.6235	0.7989048	Both
CN	nifh_Cluster40	0.0012	-0.0008	0.1143	0.0677	0.1339	0.438812	Both
CN	nifh_Cluster43	0.0108	0.0021	0.0623	0.0129	0.2753	0.5663201	Both
CN	nifh_Cluster434	0.0035	-0.0011	0.034	-0.0168	0.4234	0.7129848	Both
CN	nifh_Cluster440	0.0133	-0.0068	0.1551	0.1107	0.0773	0.321071	Both
CN	nifh_Cluster462	0.0034	-0.0009	0.0226	-0.0289	0.5158	0.7627473	Both
CN	nifh_Cluster470	0.0152	0.0002	0.0002	-0.0525	0.9562	0.9750586	Both
CN	nifh_Cluster49	0.0213	0.0015	0.0019	-0.0506	0.8496	0.9096912	Both
CN	nifh_Cluster499	0.0026	-1.00E-04	0.0002	-0.0524	0.9503	0.9750586	Both
CN	nifh_Cluster5	0.0256	-0.0014	0.0109	-0.0411	0.6519	0.8096861	Both
CN	nifh_Cluster506	0.0048	0.0001	0.0004	-0.0522	0.9297	0.9614782	Both
CN	nifh_Cluster51	0.0028	0.00E+00	1.00E-04	-0.0526	0.9745	0.9777904	Both
CN	nifh_Cluster52	0.0336	-0.0032	0.0052	-0.0472	0.7563	0.8462205	Both
CN	nifh_Cluster525	0.0033	-0.0026	0.1079	0.061	0.146	0.4509092	Both
CN	nifh_Cluster531	0.0258	0.022	0.6263	0.6066	0.00E+00	0.0009138	Both
CN	nifh_Cluster539	0.0048	0.0006	0.003	-0.0495	0.8143	0.8831105	Both
CN	nifh_Cluster578	0.0481	-0.0123	0.1105	0.0637	0.1409	0.4469625	Both
CN	nifh_Cluster58	0.3542	0.2525	0.513	0.4874	0.0003	0.0066712	Both
CN	nifh_Cluster60	0.0089	-0.0012	0.0112	-0.0408	0.648	0.8096861	Both
CN	nifh_Cluster61	0.0025	0.0002	0.0021	-0.0504	0.8422	0.9035006	Both
CN	nifh_Cluster65	0.009	0.0014	0.0129	-0.0391	0.6242	0.7989048	Both
CN	nifh_Cluster686	0.0009	-0.0003	0.0247	-0.0267	0.4965	0.7479042	Both
CN	nifh_Cluster69	0.009	-0.001	0.0063	-0.046	0.7328	0.8394725	Both
CN	nifh_Cluster70	0.0111	-0.0016	0.006	-0.0463	0.7376	0.8394725	Both
CN	nifh_Cluster717	0.003	0.0016	0.1796	0.1364	0.0555	0.2686404	Both
CN	nifh_Cluster725	0.0058	0.0044	0.4395	0.4099	0.0011	0.0174007	Both

CN	nifh_Cluster727	0.0039	-0.002	0.1226	0.0764	0.1197	0.3946363	Both
CN	nifh_Cluster73	0.0106	-0.0029	0.0438	-0.0065	0.3625	0.6544431	Both
CN	nifh_Cluster74	0.0049	0.0004	0.0019	-0.0507	0.8526	0.9097061	Both
CN	nifh_Cluster748	0.0081	-0.0026	0.1337	0.0881	0.1031	0.3643529	Both
CN	nifh_Cluster749	0.0071	0.0018	0.0673	0.0182	0.2561	0.5575579	Both
CN	nifh_Cluster755	0.0102	-0.0069	0.2888	0.2514	0.012	0.0995937	Both
CN	nifh_Cluster76	0.0029	-0.0008	0.0332	-0.0176	0.4289	0.7189601	Both
CN	nifh_Cluster79	0.0022	-1.00E-04	0.0004	-0.0522	0.9305	0.9614782	Both
CN	nifh_Cluster81	0.0055	-0.001	0.0111	-0.041	0.6498	0.8096861	Both
CN	nifh_Cluster82	0.0011	-0.0005	0.0421	-0.0083	0.3724	0.661872	Both
CN	nifh_Cluster83	0.0069	0.0012	0.0139	-0.038	0.6112	0.797851	Both
CN	nifh_Cluster86	0.0135	-0.0061	0.2183	0.1772	0.0327	0.1856208	Both
CN	nifh_Cluster868	0.0099	-0.0001	0.0002	-0.0525	0.9552	0.9750586	Both
CN	nifh_Cluster87	0.0024	0.00E+00	1.00E-04	-0.0525	0.9668	0.9750586	Both
CN	nifh_Cluster88	0.0039	-0.0019	0.078	0.0295	0.2201	0.5214893	Both
CN	nifh_Cluster9	0.0078	0.0004	0.0016	-0.051	0.8638	0.9150206	Both
CN	nifh_Cluster92	0.0051	-0.0008	0.0066	-0.0457	0.7266	0.838774	Both
CN	nifh_Cluster93	0.0036	0.0006	0.0115	-0.0405	0.6434	0.8096861	Both
CN	nifh_Cluster94	0.0047	0.0003	0.001	-0.0516	0.8909	0.9333792	Both
CN	nifh_Cluster95	0.0121	-0.0015	0.0073	-0.0449	0.7127	0.838774	Both
CN	nifh_Cluster96	0.0123	-0.0009	0.0056	-0.0468	0.7477	0.8422858	Both
CN	nifh_Cluster97	0.018	0.0126	0.3754	0.3425	0.0032	0.0379298	Both
CN	nifh_Cluster98	0.0059	0.0008	0.0164	-0.0354	0.5806	0.7907503	Both
CN	nifh_Cluster99	0.0033	0.0006	0.0102	-0.0419	0.6631	0.8184135	Both
CN	nirk_Cluster0	5.9009	-1.2619	0.7101	0.6948	0.00E+00	0.0001908	Both
CN	nirk_Cluster1	0.1708	-0.1136	0.6899	0.6735	0.00E+00	0.0002324	Both
CN	nirk_Cluster10	0.5713	-0.1404	0.3021	0.2653	0.0099	0.0909126	Both
CN	nirk_Cluster101	0.0031	-0.0002	0.0012	-0.0514	0.8832	0.930511	Both
CN	nirk_Cluster102	0.0426	-0.0154	0.1701	0.1265	0.0631	0.291076	Both
CN	nirk_Cluster103	0.0754	-0.0383	0.4392	0.4096	0.0011	0.0174007	Both
CN	nirk_Cluster104	0.0023	-0.0015	0.1249	0.0788	0.1161	0.3894679	Both
CN	nirk_Cluster105	0.0946	-0.036	0.2543	0.2151	0.0197	0.1405549	Both
CN	nirk_Cluster106	0.0592	-0.0238	0.1632	0.1192	0.0693	0.3131529	Both
CN	nirk_Cluster107	0.0891	-0.0339	0.2228	0.1819	0.0308	0.1796732	Both
CN	nirk_Cluster108	0.0151	-0.0023	0.0205	-0.031	0.5356	0.7730399	Both
CN	nirk_Cluster109	0.0115	-0.005	0.1543	0.1098	0.0781	0.3224144	Both
CN	nirk_Cluster11	0.1401	-0.0101	0.0846	0.0364	0.201	0.5201002	Both
CN	nirk_Cluster12	0.094	-0.023	0.2525	0.2132	0.0202	0.1405549	Both
CN	nirk_Cluster13	0.0408	-0.0028	0.031	-0.02	0.445	0.722729	Both
CN	nirk_Cluster14	0.0946	-0.0321	0.47	0.4421	0.0006	0.0114791	Both
CN	nirk_Cluster15	0.3358	-0.1415	0.3206	0.2848	0.0075	0.0771674	Both
CN	nirk_Cluster16	0.4727	-0.1993	0.295	0.2578	0.011	0.0965218	Both
CN	nirk_Cluster17	0.0998	-0.0015	0.0017	-0.0509	0.8606	0.9150206	Both
CN	nirk_Cluster18	0.1296	-0.0208	0.0493	-0.0008	0.3335	0.6286585	Both
CN	nirk_Cluster19	0.2776	-0.0299	0.1267	0.0808	0.1132	0.3855765	Both
CN	nirk_Cluster2	0.2032	-0.0115	0.0279	-0.0233	0.4692	0.7362147	Both
CN	nirk_Cluster20	0.1738	-0.0162	0.0315	-0.0195	0.4417	0.722729	Both
CN	nirk_Cluster21	0.1532	-0.0277	0.267	0.2284	0.0165	0.1262175	Both
CN	nirk_Cluster22	0.1291	-0.0339	0.3026	0.2659	0.0098	0.0909126	Both
CN	nirk_Cluster23	0.0673	-0.0071	0.1085	0.0616	0.1448	0.4509092	Both
CN	nirk_Cluster24	0.0654	-0.0252	0.4845	0.4574	0.0005	0.00998	Both
CN	nirk_Cluster25	0.4782	-0.1947	0.7352	0.7213	0.00E+00	0.0001032	Both
CN	nirk_Cluster26	0.105	-0.0124	0.107	0.0599	0.1479	0.4520862	Both
CN	nirk_Cluster27	0.1727	-0.0527	0.3068	0.2703	0.0092	0.0909126	Both
CN	nirk_Cluster28	0.0685	-0.0138	0.1352	0.0897	0.1011	0.3613374	Both

CN	nirk_Cluster29	0.1526	-0.0126	0.0455	-0.0047	0.353	0.6468595	Both
CN	nirk_Cluster3	0.3023	-0.0742	0.3821	0.3496	0.0028	0.035416	Both
CN	nirk_Cluster30	0.0556	-0.0235	0.2939	0.2567	0.0111	0.0965218	Both
CN	nirk_Cluster31	0.1501	0.0159	0.1399	0.0946	0.0948	0.3519325	Both
CN	nirk_Cluster32	0.1254	-0.0547	0.2534	0.2141	0.02	0.1405549	Both
CN	nirk_Cluster33	0.0638	-0.0256	0.1929	0.1504	0.0464	0.2421031	Both
CN	nirk_Cluster34	0.0416	-0.0046	0.0273	-0.0239	0.4741	0.7376224	Both
CN	nirk_Cluster35	0.1007	-0.0347	0.1401	0.0949	0.0945	0.3519325	Both
CN	nirk_Cluster36	0.0017	-0.0007	0.0467	-0.0035	0.3469	0.6411583	Both
CN	nirk_Cluster39	0.0035	-0.0007	0.0067	-0.0456	0.7248	0.838774	Both
CN	nirk_Cluster4	0.1323	-0.0255	0.1494	0.1046	0.0835	0.3395619	Both
CN	nirk_Cluster40	0.0455	-0.0167	0.3903	0.3582	0.0025	0.0323337	Both
CN	nirk_Cluster41	0.1562	-0.0481	0.5302	0.5055	0.0002	0.0053604	Both
CN	nirk_Cluster44	0.0669	-0.0404	0.5015	0.4753	0.0003	0.0080546	Both
CN	nirk_Cluster46	0.0154	-0.0068	0.1375	0.0921	0.098	0.3557214	Both
CN	nirk_Cluster48	0.003	-0.0016	0.138	0.0926	0.0973	0.3557214	Both
CN	nirk_Cluster49	0.0057	-0.0031	0.1248	0.0788	0.1162	0.3894679	Both
CN	nirk_Cluster5	0.1932	-0.0312	0.2021	0.1601	0.0409	0.2193011	Both
CN	nirk_Cluster50	0.0016	0.0003	0.0111	-0.041	0.6501	0.8096861	Both
CN	nirk_Cluster52	0.0452	-0.0172	0.3229	0.2872	0.0072	0.0758705	Both
CN	nirk_Cluster53	0.0038	-0.0005	0.0065	-0.0458	0.728	0.838774	Both
CN	nirk_Cluster54	0.002	-0.0002	0.0048	-0.0476	0.7658	0.8484465	Both
CN	nirk_Cluster55	0.0023	-0.0003	0.0036	-0.0488	0.795	0.8734719	Both
CN	nirk_Cluster56	0.0009	-0.0004	0.0313	-0.0197	0.4427	0.722729	Both
CN	nirk_Cluster57	0.0016	0.0005	0.0144	-0.0374	0.6038	0.7907503	Both
CN	nirk_Cluster58	0.0088	-0.005	0.2476	0.208	0.0217	0.1456068	Both
CN	nirk_Cluster59	0.0058	-0.002	0.0785	0.03	0.2188	0.5214893	Both
CN	nirk_Cluster6	0.1558	-0.0485	0.4863	0.4593	0.0004	0.00998	Both
CN	nirk_Cluster60	0.0006	0.0002	0.0133	-0.0386	0.6189	0.7989048	Both
CN	nirk_Cluster62	0.1417	-0.0927	0.6006	0.5796	0.00E+00	0.0014342	Both
CN	nirk_Cluster63	0.1284	-0.0786	0.8101	0.8001	0.00E+00	1.64E-05	Both
CN	nirk_Cluster64	0.0044	0.00E+00	1.00E-04	-0.0525	0.9684	0.9750586	Both
CN	nirk_Cluster65	0.0464	-0.0144	0.2516	0.2122	0.0205	0.1408722	Both
CN	nirk_Cluster66	0.0068	-0.004	0.2477	0.2081	0.0217	0.1456068	Both
CN	nirk_Cluster67	0.0174	-0.008	0.157	0.1126	0.0754	0.3200022	Both
CN	nirk_Cluster68	0.0125	-0.0084	0.4311	0.4012	0.0012	0.0185446	Both
CN	nirk_Cluster69	0.0305	-0.0117	0.3033	0.2666	0.0097	0.0909126	Both
CN	nirk_Cluster7	0.0804	-0.0242	0.2764	0.2383	0.0144	0.1137815	Both
CN	nirk_Cluster70	0.0721	-0.0573	0.4778	0.4503	0.0005	0.0105795	Both
CN	nirk_Cluster71	0.0053	-0.0017	0.0674	0.0183	0.2558	0.5575579	Both
CN	nirk_Cluster72	0.0239	-0.0115	0.4477	0.4187	0.0009	0.0158857	Both
CN	nirk_Cluster73	0.1444	-0.0749	0.4474	0.4183	0.0009	0.0158857	Both
CN	nirk_Cluster74	0.6398	-0.4438	0.7345	0.7205	0.00E+00	0.0001032	Both
CN	nirk_Cluster75	0.0147	-0.0086	0.184	0.1411	0.0523	0.2616185	Both
CN	nirk_Cluster76	0.1055	-0.0548	0.6247	0.6049	0.00E+00	0.0009138	Both
CN	nirk_Cluster77	0.0081	-0.0041	0.0539	0.0041	0.3111	0.6097896	Both
CN	nirk_Cluster78	0.2407	-0.1075	0.3911	0.359	0.0024	0.0323337	Both
CN	nirk_Cluster79	0.0069	-0.0035	0.0841	0.0359	0.2022	0.5201002	Both
CN	nirk_Cluster8	0.0982	-0.025	0.1388	0.0935	0.0963	0.3550364	Both
CN	nirk_Cluster80	0.2665	-0.133	0.3996	0.368	0.0021	0.0290314	Both
CN	nirk_Cluster82	0.0015	-0.0015	0.1618	0.1176	0.0707	0.3135888	Both
CN	nirk_Cluster83	0.004	-0.0009	0.0307	-0.0203	0.4476	0.722729	Both
CN	nirk_Cluster84	0.0207	0.0005	0.0004	-0.0523	0.9351	0.9628657	Both
CN	nirk_Cluster85	0.004	-0.0015	0.0782	0.0296	0.2197	0.5214893	Both
CN	nirk_Cluster86	0.0286	-0.0199	0.528	0.5032	0.0002	0.0053604	Both

CN	nirk_Cluster87	0.0115	-0.0077	0.4035	0.3721	0.002	0.028404	Both
CN	nirk_Cluster88	0.0032	-0.0008	0.0394	-0.0111	0.3882	0.6779851	Both
CN	nirk_Cluster89	0.0065	-0.002	0.0515	0.0016	0.3225	0.6217477	Both
CN	nirk_Cluster9	0.0517	-0.0097	0.0622	0.0129	0.2755	0.5663201	Both
CN	nirk_Cluster90	0.0067	-0.0032	0.1024	0.0552	0.1573	0.4712013	Both
CN	nirk_Cluster91	0.0374	-0.0154	0.2994	0.2625	0.0103	0.0931163	Both
CN	nirk_Cluster92	0.0072	-0.0053	0.291	0.2537	0.0116	0.0992592	Both
CN	nirk_Cluster93	0.0308	-0.0077	0.1095	0.0626	0.1429	0.4507726	Both
CN	nirk_Cluster94	0.0175	-0.0063	0.1412	0.096	0.0932	0.3505828	Both
CN	nirk_Cluster95	0.0473	-0.025	0.3628	0.3293	0.0039	0.0446769	Both
CN	nirk_Cluster96	0.0472	-0.0183	0.2437	0.2039	0.0229	0.1487402	Both
CN	nirk_Cluster97	0.0306	-0.0085	0.1293	0.0835	0.1094	0.377441	Both
CN	nirk_Cluster98	0.0181	-0.007	0.1059	0.0588	0.15	0.4562653	Both
CN	nirk_Cluster99	0.006	-0.0032	0.1594	0.1151	0.073	0.3200022	Both
CN	nirs_Cluster0	0.1409	-0.037	0.294	0.2569	0.0111	0.0965218	Both
CN	nirs_Cluster1	0.004	-0.0012	0.0284	-0.0227	0.4649	0.7333793	Both
CN	nirs_Cluster14	0.0107	-0.0024	0.0115	-0.0405	0.6438	0.8096861	Both
CN	nirs_Cluster2	0.0139	-0.0065	0.0814	0.0331	0.21	0.520961	Both
CN	nirs_Cluster26	0.0005	-1.00E-04	0.0032	-0.0492	0.8068	0.8798973	Both
CN	nirs_Cluster28	0.0071	-0.0028	0.0321	-0.0189	0.4373	0.7207178	Both
CN	nirs_Cluster3	0.1715	-0.0281	0.2339	0.1936	0.0263	0.1635862	Both
CN	nirs_Cluster36	0.0013	-0.0006	0.0161	-0.0357	0.584	0.7907503	Both
CN	nirs_Cluster37	0.0066	-0.0023	0.0138	-0.0382	0.6127	0.798	Both
CN	nirs_Cluster4	0.0002	-1.00E-04	0.0064	-0.0459	0.73	0.838774	Both
CN	nirs_Cluster50	0.002	0.0002	0.0017	-0.0509	0.861	0.9150206	Both
CN	nirs_Cluster9	0.0269	-0.0144	0.059	0.0095	0.2887	0.5864614	Both
CN	norb_Cluster0	0.0775	-0.0297	0.2405	0.2005	0.024	0.1540079	Both
CN	norb_Cluster1	1.1042	-0.4546	0.2031	0.1612	0.0403	0.2183124	Both
CN	norb_Cluster101	0.0026	-0.0014	0.0666	0.0175	0.2586	0.5600036	Both
CN	norb_Cluster102	0.0038	-0.0013	0.0582	0.0086	0.2921	0.5864614	Both
CN	norb_Cluster103	0.0018	-0.0007	0.0365	-0.0142	0.4066	0.7014925	Both
CN	norb_Cluster104	0.0023	-0.0003	0.0044	-0.048	0.7749	0.8561672	Both
CN	norb_Cluster106	0.125	-0.0288	0.0909	0.0431	0.1841	0.5055226	Both
CN	norb_Cluster107	0.0133	-0.0043	0.0841	0.0359	0.2023	0.5201002	Both
CN	norb_Cluster108	0.0052	-0.0024	0.1583	0.114	0.074	0.3200022	Both
CN	norb_Cluster109	0.0227	-0.0028	0.0161	-0.0357	0.5842	0.7907503	Both
CN	norb_Cluster11	0.0126	-0.0055	0.1441	0.0991	0.0896	0.3412453	Both
CN	norb_Cluster110	0.0099	-0.0035	0.0821	0.0338	0.2079	0.520961	Both
CN	norb_Cluster112	0.0171	-0.0044	0.0807	0.0323	0.212	0.520961	Both
CN	norb_Cluster113	0.0134	-0.0016	0.0096	-0.0426	0.6731	0.8222076	Both
CN	norb_Cluster114	0.0025	-0.0009	0.019	-0.0327	0.5516	0.780305	Both
CN	norb_Cluster115	0.0137	0.0147	0.0895	0.0415	0.1878	0.5055226	Both
CN	norb_Cluster116	0.0072	-0.0012	0.0122	-0.0398	0.6333	0.8070139	Both
CN	norb_Cluster117	0.0027	-0.0006	0.0183	-0.0333	0.5584	0.7844505	Both
CN	norb_Cluster118	0.0037	-0.001	0.041	-0.0095	0.3787	0.6688926	Both
CN	norb_Cluster119	0.019	-0.0076	0.0953	0.0476	0.1734	0.4986086	Both
CN	norb_Cluster12	0.0047	-0.0011	0.0202	-0.0313	0.5385	0.774896	Both
CN	norb_Cluster121	0.0017	-0.0007	0.026	-0.0253	0.4853	0.7450038	Both
CN	norb_Cluster122	0.0144	-0.0031	0.0432	-0.0071	0.3659	0.658127	Both
CN	norb_Cluster123	0.0053	-0.0011	0.02	-0.0316	0.5413	0.7751303	Both
CN	norb_Cluster124	0.682	-0.1426	0.0664	0.0173	0.2593	0.5600036	Both
CN	norb_Cluster125	0.004	-0.0011	0.0389	-0.0117	0.3917	0.6817498	Both
CN	norb_Cluster126	0.0082	-0.0024	0.0592	0.0097	0.2878	0.5864614	Both
CN	norb_Cluster127	0.1735	-0.0549	0.1304	0.0847	0.1077	0.3738039	Both
CN	norb_Cluster128	0.0115	-0.003	0.0775	0.0289	0.2218	0.5214893	Both

CN	norb_Cluster13	0.0045	-0.0023	0.0817	0.0334	0.209	0.520961	Both
CN	norb_Cluster131	0.0184	-0.001	0.0032	-0.0493	0.809	0.8800088	Both
CN	norb_Cluster132	1.0357	-0.3335	0.2186	0.1775	0.0326	0.1856208	Both
CN	norb_Cluster133	0.0018	-0.0008	0.0572	0.0076	0.2963	0.5864614	Both
CN	norb_Cluster135	0.0567	-0.0165	0.1115	0.0648	0.139	0.4433182	Both
CN	norb_Cluster136	0.0213	-0.0055	0.0798	0.0313	0.2148	0.520961	Both
CN	norb_Cluster139	0.0328	-0.008	0.0821	0.0338	0.2078	0.520961	Both
CN	norb_Cluster14	0.0026	-0.0017	0.1577	0.1133	0.0747	0.3200022	Both
CN	norb_Cluster140	0.0437	-0.0225	0.3488	0.3146	0.0048	0.0536271	Both
CN	norb_Cluster142	0.0015	-0.0007	0.0358	-0.0149	0.4112	0.7052288	Both
CN	norb_Cluster144	0.0098	-0.0022	0.0423	-0.0081	0.371	0.6613689	Both
CN	norb_Cluster146	0.0348	-0.0118	0.1752	0.1318	0.059	0.2761965	Both
CN	norb_Cluster15	0.0469	-0.0147	0.1138	0.0671	0.1349	0.4396248	Both
CN	norb_Cluster150	0.0157	-0.0071	0.276	0.2379	0.0145	0.1137815	Both
CN	norb_Cluster155	0.1631	-0.0369	0.0858	0.0377	0.1976	0.5201002	Both
CN	norb_Cluster16	0.0131	-0.0056	0.1084	0.0615	0.145	0.4509092	Both
CN	norb_Cluster164	0.0199	-0.0106	0.214	0.1727	0.0347	0.1934677	Both
CN	norb_Cluster165	0.0076	-0.0015	0.0179	-0.0338	0.5632	0.7859822	Both
CN	norb_Cluster166	0.0039	-0.0004	0.0036	-0.0489	0.7965	0.8734719	Both
CN	norb_Cluster169	0.0238	-0.0109	0.1466	0.1017	0.0866	0.3403133	Both
CN	norb_Cluster17	0.0028	-0.0021	0.2301	0.1896	0.0278	0.1705772	Both
CN	norb_Cluster170	0.0243	-0.005	0.0259	-0.0253	0.4856	0.7450038	Both
CN	norb_Cluster171	0.0069	-0.0019	0.0251	-0.0263	0.4931	0.7479042	Both
CN	norb_Cluster172	0.014	-0.0052	0.0764	0.0278	0.2251	0.5214893	Both
CN	norb_Cluster175	0.0458	-0.0174	0.0883	0.0403	0.1909	0.5097152	Both
CN	norb_Cluster176	0.0298	-0.0115	0.0923	0.0445	0.1807	0.5055226	Both
CN	norb_Cluster177	0.0282	-0.0076	0.045	-0.0052	0.3557	0.649714	Both
CN	norb_Cluster179	0.0022	-0.0008	0.0348	-0.016	0.418	0.7107665	Both
CN	norb_Cluster18	0.0037	-0.0025	0.2531	0.2137	0.0201	0.1405549	Both
CN	norb_Cluster180	0.0109	-0.0033	0.1013	0.054	0.1596	0.4733129	Both
CN	norb_Cluster181	0.0008	0.0008	0.0836	0.0354	0.2036	0.5201002	Both
CN	norb_Cluster182	0.0013	-0.0007	0.0586	0.009	0.2906	0.5864614	Both
CN	norb_Cluster184	0.0146	-0.0051	0.098	0.0505	0.1671	0.488012	Both
CN	norb_Cluster186	0.0011	-0.0001	0.0028	-0.0496	0.8183	0.8858369	Both
CN	norb_Cluster19	0.0049	-0.002	0.1343	0.0887	0.1023	0.3635433	Both
CN	norb_Cluster192	0.0173	-0.006	0.0796	0.0311	0.2154	0.520961	Both
CN	norb_Cluster193	0.0021	1.00E-04	0.0005	-0.0521	0.921	0.954978	Both
CN	norb_Cluster197	0.0303	-0.0059	0.0057	-0.0466	0.7448	0.841796	Both
CN	norb_Cluster2	0.0031	0.0005	0.0076	-0.0446	0.7069	0.838774	Both
CN	norb_Cluster20	0.0587	-0.0199	0.1734	0.1299	0.0605	0.2808502	Both
CN	norb_Cluster208	0.0011	-0.0003	0.0112	-0.0409	0.6486	0.8096861	Both
CN	norb_Cluster21	0.0481	-0.021	0.2139	0.1726	0.0348	0.1934677	Both
CN	norb_Cluster211	0.0104	-0.0021	0.008	-0.0442	0.6991	0.8381711	Both
CN	norb_Cluster212	0.0264	0.0062	0.0166	-0.0352	0.578	0.7907503	Both
CN	norb_Cluster214	0.0119	-0.0026	0.0144	-0.0375	0.6044	0.7907503	Both
CN	norb_Cluster217	0.0149	-0.002	0.0064	-0.0459	0.7307	0.838774	Both
CN	norb_Cluster219	0.0083	-0.002	0.0144	-0.0375	0.6045	0.7907503	Both
CN	norb_Cluster22	0.0025	-0.0016	0.0969	0.0494	0.1696	0.4928864	Both
CN	norb_Cluster220	0.0177	-0.0056	0.021	-0.0305	0.5304	0.7727341	Both
CN	norb_Cluster221	0.0177	-0.0045	0.0118	-0.0402	0.6394	0.8095113	Both
CN	norb_Cluster222	0.0074	-0.002	0.0099	-0.0422	0.6682	0.8196489	Both
CN	norb_Cluster224	0.0194	-0.0034	0.0096	-0.0425	0.6724	0.8222076	Both
CN	norb_Cluster225	0.0097	0.0021	0.0165	-0.0352	0.5787	0.7907503	Both
CN	norb_Cluster226	0.0043	-0.0006	0.0069	-0.0454	0.721	0.838774	Both
CN	norb_Cluster228	0.0031	0.0032	0.0782	0.0296	0.2197	0.5214893	Both

CN	norb_Cluster229	0.0034	-0.0007	0.0162	-0.0356	0.5828	0.7907503	Both
CN	norb_Cluster230	0.0014	-0.0008	0.0692	0.0202	0.2493	0.5509434	Both
CN	norb_Cluster231	0.0044	-0.0017	0.0396	-0.011	0.3874	0.6779851	Both
CN	norb_Cluster232	0.0031	-0.0017	0.0837	0.0355	0.2033	0.5201002	Both
CN	norb_Cluster234	0.0121	-0.0057	0.0895	0.0416	0.1876	0.5055226	Both
CN	norb_Cluster235	0.0009	-0.0003	0.0311	-0.0198	0.4441	0.722729	Both
CN	norb_Cluster238	0.0099	-0.0031	0.0306	-0.0204	0.4483	0.722729	Both
CN	norb_Cluster239	0.0081	-0.0023	0.0213	-0.0303	0.5283	0.7727341	Both
CN	norb_Cluster24	0.2637	-0.1059	0.2438	0.204	0.0229	0.1487402	Both
CN	norb_Cluster244	0.0011	-0.0006	0.0646	0.0154	0.2662	0.5636078	Both
CN	norb_Cluster247	0.0048	-0.0018	0.1127	0.066	0.1368	0.4411184	Both
CN	norb_Cluster25	0.1114	-0.0387	0.1489	0.1041	0.084	0.3395619	Both
CN	norb_Cluster250	0.0115	-0.0025	0.033	-0.0179	0.4309	0.7197813	Both
CN	norb_Cluster251	0.0052	-0.0009	0.015	-0.0368	0.5966	0.7907503	Both
CN	norb_Cluster26	0.0931	-0.0471	0.2633	0.2246	0.0174	0.1296235	Both
CN	norb_Cluster28	0.0302	-0.0026	0.0077	-0.0446	0.706	0.838774	Both
CN	norb_Cluster29	0.222	-0.0478	0.1763	0.133	0.0581	0.2755268	Both
CN	norb_Cluster31	0.0531	-0.0248	0.2571	0.218	0.019	0.1383048	Both
CN	norb_Cluster32	0.1193	-0.0543	0.1881	0.1454	0.0495	0.2539091	Both
CN	norb_Cluster33	0.4946	0.0788	0.0951	0.0475	0.1738	0.4986086	Both
CN	norb_Cluster35	0.0628	-0.0248	0.1918	0.1493	0.047	0.2434643	Both
CN	norb_Cluster36	1.1345	-0.2476	0.0923	0.0445	0.1807	0.5055226	Both
CN	norb_Cluster37	0.7473	-0.0963	0.05	0.00E+00	0.33	0.626032	Both
CN	norb_Cluster38	0.0116	-0.003	0.0892	0.0413	0.1885	0.5055226	Both
CN	norb_Cluster39	0.0426	-0.0125	0.1016	0.0543	0.1591	0.4733129	Both
CN	norb_Cluster40	0.186	-0.0682	0.181	0.1379	0.0545	0.2667773	Both
CN	norb_Cluster42	0.2864	-0.0309	0.0246	-0.0268	0.4974	0.7479042	Both
CN	norb_Cluster44	0.0182	-0.0083	0.1321	0.0864	0.1054	0.3700586	Both
CN	norb_Cluster46	0.2556	0.0165	0.006	-0.0463	0.7385	0.8394725	Both
CN	norb_Cluster47	0.555	-0.1881	0.1862	0.1433	0.0508	0.2575353	Both
CN	norb_Cluster5	0.01	-0.0037	0.1822	0.1391	0.0537	0.2660837	Both
CN	norb_Cluster51	0.0092	-0.0027	0.0323	-0.0187	0.436	0.720499	Both
CN	norb_Cluster52	0.2391	-0.0908	0.1858	0.143	0.0511	0.2575353	Both
CN	norb_Cluster56	0.014	-0.0054	0.1478	0.103	0.0853	0.3399182	Both
CN	norb_Cluster57	0.0034	-0.0005	0.013	-0.0389	0.6221	0.7989048	Both
CN	norb_Cluster59	0.0236	-0.0097	0.0837	0.0355	0.2033	0.5201002	Both
CN	norb_Cluster6	0.6862	-0.1709	0.1273	0.0814	0.1123	0.385332	Both
CN	norb_Cluster60	0.3199	-0.061	0.0288	-0.0223	0.4619	0.7321274	Both
CN	norb_Cluster61	0.482	-0.083	0.0397	-0.0108	0.3864	0.6779851	Both
CN	norb_Cluster62	0.0024	0.00E+00	0.0001	-0.0525	0.9646	0.9750586	Both
CN	norb_Cluster64	0.0326	-0.0112	0.1807	0.1376	0.0547	0.2667773	Both
CN	norb_Cluster66	0.0012	-0.0003	0.0161	-0.0357	0.5838	0.7907503	Both
CN	norb_Cluster67	0.0218	-0.0072	0.107	0.06	0.1477	0.4520862	Both
CN	norb_Cluster68	0.0034	0.0003	0.005	-0.0473	0.7596	0.8462205	Both
CN	norb_Cluster69	0.0028	-0.0015	0.105	0.0579	0.1518	0.4593601	Both
CN	norb_Cluster7	0.0709	-0.015	0.0641	0.0149	0.2681	0.5648457	Both
CN	norb_Cluster70	0.1051	-0.0316	0.1411	0.0959	0.0933	0.3505828	Both
CN	norb_Cluster71	0.1295	-0.0286	0.0928	0.045	0.1795	0.5055226	Both
CN	norb_Cluster74	0.0327	-0.0102	0.1506	0.1059	0.0822	0.3366388	Both
CN	norb_Cluster75	0.1291	-0.04	0.228	0.1874	0.0286	0.1730459	Both
CN	norb_Cluster76	0.1105	-0.0177	0.0152	-0.0366	0.594	0.7907503	Both
CN	norb_Cluster77	0.0475	-0.0048	0.0269	-0.0243	0.4777	0.7378083	Both
CN	norb_Cluster8	0.018	-0.0029	0.0243	-0.027	0.4994	0.7479042	Both
CN	norb_Cluster80	0.0015	-0.0005	0.0201	-0.0315	0.5402	0.7751303	Both
CN	norb_Cluster81	0.0012	0.0002	0.0075	-0.0448	0.7094	0.838774	Both

CN	norb_Cluster83	0.0078	-0.0034	0.0438	-0.0066	0.3627	0.6544431	Both
CN	norb_Cluster84	0.0082	-0.0015	0.0272	-0.024	0.4751	0.7376224	Both
CN	norb_Cluster85	0.0023	-0.0004	0.0106	-0.0414	0.6565	0.8120358	Both
CN	norb_Cluster86	0.031	-0.0071	0.015	-0.0368	0.5968	0.7907503	Both
CN	norb_Cluster88	0.0016	-0.0002	0.0034	-0.049	0.8009	0.8766925	Both
CN	norb_Cluster90	0.0009	-1.00E-04	0.0006	-0.052	0.9132	0.9502162	Both
CN	norb_Cluster91	0.6481	-0.1528	0.0662	0.0171	0.2601	0.5600036	Both
CN	norb_Cluster92	0.0015	-0.0005	0.0145	-0.0374	0.6033	0.7907503	Both
CN	norb_Cluster93	0.0022	-0.0007	0.0229	-0.0285	0.5124	0.7595595	Both
CN	norb_Cluster94	0.0017	-0.0004	0.0185	-0.0331	0.5564	0.783469	Both
CN	norb_Cluster96	0.0007	0.0002	0.0082	-0.0439	0.6954	0.8381711	Both
CN	norb_Cluster97	0.0119	-0.0045	0.08	0.0316	0.2142	0.520961	Both
CN	norb_Cluster98	0.0132	-0.0035	0.0325	-0.0184	0.4342	0.7197813	Both
CN	nosz_Cluster0	0.0045	-0.0005	0.008	-0.0442	0.6994	0.8381711	Both
CN	nosz_Cluster10	0.0136	0.0052	0.0896	0.0416	0.1875	0.5055226	Both
CN	nosz_Cluster102	0.0044	-0.002	0.1987	0.1565	0.0429	0.2277805	Both
CN	nosz_Cluster103	0.0455	-0.0094	0.0769	0.0283	0.2236	0.5214893	Both
CN	nosz_Cluster105	0.0016	-0.0003	0.008	-0.0442	0.6993	0.8381711	Both
CN	nosz_Cluster106	0.0073	-0.0028	0.095	0.0473	0.1741	0.4986086	Both
CN	nosz_Cluster107	0.0002	-1.00E-04	0.0064	-0.0459	0.73	0.838774	Both
CN	nosz_Cluster108	0.004	0.001	0.0301	-0.0209	0.452	0.7239842	Both
CN	nosz_Cluster109	0.0375	-0.0108	0.2216	0.1806	0.0313	0.1808651	Both
CN	nosz_Cluster11	0.0129	0.0045	0.0325	-0.0184	0.4343	0.7197813	Both
CN	nosz_Cluster110	0.0003	0.0003	0.0507	0.0007	0.3265	0.6234669	Both
CN	nosz_Cluster111	0.0061	-0.0017	0.0556	0.0059	0.3036	0.596999	Both
CN	nosz_Cluster116	0.0067	0.0008	0.0099	-0.0422	0.668	0.8196489	Both
CN	nosz_Cluster118	0.0045	-0.0013	0.0273	-0.0238	0.4738	0.7376224	Both
CN	nosz_Cluster119	0.0013	-0.0006	0.0465	-0.0037	0.3477	0.6411583	Both
CN	nosz_Cluster120	0.0071	-0.0009	0.0135	-0.0385	0.6164	0.7989048	Both
CN	nosz_Cluster123	0.0097	-0.004	0.0873	0.0392	0.1936	0.5144305	Both
CN	nosz_Cluster128	0.0047	0.0003	0.0016	-0.0509	0.8634	0.9150206	Both
CN	nosz_Cluster129	0.0494	0.001	0.0019	-0.0507	0.8527	0.9097061	Both
CN	nosz_Cluster13	0.0115	-0.0036	0.0725	0.0237	0.2379	0.5331685	Both
CN	nosz_Cluster130	0.089	-0.0221	0.013	-0.0389	0.6226	0.7989048	Both
CN	nosz_Cluster132	0.0037	-0.0011	0.08	0.0316	0.2141	0.520961	Both
CN	nosz_Cluster14	0.024	0.0162	0.074	0.0253	0.2329	0.5263988	Both
CN	nosz_Cluster16	0.0076	0.0017	0.0192	-0.0324	0.5487	0.7785497	Both
CN	nosz_Cluster17	0.0364	-0.0075	0.0577	0.0081	0.2943	0.5864614	Both
CN	nosz_Cluster18	0.0161	0.0003	0.0002	-0.0524	0.9505	0.9750586	Both
CN	nosz_Cluster20	0.0091	-0.0023	0.0287	-0.0224	0.4629	0.7321274	Both
CN	nosz_Cluster21	0.0083	-0.003	0.051	0.0011	0.3248	0.6234669	Both
CN	nosz_Cluster22	0.0267	-0.0033	0.0064	-0.0459	0.7305	0.838774	Both
CN	nosz_Cluster23	0.0141	-0.0039	0.0394	-0.0112	0.3884	0.6779851	Both
CN	nosz_Cluster24	0.0013	-0.0005	0.0298	-0.0212	0.4542	0.7242289	Both
CN	nosz_Cluster25	0.0029	0.0008	0.0205	-0.0311	0.5359	0.7730399	Both
CN	nosz_Cluster26	0.0054	-0.0005	0.0038	-0.0486	0.7897	0.8708625	Both
CN	nosz_Cluster27	0.0053	-0.0019	0.075	0.0263	0.2298	0.5214893	Both
CN	nosz_Cluster28	0.0303	-0.0093	0.0911	0.0433	0.1835	0.5055226	Both
CN	nosz_Cluster30	0.0276	-0.004	0.0195	-0.0321	0.5464	0.7785497	Both
CN	nosz_Cluster32	0.0137	-0.0012	0.006	-0.0463	0.7382	0.8394725	Both
CN	nosz_Cluster33	0.018	0.0029	0.0117	-0.0403	0.6412	0.8096861	Both
CN	nosz_Cluster34	0.0377	0.0032	0.0037	-0.0488	0.7938	0.8734719	Both
CN	nosz_Cluster35	0.0075	-0.0017	0.0311	-0.0199	0.4447	0.722729	Both
CN	nosz_Cluster36	0.0179	-0.002	0.0093	-0.0428	0.677	0.8235314	Both
CN	nosz_Cluster37	0.006	-0.0015	0.0325	-0.0184	0.4343	0.7197813	Both

CN	nosz_Cluster38	0.0128	0.0014	0.0061	-0.0462	0.7368	0.8394725	Both
CN	nosz_Cluster39	0.0537	-0.0099	0.035	-0.0158	0.4166	0.7107665	Both
CN	nosz_Cluster40	0.0218	-0.0015	0.0033	-0.0492	0.8048	0.8793568	Both
CN	nosz_Cluster41	0.0203	-0.0051	0.0424	-0.008	0.3706	0.6613689	Both
CN	nosz_Cluster42	0.0279	0.0011	0.0011	-0.0515	0.8872	0.9330765	Both
CN	nosz_Cluster43	0.0007	-0.0004	0.0259	-0.0254	0.4861	0.7450038	Both
CN	nosz_Cluster44	0.0057	-0.0031	0.1628	0.1187	0.0697	0.3131529	Both
CN	nosz_Cluster45	0.0045	-0.0004	0.005	-0.0474	0.7608	0.8462205	Both
CN	nosz_Cluster46	0.007	-0.0008	0.0119	-0.0401	0.6372	0.8095113	Both
CN	nosz_Cluster47	0.0008	-0.0004	0.0442	-0.0061	0.3601	0.6543071	Both
CN	nosz_Cluster48	0.0023	-0.0006	0.0154	-0.0365	0.5925	0.7907503	Both
CN	nosz_Cluster49	0.0014	0.0009	0.1033	0.0561	0.1554	0.4678123	Both
CN	nosz_Cluster5	0.0045	0.0016	0.0308	-0.0202	0.4468	0.722729	Both
CN	nosz_Cluster51	0.0021	-0.0003	0.0071	-0.0451	0.7161	0.838774	Both
CN	nosz_Cluster53	0.0009	-0.0003	0.016	-0.0358	0.5849	0.7907503	Both
CN	nosz_Cluster54	0.0026	-0.0009	0.0304	-0.0206	0.4497	0.7229023	Both
CN	nosz_Cluster57	0.1757	-0.0629	0.1227	0.0765	0.1195	0.3946363	Both
CN	nosz_Cluster58	0.0043	-0.0019	0.0755	0.0269	0.228	0.5214893	Both
CN	nosz_Cluster59	0.0009	0.00E+00	0.00E+00	-0.0526	0.9771	0.9787223	Both
CN	nosz_Cluster6	0.0032	0.0005	0.0072	-0.0451	0.7147	0.838774	Both
CN	nosz_Cluster60	0.0079	-0.0008	0.0054	-0.047	0.752	0.8435029	Both
CN	nosz_Cluster61	0.0178	-0.005	0.1759	0.1326	0.0584	0.2755268	Both
CN	nosz_Cluster62	0.0413	-0.0105	0.0613	0.0119	0.2792	0.5720377	Both
CN	nosz_Cluster64	0.0016	-0.0007	0.0207	-0.0309	0.5339	0.7730399	Both
CN	nosz_Cluster65	0.0065	-0.0008	0.008	-0.0442	0.7004	0.8381711	Both
CN	nosz_Cluster66	0.0324	-0.0162	0.1572	0.1128	0.0752	0.3200022	Both
CN	nosz_Cluster67	0.0521	-0.0172	0.0756	0.0269	0.2277	0.5214893	Both
CN	nosz_Cluster69	0.0061	-0.0014	0.03	-0.0211	0.4528	0.7239842	Both
CN	nosz_Cluster70	0.0479	-0.0144	0.0239	-0.0274	0.503	0.7513485	Both
CN	nosz_Cluster71	0.0083	-0.0004	0.0009	-0.0517	0.8964	0.9361018	Both
CN	nosz_Cluster72	0.0506	-0.018	0.1002	0.0528	0.1622	0.4785048	Both
CN	nosz_Cluster73	0.003	-0.0004	0.007	-0.0453	0.7187	0.838774	Both
CN	nosz_Cluster74	0.0017	-0.0005	0.027	-0.0242	0.4764	0.7377501	Both
CN	nosz_Cluster75	0.0967	-0.0311	0.0156	-0.0362	0.5898	0.7907503	Both
CN	nosz_Cluster77	0.0002	-0.0002	0.075	0.0263	0.2297	0.5214893	Both
CN	nosz_Cluster78	0.0044	-0.0014	0.0675	0.0184	0.2553	0.5575579	Both
CN	nosz_Cluster79	0.0333	-0.0074	0.0623	0.0129	0.2752	0.5663201	Both
CN	nosz_Cluster8	0.0099	-0.0003	0.0013	-0.0513	0.878	0.9266417	Both
CN	nosz_Cluster80	0.0045	0.0016	0.0359	-0.0149	0.4108	0.7052288	Both
CN	nosz_Cluster81	0.0079	-0.0014	0.0093	-0.0428	0.677	0.8235314	Both
CN	nosz_Cluster82	0.0016	-0.0008	0.0189	-0.0328	0.5528	0.780305	Both
CN	nosz_Cluster83	0.0015	-1.00E-04	0.0006	-0.052	0.9182	0.9537447	Both
CN	nosz_Cluster85	0.0022	-0.0005	0.0133	-0.0386	0.6182	0.7989048	Both
CN	nosz_Cluster86	0.0027	-0.0012	0.0579	0.0083	0.2933	0.5864614	Both
CN	nosz_Cluster87	0.003	0.0003	0.005	-0.0474	0.7616	0.8462205	Both
CN	nosz_Cluster88	0.0218	-0.0045	0.0914	0.0436	0.1828	0.5055226	Both
CN	nosz_Cluster89	0.0466	-0.0196	0.1373	0.0919	0.0983	0.3557214	Both
CN	nosz_Cluster9	0.003	-0.0003	0.0024	-0.0501	0.8324	0.8977941	Both
CN	nosz_Cluster90	0.0116	-0.0038	0.1559	0.1115	0.0765	0.3201541	Both
CN	nosz_Cluster91	0.0189	-0.0003	0.0002	-0.0525	0.9571	0.9750586	Both
CN	nosz_Cluster93	0.0393	-0.0138	0.1264	0.0804	0.1137	0.3855765	Both
CN	nosz_Cluster95	0.007	-0.0012	0.0211	-0.0304	0.5298	0.7727341	Both
CN	nosz_Cluster96	0.0066	0.0018	0.0845	0.0363	0.2011	0.5201002	Both
CN	nosz_Cluster97	0.0019	-0.001	0.0761	0.0274	0.2263	0.5214893	Both
CN	nosz_Cluster98	0.0025	-0.0012	0.0179	-0.0338	0.5629	0.7859822	Both

CN	nosz_Cluster99	0.0055	0.0009	0.0233	-0.0281	0.5086	0.7577103	Both
CN	nrfa_Cluster10	0.0804	-0.0161	0.0256	-0.0257	0.4884	0.7464685	Both
CN	nrfa_Cluster100	0.0963	-0.0324	0.0524	0.0025	0.3181	0.6174487	Both
CN	nrfa_Cluster104	0.1143	-0.0471	0.1766	0.1333	0.0579	0.2755268	Both
CN	nrfa_Cluster105	0.0062	0.0008	0.0055	-0.0468	0.7495	0.8422858	Both
CN	nrfa_Cluster109	0.2294	-0.0645	0.0993	0.0519	0.1641	0.4816792	Both
CN	nrfa_Cluster116	0.2592	-0.1107	0.3496	0.3154	0.0048	0.0536271	Both
CN	nrfa_Cluster118	0.173	-0.0631	0.0503	0.0003	0.3285	0.6252967	Both
CN	nrfa_Cluster125	0.0809	-0.0293	0.0442	-0.0061	0.3604	0.6543071	Both
CN	nrfa_Cluster154	0.0658	-0.0207	0.0497	-0.0003	0.3312	0.6262302	Both
CN	nrfa_Cluster164	0.0045	0.001	0.0179	-0.0338	0.5635	0.7859822	Both
CN	nrfa_Cluster172	0.0026	-0.0008	0.0223	-0.0292	0.5184	0.7645893	Both
CN	nrfa_Cluster175	0.0044	-0.0004	0.0025	-0.05	0.8296	0.8964937	Both
CN	nrfa_Cluster20	0.035	-0.0107	0.0573	0.0077	0.296	0.5864614	Both
CN	nrfa_Cluster26	0.1078	-0.0381	0.0522	0.0023	0.3194	0.6178519	Both
CN	nrfa_Cluster29	0.0048	-0.0014	0.0294	-0.0216	0.457	0.7268135	Both
CN	nrfa_Cluster34	0.0487	-0.018	0.0818	0.0335	0.2087	0.520961	Both
CN	nrfa_Cluster38	0.0031	-0.0005	0.0069	-0.0453	0.7194	0.838774	Both
CN	nrfa_Cluster61	0.0036	-0.0017	0.078	0.0294	0.2203	0.5214893	Both
CN	nrfa_Cluster62	0.0452	-0.0167	0.0477	-0.0024	0.3415	0.637597	Both
CN	nrfa_Cluster66	0.1082	-0.0339	0.1458	0.1008	0.0877	0.3403133	Both
CN	nrfa_Cluster69	0.003	1.00E-04	0.0001	-0.0525	0.9629	0.9750586	Both
CN	nrfa_Cluster71	0.027	-0.0028	0.005	-0.0474	0.7603	0.8462205	Both
CN	nrfa_Cluster72	0.0732	-0.0258	0.0681	0.019	0.2534	0.5575579	Both
CN	nrfa_Cluster73	0.0038	1.00E-04	0.0001	-0.0525	0.9655	0.9750586	Both
CN	nrfa_Cluster74	0.1331	-0.044	0.063	0.0137	0.2725	0.5663201	Both
CN	nrfa_Cluster76	0.0755	-0.0149	0.0192	-0.0324	0.5489	0.7785497	Both
CN	nrfa_Cluster79	0.0229	0.016	0.0645	0.0153	0.2665	0.5636078	Both
CN	nrfa_Cluster87	0.017	-0.001	0.001	-0.0516	0.891	0.9333792	Both
CN	nrfa_Cluster89	0.0018	-0.0003	0.0071	-0.0451	0.7163	0.838774	Both
CN	nrfa_Cluster92	0.0043	0.0009	0.0164	-0.0354	0.5806	0.7907503	Both
CN	nrfa_Cluster93	0.0428	-0.01	0.0158	-0.036	0.587	0.7907503	Both
CN	nrfa_Cluster94	0.0002	-0.0002	0.0274	-0.0238	0.4737	0.7376224	Both
CN	nrfa_Cluster97	0.0168	-0.0076	0.1461	0.1012	0.0873	0.3403133	Both
CN	nrfa_Cluster99	0.014	-0.0054	0.0892	0.0413	0.1885	0.5055226	Both
Latitude	amoA_A_Cluster26	0.004	-0.0056	0.5457	0.5218	0.0001	0.0034265	Both
Latitude	amoA_A_Cluster45	0.0051	-0.0051	0.3484	0.3141	0.0049	0.0500989	Both
Latitude	amoA_B_Cluster0	0.1099	0.0106	0.0441	-0.0062	0.361	0.6573517	Both
Latitude	amoA_B_Cluster1	0.0172	-0.0047	0.0748	0.0261	0.2303	0.5456953	Both
Latitude	amoA_B_Cluster10	0.0422	-0.0206	0.4473	0.4182	0.0009	0.0154592	Both
Latitude	amoA_B_Cluster11	0.0377	-0.0058	0.0327	-0.0182	0.4325	0.7085971	Both
Latitude	amoA_B_Cluster12	0.0807	-0.0029	0.0046	-0.0478	0.7709	0.8988591	Both
Latitude	amoA_B_Cluster13	0.5271	-0.0301	0.0055	-0.0468	0.7487	0.8864514	Both
Latitude	amoA_B_Cluster14	0.0275	0.00E+00	0.00E+00	-0.0526	0.9982	0.9981711	Both
Latitude	amoA_B_Cluster15	0.0318	-0.0107	0.1485	0.1037	0.0845	0.3495114	Both
Latitude	amoA_B_Cluster2	0.1721	-0.0166	0.0151	-0.0367	0.5952	0.8149128	Both
Latitude	amoA_B_Cluster20	0.0058	-0.0017	0.0354	-0.0154	0.4144	0.6886997	Both
Latitude	amoA_B_Cluster23	0.0507	-0.0096	0.0757	0.027	0.2275	0.5456953	Both
Latitude	amoA_B_Cluster3	0.0182	-0.0061	0.0934	0.0457	0.1778	0.4850028	Both
Latitude	amoA_B_Cluster4	0.0125	-0.0055	0.1381	0.0928	0.0971	0.3770195	Both
Latitude	amoA_B_Cluster5	0.0928	0.005	0.0109	-0.0412	0.653	0.8444623	Both
Latitude	amoA_B_Cluster6	0.0233	-0.0039	0.0521	0.0023	0.3194	0.6403108	Both
Latitude	amoA_B_Cluster7	0.0179	-0.0056	0.0818	0.0335	0.2088	0.5313801	Both
Latitude	amoA_B_Cluster8	0.0207	-0.0102	0.195	0.1527	0.045	0.2514283	Both
Latitude	amoA_B_Cluster9	0.0549	-0.0085	0.0271	-0.0241	0.4757	0.7443968	Both

Latitude	nifh_Cluster0	0.0332	0.0042	0.0709	0.022	0.2433	0.5651553	Both
Latitude	nifh_Cluster10	0.005	0.0026	0.0643	0.015	0.2675	0.5788647	Both
Latitude	nifh_Cluster100	0.0019	0.0005	0.021	-0.0305	0.5305	0.7919993	Both
Latitude	nifh_Cluster103	0.0383	-0.0158	0.37	0.3368	0.0034	0.0397691	Both
Latitude	nifh_Cluster1034	0.0262	0.0111	0.1329	0.0873	0.1042	0.3872157	Both
Latitude	nifh_Cluster105	0.013	0.0046	0.0872	0.0392	0.1937	0.5146485	Both
Latitude	nifh_Cluster108	0.0083	-0.0034	0.1517	0.107	0.081	0.3495114	Both
Latitude	nifh_Cluster1099	0.066	0.0031	0.0022	-0.0503	0.8385	0.9291018	Both
Latitude	nifh_Cluster11	0.0021	0.0003	0.0113	-0.0407	0.6462	0.8416253	Both
Latitude	nifh_Cluster1112	0.0029	-0.0007	0.0208	-0.0307	0.5324	0.7919993	Both
Latitude	nifh_Cluster112	0.0112	0.0031	0.0673	0.0182	0.256	0.5765352	Both
Latitude	nifh_Cluster113	0.0327	0.0002	1.00E-04	-0.0525	0.9671	0.9923584	Both
Latitude	nifh_Cluster114	0.0332	0.0047	0.1173	0.0709	0.1285	0.4266897	Both
Latitude	nifh_Cluster1141	0.0251	0.0093	0.1562	0.1118	0.0762	0.3457242	Both
Latitude	nifh_Cluster115	0.0095	-0.001	0.0171	-0.0347	0.5726	0.804377	Both
Latitude	nifh_Cluster116	0.0775	-0.0008	0.0003	-0.0524	0.9443	0.9853752	Both
Latitude	nifh_Cluster1163	0.0044	0.00E+00	0.00E+00	-0.0526	0.9919	0.9968904	Both
Latitude	nifh_Cluster1179	0.0031	0.0014	0.0634	0.0141	0.2709	0.5816791	Both
Latitude	nifh_Cluster1182	0.0204	-0.0033	0.1283	0.0824	0.1109	0.3876918	Both
Latitude	nifh_Cluster1183	0.0066	0.0017	0.0797	0.0313	0.2149	0.5350909	Both
Latitude	nifh_Cluster1197	0.0115	-0.004	0.1514	0.1068	0.0813	0.3495114	Both
Latitude	nifh_Cluster1199	0.0046	0.0014	0.0662	0.017	0.2603	0.5788647	Both
Latitude	nifh_Cluster120	0.0149	0.0088	0.0957	0.0481	0.1724	0.4819577	Both
Latitude	nifh_Cluster1203	0.0155	-0.0149	0.57	0.5474	1.00E-04	0.0028124	Both
Latitude	nifh_Cluster1206	0.0043	-0.0022	0.1612	0.117	0.0713	0.3336844	Both
Latitude	nifh_Cluster1207	0.0074	0.0021	0.0491	-0.0009	0.3344	0.6449128	Both
Latitude	nifh_Cluster1209	0.1506	-0.0269	0.2351	0.1948	0.0259	0.1716622	Both
Latitude	nifh_Cluster121	0.0095	-0.0033	0.0365	-0.0142	0.4067	0.6875706	Both
Latitude	nifh_Cluster1211	0.0056	-0.0013	0.0428	-0.0076	0.3682	0.6656997	Both
Latitude	nifh_Cluster1213	0.0026	-0.0012	0.1237	0.0776	0.1179	0.4002661	Both
Latitude	nifh_Cluster122	0.0029	-0.0012	0.0557	0.006	0.3032	0.6211945	Both
Latitude	nifh_Cluster123	0.0095	0.0031	0.0339	-0.017	0.4244	0.699435	Both
Latitude	nifh_Cluster1236	0.0234	-0.0288	0.6098	0.5893	0.00E+00	0.0013366	Both
Latitude	nifh_Cluster1238	0.0028	-0.0005	0.0124	-0.0396	0.6306	0.8360759	Both
Latitude	nifh_Cluster124	0.0091	-0.0008	0.0082	-0.0441	0.6971	0.8659124	Both
Latitude	nifh_Cluster1261	0.002	-0.0008	0.0479	-0.0022	0.3405	0.6469675	Both
Latitude	nifh_Cluster1262	0.0065	0.0032	0.1351	0.0896	0.1011	0.3814347	Both
Latitude	nifh_Cluster1266	0.0094	0.00E+00	0.00E+00	-0.0526	0.988	0.9960476	Both
Latitude	nifh_Cluster1267	0.0562	0.0117	0.0369	-0.0138	0.4044	0.6855816	Both
Latitude	nifh_Cluster127	0.0026	0.0004	0.0067	-0.0455	0.7234	0.8763507	Both
Latitude	nifh_Cluster1278	0.1602	-0.0089	0.0046	-0.0478	0.7706	0.8988591	Both
Latitude	nifh_Cluster128	0.0036	0.0008	0.0259	-0.0253	0.4855	0.7499292	Both
Latitude	nifh_Cluster1289	0.0039	-0.0006	0.0158	-0.036	0.5872	0.8127667	Both
Latitude	nifh_Cluster129	0.0077	0.0025	0.0403	-0.0102	0.3827	0.6700869	Both
Latitude	nifh_Cluster1292	0.0086	-0.0002	0.0005	-0.0521	0.9227	0.9814747	Both
Latitude	nifh_Cluster130	0.0059	0.0009	0.0055	-0.0469	0.7498	0.8864514	Both
Latitude	nifh_Cluster1305	0.0048	-0.0006	0.0125	-0.0395	0.6299	0.8360759	Both
Latitude	nifh_Cluster1306	0.005	-0.0012	0.0388	-0.0118	0.3919	0.6818215	Both
Latitude	nifh_Cluster131	0.0051	0.0004	0.0018	-0.0507	0.8546	0.9354101	Both
Latitude	nifh_Cluster1319	0.0036	0.0014	0.0577	0.0081	0.2944	0.6139403	Both
Latitude	nifh_Cluster132	0.0064	0.0025	0.0711	0.0222	0.2427	0.5651553	Both
Latitude	nifh_Cluster1320	0.0025	-0.0004	0.0054	-0.0469	0.7512	0.8864514	Both
Latitude	nifh_Cluster1323	0.0023	-0.0005	0.03	-0.0211	0.453	0.7223199	Both
Latitude	nifh_Cluster1324	0.03	-0.0066	0.0584	0.0089	0.2911	0.6134012	Both
Latitude	nifh_Cluster1336	0.0153	0.0002	0.0004	-0.0523	0.9354	0.9845766	Both

Latitude	nifh_Cluster1340	0.0363	-0.0163	0.1036	0.0564	0.1547	0.458783	Both
Latitude	nifh_Cluster1345	0.0483	0.008	0.0234	-0.028	0.5082	0.7688692	Both
Latitude	nifh_Cluster1370	0.0021	0.0013	0.1282	0.0823	0.1111	0.3876918	Both
Latitude	nifh_Cluster1375	0.0018	-0.0011	0.1832	0.1402	0.0529	0.276095	Both
Latitude	nifh_Cluster139	0.0041	-0.0006	0.0098	-0.0423	0.6694	0.8525119	Both
Latitude	nifh_Cluster140	0.0043	-0.001	0.0361	-0.0146	0.4094	0.6881154	Both
Latitude	nifh_Cluster141	0.0677	-0.0129	0.0818	0.0334	0.2089	0.5313801	Both
Latitude	nifh_Cluster148	0.0046	-0.0007	0.007	-0.0452	0.7178	0.8749838	Both
Latitude	nifh_Cluster1480	0.001	0.0003	0.0265	-0.0247	0.4807	0.7483749	Both
Latitude	nifh_Cluster152	0.0599	0.0189	0.3153	0.2793	0.0081	0.0721022	Both
Latitude	nifh_Cluster156	0.0659	0.0167	0.1137	0.067	0.135	0.4400313	Both
Latitude	nifh_Cluster1562	0.0543	0.0238	0.1301	0.0843	0.1083	0.3876918	Both
Latitude	nifh_Cluster158	0.0144	-0.0098	0.3328	0.2977	0.0062	0.0570186	Both
Latitude	nifh_Cluster16	0.0019	-0.0004	0.0102	-0.0419	0.6628	0.8471892	Both
Latitude	nifh_Cluster166	0.0172	0.0088	0.0816	0.0332	0.2094	0.5313801	Both
Latitude	nifh_Cluster205	0.0122	0.0007	0.0033	-0.0491	0.8035	0.9222935	Both
Latitude	nifh_Cluster21	0.0021	-0.0006	0.0248	-0.0265	0.4954	0.753886	Both
Latitude	nifh_Cluster222	0.006	0.003	0.0748	0.0261	0.2303	0.5456953	Both
Latitude	nifh_Cluster225	0.0189	0.0005	0.0017	-0.0509	0.8607	0.9388867	Both
Latitude	nifh_Cluster230	0.0042	-0.0015	0.0811	0.0328	0.2108	0.5313801	Both
Latitude	nifh_Cluster231	0.0022	0.0013	0.1148	0.0683	0.1329	0.438022	Both
Latitude	nifh_Cluster236	0.0027	-0.0008	0.0501	1.00E-04	0.3294	0.6449128	Both
Latitude	nifh_Cluster237	0.0058	-0.0024	0.1371	0.0917	0.0985	0.377579	Both
Latitude	nifh_Cluster243	0.0333	-0.0084	0.0654	0.0163	0.263	0.5788647	Both
Latitude	nifh_Cluster2432	0.0436	0.0046	0.0109	-0.0412	0.6529	0.8444623	Both
Latitude	nifh_Cluster2433	0.0032	0.0004	0.0041	-0.0483	0.7816	0.9077134	Both
Latitude	nifh_Cluster246	0.0057	0.0017	0.0408	-0.0097	0.3798	0.6674268	Both
Latitude	nifh_Cluster25	0.0295	0.0088	0.1468	0.1019	0.0865	0.3495114	Both
Latitude	nifh_Cluster256	0.0029	-0.0016	0.1584	0.1141	0.074	0.338344	Both
Latitude	nifh_Cluster264	0.0029	0.0018	0.2265	0.1858	0.0292	0.1913101	Both
Latitude	nifh_Cluster265	0.0736	-0.0019	0.0028	-0.0496	0.8187	0.9291018	Both
Latitude	nifh_Cluster267	0.0109	-0.0005	0.0016	-0.0509	0.8625	0.9388867	Both
Latitude	nifh_Cluster268	0.0111	0.0008	0.0074	-0.0448	0.7099	0.8707527	Both
Latitude	nifh_Cluster269	0.023	-0.0159	0.3735	0.3405	0.0032	0.0383089	Both
Latitude	nifh_Cluster272	0.0041	0.0018	0.0683	0.0193	0.2525	0.5741217	Both
Latitude	nifh_Cluster274	0.0032	0.0021	0.0409	-0.0095	0.379	0.6674268	Both
Latitude	nifh_Cluster275	0.0036	-0.0028	0.3926	0.3606	0.0024	0.0291417	Both
Latitude	nifh_Cluster278	0.0077	0.0038	0.18	0.1368	0.0553	0.2787203	Both
Latitude	nifh_Cluster279	0.006	0.0015	0.0773	0.0287	0.2225	0.5424394	Both
Latitude	nifh_Cluster28	0.002	-0.0007	0.0633	0.014	0.2711	0.5816791	Both
Latitude	nifh_Cluster280	0.0031	-0.0006	0.0086	-0.0436	0.689	0.8631098	Both
Latitude	nifh_Cluster282	0.0052	0.0008	0.0226	-0.0289	0.5157	0.7781863	Both
Latitude	nifh_Cluster287	0.0022	0.00E+00	0.0001	-0.0525	0.9619	0.9922099	Both
Latitude	nifh_Cluster290	0.011	-0.004	0.1289	0.0831	0.1099	0.3876918	Both
Latitude	nifh_Cluster297	0.0029	-0.0023	0.1111	0.0644	0.1397	0.4497192	Both
Latitude	nifh_Cluster299	0.0026	0.0007	0.0207	-0.0309	0.5343	0.7919993	Both
Latitude	nifh_Cluster303	0.002	-0.0008	0.0507	0.0007	0.3267	0.6449128	Both
Latitude	nifh_Cluster304	0.0024	-0.0003	0.0062	-0.0461	0.7334	0.8812557	Both
Latitude	nifh_Cluster313	0.0103	0.001	0.0031	-0.0494	0.8109	0.9270353	Both
Latitude	nifh_Cluster314	0.0022	0.0009	0.0548	0.0051	0.3069	0.6226285	Both
Latitude	nifh_Cluster317	0.0071	-0.0038	0.2175	0.1763	0.0331	0.2054868	Both
Latitude	nifh_Cluster32	0.0033	-0.0009	0.0489	-0.0012	0.3356	0.6449128	Both
Latitude	nifh_Cluster320	12.7419	-0.6703	0.0456	-0.0046	0.3527	0.6526336	Both
Latitude	nifh_Cluster334	0.0063	0.0048	0.1548	0.1103	0.0777	0.3473077	Both
Latitude	nifh_Cluster335	0.0075	0.0058	0.1252	0.0792	0.1155	0.4002661	Both

Latitude	nifh_Cluster338	0.0123	-0.0038	0.1118	0.0651	0.1384	0.4488116	Both
Latitude	nifh_Cluster340	0.007	0.0029	0.0246	-0.0267	0.4971	0.753886	Both
Latitude	nifh_Cluster341	0.0023	-0.0014	0.1238	0.0777	0.1178	0.4002661	Both
Latitude	nifh_Cluster35	0.0078	-0.0016	0.052	0.0021	0.3202	0.6403108	Both
Latitude	nifh_Cluster351	0.0025	0.0015	0.0946	0.047	0.1749	0.4826146	Both
Latitude	nifh_Cluster3574	0.1093	0.0178	0.0859	0.0378	0.1971	0.5169588	Both
Latitude	nifh_Cluster3691	0.0174	-0.0074	0.109	0.0621	0.1439	0.4514451	Both
Latitude	nifh_Cluster37	0.0329	-0.0019	0.0026	-0.0499	0.8273	0.9291018	Both
Latitude	nifh_Cluster374	0.0021	-0.0014	0.1105	0.0636	0.141	0.4497192	Both
Latitude	nifh_Cluster38	0.0009	0.00E+00	0.0002	-0.0524	0.9485	0.9870085	Both
Latitude	nifh_Cluster382	0.0016	-0.0006	0.0157	-0.0361	0.5882	0.8127667	Both
Latitude	nifh_Cluster384	0.0019	-0.0009	0.0778	0.0293	0.2208	0.5405886	Both
Latitude	nifh_Cluster386	0.0035	0.0031	0.2189	0.1778	0.0324	0.2036478	Both
Latitude	nifh_Cluster389	0.0031	0.0021	0.1323	0.0867	0.105	0.3872157	Both
Latitude	nifh_Cluster394	0.002	0.001	0.0428	-0.0076	0.3685	0.6656997	Both
Latitude	nifh_Cluster40	0.0012	0.0001	0.0023	-0.0502	0.835	0.9291018	Both
Latitude	nifh_Cluster43	0.0108	-0.0011	0.0163	-0.0355	0.5814	0.8127667	Both
Latitude	nifh_Cluster434	0.0035	-0.002	0.1064	0.0593	0.149	0.4515099	Both
Latitude	nifh_Cluster440	0.0133	0.0024	0.0193	-0.0323	0.5484	0.8003655	Both
Latitude	nifh_Cluster462	0.0034	0.002	0.1172	0.0707	0.1287	0.4266897	Both
Latitude	nifh_Cluster470	0.0152	-0.0024	0.0326	-0.0183	0.4336	0.7085971	Both
Latitude	nifh_Cluster49	0.0213	-0.0046	0.0184	-0.0333	0.5577	0.8003655	Both
Latitude	nifh_Cluster499	0.0026	0.0005	0.018	-0.0337	0.5624	0.8003655	Both
Latitude	nifh_Cluster5	0.0256	-0.0032	0.0615	0.0121	0.2784	0.5909363	Both
Latitude	nifh_Cluster506	0.0048	-0.0017	0.0785	0.03	0.2188	0.5400879	Both
Latitude	nifh_Cluster51	0.0028	0.0014	0.0702	0.0213	0.2457	0.5678557	Both
Latitude	nifh_Cluster52	0.0336	-0.0003	1.00E-04	-0.0526	0.9728	0.9947237	Both
Latitude	nifh_Cluster525	0.0033	0.002	0.0626	0.0133	0.2738	0.5832723	Both
Latitude	nifh_Cluster531	0.0258	0.0145	0.2715	0.2331	0.0154	0.1125754	Both
Latitude	nifh_Cluster539	0.0048	0.0043	0.1547	0.1103	0.0777	0.3473077	Both
Latitude	nifh_Cluster578	0.0481	-0.0063	0.0289	-0.0222	0.4609	0.7319159	Both
Latitude	nifh_Cluster58	0.3542	0.0713	0.0409	-0.0095	0.379	0.6674268	Both
Latitude	nifh_Cluster60	0.0089	-0.0035	0.0932	0.0455	0.1784	0.4850028	Both
Latitude	nifh_Cluster61	0.0025	0.001	0.0764	0.0278	0.2252	0.5446192	Both
Latitude	nifh_Cluster65	0.009	0.0029	0.0576	0.0081	0.2945	0.6139403	Both
Latitude	nifh_Cluster686	0.0009	0.0006	0.0942	0.0466	0.1759	0.4826146	Both
Latitude	nifh_Cluster69	0.009	-0.0052	0.1769	0.1336	0.0576	0.2879699	Both
Latitude	nifh_Cluster70	0.0111	0.0016	0.006	-0.0463	0.7384	0.8829803	Both
Latitude	nifh_Cluster717	0.003	0.0002	0.0039	-0.0485	0.7882	0.9135793	Both
Latitude	nifh_Cluster725	0.0058	0.0007	0.0102	-0.0419	0.6634	0.8471892	Both
Latitude	nifh_Cluster727	0.0039	-0.0008	0.0192	-0.0324	0.5491	0.8003655	Both
Latitude	nifh_Cluster73	0.0106	0.0022	0.025	-0.0263	0.4933	0.753886	Both
Latitude	nifh_Cluster74	0.0049	0.0011	0.0155	-0.0363	0.5908	0.8143746	Both
Latitude	nifh_Cluster748	0.0081	-0.0006	0.0079	-0.0443	0.7014	0.8675586	Both
Latitude	nifh_Cluster749	0.0071	0.0025	0.1225	0.0763	0.1199	0.4042809	Both
Latitude	nifh_Cluster755	0.0102	-0.0013	0.0105	-0.0415	0.6578	0.8455026	Both
Latitude	nifh_Cluster76	0.0029	-0.0014	0.1084	0.0615	0.145	0.4514451	Both
Latitude	nifh_Cluster79	0.0022	0.0006	0.0381	-0.0125	0.3966	0.6818215	Both
Latitude	nifh_Cluster81	0.0055	0.0025	0.063	0.0137	0.2724	0.5822376	Both
Latitude	nifh_Cluster82	0.0011	-0.0005	0.0468	-0.0034	0.3465	0.6469675	Both
Latitude	nifh_Cluster83	0.0069	-0.0003	0.0011	-0.0515	0.8871	0.9581474	Both
Latitude	nifh_Cluster86	0.0135	-0.0069	0.2801	0.2422	0.0136	0.1086093	Both
Latitude	nifh_Cluster868	0.0099	-0.002	0.0559	0.0062	0.3022	0.6211945	Both
Latitude	nifh_Cluster87	0.0024	0.0002	0.0027	-0.0498	0.8228	0.9291018	Both
Latitude	nifh_Cluster88	0.0039	-0.0022	0.1003	0.053	0.1618	0.4649985	Both

Latitude	nifh_Cluster9	0.0078	-0.0042	0.2082	0.1665	0.0376	0.226536	Both
Latitude	nifh_Cluster92	0.0051	0.0013	0.0179	-0.0338	0.5632	0.8003655	Both
Latitude	nifh_Cluster93	0.0036	0.0007	0.0188	-0.0329	0.5535	0.8003655	Both
Latitude	nifh_Cluster94	0.0047	0.0004	0.0025	-0.05	0.8306	0.9291018	Both
Latitude	nifh_Cluster95	0.0121	0.0067	0.1352	0.0897	0.1011	0.3814347	Both
Latitude	nifh_Cluster96	0.0123	-0.0014	0.0129	-0.0391	0.6244	0.8360759	Both
Latitude	nifh_Cluster97	0.018	0.0079	0.1493	0.1045	0.0836	0.3495114	Both
Latitude	nifh_Cluster98	0.0059	-0.0002	0.0008	-0.0518	0.9027	0.9683753	Both
Latitude	nifh_Cluster99	0.0033	0.0005	0.0091	-0.043	0.6801	0.8553162	Both
Latitude	nirk_Cluster0	5.9009	-0.1556	0.0108	-0.0413	0.6541	0.8444623	Both
Latitude	nirk_Cluster1	0.1708	-0.0161	0.0138	-0.0381	0.6118	0.8297456	Both
Latitude	nirk_Cluster10	0.5713	-0.1239	0.2352	0.1949	0.0259	0.1716622	Both
Latitude	nirk_Cluster101	0.0031	-0.0027	0.2875	0.25	0.0122	0.1014943	Both
Latitude	nirk_Cluster102	0.0426	-0.0066	0.0311	-0.0198	0.4441	0.7119935	Both
Latitude	nirk_Cluster103	0.0754	0.0097	0.0282	-0.0229	0.4665	0.7343219	Both
Latitude	nirk_Cluster104	0.0023	-0.0004	0.0072	-0.0451	0.7151	0.8734589	Both
Latitude	nirk_Cluster105	0.0946	-0.0101	0.0202	-0.0314	0.5388	0.7946675	Both
Latitude	nirk_Cluster106	0.0592	0.0119	0.0408	-0.0097	0.3801	0.6674268	Both
Latitude	nirk_Cluster107	0.0891	-0.0145	0.041	-0.0094	0.3785	0.6674268	Both
Latitude	nirk_Cluster108	0.0151	-0.0013	0.0072	-0.045	0.7139	0.8734589	Both
Latitude	nirk_Cluster109	0.0115	-0.0021	0.0267	-0.0245	0.479	0.7477167	Both
Latitude	nirk_Cluster11	0.1401	-0.0055	0.0247	-0.0267	0.4965	0.753886	Both
Latitude	nirk_Cluster12	0.094	-0.0126	0.076	0.0273	0.2265	0.5455299	Both
Latitude	nirk_Cluster13	0.0408	-0.0019	0.0151	-0.0367	0.5953	0.8149128	Both
Latitude	nirk_Cluster14	0.0946	-0.0035	0.0054	-0.0469	0.7509	0.8864514	Both
Latitude	nirk_Cluster15	0.3358	0.0415	0.0276	-0.0236	0.4717	0.7400987	Both
Latitude	nirk_Cluster16	0.4727	0.0849	0.0535	0.0037	0.3129	0.6300861	Both
Latitude	nirk_Cluster17	0.0998	-0.0156	0.1714	0.1278	0.0621	0.2977565	Both
Latitude	nirk_Cluster18	0.1296	-0.0024	0.0007	-0.0519	0.9122	0.9750126	Both
Latitude	nirk_Cluster19	0.2776	0.0219	0.0679	0.0188	0.254	0.5741217	Both
Latitude	nirk_Cluster2	0.2032	0.0247	0.1283	0.0824	0.1109	0.3876918	Both
Latitude	nirk_Cluster20	0.1738	-0.0198	0.0474	-0.0027	0.343	0.6469675	Both
Latitude	nirk_Cluster21	0.1532	-0.0151	0.0791	0.0306	0.2168	0.5374617	Both
Latitude	nirk_Cluster22	0.1291	0.0085	0.0191	-0.0325	0.5504	0.8003655	Both
Latitude	nirk_Cluster23	0.0673	-0.0044	0.0414	-0.009	0.3762	0.6674268	Both
Latitude	nirk_Cluster24	0.0654	-0.0044	0.0148	-0.037	0.5988	0.8158709	Both
Latitude	nirk_Cluster25	0.4782	-0.1331	0.3435	0.309	0.0052	0.0506542	Both
Latitude	nirk_Cluster26	0.105	0.0001	0.00E+00	-0.0526	0.9876	0.9960476	Both
Latitude	nirk_Cluster27	0.1727	-0.017	0.032	-0.019	0.4381	0.7094062	Both
Latitude	nirk_Cluster28	0.0685	-0.0007	0.0003	-0.0523	0.9365	0.9845766	Both
Latitude	nirk_Cluster29	0.1526	-0.0228	0.1501	0.1054	0.0826	0.3495114	Both
Latitude	nirk_Cluster3	0.3023	0.011	0.0084	-0.0438	0.6922	0.8652604	Both
Latitude	nirk_Cluster30	0.0556	0.007	0.0259	-0.0253	0.4855	0.7499292	Both
Latitude	nirk_Cluster31	0.1501	0.0132	0.0964	0.0488	0.1708	0.4819577	Both
Latitude	nirk_Cluster32	0.1254	-0.0095	0.0076	-0.0446	0.7065	0.8701908	Both
Latitude	nirk_Cluster33	0.0638	-0.0005	1.00E-04	-0.0526	0.9705	0.9940395	Both
Latitude	nirk_Cluster34	0.0416	-0.0047	0.0286	-0.0225	0.4637	0.7334683	Both
Latitude	nirk_Cluster35	0.1007	-0.051	0.3023	0.2656	0.0098	0.0839538	Both
Latitude	nirk_Cluster36	0.0017	-0.0004	0.0114	-0.0406	0.6452	0.8416253	Both
Latitude	nirk_Cluster39	0.0035	-0.0009	0.011	-0.041	0.6506	0.8444623	Both
Latitude	nirk_Cluster4	0.1323	-0.007	0.0113	-0.0407	0.6461	0.8416253	Both
Latitude	nirk_Cluster40	0.0455	-0.0037	0.0188	-0.0328	0.5534	0.8003655	Both
Latitude	nirk_Cluster41	0.1562	-0.0089	0.018	-0.0337	0.5621	0.8003655	Both
Latitude	nirk_Cluster44	0.0669	0.0111	0.0378	-0.0128	0.3984	0.6818215	Both
Latitude	nirk_Cluster46	0.0154	-0.001	0.0032	-0.0492	0.807	0.9245066	Both

Latitude	nirk_Cluster48	0.003	-0.0005	0.014	-0.0379	0.6091	0.8280281	Both
Latitude	nirk_Cluster49	0.0057	-0.0013	0.0211	-0.0304	0.5301	0.7919993	Both
Latitude	nirk_Cluster5	0.1932	-0.0132	0.0359	-0.0149	0.4109	0.6883946	Both
Latitude	nirk_Cluster50	0.0016	0.00E+00	0.00E+00	-0.0526	0.9935	0.9968904	Both
Latitude	nirk_Cluster52	0.0452	-0.0035	0.0132	-0.0388	0.6204	0.8338501	Both
Latitude	nirk_Cluster53	0.0038	-0.0023	0.129	0.0831	0.1098	0.3876918	Both
Latitude	nirk_Cluster54	0.002	-0.0009	0.0653	0.0161	0.2635	0.5788647	Both
Latitude	nirk_Cluster55	0.0023	-0.0016	0.1077	0.0607	0.1464	0.4514451	Both
Latitude	nirk_Cluster56	0.0009	-0.0004	0.032	-0.0189	0.4377	0.7094062	Both
Latitude	nirk_Cluster57	0.0016	-0.0008	0.0497	-0.0003	0.3315	0.6449128	Both
Latitude	nirk_Cluster58	0.0088	-0.0012	0.0135	-0.0384	0.6162	0.8319537	Both
Latitude	nirk_Cluster59	0.0058	0.0003	0.0018	-0.0507	0.8531	0.9354101	Both
Latitude	nirk_Cluster6	0.1558	-0.0004	0.00E+00	-0.0526	0.9826	0.9960476	Both
Latitude	nirk_Cluster60	0.0006	0.0003	0.025	-0.0263	0.4938	0.753886	Both
Latitude	nirk_Cluster62	0.1417	-0.0132	0.0122	-0.0398	0.6339	0.8386317	Both
Latitude	nirk_Cluster63	0.1284	-0.0357	0.1672	0.1234	0.0657	0.3100414	Both
Latitude	nirk_Cluster64	0.0044	0.0003	0.0028	-0.0497	0.8196	0.9291018	Both
Latitude	nirk_Cluster65	0.0464	-0.0135	0.2221	0.1812	0.031	0.1990548	Both
Latitude	nirk_Cluster66	0.0068	-0.0045	0.3058	0.2693	0.0093	0.0808259	Both
Latitude	nirk_Cluster67	0.0174	-0.0045	0.0491	-0.001	0.3345	0.6449128	Both
Latitude	nirk_Cluster68	0.0125	-0.0054	0.1753	0.1318	0.0589	0.2901713	Both
Latitude	nirk_Cluster69	0.0305	-0.0138	0.4253	0.395	0.0014	0.0195246	Both
Latitude	nirk_Cluster7	0.0804	-0.0203	0.194	0.1516	0.0457	0.2517404	Both
Latitude	nirk_Cluster70	0.0721	0.0019	0.0005	-0.0521	0.9233	0.9814747	Both
Latitude	nirk_Cluster71	0.0053	-0.0012	0.0315	-0.0194	0.4412	0.7107835	Both
Latitude	nirk_Cluster72	0.0239	-0.0056	0.1071	0.0601	0.1477	0.4514451	Both
Latitude	nirk_Cluster73	0.1444	-0.0745	0.4427	0.4133	0.001	0.0158974	Both
Latitude	nirk_Cluster74	0.6398	-0.0315	0.0037	-0.0487	0.7936	0.9136867	Both
Latitude	nirk_Cluster75	0.0147	-0.0169	0.7158	0.7009	0.00E+00	0.0007966	Both
Latitude	nirk_Cluster76	0.1055	-0.0228	0.1081	0.0611	0.1457	0.4514451	Both
Latitude	nirk_Cluster77	0.0081	-0.0062	0.1236	0.0775	0.118	0.4002661	Both
Latitude	nirk_Cluster78	0.2407	-0.0449	0.0681	0.0191	0.2531	0.5741217	Both
Latitude	nirk_Cluster79	0.0069	-0.0035	0.0836	0.0353	0.2037	0.5290731	Both
Latitude	nirk_Cluster8	0.0982	-0.0208	0.0961	0.0485	0.1714	0.4819577	Both
Latitude	nirk_Cluster80	0.2665	-0.1446	0.4721	0.4443	0.0006	0.0110301	Both
Latitude	nirk_Cluster82	0.0015	-0.0001	0.0011	-0.0515	0.8883	0.9581474	Both
Latitude	nirk_Cluster83	0.004	-0.001	0.0443	-0.006	0.3598	0.6573517	Both
Latitude	nirk_Cluster84	0.0207	0.0059	0.0608	0.0113	0.2814	0.5949901	Both
Latitude	nirk_Cluster85	0.004	-0.0037	0.4687	0.4407	0.0006	0.0114037	Both
Latitude	nirk_Cluster86	0.0286	-0.0075	0.075	0.0264	0.2295	0.5456953	Both
Latitude	nirk_Cluster87	0.0115	-0.0046	0.1419	0.0967	0.0924	0.3659427	Both
Latitude	nirk_Cluster88	0.0032	-0.0006	0.0247	-0.0266	0.4963	0.753886	Both
Latitude	nirk_Cluster89	0.0065	-0.0011	0.0158	-0.036	0.5874	0.8127667	Both
Latitude	nirk_Cluster9	0.0517	-0.0016	0.0017	-0.0509	0.8611	0.9388867	Both
Latitude	nirk_Cluster90	0.0067	0.0042	0.1808	0.1377	0.0546	0.2787203	Both
Latitude	nirk_Cluster91	0.0374	-0.0038	0.0182	-0.0335	0.5597	0.8003655	Both
Latitude	nirk_Cluster92	0.0072	0.0008	0.0069	-0.0454	0.7211	0.8763507	Both
Latitude	nirk_Cluster93	0.0308	-0.0102	0.1905	0.1479	0.0479	0.2602119	Both
Latitude	nirk_Cluster94	0.0175	-0.004	0.0553	0.0056	0.3046	0.6219067	Both
Latitude	nirk_Cluster95	0.0473	0.0109	0.0687	0.0196	0.2512	0.5741217	Both
Latitude	nirk_Cluster96	0.0472	0.0066	0.0314	-0.0196	0.4421	0.7107835	Both
Latitude	nirk_Cluster97	0.0306	0.0087	0.1349	0.0893	0.1015	0.3814347	Both
Latitude	nirk_Cluster98	0.0181	0.0045	0.0442	-0.0061	0.3604	0.6573517	Both
Latitude	nirk_Cluster99	0.006	-0.0019	0.0566	0.0069	0.2992	0.6173104	Both
Latitude	nirs_Cluster0	0.1409	0.0147	0.0468	-0.0034	0.3463	0.6469675	Both

Latitude	nirs_Cluster1	0.004	0.0011	0.0254	-0.0259	0.4903	0.753886	Both
Latitude	nirs_Cluster14	0.0107	0.0049	0.0469	-0.0033	0.346	0.6469675	Both
Latitude	nirs_Cluster2	0.0139	-0.005	0.048	-0.0021	0.3399	0.6469675	Both
Latitude	nirs_Cluster26	0.0005	0.0004	0.0781	0.0296	0.2199	0.5405401	Both
Latitude	nirs_Cluster28	0.0071	0.0002	0.0002	-0.0525	0.9553	0.9888128	Both
Latitude	nirs_Cluster3	0.1715	-0.0176	0.0915	0.0437	0.1825	0.4917692	Both
Latitude	nirs_Cluster36	0.0013	-0.0007	0.0171	-0.0346	0.5715	0.804377	Both
Latitude	nirs_Cluster37	0.0066	0.0043	0.0495	-0.0006	0.3325	0.6449128	Both
Latitude	nirs_Cluster4	0.0002	0.0002	0.0372	-0.0135	0.4025	0.6843069	Both
Latitude	nirs_Cluster50	0.002	1.00E-04	0.0005	-0.0521	0.9261	0.9827093	Both
Latitude	nirs_Cluster9	0.0269	-0.0422	0.5077	0.4818	0.0003	0.0061084	Both
Latitude	norb_Cluster0	0.0775	0.022	0.1315	0.0857	0.1062	0.3876918	Both
Latitude	norb_Cluster1	1.1042	0.4743	0.2211	0.1801	0.0315	0.1997858	Both
Latitude	norb_Cluster101	0.0026	-0.0042	0.6232	0.6034	0.00E+00	0.0012835	Both
Latitude	norb_Cluster102	0.0038	0.0008	0.0207	-0.0309	0.534	0.7919993	Both
Latitude	norb_Cluster103	0.0018	0.0002	0.0027	-0.0498	0.8231	0.9291018	Both
Latitude	norb_Cluster104	0.0023	0.0014	0.0813	0.033	0.2102	0.5313801	Both
Latitude	norb_Cluster106	0.125	-0.0301	0.0995	0.0521	0.1636	0.466218	Both
Latitude	norb_Cluster107	0.0133	-0.0065	0.1873	0.1445	0.05	0.2659714	Both
Latitude	norb_Cluster108	0.0052	-0.0011	0.0329	-0.018	0.4313	0.7085971	Both
Latitude	norb_Cluster109	0.0227	-0.0108	0.2443	0.2045	0.0228	0.159899	Both
Latitude	norb_Cluster11	0.0126	-0.0099	0.4613	0.4329	0.0007	0.0125647	Both
Latitude	norb_Cluster110	0.0099	-0.0063	0.2714	0.233	0.0155	0.1125754	Both
Latitude	norb_Cluster112	0.0171	-0.008	0.2741	0.2359	0.0149	0.1124726	Both
Latitude	norb_Cluster113	0.0134	-0.0051	0.1029	0.0557	0.1562	0.4592172	Both
Latitude	norb_Cluster114	0.0025	0.0014	0.0495	-0.0005	0.3323	0.6449128	Both
Latitude	norb_Cluster115	0.0137	0.0096	0.0381	-0.0125	0.3962	0.6818215	Both
Latitude	norb_Cluster116	0.0072	0.0035	0.1144	0.0678	0.1337	0.4380816	Both
Latitude	norb_Cluster117	0.0027	-0.0004	0.008	-0.0442	0.7	0.8675586	Both
Latitude	norb_Cluster118	0.0037	-0.0004	0.005	-0.0474	0.7611	0.894516	Both
Latitude	norb_Cluster119	0.019	-0.0162	0.4389	0.4093	0.0011	0.0165733	Both
Latitude	norb_Cluster12	0.0047	0.0016	0.0454	-0.0049	0.354	0.6526336	Both
Latitude	norb_Cluster121	0.0017	-0.0023	0.2727	0.2344	0.0152	0.1125754	Both
Latitude	norb_Cluster122	0.0144	-0.0019	0.0157	-0.0361	0.588	0.8127667	Both
Latitude	norb_Cluster123	0.0053	-0.0021	0.0804	0.032	0.2129	0.5344558	Both
Latitude	norb_Cluster124	0.682	0.2615	0.2234	0.1825	0.0305	0.1977817	Both
Latitude	norb_Cluster125	0.004	-0.0032	0.352	0.3179	0.0046	0.0482593	Both
Latitude	norb_Cluster126	0.0082	-0.0055	0.3002	0.2634	0.0101	0.0853584	Both
Latitude	norb_Cluster127	0.1735	0.0309	0.0414	-0.0091	0.3764	0.6674268	Both
Latitude	norb_Cluster128	0.0115	0.0035	0.1044	0.0572	0.1532	0.4564529	Both
Latitude	norb_Cluster13	0.0045	-0.003	0.139	0.0937	0.096	0.3770195	Both
Latitude	norb_Cluster131	0.0184	-0.0089	0.2424	0.2025	0.0234	0.1623301	Both
Latitude	norb_Cluster132	1.0357	-0.0484	0.0046	-0.0478	0.77	0.8988591	Both
Latitude	norb_Cluster133	0.0018	0.00E+00	1.00E-04	-0.0525	0.9668	0.9923584	Both
Latitude	norb_Cluster135	0.0567	0.0109	0.0484	-0.0017	0.3379	0.6469675	Both
Latitude	norb_Cluster136	0.0213	0.0006	0.0009	-0.0517	0.8972	0.9642399	Both
Latitude	norb_Cluster139	0.0328	-0.0025	0.0082	-0.044	0.6957	0.8659124	Both
Latitude	norb_Cluster14	0.0026	-0.0026	0.3541	0.3201	0.0044	0.0475268	Both
Latitude	norb_Cluster140	0.0437	0.0018	0.0022	-0.0503	0.8388	0.9291018	Both
Latitude	norb_Cluster142	0.0015	-0.0016	0.1948	0.1524	0.0452	0.2514283	Both
Latitude	norb_Cluster144	0.0098	-0.0019	0.0309	-0.0201	0.4459	0.7128868	Both
Latitude	norb_Cluster146	0.0348	0.0093	0.1094	0.0625	0.1431	0.4514451	Both
Latitude	norb_Cluster15	0.0469	0.0167	0.1474	0.1026	0.0857	0.3495114	Both
Latitude	norb_Cluster150	0.0157	0.0015	0.0117	-0.0403	0.6401	0.8416253	Both
Latitude	norb_Cluster155	0.1631	0.0572	0.2057	0.1639	0.0389	0.2301363	Both

Latitude	norb_Cluster16	0.0131	-0.0113	0.4431	0.4138	0.001	0.0158974	Both
Latitude	norb_Cluster164	0.0199	-0.0067	0.0866	0.0385	0.1954	0.5146485	Both
Latitude	norb_Cluster165	0.0076	0.001	0.0075	-0.0447	0.709	0.8707527	Both
Latitude	norb_Cluster166	0.0039	-0.0003	0.0024	-0.0501	0.834	0.9291018	Both
Latitude	norb_Cluster169	0.0238	-0.0091	0.1016	0.0543	0.1591	0.463621	Both
Latitude	norb_Cluster17	0.0028	-0.002	0.2055	0.1637	0.039	0.2301363	Both
Latitude	norb_Cluster170	0.0243	0.0036	0.0132	-0.0387	0.6201	0.8338501	Both
Latitude	norb_Cluster171	0.0069	-0.0076	0.4234	0.393	0.0014	0.0197107	Both
Latitude	norb_Cluster172	0.014	-0.0027	0.0208	-0.0307	0.5328	0.7919993	Both
Latitude	norb_Cluster175	0.0458	-0.011	0.0356	-0.0152	0.413	0.6883946	Both
Latitude	norb_Cluster176	0.0298	-0.0176	0.2152	0.1739	0.0341	0.209754	Both
Latitude	norb_Cluster177	0.0282	-0.0048	0.0176	-0.0341	0.5662	0.8003655	Both
Latitude	norb_Cluster179	0.0022	-0.0001	0.0006	-0.052	0.9172	0.9785672	Both
Latitude	norb_Cluster18	0.0037	-0.0026	0.2817	0.2439	0.0133	0.1075561	Both
Latitude	norb_Cluster180	0.0109	-0.0053	0.2679	0.2294	0.0163	0.116975	Both
Latitude	norb_Cluster181	0.0008	0.0007	0.0667	0.0176	0.2584	0.5788647	Both
Latitude	norb_Cluster182	0.0013	-0.001	0.1297	0.0838	0.1089	0.3876918	Both
Latitude	norb_Cluster184	0.0146	-0.0022	0.0175	-0.0342	0.567	0.8003655	Both
Latitude	norb_Cluster186	0.0011	0.0008	0.0945	0.0469	0.1752	0.4826146	Both
Latitude	norb_Cluster19	0.0049	-0.0022	0.1584	0.1141	0.074	0.338344	Both
Latitude	norb_Cluster192	0.0173	-0.0054	0.0646	0.0153	0.2664	0.5788647	Both
Latitude	norb_Cluster193	0.0021	-0.0005	0.0194	-0.0322	0.547	0.8003655	Both
Latitude	norb_Cluster197	0.0303	0.0152	0.0379	-0.0127	0.3976	0.6818215	Both
Latitude	norb_Cluster2	0.0031	-0.0017	0.1001	0.0527	0.1624	0.4649985	Both
Latitude	norb_Cluster20	0.0587	0.0177	0.1371	0.0916	0.0986	0.377579	Both
Latitude	norb_Cluster208	0.0011	-0.0008	0.0845	0.0363	0.2011	0.5249395	Both
Latitude	norb_Cluster21	0.0481	0.0171	0.1418	0.0967	0.0924	0.3659427	Both
Latitude	norb_Cluster211	0.0104	0.0061	0.0649	0.0156	0.2652	0.5788647	Both
Latitude	norb_Cluster212	0.0264	0.0025	0.0026	-0.0499	0.825	0.9291018	Both
Latitude	norb_Cluster214	0.0119	0.003	0.0192	-0.0324	0.5488	0.8003655	Both
Latitude	norb_Cluster217	0.0149	0.00E+00	0.00E+00	-0.0526	0.9956	0.9973217	Both
Latitude	norb_Cluster219	0.0083	0.0016	0.0091	-0.0431	0.6811	0.8553162	Both
Latitude	norb_Cluster22	0.0025	0.0004	0.0061	-0.0463	0.7375	0.8829803	Both
Latitude	norb_Cluster220	0.0177	0.0062	0.026	-0.0252	0.4846	0.7499292	Both
Latitude	norb_Cluster221	0.0177	0.0031	0.0055	-0.0469	0.7496	0.8864514	Both
Latitude	norb_Cluster222	0.0074	0.0038	0.0356	-0.0151	0.4125	0.6883946	Both
Latitude	norb_Cluster224	0.0194	-0.0005	0.0002	-0.0524	0.9516	0.9884992	Both
Latitude	norb_Cluster225	0.0097	0.0018	0.0126	-0.0394	0.6278	0.8360759	Both
Latitude	norb_Cluster226	0.0043	-0.002	0.0764	0.0278	0.2252	0.5446192	Both
Latitude	norb_Cluster228	0.0031	0.0008	0.0046	-0.0478	0.7708	0.8988591	Both
Latitude	norb_Cluster229	0.0034	-0.0009	0.0282	-0.0229	0.4667	0.7343219	Both
Latitude	norb_Cluster230	0.0014	-0.0016	0.2782	0.2402	0.014	0.1092429	Both
Latitude	norb_Cluster231	0.0044	-0.0002	0.0004	-0.0522	0.9295	0.9844606	Both
Latitude	norb_Cluster232	0.0031	1.00E-04	0.0001	-0.0525	0.965	0.9923584	Both
Latitude	norb_Cluster234	0.0121	-0.0126	0.4365	0.4069	0.0011	0.0168383	Both
Latitude	norb_Cluster235	0.0009	-0.0002	0.0096	-0.0425	0.6729	0.8525119	Both
Latitude	norb_Cluster238	0.0099	-0.0145	0.667	0.6495	0.00E+00	0.0012835	Both
Latitude	norb_Cluster239	0.0081	-0.0118	0.5448	0.5208	0.0001	0.0034265	Both
Latitude	norb_Cluster24	0.2637	0.0811	0.1428	0.0977	0.0912	0.3659253	Both
Latitude	norb_Cluster244	0.0011	-0.0015	0.3981	0.3664	0.0022	0.0277429	Both
Latitude	norb_Cluster247	0.0048	-0.0021	0.1469	0.102	0.0863	0.3495114	Both
Latitude	norb_Cluster25	0.1114	0.0426	0.1802	0.137	0.0551	0.2787203	Both
Latitude	norb_Cluster250	0.0115	-0.0037	0.0722	0.0234	0.2389	0.559363	Both
Latitude	norb_Cluster251	0.0052	-0.0036	0.239	0.199	0.0245	0.1680806	Both
Latitude	norb_Cluster26	0.0931	0.0302	0.1086	0.0617	0.1446	0.4514451	Both

Latitude	norb_Cluster28	0.0302	-0.0024	0.0064	-0.0459	0.7295	0.880151	Both
Latitude	norb_Cluster29	0.222	0.0075	0.0043	-0.0481	0.7776	0.9048669	Both
Latitude	norb_Cluster31	0.0531	0.0226	0.2138	0.1725	0.0348	0.2116903	Both
Latitude	norb_Cluster32	0.1193	0.0565	0.2039	0.162	0.0399	0.2308286	Both
Latitude	norb_Cluster33	0.4946	0.2029	0.6313	0.6119	0.00E+00	0.0012835	Both
Latitude	norb_Cluster35	0.0628	0.0196	0.1205	0.0742	0.1231	0.4126451	Both
Latitude	norb_Cluster36	1.1345	0.5119	0.3943	0.3624	0.0023	0.0289468	Both
Latitude	norb_Cluster37	0.7473	0.2823	0.429	0.3989	0.0013	0.0187624	Both
Latitude	norb_Cluster38	0.0116	0.0022	0.0499	-0.0002	0.3306	0.6449128	Both
Latitude	norb_Cluster39	0.0426	0.0049	0.0158	-0.036	0.5877	0.8127667	Both
Latitude	norb_Cluster40	0.186	0.0462	0.0833	0.035	0.2045	0.5290731	Both
Latitude	norb_Cluster42	0.2864	0.1433	0.5273	0.5024	0.0002	0.0047633	Both
Latitude	norb_Cluster44	0.0182	-0.0134	0.3441	0.3095	0.0052	0.0506542	Both
Latitude	norb_Cluster46	0.2556	0.1231	0.3348	0.2998	0.006	0.0561677	Both
Latitude	norb_Cluster47	0.555	0.1796	0.1696	0.1259	0.0636	0.3026025	Both
Latitude	norb_Cluster5	0.01	0.002	0.0548	0.0051	0.3071	0.6226285	Both
Latitude	norb_Cluster51	0.0092	-0.0078	0.2753	0.2372	0.0146	0.1118559	Both
Latitude	norb_Cluster52	0.2391	0.0919	0.1903	0.1476	0.0481	0.2602119	Both
Latitude	norb_Cluster56	0.014	0.0002	0.0003	-0.0523	0.9438	0.9853752	Both
Latitude	norb_Cluster57	0.0034	0.0004	0.0083	-0.0438	0.6937	0.8653524	Both
Latitude	norb_Cluster59	0.0236	-0.0231	0.475	0.4474	0.0005	0.0107878	Both
Latitude	norb_Cluster6	0.6862	0.2788	0.3389	0.3041	0.0056	0.0535478	Both
Latitude	norb_Cluster60	0.3199	0.211	0.345	0.3105	0.0051	0.0506542	Both
Latitude	norb_Cluster61	0.482	-0.1505	0.1306	0.0848	0.1075	0.3876918	Both
Latitude	norb_Cluster62	0.0024	0.00E+00	0.00E+00	-0.0526	0.9873	0.9960476	Both
Latitude	norb_Cluster64	0.0326	-0.0032	0.015	-0.0368	0.5967	0.8149128	Both
Latitude	norb_Cluster66	0.0012	0.0007	0.0744	0.0256	0.2317	0.5468709	Both
Latitude	norb_Cluster67	0.0218	-0.0086	0.1526	0.108	0.08	0.3495114	Both
Latitude	norb_Cluster68	0.0034	-0.0008	0.0455	-0.0047	0.353	0.6526336	Both
Latitude	norb_Cluster69	0.0028	0.0014	0.0918	0.044	0.1819	0.4917692	Both
Latitude	norb_Cluster7	0.0709	0.023	0.1507	0.106	0.0821	0.3495114	Both
Latitude	norb_Cluster70	0.1051	0.0051	0.0037	-0.0488	0.7944	0.9136867	Both
Latitude	norb_Cluster71	0.1295	-0.021	0.0497	-0.0003	0.3313	0.6449128	Both
Latitude	norb_Cluster74	0.0327	-0.0052	0.0398	-0.0108	0.3861	0.6739819	Both
Latitude	norb_Cluster75	0.1291	-0.0178	0.045	-0.0053	0.3561	0.6545633	Both
Latitude	norb_Cluster76	0.1105	0.0755	0.2779	0.2399	0.0141	0.1092429	Both
Latitude	norb_Cluster77	0.0475	0.0075	0.0647	0.0155	0.2658	0.5788647	Both
Latitude	norb_Cluster8	0.018	0.0008	0.0021	-0.0504	0.8429	0.9295628	Both
Latitude	norb_Cluster80	0.0015	-0.001	0.1009	0.0536	0.1605	0.4642662	Both
Latitude	norb_Cluster81	0.0012	0.00E+00	0.00E+00	-0.0526	0.9838	0.9960476	Both
Latitude	norb_Cluster83	0.0078	-0.0112	0.4763	0.4487	0.0005	0.0107878	Both
Latitude	norb_Cluster84	0.0082	-0.0002	0.0003	-0.0523	0.9379	0.9845766	Both
Latitude	norb_Cluster85	0.0023	0.0008	0.0409	-0.0096	0.3795	0.6674268	Both
Latitude	norb_Cluster86	0.031	0.0114	0.0382	-0.0124	0.3955	0.6818215	Both
Latitude	norb_Cluster88	0.0016	-0.0002	0.0028	-0.0497	0.8201	0.9291018	Both
Latitude	norb_Cluster90	0.0009	-0.0016	0.2352	0.195	0.0259	0.1716622	Both
Latitude	norb_Cluster91	0.6481	-0.1904	0.1028	0.0556	0.1564	0.4592172	Both
Latitude	norb_Cluster92	0.0015	0.0005	0.0117	-0.0403	0.641	0.8416253	Both
Latitude	norb_Cluster93	0.0022	0.0011	0.0505	0.0005	0.3276	0.6449128	Both
Latitude	norb_Cluster94	0.0017	-0.0009	0.0874	0.0394	0.1932	0.5146485	Both
Latitude	norb_Cluster96	0.0007	-0.0004	0.0494	-0.0006	0.3329	0.6449128	Both
Latitude	norb_Cluster97	0.0119	-0.0031	0.0378	-0.0129	0.3987	0.6818215	Both
Latitude	norb_Cluster98	0.0132	0.0037	0.0362	-0.0146	0.409	0.6881154	Both
Latitude	nosz_Cluster0	0.0045	-0.0012	0.0475	-0.0027	0.3428	0.6469675	Both
Latitude	nosz_Cluster10	0.0136	0.0032	0.0346	-0.0162	0.4195	0.6951728	Both

Latitude	nosz_Cluster102	0.0044	-0.0026	0.3232	0.2876	0.0072	0.065056	Both
Latitude	nosz_Cluster103	0.0455	-0.0145	0.183	0.14	0.0531	0.276095	Both
Latitude	nosz_Cluster105	0.0016	0.00E+00	0.0001	-0.0525	0.9605	0.9922099	Both
Latitude	nosz_Cluster106	0.0073	-0.0055	0.3643	0.3308	0.0038	0.0419421	Both
Latitude	nosz_Cluster107	0.0002	0.0002	0.0372	-0.0135	0.4025	0.6843069	Both
Latitude	nosz_Cluster108	0.004	0.0002	0.0015	-0.051	0.8672	0.94054	Both
Latitude	nosz_Cluster109	0.0375	-0.0075	0.1072	0.0602	0.1474	0.4514451	Both
Latitude	nosz_Cluster11	0.0129	0.0033	0.0176	-0.0341	0.5659	0.8003655	Both
Latitude	nosz_Cluster110	0.0003	0.0001	0.0096	-0.0426	0.6733	0.8525119	Both
Latitude	nosz_Cluster111	0.0061	-0.0043	0.3474	0.3131	0.0049	0.0500989	Both
Latitude	nosz_Cluster116	0.0067	0.00E+00	0.00E+00	-0.0526	0.9886	0.9960476	Both
Latitude	nosz_Cluster118	0.0045	0.0006	0.0067	-0.0455	0.7233	0.8763507	Both
Latitude	nosz_Cluster119	0.0013	-0.0021	0.5165	0.491	0.0002	0.0055021	Both
Latitude	nosz_Cluster120	0.0071	-0.0047	0.3578	0.324	0.0042	0.0456763	Both
Latitude	nosz_Cluster123	0.0097	-0.0096	0.5142	0.4887	0.0003	0.0055439	Both
Latitude	nosz_Cluster128	0.0047	0.0019	0.0645	0.0153	0.2665	0.5788647	Both
Latitude	nosz_Cluster129	0.0494	0.0004	0.0003	-0.0524	0.9453	0.9853752	Both
Latitude	nosz_Cluster13	0.0115	0.0041	0.0957	0.0481	0.1723	0.4819577	Both
Latitude	nosz_Cluster130	0.089	0.0238	0.0151	-0.0367	0.5956	0.8149128	Both
Latitude	nosz_Cluster132	0.0037	-0.0012	0.1014	0.0541	0.1595	0.463621	Both
Latitude	nosz_Cluster14	0.024	0.0123	0.0427	-0.0077	0.369	0.6656997	Both
Latitude	nosz_Cluster16	0.0076	0.0041	0.1053	0.0582	0.1512	0.4529022	Both
Latitude	nosz_Cluster17	0.0364	-0.0101	0.1059	0.0589	0.15	0.4515099	Both
Latitude	nosz_Cluster18	0.0161	-0.0031	0.0185	-0.0331	0.5565	0.8003655	Both
Latitude	nosz_Cluster20	0.0091	-0.0013	0.0092	-0.0429	0.679	0.8553162	Both
Latitude	nosz_Cluster21	0.0083	-0.0051	0.1478	0.103	0.0853	0.3495114	Both
Latitude	nosz_Cluster22	0.0267	0.0111	0.0722	0.0234	0.2389	0.559363	Both
Latitude	nosz_Cluster23	0.0141	-0.0081	0.172	0.1284	0.0616	0.2977565	Both
Latitude	nosz_Cluster24	0.0013	0.0001	0.0022	-0.0503	0.8386	0.9291018	Both
Latitude	nosz_Cluster25	0.0029	0.0006	0.0116	-0.0404	0.6423	0.8416253	Both
Latitude	nosz_Cluster26	0.0054	0.0002	0.0004	-0.0522	0.934	0.9845766	Both
Latitude	nosz_Cluster27	0.0053	-0.002	0.0798	0.0314	0.2147	0.5350909	Both
Latitude	nosz_Cluster28	0.0303	-1.00E-04	0.00E+00	-0.0526	0.9893	0.9960476	Both
Latitude	nosz_Cluster30	0.0276	-0.0029	0.0106	-0.0415	0.6574	0.8455026	Both
Latitude	nosz_Cluster32	0.0137	0.00E+00	0.00E+00	-0.0526	0.989	0.9960476	Both
Latitude	nosz_Cluster33	0.018	0.0057	0.0456	-0.0046	0.3527	0.6526336	Both
Latitude	nosz_Cluster34	0.0377	0.0047	0.0078	-0.0444	0.7033	0.8681363	Both
Latitude	nosz_Cluster35	0.0075	-0.0003	0.0008	-0.0518	0.9046	0.9686422	Both
Latitude	nosz_Cluster36	0.0179	0.0022	0.0116	-0.0404	0.6417	0.8416253	Both
Latitude	nosz_Cluster37	0.006	-0.0004	0.0022	-0.0504	0.8418	0.9295628	Both
Latitude	nosz_Cluster38	0.0128	-0.0078	0.1949	0.1525	0.0451	0.2514283	Both
Latitude	nosz_Cluster39	0.0537	-0.0126	0.0572	0.0076	0.2963	0.6148833	Both
Latitude	nosz_Cluster40	0.0218	-0.0009	0.0013	-0.0513	0.8788	0.9513729	Both
Latitude	nosz_Cluster41	0.0203	-0.0031	0.016	-0.0358	0.5844	0.8127667	Both
Latitude	nosz_Cluster42	0.0279	0.0027	0.0066	-0.0457	0.7257	0.8773698	Both
Latitude	nosz_Cluster43	0.0007	-0.0007	0.091	0.0432	0.1839	0.4930881	Both
Latitude	nosz_Cluster44	0.0057	-0.001	0.0183	-0.0334	0.5586	0.8003655	Both
Latitude	nosz_Cluster45	0.0045	0.0003	0.0022	-0.0503	0.8393	0.9291018	Both
Latitude	nosz_Cluster46	0.007	0.0012	0.0289	-0.0222	0.4615	0.7319159	Both
Latitude	nosz_Cluster47	0.0008	-1.00E-04	0.0024	-0.0501	0.8313	0.9291018	Both
Latitude	nosz_Cluster48	0.0023	-0.0017	0.1385	0.0932	0.0967	0.3770195	Both
Latitude	nosz_Cluster49	0.0014	0.0006	0.0581	0.0085	0.2925	0.6139403	Both
Latitude	nosz_Cluster5	0.0045	0.0021	0.0545	0.0048	0.3084	0.6231318	Both
Latitude	nosz_Cluster51	0.0021	0.0005	0.0176	-0.0341	0.5666	0.8003655	Both
Latitude	nosz_Cluster53	0.0009	0.0001	0.0019	-0.0506	0.85	0.9338705	Both

Latitude	nosz_Cluster54	0.0026	-0.0013	0.0653	0.0161	0.2637	0.5788647	Both
Latitude	nosz_Cluster57	0.1757	0.0716	0.1593	0.115	0.0731	0.338344	Both
Latitude	nosz_Cluster58	0.0043	-0.0035	0.245	0.2053	0.0225	0.159899	Both
Latitude	nosz_Cluster59	0.0009	-0.001	0.1106	0.0638	0.1407	0.4497192	Both
Latitude	nosz_Cluster6	0.0032	0.0016	0.0867	0.0386	0.1951	0.5146485	Both
Latitude	nosz_Cluster60	0.0079	-0.0022	0.0424	-0.008	0.3706	0.666714	Both
Latitude	nosz_Cluster61	0.0178	-0.0029	0.0571	0.0074	0.297	0.6148833	Both
Latitude	nosz_Cluster62	0.0413	0.0177	0.1751	0.1317	0.059	0.2901713	Both
Latitude	nosz_Cluster64	0.0016	-0.0013	0.0642	0.0149	0.2678	0.5788647	Both
Latitude	nosz_Cluster65	0.0065	-0.0005	0.003	-0.0494	0.8123	0.9270353	Both
Latitude	nosz_Cluster66	0.0324	0.017	0.1737	0.1303	0.0601	0.2932311	Both
Latitude	nosz_Cluster67	0.0521	0.0283	0.2039	0.1621	0.0399	0.2308286	Both
Latitude	nosz_Cluster69	0.0061	-0.0009	0.0125	-0.0395	0.6299	0.8360759	Both
Latitude	nosz_Cluster70	0.0479	0.0109	0.0137	-0.0382	0.6137	0.8304243	Both
Latitude	nosz_Cluster71	0.0083	0.0009	0.0053	-0.047	0.7535	0.8873989	Both
Latitude	nosz_Cluster72	0.0506	0.0207	0.1327	0.087	0.1045	0.3872157	Both
Latitude	nosz_Cluster73	0.003	-0.0015	0.0948	0.0472	0.1744	0.4826146	Both
Latitude	nosz_Cluster74	0.0017	-0.0019	0.4023	0.3709	0.002	0.0264029	Both
Latitude	nosz_Cluster75	0.0967	0.0355	0.0203	-0.0313	0.5378	0.7946675	Both
Latitude	nosz_Cluster77	0.0002	1.00E-04	0.0096	-0.0426	0.6733	0.8525119	Both
Latitude	nosz_Cluster78	0.0044	-0.0018	0.1062	0.0592	0.1493	0.4515099	Both
Latitude	nosz_Cluster79	0.0333	-0.0005	0.0003	-0.0523	0.9414	0.9853752	Both
Latitude	nosz_Cluster8	0.0099	0.00E+00	0.00E+00	-0.0526	0.9868	0.9960476	Both
Latitude	nosz_Cluster80	0.0045	0.0001	0.0002	-0.0525	0.955	0.9888128	Both
Latitude	nosz_Cluster81	0.0079	0.0038	0.07	0.0211	0.2464	0.5678557	Both
Latitude	nosz_Cluster82	0.0016	-0.0013	0.0473	-0.0029	0.3438	0.6469675	Both
Latitude	nosz_Cluster83	0.0015	-0.0019	0.2866	0.2491	0.0124	0.1014943	Both
Latitude	nosz_Cluster85	0.0022	-0.0001	0.001	-0.0516	0.8942	0.9627857	Both
Latitude	nosz_Cluster86	0.0027	-0.0005	0.0091	-0.0431	0.6814	0.8553162	Both
Latitude	nosz_Cluster87	0.003	-0.0018	0.1885	0.1458	0.0492	0.2640264	Both
Latitude	nosz_Cluster88	0.0218	-0.0039	0.0694	0.0204	0.2486	0.5708129	Both
Latitude	nosz_Cluster89	0.0466	0.0203	0.1473	0.1025	0.0858	0.3495114	Both
Latitude	nosz_Cluster9	0.003	-0.0005	0.0103	-0.0418	0.6614	0.8471892	Both
Latitude	nosz_Cluster90	0.0116	-0.0006	0.0037	-0.0487	0.7927	0.9136867	Both
Latitude	nosz_Cluster91	0.0189	0.0012	0.0024	-0.0501	0.8336	0.9291018	Both
Latitude	nosz_Cluster93	0.0393	-0.0237	0.3752	0.3423	0.0032	0.0380553	Both
Latitude	nosz_Cluster95	0.007	0.0007	0.0063	-0.046	0.7333	0.8812557	Both
Latitude	nosz_Cluster96	0.0066	-0.0027	0.1826	0.1396	0.0533	0.276095	Both
Latitude	nosz_Cluster97	0.0019	-0.0004	0.0128	-0.0391	0.6251	0.8360759	Both
Latitude	nosz_Cluster98	0.0025	-0.0016	0.0319	-0.0191	0.4389	0.7094062	Both
Latitude	nosz_Cluster99	0.0055	-0.0002	0.0015	-0.051	0.8672	0.94054	Both
Latitude	nrfa_Cluster10	0.0804	-0.0078	0.006	-0.0464	0.7393	0.8829803	Both
Latitude	nrfa_Cluster100	0.0963	-0.1108	0.613	0.5926	0.00E+00	0.0013366	Both
Latitude	nrfa_Cluster104	0.1143	-0.0868	0.6004	0.5794	0.00E+00	0.001566	Both
Latitude	nrfa_Cluster105	0.0062	-0.0008	0.0055	-0.0469	0.7502	0.8864514	Both
Latitude	nrfa_Cluster109	0.2294	-0.1561	0.5809	0.5588	1.00E-04	0.0023324	Both
Latitude	nrfa_Cluster116	0.2592	-0.1475	0.6214	0.6015	0.00E+00	0.0012835	Both
Latitude	nrfa_Cluster118	0.173	-0.2211	0.618	0.5979	0.00E+00	0.0012835	Both
Latitude	nrfa_Cluster125	0.0809	-0.1117	0.6411	0.6222	0.00E+00	0.0012835	Both
Latitude	nrfa_Cluster154	0.0658	-0.073	0.6217	0.6018	0.00E+00	0.0012835	Both
Latitude	nrfa_Cluster164	0.0045	0.0005	0.0038	-0.0486	0.7901	0.9136867	Both
Latitude	nrfa_Cluster172	0.0026	-0.0001	0.0004	-0.0522	0.9311	0.9844606	Both
Latitude	nrfa_Cluster175	0.0044	-0.0066	0.5456	0.5217	0.0001	0.0034265	Both
Latitude	nrfa_Cluster20	0.035	-0.0333	0.5553	0.5319	0.0001	0.0031588	Both
Latitude	nrfa_Cluster26	0.1078	-0.1244	0.5559	0.5326	0.0001	0.0031588	Both

Latitude	nrfa_Cluster29	0.0048	0.0028	0.1248	0.0787	0.1162	0.4002661	Both
Latitude	nrfa_Cluster34	0.0487	-0.0501	0.6383	0.6193	0.00E+00	0.0012835	Both
Latitude	nrfa_Cluster38	0.0031	-0.0035	0.367	0.3337	0.0036	0.0409445	Both
Latitude	nrfa_Cluster61	0.0036	-0.0046	0.5549	0.5315	0.0001	0.0031588	Both
Latitude	nrfa_Cluster62	0.0452	-0.0606	0.6248	0.6051	0.00E+00	0.0012835	Both
Latitude	nrfa_Cluster66	0.1082	-0.0641	0.5221	0.497	0.0002	0.0050952	Both
Latitude	nrfa_Cluster69	0.003	0.0019	0.1488	0.104	0.0842	0.3495114	Both
Latitude	nrfa_Cluster71	0.027	-0.0272	0.4602	0.4318	0.0007	0.0125647	Both
Latitude	nrfa_Cluster72	0.0732	-0.0798	0.6504	0.632	0.00E+00	0.0012835	Both
Latitude	nrfa_Cluster73	0.0038	-0.0011	0.0219	-0.0296	0.5219	0.785529	Both
Latitude	nrfa_Cluster74	0.1331	-0.1309	0.5589	0.5357	1.00E-04	0.0031588	Both
Latitude	nrfa_Cluster76	0.0755	-0.0695	0.4201	0.3896	0.0015	0.0203725	Both
Latitude	nrfa_Cluster79	0.0229	0.0113	0.0323	-0.0187	0.436	0.7094062	Both
Latitude	nrfa_Cluster87	0.017	0.0059	0.0344	-0.0164	0.421	0.695817	Both
Latitude	nrfa_Cluster89	0.0018	-0.0011	0.083	0.0347	0.2054	0.5290731	Both
Latitude	nrfa_Cluster92	0.0043	0.0003	0.002	-0.0505	0.8465	0.931734	Both
Latitude	nrfa_Cluster93	0.0428	0.0311	0.1532	0.1087	0.0793	0.3495114	Both
Latitude	nrfa_Cluster94	0.0002	-0.0005	0.2017	0.1597	0.0411	0.235434	Both
Latitude	nrfa_Cluster97	0.0168	-0.0112	0.3144	0.2783	0.0082	0.0721022	Both
Latitude	nrfa_Cluster99	0.014	-0.0117	0.4177	0.3871	0.0015	0.0207517	Both
Longitude	amoA_A_Cluster26	0.004	0.0004	0.0022	-0.0504	0.8415	0.9827843	Both
Longitude	amoA_A_Cluster45	0.0051	-0.0005	0.0035	-0.049	0.8003	0.9697956	Both
Longitude	amoA_B_Cluster0	0.1099	0.0037	0.0054	-0.047	0.7518	0.95251	Both
Longitude	amoA_B_Cluster1	0.0172	-0.0113	0.4335	0.4036	0.0012	0.0288914	Both
Longitude	amoA_B_Cluster10	0.0422	-0.01	0.1052	0.0581	0.1515	0.7397849	Both
Longitude	amoA_B_Cluster11	0.0377	-0.0202	0.3951	0.3633	0.0023	0.0486704	Both
Longitude	amoA_B_Cluster12	0.0807	-0.0056	0.0171	-0.0346	0.5718	0.9073675	Both
Longitude	amoA_B_Cluster13	0.5271	0.0067	0.0003	-0.0523	0.9436	0.9889693	Both
Longitude	amoA_B_Cluster14	0.0275	0.0053	0.0331	-0.0178	0.4298	0.9073675	Both
Longitude	amoA_B_Cluster15	0.0318	-0.0121	0.1881	0.1453	0.0495	0.4638825	Both
Longitude	amoA_B_Cluster2	0.1721	-0.0198	0.0215	-0.03	0.5259	0.9073675	Both
Longitude	amoA_B_Cluster20	0.0058	-0.0044	0.2317	0.1913	0.0272	0.3337713	Both
Longitude	amoA_B_Cluster23	0.0507	-0.0214	0.3732	0.3402	0.0033	0.0664392	Both
Longitude	amoA_B_Cluster3	0.0182	-0.013	0.4302	0.4002	0.0012	0.0293624	Both
Longitude	amoA_B_Cluster4	0.0125	-0.0045	0.092	0.0442	0.1814	0.8046692	Both
Longitude	amoA_B_Cluster5	0.0928	-0.0027	0.0033	-0.0492	0.806	0.9705301	Both
Longitude	amoA_B_Cluster6	0.0233	-0.0079	0.2144	0.173	0.0345	0.3919537	Both
Longitude	amoA_B_Cluster7	0.0179	-0.013	0.438	0.4084	0.0011	0.0277975	Both
Longitude	amoA_B_Cluster8	0.0207	-0.0186	0.6565	0.6384	0.00E+00	0.0008351	Both
Longitude	amoA_B_Cluster9	0.0549	-0.0275	0.2842	0.2466	0.0128	0.1939561	Both
Longitude	nifh_Cluster0	0.0332	-0.0017	0.0116	-0.0404	0.6424	0.9073675	Both
Longitude	nifh_Cluster10	0.005	0.0013	0.0148	-0.0371	0.5996	0.9073675	Both
Longitude	nifh_Cluster100	0.0019	0.0007	0.0326	-0.0183	0.4334	0.9073675	Both
Longitude	nifh_Cluster103	0.0383	-0.0071	0.0762	0.0276	0.2259	0.865385	Both
Longitude	nifh_Cluster1034	0.0262	0.0063	0.0424	-0.008	0.3704	0.9073675	Both
Longitude	nifh_Cluster105	0.013	0.0044	0.0787	0.0302	0.218	0.865385	Both
Longitude	nifh_Cluster108	0.0083	0.0013	0.0214	-0.0301	0.5266	0.9073675	Both
Longitude	nifh_Cluster1099	0.066	0.0017	0.0007	-0.0519	0.9086	0.9889693	Both
Longitude	nifh_Cluster11	0.0021	0.0005	0.0284	-0.0227	0.4652	0.9073675	Both
Longitude	nifh_Cluster1112	0.0029	-0.0012	0.06	0.0105	0.2847	0.9073675	Both
Longitude	nifh_Cluster112	0.0112	0.0031	0.0678	0.0187	0.2543	0.8923808	Both
Longitude	nifh_Cluster113	0.0327	-0.003	0.022	-0.0295	0.5209	0.9073675	Both
Longitude	nifh_Cluster114	0.0332	0.0028	0.0399	-0.0106	0.3852	0.9073675	Both
Longitude	nifh_Cluster1141	0.0251	0.0032	0.0186	-0.033	0.5553	0.9073675	Both
Longitude	nifh_Cluster115	0.0095	-0.0009	0.0143	-0.0376	0.6055	0.9073675	Both

Longitude	nifh_Cluster116	0.0775	-0.0298	0.3942	0.3623	0.0023	0.0486704	Both
Longitude	nifh_Cluster1163	0.0044	-0.0017	0.1303	0.0845	0.1079	0.6430341	Both
Longitude	nifh_Cluster1179	0.0031	0.0015	0.0746	0.0259	0.2308	0.8728148	Both
Longitude	nifh_Cluster1182	0.0204	-0.0014	0.0218	-0.0297	0.5234	0.9073675	Both
Longitude	nifh_Cluster1183	0.0066	0.0007	0.0151	-0.0367	0.5952	0.9073675	Both
Longitude	nifh_Cluster1197	0.0115	-0.0021	0.0428	-0.0076	0.3685	0.9073675	Both
Longitude	nifh_Cluster1199	0.0046	-0.0007	0.015	-0.0369	0.597	0.9073675	Both
Longitude	nifh_Cluster120	0.0149	0.005	0.0314	-0.0196	0.4425	0.9073675	Both
Longitude	nifh_Cluster1203	0.0155	-0.0118	0.3557	0.3218	0.0043	0.0822248	Both
Longitude	nifh_Cluster1206	0.0043	-0.004	0.5411	0.517	0.0001	0.0050173	Both
Longitude	nifh_Cluster1207	0.0074	0.0018	0.036	-0.0147	0.4098	0.9073675	Both
Longitude	nifh_Cluster1209	0.1506	-0.041	0.5452	0.5212	0.0001	0.0048878	Both
Longitude	nifh_Cluster121	0.0095	0.0018	0.0115	-0.0405	0.643	0.9073675	Both
Longitude	nifh_Cluster1211	0.0056	-0.0049	0.6232	0.6034	0.00E+00	0.0015436	Both
Longitude	nifh_Cluster1213	0.0026	-0.0008	0.0511	0.0012	0.3244	0.9073675	Both
Longitude	nifh_Cluster122	0.0029	0.0005	0.0095	-0.0426	0.6735	0.9124461	Both
Longitude	nifh_Cluster123	0.0095	0.0028	0.0271	-0.0241	0.476	0.9073675	Both
Longitude	nifh_Cluster1236	0.0234	-0.0047	0.0164	-0.0354	0.5801	0.9073675	Both
Longitude	nifh_Cluster1238	0.0028	0.0008	0.0331	-0.0177	0.4296	0.9073675	Both
Longitude	nifh_Cluster124	0.0091	-0.0017	0.0397	-0.0108	0.3863	0.9073675	Both
Longitude	nifh_Cluster1261	0.002	-0.0002	0.0021	-0.0504	0.8442	0.9827843	Both
Longitude	nifh_Cluster1262	0.0065	0.0035	0.1604	0.1163	0.072	0.5172457	Both
Longitude	nifh_Cluster1266	0.0094	0.0034	0.1167	0.0702	0.1297	0.7053789	Both
Longitude	nifh_Cluster1267	0.0562	0.0122	0.0399	-0.0106	0.3852	0.9073675	Both
Longitude	nifh_Cluster127	0.0026	0.0008	0.024	-0.0274	0.5029	0.9073675	Both
Longitude	nifh_Cluster1278	0.1602	-0.0553	0.1777	0.1345	0.057	0.4743676	Both
Longitude	nifh_Cluster128	0.0036	0.0011	0.0479	-0.0023	0.3408	0.9073675	Both
Longitude	nifh_Cluster1289	0.0039	0.0006	0.016	-0.0358	0.5844	0.9073675	Both
Longitude	nifh_Cluster129	0.0077	0.0022	0.0303	-0.0208	0.4507	0.9073675	Both
Longitude	nifh_Cluster1292	0.0086	-0.0028	0.0776	0.029	0.2216	0.865385	Both
Longitude	nifh_Cluster130	0.0059	0.0016	0.0189	-0.0327	0.5524	0.9073675	Both
Longitude	nifh_Cluster1305	0.0048	-0.0007	0.0197	-0.0319	0.5436	0.9073675	Both
Longitude	nifh_Cluster1306	0.005	-0.0007	0.0146	-0.0373	0.602	0.9073675	Both
Longitude	nifh_Cluster131	0.0051	0.0012	0.0194	-0.0322	0.5468	0.9073675	Both
Longitude	nifh_Cluster1319	0.0036	0.0006	0.0112	-0.0408	0.6476	0.9073675	Both
Longitude	nifh_Cluster132	0.0064	0.0021	0.0504	0.0004	0.3281	0.9073675	Both
Longitude	nifh_Cluster1320	0.0025	-0.0012	0.0641	0.0148	0.2683	0.9073675	Both
Longitude	nifh_Cluster1323	0.0023	-0.0012	0.1612	0.117	0.0713	0.5172457	Both
Longitude	nifh_Cluster1324	0.03	0.0087	0.1027	0.0554	0.1567	0.7397849	Both
Longitude	nifh_Cluster1336	0.0153	-0.0026	0.1091	0.0622	0.1437	0.7307538	Both
Longitude	nifh_Cluster1340	0.0363	-0.0442	0.7606	0.7481	0.00E+00	7.60E-05	Both
Longitude	nifh_Cluster1345	0.0483	0.0065	0.0152	-0.0367	0.5948	0.9073675	Both
Longitude	nifh_Cluster1370	0.0021	0.0006	0.0296	-0.0214	0.4556	0.9073675	Both
Longitude	nifh_Cluster1375	0.0018	-0.0002	0.0045	-0.0479	0.7724	0.9621867	Both
Longitude	nifh_Cluster139	0.0041	-0.0023	0.1372	0.0918	0.0984	0.6012544	Both
Longitude	nifh_Cluster140	0.0043	-0.0004	0.0073	-0.045	0.7133	0.9331847	Both
Longitude	nifh_Cluster141	0.0677	-0.0067	0.0222	-0.0293	0.5192	0.9073675	Both
Longitude	nifh_Cluster148	0.0046	-0.0033	0.1588	0.1145	0.0736	0.5172457	Both
Longitude	nifh_Cluster1480	0.001	0.0001	0.0049	-0.0474	0.7625	0.9592071	Both
Longitude	nifh_Cluster152	0.0599	0.0147	0.1905	0.1478	0.0479	0.4637206	Both
Longitude	nifh_Cluster156	0.0659	0.0221	0.1995	0.1574	0.0424	0.4456887	Both
Longitude	nifh_Cluster1562	0.0543	0.0183	0.0767	0.0281	0.2242	0.865385	Both
Longitude	nifh_Cluster158	0.0144	-0.0057	0.1131	0.0664	0.136	0.7150828	Both
Longitude	nifh_Cluster16	0.0019	0.0005	0.0162	-0.0356	0.5825	0.9073675	Both
Longitude	nifh_Cluster166	0.0172	0.0053	0.0297	-0.0213	0.4547	0.9073675	Both

Longitude	nifh_Cluster205	0.0122	-0.0002	0.0002	-0.0525	0.9573	0.9889693	Both
Longitude	nifh_Cluster21	0.0021	1.00E-04	0.0005	-0.0521	0.9208	0.9889693	Both
Longitude	nifh_Cluster222	0.006	0.0019	0.0284	-0.0228	0.4654	0.9073675	Both
Longitude	nifh_Cluster225	0.0189	-0.0024	0.0368	-0.0139	0.405	0.9073675	Both
Longitude	nifh_Cluster230	0.0042	0.0002	0.0018	-0.0507	0.8541	0.9850024	Both
Longitude	nifh_Cluster231	0.0022	-0.0001	0.0012	-0.0514	0.8826	0.9889693	Both
Longitude	nifh_Cluster236	0.0027	0.00E+00	0.0002	-0.0525	0.9576	0.9889693	Both
Longitude	nifh_Cluster237	0.0058	0.0012	0.0372	-0.0134	0.402	0.9073675	Both
Longitude	nifh_Cluster243	0.0333	0.0087	0.0706	0.0217	0.2444	0.8871655	Both
Longitude	nifh_Cluster2432	0.0436	0.0037	0.0071	-0.0451	0.7162	0.9342816	Both
Longitude	nifh_Cluster2433	0.0032	-0.0011	0.0357	-0.0151	0.4122	0.9073675	Both
Longitude	nifh_Cluster246	0.0057	0.0011	0.0173	-0.0344	0.5697	0.9073675	Both
Longitude	nifh_Cluster25	0.0295	0.0075	0.1062	0.0592	0.1494	0.7397849	Both
Longitude	nifh_Cluster256	0.0029	0.00E+00	1.00E-04	-0.0526	0.9712	0.9930474	Both
Longitude	nifh_Cluster264	0.0029	0.0011	0.0859	0.0378	0.1974	0.8320722	Both
Longitude	nifh_Cluster265	0.0736	0.0002	0.00E+00	-0.0526	0.9799	0.9951163	Both
Longitude	nifh_Cluster267	0.0109	0.0009	0.0055	-0.0468	0.7491	0.95251	Both
Longitude	nifh_Cluster268	0.0111	0.001	0.0126	-0.0393	0.6274	0.9073675	Both
Longitude	nifh_Cluster269	0.023	-0.007	0.0714	0.0226	0.2415	0.8871655	Both
Longitude	nifh_Cluster272	0.0041	0.0015	0.043	-0.0074	0.3673	0.9073675	Both
Longitude	nifh_Cluster274	0.0032	0.001	0.0102	-0.0418	0.6624	0.9114297	Both
Longitude	nifh_Cluster275	0.0036	-0.0007	0.0255	-0.0258	0.4893	0.9073675	Both
Longitude	nifh_Cluster278	0.0077	0.0014	0.0244	-0.027	0.4993	0.9073675	Both
Longitude	nifh_Cluster279	0.006	0.0005	0.0089	-0.0432	0.6838	0.9149597	Both
Longitude	nifh_Cluster28	0.002	-0.0003	0.0135	-0.0384	0.6159	0.9073675	Both
Longitude	nifh_Cluster280	0.0031	-0.0024	0.1444	0.0994	0.0892	0.5673491	Both
Longitude	nifh_Cluster282	0.0052	-0.0005	0.0097	-0.0425	0.6717	0.9124461	Both
Longitude	nifh_Cluster287	0.0022	1.00E-04	0.0007	-0.0519	0.9107	0.9889693	Both
Longitude	nifh_Cluster290	0.011	0.0015	0.0194	-0.0323	0.5475	0.9073675	Both
Longitude	nifh_Cluster297	0.0029	0.0003	0.002	-0.0505	0.8464	0.9827843	Both
Longitude	nifh_Cluster299	0.0026	0.0009	0.0368	-0.0139	0.4048	0.9073675	Both
Longitude	nifh_Cluster303	0.002	0.0005	0.0217	-0.0298	0.5245	0.9073675	Both
Longitude	nifh_Cluster304	0.0024	0.0005	0.0198	-0.0318	0.5433	0.9073675	Both
Longitude	nifh_Cluster313	0.0103	0.0025	0.0208	-0.0308	0.5332	0.9073675	Both
Longitude	nifh_Cluster314	0.0022	0.0007	0.0345	-0.0163	0.4204	0.9073675	Both
Longitude	nifh_Cluster317	0.0071	0.0009	0.0119	-0.0401	0.6381	0.9073675	Both
Longitude	nifh_Cluster32	0.0033	0.0012	0.088	0.04	0.1917	0.825468	Both
Longitude	nifh_Cluster320	12.7419	0.0909	0.0008	-0.0517	0.9008	0.9889693	Both
Longitude	nifh_Cluster334	0.0063	0.0022	0.0314	-0.0196	0.4421	0.9073675	Both
Longitude	nifh_Cluster335	0.0075	0.0028	0.0279	-0.0232	0.469	0.9073675	Both
Longitude	nifh_Cluster338	0.0123	-0.0042	0.1401	0.0948	0.0947	0.5878515	Both
Longitude	nifh_Cluster340	0.007	0.0002	0.0001	-0.0525	0.9605	0.9889693	Both
Longitude	nifh_Cluster341	0.0023	0.0007	0.0314	-0.0196	0.4426	0.9073675	Both
Longitude	nifh_Cluster35	0.0078	-0.0035	0.244	0.2042	0.0229	0.2932573	Both
Longitude	nifh_Cluster351	0.0025	0.0008	0.0271	-0.0241	0.4761	0.9073675	Both
Longitude	nifh_Cluster3574	0.1093	0.0151	0.0621	0.0127	0.2761	0.9073675	Both
Longitude	nifh_Cluster3691	0.0174	-0.0156	0.4763	0.4487	0.0005	0.0143601	Both
Longitude	nifh_Cluster37	0.0329	0.0029	0.006	-0.0463	0.7376	0.9449771	Both
Longitude	nifh_Cluster374	0.0021	-0.0036	0.7632	0.7507	0.00E+00	7.60E-05	Both
Longitude	nifh_Cluster38	0.0009	0.0003	0.0162	-0.0355	0.582	0.9073675	Both
Longitude	nifh_Cluster382	0.0016	0.0003	0.0044	-0.048	0.7762	0.9621867	Both
Longitude	nifh_Cluster384	0.0019	0.0003	0.0089	-0.0433	0.6841	0.9149597	Both
Longitude	nifh_Cluster386	0.0035	0.0013	0.0373	-0.0134	0.4017	0.9073675	Both
Longitude	nifh_Cluster389	0.0031	0.001	0.0327	-0.0182	0.4328	0.9073675	Both
Longitude	nifh_Cluster394	0.002	0.0006	0.0149	-0.037	0.5983	0.9073675	Both

Longitude	nifh_Cluster40	0.0012	0.0005	0.0554	0.0057	0.3042	0.9073675	Both
Longitude	nifh_Cluster43	0.0108	0.0006	0.0042	-0.0482	0.7805	0.9633349	Both
Longitude	nifh_Cluster434	0.0035	-0.0021	0.1248	0.0787	0.1162	0.6788367	Both
Longitude	nifh_Cluster440	0.0133	-0.0058	0.1138	0.0672	0.1348	0.7150828	Both
Longitude	nifh_Cluster462	0.0034	-1.00E-04	0.0001	-0.0525	0.9603	0.9889693	Both
Longitude	nifh_Cluster470	0.0152	-0.0079	0.3397	0.3049	0.0056	0.1024913	Both
Longitude	nifh_Cluster49	0.0213	0.004	0.0141	-0.0378	0.6084	0.9073675	Both
Longitude	nifh_Cluster499	0.0026	0.0008	0.0433	-0.0071	0.3654	0.9073675	Both
Longitude	nifh_Cluster5	0.0256	-0.0053	0.1677	0.1239	0.0653	0.5000079	Both
Longitude	nifh_Cluster506	0.0048	-0.0019	0.1056	0.0585	0.1507	0.7397849	Both
Longitude	nifh_Cluster51	0.0028	0.001	0.035	-0.0158	0.4169	0.9073675	Both
Longitude	nifh_Cluster52	0.0336	0.0092	0.0438	-0.0066	0.3628	0.9073675	Both
Longitude	nifh_Cluster525	0.0033	0.0022	0.0717	0.0228	0.2406	0.8871655	Both
Longitude	nifh_Cluster531	0.0258	0.0148	0.2844	0.2467	0.0128	0.1939561	Both
Longitude	nifh_Cluster539	0.0048	0.0018	0.0264	-0.0249	0.482	0.9073675	Both
Longitude	nifh_Cluster578	0.0481	-0.0223	0.3639	0.3304	0.0038	0.0745905	Both
Longitude	nifh_Cluster58	0.3542	0.0687	0.038	-0.0126	0.3971	0.9073675	Both
Longitude	nifh_Cluster60	0.0089	0.0007	0.0038	-0.0486	0.7899	0.9696436	Both
Longitude	nifh_Cluster61	0.0025	0.0014	0.149	0.1042	0.0839	0.5540719	Both
Longitude	nifh_Cluster65	0.009	0.0019	0.0251	-0.0262	0.4931	0.9073675	Both
Longitude	nifh_Cluster686	0.0009	0.0005	0.0598	0.0104	0.2852	0.9073675	Both
Longitude	nifh_Cluster69	0.009	0.0017	0.019	-0.0326	0.5512	0.9073675	Both
Longitude	nifh_Cluster70	0.0111	0.0028	0.0181	-0.0335	0.5605	0.9073675	Both
Longitude	nifh_Cluster717	0.003	0.0001	0.0011	-0.0515	0.8856	0.9889693	Both
Longitude	nifh_Cluster725	0.0058	0.0016	0.0559	0.0062	0.3022	0.9073675	Both
Longitude	nifh_Cluster727	0.0039	-0.0008	0.0205	-0.0311	0.5359	0.9073675	Both
Longitude	nifh_Cluster73	0.0106	0.0036	0.0681	0.0191	0.253	0.8923808	Both
Longitude	nifh_Cluster74	0.0049	0.0014	0.0275	-0.0237	0.4725	0.9073675	Both
Longitude	nifh_Cluster748	0.0081	-0.001	0.0185	-0.0332	0.557	0.9073675	Both
Longitude	nifh_Cluster749	0.0071	0.0012	0.0303	-0.0207	0.4503	0.9073675	Both
Longitude	nifh_Cluster755	0.0102	-0.007	0.2933	0.2561	0.0112	0.1788954	Both
Longitude	nifh_Cluster76	0.0029	-0.0007	0.0236	-0.0277	0.5058	0.9073675	Both
Longitude	nifh_Cluster79	0.0022	0.0009	0.083	0.0347	0.2053	0.847194	Both
Longitude	nifh_Cluster81	0.0055	0.0026	0.0683	0.0193	0.2524	0.8923808	Both
Longitude	nifh_Cluster82	0.0011	0.0004	0.0242	-0.0272	0.5009	0.9073675	Both
Longitude	nifh_Cluster83	0.0069	-0.0027	0.0768	0.0282	0.2239	0.865385	Both
Longitude	nifh_Cluster86	0.0135	-0.0099	0.5807	0.5586	1.00E-04	0.0027037	Both
Longitude	nifh_Cluster868	0.0099	0.0001	0.0002	-0.0524	0.9484	0.9889693	Both
Longitude	nifh_Cluster87	0.0024	0.0006	0.0183	-0.0334	0.5587	0.9073675	Both
Longitude	nifh_Cluster88	0.0039	-0.0057	0.7014	0.6856	0.00E+00	0.0003222	Both
Longitude	nifh_Cluster9	0.0078	-0.0014	0.022	-0.0295	0.5214	0.9073675	Both
Longitude	nifh_Cluster92	0.0051	0.0014	0.0204	-0.0311	0.5364	0.9073675	Both
Longitude	nifh_Cluster93	0.0036	0.0001	0.0006	-0.052	0.9188	0.9889693	Both
Longitude	nifh_Cluster94	0.0047	0.0012	0.0208	-0.0308	0.5329	0.9073675	Both
Longitude	nifh_Cluster95	0.0121	0.0036	0.0402	-0.0103	0.3836	0.9073675	Both
Longitude	nifh_Cluster96	0.0123	-0.0062	0.267	0.2284	0.0165	0.2369052	Both
Longitude	nifh_Cluster97	0.018	0.004	0.0379	-0.0128	0.3979	0.9073675	Both
Longitude	nifh_Cluster98	0.0059	-0.0026	0.1762	0.1328	0.0582	0.4743676	Both
Longitude	nifh_Cluster99	0.0033	0.0008	0.023	-0.0284	0.5114	0.9073675	Both
Longitude	nirk_Cluster0	5.9009	0.1773	0.014	-0.0379	0.6092	0.9073675	Both
Longitude	nirk_Cluster1	0.1708	0.0226	0.0273	-0.0239	0.4742	0.9073675	Both
Longitude	nirk_Cluster10	0.5713	-0.2197	0.7394	0.7256	0.00E+00	0.0001152	Both
Longitude	nirk_Cluster101	0.0031	-0.0007	0.022	-0.0295	0.5213	0.9073675	Both
Longitude	nirk_Cluster102	0.0426	-0.0045	0.0146	-0.0372	0.6015	0.9073675	Both
Longitude	nirk_Cluster103	0.0754	-0.0002	0.00E+00	-0.0526	0.9881	0.9960176	Both

Longitude	nirk_Cluster104	0.0023	-0.0009	0.0496	-0.0005	0.332	0.9073675	Both
Longitude	nirk_Cluster105	0.0946	-0.0044	0.0038	-0.0486	0.7897	0.9696436	Both
Longitude	nirk_Cluster106	0.0592	0.007	0.0142	-0.0377	0.6074	0.9073675	Both
Longitude	nirk_Cluster107	0.0891	-0.0105	0.0213	-0.0302	0.5281	0.9073675	Both
Longitude	nirk_Cluster108	0.0151	-0.0036	0.0529	0.0031	0.3158	0.9073675	Both
Longitude	nirk_Cluster109	0.0115	-0.0033	0.0668	0.0176	0.2581	0.8923808	Both
Longitude	nirk_Cluster11	0.1401	-0.0083	0.0559	0.0062	0.302	0.9073675	Both
Longitude	nirk_Cluster12	0.094	0.0112	0.0601	0.0107	0.284	0.9073675	Both
Longitude	nirk_Cluster13	0.0408	0.0029	0.0328	-0.0181	0.432	0.9073675	Both
Longitude	nirk_Cluster14	0.0946	-0.0178	0.1443	0.0992	0.0894	0.5673491	Both
Longitude	nirk_Cluster15	0.3358	0.0275	0.0121	-0.0399	0.6352	0.9073675	Both
Longitude	nirk_Cluster16	0.4727	0.0702	0.0365	-0.0142	0.4066	0.9073675	Both
Longitude	nirk_Cluster17	0.0998	-0.0089	0.0559	0.0062	0.302	0.9073675	Both
Longitude	nirk_Cluster18	0.1296	0.0188	0.0404	-0.0101	0.3822	0.9073675	Both
Longitude	nirk_Cluster19	0.2776	0.0233	0.0771	0.0285	0.2229	0.865385	Both
Longitude	nirk_Cluster2	0.2032	-0.0067	0.0095	-0.0426	0.6743	0.9124461	Both
Longitude	nirk_Cluster20	0.1738	-0.0348	0.146	0.1011	0.0874	0.5665468	Both
Longitude	nirk_Cluster21	0.1532	-0.0157	0.0857	0.0375	0.1979	0.8320722	Both
Longitude	nirk_Cluster22	0.1291	0.0025	0.0017	-0.0508	0.8592	0.986755	Both
Longitude	nirk_Cluster23	0.0673	-0.0048	0.0494	-0.0007	0.333	0.9073675	Both
Longitude	nirk_Cluster24	0.0654	-0.0158	0.1889	0.1462	0.0489	0.4638825	Both
Longitude	nirk_Cluster25	0.4782	-0.0763	0.113	0.0663	0.1362	0.7150828	Both
Longitude	nirk_Cluster26	0.105	0.0008	0.0004	-0.0522	0.9275	0.9889693	Both
Longitude	nirk_Cluster27	0.1727	-0.0305	0.1029	0.0557	0.1562	0.7397849	Both
Longitude	nirk_Cluster28	0.0685	-0.0148	0.1562	0.1118	0.0762	0.5224533	Both
Longitude	nirk_Cluster29	0.1526	-0.0455	0.597	0.5758	0.00E+00	0.002382	Both
Longitude	nirk_Cluster3	0.3023	-0.0401	0.1113	0.0646	0.1394	0.7150828	Both
Longitude	nirk_Cluster30	0.0556	0.0014	0.001	-0.0516	0.8932	0.9889693	Both
Longitude	nirk_Cluster31	0.1501	0.0083	0.0384	-0.0122	0.3944	0.9073675	Both
Longitude	nirk_Cluster32	0.1254	0.0171	0.0247	-0.0266	0.4963	0.9073675	Both
Longitude	nirk_Cluster33	0.0638	0.0038	0.0043	-0.0481	0.7771	0.9621867	Both
Longitude	nirk_Cluster34	0.0416	-0.0059	0.0445	-0.0058	0.3588	0.9073675	Both
Longitude	nirk_Cluster35	0.1007	-0.0279	0.0909	0.0431	0.1841	0.8046692	Both
Longitude	nirk_Cluster36	0.0017	-0.0004	0.0184	-0.0333	0.5577	0.9073675	Both
Longitude	nirk_Cluster39	0.0035	-0.0043	0.2523	0.2129	0.0203	0.2664969	Both
Longitude	nirk_Cluster4	0.1323	-0.0166	0.0628	0.0134	0.2734	0.9073675	Both
Longitude	nirk_Cluster40	0.0455	-0.005	0.0355	-0.0153	0.4134	0.9073675	Both
Longitude	nirk_Cluster41	0.1562	-0.0057	0.0073	-0.0449	0.7122	0.9331847	Both
Longitude	nirk_Cluster44	0.0669	0.0012	0.0005	-0.0521	0.9254	0.9889693	Both
Longitude	nirk_Cluster46	0.0154	-0.0034	0.034	-0.0169	0.4237	0.9073675	Both
Longitude	nirk_Cluster48	0.003	0.0002	0.0018	-0.0507	0.8548	0.9850024	Both
Longitude	nirk_Cluster49	0.0057	0.0027	0.0911	0.0432	0.1837	0.8046692	Both
Longitude	nirk_Cluster5	0.1932	-0.0104	0.0223	-0.0292	0.5187	0.9073675	Both
Longitude	nirk_Cluster50	0.0016	1.00E-04	0.0014	-0.0512	0.8732	0.9889693	Both
Longitude	nirk_Cluster52	0.0452	-0.0038	0.0156	-0.0362	0.5895	0.9073675	Both
Longitude	nirk_Cluster53	0.0038	-0.0002	0.0008	-0.0518	0.9027	0.9889693	Both
Longitude	nirk_Cluster54	0.002	-0.0005	0.0198	-0.0318	0.5434	0.9073675	Both
Longitude	nirk_Cluster55	0.0023	1.00E-04	0.0003	-0.0523	0.9426	0.9889693	Both
Longitude	nirk_Cluster56	0.0009	0.0004	0.0383	-0.0124	0.3954	0.9073675	Both
Longitude	nirk_Cluster57	0.0016	0.0004	0.0096	-0.0426	0.6732	0.9124461	Both
Longitude	nirk_Cluster58	0.0088	-0.003	0.0881	0.0401	0.1913	0.825468	Both
Longitude	nirk_Cluster59	0.0058	-0.0031	0.1967	0.1544	0.044	0.4456887	Both
Longitude	nirk_Cluster6	0.1558	-0.0293	0.1773	0.134	0.0573	0.4743676	Both
Longitude	nirk_Cluster60	0.0006	-0.0002	0.0169	-0.0348	0.5741	0.9073675	Both
Longitude	nirk_Cluster62	0.1417	-0.0093	0.006	-0.0463	0.7384	0.9449771	Both

Longitude	nirk_Cluster63	0.1284	-0.0255	0.0851	0.037	0.1994	0.8320722	Both
Longitude	nirk_Cluster64	0.0044	-0.001	0.0385	-0.0121	0.3937	0.9073675	Both
Longitude	nirk_Cluster65	0.0464	-0.0089	0.0954	0.0478	0.1732	0.7792279	Both
Longitude	nirk_Cluster66	0.0068	-0.0001	0.0003	-0.0523	0.9363	0.9889693	Both
Longitude	nirk_Cluster67	0.0174	-0.016	0.6275	0.6079	0.00E+00	0.0015436	Both
Longitude	nirk_Cluster68	0.0125	-0.0035	0.0765	0.0279	0.2247	0.865385	Both
Longitude	nirk_Cluster69	0.0305	-0.0097	0.2077	0.166	0.0379	0.4099058	Both
Longitude	nirk_Cluster7	0.0804	-0.029	0.3945	0.3626	0.0023	0.0486704	Both
Longitude	nirk_Cluster70	0.0721	0.0084	0.0102	-0.0419	0.6628	0.9114297	Both
Longitude	nirk_Cluster71	0.0053	-0.0015	0.0532	0.0034	0.3143	0.9073675	Both
Longitude	nirk_Cluster72	0.0239	-0.0026	0.0229	-0.0286	0.5129	0.9073675	Both
Longitude	nirk_Cluster73	0.1444	-0.0268	0.0575	0.0079	0.295	0.9073675	Both
Longitude	nirk_Cluster74	0.6398	0.0226	0.0019	-0.0506	0.851	0.9845049	Both
Longitude	nirk_Cluster75	0.0147	-0.0059	0.086	0.0379	0.1969	0.8320722	Both
Longitude	nirk_Cluster76	0.1055	-0.0163	0.0555	0.0058	0.304	0.9073675	Both
Longitude	nirk_Cluster77	0.0081	-0.0143	0.6698	0.6524	0.00E+00	0.0006831	Both
Longitude	nirk_Cluster78	0.2407	-0.0439	0.0652	0.016	0.2638	0.8998097	Both
Longitude	nirk_Cluster79	0.0069	-0.0092	0.5831	0.5612	1.00E-04	0.0027037	Both
Longitude	nirk_Cluster8	0.0982	-0.0161	0.0573	0.0077	0.296	0.9073675	Both
Longitude	nirk_Cluster80	0.2665	-0.039	0.0343	-0.0166	0.4219	0.9073675	Both
Longitude	nirk_Cluster82	0.0015	0.0005	0.017	-0.0348	0.5736	0.9073675	Both
Longitude	nirk_Cluster83	0.004	-0.0023	0.2094	0.1678	0.037	0.4099058	Both
Longitude	nirk_Cluster84	0.0207	0.0021	0.0076	-0.0446	0.7068	0.9329682	Both
Longitude	nirk_Cluster85	0.004	-0.0041	0.5523	0.5287	0.0001	0.0044622	Both
Longitude	nirk_Cluster86	0.0286	-0.0045	0.0264	-0.0248	0.4815	0.9073675	Both
Longitude	nirk_Cluster87	0.0115	0.0008	0.0043	-0.0481	0.7779	0.9621867	Both
Longitude	nirk_Cluster88	0.0032	-0.0007	0.0289	-0.0222	0.4615	0.9073675	Both
Longitude	nirk_Cluster89	0.0065	0.0009	0.0099	-0.0422	0.6677	0.9119159	Both
Longitude	nirk_Cluster9	0.0517	-0.0045	0.0132	-0.0387	0.6193	0.9073675	Both
Longitude	nirk_Cluster90	0.0067	0.0027	0.0729	0.0241	0.2366	0.883558	Both
Longitude	nirk_Cluster91	0.0374	0.0008	0.0008	-0.0518	0.9019	0.9889693	Both
Longitude	nirk_Cluster92	0.0072	0.0018	0.0346	-0.0163	0.4198	0.9073675	Both
Longitude	nirk_Cluster93	0.0308	-0.0053	0.0527	0.0029	0.3167	0.9073675	Both
Longitude	nirk_Cluster94	0.0175	-0.0058	0.1184	0.0719	0.1268	0.7053789	Both
Longitude	nirk_Cluster95	0.0473	0.0077	0.0349	-0.0159	0.4177	0.9073675	Both
Longitude	nirk_Cluster96	0.0472	1.00E-04	0.00E+00	-0.0526	0.9935	0.9960176	Both
Longitude	nirk_Cluster97	0.0306	0.0105	0.1958	0.1535	0.0446	0.4456887	Both
Longitude	nirk_Cluster98	0.0181	-0.0046	0.0453	-0.0049	0.3542	0.9073675	Both
Longitude	nirk_Cluster99	0.006	-0.0026	0.1	0.0526	0.1626	0.7555319	Both
Longitude	nirs_Cluster0	0.1409	-0.0228	0.1119	0.0651	0.1384	0.7150828	Both
Longitude	nirs_Cluster1	0.004	-0.0008	0.0123	-0.0397	0.6328	0.9073675	Both
Longitude	nirs_Cluster14	0.0107	0.0032	0.0201	-0.0315	0.5404	0.9073675	Both
Longitude	nirs_Cluster2	0.0139	-0.0165	0.5197	0.4944	0.0002	0.0074376	Both
Longitude	nirs_Cluster26	0.0005	0.0002	0.0133	-0.0386	0.6186	0.9073675	Both
Longitude	nirs_Cluster28	0.0071	0.0017	0.0116	-0.0404	0.6415	0.9073675	Both
Longitude	nirs_Cluster3	0.1715	-0.0304	0.273	0.2348	0.0151	0.2225994	Both
Longitude	nirs_Cluster36	0.0013	-0.0021	0.1769	0.1336	0.0576	0.4743676	Both
Longitude	nirs_Cluster37	0.0066	0.0022	0.0125	-0.0395	0.6296	0.9073675	Both
Longitude	nirs_Cluster4	0.0002	1.00E-04	0.0063	-0.046	0.7317	0.9431455	Both
Longitude	nirs_Cluster50	0.002	0.00E+00	0.0001	-0.0525	0.9628	0.9889693	Both
Longitude	nirs_Cluster9	0.0269	-1.00E-04	0.00E+00	-0.0526	0.9952	0.9960176	Both
Longitude	norb_Cluster0	0.0775	0.0311	0.2631	0.2243	0.0174	0.2391126	Both
Longitude	norb_Cluster1	1.1042	0.436	0.1868	0.144	0.0504	0.4647098	Both
Longitude	norb_Cluster101	0.0026	-0.0007	0.0171	-0.0346	0.5717	0.9073675	Both
Longitude	norb_Cluster102	0.0038	0.0016	0.0824	0.0341	0.207	0.8481424	Both

Longitude	norb_Cluster103	0.0018	0.0005	0.0168	-0.0349	0.5754	0.9073675	Both
Longitude	norb_Cluster104	0.0023	0.0001	0.0009	-0.0517	0.896	0.9889693	Both
Longitude	norb_Cluster106	0.125	-0.0024	0.0006	-0.052	0.9132	0.9889693	Both
Longitude	norb_Cluster107	0.0133	-0.0035	0.0539	0.0041	0.3112	0.9073675	Both
Longitude	norb_Cluster108	0.0052	-0.0021	0.1163	0.0698	0.1303	0.7053789	Both
Longitude	norb_Cluster109	0.0227	-0.0058	0.07	0.021	0.2466	0.8871655	Both
Longitude	norb_Cluster11	0.0126	-0.0003	0.0004	-0.0522	0.9296	0.9889693	Both
Longitude	norb_Cluster110	0.0099	-0.0048	0.1579	0.1135	0.0745	0.5172457	Both
Longitude	norb_Cluster112	0.0171	-0.0034	0.0497	-0.0003	0.3314	0.9073675	Both
Longitude	norb_Cluster113	0.0134	-0.0004	0.0006	-0.052	0.9143	0.9889693	Both
Longitude	norb_Cluster114	0.0025	0.0008	0.0175	-0.0342	0.5679	0.9073675	Both
Longitude	norb_Cluster115	0.0137	0.0044	0.008	-0.0442	0.6995	0.925294	Both
Longitude	norb_Cluster116	0.0072	0.0026	0.0601	0.0106	0.2842	0.9073675	Both
Longitude	norb_Cluster117	0.0027	-0.0004	0.0085	-0.0437	0.6905	0.9175223	Both
Longitude	norb_Cluster118	0.0037	-0.0003	0.0026	-0.0499	0.8252	0.979654	Both
Longitude	norb_Cluster119	0.019	-0.0076	0.0949	0.0472	0.1743	0.7792279	Both
Longitude	norb_Cluster12	0.0047	0.0009	0.0149	-0.0369	0.5977	0.9073675	Both
Longitude	norb_Cluster121	0.0017	0.0001	0.0009	-0.0517	0.8957	0.9889693	Both
Longitude	norb_Cluster122	0.0144	-0.0002	0.0002	-0.0524	0.946	0.9889693	Both
Longitude	norb_Cluster123	0.0053	-0.0012	0.0246	-0.0267	0.497	0.9073675	Both
Longitude	norb_Cluster124	0.682	0.2518	0.207	0.1653	0.0382	0.4099058	Both
Longitude	norb_Cluster125	0.004	-0.0007	0.0195	-0.0321	0.5462	0.9073675	Both
Longitude	norb_Cluster126	0.0082	-0.0013	0.0172	-0.0346	0.5712	0.9073675	Both
Longitude	norb_Cluster127	0.1735	0.0402	0.0699	0.0209	0.2468	0.8871655	Both
Longitude	norb_Cluster128	0.0115	0.0033	0.0951	0.0475	0.1738	0.7792279	Both
Longitude	norb_Cluster13	0.0045	0.001	0.0172	-0.0345	0.5712	0.9073675	Both
Longitude	norb_Cluster131	0.0184	0.00E+00	0.00E+00	-0.0526	0.9936	0.9960176	Both
Longitude	norb_Cluster132	1.0357	-0.0261	0.0013	-0.0512	0.8751	0.9889693	Both
Longitude	norb_Cluster133	0.0018	-0.0014	0.1804	0.1372	0.055	0.4743676	Both
Longitude	norb_Cluster135	0.0567	0.0073	0.0218	-0.0297	0.5229	0.9073675	Both
Longitude	norb_Cluster136	0.0213	0.0002	0.0001	-0.0525	0.9624	0.9889693	Both
Longitude	norb_Cluster139	0.0328	-0.003	0.0114	-0.0407	0.6456	0.9073675	Both
Longitude	norb_Cluster14	0.0026	0.0005	0.0143	-0.0375	0.605	0.9073675	Both
Longitude	norb_Cluster140	0.0437	0.0126	0.1082	0.0613	0.1454	0.733168	Both
Longitude	norb_Cluster142	0.0015	0.0003	0.0069	-0.0454	0.7209	0.9369164	Both
Longitude	norb_Cluster144	0.0098	-0.0034	0.1004	0.0531	0.1616	0.7555319	Both
Longitude	norb_Cluster146	0.0348	0.0017	0.0035	-0.0489	0.7985	0.9697956	Both
Longitude	norb_Cluster15	0.0469	0.0156	0.1283	0.0824	0.1108	0.6539153	Both
Longitude	norb_Cluster150	0.0157	-0.0016	0.0134	-0.0385	0.6168	0.9073675	Both
Longitude	norb_Cluster155	0.1631	0.055	0.1905	0.1479	0.0479	0.4637206	Both
Longitude	norb_Cluster16	0.0131	0.0014	0.0064	-0.0459	0.7297	0.9431455	Both
Longitude	norb_Cluster164	0.0199	0.006	0.0695	0.0206	0.2481	0.8871655	Both
Longitude	norb_Cluster165	0.0076	0.0023	0.0414	-0.0091	0.3765	0.9073675	Both
Longitude	norb_Cluster166	0.0039	0.001	0.0257	-0.0256	0.4875	0.9073675	Both
Longitude	norb_Cluster169	0.0238	0.0048	0.0291	-0.0221	0.4601	0.9073675	Both
Longitude	norb_Cluster17	0.0028	0.0004	0.0091	-0.043	0.6801	0.9148537	Both
Longitude	norb_Cluster170	0.0243	0.0079	0.0654	0.0163	0.263	0.8998097	Both
Longitude	norb_Cluster171	0.0069	-0.0004	0.001	-0.0516	0.8916	0.9889693	Both
Longitude	norb_Cluster172	0.014	0.0036	0.0366	-0.0141	0.4059	0.9073675	Both
Longitude	norb_Cluster175	0.0458	0.0116	0.0392	-0.0113	0.3895	0.9073675	Both
Longitude	norb_Cluster176	0.0298	0.0051	0.0183	-0.0333	0.5583	0.9073675	Both
Longitude	norb_Cluster177	0.0282	0.0048	0.018	-0.0337	0.5621	0.9073675	Both
Longitude	norb_Cluster179	0.0022	-0.0021	0.2216	0.1806	0.0313	0.3690418	Both
Longitude	norb_Cluster18	0.0037	-1.00E-04	0.0004	-0.0522	0.9347	0.9889693	Both
Longitude	norb_Cluster180	0.0109	-0.0043	0.1755	0.1322	0.0587	0.4743676	Both

Longitude	norb_Cluster181	0.0008	0.0003	0.0114	-0.0407	0.6457	0.9073675	Both
Longitude	norb_Cluster182	0.0013	0.0003	0.0143	-0.0376	0.6054	0.9073675	Both
Longitude	norb_Cluster184	0.0146	0.0026	0.0256	-0.0257	0.4885	0.9073675	Both
Longitude	norb_Cluster186	0.0011	0.0006	0.0453	-0.005	0.3544	0.9073675	Both
Longitude	norb_Cluster19	0.0049	1.00E-04	0.0002	-0.0524	0.9529	0.9889693	Both
Longitude	norb_Cluster192	0.0173	0.0042	0.0402	-0.0103	0.3833	0.9073675	Both
Longitude	norb_Cluster193	0.0021	0.0004	0.0113	-0.0408	0.6467	0.9073675	Both
Longitude	norb_Cluster197	0.0303	0.0091	0.0137	-0.0382	0.6138	0.9073675	Both
Longitude	norb_Cluster2	0.0031	0.0006	0.0134	-0.0385	0.6172	0.9073675	Both
Longitude	norb_Cluster20	0.0587	0.0187	0.1537	0.1091	0.0788	0.5344693	Both
Longitude	norb_Cluster208	0.0011	0.0001	0.0021	-0.0505	0.8452	0.9827843	Both
Longitude	norb_Cluster21	0.0481	0.0157	0.1205	0.0743	0.1231	0.7053789	Both
Longitude	norb_Cluster211	0.0104	0.0012	0.0024	-0.0501	0.8344	0.9809646	Both
Longitude	norb_Cluster212	0.0264	0.0067	0.0196	-0.032	0.5447	0.9073675	Both
Longitude	norb_Cluster214	0.0119	0.0004	0.0003	-0.0523	0.9372	0.9889693	Both
Longitude	norb_Cluster217	0.0149	0.0027	0.0109	-0.0412	0.6526	0.9102259	Both
Longitude	norb_Cluster219	0.0083	0.0022	0.0164	-0.0354	0.5803	0.9073675	Both
Longitude	norb_Cluster22	0.0025	-0.0006	0.0137	-0.0383	0.6139	0.9073675	Both
Longitude	norb_Cluster220	0.0177	0.0048	0.0157	-0.0361	0.5884	0.9073675	Both
Longitude	norb_Cluster221	0.0177	0.0032	0.0062	-0.0461	0.7343	0.9439173	Both
Longitude	norb_Cluster222	0.0074	0.0023	0.0137	-0.0383	0.6139	0.9073675	Both
Longitude	norb_Cluster224	0.0194	0.0044	0.0168	-0.0349	0.5749	0.9073675	Both
Longitude	norb_Cluster225	0.0097	0.0003	0.0003	-0.0523	0.9432	0.9889693	Both
Longitude	norb_Cluster226	0.0043	-0.0004	0.0038	-0.0486	0.7905	0.9696436	Both
Longitude	norb_Cluster228	0.0031	0.0008	0.0054	-0.047	0.7523	0.95251	Both
Longitude	norb_Cluster229	0.0034	1.00E-04	0.0003	-0.0523	0.941	0.9889693	Both
Longitude	norb_Cluster230	0.0014	-0.0007	0.0456	-0.0046	0.3526	0.9073675	Both
Longitude	norb_Cluster231	0.0044	-0.0023	0.0712	0.0223	0.2424	0.8871655	Both
Longitude	norb_Cluster232	0.0031	-0.0013	0.047	-0.0032	0.3453	0.9073675	Both
Longitude	norb_Cluster234	0.0121	-0.0008	0.0016	-0.051	0.8643	0.9889693	Both
Longitude	norb_Cluster235	0.0009	-0.0002	0.0124	-0.0395	0.6302	0.9073675	Both
Longitude	norb_Cluster238	0.0099	-0.0057	0.1031	0.0559	0.1558	0.7397849	Both
Longitude	norb_Cluster239	0.0081	-0.0027	0.0289	-0.0223	0.4616	0.9073675	Both
Longitude	norb_Cluster24	0.2637	0.0852	0.1578	0.1135	0.0745	0.5172457	Both
Longitude	norb_Cluster244	0.0011	-0.0012	0.2561	0.217	0.0192	0.2580689	Both
Longitude	norb_Cluster247	0.0048	-0.0041	0.5691	0.5464	1.00E-04	0.003279	Both
Longitude	norb_Cluster25	0.1114	0.0431	0.1848	0.1419	0.0518	0.4669139	Both
Longitude	norb_Cluster250	0.0115	-0.0031	0.0505	0.0005	0.3276	0.9073675	Both
Longitude	norb_Cluster251	0.0052	-0.0014	0.0386	-0.012	0.3935	0.9073675	Both
Longitude	norb_Cluster26	0.0931	0.0368	0.1614	0.1173	0.071	0.5172457	Both
Longitude	norb_Cluster28	0.0302	0.0012	0.0017	-0.0509	0.8596	0.986755	Both
Longitude	norb_Cluster29	0.222	0.0388	0.1166	0.0702	0.1297	0.7053789	Both
Longitude	norb_Cluster31	0.0531	0.0167	0.1169	0.0704	0.1293	0.7053789	Both
Longitude	norb_Cluster32	0.1193	0.0516	0.1701	0.1264	0.0632	0.4970871	Both
Longitude	norb_Cluster33	0.4946	0.1964	0.5916	0.5701	0.00E+00	0.002467	Both
Longitude	norb_Cluster35	0.0628	0.0218	0.1485	0.1037	0.0845	0.5540719	Both
Longitude	norb_Cluster36	1.1345	0.576	0.4992	0.4728	0.0003	0.0101313	Both
Longitude	norb_Cluster37	0.7473	0.3373	0.6127	0.5923	0.00E+00	0.0017933	Both
Longitude	norb_Cluster38	0.0116	0.0012	0.0143	-0.0376	0.6061	0.9073675	Both
Longitude	norb_Cluster39	0.0426	0.0087	0.0487	-0.0014	0.3367	0.9073675	Both
Longitude	norb_Cluster40	0.186	0.0443	0.0764	0.0277	0.2253	0.865385	Both
Longitude	norb_Cluster42	0.2864	0.1414	0.5132	0.4876	0.0003	0.0080527	Both
Longitude	norb_Cluster44	0.0182	-0.0087	0.143	0.0979	0.0909	0.5707874	Both
Longitude	norb_Cluster46	0.2556	0.0602	0.0801	0.0317	0.2139	0.865385	Both
Longitude	norb_Cluster47	0.555	0.1791	0.1687	0.1249	0.0644	0.5000079	Both

Longitude	norb_Cluster5	0.01	0.0023	0.0729	0.0241	0.2366	0.883558	Both
Longitude	norb_Cluster51	0.0092	0.0002	0.0001	-0.0525	0.9638	0.9889693	Both
Longitude	norb_Cluster52	0.2391	0.0779	0.1368	0.0914	0.0989	0.6012544	Both
Longitude	norb_Cluster56	0.014	0.0003	0.0006	-0.052	0.9141	0.9889693	Both
Longitude	norb_Cluster57	0.0034	0.0004	0.0073	-0.045	0.7133	0.9331847	Both
Longitude	norb_Cluster59	0.0236	-0.0184	0.3013	0.2645	0.01	0.1681342	Both
Longitude	norb_Cluster6	0.6862	0.3356	0.4909	0.4641	0.0004	0.0113494	Both
Longitude	norb_Cluster60	0.3199	0.1542	0.1842	0.1412	0.0522	0.4669139	Both
Longitude	norb_Cluster61	0.482	0.0427	0.0105	-0.0415	0.658	0.9114297	Both
Longitude	norb_Cluster62	0.0024	-0.0002	0.002	-0.0505	0.8479	0.9827843	Both
Longitude	norb_Cluster64	0.0326	-0.0025	0.0091	-0.043	0.6807	0.9148537	Both
Longitude	norb_Cluster66	0.0012	0.00E+00	0.00E+00	-0.0526	0.9951	0.9960176	Both
Longitude	norb_Cluster67	0.0218	-0.0091	0.1711	0.1275	0.0623	0.4970254	Both
Longitude	norb_Cluster68	0.0034	0.0005	0.0154	-0.0364	0.5916	0.9073675	Both
Longitude	norb_Cluster69	0.0028	0.0003	0.0056	-0.0467	0.747	0.9518723	Both
Longitude	norb_Cluster7	0.0709	0.0217	0.1342	0.0886	0.1025	0.6168925	Both
Longitude	norb_Cluster70	0.1051	0.0006	0.00E+00	-0.0526	0.976	0.9938755	Both
Longitude	norb_Cluster71	0.1295	0.0002	0.00E+00	-0.0526	0.9926	0.9960176	Both
Longitude	norb_Cluster74	0.0327	-0.0013	0.0023	-0.0502	0.835	0.9809646	Both
Longitude	norb_Cluster75	0.1291	0.0011	0.0002	-0.0524	0.953	0.9889693	Both
Longitude	norb_Cluster76	0.1105	0.048	0.1126	0.0659	0.1371	0.7150828	Both
Longitude	norb_Cluster77	0.0475	0.0017	0.0033	-0.0492	0.8048	0.9705301	Both
Longitude	norb_Cluster8	0.018	0.0026	0.0199	-0.0317	0.5421	0.9073675	Both
Longitude	norb_Cluster80	0.0015	0.0002	0.0034	-0.049	0.8016	0.9697956	Both
Longitude	norb_Cluster81	0.0012	0.0003	0.018	-0.0336	0.5617	0.9073675	Both
Longitude	norb_Cluster83	0.0078	-0.0011	0.0045	-0.0479	0.7733	0.9621867	Both
Longitude	norb_Cluster84	0.0082	-0.0008	0.0071	-0.0452	0.7173	0.9342816	Both
Longitude	norb_Cluster85	0.0023	0.0009	0.0494	-0.0006	0.3327	0.9073675	Both
Longitude	norb_Cluster86	0.031	0.0095	0.0263	-0.0249	0.4821	0.9073675	Both
Longitude	norb_Cluster88	0.0016	0.0004	0.0124	-0.0396	0.6313	0.9073675	Both
Longitude	norb_Cluster90	0.0009	-0.0004	0.0185	-0.0332	0.557	0.9073675	Both
Longitude	norb_Cluster91	0.6481	0.1027	0.0299	-0.0211	0.4534	0.9073675	Both
Longitude	norb_Cluster92	0.0015	-0.0007	0.0283	-0.0228	0.4659	0.9073675	Both
Longitude	norb_Cluster93	0.0022	0.001	0.0384	-0.0123	0.3949	0.9073675	Both
Longitude	norb_Cluster94	0.0017	-0.0012	0.176	0.1326	0.0583	0.4743676	Both
Longitude	norb_Cluster96	0.0007	-0.0003	0.0261	-0.0251	0.4839	0.9073675	Both
Longitude	norb_Cluster97	0.0119	-0.0086	0.2957	0.2586	0.0108	0.177503	Both
Longitude	norb_Cluster98	0.0132	0.0039	0.0396	-0.011	0.3872	0.9073675	Both
Longitude	nosz_Cluster0	0.0045	-0.0018	0.1035	0.0563	0.1551	0.7397849	Both
Longitude	nosz_Cluster10	0.0136	0.0026	0.0224	-0.029	0.5168	0.9073675	Both
Longitude	nosz_Cluster102	0.0044	-0.0025	0.302	0.2652	0.0099	0.1681342	Both
Longitude	nosz_Cluster103	0.0455	0.0059	0.0304	-0.0207	0.45	0.9073675	Both
Longitude	nosz_Cluster105	0.0016	-0.0004	0.025	-0.0263	0.4935	0.9073675	Both
Longitude	nosz_Cluster106	0.0073	0.001	0.013	-0.039	0.6231	0.9073675	Both
Longitude	nosz_Cluster107	0.0002	1.00E-04	0.0063	-0.046	0.7317	0.9431455	Both
Longitude	nosz_Cluster108	0.004	0.0007	0.0161	-0.0357	0.5842	0.9073675	Both
Longitude	nosz_Cluster109	0.0375	-0.0008	0.0011	-0.0514	0.8846	0.9889693	Both
Longitude	nosz_Cluster11	0.0129	-0.0015	0.0036	-0.0488	0.7959	0.9697956	Both
Longitude	nosz_Cluster110	0.0003	0.0002	0.0323	-0.0186	0.4357	0.9073675	Both
Longitude	nosz_Cluster111	0.0061	-0.0004	0.0024	-0.0501	0.8316	0.9809646	Both
Longitude	nosz_Cluster116	0.0067	-0.0011	0.02	-0.0316	0.5412	0.9073675	Both
Longitude	nosz_Cluster118	0.0045	0.00E+00	0.00E+00	-0.0526	0.9888	0.9960176	Both
Longitude	nosz_Cluster119	0.0013	0.00E+00	0.00E+00	-0.0526	0.9945	0.9960176	Both
Longitude	nosz_Cluster120	0.0071	-0.0013	0.027	-0.0242	0.4767	0.9073675	Both
Longitude	nosz_Cluster123	0.0097	-0.0017	0.0163	-0.0354	0.581	0.9073675	Both

Longitude	nosz_Cluster128	0.0047	0.0015	0.0405	-0.01	0.3818	0.9073675	Both
Longitude	nosz_Cluster129	0.0494	0.0115	0.2423	0.2024	0.0234	0.293909	Both
Longitude	nosz_Cluster13	0.0115	-0.0002	0.0002	-0.0524	0.9541	0.9889693	Both
Longitude	nosz_Cluster130	0.089	0.0248	0.0165	-0.0353	0.5792	0.9073675	Both
Longitude	nosz_Cluster132	0.0037	0.0001	0.0011	-0.0515	0.8871	0.9889693	Both
Longitude	nosz_Cluster14	0.024	0.0031	0.0027	-0.0498	0.8223	0.979654	Both
Longitude	nosz_Cluster16	0.0076	0.0007	0.0027	-0.0498	0.823	0.979654	Both
Longitude	nosz_Cluster17	0.0364	0.0009	0.0008	-0.0518	0.9045	0.9889693	Both
Longitude	nosz_Cluster18	0.0161	-0.0046	0.0408	-0.0097	0.3798	0.9073675	Both
Longitude	nosz_Cluster20	0.0091	0.001	0.005	-0.0474	0.7614	0.9592071	Both
Longitude	nosz_Cluster21	0.0083	0.0003	0.0006	-0.052	0.9184	0.9889693	Both
Longitude	nosz_Cluster22	0.0267	0.0039	0.0088	-0.0433	0.6854	0.9149597	Both
Longitude	nosz_Cluster23	0.0141	-0.0006	0.0009	-0.0517	0.8984	0.9889693	Both
Longitude	nosz_Cluster24	0.0013	-0.001	0.1039	0.0568	0.154	0.7397849	Both
Longitude	nosz_Cluster25	0.0029	-0.0005	0.0084	-0.0437	0.692	0.9175223	Both
Longitude	nosz_Cluster26	0.0054	0.0005	0.0031	-0.0494	0.8118	0.9734815	Both
Longitude	nosz_Cluster27	0.0053	-0.0018	0.0666	0.0175	0.2586	0.8923808	Both
Longitude	nosz_Cluster28	0.0303	0.001	0.001	-0.0516	0.8928	0.9889693	Both
Longitude	nosz_Cluster30	0.0276	-0.0049	0.0301	-0.021	0.4521	0.9073675	Both
Longitude	nosz_Cluster32	0.0137	-0.003	0.0387	-0.0119	0.3925	0.9073675	Both
Longitude	nosz_Cluster33	0.018	0.003	0.0121	-0.0399	0.6345	0.9073675	Both
Longitude	nosz_Cluster34	0.0377	0.0102	0.0369	-0.0138	0.4043	0.9073675	Both
Longitude	nosz_Cluster35	0.0075	-0.0012	0.0157	-0.0361	0.588	0.9073675	Both
Longitude	nosz_Cluster36	0.0179	0.0021	0.01	-0.0421	0.6658	0.9114297	Both
Longitude	nosz_Cluster37	0.006	-0.002	0.0561	0.0064	0.3014	0.9073675	Both
Longitude	nosz_Cluster38	0.0128	-0.0031	0.0312	-0.0198	0.4436	0.9073675	Both
Longitude	nosz_Cluster39	0.0537	-0.0111	0.0442	-0.0061	0.3605	0.9073675	Both
Longitude	nosz_Cluster40	0.0218	-0.0067	0.0667	0.0176	0.2584	0.8923808	Both
Longitude	nosz_Cluster41	0.0203	-0.0055	0.0499	-0.0001	0.3305	0.9073675	Both
Longitude	nosz_Cluster42	0.0279	0.0016	0.0023	-0.0502	0.8363	0.9809646	Both
Longitude	nosz_Cluster43	0.0007	1.00E-04	0.0006	-0.0519	0.9127	0.9889693	Both
Longitude	nosz_Cluster44	0.0057	0.001	0.0176	-0.0341	0.5661	0.9073675	Both
Longitude	nosz_Cluster45	0.0045	0.00E+00	1.00E-04	-0.0526	0.9757	0.9938755	Both
Longitude	nosz_Cluster46	0.007	-0.0005	0.0051	-0.0473	0.7585	0.9582371	Both
Longitude	nosz_Cluster47	0.0008	0.0002	0.0138	-0.0381	0.6125	0.9073675	Both
Longitude	nosz_Cluster48	0.0023	-0.0001	0.0007	-0.0519	0.9087	0.9889693	Both
Longitude	nosz_Cluster49	0.0014	-0.0004	0.0189	-0.0328	0.5528	0.9073675	Both
Longitude	nosz_Cluster5	0.0045	0.0009	0.0111	-0.0409	0.649	0.9073675	Both
Longitude	nosz_Cluster51	0.0021	0.0002	0.0031	-0.0494	0.8115	0.9734815	Both
Longitude	nosz_Cluster53	0.0009	-0.0006	0.0483	-0.0018	0.3386	0.9073675	Both
Longitude	nosz_Cluster54	0.0026	0.0005	0.0112	-0.0408	0.6481	0.9073675	Both
Longitude	nosz_Cluster57	0.1757	0.0269	0.0224	-0.029	0.5172	0.9073675	Both
Longitude	nosz_Cluster58	0.0043	0.0007	0.0104	-0.0416	0.6593	0.9114297	Both
Longitude	nosz_Cluster59	0.0009	1.00E-04	0.0004	-0.0523	0.9354	0.9889693	Both
Longitude	nosz_Cluster6	0.0032	0.0002	0.0012	-0.0513	0.8804	0.9889693	Both
Longitude	nosz_Cluster60	0.0079	-0.0031	0.0848	0.0366	0.2003	0.8320722	Both
Longitude	nosz_Cluster61	0.0178	0.0027	0.0492	-0.0009	0.334	0.9073675	Both
Longitude	nosz_Cluster62	0.0413	0.0071	0.0285	-0.0226	0.4642	0.9073675	Both
Longitude	nosz_Cluster64	0.0016	-0.0021	0.1591	0.1148	0.0733	0.5172457	Both
Longitude	nosz_Cluster65	0.0065	-0.0011	0.0138	-0.0381	0.6122	0.9073675	Both
Longitude	nosz_Cluster66	0.0324	0.0093	0.0523	0.0024	0.3188	0.9073675	Both
Longitude	nosz_Cluster67	0.0521	0.0091	0.021	-0.0306	0.5312	0.9073675	Both
Longitude	nosz_Cluster69	0.0061	0.0016	0.0401	-0.0104	0.384	0.9073675	Both
Longitude	nosz_Cluster70	0.0479	0.0061	0.0043	-0.0481	0.7769	0.9621867	Both
Longitude	nosz_Cluster71	0.0083	0.0002	0.0001	-0.0525	0.9591	0.9889693	Both

Longitude	nosz_Cluster72	0.0506	0.0099	0.0303	-0.0207	0.4503	0.9073675	Both
Longitude	nosz_Cluster73	0.003	1.00E-04	0.0003	-0.0524	0.9444	0.9889693	Both
Longitude	nosz_Cluster74	0.0017	0.0003	0.0073	-0.045	0.7128	0.9331847	Both
Longitude	nosz_Cluster75	0.0967	0.025	0.01	-0.0421	0.6656	0.9114297	Both
Longitude	nosz_Cluster77	0.0002	0.0001	0.0323	-0.0186	0.4357	0.9073675	Both
Longitude	nosz_Cluster78	0.0044	0.0009	0.0287	-0.0224	0.463	0.9073675	Both
Longitude	nosz_Cluster79	0.0333	-0.0056	0.0356	-0.0152	0.413	0.9073675	Both
Longitude	nosz_Cluster8	0.0099	-0.0002	0.0004	-0.0522	0.9288	0.9889693	Both
Longitude	nosz_Cluster80	0.0045	0.0008	0.0086	-0.0435	0.6886	0.9170765	Both
Longitude	nosz_Cluster81	0.0079	0.001	0.0044	-0.048	0.775	0.9621867	Both
Longitude	nosz_Cluster82	0.0016	-0.0033	0.3126	0.2764	0.0084	0.1504138	Both
Longitude	nosz_Cluster83	0.0015	0.0003	0.0058	-0.0465	0.742	0.9475533	Both
Longitude	nosz_Cluster85	0.0022	-0.0022	0.2162	0.175	0.0337	0.3894985	Both
Longitude	nosz_Cluster86	0.0027	-0.0022	0.197	0.1547	0.0439	0.4456887	Both
Longitude	nosz_Cluster87	0.003	-0.0013	0.0985	0.0511	0.1658	0.7588351	Both
Longitude	nosz_Cluster88	0.0218	0.003	0.0417	-0.0087	0.3746	0.9073675	Both
Longitude	nosz_Cluster89	0.0466	0.0098	0.0346	-0.0162	0.4192	0.9073675	Both
Longitude	nosz_Cluster9	0.003	-0.0002	0.0021	-0.0505	0.8448	0.9827843	Both
Longitude	nosz_Cluster90	0.0116	-0.0006	0.0035	-0.049	0.7991	0.9697956	Both
Longitude	nosz_Cluster91	0.0189	0.0005	0.0003	-0.0523	0.9363	0.9889693	Both
Longitude	nosz_Cluster93	0.0393	-0.0026	0.0044	-0.048	0.774	0.9621867	Both
Longitude	nosz_Cluster95	0.007	0.0009	0.0104	-0.0417	0.6601	0.9114297	Both
Longitude	nosz_Cluster96	0.0066	-0.0009	0.0193	-0.0324	0.5486	0.9073675	Both
Longitude	nosz_Cluster97	0.0019	-0.0003	0.0063	-0.046	0.7321	0.9431455	Both
Longitude	nosz_Cluster98	0.0025	-0.0045	0.2651	0.2264	0.0169	0.2377955	Both
Longitude	nosz_Cluster99	0.0055	-0.0007	0.0155	-0.0363	0.5911	0.9073675	Both
Longitude	nrfa_Cluster10	0.0804	-0.0036	0.0013	-0.0513	0.8774	0.9889693	Both
Longitude	nrfa_Cluster100	0.0963	-0.0074	0.0027	-0.0498	0.8221	0.979654	Both
Longitude	nrfa_Cluster104	0.1143	-0.0351	0.0985	0.0511	0.1659	0.7588351	Both
Longitude	nrfa_Cluster105	0.0062	0.00E+00	0.00E+00	-0.0526	0.996	0.9960176	Both
Longitude	nrfa_Cluster109	0.2294	-0.0975	0.227	0.1863	0.029	0.349193	Both
Longitude	nrfa_Cluster116	0.2592	-0.0749	0.1603	0.1161	0.0721	0.5172457	Both
Longitude	nrfa_Cluster118	0.173	-0.0145	0.0027	-0.0498	0.8244	0.979654	Both
Longitude	nrfa_Cluster125	0.0809	-0.0083	0.0035	-0.0489	0.7977	0.9697956	Both
Longitude	nrfa_Cluster154	0.0658	-0.0098	0.0111	-0.0409	0.649	0.9073675	Both
Longitude	nrfa_Cluster164	0.0045	0.0004	0.0025	-0.05	0.8292	0.9809646	Both
Longitude	nrfa_Cluster172	0.0026	0.0006	0.0117	-0.0403	0.6404	0.9073675	Both
Longitude	nrfa_Cluster175	0.0044	1.00E-04	1.00E-04	-0.0526	0.9704	0.9930474	Both
Longitude	nrfa_Cluster20	0.035	-0.0173	0.1509	0.1062	0.0818	0.5484298	Both
Longitude	nrfa_Cluster26	0.1078	-0.0052	0.001	-0.0516	0.8942	0.9889693	Both
Longitude	nrfa_Cluster29	0.0048	0.0022	0.0754	0.0267	0.2285	0.8697366	Both
Longitude	nrfa_Cluster34	0.0487	-0.0217	0.1198	0.0734	0.1244	0.7053789	Both
Longitude	nrfa_Cluster38	0.0031	0.0003	0.0034	-0.0491	0.8021	0.9697956	Both
Longitude	nrfa_Cluster61	0.0036	-0.0021	0.117	0.0705	0.1291	0.7053789	Both
Longitude	nrfa_Cluster62	0.0452	-0.0037	0.0024	-0.0501	0.8336	0.9809646	Both
Longitude	nrfa_Cluster66	0.1082	-0.0248	0.0779	0.0294	0.2204	0.865385	Both
Longitude	nrfa_Cluster69	0.003	0.0011	0.0503	0.0003	0.3286	0.9073675	Both
Longitude	nrfa_Cluster71	0.027	-0.0043	0.0117	-0.0403	0.64	0.9073675	Both
Longitude	nrfa_Cluster72	0.0732	-0.0162	0.0267	-0.0245	0.4789	0.9073675	Both
Longitude	nrfa_Cluster73	0.0038	0.0007	0.0105	-0.0416	0.6581	0.9114297	Both
Longitude	nrfa_Cluster74	0.1331	-0.0061	0.0012	-0.0514	0.8808	0.9889693	Both
Longitude	nrfa_Cluster76	0.0755	-0.0007	0.00E+00	-0.0526	0.977	0.9938755	Both
Longitude	nrfa_Cluster79	0.0229	0.007	0.0124	-0.0396	0.631	0.9073675	Both
Longitude	nrfa_Cluster87	0.017	0.0048	0.0231	-0.0283	0.5109	0.9073675	Both
Longitude	nrfa_Cluster89	0.0018	0.0004	0.0093	-0.0428	0.6776	0.9148337	Both

Longitude	nrfa_Cluster92	0.0043	0.0012	0.0266	-0.0246	0.4795	0.9073675	Both
Longitude	nrfa_Cluster93	0.0428	0.0165	0.043	-0.0073	0.3669	0.9073675	Both
Longitude	nrfa_Cluster94	0.0002	0.00E+00	0.0003	-0.0523	0.9432	0.9889693	Both
Longitude	nrfa_Cluster97	0.0168	0.0003	0.0002	-0.0525	0.9558	0.9889693	Both
Longitude	nrfa_Cluster99	0.014	-0.0004	0.0005	-0.0521	0.9247	0.9889693	Both
Elevation	amoA_A_Cluster26	0.004	-0.0033	0.1933	0.1508	0.0461	0.4268248	Both
Elevation	amoA_A_Cluster45	0.0051	-0.0022	0.067	0.0179	0.2572	0.7716014	Both
Elevation	amoA_B_Cluster0	0.1099	0.0004	1.00E-04	-0.0526	0.9747	0.9918486	Both
Elevation	amoA_B_Cluster1	0.0172	0.011	0.4156	0.3848	0.0016	0.0474062	Both
Elevation	amoA_B_Cluster10	0.0422	0.0017	0.0032	-0.0492	0.8068	0.9671747	Both
Elevation	amoA_B_Cluster11	0.0377	0.0209	0.4239	0.3936	0.0014	0.0440902	Both
Elevation	amoA_B_Cluster12	0.0807	0.0056	0.0172	-0.0345	0.5705	0.9215392	Both
Elevation	amoA_B_Cluster13	0.5271	-0.0084	0.0004	-0.0522	0.9287	0.990798	Both
Elevation	amoA_B_Cluster14	0.0275	-0.0051	0.0314	-0.0196	0.4426	0.8600141	Both
Elevation	amoA_B_Cluster15	0.0318	0.0096	0.1191	0.0728	0.1254	0.621871	Both
Elevation	amoA_B_Cluster2	0.1721	0.0196	0.0211	-0.0305	0.5302	0.9009061	Both
Elevation	amoA_B_Cluster20	0.0058	0.0041	0.2065	0.1647	0.0385	0.4222987	Both
Elevation	amoA_B_Cluster23	0.0507	0.0208	0.3514	0.3173	0.0046	0.1085681	Both
Elevation	amoA_B_Cluster3	0.0182	0.0129	0.4231	0.3927	0.0014	0.0440902	Both
Elevation	amoA_B_Cluster4	0.0125	0.003	0.0408	-0.0097	0.3799	0.8471095	Both
Elevation	amoA_B_Cluster5	0.0928	0.0058	0.0145	-0.0373	0.6029	0.9225412	Both
Elevation	amoA_B_Cluster6	0.0233	0.0075	0.1936	0.1511	0.046	0.4268248	Both
Elevation	amoA_B_Cluster7	0.0179	0.0121	0.382	0.3495	0.0028	0.0757918	Both
Elevation	amoA_B_Cluster8	0.0207	0.0172	0.5577	0.5344	0.0001	0.0066019	Both
Elevation	amoA_B_Cluster9	0.0549	0.0294	0.3242	0.2887	0.0071	0.1486345	Both
Elevation	nifh_Cluster0	0.0332	0.0036	0.0517	0.0018	0.3214	0.7958634	Both
Elevation	nifh_Cluster10	0.005	-0.0004	0.0017	-0.0509	0.8599	0.9737377	Both
Elevation	nifh_Cluster100	0.0019	-0.0006	0.0303	-0.0207	0.4501	0.8600141	Both
Elevation	nifh_Cluster103	0.0383	0.0014	0.0028	-0.0497	0.8212	0.9671747	Both
Elevation	nifh_Cluster1034	0.0262	-0.0021	0.0049	-0.0475	0.7626	0.9671747	Both
Elevation	nifh_Cluster105	0.013	-0.0033	0.0464	-0.0038	0.3483	0.8188088	Both
Elevation	nifh_Cluster108	0.0083	-0.0032	0.1364	0.0909	0.0995	0.6044683	Both
Elevation	nifh_Cluster1099	0.066	-0.0008	0.0002	-0.0525	0.9565	0.9918486	Both
Elevation	nifh_Cluster11	0.0021	-0.0006	0.0423	-0.0081	0.3711	0.845474	Both
Elevation	nifh_Cluster1112	0.0029	0.0011	0.053	0.0032	0.3152	0.7958634	Both
Elevation	nifh_Cluster112	0.0112	-0.002	0.029	-0.0221	0.4606	0.8600141	Both
Elevation	nifh_Cluster113	0.0327	0.0023	0.0129	-0.039	0.6239	0.9225412	Both
Elevation	nifh_Cluster114	0.0332	-0.001	0.0051	-0.0472	0.7573	0.9671747	Both
Elevation	nifh_Cluster1141	0.0251	0.0005	0.0005	-0.0521	0.9252	0.990798	Both
Elevation	nifh_Cluster115	0.0095	0.0005	0.0035	-0.049	0.7992	0.9671747	Both
Elevation	nifh_Cluster116	0.0775	0.0343	0.5227	0.4976	0.0002	0.0114378	Both
Elevation	nifh_Cluster1163	0.0044	0.002	0.1752	0.1318	0.059	0.463981	Both
Elevation	nifh_Cluster1179	0.0031	-0.0011	0.0452	-0.005	0.3547	0.8238634	Both
Elevation	nifh_Cluster1182	0.0204	0.00E+00	0.00E+00	-0.0526	0.9861	0.9947909	Both
Elevation	nifh_Cluster1183	0.0066	0.00E+00	0.00E+00	-0.0526	0.9794	0.9929059	Both
Elevation	nifh_Cluster1197	0.0115	0.0004	0.002	-0.0506	0.8493	0.9737377	Both
Elevation	nifh_Cluster1199	0.0046	0.0014	0.0718	0.0229	0.2404	0.7716014	Both
Elevation	nifh_Cluster120	0.0149	-0.0029	0.0108	-0.0413	0.6544	0.9348377	Both
Elevation	nifh_Cluster1203	0.0155	0.0065	0.1075	0.0605	0.1469	0.670426	Both
Elevation	nifh_Cluster1206	0.0043	0.0037	0.4497	0.4207	0.0009	0.0370184	Both
Elevation	nifh_Cluster1207	0.0074	-0.0011	0.0134	-0.0385	0.6172	0.9225412	Both
Elevation	nifh_Cluster1209	0.1506	0.0362	0.4246	0.3943	0.0014	0.0440902	Both
Elevation	nifh_Cluster121	0.0095	-0.0045	0.0695	0.0205	0.2482	0.7716014	Both
Elevation	nifh_Cluster1211	0.0056	0.005	0.6469	0.6283	0.00E+00	0.001312	Both
Elevation	nifh_Cluster1213	0.0026	0.0002	0.0029	-0.0496	0.8167	0.9671747	Both

Elevation	nifh_Cluster122	0.0029	-0.0014	0.0771	0.0285	0.2231	0.7608685	Both
Elevation	nifh_Cluster123	0.0095	-0.0026	0.0234	-0.028	0.5078	0.8785119	Both
Elevation	nifh_Cluster1236	0.0234	-0.0094	0.0651	0.0159	0.2642	0.7795232	Both
Elevation	nifh_Cluster1238	0.0028	-0.0014	0.0872	0.0391	0.1939	0.7331654	Both
Elevation	nifh_Cluster124	0.0091	0.0013	0.0227	-0.0288	0.5148	0.8855249	Both
Elevation	nifh_Cluster1261	0.002	-0.0002	0.0032	-0.0493	0.8077	0.9671747	Both
Elevation	nifh_Cluster1262	0.0065	-0.0024	0.0756	0.0269	0.2278	0.7636069	Both
Elevation	nifh_Cluster1266	0.0094	-0.0044	0.1908	0.1482	0.0477	0.4268248	Both
Elevation	nifh_Cluster1267	0.0562	-0.0078	0.0164	-0.0354	0.5803	0.9215392	Both
Elevation	nifh_Cluster127	0.0026	-0.0009	0.0312	-0.0198	0.4436	0.8600141	Both
Elevation	nifh_Cluster1278	0.1602	0.0566	0.1862	0.1434	0.0508	0.4329593	Both
Elevation	nifh_Cluster128	0.0036	-0.0012	0.0534	0.0036	0.3135	0.7958634	Both
Elevation	nifh_Cluster1289	0.0039	-0.001	0.0417	-0.0087	0.3745	0.8456325	Both
Elevation	nifh_Cluster129	0.0077	-0.002	0.0268	-0.0244	0.4779	0.8627904	Both
Elevation	nifh_Cluster1292	0.0086	0.0028	0.0762	0.0276	0.2258	0.7636069	Both
Elevation	nifh_Cluster130	0.0059	-0.002	0.0277	-0.0235	0.4708	0.8600178	Both
Elevation	nifh_Cluster1305	0.0048	0.0003	0.0046	-0.0478	0.7712	0.9671747	Both
Elevation	nifh_Cluster1306	0.005	0.0003	0.002	-0.0505	0.8461	0.9737377	Both
Elevation	nifh_Cluster131	0.0051	-0.0017	0.0377	-0.0129	0.3988	0.8554428	Both
Elevation	nifh_Cluster1319	0.0036	0.00E+00	1.00E-04	-0.0526	0.975	0.9918486	Both
Elevation	nifh_Cluster132	0.0064	-0.0018	0.0348	-0.016	0.4182	0.8600141	Both
Elevation	nifh_Cluster1320	0.0025	0.0012	0.0599	0.0104	0.2851	0.7870206	Both
Elevation	nifh_Cluster1323	0.0023	0.0011	0.1542	0.1097	0.0782	0.5185105	Both
Elevation	nifh_Cluster1324	0.03	-0.0139	0.2584	0.2194	0.0186	0.2746392	Both
Elevation	nifh_Cluster1336	0.0153	0.0029	0.1342	0.0886	0.1025	0.6044683	Both
Elevation	nifh_Cluster1340	0.0363	0.0449	0.7872	0.776	0.00E+00	3.64E-05	Both
Elevation	nifh_Cluster1345	0.0483	-0.0033	0.0038	-0.0486	0.7899	0.9671747	Both
Elevation	nifh_Cluster1370	0.0021	-0.0001	0.0018	-0.0508	0.8559	0.9737377	Both
Elevation	nifh_Cluster1375	0.0018	-0.0003	0.0111	-0.0409	0.649	0.9331749	Both
Elevation	nifh_Cluster139	0.0041	0.0022	0.1267	0.0807	0.1133	0.6057242	Both
Elevation	nifh_Cluster140	0.0043	-1.00E-04	0.0001	-0.0525	0.9629	0.9918486	Both
Elevation	nifh_Cluster141	0.0677	0.0019	0.0017	-0.0508	0.8578	0.9737377	Both
Elevation	nifh_Cluster148	0.0046	0.0038	0.2026	0.1606	0.0406	0.4222987	Both
Elevation	nifh_Cluster1480	0.001	0.00E+00	0.0001	-0.0525	0.9606	0.9918486	Both
Elevation	nifh_Cluster152	0.0599	-0.0089	0.0697	0.0208	0.2473	0.7716014	Both
Elevation	nifh_Cluster156	0.0659	-0.0178	0.129	0.0832	0.1098	0.6057242	Both
Elevation	nifh_Cluster1562	0.0543	-0.0095	0.0207	-0.0309	0.534	0.9009061	Both
Elevation	nifh_Cluster158	0.0144	0.0019	0.012	-0.04	0.6359	0.9287205	Both
Elevation	nifh_Cluster16	0.0019	-0.0009	0.0523	0.0024	0.3188	0.7958634	Both
Elevation	nifh_Cluster166	0.0172	-0.0036	0.0135	-0.0384	0.6154	0.9225412	Both
Elevation	nifh_Cluster205	0.0122	0.0001	0.0001	-0.0525	0.9651	0.9918486	Both
Elevation	nifh_Cluster21	0.0021	-0.0003	0.0073	-0.0449	0.7123	0.9660924	Both
Elevation	nifh_Cluster222	0.006	-0.0013	0.0136	-0.0383	0.6147	0.9225412	Both
Elevation	nifh_Cluster225	0.0189	0.0029	0.0527	0.0028	0.3169	0.7958634	Both
Elevation	nifh_Cluster230	0.0042	-0.0012	0.0515	0.0016	0.3224	0.7958634	Both
Elevation	nifh_Cluster231	0.0022	0.0006	0.0259	-0.0254	0.4862	0.8635407	Both
Elevation	nifh_Cluster236	0.0027	-0.0004	0.0104	-0.0417	0.6605	0.9413387	Both
Elevation	nifh_Cluster237	0.0058	-0.0028	0.1927	0.1502	0.0465	0.4268248	Both
Elevation	nifh_Cluster243	0.0333	-0.0144	0.1915	0.149	0.0473	0.4268248	Both
Elevation	nifh_Cluster2432	0.0436	-0.0022	0.0025	-0.05	0.8299	0.9687264	Both
Elevation	nifh_Cluster2433	0.0032	0.0013	0.0516	0.0017	0.322	0.7958634	Both
Elevation	nifh_Cluster246	0.0057	-0.0008	0.0096	-0.0425	0.6723	0.9512766	Both
Elevation	nifh_Cluster25	0.0295	-0.0051	0.0501	1.00E-04	0.3295	0.7972737	Both
Elevation	nifh_Cluster256	0.0029	-0.0009	0.0484	-0.0017	0.3379	0.8077917	Both
Elevation	nifh_Cluster264	0.0029	-0.0006	0.0292	-0.0219	0.4588	0.8600141	Both

Elevation	nifh_Cluster265	0.0736	-0.002	0.0033	-0.0492	0.806	0.9671747	Both
Elevation	nifh_Cluster267	0.0109	-0.0013	0.0099	-0.0422	0.6678	0.947175	Both
Elevation	nifh_Cluster268	0.0111	-0.001	0.0144	-0.0375	0.6045	0.9225412	Both
Elevation	nifh_Cluster269	0.023	0.00E+00	0.00E+00	-0.0526	0.9981	0.9993807	Both
Elevation	nifh_Cluster272	0.0041	-0.0009	0.0176	-0.0341	0.5664	0.9215392	Both
Elevation	nifh_Cluster274	0.0032	-0.0005	0.0025	-0.05	0.8301	0.9687264	Both
Elevation	nifh_Cluster275	0.0036	-0.0005	0.0119	-0.0402	0.6385	0.9287205	Both
Elevation	nifh_Cluster278	0.0077	0.0002	0.0003	-0.0523	0.9416	0.9914842	Both
Elevation	nifh_Cluster279	0.006	0.00E+00	0.00E+00	-0.0526	0.9899	0.9949387	Both
Elevation	nifh_Cluster28	0.002	0.00E+00	0.0002	-0.0524	0.9534	0.9918486	Both
Elevation	nifh_Cluster280	0.0031	0.0026	0.1804	0.1373	0.0549	0.4461049	Both
Elevation	nifh_Cluster282	0.0052	0.0009	0.0246	-0.0268	0.4975	0.869576	Both
Elevation	nifh_Cluster287	0.0022	-1.00E-04	0.0007	-0.0519	0.9099	0.9871445	Both
Elevation	nifh_Cluster290	0.011	-0.004	0.131	0.0853	0.1069	0.6057242	Both
Elevation	nifh_Cluster297	0.0029	-0.0017	0.0612	0.0118	0.2795	0.7870206	Both
Elevation	nifh_Cluster299	0.0026	-0.0009	0.0367	-0.014	0.4056	0.8554428	Both
Elevation	nifh_Cluster303	0.002	-0.0011	0.0968	0.0493	0.1697	0.7242057	Both
Elevation	nifh_Cluster304	0.0024	-0.0009	0.068	0.019	0.2534	0.7716014	Both
Elevation	nifh_Cluster313	0.0103	-0.0034	0.0379	-0.0128	0.3979	0.8554428	Both
Elevation	nifh_Cluster314	0.0022	-0.0006	0.024	-0.0274	0.5026	0.8722101	Both
Elevation	nifh_Cluster317	0.0071	-0.003	0.1371	0.0917	0.0984	0.6044683	Both
Elevation	nifh_Cluster32	0.0033	-0.0019	0.2185	0.1773	0.0326	0.3851629	Both
Elevation	nifh_Cluster320	12.7419	-0.5409	0.0297	-0.0214	0.4552	0.8600141	Both
Elevation	nifh_Cluster334	0.0063	-0.0008	0.0044	-0.048	0.7745	0.9671747	Both
Elevation	nifh_Cluster335	0.0075	-0.0011	0.0042	-0.0482	0.7794	0.9671747	Both
Elevation	nifh_Cluster338	0.0123	0.0033	0.083	0.0348	0.2052	0.7456773	Both
Elevation	nifh_Cluster340	0.007	0.0007	0.0012	-0.0513	0.8799	0.9849778	Both
Elevation	nifh_Cluster341	0.0023	-0.0016	0.1525	0.1079	0.0801	0.5250682	Both
Elevation	nifh_Cluster35	0.0078	0.0035	0.2386	0.1986	0.0246	0.3160521	Both
Elevation	nifh_Cluster351	0.0025	-0.0005	0.0085	-0.0437	0.6908	0.9577118	Both
Elevation	nifh_Cluster3574	0.1093	-0.0055	0.0082	-0.044	0.6963	0.9577118	Both
Elevation	nifh_Cluster3691	0.0174	0.0147	0.4247	0.3945	0.0014	0.0440902	Both
Elevation	nifh_Cluster37	0.0329	-0.0061	0.0268	-0.0244	0.4782	0.8627904	Both
Elevation	nifh_Cluster374	0.0021	0.0036	0.7782	0.7666	0.00E+00	3.64E-05	Both
Elevation	nifh_Cluster38	0.0009	-0.0003	0.0298	-0.0213	0.4547	0.8600141	Both
Elevation	nifh_Cluster382	0.0016	-0.0008	0.0287	-0.0224	0.4628	0.8600178	Both
Elevation	nifh_Cluster384	0.0019	-0.001	0.0872	0.0391	0.1938	0.7331654	Both
Elevation	nifh_Cluster386	0.0035	-0.0003	0.0027	-0.0498	0.8246	0.9681243	Both
Elevation	nifh_Cluster389	0.0031	-0.0005	0.0077	-0.0445	0.7051	0.9595565	Both
Elevation	nifh_Cluster394	0.002	-0.0004	0.0064	-0.0459	0.7309	0.9671747	Both
Elevation	nifh_Cluster40	0.0012	-0.0006	0.0683	0.0192	0.2526	0.7716014	Both
Elevation	nifh_Cluster43	0.0108	-0.0013	0.0242	-0.0271	0.5006	0.871262	Both
Elevation	nifh_Cluster434	0.0035	0.0014	0.0573	0.0077	0.2961	0.7870206	Both
Elevation	nifh_Cluster440	0.0133	0.0078	0.2059	0.1641	0.0388	0.4222987	Both
Elevation	nifh_Cluster462	0.0034	0.0008	0.0191	-0.0326	0.5506	0.9063091	Both
Elevation	nifh_Cluster470	0.0152	0.008	0.3493	0.315	0.0048	0.1085681	Both
Elevation	nifh_Cluster49	0.0213	-0.0087	0.0665	0.0174	0.2589	0.7716014	Both
Elevation	nifh_Cluster499	0.0026	-0.0009	0.05	0.00E+00	0.3297	0.7972737	Both
Elevation	nifh_Cluster5	0.0256	0.0051	0.1555	0.1111	0.0769	0.5155288	Both
Elevation	nifh_Cluster506	0.0048	0.0015	0.0602	0.0107	0.2837	0.7870206	Both
Elevation	nifh_Cluster51	0.0028	-0.0007	0.0167	-0.0351	0.5766	0.9215392	Both
Elevation	nifh_Cluster52	0.0336	-0.0134	0.0928	0.0451	0.1793	0.7266135	Both
Elevation	nifh_Cluster525	0.0033	-0.0014	0.032	-0.019	0.4379	0.8600141	Both
Elevation	nifh_Cluster531	0.0258	-0.01	0.1298	0.084	0.1087	0.6057242	Both
Elevation	nifh_Cluster539	0.0048	-0.0005	0.0019	-0.0507	0.8522	0.9737377	Both

Elevation	nifh_Cluster578	0.0481	0.0222	0.3626	0.3291	0.0039	0.0951137	Both
Elevation	nifh_Cluster58	0.3542	-0.043	0.0149	-0.037	0.5982	0.9225412	Both
Elevation	nifh_Cluster60	0.0089	-0.0031	0.0746	0.0259	0.2309	0.7697374	Both
Elevation	nifh_Cluster61	0.0025	-0.0011	0.0965	0.0489	0.1706	0.7242057	Both
Elevation	nifh_Cluster65	0.009	-0.0015	0.0153	-0.0365	0.5932	0.9225412	Both
Elevation	nifh_Cluster686	0.0009	-0.0003	0.0202	-0.0313	0.5384	0.9009061	Both
Elevation	nifh_Cluster69	0.009	-0.0052	0.1707	0.127	0.0627	0.4727139	Both
Elevation	nifh_Cluster70	0.0111	-0.0035	0.0283	-0.0228	0.4658	0.8600178	Both
Elevation	nifh_Cluster717	0.003	0.00E+00	1.00E-04	-0.0526	0.9739	0.9918486	Both
Elevation	nifh_Cluster725	0.0058	-0.0016	0.059	0.0094	0.2889	0.7870206	Both
Elevation	nifh_Cluster727	0.0039	0.0007	0.0131	-0.0388	0.6214	0.9225412	Both
Elevation	nifh_Cluster73	0.0106	-0.0037	0.0693	0.0203	0.2489	0.7716014	Both
Elevation	nifh_Cluster74	0.0049	-0.0016	0.0321	-0.0188	0.4368	0.8600141	Both
Elevation	nifh_Cluster748	0.0081	0.0008	0.0118	-0.0402	0.6396	0.9287205	Both
Elevation	nifh_Cluster749	0.0071	-0.0002	0.0012	-0.0514	0.8813	0.9849778	Both
Elevation	nifh_Cluster755	0.0102	0.008	0.3836	0.3512	0.0028	0.0757918	Both
Elevation	nifh_Cluster76	0.0029	1.00E-04	0.0005	-0.0521	0.9213	0.99014	Both
Elevation	nifh_Cluster79	0.0022	-0.0009	0.0707	0.0218	0.2441	0.7716014	Both
Elevation	nifh_Cluster81	0.0055	-0.002	0.0405	-0.01	0.3816	0.8471095	Both
Elevation	nifh_Cluster82	0.0011	-0.0007	0.0849	0.0368	0.2	0.736913	Both
Elevation	nifh_Cluster83	0.0069	0.0029	0.0845	0.0363	0.2011	0.736913	Both
Elevation	nifh_Cluster86	0.0135	0.0084	0.4227	0.3923	0.0014	0.0440902	Both
Elevation	nifh_Cluster868	0.0099	-0.001	0.0139	-0.038	0.6108	0.9225412	Both
Elevation	nifh_Cluster87	0.0024	-0.0008	0.0334	-0.0174	0.4275	0.8600141	Both
Elevation	nifh_Cluster88	0.0039	0.0057	0.6858	0.6692	0.00E+00	0.0005997	Both
Elevation	nifh_Cluster9	0.0078	-0.0004	0.0022	-0.0503	0.8384	0.971847	Both
Elevation	nifh_Cluster92	0.0051	-0.0015	0.0229	-0.0285	0.5127	0.8844509	Both
Elevation	nifh_Cluster93	0.0036	0.00E+00	0.00E+00	-0.0526	0.988	0.9947909	Both
Elevation	nifh_Cluster94	0.0047	-0.0016	0.0376	-0.013	0.3996	0.8554428	Both
Elevation	nifh_Cluster95	0.0121	-0.0016	0.0081	-0.0441	0.698	0.9577118	Both
Elevation	nifh_Cluster96	0.0123	0.0068	0.3194	0.2836	0.0076	0.1544313	Both
Elevation	nifh_Cluster97	0.018	-0.0012	0.0036	-0.0489	0.7975	0.9671747	Both
Elevation	nifh_Cluster98	0.0059	0.003	0.2323	0.1918	0.0269	0.3244605	Both
Elevation	nifh_Cluster99	0.0033	-0.001	0.0343	-0.0165	0.4214	0.8600141	Both
Elevation	nirk_Cluster0	5.9009	-0.2731	0.0333	-0.0176	0.4288	0.8600141	Both
Elevation	nirk_Cluster1	0.1708	-0.0317	0.0536	0.0038	0.3125	0.7958634	Both
Elevation	nirk_Cluster10	0.5713	0.1971	0.595	0.5737	0.00E+00	0.0035723	Both
Elevation	nirk_Cluster101	0.0031	-0.0006	0.0131	-0.0388	0.6212	0.9225412	Both
Elevation	nirk_Cluster102	0.0426	0.0007	0.0003	-0.0523	0.9388	0.9914842	Both
Elevation	nirk_Cluster103	0.0754	0.0051	0.0077	-0.0446	0.7058	0.9595565	Both
Elevation	nirk_Cluster104	0.0023	0.001	0.0526	0.0028	0.3172	0.7958634	Both
Elevation	nirk_Cluster105	0.0946	-0.002	0.0008	-0.0518	0.9016	0.9849778	Both
Elevation	nirk_Cluster106	0.0592	-0.0038	0.0042	-0.0482	0.7793	0.9671747	Both
Elevation	nirk_Cluster107	0.0891	0.0035	0.0024	-0.0501	0.8319	0.9687264	Both
Elevation	nirk_Cluster108	0.0151	0.0029	0.0342	-0.0166	0.4221	0.8600141	Both
Elevation	nirk_Cluster109	0.0115	0.0025	0.0368	-0.0139	0.4048	0.8554428	Both
Elevation	nirk_Cluster11	0.1401	0.006	0.0291	-0.022	0.4597	0.8600141	Both
Elevation	nirk_Cluster12	0.094	-0.0203	0.1958	0.1535	0.0446	0.4268248	Both
Elevation	nirk_Cluster13	0.0408	-0.0044	0.0774	0.0288	0.2221	0.7608685	Both
Elevation	nirk_Cluster14	0.0946	0.0193	0.1697	0.126	0.0635	0.4727139	Both
Elevation	nirk_Cluster15	0.3358	-0.0187	0.0056	-0.0467	0.7465	0.9671747	Both
Elevation	nirk_Cluster16	0.4727	-0.0484	0.0174	-0.0344	0.5692	0.9215392	Both
Elevation	nirk_Cluster17	0.0998	0.0009	0.0006	-0.052	0.9151	0.9871445	Both
Elevation	nirk_Cluster18	0.1296	-0.0285	0.0926	0.0449	0.1798	0.7266135	Both
Elevation	nirk_Cluster19	0.2776	-0.0189	0.0506	0.0006	0.3271	0.7972737	Both

Elevation	nirk_Cluster2	0.2032	0.0217	0.0988	0.0513	0.1653	0.7225175	Both
Elevation	nirk_Cluster20	0.1738	0.0275	0.0912	0.0434	0.1833	0.7331654	Both
Elevation	nirk_Cluster21	0.1532	0.0095	0.0312	-0.0198	0.4439	0.8600141	Both
Elevation	nirk_Cluster22	0.1291	0.00E+00	0.00E+00	-0.0526	0.9994	0.9993807	Both
Elevation	nirk_Cluster23	0.0673	0.0026	0.0145	-0.0374	0.6036	0.9225412	Both
Elevation	nirk_Cluster24	0.0654	0.0165	0.2076	0.1659	0.0379	0.4222987	Both
Elevation	nirk_Cluster25	0.4782	0.0285	0.0157	-0.0361	0.5883	0.9215392	Both
Elevation	nirk_Cluster26	0.105	-0.0017	0.002	-0.0505	0.8478	0.9737377	Both
Elevation	nirk_Cluster27	0.1727	0.0249	0.0682	0.0191	0.2529	0.7716014	Both
Elevation	nirk_Cluster28	0.0685	0.0155	0.1715	0.1279	0.062	0.4727139	Both
Elevation	nirk_Cluster29	0.1526	0.0421	0.5111	0.4854	0.0003	0.0133008	Both
Elevation	nirk_Cluster3	0.3023	0.0535	0.1985	0.1563	0.0429	0.4222987	Both
Elevation	nirk_Cluster30	0.0556	0.0007	0.0003	-0.0524	0.9457	0.9914842	Both
Elevation	nirk_Cluster31	0.1501	-0.003	0.005	-0.0473	0.7598	0.9671747	Both
Elevation	nirk_Cluster32	0.1254	-0.0287	0.0694	0.0205	0.2484	0.7716014	Both
Elevation	nirk_Cluster33	0.0638	-0.0068	0.0136	-0.0383	0.6149	0.9225412	Both
Elevation	nirk_Cluster34	0.0416	0.0032	0.013	-0.039	0.6232	0.9225412	Both
Elevation	nirk_Cluster35	0.1007	0.006	0.0042	-0.0482	0.7791	0.9671747	Both
Elevation	nirk_Cluster36	0.0017	0.0003	0.0084	-0.0438	0.6934	0.9577118	Both
Elevation	nirk_Cluster39	0.0035	0.0046	0.2862	0.2486	0.0125	0.2228335	Both
Elevation	nirk_Cluster4	0.1323	0.0129	0.0382	-0.0125	0.3961	0.8554428	Both
Elevation	nirk_Cluster40	0.0455	0.0034	0.0163	-0.0355	0.5812	0.9215392	Both
Elevation	nirk_Cluster41	0.1562	0.0011	0.0003	-0.0523	0.9437	0.9914842	Both
Elevation	nirk_Cluster44	0.0669	0.0053	0.0087	-0.0435	0.6882	0.9577118	Both
Elevation	nirk_Cluster46	0.0154	0.0031	0.0292	-0.0219	0.4593	0.8600141	Both
Elevation	nirk_Cluster48	0.003	-0.0004	0.0082	-0.044	0.6956	0.9577118	Both
Elevation	nirk_Cluster49	0.0057	-0.0036	0.1693	0.1256	0.0638	0.4727139	Both
Elevation	nirk_Cluster5	0.1932	0.0037	0.0028	-0.0496	0.8187	0.9671747	Both
Elevation	nirk_Cluster50	0.0016	-0.0002	0.0046	-0.0478	0.7708	0.9671747	Both
Elevation	nirk_Cluster52	0.0452	0.002	0.0044	-0.048	0.7753	0.9671747	Both
Elevation	nirk_Cluster53	0.0038	-0.0011	0.0278	-0.0234	0.4704	0.8600178	Both
Elevation	nirk_Cluster54	0.002	1.00E-04	0.0003	-0.0523	0.9397	0.9914842	Both
Elevation	nirk_Cluster55	0.0023	-0.001	0.0363	-0.0144	0.4081	0.8569446	Both
Elevation	nirk_Cluster56	0.0009	-0.0007	0.0887	0.0408	0.1897	0.7331654	Both
Elevation	nirk_Cluster57	0.0016	-0.0009	0.0595	0.01	0.2866	0.7870206	Both
Elevation	nirk_Cluster58	0.0088	0.0033	0.1088	0.0619	0.1443	0.670426	Both
Elevation	nirk_Cluster59	0.0058	0.0037	0.2756	0.2375	0.0145	0.2451668	Both
Elevation	nirk_Cluster6	0.1558	0.0361	0.2694	0.2309	0.0159	0.2515302	Both
Elevation	nirk_Cluster60	0.0006	0.0003	0.0419	-0.0085	0.3733	0.8456325	Both
Elevation	nirk_Cluster62	0.1417	0.0088	0.0054	-0.0469	0.7509	0.9671747	Both
Elevation	nirk_Cluster63	0.1284	0.0145	0.0278	-0.0234	0.4703	0.8600178	Both
Elevation	nirk_Cluster64	0.0044	0.0012	0.0546	0.0048	0.3081	0.7958634	Both
Elevation	nirk_Cluster65	0.0464	0.0032	0.0121	-0.0399	0.6345	0.9287205	Both
Elevation	nirk_Cluster66	0.0068	-0.0021	0.0681	0.019	0.2534	0.7716014	Both
Elevation	nirk_Cluster67	0.0174	0.0167	0.6816	0.6648	0.00E+00	0.0005997	Both
Elevation	nirk_Cluster68	0.0125	0.0018	0.0203	-0.0313	0.538	0.9009061	Both
Elevation	nirk_Cluster69	0.0305	0.0047	0.0484	-0.0017	0.3382	0.8077917	Both
Elevation	nirk_Cluster7	0.0804	0.023	0.2497	0.2102	0.0211	0.2960354	Both
Elevation	nirk_Cluster70	0.0721	-0.0051	0.0037	-0.0487	0.7931	0.9671747	Both
Elevation	nirk_Cluster71	0.0053	0.001	0.0211	-0.0304	0.5299	0.9009061	Both
Elevation	nirk_Cluster72	0.0239	0.0005	0.001	-0.0516	0.8942	0.9849778	Both
Elevation	nirk_Cluster73	0.1444	-0.0072	0.0041	-0.0483	0.782	0.9671747	Both
Elevation	nirk_Cluster74	0.6398	-0.0289	0.0031	-0.0494	0.8101	0.9671747	Both
Elevation	nirk_Cluster75	0.0147	-0.0015	0.0054	-0.0469	0.7508	0.9671747	Both
Elevation	nirk_Cluster76	0.1055	0.0075	0.0116	-0.0404	0.6417	0.9287205	Both

Elevation	nirk_Cluster77	0.0081	0.0139	0.6336	0.6143	0.00E+00	0.0015659	Both
Elevation	nirk_Cluster78	0.2407	0.0236	0.0188	-0.0328	0.553	0.9063855	Both
Elevation	nirk_Cluster79	0.0069	0.0092	0.5813	0.5592	1.00E-04	0.0043341	Both
Elevation	nirk_Cluster8	0.0982	0.0056	0.0069	-0.0454	0.7206	0.9671747	Both
Elevation	nirk_Cluster80	0.2665	-0.0303	0.0208	-0.0308	0.5331	0.9009061	Both
Elevation	nirk_Cluster82	0.0015	-0.0005	0.0194	-0.0322	0.5466	0.9056495	Both
Elevation	nirk_Cluster83	0.004	0.0021	0.1788	0.1356	0.0562	0.4477127	Both
Elevation	nirk_Cluster84	0.0207	-0.0006	0.0006	-0.052	0.9152	0.9871445	Both
Elevation	nirk_Cluster85	0.004	0.003	0.2909	0.2535	0.0116	0.2214039	Both
Elevation	nirk_Cluster86	0.0286	0.0025	0.0083	-0.0439	0.6948	0.9577118	Both
Elevation	nirk_Cluster87	0.0115	-0.0029	0.0571	0.0075	0.2969	0.7870206	Both
Elevation	nirk_Cluster88	0.0032	0.0005	0.0138	-0.0381	0.6115	0.9225412	Both
Elevation	nirk_Cluster89	0.0065	-0.002	0.0486	-0.0015	0.337	0.8077917	Both
Elevation	nirk_Cluster9	0.0517	0.0027	0.0049	-0.0475	0.7632	0.9671747	Both
Elevation	nirk_Cluster90	0.0067	-0.0012	0.0143	-0.0376	0.6062	0.9225412	Both
Elevation	nirk_Cluster91	0.0374	-0.0035	0.0157	-0.0362	0.5889	0.9215392	Both
Elevation	nirk_Cluster92	0.0072	-0.0015	0.0245	-0.0269	0.4982	0.869576	Both
Elevation	nirk_Cluster93	0.0308	0.0014	0.0035	-0.049	0.7993	0.9671747	Both
Elevation	nirk_Cluster94	0.0175	0.0044	0.0679	0.0189	0.2539	0.7716014	Both
Elevation	nirk_Cluster95	0.0473	-0.0039	0.0087	-0.0435	0.6878	0.9577118	Both
Elevation	nirk_Cluster96	0.0472	0.002	0.0029	-0.0496	0.817	0.9671747	Both
Elevation	nirk_Cluster97	0.0306	-0.0083	0.1214	0.0752	0.1216	0.6126302	Both
Elevation	nirk_Cluster98	0.0181	0.0071	0.1083	0.0613	0.1453	0.670426	Both
Elevation	nirk_Cluster99	0.006	0.0021	0.0658	0.0166	0.2616	0.7756714	Both
Elevation	nirs_Cluster0	0.1409	0.0336	0.2427	0.2029	0.0233	0.3050241	Both
Elevation	nirs_Cluster1	0.004	0.0015	0.0415	-0.0089	0.3755	0.8456325	Both
Elevation	nirs_Cluster14	0.0107	-0.0024	0.0111	-0.041	0.6501	0.9331749	Both
Elevation	nirs_Cluster2	0.0139	0.017	0.5514	0.5278	0.0001	0.0068302	Both
Elevation	nirs_Cluster26	0.0005	0.00E+00	0.0009	-0.0516	0.8947	0.9849778	Both
Elevation	nirs_Cluster28	0.0071	-0.0025	0.0259	-0.0253	0.4857	0.8635407	Both
Elevation	nirs_Cluster3	0.1715	0.026	0.2004	0.1583	0.0419	0.4222987	Both
Elevation	nirs_Cluster36	0.0013	0.0022	0.1861	0.1433	0.0509	0.4329593	Both
Elevation	nirs_Cluster37	0.0066	-0.0011	0.0031	-0.0494	0.811	0.9671747	Both
Elevation	nirs_Cluster4	0.0002	0.00E+00	0.0005	-0.0522	0.9273	0.990798	Both
Elevation	nirs_Cluster50	0.002	0.00E+00	1.00E-04	-0.0526	0.9721	0.9918486	Both
Elevation	nirs_Cluster9	0.0269	-0.0224	0.1421	0.097	0.0921	0.5778387	Both
Elevation	norb_Cluster0	0.0775	-0.0259	0.1823	0.1392	0.0536	0.4453034	Both
Elevation	norb_Cluster1	1.1042	-0.2952	0.0857	0.0375	0.1979	0.736913	Both
Elevation	norb_Cluster101	0.0026	-0.0013	0.0608	0.0114	0.2811	0.7870206	Both
Elevation	norb_Cluster102	0.0038	-0.0016	0.0828	0.0345	0.206	0.7456773	Both
Elevation	norb_Cluster103	0.0018	-0.0006	0.0274	-0.0238	0.4737	0.8617902	Both
Elevation	norb_Cluster104	0.0023	0.0004	0.0056	-0.0467	0.7461	0.9671747	Both
Elevation	norb_Cluster106	0.125	-0.0167	0.0306	-0.0204	0.4482	0.8600141	Both
Elevation	norb_Cluster107	0.0133	0.0005	0.0011	-0.0515	0.8873	0.9849778	Both
Elevation	norb_Cluster108	0.0052	0.0017	0.0783	0.0298	0.2192	0.7608685	Both
Elevation	norb_Cluster109	0.0227	0.0006	0.0008	-0.0518	0.9032	0.9849778	Both
Elevation	norb_Cluster11	0.0126	-0.0048	0.1081	0.0612	0.1456	0.670426	Both
Elevation	norb_Cluster110	0.0099	0.0023	0.0353	-0.0155	0.4151	0.8600141	Both
Elevation	norb_Cluster112	0.0171	-0.0006	0.0014	-0.0512	0.8735	0.9849778	Both
Elevation	norb_Cluster113	0.0134	-0.0028	0.0302	-0.0208	0.4511	0.8600141	Both
Elevation	norb_Cluster114	0.0025	-0.0005	0.0067	-0.0455	0.7234	0.9671747	Both
Elevation	norb_Cluster115	0.0137	-0.0016	0.001	-0.0515	0.8898	0.9849778	Both
Elevation	norb_Cluster116	0.0072	-0.0017	0.0248	-0.0265	0.495	0.869316	Both
Elevation	norb_Cluster117	0.0027	0.0001	0.0006	-0.0519	0.9127	0.9871445	Both
Elevation	norb_Cluster118	0.0037	0.00E+00	1.00E-04	-0.0526	0.97	0.9918486	Both

Elevation	norb_Cluster119	0.019	0.00E+00	0.00E+00	-0.0526	0.9935	0.9969189	Both
Elevation	norb_Cluster12	0.0047	-0.0004	0.0028	-0.0497	0.8213	0.9671747	Both
Elevation	norb_Cluster121	0.0017	-0.0013	0.0959	0.0483	0.1719	0.7244545	Both
Elevation	norb_Cluster122	0.0144	-0.0012	0.0061	-0.0462	0.736	0.9671747	Both
Elevation	norb_Cluster123	0.0053	0.00E+00	0.00E+00	-0.0526	0.9878	0.9947909	Both
Elevation	norb_Cluster124	0.682	-0.186	0.113	0.0663	0.1363	0.6535569	Both
Elevation	norb_Cluster125	0.004	-0.0009	0.03	-0.021	0.4527	0.8600141	Both
Elevation	norb_Cluster126	0.0082	-0.0016	0.0248	-0.0265	0.4951	0.869316	Both
Elevation	norb_Cluster127	0.1735	-0.0381	0.0628	0.0135	0.2733	0.7870206	Both
Elevation	norb_Cluster128	0.0115	-0.0026	0.0556	0.0059	0.3033	0.7953266	Both
Elevation	norb_Cluster13	0.0045	-0.0029	0.129	0.0831	0.1099	0.6057242	Both
Elevation	norb_Cluster131	0.0184	-0.0051	0.0795	0.031	0.2157	0.7608685	Both
Elevation	norb_Cluster132	1.0357	-0.0249	0.0012	-0.0514	0.8807	0.9849778	Both
Elevation	norb_Cluster133	0.0018	0.0016	0.2457	0.206	0.0223	0.2991059	Both
Elevation	norb_Cluster135	0.0567	-0.0054	0.0118	-0.0402	0.6391	0.9287205	Both
Elevation	norb_Cluster136	0.0213	-0.0006	0.0008	-0.0518	0.9021	0.9849778	Both
Elevation	norb_Cluster139	0.0328	0.0012	0.0018	-0.0507	0.8543	0.9737377	Both
Elevation	norb_Cluster14	0.0026	-0.002	0.2032	0.1612	0.0403	0.4222987	Both
Elevation	norb_Cluster140	0.0437	-0.0142	0.1376	0.0922	0.0978	0.6044683	Both
Elevation	norb_Cluster142	0.0015	-0.0012	0.1058	0.0587	0.1502	0.6765464	Both
Elevation	norb_Cluster144	0.0098	0.0028	0.0673	0.0183	0.256	0.7716014	Both
Elevation	norb_Cluster146	0.0348	0.0018	0.0042	-0.0482	0.7794	0.9671747	Both
Elevation	norb_Cluster15	0.0469	-0.0115	0.0692	0.0202	0.2493	0.7716014	Both
Elevation	norb_Cluster150	0.0157	0.0024	0.0313	-0.0197	0.4429	0.8600141	Both
Elevation	norb_Cluster155	0.1631	-0.0403	0.1024	0.0552	0.1573	0.6927439	Both
Elevation	norb_Cluster16	0.0131	-0.0076	0.1992	0.1571	0.0426	0.4222987	Both
Elevation	norb_Cluster164	0.0199	-0.0111	0.2354	0.1952	0.0258	0.31778	Both
Elevation	norb_Cluster165	0.0076	-0.0028	0.0595	0.01	0.2865	0.7870206	Both
Elevation	norb_Cluster166	0.0039	-0.0016	0.0703	0.0214	0.2454	0.7716014	Both
Elevation	norb_Cluster169	0.0238	-0.0113	0.1583	0.1141	0.074	0.5137635	Both
Elevation	norb_Cluster17	0.0028	-0.0015	0.1156	0.0691	0.1315	0.6360576	Both
Elevation	norb_Cluster170	0.0243	-0.0091	0.0859	0.0378	0.1973	0.736913	Both
Elevation	norb_Cluster171	0.0069	-0.0038	0.1026	0.0554	0.1569	0.6927439	Both
Elevation	norb_Cluster172	0.014	-0.0065	0.1172	0.0707	0.1288	0.6318175	Both
Elevation	norb_Cluster175	0.0458	-0.0215	0.1354	0.0899	0.1008	0.6044683	Both
Elevation	norb_Cluster176	0.0298	-0.0164	0.1854	0.1425	0.0514	0.4329593	Both
Elevation	norb_Cluster177	0.0282	-0.01	0.0772	0.0286	0.2228	0.7608685	Both
Elevation	norb_Cluster179	0.0022	0.0023	0.2774	0.2394	0.0142	0.2451668	Both
Elevation	norb_Cluster18	0.0037	-0.0012	0.0568	0.0071	0.2983	0.7870206	Both
Elevation	norb_Cluster180	0.0109	0.0022	0.0471	-0.003	0.3446	0.8165417	Both
Elevation	norb_Cluster181	0.0008	-1.00E-04	0.0008	-0.0518	0.9027	0.9849778	Both
Elevation	norb_Cluster182	0.0013	-0.0009	0.1077	0.0608	0.1463	0.670426	Both
Elevation	norb_Cluster184	0.0146	-0.0048	0.0881	0.0401	0.1913	0.7331654	Both
Elevation	norb_Cluster186	0.0011	-0.0003	0.0127	-0.0392	0.6261	0.9235587	Both
Elevation	norb_Cluster19	0.0049	-0.0012	0.0506	0.0006	0.3269	0.7972737	Both
Elevation	norb_Cluster192	0.0173	-0.0087	0.168	0.1242	0.065	0.4735892	Both
Elevation	norb_Cluster193	0.0021	-0.0009	0.0573	0.0077	0.2958	0.7870206	Both
Elevation	norb_Cluster197	0.0303	-0.0059	0.0057	-0.0466	0.7449	0.9671747	Both
Elevation	norb_Cluster2	0.0031	-0.0017	0.1085	0.0615	0.1449	0.670426	Both
Elevation	norb_Cluster20	0.0587	-0.0146	0.0942	0.0465	0.176	0.7266135	Both
Elevation	norb_Cluster208	0.0011	-0.0006	0.0508	0.0008	0.3261	0.7972737	Both
Elevation	norb_Cluster21	0.0481	-0.011	0.0584	0.0088	0.2914	0.7870206	Both
Elevation	norb_Cluster211	0.0104	0.0007	0.0009	-0.0517	0.8978	0.9849778	Both
Elevation	norb_Cluster212	0.0264	-0.009	0.035	-0.0158	0.4169	0.8600141	Both
Elevation	norb_Cluster214	0.0119	0.0002	1.00E-04	-0.0526	0.9735	0.9918486	Both

Elevation	norb_Cluster217	0.0149	-0.0042	0.0278	-0.0234	0.4703	0.8600178	Both
Elevation	norb_Cluster219	0.0083	-0.0025	0.022	-0.0294	0.5207	0.8929769	Both
Elevation	norb_Cluster22	0.0025	0.001	0.035	-0.0157	0.4165	0.8600141	Both
Elevation	norb_Cluster220	0.0177	-0.0042	0.0118	-0.0402	0.6386	0.9287205	Both
Elevation	norb_Cluster221	0.0177	-0.0037	0.0082	-0.044	0.6964	0.9577118	Both
Elevation	norb_Cluster222	0.0074	-0.0016	0.0064	-0.0459	0.731	0.9671747	Both
Elevation	norb_Cluster224	0.0194	-0.0072	0.0439	-0.0064	0.3621	0.8344594	Both
Elevation	norb_Cluster225	0.0097	-0.0002	0.0001	-0.0525	0.9613	0.9918486	Both
Elevation	norb_Cluster226	0.0043	-0.0007	0.0109	-0.0412	0.6529	0.9348377	Both
Elevation	norb_Cluster228	0.0031	-0.0009	0.0061	-0.0462	0.7368	0.9671747	Both
Elevation	norb_Cluster229	0.0034	-0.0007	0.0169	-0.0349	0.5747	0.9215392	Both
Elevation	norb_Cluster230	0.0014	-1.00E-04	0.0007	-0.0518	0.9066	0.9868401	Both
Elevation	norb_Cluster231	0.0044	0.0026	0.0849	0.0367	0.2001	0.736913	Both
Elevation	norb_Cluster232	0.0031	0.0015	0.0634	0.0141	0.2708	0.7870206	Both
Elevation	norb_Cluster234	0.0121	-0.0059	0.0945	0.0468	0.1753	0.7266135	Both
Elevation	norb_Cluster235	0.0009	1.00E-04	0.0028	-0.0497	0.8197	0.9671747	Both
Elevation	norb_Cluster238	0.0099	-0.0007	0.0016	-0.051	0.8647	0.9773841	Both
Elevation	norb_Cluster239	0.0081	-0.0028	0.0317	-0.0193	0.44	0.8600141	Both
Elevation	norb_Cluster24	0.2637	-0.0639	0.0888	0.0409	0.1894	0.7331654	Both
Elevation	norb_Cluster244	0.0011	0.0007	0.0782	0.0296	0.2197	0.7608685	Both
Elevation	norb_Cluster247	0.0048	0.0037	0.4609	0.4325	0.0007	0.0324311	Both
Elevation	norb_Cluster25	0.1114	-0.0324	0.1042	0.057	0.1535	0.6862289	Both
Elevation	norb_Cluster250	0.0115	0.0012	0.0077	-0.0446	0.7058	0.9595565	Both
Elevation	norb_Cluster251	0.0052	-0.0003	0.0012	-0.0513	0.8801	0.9849778	Both
Elevation	norb_Cluster26	0.0931	-0.03	0.107	0.06	0.1477	0.670426	Both
Elevation	norb_Cluster28	0.0302	-0.0042	0.0202	-0.0314	0.539	0.9009061	Both
Elevation	norb_Cluster29	0.222	-0.0463	0.1654	0.1215	0.0673	0.4840832	Both
Elevation	norb_Cluster31	0.0531	-0.0089	0.0331	-0.0178	0.4297	0.8600141	Both
Elevation	norb_Cluster32	0.1193	-0.0344	0.0756	0.0269	0.2278	0.7636069	Both
Elevation	norb_Cluster33	0.4946	-0.1292	0.256	0.2168	0.0193	0.2774492	Both
Elevation	norb_Cluster35	0.0628	-0.0172	0.0929	0.0452	0.179	0.7266135	Both
Elevation	norb_Cluster36	1.1345	-0.4054	0.2473	0.2077	0.0218	0.2991059	Both
Elevation	norb_Cluster37	0.7473	-0.2481	0.3315	0.2963	0.0063	0.1379341	Both
Elevation	norb_Cluster38	0.0116	-0.0007	0.0049	-0.0474	0.7624	0.9671747	Both
Elevation	norb_Cluster39	0.0426	-0.009	0.0529	0.0031	0.3157	0.7958634	Both
Elevation	norb_Cluster40	0.186	-0.0346	0.0466	-0.0036	0.3475	0.8188088	Both
Elevation	norb_Cluster42	0.2864	-0.0957	0.2352	0.195	0.0259	0.31778	Both
Elevation	norb_Cluster44	0.0182	0.003	0.0166	-0.0351	0.5773	0.9215392	Both
Elevation	norb_Cluster46	0.2556	-0.0194	0.0083	-0.0438	0.6937	0.9577118	Both
Elevation	norb_Cluster47	0.555	-0.1331	0.0931	0.0454	0.1785	0.7266135	Both
Elevation	norb_Cluster5	0.01	-0.0017	0.0371	-0.0136	0.4028	0.8554428	Both
Elevation	norb_Cluster51	0.0092	-0.0045	0.0904	0.0426	0.1853	0.7331654	Both
Elevation	norb_Cluster52	0.2391	-0.0513	0.0592	0.0097	0.2878	0.7870206	Both
Elevation	norb_Cluster56	0.014	-0.0006	0.0021	-0.0505	0.8454	0.9737377	Both
Elevation	norb_Cluster57	0.0034	-0.0004	0.0084	-0.0437	0.6921	0.9577118	Both
Elevation	norb_Cluster59	0.0236	0.0099	0.0872	0.0392	0.1937	0.7331654	Both
Elevation	norb_Cluster6	0.6862	-0.2463	0.2645	0.2258	0.0171	0.2582158	Both
Elevation	norb_Cluster60	0.3199	-0.0887	0.061	0.0116	0.2803	0.7870206	Both
Elevation	norb_Cluster61	0.482	-0.1448	0.1209	0.0746	0.1225	0.6126302	Both
Elevation	norb_Cluster62	0.0024	0.0002	0.0028	-0.0496	0.8183	0.9671747	Both
Elevation	norb_Cluster64	0.0326	0.0003	0.0001	-0.0525	0.9588	0.9918486	Both
Elevation	norb_Cluster66	0.0012	0.0003	0.0099	-0.0422	0.6674	0.947175	Both
Elevation	norb_Cluster67	0.0218	0.0058	0.0689	0.0199	0.2505	0.7716014	Both
Elevation	norb_Cluster68	0.0034	-0.0012	0.0872	0.0392	0.1936	0.7331654	Both
Elevation	norb_Cluster69	0.0028	0.0002	0.0024	-0.0501	0.8324	0.9687264	Both

Elevation	norb_Cluster7	0.0709	-0.0166	0.0783	0.0298	0.2192	0.7608685	Both
Elevation	norb_Cluster70	0.1051	-0.0013	0.0002	-0.0524	0.9476	0.9914842	Both
Elevation	norb_Cluster71	0.1295	-0.0152	0.0262	-0.0251	0.4835	0.8635407	Both
Elevation	norb_Cluster74	0.0327	-0.0022	0.0072	-0.0451	0.7152	0.9671747	Both
Elevation	norb_Cluster75	0.1291	-0.0134	0.0257	-0.0256	0.4874	0.8635407	Both
Elevation	norb_Cluster76	0.1105	-0.0247	0.0298	-0.0213	0.4543	0.8600141	Both
Elevation	norb_Cluster77	0.0475	0.0005	0.0003	-0.0523	0.939	0.9914842	Both
Elevation	norb_Cluster8	0.018	-0.0035	0.0368	-0.0139	0.405	0.8554428	Both
Elevation	norb_Cluster80	0.0015	-0.0009	0.0675	0.0185	0.2552	0.7716014	Both
Elevation	norb_Cluster81	0.0012	-0.0005	0.039	-0.0116	0.3911	0.8554428	Both
Elevation	norb_Cluster83	0.0078	-0.0046	0.0807	0.0323	0.212	0.7608685	Both
Elevation	norb_Cluster84	0.0082	0.0006	0.0035	-0.049	0.7991	0.9671747	Both
Elevation	norb_Cluster85	0.0023	-0.0008	0.0381	-0.0125	0.3963	0.8554428	Both
Elevation	norb_Cluster86	0.031	-0.0082	0.0199	-0.0316	0.5415	0.9025174	Both
Elevation	norb_Cluster88	0.0016	-0.0006	0.0405	-0.01	0.3819	0.8471095	Both
Elevation	norb_Cluster90	0.0009	-0.0003	0.0079	-0.0443	0.7021	0.9595565	Both
Elevation	norb_Cluster91	0.6481	-0.2441	0.169	0.1253	0.0641	0.4727139	Both
Elevation	norb_Cluster92	0.0015	0.001	0.053	0.0032	0.3153	0.7958634	Both
Elevation	norb_Cluster93	0.0022	-0.0007	0.0202	-0.0314	0.5389	0.9009061	Both
Elevation	norb_Cluster94	0.0017	0.001	0.1236	0.0775	0.1181	0.6057242	Both
Elevation	norb_Cluster96	0.0007	0.0001	0.0036	-0.0488	0.7952	0.9671747	Both
Elevation	norb_Cluster97	0.0119	0.0085	0.2872	0.2497	0.0123	0.2228335	Both
Elevation	norb_Cluster98	0.0132	-0.0037	0.036	-0.0147	0.4101	0.8581057	Both
Elevation	nosz_Cluster0	0.0045	0.0014	0.0572	0.0076	0.2965	0.7870206	Both
Elevation	nosz_Cluster10	0.0136	-0.0024	0.0186	-0.033	0.5555	0.907898	Both
Elevation	nosz_Cluster102	0.0044	0.0017	0.1345	0.0889	0.102	0.6044683	Both
Elevation	nosz_Cluster103	0.0455	-0.0144	0.1801	0.1369	0.0552	0.4461049	Both
Elevation	nosz_Cluster105	0.0016	0.0004	0.0257	-0.0255	0.4872	0.8635407	Both
Elevation	nosz_Cluster106	0.0073	-0.004	0.1987	0.1565	0.0429	0.4222987	Both
Elevation	nosz_Cluster107	0.0002	0.00E+00	0.0005	-0.0522	0.9273	0.990798	Both
Elevation	nosz_Cluster108	0.004	-0.001	0.0313	-0.0197	0.4432	0.8600141	Both
Elevation	nosz_Cluster109	0.0375	-0.0029	0.0157	-0.0361	0.5885	0.9215392	Both
Elevation	nosz_Cluster11	0.0129	0.0027	0.0116	-0.0405	0.6427	0.9287205	Both
Elevation	nosz_Cluster110	0.0003	-0.0002	0.0193	-0.0323	0.548	0.9056495	Both
Elevation	nosz_Cluster111	0.0061	-0.0019	0.0694	0.0205	0.2484	0.7716014	Both
Elevation	nosz_Cluster116	0.0067	0.0009	0.0132	-0.0388	0.6204	0.9225412	Both
Elevation	nosz_Cluster118	0.0045	-1.00E-04	1.00E-04	-0.0525	0.9684	0.9918486	Both
Elevation	nosz_Cluster119	0.0013	-0.0011	0.1444	0.0993	0.0893	0.5704513	Both
Elevation	nosz_Cluster120	0.0071	-0.0009	0.0134	-0.0385	0.6174	0.9225412	Both
Elevation	nosz_Cluster123	0.0097	-0.0032	0.0572	0.0076	0.2962	0.7870206	Both
Elevation	nosz_Cluster128	0.0047	-0.0012	0.0272	-0.024	0.4747	0.8617902	Both
Elevation	nosz_Cluster129	0.0494	-0.0143	0.3745	0.3416	0.0032	0.0819792	Both
Elevation	nosz_Cluster13	0.0115	0.0022	0.0282	-0.023	0.4669	0.8600178	Both
Elevation	nosz_Cluster130	0.089	-0.0241	0.0155	-0.0363	0.5904	0.9215392	Both
Elevation	nosz_Cluster132	0.0037	-0.0008	0.0408	-0.0097	0.3799	0.8471095	Both
Elevation	nosz_Cluster14	0.024	0.0003	0.00E+00	-0.0526	0.9837	0.9947909	Both
Elevation	nosz_Cluster16	0.0076	0.0006	0.0026	-0.0499	0.8254	0.9681243	Both
Elevation	nosz_Cluster17	0.0364	-0.0076	0.0606	0.0111	0.2821	0.7870206	Both
Elevation	nosz_Cluster18	0.0161	0.0029	0.016	-0.0358	0.5849	0.9215392	Both
Elevation	nosz_Cluster20	0.0091	-0.0024	0.0309	-0.0201	0.4457	0.8600141	Both
Elevation	nosz_Cluster21	0.0083	-0.0034	0.0667	0.0176	0.2582	0.7716014	Both
Elevation	nosz_Cluster22	0.0267	-0.0012	0.0009	-0.0517	0.8999	0.9849778	Both
Elevation	nosz_Cluster23	0.0141	-0.0042	0.0471	-0.003	0.3445	0.8165417	Both
Elevation	nosz_Cluster24	0.0013	0.0012	0.1472	0.1024	0.0859	0.5571879	Both
Elevation	nosz_Cluster25	0.0029	0.0007	0.0159	-0.0359	0.5855	0.9215392	Both

Elevation	nosz_Cluster26	0.0054	-0.0009	0.0115	-0.0405	0.6438	0.9287205	Both
Elevation	nosz_Cluster27	0.0053	0.0009	0.0156	-0.0362	0.5898	0.9215392	Both
Elevation	nosz_Cluster28	0.0303	-0.0024	0.0063	-0.046	0.7328	0.9671747	Both
Elevation	nosz_Cluster30	0.0276	0.0027	0.0093	-0.0428	0.6773	0.955177	Both
Elevation	nosz_Cluster32	0.0137	0.0029	0.0366	-0.0141	0.4058	0.8554428	Both
Elevation	nosz_Cluster33	0.018	-0.0021	0.0061	-0.0462	0.7367	0.9671747	Both
Elevation	nosz_Cluster34	0.0377	-0.0124	0.055	0.0053	0.3061	0.7958634	Both
Elevation	nosz_Cluster35	0.0075	0.0008	0.0066	-0.0457	0.7266	0.9671747	Both
Elevation	nosz_Cluster36	0.0179	-0.0025	0.0143	-0.0376	0.6059	0.9225412	Both
Elevation	nosz_Cluster37	0.006	0.0017	0.0445	-0.0058	0.3587	0.8298785	Both
Elevation	nosz_Cluster38	0.0128	-0.0009	0.0023	-0.0502	0.8361	0.9710473	Both
Elevation	nosz_Cluster39	0.0537	0.0042	0.0062	-0.0461	0.7336	0.9671747	Both
Elevation	nosz_Cluster40	0.0218	0.0062	0.0567	0.007	0.2988	0.7870206	Both
Elevation	nosz_Cluster41	0.0203	0.004	0.0263	-0.0249	0.4822	0.8635407	Both
Elevation	nosz_Cluster42	0.0279	-0.0025	0.0059	-0.0464	0.7413	0.9671747	Both
Elevation	nosz_Cluster43	0.0007	-0.0005	0.0401	-0.0104	0.3843	0.8491506	Both
Elevation	nosz_Cluster44	0.0057	-0.0015	0.0397	-0.0108	0.3863	0.8503318	Both
Elevation	nosz_Cluster45	0.0045	-1.00E-04	0.0001	-0.0525	0.9631	0.9918486	Both
Elevation	nosz_Cluster46	0.007	0.0009	0.0148	-0.0371	0.5996	0.9225412	Both
Elevation	nosz_Cluster47	0.0008	-0.0004	0.0366	-0.0141	0.406	0.8554428	Both
Elevation	nosz_Cluster48	0.0023	-0.0008	0.0346	-0.0162	0.4192	0.8600141	Both
Elevation	nosz_Cluster49	0.0014	0.0007	0.059	0.0094	0.2889	0.7870206	Both
Elevation	nosz_Cluster5	0.0045	-0.0005	0.0031	-0.0494	0.8115	0.9671747	Both
Elevation	nosz_Cluster51	0.0021	-0.0002	0.0017	-0.0509	0.8596	0.9737377	Both
Elevation	nosz_Cluster53	0.0009	0.0007	0.0706	0.0217	0.2444	0.7716014	Both
Elevation	nosz_Cluster54	0.0026	-0.0014	0.0792	0.0307	0.2166	0.7608685	Both
Elevation	nosz_Cluster57	0.1757	-0.0028	0.0002	-0.0524	0.9472	0.9914842	Both
Elevation	nosz_Cluster58	0.0043	-0.0028	0.1564	0.112	0.076	0.5155288	Both
Elevation	nosz_Cluster59	0.0009	-0.0006	0.0426	-0.0078	0.3696	0.8451857	Both
Elevation	nosz_Cluster6	0.0032	0.0003	0.0028	-0.0497	0.8213	0.9671747	Both
Elevation	nosz_Cluster60	0.0079	0.0022	0.0427	-0.0076	0.3686	0.8451857	Both
Elevation	nosz_Cluster61	0.0178	-0.0044	0.1325	0.0868	0.1048	0.6057242	Both
Elevation	nosz_Cluster62	0.0413	-0.0014	0.0011	-0.0515	0.8891	0.9849778	Both
Elevation	nosz_Cluster64	0.0016	0.0018	0.1243	0.0782	0.117	0.6057242	Both
Elevation	nosz_Cluster65	0.0065	0.0006	0.0045	-0.0479	0.7719	0.9671747	Both
Elevation	nosz_Cluster66	0.0324	-0.0033	0.0067	-0.0456	0.7241	0.9671747	Both
Elevation	nosz_Cluster67	0.0521	0.0012	0.0003	-0.0523	0.9361	0.9914842	Both
Elevation	nosz_Cluster69	0.0061	-0.0027	0.1167	0.0702	0.1296	0.6318175	Both
Elevation	nosz_Cluster70	0.0479	-0.0058	0.0039	-0.0485	0.7882	0.9671747	Both
Elevation	nosz_Cluster71	0.0083	-0.0004	0.0009	-0.0517	0.8962	0.9849778	Both
Elevation	nosz_Cluster72	0.0506	-0.0037	0.0042	-0.0482	0.7797	0.9671747	Both
Elevation	nosz_Cluster73	0.003	-0.0009	0.0317	-0.0193	0.4404	0.8600141	Both
Elevation	nosz_Cluster74	0.0017	-0.0013	0.1855	0.1427	0.0513	0.4329593	Both
Elevation	nosz_Cluster75	0.0967	-0.0204	0.0067	-0.0456	0.7239	0.9671747	Both
Elevation	nosz_Cluster77	0.0002	-0.0001	0.0193	-0.0323	0.548	0.9056495	Both
Elevation	nosz_Cluster78	0.0044	-0.0021	0.1439	0.0988	0.0899	0.5704513	Both
Elevation	nosz_Cluster79	0.0333	0.0053	0.0312	-0.0198	0.4435	0.8600141	Both
Elevation	nosz_Cluster8	0.0099	-0.0003	0.001	-0.0516	0.8915	0.9849778	Both
Elevation	nosz_Cluster80	0.0045	-0.0012	0.0202	-0.0313	0.5386	0.9009061	Both
Elevation	nosz_Cluster81	0.0079	0.0001	1.00E-04	-0.0525	0.9662	0.9918486	Both
Elevation	nosz_Cluster82	0.0016	0.0033	0.3112	0.2749	0.0086	0.1690746	Both
Elevation	nosz_Cluster83	0.0015	-0.0013	0.1263	0.0803	0.1139	0.6057242	Both
Elevation	nosz_Cluster85	0.0022	0.0024	0.2681	0.2296	0.0162	0.2515302	Both
Elevation	nosz_Cluster86	0.0027	0.0023	0.2071	0.1654	0.0382	0.4222987	Both
Elevation	nosz_Cluster87	0.003	0.0005	0.0156	-0.0362	0.5892	0.9215392	Both

Elevation	nosz_Cluster88	0.0218	-0.0059	0.1584	0.1141	0.0739	0.5137635	Both
Elevation	nosz_Cluster89	0.0466	-0.0029	0.0031	-0.0494	0.8118	0.9671747	Both
Elevation	nosz_Cluster9	0.003	-0.0001	0.0006	-0.052	0.9146	0.9871445	Both
Elevation	nosz_Cluster90	0.0116	0.0001	0.0002	-0.0524	0.9478	0.9914842	Both
Elevation	nosz_Cluster91	0.0189	-0.001	0.0018	-0.0507	0.8544	0.9737377	Both
Elevation	nosz_Cluster93	0.0393	-0.0098	0.0639	0.0146	0.2691	0.7870206	Both
Elevation	nosz_Cluster95	0.007	-0.001	0.0131	-0.0388	0.6208	0.9225412	Both
Elevation	nosz_Cluster96	0.0066	-0.0005	0.0056	-0.0467	0.7473	0.9671747	Both
Elevation	nosz_Cluster97	0.0019	1.00E-04	0.0004	-0.0522	0.9325	0.9914842	Both
Elevation	nosz_Cluster98	0.0025	0.0045	0.2719	0.2336	0.0153	0.2513155	Both
Elevation	nosz_Cluster99	0.0055	0.0004	0.0053	-0.0471	0.7541	0.9671747	Both
Elevation	nrfa_Cluster10	0.0804	-0.0045	0.002	-0.0505	0.8457	0.9737377	Both
Elevation	nrfa_Cluster100	0.0963	-0.0497	0.1234	0.0772	0.1185	0.6057242	Both
Elevation	nrfa_Cluster104	0.1143	-0.0032	0.0008	-0.0518	0.9012	0.9849778	Both
Elevation	nrfa_Cluster105	0.0062	-0.0008	0.0062	-0.0461	0.7341	0.9671747	Both
Elevation	nrfa_Cluster109	0.2294	0.0326	0.0253	-0.026	0.4908	0.8670483	Both
Elevation	nrfa_Cluster116	0.2592	0.0121	0.0042	-0.0482	0.7806	0.9671747	Both
Elevation	nrfa_Cluster118	0.173	-0.0995	0.125	0.0789	0.1159	0.6057242	Both
Elevation	nrfa_Cluster125	0.0809	-0.0489	0.123	0.0768	0.1191	0.6057242	Both
Elevation	nrfa_Cluster154	0.0658	-0.0275	0.0881	0.0401	0.1914	0.7331654	Both
Elevation	nrfa_Cluster164	0.0045	-0.0005	0.0053	-0.0471	0.7538	0.9671747	Both
Elevation	nrfa_Cluster172	0.0026	-0.001	0.0321	-0.0188	0.437	0.8600141	Both
Elevation	nrfa_Cluster175	0.0044	-0.0036	0.1625	0.1184	0.07	0.4977108	Both
Elevation	nrfa_Cluster20	0.035	0.0031	0.0048	-0.0476	0.7662	0.9671747	Both
Elevation	nrfa_Cluster26	0.1078	-0.0603	0.1308	0.085	0.1072	0.6057242	Both
Elevation	nrfa_Cluster29	0.0048	-0.0014	0.0312	-0.0198	0.4437	0.8600141	Both
Elevation	nrfa_Cluster34	0.0487	-0.0004	0.00E+00	-0.0526	0.9788	0.9929059	Both
Elevation	nrfa_Cluster38	0.0031	-0.0023	0.1558	0.1113	0.0766	0.5155288	Both
Elevation	nrfa_Cluster61	0.0036	0.0001	0.0006	-0.052	0.9183	0.988681	Both
Elevation	nrfa_Cluster62	0.0452	-0.0273	0.1271	0.0812	0.1126	0.6057242	Both
Elevation	nrfa_Cluster66	0.1082	-0.005	0.0032	-0.0493	0.8083	0.9671747	Both
Elevation	nrfa_Cluster69	0.003	-0.0006	0.0157	-0.0362	0.589	0.9215392	Both
Elevation	nrfa_Cluster71	0.027	-0.0096	0.0575	0.0079	0.2952	0.7870206	Both
Elevation	nrfa_Cluster72	0.0732	-0.023	0.0541	0.0043	0.3102	0.7958634	Both
Elevation	nrfa_Cluster73	0.0038	-0.0017	0.0569	0.0072	0.2979	0.7870206	Both
Elevation	nrfa_Cluster74	0.1331	-0.0629	0.1291	0.0833	0.1096	0.6057242	Both
Elevation	nrfa_Cluster76	0.0755	-0.0377	0.1237	0.0776	0.1179	0.6057242	Both
Elevation	nrfa_Cluster79	0.0229	-0.0049	0.006	-0.0463	0.738	0.9671747	Both
Elevation	nrfa_Cluster87	0.017	-0.0044	0.019	-0.0326	0.5515	0.9063091	Both
Elevation	nrfa_Cluster89	0.0018	-0.0011	0.0787	0.0302	0.2181	0.7608685	Both
Elevation	nrfa_Cluster92	0.0043	-0.0016	0.0461	-0.0041	0.3498	0.8189372	Both
Elevation	nrfa_Cluster93	0.0428	-0.0076	0.0093	-0.0429	0.6783	0.955177	Both
Elevation	nrfa_Cluster94	0.0002	-0.0002	0.0455	-0.0048	0.3534	0.8238634	Both
Elevation	nrfa_Cluster97	0.0168	-0.0062	0.0965	0.049	0.1704	0.7242057	Both
Elevation	nrfa_Cluster99	0.014	-0.0057	0.0973	0.0498	0.1686	0.7242057	Both
DNA	amoA_A_Cluster26	0.0053	-0.006	0.4861	0.4466	0.0039	0.0644571	Mineral
DNA	amoA_A_Cluster45	0.0071	-0.0061	0.4221	0.3776	0.0088	0.0821001	Mineral
DNA	amoA_B_Cluster0	0.1272	-0.0149	0.1141	0.0459	0.2183	0.3819279	Mineral
DNA	amoA_B_Cluster1	0.0205	0.0046	0.0817	0.011	0.3018	0.4636927	Mineral
DNA	amoA_B_Cluster10	0.0539	-0.0115	0.1987	0.1371	0.0958	0.2583763	Mineral
DNA	amoA_B_Cluster11	0.0486	0.0069	0.0514	-0.0216	0.4164	0.5575554	Mineral
DNA	amoA_B_Cluster12	0.093	-0.0081	0.0457	-0.0277	0.4444	0.5749316	Mineral
DNA	amoA_B_Cluster13	0.6417	-0.038	0.0108	-0.0652	0.7119	0.7925058	Mineral
DNA	amoA_B_Cluster14	0.0339	-0.0042	0.022	-0.0533	0.598	0.6945688	Mineral
DNA	amoA_B_Cluster15	0.0413	0.0046	0.0305	-0.0441	0.5334	0.6475858	Mineral

DNA	amoA_B_Cluster2	0.2143	-0.0064	0.0029	-0.0738	0.849	0.8944354	Mineral
DNA	amoA_B_Cluster20	0.0078	0.0013	0.0173	-0.0583	0.6407	0.734031	Mineral
DNA	amoA_B_Cluster23	0.0626	0.0071	0.0494	-0.0237	0.4259	0.5595807	Mineral
DNA	amoA_B_Cluster3	0.0244	0.009	0.1967	0.1349	0.0978	0.2609696	Mineral
DNA	amoA_B_Cluster4	0.0169	-0.0002	0.0002	-0.0767	0.9556	0.9737953	Mineral
DNA	amoA_B_Cluster5	0.1032	-0.0102	0.0667	-0.0051	0.3529	0.5078018	Mineral
DNA	amoA_B_Cluster6	0.0279	0.0012	0.0051	-0.0715	0.8013	0.8582317	Mineral
DNA	amoA_B_Cluster7	0.0232	0.0034	0.0263	-0.0486	0.5638	0.6678382	Mineral
DNA	amoA_B_Cluster8	0.0268	0.0085	0.1217	0.0541	0.2026	0.3726935	Mineral
DNA	amoA_B_Cluster9	0.0693	0.0166	0.1035	0.0345	0.2423	0.4083701	Mineral
DNA	nifh_Cluster0	0.0318	0.002	0.0128	-0.0631	0.6878	0.7729329	Mineral
DNA	nifh_Cluster10	0.0033	-0.0003	0.005	-0.0715	0.8022	0.8582317	Mineral
DNA	nifh_Cluster100	0.002	-0.0013	0.1446	0.0788	0.162	0.3270565	Mineral
DNA	nifh_Cluster103	0.0433	-0.0063	0.0576	-0.0149	0.3888	0.5387079	Mineral
DNA	nifh_Cluster1034	0.0337	-0.0048	0.0214	-0.0539	0.6032	0.6977768	Mineral
DNA	nifh_Cluster105	0.0164	-0.0069	0.1592	0.0945	0.1407	0.3058564	Mineral
DNA	nifh_Cluster108	0.0076	-0.0043	0.3098	0.2567	0.0312	0.1596839	Mineral
DNA	nifh_Cluster1099	0.0478	0.0204	0.1057	0.0369	0.237	0.4040847	Mineral
DNA	nifh_Cluster11	0.0025	-0.0019	0.318	0.2655	0.0286	0.1531733	Mineral
DNA	nifh_Cluster1112	0.0037	0.0005	0.0097	-0.0665	0.7272	0.8079465	Mineral
DNA	nifh_Cluster112	0.0098	0.0044	0.1154	0.0474	0.2154	0.3816213	Mineral
DNA	nifh_Cluster113	0.0374	-0.0086	0.1803	0.1172	0.1147	0.2882336	Mineral
DNA	nifh_Cluster114	0.0304	0.00E+00	0.00E+00	-0.0769	0.9918	0.9918192	Mineral
DNA	nifh_Cluster1141	0.0296	-0.0038	0.0238	-0.0513	0.5831	0.6830721	Mineral
DNA	nifh_Cluster115	0.0098	-0.0012	0.0241	-0.051	0.5809	0.6830721	Mineral
DNA	nifh_Cluster116	0.0883	0.0161	0.1176	0.0497	0.2109	0.3782494	Mineral
DNA	nifh_Cluster1163	0.0051	0.0006	0.0137	-0.0622	0.6784	0.7638179	Mineral
DNA	nifh_Cluster1179	0.0037	-0.0011	0.0336	-0.0407	0.5129	0.6331239	Mineral
DNA	nifh_Cluster1182	0.0209	-0.0001	0.0001	-0.0768	0.9708	0.9824654	Mineral
DNA	nifh_Cluster1183	0.0045	0.0024	0.3156	0.263	0.0293	0.1547132	Mineral
DNA	nifh_Cluster1197	0.0115	-0.003	0.0777	0.0067	0.3146	0.4690363	Mineral
DNA	nifh_Cluster1199	0.0048	0.0015	0.0649	-0.007	0.3594	0.5158823	Mineral
DNA	nifh_Cluster120	0.02	-0.0137	0.1806	0.1176	0.1143	0.2882336	Mineral
DNA	nifh_Cluster1203	0.0217	-0.0034	0.0274	-0.0474	0.5556	0.6633402	Mineral
DNA	nifh_Cluster1206	0.006	0.0019	0.1138	0.0457	0.2188	0.3819279	Mineral
DNA	nifh_Cluster1207	0.0078	-0.0026	0.0753	0.0042	0.3222	0.4737997	Mineral
DNA	nifh_Cluster1209	0.1617	0.0251	0.1655	0.1013	0.1324	0.2995799	Mineral
DNA	nifh_Cluster121	0.0123	-0.0126	0.4164	0.3715	0.0094	0.0832291	Mineral
DNA	nifh_Cluster1211	0.0068	0.0016	0.0542	-0.0185	0.4036	0.5486435	Mineral
DNA	nifh_Cluster1213	0.0032	-0.0014	0.1587	0.0939	0.1414	0.3058564	Mineral
DNA	nifh_Cluster122	0.0041	-0.0041	0.5088	0.471	0.0028	0.0555855	Mineral
DNA	nifh_Cluster123	0.0092	-0.0077	0.3325	0.2812	0.0244	0.1424451	Mineral
DNA	nifh_Cluster1236	0.031	-0.0229	0.3045	0.251	0.033	0.1635282	Mineral
DNA	nifh_Cluster1238	0.0034	-0.002	0.1482	0.0826	0.1566	0.3228616	Mineral
DNA	nifh_Cluster124	0.0101	-0.0022	0.079	0.0081	0.3103	0.4682376	Mineral
DNA	nifh_Cluster1261	0.0023	-0.0007	0.0235	-0.0517	0.5858	0.6830721	Mineral
DNA	nifh_Cluster1262	0.008	-0.0009	0.0085	-0.0678	0.7437	0.8201479	Mineral
DNA	nifh_Cluster1266	0.0072	-0.0037	0.2447	0.1866	0.0609	0.2091025	Mineral
DNA	nifh_Cluster1267	0.0388	0.0275	0.2306	0.1714	0.0701	0.2239632	Mineral
DNA	nifh_Cluster127	0.0033	-0.0028	0.2167	0.1564	0.0804	0.2436161	Mineral
DNA	nifh_Cluster1278	0.2105	-0.0195	0.0264	-0.0485	0.5631	0.6678382	Mineral
DNA	nifh_Cluster128	0.0043	-0.003	0.3279	0.2762	0.0257	0.1442484	Mineral
DNA	nifh_Cluster1289	0.004	-0.0001	0.0005	-0.0764	0.9366	0.9577261	Mineral
DNA	nifh_Cluster129	0.0086	-0.0057	0.2167	0.1564	0.0804	0.2436161	Mineral
DNA	nifh_Cluster1292	0.0106	-0.002	0.0325	-0.0419	0.5203	0.6375337	Mineral

DNA	nifh_Cluster130	0.0071	-0.0064	0.2293	0.17	0.071	0.2239632	Mineral
DNA	nifh_Cluster1305	0.0048	-0.0008	0.0237	-0.0514	0.5842	0.6830721	Mineral
DNA	nifh_Cluster1306	0.0055	-0.0002	0.001	-0.0759	0.9121	0.9440729	Mineral
DNA	nifh_Cluster131	0.0067	-0.0057	0.3206	0.2683	0.0278	0.1503399	Mineral
DNA	nifh_Cluster1319	0.0048	0.0003	0.0026	-0.0741	0.8575	0.9001887	Mineral
DNA	nifh_Cluster132	0.0064	-0.0049	0.2903	0.2357	0.0382	0.1719969	Mineral
DNA	nifh_Cluster1320	0.0028	-0.0005	0.0073	-0.069	0.7617	0.8282451	Mineral
DNA	nifh_Cluster1323	0.0022	0.001	0.1139	0.0458	0.2185	0.3819279	Mineral
DNA	nifh_Cluster1324	0.0235	-0.0129	0.2941	0.2398	0.0367	0.1719969	Mineral
DNA	nifh_Cluster1336	0.0159	0.0008	0.0081	-0.0682	0.7496	0.8251186	Mineral
DNA	nifh_Cluster1340	0.0478	0.0309	0.3035	0.25	0.0333	0.1636809	Mineral
DNA	nifh_Cluster1345	0.033	0.019	0.1757	0.1123	0.1199	0.2886606	Mineral
DNA	nifh_Cluster1370	0.0022	-0.0003	0.0061	-0.0704	0.7828	0.8459277	Mineral
DNA	nifh_Cluster1375	0.0017	1.00E-04	0.0012	-0.0756	0.9008	0.9389877	Mineral
DNA	nifh_Cluster139	0.0038	0.0001	0.0005	-0.0763	0.9345	0.9572243	Mineral
DNA	nifh_Cluster140	0.0051	-0.0009	0.026	-0.0489	0.566	0.6678382	Mineral
DNA	nifh_Cluster141	0.0808	-0.0107	0.1555	0.0905	0.1458	0.3106116	Mineral
DNA	nifh_Cluster148	0.006	0.003	0.0964	0.0269	0.26	0.4272382	Mineral
DNA	nifh_Cluster1480	0.0006	0.001	0.4345	0.3911	0.0075	0.0791684	Mineral
DNA	nifh_Cluster152	0.0602	-0.0073	0.0339	-0.0404	0.5111	0.6321201	Mineral
DNA	nifh_Cluster156	0.0459	0.0072	0.0509	-0.0221	0.4188	0.5575554	Mineral
DNA	nifh_Cluster1562	0.0293	0.0286	0.3498	0.2998	0.0202	0.1273795	Mineral
DNA	nifh_Cluster158	0.019	-0.005	0.0768	0.0058	0.3173	0.4703899	Mineral
DNA	nifh_Cluster16	0.0023	-0.0021	0.2149	0.1545	0.0818	0.2449362	Mineral
DNA	nifh_Cluster166	0.0165	-0.0128	0.2442	0.1861	0.0611	0.2091025	Mineral
DNA	nifh_Cluster205	0.0121	-0.0019	0.0453	-0.0282	0.4464	0.5763132	Mineral
DNA	nifh_Cluster21	0.0022	-0.0008	0.0563	-0.0163	0.3944	0.5402231	Mineral
DNA	nifh_Cluster222	0.0069	-0.0053	0.2032	0.1419	0.0917	0.25399	Mineral
DNA	nifh_Cluster225	0.021	0.0015	0.0119	-0.0641	0.6987	0.7822031	Mineral
DNA	nifh_Cluster230	0.0052	-0.0024	0.1836	0.1208	0.111	0.2836198	Mineral
DNA	nifh_Cluster231	0.0019	-0.0005	0.0189	-0.0566	0.6252	0.7203925	Mineral
DNA	nifh_Cluster236	0.0034	-0.0012	0.0809	0.0102	0.3041	0.4656185	Mineral
DNA	nifh_Cluster237	0.007	-0.0041	0.3219	0.2698	0.0274	0.1502208	Mineral
DNA	nifh_Cluster243	0.0253	-0.0097	0.1	0.0307	0.2509	0.4181748	Mineral
DNA	nifh_Cluster2432	0.0317	0.019	0.2034	0.1421	0.0916	0.25399	Mineral
DNA	nifh_Cluster2433	0.0028	0.0005	0.0075	-0.0688	0.7584	0.8282451	Mineral
DNA	nifh_Cluster246	0.0068	-0.0036	0.1482	0.0827	0.1565	0.3228616	Mineral
DNA	nifh_Cluster25	0.0195	-0.0022	0.0331	-0.0413	0.5163	0.6345873	Mineral
DNA	nifh_Cluster256	0.0023	-0.002	0.3145	0.2617	0.0297	0.1548238	Mineral
DNA	nifh_Cluster264	0.0025	-0.0013	0.1611	0.0966	0.1381	0.3058564	Mineral
DNA	nifh_Cluster265	0.0702	-0.0007	0.0006	-0.0762	0.9282	0.9547614	Mineral
DNA	nifh_Cluster267	0.0071	0.0002	0.0006	-0.0762	0.9289	0.9547614	Mineral
DNA	nifh_Cluster268	0.0109	0.00E+00	0.00E+00	-0.0769	0.991	0.9918192	Mineral
DNA	nifh_Cluster269	0.0283	-0.0088	0.0896	0.0196	0.2784	0.4404301	Mineral
DNA	nifh_Cluster272	0.0012	0.0009	0.1281	0.061	0.1903	0.3586982	Mineral
DNA	nifh_Cluster274	0.0014	-0.0012	0.0773	0.0064	0.3156	0.4690363	Mineral
DNA	nifh_Cluster275	0.0051	-0.0008	0.0324	-0.042	0.5208	0.6375337	Mineral
DNA	nifh_Cluster278	0.0056	0.0032	0.207	0.146	0.0883	0.2509425	Mineral
DNA	nifh_Cluster279	0.0058	0.0001	0.0005	-0.0764	0.9383	0.9578058	Mineral
DNA	nifh_Cluster28	0.0017	-0.0009	0.0836	0.0131	0.2958	0.4581236	Mineral
DNA	nifh_Cluster280	0.0034	0.0021	0.0924	0.0226	0.2708	0.4351912	Mineral
DNA	nifh_Cluster282	0.0052	-0.0009	0.0297	-0.045	0.5393	0.652791	Mineral
DNA	nifh_Cluster287	0.003	0.0007	0.0344	-0.0399	0.5081	0.629778	Mineral
DNA	nifh_Cluster290	0.0096	-0.0058	0.223	0.1632	0.0755	0.2344914	Mineral
DNA	nifh_Cluster297	0.004	-0.0043	0.2793	0.2239	0.0428	0.18181	Mineral

DNA	nifh_Cluster299	0.0025	-0.001	0.0698	-0.0018	0.3415	0.4950147	Mineral
DNA	nifh_Cluster303	0.002	-0.0006	0.0391	-0.0349	0.4802	0.6027989	Mineral
DNA	nifh_Cluster304	0.0025	-0.0025	0.4022	0.3562	0.0111	0.0911012	Mineral
DNA	nifh_Cluster313	0.0129	-0.01	0.2497	0.1919	0.0579	0.2091025	Mineral
DNA	nifh_Cluster314	0.0027	-0.0014	0.1183	0.0504	0.2095	0.3771789	Mineral
DNA	nifh_Cluster317	0.0089	-0.005	0.3154	0.2627	0.0294	0.1547132	Mineral
DNA	nifh_Cluster32	0.0029	-0.0018	0.2637	0.207	0.0503	0.1989872	Mineral
DNA	nifh_Cluster320	13.944	-2.2697	0.7355	0.7152	0.00E+00	0.0072041	Mineral
DNA	nifh_Cluster334	0.0084	-0.0049	0.1201	0.0524	0.2057	0.3756734	Mineral
DNA	nifh_Cluster335	0.0059	-0.0038	0.143	0.077	0.1647	0.3274137	Mineral
DNA	nifh_Cluster338	0.0151	0.0001	1.00E-04	-0.0768	0.9732	0.9825338	Mineral
DNA	nifh_Cluster340	0.0041	-0.0009	0.04	-0.0339	0.4749	0.5999633	Mineral
DNA	nifh_Cluster341	0.0021	-0.0007	0.0358	-0.0384	0.4994	0.6215622	Mineral
DNA	nifh_Cluster35	0.0079	0.0027	0.1686	0.1047	0.1284	0.2947353	Mineral
DNA	nifh_Cluster351	0.0035	-0.0021	0.1513	0.0861	0.1518	0.3187596	Mineral
DNA	nifh_Cluster3574	0.1191	0.0156	0.0616	-0.0106	0.3726	0.5239593	Mineral
DNA	nifh_Cluster3691	0.0224	0.0056	0.0527	-0.0202	0.4106	0.5556614	Mineral
DNA	nifh_Cluster37	0.0374	-0.0221	0.2897	0.235	0.0385	0.1719969	Mineral
DNA	nifh_Cluster374	0.0029	0.0024	0.2731	0.2172	0.0456	0.1910026	Mineral
DNA	nifh_Cluster38	0.0008	-0.0004	0.0605	-0.0118	0.3769	0.5256565	Mineral
DNA	nifh_Cluster382	0.0023	-0.0024	0.2165	0.1562	0.0805	0.2436161	Mineral
DNA	nifh_Cluster384	0.0023	-0.0023	0.4211	0.3766	0.0089	0.0821001	Mineral
DNA	nifh_Cluster386	0.0041	-0.0023	0.0991	0.0298	0.2532	0.4196296	Mineral
DNA	nifh_Cluster389	0.003	-0.0022	0.1152	0.0471	0.2159	0.3816213	Mineral
DNA	nifh_Cluster394	0.0027	-0.002	0.1244	0.0571	0.1972	0.3669523	Mineral
DNA	nifh_Cluster40	0.0014	-0.0006	0.0574	-0.0151	0.3899	0.5387079	Mineral
DNA	nifh_Cluster43	0.0107	-0.0026	0.0883	0.0182	0.282	0.4448802	Mineral
DNA	nifh_Cluster434	0.0048	-0.0008	0.016	-0.0597	0.6537	0.7402484	Mineral
DNA	nifh_Cluster440	0.0171	0.0013	0.005	-0.0716	0.803	0.8582317	Mineral
DNA	nifh_Cluster462	0.0048	-0.001	0.0235	-0.0516	0.5851	0.6830721	Mineral
DNA	nifh_Cluster470	0.017	0.0053	0.128	0.0609	0.1905	0.3586982	Mineral
DNA	nifh_Cluster49	0.0223	-0.0229	0.4573	0.4156	0.0056	0.0693169	Mineral
DNA	nifh_Cluster499	0.0028	-0.0023	0.2865	0.2316	0.0398	0.1756501	Mineral
DNA	nifh_Cluster5	0.0263	0.0083	0.3425	0.2919	0.0219	0.1333045	Mineral
DNA	nifh_Cluster506	0.0058	0.0017	0.0633	-0.0087	0.3656	0.5197188	Mineral
DNA	nifh_Cluster51	0.0031	-0.0022	0.137	0.0706	0.1744	0.3396259	Mineral
DNA	nifh_Cluster52	0.0396	-0.0318	0.427	0.3829	0.0083	0.0821001	Mineral
DNA	nifh_Cluster525	0.0025	-0.0009	0.0314	-0.0431	0.5276	0.6431987	Mineral
DNA	nifh_Cluster531	0.0129	0.0063	0.1586	0.0939	0.1415	0.3058564	Mineral
DNA	nifh_Cluster539	0.0056	-0.0031	0.061	-0.0112	0.3748	0.5239593	Mineral
DNA	nifh_Cluster578	0.0593	0.0092	0.0612	-0.011	0.3739	0.5239593	Mineral
DNA	nifh_Cluster58	0.2389	0.1532	0.2478	0.19	0.059	0.2091025	Mineral
DNA	nifh_Cluster60	0.011	-0.009	0.502	0.4637	0.0031	0.0555855	Mineral
DNA	nifh_Cluster61	0.0017	-0.0003	0.0169	-0.0587	0.6439	0.7362654	Mineral
DNA	nifh_Cluster65	0.0085	-0.005	0.2552	0.1979	0.0548	0.2071598	Mineral
DNA	nifh_Cluster686	0.0013	1.00E-04	0.0013	-0.0755	0.8989	0.9386551	Mineral
DNA	nifh_Cluster69	0.0092	-0.0106	0.5988	0.5679	0.0007	0.0322891	Mineral
DNA	nifh_Cluster70	0.0144	-0.0127	0.2838	0.2287	0.0409	0.1785363	Mineral
DNA	nifh_Cluster717	0.0024	0.0008	0.0469	-0.0264	0.438	0.5691642	Mineral
DNA	nifh_Cluster725	0.0035	-0.0011	0.0791	0.0082	0.31	0.4682376	Mineral
DNA	nifh_Cluster727	0.0038	0.0005	0.0095	-0.0667	0.7302	0.8098084	Mineral
DNA	nifh_Cluster73	0.0133	-0.0076	0.2409	0.1825	0.0632	0.2106617	Mineral
DNA	nifh_Cluster74	0.005	-0.0039	0.2497	0.192	0.0579	0.2091025	Mineral
DNA	nifh_Cluster748	0.0104	-1.00E-04	0.0002	-0.0767	0.9613	0.9761781	Mineral
DNA	nifh_Cluster749	0.0055	0.0016	0.0611	-0.0111	0.3745	0.5239593	Mineral

DNA	nifh_Cluster755	0.0118	0.0056	0.2078	0.1469	0.0877	0.2509425	Mineral
DNA	nifh_Cluster76	0.0033	-0.0009	0.0351	-0.0391	0.5035	0.6254406	Mineral
DNA	nifh_Cluster79	0.002	-0.0011	0.1503	0.0849	0.1534	0.3200742	Mineral
DNA	nifh_Cluster81	0.0038	-0.003	0.2474	0.1895	0.0592	0.2091025	Mineral
DNA	nifh_Cluster82	0.0014	-0.0011	0.1396	0.0734	0.1702	0.3335642	Mineral
DNA	nifh_Cluster83	0.0055	0.0012	0.0296	-0.0451	0.5399	0.652791	Mineral
DNA	nifh_Cluster86	0.0161	0.0033	0.0564	-0.0162	0.3943	0.5402231	Mineral
DNA	nifh_Cluster868	0.0098	0.0003	0.0008	-0.0761	0.921	0.949988	Mineral
DNA	nifh_Cluster87	0.003	-0.0021	0.1757	0.1123	0.1199	0.2886606	Mineral
DNA	nifh_Cluster88	0.0054	0.0028	0.1403	0.0742	0.1689	0.3333792	Mineral
DNA	nifh_Cluster9	0.0077	-0.0016	0.0222	-0.053	0.5958	0.6932878	Mineral
DNA	nifh_Cluster92	0.0068	-0.0042	0.1433	0.0774	0.1641	0.3274137	Mineral
DNA	nifh_Cluster93	0.0035	-0.0013	0.0765	0.0055	0.3182	0.4704579	Mineral
DNA	nifh_Cluster94	0.005	-0.0044	0.2989	0.245	0.0349	0.1703897	Mineral
DNA	nifh_Cluster95	0.0149	-0.0051	0.0631	-0.009	0.3667	0.5200231	Mineral
DNA	nifh_Cluster96	0.0131	0.0067	0.2463	0.1883	0.0599	0.2091025	Mineral
DNA	nifh_Cluster97	0.0125	0.0027	0.1291	0.0621	0.1884	0.3578316	Mineral
DNA	nifh_Cluster98	0.0049	0.0023	0.1845	0.1218	0.1101	0.2836134	Mineral
DNA	nifh_Cluster99	0.0035	-0.0031	0.2686	0.2123	0.0478	0.195923	Mineral
DNA	nirk_Cluster0	6.5686	-0.5793	0.3493	0.2993	0.0203	0.1273795	Mineral
DNA	nirk_Cluster1	0.2105	-0.0536	0.201	0.1395	0.0937	0.2571861	Mineral
DNA	nirk_Cluster10	0.6649	0.0769	0.1073	0.0386	0.2334	0.3996434	Mineral
DNA	nirk_Cluster101	0.004	-0.0014	0.0614	-0.0108	0.373	0.5239593	Mineral
DNA	nirk_Cluster102	0.055	-0.0197	0.2862	0.2313	0.0399	0.1756501	Mineral
DNA	nirk_Cluster103	0.09	-0.0132	0.0638	-0.0083	0.3639	0.5197188	Mineral
DNA	nirk_Cluster104	0.0028	0.0005	0.0125	-0.0634	0.6913	0.7754087	Mineral
DNA	nirk_Cluster105	0.1172	-0.043	0.3817	0.3341	0.0141	0.1053854	Mineral
DNA	nirk_Cluster106	0.073	-0.0242	0.1443	0.0785	0.1625	0.3270565	Mineral
DNA	nirk_Cluster107	0.1123	-0.0309	0.1869	0.1244	0.1075	0.2782619	Mineral
DNA	nirk_Cluster108	0.0184	-0.0057	0.1212	0.0536	0.2035	0.3728324	Mineral
DNA	nirk_Cluster109	0.0158	-0.0039	0.0938	0.0241	0.2669	0.4338811	Mineral
DNA	nirk_Cluster11	0.1509	-0.0054	0.0292	-0.0455	0.5427	0.6547633	Mineral
DNA	nirk_Cluster12	0.1052	-0.0351	0.6451	0.6178	0.0003	0.0166787	Mineral
DNA	nirk_Cluster13	0.0418	-0.0033	0.0427	-0.0309	0.4598	0.5866281	Mineral
DNA	nirk_Cluster14	0.1084	0.0063	0.0362	-0.038	0.4972	0.6202329	Mineral
DNA	nirk_Cluster15	0.4141	-0.1119	0.221	0.1611	0.077	0.2378122	Mineral
DNA	nirk_Cluster16	0.5816	-0.1584	0.1885	0.126	0.1059	0.2752756	Mineral
DNA	nirk_Cluster17	0.1044	-0.0184	0.2583	0.2013	0.0531	0.204853	Mineral
DNA	nirk_Cluster18	0.1461	-0.0766	0.6493	0.6223	0.0003	0.0166787	Mineral
DNA	nirk_Cluster19	0.2953	-0.0375	0.2067	0.1456	0.0887	0.2509425	Mineral
DNA	nirk_Cluster2	0.2004	0.0344	0.2552	0.1979	0.0548	0.2071598	Mineral
DNA	nirk_Cluster20	0.1934	-0.0156	0.0506	-0.0224	0.4201	0.5575554	Mineral
DNA	nirk_Cluster21	0.1711	-0.0189	0.1808	0.1178	0.1141	0.2882336	Mineral
DNA	nirk_Cluster22	0.1469	-0.0247	0.1588	0.0941	0.1412	0.3058564	Mineral
DNA	nirk_Cluster23	0.0729	-0.0091	0.1799	0.1169	0.115	0.2882336	Mineral
DNA	nirk_Cluster24	0.0753	0.0046	0.0264	-0.0485	0.563	0.6678382	Mineral
DNA	nirk_Cluster25	0.5519	-0.0533	0.1011	0.0319	0.2482	0.414881	Mineral
DNA	nirk_Cluster26	0.1119	-0.0172	0.2536	0.1961	0.0557	0.2091025	Mineral
DNA	nirk_Cluster27	0.2066	-0.0285	0.1076	0.039	0.2326	0.3996434	Mineral
DNA	nirk_Cluster28	0.0808	-0.0055	0.0236	-0.0515	0.5843	0.6830721	Mineral
DNA	nirk_Cluster29	0.162	0.0242	0.1493	0.0838	0.1549	0.3217849	Mineral
DNA	nirk_Cluster3	0.3427	0.0343	0.1231	0.0556	0.1999	0.3698816	Mineral
DNA	nirk_Cluster30	0.0712	-0.0159	0.1598	0.0951	0.1399	0.3058564	Mineral
DNA	nirk_Cluster31	0.1423	0.0124	0.0777	0.0067	0.3144	0.4690363	Mineral
DNA	nirk_Cluster32	0.1568	-0.0797	0.545	0.51	0.0017	0.0493561	Mineral

DNA	nirk_Cluster33	0.0812	-0.036	0.3611	0.312	0.0178	0.1208749	Mineral
DNA	nirk_Cluster34	0.0462	-0.0142	0.2465	0.1886	0.0597	0.2091025	Mineral
DNA	nirk_Cluster35	0.1282	-0.0425	0.199	0.1373	0.0956	0.2583763	Mineral
DNA	nirk_Cluster36	0.0021	-0.0007	0.0388	-0.0351	0.4815	0.6032029	Mineral
DNA	nirk_Cluster39	0.0045	0.0031	0.0942	0.0246	0.2657	0.4330766	Mineral
DNA	nirk_Cluster4	0.1521	-0.0278	0.1753	0.1119	0.1204	0.2886606	Mineral
DNA	nirk_Cluster40	0.0544	-0.0131	0.2938	0.2394	0.0369	0.1719969	Mineral
DNA	nirk_Cluster41	0.1797	-0.032	0.3772	0.3293	0.0149	0.1096246	Mineral
DNA	nirk_Cluster44	0.0756	-0.0019	0.0019	-0.0749	0.8768	0.9172221	Mineral
DNA	nirk_Cluster46	0.0193	-0.0044	0.0499	-0.0231	0.4234	0.5575554	Mineral
DNA	nirk_Cluster48	0.003	-0.0006	0.0166	-0.0591	0.6477	0.7380427	Mineral
DNA	nirk_Cluster49	0.0072	-0.0022	0.0502	-0.0228	0.4219	0.5575554	Mineral
DNA	nirk_Cluster5	0.2174	-0.0269	0.1771	0.1138	0.1182	0.2886606	Mineral
DNA	nirk_Cluster50	0.0014	-0.0007	0.0719	0.0005	0.334	0.4853745	Mineral
DNA	nirk_Cluster52	0.0551	-0.0125	0.198	0.1363	0.0965	0.2587546	Mineral
DNA	nirk_Cluster53	0.0039	-0.0031	0.2016	0.1402	0.0931	0.2567674	Mineral
DNA	nirk_Cluster54	0.0024	-0.0011	0.0901	0.0202	0.2769	0.4403707	Mineral
DNA	nirk_Cluster55	0.0028	-0.0019	0.1037	0.0348	0.2417	0.4083701	Mineral
DNA	nirk_Cluster56	0.0009	-0.0003	0.0216	-0.0536	0.6009	0.6964957	Mineral
DNA	nirk_Cluster57	0.0017	-0.0016	0.1557	0.0907	0.1456	0.3106116	Mineral
DNA	nirk_Cluster58	0.0102	0.0034	0.0921	0.0222	0.2716	0.4351912	Mineral
DNA	nirk_Cluster59	0.007	0.0007	0.0088	-0.0674	0.7392	0.8167114	Mineral
DNA	nirk_Cluster6	0.1713	0.0252	0.1625	0.098	0.1363	0.3038341	Mineral
DNA	nirk_Cluster60	0.0006	1.00E-04	0.004	-0.0726	0.8231	0.8734229	Mineral
DNA	nirk_Cluster62	0.1789	0.0006	0.00E+00	-0.0769	0.9849	0.9899792	Mineral
DNA	nirk_Cluster63	0.1586	-0.0116	0.0394	-0.0345	0.4784	0.6017842	Mineral
DNA	nirk_Cluster64	0.0043	-0.0007	0.018	-0.0576	0.6338	0.7289482	Mineral
DNA	nirk_Cluster65	0.0531	-0.0117	0.2143	0.1538	0.0823	0.2452363	Mineral
DNA	nirk_Cluster66	0.0095	-0.0053	0.4383	0.3951	0.0072	0.0782556	Mineral
DNA	nirk_Cluster67	0.0232	0.0093	0.1931	0.1311	0.1012	0.267716	Mineral
DNA	nirk_Cluster68	0.0158	-0.0016	0.0164	-0.0592	0.6489	0.7380427	Mineral
DNA	nirk_Cluster69	0.0362	-0.0036	0.0315	-0.043	0.5267	0.6431987	Mineral
DNA	nirk_Cluster7	0.096	-0.0022	0.0026	-0.0741	0.8558	0.8999912	Mineral
DNA	nirk_Cluster70	0.0892	0.0021	0.0006	-0.0763	0.9312	0.9555353	Mineral
DNA	nirk_Cluster71	0.0071	-0.0021	0.0898	0.0198	0.2778	0.4404301	Mineral
DNA	nirk_Cluster72	0.0278	-0.0035	0.0505	-0.0226	0.4209	0.5575554	Mineral
DNA	nirk_Cluster73	0.1947	-0.0749	0.7012	0.6783	1.00E-04	0.0082679	Mineral
DNA	nirk_Cluster74	0.8012	-0.0986	0.05	-0.023	0.4229	0.5575554	Mineral
DNA	nirk_Cluster75	0.0186	-0.0098	0.2064	0.1454	0.0889	0.2509425	Mineral
DNA	nirk_Cluster76	0.1377	-0.0275	0.293	0.2386	0.0372	0.1719969	Mineral
DNA	nirk_Cluster77	0.0113	0.0092	0.2116	0.1509	0.0845	0.2504863	Mineral
DNA	nirk_Cluster78	0.3126	-0.0917	0.414	0.369	0.0097	0.0837336	Mineral
DNA	nirk_Cluster79	0.0096	0.0055	0.1676	0.1036	0.1297	0.2964916	Mineral
DNA	nirk_Cluster8	0.1182	-0.0351	0.2719	0.2159	0.0462	0.1920384	Mineral
DNA	nirk_Cluster80	0.3544	-0.1536	0.7147	0.6928	1.00E-04	0.0072041	Mineral
DNA	nirk_Cluster82	0.0021	0.0001	0.0011	-0.0757	0.907	0.9421078	Mineral
DNA	nirk_Cluster83	0.0045	0.00E+00	0.00E+00	-0.0769	0.9809	0.987584	Mineral
DNA	nirk_Cluster84	0.0235	-0.0077	0.0958	0.0263	0.2615	0.4281345	Mineral
DNA	nirk_Cluster85	0.0053	0.0005	0.0074	-0.0689	0.7599	0.8282451	Mineral
DNA	nirk_Cluster86	0.0352	-0.002	0.0063	-0.0702	0.7793	0.843693	Mineral
DNA	nirk_Cluster87	0.0138	-0.0043	0.129	0.062	0.1886	0.3578316	Mineral
DNA	nirk_Cluster88	0.0035	-0.0004	0.0112	-0.0648	0.7069	0.7883638	Mineral
DNA	nirk_Cluster89	0.0087	-0.0056	0.3286	0.2769	0.0255	0.1442484	Mineral
DNA	nirk_Cluster9	0.061	-0.0126	0.1071	0.0385	0.2337	0.3996434	Mineral
DNA	nirk_Cluster90	0.0087	-0.0031	0.0804	0.0096	0.3059	0.4663759	Mineral

DNA	nirk_Cluster91	0.044	-0.0134	0.2652	0.2087	0.0495	0.1971836	Mineral
DNA	nirk_Cluster92	0.009	-0.0009	0.0075	-0.0689	0.7591	0.8282451	Mineral
DNA	nirk_Cluster93	0.0357	-0.0049	0.0368	-0.0372	0.4931	0.6164278	Mineral
DNA	nirk_Cluster94	0.0222	-0.0042	0.0618	-0.0104	0.3717	0.5239593	Mineral
DNA	nirk_Cluster95	0.0566	-0.0119	0.0831	0.0126	0.2974	0.4592737	Mineral
DNA	nirk_Cluster96	0.0588	-0.0152	0.1783	0.115	0.117	0.2886606	Mineral
DNA	nirk_Cluster97	0.0329	-0.0108	0.1716	0.1078	0.1248	0.2911174	Mineral
DNA	nirk_Cluster98	0.0225	-0.0014	0.0038	-0.0729	0.8279	0.8753505	Mineral
DNA	nirk_Cluster99	0.0074	-0.0003	0.001	-0.0758	0.9096	0.9432131	Mineral
DNA	nirs_Cluster0	0.1586	0.0126	0.0402	-0.0337	0.4739	0.5999633	Mineral
DNA	nirs_Cluster1	0.0051	0.0007	0.0073	-0.0691	0.7623	0.8282451	Mineral
DNA	nirs_Cluster14	0.0142	-0.0111	0.1793	0.1162	0.1158	0.2882336	Mineral
DNA	nirs_Cluster2	0.0174	0.0088	0.1148	0.0467	0.2167	0.3816341	Mineral
DNA	nirs_Cluster26	0.0007	-0.0004	0.0518	-0.0212	0.4147	0.5575554	Mineral
DNA	nirs_Cluster28	0.0099	-0.0092	0.2661	0.2096	0.049	0.1968588	Mineral
DNA	nirs_Cluster3	0.1914	0.0006	0.0002	-0.0768	0.9654	0.9786297	Mineral
DNA	nirs_Cluster36	0.0018	0.0015	0.0683	-0.0034	0.3469	0.5003761	Mineral
DNA	nirs_Cluster37	0.0092	-0.0072	0.0988	0.0295	0.2539	0.4196296	Mineral
DNA	nirs_Cluster4	0.0003	-0.0002	0.0418	-0.0319	0.4648	0.5897631	Mineral
DNA	nirs_Cluster50	0.0024	0.0003	0.0043	-0.0723	0.8174	0.8704803	Mineral
DNA	nirs_Cluster9	0.0376	-0.046	0.4609	0.4194	0.0054	0.067866	Mineral
DNA	norb_Cluster0	0.0848	-0.0265	0.1837	0.121	0.1109	0.2836198	Mineral
DNA	norb_Cluster1	1.2746	-0.4074	0.1476	0.082	0.1575	0.3228616	Mineral
DNA	norb_Cluster101	0.0037	-0.0034	0.3218	0.2697	0.0274	0.1502208	Mineral
DNA	norb_Cluster102	0.005	-0.0029	0.2339	0.1749	0.0678	0.2210232	Mineral
DNA	norb_Cluster103	0.0025	-0.0017	0.1623	0.0979	0.1365	0.3038341	Mineral
DNA	norb_Cluster104	0.0025	-0.0007	0.0147	-0.061	0.6664	0.7532205	Mineral
DNA	norb_Cluster106	0.1468	-0.0704	0.6381	0.6103	0.0004	0.0174363	Mineral
DNA	norb_Cluster107	0.0163	-0.0066	0.1577	0.0929	0.1428	0.3074506	Mineral
DNA	norb_Cluster108	0.0069	-0.0013	0.0396	-0.0343	0.4773	0.6017562	Mineral
DNA	norb_Cluster109	0.0282	-0.0092	0.1596	0.095	0.1401	0.3058564	Mineral
DNA	norb_Cluster11	0.0156	-0.0105	0.4463	0.4037	0.0065	0.0739741	Mineral
DNA	norb_Cluster110	0.0128	-0.0047	0.1264	0.0592	0.1935	0.3622499	Mineral
DNA	norb_Cluster112	0.0215	-0.0092	0.4369	0.3936	0.0073	0.0782556	Mineral
DNA	norb_Cluster113	0.0157	-0.0101	0.4692	0.4284	0.0048	0.0648174	Mineral
DNA	norb_Cluster114	0.0035	-0.0025	0.1162	0.0482	0.2137	0.3808736	Mineral
DNA	norb_Cluster115	0.0034	-0.0008	0.0635	-0.0085	0.3649	0.5197188	Mineral
DNA	norb_Cluster116	0.0083	-0.0036	0.1216	0.054	0.2028	0.3726935	Mineral
DNA	norb_Cluster117	0.0034	-0.0019	0.1312	0.0644	0.1845	0.3523133	Mineral
DNA	norb_Cluster118	0.0044	-0.0018	0.1031	0.0341	0.2432	0.4087649	Mineral
DNA	norb_Cluster119	0.0263	-0.0152	0.3494	0.2993	0.0203	0.1273795	Mineral
DNA	norb_Cluster12	0.005	-0.0021	0.0683	-0.0034	0.3468	0.5003761	Mineral
DNA	norb_Cluster121	0.0023	-0.002	0.1647	0.1005	0.1334	0.3003422	Mineral
DNA	norb_Cluster122	0.0184	-0.0078	0.2441	0.1859	0.0612	0.2091025	Mineral
DNA	norb_Cluster123	0.0071	-0.0038	0.2052	0.1441	0.0899	0.2526413	Mineral
DNA	norb_Cluster124	0.7452	-0.2955	0.2429	0.1847	0.0619	0.2091139	Mineral
DNA	norb_Cluster125	0.0052	-0.004	0.4807	0.4408	0.0042	0.0644571	Mineral
DNA	norb_Cluster126	0.011	-0.0074	0.4843	0.4447	0.004	0.0644571	Mineral
DNA	norb_Cluster127	0.2041	-0.0971	0.3506	0.3007	0.02	0.1273795	Mineral
DNA	norb_Cluster128	0.0123	-0.0048	0.1763	0.1129	0.1192	0.2886606	Mineral
DNA	norb_Cluster13	0.0051	-0.0053	0.3527	0.3029	0.0196	0.1273795	Mineral
DNA	norb_Cluster131	0.0209	-0.0113	0.3938	0.3472	0.0123	0.093996	Mineral
DNA	norb_Cluster132	1.2771	-0.4338	0.4193	0.3746	0.0091	0.0821998	Mineral
DNA	norb_Cluster133	0.0023	0.0007	0.0335	-0.0409	0.5141	0.6332293	Mineral
DNA	norb_Cluster135	0.0703	-0.0253	0.2524	0.1949	0.0563	0.2091025	Mineral

DNA	norb_Cluster136	0.025	-0.0044	0.0426	-0.031	0.4604	0.5866281	Mineral
DNA	norb_Cluster139	0.0391	-0.0124	0.1746	0.1111	0.1212	0.2890378	Mineral
DNA	norb_Cluster14	0.0037	-0.0033	0.4773	0.4371	0.0043	0.0646012	Mineral
DNA	norb_Cluster140	0.0485	-0.0222	0.4456	0.403	0.0065	0.0739741	Mineral
DNA	norb_Cluster142	0.0018	-0.0022	0.3044	0.2509	0.033	0.1635282	Mineral
DNA	norb_Cluster144	0.0122	-0.0008	0.0056	-0.0709	0.7917	0.8521208	Mineral
DNA	norb_Cluster146	0.0418	-0.0064	0.0563	-0.0163	0.3946	0.5402231	Mineral
DNA	norb_Cluster15	0.0502	-0.0217	0.2427	0.1845	0.0621	0.2091139	Mineral
DNA	norb_Cluster150	0.0199	-0.0029	0.0515	-0.0215	0.4161	0.5575554	Mineral
DNA	norb_Cluster155	0.1729	-0.0635	0.2457	0.1877	0.0602	0.2091025	Mineral
DNA	norb_Cluster16	0.0165	-0.0145	0.5827	0.5506	0.0009	0.0361945	Mineral
DNA	norb_Cluster164	0.0242	-0.0205	0.7373	0.7171	0.00E+00	0.0072041	Mineral
DNA	norb_Cluster165	0.0092	-0.0077	0.3753	0.3272	0.0152	0.1106506	Mineral
DNA	norb_Cluster166	0.0046	-0.0039	0.3693	0.3207	0.0163	0.1148462	Mineral
DNA	norb_Cluster169	0.0309	-0.0252	0.6764	0.6515	0.0002	0.0109647	Mineral
DNA	norb_Cluster17	0.0035	-0.0024	0.2597	0.2027	0.0523	0.2044877	Mineral
DNA	norb_Cluster170	0.0299	-0.0224	0.4673	0.4263	0.005	0.0650111	Mineral
DNA	norb_Cluster171	0.0089	-0.0094	0.5031	0.4649	0.0031	0.0555855	Mineral
DNA	norb_Cluster172	0.0179	-0.015	0.509	0.4713	0.0028	0.0555855	Mineral
DNA	norb_Cluster175	0.0595	-0.047	0.5435	0.5083	0.0017	0.0493561	Mineral
DNA	norb_Cluster176	0.0392	-0.0349	0.7152	0.6933	1.00E-04	0.0072041	Mineral
DNA	norb_Cluster177	0.0361	-0.0264	0.4714	0.4307	0.0047	0.0646012	Mineral
DNA	norb_Cluster179	0.003	0.0011	0.0473	-0.026	0.4363	0.5682575	Mineral
DNA	norb_Cluster18	0.0047	-0.0027	0.2582	0.2011	0.0531	0.204853	Mineral
DNA	norb_Cluster180	0.0133	-0.0013	0.0144	-0.0614	0.67	0.7558178	Mineral
DNA	norb_Cluster181	0.0003	-0.0002	0.0273	-0.0476	0.5565	0.6633402	Mineral
DNA	norb_Cluster182	0.0018	-0.0011	0.1345	0.0679	0.1787	0.3468896	Mineral
DNA	norb_Cluster184	0.0175	-0.0109	0.3971	0.3507	0.0118	0.0932569	Mineral
DNA	norb_Cluster186	0.0011	-0.0006	0.051	-0.022	0.4185	0.5575554	Mineral
DNA	norb_Cluster19	0.0058	-0.0025	0.1914	0.1292	0.1029	0.2698008	Mineral
DNA	norb_Cluster192	0.0221	-0.0195	0.7156	0.6937	1.00E-04	0.0072041	Mineral
DNA	norb_Cluster193	0.0022	-0.002	0.3095	0.2564	0.0313	0.1596839	Mineral
DNA	norb_Cluster197	0.0398	-0.0269	0.0872	0.017	0.2854	0.4481406	Mineral
DNA	norb_Cluster2	0.0032	-0.0025	0.2043	0.1431	0.0908	0.2538312	Mineral
DNA	norb_Cluster20	0.0665	-0.0243	0.2287	0.1694	0.0714	0.2239632	Mineral
DNA	norb_Cluster208	0.0015	-0.0017	0.2677	0.2114	0.0482	0.1959639	Mineral
DNA	norb_Cluster21	0.0542	-0.0194	0.1935	0.1314	0.1009	0.267716	Mineral
DNA	norb_Cluster211	0.0141	-0.0067	0.06	-0.0123	0.379	0.5273392	Mineral
DNA	norb_Cluster212	0.0252	-0.0239	0.4008	0.3547	0.0113	0.0913463	Mineral
DNA	norb_Cluster214	0.0163	-0.008	0.1104	0.042	0.2262	0.3914531	Mineral
DNA	norb_Cluster217	0.0186	-0.0142	0.2523	0.1948	0.0564	0.2091025	Mineral
DNA	norb_Cluster219	0.0108	-0.0096	0.245	0.1869	0.0607	0.2091025	Mineral
DNA	norb_Cluster22	0.0028	0.0007	0.0163	-0.0594	0.6505	0.7380427	Mineral
DNA	norb_Cluster220	0.0248	-0.0182	0.1704	0.1066	0.1262	0.2911174	Mineral
DNA	norb_Cluster221	0.0248	-0.0163	0.119	0.0512	0.208	0.3771789	Mineral
DNA	norb_Cluster222	0.0104	-0.0072	0.0957	0.0261	0.262	0.4281345	Mineral
DNA	norb_Cluster224	0.0238	-0.0224	0.3478	0.2976	0.0207	0.1273795	Mineral
DNA	norb_Cluster225	0.0095	-0.005	0.1711	0.1073	0.1254	0.2911174	Mineral
DNA	norb_Cluster226	0.0049	-0.0036	0.2381	0.1795	0.065	0.2142507	Mineral
DNA	norb_Cluster228	0.0009	-0.0014	0.1461	0.0804	0.1597	0.3248558	Mineral
DNA	norb_Cluster229	0.004	-0.0019	0.1123	0.044	0.222	0.3864148	Mineral
DNA	norb_Cluster230	0.0019	-0.0011	0.1019	0.0328	0.2462	0.4126367	Mineral
DNA	norb_Cluster231	0.0061	0.00E+00	0.00E+00	-0.0769	0.9898	0.9918192	Mineral
DNA	norb_Cluster232	0.0044	0.0005	0.0055	-0.071	0.7929	0.8521208	Mineral
DNA	norb_Cluster234	0.017	-0.0144	0.4809	0.441	0.0041	0.0644571	Mineral

DNA	norb_Cluster235	0.0012	-0.0006	0.0742	0.003	0.326	0.4760979	Mineral
DNA	norb_Cluster238	0.0135	-0.0082	0.1703	0.1065	0.1263	0.2911174	Mineral
DNA	norb_Cluster239	0.0109	-0.0088	0.2324	0.1734	0.0688	0.2229066	Mineral
DNA	norb_Cluster24	0.3014	-0.0895	0.1756	0.1122	0.12	0.2886606	Mineral
DNA	norb_Cluster244	0.0015	-1.00E-04	0.0009	-0.0759	0.9151	0.9455343	Mineral
DNA	norb_Cluster247	0.006	0.001	0.0273	-0.0475	0.5559	0.6633402	Mineral
DNA	norb_Cluster25	0.1263	-0.0468	0.1916	0.1294	0.1027	0.2698008	Mineral
DNA	norb_Cluster250	0.0146	-0.0068	0.2275	0.1681	0.0722	0.2254157	Mineral
DNA	norb_Cluster251	0.0064	-0.0041	0.2439	0.1858	0.0613	0.2091025	Mineral
DNA	norb_Cluster26	0.1134	-0.0445	0.23	0.1708	0.0704	0.2239632	Mineral
DNA	norb_Cluster28	0.0355	-0.0177	0.3676	0.319	0.0166	0.1150286	Mineral
DNA	norb_Cluster29	0.2454	-0.0842	0.5528	0.5184	0.0015	0.0487845	Mineral
DNA	norb_Cluster31	0.0632	-0.0117	0.0575	-0.015	0.3892	0.5387079	Mineral
DNA	norb_Cluster32	0.1401	-0.0437	0.1119	0.0436	0.2229	0.3868328	Mineral
DNA	norb_Cluster33	0.434	0.0284	0.0114	-0.0646	0.7045	0.78724	Mineral
DNA	norb_Cluster35	0.0746	-0.0271	0.2152	0.1548	0.0816	0.2449362	Mineral
DNA	norb_Cluster36	1.1002	-0.1135	0.0164	-0.0593	0.6495	0.7380427	Mineral
DNA	norb_Cluster37	0.7173	-0.0684	0.021	-0.0543	0.6061	0.6997999	Mineral
DNA	norb_Cluster38	0.0138	-0.0043	0.1654	0.1012	0.1325	0.2995799	Mineral
DNA	norb_Cluster39	0.0465	-0.0202	0.3075	0.2542	0.0319	0.1610736	Mineral
DNA	norb_Cluster40	0.2213	-0.0808	0.234	0.1751	0.0677	0.2210232	Mineral
DNA	norb_Cluster42	0.2825	-0.0381	0.0288	-0.0459	0.5453	0.6566176	Mineral
DNA	norb_Cluster44	0.0249	-0.01	0.1723	0.1087	0.1239	0.2911174	Mineral
DNA	norb_Cluster46	0.2575	-0.0488	0.0486	-0.0246	0.4299	0.563613	Mineral
DNA	norb_Cluster47	0.6453	-0.2114	0.2078	0.1469	0.0877	0.2509425	Mineral
DNA	norb_Cluster5	0.0115	-0.0023	0.0551	-0.0176	0.3998	0.5460144	Mineral
DNA	norb_Cluster51	0.0122	-0.0109	0.4207	0.3761	0.0089	0.0821001	Mineral
DNA	norb_Cluster52	0.2809	-0.0916	0.1662	0.1021	0.1315	0.2994775	Mineral
DNA	norb_Cluster56	0.0178	-0.0053	0.1328	0.0661	0.1817	0.3514734	Mineral
DNA	norb_Cluster57	0.004	-0.0015	0.1475	0.0819	0.1576	0.3228616	Mineral
DNA	norb_Cluster59	0.0322	-0.0023	0.0039	-0.0727	0.8246	0.8734229	Mineral
DNA	norb_Cluster6	0.6934	-0.0884	0.0284	-0.0463	0.5479	0.6583871	Mineral
DNA	norb_Cluster60	0.345	-0.1405	0.1313	0.0644	0.1845	0.3523133	Mineral
DNA	norb_Cluster61	0.5486	-0.3368	0.6766	0.6518	0.0002	0.0109647	Mineral
DNA	norb_Cluster62	0.003	0.0012	0.0928	0.023	0.2697	0.4351912	Mineral
DNA	norb_Cluster64	0.0407	-0.0106	0.1778	0.1145	0.1175	0.2886606	Mineral
DNA	norb_Cluster66	0.0016	-0.0007	0.0481	-0.0251	0.4322	0.5654108	Mineral
DNA	norb_Cluster67	0.0279	-0.0055	0.0572	-0.0154	0.3908	0.5387477	Mineral
DNA	norb_Cluster68	0.003	-0.0022	0.2911	0.2366	0.0379	0.1719969	Mineral
DNA	norb_Cluster69	0.0032	-0.0004	0.0073	-0.069	0.7614	0.8282451	Mineral
DNA	norb_Cluster7	0.0786	-0.0328	0.2447	0.1866	0.0609	0.2091025	Mineral
DNA	norb_Cluster70	0.1262	-0.0411	0.2691	0.2129	0.0476	0.195923	Mineral
DNA	norb_Cluster71	0.1512	-0.0633	0.5415	0.5062	0.0018	0.0493561	Mineral
DNA	norb_Cluster74	0.0387	-0.0132	0.2911	0.2366	0.0379	0.1719969	Mineral
DNA	norb_Cluster75	0.1531	-0.0577	0.5801	0.5478	0.001	0.0361945	Mineral
DNA	norb_Cluster76	0.1215	-0.0536	0.1188	0.051	0.2084	0.3771789	Mineral
DNA	norb_Cluster77	0.0492	-0.0081	0.0752	0.0041	0.3225	0.4737997	Mineral
DNA	norb_Cluster8	0.0206	-0.0113	0.3688	0.3202	0.0164	0.1148462	Mineral
DNA	norb_Cluster80	0.0021	-0.0023	0.3612	0.3121	0.0178	0.1208749	Mineral
DNA	norb_Cluster81	0.0013	-0.001	0.1727	0.109	0.1235	0.2911174	Mineral
DNA	norb_Cluster83	0.011	-0.0118	0.4046	0.3588	0.0108	0.0897808	Mineral
DNA	norb_Cluster84	0.0093	-0.003	0.079	0.0082	0.3102	0.4682376	Mineral
DNA	norb_Cluster85	0.0025	-0.0017	0.1596	0.095	0.1401	0.3058564	Mineral
DNA	norb_Cluster86	0.041	-0.0321	0.2318	0.1727	0.0692	0.2230355	Mineral
DNA	norb_Cluster88	0.0019	-0.0016	0.2004	0.1389	0.0943	0.2575746	Mineral

DNA	norb_Cluster90	0.0013	-0.0012	0.0914	0.0215	0.2733	0.4358414	Mineral
DNA	norb_Cluster91	0.7571	-0.5297	0.714	0.692	1.00E-04	0.0072041	Mineral
DNA	norb_Cluster92	0.0021	-0.0003	0.0029	-0.0738	0.8478	0.8944354	Mineral
DNA	norb_Cluster93	0.0028	-0.0016	0.0827	0.0122	0.2985	0.4598658	Mineral
DNA	norb_Cluster94	0.002	0.0007	0.0427	-0.0309	0.4598	0.5866281	Mineral
DNA	norb_Cluster96	0.0006	-0.0002	0.0175	-0.0581	0.6383	0.7326801	Mineral
DNA	norb_Cluster97	0.0162	0.0039	0.0516	-0.0213	0.4155	0.5575554	Mineral
DNA	norb_Cluster98	0.0176	-0.0123	0.3215	0.2694	0.0275	0.1502208	Mineral
DNA	nosz_Cluster0	0.0057	-0.0014	0.0476	-0.0257	0.4347	0.5674006	Mineral
DNA	nosz_Cluster10	0.0128	-0.0059	0.2099	0.1492	0.0859	0.2509425	Mineral
DNA	nosz_Cluster102	0.0057	-0.0004	0.0077	-0.0686	0.7551	0.8282451	Mineral
DNA	nosz_Cluster103	0.0496	-0.0217	0.33	0.2785	0.0251	0.1437605	Mineral
DNA	nosz_Cluster105	0.0016	0.00E+00	1.00E-04	-0.0768	0.9742	0.9825338	Mineral
DNA	nosz_Cluster106	0.0088	-0.0059	0.3388	0.288	0.0228	0.1373463	Mineral
DNA	nosz_Cluster107	0.0003	-0.0002	0.0418	-0.0319	0.4648	0.5897631	Mineral
DNA	nosz_Cluster108	0.0043	-0.0024	0.1405	0.0744	0.1686	0.3333792	Mineral
DNA	nosz_Cluster109	0.0416	-0.0104	0.2289	0.1696	0.0712	0.2239632	Mineral
DNA	nosz_Cluster11	0.0104	-0.0005	0.0021	-0.0746	0.8702	0.9119674	Mineral
DNA	nosz_Cluster110	0.0004	0.0008	0.3318	0.2804	0.0246	0.1424451	Mineral
DNA	nosz_Cluster111	0.0078	-0.005	0.4246	0.3804	0.0085	0.0821001	Mineral
DNA	nosz_Cluster116	0.0074	-0.002	0.0965	0.027	0.2597	0.4272382	Mineral
DNA	nosz_Cluster118	0.0064	-0.0038	0.1886	0.1262	0.1058	0.2752756	Mineral
DNA	nosz_Cluster119	0.0019	-0.0024	0.5106	0.4729	0.0028	0.0555855	Mineral
DNA	nosz_Cluster120	0.0083	-0.0039	0.1986	0.137	0.0959	0.2583763	Mineral
DNA	nosz_Cluster123	0.0136	-0.0106	0.5597	0.5258	0.0013	0.0464601	Mineral
DNA	nosz_Cluster128	0.005	-0.0037	0.2945	0.2402	0.0366	0.1719969	Mineral
DNA	nosz_Cluster129	0.05	-0.0174	0.4736	0.4332	0.0046	0.0646012	Mineral
DNA	nosz_Cluster13	0.0124	0.001	0.0068	-0.0696	0.7699	0.8350164	Mineral
DNA	nosz_Cluster130	0.1138	-0.0885	0.1532	0.0881	0.149	0.3140593	Mineral
DNA	nosz_Cluster132	0.004	-0.0021	0.2422	0.1839	0.0624	0.2091139	Mineral
DNA	nosz_Cluster14	0.0149	-0.006	0.1041	0.0352	0.2408	0.4082082	Mineral
DNA	nosz_Cluster16	0.008	-0.0024	0.0439	-0.0296	0.4535	0.5841986	Mineral
DNA	nosz_Cluster17	0.0427	-0.0228	0.4712	0.4306	0.0047	0.0646012	Mineral
DNA	nosz_Cluster18	0.0184	-0.0073	0.1098	0.0414	0.2276	0.3925777	Mineral
DNA	nosz_Cluster20	0.0117	-0.0083	0.2828	0.2276	0.0413	0.1785363	Mineral
DNA	nosz_Cluster21	0.0117	-0.0103	0.5043	0.4661	0.003	0.0555855	Mineral
DNA	nosz_Cluster22	0.0325	-0.0172	0.1399	0.0737	0.1697	0.3335642	Mineral
DNA	nosz_Cluster23	0.019	-0.0151	0.5122	0.4747	0.0027	0.0555855	Mineral
DNA	nosz_Cluster24	0.0019	0.0003	0.0057	-0.0708	0.7892	0.8512334	Mineral
DNA	nosz_Cluster25	0.0026	-0.0004	0.0088	-0.0674	0.7392	0.8167114	Mineral
DNA	nosz_Cluster26	0.0068	-0.0047	0.239	0.1804	0.0644	0.2135922	Mineral
DNA	nosz_Cluster27	0.007	-0.0031	0.1634	0.099	0.1351	0.303027	Mineral
DNA	nosz_Cluster28	0.0358	-0.0174	0.2906	0.236	0.0381	0.1719969	Mineral
DNA	nosz_Cluster30	0.0341	-0.0119	0.1734	0.1099	0.1226	0.2903849	Mineral
DNA	nosz_Cluster32	0.016	-0.0031	0.0432	-0.0304	0.4572	0.5864469	Mineral
DNA	nosz_Cluster33	0.0187	-0.0107	0.2464	0.1884	0.0598	0.2091025	Mineral
DNA	nosz_Cluster34	0.0387	-0.0316	0.3967	0.3503	0.0119	0.0932569	Mineral
DNA	nosz_Cluster35	0.0097	-0.004	0.1547	0.0897	0.1469	0.3117201	Mineral
DNA	nosz_Cluster36	0.0213	-0.0122	0.3476	0.2974	0.0207	0.1273795	Mineral
DNA	nosz_Cluster37	0.0077	-0.0027	0.092	0.0222	0.2717	0.4351912	Mineral
DNA	nosz_Cluster38	0.0144	-0.009	0.2081	0.1472	0.0874	0.2509425	Mineral
DNA	nosz_Cluster39	0.0653	-0.0265	0.2563	0.1991	0.0541	0.2071598	Mineral
DNA	nosz_Cluster40	0.0262	-0.0044	0.026	-0.0489	0.5658	0.6678382	Mineral
DNA	nosz_Cluster41	0.0258	-0.0078	0.0844	0.0139	0.2937	0.4566011	Mineral
DNA	nosz_Cluster42	0.03	-0.018	0.3338	0.2825	0.0241	0.1422059	Mineral

DNA	nosz_Cluster43	0.001	-0.0011	0.1743	0.1108	0.1215	0.2890378	Mineral
DNA	nosz_Cluster44	0.0068	-0.0024	0.0844	0.0139	0.2936	0.4566011	Mineral
DNA	nosz_Cluster45	0.0056	-0.0024	0.1151	0.047	0.216	0.3816213	Mineral
DNA	nosz_Cluster46	0.0082	-0.002	0.0782	0.0073	0.3127	0.4690363	Mineral
DNA	nosz_Cluster47	0.0012	-0.0008	0.144	0.0782	0.163	0.3270565	Mineral
DNA	nosz_Cluster48	0.0028	-0.0022	0.1797	0.1166	0.1153	0.2882336	Mineral
DNA	nosz_Cluster49	0.0012	0.0002	0.0043	-0.0723	0.8163	0.8704803	Mineral
DNA	nosz_Cluster5	0.004	-0.0024	0.1261	0.0589	0.194	0.3622499	Mineral
DNA	nosz_Cluster51	0.003	-0.0013	0.0919	0.022	0.2722	0.4351912	Mineral
DNA	nosz_Cluster53	0.0012	-0.0001	0.0012	-0.0756	0.9028	0.9393799	Mineral
DNA	nosz_Cluster54	0.0037	-0.0026	0.2081	0.1472	0.0874	0.2509425	Mineral
DNA	nosz_Cluster57	0.2232	-0.056	0.0871	0.0169	0.2856	0.4481406	Mineral
DNA	nosz_Cluster58	0.0053	-0.0056	0.4736	0.4331	0.0046	0.0646012	Mineral
DNA	nosz_Cluster59	0.0008	-0.0009	0.0749	0.0037	0.3236	0.4737997	Mineral
DNA	nosz_Cluster6	0.0034	-0.0017	0.0843	0.0139	0.2938	0.4566011	Mineral
DNA	nosz_Cluster60	0.0099	-0.0025	0.0433	-0.0303	0.4567	0.5864469	Mineral
DNA	nosz_Cluster61	0.0178	-0.0067	0.337	0.286	0.0233	0.1387354	Mineral
DNA	nosz_Cluster62	0.0493	-0.0133	0.0842	0.0138	0.2941	0.4566011	Mineral
DNA	nosz_Cluster64	0.0023	0.0017	0.0777	0.0068	0.3144	0.4690363	Mineral
DNA	nosz_Cluster65	0.0083	-0.0028	0.0808	0.0101	0.3046	0.4656185	Mineral
DNA	nosz_Cluster66	0.0428	-0.0112	0.0641	-0.0079	0.3625	0.5191117	Mineral
DNA	nosz_Cluster67	0.0669	-0.0159	0.0537	-0.0191	0.4061	0.5507942	Mineral
DNA	nosz_Cluster69	0.008	-0.0062	0.5356	0.4999	0.0019	0.0493561	Mineral
DNA	nosz_Cluster70	0.0655	-0.0448	0.1783	0.1151	0.1169	0.2886606	Mineral
DNA	nosz_Cluster71	0.0101	-0.0057	0.1571	0.0922	0.1436	0.3081534	Mineral
DNA	nosz_Cluster72	0.066	-0.0212	0.123	0.0555	0.2	0.3698816	Mineral
DNA	nosz_Cluster73	0.0031	-0.0017	0.1182	0.0503	0.2097	0.3771789	Mineral
DNA	nosz_Cluster74	0.0017	-0.0021	0.4208	0.3763	0.0089	0.0821001	Mineral
DNA	nosz_Cluster75	0.132	-0.1055	0.1324	0.0656	0.1825	0.3518351	Mineral
DNA	nosz_Cluster77	0.0002	1.00E-04	0.004	-0.0727	0.8237	0.8734229	Mineral
DNA	nosz_Cluster78	0.0054	-0.0039	0.4158	0.3708	0.0095	0.0832291	Mineral
DNA	nosz_Cluster79	0.0401	-0.0072	0.0513	-0.0216	0.4168	0.5575554	Mineral
DNA	nosz_Cluster8	0.0106	-0.0042	0.2618	0.2051	0.0512	0.2013827	Mineral
DNA	nosz_Cluster80	0.0039	-0.002	0.1381	0.0718	0.1725	0.3370755	Mineral
DNA	nosz_Cluster81	0.0099	-0.0044	0.0719	0.0005	0.334	0.4853745	Mineral
DNA	nosz_Cluster82	0.0023	0.0025	0.1278	0.0607	0.1909	0.3586982	Mineral
DNA	nosz_Cluster83	0.0014	-0.0021	0.2825	0.2273	0.0415	0.1785363	Mineral
DNA	nosz_Cluster85	0.0027	0.0009	0.0277	-0.0471	0.5535	0.6633402	Mineral
DNA	nosz_Cluster86	0.0038	1.00E-04	0.0002	-0.0767	0.9612	0.9761781	Mineral
DNA	nosz_Cluster87	0.0034	-0.0008	0.0307	-0.0439	0.5322	0.6474077	Mineral
DNA	nosz_Cluster88	0.0246	-0.0109	0.5049	0.4668	0.003	0.0555855	Mineral
DNA	nosz_Cluster89	0.0607	-0.0153	0.0749	0.0038	0.3235	0.4737997	Mineral
DNA	nosz_Cluster9	0.0034	-0.0017	0.0801	0.0093	0.3068	0.4665805	Mineral
DNA	nosz_Cluster90	0.0139	-0.0027	0.0773	0.0064	0.3155	0.4690363	Mineral
DNA	nosz_Cluster91	0.0232	-0.009	0.1172	0.0492	0.2118	0.3785886	Mineral
DNA	nosz_Cluster93	0.0488	-0.0275	0.4258	0.3816	0.0084	0.0821001	Mineral
DNA	nosz_Cluster95	0.0094	-0.0033	0.1429	0.0769	0.1648	0.3274137	Mineral
DNA	nosz_Cluster96	0.0064	-0.002	0.1313	0.0645	0.1843	0.3523133	Mineral
DNA	nosz_Cluster97	0.0026	-0.0009	0.0546	-0.0181	0.4017	0.5473778	Mineral
DNA	nosz_Cluster98	0.0035	0.0033	0.1054	0.0366	0.2377	0.4040847	Mineral
DNA	nosz_Cluster99	0.0061	-0.0012	0.0459	-0.0275	0.4432	0.5746355	Mineral
DNA	nrfa_Cluster10	0.0978	-0.0572	0.2975	0.2434	0.0355	0.1708502	Mineral
DNA	nrfa_Cluster100	0.1249	-0.1139	0.5139	0.4765	0.0026	0.0555855	Mineral
DNA	nrfa_Cluster104	0.1478	-0.059	0.2672	0.2108	0.0485	0.1959639	Mineral
DNA	nrfa_Cluster105	0.006	-0.0038	0.2102	0.1494	0.0856	0.2509425	Mineral

DNA	nrfa_Cluster109	0.2753	-0.0789	0.1541	0.0891	0.1478	0.3125041	Mineral
DNA	nrfa_Cluster116	0.3208	-0.1019	0.3519	0.302	0.0198	0.1273795	Mineral
DNA	nrfa_Cluster118	0.2298	-0.223	0.4959	0.4571	0.0034	0.0587245	Mineral
DNA	nrfa_Cluster125	0.1081	-0.1054	0.4463	0.4037	0.0065	0.0739741	Mineral
DNA	nrfa_Cluster154	0.0837	-0.07	0.4606	0.4191	0.0054	0.067866	Mineral
DNA	nrfa_Cluster164	0.0044	-0.0031	0.2971	0.243	0.0356	0.1708502	Mineral
DNA	nrfa_Cluster172	0.0036	-0.0033	0.2775	0.2219	0.0436	0.1839289	Mineral
DNA	nrfa_Cluster175	0.0053	-0.007	0.4444	0.4017	0.0066	0.0739741	Mineral
DNA	nrfa_Cluster20	0.045	-0.019	0.1502	0.0848	0.1535	0.3200742	Mineral
DNA	nrfa_Cluster26	0.1433	-0.1366	0.5363	0.5006	0.0019	0.0493561	Mineral
DNA	nrfa_Cluster29	0.0059	-0.0027	0.0927	0.0229	0.2698	0.4351912	Mineral
DNA	nrfa_Cluster34	0.0642	-0.0312	0.2091	0.1483	0.0866	0.2509425	Mineral
DNA	nrfa_Cluster38	0.0033	-0.0039	0.3707	0.3223	0.016	0.1148462	Mineral
DNA	nrfa_Cluster61	0.0047	-0.0022	0.0991	0.0299	0.253	0.4196296	Mineral
DNA	nrfa_Cluster62	0.0589	-0.0588	0.4544	0.4124	0.0059	0.0704794	Mineral
DNA	nrfa_Cluster66	0.1282	-0.0524	0.3854	0.3381	0.0135	0.102304	Mineral
DNA	nrfa_Cluster69	0.0035	-0.0021	0.1449	0.0791	0.1616	0.3270565	Mineral
DNA	nrfa_Cluster71	0.0322	-0.0259	0.3587	0.3094	0.0183	0.1227947	Mineral
DNA	nrfa_Cluster72	0.0969	-0.071	0.4271	0.3831	0.0082	0.0821001	Mineral
DNA	nrfa_Cluster73	0.0042	-0.0041	0.3091	0.2559	0.0314	0.1596839	Mineral
DNA	nrfa_Cluster74	0.1692	-0.1405	0.5313	0.4952	0.0021	0.0504317	Mineral
DNA	nrfa_Cluster76	0.0922	-0.0897	0.5793	0.5469	0.001	0.0361945	Mineral
DNA	nrfa_Cluster79	0.0138	-0.0124	0.1714	0.1076	0.125	0.2911174	Mineral
DNA	nrfa_Cluster87	0.0207	-0.0166	0.2182	0.1581	0.0791	0.2431902	Mineral
DNA	nrfa_Cluster89	0.0023	-0.0024	0.2803	0.2249	0.0424	0.1812709	Mineral
DNA	nrfa_Cluster92	0.0041	-0.0033	0.4117	0.3665	0.0099	0.0838368	Mineral
DNA	nrfa_Cluster93	0.0581	-0.031	0.1187	0.0509	0.2085	0.3771789	Mineral
DNA	nrfa_Cluster94	0.0003	-0.0005	0.1461	0.0804	0.1597	0.3248558	Mineral
DNA	nrfa_Cluster97	0.0225	-0.0133	0.3945	0.3479	0.0122	0.093996	Mineral
DNA	nrfa_Cluster99	0.0184	-0.0127	0.4115	0.3662	0.0099	0.0838368	Mineral
Water_content	amoA_A_Cluster26	0.0053	-0.004	0.2133	0.1528	0.0831	0.865544	Mineral
Water_content	amoA_A_Cluster45	0.0071	-0.003	0.1026	0.0336	0.2444	0.9957818	Mineral
Water_content	amoA_B_Cluster0	0.1272	-0.0095	0.0465	-0.0269	0.4404	0.9957818	Mineral
Water_content	amoA_B_Cluster1	0.0205	-0.0011	0.0046	-0.072	0.8108	0.9957818	Mineral
Water_content	amoA_B_Cluster10	0.0539	-0.0171	0.437	0.3937	0.0073	0.3306417	Mineral
Water_content	amoA_B_Cluster11	0.0486	-0.0046	0.0232	-0.0519	0.5876	0.9957818	Mineral
Water_content	amoA_B_Cluster12	0.093	-0.0205	0.294	0.2397	0.0368	0.7734922	Mineral
Water_content	amoA_B_Cluster13	0.6417	-0.1686	0.2137	0.1532	0.0828	0.865544	Mineral
Water_content	amoA_B_Cluster14	0.0339	-0.0106	0.143	0.0771	0.1645	0.9244709	Mineral
Water_content	amoA_B_Cluster15	0.0413	-0.0082	0.0986	0.0293	0.2543	0.9957818	Mineral
Water_content	amoA_B_Cluster2	0.2143	-0.0589	0.2479	0.1901	0.0589	0.865544	Mineral
Water_content	amoA_B_Cluster20	0.0078	0.0008	0.0064	-0.0701	0.7774	0.9957818	Mineral
Water_content	amoA_B_Cluster23	0.0626	-0.0065	0.0407	-0.033	0.4706	0.9957818	Mineral
Water_content	amoA_B_Cluster3	0.0244	-0.0049	0.0588	-0.0135	0.3837	0.9957818	Mineral
Water_content	amoA_B_Cluster4	0.0169	-0.0065	0.1778	0.1145	0.1175	0.8886646	Mineral
Water_content	amoA_B_Cluster5	0.1032	-0.0133	0.1144	0.0462	0.2176	0.9467997	Mineral
Water_content	amoA_B_Cluster6	0.0279	-0.0008	0.0022	-0.0745	0.8674	0.9957818	Mineral
Water_content	amoA_B_Cluster7	0.0232	0.0012	0.0033	-0.0734	0.8393	0.9957818	Mineral
Water_content	amoA_B_Cluster8	0.0268	-0.0021	0.0074	-0.0689	0.7603	0.9957818	Mineral
Water_content	amoA_B_Cluster9	0.0693	-0.0055	0.0114	-0.0647	0.7053	0.9957818	Mineral
Water_content	nifh_Cluster0	0.0318	0.0071	0.1662	0.1021	0.1314	0.8913283	Mineral
Water_content	nifh_Cluster10	0.0033	0.0007	0.0249	-0.0501	0.574	0.9957818	Mineral
Water_content	nifh_Cluster100	0.002	-1.00E-04	0.0004	-0.0765	0.947	0.9957818	Mineral
Water_content	nifh_Cluster103	0.0433	-0.0147	0.3175	0.265	0.0287	0.6596202	Mineral
Water_content	nifh_Cluster1034	0.0337	-0.0033	0.0099	-0.0663	0.7242	0.9957818	Mineral

Water_content	nifh_Cluster105	0.0164	-0.0018	0.0108	-0.0653	0.7122	0.9957818	Mineral
Water_content	nifh_Cluster108	0.0076	-0.0027	0.1257	0.0584	0.1948	0.9455824	Mineral
Water_content	nifh_Cluster1099	0.0478	0.0244	0.1508	0.0855	0.1526	0.9129331	Mineral
Water_content	nifh_Cluster11	0.0025	-0.0002	0.0053	-0.0712	0.7961	0.9957818	Mineral
Water_content	nifh_Cluster1112	0.0037	-0.0008	0.0192	-0.0563	0.6225	0.9957818	Mineral
Water_content	nifh_Cluster112	0.0098	0.0091	0.4808	0.4409	0.0041	0.2227812	Mineral
Water_content	nifh_Cluster113	0.0374	0.0009	0.0021	-0.0747	0.8711	0.9957818	Mineral
Water_content	nifh_Cluster114	0.0304	0.0026	0.0353	-0.0389	0.5023	0.9957818	Mineral
Water_content	nifh_Cluster1141	0.0296	0.002	0.0065	-0.0699	0.7751	0.9957818	Mineral
Water_content	nifh_Cluster115	0.0098	-0.0017	0.0445	-0.029	0.4502	0.9957818	Mineral
Water_content	nifh_Cluster116	0.0883	0.0002	0.00E+00	-0.0769	0.9896	0.9977707	Mineral
Water_content	nifh_Cluster1163	0.0051	1.00E-04	0.0001	-0.0768	0.9714	0.9962612	Mineral
Water_content	nifh_Cluster1179	0.0037	0.0001	0.0003	-0.0766	0.9509	0.9957818	Mineral
Water_content	nifh_Cluster1182	0.0209	-0.0005	0.0029	-0.0738	0.8479	0.9957818	Mineral
Water_content	nifh_Cluster1183	0.0045	0.0021	0.2493	0.1916	0.0581	0.865544	Mineral
Water_content	nifh_Cluster1197	0.0115	-0.0038	0.1245	0.0571	0.1971	0.9455824	Mineral
Water_content	nifh_Cluster1199	0.0048	0.002	0.1141	0.0459	0.2182	0.9467997	Mineral
Water_content	nifh_Cluster120	0.02	0.0017	0.0029	-0.0738	0.8496	0.9957818	Mineral
Water_content	nifh_Cluster1203	0.0217	-0.0082	0.164	0.0997	0.1343	0.8935183	Mineral
Water_content	nifh_Cluster1206	0.006	-0.0005	0.0078	-0.0685	0.7544	0.9957818	Mineral
Water_content	nifh_Cluster1207	0.0078	-0.0003	0.001	-0.0758	0.9094	0.9957818	Mineral
Water_content	nifh_Cluster1209	0.1617	-0.0073	0.014	-0.0619	0.675	0.9957818	Mineral
Water_content	nifh_Cluster121	0.0123	-0.005	0.0659	-0.0059	0.3557	0.9957818	Mineral
Water_content	nifh_Cluster1211	0.0068	0.0002	0.0012	-0.0756	0.9019	0.9957818	Mineral
Water_content	nifh_Cluster1213	0.0032	-0.0013	0.1343	0.0677	0.179	0.94319	Mineral
Water_content	nifh_Cluster122	0.0041	-0.0012	0.046	-0.0274	0.4429	0.9957818	Mineral
Water_content	nifh_Cluster123	0.0092	-0.0013	0.0095	-0.0667	0.7303	0.9957818	Mineral
Water_content	nifh_Cluster1236	0.031	-0.0147	0.1252	0.0579	0.1958	0.9455824	Mineral
Water_content	nifh_Cluster1238	0.0034	-0.0005	0.01	-0.0662	0.7231	0.9957818	Mineral
Water_content	nifh_Cluster124	0.0101	-0.0002	0.0007	-0.0762	0.9267	0.9957818	Mineral
Water_content	nifh_Cluster1261	0.0023	-0.0006	0.017	-0.0586	0.6428	0.9957818	Mineral
Water_content	nifh_Cluster1262	0.008	0.0013	0.0189	-0.0566	0.6253	0.9957818	Mineral
Water_content	nifh_Cluster1266	0.0072	-0.0019	0.0662	-0.0056	0.3545	0.9957818	Mineral
Water_content	nifh_Cluster1267	0.0388	0.0367	0.4106	0.3653	0.0101	0.3954977	Mineral
Water_content	nifh_Cluster127	0.0033	0.0003	0.0024	-0.0743	0.8623	0.9957818	Mineral
Water_content	nifh_Cluster1278	0.2105	-0.0049	0.0016	-0.0752	0.8861	0.9957818	Mineral
Water_content	nifh_Cluster128	0.0043	-0.0007	0.0187	-0.0568	0.6267	0.9957818	Mineral
Water_content	nifh_Cluster1289	0.004	-0.0004	0.0064	-0.0701	0.7776	0.9957818	Mineral
Water_content	nifh_Cluster129	0.0086	-0.0003	0.0007	-0.0761	0.9231	0.9957818	Mineral
Water_content	nifh_Cluster1292	0.0106	-0.001	0.0087	-0.0675	0.7408	0.9957818	Mineral
Water_content	nifh_Cluster130	0.0071	-0.0022	0.0278	-0.0469	0.5523	0.9957818	Mineral
Water_content	nifh_Cluster1305	0.0048	-0.0003	0.0034	-0.0732	0.8359	0.9957818	Mineral
Water_content	nifh_Cluster1306	0.0055	0.0001	0.0004	-0.0764	0.9404	0.9957818	Mineral
Water_content	nifh_Cluster131	0.0067	-0.0006	0.003	-0.0736	0.8451	0.9957818	Mineral
Water_content	nifh_Cluster1319	0.0048	-0.0009	0.0206	-0.0548	0.6101	0.9957818	Mineral
Water_content	nifh_Cluster132	0.0064	-0.0006	0.0051	-0.0715	0.8007	0.9957818	Mineral
Water_content	nifh_Cluster1320	0.0028	0.0003	0.0023	-0.0744	0.8642	0.9957818	Mineral
Water_content	nifh_Cluster1323	0.0022	-0.0004	0.0155	-0.0603	0.6589	0.9957818	Mineral
Water_content	nifh_Cluster1324	0.0235	-0.0052	0.0483	-0.0249	0.4314	0.9957818	Mineral
Water_content	nifh_Cluster1336	0.0159	0.0004	0.002	-0.0748	0.8749	0.9957818	Mineral
Water_content	nifh_Cluster1340	0.0478	-0.0013	0.0006	-0.0763	0.9335	0.9957818	Mineral
Water_content	nifh_Cluster1345	0.033	0.0241	0.2835	0.2284	0.041	0.7734922	Mineral
Water_content	nifh_Cluster1370	0.0022	0.0006	0.0208	-0.0545	0.6083	0.9957818	Mineral
Water_content	nifh_Cluster1375	0.0017	0.0002	0.008	-0.0683	0.7514	0.9957818	Mineral
Water_content	nifh_Cluster139	0.0038	-0.001	0.0267	-0.0482	0.561	0.9957818	Mineral

Water_content	nifh_Cluster140	0.0051	-0.0004	0.004	-0.0726	0.8218	0.9957818	Mineral
Water_content	nifh_Cluster141	0.0808	-0.0144	0.2813	0.226	0.042	0.7734922	Mineral
Water_content	nifh_Cluster148	0.006	-0.0004	0.0022	-0.0746	0.869	0.9957818	Mineral
Water_content	nifh_Cluster1480	0.0006	0.0012	0.5359	0.5002	0.0019	0.2227812	Mineral
Water_content	nifh_Cluster152	0.0602	0.0029	0.0056	-0.0709	0.7913	0.9957818	Mineral
Water_content	nifh_Cluster156	0.0459	0.02	0.3944	0.3478	0.0122	0.4315249	Mineral
Water_content	nifh_Cluster1562	0.0293	0.0383	0.6267	0.598	0.0004	0.1289925	Mineral
Water_content	nifh_Cluster158	0.019	-0.0055	0.0923	0.0225	0.2709	0.9957818	Mineral
Water_content	nifh_Cluster16	0.0023	-0.0013	0.0775	0.0065	0.3151	0.9957818	Mineral
Water_content	nifh_Cluster166	0.0165	-0.0008	0.001	-0.0759	0.912	0.9957818	Mineral
Water_content	nifh_Cluster205	0.0121	-0.0013	0.02	-0.0554	0.6154	0.9957818	Mineral
Water_content	nifh_Cluster21	0.0022	-0.001	0.0823	0.0117	0.2999	0.9957818	Mineral
Water_content	nifh_Cluster222	0.0069	-0.0006	0.0025	-0.0742	0.8583	0.9957818	Mineral
Water_content	nifh_Cluster225	0.021	0.0016	0.0136	-0.0623	0.6793	0.9957818	Mineral
Water_content	nifh_Cluster230	0.0052	-0.0006	0.0129	-0.063	0.6867	0.9957818	Mineral
Water_content	nifh_Cluster231	0.0019	0.0002	0.0023	-0.0744	0.8642	0.9957818	Mineral
Water_content	nifh_Cluster236	0.0034	-0.0011	0.0673	-0.0044	0.3505	0.9957818	Mineral
Water_content	nifh_Cluster237	0.007	-0.002	0.0824	0.0118	0.2997	0.9957818	Mineral
Water_content	nifh_Cluster243	0.0253	-0.0031	0.0101	-0.0661	0.7222	0.9957818	Mineral
Water_content	nifh_Cluster2432	0.0317	0.0246	0.3414	0.2907	0.0222	0.6064434	Mineral
Water_content	nifh_Cluster2433	0.0028	0.0001	0.0004	-0.0765	0.9432	0.9957818	Mineral
Water_content	nifh_Cluster246	0.0068	0.00E+00	0.00E+00	-0.0769	0.9951	0.9983685	Mineral
Water_content	nifh_Cluster25	0.0195	0.0007	0.0037	-0.0729	0.8289	0.9957818	Mineral
Water_content	nifh_Cluster256	0.0023	-0.0015	0.1718	0.1081	0.1245	0.8913283	Mineral
Water_content	nifh_Cluster264	0.0025	0.00E+00	0.0002	-0.0767	0.9576	0.9957818	Mineral
Water_content	nifh_Cluster265	0.0702	0.0097	0.1196	0.0518	0.2068	0.9467997	Mineral
Water_content	nifh_Cluster267	0.0071	-0.0002	0.0007	-0.0762	0.9275	0.9957818	Mineral
Water_content	nifh_Cluster268	0.0109	0.0029	0.1751	0.1116	0.1206	0.8913283	Mineral
Water_content	nifh_Cluster269	0.0283	-0.007	0.0573	-0.0152	0.3902	0.9957818	Mineral
Water_content	nifh_Cluster272	0.0012	0.0014	0.2824	0.2272	0.0415	0.7734922	Mineral
Water_content	nifh_Cluster274	0.0014	-0.0001	0.0009	-0.0759	0.915	0.9957818	Mineral
Water_content	nifh_Cluster275	0.0051	-0.0015	0.1012	0.0321	0.2478	0.9957818	Mineral
Water_content	nifh_Cluster278	0.0056	0.0051	0.5165	0.4793	0.0025	0.2227812	Mineral
Water_content	nifh_Cluster279	0.0058	0.0028	0.199	0.1374	0.0956	0.8886646	Mineral
Water_content	nifh_Cluster28	0.0017	-0.0007	0.0607	-0.0116	0.3761	0.9957818	Mineral
Water_content	nifh_Cluster280	0.0034	-0.0005	0.0056	-0.0709	0.7903	0.9957818	Mineral
Water_content	nifh_Cluster282	0.0052	-0.0002	0.0013	-0.0755	0.8968	0.9957818	Mineral
Water_content	nifh_Cluster287	0.003	0.001	0.0635	-0.0086	0.365	0.9957818	Mineral
Water_content	nifh_Cluster290	0.0096	-0.0039	0.1012	0.0321	0.2478	0.9957818	Mineral
Water_content	nifh_Cluster297	0.004	-0.0022	0.0768	0.0058	0.3173	0.9957818	Mineral
Water_content	nifh_Cluster299	0.0025	0.0006	0.0218	-0.0535	0.5999	0.9957818	Mineral
Water_content	nifh_Cluster303	0.002	0.0006	0.0351	-0.0392	0.504	0.9957818	Mineral
Water_content	nifh_Cluster304	0.0025	-0.0011	0.0873	0.0171	0.285	0.9957818	Mineral
Water_content	nifh_Cluster313	0.0129	-1.00E-04	0.00E+00	-0.0769	0.991	0.9977707	Mineral
Water_content	nifh_Cluster314	0.0027	0.0005	0.016	-0.0597	0.653	0.9957818	Mineral
Water_content	nifh_Cluster317	0.0089	-0.0043	0.2312	0.1721	0.0696	0.865544	Mineral
Water_content	nifh_Cluster32	0.0029	-0.0005	0.023	-0.0521	0.5894	0.9957818	Mineral
Water_content	nifh_Cluster320	13.944	-1.2053	0.2074	0.1464	0.088	0.865544	Mineral
Water_content	nifh_Cluster334	0.0084	0.0024	0.0282	-0.0466	0.5499	0.9957818	Mineral
Water_content	nifh_Cluster335	0.0059	0.0003	0.001	-0.0758	0.9104	0.9957818	Mineral
Water_content	nifh_Cluster338	0.0151	-0.0012	0.0093	-0.0669	0.7319	0.9957818	Mineral
Water_content	nifh_Cluster340	0.0041	-0.0009	0.0381	-0.0359	0.4859	0.9957818	Mineral
Water_content	nifh_Cluster341	0.0021	0.0005	0.0216	-0.0537	0.6013	0.9957818	Mineral
Water_content	nifh_Cluster35	0.0079	0.0006	0.0091	-0.0671	0.7346	0.9957818	Mineral
Water_content	nifh_Cluster351	0.0035	0.0008	0.0188	-0.0567	0.6265	0.9957818	Mineral

Water_content	nifh_Cluster3574	0.1191	-0.0016	0.0007	-0.0762	0.9267	0.9957818	Mineral
Water_content	nifh_Cluster3691	0.0224	-0.0038	0.0236	-0.0515	0.5849	0.9957818	Mineral
Water_content	nifh_Cluster37	0.0374	-0.0049	0.0142	-0.0616	0.6718	0.9957818	Mineral
Water_content	nifh_Cluster374	0.0029	-1.00E-04	0.0002	-0.0767	0.9612	0.9957818	Mineral
Water_content	nifh_Cluster38	0.0008	0.0001	0.0036	-0.0731	0.8325	0.9957818	Mineral
Water_content	nifh_Cluster382	0.0023	-0.0012	0.0543	-0.0185	0.4035	0.9957818	Mineral
Water_content	nifh_Cluster384	0.0023	-0.0007	0.0385	-0.0355	0.4833	0.9957818	Mineral
Water_content	nifh_Cluster386	0.0041	0.0012	0.0259	-0.0491	0.5669	0.9957818	Mineral
Water_content	nifh_Cluster389	0.003	0.0007	0.0128	-0.0632	0.6883	0.9957818	Mineral
Water_content	nifh_Cluster394	0.0027	0.0004	0.0049	-0.0716	0.8036	0.9957818	Mineral
Water_content	nifh_Cluster40	0.0014	-0.0003	0.0138	-0.062	0.6764	0.9957818	Mineral
Water_content	nifh_Cluster43	0.0107	0.0008	0.0085	-0.0677	0.7436	0.9957818	Mineral
Water_content	nifh_Cluster434	0.0048	-0.0014	0.0459	-0.0274	0.443	0.9957818	Mineral
Water_content	nifh_Cluster440	0.0171	0.0013	0.0047	-0.0719	0.8081	0.9957818	Mineral
Water_content	nifh_Cluster462	0.0048	0.0013	0.0378	-0.0363	0.4877	0.9957818	Mineral
Water_content	nifh_Cluster470	0.017	0.0023	0.0232	-0.0519	0.5875	0.9957818	Mineral
Water_content	nifh_Cluster49	0.0223	-0.0092	0.0731	0.0018	0.3296	0.9957818	Mineral
Water_content	nifh_Cluster499	0.0028	-0.0001	0.0009	-0.076	0.9175	0.9957818	Mineral
Water_content	nifh_Cluster5	0.0263	0.005	0.1236	0.0562	0.1989	0.9461457	Mineral
Water_content	nifh_Cluster506	0.0058	0.001	0.0227	-0.0524	0.5916	0.9957818	Mineral
Water_content	nifh_Cluster51	0.0031	-0.0005	0.0081	-0.0682	0.7494	0.9957818	Mineral
Water_content	nifh_Cluster52	0.0396	-0.0049	0.0101	-0.066	0.721	0.9957818	Mineral
Water_content	nifh_Cluster525	0.0025	0.0002	0.0013	-0.0755	0.898	0.9957818	Mineral
Water_content	nifh_Cluster531	0.0129	0.0114	0.5254	0.4889	0.0022	0.2227812	Mineral
Water_content	nifh_Cluster539	0.0056	0.0012	0.009	-0.0673	0.737	0.9957818	Mineral
Water_content	nifh_Cluster578	0.0593	0.0103	0.0763	0.0053	0.3188	0.9957818	Mineral
Water_content	nifh_Cluster58	0.2389	0.2134	0.4805	0.4405	0.0042	0.2227812	Mineral
Water_content	nifh_Cluster60	0.011	-0.0034	0.073	0.0017	0.3302	0.9957818	Mineral
Water_content	nifh_Cluster61	0.0017	0.0006	0.0496	-0.0235	0.425	0.9957818	Mineral
Water_content	nifh_Cluster65	0.0085	0.0003	0.0012	-0.0757	0.9033	0.9957818	Mineral
Water_content	nifh_Cluster686	0.0013	0.0003	0.0146	-0.0612	0.668	0.9957818	Mineral
Water_content	nifh_Cluster69	0.0092	-0.0059	0.1868	0.1243	0.1076	0.8886646	Mineral
Water_content	nifh_Cluster70	0.0144	-0.0029	0.0152	-0.0606	0.6617	0.9957818	Mineral
Water_content	nifh_Cluster717	0.0024	0.0003	0.0059	-0.0706	0.7857	0.9957818	Mineral
Water_content	nifh_Cluster725	0.0035	0.0003	0.0049	-0.0716	0.8036	0.9957818	Mineral
Water_content	nifh_Cluster727	0.0038	-0.0003	0.0043	-0.0723	0.816	0.9957818	Mineral
Water_content	nifh_Cluster73	0.0133	-0.0013	0.0068	-0.0696	0.7696	0.9957818	Mineral
Water_content	nifh_Cluster74	0.005	-0.001	0.0174	-0.0582	0.6391	0.9957818	Mineral
Water_content	nifh_Cluster748	0.0104	0.001	0.0196	-0.0558	0.6185	0.9957818	Mineral
Water_content	nifh_Cluster749	0.0055	0.0018	0.0792	0.0084	0.3094	0.9957818	Mineral
Water_content	nifh_Cluster755	0.0118	-0.0003	0.0007	-0.0761	0.9235	0.9957818	Mineral
Water_content	nifh_Cluster76	0.0033	-0.0013	0.0712	-0.0003	0.3365	0.9957818	Mineral
Water_content	nifh_Cluster79	0.002	-0.0004	0.024	-0.051	0.5811	0.9957818	Mineral
Water_content	nifh_Cluster81	0.0038	0.00E+00	0.00E+00	-0.0769	0.9984	0.9983685	Mineral
Water_content	nifh_Cluster82	0.0014	-0.0006	0.0413	-0.0324	0.4673	0.9957818	Mineral
Water_content	nifh_Cluster83	0.0055	-0.0009	0.0155	-0.0602	0.6581	0.9957818	Mineral
Water_content	nifh_Cluster86	0.0161	-0.0027	0.0371	-0.037	0.4915	0.9957818	Mineral
Water_content	nifh_Cluster868	0.0098	0.0009	0.0084	-0.0679	0.7455	0.9957818	Mineral
Water_content	nifh_Cluster87	0.003	0.00E+00	0.00E+00	-0.0769	0.9947	0.9983685	Mineral
Water_content	nifh_Cluster88	0.0054	0.0005	0.0047	-0.0719	0.8083	0.9957818	Mineral
Water_content	nifh_Cluster9	0.0077	-0.0034	0.1051	0.0363	0.2384	0.9957818	Mineral
Water_content	nifh_Cluster92	0.0068	1.00E-04	1.00E-04	-0.0769	0.9786	0.9962612	Mineral
Water_content	nifh_Cluster93	0.0035	1.00E-04	0.0002	-0.0767	0.957	0.9957818	Mineral
Water_content	nifh_Cluster94	0.005	-0.0019	0.0549	-0.0178	0.4008	0.9957818	Mineral
Water_content	nifh_Cluster95	0.0149	0.0012	0.0035	-0.0731	0.833	0.9957818	Mineral

Water_content	nifh_Cluster96	0.0131	0.0032	0.054	-0.0188	0.4047	0.9957818	Mineral
Water_content	nifh_Cluster97	0.0125	0.0039	0.272	0.216	0.0462	0.8019741	Mineral
Water_content	nifh_Cluster98	0.0049	0.0003	0.0036	-0.073	0.8316	0.9957818	Mineral
Water_content	nifh_Cluster99	0.0035	-0.0009	0.024	-0.0511	0.5818	0.9957818	Mineral
Water_content	nirk_Cluster0	6.5686	-0.4195	0.1832	0.1203	0.1115	0.8886646	Mineral
Water_content	nirk_Cluster1	0.2105	-0.0719	0.3619	0.3128	0.0177	0.5486397	Mineral
Water_content	nirk_Cluster10	0.6649	-0.0346	0.0217	-0.0536	0.6006	0.9957818	Mineral
Water_content	nirk_Cluster101	0.004	-0.0005	0.0091	-0.0671	0.7353	0.9957818	Mineral
Water_content	nirk_Cluster102	0.055	-0.0105	0.0813	0.0106	0.3029	0.9957818	Mineral
Water_content	nirk_Cluster103	0.09	-0.0093	0.0316	-0.0429	0.5262	0.9957818	Mineral
Water_content	nirk_Cluster104	0.0028	-0.0004	0.0073	-0.069	0.7616	0.9957818	Mineral
Water_content	nirk_Cluster105	0.1172	-0.0214	0.0941	0.0244	0.2661	0.9957818	Mineral
Water_content	nirk_Cluster106	0.073	-0.0077	0.0148	-0.061	0.666	0.9957818	Mineral
Water_content	nirk_Cluster107	0.1123	-0.0221	0.0957	0.0261	0.2619	0.9957818	Mineral
Water_content	nirk_Cluster108	0.0184	-0.0011	0.0042	-0.0724	0.8176	0.9957818	Mineral
Water_content	nirk_Cluster109	0.0158	-0.0023	0.0334	-0.041	0.5146	0.9957818	Mineral
Water_content	nirk_Cluster11	0.1509	-0.0011	0.0011	-0.0757	0.9054	0.9957818	Mineral
Water_content	nirk_Cluster12	0.1052	-0.0187	0.1842	0.1215	0.1103	0.8886646	Mineral
Water_content	nirk_Cluster13	0.0418	0.0008	0.0025	-0.0742	0.8589	0.9957818	Mineral
Water_content	nirk_Cluster14	0.1084	-0.0037	0.0127	-0.0633	0.6894	0.9957818	Mineral
Water_content	nirk_Cluster15	0.4141	-0.0291	0.0149	-0.0608	0.6644	0.9957818	Mineral
Water_content	nirk_Cluster16	0.5816	-0.0318	0.0076	-0.0687	0.7573	0.9957818	Mineral
Water_content	nirk_Cluster17	0.1044	-0.0066	0.0334	-0.0409	0.5143	0.9957818	Mineral
Water_content	nirk_Cluster18	0.1461	-0.0292	0.0942	0.0246	0.2657	0.9957818	Mineral
Water_content	nirk_Cluster19	0.2953	-0.0007	1.00E-04	-0.0768	0.9766	0.9962612	Mineral
Water_content	nirk_Cluster2	0.2004	0.0264	0.1504	0.0851	0.1532	0.9129331	Mineral
Water_content	nirk_Cluster20	0.1934	-0.0171	0.061	-0.0113	0.375	0.9957818	Mineral
Water_content	nirk_Cluster21	0.1711	-0.0153	0.1186	0.0508	0.2088	0.9467997	Mineral
Water_content	nirk_Cluster22	0.1469	-0.0113	0.0329	-0.0415	0.5174	0.9957818	Mineral
Water_content	nirk_Cluster23	0.0729	-0.0054	0.0638	-0.0082	0.3638	0.9957818	Mineral
Water_content	nirk_Cluster24	0.0753	-0.0058	0.0412	-0.0326	0.4684	0.9957818	Mineral
Water_content	nirk_Cluster25	0.5519	-0.1225	0.5332	0.4973	0.002	0.2227812	Mineral
Water_content	nirk_Cluster26	0.1119	-0.0084	0.0604	-0.0119	0.3773	0.9957818	Mineral
Water_content	nirk_Cluster27	0.2066	-0.0087	0.01	-0.0662	0.723	0.9957818	Mineral
Water_content	nirk_Cluster28	0.0808	-0.004	0.0127	-0.0632	0.6888	0.9957818	Mineral
Water_content	nirk_Cluster29	0.162	0.0082	0.0171	-0.0585	0.6422	0.9957818	Mineral
Water_content	nirk_Cluster3	0.3427	0.0195	0.0399	-0.034	0.4757	0.9957818	Mineral
Water_content	nirk_Cluster30	0.0712	-0.0021	0.0027	-0.074	0.8533	0.9957818	Mineral
Water_content	nirk_Cluster31	0.1423	0.0102	0.0524	-0.0205	0.4121	0.9957818	Mineral
Water_content	nirk_Cluster32	0.1568	-0.0298	0.0762	0.0052	0.3192	0.9957818	Mineral
Water_content	nirk_Cluster33	0.0812	-0.017	0.0804	0.0097	0.3058	0.9957818	Mineral
Water_content	nirk_Cluster34	0.0462	-0.0077	0.0723	0.0009	0.3325	0.9957818	Mineral
Water_content	nirk_Cluster35	0.1282	-0.0344	0.1304	0.0636	0.186	0.9455824	Mineral
Water_content	nirk_Cluster36	0.0021	-0.0002	0.0035	-0.0732	0.835	0.9957818	Mineral
Water_content	nirk_Cluster39	0.0045	0.0018	0.0325	-0.0419	0.5202	0.9957818	Mineral
Water_content	nirk_Cluster4	0.1521	-0.0088	0.0177	-0.0578	0.6363	0.9957818	Mineral
Water_content	nirk_Cluster40	0.0544	-0.0098	0.1634	0.0991	0.1351	0.8935183	Mineral
Water_content	nirk_Cluster41	0.1797	-0.0244	0.2186	0.1585	0.0788	0.865544	Mineral
Water_content	nirk_Cluster44	0.0756	-0.0111	0.0643	-0.0076	0.3617	0.9957818	Mineral
Water_content	nirk_Cluster46	0.0193	-0.0023	0.014	-0.0618	0.6741	0.9957818	Mineral
Water_content	nirk_Cluster48	0.003	-0.0011	0.0666	-0.0052	0.3531	0.9957818	Mineral
Water_content	nirk_Cluster49	0.0072	-0.0034	0.1178	0.0499	0.2104	0.9467997	Mineral
Water_content	nirk_Cluster5	0.2174	-0.0127	0.0394	-0.0345	0.478	0.9957818	Mineral
Water_content	nirk_Cluster50	0.0014	-0.0003	0.0112	-0.0649	0.7074	0.9957818	Mineral
Water_content	nirk_Cluster52	0.0551	-0.0067	0.0573	-0.0153	0.3904	0.9957818	Mineral

Water_content	nirk_Cluster53	0.0039	-0.0019	0.0726	0.0012	0.3316	0.9957818	Mineral
Water_content	nirk_Cluster54	0.0024	-0.0013	0.1299	0.063	0.187	0.9455824	Mineral
Water_content	nirk_Cluster55	0.0028	-0.002	0.1154	0.0474	0.2154	0.9467997	Mineral
Water_content	nirk_Cluster56	0.0009	-0.0005	0.0522	-0.0207	0.4128	0.9957818	Mineral
Water_content	nirk_Cluster57	0.0017	-0.0009	0.0525	-0.0204	0.4114	0.9957818	Mineral
Water_content	nirk_Cluster58	0.0102	-0.0032	0.0841	0.0137	0.2943	0.9957818	Mineral
Water_content	nirk_Cluster59	0.007	-0.0004	0.0034	-0.0733	0.8376	0.9957818	Mineral
Water_content	nirk_Cluster6	0.1713	-0.007	0.0125	-0.0634	0.6911	0.9957818	Mineral
Water_content	nirk_Cluster60	0.0006	0.0001	0.0083	-0.068	0.7469	0.9957818	Mineral
Water_content	nirk_Cluster62	0.1789	-0.0494	0.1839	0.1212	0.1107	0.8886646	Mineral
Water_content	nirk_Cluster63	0.1586	-0.041	0.4914	0.4523	0.0036	0.2227812	Mineral
Water_content	nirk_Cluster64	0.0043	-0.0004	0.0058	-0.0707	0.7879	0.9957818	Mineral
Water_content	nirk_Cluster65	0.0531	-0.0114	0.2005	0.139	0.0941	0.8886646	Mineral
Water_content	nirk_Cluster66	0.0095	-0.0052	0.4247	0.3805	0.0085	0.3572571	Mineral
Water_content	nirk_Cluster67	0.0232	0.0013	0.0038	-0.0728	0.8271	0.9957818	Mineral
Water_content	nirk_Cluster68	0.0158	-0.0054	0.1904	0.1281	0.1039	0.8886646	Mineral
Water_content	nirk_Cluster69	0.0362	-0.0127	0.3926	0.3459	0.0124	0.4315249	Mineral
Water_content	nirk_Cluster7	0.096	-0.0074	0.031	-0.0435	0.5302	0.9957818	Mineral
Water_content	nirk_Cluster70	0.0892	-0.0328	0.1388	0.0725	0.1715	0.928126	Mineral
Water_content	nirk_Cluster71	0.0071	-0.0008	0.0124	-0.0635	0.6925	0.9957818	Mineral
Water_content	nirk_Cluster72	0.0278	-0.0097	0.3755	0.3275	0.0151	0.4963464	Mineral
Water_content	nirk_Cluster73	0.1947	-0.0616	0.4748	0.4344	0.0045	0.2227812	Mineral
Water_content	nirk_Cluster74	0.8012	-0.2194	0.2475	0.1896	0.0592	0.865544	Mineral
Water_content	nirk_Cluster75	0.0186	-0.0112	0.2691	0.2128	0.0476	0.8019741	Mineral
Water_content	nirk_Cluster76	0.1377	-0.0302	0.3532	0.3035	0.0195	0.5740941	Mineral
Water_content	nirk_Cluster77	0.0113	0.0013	0.0046	-0.072	0.8112	0.9957818	Mineral
Water_content	nirk_Cluster78	0.3126	-0.0575	0.1625	0.098	0.1363	0.8935183	Mineral
Water_content	nirk_Cluster79	0.0096	0.0007	0.0025	-0.0743	0.8603	0.9957818	Mineral
Water_content	nirk_Cluster8	0.1182	-0.0115	0.0294	-0.0452	0.5409	0.9957818	Mineral
Water_content	nirk_Cluster80	0.3544	-0.1253	0.4758	0.4355	0.0044	0.2227812	Mineral
Water_content	nirk_Cluster82	0.0021	-0.0014	0.101	0.0318	0.2484	0.9957818	Mineral
Water_content	nirk_Cluster83	0.0045	-0.0011	0.0418	-0.0319	0.4648	0.9957818	Mineral
Water_content	nirk_Cluster84	0.0235	0.0029	0.0136	-0.0623	0.6788	0.9957818	Mineral
Water_content	nirk_Cluster85	0.0053	-0.0018	0.091	0.0211	0.2745	0.9957818	Mineral
Water_content	nirk_Cluster86	0.0352	-0.0114	0.1958	0.134	0.0986	0.8886646	Mineral
Water_content	nirk_Cluster87	0.0138	-0.0062	0.2707	0.2146	0.0468	0.8019741	Mineral
Water_content	nirk_Cluster88	0.0035	-0.0004	0.0108	-0.0652	0.7119	0.9957818	Mineral
Water_content	nirk_Cluster89	0.0087	-0.0004	0.0019	-0.0749	0.8777	0.9957818	Mineral
Water_content	nirk_Cluster9	0.061	-0.0002	0.00E+00	-0.0769	0.9833	0.9962612	Mineral
Water_content	nirk_Cluster90	0.0087	-0.0002	0.0002	-0.0767	0.9589	0.9957818	Mineral
Water_content	nirk_Cluster91	0.044	-0.0077	0.0875	0.0173	0.2845	0.9957818	Mineral
Water_content	nirk_Cluster92	0.009	-0.0026	0.0578	-0.0147	0.3881	0.9957818	Mineral
Water_content	nirk_Cluster93	0.0357	-0.0057	0.0502	-0.0229	0.4224	0.9957818	Mineral
Water_content	nirk_Cluster94	0.0222	-0.0028	0.0282	-0.0466	0.5499	0.9957818	Mineral
Water_content	nirk_Cluster95	0.0566	-0.0124	0.0908	0.0208	0.2752	0.9957818	Mineral
Water_content	nirk_Cluster96	0.0588	-0.0046	0.0164	-0.0592	0.649	0.9957818	Mineral
Water_content	nirk_Cluster97	0.0329	-0.0028	0.0116	-0.0645	0.703	0.9957818	Mineral
Water_content	nirk_Cluster98	0.0225	0.0032	0.0193	-0.0561	0.6213	0.9957818	Mineral
Water_content	nirk_Cluster99	0.0074	-0.0003	0.0015	-0.0754	0.8924	0.9957818	Mineral
Water_content	nirs_Cluster0	0.1586	0.0039	0.0038	-0.0729	0.8282	0.9957818	Mineral
Water_content	nirs_Cluster1	0.0051	0.0006	0.006	-0.0704	0.783	0.9957818	Mineral
Water_content	nirs_Cluster14	0.0142	-0.0012	0.002	-0.0747	0.8734	0.9957818	Mineral
Water_content	nirs_Cluster2	0.0174	0.0006	0.0006	-0.0763	0.9319	0.9957818	Mineral
Water_content	nirs_Cluster26	0.0007	0.0004	0.0394	-0.0344	0.478	0.9957818	Mineral
Water_content	nirs_Cluster28	0.0099	-0.0028	0.024	-0.0511	0.5812	0.9957818	Mineral

Water_content	nirs_Cluster3	0.1914	0.0058	0.0132	-0.0627	0.6835	0.9957818	Mineral
Water_content	nirs_Cluster36	0.0018	0.0003	0.0029	-0.0738	0.8492	0.9957818	Mineral
Water_content	nirs_Cluster37	0.0092	-0.0009	0.0017	-0.0751	0.8852	0.9957818	Mineral
Water_content	nirs_Cluster4	0.0003	-1.00E-04	0.0031	-0.0735	0.8427	0.9957818	Mineral
Water_content	nirs_Cluster50	0.0024	0.0016	0.1183	0.0505	0.2094	0.9467997	Mineral
Water_content	nirs_Cluster9	0.0376	-0.0286	0.1783	0.1151	0.1169	0.8886646	Mineral
Water_content	norb_Cluster0	0.0848	-0.0096	0.0243	-0.0508	0.5794	0.9957818	Mineral
Water_content	norb_Cluster1	1.2746	-0.0603	0.0032	-0.0734	0.8404	0.9957818	Mineral
Water_content	norb_Cluster101	0.0037	-0.0029	0.2324	0.1734	0.0688	0.865544	Mineral
Water_content	norb_Cluster102	0.005	-0.0002	0.0012	-0.0756	0.9026	0.9957818	Mineral
Water_content	norb_Cluster103	0.0025	-0.0006	0.0168	-0.0589	0.6456	0.9957818	Mineral
Water_content	norb_Cluster104	0.0025	0.0013	0.052	-0.0209	0.4138	0.9957818	Mineral
Water_content	norb_Cluster106	0.1468	-0.0332	0.1419	0.0758	0.1664	0.9264017	Mineral
Water_content	norb_Cluster107	0.0163	-0.0024	0.0213	-0.054	0.6038	0.9957818	Mineral
Water_content	norb_Cluster108	0.0069	-0.0002	0.0009	-0.076	0.916	0.9957818	Mineral
Water_content	norb_Cluster109	0.0282	-0.0029	0.0155	-0.0602	0.6583	0.9957818	Mineral
Water_content	norb_Cluster11	0.0156	-0.0064	0.1669	0.1028	0.1306	0.8913283	Mineral
Water_content	norb_Cluster110	0.0128	-0.0042	0.103	0.034	0.2436	0.9957818	Mineral
Water_content	norb_Cluster112	0.0215	-0.0061	0.1892	0.1268	0.1052	0.8886646	Mineral
Water_content	norb_Cluster113	0.0157	-0.0056	0.144	0.0781	0.163	0.9244709	Mineral
Water_content	norb_Cluster114	0.0035	0.0002	0.0006	-0.0762	0.9282	0.9957818	Mineral
Water_content	norb_Cluster115	0.0034	-0.0003	0.0111	-0.065	0.7087	0.9957818	Mineral
Water_content	norb_Cluster116	0.0083	-0.001	0.0089	-0.0674	0.7385	0.9957818	Mineral
Water_content	norb_Cluster117	0.0034	-0.0007	0.0168	-0.0588	0.6454	0.9957818	Mineral
Water_content	norb_Cluster118	0.0044	-0.0003	0.002	-0.0747	0.8734	0.9957818	Mineral
Water_content	norb_Cluster119	0.0263	-0.0091	0.1262	0.059	0.1937	0.9455824	Mineral
Water_content	norb_Cluster12	0.005	0.0003	0.0013	-0.0755	0.8981	0.9957818	Mineral
Water_content	norb_Cluster121	0.0023	-0.0011	0.0473	-0.026	0.4363	0.9957818	Mineral
Water_content	norb_Cluster122	0.0184	-0.0015	0.0092	-0.067	0.7337	0.9957818	Mineral
Water_content	norb_Cluster123	0.0071	-0.0017	0.0415	-0.0323	0.4667	0.9957818	Mineral
Water_content	norb_Cluster124	0.7452	-0.0339	0.0032	-0.0735	0.8415	0.9957818	Mineral
Water_content	norb_Cluster125	0.0052	-0.0022	0.1437	0.0778	0.1635	0.9244709	Mineral
Water_content	norb_Cluster126	0.011	-0.0037	0.1184	0.0506	0.2091	0.9467997	Mineral
Water_content	norb_Cluster127	0.2041	-0.0198	0.0146	-0.0612	0.6683	0.9957818	Mineral
Water_content	norb_Cluster128	0.0123	-0.0008	0.0045	-0.0721	0.8132	0.9957818	Mineral
Water_content	norb_Cluster13	0.0051	-0.0029	0.1063	0.0375	0.2357	0.9957818	Mineral
Water_content	norb_Cluster131	0.0209	-0.0049	0.0738	0.0025	0.3275	0.9957818	Mineral
Water_content	norb_Cluster132	1.2771	-0.1481	0.0489	-0.0243	0.4285	0.9957818	Mineral
Water_content	norb_Cluster133	0.0023	0.0002	0.0026	-0.0741	0.8558	0.9957818	Mineral
Water_content	norb_Cluster135	0.0703	-0.0037	0.0053	-0.0712	0.796	0.9957818	Mineral
Water_content	norb_Cluster136	0.025	0.002	0.0087	-0.0676	0.741	0.9957818	Mineral
Water_content	norb_Cluster139	0.0391	-0.0058	0.0385	-0.0354	0.4831	0.9957818	Mineral
Water_content	norb_Cluster14	0.0037	-0.0026	0.2974	0.2434	0.0355	0.7734922	Mineral
Water_content	norb_Cluster140	0.0485	-0.0114	0.1178	0.05	0.2104	0.9467997	Mineral
Water_content	norb_Cluster142	0.0018	-0.0015	0.145	0.0793	0.1614	0.9244709	Mineral
Water_content	norb_Cluster144	0.0122	-0.0007	0.0045	-0.072	0.8116	0.9957818	Mineral
Water_content	norb_Cluster146	0.0418	-0.0003	0.0001	-0.0768	0.9689	0.9962612	Mineral
Water_content	norb_Cluster15	0.0502	-0.0032	0.0053	-0.0713	0.7975	0.9957818	Mineral
Water_content	norb_Cluster150	0.0199	-0.0011	0.0077	-0.0686	0.7557	0.9957818	Mineral
Water_content	norb_Cluster155	0.1729	-0.0132	0.0107	-0.0654	0.7143	0.9957818	Mineral
Water_content	norb_Cluster16	0.0165	-0.0107	0.3192	0.2668	0.0282	0.6596202	Mineral
Water_content	norb_Cluster164	0.0242	-0.0099	0.1708	0.107	0.1257	0.8913283	Mineral
Water_content	norb_Cluster165	0.0092	-0.0022	0.0295	-0.0452	0.5406	0.9957818	Mineral
Water_content	norb_Cluster166	0.0046	-0.0008	0.0139	-0.062	0.6761	0.9957818	Mineral
Water_content	norb_Cluster169	0.0309	-0.0123	0.1613	0.0968	0.1378	0.8935636	Mineral

Water_content	norb_Cluster17	0.0035	-0.0017	0.1298	0.0629	0.1871	0.9455824	Mineral
Water_content	norb_Cluster170	0.0299	-0.0045	0.0188	-0.0566	0.6256	0.9957818	Mineral
Water_content	norb_Cluster171	0.0089	-0.0047	0.1246	0.0572	0.1969	0.9455824	Mineral
Water_content	norb_Cluster172	0.0179	-0.0034	0.0269	-0.048	0.5593	0.9957818	Mineral
Water_content	norb_Cluster175	0.0595	-0.0151	0.056	-0.0167	0.396	0.9957818	Mineral
Water_content	norb_Cluster176	0.0392	-0.0161	0.1526	0.0874	0.15	0.9129331	Mineral
Water_content	norb_Cluster177	0.0361	-0.0073	0.0361	-0.038	0.4975	0.9957818	Mineral
Water_content	norb_Cluster179	0.003	0.001	0.0379	-0.0361	0.4867	0.9957818	Mineral
Water_content	norb_Cluster18	0.0047	-0.0031	0.3396	0.2888	0.0226	0.6064434	Mineral
Water_content	norb_Cluster180	0.0133	0.0002	0.0003	-0.0766	0.9519	0.9957818	Mineral
Water_content	norb_Cluster181	0.0003	1.00E-04	0.0036	-0.073	0.8315	0.9957818	Mineral
Water_content	norb_Cluster182	0.0018	-0.0009	0.0866	0.0164	0.2869	0.9957818	Mineral
Water_content	norb_Cluster184	0.0175	-0.0045	0.0694	-0.0022	0.3429	0.9957818	Mineral
Water_content	norb_Cluster186	0.0011	-0.0004	0.0235	-0.0516	0.5856	0.9957818	Mineral
Water_content	norb_Cluster19	0.0058	-0.0029	0.264	0.2074	0.0501	0.820667	Mineral
Water_content	norb_Cluster192	0.0221	-0.0074	0.1039	0.035	0.2413	0.9957818	Mineral
Water_content	norb_Cluster193	0.0022	-0.0006	0.0268	-0.048	0.5596	0.9957818	Mineral
Water_content	norb_Cluster197	0.0398	0.0121	0.0177	-0.0579	0.6364	0.9957818	Mineral
Water_content	norb_Cluster2	0.0032	-0.0016	0.0823	0.0117	0.2999	0.9957818	Mineral
Water_content	norb_Cluster20	0.0665	-0.006	0.0139	-0.062	0.6758	0.9957818	Mineral
Water_content	norb_Cluster208	0.0015	-0.0007	0.0527	-0.0202	0.4105	0.9957818	Mineral
Water_content	norb_Cluster21	0.0542	-0.0039	0.0079	-0.0684	0.7527	0.9957818	Mineral
Water_content	norb_Cluster211	0.0141	0.0052	0.0358	-0.0384	0.4994	0.9957818	Mineral
Water_content	norb_Cluster212	0.0252	-0.0079	0.0435	-0.03	0.4555	0.9957818	Mineral
Water_content	norb_Cluster214	0.0163	0.0021	0.0072	-0.0691	0.763	0.9957818	Mineral
Water_content	norb_Cluster217	0.0186	-0.0008	0.0008	-0.076	0.9188	0.9957818	Mineral
Water_content	norb_Cluster219	0.0108	-0.0025	0.0161	-0.0596	0.6525	0.9957818	Mineral
Water_content	norb_Cluster22	0.0028	0.0006	0.01	-0.0661	0.7223	0.9957818	Mineral
Water_content	norb_Cluster220	0.0248	0.0025	0.0032	-0.0735	0.8416	0.9957818	Mineral
Water_content	norb_Cluster221	0.0248	0.0052	0.012	-0.0641	0.6981	0.9957818	Mineral
Water_content	norb_Cluster222	0.0104	0.0032	0.019	-0.0565	0.6244	0.9957818	Mineral
Water_content	norb_Cluster224	0.0238	-0.0057	0.0224	-0.0528	0.5944	0.9957818	Mineral
Water_content	norb_Cluster225	0.0095	-0.0007	0.0039	-0.0727	0.8246	0.9957818	Mineral
Water_content	norb_Cluster226	0.0049	-0.0019	0.0674	-0.0043	0.35	0.9957818	Mineral
Water_content	norb_Cluster228	0.0009	-0.0011	0.0836	0.0131	0.2959	0.9957818	Mineral
Water_content	norb_Cluster229	0.004	-0.0014	0.0625	-0.0097	0.369	0.9957818	Mineral
Water_content	norb_Cluster230	0.0019	-0.0007	0.0383	-0.0357	0.4846	0.9957818	Mineral
Water_content	norb_Cluster231	0.0061	-0.0004	0.0015	-0.0753	0.8908	0.9957818	Mineral
Water_content	norb_Cluster232	0.0044	-0.0003	0.002	-0.0748	0.8757	0.9957818	Mineral
Water_content	norb_Cluster234	0.017	-0.0074	0.1279	0.0609	0.1905	0.9455824	Mineral
Water_content	norb_Cluster235	0.0012	0.00E+00	0.0001	-0.0768	0.9703	0.9962612	Mineral
Water_content	norb_Cluster238	0.0135	-0.0072	0.1286	0.0616	0.1893	0.9455824	Mineral
Water_content	norb_Cluster239	0.0109	-0.0068	0.1404	0.0743	0.1687	0.928126	Mineral
Water_content	norb_Cluster24	0.3014	-0.0206	0.0093	-0.0669	0.7322	0.9957818	Mineral
Water_content	norb_Cluster244	0.0015	-0.0006	0.0543	-0.0184	0.4033	0.9957818	Mineral
Water_content	norb_Cluster247	0.006	0.0002	0.0008	-0.0761	0.922	0.9957818	Mineral
Water_content	norb_Cluster25	0.1263	-0.0031	0.0008	-0.076	0.9193	0.9957818	Mineral
Water_content	norb_Cluster250	0.0146	-0.0026	0.0339	-0.0405	0.5116	0.9957818	Mineral
Water_content	norb_Cluster251	0.0064	-0.003	0.1361	0.0697	0.1759	0.94319	Mineral
Water_content	norb_Cluster26	0.1134	-0.0124	0.0178	-0.0577	0.6353	0.9957818	Mineral
Water_content	norb_Cluster28	0.0355	-0.006	0.0418	-0.0319	0.4646	0.9957818	Mineral
Water_content	norb_Cluster29	0.2454	-0.0288	0.0647	-0.0073	0.3603	0.9957818	Mineral
Water_content	norb_Cluster31	0.0632	0.0009	0.0004	-0.0765	0.9471	0.9957818	Mineral
Water_content	norb_Cluster32	0.1401	-0.003	0.0005	-0.0763	0.935	0.9957818	Mineral
Water_content	norb_Cluster33	0.434	0.1084	0.1671	0.103	0.1304	0.8913283	Mineral

Water_content	norb_Cluster35	0.0746	-0.0013	0.0005	-0.0764	0.9385	0.9957818	Mineral
Water_content	norb_Cluster36	1.1002	-0.0255	0.0008	-0.076	0.9189	0.9957818	Mineral
Water_content	norb_Cluster37	0.7173	0.0135	0.0008	-0.076	0.9193	0.9957818	Mineral
Water_content	norb_Cluster38	0.0138	-0.0009	0.0078	-0.0685	0.7545	0.9957818	Mineral
Water_content	norb_Cluster39	0.0465	0.0006	0.0002	-0.0767	0.9556	0.9957818	Mineral
Water_content	norb_Cluster40	0.2213	-0.0115	0.0048	-0.0718	0.8071	0.9957818	Mineral
Water_content	norb_Cluster42	0.2825	0.0335	0.0223	-0.0529	0.5951	0.9957818	Mineral
Water_content	norb_Cluster44	0.0249	-0.0081	0.1148	0.0467	0.2168	0.9467997	Mineral
Water_content	norb_Cluster46	0.2575	0.036	0.0264	-0.0485	0.563	0.9957818	Mineral
Water_content	norb_Cluster47	0.6453	-0.0469	0.0102	-0.0659	0.72	0.9957818	Mineral
Water_content	norb_Cluster5	0.0115	-0.0023	0.0549	-0.0178	0.4007	0.9957818	Mineral
Water_content	norb_Cluster51	0.0122	-0.0044	0.0679	-0.0038	0.3483	0.9957818	Mineral
Water_content	norb_Cluster52	0.2809	-0.0135	0.0036	-0.073	0.8313	0.9957818	Mineral
Water_content	norb_Cluster56	0.0178	-0.0022	0.0223	-0.0529	0.5955	0.9957818	Mineral
Water_content	norb_Cluster57	0.004	-0.0008	0.0375	-0.0366	0.4894	0.9957818	Mineral
Water_content	norb_Cluster59	0.0322	-0.0063	0.0302	-0.0444	0.5359	0.9957818	Mineral
Water_content	norb_Cluster6	0.6934	-0.0147	0.0008	-0.0761	0.921	0.9957818	Mineral
Water_content	norb_Cluster60	0.345	-0.0019	0.00E+00	-0.0769	0.9861	0.9962612	Mineral
Water_content	norb_Cluster61	0.5486	-0.156	0.1451	0.0794	0.1612	0.9244709	Mineral
Water_content	norb_Cluster62	0.003	0.0023	0.3224	0.2703	0.0272	0.6596202	Mineral
Water_content	norb_Cluster64	0.0407	-0.0023	0.0084	-0.0679	0.7458	0.9957818	Mineral
Water_content	norb_Cluster66	0.0016	0.0003	0.0076	-0.0688	0.7578	0.9957818	Mineral
Water_content	norb_Cluster67	0.0279	-0.0025	0.012	-0.064	0.6974	0.9957818	Mineral
Water_content	norb_Cluster68	0.003	-0.0013	0.0957	0.0262	0.2618	0.9957818	Mineral
Water_content	norb_Cluster69	0.0032	0.0006	0.0156	-0.0601	0.6572	0.9957818	Mineral
Water_content	norb_Cluster7	0.0786	-0.0048	0.0051	-0.0714	0.7994	0.9957818	Mineral
Water_content	norb_Cluster70	0.1262	-0.0152	0.0369	-0.0372	0.4928	0.9957818	Mineral
Water_content	norb_Cluster71	0.1512	-0.0258	0.0901	0.0201	0.2772	0.9957818	Mineral
Water_content	norb_Cluster74	0.0387	-0.0062	0.0635	-0.0086	0.3651	0.9957818	Mineral
Water_content	norb_Cluster75	0.1531	-0.0259	0.1165	0.0486	0.213	0.9467997	Mineral
Water_content	norb_Cluster76	0.1215	0.0139	0.0081	-0.0683	0.7505	0.9957818	Mineral
Water_content	norb_Cluster77	0.0492	0.0012	0.0017	-0.075	0.8826	0.9957818	Mineral
Water_content	norb_Cluster8	0.0206	-0.0047	0.0635	-0.0085	0.3648	0.9957818	Mineral
Water_content	norb_Cluster80	0.0021	-0.0006	0.0289	-0.0458	0.5449	0.9957818	Mineral
Water_content	norb_Cluster81	0.0013	-0.0005	0.0456	-0.0278	0.4445	0.9957818	Mineral
Water_content	norb_Cluster83	0.011	-0.0086	0.2168	0.1566	0.0803	0.865544	Mineral
Water_content	norb_Cluster84	0.0093	1.00E-04	0.00E+00	-0.0769	0.985	0.9962612	Mineral
Water_content	norb_Cluster85	0.0025	-0.0004	0.0098	-0.0664	0.7255	0.9957818	Mineral
Water_content	norb_Cluster86	0.041	0.0004	0.00E+00	-0.0769	0.983	0.9962612	Mineral
Water_content	norb_Cluster88	0.0019	-0.0007	0.0364	-0.0378	0.4961	0.9957818	Mineral
Water_content	norb_Cluster90	0.0013	-0.001	0.064	-0.008	0.3631	0.9957818	Mineral
Water_content	norb_Cluster91	0.7571	-0.2047	0.1066	0.0379	0.2349	0.9957818	Mineral
Water_content	norb_Cluster92	0.0021	0.0009	0.0367	-0.0374	0.4938	0.9957818	Mineral
Water_content	norb_Cluster93	0.0028	-0.0009	0.0267	-0.0482	0.5609	0.9957818	Mineral
Water_content	norb_Cluster94	0.002	-0.0007	0.0473	-0.026	0.4362	0.9957818	Mineral
Water_content	norb_Cluster96	0.0006	-0.0003	0.0457	-0.0278	0.4445	0.9957818	Mineral
Water_content	norb_Cluster97	0.0162	0.0004	0.0007	-0.0762	0.9255	0.9957818	Mineral
Water_content	norb_Cluster98	0.0176	-0.0016	0.0056	-0.0709	0.7904	0.9957818	Mineral
Water_content	nosz_Cluster0	0.0057	-0.0005	0.0068	-0.0696	0.77	0.9957818	Mineral
Water_content	nosz_Cluster10	0.0128	0.0005	0.0017	-0.0751	0.8853	0.9957818	Mineral
Water_content	nosz_Cluster102	0.0057	-0.0022	0.2419	0.1836	0.0626	0.865544	Mineral
Water_content	nosz_Cluster103	0.0496	-0.0166	0.1927	0.1306	0.1016	0.8886646	Mineral
Water_content	nosz_Cluster105	0.0016	-0.0001	0.0016	-0.0752	0.8883	0.9957818	Mineral
Water_content	nosz_Cluster106	0.0088	-0.0037	0.1325	0.0658	0.1822	0.9455824	Mineral
Water_content	nosz_Cluster107	0.0003	-1.00E-04	0.0031	-0.0735	0.8427	0.9957818	Mineral

Water_content	nosz_Cluster108	0.0043	0.0007	0.0114	-0.0647	0.705	0.9957818	Mineral
Water_content	nosz_Cluster109	0.0416	-0.0104	0.2294	0.1701	0.0709	0.865544	Mineral
Water_content	nosz_Cluster11	0.0104	-0.0017	0.0281	-0.0467	0.5506	0.9957818	Mineral
Water_content	nosz_Cluster110	0.0004	0.0012	0.7243	0.7031	1.00E-04	0.0338965	Mineral
Water_content	nosz_Cluster111	0.0078	-0.003	0.1581	0.0933	0.1423	0.9122892	Mineral
Water_content	nosz_Cluster116	0.0074	0.0007	0.0127	-0.0632	0.6889	0.9957818	Mineral
Water_content	nosz_Cluster118	0.0064	1.00E-04	0.00E+00	-0.0769	0.9823	0.9962612	Mineral
Water_content	nosz_Cluster119	0.0019	-0.0016	0.2247	0.1651	0.0742	0.865544	Mineral
Water_content	nosz_Cluster120	0.0083	-0.003	0.1149	0.0469	0.2164	0.9467997	Mineral
Water_content	nosz_Cluster123	0.0136	-0.0065	0.2077	0.1467	0.0878	0.865544	Mineral
Water_content	nosz_Cluster128	0.005	-1.00E-04	0.0002	-0.0767	0.9637	0.9957818	Mineral
Water_content	nosz_Cluster129	0.05	-0.0065	0.0654	-0.0064	0.3574	0.9957818	Mineral
Water_content	nosz_Cluster13	0.0124	-0.002	0.0253	-0.0497	0.5713	0.9957818	Mineral
Water_content	nosz_Cluster130	0.1138	-0.025	0.0122	-0.0637	0.6948	0.9957818	Mineral
Water_content	nosz_Cluster132	0.004	-0.0018	0.1702	0.1064	0.1264	0.8913283	Mineral
Water_content	nosz_Cluster14	0.0149	-0.0004	0.0004	-0.0765	0.9421	0.9957818	Mineral
Water_content	nosz_Cluster16	0.008	0.0031	0.0725	0.0012	0.3318	0.9957818	Mineral
Water_content	nosz_Cluster17	0.0427	-0.0124	0.1389	0.0726	0.1713	0.928126	Mineral
Water_content	nosz_Cluster18	0.0184	-0.002	0.0086	-0.0677	0.7423	0.9957818	Mineral
Water_content	nosz_Cluster20	0.0117	-0.0006	0.0014	-0.0755	0.896	0.9957818	Mineral
Water_content	nosz_Cluster21	0.0117	-0.0043	0.0874	0.0172	0.2846	0.9957818	Mineral
Water_content	nosz_Cluster22	0.0325	0.0037	0.0065	-0.0699	0.7755	0.9957818	Mineral
Water_content	nosz_Cluster23	0.019	-0.0052	0.0598	-0.0126	0.3799	0.9957818	Mineral
Water_content	nosz_Cluster24	0.0019	0.0001	0.0012	-0.0756	0.9012	0.9957818	Mineral
Water_content	nosz_Cluster25	0.0026	-0.0002	0.0026	-0.0741	0.857	0.9957818	Mineral
Water_content	nosz_Cluster26	0.0068	-0.0009	0.0084	-0.0678	0.7447	0.9957818	Mineral
Water_content	nosz_Cluster27	0.007	-0.0013	0.0271	-0.0477	0.5574	0.9957818	Mineral
Water_content	nosz_Cluster28	0.0358	-0.0028	0.0074	-0.069	0.7607	0.9957818	Mineral
Water_content	nosz_Cluster30	0.0341	-0.0015	0.0026	-0.0741	0.8574	0.9957818	Mineral
Water_content	nosz_Cluster32	0.016	-0.0003	0.0005	-0.0764	0.9379	0.9957818	Mineral
Water_content	nosz_Cluster33	0.0187	-0.0005	0.0006	-0.0763	0.9325	0.9957818	Mineral
Water_content	nosz_Cluster34	0.0387	-0.0045	0.0081	-0.0682	0.7499	0.9957818	Mineral
Water_content	nosz_Cluster35	0.0097	-0.0003	0.0006	-0.0763	0.9303	0.9957818	Mineral
Water_content	nosz_Cluster36	0.0213	-0.0036	0.0297	-0.0449	0.539	0.9957818	Mineral
Water_content	nosz_Cluster37	0.0077	-0.0007	0.0062	-0.0702	0.7802	0.9957818	Mineral
Water_content	nosz_Cluster38	0.0144	-0.0054	0.0734	0.0022	0.3286	0.9957818	Mineral
Water_content	nosz_Cluster39	0.0653	-0.0086	0.0268	-0.0481	0.56	0.9957818	Mineral
Water_content	nosz_Cluster40	0.0262	0.0027	0.0095	-0.0667	0.7292	0.9957818	Mineral
Water_content	nosz_Cluster41	0.0258	-0.0022	0.0066	-0.0698	0.7729	0.9957818	Mineral
Water_content	nosz_Cluster42	0.03	-0.0036	0.0132	-0.0627	0.6832	0.9957818	Mineral
Water_content	nosz_Cluster43	0.001	-0.0004	0.024	-0.0511	0.5815	0.9957818	Mineral
Water_content	nosz_Cluster44	0.0068	-0.0035	0.1784	0.1152	0.1168	0.8886646	Mineral
Water_content	nosz_Cluster45	0.0056	0.0006	0.0072	-0.0691	0.7631	0.9957818	Mineral
Water_content	nosz_Cluster46	0.0082	-0.0002	0.001	-0.0758	0.9093	0.9957818	Mineral
Water_content	nosz_Cluster47	0.0012	-1.00E-04	0.002	-0.0748	0.8745	0.9957818	Mineral
Water_content	nosz_Cluster48	0.0028	-0.0009	0.0308	-0.0437	0.5315	0.9957818	Mineral
Water_content	nosz_Cluster49	0.0012	0.0004	0.0291	-0.0456	0.5432	0.9957818	Mineral
Water_content	nosz_Cluster5	0.004	0.0004	0.0039	-0.0727	0.8243	0.9957818	Mineral
Water_content	nosz_Cluster51	0.003	0.0006	0.0167	-0.0589	0.646	0.9957818	Mineral
Water_content	nosz_Cluster53	0.0012	-0.0003	0.0093	-0.067	0.733	0.9957818	Mineral
Water_content	nosz_Cluster54	0.0037	0.00E+00	0.00E+00	-0.0769	0.9844	0.9962612	Mineral
Water_content	nosz_Cluster57	0.2232	0.0046	0.0006	-0.0763	0.931	0.9957818	Mineral
Water_content	nosz_Cluster58	0.0053	-0.0033	0.1687	0.1048	0.1283	0.8913283	Mineral
Water_content	nosz_Cluster59	0.0008	-0.0006	0.0304	-0.0442	0.5343	0.9957818	Mineral
Water_content	nosz_Cluster6	0.0034	0.0001	0.0006	-0.0762	0.9286	0.9957818	Mineral

Water_content	nosz_Cluster60	0.0099	-1.00E-04	0.00E+00	-0.0769	0.9855	0.9962612	Mineral
Water_content	nosz_Cluster61	0.0178	-0.008	0.4741	0.4337	0.0045	0.2227812	Mineral
Water_content	nosz_Cluster62	0.0493	0.0028	0.0038	-0.0728	0.8268	0.9957818	Mineral
Water_content	nosz_Cluster64	0.0023	0.0003	0.0019	-0.0749	0.8772	0.9957818	Mineral
Water_content	nosz_Cluster65	0.0083	0.0013	0.0167	-0.0589	0.6459	0.9957818	Mineral
Water_content	nosz_Cluster66	0.0428	-0.0006	0.0002	-0.0767	0.9636	0.9957818	Mineral
Water_content	nosz_Cluster67	0.0669	0.0048	0.005	-0.0716	0.8032	0.9957818	Mineral
Water_content	nosz_Cluster69	0.008	-0.0016	0.0362	-0.0379	0.4968	0.9957818	Mineral
Water_content	nosz_Cluster70	0.0655	0.0021	0.0004	-0.0765	0.9432	0.9957818	Mineral
Water_content	nosz_Cluster71	0.0101	-0.0003	0.0005	-0.0764	0.9379	0.9957818	Mineral
Water_content	nosz_Cluster72	0.066	-0.0011	0.0003	-0.0766	0.9507	0.9957818	Mineral
Water_content	nosz_Cluster73	0.0031	-0.0015	0.0924	0.0226	0.2706	0.9957818	Mineral
Water_content	nosz_Cluster74	0.0017	-0.0016	0.2339	0.175	0.0678	0.865544	Mineral
Water_content	nosz_Cluster75	0.132	-0.0214	0.0054	-0.0711	0.7938	0.9957818	Mineral
Water_content	nosz_Cluster77	0.0002	-0.0001	0.0191	-0.0563	0.6231	0.9957818	Mineral
Water_content	nosz_Cluster78	0.0054	-0.0017	0.0812	0.0106	0.3032	0.9957818	Mineral
Water_content	nosz_Cluster79	0.0401	0.00E+00	0.00E+00	-0.0769	0.9975	0.9983685	Mineral
Water_content	nosz_Cluster8	0.0106	-0.0013	0.0239	-0.0511	0.5819	0.9957818	Mineral
Water_content	nosz_Cluster80	0.0039	0.00E+00	0.00E+00	-0.0769	0.9856	0.9962612	Mineral
Water_content	nosz_Cluster81	0.0099	0.0006	0.0015	-0.0753	0.8904	0.9957818	Mineral
Water_content	nosz_Cluster82	0.0023	0.0003	0.0016	-0.0752	0.8862	0.9957818	Mineral
Water_content	nosz_Cluster83	0.0014	-0.0016	0.1522	0.087	0.1505	0.9129331	Mineral
Water_content	nosz_Cluster85	0.0027	0.0003	0.0039	-0.0728	0.8261	0.9957818	Mineral
Water_content	nosz_Cluster86	0.0038	0.0003	0.0033	-0.0734	0.8393	0.9957818	Mineral
Water_content	nosz_Cluster87	0.0034	-0.0007	0.0223	-0.053	0.5957	0.9957818	Mineral
Water_content	nosz_Cluster88	0.0246	-0.0063	0.1697	0.1058	0.1271	0.8913283	Mineral
Water_content	nosz_Cluster89	0.0607	0.0021	0.0014	-0.0755	0.8961	0.9957818	Mineral
Water_content	nosz_Cluster9	0.0034	0.0001	0.0003	-0.0766	0.9499	0.9957818	Mineral
Water_content	nosz_Cluster90	0.0139	-0.0014	0.0205	-0.0549	0.6111	0.9957818	Mineral
Water_content	nosz_Cluster91	0.0232	0.0043	0.0275	-0.0473	0.5548	0.9957818	Mineral
Water_content	nosz_Cluster93	0.0488	-0.0182	0.1876	0.1251	0.1068	0.8886646	Mineral
Water_content	nosz_Cluster95	0.0094	0.0007	0.0071	-0.0693	0.7649	0.9957818	Mineral
Water_content	nosz_Cluster96	0.0064	-0.0016	0.0813	0.0106	0.303	0.9957818	Mineral
Water_content	nosz_Cluster97	0.0026	-0.0003	0.0065	-0.07	0.7759	0.9957818	Mineral
Water_content	nosz_Cluster98	0.0035	0.0008	0.0059	-0.0706	0.7853	0.9957818	Mineral
Water_content	nosz_Cluster99	0.0061	0.0012	0.0395	-0.0344	0.4779	0.9957818	Mineral
Water_content	nrfa_Cluster10	0.0978	-0.0214	0.0415	-0.0323	0.4666	0.9957818	Mineral
Water_content	nrfa_Cluster100	0.1249	-0.0782	0.2421	0.1838	0.0625	0.865544	Mineral
Water_content	nrfa_Cluster104	0.1478	-0.0565	0.2451	0.187	0.0606	0.865544	Mineral
Water_content	nrfa_Cluster105	0.006	-0.0018	0.0475	-0.0257	0.435	0.9957818	Mineral
Water_content	nrfa_Cluster109	0.2753	-0.0857	0.1817	0.1188	0.1131	0.8886646	Mineral
Water_content	nrfa_Cluster116	0.3208	-0.0966	0.3163	0.2638	0.0291	0.6596202	Mineral
Water_content	nrfa_Cluster118	0.2298	-0.1499	0.2239	0.1642	0.0748	0.865544	Mineral
Water_content	nrfa_Cluster125	0.1081	-0.0686	0.1892	0.1268	0.1052	0.8886646	Mineral
Water_content	nrfa_Cluster154	0.0837	-0.048	0.2168	0.1566	0.0803	0.865544	Mineral
Water_content	nrfa_Cluster164	0.0044	-0.001	0.0292	-0.0455	0.5427	0.9957818	Mineral
Water_content	nrfa_Cluster172	0.0036	-0.0008	0.0176	-0.058	0.6373	0.9957818	Mineral
Water_content	nrfa_Cluster175	0.0053	-0.0047	0.2053	0.1442	0.0899	0.8691029	Mineral
Water_content	nrfa_Cluster20	0.045	-0.018	0.1346	0.068	0.1786	0.94319	Mineral
Water_content	nrfa_Cluster26	0.1433	-0.0856	0.2106	0.1499	0.0853	0.865544	Mineral
Water_content	nrfa_Cluster29	0.0059	0.0001	0.0002	-0.0767	0.9637	0.9957818	Mineral
Water_content	nrfa_Cluster34	0.0642	-0.0261	0.1464	0.0808	0.1592	0.9244709	Mineral
Water_content	nrfa_Cluster38	0.0033	-0.0025	0.1568	0.092	0.144	0.9129331	Mineral
Water_content	nrfa_Cluster61	0.0047	-0.0021	0.092	0.0222	0.2717	0.9957818	Mineral
Water_content	nrfa_Cluster62	0.0589	-0.0405	0.2159	0.1556	0.081	0.865544	Mineral

Water_content	nrfa_Cluster66	0.1282	-0.0455	0.2903	0.2357	0.0382	0.7734922	Mineral
Water_content	nrfa_Cluster69	0.0035	0.0008	0.0198	-0.0556	0.6166	0.9957818	Mineral
Water_content	nrfa_Cluster71	0.0322	-0.0169	0.1536	0.0885	0.1485	0.9129331	Mineral
Water_content	nrfa_Cluster72	0.0969	-0.0509	0.2196	0.1596	0.078	0.865544	Mineral
Water_content	nrfa_Cluster73	0.0042	-0.0019	0.0636	-0.0084	0.3645	0.9957818	Mineral
Water_content	nrfa_Cluster74	0.1692	-0.0892	0.2143	0.1539	0.0823	0.865544	Mineral
Water_content	nrfa_Cluster76	0.0922	-0.0543	0.212	0.1514	0.0842	0.865544	Mineral
Water_content	nrfa_Cluster79	0.0138	-0.0031	0.0108	-0.0652	0.7118	0.9957818	Mineral
Water_content	nrfa_Cluster87	0.0207	-0.0024	0.0046	-0.0719	0.8097	0.9957818	Mineral
Water_content	nrfa_Cluster89	0.0023	-0.0009	0.0456	-0.0278	0.4447	0.9957818	Mineral
Water_content	nrfa_Cluster92	0.0041	-0.0013	0.0648	-0.0071	0.3597	0.9957818	Mineral
Water_content	nrfa_Cluster93	0.0581	0.0103	0.013	-0.0629	0.686	0.9957818	Mineral
Water_content	nrfa_Cluster94	0.0003	-0.0004	0.0836	0.0131	0.2959	0.9957818	Mineral
Water_content	nrfa_Cluster97	0.0225	-0.0094	0.1959	0.134	0.0985	0.8886646	Mineral
Water_content	nrfa_Cluster99	0.0184	-0.0077	0.1543	0.0892	0.1475	0.9129331	Mineral
Bulk_density	amoA_A_Cluster26	0.0072	-0.005	0.2806	0.2007	0.0937	0.2729786	Mineral
Bulk_density	amoA_A_Cluster45	0.0089	-0.0069	0.4542	0.3935	0.023	0.2148116	Mineral
Bulk_density	amoA_B_Cluster0	0.1358	-0.0076	0.0255	-0.0828	0.639	0.7155322	Mineral
Bulk_density	amoA_B_Cluster1	0.0132	-0.0063	0.4224	0.3582	0.0304	0.2466524	Mineral
Bulk_density	amoA_B_Cluster10	0.0531	-0.0035	0.0142	-0.0954	0.7275	0.7935594	Mineral
Bulk_density	amoA_B_Cluster11	0.0373	-0.0048	0.043	-0.0633	0.5405	0.6510627	Mineral
Bulk_density	amoA_B_Cluster12	0.0925	0.0042	0.0121	-0.0976	0.7472	0.8118058	Mineral
Bulk_density	amoA_B_Cluster13	0.6645	0.2216	0.2839	0.2044	0.0915	0.2713087	Mineral
Bulk_density	amoA_B_Cluster14	0.0382	0.017	0.3076	0.2307	0.0766	0.2644395	Mineral
Bulk_density	amoA_B_Cluster15	0.0354	0.0068	0.0916	-0.0093	0.3655	0.5225487	Mineral
Bulk_density	amoA_B_Cluster2	0.2073	0.0613	0.2136	0.1262	0.1524	0.323981	Mineral
Bulk_density	amoA_B_Cluster20	0.0057	-0.0044	0.5486	0.4984	0.0091	0.1537612	Mineral
Bulk_density	amoA_B_Cluster23	0.0506	-0.0017	0.006	-0.1044	0.8208	0.8679243	Mineral
Bulk_density	amoA_B_Cluster3	0.0161	0.0084	0.3448	0.272	0.0575	0.2551649	Mineral
Bulk_density	amoA_B_Cluster4	0.0149	0.0073	0.2158	0.1287	0.15	0.3212499	Mineral
Bulk_density	amoA_B_Cluster5	0.1038	-0.0076	0.0324	-0.0751	0.5962	0.6818783	Mineral
Bulk_density	amoA_B_Cluster6	0.0237	-0.0006	0.0021	-0.1088	0.8931	0.9164532	Mineral
Bulk_density	amoA_B_Cluster7	0.0164	-0.0092	0.2787	0.1985	0.0951	0.2729786	Mineral
Bulk_density	amoA_B_Cluster8	0.0152	-0.0018	0.0781	-0.0244	0.4053	0.5539252	Mineral
Bulk_density	amoA_B_Cluster9	0.0514	0.0125	0.1001	0.0002	0.3431	0.5026444	Mineral
Bulk_density	nifh_Cluster0	0.0302	-0.0041	0.0462	-0.0598	0.5258	0.6397222	Mineral
Bulk_density	nifh_Cluster10	0.0037	-0.0003	0.004	-0.1066	0.8526	0.8905392	Mineral
Bulk_density	nifh_Cluster100	0.0027	-0.001	0.0721	-0.031	0.4246	0.5683901	Mineral
Bulk_density	nifh_Cluster103	0.0404	0.0037	0.0203	-0.0886	0.6762	0.7528996	Mineral
Bulk_density	nifh_Cluster1034	0.0402	-0.0013	0.0013	-0.1096	0.9148	0.9322557	Mineral
Bulk_density	nifh_Cluster105	0.0214	-0.0043	0.0599	-0.0446	0.4683	0.6022476	Mineral
Bulk_density	nifh_Cluster108	0.0091	-0.0034	0.1592	0.0658	0.2241	0.3904858	Mineral
Bulk_density	nifh_Cluster1099	0.0427	0.0062	0.0159	-0.0935	0.7121	0.7796267	Mineral
Bulk_density	nifh_Cluster11	0.0034	-0.0026	0.5504	0.5005	0.0089	0.1537612	Mineral
Bulk_density	nifh_Cluster1112	0.003	0.0004	0.0086	-0.1015	0.7859	0.8385892	Mineral
Bulk_density	nifh_Cluster112	0.0111	0.0027	0.0332	-0.0742	0.5916	0.6805987	Mineral
Bulk_density	nifh_Cluster113	0.0394	-0.0172	0.5846	0.5385	0.0061	0.1537612	Mineral
Bulk_density	nifh_Cluster114	0.031	-0.0024	0.0296	-0.0783	0.6131	0.6944801	Mineral
Bulk_density	nifh_Cluster1141	0.0329	-0.0031	0.0118	-0.098	0.7506	0.8126853	Mineral
Bulk_density	nifh_Cluster115	0.0096	0.0013	0.0382	-0.0687	0.5647	0.6610552	Mineral
Bulk_density	nifh_Cluster116	0.0694	-0.0136	0.1369	0.041	0.2627	0.428622	Mineral
Bulk_density	nifh_Cluster1163	0.004	-0.0013	0.066	-0.0378	0.4458	0.587369	Mineral
Bulk_density	nifh_Cluster1179	0.0051	-0.0004	0.004	-0.1067	0.8543	0.8905392	Mineral
Bulk_density	nifh_Cluster1182	0.0204	0.0018	0.0355	-0.0717	0.5792	0.669405	Mineral
Bulk_density	nifh_Cluster1183	0.0039	0.0009	0.0457	-0.0604	0.5281	0.6400383	Mineral

Bulk_density	nifh_Cluster1197	0.0106	-0.0021	0.0481	-0.0577	0.517	0.6343742	Mineral
Bulk_density	nifh_Cluster1199	0.0041	-0.0013	0.041	-0.0655	0.5502	0.6510747	Mineral
Bulk_density	nifh_Cluster120	0.0273	-0.019	0.2902	0.2113	0.0873	0.2713087	Mineral
Bulk_density	nifh_Cluster1203	0.017	-0.0056	0.0761	-0.0266	0.4117	0.5569329	Mineral
Bulk_density	nifh_Cluster1206	0.0037	-0.0009	0.041	-0.0656	0.5506	0.6510747	Mineral
Bulk_density	nifh_Cluster1207	0.0091	-0.0005	0.0022	-0.1087	0.8909	0.9157767	Mineral
Bulk_density	nifh_Cluster1209	0.1348	0.0122	0.0771	-0.0254	0.4083	0.5540838	Mineral
Bulk_density	nifh_Cluster121	0.0167	-0.0126	0.3502	0.278	0.0551	0.2551649	Mineral
Bulk_density	nifh_Cluster1211	0.0038	-0.003	0.4119	0.3466	0.0333	0.2492719	Mineral
Bulk_density	nifh_Cluster1213	0.0032	-0.0021	0.329	0.2545	0.065	0.2551649	Mineral
Bulk_density	nifh_Cluster122	0.0056	-0.0043	0.5133	0.4592	0.0131	0.1797301	Mineral
Bulk_density	nifh_Cluster123	0.0125	-0.0089	0.3968	0.3298	0.0378	0.2492719	Mineral
Bulk_density	nifh_Cluster1236	0.036	-0.022	0.2174	0.1304	0.1483	0.3212499	Mineral
Bulk_density	nifh_Cluster1238	0.0046	-0.0018	0.1048	0.0054	0.3314	0.4904655	Mineral
Bulk_density	nifh_Cluster124	0.0099	-0.0032	0.2723	0.1914	0.0997	0.2742109	Mineral
Bulk_density	nifh_Cluster1261	0.0024	-0.0012	0.069	-0.0344	0.4351	0.5779193	Mineral
Bulk_density	nifh_Cluster1262	0.0105	0.0047	0.2203	0.1336	0.1453	0.3205114	Mineral
Bulk_density	nifh_Cluster1266	0.0098	-0.0009	0.0171	-0.0921	0.7014	0.7722197	Mineral
Bulk_density	nifh_Cluster1267	0.0389	0.0195	0.1214	0.0237	0.2938	0.4557298	Mineral
Bulk_density	nifh_Cluster127	0.0044	-0.0036	0.2988	0.2209	0.0818	0.269598	Mineral
Bulk_density	nifh_Cluster1278	0.1942	-0.0819	0.4029	0.3365	0.0359	0.2492719	Mineral
Bulk_density	nifh_Cluster128	0.0058	-0.0035	0.4395	0.3772	0.0262	0.2269574	Mineral
Bulk_density	nifh_Cluster1289	0.0046	0.0021	0.2289	0.1433	0.1366	0.3154091	Mineral
Bulk_density	nifh_Cluster129	0.0117	-0.0091	0.4812	0.4236	0.0179	0.1989595	Mineral
Bulk_density	nifh_Cluster1292	0.01	-0.0061	0.3119	0.2355	0.0741	0.2627045	Mineral
Bulk_density	nifh_Cluster130	0.0097	-0.006	0.1666	0.074	0.2127	0.3809504	Mineral
Bulk_density	nifh_Cluster1305	0.0048	-0.0019	0.1739	0.0821	0.202	0.3726962	Mineral
Bulk_density	nifh_Cluster1306	0.0053	-0.0023	0.1266	0.0295	0.2829	0.4456161	Mineral
Bulk_density	nifh_Cluster131	0.0092	-0.0072	0.4399	0.3777	0.0261	0.2269574	Mineral
Bulk_density	nifh_Cluster1319	0.0053	0.0023	0.1156	0.0173	0.3064	0.4686996	Mineral
Bulk_density	nifh_Cluster132	0.0087	-0.0049	0.2616	0.1795	0.1078	0.2834985	Mineral
Bulk_density	nifh_Cluster1320	0.0022	-0.0018	0.2001	0.1112	0.1677	0.3360388	Mineral
Bulk_density	nifh_Cluster1323	0.0012	0.0004	0.0409	-0.0657	0.551	0.6510747	Mineral
Bulk_density	nifh_Cluster1324	0.0298	-0.0073	0.0858	-0.0157	0.3819	0.5347096	Mineral
Bulk_density	nifh_Cluster1336	0.0145	-0.0027	0.1852	0.0947	0.1864	0.3566502	Mineral
Bulk_density	nifh_Cluster1340	0.0173	0.0082	0.2733	0.1925	0.099	0.2742109	Mineral
Bulk_density	nifh_Cluster1345	0.0306	0.0101	0.063	-0.0411	0.4565	0.5938977	Mineral
Bulk_density	nifh_Cluster1370	0.0027	-0.0008	0.0313	-0.0764	0.6029	0.6855214	Mineral
Bulk_density	nifh_Cluster1375	0.0015	-0.0001	0.002	-0.1088	0.8951	0.9168926	Mineral
Bulk_density	nifh_Cluster139	0.0024	-0.0017	0.1439	0.0487	0.25	0.4146451	Mineral
Bulk_density	nifh_Cluster140	0.0054	-0.0026	0.1801	0.089	0.1932	0.3620835	Mineral
Bulk_density	nifh_Cluster141	0.0815	-0.0107	0.149	0.0544	0.2411	0.4079933	Mineral
Bulk_density	nifh_Cluster148	0.0036	0.0032	0.2507	0.1675	0.1167	0.2949359	Mineral
Bulk_density	nifh_Cluster1480	0.0005	0.0007	0.2208	0.1343	0.1447	0.3205114	Mineral
Bulk_density	nifh_Cluster152	0.0709	-0.0107	0.0689	-0.0346	0.4356	0.5779193	Mineral
Bulk_density	nifh_Cluster156	0.0537	0.0119	0.1262	0.0292	0.2836	0.4456161	Mineral
Bulk_density	nifh_Cluster1562	0.0305	0.0231	0.1945	0.105	0.1745	0.3437937	Mineral
Bulk_density	nifh_Cluster158	0.0176	-0.0024	0.0161	-0.0932	0.7102	0.7789524	Mineral
Bulk_density	nifh_Cluster16	0.0032	-0.0016	0.1059	0.0066	0.3288	0.4902523	Mineral
Bulk_density	nifh_Cluster166	0.0225	-0.017	0.3619	0.291	0.0502	0.2551649	Mineral
Bulk_density	nifh_Cluster205	0.0127	-0.0035	0.2223	0.1359	0.1432	0.3205114	Mineral
Bulk_density	nifh_Cluster21	0.0022	0.00E+00	1.00E-04	-0.111	0.9784	0.9817384	Mineral
Bulk_density	nifh_Cluster222	0.0095	-0.007	0.293	0.2145	0.0855	0.269598	Mineral
Bulk_density	nifh_Cluster225	0.0202	-0.0017	0.0185	-0.0906	0.6902	0.7641405	Mineral
Bulk_density	nifh_Cluster230	0.0063	-0.0033	0.3258	0.2509	0.0667	0.2551649	Mineral

Bulk_density	nifh_Cluster231	0.0019	-0.0014	0.1666	0.074	0.2127	0.3809504	Mineral
Bulk_density	nifh_Cluster236	0.0039	-0.001	0.0662	-0.0376	0.4451	0.587369	Mineral
Bulk_density	nifh_Cluster237	0.0091	-0.0025	0.1275	0.0306	0.281	0.4456161	Mineral
Bulk_density	nifh_Cluster243	0.0306	-0.0038	0.012	-0.0978	0.7484	0.8118058	Mineral
Bulk_density	nifh_Cluster2432	0.0298	0.0092	0.0524	-0.0529	0.4984	0.6232233	Mineral
Bulk_density	nifh_Cluster2433	0.0021	-0.0011	0.0527	-0.0525	0.497	0.6232233	Mineral
Bulk_density	nifh_Cluster246	0.0086	-0.0049	0.2235	0.1372	0.142	0.3205114	Mineral
Bulk_density	nifh_Cluster25	0.0218	-0.0023	0.0411	-0.0654	0.5499	0.6510747	Mineral
Bulk_density	nifh_Cluster256	0.0025	-0.0019	0.2758	0.1953	0.0971	0.2742109	Mineral
Bulk_density	nifh_Cluster264	0.0033	-0.0016	0.2476	0.1639	0.1194	0.2955092	Mineral
Bulk_density	nifh_Cluster265	0.0711	-0.0081	0.0641	-0.0399	0.4525	0.5922306	Mineral
Bulk_density	nifh_Cluster267	0.0063	-0.0018	0.0833	-0.0185	0.3893	0.5408385	Mineral
Bulk_density	nifh_Cluster268	0.012	-0.0015	0.046	-0.06	0.5266	0.6397222	Mineral
Bulk_density	nifh_Cluster269	0.0279	-0.013	0.1427	0.0475	0.252	0.4146451	Mineral
Bulk_density	nifh_Cluster272	0.0013	0.0006	0.038	-0.0689	0.5657	0.6610552	Mineral
Bulk_density	nifh_Cluster274	0.0019	-0.0014	0.083	-0.0189	0.3904	0.5410042	Mineral
Bulk_density	nifh_Cluster275	0.0054	0.0004	0.0066	-0.1038	0.8122	0.8604369	Mineral
Bulk_density	nifh_Cluster278	0.0055	-0.0004	0.003	-0.1077	0.8722	0.9018263	Mineral
Bulk_density	nifh_Cluster279	0.0061	-0.0023	0.1132	0.0146	0.3118	0.4730011	Mineral
Bulk_density	nifh_Cluster28	0.0016	-0.0012	0.189	0.0988	0.1815	0.3545431	Mineral
Bulk_density	nifh_Cluster280	0.0014	0.0007	0.0803	-0.0218	0.3983	0.5481416	Mineral
Bulk_density	nifh_Cluster282	0.005	-0.0021	0.1268	0.0298	0.2825	0.4456161	Mineral
Bulk_density	nifh_Cluster287	0.0034	0.0003	0.0047	-0.1059	0.8411	0.8830619	Mineral
Bulk_density	nifh_Cluster290	0.0114	-0.0037	0.0787	-0.0237	0.4035	0.5527454	Mineral
Bulk_density	nifh_Cluster297	0.0055	-0.0042	0.211	0.1234	0.1552	0.32741	Mineral
Bulk_density	nifh_Cluster299	0.0035	-0.001	0.0623	-0.0419	0.4593	0.5938977	Mineral
Bulk_density	nifh_Cluster303	0.0028	-0.0007	0.0476	-0.0583	0.5195	0.6353657	Mineral
Bulk_density	nifh_Cluster304	0.0034	-0.0026	0.3775	0.3084	0.0443	0.2551649	Mineral
Bulk_density	nifh_Cluster313	0.0176	-0.0138	0.4025	0.3362	0.036	0.2492719	Mineral
Bulk_density	nifh_Cluster314	0.0037	-0.0025	0.3229	0.2476	0.0682	0.2562377	Mineral
Bulk_density	nifh_Cluster317	0.011	-0.0028	0.0891	-0.0121	0.3726	0.5298403	Mineral
Bulk_density	nifh_Cluster32	0.0039	-0.0012	0.1138	0.0154	0.3103	0.4722357	Mineral
Bulk_density	nifh_Cluster320	14.7668	-2.1122	0.6803	0.6448	0.0018	0.1416317	Mineral
Bulk_density	nifh_Cluster334	0.0115	-0.009	0.3416	0.2685	0.059	0.2551649	Mineral
Bulk_density	nifh_Cluster335	0.0081	-0.0049	0.1965	0.1072	0.1721	0.3413586	Mineral
Bulk_density	nifh_Cluster338	0.0131	0.0014	0.0147	-0.0947	0.7222	0.7892382	Mineral
Bulk_density	nifh_Cluster340	0.0038	-0.0021	0.2306	0.1451	0.1349	0.3141319	Mineral
Bulk_density	nifh_Cluster341	0.0029	-0.0003	0.0049	-0.1057	0.8388	0.8830619	Mineral
Bulk_density	nifh_Cluster35	0.0051	-0.0018	0.2017	0.113	0.1658	0.3360388	Mineral
Bulk_density	nifh_Cluster351	0.0048	-0.0038	0.3964	0.3293	0.0379	0.2492719	Mineral
Bulk_density	nifh_Cluster3574	0.1248	0.0556	0.5865	0.5405	0.006	0.1537612	Mineral
Bulk_density	nifh_Cluster3691	0.0129	-0.0044	0.1619	0.0687	0.2199	0.3866931	Mineral
Bulk_density	nifh_Cluster37	0.0452	-0.0261	0.3355	0.2617	0.0618	0.2551649	Mineral
Bulk_density	nifh_Cluster374	0.0004	0.0007	0.1978	0.1087	0.1705	0.3391926	Mineral
Bulk_density	nifh_Cluster38	0.0011	-0.0004	0.046	-0.0601	0.5268	0.6397222	Mineral
Bulk_density	nifh_Cluster382	0.0031	-0.0023	0.1453	0.0503	0.2475	0.4134182	Mineral
Bulk_density	nifh_Cluster384	0.0032	-0.0025	0.4048	0.3387	0.0353	0.2492719	Mineral
Bulk_density	nifh_Cluster386	0.0056	-0.0043	0.2933	0.2148	0.0853	0.269598	Mineral
Bulk_density	nifh_Cluster389	0.0041	-0.0033	0.2011	0.1123	0.1666	0.3360388	Mineral
Bulk_density	nifh_Cluster394	0.0037	-0.0029	0.1937	0.1041	0.1756	0.3446732	Mineral
Bulk_density	nifh_Cluster40	0.0019	-0.0008	0.0857	-0.0158	0.3822	0.5347096	Mineral
Bulk_density	nifh_Cluster43	0.0116	-0.0032	0.1274	0.0305	0.2812	0.4456161	Mineral
Bulk_density	nifh_Cluster434	0.0042	-0.0023	0.1473	0.0525	0.2439	0.4093247	Mineral
Bulk_density	nifh_Cluster440	0.0138	-0.0054	0.0935	-0.0072	0.3605	0.517984	Mineral
Bulk_density	nifh_Cluster462	0.0054	-0.0039	0.2806	0.2007	0.0937	0.2729786	Mineral

Bulk_density	nifh_Cluster470	0.0119	-0.001	0.0192	-0.0898	0.6845	0.7606915	Mineral
Bulk_density	nifh_Cluster49	0.03	-0.023	0.3908	0.3231	0.0397	0.2542261	Mineral
Bulk_density	nifh_Cluster499	0.0038	-0.0029	0.3965	0.3294	0.0379	0.2492719	Mineral
Bulk_density	nifh_Cluster5	0.0218	0.0022	0.0328	-0.0747	0.5941	0.6818783	Mineral
Bulk_density	nifh_Cluster506	0.0049	0.0005	0.0112	-0.0986	0.7563	0.8173895	Mineral
Bulk_density	nifh_Cluster51	0.0042	-0.0019	0.0749	-0.0278	0.4153	0.5585144	Mineral
Bulk_density	nifh_Cluster52	0.054	-0.0358	0.5231	0.4701	0.0119	0.1750427	Mineral
Bulk_density	nifh_Cluster525	0.0034	-0.001	0.0287	-0.0792	0.6185	0.6984709	Mineral
Bulk_density	nifh_Cluster531	0.0172	0.0115	0.4923	0.4359	0.0161	0.1875478	Mineral
Bulk_density	nifh_Cluster539	0.0076	-0.0057	0.1603	0.067	0.2224	0.3888823	Mineral
Bulk_density	nifh_Cluster578	0.0483	-0.0214	0.4733	0.4148	0.0193	0.2027218	Mineral
Bulk_density	nifh_Cluster58	0.2354	0.0924	0.0826	-0.0193	0.3914	0.5411581	Mineral
Bulk_density	nifh_Cluster60	0.0139	-0.0096	0.515	0.4611	0.0129	0.1797301	Mineral
Bulk_density	nifh_Cluster61	0.0023	0.0005	0.0384	-0.0685	0.5637	0.6610552	Mineral
Bulk_density	nifh_Cluster65	0.0107	-0.0079	0.5684	0.5205	0.0074	0.1537612	Mineral
Bulk_density	nifh_Cluster686	0.0017	0.00E+00	0.0001	-0.111	0.9721	0.9804252	Mineral
Bulk_density	nifh_Cluster69	0.0126	-0.0096	0.4294	0.366	0.0286	0.2407924	Mineral
Bulk_density	nifh_Cluster70	0.0196	-0.0146	0.3113	0.2348	0.0745	0.2627045	Mineral
Bulk_density	nifh_Cluster717	0.0022	0.0018	0.3249	0.2499	0.0671	0.2551649	Mineral
Bulk_density	nifh_Cluster725	0.0039	-0.0015	0.1098	0.0109	0.3195	0.4823695	Mineral
Bulk_density	nifh_Cluster727	0.003	-0.0006	0.0247	-0.0837	0.6445	0.7203547	Mineral
Bulk_density	nifh_Cluster73	0.0178	-0.0058	0.1341	0.0379	0.268	0.4322215	Mineral
Bulk_density	nifh_Cluster74	0.0068	-0.0039	0.2139	0.1265	0.1521	0.323981	Mineral
Bulk_density	nifh_Cluster748	0.0107	-0.0006	0.018	-0.0911	0.6938	0.7652046	Mineral
Bulk_density	nifh_Cluster749	0.0055	0.0029	0.1807	0.0896	0.1925	0.3620835	Mineral
Bulk_density	nifh_Cluster755	0.0062	0.0034	0.2081	0.1202	0.1584	0.3274525	Mineral
Bulk_density	nifh_Cluster76	0.003	-0.0002	0.0037	-0.107	0.8584	0.8917142	Mineral
Bulk_density	nifh_Cluster79	0.0028	-0.0009	0.0969	-0.0035	0.3515	0.512516	Mineral
Bulk_density	nifh_Cluster81	0.0052	-0.004	0.3752	0.3058	0.0451	0.2551649	Mineral
Bulk_density	nifh_Cluster82	0.0019	-1.00E-04	0.0003	-0.1108	0.9592	0.969027	Mineral
Bulk_density	nifh_Cluster83	0.0032	-0.0012	0.0888	-0.0124	0.3733	0.5298403	Mineral
Bulk_density	nifh_Cluster86	0.0098	-0.0016	0.0366	-0.0704	0.5729	0.6655398	Mineral
Bulk_density	nifh_Cluster868	0.01	0.0003	0.0007	-0.1103	0.939	0.9509128	Mineral
Bulk_density	nifh_Cluster87	0.0041	-0.0033	0.3566	0.2851	0.0524	0.2551649	Mineral
Bulk_density	nifh_Cluster88	0.0018	-0.0013	0.3054	0.2282	0.0779	0.2644395	Mineral
Bulk_density	nifh_Cluster9	0.0071	-0.0012	0.0109	-0.099	0.7596	0.8192478	Mineral
Bulk_density	nifh_Cluster92	0.0093	-0.0072	0.3572	0.2858	0.0521	0.2551649	Mineral
Bulk_density	nifh_Cluster93	0.0039	-0.003	0.3675	0.2972	0.048	0.2551649	Mineral
Bulk_density	nifh_Cluster94	0.0069	-0.0047	0.2855	0.2062	0.0904	0.2713087	Mineral
Bulk_density	nifh_Cluster95	0.0191	-0.0063	0.0812	-0.0209	0.3957	0.5457587	Mineral
Bulk_density	nifh_Cluster96	0.0084	0.0014	0.0425	-0.0639	0.5431	0.6510747	Mineral
Bulk_density	nifh_Cluster97	0.0133	0.0036	0.2312	0.1458	0.1344	0.3140233	Mineral
Bulk_density	nifh_Cluster98	0.0025	1.00E-04	0.0007	-0.1104	0.9396	0.9509128	Mineral
Bulk_density	nifh_Cluster99	0.0047	-0.0035	0.2836	0.2041	0.0917	0.2713087	Mineral
Bulk_density	nirk_Cluster0	6.9525	-0.0362	0.0019	-0.109	0.8982	0.9184838	Mineral
Bulk_density	nirk_Cluster1	0.2397	0.0404	0.1005	0.0005	0.3422	0.5026444	Mineral
Bulk_density	nirk_Cluster10	0.5433	-0.0696	0.3215	0.2461	0.0689	0.2562377	Mineral
Bulk_density	nirk_Cluster101	0.0044	-0.0024	0.1786	0.0873	0.1954	0.362466	Mineral
Bulk_density	nirk_Cluster102	0.0597	-0.0229	0.3149	0.2388	0.0725	0.261904	Mineral
Bulk_density	nirk_Cluster103	0.0936	-0.0018	0.001	-0.11	0.9252	0.9411333	Mineral
Bulk_density	nirk_Cluster104	0.0023	0.00E+00	1.00E-04	-0.111	0.9783	0.9817384	Mineral
Bulk_density	nirk_Cluster105	0.1281	-0.0444	0.3249	0.2499	0.0671	0.2551649	Mineral
Bulk_density	nirk_Cluster106	0.0846	-0.0244	0.1188	0.0209	0.2993	0.461502	Mineral
Bulk_density	nirk_Cluster107	0.1178	-0.0338	0.1724	0.0805	0.2041	0.3744721	Mineral
Bulk_density	nirk_Cluster108	0.0183	-0.0097	0.3725	0.3028	0.0461	0.2551649	Mineral

Bulk_density	nirk_Cluster109	0.0158	-0.0072	0.3055	0.2283	0.0779	0.2644395	Mineral
Bulk_density	nirk_Cluster11	0.1512	-0.0169	0.2191	0.1323	0.1465	0.3212499	Mineral
Bulk_density	nirk_Cluster12	0.1219	-0.0163	0.1883	0.0981	0.1824	0.3545431	Mineral
Bulk_density	nirk_Cluster13	0.0449	-0.0043	0.067	-0.0367	0.4423	0.5854165	Mineral
Bulk_density	nirk_Cluster14	0.0996	-0.0053	0.0276	-0.0805	0.6255	0.7031162	Mineral
Bulk_density	nirk_Cluster15	0.4719	-0.1129	0.2005	0.1116	0.1673	0.3360388	Mineral
Bulk_density	nirk_Cluster16	0.6796	-0.1043	0.0774	-0.0252	0.4076	0.5540838	Mineral
Bulk_density	nirk_Cluster17	0.1052	-0.0253	0.4012	0.3346	0.0364	0.2492719	Mineral
Bulk_density	nirk_Cluster18	0.1779	-0.073	0.644	0.6045	0.0029	0.1416317	Mineral
Bulk_density	nirk_Cluster19	0.3215	-0.0355	0.2229	0.1366	0.1426	0.3205114	Mineral
Bulk_density	nirk_Cluster2	0.1869	0.0062	0.0102	-0.0998	0.7674	0.8233638	Mineral
Bulk_density	nirk_Cluster20	0.1832	-0.0371	0.3958	0.3287	0.0381	0.2492719	Mineral
Bulk_density	nirk_Cluster21	0.1706	-0.0235	0.2635	0.1817	0.1063	0.2808347	Mineral
Bulk_density	nirk_Cluster22	0.1566	-0.0179	0.0652	-0.0387	0.4487	0.5893969	Mineral
Bulk_density	nirk_Cluster23	0.0736	-0.0118	0.3424	0.2693	0.0586	0.2551649	Mineral
Bulk_density	nirk_Cluster24	0.0667	-0.0038	0.0207	-0.0881	0.6728	0.7505107	Mineral
Bulk_density	nirk_Cluster25	0.5298	-0.0194	0.0107	-0.0992	0.7622	0.8192478	Mineral
Bulk_density	nirk_Cluster26	0.1167	-0.015	0.1657	0.073	0.2141	0.3809504	Mineral
Bulk_density	nirk_Cluster27	0.2027	-0.0624	0.4435	0.3817	0.0253	0.2256222	Mineral
Bulk_density	nirk_Cluster28	0.0768	-0.0215	0.3392	0.2657	0.0601	0.2551649	Mineral
Bulk_density	nirk_Cluster29	0.1331	-0.0117	0.0867	-0.0148	0.3794	0.5333713	Mineral
Bulk_density	nirk_Cluster3	0.3218	-0.0066	0.004	-0.1067	0.8532	0.8905392	Mineral
Bulk_density	nirk_Cluster30	0.0793	-0.0182	0.1695	0.0772	0.2084	0.3806501	Mineral
Bulk_density	nirk_Cluster31	0.1435	0.0202	0.1872	0.0969	0.1838	0.3561937	Mineral
Bulk_density	nirk_Cluster32	0.1912	-0.0725	0.4734	0.4149	0.0193	0.2027218	Mineral
Bulk_density	nirk_Cluster33	0.0948	-0.033	0.2572	0.1747	0.1113	0.2875608	Mineral
Bulk_density	nirk_Cluster34	0.0468	-0.0215	0.4165	0.3517	0.032	0.2492719	Mineral
Bulk_density	nirk_Cluster35	0.1272	-0.0503	0.2112	0.1235	0.155	0.32741	Mineral
Bulk_density	nirk_Cluster36	0.002	-0.0015	0.1886	0.0984	0.182	0.3545431	Mineral
Bulk_density	nirk_Cluster39	0.0016	-0.0011	0.0474	-0.0584	0.5199	0.6353657	Mineral
Bulk_density	nirk_Cluster4	0.1535	-0.0565	0.5556	0.5062	0.0085	0.1537612	Mineral
Bulk_density	nirk_Cluster40	0.0556	-0.0151	0.2976	0.2196	0.0826	0.269598	Mineral
Bulk_density	nirk_Cluster41	0.1875	-0.027	0.2347	0.1497	0.131	0.3110571	Mineral
Bulk_density	nirk_Cluster44	0.0754	0.012	0.058	-0.0467	0.4756	0.6088857	Mineral
Bulk_density	nirk_Cluster46	0.0189	-0.0085	0.1519	0.0577	0.236	0.4055751	Mineral
Bulk_density	nirk_Cluster48	0.0031	0.0008	0.0402	-0.0664	0.5544	0.651722	Mineral
Bulk_density	nirk_Cluster49	0.0098	0.0024	0.0502	-0.0553	0.5077	0.630245	Mineral
Bulk_density	nirk_Cluster5	0.224	-0.0389	0.2989	0.221	0.0818	0.269598	Mineral
Bulk_density	nirk_Cluster50	0.0016	-0.0011	0.1796	0.0884	0.194	0.3620835	Mineral
Bulk_density	nirk_Cluster52	0.0577	-0.0145	0.2742	0.1936	0.0983	0.2742109	Mineral
Bulk_density	nirk_Cluster53	0.0045	-0.0034	0.2018	0.1131	0.1657	0.3360388	Mineral
Bulk_density	nirk_Cluster54	0.0024	-0.0011	0.1029	0.0032	0.3361	0.4961335	Mineral
Bulk_density	nirk_Cluster55	0.0034	-0.0008	0.013	-0.0967	0.7388	0.8043412	Mineral
Bulk_density	nirk_Cluster56	0.0012	0.0005	0.036	-0.0711	0.5762	0.6680548	Mineral
Bulk_density	nirk_Cluster57	0.0023	-0.0008	0.0269	-0.0812	0.63	0.7068022	Mineral
Bulk_density	nirk_Cluster58	0.0077	0.0062	0.3728	0.3031	0.046	0.2551649	Mineral
Bulk_density	nirk_Cluster59	0.0052	-0.0015	0.0405	-0.0661	0.5527	0.6510747	Mineral
Bulk_density	nirk_Cluster6	0.1499	0.0078	0.0187	-0.0903	0.6884	0.7636156	Mineral
Bulk_density	nirk_Cluster60	0.0004	-0.0003	0.0485	-0.0573	0.5154	0.6337615	Mineral
Bulk_density	nirk_Cluster62	0.1782	0.0778	0.3367	0.263	0.0613	0.2551649	Mineral
Bulk_density	nirk_Cluster63	0.1505	0.0123	0.0354	-0.0718	0.5796	0.669405	Mineral
Bulk_density	nirk_Cluster64	0.0037	-0.0018	0.1281	0.0312	0.2799	0.4456161	Mineral
Bulk_density	nirk_Cluster65	0.0518	-0.0167	0.3222	0.2468	0.0686	0.2562377	Mineral
Bulk_density	nirk_Cluster66	0.0113	-0.0019	0.0495	-0.0561	0.5106	0.6305312	Mineral
Bulk_density	nirk_Cluster67	0.0131	-0.0022	0.0547	-0.0503	0.4887	0.619847	Mineral

Bulk_density	nirk_Cluster68	0.0144	0.002	0.0316	-0.076	0.6009	0.6845409	Mineral
Bulk_density	nirk_Cluster69	0.0325	-0.0019	0.0084	-0.1018	0.7892	0.8405503	Mineral
Bulk_density	nirk_Cluster7	0.0837	-0.027	0.5635	0.515	0.0078	0.1537612	Mineral
Bulk_density	nirk_Cluster70	0.094	0.0693	0.4482	0.3868	0.0243	0.2232207	Mineral
Bulk_density	nirk_Cluster71	0.0072	-0.0043	0.3169	0.241	0.0714	0.2611641	Mineral
Bulk_density	nirk_Cluster72	0.0275	0.0009	0.003	-0.1078	0.8727	0.9018263	Mineral
Bulk_density	nirk_Cluster73	0.2073	-0.0562	0.3052	0.228	0.078	0.2644395	Mineral
Bulk_density	nirk_Cluster74	0.8561	0.1922	0.1501	0.0557	0.2391	0.4079933	Mineral
Bulk_density	nirk_Cluster75	0.018	-0.0083	0.1076	0.0085	0.3247	0.4871704	Mineral
Bulk_density	nirk_Cluster76	0.141	-0.0154	0.0759	-0.0268	0.4123	0.5569329	Mineral
Bulk_density	nirk_Cluster77	0.0018	-0.0014	0.1794	0.0882	0.1943	0.3620835	Mineral
Bulk_density	nirk_Cluster78	0.3226	-0.1295	0.6442	0.6047	0.0029	0.1416317	Mineral
Bulk_density	nirk_Cluster79	0.0036	-0.0001	0.0016	-0.1094	0.9078	0.9266915	Mineral
Bulk_density	nirk_Cluster8	0.1221	-0.0604	0.6164	0.5738	0.0042	0.1493385	Mineral
Bulk_density	nirk_Cluster80	0.3876	-0.1294	0.4037	0.3374	0.0357	0.2492719	Mineral
Bulk_density	nirk_Cluster82	0.0025	0.0019	0.1447	0.0496	0.2485	0.4135272	Mineral
Bulk_density	nirk_Cluster83	0.0032	-0.0009	0.1075	0.0083	0.3251	0.4871704	Mineral
Bulk_density	nirk_Cluster84	0.0274	-0.0118	0.1809	0.0899	0.1922	0.3620835	Mineral
Bulk_density	nirk_Cluster85	0.0031	-0.0017	0.1175	0.0195	0.302	0.4644819	Mineral
Bulk_density	nirk_Cluster86	0.0333	0.0113	0.1491	0.0545	0.2409	0.4079933	Mineral
Bulk_density	nirk_Cluster87	0.0155	0.0024	0.0436	-0.0626	0.5376	0.648839	Mineral
Bulk_density	nirk_Cluster88	0.0032	-0.0007	0.0448	-0.0614	0.5322	0.6437065	Mineral
Bulk_density	nirk_Cluster89	0.0111	-0.0069	0.465	0.4056	0.0208	0.2047749	Mineral
Bulk_density	nirk_Cluster9	0.0646	-0.0263	0.5387	0.4874	0.0101	0.1572038	Mineral
Bulk_density	nirk_Cluster90	0.0112	-0.0004	0.0009	-0.1101	0.9312	0.9456495	Mineral
Bulk_density	nirk_Cluster91	0.0491	-0.0141	0.285	0.2056	0.0907	0.2713087	Mineral
Bulk_density	nirk_Cluster92	0.0106	0.0055	0.2323	0.147	0.1333	0.3133184	Mineral
Bulk_density	nirk_Cluster93	0.0346	0.0014	0.0032	-0.1076	0.8698	0.9018263	Mineral
Bulk_density	nirk_Cluster94	0.0209	-0.008	0.3521	0.2801	0.0543	0.2551649	Mineral
Bulk_density	nirk_Cluster95	0.0648	0.003	0.0046	-0.106	0.8426	0.8830619	Mineral
Bulk_density	nirk_Cluster96	0.0644	-0.0183	0.2014	0.1126	0.1662	0.3360388	Mineral
Bulk_density	nirk_Cluster97	0.0412	-0.0015	0.0038	-0.1069	0.8575	0.8917142	Mineral
Bulk_density	nirk_Cluster98	0.0209	-0.0117	0.2005	0.1116	0.1673	0.3360388	Mineral
Bulk_density	nirk_Cluster99	0.0061	-0.0017	0.0776	-0.0249	0.4068	0.5540838	Mineral
Bulk_density	nirs_Cluster0	0.1455	-0.0134	0.0407	-0.0659	0.5522	0.6510747	Mineral
Bulk_density	nirs_Cluster1	0.0047	-0.0007	0.007	-0.1034	0.807	0.8579873	Mineral
Bulk_density	nirs_Cluster14	0.0193	-0.0142	0.238	0.1533	0.128	0.3080612	Mineral
Bulk_density	nirs_Cluster2	0.0062	-0.0031	0.1287	0.0319	0.2787	0.4456161	Mineral
Bulk_density	nirs_Cluster26	0.0009	-0.0008	0.1342	0.038	0.2678	0.4322215	Mineral
Bulk_density	nirs_Cluster28	0.0135	-0.0099	0.2543	0.1714	0.1137	0.2906842	Mineral
Bulk_density	nirs_Cluster3	0.1792	-0.036	0.6339	0.5932	0.0034	0.1416317	Mineral
Bulk_density	nirs_Cluster36	0.0004	-0.0003	0.0415	-0.065	0.5478	0.6510747	Mineral
Bulk_density	nirs_Cluster37	0.0126	-0.0089	0.1168	0.0187	0.3037	0.4657666	Mineral
Bulk_density	nirs_Cluster4	0.0004	-0.0003	0.0415	-0.065	0.5478	0.6510747	Mineral
Bulk_density	nirs_Cluster50	0.0026	-0.0005	0.0101	-0.0999	0.7692	0.8237111	Mineral
Bulk_density	nirs_Cluster9	0.0513	-0.0397	0.2781	0.1979	0.0955	0.2729786	Mineral
Bulk_density	norb_Cluster0	0.1091	0.00E+00	0.00E+00	-0.1111	0.9993	0.9993069	Mineral
Bulk_density	norb_Cluster1	1.6436	-0.1945	0.0375	-0.0694	0.5683	0.6628317	Mineral
Bulk_density	norb_Cluster101	0.0043	-0.0021	0.0955	-0.005	0.3551	0.5163981	Mineral
Bulk_density	norb_Cluster102	0.0068	-0.0014	0.0517	-0.0537	0.5013	0.62561	Mineral
Bulk_density	norb_Cluster103	0.0034	-0.0022	0.225	0.1389	0.1404	0.3205114	Mineral
Bulk_density	norb_Cluster104	0.0028	-0.0024	0.1476	0.0529	0.2434	0.4093247	Mineral
Bulk_density	norb_Cluster106	0.167	-0.0718	0.6607	0.623	0.0024	0.1416317	Mineral
Bulk_density	norb_Cluster107	0.0165	-0.0101	0.3665	0.2962	0.0484	0.2551649	Mineral
Bulk_density	norb_Cluster108	0.0065	-0.0043	0.4551	0.3946	0.0228	0.2148116	Mineral

Bulk_density	norb_Cluster109	0.029	-0.0184	0.573	0.5255	0.007	0.1537612	Mineral
Bulk_density	norb_Cluster11	0.0185	-0.0081	0.2378	0.1531	0.1281	0.3080612	Mineral
Bulk_density	norb_Cluster110	0.0115	-0.0084	0.3443	0.2714	0.0578	0.2551649	Mineral
Bulk_density	norb_Cluster112	0.0229	-0.0104	0.4919	0.4355	0.0162	0.1875478	Mineral
Bulk_density	norb_Cluster113	0.0185	-0.0094	0.3402	0.2669	0.0596	0.2551649	Mineral
Bulk_density	norb_Cluster114	0.0048	-0.0033	0.1577	0.0641	0.2266	0.3925288	Mineral
Bulk_density	norb_Cluster115	0.0039	-0.0008	0.0634	-0.0407	0.4553	0.5938977	Mineral
Bulk_density	norb_Cluster116	0.0109	-0.0025	0.0501	-0.0555	0.5083	0.630245	Mineral
Bulk_density	norb_Cluster117	0.0037	-0.0028	0.2539	0.171	0.114	0.2906842	Mineral
Bulk_density	norb_Cluster118	0.0047	-0.0026	0.2321	0.1467	0.1335	0.3133184	Mineral
Bulk_density	norb_Cluster119	0.0272	-0.0207	0.5967	0.5519	0.0053	0.1537612	Mineral
Bulk_density	norb_Cluster12	0.0058	-0.0034	0.1451	0.0501	0.2478	0.4134182	Mineral
Bulk_density	norb_Cluster121	0.0032	-0.0017	0.0869	-0.0145	0.3787	0.5333713	Mineral
Bulk_density	norb_Cluster122	0.021	-0.0085	0.2644	0.1827	0.1056	0.2802181	Mineral
Bulk_density	norb_Cluster123	0.0077	-0.0054	0.358	0.2867	0.0518	0.2551649	Mineral
Bulk_density	norb_Cluster124	0.9665	-0.1726	0.0995	-0.0006	0.3448	0.5039857	Mineral
Bulk_density	norb_Cluster125	0.0061	-0.0046	0.5425	0.4917	0.0097	0.1572038	Mineral
Bulk_density	norb_Cluster126	0.0126	-0.0084	0.5173	0.4636	0.0126	0.1797301	Mineral
Bulk_density	norb_Cluster127	0.2548	-0.0911	0.321	0.2456	0.0692	0.2562377	Mineral
Bulk_density	norb_Cluster128	0.0154	-0.0064	0.2888	0.2098	0.0882	0.2713087	Mineral
Bulk_density	norb_Cluster13	0.007	-0.0047	0.2321	0.1468	0.1335	0.3133184	Mineral
Bulk_density	norb_Cluster131	0.0245	-0.0106	0.3131	0.2368	0.0735	0.2627045	Mineral
Bulk_density	norb_Cluster132	1.4155	-0.5248	0.5587	0.5096	0.0082	0.1537612	Mineral
Bulk_density	norb_Cluster133	0.0014	-0.0011	0.2176	0.1306	0.1481	0.3212499	Mineral
Bulk_density	norb_Cluster135	0.0837	-0.0276	0.2969	0.2188	0.083	0.269598	Mineral
Bulk_density	norb_Cluster136	0.0277	-0.0121	0.3021	0.2245	0.0799	0.2673264	Mineral
Bulk_density	norb_Cluster139	0.0415	-0.0191	0.3698	0.2998	0.0472	0.2551649	Mineral
Bulk_density	norb_Cluster14	0.0051	-0.0016	0.1095	0.0106	0.3202	0.4823695	Mineral
Bulk_density	norb_Cluster140	0.0605	-0.0164	0.2928	0.2143	0.0856	0.269598	Mineral
Bulk_density	norb_Cluster142	0.0024	-0.0015	0.1048	0.0054	0.3314	0.4904655	Mineral
Bulk_density	norb_Cluster144	0.0113	-0.0036	0.1417	0.0463	0.2539	0.4163373	Mineral
Bulk_density	norb_Cluster146	0.0458	-0.0087	0.0935	-0.0073	0.3606	0.517984	Mineral
Bulk_density	norb_Cluster15	0.064	-0.0215	0.2492	0.1658	0.118	0.2954105	Mineral
Bulk_density	norb_Cluster150	0.0203	-0.0032	0.0525	-0.0528	0.498	0.6232233	Mineral
Bulk_density	norb_Cluster155	0.2191	-0.0295	0.0627	-0.0415	0.4577	0.5938977	Mineral
Bulk_density	norb_Cluster16	0.0214	-0.0104	0.2705	0.1894	0.101	0.2742109	Mineral
Bulk_density	norb_Cluster164	0.033	-0.0154	0.4962	0.4402	0.0155	0.1875478	Mineral
Bulk_density	norb_Cluster165	0.0126	-0.0075	0.3259	0.251	0.0666	0.2551649	Mineral
Bulk_density	norb_Cluster166	0.0062	-0.0049	0.5057	0.4508	0.0141	0.1875478	Mineral
Bulk_density	norb_Cluster169	0.041	-0.0206	0.4826	0.4251	0.0177	0.1989595	Mineral
Bulk_density	norb_Cluster17	0.0045	-0.0009	0.0319	-0.0757	0.5993	0.6840885	Mineral
Bulk_density	norb_Cluster170	0.0407	-0.0186	0.3421	0.269	0.0588	0.2551649	Mineral
Bulk_density	norb_Cluster171	0.0114	-0.009	0.3805	0.3117	0.0432	0.2551649	Mineral
Bulk_density	norb_Cluster172	0.0244	-0.0154	0.5361	0.4845	0.0104	0.1572038	Mineral
Bulk_density	norb_Cluster175	0.0804	-0.0426	0.469	0.41	0.0201	0.2038382	Mineral
Bulk_density	norb_Cluster176	0.0527	-0.0319	0.6262	0.5846	0.0037	0.1459043	Mineral
Bulk_density	norb_Cluster177	0.0476	-0.0306	0.6143	0.5714	0.0043	0.1493385	Mineral
Bulk_density	norb_Cluster179	0.0018	-0.0015	0.2006	0.1118	0.1671	0.3360388	Mineral
Bulk_density	norb_Cluster18	0.0055	-0.0009	0.0302	-0.0776	0.6095	0.6917508	Mineral
Bulk_density	norb_Cluster180	0.0119	-0.0058	0.2532	0.1702	0.1146	0.2910162	Mineral
Bulk_density	norb_Cluster181	0.0004	-0.0003	0.0485	-0.0573	0.5154	0.6337615	Mineral
Bulk_density	norb_Cluster182	0.0024	-0.0005	0.0181	-0.091	0.6931	0.7652046	Mineral
Bulk_density	norb_Cluster184	0.0224	-0.0112	0.4038	0.3375	0.0356	0.2492719	Mineral
Bulk_density	norb_Cluster186	0.0016	0.0004	0.0162	-0.0931	0.7089	0.7789524	Mineral
Bulk_density	norb_Cluster19	0.0066	-0.0015	0.0621	-0.0421	0.4598	0.5938977	Mineral

Bulk_density	norb_Cluster192	0.0302	-0.0185	0.7233	0.6926	0.0009	0.1416317	Mineral
Bulk_density	norb_Cluster193	0.003	-0.0023	0.3342	0.2602	0.0625	0.2551649	Mineral
Bulk_density	norb_Cluster197	0.0536	-0.0401	0.1479	0.0532	0.2429	0.4093247	Mineral
Bulk_density	norb_Cluster2	0.0043	-0.0023	0.1427	0.0475	0.252	0.4146451	Mineral
Bulk_density	norb_Cluster20	0.0842	-0.0166	0.1209	0.0232	0.2948	0.4557298	Mineral
Bulk_density	norb_Cluster208	0.002	-0.0015	0.1784	0.0871	0.1957	0.362466	Mineral
Bulk_density	norb_Cluster21	0.0681	-0.0175	0.1624	0.0693	0.2192	0.386513	Mineral
Bulk_density	norb_Cluster211	0.0174	-0.0142	0.1982	0.1091	0.17	0.3391926	Mineral
Bulk_density	norb_Cluster212	0.0343	-0.0239	0.3459	0.2732	0.057	0.2551649	Mineral
Bulk_density	norb_Cluster214	0.0194	-0.0136	0.2426	0.1584	0.1238	0.3013114	Mineral
Bulk_density	norb_Cluster217	0.0243	-0.0168	0.2865	0.2072	0.0898	0.2713087	Mineral
Bulk_density	norb_Cluster219	0.0148	-0.0109	0.2543	0.1714	0.1137	0.2906842	Mineral
Bulk_density	norb_Cluster22	0.0023	0.00E+00	0.00E+00	-0.1111	0.987	0.9886705	Mineral
Bulk_density	norb_Cluster220	0.0335	-0.025	0.2599	0.1777	0.1091	0.2857119	Mineral
Bulk_density	norb_Cluster221	0.0323	-0.0247	0.2105	0.1228	0.1558	0.32741	Mineral
Bulk_density	norb_Cluster222	0.0142	-0.0113	0.1851	0.0946	0.1865	0.3566502	Mineral
Bulk_density	norb_Cluster224	0.0325	-0.0247	0.3547	0.283	0.0532	0.2551649	Mineral
Bulk_density	norb_Cluster225	0.0108	-0.0078	0.3488	0.2764	0.0557	0.2551649	Mineral
Bulk_density	norb_Cluster226	0.0057	-0.0043	0.2656	0.184	0.1047	0.2790357	Mineral
Bulk_density	norb_Cluster228	0.0013	-0.001	0.0538	-0.0513	0.4925	0.619847	Mineral
Bulk_density	norb_Cluster229	0.0047	-0.0015	0.0606	-0.0438	0.4656	0.6001419	Mineral
Bulk_density	norb_Cluster230	0.002	-0.0015	0.1651	0.0723	0.215	0.3809504	Mineral
Bulk_density	norb_Cluster231	0.0051	-0.0019	0.0903	-0.0108	0.3692	0.5266047	Mineral
Bulk_density	norb_Cluster232	0.0039	-0.0012	0.0697	-0.0337	0.4327	0.5766536	Mineral
Bulk_density	norb_Cluster234	0.0212	-0.0126	0.3086	0.2318	0.076	0.2644395	Mineral
Bulk_density	norb_Cluster235	0.0013	-0.0011	0.2159	0.1288	0.1499	0.3212499	Mineral
Bulk_density	norb_Cluster238	0.0131	-0.0098	0.1829	0.0921	0.1895	0.3612863	Mineral
Bulk_density	norb_Cluster239	0.0121	-0.0079	0.1534	0.0593	0.2336	0.4035194	Mineral
Bulk_density	norb_Cluster24	0.3779	-0.0543	0.0753	-0.0275	0.4143	0.5583703	Mineral
Bulk_density	norb_Cluster244	0.001	-0.0007	0.1216	0.024	0.2933	0.4557298	Mineral
Bulk_density	norb_Cluster247	0.0038	-0.003	0.5485	0.4983	0.0091	0.1537612	Mineral
Bulk_density	norb_Cluster25	0.1648	-0.0406	0.1688	0.0765	0.2093	0.3806501	Mineral
Bulk_density	norb_Cluster250	0.015	-0.0101	0.3837	0.3152	0.0421	0.2551649	Mineral
Bulk_density	norb_Cluster251	0.0067	-0.0031	0.1211	0.0235	0.2943	0.4557298	Mineral
Bulk_density	norb_Cluster26	0.1486	-0.0219	0.0703	-0.0331	0.4309	0.575487	Mineral
Bulk_density	norb_Cluster28	0.042	-0.0216	0.7215	0.6905	0.0009	0.1416317	Mineral
Bulk_density	norb_Cluster29	0.2924	-0.0561	0.3935	0.3261	0.0388	0.2513551	Mineral
Bulk_density	norb_Cluster31	0.0774	-0.004	0.0068	-0.1036	0.81	0.8596502	Mineral
Bulk_density	norb_Cluster32	0.1832	-0.0214	0.0286	-0.0793	0.619	0.6984709	Mineral
Bulk_density	norb_Cluster33	0.5336	0.1533	0.4215	0.3572	0.0307	0.2466524	Mineral
Bulk_density	norb_Cluster35	0.0958	-0.0205	0.1514	0.0571	0.2369	0.4055751	Mineral
Bulk_density	norb_Cluster36	1.428	0.4844	0.3568	0.2853	0.0523	0.2551649	Mineral
Bulk_density	norb_Cluster37	0.9126	0.2293	0.3468	0.2742	0.0566	0.2551649	Mineral
Bulk_density	norb_Cluster38	0.016	-0.0051	0.2091	0.1212	0.1573	0.32741	Mineral
Bulk_density	norb_Cluster39	0.0566	-0.0255	0.4915	0.435	0.0162	0.1875478	Mineral
Bulk_density	norb_Cluster40	0.273	-0.0706	0.1857	0.0953	0.1857	0.3566502	Mineral
Bulk_density	norb_Cluster42	0.3718	0.0431	0.0496	-0.056	0.5105	0.6305312	Mineral
Bulk_density	norb_Cluster44	0.0237	-0.0154	0.3834	0.3148	0.0422	0.2551649	Mineral
Bulk_density	norb_Cluster46	0.3059	-0.0716	0.0942	-0.0064	0.3585	0.517984	Mineral
Bulk_density	norb_Cluster47	0.8152	-0.1345	0.1006	0.0006	0.342	0.5026444	Mineral
Bulk_density	norb_Cluster5	0.0134	0.0024	0.0542	-0.0509	0.4908	0.619847	Mineral
Bulk_density	norb_Cluster51	0.0155	-0.0107	0.3334	0.2593	0.0629	0.2551649	Mineral
Bulk_density	norb_Cluster52	0.3539	-0.0635	0.0835	-0.0183	0.3888	0.5408385	Mineral
Bulk_density	norb_Cluster56	0.0201	-0.0064	0.2185	0.1317	0.1471	0.3212499	Mineral
Bulk_density	norb_Cluster57	0.0047	-0.0016	0.1428	0.0475	0.2519	0.4146451	Mineral

Bulk_density	norb_Cluster59	0.025	-0.0144	0.1735	0.0817	0.2025	0.3726962	Mineral
Bulk_density	norb_Cluster6	0.8985	0.2746	0.3576	0.2862	0.052	0.2551649	Mineral
Bulk_density	norb_Cluster60	0.4675	-0.0739	0.0368	-0.0702	0.572	0.6655398	Mineral
Bulk_density	norb_Cluster61	0.6714	-0.3153	0.5927	0.5474	0.0056	0.1537612	Mineral
Bulk_density	norb_Cluster62	0.0031	0.0008	0.0509	-0.0546	0.5048	0.6286028	Mineral
Bulk_density	norb_Cluster64	0.0442	-0.0167	0.4277	0.3641	0.029	0.2409357	Mineral
Bulk_density	norb_Cluster66	0.0019	-0.0014	0.1672	0.0747	0.2117	0.3809504	Mineral
Bulk_density	norb_Cluster67	0.0256	-0.0161	0.5374	0.486	0.0103	0.1572038	Mineral
Bulk_density	norb_Cluster68	0.0037	-0.0029	0.3994	0.3326	0.037	0.2492719	Mineral
Bulk_density	norb_Cluster69	0.0035	-0.0014	0.0872	-0.0143	0.3781	0.5333713	Mineral
Bulk_density	norb_Cluster7	0.1	-0.0314	0.2355	0.1505	0.1303	0.3106899	Mineral
Bulk_density	norb_Cluster70	0.1398	-0.0396	0.228	0.1422	0.1375	0.3160908	Mineral
Bulk_density	norb_Cluster71	0.1719	-0.0714	0.7103	0.6781	0.0011	0.1416317	Mineral
Bulk_density	norb_Cluster74	0.0429	-0.0209	0.6521	0.6135	0.0026	0.1416317	Mineral
Bulk_density	norb_Cluster75	0.1722	-0.0577	0.5691	0.5212	0.0073	0.1537612	Mineral
Bulk_density	norb_Cluster76	0.163	-0.0502	0.0945	-0.0062	0.3579	0.517984	Mineral
Bulk_density	norb_Cluster77	0.0524	-0.0167	0.3425	0.2694	0.0586	0.2551649	Mineral
Bulk_density	norb_Cluster8	0.0253	-0.0122	0.4117	0.3463	0.0333	0.2492719	Mineral
Bulk_density	norb_Cluster80	0.0029	-0.0023	0.3071	0.2301	0.0769	0.2644395	Mineral
Bulk_density	norb_Cluster81	0.0017	-0.001	0.1262	0.0291	0.2837	0.4456161	Mineral
Bulk_density	norb_Cluster83	0.0137	-0.0095	0.2081	0.1201	0.1584	0.3274525	Mineral
Bulk_density	norb_Cluster84	0.0097	-0.0051	0.1799	0.0887	0.1936	0.3620835	Mineral
Bulk_density	norb_Cluster85	0.0034	-0.0018	0.1497	0.0552	0.2399	0.4079933	Mineral
Bulk_density	norb_Cluster86	0.0559	-0.0407	0.3124	0.236	0.0738	0.2627045	Mineral
Bulk_density	norb_Cluster88	0.0026	-0.0019	0.2488	0.1653	0.1184	0.2954105	Mineral
Bulk_density	norb_Cluster90	0.0013	-0.0011	0.0564	-0.0485	0.4821	0.6146234	Mineral
Bulk_density	norb_Cluster91	0.9677	-0.5114	0.717	0.6856	0.001	0.1416317	Mineral
Bulk_density	norb_Cluster92	0.0019	-0.0016	0.1051	0.0057	0.3306	0.4904655	Mineral
Bulk_density	norb_Cluster93	0.0038	-0.0011	0.0282	-0.0798	0.6219	0.7003244	Mineral
Bulk_density	norb_Cluster94	0.0012	0.0002	0.0048	-0.1058	0.8394	0.8830619	Mineral
Bulk_density	norb_Cluster96	0.0005	-0.0003	0.0538	-0.0513	0.4925	0.619847	Mineral
Bulk_density	norb_Cluster97	0.0116	-0.0045	0.2092	0.1213	0.1572	0.32741	Mineral
Bulk_density	norb_Cluster98	0.0237	-0.0138	0.3769	0.3077	0.0445	0.2551649	Mineral
Bulk_density	nosz_Cluster0	0.0052	-0.0036	0.277	0.1967	0.0963	0.2739806	Mineral
Bulk_density	nosz_Cluster10	0.0159	-0.0073	0.2937	0.2152	0.085	0.269598	Mineral
Bulk_density	nosz_Cluster102	0.0046	-0.0002	0.0046	-0.106	0.8424	0.8830619	Mineral
Bulk_density	nosz_Cluster103	0.0586	-0.0067	0.0349	-0.0723	0.5822	0.6710297	Mineral
Bulk_density	nosz_Cluster105	0.0014	-0.0011	0.1949	0.1055	0.174	0.3437937	Mineral
Bulk_density	nosz_Cluster106	0.0112	-0.0043	0.1602	0.0669	0.2225	0.3888823	Mineral
Bulk_density	nosz_Cluster107	0.0004	-0.0003	0.0415	-0.065	0.5478	0.6510747	Mineral
Bulk_density	nosz_Cluster108	0.0056	-0.0037	0.2736	0.1929	0.0988	0.2742109	Mineral
Bulk_density	nosz_Cluster109	0.0438	-0.0011	0.0024	-0.1084	0.8851	0.9129993	Mineral
Bulk_density	nosz_Cluster11	0.0093	-0.0026	0.0939	-0.0067	0.3593	0.517984	Mineral
Bulk_density	nosz_Cluster110	0.0005	0.0008	0.2208	0.1343	0.1447	0.3205114	Mineral
Bulk_density	nosz_Cluster111	0.0092	-0.004	0.2476	0.164	0.1194	0.2955092	Mineral
Bulk_density	nosz_Cluster116	0.0076	-0.0048	0.4709	0.4121	0.0197	0.2037034	Mineral
Bulk_density	nosz_Cluster118	0.0077	-0.006	0.4082	0.3425	0.0343	0.2492719	Mineral
Bulk_density	nosz_Cluster119	0.0025	-0.0019	0.2753	0.1948	0.0975	0.2742109	Mineral
Bulk_density	nosz_Cluster120	0.0087	-0.0045	0.3336	0.2595	0.0628	0.2551649	Mineral
Bulk_density	nosz_Cluster123	0.0161	-0.011	0.496	0.44	0.0156	0.1875478	Mineral
Bulk_density	nosz_Cluster128	0.0068	-0.0043	0.3541	0.2823	0.0535	0.2551649	Mineral
Bulk_density	nosz_Cluster129	0.0613	-0.0097	0.2656	0.184	0.1047	0.2790357	Mineral
Bulk_density	nosz_Cluster13	0.0119	0.004	0.1149	0.0165	0.3079	0.4698527	Mineral
Bulk_density	nosz_Cluster130	0.1523	-0.0909	0.1262	0.0291	0.2836	0.4456161	Mineral
Bulk_density	nosz_Cluster132	0.0044	0.00E+00	1.00E-04	-0.111	0.9776	0.9817384	Mineral

Bulk_density	nosz_Cluster14	0.0165	-0.0123	0.3554	0.2838	0.0529	0.2551649	Mineral
Bulk_density	nosz_Cluster16	0.0092	-0.0061	0.2467	0.163	0.1201	0.2960732	Mineral
Bulk_density	nosz_Cluster17	0.0506	-0.0259	0.5558	0.5065	0.0084	0.1537612	Mineral
Bulk_density	nosz_Cluster18	0.0181	-0.014	0.3308	0.2564	0.0642	0.2551649	Mineral
Bulk_density	nosz_Cluster20	0.0149	-0.0119	0.4921	0.4357	0.0161	0.1875478	Mineral
Bulk_density	nosz_Cluster21	0.015	-0.0105	0.4585	0.3983	0.0221	0.2134003	Mineral
Bulk_density	nosz_Cluster22	0.0403	-0.0287	0.305	0.2278	0.0781	0.2644395	Mineral
Bulk_density	nosz_Cluster23	0.0235	-0.0185	0.6382	0.598	0.0032	0.1416317	Mineral
Bulk_density	nosz_Cluster24	0.0014	-0.001	0.0845	-0.0172	0.3858	0.5384389	Mineral
Bulk_density	nosz_Cluster25	0.0023	-0.0017	0.1364	0.0405	0.2636	0.42891	Mineral
Bulk_density	nosz_Cluster26	0.0086	-0.0065	0.3773	0.3082	0.0444	0.2551649	Mineral
Bulk_density	nosz_Cluster27	0.007	-0.0051	0.3382	0.2647	0.0606	0.2551649	Mineral
Bulk_density	nosz_Cluster28	0.0411	-0.021	0.3351	0.2612	0.062	0.2551649	Mineral
Bulk_density	nosz_Cluster30	0.0362	-0.0239	0.6685	0.6316	0.0021	0.1416317	Mineral
Bulk_density	nosz_Cluster32	0.0157	-0.007	0.2415	0.1572	0.1247	0.3023771	Mineral
Bulk_density	nosz_Cluster33	0.0234	-0.0155	0.4439	0.3821	0.0252	0.2256222	Mineral
Bulk_density	nosz_Cluster34	0.052	-0.0364	0.4738	0.4154	0.0192	0.2027218	Mineral
Bulk_density	nosz_Cluster35	0.0106	-0.0079	0.5578	0.5087	0.0083	0.1537612	Mineral
Bulk_density	nosz_Cluster36	0.0261	-0.0133	0.3641	0.2935	0.0493	0.2551649	Mineral
Bulk_density	nosz_Cluster37	0.0076	-0.0057	0.3298	0.2553	0.0647	0.2551649	Mineral
Bulk_density	nosz_Cluster38	0.0151	-0.0117	0.2855	0.2061	0.0904	0.2713087	Mineral
Bulk_density	nosz_Cluster39	0.0679	-0.044	0.6347	0.5941	0.0033	0.1416317	Mineral
Bulk_density	nosz_Cluster40	0.0252	-0.0182	0.4963	0.4403	0.0155	0.1875478	Mineral
Bulk_density	nosz_Cluster41	0.0258	-0.016	0.3673	0.297	0.0481	0.2551649	Mineral
Bulk_density	nosz_Cluster42	0.0357	-0.0259	0.577	0.5299	0.0067	0.1537612	Mineral
Bulk_density	nosz_Cluster43	0.0013	-0.0011	0.1228	0.0253	0.2907	0.4542122	Mineral
Bulk_density	nosz_Cluster44	0.0078	0.0022	0.058	-0.0466	0.4755	0.6088857	Mineral
Bulk_density	nosz_Cluster45	0.0064	-0.0047	0.4202	0.3558	0.031	0.2466524	Mineral
Bulk_density	nosz_Cluster46	0.0087	-0.0045	0.3305	0.2561	0.0643	0.2551649	Mineral
Bulk_density	nosz_Cluster47	0.0016	-0.0009	0.1349	0.0388	0.2664	0.4322215	Mineral
Bulk_density	nosz_Cluster48	0.0035	-0.0027	0.2206	0.134	0.145	0.3205114	Mineral
Bulk_density	nosz_Cluster49	0.001	-0.0008	0.1338	0.0376	0.2686	0.4322215	Mineral
Bulk_density	nosz_Cluster5	0.0051	-0.004	0.2839	0.2043	0.0915	0.2713087	Mineral
Bulk_density	nosz_Cluster51	0.0037	-0.0025	0.2674	0.1859	0.1034	0.2780018	Mineral
Bulk_density	nosz_Cluster53	0.0009	-0.0006	0.0415	-0.065	0.5478	0.6510747	Mineral
Bulk_density	nosz_Cluster54	0.005	-0.0028	0.2046	0.1163	0.1624	0.3344697	Mineral
Bulk_density	nosz_Cluster57	0.2638	-0.0732	0.1239	0.0265	0.2885	0.4519446	Mineral
Bulk_density	nosz_Cluster58	0.0072	-0.0048	0.3041	0.2267	0.0787	0.2648272	Mineral
Bulk_density	nosz_Cluster59	0.0011	-0.0009	0.0559	-0.049	0.4838	0.6154946	Mineral
Bulk_density	nosz_Cluster6	0.004	-0.003	0.2168	0.1298	0.1489	0.3212499	Mineral
Bulk_density	nosz_Cluster60	0.0093	-0.0069	0.551	0.5011	0.0089	0.1537612	Mineral
Bulk_density	nosz_Cluster61	0.02	0.0006	0.0023	-0.1086	0.8887	0.9150945	Mineral
Bulk_density	nosz_Cluster62	0.0586	-0.0203	0.1688	0.0764	0.2094	0.3806501	Mineral
Bulk_density	nosz_Cluster64	0.001	0.0003	0.0325	-0.075	0.5958	0.6818783	Mineral
Bulk_density	nosz_Cluster65	0.009	-0.0061	0.3402	0.2669	0.0596	0.2551649	Mineral
Bulk_density	nosz_Cluster66	0.0529	-0.0045	0.0092	-0.1009	0.7788	0.8324851	Mineral
Bulk_density	nosz_Cluster67	0.0793	-0.0255	0.1129	0.0143	0.3124	0.4730011	Mineral
Bulk_density	nosz_Cluster69	0.0109	-0.0063	0.5994	0.5549	0.0052	0.1537612	Mineral
Bulk_density	nosz_Cluster70	0.0832	-0.064	0.2839	0.2043	0.0915	0.2713087	Mineral
Bulk_density	nosz_Cluster71	0.0121	-0.0093	0.3365	0.2627	0.0614	0.2551649	Mineral
Bulk_density	nosz_Cluster72	0.0809	-0.0275	0.1852	0.0947	0.1864	0.3566502	Mineral
Bulk_density	nosz_Cluster73	0.0034	-0.0015	0.0787	-0.0236	0.4032	0.5527454	Mineral
Bulk_density	nosz_Cluster74	0.0023	-0.0014	0.1516	0.0573	0.2365	0.4055751	Mineral
Bulk_density	nosz_Cluster75	0.1765	-0.1244	0.1414	0.046	0.2545	0.4163373	Mineral
Bulk_density	nosz_Cluster77	0.0003	0.0005	0.2208	0.1343	0.1447	0.3205114	Mineral

Bulk_density	nosz_Cluster78	0.007	-0.0031	0.2372	0.1524	0.1287	0.3081108	Mineral
Bulk_density	nosz_Cluster79	0.0403	-0.0165	0.3254	0.2504	0.0669	0.2551649	Mineral
Bulk_density	nosz_Cluster8	0.0118	-0.0073	0.6707	0.6341	0.002	0.1416317	Mineral
Bulk_density	nosz_Cluster80	0.0049	-0.003	0.2715	0.1905	0.1003	0.2742109	Mineral
Bulk_density	nosz_Cluster81	0.012	-0.0086	0.2095	0.1217	0.1569	0.32741	Mineral
Bulk_density	nosz_Cluster82	0	0	#VALUE!	#VALUE!	#VALUE!	NA	Mineral
Bulk_density	nosz_Cluster83	0.002	-0.0015	0.107	0.0078	0.3261	0.4875616	Mineral
Bulk_density	nosz_Cluster85	0.0013	-0.0009	0.0877	-0.0136	0.3765	0.5330748	Mineral
Bulk_density	nosz_Cluster86	0.0027	-0.0021	0.1661	0.0734	0.2135	0.3809504	Mineral
Bulk_density	nosz_Cluster87	0.003	-0.0019	0.1673	0.0747	0.2117	0.3809504	Mineral
Bulk_density	nosz_Cluster88	0.0295	-0.009	0.4648	0.4053	0.0209	0.2047749	Mineral
Bulk_density	nosz_Cluster89	0.0732	-0.0149	0.0626	-0.0415	0.4579	0.5938977	Mineral
Bulk_density	nosz_Cluster9	0.0037	-0.0031	0.2275	0.1417	0.1379	0.3160908	Mineral
Bulk_density	nosz_Cluster90	0.0149	-0.0024	0.0578	-0.0469	0.4766	0.6088857	Mineral
Bulk_density	nosz_Cluster91	0.0272	-0.0155	0.2782	0.198	0.0954	0.2729786	Mineral
Bulk_density	nosz_Cluster93	0.0561	-0.0254	0.2929	0.2143	0.0856	0.269598	Mineral
Bulk_density	nosz_Cluster95	0.0114	-0.0034	0.158	0.0644	0.2261	0.3925288	Mineral
Bulk_density	nosz_Cluster96	0.0064	-0.0024	0.1648	0.072	0.2154	0.3809504	Mineral
Bulk_density	nosz_Cluster97	0.0029	-0.0011	0.065	-0.0389	0.4493	0.5893969	Mineral
Bulk_density	nosz_Cluster98	0.0005	-0.0004	0.0728	-0.0302	0.4223	0.5666121	Mineral
Bulk_density	nosz_Cluster99	0.0063	-0.0039	0.4339	0.371	0.0275	0.2348435	Mineral
Bulk_density	nrfa_Cluster10	0.1125	-0.0729	0.3728	0.3031	0.046	0.2551649	Mineral
Bulk_density	nrfa_Cluster100	0.1542	-0.0927	0.2709	0.1899	0.1007	0.2742109	Mineral
Bulk_density	nrfa_Cluster104	0.149	-0.0585	0.2164	0.1294	0.1493	0.3212499	Mineral
Bulk_density	nrfa_Cluster105	0.007	-0.0052	0.3423	0.2692	0.0587	0.2551649	Mineral
Bulk_density	nrfa_Cluster109	0.2489	-0.1295	0.336	0.2623	0.0616	0.2551649	Mineral
Bulk_density	nrfa_Cluster116	0.313	-0.1202	0.3709	0.301	0.0467	0.2551649	Mineral
Bulk_density	nrfa_Cluster118	0.2884	-0.1839	0.2681	0.1867	0.1028	0.2778408	Mineral
Bulk_density	nrfa_Cluster125	0.136	-0.0925	0.2711	0.1901	0.1005	0.2742109	Mineral
Bulk_density	nrfa_Cluster154	0.0997	-0.0658	0.317	0.2411	0.0713	0.2611641	Mineral
Bulk_density	nrfa_Cluster164	0.0053	-0.0033	0.2947	0.2163	0.0844	0.269598	Mineral
Bulk_density	nrfa_Cluster172	0.005	-0.0038	0.3156	0.2395	0.0721	0.261904	Mineral
Bulk_density	nrfa_Cluster175	0.0072	-0.0056	0.2298	0.1442	0.1357	0.3146411	Mineral
Bulk_density	nrfa_Cluster20	0.0418	-0.0271	0.2576	0.1751	0.111	0.2875608	Mineral
Bulk_density	nrfa_Cluster26	0.1816	-0.1186	0.3307	0.2564	0.0642	0.2551649	Mineral
Bulk_density	nrfa_Cluster29	0.0081	-0.001	0.0107	-0.0992	0.7616	0.8192478	Mineral
Bulk_density	nrfa_Cluster34	0.0628	-0.0395	0.2577	0.1753	0.1109	0.2875608	Mineral
Bulk_density	nrfa_Cluster38	0.0045	-0.0035	0.243	0.1589	0.1234	0.3013114	Mineral
Bulk_density	nrfa_Cluster61	0.0043	-0.0034	0.2793	0.1992	0.0947	0.2729786	Mineral
Bulk_density	nrfa_Cluster62	0.074	-0.0491	0.2494	0.166	0.1178	0.2954105	Mineral
Bulk_density	nrfa_Cluster66	0.1308	-0.0594	0.3741	0.3046	0.0455	0.2551649	Mineral
Bulk_density	nrfa_Cluster69	0.0047	-0.0037	0.3984	0.3316	0.0373	0.2492719	Mineral
Bulk_density	nrfa_Cluster71	0.0376	-0.0264	0.2789	0.1988	0.0949	0.2729786	Mineral
Bulk_density	nrfa_Cluster72	0.1104	-0.068	0.295	0.2167	0.0842	0.269598	Mineral
Bulk_density	nrfa_Cluster73	0.0057	-0.0044	0.2911	0.2124	0.0867	0.2713087	Mineral
Bulk_density	nrfa_Cluster74	0.2091	-0.1219	0.3274	0.2526	0.0659	0.2551649	Mineral
Bulk_density	nrfa_Cluster76	0.1171	-0.0786	0.3703	0.3004	0.0469	0.2551649	Mineral
Bulk_density	nrfa_Cluster79	0.0188	-0.0138	0.166	0.0733	0.2136	0.3809504	Mineral
Bulk_density	nrfa_Cluster87	0.0282	-0.0209	0.2852	0.2058	0.0906	0.2713087	Mineral
Bulk_density	nrfa_Cluster89	0.0031	-0.0024	0.2241	0.1378	0.1414	0.3205114	Mineral
Bulk_density	nrfa_Cluster92	0.0055	-0.0031	0.3428	0.2697	0.0584	0.2551649	Mineral
Bulk_density	nrfa_Cluster93	0.0792	-0.0484	0.246	0.1622	0.1208	0.2964281	Mineral
Bulk_density	nrfa_Cluster94	0.0004	-0.0003	0.0538	-0.0513	0.4925	0.619847	Mineral
Bulk_density	nrfa_Cluster97	0.0272	-0.0097	0.1806	0.0895	0.1927	0.3620835	Mineral
Bulk_density	nrfa_Cluster99	0.0222	-0.0066	0.0925	-0.0084	0.3633	0.5206666	Mineral

pH	amoA_A_Cluster26	0.0053	-0.0052	0.3573	0.3078	0.0186	0.3921627	Mineral
pH	amoA_A_Cluster45	0.0071	-0.0036	0.1462	0.0806	0.1595	0.6951961	Mineral
pH	amoA_B_Cluster0	0.1272	0.0168	0.1447	0.0789	0.1619	0.6951961	Mineral
pH	amoA_B_Cluster1	0.0205	0.0108	0.4547	0.4127	0.0058	0.2011387	Mineral
pH	amoA_B_Cluster10	0.0539	-0.0006	0.0006	-0.0763	0.9335	0.9954346	Mineral
pH	amoA_B_Cluster11	0.0486	0.0227	0.5607	0.5269	0.0013	0.0706769	Mineral
pH	amoA_B_Cluster12	0.093	0.0194	0.2625	0.2057	0.0509	0.4963949	Mineral
pH	amoA_B_Cluster13	0.6417	0.0929	0.0649	-0.0071	0.3596	0.8697242	Mineral
pH	amoA_B_Cluster14	0.0339	0.0018	0.0041	-0.0725	0.8196	0.9859838	Mineral
pH	amoA_B_Cluster15	0.0413	0.0121	0.2126	0.1521	0.0836	0.5787959	Mineral
pH	amoA_B_Cluster2	0.2143	0.0476	0.1622	0.0978	0.1366	0.655222	Mineral
pH	amoA_B_Cluster20	0.0078	0.0043	0.1855	0.1229	0.109	0.6064477	Mineral
pH	amoA_B_Cluster23	0.0626	0.0246	0.5871	0.5553	0.0009	0.0607258	Mineral
pH	amoA_B_Cluster3	0.0244	0.0145	0.5162	0.479	0.0025	0.1156855	Mineral
pH	amoA_B_Cluster4	0.0169	0.0025	0.0268	-0.0481	0.5601	0.9621055	Mineral
pH	amoA_B_Cluster5	0.1032	0.0222	0.3171	0.2645	0.0288	0.4568573	Mineral
pH	amoA_B_Cluster6	0.0279	0.0071	0.1711	0.1073	0.1254	0.6384894	Mineral
pH	amoA_B_Cluster7	0.0232	0.0096	0.2141	0.1537	0.0824	0.5787959	Mineral
pH	amoA_B_Cluster8	0.0268	0.0147	0.3595	0.3103	0.0181	0.3921627	Mineral
pH	amoA_B_Cluster9	0.0693	0.0329	0.4071	0.3615	0.0105	0.2727276	Mineral
pH	nifh_Cluster0	0.0318	0.0003	0.0003	-0.0766	0.9476	0.9954346	Mineral
pH	nifh_Cluster10	0.0033	0.0002	0.0027	-0.074	0.8541	0.9942937	Mineral
pH	nifh_Cluster100	0.002	-0.0008	0.0559	-0.0167	0.3963	0.8738103	Mineral
pH	nifh_Cluster103	0.0433	0.001	0.0015	-0.0753	0.891	0.9954346	Mineral
pH	nifh_Cluster1034	0.0337	0.0077	0.0538	-0.0189	0.4053	0.8760291	Mineral
pH	nifh_Cluster105	0.0164	-0.0002	1.00E-04	-0.0768	0.9745	0.9954346	Mineral
pH	nifh_Cluster108	0.0076	-0.0039	0.26	0.2031	0.0522	0.4963949	Mineral
pH	nifh_Cluster1099	0.0478	-0.0187	0.0887	0.0186	0.2811	0.855624	Mineral
pH	nifh_Cluster11	0.0025	-0.0003	0.0066	-0.0698	0.7731	0.9859838	Mineral
pH	nifh_Cluster1112	0.0037	0.0018	0.1159	0.0479	0.2144	0.7946499	Mineral
pH	nifh_Cluster112	0.0098	-0.0053	0.1661	0.102	0.1316	0.655222	Mineral
pH	nifh_Cluster113	0.0374	0.0015	0.0055	-0.071	0.7925	0.9859838	Mineral
pH	nifh_Cluster114	0.0304	0.0018	0.017	-0.0586	0.6434	0.9834364	Mineral
pH	nifh_Cluster1141	0.0296	0.0062	0.0632	-0.0089	0.3661	0.8697242	Mineral
pH	nifh_Cluster115	0.0098	-0.0012	0.0236	-0.0515	0.5849	0.9704872	Mineral
pH	nifh_Cluster116	0.0883	0.0409	0.76	0.7416	0.00E+00	0.0081552	Mineral
pH	nifh_Cluster1163	0.0051	0.0029	0.2951	0.2409	0.0364	0.4731494	Mineral
pH	nifh_Cluster1179	0.0037	1.00E-04	0.0002	-0.0768	0.9652	0.9954346	Mineral
pH	nifh_Cluster1182	0.0209	-0.0016	0.024	-0.0511	0.5816	0.9704872	Mineral
pH	nifh_Cluster1183	0.0045	0.0001	0.0006	-0.0762	0.9293	0.9954346	Mineral
pH	nifh_Cluster1197	0.0115	-0.0001	0.0001	-0.0768	0.9667	0.9954346	Mineral
pH	nifh_Cluster1199	0.0048	0.0016	0.0677	-0.004	0.349	0.8697242	Mineral
pH	nifh_Cluster120	0.02	0.0026	0.0065	-0.07	0.776	0.9859838	Mineral
pH	nifh_Cluster1203	0.0217	0.0008	0.0015	-0.0753	0.89	0.9954346	Mineral
pH	nifh_Cluster1206	0.006	0.0033	0.3363	0.2853	0.0234	0.4335852	Mineral
pH	nifh_Cluster1207	0.0078	0.0001	0.0001	-0.0768	0.9662	0.9954346	Mineral
pH	nifh_Cluster1209	0.1617	0.0214	0.1208	0.0531	0.2044	0.7779114	Mineral
pH	nifh_Cluster121	0.0123	-0.0049	0.062	-0.0102	0.3709	0.8697242	Mineral
pH	nifh_Cluster1211	0.0068	0.0038	0.3158	0.2632	0.0292	0.4568573	Mineral
pH	nifh_Cluster1213	0.0032	0.00E+00	0.00E+00	-0.0769	0.9812	0.9954346	Mineral
pH	nifh_Cluster122	0.0041	-0.0016	0.0793	0.0085	0.3092	0.855624	Mineral
pH	nifh_Cluster123	0.0092	-0.0015	0.012	-0.064	0.6976	0.9859838	Mineral
pH	nifh_Cluster1236	0.031	-0.0224	0.2931	0.2387	0.0371	0.4731494	Mineral
pH	nifh_Cluster1238	0.0034	-0.0018	0.1141	0.046	0.2181	0.7946499	Mineral
pH	nifh_Cluster124	0.0101	-0.0002	0.0006	-0.0763	0.9336	0.9954346	Mineral

pH	nifh_Cluster1261	0.0023	0.00E+00	0.00E+00	-0.0769	0.9899	0.9954346	Mineral
pH	nifh_Cluster1262	0.008	-0.0016	0.0292	-0.0455	0.5425	0.9621055	Mineral
pH	nifh_Cluster1266	0.0072	-0.0047	0.4023	0.3564	0.0111	0.2727276	Mineral
pH	nifh_Cluster1267	0.0388	-0.025	0.1901	0.1278	0.1043	0.6064477	Mineral
pH	nifh_Cluster127	0.0033	-0.0008	0.0164	-0.0593	0.6492	0.9834364	Mineral
pH	nifh_Cluster1278	0.2105	0.0678	0.3198	0.2675	0.028	0.4568573	Mineral
pH	nifh_Cluster128	0.0043	-0.0003	0.0025	-0.0742	0.8584	0.9942937	Mineral
pH	nifh_Cluster1289	0.004	-0.0014	0.0925	0.0227	0.2703	0.855624	Mineral
pH	nifh_Cluster129	0.0086	-0.0012	0.0103	-0.0658	0.7189	0.9859838	Mineral
pH	nifh_Cluster1292	0.0106	0.003	0.073	0.0017	0.3301	0.8697242	Mineral
pH	nifh_Cluster130	0.0071	-0.0004	0.0007	-0.0762	0.925	0.9954346	Mineral
pH	nifh_Cluster1305	0.0048	-0.0005	0.009	-0.0673	0.7371	0.9859838	Mineral
pH	nifh_Cluster1306	0.0055	-0.0004	0.0042	-0.0724	0.8183	0.9859838	Mineral
pH	nifh_Cluster131	0.0067	-0.0014	0.0197	-0.0557	0.6178	0.97187	Mineral
pH	nifh_Cluster1319	0.0048	0.0017	0.0685	-0.0032	0.3461	0.8697242	Mineral
pH	nifh_Cluster132	0.0064	-0.0004	0.0022	-0.0745	0.8675	0.9942937	Mineral
pH	nifh_Cluster1320	0.0028	0.0008	0.0195	-0.0559	0.6198	0.97187	Mineral
pH	nifh_Cluster1323	0.0022	0.0012	0.1646	0.1003	0.1336	0.655222	Mineral
pH	nifh_Cluster1324	0.0235	-0.0182	0.5842	0.5522	0.0009	0.0607258	Mineral
pH	nifh_Cluster1336	0.0159	0.0019	0.0442	-0.0293	0.4521	0.9116712	Mineral
pH	nifh_Cluster1340	0.0478	0.04	0.5081	0.4702	0.0029	0.1204748	Mineral
pH	nifh_Cluster1345	0.033	-0.0147	0.1051	0.0362	0.2385	0.8192616	Mineral
pH	nifh_Cluster1370	0.0022	0.0007	0.0377	-0.0363	0.4881	0.9324575	Mineral
pH	nifh_Cluster1375	0.0017	-0.0012	0.2194	0.1594	0.0782	0.5707711	Mineral
pH	nifh_Cluster139	0.0038	0.0025	0.1628	0.0984	0.1359	0.655222	Mineral
pH	nifh_Cluster140	0.0051	-0.0002	0.0016	-0.0752	0.8875	0.9954346	Mineral
pH	nifh_Cluster141	0.0808	0.0034	0.016	-0.0596	0.6529	0.9834364	Mineral
pH	nifh_Cluster148	0.006	0.0037	0.1478	0.0823	0.1571	0.6951961	Mineral
pH	nifh_Cluster1480	0.0006	-0.0005	0.101	0.0319	0.2483	0.8381873	Mineral
pH	nifh_Cluster152	0.0602	0.0032	0.0066	-0.0698	0.7731	0.9859838	Mineral
pH	nifh_Cluster156	0.0459	-0.0243	0.5829	0.5508	0.0009	0.0607258	Mineral
pH	nifh_Cluster1562	0.0293	-0.0215	0.1974	0.1357	0.097	0.6064477	Mineral
pH	nifh_Cluster158	0.019	-0.0026	0.0212	-0.0541	0.605	0.9707448	Mineral
pH	nifh_Cluster16	0.0023	-0.0008	0.0326	-0.0419	0.5198	0.9554875	Mineral
pH	nifh_Cluster166	0.0165	0.0007	0.0006	-0.0762	0.9288	0.9954346	Mineral
pH	nifh_Cluster205	0.0121	0.0014	0.0243	-0.0507	0.5789	0.9704872	Mineral
pH	nifh_Cluster21	0.0022	-0.0003	0.0097	-0.0665	0.7271	0.9859838	Mineral
pH	nifh_Cluster222	0.0069	0.001	0.0078	-0.0685	0.7546	0.9859838	Mineral
pH	nifh_Cluster225	0.021	0.0014	0.0101	-0.066	0.721	0.9859838	Mineral
pH	nifh_Cluster230	0.0052	-0.0021	0.1406	0.0745	0.1685	0.6951961	Mineral
pH	nifh_Cluster231	0.0019	0.0011	0.0926	0.0228	0.2702	0.855624	Mineral
pH	nifh_Cluster236	0.0034	-0.0002	0.0021	-0.0746	0.8704	0.9952249	Mineral
pH	nifh_Cluster237	0.007	-0.0033	0.2087	0.1478	0.0869	0.5826888	Mineral
pH	nifh_Cluster243	0.0253	-0.0224	0.5326	0.4967	0.002	0.0988531	Mineral
pH	nifh_Cluster2432	0.0317	-0.0184	0.1893	0.1269	0.1051	0.6064477	Mineral
pH	nifh_Cluster2433	0.0028	0.0015	0.0611	-0.0111	0.3743	0.8697242	Mineral
pH	nifh_Cluster246	0.0068	-0.0003	0.0013	-0.0755	0.8984	0.9954346	Mineral
pH	nifh_Cluster25	0.0195	-0.0031	0.065	-0.0069	0.359	0.8697242	Mineral
pH	nifh_Cluster256	0.0023	-0.0012	0.1181	0.0502	0.2099	0.7886498	Mineral
pH	nifh_Cluster264	0.0025	-1.00E-04	0.0006	-0.0763	0.9313	0.9954346	Mineral
pH	nifh_Cluster265	0.0702	-0.0129	0.2117	0.1511	0.0844	0.5787959	Mineral
pH	nifh_Cluster267	0.0071	-0.0008	0.012	-0.064	0.6976	0.9859838	Mineral
pH	nifh_Cluster268	0.0109	-0.0023	0.1046	0.0357	0.2397	0.8192616	Mineral
pH	nifh_Cluster269	0.0283	-0.0072	0.0595	-0.0129	0.381	0.8713473	Mineral
pH	nifh_Cluster272	0.0012	-0.0011	0.1876	0.1251	0.1068	0.6064477	Mineral

pH	nifh_Cluster274	0.0014	-0.0002	0.0026	-0.0742	0.8581	0.9942937	Mineral
pH	nifh_Cluster275	0.0051	-0.0013	0.0804	0.0097	0.3057	0.855624	Mineral
pH	nifh_Cluster278	0.0056	-0.0006	0.0061	-0.0704	0.7822	0.9859838	Mineral
pH	nifh_Cluster279	0.0058	-0.0007	0.0109	-0.0651	0.7107	0.9859838	Mineral
pH	nifh_Cluster28	0.0017	-0.0005	0.0232	-0.052	0.5881	0.9704872	Mineral
pH	nifh_Cluster280	0.0034	0.003	0.1792	0.1161	0.1159	0.6270888	Mineral
pH	nifh_Cluster282	0.0052	0.0015	0.0805	0.0098	0.3054	0.855624	Mineral
pH	nifh_Cluster287	0.003	-0.0003	0.0043	-0.0723	0.8168	0.9859838	Mineral
pH	nifh_Cluster290	0.0096	-0.0058	0.2233	0.1636	0.0753	0.5707711	Mineral
pH	nifh_Cluster297	0.004	-0.0024	0.0921	0.0223	0.2715	0.855624	Mineral
pH	nifh_Cluster299	0.0025	-0.0009	0.0599	-0.0124	0.3792	0.8706018	Mineral
pH	nifh_Cluster303	0.002	-0.0024	0.6586	0.6324	0.0002	0.0351971	Mineral
pH	nifh_Cluster304	0.0025	-0.0009	0.0539	-0.0189	0.405	0.8760291	Mineral
pH	nifh_Cluster313	0.0129	-0.003	0.0222	-0.0531	0.5965	0.9707448	Mineral
pH	nifh_Cluster314	0.0027	-0.0005	0.0166	-0.059	0.6469	0.9834364	Mineral
pH	nifh_Cluster317	0.0089	-0.0023	0.066	-0.0058	0.3552	0.8697242	Mineral
pH	nifh_Cluster32	0.0029	-0.0024	0.4908	0.4516	0.0036	0.1427123	Mineral
pH	nifh_Cluster320	13.944	-0.2464	0.0087	-0.0676	0.7414	0.9859838	Mineral
pH	nifh_Cluster334	0.0084	0.0011	0.0062	-0.0702	0.7799	0.9859838	Mineral
pH	nifh_Cluster335	0.0059	0.0013	0.0158	-0.0599	0.6555	0.9834364	Mineral
pH	nifh_Cluster338	0.0151	0.0011	0.0086	-0.0677	0.7423	0.9859838	Mineral
pH	nifh_Cluster340	0.0041	0.0013	0.0809	0.0102	0.3043	0.855624	Mineral
pH	nifh_Cluster341	0.0021	-0.0032	0.753	0.734	0.00E+00	0.0081552	Mineral
pH	nifh_Cluster35	0.0079	0.0033	0.2608	0.204	0.0517	0.4963949	Mineral
pH	nifh_Cluster351	0.0035	1.00E-04	0.0002	-0.0767	0.9571	0.9954346	Mineral
pH	nifh_Cluster3574	0.1191	0.015	0.0571	-0.0154	0.3909	0.8738103	Mineral
pH	nifh_Cluster3691	0.0224	0.0122	0.2475	0.1896	0.0592	0.5288024	Mineral
pH	nifh_Cluster37	0.0374	-0.0069	0.0284	-0.0463	0.5481	0.9621055	Mineral
pH	nifh_Cluster374	0.0029	0.003	0.4229	0.3785	0.0087	0.2557833	Mineral
pH	nifh_Cluster38	0.0008	-0.0007	0.1314	0.0646	0.1843	0.741891	Mineral
pH	nifh_Cluster382	0.0023	-0.0005	0.0087	-0.0675	0.7406	0.9859838	Mineral
pH	nifh_Cluster384	0.0023	-0.0013	0.1356	0.0691	0.1769	0.719742	Mineral
pH	nifh_Cluster386	0.0041	0.001	0.019	-0.0565	0.6242	0.97187	Mineral
pH	nifh_Cluster389	0.003	0.00E+00	0.00E+00	-0.0769	0.9846	0.9954346	Mineral
pH	nifh_Cluster394	0.0027	1.00E-04	0.0002	-0.0767	0.9558	0.9954346	Mineral
pH	nifh_Cluster40	0.0014	-0.0002	0.0062	-0.0703	0.7807	0.9859838	Mineral
pH	nifh_Cluster43	0.0107	-0.0032	0.1363	0.0698	0.1757	0.719742	Mineral
pH	nifh_Cluster434	0.0048	0.0003	0.0015	-0.0754	0.8925	0.9954346	Mineral
pH	nifh_Cluster440	0.0171	0.0099	0.2864	0.2315	0.0398	0.4731494	Mineral
pH	nifh_Cluster462	0.0048	0.0018	0.074	0.0028	0.3265	0.8678504	Mineral
pH	nifh_Cluster470	0.017	0.0043	0.0836	0.0131	0.296	0.855624	Mineral
pH	nifh_Cluster49	0.0223	-0.0106	0.099	0.0297	0.2533	0.8394299	Mineral
pH	nifh_Cluster499	0.0028	-0.0002	0.0029	-0.0738	0.8478	0.9942937	Mineral
pH	nifh_Cluster5	0.0263	0.0018	0.0158	-0.0599	0.6549	0.9834364	Mineral
pH	nifh_Cluster506	0.0058	-0.0001	0.0004	-0.0764	0.9402	0.9954346	Mineral
pH	nifh_Cluster51	0.0031	0.0007	0.0138	-0.062	0.6764	0.9859838	Mineral
pH	nifh_Cluster52	0.0396	-0.0117	0.0577	-0.0148	0.3885	0.8738103	Mineral
pH	nifh_Cluster525	0.0025	0.0004	0.0071	-0.0693	0.7651	0.9859838	Mineral
pH	nifh_Cluster531	0.0129	-0.0093	0.3525	0.3027	0.0196	0.3992826	Mineral
pH	nifh_Cluster539	0.0056	0.0015	0.0148	-0.061	0.666	0.9859838	Mineral
pH	nifh_Cluster578	0.0593	0.0174	0.2192	0.1592	0.0784	0.5707711	Mineral
pH	nifh_Cluster58	0.2389	-0.1376	0.1997	0.1382	0.0949	0.6064477	Mineral
pH	nifh_Cluster60	0.011	-0.0041	0.1054	0.0366	0.2377	0.8192616	Mineral
pH	nifh_Cluster61	0.0017	-0.001	0.1633	0.099	0.1352	0.655222	Mineral
pH	nifh_Cluster65	0.0085	1.00E-04	1.00E-04	-0.0768	0.9756	0.9954346	Mineral

pH	nifh_Cluster686	0.0013	-1.00E-04	0.0008	-0.0761	0.9205	0.9954346	Mineral
pH	nifh_Cluster69	0.0092	-0.0063	0.2101	0.1494	0.0857	0.5813067	Mineral
pH	nifh_Cluster70	0.0144	0.0005	0.0004	-0.0765	0.9456	0.9954346	Mineral
pH	nifh_Cluster717	0.0024	-0.0006	0.0247	-0.0503	0.5756	0.9704872	Mineral
pH	nifh_Cluster725	0.0035	-0.002	0.2319	0.1728	0.0691	0.5512046	Mineral
pH	nifh_Cluster727	0.0038	0.0003	0.0031	-0.0736	0.8439	0.9942937	Mineral
pH	nifh_Cluster73	0.0133	-0.0016	0.0103	-0.0658	0.7191	0.9859838	Mineral
pH	nifh_Cluster74	0.005	-0.0004	0.0025	-0.0743	0.8607	0.9942937	Mineral
pH	nifh_Cluster748	0.0104	0.0004	0.0041	-0.0725	0.8205	0.9859838	Mineral
pH	nifh_Cluster749	0.0055	0.00E+00	1.00E-04	-0.0769	0.9795	0.9954346	Mineral
pH	nifh_Cluster755	0.0118	0.0078	0.4056	0.3599	0.0107	0.2727276	Mineral
pH	nifh_Cluster76	0.0033	-0.0003	0.005	-0.0715	0.8024	0.9859838	Mineral
pH	nifh_Cluster79	0.002	-0.0004	0.0186	-0.0569	0.628	0.9725055	Mineral
pH	nifh_Cluster81	0.0038	-0.0003	0.0019	-0.0748	0.8763	0.9954346	Mineral
pH	nifh_Cluster82	0.0014	-0.001	0.13	0.0631	0.1868	0.741891	Mineral
pH	nifh_Cluster83	0.0055	0.0024	0.1171	0.0492	0.2118	0.7909581	Mineral
pH	nifh_Cluster86	0.0161	0.0072	0.2676	0.2113	0.0483	0.4852281	Mineral
pH	nifh_Cluster868	0.0098	-0.0027	0.0822	0.0116	0.3002	0.855624	Mineral
pH	nifh_Cluster87	0.003	-0.0008	0.0264	-0.0484	0.5626	0.9621055	Mineral
pH	nifh_Cluster88	0.0054	0.0046	0.3639	0.315	0.0173	0.3921627	Mineral
pH	nifh_Cluster9	0.0077	-0.0018	0.0288	-0.0459	0.5453	0.9621055	Mineral
pH	nifh_Cluster92	0.0068	-0.0009	0.0065	-0.07	0.776	0.9859838	Mineral
pH	nifh_Cluster93	0.0035	0.0009	0.0395	-0.0344	0.4776	0.9299126	Mineral
pH	nifh_Cluster94	0.005	-0.0009	0.0115	-0.0645	0.7032	0.9859838	Mineral
pH	nifh_Cluster95	0.0149	0.0015	0.0054	-0.0711	0.7952	0.9859838	Mineral
pH	nifh_Cluster96	0.0131	0.0043	0.0991	0.0298	0.2531	0.8394299	Mineral
pH	nifh_Cluster97	0.0125	-0.0012	0.0271	-0.0478	0.5578	0.9621055	Mineral
pH	nifh_Cluster98	0.0049	0.0026	0.2349	0.1761	0.0671	0.5496702	Mineral
pH	nifh_Cluster99	0.0035	-0.0006	0.0116	-0.0644	0.702	0.9859838	Mineral
pH	nirk_Cluster0	6.5686	0.0072	1.00E-04	-0.0769	0.9792	0.9954346	Mineral
pH	nirk_Cluster1	0.2105	0.0066	0.003	-0.0736	0.8452	0.9942937	Mineral
pH	nirk_Cluster10	0.6649	0.1584	0.4556	0.4137	0.0058	0.2011387	Mineral
pH	nirk_Cluster101	0.004	-0.0026	0.2164	0.1561	0.0806	0.5787959	Mineral
pH	nirk_Cluster102	0.055	0.007	0.0367	-0.0374	0.4937	0.936397	Mineral
pH	nirk_Cluster103	0.09	0.0187	0.1286	0.0616	0.1894	0.741891	Mineral
pH	nirk_Cluster104	0.0028	0.0012	0.064	-0.0081	0.3632	0.8697242	Mineral
pH	nirk_Cluster105	0.1172	0.0128	0.0338	-0.0406	0.5121	0.9501544	Mineral
pH	nirk_Cluster106	0.073	0.0164	0.066	-0.0058	0.3552	0.8697242	Mineral
pH	nirk_Cluster107	0.1123	0.017	0.0561	-0.0165	0.3952	0.8738103	Mineral
pH	nirk_Cluster108	0.0184	0.002	0.0146	-0.0612	0.668	0.9859838	Mineral
pH	nirk_Cluster109	0.0158	0.0042	0.108	0.0394	0.2317	0.8165349	Mineral
pH	nirk_Cluster11	0.1509	0.0028	0.0077	-0.0687	0.7565	0.9859838	Mineral
pH	nirk_Cluster12	0.1052	-0.0175	0.1611	0.0966	0.1381	0.6572345	Mineral
pH	nirk_Cluster13	0.0418	-0.0035	0.0479	-0.0253	0.4331	0.8964971	Mineral
pH	nirk_Cluster14	0.1084	0.0251	0.5712	0.5382	0.0011	0.0659193	Mineral
pH	nirk_Cluster15	0.4141	0.0497	0.0436	-0.03	0.4551	0.9133549	Mineral
pH	nirk_Cluster16	0.5816	0.0502	0.0189	-0.0565	0.6247	0.97187	Mineral
pH	nirk_Cluster17	0.1044	-0.0105	0.0843	0.0138	0.2939	0.855624	Mineral
pH	nirk_Cluster18	0.1461	-0.016	0.0282	-0.0465	0.5494	0.9621055	Mineral
pH	nirk_Cluster19	0.2953	-0.007	0.0073	-0.0691	0.7626	0.9859838	Mineral
pH	nirk_Cluster2	0.2004	0.0262	0.1483	0.0828	0.1563	0.6951961	Mineral
pH	nirk_Cluster20	0.1934	0.0229	0.1087	0.0402	0.2301	0.8165349	Mineral
pH	nirk_Cluster21	0.1711	0.0062	0.0192	-0.0562	0.6222	0.97187	Mineral
pH	nirk_Cluster22	0.1469	0.0146	0.0555	-0.0172	0.3981	0.8738103	Mineral
pH	nirk_Cluster23	0.0729	0.0043	0.04	-0.0339	0.4749	0.9285205	Mineral

pH	nirk_Cluster24	0.0753	0.0237	0.6907	0.6669	0.0001	0.0243287	Mineral
pH	nirk_Cluster25	0.5519	0.0252	0.0225	-0.0527	0.5935	0.9707448	Mineral
pH	nirk_Cluster26	0.1119	0.0044	0.0169	-0.0587	0.6443	0.9834364	Mineral
pH	nirk_Cluster27	0.2066	0.0287	0.1089	0.0403	0.2297	0.8165349	Mineral
pH	nirk_Cluster28	0.0808	0.0201	0.3205	0.2682	0.0278	0.4568573	Mineral
pH	nirk_Cluster29	0.162	0.026	0.1716	0.1078	0.1248	0.6384894	Mineral
pH	nirk_Cluster3	0.3427	0.0621	0.4037	0.3578	0.0109	0.2727276	Mineral
pH	nirk_Cluster30	0.0712	0.011	0.0758	0.0048	0.3205	0.855624	Mineral
pH	nirk_Cluster31	0.1423	-0.0038	0.0073	-0.0691	0.7622	0.9859838	Mineral
pH	nirk_Cluster32	0.1568	-0.0086	0.0063	-0.0702	0.7788	0.9859838	Mineral
pH	nirk_Cluster33	0.0812	0.0072	0.0146	-0.0612	0.6679	0.9859838	Mineral
pH	nirk_Cluster34	0.0462	0.0067	0.0541	-0.0186	0.404	0.8760291	Mineral
pH	nirk_Cluster35	0.1282	-0.0032	0.0011	-0.0757	0.9048	0.9954346	Mineral
pH	nirk_Cluster36	0.0021	0.0008	0.0488	-0.0243	0.4287	0.8906315	Mineral
pH	nirk_Cluster39	0.0045	0.0029	0.0825	0.0119	0.2992	0.855624	Mineral
pH	nirk_Cluster4	0.1521	0.0185	0.0774	0.0064	0.3154	0.855624	Mineral
pH	nirk_Cluster40	0.0544	0.0077	0.1007	0.0316	0.249	0.8381873	Mineral
pH	nirk_Cluster41	0.1797	0.017	0.1071	0.0384	0.2339	0.8165349	Mineral
pH	nirk_Cluster44	0.0756	0.0246	0.3152	0.2625	0.0294	0.4568573	Mineral
pH	nirk_Cluster46	0.0193	0.0064	0.1044	0.0355	0.2402	0.8192616	Mineral
pH	nirk_Cluster48	0.003	0.00E+00	0.00E+00	-0.0769	0.9999	0.9999179	Mineral
pH	nirk_Cluster49	0.0072	-0.0023	0.0512	-0.0218	0.4175	0.889307	Mineral
pH	nirk_Cluster5	0.2174	0.0091	0.0201	-0.0552	0.6138	0.97187	Mineral
pH	nirk_Cluster50	0.0014	0.0002	0.0083	-0.068	0.7463	0.9859838	Mineral
pH	nirk_Cluster52	0.0551	0.008	0.0814	0.0108	0.3026	0.855624	Mineral
pH	nirk_Cluster53	0.0039	-0.0017	0.0613	-0.0109	0.3736	0.8697242	Mineral
pH	nirk_Cluster54	0.0024	0.0004	0.0093	-0.0669	0.7322	0.9859838	Mineral
pH	nirk_Cluster55	0.0028	-0.0012	0.0411	-0.0327	0.4688	0.9244229	Mineral
pH	nirk_Cluster56	0.0009	-0.0003	0.0124	-0.0636	0.6932	0.9859838	Mineral
pH	nirk_Cluster57	0.0017	-0.0011	0.0712	-0.0002	0.3364	0.8697242	Mineral
pH	nirk_Cluster58	0.0102	0.006	0.2982	0.2443	0.0352	0.4731494	Mineral
pH	nirk_Cluster59	0.007	0.005	0.4464	0.4038	0.0065	0.2011387	Mineral
pH	nirk_Cluster6	0.1713	0.0504	0.6473	0.6202	0.0003	0.0351971	Mineral
pH	nirk_Cluster60	0.0006	0.0005	0.0968	0.0274	0.2589	0.8529427	Mineral
pH	nirk_Cluster62	0.1789	0.0346	0.0903	0.0204	0.2764	0.855624	Mineral
pH	nirk_Cluster63	0.1586	0.0231	0.1554	0.0904	0.146	0.6729478	Mineral
pH	nirk_Cluster64	0.0043	0.0011	0.0409	-0.0329	0.47	0.9244229	Mineral
pH	nirk_Cluster65	0.0531	0.0021	0.007	-0.0694	0.7665	0.9859838	Mineral
pH	nirk_Cluster66	0.0095	-0.0019	0.0559	-0.0167	0.3963	0.8738103	Mineral
pH	nirk_Cluster67	0.0232	0.0143	0.4494	0.407	0.0062	0.2011387	Mineral
pH	nirk_Cluster68	0.0158	0.0032	0.0667	-0.0051	0.3526	0.8697242	Mineral
pH	nirk_Cluster69	0.0362	0.005	0.0602	-0.0121	0.3782	0.8706018	Mineral
pH	nirk_Cluster7	0.096	0.02	0.2251	0.1655	0.074	0.5707711	Mineral
pH	nirk_Cluster70	0.0892	0.0207	0.0554	-0.0173	0.3984	0.8738103	Mineral
pH	nirk_Cluster71	0.0071	0.0011	0.0232	-0.052	0.5881	0.9704872	Mineral
pH	nirk_Cluster72	0.0278	0.0039	0.0612	-0.011	0.3741	0.8697242	Mineral
pH	nirk_Cluster73	0.1947	-0.0165	0.034	-0.0403	0.5107	0.9501544	Mineral
pH	nirk_Cluster74	0.8012	0.1243	0.0795	0.0087	0.3087	0.855624	Mineral
pH	nirk_Cluster75	0.0186	-0.0064	0.0882	0.0181	0.2823	0.855624	Mineral
pH	nirk_Cluster76	0.1377	0.0148	0.0852	0.0149	0.291	0.855624	Mineral
pH	nirk_Cluster77	0.0113	0.0116	0.3377	0.2867	0.0231	0.4335852	Mineral
pH	nirk_Cluster78	0.3126	0.0381	0.0715	1.00E-04	0.3353	0.8697242	Mineral
pH	nirk_Cluster79	0.0096	0.0081	0.361	0.3119	0.0178	0.3921627	Mineral
pH	nirk_Cluster8	0.1182	0.0017	0.0006	-0.0762	0.9294	0.9954346	Mineral
pH	nirk_Cluster80	0.3544	-0.0436	0.0575	-0.015	0.3895	0.8738103	Mineral

pH	nirk_Cluster82	0.0021	0.0005	0.0111	-0.0649	0.7084	0.9859838	Mineral
pH	nirk_Cluster83	0.0045	0.0019	0.1302	0.0633	0.1864	0.741891	Mineral
pH	nirk_Cluster84	0.0235	0.0029	0.0131	-0.0628	0.685	0.9859838	Mineral
pH	nirk_Cluster85	0.0053	0.0016	0.0712	-0.0003	0.3364	0.8697242	Mineral
pH	nirk_Cluster86	0.0352	0.0033	0.0164	-0.0592	0.6489	0.9834364	Mineral
pH	nirk_Cluster87	0.0138	-0.0022	0.0347	-0.0395	0.5061	0.9468854	Mineral
pH	nirk_Cluster88	0.0035	0.0004	0.0091	-0.0672	0.7358	0.9859838	Mineral
pH	nirk_Cluster89	0.0087	-0.0016	0.0281	-0.0467	0.5505	0.9621055	Mineral
pH	nirk_Cluster9	0.061	0.0025	0.0041	-0.0725	0.8197	0.9859838	Mineral
pH	nirk_Cluster90	0.0087	0.0019	0.0281	-0.0467	0.5506	0.9621055	Mineral
pH	nirk_Cluster91	0.044	0.0029	0.0126	-0.0634	0.6906	0.9859838	Mineral
pH	nirk_Cluster92	0.009	0.0018	0.0272	-0.0476	0.5568	0.9621055	Mineral
pH	nirk_Cluster93	0.0357	-0.0024	0.0089	-0.0673	0.7379	0.9859838	Mineral
pH	nirk_Cluster94	0.0222	0.0028	0.0271	-0.0477	0.5574	0.9621055	Mineral
pH	nirk_Cluster95	0.0566	0.0135	0.1072	0.0385	0.2336	0.8165349	Mineral
pH	nirk_Cluster96	0.0588	0.0126	0.1233	0.0559	0.1994	0.7638867	Mineral
pH	nirk_Cluster97	0.0329	0.002	0.0059	-0.0706	0.7852	0.9859838	Mineral
pH	nirk_Cluster98	0.0225	0.0104	0.1988	0.1372	0.0957	0.6064477	Mineral
pH	nirk_Cluster99	0.0074	0.0023	0.0688	-0.0028	0.3448	0.8697242	Mineral
pH	nirs_Cluster0	0.1586	0.048	0.5841	0.5521	0.0009	0.0607258	Mineral
pH	nirs_Cluster1	0.0051	0.0018	0.0496	-0.0235	0.4248	0.8906315	Mineral
pH	nirs_Cluster14	0.0142	0.0027	0.0108	-0.0653	0.713	0.9859838	Mineral
pH	nirs_Cluster2	0.0174	0.0143	0.299	0.2451	0.0349	0.4731494	Mineral
pH	nirs_Cluster26	0.0007	0.00E+00	0.0006	-0.0763	0.9297	0.9954346	Mineral
pH	nirs_Cluster28	0.0099	-1.00E-04	0.00E+00	-0.0769	0.9878	0.9954346	Mineral
pH	nirs_Cluster3	0.1914	0.0125	0.0614	-0.0108	0.3734	0.8697242	Mineral
pH	nirs_Cluster36	0.0018	0.0026	0.1918	0.1297	0.1025	0.6064477	Mineral
pH	nirs_Cluster37	0.0092	0.0043	0.0355	-0.0386	0.501	0.941425	Mineral
pH	nirs_Cluster4	0.0003	0.0002	0.0441	-0.0295	0.4527	0.9116712	Mineral
pH	nirs_Cluster50	0.0024	-0.0008	0.0286	-0.0461	0.5468	0.9621055	Mineral
pH	nirs_Cluster9	0.0376	-0.0351	0.2683	0.212	0.048	0.4852281	Mineral
pH	norb_Cluster0	0.0848	-0.0046	0.0055	-0.071	0.7931	0.9859838	Mineral
pH	norb_Cluster1	1.2746	0.1125	0.0113	-0.0648	0.7067	0.9859838	Mineral
pH	norb_Cluster101	0.0037	-0.0029	0.2373	0.1786	0.0655	0.5496702	Mineral
pH	norb_Cluster102	0.005	-0.001	0.0285	-0.0463	0.5478	0.9621055	Mineral
pH	norb_Cluster103	0.0025	-0.0003	0.0063	-0.0701	0.7786	0.9859838	Mineral
pH	norb_Cluster104	0.0025	0.0005	0.0087	-0.0675	0.7403	0.9859838	Mineral
pH	norb_Cluster106	0.1468	-0.0209	0.0561	-0.0165	0.3955	0.8738103	Mineral
pH	norb_Cluster107	0.0163	-0.0017	0.0109	-0.0652	0.7113	0.9859838	Mineral
pH	norb_Cluster108	0.0069	0.0018	0.0817	0.0111	0.3017	0.855624	Mineral
pH	norb_Cluster109	0.0282	-0.0046	0.0399	-0.0339	0.4753	0.9285205	Mineral
pH	norb_Cluster11	0.0156	-0.0085	0.2973	0.2433	0.0355	0.4731494	Mineral
pH	norb_Cluster110	0.0128	0.0004	0.0008	-0.0761	0.9212	0.9954346	Mineral
pH	norb_Cluster112	0.0215	-0.0035	0.0646	-0.0074	0.3607	0.8697242	Mineral
pH	norb_Cluster113	0.0157	-0.0037	0.062	-0.0102	0.371	0.8697242	Mineral
pH	norb_Cluster114	0.0035	0.0011	0.0204	-0.0549	0.6115	0.97187	Mineral
pH	norb_Cluster115	0.0034	0.00E+00	1.00E-04	-0.0769	0.9785	0.9954346	Mineral
pH	norb_Cluster116	0.0083	0.0018	0.0316	-0.0429	0.526	0.960721	Mineral
pH	norb_Cluster117	0.0034	0.00E+00	0.00E+00	-0.0769	0.9904	0.9954346	Mineral
pH	norb_Cluster118	0.0044	0.0002	0.001	-0.0759	0.9116	0.9954346	Mineral
pH	norb_Cluster119	0.0263	-0.0051	0.0388	-0.0351	0.4814	0.931241	Mineral
pH	norb_Cluster12	0.005	0.0012	0.0219	-0.0534	0.599	0.9707448	Mineral
pH	norb_Cluster121	0.0023	-0.0017	0.1199	0.0522	0.2061	0.7796537	Mineral
pH	norb_Cluster122	0.0184	-0.0021	0.0172	-0.0584	0.6414	0.9834364	Mineral
pH	norb_Cluster123	0.0071	-0.0002	0.0007	-0.0762	0.9249	0.9954346	Mineral

pH	norb_Cluster124	0.7452	0.0078	0.0002	-0.0767	0.9634	0.9954346	Mineral
pH	norb_Cluster125	0.0052	-0.0017	0.089	0.019	0.28	0.855624	Mineral
pH	norb_Cluster126	0.011	-0.0029	0.0762	0.0051	0.3195	0.855624	Mineral
pH	norb_Cluster127	0.2041	0.0019	0.0001	-0.0768	0.9681	0.9954346	Mineral
pH	norb_Cluster128	0.0123	0.0011	0.0093	-0.0669	0.7325	0.9859838	Mineral
pH	norb_Cluster13	0.0051	-0.0039	0.1924	0.1303	0.1019	0.6064477	Mineral
pH	norb_Cluster131	0.0209	-0.0088	0.239	0.1805	0.0644	0.5496702	Mineral
pH	norb_Cluster132	1.2771	0.0533	0.0063	-0.0701	0.7779	0.9859838	Mineral
pH	norb_Cluster133	0.0023	0.0016	0.1912	0.129	0.1031	0.6064477	Mineral
pH	norb_Cluster135	0.0703	0.0043	0.0072	-0.0692	0.7643	0.9859838	Mineral
pH	norb_Cluster136	0.025	-0.0001	0.00E+00	-0.0769	0.9849	0.9954346	Mineral
pH	norb_Cluster139	0.0391	0.0068	0.0527	-0.0201	0.4103	0.8803357	Mineral
pH	norb_Cluster14	0.0037	-0.0021	0.1874	0.1249	0.107	0.6064477	Mineral
pH	norb_Cluster140	0.0485	-0.0033	0.0102	-0.066	0.7209	0.9859838	Mineral
pH	norb_Cluster142	0.0018	-0.0013	0.1095	0.041	0.2284	0.8165349	Mineral
pH	norb_Cluster144	0.0122	0.0032	0.0934	0.0237	0.268	0.855624	Mineral
pH	norb_Cluster146	0.0418	0.0078	0.0834	0.0129	0.2966	0.855624	Mineral
pH	norb_Cluster15	0.0502	0.0013	0.0009	-0.076	0.9175	0.9954346	Mineral
pH	norb_Cluster150	0.0199	0.0052	0.1584	0.0937	0.1417	0.6653919	Mineral
pH	norb_Cluster155	0.1729	0.0057	0.002	-0.0748	0.8755	0.9954346	Mineral
pH	norb_Cluster16	0.0165	-0.0089	0.2195	0.1595	0.0781	0.5707711	Mineral
pH	norb_Cluster164	0.0242	-0.01	0.1758	0.1124	0.1198	0.6367254	Mineral
pH	norb_Cluster165	0.0092	-0.0014	0.0128	-0.0632	0.6883	0.9859838	Mineral
pH	norb_Cluster166	0.0046	-0.0014	0.0493	-0.0239	0.4265	0.8906315	Mineral
pH	norb_Cluster169	0.0309	-0.0103	0.1141	0.046	0.2182	0.7946499	Mineral
pH	norb_Cluster17	0.0035	-0.0018	0.1474	0.0818	0.1578	0.6951961	Mineral
pH	norb_Cluster170	0.0299	-0.0067	0.0422	-0.0315	0.4626	0.9221125	Mineral
pH	norb_Cluster171	0.0089	-0.0072	0.2947	0.2404	0.0365	0.4731494	Mineral
pH	norb_Cluster172	0.0179	-0.0055	0.0677	-0.004	0.349	0.8697242	Mineral
pH	norb_Cluster175	0.0595	-0.0177	0.0769	0.0059	0.3169	0.855624	Mineral
pH	norb_Cluster176	0.0392	-0.018	0.1908	0.1286	0.1035	0.6064477	Mineral
pH	norb_Cluster177	0.0361	-0.0072	0.0346	-0.0397	0.5071	0.9468854	Mineral
pH	norb_Cluster179	0.003	0.0019	0.1469	0.0813	0.1584	0.6951961	Mineral
pH	norb_Cluster18	0.0047	-0.0016	0.0889	0.0189	0.2803	0.855624	Mineral
pH	norb_Cluster180	0.0133	1.00E-04	0.00E+00	-0.0769	0.9816	0.9954346	Mineral
pH	norb_Cluster181	0.0003	1.00E-04	0.0044	-0.0722	0.814	0.9859838	Mineral
pH	norb_Cluster182	0.0018	-0.0009	0.08	0.0092	0.3071	0.855624	Mineral
pH	norb_Cluster184	0.0175	-0.0037	0.0453	-0.0282	0.4464	0.9116712	Mineral
pH	norb_Cluster186	0.0011	0.0004	0.0236	-0.0515	0.5847	0.9704872	Mineral
pH	norb_Cluster19	0.0058	-0.0008	0.0211	-0.0541	0.6051	0.9707448	Mineral
pH	norb_Cluster192	0.0221	-0.0078	0.115	0.047	0.2162	0.7946499	Mineral
pH	norb_Cluster193	0.0022	-0.0009	0.0686	-0.003	0.3457	0.8697242	Mineral
pH	norb_Cluster197	0.0398	-0.0024	0.0007	-0.0762	0.9246	0.9954346	Mineral
pH	norb_Cluster2	0.0032	-0.0022	0.1519	0.0866	0.151	0.6906553	Mineral
pH	norb_Cluster20	0.0665	0.0021	0.0017	-0.0751	0.8848	0.9954346	Mineral
pH	norb_Cluster208	0.0015	-0.0009	0.0786	0.0077	0.3116	0.855624	Mineral
pH	norb_Cluster21	0.0542	0.0061	0.019	-0.0565	0.6244	0.97187	Mineral
pH	norb_Cluster211	0.0141	0.0025	0.0083	-0.068	0.7469	0.9859838	Mineral
pH	norb_Cluster212	0.0252	-0.0068	0.0323	-0.0421	0.5216	0.955678	Mineral
pH	norb_Cluster214	0.0163	0.0014	0.0032	-0.0735	0.8421	0.9942937	Mineral
pH	norb_Cluster217	0.0186	-0.0035	0.0154	-0.0603	0.6592	0.9845819	Mineral
pH	norb_Cluster219	0.0108	0.0001	0.00E+00	-0.0769	0.9811	0.9954346	Mineral
pH	norb_Cluster22	0.0028	0.0016	0.0849	0.0145	0.2921	0.855624	Mineral
pH	norb_Cluster220	0.0248	1.00E-04	0.00E+00	-0.0769	0.9951	0.9984798	Mineral
pH	norb_Cluster221	0.0248	-0.0051	0.0115	-0.0645	0.7034	0.9859838	Mineral

pH	norb_Cluster222	0.0104	-0.0006	0.0006	-0.0763	0.9308	0.9954346	Mineral
pH	norb_Cluster224	0.0238	-0.0056	0.0215	-0.0538	0.6021	0.9707448	Mineral
pH	norb_Cluster225	0.0095	0.0008	0.0043	-0.0723	0.8166	0.9859838	Mineral
pH	norb_Cluster226	0.0049	-0.0019	0.0622	-0.01	0.3702	0.8697242	Mineral
pH	norb_Cluster228	0.0009	-0.0014	0.1411	0.075	0.1677	0.6951961	Mineral
pH	norb_Cluster229	0.004	-0.0012	0.047	-0.0264	0.4379	0.9033878	Mineral
pH	norb_Cluster230	0.0019	-0.0007	0.036	-0.0382	0.4983	0.9392782	Mineral
pH	norb_Cluster231	0.0061	0.003	0.092	0.0221	0.2719	0.855624	Mineral
pH	norb_Cluster232	0.0044	0.0027	0.1759	0.1125	0.1197	0.6367254	Mineral
pH	norb_Cluster234	0.017	-0.0105	0.2545	0.1972	0.0551	0.5163428	Mineral
pH	norb_Cluster235	0.0012	-0.0001	0.0047	-0.0718	0.8077	0.9859838	Mineral
pH	norb_Cluster238	0.0135	-0.0059	0.0865	0.0162	0.2874	0.855624	Mineral
pH	norb_Cluster239	0.0109	-0.0071	0.1506	0.0852	0.153	0.6941802	Mineral
pH	norb_Cluster24	0.3014	0.0105	0.0024	-0.0743	0.8613	0.9942937	Mineral
pH	norb_Cluster244	0.0015	0.00E+00	0.00E+00	-0.0769	0.9995	0.9999179	Mineral
pH	norb_Cluster247	0.006	0.0026	0.1963	0.1345	0.0981	0.6064477	Mineral
pH	norb_Cluster25	0.1263	0.0037	0.0012	-0.0756	0.9022	0.9954346	Mineral
pH	norb_Cluster250	0.0146	-0.0009	0.0042	-0.0725	0.8195	0.9859838	Mineral
pH	norb_Cluster251	0.0064	-0.0018	0.0498	-0.0233	0.424	0.8906315	Mineral
pH	norb_Cluster26	0.1134	0.0024	0.0007	-0.0762	0.9278	0.9954346	Mineral
pH	norb_Cluster28	0.0355	-0.0041	0.0193	-0.0562	0.6216	0.97187	Mineral
pH	norb_Cluster29	0.2454	-0.0322	0.0808	0.0101	0.3045	0.855624	Mineral
pH	norb_Cluster31	0.0632	0.0095	0.0377	-0.0363	0.4878	0.9324575	Mineral
pH	norb_Cluster32	0.1401	0.0144	0.0122	-0.0637	0.6947	0.9859838	Mineral
pH	norb_Cluster33	0.434	-0.0697	0.0691	-0.0025	0.3439	0.8697242	Mineral
pH	norb_Cluster35	0.0746	-0.002	0.0012	-0.0757	0.9036	0.9954346	Mineral
pH	norb_Cluster36	1.1002	-0.036	0.0016	-0.0752	0.8859	0.9954346	Mineral
pH	norb_Cluster37	0.7173	-0.0568	0.0145	-0.0613	0.6691	0.9859838	Mineral
pH	norb_Cluster38	0.0138	0.0016	0.0232	-0.0519	0.5875	0.9704872	Mineral
pH	norb_Cluster39	0.0465	-0.005	0.0188	-0.0567	0.626	0.97187	Mineral
pH	norb_Cluster40	0.2213	0.0125	0.0056	-0.0709	0.7905	0.9859838	Mineral
pH	norb_Cluster42	0.2825	-0.0148	0.0043	-0.0722	0.8156	0.9859838	Mineral
pH	norb_Cluster44	0.0249	-0.0015	0.0038	-0.0728	0.8271	0.9877788	Mineral
pH	norb_Cluster46	0.2575	0.04	0.0326	-0.0418	0.5193	0.9554875	Mineral
pH	norb_Cluster47	0.6453	0.0321	0.0048	-0.0718	0.8061	0.9859838	Mineral
pH	norb_Cluster5	0.0115	0.0016	0.0293	-0.0454	0.5419	0.9621055	Mineral
pH	norb_Cluster51	0.0122	-0.0066	0.1569	0.0921	0.1438	0.6681394	Mineral
pH	norb_Cluster52	0.2809	0.0255	0.0129	-0.0631	0.6874	0.9859838	Mineral
pH	norb_Cluster56	0.0178	0.0014	0.0088	-0.0674	0.7388	0.9859838	Mineral
pH	norb_Cluster57	0.004	0.0003	0.007	-0.0694	0.7675	0.9859838	Mineral
pH	norb_Cluster59	0.0322	0.0022	0.0035	-0.0731	0.8333	0.9921435	Mineral
pH	norb_Cluster6	0.6934	-0.0525	0.01	-0.0661	0.7224	0.9859838	Mineral
pH	norb_Cluster60	0.345	0.0565	0.0213	-0.054	0.604	0.9707448	Mineral
pH	norb_Cluster61	0.5486	-0.1776	0.1881	0.1257	0.1063	0.6064477	Mineral
pH	norb_Cluster62	0.003	-0.0015	0.1283	0.0613	0.1899	0.741891	Mineral
pH	norb_Cluster64	0.0407	0.0004	0.0002	-0.0767	0.9554	0.9954346	Mineral
pH	norb_Cluster66	0.0016	0.0009	0.0759	0.0048	0.3203	0.855624	Mineral
pH	norb_Cluster67	0.0279	0.0039	0.029	-0.0457	0.544	0.9621055	Mineral
pH	norb_Cluster68	0.003	-0.0015	0.1254	0.0581	0.1953	0.7581812	Mineral
pH	norb_Cluster69	0.0032	0.0013	0.082	0.0114	0.3008	0.855624	Mineral
pH	norb_Cluster7	0.0786	0.0043	0.0042	-0.0725	0.8195	0.9859838	Mineral
pH	norb_Cluster70	0.1262	0.0111	0.0196	-0.0558	0.6189	0.97187	Mineral
pH	norb_Cluster71	0.1512	-0.0147	0.0292	-0.0455	0.5429	0.9621055	Mineral
pH	norb_Cluster74	0.0387	-0.0012	0.0022	-0.0745	0.8667	0.9942937	Mineral
pH	norb_Cluster75	0.1531	-0.0084	0.0123	-0.0637	0.6942	0.9859838	Mineral

pH	norb_Cluster76	0.1215	0.0209	0.0181	-0.0575	0.6328	0.9773011	Mineral
pH	norb_Cluster77	0.0492	0.0052	0.0313	-0.0433	0.5285	0.9621055	Mineral
pH	norb_Cluster8	0.0206	1.00E-04	0.00E+00	-0.0769	0.987	0.9954346	Mineral
pH	norb_Cluster80	0.0021	-0.0015	0.1582	0.0934	0.1421	0.6653919	Mineral
pH	norb_Cluster81	0.0013	-0.0003	0.0127	-0.0632	0.6892	0.9859838	Mineral
pH	norb_Cluster83	0.011	-0.0084	0.2065	0.1454	0.0888	0.588963	Mineral
pH	norb_Cluster84	0.0093	0.0007	0.0039	-0.0727	0.8245	0.9866917	Mineral
pH	norb_Cluster85	0.0025	0.0002	0.0028	-0.0739	0.8514	0.9942937	Mineral
pH	norb_Cluster86	0.041	0.002	0.0009	-0.076	0.9167	0.9954346	Mineral
pH	norb_Cluster88	0.0019	-0.0003	0.0086	-0.0676	0.742	0.9859838	Mineral
pH	norb_Cluster90	0.0013	-0.0009	0.0514	-0.0215	0.4164	0.889307	Mineral
pH	norb_Cluster91	0.7571	-0.2673	0.1819	0.1189	0.1129	0.6227428	Mineral
pH	norb_Cluster92	0.0021	0.0008	0.0274	-0.0474	0.5555	0.9621055	Mineral
pH	norb_Cluster93	0.0028	0.0009	0.0231	-0.0521	0.5889	0.9704872	Mineral
pH	norb_Cluster94	0.002	0.0009	0.0781	0.0072	0.313	0.855624	Mineral
pH	norb_Cluster96	0.0006	-0.0002	0.0211	-0.0542	0.6055	0.9707448	Mineral
pH	norb_Cluster97	0.0162	0.007	0.1714	0.1077	0.125	0.6384894	Mineral
pH	norb_Cluster98	0.0176	0.0002	1.00E-04	-0.0768	0.9759	0.9954346	Mineral
pH	nosz_Cluster0	0.0057	0.0015	0.0608	-0.0114	0.3756	0.8697242	Mineral
pH	nosz_Cluster10	0.0128	-0.002	0.0233	-0.0519	0.5873	0.9704872	Mineral
pH	nosz_Cluster102	0.0057	0.0009	0.0441	-0.0294	0.4526	0.9116712	Mineral
pH	nosz_Cluster103	0.0496	-0.0184	0.2379	0.1792	0.0652	0.5496702	Mineral
pH	nosz_Cluster105	0.0016	0.0007	0.0574	-0.0151	0.3896	0.8738103	Mineral
pH	nosz_Cluster106	0.0088	-0.0054	0.2857	0.2308	0.0401	0.4731494	Mineral
pH	nosz_Cluster107	0.0003	0.0002	0.0441	-0.0295	0.4527	0.9116712	Mineral
pH	nosz_Cluster108	0.0043	-0.0013	0.0412	-0.0326	0.4683	0.9244229	Mineral
pH	nosz_Cluster109	0.0416	-0.0035	0.0265	-0.0484	0.5623	0.9621055	Mineral
pH	nosz_Cluster11	0.0104	0.0041	0.171	0.1072	0.1255	0.6384894	Mineral
pH	nosz_Cluster110	0.0004	-0.0008	0.3332	0.2819	0.0243	0.4335852	Mineral
pH	nosz_Cluster111	0.0078	-0.0032	0.1732	0.1096	0.1228	0.6384894	Mineral
pH	nosz_Cluster116	0.0074	-0.0001	0.0003	-0.0766	0.9534	0.9954346	Mineral
pH	nosz_Cluster118	0.0064	0.0007	0.0057	-0.0708	0.7899	0.9859838	Mineral
pH	nosz_Cluster119	0.0019	-0.0017	0.2505	0.1928	0.0574	0.5286529	Mineral
pH	nosz_Cluster120	0.0083	-0.0027	0.0955	0.0259	0.2624	0.8529427	Mineral
pH	nosz_Cluster123	0.0136	-0.0063	0.2007	0.1392	0.094	0.6064477	Mineral
pH	nosz_Cluster128	0.005	-0.0005	0.0045	-0.0721	0.8118	0.9859838	Mineral
pH	nosz_Cluster129	0.05	-0.0126	0.2491	0.1913	0.0582	0.5286529	Mineral
pH	nosz_Cluster13	0.0124	0.0065	0.2794	0.2239	0.0428	0.4852281	Mineral
pH	nosz_Cluster130	0.1138	0.0177	0.0061	-0.0703	0.7815	0.9859838	Mineral
pH	nosz_Cluster132	0.004	-0.0011	0.0657	-0.0062	0.3565	0.8697242	Mineral
pH	nosz_Cluster14	0.0149	0.0041	0.049	-0.0242	0.428	0.8906315	Mineral
pH	nosz_Cluster16	0.008	0.001	0.0074	-0.069	0.7608	0.9859838	Mineral
pH	nosz_Cluster17	0.0427	-0.006	0.0326	-0.0418	0.5197	0.9554875	Mineral
pH	nosz_Cluster18	0.0184	0.0029	0.0179	-0.0576	0.6344	0.9773011	Mineral
pH	nosz_Cluster20	0.0117	-0.0022	0.0197	-0.0557	0.6178	0.97187	Mineral
pH	nosz_Cluster21	0.0117	-0.0045	0.0952	0.0256	0.2631	0.8529427	Mineral
pH	nosz_Cluster22	0.0325	0.0068	0.0216	-0.0536	0.6008	0.9707448	Mineral
pH	nosz_Cluster23	0.019	-0.0062	0.0861	0.0158	0.2886	0.855624	Mineral
pH	nosz_Cluster24	0.0019	0.0019	0.2718	0.2158	0.0463	0.4852281	Mineral
pH	nosz_Cluster25	0.0026	0.0016	0.1407	0.0746	0.1683	0.6951961	Mineral
pH	nosz_Cluster26	0.0068	0.0004	0.0014	-0.0754	0.8947	0.9954346	Mineral
pH	nosz_Cluster27	0.007	0.0004	0.0028	-0.0739	0.8522	0.9942937	Mineral
pH	nosz_Cluster28	0.0358	-0.0004	0.0002	-0.0767	0.9634	0.9954346	Mineral
pH	nosz_Cluster30	0.0341	0.0029	0.01	-0.0661	0.7223	0.9859838	Mineral
pH	nosz_Cluster32	0.016	0.0035	0.0546	-0.0181	0.4018	0.8760291	Mineral

pH	nosz_Cluster33	0.0187	0.001	0.0022	-0.0745	0.8679	0.9942937	Mineral
pH	nosz_Cluster34	0.0387	-0.0086	0.0294	-0.0453	0.5414	0.9621055	Mineral
pH	nosz_Cluster35	0.0097	0.0013	0.0157	-0.0601	0.6567	0.9834364	Mineral
pH	nosz_Cluster36	0.0213	0.0013	0.0039	-0.0727	0.824	0.9866917	Mineral
pH	nosz_Cluster37	0.0077	0.0026	0.0841	0.0136	0.2944	0.855624	Mineral
pH	nosz_Cluster38	0.0144	-0.003	0.0231	-0.052	0.5885	0.9704872	Mineral
pH	nosz_Cluster39	0.0653	0.0013	0.0006	-0.0763	0.9309	0.9954346	Mineral
pH	nosz_Cluster40	0.0262	0.0055	0.0409	-0.0329	0.4697	0.9244229	Mineral
pH	nosz_Cluster41	0.0258	0.0052	0.0376	-0.0364	0.4884	0.9324575	Mineral
pH	nosz_Cluster42	0.03	-0.0001	0.00E+00	-0.0769	0.9895	0.9954346	Mineral
pH	nosz_Cluster43	0.001	-0.001	0.1236	0.0562	0.1988	0.7638867	Mineral
pH	nosz_Cluster44	0.0068	0.0002	0.0005	-0.0764	0.9371	0.9954346	Mineral
pH	nosz_Cluster45	0.0056	0.0006	0.0068	-0.0696	0.7695	0.9859838	Mineral
pH	nosz_Cluster46	0.0082	0.0019	0.0681	-0.0036	0.3476	0.8697242	Mineral
pH	nosz_Cluster47	0.0012	-0.0001	0.0027	-0.074	0.8538	0.9942937	Mineral
pH	nosz_Cluster48	0.0028	-0.001	0.039	-0.035	0.4807	0.931241	Mineral
pH	nosz_Cluster49	0.0012	0.0006	0.0501	-0.023	0.4228	0.8906315	Mineral
pH	nosz_Cluster5	0.004	0.00E+00	0.00E+00	-0.0769	0.9901	0.9954346	Mineral
pH	nosz_Cluster51	0.003	0.00E+00	1.00E-04	-0.0768	0.9727	0.9954346	Mineral
pH	nosz_Cluster53	0.0012	0.0012	0.1297	0.0627	0.1874	0.741891	Mineral
pH	nosz_Cluster54	0.0037	-0.0027	0.2137	0.1532	0.0828	0.5787959	Mineral
pH	nosz_Cluster57	0.2232	0.0467	0.0607	-0.0115	0.3759	0.8697242	Mineral
pH	nosz_Cluster58	0.0053	-0.0033	0.1667	0.1027	0.1308	0.655222	Mineral
pH	nosz_Cluster59	0.0008	-0.0008	0.0623	-0.0098	0.3697	0.8697242	Mineral
pH	nosz_Cluster6	0.0034	0.0012	0.043	-0.0306	0.4582	0.9163191	Mineral
pH	nosz_Cluster60	0.0099	0.0007	0.0032	-0.0735	0.8414	0.9942937	Mineral
pH	nosz_Cluster61	0.0178	-0.0022	0.0365	-0.0376	0.4952	0.936397	Mineral
pH	nosz_Cluster62	0.0493	0.009	0.0382	-0.0357	0.4849	0.9324575	Mineral
pH	nosz_Cluster64	0.0023	0.001	0.0288	-0.046	0.5457	0.9621055	Mineral
pH	nosz_Cluster65	0.0083	-0.0005	0.0032	-0.0735	0.8416	0.9942937	Mineral
pH	nosz_Cluster66	0.0428	0.0094	0.0452	-0.0282	0.4467	0.9116712	Mineral
pH	nosz_Cluster67	0.0669	0.0175	0.0646	-0.0074	0.3607	0.8697242	Mineral
pH	nosz_Cluster69	0.008	-0.0022	0.0686	-0.003	0.3456	0.8697242	Mineral
pH	nosz_Cluster70	0.0655	0.005	0.0023	-0.0745	0.8662	0.9942937	Mineral
pH	nosz_Cluster71	0.0101	0.0012	0.0065	-0.0699	0.775	0.9859838	Mineral
pH	nosz_Cluster72	0.066	0.0116	0.0369	-0.0372	0.4929	0.936397	Mineral
pH	nosz_Cluster73	0.0031	-0.0005	0.0105	-0.0656	0.7166	0.9859838	Mineral
pH	nosz_Cluster74	0.0017	-0.0019	0.3346	0.2834	0.0239	0.4335852	Mineral
pH	nosz_Cluster75	0.132	0.035	0.0146	-0.0612	0.6678	0.9859838	Mineral
pH	nosz_Cluster77	0.0002	1.00E-04	0.0077	-0.0686	0.7558	0.9859838	Mineral
pH	nosz_Cluster78	0.0054	-0.0017	0.0785	0.0076	0.3117	0.855624	Mineral
pH	nosz_Cluster79	0.0401	0.004	0.0157	-0.06	0.656	0.9834364	Mineral
pH	nosz_Cluster8	0.0106	0.0005	0.0035	-0.0732	0.8341	0.9921435	Mineral
pH	nosz_Cluster80	0.0039	-0.0014	0.0727	0.0014	0.331	0.8697242	Mineral
pH	nosz_Cluster81	0.0099	0.0027	0.0261	-0.0488	0.5653	0.9639666	Mineral
pH	nosz_Cluster82	0.0023	0.0033	0.2267	0.1672	0.0728	0.5707711	Mineral
pH	nosz_Cluster83	0.0014	-0.002	0.2362	0.1775	0.0662	0.5496702	Mineral
pH	nosz_Cluster85	0.0027	0.0025	0.2224	0.1626	0.0759	0.5707711	Mineral
pH	nosz_Cluster86	0.0038	0.0021	0.142	0.076	0.1662	0.6951961	Mineral
pH	nosz_Cluster87	0.0034	-1.00E-04	0.0002	-0.0767	0.9577	0.9954346	Mineral
pH	nosz_Cluster88	0.0246	-0.0048	0.0959	0.0263	0.2614	0.8529427	Mineral
pH	nosz_Cluster89	0.0607	0.0085	0.0233	-0.0518	0.5868	0.9704872	Mineral
pH	nosz_Cluster9	0.0034	-0.0006	0.0097	-0.0665	0.7272	0.9859838	Mineral
pH	nosz_Cluster90	0.0139	0.0002	0.0004	-0.0765	0.9448	0.9954346	Mineral
pH	nosz_Cluster91	0.0232	-0.0017	0.0041	-0.0725	0.82	0.9859838	Mineral

pH	nosz_Cluster93	0.0488	-0.0171	0.1644	0.1001	0.1338	0.655222	Mineral
pH	nosz_Cluster95	0.0094	-0.0014	0.0272	-0.0477	0.5571	0.9621055	Mineral
pH	nosz_Cluster96	0.0064	-0.0021	0.1442	0.0784	0.1627	0.6951961	Mineral
pH	nosz_Cluster97	0.0026	0.00E+00	1.00E-04	-0.0768	0.9746	0.9954346	Mineral
pH	nosz_Cluster98	0.0035	0.0046	0.2047	0.1435	0.0904	0.5926154	Mineral
pH	nosz_Cluster99	0.0061	-0.0006	0.0099	-0.0662	0.7241	0.9859838	Mineral
pH	nrfa_Cluster10	0.0978	0.0018	0.0003	-0.0766	0.9523	0.9954346	Mineral
pH	nrfa_Cluster100	0.1249	-0.0823	0.2682	0.2119	0.048	0.4852281	Mineral
pH	nrfa_Cluster104	0.1478	-0.0241	0.0446	-0.0289	0.45	0.9116712	Mineral
pH	nrfa_Cluster105	0.006	-0.0006	0.0045	-0.072	0.8114	0.9859838	Mineral
pH	nrfa_Cluster109	0.2753	-0.0102	0.0026	-0.0742	0.8579	0.9942937	Mineral
pH	nrfa_Cluster116	0.3208	-0.0158	0.0084	-0.0679	0.7452	0.9859838	Mineral
pH	nrfa_Cluster118	0.2298	-0.1664	0.2761	0.2205	0.0442	0.4852281	Mineral
pH	nrfa_Cluster125	0.1081	-0.0842	0.2849	0.2299	0.0404	0.4731494	Mineral
pH	nrfa_Cluster154	0.0837	-0.0497	0.2322	0.1731	0.069	0.5512046	Mineral
pH	nrfa_Cluster164	0.0044	-0.0002	0.001	-0.0758	0.9102	0.9954346	Mineral
pH	nrfa_Cluster172	0.0036	-0.0005	0.0071	-0.0693	0.7651	0.9859838	Mineral
pH	nrfa_Cluster175	0.0053	-0.0056	0.2904	0.2358	0.0382	0.4731494	Mineral
pH	nrfa_Cluster20	0.045	-0.0073	0.0222	-0.053	0.5959	0.9707448	Mineral
pH	nrfa_Cluster26	0.1433	-0.0964	0.2671	0.2107	0.0485	0.4852281	Mineral
pH	nrfa_Cluster29	0.0059	0.0004	0.0018	-0.0749	0.879	0.9954346	Mineral
pH	nrfa_Cluster34	0.0642	-0.0158	0.0535	-0.0193	0.407	0.8762816	Mineral
pH	nrfa_Cluster38	0.0033	-0.0034	0.2853	0.2304	0.0403	0.4731494	Mineral
pH	nrfa_Cluster61	0.0047	-0.0017	0.0568	-0.0158	0.3925	0.8738103	Mineral
pH	nrfa_Cluster62	0.0589	-0.0453	0.2691	0.2129	0.0476	0.4852281	Mineral
pH	nrfa_Cluster66	0.1282	-0.0188	0.0495	-0.0236	0.4256	0.8906315	Mineral
pH	nrfa_Cluster69	0.0035	1.00E-04	0.0003	-0.0766	0.9548	0.9954346	Mineral
pH	nrfa_Cluster71	0.0322	-0.0186	0.1856	0.123	0.1089	0.6064477	Mineral
pH	nrfa_Cluster72	0.0969	-0.046	0.1792	0.1161	0.1158	0.6270888	Mineral
pH	nrfa_Cluster73	0.0042	-0.0025	0.1125	0.0442	0.2217	0.8024643	Mineral
pH	nrfa_Cluster74	0.1692	-0.1027	0.2841	0.229	0.0408	0.4731494	Mineral
pH	nrfa_Cluster76	0.0922	-0.0574	0.2373	0.1786	0.0655	0.5496702	Mineral
pH	nrfa_Cluster79	0.0138	-0.002	0.0043	-0.0723	0.8171	0.9859838	Mineral
pH	nrfa_Cluster87	0.0207	0.0012	0.0012	-0.0756	0.9029	0.9954346	Mineral
pH	nrfa_Cluster89	0.0023	-0.0012	0.0766	0.0056	0.3179	0.855624	Mineral
pH	nrfa_Cluster92	0.0041	-0.0016	0.1003	0.0311	0.25	0.8381873	Mineral
pH	nrfa_Cluster93	0.0581	0.008	0.0078	-0.0685	0.754	0.9859838	Mineral
pH	nrfa_Cluster94	0.0003	-0.0005	0.1411	0.075	0.1677	0.6951961	Mineral
pH	nrfa_Cluster97	0.0225	-0.0081	0.1452	0.0794	0.1611	0.6951961	Mineral
pH	nrfa_Cluster99	0.0184	-0.0105	0.2838	0.2287	0.0409	0.4731494	Mineral
C	amoA_A_Cluster26	0.0053	-0.0025	0.0818	0.0111	0.3015	0.6440917	Mineral
C	amoA_A_Cluster45	0.0071	-0.0031	0.1059	0.0371	0.2367	0.6440917	Mineral
C	amoA_B_Cluster0	0.1272	-0.0233	0.2798	0.2244	0.0426	0.6440917	Mineral
C	amoA_B_Cluster1	0.0205	-0.0034	0.0463	-0.0271	0.4412	0.6657796	Mineral
C	amoA_B_Cluster10	0.0539	-0.014	0.2917	0.2373	0.0377	0.6440917	Mineral
C	amoA_B_Cluster11	0.0486	-0.0094	0.0971	0.0276	0.2582	0.6440917	Mineral
C	amoA_B_Cluster12	0.093	-0.0218	0.3318	0.2804	0.0246	0.6440917	Mineral
C	amoA_B_Cluster13	0.6417	-0.1466	0.1615	0.097	0.1376	0.6440917	Mineral
C	amoA_B_Cluster14	0.0339	-0.0082	0.0854	0.015	0.2907	0.6440917	Mineral
C	amoA_B_Cluster15	0.0413	-0.0076	0.0852	0.0148	0.2911	0.6440917	Mineral
C	amoA_B_Cluster2	0.2143	-0.0531	0.2013	0.1399	0.0934	0.6440917	Mineral
C	amoA_B_Cluster20	0.0078	-0.0017	0.0279	-0.0469	0.5517	0.7132088	Mineral
C	amoA_B_Cluster23	0.0626	-0.0118	0.1359	0.0694	0.1764	0.6440917	Mineral
C	amoA_B_Cluster3	0.0244	-0.0037	0.0335	-0.0408	0.5135	0.6885539	Mineral
C	amoA_B_Cluster4	0.0169	-0.004	0.0694	-0.0022	0.3427	0.6440917	Mineral

C	amoA_B_Cluster5	0.1032	-0.0244	0.384	0.3366	0.0137	0.4147775	Mineral
C	amoA_B_Cluster6	0.0279	-0.004	0.0556	-0.017	0.3975	0.6525012	Mineral
C	amoA_B_Cluster7	0.0232	-0.0044	0.045	-0.0285	0.4478	0.6722946	Mineral
C	amoA_B_Cluster8	0.0268	-0.0034	0.0189	-0.0566	0.6255	0.7578089	Mineral
C	amoA_B_Cluster9	0.0693	-0.0124	0.0581	-0.0144	0.3868	0.6448159	Mineral
C	nifh_Cluster0	0.0318	0.0036	0.0442	-0.0293	0.4518	0.672688	Mineral
C	nifh_Cluster10	0.0033	0.0004	0.006	-0.0704	0.7829	0.8601834	Mineral
C	nifh_Cluster100	0.002	-0.0007	0.0414	-0.0323	0.4669	0.6736646	Mineral
C	nifh_Cluster103	0.0433	-0.0086	0.1089	0.0404	0.2296	0.6440917	Mineral
C	nifh_Cluster1034	0.0337	-0.0104	0.1002	0.031	0.2504	0.6440917	Mineral
C	nifh_Cluster105	0.0164	-0.0043	0.0614	-0.0107	0.373	0.6440917	Mineral
C	nifh_Cluster108	0.0076	-0.001	0.0164	-0.0592	0.649	0.7756324	Mineral
C	nifh_Cluster1099	0.0478	0.0316	0.2536	0.1962	0.0556	0.6440917	Mineral
C	nifh_Cluster11	0.0025	-0.0011	0.1035	0.0346	0.2422	0.6440917	Mineral
C	nifh_Cluster1112	0.0037	-0.0008	0.0232	-0.0519	0.5879	0.7395521	Mineral
C	nifh_Cluster112	0.0098	0.0095	0.5285	0.4923	0.0021	0.1399905	Mineral
C	nifh_Cluster113	0.0374	-0.0058	0.0811	0.0104	0.3036	0.6440917	Mineral
C	nifh_Cluster114	0.0304	0.0001	1.00E-04	-0.0768	0.9749	0.9849088	Mineral
C	nifh_Cluster1141	0.0296	-0.0073	0.0882	0.018	0.2825	0.6440917	Mineral
C	nifh_Cluster115	0.0098	-0.0002	0.0004	-0.0765	0.9434	0.966933	Mineral
C	nifh_Cluster116	0.0883	-0.0113	0.0581	-0.0144	0.3869	0.6448159	Mineral
C	nifh_Cluster1163	0.0051	-0.0012	0.0522	-0.0208	0.413	0.653328	Mineral
C	nifh_Cluster1179	0.0037	-0.0013	0.0434	-0.0302	0.4563	0.6736646	Mineral
C	nifh_Cluster1182	0.0209	0.0014	0.0181	-0.0574	0.6324	0.7645867	Mineral
C	nifh_Cluster1183	0.0045	0.0023	0.2872	0.2324	0.0395	0.6440917	Mineral
C	nifh_Cluster1197	0.0115	-0.0032	0.0873	0.0171	0.2849	0.6440917	Mineral
C	nifh_Cluster1199	0.0048	0.0011	0.031	-0.0435	0.53	0.7010972	Mineral
C	nifh_Cluster120	0.02	-0.0085	0.0697	-0.0019	0.3417	0.6440917	Mineral
C	nifh_Cluster1203	0.0217	-0.0048	0.0549	-0.0178	0.4007	0.6530519	Mineral
C	nifh_Cluster1206	0.006	-0.0009	0.0258	-0.0491	0.5674	0.7238713	Mineral
C	nifh_Cluster1207	0.0078	-0.0025	0.0682	-0.0035	0.3471	0.6440917	Mineral
C	nifh_Cluster1209	0.1617	0.0029	0.0023	-0.0745	0.8661	0.9076297	Mineral
C	nifh_Cluster121	0.0123	-0.0059	0.0898	0.0198	0.2778	0.6440917	Mineral
C	nifh_Cluster1211	0.0068	-0.001	0.0202	-0.0552	0.6133	0.7578017	Mineral
C	nifh_Cluster1213	0.0032	-0.001	0.0754	0.0043	0.3219	0.6440917	Mineral
C	nifh_Cluster122	0.0041	-0.0019	0.1139	0.0458	0.2185	0.6440917	Mineral
C	nifh_Cluster123	0.0092	-0.0041	0.0935	0.0238	0.2677	0.6440917	Mineral
C	nifh_Cluster1236	0.031	-0.007	0.0289	-0.0458	0.545	0.7097936	Mineral
C	nifh_Cluster1238	0.0034	-0.0004	0.0071	-0.0693	0.7655	0.8473744	Mineral
C	nifh_Cluster124	0.0101	-0.0015	0.0357	-0.0385	0.5003	0.6838422	Mineral
C	nifh_Cluster1261	0.0023	-0.0008	0.035	-0.0393	0.5046	0.6852504	Mineral
C	nifh_Cluster1262	0.008	0.0002	0.0004	-0.0765	0.9437	0.966933	Mineral
C	nifh_Cluster1266	0.0072	-0.0005	0.0039	-0.0727	0.8255	0.880713	Mineral
C	nifh_Cluster1267	0.0388	0.0433	0.57	0.5369	0.0011	0.0960099	Mineral
C	nifh_Cluster127	0.0033	-0.0014	0.0532	-0.0197	0.4084	0.653328	Mineral
C	nifh_Cluster1278	0.2105	-0.0495	0.1705	0.1067	0.1261	0.6440917	Mineral
C	nifh_Cluster128	0.0043	-0.0018	0.1189	0.0511	0.2081	0.6440917	Mineral
C	nifh_Cluster1289	0.004	0.0006	0.0157	-0.06	0.6562	0.7789551	Mineral
C	nifh_Cluster129	0.0086	-0.0035	0.0819	0.0113	0.301	0.6440917	Mineral
C	nifh_Cluster1292	0.0106	-0.0024	0.0466	-0.0267	0.4397	0.6651262	Mineral
C	nifh_Cluster130	0.0071	-0.0034	0.0663	-0.0056	0.3543	0.6440917	Mineral
C	nifh_Cluster1305	0.0048	0.00E+00	0.00E+00	-0.0769	0.9973	0.9972741	Mineral
C	nifh_Cluster1306	0.0055	0.00E+00	1.00E-04	-0.0769	0.9796	0.986571	Mineral
C	nifh_Cluster131	0.0067	-0.003	0.0882	0.0181	0.2824	0.6440917	Mineral
C	nifh_Cluster1319	0.0048	-0.0012	0.0365	-0.0376	0.4951	0.6838422	Mineral

C	nifh_Cluster132	0.0064	-0.0027	0.0911	0.0212	0.2742	0.6440917	Mineral
C	nifh_Cluster1320	0.0028	-0.0006	0.0134	-0.0625	0.6816	0.7931895	Mineral
C	nifh_Cluster1323	0.0022	-0.0003	0.011	-0.065	0.7095	0.811287	Mineral
C	nifh_Cluster1324	0.0235	0.0011	0.002	-0.0747	0.8737	0.9123538	Mineral
C	nifh_Cluster1336	0.0159	-0.0004	0.0017	-0.0751	0.8829	0.9186642	Mineral
C	nifh_Cluster1340	0.0478	0.0003	0.00E+00	-0.0769	0.986	0.9902866	Mineral
C	nifh_Cluster1345	0.033	0.0283	0.3911	0.3443	0.0127	0.4147482	Mineral
C	nifh_Cluster1370	0.0022	-0.0002	0.0026	-0.0741	0.857	0.9013295	Mineral
C	nifh_Cluster1375	0.0017	0.0009	0.1325	0.0658	0.1822	0.6440917	Mineral
C	nifh_Cluster139	0.0038	-0.0007	0.0122	-0.0638	0.6954	0.7981854	Mineral
C	nifh_Cluster140	0.0051	-0.001	0.0293	-0.0454	0.542	0.7097936	Mineral
C	nifh_Cluster141	0.0808	-0.0144	0.2825	0.2273	0.0414	0.6440917	Mineral
C	nifh_Cluster148	0.006	-0.0008	0.0079	-0.0684	0.7532	0.8416037	Mineral
C	nifh_Cluster1480	0.0006	0.0013	0.667	0.6414	0.0002	0.0369628	Mineral
C	nifh_Cluster152	0.0602	-0.0055	0.0196	-0.0559	0.6191	0.7578089	Mineral
C	nifh_Cluster156	0.0459	0.025	0.6182	0.5888	0.0005	0.0500459	Mineral
C	nifh_Cluster1562	0.0293	0.043	0.7906	0.7745	0.00E+00	0.0027319	Mineral
C	nifh_Cluster158	0.019	-0.0044	0.0586	-0.0138	0.3848	0.6448159	Mineral
C	nifh_Cluster16	0.0023	-0.0011	0.0587	-0.0137	0.3842	0.6448159	Mineral
C	nifh_Cluster166	0.0165	-0.0073	0.0794	0.0086	0.3088	0.6440917	Mineral
C	nifh_Cluster205	0.0121	-0.0024	0.0723	0.0009	0.3325	0.6440917	Mineral
C	nifh_Cluster21	0.0022	-0.0007	0.0426	-0.031	0.4603	0.6736646	Mineral
C	nifh_Cluster222	0.0069	-0.0031	0.0678	-0.0039	0.3486	0.6440917	Mineral
C	nifh_Cluster225	0.021	0.0003	0.0004	-0.0765	0.944	0.966933	Mineral
C	nifh_Cluster230	0.0052	-0.0004	0.0042	-0.0724	0.8186	0.8781415	Mineral
C	nifh_Cluster231	0.0019	-0.0005	0.0228	-0.0524	0.5911	0.7396067	Mineral
C	nifh_Cluster236	0.0034	-0.0012	0.0828	0.0122	0.2984	0.6440917	Mineral
C	nifh_Cluster237	0.007	-0.0013	0.0351	-0.0391	0.5035	0.6852504	Mineral
C	nifh_Cluster243	0.0253	0.0057	0.0344	-0.0399	0.5084	0.6863317	Mineral
C	nifh_Cluster2432	0.0317	0.03	0.505	0.4669	0.003	0.17614	Mineral
C	nifh_Cluster2433	0.0028	-0.0002	0.0008	-0.076	0.918	0.9485136	Mineral
C	nifh_Cluster246	0.0068	-0.0026	0.0767	0.0057	0.3176	0.6440917	Mineral
C	nifh_Cluster25	0.0195	-0.0011	0.0076	-0.0687	0.7571	0.8421773	Mineral
C	nifh_Cluster256	0.0023	-0.0009	0.0685	-0.0031	0.346	0.6440917	Mineral
C	nifh_Cluster264	0.0025	-0.0009	0.085	0.0146	0.2917	0.6440917	Mineral
C	nifh_Cluster265	0.0702	0.0099	0.1228	0.0553	0.2003	0.6440917	Mineral
C	nifh_Cluster267	0.0071	0.0005	0.0044	-0.0722	0.8152	0.8777196	Mineral
C	nifh_Cluster268	0.0109	0.0025	0.1247	0.0574	0.1967	0.6440917	Mineral
C	nifh_Cluster269	0.0283	-0.0034	0.0134	-0.0625	0.681	0.7931895	Mineral
C	nifh_Cluster272	0.0012	0.0016	0.4009	0.3549	0.0113	0.3914386	Mineral
C	nifh_Cluster274	0.0014	-0.0007	0.0263	-0.0486	0.5638	0.7231387	Mineral
C	nifh_Cluster275	0.0051	-1.00E-04	0.0002	-0.0767	0.9618	0.9733915	Mineral
C	nifh_Cluster278	0.0056	0.0036	0.2635	0.2069	0.0503	0.6440917	Mineral
C	nifh_Cluster279	0.0058	0.0009	0.0189	-0.0566	0.6251	0.7578089	Mineral
C	nifh_Cluster28	0.0017	-0.0006	0.0361	-0.038	0.4976	0.6838422	Mineral
C	nifh_Cluster280	0.0034	0.0004	0.0033	-0.0734	0.839	0.8871144	Mineral
C	nifh_Cluster282	0.0052	-0.0014	0.0706	-0.0009	0.3385	0.6440917	Mineral
C	nifh_Cluster287	0.003	0.0011	0.079	0.0082	0.3102	0.6440917	Mineral
C	nifh_Cluster290	0.0096	-0.0012	0.0103	-0.0658	0.7184	0.8151138	Mineral
C	nifh_Cluster297	0.004	-0.0019	0.0553	-0.0173	0.3988	0.6525012	Mineral
C	nifh_Cluster299	0.0025	0.0004	0.0104	-0.0657	0.7176	0.8151138	Mineral
C	nifh_Cluster303	0.002	0.001	0.1212	0.0536	0.2036	0.6440917	Mineral
C	nifh_Cluster304	0.0025	-0.0012	0.0896	0.0195	0.2785	0.6440917	Mineral
C	nifh_Cluster313	0.0129	-0.0057	0.0801	0.0094	0.3067	0.6440917	Mineral
C	nifh_Cluster314	0.0027	-0.0006	0.0201	-0.0552	0.6139	0.7578017	Mineral

C	nifh_Cluster317	0.0089	-0.0024	0.0707	-0.0008	0.3382	0.6440917	Mineral
C	nifh_Cluster32	0.0029	-0.0002	0.0039	-0.0727	0.825	0.880713	Mineral
C	nifh_Cluster320	13.944	-1.7871	0.456	0.4142	0.0057	0.2820262	Mineral
C	nifh_Cluster334	0.0084	-0.0033	0.0567	-0.0159	0.3929	0.6511707	Mineral
C	nifh_Cluster335	0.0059	-0.0025	0.064	-0.008	0.363	0.6440917	Mineral
C	nifh_Cluster338	0.0151	-0.0016	0.0171	-0.0585	0.6426	0.7737004	Mineral
C	nifh_Cluster340	0.0041	-0.0011	0.0593	-0.013	0.3817	0.6448159	Mineral
C	nifh_Cluster341	0.0021	0.0016	0.1758	0.1124	0.1198	0.6440917	Mineral
C	nifh_Cluster35	0.0079	0.0002	0.0007	-0.0762	0.9259	0.9550092	Mineral
C	nifh_Cluster351	0.0035	-0.0014	0.0629	-0.0091	0.3671	0.6440917	Mineral
C	nifh_Cluster3574	0.1191	0.0011	0.0003	-0.0766	0.9505	0.968545	Mineral
C	nifh_Cluster3691	0.0224	-0.0028	0.0127	-0.0632	0.6888	0.7955733	Mineral
C	nifh_Cluster37	0.0374	-0.0093	0.0511	-0.0219	0.418	0.653328	Mineral
C	nifh_Cluster374	0.0029	0.00E+00	1.00E-04	-0.0769	0.9799	0.986571	Mineral
C	nifh_Cluster38	0.0008	0.0001	0.0037	-0.0729	0.8287	0.8825483	Mineral
C	nifh_Cluster382	0.0023	-0.0011	0.0474	-0.0259	0.4356	0.6625813	Mineral
C	nifh_Cluster384	0.0023	-0.0011	0.0901	0.0201	0.2771	0.6440917	Mineral
C	nifh_Cluster386	0.0041	-0.0016	0.0524	-0.0205	0.4118	0.653328	Mineral
C	nifh_Cluster389	0.003	-0.0013	0.0415	-0.0323	0.4667	0.6736646	Mineral
C	nifh_Cluster394	0.0027	-0.0012	0.0445	-0.029	0.4502	0.6725787	Mineral
C	nifh_Cluster40	0.0014	-0.0005	0.0494	-0.0237	0.4258	0.6559	Mineral
C	nifh_Cluster43	0.0107	0.0003	0.0012	-0.0756	0.9008	0.9340935	Mineral
C	nifh_Cluster434	0.0048	-0.0011	0.0258	-0.0491	0.5675	0.7238713	Mineral
C	nifh_Cluster440	0.0171	-0.0034	0.0336	-0.0407	0.5129	0.6885539	Mineral
C	nifh_Cluster462	0.0048	-0.0015	0.051	-0.022	0.4184	0.653328	Mineral
C	nifh_Cluster470	0.017	0.0012	0.0067	-0.0697	0.7721	0.8530768	Mineral
C	nifh_Cluster49	0.0223	-0.0104	0.0939	0.0242	0.2666	0.6440917	Mineral
C	nifh_Cluster499	0.0028	-0.0013	0.0843	0.0138	0.294	0.6440917	Mineral
C	nifh_Cluster5	0.0263	0.0064	0.2074	0.1464	0.088	0.6440917	Mineral
C	nifh_Cluster506	0.0058	0.0017	0.0692	-0.0024	0.3434	0.6440917	Mineral
C	nifh_Cluster51	0.0031	-0.0014	0.054	-0.0188	0.4046	0.653328	Mineral
C	nifh_Cluster52	0.0396	-0.0132	0.0737	0.0025	0.3276	0.6440917	Mineral
C	nifh_Cluster525	0.0025	-0.0009	0.0307	-0.0439	0.5322	0.7024195	Mineral
C	nifh_Cluster531	0.0129	0.0125	0.6316	0.6032	0.0004	0.0472096	Mineral
C	nifh_Cluster539	0.0056	-0.0024	0.0364	-0.0377	0.4958	0.6838422	Mineral
C	nifh_Cluster578	0.0593	-0.0016	0.0018	-0.0749	0.8791	0.9163363	Mineral
C	nifh_Cluster58	0.2389	0.2494	0.6563	0.6299	0.0003	0.0369628	Mineral
C	nifh_Cluster60	0.011	-0.0036	0.08	0.0092	0.3071	0.6440917	Mineral
C	nifh_Cluster61	0.0017	0.0007	0.0743	0.0031	0.3257	0.6440917	Mineral
C	nifh_Cluster65	0.0085	-0.0029	0.0823	0.0117	0.3	0.6440917	Mineral
C	nifh_Cluster686	0.0013	1.00E-04	0.0009	-0.076	0.9171	0.9485136	Mineral
C	nifh_Cluster69	0.0092	-0.0045	0.1066	0.0379	0.2349	0.6440917	Mineral
C	nifh_Cluster70	0.0144	-0.0066	0.0762	0.0052	0.3192	0.6440917	Mineral
C	nifh_Cluster717	0.0024	0.0008	0.0486	-0.0246	0.4299	0.6587953	Mineral
C	nifh_Cluster725	0.0035	0.0007	0.0319	-0.0426	0.5243	0.6966928	Mineral
C	nifh_Cluster727	0.0038	-0.0003	0.0042	-0.0724	0.8178	0.8781415	Mineral
C	nifh_Cluster73	0.0133	-0.0049	0.0997	0.0305	0.2515	0.6440917	Mineral
C	nifh_Cluster74	0.005	-0.0023	0.0873	0.0171	0.2849	0.6440917	Mineral
C	nifh_Cluster748	0.0104	-0.0001	0.0004	-0.0765	0.9425	0.966933	Mineral
C	nifh_Cluster749	0.0055	0.0019	0.0866	0.0163	0.2871	0.6440917	Mineral
C	nifh_Cluster755	0.0118	-0.0008	0.004	-0.0726	0.8223	0.8805316	Mineral
C	nifh_Cluster76	0.0033	-0.0009	0.0357	-0.0385	0.5001	0.6838422	Mineral
C	nifh_Cluster79	0.002	-0.0008	0.071	-0.0005	0.3371	0.6440917	Mineral
C	nifh_Cluster81	0.0038	-0.0017	0.0771	0.0062	0.3162	0.6440917	Mineral
C	nifh_Cluster82	0.0014	-0.0001	0.0027	-0.074	0.8548	0.9005616	Mineral

C	nifh_Cluster83	0.0055	-0.0009	0.0152	-0.0606	0.662	0.7831606	Mineral
C	nifh_Cluster86	0.0161	-0.0026	0.0356	-0.0386	0.5007	0.6838422	Mineral
C	nifh_Cluster868	0.0098	0.0017	0.0344	-0.0399	0.5082	0.6863317	Mineral
C	nifh_Cluster87	0.003	-0.0013	0.0647	-0.0073	0.3604	0.6440917	Mineral
C	nifh_Cluster88	0.0054	-0.0004	0.0034	-0.0732	0.8355	0.8864719	Mineral
C	nifh_Cluster9	0.0077	-0.0007	0.0044	-0.0722	0.8145	0.8777196	Mineral
C	nifh_Cluster92	0.0068	-0.0028	0.0649	-0.0071	0.3596	0.6440917	Mineral
C	nifh_Cluster93	0.0035	-0.0012	0.0683	-0.0034	0.3468	0.6440917	Mineral
C	nifh_Cluster94	0.005	-0.0023	0.0795	0.0087	0.3087	0.6440917	Mineral
C	nifh_Cluster95	0.0149	-0.004	0.0389	-0.035	0.481	0.6838422	Mineral
C	nifh_Cluster96	0.0131	0.0041	0.0907	0.0208	0.2754	0.6440917	Mineral
C	nifh_Cluster97	0.0125	0.0034	0.2113	0.1507	0.0847	0.6440917	Mineral
C	nifh_Cluster98	0.0049	0.0004	0.0057	-0.0708	0.7895	0.8642272	Mineral
C	nifh_Cluster99	0.0035	-0.0016	0.0736	0.0024	0.3279	0.6440917	Mineral
C	nirk_Cluster0	6.5686	-0.4925	0.2525	0.195	0.0563	0.6440917	Mineral
C	nirk_Cluster1	0.2105	-0.0633	0.2808	0.2255	0.0422	0.6440917	Mineral
C	nirk_Cluster10	0.6649	-0.0488	0.0433	-0.0303	0.4568	0.6736646	Mineral
C	nirk_Cluster101	0.004	0.00E+00	0.00E+00	-0.0769	0.9869	0.9902866	Mineral
C	nirk_Cluster102	0.055	-0.0167	0.2052	0.144	0.09	0.6440917	Mineral
C	nirk_Cluster103	0.09	-0.0235	0.2035	0.1422	0.0915	0.6440917	Mineral
C	nirk_Cluster104	0.0028	-0.0006	0.0164	-0.0593	0.6494	0.7756324	Mineral
C	nirk_Cluster105	0.1172	-0.036	0.267	0.2106	0.0486	0.6440917	Mineral
C	nirk_Cluster106	0.073	-0.0236	0.1373	0.071	0.1739	0.6440917	Mineral
C	nirk_Cluster107	0.1123	-0.0291	0.1652	0.101	0.1327	0.6440917	Mineral
C	nirk_Cluster108	0.0184	-0.005	0.0927	0.0229	0.27	0.6440917	Mineral
C	nirk_Cluster109	0.0158	-0.0048	0.1387	0.0724	0.1716	0.6440917	Mineral
C	nirk_Cluster11	0.1509	-0.0078	0.0598	-0.0125	0.3797	0.6448159	Mineral
C	nirk_Cluster12	0.1052	-0.0163	0.1399	0.0737	0.1696	0.6440917	Mineral
C	nirk_Cluster13	0.0418	0.0003	0.0003	-0.0765	0.9474	0.968545	Mineral
C	nirk_Cluster14	0.1084	-0.0125	0.1404	0.0743	0.1687	0.6440917	Mineral
C	nirk_Cluster15	0.4141	-0.1169	0.241	0.1826	0.0631	0.6440917	Mineral
C	nirk_Cluster16	0.5816	-0.1511	0.1714	0.1076	0.125	0.6440917	Mineral
C	nirk_Cluster17	0.1044	-0.0087	0.0573	-0.0152	0.39	0.6481547	Mineral
C	nirk_Cluster18	0.1461	-0.0463	0.2371	0.1784	0.0656	0.6440917	Mineral
C	nirk_Cluster19	0.2953	-0.0222	0.0721	0.0007	0.3333	0.6440917	Mineral
C	nirk_Cluster2	0.2004	0.016	0.0552	-0.0175	0.3992	0.6525012	Mineral
C	nirk_Cluster20	0.1934	-0.0318	0.2103	0.1495	0.0856	0.6440917	Mineral
C	nirk_Cluster21	0.1711	-0.0228	0.2629	0.2062	0.0506	0.6440917	Mineral
C	nirk_Cluster22	0.1469	-0.0282	0.2066	0.1456	0.0887	0.6440917	Mineral
C	nirk_Cluster23	0.0729	-0.0111	0.2686	0.2123	0.0478	0.6440917	Mineral
C	nirk_Cluster24	0.0753	-0.012	0.1765	0.1132	0.119	0.6440917	Mineral
C	nirk_Cluster25	0.5519	-0.0882	0.2766	0.2209	0.0441	0.6440917	Mineral
C	nirk_Cluster26	0.1119	-0.0128	0.1404	0.0743	0.1688	0.6440917	Mineral
C	nirk_Cluster27	0.2066	-0.0373	0.1847	0.122	0.1099	0.6440917	Mineral
C	nirk_Cluster28	0.0808	-0.0139	0.1545	0.0895	0.1472	0.6440917	Mineral
C	nirk_Cluster29	0.162	0.0043	0.0046	-0.0719	0.8095	0.8763878	Mineral
C	nirk_Cluster3	0.3427	-0.0059	0.0036	-0.073	0.8315	0.8838969	Mineral
C	nirk_Cluster30	0.0712	-0.0158	0.1574	0.0926	0.1432	0.6440917	Mineral
C	nirk_Cluster31	0.1423	0.0195	0.1909	0.1287	0.1034	0.6440917	Mineral
C	nirk_Cluster32	0.1568	-0.0541	0.2512	0.1936	0.057	0.6440917	Mineral
C	nirk_Cluster33	0.0812	-0.028	0.2172	0.157	0.0799	0.6440917	Mineral
C	nirk_Cluster34	0.0462	-0.0119	0.1729	0.1093	0.1232	0.6440917	Mineral
C	nirk_Cluster35	0.1282	-0.0314	0.1091	0.0406	0.2292	0.6440917	Mineral
C	nirk_Cluster36	0.0021	-0.0006	0.0289	-0.0458	0.5447	0.7097936	Mineral
C	nirk_Cluster39	0.0045	0.0002	0.0002	-0.0767	0.9561	0.9692913	Mineral

C	nirk_Cluster4	0.1521	-0.0305	0.2107	0.15	0.0852	0.6440917	Mineral
C	nirk_Cluster40	0.0544	-0.0158	0.4252	0.3809	0.0084	0.331721	Mineral
C	nirk_Cluster41	0.1797	-0.0354	0.4617	0.4203	0.0053	0.2820262	Mineral
C	nirk_Cluster44	0.0756	-0.0159	0.1314	0.0646	0.1843	0.6440917	Mineral
C	nirk_Cluster46	0.0193	-0.0051	0.0671	-0.0047	0.3512	0.6440917	Mineral
C	nirk_Cluster48	0.003	-0.0008	0.0378	-0.0362	0.4876	0.6838422	Mineral
C	nirk_Cluster49	0.0072	-0.0015	0.024	-0.0511	0.5814	0.7336865	Mineral
C	nirk_Cluster5	0.2174	-0.0201	0.0993	0.0301	0.2525	0.6440917	Mineral
C	nirk_Cluster50	0.0014	-0.0005	0.0481	-0.0251	0.4322	0.6605735	Mineral
C	nirk_Cluster52	0.0551	-0.0143	0.2593	0.2023	0.0525	0.6440917	Mineral
C	nirk_Cluster53	0.0039	-0.0016	0.053	-0.0198	0.4091	0.653328	Mineral
C	nirk_Cluster54	0.0024	-0.0007	0.0374	-0.0366	0.4897	0.6838422	Mineral
C	nirk_Cluster55	0.0028	-0.001	0.0279	-0.0469	0.5521	0.7132088	Mineral
C	nirk_Cluster56	0.0009	-0.0004	0.0223	-0.0529	0.5949	0.7420891	Mineral
C	nirk_Cluster57	0.0017	-0.0007	0.0292	-0.0455	0.5427	0.7097936	Mineral
C	nirk_Cluster58	0.0102	-0.0017	0.024	-0.0511	0.5816	0.7336865	Mineral
C	nirk_Cluster59	0.007	-0.0015	0.0383	-0.0357	0.4845	0.6838422	Mineral
C	nirk_Cluster6	0.1713	-0.0175	0.0783	0.0074	0.3123	0.6440917	Mineral
C	nirk_Cluster60	0.0006	-0.0001	0.0089	-0.0673	0.7379	0.8276658	Mineral
C	nirk_Cluster62	0.1789	-0.0419	0.1321	0.0653	0.183	0.6440917	Mineral
C	nirk_Cluster63	0.1586	-0.0362	0.382	0.3345	0.0141	0.4147775	Mineral
C	nirk_Cluster64	0.0043	-0.001	0.0322	-0.0423	0.5224	0.6957141	Mineral
C	nirk_Cluster65	0.0531	-0.0141	0.307	0.2537	0.0321	0.6440917	Mineral
C	nirk_Cluster66	0.0095	-0.0035	0.1928	0.1307	0.1015	0.6440917	Mineral
C	nirk_Cluster67	0.0232	-0.0014	0.0045	-0.0721	0.8128	0.8777196	Mineral
C	nirk_Cluster68	0.0158	-0.0041	0.1118	0.0435	0.2232	0.6440917	Mineral
C	nirk_Cluster69	0.0362	-0.0083	0.1654	0.1012	0.1325	0.6440917	Mineral
C	nirk_Cluster7	0.096	-0.0135	0.1018	0.0328	0.2463	0.6440917	Mineral
C	nirk_Cluster70	0.0892	-0.0228	0.0673	-0.0044	0.3504	0.6440917	Mineral
C	nirk_Cluster71	0.0071	-0.0021	0.0952	0.0256	0.2631	0.6440917	Mineral
C	nirk_Cluster72	0.0278	-0.0075	0.2259	0.1663	0.0734	0.6440917	Mineral
C	nirk_Cluster73	0.1947	-0.0592	0.4381	0.3949	0.0072	0.3106989	Mineral
C	nirk_Cluster74	0.8012	-0.2196	0.248	0.1902	0.0589	0.6440917	Mineral
C	nirk_Cluster75	0.0186	-0.006	0.0771	0.0061	0.3165	0.6440917	Mineral
C	nirk_Cluster76	0.1377	-0.0373	0.5387	0.5032	0.0018	0.1356539	Mineral
C	nirk_Cluster77	0.0113	-0.0003	0.0003	-0.0766	0.9534	0.969274	Mineral
C	nirk_Cluster78	0.3126	-0.0941	0.4361	0.3927	0.0074	0.3106989	Mineral
C	nirk_Cluster79	0.0096	-0.001	0.005	-0.0715	0.8021	0.8714803	Mineral
C	nirk_Cluster8	0.1182	-0.0274	0.1657	0.1015	0.1321	0.6440917	Mineral
C	nirk_Cluster80	0.3544	-0.1153	0.4026	0.3567	0.0111	0.3914386	Mineral
C	nirk_Cluster82	0.0021	-0.0006	0.0171	-0.0585	0.6425	0.7737004	Mineral
C	nirk_Cluster83	0.0045	-0.0008	0.0243	-0.0507	0.5789	0.7336865	Mineral
C	nirk_Cluster84	0.0235	-0.0035	0.0196	-0.0558	0.6188	0.7578089	Mineral
C	nirk_Cluster85	0.0053	-0.0008	0.0198	-0.0556	0.6169	0.7578089	Mineral
C	nirk_Cluster86	0.0352	-0.0088	0.1156	0.0476	0.215	0.6440917	Mineral
C	nirk_Cluster87	0.0138	-0.0039	0.1089	0.0404	0.2296	0.6440917	Mineral
C	nirk_Cluster88	0.0035	-0.0005	0.0138	-0.0621	0.677	0.7910022	Mineral
C	nirk_Cluster89	0.0087	-0.003	0.0966	0.0271	0.2594	0.6440917	Mineral
C	nirk_Cluster9	0.061	-0.0111	0.0833	0.0128	0.2968	0.6440917	Mineral
C	nirk_Cluster90	0.0087	-0.0029	0.0692	-0.0024	0.3436	0.6440917	Mineral
C	nirk_Cluster91	0.044	-0.0138	0.2809	0.2255	0.0422	0.6440917	Mineral
C	nirk_Cluster92	0.009	-0.0028	0.0668	-0.005	0.3523	0.6440917	Mineral
C	nirk_Cluster93	0.0357	-0.0054	0.0445	-0.029	0.4503	0.6725787	Mineral
C	nirk_Cluster94	0.0222	-0.0062	0.1358	0.0693	0.1766	0.6440917	Mineral
C	nirk_Cluster95	0.0566	-0.0175	0.18	0.1169	0.115	0.6440917	Mineral

C	nirk_Cluster96	0.0588	-0.0167	0.2154	0.155	0.0814	0.6440917	Mineral
C	nirk_Cluster97	0.0329	-0.0083	0.0996	0.0303	0.2519	0.6440917	Mineral
C	nirk_Cluster98	0.0225	-0.0055	0.0553	-0.0173	0.3986	0.6525012	Mineral
C	nirk_Cluster99	0.0074	-0.0013	0.0228	-0.0524	0.5914	0.7396067	Mineral
C	nirs_Cluster0	0.1586	-0.017	0.0729	0.0016	0.3303	0.6440917	Mineral
C	nirs_Cluster1	0.0051	-0.0012	0.0202	-0.0551	0.613	0.7578017	Mineral
C	nirs_Cluster14	0.0142	-0.0065	0.0612	-0.011	0.3738	0.6440917	Mineral
C	nirs_Cluster2	0.0174	-0.0015	0.0032	-0.0735	0.8417	0.8883426	Mineral
C	nirs_Cluster26	0.0007	-0.0003	0.0227	-0.0524	0.5917	0.7396067	Mineral
C	nirs_Cluster28	0.0099	-0.0047	0.07	-0.0015	0.3406	0.6440917	Mineral
C	nirs_Cluster3	0.1914	-0.0043	0.0074	-0.069	0.7611	0.8440688	Mineral
C	nirs_Cluster36	0.0018	-1.00E-04	0.0003	-0.0766	0.9545	0.969274	Mineral
C	nirs_Cluster37	0.0092	-0.0043	0.0357	-0.0385	0.5003	0.6838422	Mineral
C	nirs_Cluster4	0.0003	-0.0001	0.0143	-0.0615	0.671	0.7886597	Mineral
C	nirs_Cluster50	0.0024	0.0011	0.0543	-0.0184	0.4031	0.653328	Mineral
C	nirs_Cluster9	0.0376	-0.0187	0.0762	0.0051	0.3193	0.6440917	Mineral
C	norb_Cluster0	0.0848	-0.021	0.1158	0.0477	0.2147	0.6440917	Mineral
C	norb_Cluster1	1.2746	-0.3732	0.1238	0.0564	0.1984	0.6440917	Mineral
C	norb_Cluster101	0.0037	-0.0015	0.0628	-0.0093	0.3676	0.6440917	Mineral
C	norb_Cluster102	0.005	-0.001	0.0282	-0.0466	0.5497	0.7132088	Mineral
C	norb_Cluster103	0.0025	-0.0011	0.062	-0.0102	0.371	0.6440917	Mineral
C	norb_Cluster104	0.0025	-0.0007	0.016	-0.0597	0.653	0.7780364	Mineral
C	norb_Cluster106	0.1468	-0.0468	0.2818	0.2266	0.0417	0.6440917	Mineral
C	norb_Cluster107	0.0163	-0.0052	0.1	0.0307	0.2509	0.6440917	Mineral
C	norb_Cluster108	0.0069	-0.0019	0.0918	0.022	0.2723	0.6440917	Mineral
C	norb_Cluster109	0.0282	-0.0042	0.0328	-0.0416	0.5183	0.6918461	Mineral
C	norb_Cluster11	0.0156	-0.0041	0.0698	-0.0018	0.3414	0.6440917	Mineral
C	norb_Cluster110	0.0128	-0.0038	0.0825	0.0119	0.2992	0.6440917	Mineral
C	norb_Cluster112	0.0215	-0.0071	0.2571	0.1999	0.0537	0.6440917	Mineral
C	norb_Cluster113	0.0157	-0.0057	0.1512	0.0859	0.152	0.6440917	Mineral
C	norb_Cluster114	0.0035	-0.0015	0.0417	-0.032	0.4654	0.6736646	Mineral
C	norb_Cluster115	0.0034	-0.0007	0.0429	-0.0307	0.4589	0.6736646	Mineral
C	norb_Cluster116	0.0083	-0.0033	0.0979	0.0285	0.2563	0.6440917	Mineral
C	norb_Cluster117	0.0034	-0.0013	0.0599	-0.0124	0.3794	0.6448159	Mineral
C	norb_Cluster118	0.0044	-0.0014	0.0613	-0.0109	0.3735	0.6440917	Mineral
C	norb_Cluster119	0.0263	-0.0092	0.1277	0.0606	0.191	0.6440917	Mineral
C	norb_Cluster12	0.005	-0.0015	0.0349	-0.0394	0.5052	0.6852504	Mineral
C	norb_Cluster121	0.0023	-0.0006	0.0139	-0.062	0.6757	0.7910022	Mineral
C	norb_Cluster122	0.0184	-0.0052	0.1078	0.0392	0.2322	0.6440917	Mineral
C	norb_Cluster123	0.0071	-0.0021	0.0622	-0.01	0.3701	0.6440917	Mineral
C	norb_Cluster124	0.7452	-0.2154	0.129	0.062	0.1885	0.6440917	Mineral
C	norb_Cluster125	0.0052	-0.0021	0.1339	0.0672	0.1799	0.6440917	Mineral
C	norb_Cluster126	0.011	-0.0041	0.1505	0.0852	0.153	0.6440917	Mineral
C	norb_Cluster127	0.2041	-0.0729	0.1976	0.1359	0.0969	0.6440917	Mineral
C	norb_Cluster128	0.0123	-0.0039	0.1142	0.046	0.2181	0.6440917	Mineral
C	norb_Cluster13	0.0051	-0.0024	0.0728	0.0015	0.3306	0.6440917	Mineral
C	norb_Cluster131	0.0209	-0.0034	0.0358	-0.0384	0.4997	0.6838422	Mineral
C	norb_Cluster132	1.2771	-0.3674	0.3009	0.2471	0.0342	0.6440917	Mineral
C	norb_Cluster133	0.0023	-0.0003	0.0053	-0.0712	0.7963	0.8684668	Mineral
C	norb_Cluster135	0.0703	-0.0213	0.1792	0.1161	0.1158	0.6440917	Mineral
C	norb_Cluster136	0.025	-0.0047	0.0474	-0.0259	0.4357	0.6625813	Mineral
C	norb_Cluster139	0.0391	-0.0123	0.171	0.1072	0.1255	0.6440917	Mineral
C	norb_Cluster14	0.0037	-0.0017	0.1246	0.0572	0.197	0.6440917	Mineral
C	norb_Cluster140	0.0485	-0.0175	0.2763	0.2207	0.0442	0.6440917	Mineral
C	norb_Cluster142	0.0018	-0.0009	0.0441	-0.0295	0.4526	0.672688	Mineral

C	norb_Cluster144	0.0122	-0.0024	0.0516	-0.0213	0.4154	0.653328	Mineral
C	norb_Cluster146	0.0418	-0.0101	0.1379	0.0716	0.1729	0.6440917	Mineral
C	norb_Cluster15	0.0502	-0.0177	0.1621	0.0977	0.1368	0.6440917	Mineral
C	norb_Cluster150	0.0199	-0.0051	0.1527	0.0875	0.1498	0.6440917	Mineral
C	norb_Cluster155	0.1729	-0.05	0.1519	0.0867	0.1509	0.6440917	Mineral
C	norb_Cluster16	0.0165	-0.0071	0.1376	0.0713	0.1735	0.6440917	Mineral
C	norb_Cluster164	0.0242	-0.0107	0.1999	0.1383	0.0948	0.6440917	Mineral
C	norb_Cluster165	0.0092	-0.0041	0.1085	0.0399	0.2307	0.6440917	Mineral
C	norb_Cluster166	0.0046	-0.002	0.0962	0.0266	0.2606	0.6440917	Mineral
C	norb_Cluster169	0.0309	-0.0135	0.1936	0.1316	0.1008	0.6440917	Mineral
C	norb_Cluster17	0.0035	-0.0014	0.0929	0.0231	0.2694	0.6440917	Mineral
C	norb_Cluster170	0.0299	-0.0123	0.1416	0.0755	0.1669	0.6440917	Mineral
C	norb_Cluster171	0.0089	-0.0039	0.0892	0.0191	0.2796	0.6440917	Mineral
C	norb_Cluster172	0.0179	-0.0079	0.1395	0.0733	0.1702	0.6440917	Mineral
C	norb_Cluster175	0.0595	-0.0238	0.1399	0.0737	0.1696	0.6440917	Mineral
C	norb_Cluster176	0.0392	-0.017	0.1706	0.1067	0.126	0.6440917	Mineral
C	norb_Cluster177	0.0361	-0.0151	0.1545	0.0895	0.1472	0.6440917	Mineral
C	norb_Cluster179	0.003	-0.0004	0.0076	-0.0688	0.758	0.8421773	Mineral
C	norb_Cluster18	0.0047	-0.0016	0.0925	0.0227	0.2705	0.6440917	Mineral
C	norb_Cluster180	0.0133	-0.0008	0.0051	-0.0715	0.8012	0.8714803	Mineral
C	norb_Cluster181	0.0003	-0.0002	0.0151	-0.0606	0.6624	0.7831606	Mineral
C	norb_Cluster182	0.0018	-0.0007	0.0534	-0.0194	0.4074	0.653328	Mineral
C	norb_Cluster184	0.0175	-0.0069	0.1595	0.0948	0.1403	0.6440917	Mineral
C	norb_Cluster186	0.0011	-0.0005	0.0272	-0.0476	0.557	0.7159809	Mineral
C	norb_Cluster19	0.0058	-0.002	0.1252	0.0579	0.1957	0.6440917	Mineral
C	norb_Cluster192	0.0221	-0.0099	0.1833	0.1205	0.1113	0.6440917	Mineral
C	norb_Cluster193	0.0022	-0.001	0.0777	0.0067	0.3145	0.6440917	Mineral
C	norb_Cluster197	0.0398	-0.0151	0.0275	-0.0473	0.5546	0.7144112	Mineral
C	norb_Cluster2	0.0032	-0.0014	0.0595	-0.0128	0.3809	0.6448159	Mineral
C	norb_Cluster20	0.0665	-0.0212	0.1742	0.1106	0.1217	0.6440917	Mineral
C	norb_Cluster208	0.0015	-0.0007	0.0518	-0.0212	0.4148	0.653328	Mineral
C	norb_Cluster21	0.0542	-0.0178	0.1631	0.0987	0.1355	0.6440917	Mineral
C	norb_Cluster211	0.0141	-0.0049	0.0311	-0.0434	0.5293	0.7010972	Mineral
C	norb_Cluster212	0.0252	-0.0119	0.0993	0.0301	0.2525	0.6440917	Mineral
C	norb_Cluster214	0.0163	-0.0054	0.0503	-0.0227	0.4215	0.653328	Mineral
C	norb_Cluster217	0.0186	-0.0074	0.0679	-0.0038	0.3484	0.6440917	Mineral
C	norb_Cluster219	0.0108	-0.0051	0.0684	-0.0032	0.3463	0.6440917	Mineral
C	norb_Cluster22	0.0028	-0.0005	0.0077	-0.0687	0.7564	0.8421773	Mineral
C	norb_Cluster220	0.0248	-0.0101	0.0522	-0.0207	0.4127	0.653328	Mineral
C	norb_Cluster221	0.0248	-0.0091	0.0371	-0.0369	0.4913	0.6838422	Mineral
C	norb_Cluster222	0.0104	-0.004	0.0301	-0.0445	0.5363	0.7063347	Mineral
C	norb_Cluster224	0.0238	-0.011	0.0841	0.0136	0.2945	0.6440917	Mineral
C	norb_Cluster225	0.0095	-0.003	0.063	-0.0091	0.367	0.6440917	Mineral
C	norb_Cluster226	0.0049	-0.002	0.0695	-0.0021	0.3424	0.6440917	Mineral
C	norb_Cluster228	0.0009	-0.0005	0.019	-0.0564	0.6239	0.7578089	Mineral
C	norb_Cluster229	0.004	-0.0014	0.0634	-0.0087	0.3654	0.6440917	Mineral
C	norb_Cluster230	0.0019	-0.0007	0.0408	-0.033	0.4704	0.6736646	Mineral
C	norb_Cluster231	0.0061	-0.0016	0.0247	-0.0503	0.5756	0.7319416	Mineral
C	norb_Cluster232	0.0044	-0.001	0.0257	-0.0492	0.5681	0.7238713	Mineral
C	norb_Cluster234	0.017	-0.0064	0.0955	0.0259	0.2625	0.6440917	Mineral
C	norb_Cluster235	0.0012	-0.0004	0.0411	-0.0327	0.4688	0.6736646	Mineral
C	norb_Cluster238	0.0135	-0.0041	0.0413	-0.0324	0.4675	0.6736646	Mineral
C	norb_Cluster239	0.0109	-0.0037	0.0418	-0.0319	0.4646	0.6736646	Mineral
C	norb_Cluster24	0.3014	-0.0801	0.1407	0.0746	0.1683	0.6440917	Mineral
C	norb_Cluster244	0.0015	-0.0003	0.014	-0.0618	0.674	0.7906351	Mineral

C	norb_Cluster247	0.006	-0.0011	0.0364	-0.0377	0.4957	0.6838422	Mineral
C	norb_Cluster25	0.1263	-0.0375	0.1233	0.0559	0.1993	0.6440917	Mineral
C	norb_Cluster250	0.0146	-0.0045	0.099	0.0297	0.2533	0.6440917	Mineral
C	norb_Cluster251	0.0064	-0.0022	0.0744	0.0032	0.3255	0.6440917	Mineral
C	norb_Cluster26	0.1134	-0.0343	0.1364	0.0699	0.1755	0.6440917	Mineral
C	norb_Cluster28	0.0355	-0.0127	0.1883	0.1258	0.1061	0.6440917	Mineral
C	norb_Cluster29	0.2454	-0.0521	0.2119	0.1513	0.0842	0.6440917	Mineral
C	norb_Cluster31	0.0632	-0.0141	0.0836	0.0131	0.2958	0.6440917	Mineral
C	norb_Cluster32	0.1401	-0.0397	0.0923	0.0225	0.2709	0.6440917	Mineral
C	norb_Cluster33	0.434	0.0859	0.1048	0.0359	0.2392	0.6440917	Mineral
C	norb_Cluster35	0.0746	-0.0189	0.1042	0.0353	0.2406	0.6440917	Mineral
C	norb_Cluster36	1.1002	-0.0911	0.0106	-0.0656	0.7156	0.8151001	Mineral
C	norb_Cluster37	0.7173	-0.0213	0.002	-0.0747	0.8731	0.9123538	Mineral
C	norb_Cluster38	0.0138	-0.0039	0.1383	0.072	0.1722	0.6440917	Mineral
C	norb_Cluster39	0.0465	-0.0092	0.0639	-0.0081	0.3634	0.6440917	Mineral
C	norb_Cluster40	0.2213	-0.067	0.1606	0.0961	0.1387	0.6440917	Mineral
C	norb_Cluster42	0.2825	-0.013	0.0034	-0.0733	0.8369	0.8864719	Mineral
C	norb_Cluster44	0.0249	-0.0079	0.1095	0.0411	0.2282	0.6440917	Mineral
C	norb_Cluster46	0.2575	-0.0432	0.038	-0.036	0.4864	0.6838422	Mineral
C	norb_Cluster47	0.6453	-0.185	0.1593	0.0946	0.1406	0.6440917	Mineral
C	norb_Cluster5	0.0115	-0.0024	0.0602	-0.0121	0.3782	0.6448159	Mineral
C	norb_Cluster51	0.0122	-0.0046	0.0757	0.0046	0.3211	0.6440917	Mineral
C	norb_Cluster52	0.2809	-0.0849	0.1425	0.0765	0.1654	0.6440917	Mineral
C	norb_Cluster56	0.0178	-0.0059	0.1596	0.095	0.1401	0.6440917	Mineral
C	norb_Cluster57	0.004	-0.0013	0.1134	0.0452	0.2196	0.6440917	Mineral
C	norb_Cluster59	0.0322	-0.0056	0.0239	-0.0512	0.582	0.7336865	Mineral
C	norb_Cluster6	0.6934	-0.0518	0.0098	-0.0664	0.7262	0.8191916	Mineral
C	norb_Cluster60	0.345	-0.1188	0.094	0.0243	0.2665	0.6440917	Mineral
C	norb_Cluster61	0.5486	-0.1681	0.1686	0.1047	0.1284	0.6440917	Mineral
C	norb_Cluster62	0.003	0.002	0.2397	0.1812	0.064	0.6440917	Mineral
C	norb_Cluster64	0.0407	-0.0109	0.1881	0.1257	0.1063	0.6440917	Mineral
C	norb_Cluster66	0.0016	-0.0006	0.0369	-0.0372	0.4931	0.6838422	Mineral
C	norb_Cluster67	0.0279	-0.0072	0.0998	0.0305	0.2514	0.6440917	Mineral
C	norb_Cluster68	0.003	-0.0012	0.0827	0.0122	0.2986	0.6440917	Mineral
C	norb_Cluster69	0.0032	-0.0009	0.0373	-0.0368	0.4904	0.6838422	Mineral
C	norb_Cluster7	0.0786	-0.0254	0.1466	0.081	0.1589	0.6440917	Mineral
C	norb_Cluster70	0.1262	-0.0367	0.2148	0.1544	0.0819	0.6440917	Mineral
C	norb_Cluster71	0.1512	-0.0448	0.2717	0.2157	0.0463	0.6440917	Mineral
C	norb_Cluster74	0.0387	-0.0104	0.1806	0.1176	0.1143	0.6440917	Mineral
C	norb_Cluster75	0.1531	-0.0444	0.3435	0.293	0.0217	0.6091204	Mineral
C	norb_Cluster76	0.1215	-0.0388	0.0624	-0.0098	0.3694	0.6440917	Mineral
C	norb_Cluster77	0.0492	-0.0094	0.1015	0.0323	0.2472	0.6440917	Mineral
C	norb_Cluster8	0.0206	-0.0074	0.1561	0.0912	0.1449	0.6440917	Mineral
C	norb_Cluster80	0.0021	-0.0009	0.0633	-0.0087	0.3656	0.6440917	Mineral
C	norb_Cluster81	0.0013	-0.0005	0.0469	-0.0264	0.4382	0.6646531	Mineral
C	norb_Cluster83	0.011	-0.005	0.0717	0.0003	0.3347	0.6440917	Mineral
C	norb_Cluster84	0.0093	-0.0027	0.0662	-0.0056	0.3545	0.6440917	Mineral
C	norb_Cluster85	0.0025	-0.001	0.0538	-0.0189	0.4053	0.653328	Mineral
C	norb_Cluster86	0.041	-0.0172	0.0666	-0.0052	0.353	0.6440917	Mineral
C	norb_Cluster88	0.0019	-0.0008	0.0594	-0.0129	0.3813	0.6448159	Mineral
C	norb_Cluster90	0.0013	-0.0005	0.0147	-0.0611	0.6665	0.786505	Mineral
C	norb_Cluster91	0.7571	-0.253	0.1629	0.0985	0.1358	0.6440917	Mineral
C	norb_Cluster92	0.0021	-0.0005	0.0099	-0.0663	0.7243	0.818667	Mineral
C	norb_Cluster93	0.0028	-0.0011	0.0394	-0.0344	0.478	0.6827951	Mineral
C	norb_Cluster94	0.002	-0.0003	0.0063	-0.0702	0.779	0.8590855	Mineral

C	norb_Cluster96	0.0006	-0.0002	0.0123	-0.0636	0.6936	0.797704	Mineral
C	norb_Cluster97	0.0162	-0.0019	0.013	-0.0629	0.6855	0.7955733	Mineral
C	norb_Cluster98	0.0176	-0.0074	0.1157	0.0476	0.2149	0.6440917	Mineral
C	nosz_Cluster0	0.0057	-0.0011	0.029	-0.0457	0.5442	0.7097936	Mineral
C	nosz_Cluster10	0.0128	-0.0017	0.0166	-0.0591	0.6473	0.7756324	Mineral
C	nosz_Cluster102	0.0057	-0.0013	0.0867	0.0164	0.2869	0.6440917	Mineral
C	nosz_Cluster103	0.0496	-0.0106	0.0783	0.0074	0.3124	0.6440917	Mineral
C	nosz_Cluster105	0.0016	-0.0004	0.019	-0.0564	0.624	0.7578089	Mineral
C	nosz_Cluster106	0.0088	-0.0023	0.0521	-0.0208	0.4132	0.653328	Mineral
C	nosz_Cluster107	0.0003	-0.0001	0.0143	-0.0615	0.671	0.7886597	Mineral
C	nosz_Cluster108	0.0043	-0.0006	0.0095	-0.0666	0.7291	0.8209199	Mineral
C	nosz_Cluster109	0.0416	-0.0077	0.1267	0.0595	0.1929	0.6440917	Mineral
C	nosz_Cluster11	0.0104	-0.0028	0.0764	0.0054	0.3185	0.6440917	Mineral
C	nosz_Cluster110	0.0004	0.0014	0.9584	0.9552	0.00E+00	1.38E-07	Mineral
C	nosz_Cluster111	0.0078	-0.0021	0.074	0.0028	0.3266	0.6440917	Mineral
C	nosz_Cluster116	0.0074	-0.0007	0.0113	-0.0648	0.7062	0.8090087	Mineral
C	nosz_Cluster118	0.0064	-0.0023	0.0673	-0.0045	0.3506	0.6440917	Mineral
C	nosz_Cluster119	0.0019	-0.0009	0.0785	0.0076	0.3118	0.6440917	Mineral
C	nosz_Cluster120	0.0083	-0.0023	0.0705	-0.0011	0.339	0.6440917	Mineral
C	nosz_Cluster123	0.0136	-0.0054	0.1458	0.08	0.1603	0.6440917	Mineral
C	nosz_Cluster128	0.005	-0.0022	0.0996	0.0303	0.2519	0.6440917	Mineral
C	nosz_Cluster129	0.05	-0.0065	0.066	-0.0059	0.3555	0.6440917	Mineral
C	nosz_Cluster13	0.0124	-0.0028	0.0505	-0.0226	0.4209	0.653328	Mineral
C	nosz_Cluster130	0.1138	-0.0459	0.0413	-0.0325	0.4677	0.6736646	Mineral
C	nosz_Cluster132	0.004	-0.0014	0.1011	0.032	0.248	0.6440917	Mineral
C	nosz_Cluster14	0.0149	-0.0054	0.082	0.0114	0.3007	0.6440917	Mineral
C	nosz_Cluster16	0.008	-0.0011	0.0092	-0.067	0.7342	0.8250489	Mineral
C	nosz_Cluster17	0.0427	-0.014	0.1789	0.1158	0.1162	0.6440917	Mineral
C	nosz_Cluster18	0.0184	-0.0058	0.0693	-0.0022	0.343	0.6440917	Mineral
C	nosz_Cluster20	0.0117	-0.0046	0.0881	0.018	0.2826	0.6440917	Mineral
C	nosz_Cluster21	0.0117	-0.0049	0.1147	0.0466	0.217	0.6440917	Mineral
C	nosz_Cluster22	0.0325	-0.0121	0.0695	-0.0021	0.3424	0.6440917	Mineral
C	nosz_Cluster23	0.019	-0.0076	0.128	0.0609	0.1904	0.6440917	Mineral
C	nosz_Cluster24	0.0019	-0.0004	0.0159	-0.0598	0.6541	0.7780364	Mineral
C	nosz_Cluster25	0.0026	-0.0008	0.0331	-0.0413	0.5164	0.6908708	Mineral
C	nosz_Cluster26	0.0068	-0.0027	0.0813	0.0106	0.303	0.6440917	Mineral
C	nosz_Cluster27	0.007	-0.0022	0.0814	0.0107	0.3026	0.6440917	Mineral
C	nosz_Cluster28	0.0358	-0.0108	0.1129	0.0447	0.2207	0.6440917	Mineral
C	nosz_Cluster30	0.0341	-0.0107	0.1397	0.0736	0.1699	0.6440917	Mineral
C	nosz_Cluster32	0.016	-0.0037	0.0626	-0.0095	0.3683	0.6440917	Mineral
C	nosz_Cluster33	0.0187	-0.0073	0.1142	0.046	0.218	0.6440917	Mineral
C	nosz_Cluster34	0.0387	-0.0164	0.1068	0.0381	0.2345	0.6440917	Mineral
C	nosz_Cluster35	0.0097	-0.0034	0.1099	0.0415	0.2274	0.6440917	Mineral
C	nosz_Cluster36	0.0213	-0.0081	0.1545	0.0895	0.1472	0.6440917	Mineral
C	nosz_Cluster37	0.0077	-0.0023	0.0685	-0.0031	0.346	0.6440917	Mineral
C	nosz_Cluster38	0.0144	-0.0051	0.0658	-0.006	0.356	0.6440917	Mineral
C	nosz_Cluster39	0.0653	-0.0205	0.1531	0.088	0.1492	0.6440917	Mineral
C	nosz_Cluster40	0.0262	-0.007	0.0665	-0.0053	0.3534	0.6440917	Mineral
C	nosz_Cluster41	0.0258	-0.0079	0.0859	0.0156	0.289	0.6440917	Mineral
C	nosz_Cluster42	0.03	-0.0111	0.1268	0.0597	0.1926	0.6440917	Mineral
C	nosz_Cluster43	0.001	-0.0005	0.0278	-0.047	0.5524	0.7132088	Mineral
C	nosz_Cluster44	0.0068	-0.0022	0.0707	-0.0008	0.338	0.6440917	Mineral
C	nosz_Cluster45	0.0056	-0.0014	0.0422	-0.0315	0.4625	0.6736646	Mineral
C	nosz_Cluster46	0.0082	-0.0026	0.132	0.0653	0.1831	0.6440917	Mineral
C	nosz_Cluster47	0.0012	-0.0005	0.0499	-0.0232	0.4238	0.6545066	Mineral

C	nosz_Cluster48	0.0028	-0.0012	0.056	-0.0166	0.3957	0.6525012	Mineral
C	nosz_Cluster49	0.0012	-0.0003	0.0109	-0.0652	0.7112	0.8116444	Mineral
C	nosz_Cluster5	0.004	-0.0016	0.0611	-0.0111	0.3744	0.6440917	Mineral
C	nosz_Cluster51	0.003	-0.0006	0.0221	-0.0531	0.5971	0.74321	Mineral
C	nosz_Cluster53	0.0012	-0.0002	0.0056	-0.0709	0.7915	0.8648368	Mineral
C	nosz_Cluster54	0.0037	-0.0003	0.0024	-0.0743	0.8611	0.9039765	Mineral
C	nosz_Cluster57	0.2232	-0.0697	0.1349	0.0683	0.1781	0.6440917	Mineral
C	nosz_Cluster58	0.0053	-0.0026	0.1018	0.0328	0.2463	0.6440917	Mineral
C	nosz_Cluster59	0.0008	-0.0004	0.0165	-0.0591	0.6478	0.7756324	Mineral
C	nosz_Cluster6	0.0034	-0.0013	0.0491	-0.0241	0.4276	0.6569589	Mineral
C	nosz_Cluster60	0.0099	-0.0024	0.0408	-0.0329	0.4701	0.6736646	Mineral
C	nosz_Cluster61	0.0178	-0.0058	0.2524	0.1949	0.0563	0.6440917	Mineral
C	nosz_Cluster62	0.0493	-0.0149	0.1047	0.0358	0.2395	0.6440917	Mineral
C	nosz_Cluster64	0.0023	0.0002	0.0015	-0.0753	0.8921	0.9266204	Mineral
C	nosz_Cluster65	0.0083	-0.0014	0.0195	-0.056	0.6201	0.7578089	Mineral
C	nosz_Cluster66	0.0428	-0.014	0.1009	0.0317	0.2487	0.6440917	Mineral
C	nosz_Cluster67	0.0669	-0.0217	0.0994	0.0301	0.2525	0.6440917	Mineral
C	nosz_Cluster69	0.008	-0.0034	0.1576	0.0928	0.1429	0.6440917	Mineral
C	nosz_Cluster70	0.0655	-0.0257	0.0586	-0.0138	0.3845	0.6448159	Mineral
C	nosz_Cluster71	0.0101	-0.0038	0.0693	-0.0023	0.3431	0.6440917	Mineral
C	nosz_Cluster72	0.066	-0.0225	0.1382	0.0719	0.1724	0.6440917	Mineral
C	nosz_Cluster73	0.0031	-0.0011	0.0502	-0.0228	0.4219	0.653328	Mineral
C	nosz_Cluster74	0.0017	-0.0008	0.0648	-0.0071	0.3599	0.6440917	Mineral
C	nosz_Cluster75	0.132	-0.0563	0.0377	-0.0363	0.4879	0.6838422	Mineral
C	nosz_Cluster77	0.0002	-1.00E-04	0.0059	-0.0706	0.7856	0.8615809	Mineral
C	nosz_Cluster78	0.0054	-0.0022	0.1313	0.0645	0.1844	0.6440917	Mineral
C	nosz_Cluster79	0.0401	-0.009	0.0809	0.0102	0.3042	0.6440917	Mineral
C	nosz_Cluster8	0.0106	-0.0035	0.189	0.1266	0.1054	0.6440917	Mineral
C	nosz_Cluster80	0.0039	-0.0006	0.0126	-0.0633	0.6902	0.7955733	Mineral
C	nosz_Cluster81	0.0099	-0.0038	0.0532	-0.0196	0.4082	0.653328	Mineral
C	nosz_Cluster82	0.0023	0.0001	0.0003	-0.0766	0.9489	0.968545	Mineral
C	nosz_Cluster83	0.0014	-0.0007	0.0341	-0.0402	0.51	0.6870453	Mineral
C	nosz_Cluster85	0.0027	-0.0004	0.0061	-0.0704	0.7821	0.8601834	Mineral
C	nosz_Cluster86	0.0038	-0.0008	0.0216	-0.0536	0.6008	0.7462821	Mineral
C	nosz_Cluster87	0.0034	-0.0005	0.0127	-0.0633	0.6895	0.7955733	Mineral
C	nosz_Cluster88	0.0246	-0.0058	0.1445	0.0786	0.1623	0.6440917	Mineral
C	nosz_Cluster89	0.0607	-0.0165	0.0866	0.0163	0.287	0.6440917	Mineral
C	nosz_Cluster9	0.0034	-0.0011	0.0365	-0.0377	0.4955	0.6838422	Mineral
C	nosz_Cluster90	0.0139	-0.0028	0.083	0.0125	0.2976	0.6440917	Mineral
C	nosz_Cluster91	0.0232	-0.0024	0.0084	-0.0679	0.7459	0.835054	Mineral
C	nosz_Cluster93	0.0488	-0.0139	0.1086	0.0401	0.2302	0.6440917	Mineral
C	nosz_Cluster95	0.0094	-0.001	0.0126	-0.0634	0.6904	0.7955733	Mineral
C	nosz_Cluster96	0.0064	-0.0006	0.0099	-0.0662	0.7239	0.818667	Mineral
C	nosz_Cluster97	0.0026	-0.0008	0.0437	-0.0299	0.4546	0.6736646	Mineral
C	nosz_Cluster98	0.0035	0.00E+00	0.00E+00	-0.0769	0.9897	0.9913702	Mineral
C	nosz_Cluster99	0.0061	-0.0004	0.0047	-0.0719	0.8087	0.8763878	Mineral
C	nrfa_Cluster10	0.0978	-0.036	0.1178	0.0499	0.2104	0.6440917	Mineral
C	nrfa_Cluster100	0.1249	-0.0511	0.1036	0.0346	0.2421	0.6440917	Mineral
C	nrfa_Cluster104	0.1478	-0.0427	0.1399	0.0738	0.1695	0.6440917	Mineral
C	nrfa_Cluster105	0.006	-0.0024	0.0864	0.0161	0.2876	0.6440917	Mineral
C	nrfa_Cluster109	0.2753	-0.07	0.1213	0.0537	0.2032	0.6440917	Mineral
C	nrfa_Cluster116	0.3208	-0.0878	0.2613	0.2045	0.0515	0.6440917	Mineral
C	nrfa_Cluster118	0.2298	-0.0963	0.0924	0.0226	0.2706	0.6440917	Mineral
C	nrfa_Cluster125	0.1081	-0.0415	0.0692	-0.0024	0.3436	0.6440917	Mineral
C	nrfa_Cluster154	0.0837	-0.0323	0.0984	0.0291	0.2548	0.6440917	Mineral

C	nrfa_Cluster164	0.0044	-0.0017	0.0911	0.0211	0.2743	0.6440917	Mineral
C	nrfa_Cluster172	0.0036	-0.0016	0.0656	-0.0063	0.3569	0.6440917	Mineral
C	nrfa_Cluster175	0.0053	-0.0026	0.0646	-0.0074	0.3608	0.6440917	Mineral
C	nrfa_Cluster20	0.045	-0.0133	0.0742	0.0029	0.3262	0.6440917	Mineral
C	nrfa_Cluster26	0.1433	-0.0595	0.1018	0.0327	0.2464	0.6440917	Mineral
C	nrfa_Cluster29	0.0059	-0.0023	0.0713	-0.0002	0.3362	0.6440917	Mineral
C	nrfa_Cluster34	0.0642	-0.0196	0.0822	0.0116	0.3	0.6440917	Mineral
C	nrfa_Cluster38	0.0033	-0.0016	0.0614	-0.0108	0.3733	0.6440917	Mineral
C	nrfa_Cluster61	0.0047	-0.0015	0.0453	-0.0281	0.4461	0.671495	Mineral
C	nrfa_Cluster62	0.0589	-0.0259	0.0883	0.0182	0.2821	0.6440917	Mineral
C	nrfa_Cluster66	0.1282	-0.0374	0.1965	0.1347	0.098	0.6440917	Mineral
C	nrfa_Cluster69	0.0035	-0.0014	0.0626	-0.0095	0.3683	0.6440917	Mineral
C	nrfa_Cluster71	0.0322	-0.0111	0.0661	-0.0057	0.3548	0.6440917	Mineral
C	nrfa_Cluster72	0.0969	-0.0355	0.1064	0.0376	0.2355	0.6440917	Mineral
C	nrfa_Cluster73	0.0042	-0.002	0.0705	-0.001	0.3389	0.6440917	Mineral
C	nrfa_Cluster74	0.1692	-0.0609	0.0999	0.0306	0.2512	0.6440917	Mineral
C	nrfa_Cluster76	0.0922	-0.0389	0.109	0.0405	0.2294	0.6440917	Mineral
C	nrfa_Cluster79	0.0138	-0.0067	0.0506	-0.0225	0.4203	0.653328	Mineral
C	nrfa_Cluster87	0.0207	-0.0095	0.0711	-0.0003	0.3367	0.6440917	Mineral
C	nrfa_Cluster89	0.0023	-0.0011	0.0633	-0.0088	0.3658	0.6440917	Mineral
C	nrfa_Cluster92	0.0041	-0.0018	0.1221	0.0546	0.2017	0.6440917	Mineral
C	nrfa_Cluster93	0.0581	-0.0233	0.0669	-0.0049	0.352	0.6440917	Mineral
C	nrfa_Cluster94	0.0003	-0.0002	0.019	-0.0564	0.6239	0.7578089	Mineral
C	nrfa_Cluster97	0.0225	-0.0083	0.1543	0.0893	0.1475	0.6440917	Mineral
C	nrfa_Cluster99	0.0184	-0.0057	0.0829	0.0124	0.298	0.6440917	Mineral
N	amoA_A_Cluster26	0.0053	-0.0035	0.162	0.0976	0.1369	0.426386	Mineral
N	amoA_A_Cluster45	0.0071	-0.0044	0.2121	0.1515	0.0841	0.426386	Mineral
N	amoA_B_Cluster0	0.1272	-0.0227	0.2639	0.2073	0.0501	0.426386	Mineral
N	amoA_B_Cluster1	0.0205	-0.0028	0.0303	-0.0443	0.5349	0.6687275	Mineral
N	amoA_B_Cluster10	0.0539	-0.0123	0.225	0.1654	0.074	0.426386	Mineral
N	amoA_B_Cluster11	0.0486	-0.0062	0.0426	-0.0311	0.4607	0.6205858	Mineral
N	amoA_B_Cluster12	0.093	-0.016	0.1801	0.1171	0.1148	0.426386	Mineral
N	amoA_B_Cluster13	0.6417	-0.0776	0.0452	-0.0282	0.4468	0.6115826	Mineral
N	amoA_B_Cluster14	0.0339	-0.0047	0.0287	-0.0461	0.5464	0.6745665	Mineral
N	amoA_B_Cluster15	0.0413	-0.003	0.0136	-0.0623	0.6794	0.7798035	Mineral
N	amoA_B_Cluster2	0.2143	-0.0309	0.0683	-0.0034	0.3468	0.5357105	Mineral
N	amoA_B_Cluster20	0.0078	-0.002	0.0397	-0.0341	0.4763	0.6304315	Mineral
N	amoA_B_Cluster23	0.0626	-0.0076	0.0559	-0.0167	0.3963	0.5730466	Mineral
N	amoA_B_Cluster3	0.0244	0.00E+00	0.00E+00	-0.0769	0.9955	0.9955341	Mineral
N	amoA_B_Cluster4	0.0169	-0.0018	0.0145	-0.0613	0.6685	0.771853	Mineral
N	amoA_B_Cluster5	0.1032	-0.021	0.2843	0.2292	0.0407	0.426386	Mineral
N	amoA_B_Cluster6	0.0279	-0.0032	0.0341	-0.0402	0.51	0.651282	Mineral
N	amoA_B_Cluster7	0.0232	-0.0041	0.0394	-0.0345	0.4784	0.6304315	Mineral
N	amoA_B_Cluster8	0.0268	-0.001	0.0016	-0.0752	0.8881	0.9274364	Mineral
N	amoA_B_Cluster9	0.0693	-0.0051	0.0099	-0.0663	0.7246	0.8096965	Mineral
N	nifh_Cluster0	0.0318	0.0021	0.0143	-0.0615	0.6713	0.7736242	Mineral
N	nifh_Cluster10	0.0033	0.0002	0.0026	-0.0742	0.858	0.9137072	Mineral
N	nifh_Cluster100	0.002	-0.001	0.077	0.006	0.3166	0.5146043	Mineral
N	nifh_Cluster103	0.0433	-0.0062	0.0566	-0.016	0.3933	0.5730466	Mineral
N	nifh_Cluster1034	0.0337	-0.0098	0.0885	0.0184	0.2816	0.4949814	Mineral
N	nifh_Cluster105	0.0164	-0.0056	0.1048	0.0359	0.2392	0.4679294	Mineral
N	nifh_Cluster108	0.0076	-0.0019	0.0592	-0.0132	0.3824	0.5653928	Mineral
N	nifh_Cluster1099	0.0478	0.0274	0.1907	0.1284	0.1037	0.426386	Mineral
N	nifh_Cluster11	0.0025	-0.0016	0.2171	0.1569	0.08	0.426386	Mineral
N	nifh_Cluster1112	0.0037	-0.0006	0.0133	-0.0626	0.682	0.7798035	Mineral

N	nifh_Cluster112	0.0098	0.0081	0.3866	0.3394	0.0133	0.3576519	Mineral
N	nifh_Cluster113	0.0374	-0.0082	0.162	0.0976	0.1369	0.426386	Mineral
N	nifh_Cluster114	0.0304	0.0002	0.0002	-0.0767	0.9639	0.9822024	Mineral
N	nifh_Cluster1141	0.0296	-0.0076	0.0957	0.0261	0.2619	0.4794658	Mineral
N	nifh_Cluster115	0.0098	-0.0001	0.0002	-0.0767	0.9586	0.9813987	Mineral
N	nifh_Cluster116	0.0883	-0.0084	0.0322	-0.0423	0.5225	0.6615588	Mineral
N	nifh_Cluster1163	0.0051	-0.0012	0.0516	-0.0214	0.4157	0.5844847	Mineral
N	nifh_Cluster1179	0.0037	-0.0009	0.0229	-0.0523	0.5907	0.7127298	Mineral
N	nifh_Cluster1182	0.0209	0.0017	0.0282	-0.0465	0.5494	0.6745665	Mineral
N	nifh_Cluster1183	0.0045	0.0022	0.2627	0.206	0.0507	0.426386	Mineral
N	nifh_Cluster1197	0.0115	-0.0028	0.067	-0.0048	0.3516	0.5412478	Mineral
N	nifh_Cluster1199	0.0048	0.0009	0.023	-0.0521	0.5895	0.7127298	Mineral
N	nifh_Cluster120	0.02	-0.0122	0.1426	0.0766	0.1652	0.4275837	Mineral
N	nifh_Cluster1203	0.0217	-0.0048	0.0549	-0.0178	0.4008	0.5730466	Mineral
N	nifh_Cluster1206	0.006	-0.0005	0.0077	-0.0686	0.7559	0.8367111	Mineral
N	nifh_Cluster1207	0.0078	-0.0028	0.0854	0.015	0.2906	0.5024625	Mineral
N	nifh_Cluster1209	0.1617	0.0062	0.0101	-0.0661	0.7219	0.8081462	Mineral
N	nifh_Cluster121	0.0123	-0.0084	0.187	0.1245	0.1074	0.426386	Mineral
N	nifh_Cluster1211	0.0068	-0.0009	0.0169	-0.0587	0.644	0.7546775	Mineral
N	nifh_Cluster1213	0.0032	-0.0012	0.1206	0.053	0.2047	0.4344169	Mineral
N	nifh_Cluster122	0.0041	-0.0028	0.2384	0.1798	0.0648	0.426386	Mineral
N	nifh_Cluster123	0.0092	-0.0058	0.1889	0.1265	0.1054	0.426386	Mineral
N	nifh_Cluster1236	0.031	-0.0119	0.0826	0.012	0.2991	0.5056436	Mineral
N	nifh_Cluster1238	0.0034	-0.001	0.0349	-0.0393	0.505	0.6494608	Mineral
N	nifh_Cluster124	0.0101	-0.0021	0.0721	0.0007	0.3332	0.5256557	Mineral
N	nifh_Cluster1261	0.0023	-0.001	0.0554	-0.0173	0.3984	0.5730466	Mineral
N	nifh_Cluster1262	0.008	0.0004	0.0018	-0.0749	0.8795	0.9232788	Mineral
N	nifh_Cluster1266	0.0072	-0.001	0.0176	-0.058	0.6375	0.7522262	Mineral
N	nifh_Cluster1267	0.0388	0.0397	0.4797	0.4397	0.0042	0.2032625	Mineral
N	nifh_Cluster127	0.0033	-0.002	0.1148	0.0468	0.2166	0.4471191	Mineral
N	nifh_Cluster1278	0.2105	-0.0539	0.2024	0.141	0.0925	0.426386	Mineral
N	nifh_Cluster128	0.0043	-0.0025	0.2211	0.1612	0.0769	0.426386	Mineral
N	nifh_Cluster1289	0.004	0.0009	0.0374	-0.0366	0.4896	0.6362612	Mineral
N	nifh_Cluster129	0.0086	-0.0052	0.1765	0.1132	0.1189	0.426386	Mineral
N	nifh_Cluster1292	0.0106	-0.003	0.0725	0.0012	0.3318	0.5256557	Mineral
N	nifh_Cluster130	0.0071	-0.0048	0.1309	0.0641	0.1851	0.4285429	Mineral
N	nifh_Cluster1305	0.0048	-0.0004	0.006	-0.0705	0.7842	0.8568169	Mineral
N	nifh_Cluster1306	0.0055	-0.0003	0.0019	-0.0749	0.8771	0.9232788	Mineral
N	nifh_Cluster131	0.0067	-0.0043	0.1848	0.122	0.1098	0.426386	Mineral
N	nifh_Cluster1319	0.0048	-0.0008	0.0155	-0.0603	0.6589	0.766802	Mineral
N	nifh_Cluster132	0.0064	-0.0038	0.1716	0.1079	0.1247	0.426386	Mineral
N	nifh_Cluster1320	0.0028	-0.0007	0.0173	-0.0583	0.6408	0.753125	Mineral
N	nifh_Cluster1323	0.0022	0.00E+00	0.00E+00	-0.0769	0.987	0.9920825	Mineral
N	nifh_Cluster1324	0.0235	-0.0025	0.0108	-0.0653	0.7129	0.8042209	Mineral
N	nifh_Cluster1336	0.0159	-0.0005	0.0031	-0.0736	0.844	0.9054343	Mineral
N	nifh_Cluster1340	0.0478	0.0077	0.0189	-0.0565	0.6248	0.7393832	Mineral
N	nifh_Cluster1345	0.033	0.0252	0.3102	0.2571	0.031	0.426386	Mineral
N	nifh_Cluster1370	0.0022	-0.0003	0.0075	-0.0688	0.7584	0.8379306	Mineral
N	nifh_Cluster1375	0.0017	0.0007	0.0846	0.0142	0.293	0.5024625	Mineral
N	nifh_Cluster139	0.0038	-0.0007	0.013	-0.0629	0.6852	0.7819645	Mineral
N	nifh_Cluster140	0.0051	-0.0013	0.0523	-0.0206	0.4124	0.5844847	Mineral
N	nifh_Cluster141	0.0808	-0.0143	0.278	0.2224	0.0434	0.426386	Mineral
N	nifh_Cluster148	0.006	-1.00E-04	0.00E+00	-0.0769	0.9852	0.9920825	Mineral
N	nifh_Cluster1480	0.0006	0.0012	0.6129	0.5831	0.0006	0.0950058	Mineral
N	nifh_Cluster152	0.0602	-0.0074	0.0348	-0.0395	0.5058	0.6494608	Mineral

N	nifh_Cluster156	0.0459	0.0219	0.475	0.4346	0.0045	0.2032625	Mineral
N	nifh_Cluster1562	0.0293	0.0403	0.6948	0.6713	0.0001	0.0333949	Mineral
N	nifh_Cluster158	0.019	-0.0048	0.0694	-0.0022	0.3427	0.532051	Mineral
N	nifh_Cluster16	0.0023	-0.0014	0.1029	0.0339	0.2436	0.4708967	Mineral
N	nifh_Cluster166	0.0165	-0.0106	0.1671	0.103	0.1303	0.426386	Mineral
N	nifh_Cluster205	0.0121	-0.0029	0.1009	0.0318	0.2486	0.4735363	Mineral
N	nifh_Cluster21	0.0022	-0.0007	0.044	-0.0296	0.4531	0.6188563	Mineral
N	nifh_Cluster222	0.0069	-0.0044	0.1413	0.0753	0.1673	0.4275837	Mineral
N	nifh_Cluster225	0.021	0.0006	0.0022	-0.0745	0.8672	0.9169314	Mineral
N	nifh_Cluster230	0.0052	-0.0011	0.0391	-0.0348	0.4798	0.6304315	Mineral
N	nifh_Cluster231	0.0019	-0.0007	0.0404	-0.0334	0.4724	0.6303784	Mineral
N	nifh_Cluster236	0.0034	-0.0014	0.1147	0.0466	0.2168	0.4471191	Mineral
N	nifh_Cluster237	0.007	-0.0018	0.062	-0.0102	0.3709	0.5539758	Mineral
N	nifh_Cluster243	0.0253	0.0026	0.0071	-0.0693	0.7657	0.84126	Mineral
N	nifh_Cluster2432	0.0317	0.0268	0.4026	0.3567	0.0111	0.3309706	Mineral
N	nifh_Cluster2433	0.0028	-0.0004	0.0034	-0.0732	0.8354	0.9010502	Mineral
N	nifh_Cluster246	0.0068	-0.0035	0.1347	0.0682	0.1784	0.4275837	Mineral
N	nifh_Cluster25	0.0195	-0.0015	0.0157	-0.06	0.656	0.7663995	Mineral
N	nifh_Cluster256	0.0023	-0.0013	0.1244	0.057	0.1973	0.4296198	Mineral
N	nifh_Cluster264	0.0025	-0.0012	0.1327	0.066	0.1819	0.4275837	Mineral
N	nifh_Cluster265	0.0702	0.0061	0.047	-0.0263	0.4377	0.6048233	Mineral
N	nifh_Cluster267	0.0071	0.0001	0.0003	-0.0767	0.9553	0.9813987	Mineral
N	nifh_Cluster268	0.0109	0.0016	0.0515	-0.0215	0.4161	0.5844847	Mineral
N	nifh_Cluster269	0.0283	-0.0055	0.0358	-0.0384	0.4997	0.6479911	Mineral
N	nifh_Cluster272	0.0012	0.0015	0.3227	0.2706	0.0271	0.426386	Mineral
N	nifh_Cluster274	0.0014	-0.001	0.0548	-0.0179	0.4011	0.5730466	Mineral
N	nifh_Cluster275	0.0051	-0.0002	0.0011	-0.0758	0.9074	0.9367625	Mineral
N	nifh_Cluster278	0.0056	0.0031	0.1886	0.1262	0.1057	0.426386	Mineral
N	nifh_Cluster279	0.0058	0.0003	0.0028	-0.0739	0.8507	0.909276	Mineral
N	nifh_Cluster28	0.0017	-0.0008	0.0685	-0.0032	0.3461	0.5357105	Mineral
N	nifh_Cluster280	0.0034	0.0009	0.0155	-0.0603	0.6589	0.766802	Mineral
N	nifh_Cluster282	0.0052	-0.0017	0.0992	0.0299	0.2528	0.4735363	Mineral
N	nifh_Cluster287	0.003	0.0009	0.0559	-0.0167	0.3963	0.5730466	Mineral
N	nifh_Cluster290	0.0096	-0.0025	0.0406	-0.0332	0.4714	0.6303784	Mineral
N	nifh_Cluster297	0.004	-0.0027	0.1132	0.045	0.2201	0.447823	Mineral
N	nifh_Cluster299	0.0025	-1.00E-04	0.0002	-0.0767	0.9614	0.9813987	Mineral
N	nifh_Cluster303	0.002	0.0006	0.0419	-0.0318	0.4644	0.6241372	Mineral
N	nifh_Cluster304	0.0025	-0.0017	0.1871	0.1245	0.1074	0.426386	Mineral
N	nifh_Cluster313	0.0129	-0.0083	0.1712	0.1075	0.1252	0.426386	Mineral
N	nifh_Cluster314	0.0027	-0.0011	0.073	0.0017	0.3302	0.5256557	Mineral
N	nifh_Cluster317	0.0089	-0.0028	0.0967	0.0272	0.2592	0.4779076	Mineral
N	nifh_Cluster32	0.0029	-0.0005	0.0191	-0.0563	0.6229	0.7393832	Mineral
N	nifh_Cluster320	13.944	-2.0321	0.5896	0.558	0.0008	0.0950058	Mineral
N	nifh_Cluster334	0.0084	-0.0049	0.122	0.0544	0.202	0.4302621	Mineral
N	nifh_Cluster335	0.0059	-0.0036	0.1274	0.0603	0.1915	0.4285429	Mineral
N	nifh_Cluster338	0.0151	-0.0012	0.0104	-0.0657	0.7176	0.8064623	Mineral
N	nifh_Cluster340	0.0041	-0.0013	0.0813	0.0106	0.3031	0.5062103	Mineral
N	nifh_Cluster341	0.0021	0.0011	0.0848	0.0143	0.2925	0.5024625	Mineral
N	nifh_Cluster35	0.0079	0.0002	0.0014	-0.0754	0.8938	0.9317423	Mineral
N	nifh_Cluster351	0.0035	-0.002	0.1354	0.0689	0.1773	0.4275837	Mineral
N	nifh_Cluster3574	0.1191	0.0131	0.0434	-0.0302	0.4561	0.6193291	Mineral
N	nifh_Cluster3691	0.0224	-0.001	0.0017	-0.0751	0.8844	0.9252057	Mineral
N	nifh_Cluster37	0.0374	-0.0144	0.1236	0.0562	0.1988	0.4302621	Mineral
N	nifh_Cluster374	0.0029	0.0005	0.0102	-0.066	0.7205	0.8081462	Mineral
N	nifh_Cluster38	0.0008	-1.00E-04	0.0013	-0.0755	0.8974	0.9321677	Mineral

N	nifh_Cluster382	0.0023	-0.0016	0.0955	0.0259	0.2625	0.4794658	Mineral
N	nifh_Cluster384	0.0023	-0.0016	0.1919	0.1298	0.1024	0.426386	Mineral
N	nifh_Cluster386	0.0041	-0.0024	0.1123	0.044	0.2221	0.4502788	Mineral
N	nifh_Cluster389	0.003	-0.0019	0.0884	0.0183	0.2819	0.4949814	Mineral
N	nifh_Cluster394	0.0027	-0.0018	0.0941	0.0244	0.2662	0.4822041	Mineral
N	nifh_Cluster40	0.0014	-0.0007	0.0778	0.0069	0.3141	0.5146043	Mineral
N	nifh_Cluster43	0.0107	-0.0008	0.009	-0.0672	0.7367	0.8201316	Mineral
N	nifh_Cluster434	0.0048	-0.0013	0.0389	-0.035	0.4808	0.6304315	Mineral
N	nifh_Cluster440	0.0171	-0.0032	0.0294	-0.0453	0.5414	0.6739536	Mineral
N	nifh_Cluster462	0.0048	-0.002	0.0932	0.0235	0.2684	0.4822041	Mineral
N	nifh_Cluster470	0.017	0.0016	0.0112	-0.0648	0.7072	0.8008395	Mineral
N	nifh_Cluster49	0.0223	-0.0149	0.1944	0.1325	0.0999	0.426386	Mineral
N	nifh_Cluster499	0.0028	-0.0018	0.1761	0.1127	0.1194	0.426386	Mineral
N	nifh_Cluster5	0.0263	0.0073	0.2695	0.2133	0.0474	0.426386	Mineral
N	nifh_Cluster506	0.0058	0.0016	0.0624	-0.0097	0.3692	0.5534866	Mineral
N	nifh_Cluster51	0.0031	-0.0019	0.1008	0.0316	0.2488	0.4735363	Mineral
N	nifh_Cluster52	0.0396	-0.0209	0.1851	0.1224	0.1094	0.426386	Mineral
N	nifh_Cluster525	0.0025	-0.0011	0.0426	-0.031	0.4605	0.6205858	Mineral
N	nifh_Cluster531	0.0129	0.0122	0.6029	0.5723	0.0007	0.0950058	Mineral
N	nifh_Cluster539	0.0056	-0.0035	0.0774	0.0064	0.3155	0.5146043	Mineral
N	nifh_Cluster578	0.0593	-0.0033	0.008	-0.0683	0.7517	0.8352615	Mineral
N	nifh_Cluster58	0.2389	0.2253	0.5356	0.4999	0.0019	0.1551083	Mineral
N	nifh_Cluster60	0.011	-0.0055	0.186	0.1233	0.1085	0.426386	Mineral
N	nifh_Cluster61	0.0017	0.0004	0.0303	-0.0443	0.535	0.6687275	Mineral
N	nifh_Cluster65	0.0085	-0.0043	0.1868	0.1243	0.1076	0.426386	Mineral
N	nifh_Cluster686	0.0013	0.00E+00	0.00E+00	-0.0769	0.9928	0.9955341	Mineral
N	nifh_Cluster69	0.0092	-0.0064	0.2217	0.1619	0.0765	0.426386	Mineral
N	nifh_Cluster70	0.0144	-0.0095	0.1592	0.0946	0.1406	0.426386	Mineral
N	nifh_Cluster717	0.0024	0.001	0.0715	0.00E+00	0.3355	0.5277865	Mineral
N	nifh_Cluster725	0.0035	0.0002	0.0024	-0.0743	0.8615	0.9158143	Mineral
N	nifh_Cluster727	0.0038	-0.0002	0.0014	-0.0755	0.8963	0.9321677	Mineral
N	nifh_Cluster73	0.0133	-0.0065	0.1776	0.1143	0.1177	0.426386	Mineral
N	nifh_Cluster74	0.005	-0.003	0.1483	0.0827	0.1564	0.4275837	Mineral
N	nifh_Cluster748	0.0104	-1.00E-04	0.0001	-0.0768	0.9671	0.983775	Mineral
N	nifh_Cluster749	0.0055	0.0019	0.0847	0.0143	0.2927	0.5024625	Mineral
N	nifh_Cluster755	0.0118	0.0005	0.002	-0.0748	0.874	0.9224517	Mineral
N	nifh_Cluster76	0.0033	-0.0006	0.0174	-0.0582	0.6395	0.7531027	Mineral
N	nifh_Cluster79	0.002	-0.0009	0.0957	0.0261	0.2619	0.4794658	Mineral
N	nifh_Cluster81	0.0038	-0.0024	0.1643	0.1	0.1339	0.426386	Mineral
N	nifh_Cluster82	0.0014	-0.0003	0.0151	-0.0607	0.6628	0.7697445	Mineral
N	nifh_Cluster83	0.0055	-0.0006	0.0063	-0.0702	0.7788	0.8524545	Mineral
N	nifh_Cluster86	0.0161	-0.002	0.0214	-0.0539	0.6031	0.7232392	Mineral
N	nifh_Cluster868	0.0098	0.0019	0.0392	-0.0347	0.4792	0.6304315	Mineral
N	nifh_Cluster87	0.003	-0.0019	0.141	0.075	0.1677	0.4275837	Mineral
N	nifh_Cluster88	0.0054	0.00E+00	0.00E+00	-0.0769	0.9827	0.9920825	Mineral
N	nifh_Cluster9	0.0077	-0.0009	0.0072	-0.0692	0.7635	0.840709	Mineral
N	nifh_Cluster92	0.0068	-0.0041	0.14	0.0738	0.1695	0.4275837	Mineral
N	nifh_Cluster93	0.0035	-0.0017	0.1296	0.0627	0.1874	0.4285429	Mineral
N	nifh_Cluster94	0.005	-0.0032	0.1553	0.0903	0.1461	0.4275837	Mineral
N	nifh_Cluster95	0.0149	-0.0058	0.0834	0.0128	0.2967	0.5047519	Mineral
N	nifh_Cluster96	0.0131	0.0049	0.1284	0.0613	0.1897	0.4285429	Mineral
N	nifh_Cluster97	0.0125	0.0036	0.2325	0.1735	0.0687	0.426386	Mineral
N	nifh_Cluster98	0.0049	0.0008	0.0229	-0.0523	0.5904	0.7127298	Mineral
N	nifh_Cluster99	0.0035	-0.0023	0.1542	0.0891	0.1477	0.4275837	Mineral
N	nirk_Cluster0	6.5686	-0.4606	0.2209	0.1609	0.0771	0.426386	Mineral

N	nirk_Cluster1	0.2105	-0.0477	0.159	0.0943	0.1409	0.426386	Mineral
N	nirk_Cluster10	0.6649	-0.0305	0.0169	-0.0588	0.6447	0.7546775	Mineral
N	nirk_Cluster101	0.004	-0.0006	0.011	-0.065	0.7094	0.8018138	Mineral
N	nirk_Cluster102	0.055	-0.0191	0.2684	0.2121	0.0479	0.426386	Mineral
N	nirk_Cluster103	0.09	-0.0212	0.1655	0.1013	0.1324	0.426386	Mineral
N	nirk_Cluster104	0.0028	-0.0004	0.0064	-0.07	0.7766	0.8517115	Mineral
N	nirk_Cluster105	0.1172	-0.0413	0.3516	0.3017	0.0198	0.426386	Mineral
N	nirk_Cluster106	0.073	-0.0256	0.1614	0.0969	0.1376	0.426386	Mineral
N	nirk_Cluster107	0.1123	-0.0337	0.2218	0.1619	0.0764	0.426386	Mineral
N	nirk_Cluster108	0.0184	-0.006	0.1337	0.0671	0.1801	0.4275837	Mineral
N	nirk_Cluster109	0.0158	-0.0054	0.1758	0.1124	0.1198	0.426386	Mineral
N	nirk_Cluster11	0.1509	-0.0082	0.0668	-0.005	0.3523	0.5412478	Mineral
N	nirk_Cluster12	0.1052	-0.0206	0.2215	0.1616	0.0766	0.426386	Mineral
N	nirk_Cluster13	0.0418	-0.0008	0.0026	-0.0741	0.8564	0.9137072	Mineral
N	nirk_Cluster14	0.1084	-0.0096	0.0831	0.0126	0.2974	0.5047519	Mineral
N	nirk_Cluster15	0.4141	-0.1244	0.273	0.2171	0.0457	0.426386	Mineral
N	nirk_Cluster16	0.5816	-0.1601	0.1924	0.1303	0.1019	0.426386	Mineral
N	nirk_Cluster17	0.1044	-0.0141	0.1516	0.0863	0.1515	0.4275837	Mineral
N	nirk_Cluster18	0.1461	-0.0602	0.4014	0.3553	0.0112	0.3309706	Mineral
N	nirk_Cluster19	0.2953	-0.0284	0.1182	0.0504	0.2095	0.4415279	Mineral
N	nirk_Cluster2	0.2004	0.0194	0.0811	0.0104	0.3036	0.5062103	Mineral
N	nirk_Cluster20	0.1934	-0.0335	0.2334	0.1744	0.0681	0.426386	Mineral
N	nirk_Cluster21	0.1711	-0.0255	0.3301	0.2786	0.0251	0.426386	Mineral
N	nirk_Cluster22	0.1469	-0.0297	0.2283	0.1689	0.0717	0.426386	Mineral
N	nirk_Cluster23	0.0729	-0.0123	0.3293	0.2777	0.0253	0.426386	Mineral
N	nirk_Cluster24	0.0753	-0.0086	0.0917	0.0218	0.2727	0.4831174	Mineral
N	nirk_Cluster25	0.5519	-0.0784	0.2185	0.1584	0.0789	0.426386	Mineral
N	nirk_Cluster26	0.1119	-0.0145	0.1796	0.1165	0.1155	0.426386	Mineral
N	nirk_Cluster27	0.2066	-0.0422	0.2365	0.1778	0.0661	0.426386	Mineral
N	nirk_Cluster28	0.0808	-0.0146	0.1693	0.1054	0.1276	0.426386	Mineral
N	nirk_Cluster29	0.162	0.0056	0.0079	-0.0684	0.7533	0.8353717	Mineral
N	nirk_Cluster3	0.3427	-0.0007	1.00E-04	-0.0769	0.9793	0.9920825	Mineral
N	nirk_Cluster30	0.0712	-0.0178	0.2006	0.1391	0.0941	0.426386	Mineral
N	nirk_Cluster31	0.1423	0.0192	0.1858	0.1232	0.1087	0.426386	Mineral
N	nirk_Cluster32	0.1568	-0.0645	0.3577	0.3083	0.0185	0.426386	Mineral
N	nirk_Cluster33	0.0812	-0.0312	0.2701	0.2139	0.0471	0.426386	Mineral
N	nirk_Cluster34	0.0462	-0.0148	0.2662	0.2098	0.049	0.426386	Mineral
N	nirk_Cluster35	0.1282	-0.0365	0.1473	0.0817	0.1579	0.4275837	Mineral
N	nirk_Cluster36	0.0021	-0.0008	0.0502	-0.0229	0.4222	0.5902891	Mineral
N	nirk_Cluster39	0.0045	0.0003	0.0011	-0.0757	0.9047	0.9363969	Mineral
N	nirk_Cluster4	0.1521	-0.0357	0.2901	0.2355	0.0383	0.426386	Mineral
N	nirk_Cluster40	0.0544	-0.0171	0.4968	0.4581	0.0033	0.1792043	Mineral
N	nirk_Cluster41	0.1797	-0.0356	0.4658	0.4248	0.0051	0.2092856	Mineral
N	nirk_Cluster44	0.0756	-0.0104	0.0557	-0.0169	0.397	0.5730466	Mineral
N	nirk_Cluster46	0.0193	-0.0058	0.088	0.0179	0.2829	0.4952063	Mineral
N	nirk_Cluster48	0.003	-0.0003	0.0051	-0.0715	0.8012	0.8689019	Mineral
N	nirk_Cluster49	0.0072	-0.001	0.0094	-0.0668	0.7315	0.8158842	Mineral
N	nirk_Cluster5	0.2174	-0.0256	0.1603	0.0957	0.1392	0.426386	Mineral
N	nirk_Cluster50	0.0014	-0.0008	0.0932	0.0235	0.2684	0.4822041	Mineral
N	nirk_Cluster52	0.0551	-0.0157	0.3114	0.2584	0.0306	0.426386	Mineral
N	nirk_Cluster53	0.0039	-0.0023	0.108	0.0394	0.2316	0.4601428	Mineral
N	nirk_Cluster54	0.0024	-0.0008	0.0534	-0.0195	0.4075	0.580762	Mineral
N	nirk_Cluster55	0.0028	-0.001	0.028	-0.0468	0.5511	0.6745665	Mineral
N	nirk_Cluster56	0.0009	-0.0001	0.0029	-0.0738	0.848	0.908023	Mineral
N	nirk_Cluster57	0.0017	-0.0008	0.0338	-0.0405	0.512	0.6524423	Mineral

N	nirk_Cluster58	0.0102	0.0005	0.0018	-0.075	0.8816	0.9239134	Mineral
N	nirk_Cluster59	0.007	-0.0013	0.0305	-0.0441	0.5339	0.6687275	Mineral
N	nirk_Cluster6	0.1713	-0.0072	0.0133	-0.0626	0.6818	0.7798035	Mineral
N	nirk_Cluster60	0.0006	-0.0002	0.0128	-0.0631	0.6875	0.7830655	Mineral
N	nirk_Cluster62	0.1789	-0.0193	0.0281	-0.0466	0.5502	0.6745665	Mineral
N	nirk_Cluster63	0.1586	-0.0272	0.2161	0.1558	0.0808	0.426386	Mineral
N	nirk_Cluster64	0.0043	-0.0011	0.0438	-0.0298	0.4542	0.6188563	Mineral
N	nirk_Cluster65	0.0531	-0.0145	0.3286	0.277	0.0255	0.426386	Mineral
N	nirk_Cluster66	0.0095	-0.0039	0.2294	0.1701	0.0709	0.426386	Mineral
N	nirk_Cluster67	0.0232	0.0007	0.0011	-0.0758	0.9082	0.9367625	Mineral
N	nirk_Cluster68	0.0158	-0.0029	0.0554	-0.0173	0.3985	0.5730466	Mineral
N	nirk_Cluster69	0.0362	-0.0067	0.1086	0.0401	0.2303	0.4590432	Mineral
N	nirk_Cluster7	0.096	-0.0149	0.1248	0.0574	0.1966	0.4296198	Mineral
N	nirk_Cluster70	0.0892	-0.005	0.0032	-0.0735	0.842	0.9049312	Mineral
N	nirk_Cluster71	0.0071	-0.0027	0.153	0.0879	0.1493	0.4275837	Mineral
N	nirk_Cluster72	0.0278	-0.0058	0.1334	0.0668	0.1806	0.4275837	Mineral
N	nirk_Cluster73	0.1947	-0.065	0.5296	0.4934	0.0021	0.1551083	Mineral
N	nirk_Cluster74	0.8012	-0.1413	0.1027	0.0337	0.2442	0.4708967	Mineral
N	nirk_Cluster75	0.0186	-0.0065	0.0927	0.0229	0.27	0.4822041	Mineral
N	nirk_Cluster76	0.1377	-0.0343	0.4568	0.415	0.0057	0.2092856	Mineral
N	nirk_Cluster77	0.0113	0.0009	0.0019	-0.0749	0.8789	0.9232788	Mineral
N	nirk_Cluster78	0.3126	-0.1086	0.5803	0.548	0.001	0.0950058	Mineral
N	nirk_Cluster79	0.0096	0.00E+00	0.00E+00	-0.0769	0.994	0.9955341	Mineral
N	nirk_Cluster8	0.1182	-0.0351	0.2719	0.2158	0.0462	0.426386	Mineral
N	nirk_Cluster80	0.3544	-0.129	0.5039	0.4657	0.003	0.1792043	Mineral
N	nirk_Cluster82	0.0021	0.00E+00	0.00E+00	-0.0769	0.9821	0.9920825	Mineral
N	nirk_Cluster83	0.0045	-0.0007	0.0196	-0.0558	0.6185	0.7356877	Mineral
N	nirk_Cluster84	0.0235	-0.0059	0.0555	-0.0172	0.3981	0.5730466	Mineral
N	nirk_Cluster85	0.0053	-0.0007	0.0149	-0.0609	0.6645	0.7702385	Mineral
N	nirk_Cluster86	0.0352	-0.0059	0.0522	-0.0207	0.4129	0.5844847	Mineral
N	nirk_Cluster87	0.0138	-0.0036	0.0922	0.0223	0.2713	0.4822041	Mineral
N	nirk_Cluster88	0.0035	-0.0006	0.0244	-0.0506	0.5783	0.703467	Mineral
N	nirk_Cluster89	0.0087	-0.0044	0.2034	0.1422	0.0915	0.426386	Mineral
N	nirk_Cluster9	0.061	-0.0147	0.146	0.0803	0.1599	0.4275837	Mineral
N	nirk_Cluster90	0.0087	-0.003	0.0723	0.0009	0.3326	0.5256557	Mineral
N	nirk_Cluster91	0.044	-0.0143	0.3032	0.2495	0.0334	0.426386	Mineral
N	nirk_Cluster92	0.009	-0.0012	0.0117	-0.0643	0.7014	0.7958066	Mineral
N	nirk_Cluster93	0.0357	-0.0053	0.0431	-0.0305	0.4577	0.6193291	Mineral
N	nirk_Cluster94	0.0222	-0.0071	0.1742	0.1107	0.1216	0.426386	Mineral
N	nirk_Cluster95	0.0566	-0.0154	0.1409	0.0749	0.1679	0.4275837	Mineral
N	nirk_Cluster96	0.0588	-0.0182	0.2558	0.1985	0.0544	0.426386	Mineral
N	nirk_Cluster97	0.0329	-0.0082	0.0978	0.0284	0.2564	0.477225	Mineral
N	nirk_Cluster98	0.0225	-0.0062	0.0704	-0.0011	0.339	0.5277865	Mineral
N	nirk_Cluster99	0.0074	-0.0015	0.0298	-0.0448	0.5384	0.6715827	Mineral
N	nirs_Cluster0	0.1586	-0.014	0.0494	-0.0237	0.4257	0.5924151	Mineral
N	nirs_Cluster1	0.0051	-0.0012	0.0203	-0.055	0.6123	0.7312708	Mineral
N	nirs_Cluster14	0.0142	-0.0093	0.1268	0.0596	0.1927	0.4290739	Mineral
N	nirs_Cluster2	0.0174	0.0002	0.00E+00	-0.0769	0.9824	0.9920825	Mineral
N	nirs_Cluster26	0.0007	-0.0004	0.0498	-0.0233	0.424	0.5914194	Mineral
N	nirs_Cluster28	0.0099	-0.0067	0.1435	0.0777	0.1637	0.4275837	Mineral
N	nirs_Cluster3	0.1914	-0.0091	0.0322	-0.0422	0.522	0.6615588	Mineral
N	nirs_Cluster36	0.0018	1.00E-04	0.0002	-0.0767	0.9599	0.9813987	Mineral
N	nirs_Cluster37	0.0092	-0.0061	0.0725	0.0012	0.3318	0.5256557	Mineral
N	nirs_Cluster4	0.0003	-0.0002	0.0284	-0.0463	0.5483	0.6745665	Mineral
N	nirs_Cluster50	0.0024	0.0007	0.0255	-0.0495	0.57	0.694843	Mineral

N	nirs_Cluster9	0.0376	-0.0269	0.1577	0.0929	0.1427	0.4273808	Mineral
N	norb_Cluster0	0.0848	-0.0219	0.1252	0.0579	0.1956	0.4296198	Mineral
N	norb_Cluster1	1.2746	-0.3851	0.1319	0.0651	0.1834	0.4276218	Mineral
N	norb_Cluster101	0.0037	-0.002	0.1134	0.0452	0.2198	0.447823	Mineral
N	norb_Cluster102	0.005	-0.0017	0.0746	0.0034	0.3248	0.5238414	Mineral
N	norb_Cluster103	0.0025	-0.0015	0.1143	0.0462	0.2178	0.4471191	Mineral
N	norb_Cluster104	0.0025	-0.001	0.0303	-0.0443	0.535	0.6687275	Mineral
N	norb_Cluster106	0.1468	-0.0598	0.4612	0.4198	0.0054	0.2092856	Mineral
N	norb_Cluster107	0.0163	-0.0066	0.1568	0.092	0.1439	0.4275837	Mineral
N	norb_Cluster108	0.0069	-0.0023	0.1356	0.0692	0.1768	0.4275837	Mineral
N	norb_Cluster109	0.0282	-0.0075	0.1061	0.0374	0.236	0.4657618	Mineral
N	norb_Cluster11	0.0156	-0.0057	0.1304	0.0635	0.1861	0.4285429	Mineral
N	norb_Cluster110	0.0128	-0.0049	0.1364	0.07	0.1755	0.4275837	Mineral
N	norb_Cluster112	0.0215	-0.0087	0.3943	0.3478	0.0122	0.3423957	Mineral
N	norb_Cluster113	0.0157	-0.0078	0.2786	0.2231	0.0431	0.426386	Mineral
N	norb_Cluster114	0.0035	-0.0021	0.0793	0.0085	0.3091	0.5108572	Mineral
N	norb_Cluster115	0.0034	-0.0007	0.0491	-0.0241	0.4274	0.5933859	Mineral
N	norb_Cluster116	0.0083	-0.0035	0.1141	0.0459	0.2183	0.4471191	Mineral
N	norb_Cluster117	0.0034	-0.0018	0.1158	0.0478	0.2146	0.4471191	Mineral
N	norb_Cluster118	0.0044	-0.0016	0.0805	0.0098	0.3054	0.5075993	Mineral
N	norb_Cluster119	0.0263	-0.0122	0.2264	0.1668	0.073	0.426386	Mineral
N	norb_Cluster12	0.005	-0.0018	0.0509	-0.0221	0.4188	0.5869433	Mineral
N	norb_Cluster121	0.0023	-0.001	0.0393	-0.0346	0.479	0.6304315	Mineral
N	norb_Cluster122	0.0184	-0.0068	0.1835	0.1207	0.1112	0.426386	Mineral
N	norb_Cluster123	0.0071	-0.0031	0.1375	0.0711	0.1737	0.4275837	Mineral
N	norb_Cluster124	0.7452	-0.2443	0.1661	0.1019	0.1316	0.426386	Mineral
N	norb_Cluster125	0.0052	-0.003	0.2656	0.2091	0.0493	0.426386	Mineral
N	norb_Cluster126	0.011	-0.0054	0.2598	0.2029	0.0522	0.426386	Mineral
N	norb_Cluster127	0.2041	-0.0868	0.28	0.2247	0.0425	0.426386	Mineral
N	norb_Cluster128	0.0123	-0.0047	0.1631	0.0988	0.1354	0.426386	Mineral
N	norb_Cluster13	0.0051	-0.0034	0.1427	0.0768	0.165	0.4275837	Mineral
N	norb_Cluster131	0.0209	-0.0058	0.1036	0.0347	0.242	0.4708967	Mineral
N	norb_Cluster132	1.2771	-0.4459	0.4431	0.4003	0.0068	0.234435	Mineral
N	norb_Cluster133	0.0023	-0.0003	0.0052	-0.0713	0.7976	0.8682004	Mineral
N	norb_Cluster135	0.0703	-0.0256	0.2587	0.2017	0.0529	0.426386	Mineral
N	norb_Cluster136	0.025	-0.0067	0.0979	0.0285	0.2562	0.477225	Mineral
N	norb_Cluster139	0.0391	-0.0142	0.2287	0.1693	0.0714	0.426386	Mineral
N	norb_Cluster14	0.0037	-0.0019	0.1606	0.096	0.1388	0.426386	Mineral
N	norb_Cluster140	0.0485	-0.0185	0.311	0.258	0.0308	0.426386	Mineral
N	norb_Cluster142	0.0018	-0.0011	0.0798	0.009	0.3076	0.5097562	Mineral
N	norb_Cluster144	0.0122	-0.0027	0.0651	-0.0068	0.3588	0.5483623	Mineral
N	norb_Cluster146	0.0418	-0.0109	0.1598	0.0951	0.1399	0.426386	Mineral
N	norb_Cluster15	0.0502	-0.0217	0.2442	0.1861	0.0612	0.426386	Mineral
N	norb_Cluster150	0.0199	-0.0053	0.167	0.1029	0.1305	0.426386	Mineral
N	norb_Cluster155	0.1729	-0.0548	0.183	0.1202	0.1116	0.426386	Mineral
N	norb_Cluster16	0.0165	-0.0092	0.2346	0.1758	0.0673	0.426386	Mineral
N	norb_Cluster164	0.0242	-0.0139	0.3374	0.2864	0.0232	0.426386	Mineral
N	norb_Cluster165	0.0092	-0.0057	0.2064	0.1453	0.0889	0.426386	Mineral
N	norb_Cluster166	0.0046	-0.0029	0.2015	0.1401	0.0933	0.426386	Mineral
N	norb_Cluster169	0.0309	-0.0179	0.3415	0.2908	0.0222	0.426386	Mineral
N	norb_Cluster17	0.0035	-0.0016	0.1103	0.0419	0.2265	0.4560797	Mineral
N	norb_Cluster170	0.0299	-0.0164	0.2514	0.1938	0.0569	0.426386	Mineral
N	norb_Cluster171	0.0089	-0.0056	0.1823	0.1194	0.1124	0.426386	Mineral
N	norb_Cluster172	0.0179	-0.0106	0.2562	0.199	0.0542	0.426386	Mineral
N	norb_Cluster175	0.0595	-0.0328	0.2655	0.209	0.0493	0.426386	Mineral

N	norb_Cluster176	0.0392	-0.0228	0.3052	0.2517	0.0327	0.426386	Mineral
N	norb_Cluster177	0.0361	-0.0208	0.2933	0.2389	0.0371	0.426386	Mineral
N	norb_Cluster179	0.003	-0.0004	0.0057	-0.0708	0.789	0.8604667	Mineral
N	norb_Cluster18	0.0047	-0.0017	0.1011	0.0319	0.2482	0.4735363	Mineral
N	norb_Cluster180	0.0133	-0.0016	0.0216	-0.0537	0.6015	0.7227579	Mineral
N	norb_Cluster181	0.0003	-0.0002	0.0319	-0.0425	0.524	0.6620357	Mineral
N	norb_Cluster182	0.0018	-0.0007	0.0465	-0.0269	0.4404	0.6070968	Mineral
N	norb_Cluster184	0.0175	-0.0087	0.2537	0.1963	0.0556	0.426386	Mineral
N	norb_Cluster186	0.0011	-0.0005	0.0282	-0.0466	0.55	0.6745665	Mineral
N	norb_Cluster19	0.0058	-0.0023	0.1633	0.0989	0.1353	0.426386	Mineral
N	norb_Cluster192	0.0221	-0.0135	0.3415	0.2909	0.0221	0.426386	Mineral
N	norb_Cluster193	0.0022	-0.0015	0.162	0.0975	0.137	0.426386	Mineral
N	norb_Cluster197	0.0398	-0.0218	0.0572	-0.0154	0.3908	0.5730466	Mineral
N	norb_Cluster2	0.0032	-0.0017	0.0932	0.0234	0.2686	0.4822041	Mineral
N	norb_Cluster20	0.0665	-0.0224	0.1948	0.1329	0.0995	0.426386	Mineral
N	norb_Cluster208	0.0015	-0.0011	0.1096	0.0411	0.228	0.45628	Mineral
N	norb_Cluster21	0.0542	-0.0207	0.2196	0.1596	0.0781	0.426386	Mineral
N	norb_Cluster211	0.0141	-0.007	0.0637	-0.0083	0.3642	0.5534866	Mineral
N	norb_Cluster212	0.0252	-0.0169	0.2006	0.1391	0.0941	0.426386	Mineral
N	norb_Cluster214	0.0163	-0.0075	0.0971	0.0276	0.2583	0.4779076	Mineral
N	norb_Cluster217	0.0186	-0.0105	0.136	0.0695	0.1762	0.4275837	Mineral
N	norb_Cluster219	0.0108	-0.0073	0.1408	0.0747	0.1681	0.4275837	Mineral
N	norb_Cluster22	0.0028	-0.0004	0.0041	-0.0725	0.82	0.8861144	Mineral
N	norb_Cluster220	0.0248	-0.0144	0.1073	0.0386	0.2334	0.4620957	Mineral
N	norb_Cluster221	0.0248	-0.013	0.076	0.0049	0.3201	0.5187823	Mineral
N	norb_Cluster222	0.0104	-0.0058	0.063	-0.009	0.3667	0.5534866	Mineral
N	norb_Cluster224	0.0238	-0.0159	0.1739	0.1103	0.1221	0.426386	Mineral
N	norb_Cluster225	0.0095	-0.0044	0.1344	0.0678	0.1789	0.4275837	Mineral
N	norb_Cluster226	0.0049	-0.0027	0.1322	0.0654	0.1829	0.4276218	Mineral
N	norb_Cluster228	0.0009	-0.0007	0.0383	-0.0357	0.4847	0.6312541	Mineral
N	norb_Cluster229	0.004	-0.0014	0.0611	-0.0112	0.3746	0.5581232	Mineral
N	norb_Cluster230	0.0019	-0.0009	0.071	-0.0005	0.3372	0.5277865	Mineral
N	norb_Cluster231	0.0061	-0.0019	0.0347	-0.0396	0.5064	0.6494608	Mineral
N	norb_Cluster232	0.0044	-0.001	0.0235	-0.0516	0.5856	0.7108978	Mineral
N	norb_Cluster234	0.017	-0.009	0.1879	0.1254	0.1065	0.426386	Mineral
N	norb_Cluster235	0.0012	-0.0006	0.0811	0.0104	0.3035	0.5062103	Mineral
N	norb_Cluster238	0.0135	-0.0057	0.0811	0.0104	0.3037	0.5062103	Mineral
N	norb_Cluster239	0.0109	-0.0054	0.0877	0.0175	0.2839	0.4955613	Mineral
N	norb_Cluster24	0.3014	-0.087	0.1657	0.1016	0.1321	0.426386	Mineral
N	norb_Cluster244	0.0015	-0.0004	0.0198	-0.0556	0.6166	0.7348914	Mineral
N	norb_Cluster247	0.006	-0.0012	0.0408	-0.033	0.4703	0.6303784	Mineral
N	norb_Cluster25	0.1263	-0.0436	0.1661	0.1019	0.1316	0.426386	Mineral
N	norb_Cluster250	0.0146	-0.0058	0.1656	0.1014	0.1322	0.426386	Mineral
N	norb_Cluster251	0.0064	-0.0029	0.1244	0.0571	0.1972	0.4296198	Mineral
N	norb_Cluster26	0.1134	-0.0375	0.1634	0.0991	0.1351	0.426386	Mineral
N	norb_Cluster28	0.0355	-0.0166	0.323	0.271	0.0271	0.426386	Mineral
N	norb_Cluster29	0.2454	-0.065	0.3299	0.2783	0.0251	0.426386	Mineral
N	norb_Cluster31	0.0632	-0.0143	0.0851	0.0147	0.2915	0.5024625	Mineral
N	norb_Cluster32	0.1401	-0.0413	0.1002	0.031	0.2503	0.4735363	Mineral
N	norb_Cluster33	0.434	0.0908	0.117	0.0491	0.212	0.4436039	Mineral
N	norb_Cluster35	0.0746	-0.0232	0.1582	0.0935	0.142	0.4273808	Mineral
N	norb_Cluster36	1.1002	-0.0178	0.0004	-0.0765	0.9434	0.9714023	Mineral
N	norb_Cluster37	0.7173	0.0023	0.00E+00	-0.0769	0.9863	0.9920825	Mineral
N	norb_Cluster38	0.0138	-0.0047	0.2013	0.1399	0.0934	0.426386	Mineral
N	norb_Cluster39	0.0465	-0.0152	0.1741	0.1105	0.1218	0.426386	Mineral

N	norb_Cluster40	0.2213	-0.0766	0.2099	0.1491	0.0859	0.426386	Mineral
N	norb_Cluster42	0.2825	-0.0109	0.0024	-0.0744	0.8638	0.9165838	Mineral
N	norb_Cluster44	0.0249	-0.0104	0.1883	0.1259	0.1061	0.426386	Mineral
N	norb_Cluster46	0.2575	-0.0561	0.0642	-0.0078	0.3623	0.552291	Mineral
N	norb_Cluster47	0.6453	-0.2011	0.188	0.1256	0.1064	0.426386	Mineral
N	norb_Cluster5	0.0115	-0.002	0.0432	-0.0304	0.4575	0.6193291	Mineral
N	norb_Cluster51	0.0122	-0.0066	0.1527	0.0875	0.1498	0.4275837	Mineral
N	norb_Cluster52	0.2809	-0.0937	0.1739	0.1103	0.122	0.426386	Mineral
N	norb_Cluster56	0.0178	-0.0072	0.2404	0.182	0.0635	0.426386	Mineral
N	norb_Cluster57	0.004	-0.0014	0.1285	0.0614	0.1896	0.4285429	Mineral
N	norb_Cluster59	0.0322	-0.0071	0.0383	-0.0356	0.4843	0.6312541	Mineral
N	norb_Cluster6	0.6934	-0.018	0.0012	-0.0757	0.9033	0.9363969	Mineral
N	norb_Cluster60	0.345	-0.1283	0.1096	0.0411	0.2281	0.45628	Mineral
N	norb_Cluster61	0.5486	-0.2326	0.3227	0.2706	0.0271	0.426386	Mineral
N	norb_Cluster62	0.003	0.0018	0.193	0.1309	0.1014	0.426386	Mineral
N	norb_Cluster64	0.0407	-0.0127	0.2557	0.1984	0.0545	0.426386	Mineral
N	norb_Cluster66	0.0016	-0.0008	0.0729	0.0016	0.3305	0.5256557	Mineral
N	norb_Cluster67	0.0279	-0.009	0.1528	0.0876	0.1497	0.4275837	Mineral
N	norb_Cluster68	0.003	-0.0017	0.1713	0.1075	0.1252	0.426386	Mineral
N	norb_Cluster69	0.0032	-0.0011	0.0517	-0.0212	0.4149	0.5844847	Mineral
N	norb_Cluster7	0.0786	-0.031	0.2183	0.1582	0.0791	0.426386	Mineral
N	norb_Cluster70	0.1262	-0.0426	0.2892	0.2345	0.0387	0.426386	Mineral
N	norb_Cluster71	0.1512	-0.0562	0.4265	0.3824	0.0083	0.2720524	Mineral
N	norb_Cluster74	0.0387	-0.0135	0.3053	0.2518	0.0327	0.426386	Mineral
N	norb_Cluster75	0.1531	-0.0536	0.5	0.4615	0.0032	0.1792043	Mineral
N	norb_Cluster76	0.1215	-0.0472	0.0922	0.0224	0.2712	0.4822041	Mineral
N	norb_Cluster77	0.0492	-0.0114	0.1498	0.0844	0.1541	0.4275837	Mineral
N	norb_Cluster8	0.0206	-0.0095	0.2589	0.2019	0.0527	0.426386	Mineral
N	norb_Cluster80	0.0021	-0.0014	0.1354	0.0689	0.1772	0.4275837	Mineral
N	norb_Cluster81	0.0013	-0.0007	0.0778	0.0069	0.3141	0.5146043	Mineral
N	norb_Cluster83	0.011	-0.0069	0.1383	0.072	0.1723	0.4275837	Mineral
N	norb_Cluster84	0.0093	-0.0032	0.0945	0.0249	0.265	0.4822041	Mineral
N	norb_Cluster85	0.0025	-0.0013	0.0867	0.0165	0.2867	0.4989288	Mineral
N	norb_Cluster86	0.041	-0.0248	0.1378	0.0715	0.1731	0.4275837	Mineral
N	norb_Cluster88	0.0019	-0.0012	0.1226	0.0551	0.2007	0.4302621	Mineral
N	norb_Cluster90	0.0013	-0.0007	0.0304	-0.0442	0.5342	0.6687275	Mineral
N	norb_Cluster91	0.7571	-0.3563	0.3231	0.2711	0.027	0.426386	Mineral
N	norb_Cluster92	0.0021	-0.0006	0.0146	-0.0612	0.6677	0.771853	Mineral
N	norb_Cluster93	0.0028	-0.0013	0.0518	-0.0212	0.4147	0.5844847	Mineral
N	norb_Cluster94	0.002	0.00E+00	0.0002	-0.0767	0.9574	0.9813987	Mineral
N	norb_Cluster96	0.0006	-0.0002	0.0206	-0.0547	0.6096	0.7295084	Mineral
N	norb_Cluster97	0.0162	-0.0019	0.0119	-0.0642	0.6993	0.7949202	Mineral
N	norb_Cluster98	0.0176	-0.0099	0.2078	0.1469	0.0877	0.426386	Mineral
N	nosz_Cluster0	0.0057	-0.0016	0.0628	-0.0092	0.3675	0.5534866	Mineral
N	nosz_Cluster10	0.0128	-0.0035	0.0745	0.0033	0.325	0.5238414	Mineral
N	nosz_Cluster102	0.0057	-0.0011	0.0585	-0.0139	0.385	0.5678316	Mineral
N	nosz_Cluster103	0.0496	-0.0138	0.1329	0.0662	0.1816	0.4275837	Mineral
N	nosz_Cluster105	0.0016	-0.0005	0.0289	-0.0458	0.545	0.6745665	Mineral
N	nosz_Cluster106	0.0088	-0.0032	0.0993	0.0301	0.2525	0.4735363	Mineral
N	nosz_Cluster107	0.0003	-0.0002	0.0284	-0.0463	0.5483	0.6745665	Mineral
N	nosz_Cluster108	0.0043	-0.0014	0.0462	-0.0272	0.4418	0.6075908	Mineral
N	nosz_Cluster109	0.0416	-0.0086	0.1559	0.0909	0.1453	0.4275837	Mineral
N	nosz_Cluster11	0.0104	-0.0025	0.0634	-0.0087	0.3655	0.5534866	Mineral
N	nosz_Cluster110	0.0004	0.0013	0.8474	0.8357	0.00E+00	0.0006773	Mineral
N	nosz_Cluster111	0.0078	-0.003	0.152	0.0868	0.1508	0.4275837	Mineral

N	nosz_Cluster116	0.0074	-0.0015	0.0557	-0.0169	0.397	0.5730466	Mineral
N	nosz_Cluster118	0.0064	-0.0032	0.1359	0.0695	0.1763	0.4275837	Mineral
N	nosz_Cluster119	0.0019	-0.0013	0.1627	0.0983	0.136	0.426386	Mineral
N	nosz_Cluster120	0.0083	-0.0032	0.1341	0.0674	0.1795	0.4275837	Mineral
N	nosz_Cluster123	0.0136	-0.0075	0.2773	0.2217	0.0437	0.426386	Mineral
N	nosz_Cluster128	0.005	-0.003	0.1991	0.1374	0.0955	0.426386	Mineral
N	nosz_Cluster129	0.05	-0.0088	0.1224	0.0549	0.2011	0.4302621	Mineral
N	nosz_Cluster13	0.0124	-0.0015	0.014	-0.0618	0.6742	0.7753495	Mineral
N	nosz_Cluster130	0.1138	-0.0644	0.0811	0.0104	0.3037	0.5062103	Mineral
N	nosz_Cluster132	0.004	-0.0013	0.0994	0.0302	0.2523	0.4735363	Mineral
N	nosz_Cluster14	0.0149	-0.0073	0.1522	0.087	0.1505	0.4275837	Mineral
N	nosz_Cluster16	0.008	-0.0021	0.035	-0.0392	0.5043	0.6494608	Mineral
N	nosz_Cluster17	0.0427	-0.0179	0.2899	0.2352	0.0384	0.426386	Mineral
N	nosz_Cluster18	0.0184	-0.0078	0.1257	0.0585	0.1947	0.4296198	Mineral
N	nosz_Cluster20	0.0117	-0.0067	0.1842	0.1214	0.1104	0.426386	Mineral
N	nosz_Cluster21	0.0117	-0.0068	0.2177	0.1575	0.0796	0.426386	Mineral
N	nosz_Cluster22	0.0325	-0.0168	0.1335	0.0668	0.1806	0.4275837	Mineral
N	nosz_Cluster23	0.019	-0.0108	0.263	0.2063	0.0506	0.426386	Mineral
N	nosz_Cluster24	0.0019	-0.0005	0.022	-0.0532	0.5976	0.7195523	Mineral
N	nosz_Cluster25	0.0026	-0.001	0.0548	-0.0179	0.4009	0.5730466	Mineral
N	nosz_Cluster26	0.0068	-0.0039	0.1675	0.1034	0.1298	0.426386	Mineral
N	nosz_Cluster27	0.007	-0.0028	0.1349	0.0684	0.178	0.4275837	Mineral
N	nosz_Cluster28	0.0358	-0.0142	0.1929	0.1308	0.1014	0.426386	Mineral
N	nosz_Cluster30	0.0341	-0.0142	0.2477	0.1898	0.0591	0.426386	Mineral
N	nosz_Cluster32	0.016	-0.0044	0.0886	0.0185	0.2812	0.4949814	Mineral
N	nosz_Cluster33	0.0187	-0.0101	0.2191	0.159	0.0785	0.426386	Mineral
N	nosz_Cluster34	0.0387	-0.0228	0.2061	0.1451	0.0891	0.426386	Mineral
N	nosz_Cluster35	0.0097	-0.0045	0.2013	0.1398	0.0935	0.426386	Mineral
N	nosz_Cluster36	0.0213	-0.0101	0.2395	0.1811	0.0641	0.426386	Mineral
N	nosz_Cluster37	0.0077	-0.0031	0.1178	0.05	0.2104	0.4416616	Mineral
N	nosz_Cluster38	0.0144	-0.0071	0.1273	0.0602	0.1918	0.4285429	Mineral
N	nosz_Cluster39	0.0653	-0.0271	0.2678	0.2115	0.0482	0.426386	Mineral
N	nosz_Cluster40	0.0262	-0.0095	0.1221	0.0546	0.2017	0.4302621	Mineral
N	nosz_Cluster41	0.0258	-0.0102	0.144	0.0781	0.1631	0.4275837	Mineral
N	nosz_Cluster42	0.03	-0.0151	0.2374	0.1787	0.0655	0.426386	Mineral
N	nosz_Cluster43	0.001	-0.0007	0.058	-0.0145	0.3874	0.5699818	Mineral
N	nosz_Cluster44	0.0068	-0.0021	0.0623	-0.0098	0.3696	0.5534866	Mineral
N	nosz_Cluster45	0.0056	-0.0022	0.1001	0.0309	0.2506	0.4735363	Mineral
N	nosz_Cluster46	0.0082	-0.003	0.1834	0.1206	0.1112	0.426386	Mineral
N	nosz_Cluster47	0.0012	-0.0006	0.0784	0.0076	0.312	0.5141826	Mineral
N	nosz_Cluster48	0.0028	-0.0017	0.1117	0.0433	0.2235	0.45156	Mineral
N	nosz_Cluster49	0.0012	-0.0004	0.0189	-0.0566	0.6253	0.7393832	Mineral
N	nosz_Cluster5	0.004	-0.0024	0.1274	0.0603	0.1916	0.4285429	Mineral
N	nosz_Cluster51	0.003	-0.0011	0.0707	-0.0008	0.3383	0.5277865	Mineral
N	nosz_Cluster53	0.0012	-0.0003	0.0072	-0.0692	0.7638	0.840709	Mineral
N	nosz_Cluster54	0.0037	-0.0011	0.0334	-0.041	0.5144	0.6541238	Mineral
N	nosz_Cluster57	0.2232	-0.0763	0.1617	0.0972	0.1373	0.426386	Mineral
N	nosz_Cluster58	0.0053	-0.0036	0.1984	0.1368	0.0961	0.426386	Mineral
N	nosz_Cluster59	0.0008	-0.0006	0.0344	-0.0399	0.5081	0.6502929	Mineral
N	nosz_Cluster6	0.0034	-0.0018	0.0967	0.0272	0.2592	0.4779076	Mineral
N	nosz_Cluster60	0.0099	-0.0034	0.0843	0.0139	0.2938	0.5024625	Mineral
N	nosz_Cluster61	0.0178	-0.0057	0.2446	0.1865	0.0609	0.426386	Mineral
N	nosz_Cluster62	0.0493	-0.0175	0.1452	0.0795	0.1611	0.4275837	Mineral
N	nosz_Cluster64	0.0023	0.0004	0.0049	-0.0716	0.804	0.8704201	Mineral
N	nosz_Cluster65	0.0083	-0.0025	0.0657	-0.0062	0.3566	0.5464169	Mineral

N	nosz_Cluster66	0.0428	-0.0135	0.0929	0.0232	0.2692	0.4822041	Mineral
N	nosz_Cluster67	0.0669	-0.0248	0.1301	0.0631	0.1867	0.4285429	Mineral
N	nosz_Cluster69	0.008	-0.0044	0.2634	0.2067	0.0504	0.426386	Mineral
N	nosz_Cluster70	0.0655	-0.0365	0.1186	0.0508	0.2088	0.4415279	Mineral
N	nosz_Cluster71	0.0101	-0.0053	0.1377	0.0714	0.1732	0.4275837	Mineral
N	nosz_Cluster72	0.066	-0.0253	0.1745	0.1111	0.1212	0.426386	Mineral
N	nosz_Cluster73	0.0031	-0.0012	0.0627	-0.0094	0.3679	0.5534866	Mineral
N	nosz_Cluster74	0.0017	-0.0011	0.1148	0.0467	0.2167	0.4471191	Mineral
N	nosz_Cluster75	0.132	-0.0805	0.0771	0.0061	0.3164	0.5146043	Mineral
N	nosz_Cluster77	0.0002	1.00E-04	0.0106	-0.0655	0.7151	0.8052051	Mineral
N	nosz_Cluster78	0.0054	-0.0025	0.173	0.1094	0.123	0.426386	Mineral
N	nosz_Cluster79	0.0401	-0.0112	0.1252	0.0579	0.1957	0.4296198	Mineral
N	nosz_Cluster8	0.0106	-0.0045	0.2977	0.2437	0.0354	0.426386	Mineral
N	nosz_Cluster80	0.0039	-0.0013	0.0601	-0.0122	0.3785	0.5610456	Mineral
N	nosz_Cluster81	0.0099	-0.0053	0.1047	0.0358	0.2395	0.4679294	Mineral
N	nosz_Cluster82	0.0023	0.0005	0.0051	-0.0715	0.8011	0.8689019	Mineral
N	nosz_Cluster83	0.0014	-0.0011	0.0706	-0.0009	0.3385	0.5277865	Mineral
N	nosz_Cluster85	0.0027	-0.0003	0.0032	-0.0735	0.842	0.9049312	Mineral
N	nosz_Cluster86	0.0038	-0.0009	0.027	-0.0479	0.5587	0.6824396	Mineral
N	nosz_Cluster87	0.0034	-0.0009	0.0352	-0.039	0.5033	0.6494608	Mineral
N	nosz_Cluster88	0.0246	-0.0077	0.2492	0.1914	0.0582	0.426386	Mineral
N	nosz_Cluster89	0.0607	-0.0179	0.1027	0.0337	0.2442	0.4708967	Mineral
N	nosz_Cluster9	0.0034	-0.0016	0.0726	0.0012	0.3316	0.5256557	Mineral
N	nosz_Cluster90	0.0139	-0.0031	0.102	0.0329	0.2459	0.4726442	Mineral
N	nosz_Cluster91	0.0232	-0.0057	0.0479	-0.0253	0.4333	0.6000813	Mineral
N	nosz_Cluster93	0.0488	-0.0189	0.2013	0.1399	0.0934	0.426386	Mineral
N	nosz_Cluster95	0.0094	-0.0022	0.0608	-0.0114	0.3756	0.5581232	Mineral
N	nosz_Cluster96	0.0064	-0.0011	0.0403	-0.0336	0.4733	0.6303784	Mineral
N	nosz_Cluster97	0.0026	-0.0009	0.0459	-0.0275	0.4433	0.6082364	Mineral
N	nosz_Cluster98	0.0035	0.0005	0.0022	-0.0745	0.867	0.9169314	Mineral
N	nosz_Cluster99	0.0061	-0.0011	0.039	-0.0349	0.4803	0.6304315	Mineral
N	nrfa_Cluster10	0.0978	-0.0484	0.2127	0.1522	0.0835	0.426386	Mineral
N	nrfa_Cluster100	0.1249	-0.0694	0.1906	0.1284	0.1037	0.426386	Mineral
N	nrfa_Cluster104	0.1478	-0.0508	0.1986	0.1369	0.096	0.426386	Mineral
N	nrfa_Cluster105	0.006	-0.0034	0.1656	0.1014	0.1322	0.426386	Mineral
N	nrfa_Cluster109	0.2753	-0.0842	0.1756	0.1122	0.12	0.426386	Mineral
N	nrfa_Cluster116	0.3208	-0.0986	0.3298	0.2782	0.0252	0.426386	Mineral
N	nrfa_Cluster118	0.2298	-0.1331	0.1767	0.1134	0.1187	0.426386	Mineral
N	nrfa_Cluster125	0.1081	-0.0609	0.1488	0.0834	0.1555	0.4275837	Mineral
N	nrfa_Cluster154	0.0837	-0.0443	0.1846	0.1219	0.1099	0.426386	Mineral
N	nrfa_Cluster164	0.0044	-0.0022	0.1512	0.0859	0.152	0.4275837	Mineral
N	nrfa_Cluster172	0.0036	-0.0023	0.1373	0.071	0.1739	0.4275837	Mineral
N	nrfa_Cluster175	0.0053	-0.0038	0.1356	0.0692	0.1768	0.4275837	Mineral
N	nrfa_Cluster20	0.045	-0.0172	0.1234	0.0559	0.1993	0.4302621	Mineral
N	nrfa_Cluster26	0.1433	-0.0834	0.1999	0.1384	0.0947	0.426386	Mineral
N	nrfa_Cluster29	0.0059	-0.0024	0.0741	0.0029	0.3262	0.5243918	Mineral
N	nrfa_Cluster34	0.0642	-0.0251	0.1347	0.0681	0.1785	0.4275837	Mineral
N	nrfa_Cluster38	0.0033	-0.0023	0.13	0.0631	0.1867	0.4285429	Mineral
N	nrfa_Cluster61	0.0047	-0.002	0.083	0.0125	0.2977	0.5047519	Mineral
N	nrfa_Cluster62	0.0589	-0.0357	0.1677	0.1036	0.1296	0.426386	Mineral
N	nrfa_Cluster66	0.1282	-0.0441	0.273	0.217	0.0457	0.426386	Mineral
N	nrfa_Cluster69	0.0035	-0.002	0.1349	0.0684	0.1781	0.4275837	Mineral
N	nrfa_Cluster71	0.0322	-0.0161	0.1394	0.0732	0.1705	0.4275837	Mineral
N	nrfa_Cluster72	0.0969	-0.0464	0.182	0.1191	0.1128	0.426386	Mineral
N	nrfa_Cluster73	0.0042	-0.0028	0.1478	0.0822	0.1572	0.4275837	Mineral

N	nrfa_Cluster74	0.1692	-0.0863	0.2006	0.1392	0.094	0.426386	Mineral
N	nrfa_Cluster76	0.0922	-0.0554	0.2212	0.1613	0.0769	0.426386	Mineral
N	nrfa_Cluster79	0.0138	-0.0097	0.1047	0.0358	0.2395	0.4679294	Mineral
N	nrfa_Cluster87	0.0207	-0.0137	0.1487	0.0832	0.1558	0.4275837	Mineral
N	nrfa_Cluster89	0.0023	-0.0016	0.1335	0.0668	0.1806	0.4275837	Mineral
N	nrfa_Cluster92	0.0041	-0.0025	0.2405	0.182	0.0635	0.426386	Mineral
N	nrfa_Cluster93	0.0581	-0.0323	0.1286	0.0616	0.1893	0.4285429	Mineral
N	nrfa_Cluster94	0.0003	-0.0002	0.0383	-0.0357	0.4847	0.6312541	Mineral
N	nrfa_Cluster97	0.0225	-0.0105	0.2432	0.1849	0.0618	0.426386	Mineral
N	nrfa_Cluster99	0.0184	-0.0077	0.1514	0.0862	0.1517	0.4275837	Mineral
CN	amoA_A_Cluster26	0.0053	-0.0002	0.0004	-0.0764	0.9407	0.9983781	Mineral
CN	amoA_A_Cluster45	0.0071	-0.0006	0.0044	-0.0722	0.8145	0.9983781	Mineral
CN	amoA_B_Cluster0	0.1272	-0.0134	0.0918	0.0219	0.2725	0.9983781	Mineral
CN	amoA_B_Cluster1	0.0205	-0.0004	0.0005	-0.0764	0.9378	0.9983781	Mineral
CN	amoA_B_Cluster10	0.0539	-0.0163	0.3941	0.3475	0.0122	0.3795337	Mineral
CN	amoA_B_Cluster11	0.0486	-0.0072	0.0564	-0.0162	0.3941	0.9983781	Mineral
CN	amoA_B_Cluster12	0.093	-0.0246	0.4223	0.3778	0.0087	0.3435905	Mineral
CN	amoA_B_Cluster13	0.6417	-0.2825	0.5996	0.5688	0.0007	0.1126225	Mineral
CN	amoA_B_Cluster14	0.0339	-0.0147	0.2756	0.2199	0.0445	0.7502017	Mineral
CN	amoA_B_Cluster15	0.0413	-0.0165	0.3956	0.3491	0.012	0.3795337	Mineral
CN	amoA_B_Cluster2	0.2143	-0.0901	0.581	0.5488	0.001	0.1126225	Mineral
CN	amoA_B_Cluster20	0.0078	0.0006	0.0037	-0.0729	0.8294	0.9983781	Mineral
CN	amoA_B_Cluster23	0.0626	-0.0147	0.2099	0.1491	0.0859	0.9983781	Mineral
CN	amoA_B_Cluster3	0.0244	-0.0099	0.2387	0.1801	0.0646	0.9738609	Mineral
CN	amoA_B_Cluster4	0.0169	-0.0089	0.3375	0.2865	0.0231	0.514492	Mineral
CN	amoA_B_Cluster5	0.1032	-0.021	0.2834	0.2282	0.0411	0.7344159	Mineral
CN	amoA_B_Cluster6	0.0279	-0.0038	0.0503	-0.0228	0.4217	0.9983781	Mineral
CN	amoA_B_Cluster7	0.0232	-0.0012	0.0031	-0.0736	0.8431	0.9983781	Mineral
CN	amoA_B_Cluster8	0.0268	-0.0059	0.0586	-0.0138	0.3848	0.9983781	Mineral
CN	amoA_B_Cluster9	0.0693	-0.0211	0.1678	0.1038	0.1295	0.9983781	Mineral
CN	nifh_Cluster0	0.0318	0.0093	0.2888	0.234	0.0389	0.7344159	Mineral
CN	nifh_Cluster10	0.0033	0.0005	0.0109	-0.0652	0.711	0.9983781	Mineral
CN	nifh_Cluster100	0.002	0.0007	0.037	-0.0371	0.4923	0.9983781	Mineral
CN	nifh_Cluster103	0.0433	-0.0155	0.3514	0.3015	0.0199	0.5095401	Mineral
CN	nifh_Cluster1034	0.0337	-0.0067	0.041	-0.0328	0.4693	0.9983781	Mineral
CN	nifh_Cluster105	0.0164	0.0002	1.00E-04	-0.0768	0.9734	0.9983781	Mineral
CN	nifh_Cluster108	0.0076	0.0004	0.0021	-0.0746	0.8706	0.9983781	Mineral
CN	nifh_Cluster1099	0.0478	0.0308	0.241	0.1826	0.0631	0.9738609	Mineral
CN	nifh_Cluster11	0.0025	0.0004	0.0118	-0.0642	0.6995	0.9983781	Mineral
CN	nifh_Cluster1112	0.0037	-0.0008	0.0212	-0.0541	0.6045	0.9983781	Mineral
CN	nifh_Cluster112	0.0098	0.0084	0.4167	0.3719	0.0093	0.3445416	Mineral
CN	nifh_Cluster113	0.0374	0.0041	0.0399	-0.0339	0.4753	0.9983781	Mineral
CN	nifh_Cluster114	0.0304	0.0014	0.0108	-0.0653	0.7124	0.9983781	Mineral
CN	nifh_Cluster1141	0.0296	-0.0019	0.0058	-0.0707	0.787	0.9983781	Mineral
CN	nifh_Cluster115	0.0098	-0.0007	0.0075	-0.0688	0.7583	0.9983781	Mineral
CN	nifh_Cluster116	0.0883	-0.0052	0.0122	-0.0638	0.6952	0.9983781	Mineral
CN	nifh_Cluster1163	0.0051	-0.0005	0.0073	-0.0691	0.7625	0.9983781	Mineral
CN	nifh_Cluster1179	0.0037	-0.0008	0.0181	-0.0574	0.6326	0.9983781	Mineral
CN	nifh_Cluster1182	0.0209	0.0008	0.0056	-0.0709	0.7906	0.9983781	Mineral
CN	nifh_Cluster1183	0.0045	0.0021	0.2365	0.1778	0.066	0.9738609	Mineral
CN	nifh_Cluster1197	0.0115	-0.0028	0.0638	-0.0082	0.3637	0.9983781	Mineral
CN	nifh_Cluster1199	0.0048	0.002	0.116	0.048	0.2141	0.9983781	Mineral
CN	nifh_Cluster120	0.02	0.0063	0.0383	-0.0357	0.4845	0.9983781	Mineral
CN	nifh_Cluster1203	0.0217	-0.0012	0.0036	-0.073	0.8319	0.9983781	Mineral
CN	nifh_Cluster1206	0.006	-0.0014	0.0613	-0.0109	0.3736	0.9983781	Mineral

CN	nifh_Cluster1207	0.0078	-0.001	0.0122	-0.0638	0.6956	0.9983781	Mineral
CN	nifh_Cluster1209	0.1617	-0.0125	0.0413	-0.0325	0.4678	0.9983781	Mineral
CN	nifh_Cluster121	0.0123	0.0018	0.0089	-0.0674	0.7386	0.9983781	Mineral
CN	nifh_Cluster1211	0.0068	0.0002	0.0006	-0.0762	0.9283	0.9983781	Mineral
CN	nifh_Cluster1213	0.0032	0.0003	0.0058	-0.0707	0.7873	0.9983781	Mineral
CN	nifh_Cluster122	0.0041	0.0005	0.0088	-0.0675	0.74	0.9983781	Mineral
CN	nifh_Cluster123	0.0092	0.0014	0.0106	-0.0655	0.7152	0.9983781	Mineral
CN	nifh_Cluster1236	0.031	-0.0007	0.0002	-0.0767	0.9557	0.9983781	Mineral
CN	nifh_Cluster1238	0.0034	0.0014	0.0736	0.0023	0.3281	0.9983781	Mineral
CN	nifh_Cluster124	0.0101	0.0005	0.0036	-0.073	0.8316	0.9983781	Mineral
CN	nifh_Cluster1261	0.0023	-0.0005	0.0114	-0.0647	0.705	0.9983781	Mineral
CN	nifh_Cluster1262	0.008	-0.0012	0.0168	-0.0589	0.6455	0.9983781	Mineral
CN	nifh_Cluster1266	0.0072	-0.0012	0.0244	-0.0506	0.5782	0.9983781	Mineral
CN	nifh_Cluster1267	0.0388	0.0334	0.3393	0.2885	0.0227	0.514492	Mineral
CN	nifh_Cluster127	0.0033	0.0014	0.0546	-0.0181	0.4019	0.9983781	Mineral
CN	nifh_Cluster1278	0.2105	-0.0023	0.0004	-0.0765	0.9451	0.9983781	Mineral
CN	nifh_Cluster128	0.0043	0.00E+00	0.00E+00	-0.0769	0.989	0.9983781	Mineral
CN	nifh_Cluster1289	0.004	-0.0005	0.0119	-0.0641	0.6986	0.9983781	Mineral
CN	nifh_Cluster129	0.0086	0.0034	0.0786	0.0077	0.3115	0.9983781	Mineral
CN	nifh_Cluster1292	0.0106	0.0019	0.0306	-0.044	0.5332	0.9983781	Mineral
CN	nifh_Cluster130	0.0071	-0.0002	0.0003	-0.0766	0.9479	0.9983781	Mineral
CN	nifh_Cluster1305	0.0048	0.0003	0.0045	-0.072	0.8112	0.9983781	Mineral
CN	nifh_Cluster1306	0.0055	0.0015	0.0575	-0.015	0.3893	0.9983781	Mineral
CN	nifh_Cluster131	0.0067	0.0009	0.0076	-0.0687	0.7567	0.9983781	Mineral
CN	nifh_Cluster1319	0.0048	-0.0025	0.1468	0.0811	0.1587	0.9983781	Mineral
CN	nifh_Cluster132	0.0064	0.00E+00	0.00E+00	-0.0769	0.9863	0.9983781	Mineral
CN	nifh_Cluster1320	0.0028	0.0005	0.0076	-0.0688	0.7578	0.9983781	Mineral
CN	nifh_Cluster1323	0.0022	-0.0011	0.1347	0.0681	0.1785	0.9983781	Mineral
CN	nifh_Cluster1324	0.0235	0.0052	0.0476	-0.0257	0.4347	0.9983781	Mineral
CN	nifh_Cluster1336	0.0159	0.001	0.0119	-0.0641	0.6982	0.9983781	Mineral
CN	nifh_Cluster1340	0.0478	-0.0111	0.0393	-0.0346	0.4786	0.9983781	Mineral
CN	nifh_Cluster1345	0.033	0.0235	0.2686	0.2124	0.0478	0.7832341	Mineral
CN	nifh_Cluster1370	0.0022	0.0003	0.0061	-0.0703	0.7818	0.9983781	Mineral
CN	nifh_Cluster1375	0.0017	0.0012	0.2192	0.1591	0.0784	0.9983781	Mineral
CN	nifh_Cluster139	0.0038	0.0002	0.0007	-0.0761	0.9232	0.9983781	Mineral
CN	nifh_Cluster140	0.0051	0.0011	0.0341	-0.0402	0.5102	0.9983781	Mineral
CN	nifh_Cluster141	0.0808	-0.008	0.0878	0.0176	0.2837	0.9983781	Mineral
CN	nifh_Cluster148	0.006	-0.0023	0.0558	-0.0168	0.3965	0.9983781	Mineral
CN	nifh_Cluster1480	0.0006	0.001	0.386	0.3387	0.0134	0.3963807	Mineral
CN	nifh_Cluster152	0.0602	0.0049	0.0156	-0.0601	0.6577	0.9983781	Mineral
CN	nifh_Cluster156	0.0459	0.0196	0.3815	0.3339	0.0141	0.3974991	Mineral
CN	nifh_Cluster1562	0.0293	0.0329	0.461	0.4195	0.0054	0.26818	Mineral
CN	nifh_Cluster158	0.019	-0.0045	0.0606	-0.0117	0.3766	0.9983781	Mineral
CN	nifh_Cluster16	0.0023	-0.0007	0.0233	-0.0519	0.5872	0.9983781	Mineral
CN	nifh_Cluster166	0.0165	0.0054	0.043	-0.0307	0.4586	0.9983781	Mineral
CN	nifh_Cluster205	0.0121	-0.0005	0.0028	-0.0739	0.8512	0.9983781	Mineral
CN	nifh_Cluster21	0.0022	-0.0012	0.134	0.0674	0.1796	0.9983781	Mineral
CN	nifh_Cluster222	0.0069	0.002	0.0292	-0.0455	0.5426	0.9983781	Mineral
CN	nifh_Cluster225	0.021	0.0016	0.0138	-0.0621	0.6772	0.9983781	Mineral
CN	nifh_Cluster230	0.0052	0.0021	0.1419	0.0759	0.1664	0.9983781	Mineral
CN	nifh_Cluster231	0.0019	0.0005	0.0216	-0.0537	0.6015	0.9983781	Mineral
CN	nifh_Cluster236	0.0034	-0.001	0.0644	-0.0076	0.3615	0.9983781	Mineral
CN	nifh_Cluster237	0.007	0.0006	0.0074	-0.0689	0.7603	0.9983781	Mineral
CN	nifh_Cluster243	0.0253	0.0068	0.0492	-0.0239	0.4267	0.9983781	Mineral
CN	nifh_Cluster2432	0.0317	0.0267	0.4009	0.3548	0.0113	0.3795337	Mineral

CN	nifh_Cluster2433	0.0028	0.0004	0.0045	-0.0721	0.8127	0.9983781	Mineral
CN	nifh_Cluster246	0.0068	0.0013	0.0196	-0.0558	0.6187	0.9983781	Mineral
CN	nifh_Cluster25	0.0195	-0.0005	0.002	-0.0747	0.8733	0.9983781	Mineral
CN	nifh_Cluster256	0.0023	-0.0003	0.0074	-0.069	0.761	0.9983781	Mineral
CN	nifh_Cluster264	0.0025	0.0001	0.0012	-0.0756	0.9023	0.9983781	Mineral
CN	nifh_Cluster265	0.0702	0.0186	0.4377	0.3944	0.0072	0.3045088	Mineral
CN	nifh_Cluster267	0.0071	0.0007	0.0102	-0.066	0.7206	0.9983781	Mineral
CN	nifh_Cluster268	0.0109	0.0048	0.4664	0.4254	0.005	0.26818	Mineral
CN	nifh_Cluster269	0.0283	0.008	0.0753	0.0042	0.3223	0.9983781	Mineral
CN	nifh_Cluster272	0.0012	0.0011	0.1986	0.1369	0.096	0.9983781	Mineral
CN	nifh_Cluster274	0.0014	0.0002	0.0025	-0.0742	0.8584	0.9983781	Mineral
CN	nifh_Cluster275	0.0051	-0.0003	0.0054	-0.0711	0.7947	0.9983781	Mineral
CN	nifh_Cluster278	0.0056	0.0047	0.4523	0.4102	0.006	0.2728127	Mineral
CN	nifh_Cluster279	0.0058	0.0022	0.1256	0.0583	0.1949	0.9983781	Mineral
CN	nifh_Cluster28	0.0017	0.0004	0.0164	-0.0593	0.6494	0.9983781	Mineral
CN	nifh_Cluster280	0.0034	-0.0004	0.0027	-0.074	0.8541	0.9983781	Mineral
CN	nifh_Cluster282	0.0052	-0.0004	0.0059	-0.0706	0.7855	0.9983781	Mineral
CN	nifh_Cluster287	0.003	0.0008	0.0481	-0.0251	0.4321	0.9983781	Mineral
CN	nifh_Cluster290	0.0096	0.0004	0.0009	-0.076	0.9165	0.9983781	Mineral
CN	nifh_Cluster297	0.004	-0.0007	0.0085	-0.0678	0.744	0.9983781	Mineral
CN	nifh_Cluster299	0.0025	0.0009	0.054	-0.0188	0.4046	0.9983781	Mineral
CN	nifh_Cluster303	0.002	0.0016	0.2939	0.2396	0.0368	0.7344159	Mineral
CN	nifh_Cluster304	0.0025	0.0005	0.0171	-0.0585	0.6424	0.9983781	Mineral
CN	nifh_Cluster313	0.0129	0.0036	0.0331	-0.0413	0.5163	0.9983781	Mineral
CN	nifh_Cluster314	0.0027	0.0018	0.1973	0.1356	0.0972	0.9983781	Mineral
CN	nifh_Cluster317	0.0089	-0.0007	0.0058	-0.0706	0.7867	0.9983781	Mineral
CN	nifh_Cluster32	0.0029	0.0004	0.0103	-0.0658	0.7186	0.9983781	Mineral
CN	nifh_Cluster320	13.944	-0.522	0.0389	-0.035	0.481	0.9983781	Mineral
CN	nifh_Cluster334	0.0084	0.0036	0.0642	-0.0077	0.362	0.9983781	Mineral
CN	nifh_Cluster335	0.0059	0.0013	0.0181	-0.0574	0.6322	0.9983781	Mineral
CN	nifh_Cluster338	0.0151	-0.0018	0.0221	-0.0531	0.5966	0.9983781	Mineral
CN	nifh_Cluster340	0.0041	-0.0005	0.012	-0.064	0.6972	0.9983781	Mineral
CN	nifh_Cluster341	0.0021	0.0016	0.1812	0.1182	0.1137	0.9983781	Mineral
CN	nifh_Cluster35	0.0079	0.0005	0.0049	-0.0716	0.8038	0.9983781	Mineral
CN	nifh_Cluster351	0.0035	0.0013	0.0571	-0.0155	0.3913	0.9983781	Mineral
CN	nifh_Cluster3574	0.1191	-0.0281	0.1998	0.1382	0.0948	0.9983781	Mineral
CN	nifh_Cluster3691	0.0224	-0.0024	0.0097	-0.0665	0.7273	0.9983781	Mineral
CN	nifh_Cluster37	0.0374	0.0043	0.011	-0.0651	0.7105	0.9983781	Mineral
CN	nifh_Cluster374	0.0029	-0.0006	0.0183	-0.0572	0.6303	0.9983781	Mineral
CN	nifh_Cluster38	0.0008	0.0003	0.0235	-0.0516	0.5851	0.9983781	Mineral
CN	nifh_Cluster382	0.0023	-0.0003	0.0036	-0.0731	0.8328	0.9983781	Mineral
CN	nifh_Cluster384	0.0023	0.0005	0.0222	-0.053	0.596	0.9983781	Mineral
CN	nifh_Cluster386	0.0041	0.0018	0.063	-0.0091	0.3669	0.9983781	Mineral
CN	nifh_Cluster389	0.003	0.001	0.0256	-0.0494	0.5691	0.9983781	Mineral
CN	nifh_Cluster394	0.0027	0.0008	0.0192	-0.0563	0.6226	0.9983781	Mineral
CN	nifh_Cluster40	0.0014	-0.0002	0.0056	-0.0709	0.7905	0.9983781	Mineral
CN	nifh_Cluster43	0.0107	0.0033	0.1432	0.0773	0.1643	0.9983781	Mineral
CN	nifh_Cluster434	0.0048	0.0011	0.0295	-0.0451	0.5403	0.9983781	Mineral
CN	nifh_Cluster440	0.0171	-0.0003	0.0003	-0.0767	0.9552	0.9983781	Mineral
CN	nifh_Cluster462	0.0048	0.0016	0.0632	-0.0088	0.366	0.9983781	Mineral
CN	nifh_Cluster470	0.017	0.0018	0.0145	-0.0613	0.6692	0.9983781	Mineral
CN	nifh_Cluster49	0.0223	0.0011	0.0011	-0.0757	0.9047	0.9983781	Mineral
CN	nifh_Cluster499	0.0028	0.0004	0.0076	-0.0688	0.7579	0.9983781	Mineral
CN	nifh_Cluster5	0.0263	0.0044	0.0967	0.0272	0.2593	0.9983781	Mineral
CN	nifh_Cluster506	0.0058	0.002	0.089	0.0189	0.2802	0.9983781	Mineral

CN	nifh_Cluster51	0.0031	0.00E+00	1.00E-04	-0.0769	0.9792	0.9983781	Mineral
CN	nifh_Cluster52	0.0396	0.0071	0.0212	-0.0541	0.6048	0.9983781	Mineral
CN	nifh_Cluster525	0.0025	-0.0001	0.0008	-0.0761	0.9204	0.9983781	Mineral
CN	nifh_Cluster531	0.0129	0.0073	0.2175	0.1573	0.0797	0.9983781	Mineral
CN	nifh_Cluster539	0.0056	0.0024	0.0351	-0.0391	0.5035	0.9983781	Mineral
CN	nifh_Cluster578	0.0593	0.0114	0.094	0.0243	0.2663	0.9983781	Mineral
CN	nifh_Cluster58	0.2389	0.2087	0.4599	0.4184	0.0055	0.26818	Mineral
CN	nifh_Cluster60	0.011	0.0017	0.0178	-0.0578	0.6356	0.9983781	Mineral
CN	nifh_Cluster61	0.0017	0.0005	0.0438	-0.0297	0.4539	0.9983781	Mineral
CN	nifh_Cluster65	0.0085	0.0023	0.0559	-0.0168	0.3964	0.9983781	Mineral
CN	nifh_Cluster686	0.0013	0.0002	0.0074	-0.069	0.7607	0.9983781	Mineral
CN	nifh_Cluster69	0.0092	0.0003	0.0004	-0.0764	0.9407	0.9983781	Mineral
CN	nifh_Cluster70	0.0144	0.0045	0.0358	-0.0384	0.4994	0.9983781	Mineral
CN	nifh_Cluster717	0.0024	0.0004	0.0122	-0.0638	0.6951	0.9983781	Mineral
CN	nifh_Cluster725	0.0035	0.0015	0.1429	0.077	0.1647	0.9983781	Mineral
CN	nifh_Cluster727	0.0038	0.00E+00	1.00E-04	-0.0768	0.9761	0.9983781	Mineral
CN	nifh_Cluster73	0.0133	-0.0011	0.0053	-0.0712	0.7962	0.9983781	Mineral
CN	nifh_Cluster74	0.005	-0.0003	0.0017	-0.0751	0.8841	0.9983781	Mineral
CN	nifh_Cluster748	0.0104	-0.0009	0.0172	-0.0584	0.6415	0.9983781	Mineral
CN	nifh_Cluster749	0.0055	0.0005	0.0056	-0.0709	0.7912	0.9983781	Mineral
CN	nifh_Cluster755	0.0118	-0.0032	0.0692	-0.0024	0.3436	0.9983781	Mineral
CN	nifh_Cluster76	0.0033	-0.0013	0.0725	0.0011	0.3319	0.9983781	Mineral
CN	nifh_Cluster79	0.002	0.0003	0.0089	-0.0673	0.7378	0.9983781	Mineral
CN	nifh_Cluster81	0.0038	0.0015	0.0614	-0.0108	0.3731	0.9983781	Mineral
CN	nifh_Cluster82	0.0014	-0.0004	0.0167	-0.059	0.6465	0.9983781	Mineral
CN	nifh_Cluster83	0.0055	-0.0017	0.0594	-0.013	0.3815	0.9983781	Mineral
CN	nifh_Cluster86	0.0161	-0.0031	0.0494	-0.0237	0.426	0.9983781	Mineral
CN	nifh_Cluster868	0.0098	0.0027	0.0821	0.0114	0.3006	0.9983781	Mineral
CN	nifh_Cluster87	0.003	0.0013	0.0732	0.0019	0.3294	0.9983781	Mineral
CN	nifh_Cluster88	0.0054	-0.0005	0.0052	-0.0713	0.7987	0.9983781	Mineral
CN	nifh_Cluster9	0.0077	0.0004	0.0017	-0.0751	0.884	0.9983781	Mineral
CN	nifh_Cluster92	0.0068	0.0026	0.0535	-0.0193	0.4067	0.9983781	Mineral
CN	nifh_Cluster93	0.0035	0.0004	0.0074	-0.0689	0.76	0.9983781	Mineral
CN	nifh_Cluster94	0.005	0.0005	0.0043	-0.0723	0.8163	0.9983781	Mineral
CN	nifh_Cluster95	0.0149	0.0031	0.0233	-0.0519	0.5874	0.9983781	Mineral
CN	nifh_Cluster96	0.0131	0.0025	0.0329	-0.0414	0.5174	0.9983781	Mineral
CN	nifh_Cluster97	0.0125	0.0024	0.1043	0.0354	0.2403	0.9983781	Mineral
CN	nifh_Cluster98	0.0049	0.0005	0.0083	-0.0679	0.7461	0.9983781	Mineral
CN	nifh_Cluster99	0.0035	0.0009	0.0222	-0.053	0.5961	0.9983781	Mineral
CN	nirk_Cluster0	6.5686	-0.4498	0.2106	0.1499	0.0853	0.9983781	Mineral
CN	nirk_Cluster1	0.2105	-0.092	0.5928	0.5615	0.0008	0.1126225	Mineral
CN	nirk_Cluster10	0.6649	-0.0259	0.0121	-0.0639	0.6959	0.9983781	Mineral
CN	nirk_Cluster101	0.004	0.0018	0.1092	0.0407	0.229	0.9983781	Mineral
CN	nirk_Cluster102	0.055	-0.0018	0.0025	-0.0742	0.8594	0.9983781	Mineral
CN	nirk_Cluster103	0.09	-0.0171	0.1077	0.0391	0.2323	0.9983781	Mineral
CN	nirk_Cluster104	0.0028	-0.0008	0.0286	-0.0461	0.5468	0.9983781	Mineral
CN	nirk_Cluster105	0.1172	-0.0025	0.0013	-0.0755	0.899	0.9983781	Mineral
CN	nirk_Cluster106	0.073	-0.0063	0.0098	-0.0664	0.7255	0.9983781	Mineral
CN	nirk_Cluster107	0.1123	-0.0037	0.0027	-0.074	0.8535	0.9983781	Mineral
CN	nirk_Cluster108	0.0184	0.0002	0.0002	-0.0767	0.9596	0.9983781	Mineral
CN	nirk_Cluster109	0.0158	0.0005	0.0017	-0.0751	0.8828	0.9983781	Mineral
CN	nirk_Cluster11	0.1509	0.0054	0.0287	-0.046	0.5462	0.9983781	Mineral
CN	nirk_Cluster12	0.1052	-0.006	0.019	-0.0565	0.6243	0.9983781	Mineral
CN	nirk_Cluster13	0.0418	0.0017	0.0111	-0.0649	0.7084	0.9983781	Mineral
CN	nirk_Cluster14	0.1084	-0.009	0.074	0.0027	0.3268	0.9983781	Mineral

CN	nirk_Cluster15	0.4141	-0.0339	0.0202	-0.0551	0.613	0.9983781	Mineral
CN	nirk_Cluster16	0.5816	-0.0668	0.0335	-0.0408	0.5137	0.9983781	Mineral
CN	nirk_Cluster17	0.1044	0.0071	0.0377	-0.0363	0.4878	0.9983781	Mineral
CN	nirk_Cluster18	0.1461	0.003	0.001	-0.0758	0.9098	0.9983781	Mineral
CN	nirk_Cluster19	0.2953	0.008	0.0093	-0.0669	0.7325	0.9983781	Mineral
CN	nirk_Cluster2	0.2004	0.0114	0.0282	-0.0466	0.55	0.9983781	Mineral
CN	nirk_Cluster20	0.1934	-0.0077	0.0122	-0.0637	0.6946	0.9983781	Mineral
CN	nirk_Cluster21	0.1711	-0.0107	0.0581	-0.0143	0.3867	0.9983781	Mineral
CN	nirk_Cluster22	0.1469	-0.0168	0.0731	0.0018	0.3298	0.9983781	Mineral
CN	nirk_Cluster23	0.0729	-0.0023	0.0114	-0.0646	0.7048	0.9983781	Mineral
CN	nirk_Cluster24	0.0753	-0.0104	0.1323	0.0655	0.1827	0.9983781	Mineral
CN	nirk_Cluster25	0.5519	-0.1192	0.5046	0.4665	0.003	0.1967267	Mineral
CN	nirk_Cluster26	0.1119	0.0014	0.0018	-0.075	0.8808	0.9983781	Mineral
CN	nirk_Cluster27	0.2066	-0.0008	1.00E-04	-0.0768	0.9734	0.9983781	Mineral
CN	nirk_Cluster28	0.0808	0.0005	0.0002	-0.0767	0.9601	0.9983781	Mineral
CN	nirk_Cluster29	0.162	0.0079	0.0161	-0.0596	0.6526	0.9983781	Mineral
CN	nirk_Cluster3	0.3427	0.0061	0.0039	-0.0727	0.8254	0.9983781	Mineral
CN	nirk_Cluster30	0.0712	-0.0007	0.0003	-0.0766	0.9479	0.9983781	Mineral
CN	nirk_Cluster31	0.1423	0.008	0.0322	-0.0423	0.5225	0.9983781	Mineral
CN	nirk_Cluster32	0.1568	-0.0097	0.0081	-0.0682	0.7502	0.9983781	Mineral
CN	nirk_Cluster33	0.0812	-0.0033	0.0031	-0.0736	0.8443	0.9983781	Mineral
CN	nirk_Cluster34	0.0462	0.0009	0.001	-0.0758	0.9103	0.9983781	Mineral
CN	nirk_Cluster35	0.1282	0.009	0.0089	-0.0674	0.7384	0.9983781	Mineral
CN	nirk_Cluster36	0.0021	0.0006	0.0306	-0.0439	0.5327	0.9983781	Mineral
CN	nirk_Cluster39	0.0045	0.0008	0.0066	-0.0698	0.7732	0.9983781	Mineral
CN	nirk_Cluster4	0.1521	0.0011	0.0003	-0.0767	0.9552	0.9983781	Mineral
CN	nirk_Cluster40	0.0544	-0.0064	0.0706	-0.0009	0.3386	0.9983781	Mineral
CN	nirk_Cluster41	0.1797	-0.0195	0.1406	0.0745	0.1685	0.9983781	Mineral
CN	nirk_Cluster44	0.0756	-0.0236	0.2885	0.2338	0.0389	0.7344159	Mineral
CN	nirk_Cluster46	0.0193	0.0007	0.0011	-0.0757	0.9056	0.9983781	Mineral
CN	nirk_Cluster48	0.003	-0.0015	0.1258	0.0585	0.1946	0.9983781	Mineral
CN	nirk_Cluster49	0.0072	-0.0026	0.0679	-0.0038	0.3484	0.9983781	Mineral
CN	nirk_Cluster5	0.2174	-0.0018	0.0008	-0.0761	0.9227	0.9983781	Mineral
CN	nirk_Cluster50	0.0014	0.00E+00	0.00E+00	-0.0769	0.9992	0.9992181	Mineral
CN	nirk_Cluster52	0.0551	-0.0048	0.0291	-0.0455	0.543	0.9983781	Mineral
CN	nirk_Cluster53	0.0039	0.0013	0.0358	-0.0384	0.4997	0.9983781	Mineral
CN	nirk_Cluster54	0.0024	-1.00E-04	0.0004	-0.0765	0.9467	0.9983781	Mineral
CN	nirk_Cluster55	0.0028	0.0004	0.0057	-0.0708	0.7891	0.9983781	Mineral
CN	nirk_Cluster56	0.0009	-0.0009	0.1509	0.0856	0.1525	0.9983781	Mineral
CN	nirk_Cluster57	0.0017	0.0005	0.0155	-0.0602	0.6586	0.9983781	Mineral
CN	nirk_Cluster58	0.0102	-0.0057	0.2615	0.2047	0.0514	0.8193922	Mineral
CN	nirk_Cluster59	0.007	-0.0007	0.0095	-0.0667	0.7298	0.9983781	Mineral
CN	nirk_Cluster6	0.1713	-0.0264	0.1773	0.114	0.1181	0.9983781	Mineral
CN	nirk_Cluster60	0.0006	1.00E-04	0.0035	-0.0731	0.8336	0.9983781	Mineral
CN	nirk_Cluster62	0.1789	-0.0864	0.5634	0.5298	0.0013	0.124292	Mineral
CN	nirk_Cluster63	0.1586	-0.0452	0.5963	0.5653	0.0007	0.1126225	Mineral
CN	nirk_Cluster64	0.0043	-1.00E-04	0.0002	-0.0767	0.9559	0.9983781	Mineral
CN	nirk_Cluster65	0.0531	-0.0039	0.0238	-0.0513	0.5831	0.9983781	Mineral
CN	nirk_Cluster66	0.0095	-0.0027	0.1136	0.0454	0.2192	0.9983781	Mineral
CN	nirk_Cluster67	0.0232	-0.0018	0.0068	-0.0696	0.7696	0.9983781	Mineral
CN	nirk_Cluster68	0.0158	-0.0053	0.1829	0.1201	0.1118	0.9983781	Mineral
CN	nirk_Cluster69	0.0362	-0.0089	0.1917	0.1295	0.1027	0.9983781	Mineral
CN	nirk_Cluster7	0.096	-0.0018	0.0019	-0.0749	0.8773	0.9983781	Mineral
CN	nirk_Cluster70	0.0892	-0.0646	0.5384	0.5029	0.0018	0.1362858	Mineral
CN	nirk_Cluster71	0.0071	-0.0002	0.0009	-0.076	0.9171	0.9983781	Mineral

CN	nirk_Cluster72	0.0278	-0.0083	0.2784	0.2229	0.0432	0.750108	Mineral
CN	nirk_Cluster73	0.1947	-0.0273	0.0934	0.0236	0.268	0.9983781	Mineral
CN	nirk_Cluster74	0.8012	-0.3528	0.6401	0.6124	0.0003	0.1126225	Mineral
CN	nirk_Cluster75	0.0186	-0.0029	0.0176	-0.0579	0.6372	0.9983781	Mineral
CN	nirk_Cluster76	0.1377	-0.0271	0.2854	0.2304	0.0402	0.7344159	Mineral
CN	nirk_Cluster77	0.0113	-0.0013	0.0045	-0.0721	0.8123	0.9983781	Mineral
CN	nirk_Cluster78	0.3126	-0.0139	0.0096	-0.0666	0.7287	0.9983781	Mineral
CN	nirk_Cluster79	0.0096	-0.0013	0.0089	-0.0673	0.7375	0.9983781	Mineral
CN	nirk_Cluster8	0.1182	0.0049	0.0053	-0.0712	0.7967	0.9983781	Mineral
CN	nirk_Cluster80	0.3544	-0.0526	0.0837	0.0132	0.2957	0.9983781	Mineral
CN	nirk_Cluster82	0.0021	-0.0023	0.2859	0.231	0.04	0.7344159	Mineral
CN	nirk_Cluster83	0.0045	-1.00E-04	0.0001	-0.0768	0.97	0.9983781	Mineral
CN	nirk_Cluster84	0.0235	0.0054	0.0472	-0.0261	0.4368	0.9983781	Mineral
CN	nirk_Cluster85	0.0053	0.00E+00	0.00E+00	-0.0769	0.9829	0.9983781	Mineral
CN	nirk_Cluster86	0.0352	-0.015	0.3359	0.2848	0.0235	0.514492	Mineral
CN	nirk_Cluster87	0.0138	-0.0056	0.2238	0.1641	0.0749	0.9983781	Mineral
CN	nirk_Cluster88	0.0035	0.0003	0.006	-0.0704	0.7834	0.9983781	Mineral
CN	nirk_Cluster89	0.0087	0.0011	0.0119	-0.0641	0.6982	0.9983781	Mineral
CN	nirk_Cluster9	0.061	0.0038	0.0097	-0.0665	0.7269	0.9983781	Mineral
CN	nirk_Cluster90	0.0087	-0.0013	0.0134	-0.0625	0.6812	0.9983781	Mineral
CN	nirk_Cluster91	0.044	-0.0051	0.0389	-0.035	0.4812	0.9983781	Mineral
CN	nirk_Cluster92	0.009	-0.0063	0.3385	0.2876	0.0229	0.514492	Mineral
CN	nirk_Cluster93	0.0357	-0.0095	0.1385	0.0722	0.1719	0.9983781	Mineral
CN	nirk_Cluster94	0.0222	-0.001	0.0033	-0.0733	0.8382	0.9983781	Mineral
CN	nirk_Cluster95	0.0566	-0.0182	0.1962	0.1343	0.0982	0.9983781	Mineral
CN	nirk_Cluster96	0.0588	-0.0015	0.0017	-0.0751	0.885	0.9983781	Mineral
CN	nirk_Cluster97	0.0329	-0.0035	0.0181	-0.0574	0.6324	0.9983781	Mineral
CN	nirk_Cluster98	0.0225	0.0025	0.0115	-0.0645	0.7036	0.9983781	Mineral
CN	nirk_Cluster99	0.0074	-0.0005	0.0031	-0.0735	0.8428	0.9983781	Mineral
CN	nirs_Cluster0	0.1586	-0.004	0.004	-0.0726	0.8222	0.9983781	Mineral
CN	nirs_Cluster1	0.0051	0.00E+00	0.00E+00	-0.0769	0.995	0.9983781	Mineral
CN	nirs_Cluster14	0.0142	0.0033	0.0157	-0.0601	0.6568	0.9983781	Mineral
CN	nirs_Cluster2	0.0174	-0.0024	0.0081	-0.0682	0.7493	0.9983781	Mineral
CN	nirs_Cluster26	0.0007	0.0003	0.0366	-0.0375	0.4946	0.9983781	Mineral
CN	nirs_Cluster28	0.0099	0.0009	0.0028	-0.0739	0.8523	0.9983781	Mineral
CN	nirs_Cluster3	0.1914	0.017	0.1136	0.0455	0.2192	0.9983781	Mineral
CN	nirs_Cluster36	0.0018	-0.0002	0.0008	-0.0761	0.9221	0.9983781	Mineral
CN	nirs_Cluster37	0.0092	0.0017	0.0054	-0.0711	0.7942	0.9983781	Mineral
CN	nirs_Cluster4	0.0003	0.00E+00	0.0014	-0.0754	0.8957	0.9983781	Mineral
CN	nirs_Cluster50	0.0024	0.0013	0.0876	0.0174	0.2842	0.9983781	Mineral
CN	nirs_Cluster9	0.0376	-0.0069	0.0103	-0.0659	0.7194	0.9983781	Mineral
CN	norb_Cluster0	0.0848	-0.0162	0.069	-0.0026	0.3441	0.9983781	Mineral
CN	norb_Cluster1	1.2746	-0.1505	0.0201	-0.0552	0.6139	0.9983781	Mineral
CN	norb_Cluster101	0.0037	-0.0006	0.0099	-0.0662	0.7237	0.9983781	Mineral
CN	norb_Cluster102	0.005	0.0014	0.0536	-0.0192	0.4064	0.9983781	Mineral
CN	norb_Cluster103	0.0025	0.0002	0.0021	-0.0747	0.8724	0.9983781	Mineral
CN	norb_Cluster104	0.0025	0.001	0.0312	-0.0434	0.5292	0.9983781	Mineral
CN	norb_Cluster106	0.1468	-0.0035	0.0015	-0.0753	0.8894	0.9983781	Mineral
CN	norb_Cluster107	0.0163	0.0005	0.0008	-0.0761	0.9219	0.9983781	Mineral
CN	norb_Cluster108	0.0069	0.0002	0.0006	-0.0762	0.9282	0.9983781	Mineral
CN	norb_Cluster109	0.0282	0.0057	0.0625	-0.0096	0.3689	0.9983781	Mineral
CN	norb_Cluster11	0.0156	-0.0008	0.0029	-0.0738	0.8493	0.9983781	Mineral
CN	norb_Cluster110	0.0128	0.001	0.0054	-0.0711	0.794	0.9983781	Mineral
CN	norb_Cluster112	0.0215	-0.0004	0.0007	-0.0762	0.9247	0.9983781	Mineral
CN	norb_Cluster113	0.0157	0.0003	0.0004	-0.0765	0.9458	0.9983781	Mineral

CN	norb_Cluster114	0.0035	0.0006	0.0077	-0.0686	0.7561	0.9983781	Mineral
CN	norb_Cluster115	0.0034	-0.0006	0.0313	-0.0432	0.528	0.9983781	Mineral
CN	norb_Cluster116	0.0083	-0.0015	0.0201	-0.0552	0.6139	0.9983781	Mineral
CN	norb_Cluster117	0.0034	1.00E-04	0.0001	-0.0768	0.9701	0.9983781	Mineral
CN	norb_Cluster118	0.0044	0.0009	0.0275	-0.0473	0.5545	0.9983781	Mineral
CN	norb_Cluster119	0.0263	0.0008	0.001	-0.0759	0.9122	0.9983781	Mineral
CN	norb_Cluster12	0.005	0.0013	0.0256	-0.0494	0.5689	0.9983781	Mineral
CN	norb_Cluster121	0.0023	1.00E-04	0.0003	-0.0766	0.9541	0.9983781	Mineral
CN	norb_Cluster122	0.0184	0.0021	0.0178	-0.0577	0.635	0.9983781	Mineral
CN	norb_Cluster123	0.0071	0.002	0.0591	-0.0132	0.3825	0.9983781	Mineral
CN	norb_Cluster124	0.7452	-0.0424	0.005	-0.0715	0.8023	0.9983781	Mineral
CN	norb_Cluster125	0.0052	0.0005	0.007	-0.0694	0.7664	0.9983781	Mineral
CN	norb_Cluster126	0.011	0.0014	0.0176	-0.0579	0.6371	0.9983781	Mineral
CN	norb_Cluster127	0.2041	-0.0083	0.0026	-0.0742	0.8579	0.9983781	Mineral
CN	norb_Cluster128	0.0123	0.0003	0.0005	-0.0764	0.9384	0.9983781	Mineral
CN	norb_Cluster13	0.0051	-0.0007	0.0064	-0.07	0.7771	0.9983781	Mineral
CN	norb_Cluster131	0.0209	0.0028	0.025	-0.05	0.5736	0.9983781	Mineral
CN	norb_Cluster132	1.2771	-0.0084	0.0002	-0.0768	0.9644	0.9983781	Mineral
CN	norb_Cluster133	0.0023	0.0005	0.0206	-0.0547	0.6095	0.9983781	Mineral
CN	norb_Cluster135	0.0703	0.0024	0.0022	-0.0746	0.8685	0.9983781	Mineral
CN	norb_Cluster136	0.025	0.0028	0.0172	-0.0584	0.6415	0.9983781	Mineral
CN	norb_Cluster139	0.0391	0.0034	0.013	-0.063	0.6863	0.9983781	Mineral
CN	norb_Cluster14	0.0037	-0.0015	0.0999	0.0306	0.2512	0.9983781	Mineral
CN	norb_Cluster140	0.0485	-0.0089	0.0725	0.0011	0.3319	0.9983781	Mineral
CN	norb_Cluster142	0.0018	0.0005	0.0157	-0.0601	0.6567	0.9983781	Mineral
CN	norb_Cluster144	0.0122	1.00E-04	0.00E+00	-0.0769	0.9838	0.9983781	Mineral
CN	norb_Cluster146	0.0418	-0.0009	0.001	-0.0758	0.909	0.9983781	Mineral
CN	norb_Cluster15	0.0502	-0.0019	0.0019	-0.0749	0.8789	0.9983781	Mineral
CN	norb_Cluster150	0.0199	-0.0007	0.0029	-0.0738	0.8479	0.9983781	Mineral
CN	norb_Cluster155	0.1729	-0.0178	0.0192	-0.0562	0.6223	0.9983781	Mineral
CN	norb_Cluster16	0.0165	-0.003	0.0245	-0.0505	0.5774	0.9983781	Mineral
CN	norb_Cluster164	0.0242	-0.0037	0.0235	-0.0517	0.5858	0.9983781	Mineral
CN	norb_Cluster165	0.0092	0.0011	0.0078	-0.0686	0.755	0.9983781	Mineral
CN	norb_Cluster166	0.0046	0.0003	0.0016	-0.0752	0.8886	0.9983781	Mineral
CN	norb_Cluster169	0.0309	-0.0034	0.0124	-0.0635	0.6924	0.9983781	Mineral
CN	norb_Cluster17	0.0035	-0.0017	0.1349	0.0684	0.178	0.9983781	Mineral
CN	norb_Cluster170	0.0299	0.0016	0.0024	-0.0743	0.8615	0.9983781	Mineral
CN	norb_Cluster171	0.0089	0.0003	0.0005	-0.0764	0.9375	0.9983781	Mineral
CN	norb_Cluster172	0.0179	0.001	0.0022	-0.0746	0.8687	0.9983781	Mineral
CN	norb_Cluster175	0.0595	0.0005	1.00E-04	-0.0768	0.9765	0.9983781	Mineral
CN	norb_Cluster176	0.0392	0.00E+00	0.00E+00	-0.0769	0.9984	0.9992181	Mineral
CN	norb_Cluster177	0.0361	0.002	0.0026	-0.0741	0.8564	0.9983781	Mineral
CN	norb_Cluster179	0.003	0.0003	0.0044	-0.0722	0.8151	0.9983781	Mineral
CN	norb_Cluster18	0.0047	-0.0018	0.1154	0.0473	0.2155	0.9983781	Mineral
CN	norb_Cluster180	0.0133	0.0015	0.0184	-0.0571	0.6299	0.9983781	Mineral
CN	norb_Cluster181	0.0003	0.0001	0.0103	-0.0658	0.719	0.9983781	Mineral
CN	norb_Cluster182	0.0018	-0.0002	0.0064	-0.07	0.7762	0.9983781	Mineral
CN	norb_Cluster184	0.0175	-0.0018	0.0115	-0.0646	0.7042	0.9983781	Mineral
CN	norb_Cluster186	0.0011	-0.0005	0.0343	-0.04	0.509	0.9983781	Mineral
CN	norb_Cluster19	0.0058	-0.002	0.119	0.0512	0.208	0.9983781	Mineral
CN	norb_Cluster192	0.0221	0.0017	0.0052	-0.0713	0.7985	0.9983781	Mineral
CN	norb_Cluster193	0.0022	-0.0001	0.0017	-0.0751	0.8856	0.9983781	Mineral
CN	norb_Cluster197	0.0398	0.012	0.0172	-0.0584	0.6416	0.9983781	Mineral
CN	norb_Cluster2	0.0032	-0.0003	0.0034	-0.0733	0.8366	0.9983781	Mineral
CN	norb_Cluster20	0.0665	-0.0082	0.026	-0.0489	0.5659	0.9983781	Mineral

CN	norb_Cluster208	0.0015	0.0005	0.0218	-0.0534	0.5994	0.9983781	Mineral
CN	norb_Cluster21	0.0542	-0.003	0.0047	-0.0719	0.8083	0.9983781	Mineral
CN	norb_Cluster211	0.0141	0.0051	0.0349	-0.0393	0.5048	0.9983781	Mineral
CN	norb_Cluster212	0.0252	0.0035	0.0088	-0.0675	0.7396	0.9983781	Mineral
CN	norb_Cluster214	0.0163	0.0058	0.058	-0.0145	0.3874	0.9983781	Mineral
CN	norb_Cluster217	0.0186	0.0036	0.0164	-0.0593	0.6494	0.9983781	Mineral
CN	norb_Cluster219	0.0108	0.0015	0.0061	-0.0704	0.7822	0.9983781	Mineral
CN	norb_Cluster22	0.0028	-0.0004	0.0059	-0.0706	0.7859	0.9983781	Mineral
CN	norb_Cluster220	0.0248	0.006	0.0187	-0.0568	0.6272	0.9983781	Mineral
CN	norb_Cluster221	0.0248	0.009	0.0362	-0.0379	0.497	0.9983781	Mineral
CN	norb_Cluster222	0.0104	0.0035	0.0229	-0.0523	0.5904	0.9983781	Mineral
CN	norb_Cluster224	0.0238	0.0012	0.0009	-0.0759	0.9143	0.9983781	Mineral
CN	norb_Cluster225	0.0095	0.0015	0.0161	-0.0596	0.6524	0.9983781	Mineral
CN	norb_Cluster226	0.0049	-0.0008	0.011	-0.0651	0.7105	0.9983781	Mineral
CN	norb_Cluster228	0.0009	-0.0006	0.0249	-0.0501	0.5744	0.9983781	Mineral
CN	norb_Cluster229	0.004	-0.0007	0.0137	-0.0622	0.6778	0.9983781	Mineral
CN	norb_Cluster230	0.0019	-0.0006	0.0256	-0.0493	0.5688	0.9983781	Mineral
CN	norb_Cluster231	0.0061	0.0005	0.0024	-0.0744	0.8628	0.9983781	Mineral
CN	norb_Cluster232	0.0044	-0.0008	0.0145	-0.0613	0.6694	0.9983781	Mineral
CN	norb_Cluster234	0.017	-0.0009	0.002	-0.0748	0.8753	0.9983781	Mineral
CN	norb_Cluster235	0.0012	0.0002	0.0054	-0.0711	0.7951	0.9983781	Mineral
CN	norb_Cluster238	0.0135	0.0024	0.0144	-0.0615	0.6707	0.9983781	Mineral
CN	norb_Cluster239	0.0109	0.0022	0.0148	-0.061	0.6659	0.9983781	Mineral
CN	norb_Cluster24	0.3014	-0.031	0.0211	-0.0542	0.6054	0.9983781	Mineral
CN	norb_Cluster244	0.0015	-0.0003	0.0156	-0.0601	0.657	0.9983781	Mineral
CN	norb_Cluster247	0.006	-0.0002	0.0014	-0.0755	0.8962	0.9983781	Mineral
CN	norb_Cluster25	0.1263	-0.0051	0.0023	-0.0744	0.865	0.9983781	Mineral
CN	norb_Cluster250	0.0146	0.0012	0.0078	-0.0685	0.7543	0.9983781	Mineral
CN	norb_Cluster251	0.0064	0.0005	0.0035	-0.0731	0.8331	0.9983781	Mineral
CN	norb_Cluster26	0.1134	-0.015	0.0261	-0.0488	0.5649	0.9983781	Mineral
CN	norb_Cluster28	0.0355	0.003	0.0104	-0.0657	0.7179	0.9983781	Mineral
CN	norb_Cluster29	0.2454	-0.0154	0.0185	-0.057	0.629	0.9983781	Mineral
CN	norb_Cluster31	0.0632	-0.0062	0.0161	-0.0595	0.6519	0.9983781	Mineral
CN	norb_Cluster32	0.1401	-0.0102	0.0062	-0.0703	0.7811	0.9983781	Mineral
CN	norb_Cluster33	0.434	0.0304	0.0131	-0.0628	0.6846	0.9983781	Mineral
CN	norb_Cluster35	0.0746	-0.001	0.0003	-0.0766	0.951	0.9983781	Mineral
CN	norb_Cluster36	1.1002	-0.2838	0.1024	0.0333	0.2451	0.9983781	Mineral
CN	norb_Cluster37	0.7173	-0.1033	0.048	-0.0252	0.4325	0.9983781	Mineral
CN	norb_Cluster38	0.0138	0.0006	0.0037	-0.073	0.8305	0.9983781	Mineral
CN	norb_Cluster39	0.0465	0.0099	0.0733	0.002	0.3291	0.9983781	Mineral
CN	norb_Cluster40	0.2213	-0.0146	0.0076	-0.0687	0.757	0.9983781	Mineral
CN	norb_Cluster42	0.2825	-0.0082	0.0014	-0.0755	0.8966	0.9983781	Mineral
CN	norb_Cluster44	0.0249	1.00E-04	0.00E+00	-0.0769	0.9941	0.9983781	Mineral
CN	norb_Cluster46	0.2575	0.0297	0.018	-0.0575	0.6336	0.9983781	Mineral
CN	norb_Cluster47	0.6453	-0.0562	0.0147	-0.0611	0.6668	0.9983781	Mineral
CN	norb_Cluster5	0.0115	-0.004	0.1729	0.1093	0.1232	0.9983781	Mineral
CN	norb_Cluster51	0.0122	0.0031	0.0352	-0.039	0.5031	0.9983781	Mineral
CN	norb_Cluster52	0.2809	-0.0256	0.013	-0.0629	0.6858	0.9983781	Mineral
CN	norb_Cluster56	0.0178	-0.0003	0.0005	-0.0764	0.9394	0.9983781	Mineral
CN	norb_Cluster57	0.004	-0.0001	0.0009	-0.0759	0.914	0.9983781	Mineral
CN	norb_Cluster59	0.0322	0.0005	0.0002	-0.0767	0.9586	0.9983781	Mineral
CN	norb_Cluster6	0.6934	-0.1554	0.0879	0.0178	0.2832	0.9983781	Mineral
CN	norb_Cluster60	0.345	-0.0294	0.0058	-0.0707	0.7881	0.9983781	Mineral
CN	norb_Cluster61	0.5486	-0.0148	0.0013	-0.0755	0.898	0.9983781	Mineral
CN	norb_Cluster62	0.003	0.0015	0.1437	0.0778	0.1635	0.9983781	Mineral

CN	norb_Cluster64	0.0407	-0.0021	0.0072	-0.0691	0.763	0.9983781	Mineral
CN	norb_Cluster66	0.0016	0.0005	0.0238	-0.0513	0.5834	0.9983781	Mineral
CN	norb_Cluster67	0.0279	0.0031	0.0183	-0.0572	0.6308	0.9983781	Mineral
CN	norb_Cluster68	0.003	0.0004	0.0115	-0.0646	0.7039	0.9983781	Mineral
CN	norb_Cluster69	0.0032	0.0004	0.0073	-0.0691	0.7624	0.9983781	Mineral
CN	norb_Cluster7	0.0786	-0.001	0.0002	-0.0767	0.9575	0.9983781	Mineral
CN	norb_Cluster70	0.1262	-0.0041	0.0027	-0.074	0.8531	0.9983781	Mineral
CN	norb_Cluster71	0.1512	-0.0054	0.0039	-0.0727	0.8254	0.9983781	Mineral
CN	norb_Cluster74	0.0387	-0.0009	0.0013	-0.0755	0.8989	0.9983781	Mineral
CN	norb_Cluster75	0.1531	-0.01	0.0172	-0.0583	0.6408	0.9983781	Mineral
CN	norb_Cluster76	0.1215	0.0101	0.0042	-0.0724	0.8181	0.9983781	Mineral
CN	norb_Cluster77	0.0492	0.0028	0.0087	-0.0675	0.7406	0.9983781	Mineral
CN	norb_Cluster8	0.0206	0.002	0.0111	-0.0649	0.7082	0.9983781	Mineral
CN	norb_Cluster80	0.0021	0.0005	0.0199	-0.0555	0.6158	0.9983781	Mineral
CN	norb_Cluster81	0.0013	0.0005	0.0434	-0.0301	0.456	0.9983781	Mineral
CN	norb_Cluster83	0.011	0.0001	1.00E-04	-0.0769	0.978	0.9983781	Mineral
CN	norb_Cluster84	0.0093	0.0005	0.0026	-0.0741	0.8555	0.9983781	Mineral
CN	norb_Cluster85	0.0025	0.0007	0.0261	-0.0488	0.5651	0.9983781	Mineral
CN	norb_Cluster86	0.041	0.0107	0.0256	-0.0493	0.5687	0.9983781	Mineral
CN	norb_Cluster88	0.0019	0.00E+00	0.00E+00	-0.0769	0.993	0.9983781	Mineral
CN	norb_Cluster90	0.0013	0.0008	0.0474	-0.0259	0.4356	0.9983781	Mineral
CN	norb_Cluster91	0.7571	0.0296	0.0022	-0.0745	0.8673	0.9983781	Mineral
CN	norb_Cluster92	0.0021	0.0004	0.0079	-0.0684	0.7526	0.9983781	Mineral
CN	norb_Cluster93	0.0028	-0.0008	0.0186	-0.0569	0.6283	0.9983781	Mineral
CN	norb_Cluster94	0.002	-0.0008	0.0569	-0.0157	0.392	0.9983781	Mineral
CN	norb_Cluster96	0.0006	-0.0002	0.0142	-0.0616	0.6724	0.9983781	Mineral
CN	norb_Cluster97	0.0162	0.0023	0.0181	-0.0575	0.633	0.9983781	Mineral
CN	norb_Cluster98	0.0176	0.0027	0.0161	-0.0596	0.6523	0.9983781	Mineral
CN	nosz_Cluster0	0.0057	0.0017	0.0753	0.0042	0.3223	0.9983781	Mineral
CN	nosz_Cluster10	0.0128	0.0048	0.1414	0.0754	0.1672	0.9983781	Mineral
CN	nosz_Cluster102	0.0057	-0.0017	0.1425	0.0765	0.1654	0.9983781	Mineral
CN	nosz_Cluster103	0.0496	-0.0092	0.0587	-0.0137	0.3842	0.9983781	Mineral
CN	nosz_Cluster105	0.0016	0.00E+00	0.0002	-0.0767	0.9604	0.9983781	Mineral
CN	nosz_Cluster106	0.0088	-0.0005	0.0027	-0.074	0.8538	0.9983781	Mineral
CN	nosz_Cluster107	0.0003	0.00E+00	0.0014	-0.0754	0.8957	0.9983781	Mineral
CN	nosz_Cluster108	0.0043	0.0026	0.1564	0.0915	0.1446	0.9983781	Mineral
CN	nosz_Cluster109	0.0416	-0.0083	0.1454	0.0797	0.1607	0.9983781	Mineral
CN	nosz_Cluster11	0.0104	-0.0012	0.015	-0.0608	0.6638	0.9983781	Mineral
CN	nosz_Cluster110	0.0004	0.0011	0.5389	0.5035	0.0018	0.1362858	Mineral
CN	nosz_Cluster111	0.0078	-0.0008	0.012	-0.064	0.6981	0.9983781	Mineral
CN	nosz_Cluster116	0.0074	0.0029	0.2037	0.1425	0.0913	0.9983781	Mineral
CN	nosz_Cluster118	0.0064	0.0019	0.0492	-0.024	0.4271	0.9983781	Mineral
CN	nosz_Cluster119	0.0019	-0.0001	0.0011	-0.0757	0.905	0.9983781	Mineral
CN	nosz_Cluster120	0.0083	0.0003	0.001	-0.0759	0.9113	0.9983781	Mineral
CN	nosz_Cluster123	0.0136	0.0008	0.0035	-0.0731	0.8331	0.9983781	Mineral
CN	nosz_Cluster128	0.005	0.0006	0.0078	-0.0685	0.7544	0.9983781	Mineral
CN	nosz_Cluster129	0.05	-0.0032	0.0158	-0.0599	0.6549	0.9983781	Mineral
CN	nosz_Cluster13	0.0124	-0.0045	0.1337	0.0671	0.1802	0.9983781	Mineral
CN	nosz_Cluster130	0.1138	0.0073	0.001	-0.0758	0.9093	0.9983781	Mineral
CN	nosz_Cluster132	0.004	-0.0016	0.1373	0.0709	0.174	0.9983781	Mineral
CN	nosz_Cluster14	0.0149	0.0015	0.0067	-0.0698	0.7725	0.9983781	Mineral
CN	nosz_Cluster16	0.008	0.0037	0.1043	0.0354	0.2403	0.9983781	Mineral
CN	nosz_Cluster17	0.0427	-0.0023	0.005	-0.0716	0.8031	0.9983781	Mineral
CN	nosz_Cluster18	0.0184	0.0043	0.0386	-0.0354	0.4828	0.9983781	Mineral
CN	nosz_Cluster20	0.0117	0.0026	0.0274	-0.0474	0.5555	0.9983781	Mineral

CN	nosz_Cluster21	0.0117	0.0018	0.0158	-0.0599	0.6552	0.9983781	Mineral
CN	nosz_Cluster22	0.0325	0.0078	0.0288	-0.0459	0.5454	0.9983781	Mineral
CN	nosz_Cluster23	0.019	0.0053	0.064	-0.008	0.3631	0.9983781	Mineral
CN	nosz_Cluster24	0.0019	0.0002	0.002	-0.0748	0.8743	0.9983781	Mineral
CN	nosz_Cluster25	0.0026	0.00E+00	0.00E+00	-0.0769	0.995	0.9983781	Mineral
CN	nosz_Cluster26	0.0068	0.002	0.0432	-0.0305	0.4575	0.9983781	Mineral
CN	nosz_Cluster27	0.007	-0.0004	0.0029	-0.0738	0.8497	0.9983781	Mineral
CN	nosz_Cluster28	0.0358	0.0023	0.0052	-0.0713	0.7983	0.9983781	Mineral
CN	nosz_Cluster30	0.0341	0.0043	0.023	-0.0521	0.5893	0.9983781	Mineral
CN	nosz_Cluster32	0.016	0.0009	0.0039	-0.0727	0.8248	0.9983781	Mineral
CN	nosz_Cluster33	0.0187	0.0048	0.0496	-0.0235	0.4248	0.9983781	Mineral
CN	nosz_Cluster34	0.0387	0.0052	0.0109	-0.0651	0.7106	0.9983781	Mineral
CN	nosz_Cluster35	0.0097	0.0015	0.0225	-0.0527	0.5938	0.9983781	Mineral
CN	nosz_Cluster36	0.0213	0.0012	0.0033	-0.0734	0.8389	0.9983781	Mineral
CN	nosz_Cluster37	0.0077	0.0003	0.0011	-0.0757	0.907	0.9983781	Mineral
CN	nosz_Cluster38	0.0144	0.0043	0.0467	-0.0267	0.4393	0.9983781	Mineral
CN	nosz_Cluster39	0.0653	0.0089	0.0285	-0.0462	0.5472	0.9983781	Mineral
CN	nosz_Cluster40	0.0262	0.0054	0.0391	-0.0348	0.4799	0.9983781	Mineral
CN	nosz_Cluster41	0.0258	0.0038	0.0199	-0.0555	0.6159	0.9983781	Mineral
CN	nosz_Cluster42	0.03	0.0052	0.0277	-0.0471	0.553	0.9983781	Mineral
CN	nosz_Cluster43	0.001	-0.0002	0.0038	-0.0729	0.8281	0.9983781	Mineral
CN	nosz_Cluster44	0.0068	-0.0034	0.1715	0.1078	0.1249	0.9983781	Mineral
CN	nosz_Cluster45	0.0056	0.0017	0.0629	-0.0092	0.3674	0.9983781	Mineral
CN	nosz_Cluster46	0.0082	0.0007	0.0097	-0.0665	0.7268	0.9983781	Mineral
CN	nosz_Cluster47	0.0012	-1.00E-04	0.0015	-0.0753	0.8914	0.9983781	Mineral
CN	nosz_Cluster48	0.0028	-0.0004	0.0074	-0.069	0.7608	0.9983781	Mineral
CN	nosz_Cluster49	0.0012	0.0004	0.0223	-0.0529	0.5954	0.9983781	Mineral
CN	nosz_Cluster5	0.004	0.001	0.0225	-0.0527	0.5934	0.9983781	Mineral
CN	nosz_Cluster51	0.003	0.0016	0.1469	0.0813	0.1585	0.9983781	Mineral
CN	nosz_Cluster53	0.0012	1.00E-04	0.0008	-0.076	0.9195	0.9983781	Mineral
CN	nosz_Cluster54	0.0037	0.0007	0.0157	-0.06	0.6564	0.9983781	Mineral
CN	nosz_Cluster57	0.2232	-0.0017	1.00E-04	-0.0768	0.9742	0.9983781	Mineral
CN	nosz_Cluster58	0.0053	-0.001	0.0165	-0.0591	0.648	0.9983781	Mineral
CN	nosz_Cluster59	0.0008	-0.0004	0.0133	-0.0626	0.6823	0.9983781	Mineral
CN	nosz_Cluster6	0.0034	0.0007	0.0146	-0.0612	0.6683	0.9983781	Mineral
CN	nosz_Cluster60	0.0099	0.003	0.0624	-0.0097	0.3691	0.9983781	Mineral
CN	nosz_Cluster61	0.0178	-0.0071	0.3722	0.3239	0.0157	0.42175	Mineral
CN	nosz_Cluster62	0.0493	0.0041	0.0078	-0.0685	0.7539	0.9983781	Mineral
CN	nosz_Cluster64	0.0023	-1.00E-04	0.0001	-0.0768	0.9664	0.9983781	Mineral
CN	nosz_Cluster65	0.0083	0.0027	0.078	0.0071	0.3135	0.9983781	Mineral
CN	nosz_Cluster66	0.0428	-0.0056	0.0162	-0.0595	0.651	0.9983781	Mineral
CN	nosz_Cluster67	0.0669	0.0028	0.0017	-0.0751	0.8843	0.9983781	Mineral
CN	nosz_Cluster69	0.008	0.0015	0.0292	-0.0455	0.5428	0.9983781	Mineral
CN	nosz_Cluster70	0.0655	0.0143	0.0181	-0.0574	0.6323	0.9983781	Mineral
CN	nosz_Cluster71	0.0101	0.0019	0.0169	-0.0587	0.6443	0.9983781	Mineral
CN	nosz_Cluster72	0.066	-0.0008	0.0002	-0.0768	0.965	0.9983781	Mineral
CN	nosz_Cluster73	0.0031	-0.0011	0.0491	-0.024	0.4272	0.9983781	Mineral
CN	nosz_Cluster74	0.0017	-0.0004	0.0121	-0.0639	0.696	0.9983781	Mineral
CN	nosz_Cluster75	0.132	0.0236	0.0066	-0.0698	0.7733	0.9983781	Mineral
CN	nosz_Cluster77	0.0002	-0.0004	0.1644	0.1002	0.1337	0.9983781	Mineral
CN	nosz_Cluster78	0.0054	-0.0009	0.0223	-0.0529	0.5949	0.9983781	Mineral
CN	nosz_Cluster79	0.0401	0.0014	0.0019	-0.0748	0.8761	0.9983781	Mineral
CN	nosz_Cluster8	0.0106	0.0013	0.025	-0.05	0.5734	0.9983781	Mineral
CN	nosz_Cluster80	0.0039	0.0007	0.0149	-0.0609	0.6646	0.9983781	Mineral
CN	nosz_Cluster81	0.0099	0.0032	0.0384	-0.0355	0.4838	0.9983781	Mineral

CN	nosz_Cluster82	0.0023	-0.0002	0.0012	-0.0756	0.9011	0.9983781	Mineral
CN	nosz_Cluster83	0.0014	-0.0002	0.002	-0.0747	0.8729	0.9983781	Mineral
CN	nosz_Cluster85	0.0027	-0.0003	0.0029	-0.0738	0.8495	0.9983781	Mineral
CN	nosz_Cluster86	0.0038	0.00E+00	0.00E+00	-0.0769	0.9928	0.9983781	Mineral
CN	nosz_Cluster87	0.0034	0.0011	0.0592	-0.0132	0.3824	0.9983781	Mineral
CN	nosz_Cluster88	0.0246	-0.0017	0.0123	-0.0637	0.6943	0.9983781	Mineral
CN	nosz_Cluster89	0.0607	0.0022	0.0016	-0.0752	0.8871	0.9983781	Mineral
CN	nosz_Cluster9	0.0034	0.0003	0.0031	-0.0736	0.8431	0.9983781	Mineral
CN	nosz_Cluster90	0.0139	-0.0014	0.021	-0.0543	0.6063	0.9983781	Mineral
CN	nosz_Cluster91	0.0232	0.01	0.1454	0.0796	0.1609	0.9983781	Mineral
CN	nosz_Cluster93	0.0488	-0.0062	0.0216	-0.0537	0.6015	0.9983781	Mineral
CN	nosz_Cluster95	0.0094	0.0033	0.1426	0.0766	0.1653	0.9983781	Mineral
CN	nosz_Cluster96	0.0064	0.0006	0.01	-0.0662	0.7234	0.9983781	Mineral
CN	nosz_Cluster97	0.0026	-0.0004	0.0121	-0.0639	0.6968	0.9983781	Mineral
CN	nosz_Cluster98	0.0035	-0.0001	0.0002	-0.0767	0.9594	0.9983781	Mineral
CN	nosz_Cluster99	0.0061	0.0022	0.1414	0.0754	0.1672	0.9983781	Mineral
CN	nrfa_Cluster10	0.0978	0.0026	0.0006	-0.0763	0.93	0.9983781	Mineral
CN	nrfa_Cluster100	0.1249	-0.0015	1.00E-04	-0.0768	0.9732	0.9983781	Mineral
CN	nrfa_Cluster104	0.1478	-0.0049	0.0019	-0.0749	0.8788	0.9983781	Mineral
CN	nrfa_Cluster105	0.006	-0.0006	0.0044	-0.0722	0.8149	0.9983781	Mineral
CN	nrfa_Cluster109	0.2753	-0.004	0.0004	-0.0765	0.9432	0.9983781	Mineral
CN	nrfa_Cluster116	0.3208	-0.0294	0.0293	-0.0453	0.5416	0.9983781	Mineral
CN	nrfa_Cluster118	0.2298	-0.0008	0.00E+00	-0.0769	0.9933	0.9983781	Mineral
CN	nrfa_Cluster125	0.1081	0.0007	0.00E+00	-0.0769	0.9874	0.9983781	Mineral
CN	nrfa_Cluster154	0.0837	-0.0061	0.0035	-0.0731	0.8336	0.9983781	Mineral
CN	nrfa_Cluster164	0.0044	0.0009	0.0245	-0.0506	0.5776	0.9983781	Mineral
CN	nrfa_Cluster172	0.0036	0.0009	0.0218	-0.0534	0.5991	0.9983781	Mineral
CN	nrfa_Cluster175	0.0053	0.0001	1.00E-04	-0.0768	0.9727	0.9983781	Mineral
CN	nrfa_Cluster20	0.045	0.0051	0.011	-0.065	0.7095	0.9983781	Mineral
CN	nrfa_Cluster26	0.1433	0.0071	0.0015	-0.0753	0.8921	0.9983781	Mineral
CN	nrfa_Cluster29	0.0059	-0.0008	0.0091	-0.0671	0.7346	0.9983781	Mineral
CN	nrfa_Cluster34	0.0642	0.0053	0.0059	-0.0705	0.7851	0.9983781	Mineral
CN	nrfa_Cluster38	0.0033	1.00E-04	0.0001	-0.0768	0.9694	0.9983781	Mineral
CN	nrfa_Cluster61	0.0047	0.0002	0.0005	-0.0764	0.9381	0.9983781	Mineral
CN	nrfa_Cluster62	0.0589	-0.0021	0.0006	-0.0763	0.9319	0.9983781	Mineral
CN	nrfa_Cluster66	0.1282	-0.0074	0.0076	-0.0687	0.7568	0.9983781	Mineral
CN	nrfa_Cluster69	0.0035	0.0013	0.0603	-0.012	0.3778	0.9983781	Mineral
CN	nrfa_Cluster71	0.0322	0.004	0.0088	-0.0675	0.7399	0.9983781	Mineral
CN	nrfa_Cluster72	0.0969	0.0023	0.0004	-0.0764	0.9402	0.9983781	Mineral
CN	nrfa_Cluster73	0.0042	0.0004	0.0034	-0.0733	0.8371	0.9983781	Mineral
CN	nrfa_Cluster74	0.1692	-0.0067	0.0012	-0.0756	0.9026	0.9983781	Mineral
CN	nrfa_Cluster76	0.0922	-0.0021	0.0003	-0.0766	0.9489	0.9983781	Mineral
CN	nrfa_Cluster79	0.0138	0.0014	0.0023	-0.0744	0.8645	0.9983781	Mineral
CN	nrfa_Cluster87	0.0207	0.006	0.0284	-0.0464	0.5485	0.9983781	Mineral
CN	nrfa_Cluster89	0.0023	0.0004	0.009	-0.0672	0.7366	0.9983781	Mineral
CN	nrfa_Cluster92	0.0041	0.0004	0.0056	-0.0709	0.791	0.9983781	Mineral
CN	nrfa_Cluster93	0.0581	0.0172	0.0366	-0.0375	0.4947	0.9983781	Mineral
CN	nrfa_Cluster94	0.0003	-0.0002	0.0249	-0.0501	0.5744	0.9983781	Mineral
CN	nrfa_Cluster97	0.0225	-0.0041	0.0376	-0.0364	0.4887	0.9983781	Mineral
CN	nrfa_Cluster99	0.0184	-0.0027	0.0183	-0.0572	0.6308	0.9983781	Mineral
Latitude	amoA_A_Cluster26	0.0053	-0.0062	0.5188	0.4818	0.0025	0.0415283	Mineral
Latitude	amoA_A_Cluster45	0.0071	-0.0049	0.2676	0.2113	0.0483	0.2243071	Mineral
Latitude	amoA_B_Cluster0	0.1272	0.0304	0.4734	0.4328	0.0046	0.0574803	Mineral
Latitude	amoA_B_Cluster1	0.0205	-0.0025	0.0252	-0.0497	0.5717	0.8231248	Mineral
Latitude	amoA_B_Cluster10	0.0539	-0.0164	0.4025	0.3565	0.0111	0.0947236	Mineral

Latitude	amoA_B_Cluster11	0.0486	0.0025	0.0067	-0.0698	0.7726	0.9130755	Mineral
Latitude	amoA_B_Cluster12	0.093	0.0078	0.0426	-0.031	0.4604	0.7655881	Mineral
Latitude	amoA_B_Cluster13	0.6417	0.0736	0.0407	-0.0331	0.4711	0.7699471	Mineral
Latitude	amoA_B_Cluster14	0.0339	0.0065	0.0541	-0.0187	0.4041	0.7381723	Mineral
Latitude	amoA_B_Cluster15	0.0413	-0.0053	0.0414	-0.0323	0.4669	0.7699471	Mineral
Latitude	amoA_B_Cluster2	0.2143	0.0197	0.0277	-0.0471	0.5533	0.8157013	Mineral
Latitude	amoA_B_Cluster20	0.0078	-0.0004	0.0017	-0.0751	0.8827	0.9616149	Mineral
Latitude	amoA_B_Cluster23	0.0626	-0.0017	0.0027	-0.074	0.8542	0.9485146	Mineral
Latitude	amoA_B_Cluster3	0.0244	-0.0025	0.0153	-0.0605	0.6608	0.8493898	Mineral
Latitude	amoA_B_Cluster4	0.0169	-0.0033	0.0462	-0.0272	0.4419	0.7556857	Mineral
Latitude	amoA_B_Cluster5	0.1032	0.0176	0.1989	0.1373	0.0956	0.3292636	Mineral
Latitude	amoA_B_Cluster6	0.0279	-0.0007	0.0015	-0.0753	0.8897	0.9616149	Mineral
Latitude	amoA_B_Cluster7	0.0232	-0.0028	0.0188	-0.0567	0.6262	0.8399397	Mineral
Latitude	amoA_B_Cluster8	0.0268	-0.0078	0.101	0.0319	0.2483	0.5836709	Mineral
Latitude	amoA_B_Cluster9	0.0693	0.0027	0.0027	-0.0741	0.8553	0.9485146	Mineral
Latitude	nifh_Cluster0	0.0318	0.0038	0.0479	-0.0254	0.4334	0.7526909	Mineral
Latitude	nifh_Cluster10	0.0033	0.0014	0.0982	0.0288	0.2555	0.58651	Mineral
Latitude	nifh_Cluster100	0.002	0.0006	0.0269	-0.048	0.5594	0.8163038	Mineral
Latitude	nifh_Cluster103	0.0433	-0.0156	0.3565	0.307	0.0188	0.1322235	Mineral
Latitude	nifh_Cluster1034	0.0337	0.0216	0.4274	0.3834	0.0082	0.0768221	Mineral
Latitude	nifh_Cluster105	0.0164	0.0089	0.2689	0.2127	0.0477	0.2231319	Mineral
Latitude	nifh_Cluster108	0.0076	-0.0049	0.3969	0.3505	0.0118	0.0983215	Mineral
Latitude	nifh_Cluster1099	0.0478	-0.0141	0.0503	-0.0228	0.4217	0.7515869	Mineral
Latitude	nifh_Cluster11	0.0025	0.0007	0.0497	-0.0234	0.4244	0.7526909	Mineral
Latitude	nifh_Cluster1112	0.0037	-0.0001	0.0006	-0.0763	0.9336	0.9714871	Mineral
Latitude	nifh_Cluster112	0.0098	0.0031	0.0547	-0.018	0.4015	0.7380322	Mineral
Latitude	nifh_Cluster113	0.0374	0.0034	0.0277	-0.0471	0.5536	0.8157013	Mineral
Latitude	nifh_Cluster114	0.0304	0.0042	0.0925	0.0227	0.2703	0.5983433	Mineral
Latitude	nifh_Cluster1141	0.0296	0.0168	0.4664	0.4254	0.005	0.0598955	Mineral
Latitude	nifh_Cluster115	0.0098	-0.0016	0.0395	-0.0343	0.4774	0.7740758	Mineral
Latitude	nifh_Cluster116	0.0883	0.0089	0.036	-0.0382	0.4985	0.774411	Mineral
Latitude	nifh_Cluster1163	0.0051	0.0007	0.0171	-0.0586	0.6427	0.844867	Mineral
Latitude	nifh_Cluster1179	0.0037	0.0023	0.1414	0.0753	0.1672	0.4760351	Mineral
Latitude	nifh_Cluster1182	0.0209	-0.004	0.1564	0.0915	0.1446	0.4308813	Mineral
Latitude	nifh_Cluster1183	0.0045	0.0004	0.0095	-0.0667	0.7299	0.8879017	Mineral
Latitude	nifh_Cluster1197	0.0115	-0.0053	0.2371	0.1784	0.0656	0.2708221	Mineral
Latitude	nifh_Cluster1199	0.0048	0.0022	0.1338	0.0671	0.18	0.4906141	Mineral
Latitude	nifh_Cluster120	0.02	0.016	0.2456	0.1875	0.0603	0.2548116	Mineral
Latitude	nifh_Cluster1203	0.0217	-0.0142	0.4914	0.4523	0.0036	0.0493365	Mineral
Latitude	nifh_Cluster1206	0.006	-0.0014	0.0614	-0.0108	0.3731	0.7026992	Mineral
Latitude	nifh_Cluster1207	0.0078	0.0033	0.1196	0.0519	0.2067	0.5189062	Mineral
Latitude	nifh_Cluster1209	0.1617	-0.0262	0.1808	0.1178	0.1141	0.3619644	Mineral
Latitude	nifh_Cluster121	0.0123	-0.002	0.0105	-0.0656	0.7165	0.8791838	Mineral
Latitude	nifh_Cluster1211	0.0068	-0.0007	0.0116	-0.0644	0.7021	0.8780464	Mineral
Latitude	nifh_Cluster1213	0.0032	-0.0012	0.1135	0.0453	0.2195	0.5464117	Mineral
Latitude	nifh_Cluster122	0.0041	-0.0006	0.0099	-0.0663	0.7248	0.8846062	Mineral
Latitude	nifh_Cluster123	0.0092	0.0029	0.049	-0.0241	0.4277	0.7526909	Mineral
Latitude	nifh_Cluster1236	0.031	-0.0314	0.5756	0.5429	0.001	0.029265	Mineral
Latitude	nifh_Cluster1238	0.0034	-0.0002	0.0014	-0.0754	0.8938	0.9616149	Mineral
Latitude	nifh_Cluster124	0.0101	-0.0007	0.0068	-0.0696	0.7696	0.9127308	Mineral
Latitude	nifh_Cluster1261	0.0023	-0.0007	0.0281	-0.0467	0.5505	0.8157013	Mineral
Latitude	nifh_Cluster1262	0.008	0.0056	0.3396	0.2888	0.0226	0.1435602	Mineral
Latitude	nifh_Cluster1266	0.0072	-0.0028	0.1405	0.0744	0.1687	0.476204	Mineral
Latitude	nifh_Cluster1267	0.0388	-0.0001	0.00E+00	-0.0769	0.9945	0.9944876	Mineral
Latitude	nifh_Cluster127	0.0033	0.0012	0.0431	-0.0305	0.4577	0.7649324	Mineral

Latitude	nifh_Cluster1278	0.2105	0.0337	0.0791	0.0082	0.31	0.6432892	Mineral
Latitude	nifh_Cluster128	0.0043	0.0015	0.0843	0.0139	0.2938	0.6311613	Mineral
Latitude	nifh_Cluster1289	0.004	-0.0009	0.0358	-0.0384	0.4994	0.774411	Mineral
Latitude	nifh_Cluster129	0.0086	0.0036	0.0842	0.0137	0.2942	0.6311613	Mineral
Latitude	nifh_Cluster1292	0.0106	0.0013	0.0144	-0.0614	0.6704	0.8524441	Mineral
Latitude	nifh_Cluster130	0.0071	0.002	0.0225	-0.0527	0.5934	0.8287537	Mineral
Latitude	nifh_Cluster1305	0.0048	-0.0011	0.0487	-0.0244	0.4292	0.7526909	Mineral
Latitude	nifh_Cluster1306	0.0055	-0.0008	0.0162	-0.0594	0.6508	0.844867	Mineral
Latitude	nifh_Cluster131	0.0067	0.0019	0.0351	-0.0392	0.504	0.774411	Mineral
Latitude	nifh_Cluster1319	0.0048	0.0028	0.18	0.1169	0.115	0.3629606	Mineral
Latitude	nifh_Cluster132	0.0064	0.0028	0.0959	0.0263	0.2614	0.592764	Mineral
Latitude	nifh_Cluster1320	0.0028	-0.0002	0.0015	-0.0754	0.8927	0.9616149	Mineral
Latitude	nifh_Cluster1323	0.0022	-0.0009	0.0936	0.0239	0.2674	0.5954729	Mineral
Latitude	nifh_Cluster1324	0.0235	-0.0146	0.3798	0.3321	0.0144	0.1141129	Mineral
Latitude	nifh_Cluster1336	0.0159	0.0004	0.0021	-0.0747	0.8726	0.9587053	Mineral
Latitude	nifh_Cluster1340	0.0478	-0.0111	0.0391	-0.0348	0.48	0.7740758	Mineral
Latitude	nifh_Cluster1345	0.033	-0.003	0.0043	-0.0723	0.8171	0.9379654	Mineral
Latitude	nifh_Cluster1370	0.0022	0.0018	0.2087	0.1479	0.0869	0.3204252	Mineral
Latitude	nifh_Cluster1375	0.0017	-0.0014	0.2929	0.2385	0.0372	0.1892053	Mineral
Latitude	nifh_Cluster139	0.0038	-0.0014	0.0513	-0.0217	0.4168	0.7483419	Mineral
Latitude	nifh_Cluster140	0.0051	-0.0005	0.0086	-0.0677	0.7424	0.8976246	Mineral
Latitude	nifh_Cluster141	0.0808	-0.0033	0.0148	-0.061	0.6663	0.8524441	Mineral
Latitude	nifh_Cluster148	0.006	0.0004	0.0021	-0.0746	0.8703	0.957939	Mineral
Latitude	nifh_Cluster1480	0.0006	0.0001	0.0044	-0.0722	0.8141	0.9379654	Mineral
Latitude	nifh_Cluster152	0.0602	0.0252	0.4066	0.3609	0.0106	0.0915509	Mineral
Latitude	nifh_Cluster156	0.0459	0.0034	0.0113	-0.0648	0.7066	0.8780464	Mineral
Latitude	nifh_Cluster1562	0.0293	0.0076	0.0249	-0.0502	0.5747	0.8231248	Mineral
Latitude	nifh_Cluster158	0.019	-0.009	0.2453	0.1873	0.0605	0.2548116	Mineral
Latitude	nifh_Cluster16	0.0023	-0.0003	0.0035	-0.0732	0.835	0.947348	Mineral
Latitude	nifh_Cluster166	0.0165	0.0094	0.1322	0.0654	0.1829	0.493791	Mineral
Latitude	nifh_Cluster205	0.0121	0.0003	0.0015	-0.0753	0.8914	0.9616149	Mineral
Latitude	nifh_Cluster21	0.0022	-0.0005	0.0223	-0.0529	0.5956	0.8287537	Mineral
Latitude	nifh_Cluster222	0.0069	0.0045	0.1426	0.0767	0.1652	0.4754445	Mineral
Latitude	nifh_Cluster225	0.021	0.0023	0.0278	-0.047	0.5528	0.8157013	Mineral
Latitude	nifh_Cluster230	0.0052	-0.0011	0.0367	-0.0374	0.4942	0.774411	Mineral
Latitude	nifh_Cluster231	0.0019	0.0012	0.1217	0.0541	0.2025	0.5141763	Mineral
Latitude	nifh_Cluster236	0.0034	-0.0004	0.0082	-0.0681	0.7483	0.9016193	Mineral
Latitude	nifh_Cluster237	0.007	-0.0022	0.0987	0.0294	0.254	0.5854185	Mineral
Latitude	nifh_Cluster243	0.0253	-0.0179	0.3416	0.2909	0.0221	0.143541	Mineral
Latitude	nifh_Cluster2432	0.0317	-0.006	0.0203	-0.0551	0.6125	0.8364341	Mineral
Latitude	nifh_Cluster2433	0.0028	-1.00E-04	0.0002	-0.0768	0.9651	0.9831072	Mineral
Latitude	nifh_Cluster246	0.0068	0.0031	0.1095	0.041	0.2283	0.5589457	Mineral
Latitude	nifh_Cluster25	0.0195	0.0013	0.0115	-0.0645	0.7036	0.8780464	Mineral
Latitude	nifh_Cluster256	0.0023	-0.0025	0.509	0.4713	0.0028	0.0418851	Mineral
Latitude	nifh_Cluster264	0.0025	0.0016	0.26	0.2031	0.0521	0.231991	Mineral
Latitude	nifh_Cluster265	0.0702	-0.0069	0.0609	-0.0113	0.3752	0.7026992	Mineral
Latitude	nifh_Cluster267	0.0071	-0.0037	0.2582	0.2012	0.0531	0.2338362	Mineral
Latitude	nifh_Cluster268	0.0109	0.0004	0.0038	-0.0728	0.8275	0.944327	Mineral
Latitude	nifh_Cluster269	0.0283	-0.0162	0.3062	0.2528	0.0324	0.172091	Mineral
Latitude	nifh_Cluster272	0.0012	-0.0006	0.0475	-0.0258	0.4352	0.7526909	Mineral
Latitude	nifh_Cluster274	0.0014	0.0006	0.0193	-0.0561	0.6215	0.8399397	Mineral
Latitude	nifh_Cluster275	0.0051	-0.0025	0.286	0.2311	0.04	0.1965878	Mineral
Latitude	nifh_Cluster278	0.0056	0.0031	0.1949	0.133	0.0995	0.3353205	Mineral
Latitude	nifh_Cluster279	0.0058	0.0018	0.0813	0.0106	0.303	0.6432892	Mineral
Latitude	nifh_Cluster28	0.0017	-0.0013	0.1921	0.1299	0.1023	0.3397356	Mineral

Latitude	nifh_Cluster280	0.0034	-0.0003	0.0021	-0.0746	0.8698	0.957939	Mineral
Latitude	nifh_Cluster282	0.0052	0.0009	0.0273	-0.0475	0.556	0.8160211	Mineral
Latitude	nifh_Cluster287	0.003	0.0008	0.0451	-0.0284	0.4474	0.7579666	Mineral
Latitude	nifh_Cluster290	0.0096	-0.0068	0.31	0.2569	0.0311	0.1682984	Mineral
Latitude	nifh_Cluster297	0.004	-0.0021	0.0663	-0.0056	0.3543	0.6831832	Mineral
Latitude	nifh_Cluster299	0.0025	0.0006	0.025	-0.05	0.5733	0.8231248	Mineral
Latitude	nifh_Cluster303	0.002	-0.0012	0.1701	0.1063	0.1265	0.3896426	Mineral
Latitude	nifh_Cluster304	0.0025	-0.0004	0.0105	-0.0657	0.7168	0.8791838	Mineral
Latitude	nifh_Cluster313	0.0129	0.0033	0.0267	-0.0481	0.5603	0.8163038	Mineral
Latitude	nifh_Cluster314	0.0027	0.0015	0.1335	0.0669	0.1804	0.4906141	Mineral
Latitude	nifh_Cluster317	0.0089	-0.0034	0.146	0.0803	0.1599	0.4666151	Mineral
Latitude	nifh_Cluster32	0.0029	-0.0015	0.187	0.1245	0.1074	0.3444109	Mineral
Latitude	nifh_Cluster320	13.944	0.2199	0.0069	-0.0695	0.7685	0.9127308	Mineral
Latitude	nifh_Cluster334	0.0084	0.0082	0.3398	0.289	0.0226	0.1435602	Mineral
Latitude	nifh_Cluster335	0.0059	0.0054	0.2906	0.236	0.0381	0.1921712	Mineral
Latitude	nifh_Cluster338	0.0151	-0.0024	0.0383	-0.0357	0.4848	0.7740758	Mineral
Latitude	nifh_Cluster340	0.0041	1.00E-04	0.0004	-0.0764	0.9408	0.9754359	Mineral
Latitude	nifh_Cluster341	0.0021	-0.0021	0.3233	0.2713	0.027	0.1591897	Mineral
Latitude	nifh_Cluster35	0.0079	-0.0016	0.0611	-0.0111	0.3744	0.7026992	Mineral
Latitude	nifh_Cluster351	0.0035	0.0029	0.2697	0.2135	0.0473	0.2231286	Mineral
Latitude	nifh_Cluster3574	0.1191	0.0354	0.3166	0.264	0.029	0.1657144	Mineral
Latitude	nifh_Cluster3691	0.0224	-0.0052	0.0442	-0.0293	0.4518	0.7594138	Mineral
Latitude	nifh_Cluster37	0.0374	0.0008	0.0004	-0.0765	0.9459	0.9754359	Mineral
Latitude	nifh_Cluster374	0.0029	-0.0011	0.0527	-0.0202	0.4106	0.7430972	Mineral
Latitude	nifh_Cluster38	0.0008	-1.00E-04	0.0027	-0.074	0.8542	0.9485146	Mineral
Latitude	nifh_Cluster382	0.0023	-0.0002	0.001	-0.0758	0.9098	0.9661459	Mineral
Latitude	nifh_Cluster384	0.0023	-0.0009	0.0692	-0.0024	0.3435	0.6801332	Mineral
Latitude	nifh_Cluster386	0.0041	0.0044	0.3793	0.3316	0.0145	0.1141129	Mineral
Latitude	nifh_Cluster389	0.003	0.0024	0.143	0.0771	0.1645	0.4754445	Mineral
Latitude	nifh_Cluster394	0.0027	0.0021	0.1265	0.0593	0.1933	0.5024191	Mineral
Latitude	nifh_Cluster40	0.0014	0.0004	0.0234	-0.0517	0.586	0.8287537	Mineral
Latitude	nifh_Cluster43	0.0107	-0.0015	0.028	-0.0468	0.5513	0.8157013	Mineral
Latitude	nifh_Cluster434	0.0048	-0.0014	0.0422	-0.0315	0.4626	0.7666798	Mineral
Latitude	nifh_Cluster440	0.0171	0.0068	0.1358	0.0694	0.1765	0.4864908	Mineral
Latitude	nifh_Cluster462	0.0048	0.0039	0.3479	0.2978	0.0206	0.1389259	Mineral
Latitude	nifh_Cluster470	0.017	-0.002	0.0188	-0.0567	0.6263	0.8399397	Mineral
Latitude	nifh_Cluster49	0.0223	-0.0066	0.0381	-0.0359	0.4855	0.7740758	Mineral
Latitude	nifh_Cluster499	0.0028	0.0009	0.0453	-0.0281	0.4463	0.7579666	Mineral
Latitude	nifh_Cluster5	0.0263	-0.003	0.0448	-0.0287	0.4488	0.7579666	Mineral
Latitude	nifh_Cluster506	0.0058	-0.0014	0.0483	-0.0249	0.4313	0.7526909	Mineral
Latitude	nifh_Cluster51	0.0031	0.002	0.1071	0.0384	0.2337	0.5605812	Mineral
Latitude	nifh_Cluster52	0.0396	0.004	0.0069	-0.0695	0.768	0.9127308	Mineral
Latitude	nifh_Cluster525	0.0025	0.0024	0.2074	0.1464	0.088	0.322504	Mineral
Latitude	nifh_Cluster531	0.0129	0.0074	0.221	0.1611	0.077	0.2988429	Mineral
Latitude	nifh_Cluster539	0.0056	0.0061	0.2333	0.1744	0.0681	0.2792224	Mineral
Latitude	nifh_Cluster578	0.0593	0.0019	0.0027	-0.074	0.8531	0.9485146	Mineral
Latitude	nifh_Cluster58	0.2389	-0.0111	0.0013	-0.0755	0.8985	0.9616149	Mineral
Latitude	nifh_Cluster60	0.011	-0.003	0.0543	-0.0185	0.4034	0.7381723	Mineral
Latitude	nifh_Cluster61	0.0017	0.0007	0.073	0.0017	0.33	0.6714789	Mineral
Latitude	nifh_Cluster65	0.0085	0.0028	0.0811	0.0105	0.3035	0.6432892	Mineral
Latitude	nifh_Cluster686	0.0013	0.0012	0.2387	0.1802	0.0646	0.2683858	Mineral
Latitude	nifh_Cluster69	0.0092	-0.007	0.2634	0.2067	0.0504	0.2286972	Mineral
Latitude	nifh_Cluster70	0.0144	0.0048	0.0407	-0.0331	0.4708	0.7699471	Mineral
Latitude	nifh_Cluster717	0.0024	-0.0005	0.0165	-0.0591	0.648	0.844867	Mineral
Latitude	nifh_Cluster725	0.0035	-0.0013	0.0992	0.0299	0.253	0.585331	Mineral

Latitude	nifh_Cluster727	0.0038	-0.0009	0.0381	-0.0359	0.4858	0.7740758	Mineral
Latitude	nifh_Cluster73	0.0133	0.0051	0.1096	0.0411	0.2281	0.5589457	Mineral
Latitude	nifh_Cluster74	0.005	0.0011	0.0201	-0.0552	0.6139	0.8364341	Mineral
Latitude	nifh_Cluster748	0.0104	0.0012	0.0327	-0.0417	0.5191	0.7914343	Mineral
Latitude	nifh_Cluster749	0.0055	0.0015	0.0549	-0.0178	0.4005	0.7380322	Mineral
Latitude	nifh_Cluster755	0.0118	0.0002	0.0003	-0.0766	0.9506	0.9754359	Mineral
Latitude	nifh_Cluster76	0.0033	-0.0015	0.1046	0.0357	0.2397	0.5726043	Mineral
Latitude	nifh_Cluster79	0.002	0.0006	0.0457	-0.0277	0.4443	0.757638	Mineral
Latitude	nifh_Cluster81	0.0038	0.0019	0.1021	0.033	0.2458	0.5828896	Mineral
Latitude	nifh_Cluster82	0.0014	-0.0004	0.0188	-0.0567	0.6264	0.8399397	Mineral
Latitude	nifh_Cluster83	0.0055	-0.0022	0.0997	0.0305	0.2515	0.5848992	Mineral
Latitude	nifh_Cluster86	0.0161	-0.0065	0.2214	0.1615	0.0767	0.2988429	Mineral
Latitude	nifh_Cluster868	0.0098	-0.0024	0.0666	-0.0052	0.3529	0.6831832	Mineral
Latitude	nifh_Cluster87	0.003	0.0007	0.0226	-0.0526	0.593	0.8287537	Mineral
Latitude	nifh_Cluster88	0.0054	-0.0015	0.039	-0.035	0.4807	0.7740758	Mineral
Latitude	nifh_Cluster9	0.0077	-0.0058	0.3139	0.2611	0.0298	0.1665718	Mineral
Latitude	nifh_Cluster92	0.0068	0.0031	0.0792	0.0084	0.3096	0.6432892	Mineral
Latitude	nifh_Cluster93	0.0035	0.0007	0.0227	-0.0524	0.5917	0.8287537	Mineral
Latitude	nifh_Cluster94	0.005	0.0005	0.0044	-0.0722	0.8146	0.9379654	Mineral
Latitude	nifh_Cluster95	0.0149	0.0111	0.2986	0.2447	0.0351	0.1814105	Mineral
Latitude	nifh_Cluster96	0.0131	-0.001	0.0056	-0.0709	0.7912	0.9280703	Mineral
Latitude	nifh_Cluster97	0.0125	0.0041	0.3133	0.2605	0.03	0.1665718	Mineral
Latitude	nifh_Cluster98	0.0049	-0.0012	0.0486	-0.0245	0.4296	0.7526909	Mineral
Latitude	nifh_Cluster99	0.0035	0.0006	0.0109	-0.0651	0.7106	0.8780464	Mineral
Latitude	nirk_Cluster0	6.5686	0.4544	0.2149	0.1545	0.0818	0.3101491	Mineral
Latitude	nirk_Cluster1	0.2105	0.0179	0.0223	-0.0529	0.595	0.8287537	Mineral
Latitude	nirk_Cluster10	0.6649	-0.0792	0.1138	0.0457	0.2188	0.5464117	Mineral
Latitude	nirk_Cluster101	0.004	-0.0028	0.2635	0.2068	0.0503	0.2286972	Mineral
Latitude	nirk_Cluster102	0.055	0.0021	0.0032	-0.0735	0.842	0.9479523	Mineral
Latitude	nirk_Cluster103	0.09	0.0276	0.281	0.2256	0.0421	0.2053494	Mineral
Latitude	nirk_Cluster104	0.0028	1.00E-04	0.0004	-0.0765	0.9435	0.9754359	Mineral
Latitude	nirk_Cluster105	0.1172	0.0078	0.0125	-0.0634	0.6911	0.8720941	Mineral
Latitude	nirk_Cluster106	0.073	0.0288	0.2039	0.1427	0.0911	0.3236878	Mineral
Latitude	nirk_Cluster107	0.1123	0.0022	0.0009	-0.0759	0.9147	0.9661459	Mineral
Latitude	nirk_Cluster108	0.0184	0.0006	0.0013	-0.0756	0.8997	0.9616149	Mineral
Latitude	nirk_Cluster109	0.0158	0.0011	0.0071	-0.0693	0.766	0.9127308	Mineral
Latitude	nirk_Cluster11	0.1509	0.0018	0.0031	-0.0736	0.8437	0.9479523	Mineral
Latitude	nirk_Cluster12	0.1052	-0.0065	0.0221	-0.0531	0.5971	0.8288571	Mineral
Latitude	nirk_Cluster13	0.0418	-0.0013	0.0066	-0.0698	0.7738	0.9130755	Mineral
Latitude	nirk_Cluster14	0.1084	0.0084	0.0634	-0.0087	0.3654	0.6955098	Mineral
Latitude	nirk_Cluster15	0.4141	0.1279	0.2887	0.2339	0.0389	0.1944261	Mineral
Latitude	nirk_Cluster16	0.5816	0.2141	0.3443	0.2939	0.0215	0.1407517	Mineral
Latitude	nirk_Cluster17	0.1044	-0.0185	0.2597	0.2028	0.0523	0.231991	Mineral
Latitude	nirk_Cluster18	0.1461	0.009	0.0089	-0.0673	0.7377	0.8937701	Mineral
Latitude	nirk_Cluster19	0.2953	0.0455	0.3039	0.2503	0.0332	0.1747216	Mineral
Latitude	nirk_Cluster2	0.2004	0.034	0.2496	0.1919	0.0579	0.2494221	Mineral
Latitude	nirk_Cluster20	0.1934	-0.0143	0.0426	-0.0311	0.4607	0.7655881	Mineral
Latitude	nirk_Cluster21	0.1711	-0.0055	0.0156	-0.0601	0.6574	0.846898	Mineral
Latitude	nirk_Cluster22	0.1469	0.0277	0.1994	0.1378	0.0952	0.3292636	Mineral
Latitude	nirk_Cluster23	0.0729	-0.0015	0.0046	-0.0719	0.8093	0.9379654	Mineral
Latitude	nirk_Cluster24	0.0753	0.0036	0.016	-0.0597	0.653	0.844867	Mineral
Latitude	nirk_Cluster25	0.5519	-0.1027	0.375	0.3269	0.0152	0.1168073	Mineral
Latitude	nirk_Cluster26	0.1119	0.0067	0.0389	-0.035	0.481	0.7740758	Mineral
Latitude	nirk_Cluster27	0.2066	0.0093	0.0115	-0.0645	0.7033	0.8780464	Mineral
Latitude	nirk_Cluster28	0.0808	0.0091	0.0651	-0.0068	0.3586	0.6869291	Mineral

Latitude	nirk_Cluster29	0.162	-0.0225	0.1286	0.0616	0.1893	0.4963568	Mineral
Latitude	nirk_Cluster3	0.3427	0.0552	0.3195	0.2671	0.0281	0.1626487	Mineral
Latitude	nirk_Cluster30	0.0712	0.0237	0.3554	0.3058	0.019	0.1322235	Mineral
Latitude	nirk_Cluster31	0.1423	0.0091	0.0413	-0.0324	0.4675	0.7699471	Mineral
Latitude	nirk_Cluster32	0.1568	0.0166	0.0237	-0.0514	0.5838	0.8287537	Mineral
Latitude	nirk_Cluster33	0.0812	0.0152	0.0645	-0.0075	0.3612	0.6896816	Mineral
Latitude	nirk_Cluster34	0.0462	-0.003	0.0108	-0.0653	0.713	0.8782144	Mineral
Latitude	nirk_Cluster35	0.1282	-0.0424	0.1986	0.1369	0.096	0.3292636	Mineral
Latitude	nirk_Cluster36	0.0021	-1.00E-04	0.0006	-0.0763	0.9298	0.9714699	Mineral
Latitude	nirk_Cluster39	0.0045	-0.0004	0.0015	-0.0754	0.8927	0.9616149	Mineral
Latitude	nirk_Cluster4	0.1521	0.0079	0.0141	-0.0617	0.6729	0.8538155	Mineral
Latitude	nirk_Cluster40	0.0544	0.0034	0.0193	-0.0561	0.6213	0.8399397	Mineral
Latitude	nirk_Cluster41	0.1797	0.0101	0.0374	-0.0367	0.4901	0.774411	Mineral
Latitude	nirk_Cluster44	0.0756	0.0251	0.3281	0.2764	0.0256	0.1542512	Mineral
Latitude	nirk_Cluster46	0.0193	0.0026	0.0169	-0.0588	0.6447	0.844867	Mineral
Latitude	nirk_Cluster48	0.003	-0.0006	0.0169	-0.0588	0.6445	0.844867	Mineral
Latitude	nirk_Cluster49	0.0072	-0.0004	0.0013	-0.0755	0.898	0.9616149	Mineral
Latitude	nirk_Cluster5	0.2174	0.004	0.0039	-0.0727	0.8245	0.9427147	Mineral
Latitude	nirk_Cluster50	0.0014	-0.0001	0.0031	-0.0736	0.8449	0.9479523	Mineral
Latitude	nirk_Cluster52	0.0551	0.0046	0.0262	-0.0487	0.5647	0.8192031	Mineral
Latitude	nirk_Cluster53	0.0039	-0.003	0.1892	0.1268	0.1052	0.3429321	Mineral
Latitude	nirk_Cluster54	0.0024	-0.0009	0.0577	-0.0148	0.3884	0.7229311	Mineral
Latitude	nirk_Cluster55	0.0028	-0.0018	0.0975	0.0281	0.2571	0.5879389	Mineral
Latitude	nirk_Cluster56	0.0009	-0.0004	0.0321	-0.0424	0.5231	0.7954967	Mineral
Latitude	nirk_Cluster57	0.0017	-0.0011	0.0676	-0.0041	0.3494	0.6831832	Mineral
Latitude	nirk_Cluster58	0.0102	-0.0002	0.0003	-0.0766	0.9536	0.9767645	Mineral
Latitude	nirk_Cluster59	0.007	0.0014	0.0331	-0.0412	0.5162	0.7890164	Mineral
Latitude	nirk_Cluster6	0.1713	0.0165	0.0694	-0.0021	0.3426	0.6801332	Mineral
Latitude	nirk_Cluster60	0.0006	0.0002	0.0211	-0.0542	0.6059	0.8343172	Mineral
Latitude	nirk_Cluster62	0.1789	0.0175	0.0232	-0.052	0.588	0.8287537	Mineral
Latitude	nirk_Cluster63	0.1586	-0.017	0.0843	0.0139	0.2938	0.6311613	Mineral
Latitude	nirk_Cluster64	0.0043	0.0002	0.0013	-0.0755	0.8975	0.9616149	Mineral
Latitude	nirk_Cluster65	0.0531	-0.0119	0.2193	0.1592	0.0783	0.3015145	Mineral
Latitude	nirk_Cluster66	0.0095	-0.0035	0.1878	0.1253	0.1066	0.3444109	Mineral
Latitude	nirk_Cluster67	0.0232	-0.0007	0.0012	-0.0757	0.9034	0.9638112	Mineral
Latitude	nirk_Cluster68	0.0158	-0.0039	0.0968	0.0274	0.2589	0.5897988	Mineral
Latitude	nirk_Cluster69	0.0362	-0.0134	0.432	0.3883	0.0078	0.0759275	Mineral
Latitude	nirk_Cluster7	0.096	-0.013	0.0956	0.026	0.2622	0.592764	Mineral
Latitude	nirk_Cluster70	0.0892	0.0198	0.0504	-0.0227	0.4214	0.7515869	Mineral
Latitude	nirk_Cluster71	0.0071	-1.00E-04	1.00E-04	-0.0768	0.9752	0.9886337	Mineral
Latitude	nirk_Cluster72	0.0278	-0.0038	0.0586	-0.0138	0.3846	0.7180786	Mineral
Latitude	nirk_Cluster73	0.1947	-0.0526	0.3465	0.2962	0.021	0.1390693	Mineral
Latitude	nirk_Cluster74	0.8012	0.1176	0.0712	-0.0003	0.3364	0.6797972	Mineral
Latitude	nirk_Cluster75	0.0186	-0.0185	0.7429	0.7231	0.00E+00	0.0167059	Mineral
Latitude	nirk_Cluster76	0.1377	-0.0005	1.00E-04	-0.0768	0.973	0.9880257	Mineral
Latitude	nirk_Cluster77	0.0113	-0.0053	0.0701	-0.0014	0.3401	0.6801332	Mineral
Latitude	nirk_Cluster78	0.3126	0.0068	0.0023	-0.0744	0.8651	0.9558345	Mineral
Latitude	nirk_Cluster79	0.0096	-0.0022	0.0268	-0.0481	0.5602	0.8163038	Mineral
Latitude	nirk_Cluster8	0.1182	-0.0101	0.0226	-0.0526	0.5929	0.8287537	Mineral
Latitude	nirk_Cluster80	0.3544	-0.1115	0.3763	0.3283	0.015	0.1165356	Mineral
Latitude	nirk_Cluster82	0.0021	0.0004	0.0068	-0.0696	0.7704	0.9127308	Mineral
Latitude	nirk_Cluster83	0.0045	-0.001	0.0382	-0.0358	0.4849	0.7740758	Mineral
Latitude	nirk_Cluster84	0.0235	0.0097	0.1497	0.0843	0.1543	0.4529008	Mineral
Latitude	nirk_Cluster85	0.0053	-0.0037	0.3899	0.3429	0.0128	0.10521	Mineral
Latitude	nirk_Cluster86	0.0352	-0.0031	0.0145	-0.0613	0.6692	0.8524441	Mineral

Latitude	nirk_Cluster87	0.0138	-0.0036	0.0944	0.0247	0.2653	0.5952155	Mineral
Latitude	nirk_Cluster88	0.0035	-0.0005	0.0175	-0.0581	0.6387	0.844867	Mineral
Latitude	nirk_Cluster89	0.0087	0.0005	0.0029	-0.0738	0.8497	0.9485146	Mineral
Latitude	nirk_Cluster9	0.061	0.0051	0.0177	-0.0579	0.6366	0.844867	Mineral
Latitude	nirk_Cluster90	0.0087	0.0075	0.4539	0.4119	0.0059	0.0665141	Mineral
Latitude	nirk_Cluster91	0.044	0.0011	0.0017	-0.0751	0.8838	0.9616149	Mineral
Latitude	nirk_Cluster92	0.009	0.0028	0.0652	-0.0067	0.3584	0.6869291	Mineral
Latitude	nirk_Cluster93	0.0357	-0.0092	0.1302	0.0633	0.1864	0.493791	Mineral
Latitude	nirk_Cluster94	0.0222	-0.0012	0.0051	-0.0714	0.7999	0.9345938	Mineral
Latitude	nirk_Cluster95	0.0566	0.023	0.3119	0.2589	0.0305	0.1665718	Mineral
Latitude	nirk_Cluster96	0.0588	0.0192	0.2869	0.2321	0.0396	0.1963126	Mineral
Latitude	nirk_Cluster97	0.0329	0.0142	0.2937	0.2394	0.0369	0.1892053	Mineral
Latitude	nirk_Cluster98	0.0225	0.0103	0.1956	0.1338	0.0988	0.3348813	Mineral
Latitude	nirk_Cluster99	0.0074	-0.0011	0.0174	-0.0582	0.6391	0.844867	Mineral
Latitude	nirs_Cluster0	0.1586	0.0363	0.3342	0.2829	0.024	0.1490431	Mineral
Latitude	nirs_Cluster1	0.0051	0.0026	0.1003	0.0311	0.2501	0.5848992	Mineral
Latitude	nirs_Cluster14	0.0142	0.0094	0.1301	0.0632	0.1866	0.493791	Mineral
Latitude	nirs_Cluster2	0.0174	-0.0034	0.0166	-0.059	0.6471	0.844867	Mineral
Latitude	nirs_Cluster26	0.0007	0.0007	0.1687	0.1048	0.1283	0.3914169	Mineral
Latitude	nirs_Cluster28	0.0099	0.0028	0.0242	-0.0508	0.5797	0.8280766	Mineral
Latitude	nirs_Cluster3	0.1914	-0.0042	0.0069	-0.0695	0.7679	0.9127308	Mineral
Latitude	nirs_Cluster36	0.0018	-0.0004	0.0048	-0.0718	0.8063	0.9379654	Mineral
Latitude	nirs_Cluster37	0.0092	0.0081	0.1252	0.0579	0.1957	0.5040781	Mineral
Latitude	nirs_Cluster4	0.0003	0.0003	0.0785	0.0076	0.3118	0.6432892	Mineral
Latitude	nirs_Cluster50	0.0024	0.0004	0.0082	-0.0681	0.7488	0.9016193	Mineral
Latitude	nirs_Cluster9	0.0376	-0.0464	0.4684	0.4275	0.0049	0.0598955	Mineral
Latitude	norb_Cluster0	0.0848	0.0373	0.3648	0.3159	0.0171	0.1265532	Mineral
Latitude	norb_Cluster1	1.2746	0.8005	0.5697	0.5366	0.0011	0.0304821	Mineral
Latitude	norb_Cluster101	0.0037	-0.0046	0.5901	0.5586	0.0008	0.0278134	Mineral
Latitude	norb_Cluster102	0.005	0.0021	0.1246	0.0573	0.1969	0.5049849	Mineral
Latitude	norb_Cluster103	0.0025	0.0009	0.0432	-0.0304	0.4575	0.7649324	Mineral
Latitude	norb_Cluster104	0.0025	0.002	0.1308	0.064	0.1853	0.493791	Mineral
Latitude	norb_Cluster106	0.1468	-0.0233	0.0697	-0.0019	0.3418	0.6801332	Mineral
Latitude	norb_Cluster107	0.0163	-0.0058	0.1214	0.0538	0.2031	0.5141763	Mineral
Latitude	norb_Cluster108	0.0069	0.0001	0.0004	-0.0765	0.9455	0.9754359	Mineral
Latitude	norb_Cluster109	0.0282	-0.0094	0.1684	0.1044	0.1287	0.3914169	Mineral
Latitude	norb_Cluster11	0.0156	-0.0104	0.4415	0.3986	0.0069	0.071319	Mineral
Latitude	norb_Cluster110	0.0128	-0.0056	0.1773	0.114	0.118	0.3704296	Mineral
Latitude	norb_Cluster112	0.0215	-0.0072	0.2642	0.2076	0.05	0.2286972	Mineral
Latitude	norb_Cluster113	0.0157	-0.0053	0.1307	0.0638	0.1855	0.493791	Mineral
Latitude	norb_Cluster114	0.0035	0.0028	0.141	0.0749	0.1678	0.4760351	Mineral
Latitude	norb_Cluster115	0.0034	0.0006	0.0359	-0.0383	0.499	0.774411	Mineral
Latitude	norb_Cluster116	0.0083	0.0052	0.2539	0.1965	0.0555	0.2406695	Mineral
Latitude	norb_Cluster117	0.0034	0.00E+00	0.00E+00	-0.0769	0.9897	0.992349	Mineral
Latitude	norb_Cluster118	0.0044	0.0003	0.003	-0.0737	0.8455	0.9479523	Mineral
Latitude	norb_Cluster119	0.0263	-0.0152	0.3487	0.2986	0.0205	0.1389259	Mineral
Latitude	norb_Cluster12	0.005	0.0027	0.1099	0.0414	0.2275	0.5589457	Mineral
Latitude	norb_Cluster121	0.0023	-0.0024	0.2304	0.1712	0.0702	0.2854454	Mineral
Latitude	norb_Cluster122	0.0184	0.0009	0.0036	-0.0731	0.8328	0.9467697	Mineral
Latitude	norb_Cluster123	0.0071	-0.0014	0.0277	-0.0471	0.5531	0.8157013	Mineral
Latitude	norb_Cluster124	0.7452	0.3968	0.438	0.3948	0.0072	0.073228	Mineral
Latitude	norb_Cluster125	0.0052	-0.0032	0.3066	0.2533	0.0322	0.172091	Mineral
Latitude	norb_Cluster126	0.011	-0.0049	0.2102	0.1494	0.0857	0.3178384	Mineral
Latitude	norb_Cluster127	0.2041	0.0685	0.1744	0.1108	0.1215	0.3791831	Mineral
Latitude	norb_Cluster128	0.0123	0.0055	0.2294	0.1701	0.0709	0.2858062	Mineral

Latitude	norb_Cluster13	0.0051	-0.0033	0.1369	0.0706	0.1746	0.4858232	Mineral
Latitude	norb_Cluster131	0.0209	-0.0102	0.3209	0.2687	0.0277	0.1617168	Mineral
Latitude	norb_Cluster132	1.2771	0.146	0.0475	-0.0257	0.4351	0.7526909	Mineral
Latitude	norb_Cluster133	0.0023	0.0005	0.0181	-0.0574	0.6324	0.844867	Mineral
Latitude	norb_Cluster135	0.0703	0.0258	0.2624	0.2057	0.0509	0.2292918	Mineral
Latitude	norb_Cluster136	0.025	0.0043	0.0394	-0.0345	0.4784	0.7740758	Mineral
Latitude	norb_Cluster139	0.0391	0.0021	0.0052	-0.0713	0.798	0.9341496	Mineral
Latitude	norb_Cluster14	0.0037	-0.0025	0.2714	0.2153	0.0465	0.222855	Mineral
Latitude	norb_Cluster140	0.0485	0.0086	0.0667	-0.005	0.3525	0.6831832	Mineral
Latitude	norb_Cluster142	0.0018	-0.0018	0.1977	0.136	0.0968	0.3301093	Mineral
Latitude	norb_Cluster144	0.0122	-0.0007	0.004	-0.0726	0.8225	0.9422714	Mineral
Latitude	norb_Cluster146	0.0418	0.0183	0.4528	0.4107	0.006	0.0665141	Mineral
Latitude	norb_Cluster15	0.0502	0.0256	0.3378	0.2869	0.0231	0.1447509	Mineral
Latitude	norb_Cluster150	0.0199	0.0059	0.2059	0.1448	0.0893	0.322504	Mineral
Latitude	norb_Cluster155	0.1729	0.0827	0.416	0.3711	0.0094	0.0842531	Mineral
Latitude	norb_Cluster16	0.0165	-0.0118	0.3859	0.3387	0.0134	0.1086494	Mineral
Latitude	norb_Cluster164	0.0242	-0.0046	0.0379	-0.0361	0.4867	0.7740758	Mineral
Latitude	norb_Cluster165	0.0092	0.0025	0.0411	-0.0327	0.4686	0.7699471	Mineral
Latitude	norb_Cluster166	0.0046	0.00E+00	0.00E+00	-0.0769	0.9826	0.989311	Mineral
Latitude	norb_Cluster169	0.0309	-0.0057	0.0353	-0.039	0.5028	0.774411	Mineral
Latitude	norb_Cluster17	0.0035	-0.0018	0.151	0.0857	0.1522	0.4491273	Mineral
Latitude	norb_Cluster170	0.0299	0.0089	0.0737	0.0025	0.3275	0.6686846	Mineral
Latitude	norb_Cluster171	0.0089	-0.0085	0.4145	0.3695	0.0096	0.0844796	Mineral
Latitude	norb_Cluster172	0.0179	-0.0002	1.00E-04	-0.0769	0.9787	0.989311	Mineral
Latitude	norb_Cluster175	0.0595	-0.0026	0.0017	-0.0751	0.8833	0.9616149	Mineral
Latitude	norb_Cluster176	0.0392	-0.0151	0.1347	0.0682	0.1783	0.4893958	Mineral
Latitude	norb_Cluster177	0.0361	0.0001	0.00E+00	-0.0769	0.9907	0.992349	Mineral
Latitude	norb_Cluster179	0.003	0.0006	0.015	-0.0608	0.6637	0.8512971	Mineral
Latitude	norb_Cluster18	0.0047	-0.0024	0.2055	0.1444	0.0896	0.322504	Mineral
Latitude	norb_Cluster180	0.0133	-0.0049	0.2065	0.1455	0.0888	0.322504	Mineral
Latitude	norb_Cluster181	0.0003	0.0004	0.0785	0.0076	0.3118	0.6432892	Mineral
Latitude	norb_Cluster182	0.0018	-0.0008	0.0741	0.0029	0.3263	0.6685074	Mineral
Latitude	norb_Cluster184	0.0175	-0.0004	0.0006	-0.0762	0.9283	0.9714699	Mineral
Latitude	norb_Cluster186	0.0011	0.001	0.1361	0.0696	0.176	0.4864908	Mineral
Latitude	norb_Cluster19	0.0058	-0.0022	0.1526	0.0874	0.15	0.4446631	Mineral
Latitude	norb_Cluster192	0.0221	-0.0029	0.0162	-0.0594	0.651	0.844867	Mineral
Latitude	norb_Cluster193	0.0022	-0.0008	0.048	-0.0252	0.4327	0.7526909	Mineral
Latitude	norb_Cluster197	0.0398	0.0281	0.0948	0.0252	0.2641	0.5947713	Mineral
Latitude	norb_Cluster2	0.0032	-0.0024	0.1833	0.1205	0.1113	0.3550055	Mineral
Latitude	norb_Cluster20	0.0665	0.0306	0.3634	0.3144	0.0174	0.1265532	Mineral
Latitude	norb_Cluster208	0.0015	-0.0007	0.0464	-0.0269	0.4406	0.7556857	Mineral
Latitude	norb_Cluster21	0.0542	0.0295	0.4443	0.4015	0.0067	0.0701141	Mineral
Latitude	norb_Cluster211	0.0141	0.0113	0.1674	0.1033	0.13	0.3931888	Mineral
Latitude	norb_Cluster212	0.0252	-0.0002	0.00E+00	-0.0769	0.9813	0.989311	Mineral
Latitude	norb_Cluster214	0.0163	0.0077	0.1019	0.0329	0.2461	0.5828896	Mineral
Latitude	norb_Cluster217	0.0186	0.0028	0.0098	-0.0664	0.7257	0.8846062	Mineral
Latitude	norb_Cluster219	0.0108	0.0042	0.0473	-0.026	0.4363	0.7526909	Mineral
Latitude	norb_Cluster22	0.0028	0.001	0.0289	-0.0458	0.5449	0.8157013	Mineral
Latitude	norb_Cluster220	0.0248	0.0145	0.1079	0.0393	0.2318	0.5605812	Mineral
Latitude	norb_Cluster221	0.0248	0.0103	0.047	-0.0263	0.4376	0.7527699	Mineral
Latitude	norb_Cluster222	0.0104	0.0076	0.1076	0.0389	0.2327	0.5605812	Mineral
Latitude	norb_Cluster224	0.0238	0.0025	0.0043	-0.0723	0.8168	0.9379654	Mineral
Latitude	norb_Cluster225	0.0095	0.0013	0.0122	-0.0638	0.6951	0.8744197	Mineral
Latitude	norb_Cluster226	0.0049	-0.0023	0.0996	0.0304	0.2518	0.5848992	Mineral
Latitude	norb_Cluster228	0.0009	-0.0016	0.1907	0.1284	0.1036	0.3397356	Mineral

Latitude	norb_Cluster229	0.004	-0.0008	0.021	-0.0543	0.6064	0.8343172	Mineral
Latitude	norb_Cluster230	0.0019	-0.0017	0.2235	0.1637	0.0752	0.295614	Mineral
Latitude	norb_Cluster231	0.0061	0.0013	0.017	-0.0586	0.6429	0.844867	Mineral
Latitude	norb_Cluster232	0.0044	0.0012	0.0334	-0.041	0.5144	0.7883713	Mineral
Latitude	norb_Cluster234	0.017	-0.0124	0.3552	0.3056	0.019	0.1322235	Mineral
Latitude	norb_Cluster235	0.0012	1.00E-04	0.001	-0.0758	0.911	0.9661459	Mineral
Latitude	norb_Cluster238	0.0135	-0.0159	0.6338	0.6057	0.0004	0.0257142	Mineral
Latitude	norb_Cluster239	0.0109	-0.013	0.5085	0.4707	0.0028	0.0418851	Mineral
Latitude	norb_Cluster24	0.3014	0.1458	0.4655	0.4244	0.0051	0.0598955	Mineral
Latitude	norb_Cluster244	0.0015	-0.0016	0.3476	0.2974	0.0207	0.1389259	Mineral
Latitude	norb_Cluster247	0.006	-0.0017	0.0831	0.0125	0.2976	0.6360896	Mineral
Latitude	norb_Cluster25	0.1263	0.0715	0.4476	0.4051	0.0064	0.0691537	Mineral
Latitude	norb_Cluster250	0.0146	-0.0025	0.0301	-0.0445	0.536	0.8047332	Mineral
Latitude	norb_Cluster251	0.0064	-0.0038	0.2113	0.1506	0.0848	0.3164845	Mineral
Latitude	norb_Cluster26	0.1134	0.0601	0.4193	0.3747	0.0091	0.0821729	Mineral
Latitude	norb_Cluster28	0.0355	0.0003	0.0001	-0.0768	0.9663	0.9831072	Mineral
Latitude	norb_Cluster29	0.2454	0.0295	0.0677	-0.004	0.3488	0.6831832	Mineral
Latitude	norb_Cluster31	0.0632	0.0401	0.6736	0.6485	0.0002	0.0257142	Mineral
Latitude	norb_Cluster32	0.1401	0.0966	0.5478	0.513	0.0016	0.0338405	Mineral
Latitude	norb_Cluster33	0.434	0.2105	0.6292	0.6007	0.0004	0.0257142	Mineral
Latitude	norb_Cluster35	0.0746	0.0379	0.4202	0.3756	0.009	0.0821729	Mineral
Latitude	norb_Cluster36	1.1002	0.6681	0.5673	0.534	0.0012	0.0304821	Mineral
Latitude	norb_Cluster37	0.7173	0.3603	0.5841	0.5521	0.0009	0.0282093	Mineral
Latitude	norb_Cluster38	0.0138	0.0047	0.2022	0.1408	0.0927	0.3253785	Mineral
Latitude	norb_Cluster39	0.0465	0.0109	0.0898	0.0198	0.2779	0.6088651	Mineral
Latitude	norb_Cluster40	0.2213	0.0934	0.3121	0.2591	0.0304	0.1665718	Mineral
Latitude	norb_Cluster42	0.2825	0.1899	0.7172	0.6954	1.00E-04	0.0167059	Mineral
Latitude	norb_Cluster44	0.0249	-0.0117	0.2393	0.1808	0.0642	0.2683858	Mineral
Latitude	norb_Cluster46	0.2575	0.1571	0.5033	0.4651	0.0031	0.0429353	Mineral
Latitude	norb_Cluster47	0.6453	0.3224	0.4836	0.4439	0.004	0.0536015	Mineral
Latitude	norb_Cluster5	0.0115	0.0042	0.1873	0.1248	0.1071	0.3444109	Mineral
Latitude	norb_Cluster51	0.0122	-0.0076	0.2028	0.1415	0.0921	0.3252643	Mineral
Latitude	norb_Cluster52	0.2809	0.1621	0.5199	0.483	0.0024	0.0415283	Mineral
Latitude	norb_Cluster56	0.0178	0.0039	0.0701	-0.0014	0.3403	0.6801332	Mineral
Latitude	norb_Cluster57	0.004	0.0009	0.0489	-0.0243	0.4284	0.7526909	Mineral
Latitude	norb_Cluster59	0.0322	-0.023	0.3973	0.3509	0.0118	0.0983215	Mineral
Latitude	norb_Cluster6	0.6934	0.3879	0.5481	0.5133	0.0016	0.0338405	Mineral
Latitude	norb_Cluster60	0.345	0.2954	0.5806	0.5484	0.001	0.0283317	Mineral
Latitude	norb_Cluster61	0.5486	-0.1519	0.1376	0.0713	0.1734	0.4849558	Mineral
Latitude	norb_Cluster62	0.003	0.0005	0.0125	-0.0635	0.6918	0.8720941	Mineral
Latitude	norb_Cluster64	0.0407	0.0026	0.011	-0.0651	0.7096	0.8780464	Mineral
Latitude	norb_Cluster66	0.0016	0.0014	0.2006	0.1391	0.0941	0.3284779	Mineral
Latitude	norb_Cluster67	0.0279	-0.0059	0.0665	-0.0053	0.3534	0.6831832	Mineral
Latitude	norb_Cluster68	0.003	-0.0016	0.1456	0.0798	0.1605	0.4666151	Mineral
Latitude	norb_Cluster69	0.0032	0.0023	0.2464	0.1884	0.0598	0.2548116	Mineral
Latitude	norb_Cluster7	0.0786	0.0373	0.3159	0.2633	0.0292	0.1657144	Mineral
Latitude	norb_Cluster70	0.1262	0.024	0.0919	0.022	0.2722	0.5991845	Mineral
Latitude	norb_Cluster71	0.1512	-0.0109	0.0161	-0.0595	0.6518	0.844867	Mineral
Latitude	norb_Cluster74	0.0387	-0.0018	0.0057	-0.0708	0.7893	0.9276422	Mineral
Latitude	norb_Cluster75	0.1531	-0.0029	0.0014	-0.0754	0.8939	0.9616149	Mineral
Latitude	norb_Cluster76	0.1215	0.1074	0.4779	0.4377	0.0043	0.0555827	Mineral
Latitude	norb_Cluster77	0.0492	0.0111	0.142	0.076	0.1663	0.4760351	Mineral
Latitude	norb_Cluster8	0.0206	0.0029	0.0249	-0.0501	0.5743	0.8231248	Mineral
Latitude	norb_Cluster80	0.0021	-0.0009	0.0516	-0.0214	0.4157	0.7483419	Mineral
Latitude	norb_Cluster81	0.0013	0.00E+00	0.00E+00	-0.0769	0.9823	0.989311	Mineral

Latitude	norb_Cluster83	0.011	-0.0121	0.4275	0.3834	0.0082	0.0768221	Mineral
Latitude	norb_Cluster84	0.0093	0.0007	0.0045	-0.0721	0.8127	0.9379654	Mineral
Latitude	norb_Cluster85	0.0025	0.0012	0.0764	0.0054	0.3186	0.6548989	Mineral
Latitude	norb_Cluster86	0.041	0.0236	0.1255	0.0583	0.1951	0.5040781	Mineral
Latitude	norb_Cluster88	0.0019	-1.00E-04	0.0003	-0.0766	0.9484	0.9754359	Mineral
Latitude	norb_Cluster90	0.0013	-0.0018	0.2146	0.1542	0.082	0.3101491	Mineral
Latitude	norb_Cluster91	0.7571	-0.1619	0.0667	-0.005	0.3525	0.6831832	Mineral
Latitude	norb_Cluster92	0.0021	0.0011	0.0536	-0.0191	0.4062	0.7396584	Mineral
Latitude	norb_Cluster93	0.0028	0.0018	0.1072	0.0385	0.2336	0.5605812	Mineral
Latitude	norb_Cluster94	0.002	-0.001	0.0924	0.0226	0.2708	0.5983433	Mineral
Latitude	norb_Cluster96	0.0006	-0.0007	0.1933	0.1313	0.101	0.3386744	Mineral
Latitude	norb_Cluster97	0.0162	-0.0002	0.0001	-0.0768	0.9664	0.9831072	Mineral
Latitude	norb_Cluster98	0.0176	0.0086	0.1595	0.0948	0.1403	0.4201778	Mineral
Latitude	nosz_Cluster0	0.0057	-0.0007	0.0111	-0.0649	0.7081	0.8780464	Mineral
Latitude	nosz_Cluster10	0.0128	0.0024	0.0353	-0.0389	0.5026	0.774411	Mineral
Latitude	nosz_Cluster102	0.0057	-0.0023	0.2553	0.198	0.0547	0.2391111	Mineral
Latitude	nosz_Cluster103	0.0496	-0.0157	0.1721	0.1084	0.1242	0.385542	Mineral
Latitude	nosz_Cluster105	0.0016	-1.00E-04	0.0006	-0.0763	0.9303	0.9714699	Mineral
Latitude	nosz_Cluster106	0.0088	-0.0058	0.3255	0.2736	0.0264	0.1571404	Mineral
Latitude	nosz_Cluster107	0.0003	0.0003	0.0785	0.0076	0.3118	0.6432892	Mineral
Latitude	nosz_Cluster108	0.0043	0.0004	0.0037	-0.073	0.8302	0.9456064	Mineral
Latitude	nosz_Cluster109	0.0416	-0.0064	0.0853	0.0149	0.2909	0.6309061	Mineral
Latitude	nosz_Cluster11	0.0104	0.0007	0.0047	-0.0719	0.8091	0.9379654	Mineral
Latitude	nosz_Cluster110	0.0004	0.0003	0.0303	-0.0443	0.535	0.8047332	Mineral
Latitude	nosz_Cluster111	0.0078	-0.0044	0.3328	0.2814	0.0244	0.1497636	Mineral
Latitude	nosz_Cluster116	0.0074	0.0002	0.0014	-0.0754	0.8932	0.9616149	Mineral
Latitude	nosz_Cluster118	0.0064	0.0025	0.0798	0.009	0.3077	0.6432892	Mineral
Latitude	nosz_Cluster119	0.0019	-0.0023	0.4775	0.4373	0.0043	0.0555827	Mineral
Latitude	nosz_Cluster120	0.0083	-0.005	0.331	0.2795	0.0248	0.1511406	Mineral
Latitude	nosz_Cluster123	0.0136	-0.0093	0.431	0.3872	0.0079	0.0759275	Mineral
Latitude	nosz_Cluster128	0.005	0.0025	0.131	0.0641	0.185	0.493791	Mineral
Latitude	nosz_Cluster129	0.05	0.0001	0.00E+00	-0.0769	0.9846	0.9896656	Mineral
Latitude	nosz_Cluster13	0.0124	0.0059	0.2242	0.1645	0.0746	0.2954047	Mineral
Latitude	nosz_Cluster130	0.1138	0.0521	0.0531	-0.0197	0.4084	0.7414708	Mineral
Latitude	nosz_Cluster132	0.004	-0.0015	0.1208	0.0531	0.2044	0.5153147	Mineral
Latitude	nosz_Cluster14	0.0149	0.0045	0.0571	-0.0154	0.3911	0.7256538	Mineral
Latitude	nosz_Cluster16	0.008	0.0052	0.2048	0.1437	0.0903	0.3228037	Mineral
Latitude	nosz_Cluster17	0.0427	-0.0086	0.0677	-0.004	0.349	0.6831832	Mineral
Latitude	nosz_Cluster18	0.0184	-0.0029	0.0179	-0.0576	0.6342	0.844867	Mineral
Latitude	nosz_Cluster20	0.0117	0.0006	0.0015	-0.0753	0.8901	0.9616149	Mineral
Latitude	nosz_Cluster21	0.0117	-0.0038	0.0692	-0.0024	0.3433	0.6801332	Mineral
Latitude	nosz_Cluster22	0.0325	0.019	0.1699	0.1061	0.1268	0.3896426	Mineral
Latitude	nosz_Cluster23	0.019	-0.0063	0.0895	0.0195	0.2786	0.6088651	Mineral
Latitude	nosz_Cluster24	0.0019	0.0007	0.0352	-0.039	0.5029	0.774411	Mineral
Latitude	nosz_Cluster25	0.0026	0.0002	0.0025	-0.0742	0.8584	0.9501979	Mineral
Latitude	nosz_Cluster26	0.0068	0.0013	0.019	-0.0565	0.6242	0.8399397	Mineral
Latitude	nosz_Cluster27	0.007	-0.0012	0.0248	-0.0502	0.5748	0.8231248	Mineral
Latitude	nosz_Cluster28	0.0358	0.0046	0.0206	-0.0547	0.6095	0.8343172	Mineral
Latitude	nosz_Cluster30	0.0341	0.0009	0.001	-0.0759	0.9131	0.9661459	Mineral
Latitude	nosz_Cluster32	0.016	0.0015	0.0099	-0.0663	0.7246	0.8846062	Mineral
Latitude	nosz_Cluster33	0.0187	0.0069	0.1016	0.0325	0.247	0.5828896	Mineral
Latitude	nosz_Cluster34	0.0387	0.0052	0.0109	-0.0652	0.7114	0.8780464	Mineral
Latitude	nosz_Cluster35	0.0097	0.0015	0.0207	-0.0546	0.6088	0.8343172	Mineral
Latitude	nosz_Cluster36	0.0213	0.0051	0.0616	-0.0106	0.3724	0.7026992	Mineral
Latitude	nosz_Cluster37	0.0077	0.0008	0.0079	-0.0684	0.753	0.9048139	Mineral

Latitude	nosz_Cluster38	0.0144	-0.0094	0.2248	0.1652	0.0742	0.2954047	Mineral
Latitude	nosz_Cluster39	0.0653	-0.0077	0.0218	-0.0535	0.5999	0.8308126	Mineral
Latitude	nosz_Cluster40	0.0262	0.0018	0.0046	-0.0719	0.8095	0.9379654	Mineral
Latitude	nosz_Cluster41	0.0258	0.0005	0.0003	-0.0766	0.9506	0.9754359	Mineral
Latitude	nosz_Cluster42	0.03	0.004	0.0163	-0.0594	0.6507	0.844867	Mineral
Latitude	nosz_Cluster43	0.001	-0.0007	0.0619	-0.0102	0.3712	0.7026992	Mineral
Latitude	nosz_Cluster44	0.0068	-0.0001	0.0002	-0.0767	0.9626	0.9831072	Mineral
Latitude	nosz_Cluster45	0.0056	0.0013	0.037	-0.0371	0.4921	0.774411	Mineral
Latitude	nosz_Cluster46	0.0082	0.0026	0.1287	0.0617	0.1892	0.4963568	Mineral
Latitude	nosz_Cluster47	0.0012	0.0002	0.0061	-0.0704	0.7826	0.9216351	Mineral
Latitude	nosz_Cluster48	0.0028	-0.0018	0.1265	0.0594	0.1932	0.5024191	Mineral
Latitude	nosz_Cluster49	0.0012	0.0005	0.0374	-0.0366	0.4897	0.774411	Mineral
Latitude	nosz_Cluster5	0.004	0.0019	0.0797	0.0089	0.308	0.6432892	Mineral
Latitude	nosz_Cluster51	0.003	0.0014	0.1091	0.0405	0.2293	0.5590776	Mineral
Latitude	nosz_Cluster53	0.0012	0.0005	0.0208	-0.0545	0.6081	0.8343172	Mineral
Latitude	nosz_Cluster54	0.0037	-0.0008	0.0194	-0.0561	0.6207	0.8399397	Mineral
Latitude	nosz_Cluster57	0.2232	0.1357	0.5119	0.4743	0.0027	0.0418851	Mineral
Latitude	nosz_Cluster58	0.0053	-0.0038	0.2188	0.1587	0.0787	0.3015145	Mineral
Latitude	nosz_Cluster59	0.0008	-0.0014	0.1907	0.1284	0.1036	0.3397356	Mineral
Latitude	nosz_Cluster6	0.0034	0.002	0.1239	0.0565	0.1982	0.506233	Mineral
Latitude	nosz_Cluster60	0.0099	-0.0011	0.0093	-0.067	0.7331	0.8899491	Mineral
Latitude	nosz_Cluster61	0.0178	-0.0035	0.0936	0.0239	0.2675	0.5954729	Mineral
Latitude	nosz_Cluster62	0.0493	0.0303	0.4338	0.3902	0.0076	0.0758409	Mineral
Latitude	nosz_Cluster64	0.0023	-0.0012	0.0367	-0.0374	0.494	0.774411	Mineral
Latitude	nosz_Cluster65	0.0083	0.0008	0.0069	-0.0694	0.7678	0.9127308	Mineral
Latitude	nosz_Cluster66	0.0428	0.032	0.5254	0.4888	0.0022	0.0412228	Mineral
Latitude	nosz_Cluster67	0.0669	0.0504	0.5384	0.5029	0.0018	0.0376008	Mineral
Latitude	nosz_Cluster69	0.008	0.0005	0.003	-0.0737	0.8467	0.9479523	Mineral
Latitude	nosz_Cluster70	0.0655	0.0298	0.0789	0.0081	0.3105	0.6432892	Mineral
Latitude	nosz_Cluster71	0.0101	0.0025	0.0303	-0.0443	0.5348	0.8047332	Mineral
Latitude	nosz_Cluster72	0.066	0.0405	0.4468	0.4043	0.0064	0.0691537	Mineral
Latitude	nosz_Cluster73	0.0031	-0.0018	0.1381	0.0718	0.1727	0.4849558	Mineral
Latitude	nosz_Cluster74	0.0017	-0.0026	0.6225	0.5935	0.0005	0.0257142	Mineral
Latitude	nosz_Cluster75	0.132	0.0777	0.0719	0.0005	0.334	0.6770954	Mineral
Latitude	nosz_Cluster77	0.0002	0.0002	0.0303	-0.0443	0.535	0.8047332	Mineral
Latitude	nosz_Cluster78	0.0054	-0.0014	0.0512	-0.0218	0.4173	0.7483419	Mineral
Latitude	nosz_Cluster79	0.0401	0.0048	0.0228	-0.0523	0.591	0.8287537	Mineral
Latitude	nosz_Cluster8	0.0106	0.0002	0.0007	-0.0762	0.9266	0.9714699	Mineral
Latitude	nosz_Cluster80	0.0039	-0.0008	0.0239	-0.0512	0.5822	0.8287537	Mineral
Latitude	nosz_Cluster81	0.0099	0.0067	0.1656	0.1014	0.1322	0.397928	Mineral
Latitude	nosz_Cluster82	0.0023	-0.0011	0.0261	-0.0488	0.5651	0.8192031	Mineral
Latitude	nosz_Cluster83	0.0014	-0.0024	0.3643	0.3154	0.0172	0.1265532	Mineral
Latitude	nosz_Cluster85	0.0027	0.0002	0.001	-0.0759	0.9114	0.9661459	Mineral
Latitude	nosz_Cluster86	0.0038	0.0003	0.0034	-0.0733	0.8366	0.947348	Mineral
Latitude	nosz_Cluster87	0.0034	-0.0022	0.2289	0.1696	0.0712	0.2858062	Mineral
Latitude	nosz_Cluster88	0.0246	-0.0026	0.0275	-0.0473	0.5544	0.8157013	Mineral
Latitude	nosz_Cluster89	0.0607	0.0397	0.5047	0.4666	0.003	0.0429353	Mineral
Latitude	nosz_Cluster9	0.0034	-0.0003	0.0031	-0.0736	0.8433	0.9479523	Mineral
Latitude	nosz_Cluster90	0.0139	0.0012	0.0145	-0.0613	0.6687	0.8524441	Mineral
Latitude	nosz_Cluster91	0.0232	0.0049	0.0355	-0.0387	0.5014	0.774411	Mineral
Latitude	nosz_Cluster93	0.0488	-0.0231	0.3005	0.2467	0.0344	0.1794034	Mineral
Latitude	nosz_Cluster95	0.0094	0.0029	0.111	0.0426	0.2249	0.5575721	Mineral
Latitude	nosz_Cluster96	0.0064	-0.004	0.5119	0.4744	0.0027	0.0418851	Mineral
Latitude	nosz_Cluster97	0.0026	0.0001	0.0009	-0.076	0.9154	0.9661459	Mineral
Latitude	nosz_Cluster98	0.0035	-0.0012	0.013	-0.0629	0.6852	0.8675868	Mineral

Latitude	nosz_Cluster99	0.0061	-0.0001	0.0005	-0.0764	0.9392	0.9754359	Mineral
Latitude	nrfa_Cluster10	0.0978	0.0027	0.0006	-0.0762	0.9286	0.9714699	Mineral
Latitude	nrfa_Cluster100	0.1249	-0.1218	0.5882	0.5565	0.0008	0.0278134	Mineral
Latitude	nrfa_Cluster104	0.1478	-0.085	0.5561	0.522	0.0014	0.0338405	Mineral
Latitude	nrfa_Cluster105	0.006	-0.0018	0.0447	-0.0288	0.4496	0.7579666	Mineral
Latitude	nrfa_Cluster109	0.2753	-0.169	0.7076	0.6851	1.00E-04	0.0167059	Mineral
Latitude	nrfa_Cluster116	0.3208	-0.138	0.6455	0.6182	0.0003	0.0257142	Mineral
Latitude	nrfa_Cluster118	0.2298	-0.2434	0.5908	0.5593	0.0008	0.0278134	Mineral
Latitude	nrfa_Cluster125	0.1081	-0.1241	0.6179	0.5885	0.0005	0.0257142	Mineral
Latitude	nrfa_Cluster154	0.0837	-0.0821	0.6338	0.6056	0.0004	0.0257142	Mineral
Latitude	nrfa_Cluster164	0.0044	0.0001	0.0006	-0.0763	0.9327	0.9714871	Mineral
Latitude	nrfa_Cluster172	0.0036	0.0008	0.0156	-0.0601	0.6572	0.846898	Mineral
Latitude	nrfa_Cluster175	0.0053	-0.0081	0.6059	0.5755	0.0006	0.0265792	Mineral
Latitude	nrfa_Cluster20	0.045	-0.0353	0.5186	0.4816	0.0025	0.0415283	Mineral
Latitude	nrfa_Cluster26	0.1433	-0.1336	0.5131	0.4757	0.0027	0.0418851	Mineral
Latitude	nrfa_Cluster29	0.0059	0.0045	0.2706	0.2145	0.0468	0.222855	Mineral
Latitude	nrfa_Cluster34	0.0642	-0.0524	0.589	0.5574	0.0008	0.0278134	Mineral
Latitude	nrfa_Cluster38	0.0033	-0.0046	0.5318	0.4958	0.002	0.0400165	Mineral
Latitude	nrfa_Cluster61	0.0047	-0.0051	0.5284	0.4921	0.0021	0.0407043	Mineral
Latitude	nrfa_Cluster62	0.0589	-0.0683	0.6121	0.5822	0.0006	0.0257142	Mineral
Latitude	nrfa_Cluster66	0.1282	-0.068	0.649	0.622	0.0003	0.0257142	Mineral
Latitude	nrfa_Cluster69	0.0035	0.0029	0.2789	0.2235	0.043	0.2079125	Mineral
Latitude	nrfa_Cluster71	0.0322	-0.0322	0.5531	0.5187	0.0015	0.0338405	Mineral
Latitude	nrfa_Cluster72	0.0969	-0.0851	0.6122	0.5823	0.0006	0.0257142	Mineral
Latitude	nrfa_Cluster73	0.0042	-0.0013	0.0305	-0.0441	0.5338	0.8047332	Mineral
Latitude	nrfa_Cluster74	0.1692	-0.1429	0.5492	0.5145	0.0016	0.0338405	Mineral
Latitude	nrfa_Cluster76	0.0922	-0.0796	0.4566	0.4148	0.0057	0.0658388	Mineral
Latitude	nrfa_Cluster79	0.0138	0.0032	0.0114	-0.0646	0.7048	0.8780464	Mineral
Latitude	nrfa_Cluster87	0.0207	0.0104	0.0864	0.0161	0.2876	0.6262469	Mineral
Latitude	nrfa_Cluster89	0.0023	-0.001	0.0549	-0.0178	0.4007	0.7380322	Mineral
Latitude	nrfa_Cluster92	0.0041	-0.0001	0.0007	-0.0762	0.9262	0.9714699	Mineral
Latitude	nrfa_Cluster93	0.0581	0.0544	0.3648	0.316	0.0171	0.1265532	Mineral
Latitude	nrfa_Cluster94	0.0003	-0.0005	0.1907	0.1284	0.1036	0.3397356	Mineral
Latitude	nrfa_Cluster97	0.0225	-0.0098	0.2137	0.1532	0.0828	0.3110776	Mineral
Latitude	nrfa_Cluster99	0.0184	-0.0119	0.361	0.3118	0.0179	0.1284837	Mineral
Longitude	amoA_A_Cluster26	0.0053	0.0014	0.0246	-0.0504	0.5764	0.8523211	Mineral
Longitude	amoA_A_Cluster45	0.0071	0.0009	0.0094	-0.0668	0.7304	0.896443	Mineral
Longitude	amoA_B_Cluster0	0.1272	0.0171	0.1497	0.0843	0.1543	0.7069881	Mineral
Longitude	amoA_B_Cluster1	0.0205	-0.0128	0.6426	0.6151	0.0003	0.017437	Mineral
Longitude	amoA_B_Cluster10	0.0539	-0.0044	0.0289	-0.0458	0.5446	0.8471771	Mineral
Longitude	amoA_B_Cluster11	0.0486	-0.0183	0.3649	0.3161	0.0171	0.2289948	Mineral
Longitude	amoA_B_Cluster12	0.093	0.0013	0.0013	-0.0756	0.9	0.9526986	Mineral
Longitude	amoA_B_Cluster13	0.6417	0.0837	0.0527	-0.0202	0.4107	0.8052395	Mineral
Longitude	amoA_B_Cluster14	0.0339	0.0107	0.1462	0.0806	0.1595	0.7128821	Mineral
Longitude	amoA_B_Cluster15	0.0413	-0.0089	0.1153	0.0472	0.2157	0.7973679	Mineral
Longitude	amoA_B_Cluster2	0.2143	0.0022	0.0003	-0.0765	0.9474	0.9789569	Mineral
Longitude	amoA_B_Cluster20	0.0078	-0.0042	0.1719	0.1082	0.1244	0.6704217	Mineral
Longitude	amoA_B_Cluster23	0.0626	-0.0193	0.359	0.3097	0.0183	0.2341154	Mineral
Longitude	amoA_B_Cluster3	0.0244	-0.0121	0.3598	0.3106	0.0181	0.2341154	Mineral
Longitude	amoA_B_Cluster4	0.0169	-0.0026	0.0288	-0.0459	0.5456	0.8471771	Mineral
Longitude	amoA_B_Cluster5	0.1032	0.0026	0.0045	-0.0721	0.8126	0.9259972	Mineral
Longitude	amoA_B_Cluster6	0.0279	-0.007	0.1679	0.1039	0.1293	0.6704217	Mineral
Longitude	amoA_B_Cluster7	0.0232	-0.0126	0.3694	0.3209	0.0162	0.2289948	Mineral
Longitude	amoA_B_Cluster8	0.0268	-0.0197	0.6531	0.6265	0.0003	0.017437	Mineral
Longitude	amoA_B_Cluster9	0.0693	-0.0259	0.2531	0.1957	0.0559	0.4898286	Mineral

Longitude	nifh_Cluster0	0.0318	-0.0028	0.0257	-0.0493	0.5684	0.8476038	Mineral
Longitude	nifh_Cluster10	0.0033	0.0008	0.0302	-0.0444	0.5358	0.8471771	Mineral
Longitude	nifh_Cluster100	0.002	0.001	0.09	0.02	0.2772	0.8052395	Mineral
Longitude	nifh_Cluster103	0.0433	-0.0063	0.0592	-0.0132	0.3823	0.8052395	Mineral
Longitude	nifh_Cluster1034	0.0337	0.0136	0.1693	0.1054	0.1275	0.6704217	Mineral
Longitude	nifh_Cluster105	0.0164	0.0083	0.2336	0.1746	0.068	0.549524	Mineral
Longitude	nifh_Cluster108	0.0076	0.001	0.0166	-0.059	0.6468	0.8775051	Mineral
Longitude	nifh_Cluster1099	0.0478	-0.0096	0.0232	-0.0519	0.5875	0.8599568	Mineral
Longitude	nifh_Cluster11	0.0025	0.0011	0.1077	0.039	0.2324	0.8027796	Mineral
Longitude	nifh_Cluster1112	0.0037	-0.001	0.0314	-0.0431	0.5274	0.8471771	Mineral
Longitude	nifh_Cluster112	0.0098	0.0028	0.0444	-0.0291	0.4511	0.810923	Mineral
Longitude	nifh_Cluster113	0.0374	0.0007	0.0013	-0.0755	0.8982	0.9526986	Mineral
Longitude	nifh_Cluster114	0.0304	0.001	0.0055	-0.071	0.7921	0.9163049	Mineral
Longitude	nifh_Cluster1141	0.0296	0.0071	0.0846	0.0142	0.293	0.8052395	Mineral
Longitude	nifh_Cluster115	0.0098	-0.0004	0.0031	-0.0735	0.8428	0.9392862	Mineral
Longitude	nifh_Cluster116	0.0883	-0.0306	0.4247	0.3805	0.0085	0.1667487	Mineral
Longitude	nifh_Cluster1163	0.0051	-0.0019	0.1236	0.0562	0.1988	0.7720497	Mineral
Longitude	nifh_Cluster1179	0.0037	0.0024	0.1495	0.0841	0.1546	0.7069881	Mineral
Longitude	nifh_Cluster1182	0.0209	-0.0013	0.0163	-0.0594	0.6505	0.8775051	Mineral
Longitude	nifh_Cluster1183	0.0045	-0.0007	0.0256	-0.0493	0.5688	0.8476038	Mineral
Longitude	nifh_Cluster1197	0.0115	-0.0025	0.0528	-0.0201	0.4102	0.8052395	Mineral
Longitude	nifh_Cluster1199	0.0048	-0.0009	0.0241	-0.051	0.5809	0.8567643	Mineral
Longitude	nifh_Cluster120	0.02	0.0107	0.109	0.0404	0.2295	0.8027796	Mineral
Longitude	nifh_Cluster1203	0.0217	-0.0105	0.2663	0.2098	0.049	0.458431	Mineral
Longitude	nifh_Cluster1206	0.006	-0.0039	0.4778	0.4377	0.0043	0.1018479	Mineral
Longitude	nifh_Cluster1207	0.0078	0.0025	0.0698	-0.0017	0.3413	0.8052395	Mineral
Longitude	nifh_Cluster1209	0.1617	-0.0441	0.5104	0.4727	0.0028	0.0709925	Mineral
Longitude	nifh_Cluster121	0.0123	0.0046	0.055	-0.0176	0.4	0.8052395	Mineral
Longitude	nifh_Cluster1211	0.0068	-0.0053	0.6095	0.5795	0.0006	0.023295	Mineral
Longitude	nifh_Cluster1213	0.0032	-0.0005	0.0175	-0.0581	0.6385	0.8775051	Mineral
Longitude	nifh_Cluster122	0.0041	0.0016	0.0745	0.0033	0.3251	0.8052395	Mineral
Longitude	nifh_Cluster123	0.0092	0.0042	0.101	0.0319	0.2483	0.8052395	Mineral
Longitude	nifh_Cluster1236	0.031	-0.0004	1.00E-04	-0.0768	0.9733	0.9825468	Mineral
Longitude	nifh_Cluster1238	0.0034	0.0016	0.0963	0.0268	0.2603	0.8052395	Mineral
Longitude	nifh_Cluster124	0.0101	-0.001	0.0146	-0.0612	0.6683	0.8775051	Mineral
Longitude	nifh_Cluster1261	0.0023	-1.00E-04	0.0004	-0.0765	0.9461	0.9789569	Mineral
Longitude	nifh_Cluster1262	0.008	0.0056	0.338	0.2871	0.023	0.2828986	Mineral
Longitude	nifh_Cluster1266	0.0072	0.0036	0.2339	0.175	0.0678	0.549524	Mineral
Longitude	nifh_Cluster1267	0.0388	0.0028	0.0023	-0.0744	0.8643	0.9456804	Mineral
Longitude	nifh_Cluster127	0.0033	0.0015	0.0625	-0.0096	0.3688	0.8052395	Mineral
Longitude	nifh_Cluster1278	0.2105	-0.0343	0.0817	0.011	0.3018	0.8052395	Mineral
Longitude	nifh_Cluster128	0.0043	0.0021	0.1642	0.0999	0.1341	0.6704217	Mineral
Longitude	nifh_Cluster1289	0.004	0.0011	0.0559	-0.0167	0.3961	0.8052395	Mineral
Longitude	nifh_Cluster129	0.0086	0.0039	0.1022	0.0332	0.2453	0.8052395	Mineral
Longitude	nifh_Cluster1292	0.0106	-0.0019	0.0296	-0.0451	0.5401	0.8471771	Mineral
Longitude	nifh_Cluster130	0.0071	0.0033	0.0611	-0.0111	0.3743	0.8052395	Mineral
Longitude	nifh_Cluster1305	0.0048	-0.0005	0.0103	-0.0658	0.719	0.8948981	Mineral
Longitude	nifh_Cluster1306	0.0055	-0.0008	0.0176	-0.058	0.6374	0.8775051	Mineral
Longitude	nifh_Cluster131	0.0067	0.003	0.087	0.0168	0.2858	0.8052395	Mineral
Longitude	nifh_Cluster1319	0.0048	0.0017	0.0707	-0.0008	0.338	0.8052395	Mineral
Longitude	nifh_Cluster132	0.0064	0.0033	0.1318	0.065	0.1835	0.7595118	Mineral
Longitude	nifh_Cluster1320	0.0028	-0.0013	0.0581	-0.0144	0.387	0.8052395	Mineral
Longitude	nifh_Cluster1323	0.0022	-0.0015	0.2685	0.2122	0.0478	0.4554303	Mineral
Longitude	nifh_Cluster1324	0.0235	0.0065	0.0752	0.004	0.3228	0.8052395	Mineral
Longitude	nifh_Cluster1336	0.0159	-0.0026	0.0876	0.0175	0.284	0.8052395	Mineral

Longitude	nifh_Cluster1340	0.0478	-0.0486	0.7516	0.7325	0.00E+00	0.0064975	Mineral
Longitude	nifh_Cluster1345	0.033	-0.0031	0.0048	-0.0718	0.8064	0.9259972	Mineral
Longitude	nifh_Cluster1370	0.0022	0.0009	0.0517	-0.0212	0.4149	0.8052395	Mineral
Longitude	nifh_Cluster1375	0.0017	-0.0004	0.0307	-0.0439	0.5324	0.8471771	Mineral
Longitude	nifh_Cluster139	0.0038	-0.0028	0.1968	0.135	0.0977	0.6041774	Mineral
Longitude	nifh_Cluster140	0.0051	0.00E+00	0.00E+00	-0.0769	0.9978	0.999544	Mineral
Longitude	nifh_Cluster141	0.0808	-0.001	0.0013	-0.0755	0.8974	0.9526986	Mineral
Longitude	nifh_Cluster148	0.006	-0.0033	0.1179	0.05	0.2102	0.7899765	Mineral
Longitude	nifh_Cluster1480	0.0006	-1.00E-04	0.0028	-0.0739	0.8519	0.9394389	Mineral
Longitude	nifh_Cluster152	0.0602	0.0192	0.2372	0.1785	0.0656	0.5449657	Mineral
Longitude	nifh_Cluster156	0.0459	0.0145	0.2071	0.1461	0.0883	0.592125	Mineral
Longitude	nifh_Cluster1562	0.0293	0.0064	0.0174	-0.0581	0.6389	0.8775051	Mineral
Longitude	nifh_Cluster158	0.019	-0.0038	0.0446	-0.0289	0.4498	0.810923	Mineral
Longitude	nifh_Cluster16	0.0023	0.001	0.0536	-0.0192	0.4066	0.8052395	Mineral
Longitude	nifh_Cluster166	0.0165	0.008	0.0938	0.0241	0.2668	0.8052395	Mineral
Longitude	nifh_Cluster205	0.0121	0.0003	0.0009	-0.0759	0.9133	0.9587503	Mineral
Longitude	nifh_Cluster21	0.0022	0.00E+00	0.0001	-0.0768	0.9687	0.9825468	Mineral
Longitude	nifh_Cluster222	0.0069	0.0034	0.0838	0.0134	0.2952	0.8052395	Mineral
Longitude	nifh_Cluster225	0.021	-0.0012	0.0081	-0.0682	0.7499	0.9047477	Mineral
Longitude	nifh_Cluster230	0.0052	0.0011	0.0359	-0.0383	0.4988	0.8348441	Mineral
Longitude	nifh_Cluster231	0.0019	-0.0001	0.0017	-0.0751	0.8839	0.9526986	Mineral
Longitude	nifh_Cluster236	0.0034	0.0005	0.0132	-0.0627	0.6834	0.8862043	Mineral
Longitude	nifh_Cluster237	0.007	0.0026	0.1326	0.0659	0.1821	0.7595118	Mineral
Longitude	nifh_Cluster243	0.0253	0.005	0.0262	-0.0488	0.5647	0.8476038	Mineral
Longitude	nifh_Cluster2432	0.0317	-0.0028	0.0044	-0.0722	0.8144	0.9259972	Mineral
Longitude	nifh_Cluster2433	0.0028	-0.0014	0.0519	-0.021	0.414	0.8052395	Mineral
Longitude	nifh_Cluster246	0.0068	0.0024	0.0629	-0.0092	0.3674	0.8052395	Mineral
Longitude	nifh_Cluster25	0.0195	0.0036	0.0905	0.0206	0.2758	0.8052395	Mineral
Longitude	nifh_Cluster256	0.0023	-0.0004	0.0121	-0.0639	0.6969	0.8900961	Mineral
Longitude	nifh_Cluster264	0.0025	0.0014	0.1968	0.135	0.0977	0.6041774	Mineral
Longitude	nifh_Cluster265	0.0702	-0.001	0.0013	-0.0755	0.8991	0.9526986	Mineral
Longitude	nifh_Cluster267	0.0071	-0.0021	0.0835	0.013	0.2962	0.8052395	Mineral
Longitude	nifh_Cluster268	0.0109	0.0015	0.0486	-0.0246	0.4298	0.810923	Mineral
Longitude	nifh_Cluster269	0.0283	-0.0051	0.03	-0.0446	0.5369	0.8471771	Mineral
Longitude	nifh_Cluster272	0.0012	0.0001	0.0018	-0.075	0.8811	0.9526986	Mineral
Longitude	nifh_Cluster274	0.0014	0.0006	0.0231	-0.052	0.5887	0.8599568	Mineral
Longitude	nifh_Cluster275	0.0051	0.0002	0.0015	-0.0754	0.8926	0.9526986	Mineral
Longitude	nifh_Cluster278	0.0056	0.0002	0.0007	-0.0761	0.9239	0.9673696	Mineral
Longitude	nifh_Cluster279	0.0058	0.0004	0.0048	-0.0717	0.8056	0.9259972	Mineral
Longitude	nifh_Cluster28	0.0017	-0.0006	0.035	-0.0392	0.5044	0.8374309	Mineral
Longitude	nifh_Cluster280	0.0034	-0.003	0.191	0.1288	0.1033	0.6097173	Mineral
Longitude	nifh_Cluster282	0.0052	-0.0005	0.0081	-0.0682	0.7491	0.9047477	Mineral
Longitude	nifh_Cluster287	0.003	0.0008	0.042	-0.0317	0.4638	0.810923	Mineral
Longitude	nifh_Cluster290	0.0096	0.0012	0.0094	-0.0668	0.7309	0.896443	Mineral
Longitude	nifh_Cluster297	0.004	0.0013	0.0255	-0.0494	0.5696	0.8476038	Mineral
Longitude	nifh_Cluster299	0.0025	0.0013	0.1259	0.0586	0.1945	0.7705672	Mineral
Longitude	nifh_Cluster303	0.002	0.0008	0.079	0.0082	0.3101	0.8052395	Mineral
Longitude	nifh_Cluster304	0.0025	0.0009	0.0591	-0.0133	0.3826	0.8052395	Mineral
Longitude	nifh_Cluster313	0.0129	0.0056	0.0786	0.0077	0.3114	0.8052395	Mineral
Longitude	nifh_Cluster314	0.0027	0.0014	0.1095	0.041	0.2284	0.8027796	Mineral
Longitude	nifh_Cluster317	0.0089	0.0024	0.0753	0.0042	0.3221	0.8052395	Mineral
Longitude	nifh_Cluster32	0.0029	0.0012	0.1264	0.0592	0.1934	0.7705672	Mineral
Longitude	nifh_Cluster320	13.944	0.9929	0.1407	0.0746	0.1682	0.7298507	Mineral
Longitude	nifh_Cluster334	0.0084	0.0045	0.1046	0.0357	0.2397	0.8052395	Mineral
Longitude	nifh_Cluster335	0.0059	0.0033	0.1109	0.0425	0.2251	0.8027796	Mineral

Longitude	nifh_Cluster338	0.0151	-0.0035	0.0798	0.009	0.3078	0.8052395	Mineral
Longitude	nifh_Cluster340	0.0041	-0.0008	0.0269	-0.048	0.5592	0.8476038	Mineral
Longitude	nifh_Cluster341	0.0021	0.0008	0.0491	-0.024	0.4273	0.810923	Mineral
Longitude	nifh_Cluster35	0.0079	-0.005	0.5828	0.5507	0.0009	0.0321953	Mineral
Longitude	nifh_Cluster351	0.0035	0.0018	0.1076	0.0389	0.2327	0.8027796	Mineral
Longitude	nifh_Cluster3574	0.1191	0.0236	0.1409	0.0748	0.168	0.7298507	Mineral
Longitude	nifh_Cluster3691	0.0224	-0.0166	0.4528	0.4107	0.006	0.1259382	Mineral
Longitude	nifh_Cluster37	0.0374	0.008	0.0382	-0.0358	0.485	0.8258412	Mineral
Longitude	nifh_Cluster374	0.0029	-0.004	0.7463	0.7268	0.00E+00	0.0064975	Mineral
Longitude	nifh_Cluster38	0.0008	0.0004	0.0402	-0.0336	0.4736	0.8169776	Mineral
Longitude	nifh_Cluster382	0.0023	0.0009	0.029	-0.0457	0.5437	0.8471771	Mineral
Longitude	nifh_Cluster384	0.0023	0.0008	0.0474	-0.0258	0.4356	0.810923	Mineral
Longitude	nifh_Cluster386	0.0041	0.0023	0.1	0.0308	0.2508	0.8052395	Mineral
Longitude	nifh_Cluster389	0.003	0.0016	0.0595	-0.0128	0.3809	0.8052395	Mineral
Longitude	nifh_Cluster394	0.0027	0.0014	0.0581	-0.0143	0.3867	0.8052395	Mineral
Longitude	nifh_Cluster40	0.0014	0.0008	0.1001	0.0309	0.2505	0.8052395	Mineral
Longitude	nifh_Cluster43	0.0107	0.0006	0.0039	-0.0727	0.824	0.9259972	Mineral
Longitude	nifh_Cluster434	0.0048	-0.0017	0.0637	-0.0083	0.364	0.8052395	Mineral
Longitude	nifh_Cluster440	0.0171	-0.005	0.073	0.0017	0.3299	0.8052395	Mineral
Longitude	nifh_Cluster462	0.0048	0.001	0.0213	-0.054	0.6041	0.8683974	Mineral
Longitude	nifh_Cluster470	0.017	-0.0084	0.319	0.2666	0.0283	0.3126969	Mineral
Longitude	nifh_Cluster49	0.0223	0.0074	0.0473	-0.026	0.4364	0.810923	Mineral
Longitude	nifh_Cluster499	0.0028	0.0013	0.0839	0.0134	0.2951	0.8052395	Mineral
Longitude	nifh_Cluster5	0.0263	-0.0071	0.2522	0.1946	0.0565	0.4898286	Mineral
Longitude	nifh_Cluster506	0.0058	-0.0016	0.0572	-0.0153	0.3905	0.8052395	Mineral
Longitude	nifh_Cluster51	0.0031	0.0017	0.0788	0.008	0.3107	0.8052395	Mineral
Longitude	nifh_Cluster52	0.0396	0.0172	0.1258	0.0585	0.1946	0.7705672	Mineral
Longitude	nifh_Cluster525	0.0025	0.0017	0.1048	0.036	0.2391	0.8052395	Mineral
Longitude	nifh_Cluster531	0.0129	0.0096	0.3744	0.3263	0.0153	0.2289948	Mineral
Longitude	nifh_Cluster539	0.0056	0.0031	0.0615	-0.0107	0.3727	0.8052395	Mineral
Longitude	nifh_Cluster578	0.0593	-0.0207	0.3111	0.2581	0.0307	0.3297763	Mineral
Longitude	nifh_Cluster58	0.2389	0.0044	0.0002	-0.0767	0.9598	0.9812024	Mineral
Longitude	nifh_Cluster60	0.011	0.0026	0.0434	-0.0302	0.4561	0.810923	Mineral
Longitude	nifh_Cluster61	0.0017	0.0011	0.2028	0.1414	0.0921	0.6030881	Mineral
Longitude	nifh_Cluster65	0.0085	0.0026	0.0709	-0.0006	0.3374	0.8052395	Mineral
Longitude	nifh_Cluster686	0.0013	0.0009	0.1555	0.0905	0.1458	0.6828758	Mineral
Longitude	nifh_Cluster69	0.0092	0.0026	0.0375	-0.0366	0.4894	0.8258412	Mineral
Longitude	nifh_Cluster70	0.0144	0.0064	0.0728	0.0015	0.3307	0.8052395	Mineral
Longitude	nifh_Cluster717	0.0024	-1.00E-04	0.0007	-0.0762	0.9252	0.9673696	Mineral
Longitude	nifh_Cluster725	0.0035	0.0003	0.0058	-0.0707	0.788	0.9151759	Mineral
Longitude	nifh_Cluster727	0.0038	-0.0014	0.0821	0.0114	0.3006	0.8052395	Mineral
Longitude	nifh_Cluster73	0.0133	0.007	0.2012	0.1398	0.0935	0.6030881	Mineral
Longitude	nifh_Cluster74	0.005	0.0023	0.09	0.02	0.2772	0.8052395	Mineral
Longitude	nifh_Cluster748	0.0104	0.0005	0.0061	-0.0703	0.7812	0.9151759	Mineral
Longitude	nifh_Cluster749	0.0055	0.0007	0.0106	-0.0655	0.7154	0.8942401	Mineral
Longitude	nifh_Cluster755	0.0118	-0.0083	0.4545	0.4125	0.0058	0.1259382	Mineral
Longitude	nifh_Cluster76	0.0033	-0.0006	0.0145	-0.0613	0.6693	0.8775051	Mineral
Longitude	nifh_Cluster79	0.002	0.0012	0.1666	0.1024	0.131	0.6704217	Mineral
Longitude	nifh_Cluster81	0.0038	0.0018	0.0896	0.0195	0.2785	0.8052395	Mineral
Longitude	nifh_Cluster82	0.0014	0.0007	0.0656	-0.0063	0.3568	0.8052395	Mineral
Longitude	nifh_Cluster83	0.0055	-0.004	0.3221	0.27	0.0273	0.3100288	Mineral
Longitude	nifh_Cluster86	0.0161	-0.0111	0.6381	0.6103	0.0004	0.017437	Mineral
Longitude	nifh_Cluster868	0.0098	-0.0001	0.0003	-0.0766	0.9551	0.9800449	Mineral
Longitude	nifh_Cluster87	0.003	0.0013	0.0696	-0.002	0.342	0.8052395	Mineral
Longitude	nifh_Cluster88	0.0054	-0.0062	0.6708	0.6455	0.0002	0.017437	Mineral

Longitude	nifh_Cluster9	0.0077	-0.0018	0.0298	-0.0449	0.5386	0.8471771	Mineral
Longitude	nifh_Cluster92	0.0068	0.0032	0.0821	0.0115	0.3005	0.8052395	Mineral
Longitude	nifh_Cluster93	0.0035	0.0003	0.0029	-0.0738	0.85	0.9392862	Mineral
Longitude	nifh_Cluster94	0.005	0.0021	0.0691	-0.0025	0.3439	0.8052395	Mineral
Longitude	nifh_Cluster95	0.0149	0.0071	0.1227	0.0553	0.2005	0.7730131	Mineral
Longitude	nifh_Cluster96	0.0131	-0.0075	0.3028	0.2492	0.0335	0.3534458	Mineral
Longitude	nifh_Cluster97	0.0125	0.0025	0.1155	0.0474	0.2153	0.7973679	Mineral
Longitude	nifh_Cluster98	0.0049	-0.004	0.5486	0.5139	0.0016	0.0492012	Mineral
Longitude	nifh_Cluster99	0.0035	0.0015	0.0604	-0.0119	0.3774	0.8052395	Mineral
Longitude	nirk_Cluster0	6.5686	0.6685	0.4652	0.424	0.0051	0.1156481	Mineral
Longitude	nirk_Cluster1	0.2105	0.0555	0.2155	0.1552	0.0813	0.5716535	Mineral
Longitude	nirk_Cluster10	0.6649	-0.2149	0.8387	0.8263	0.00E+00	0.0009769	Mineral
Longitude	nirk_Cluster101	0.004	-0.0002	0.0014	-0.0754	0.8957	0.9526986	Mineral
Longitude	nirk_Cluster102	0.055	0.0039	0.011	-0.0651	0.7102	0.8934386	Mineral
Longitude	nirk_Cluster103	0.09	0.009	0.0301	-0.0445	0.5366	0.8471771	Mineral
Longitude	nirk_Cluster104	0.0028	-0.0009	0.0326	-0.0418	0.5193	0.8463767	Mineral
Longitude	nirk_Cluster105	0.1172	0.0104	0.0225	-0.0527	0.594	0.8632482	Mineral
Longitude	nirk_Cluster106	0.073	0.0187	0.0861	0.0158	0.2885	0.8052395	Mineral
Longitude	nirk_Cluster107	0.1123	0.0032	0.002	-0.0748	0.8749	0.9506414	Mineral
Longitude	nirk_Cluster108	0.0184	-0.0016	0.0093	-0.0669	0.7323	0.896443	Mineral
Longitude	nirk_Cluster109	0.0158	-0.001	0.0067	-0.0698	0.7725	0.9146383	Mineral
Longitude	nirk_Cluster11	0.1509	-0.002	0.004	-0.0727	0.8239	0.9259972	Mineral
Longitude	nirk_Cluster12	0.1052	0.0228	0.272	0.216	0.0462	0.4539683	Mineral
Longitude	nirk_Cluster13	0.0418	0.0042	0.0667	-0.0051	0.3528	0.8052395	Mineral
Longitude	nirk_Cluster14	0.1084	-0.0132	0.1578	0.0931	0.1426	0.6828758	Mineral
Longitude	nirk_Cluster15	0.4141	0.0914	0.1475	0.0819	0.1576	0.7099334	Mineral
Longitude	nirk_Cluster16	0.5816	0.1684	0.2129	0.1524	0.0834	0.57213	Mineral
Longitude	nirk_Cluster17	0.1044	-0.0059	0.0261	-0.0488	0.5652	0.8476038	Mineral
Longitude	nirk_Cluster18	0.1461	0.0394	0.1721	0.1084	0.1241	0.6704217	Mineral
Longitude	nirk_Cluster19	0.2953	0.0428	0.2685	0.2122	0.0479	0.4554303	Mineral
Longitude	nirk_Cluster2	0.2004	-0.0149	0.0477	-0.0256	0.4345	0.810923	Mineral
Longitude	nirk_Cluster20	0.1934	-0.0244	0.1237	0.0563	0.1986	0.7720497	Mineral
Longitude	nirk_Cluster21	0.1711	-0.0051	0.0134	-0.0625	0.6815	0.8862043	Mineral
Longitude	nirk_Cluster22	0.1469	0.0162	0.0681	-0.0036	0.3475	0.8052395	Mineral
Longitude	nirk_Cluster23	0.0729	-0.0012	0.0034	-0.0733	0.8376	0.9359075	Mineral
Longitude	nirk_Cluster24	0.0753	-0.0135	0.2246	0.1649	0.0743	0.5683671	Mineral
Longitude	nirk_Cluster25	0.5519	-0.0508	0.0915	0.0217	0.273	0.8052395	Mineral
Longitude	nirk_Cluster26	0.1119	0.0059	0.0297	-0.0449	0.5389	0.8471771	Mineral
Longitude	nirk_Cluster27	0.2066	-0.0155	0.0317	-0.0428	0.5257	0.8471771	Mineral
Longitude	nirk_Cluster28	0.0808	-0.0087	0.0599	-0.0124	0.3793	0.8052395	Mineral
Longitude	nirk_Cluster29	0.162	-0.0509	0.6603	0.6342	0.0002	0.017437	Mineral
Longitude	nirk_Cluster3	0.3427	-0.0261	0.0714	0.00E+00	0.3355	0.8052395	Mineral
Longitude	nirk_Cluster30	0.0712	0.0131	0.1079	0.0392	0.2321	0.8027796	Mineral
Longitude	nirk_Cluster31	0.1423	0.0063	0.0202	-0.0552	0.6138	0.8683974	Mineral
Longitude	nirk_Cluster32	0.1568	0.0447	0.172	0.1083	0.1243	0.6704217	Mineral
Longitude	nirk_Cluster33	0.0812	0.0177	0.0876	0.0174	0.2842	0.8052395	Mineral
Longitude	nirk_Cluster34	0.0462	-0.0032	0.0126	-0.0633	0.6901	0.8881936	Mineral
Longitude	nirk_Cluster35	0.1282	-0.0158	0.0276	-0.0472	0.554	0.8476038	Mineral
Longitude	nirk_Cluster36	0.0021	-0.0004	0.0113	-0.0647	0.7057	0.89055	Mineral
Longitude	nirk_Cluster39	0.0045	-0.0048	0.2246	0.165	0.0743	0.5683671	Mineral
Longitude	nirk_Cluster4	0.1521	-0.0059	0.0079	-0.0684	0.7521	0.9055403	Mineral
Longitude	nirk_Cluster40	0.0544	0.00E+00	0.00E+00	-0.0769	0.9945	0.999544	Mineral
Longitude	nirk_Cluster41	0.1797	0.0095	0.0335	-0.0408	0.5137	0.8425891	Mineral
Longitude	nirk_Cluster44	0.0756	0.0054	0.0152	-0.0605	0.6612	0.8775051	Mineral
Longitude	nirk_Cluster46	0.0193	-0.0018	0.0083	-0.0679	0.7461	0.9047477	Mineral

Longitude	nirk_Cluster48	0.003	0.0002	0.0023	-0.0745	0.8655	0.9456804	Mineral
Longitude	nirk_Cluster49	0.0072	0.0046	0.2142	0.1537	0.0824	0.5716535	Mineral
Longitude	nirk_Cluster5	0.2174	0.005	0.0062	-0.0703	0.781	0.9151759	Mineral
Longitude	nirk_Cluster50	0.0014	0.00E+00	0.00E+00	-0.0769	0.9969	0.999544	Mineral
Longitude	nirk_Cluster52	0.0551	0.0023	0.0066	-0.0698	0.7736	0.9146383	Mineral
Longitude	nirk_Cluster53	0.0039	-0.0002	0.0006	-0.0763	0.9315	0.9710407	Mineral
Longitude	nirk_Cluster54	0.0024	-0.0002	0.0039	-0.0728	0.8257	0.9261883	Mineral
Longitude	nirk_Cluster55	0.0028	0.0006	0.0106	-0.0655	0.7151	0.8942401	Mineral
Longitude	nirk_Cluster56	0.0009	0.0005	0.048	-0.0253	0.4329	0.810923	Mineral
Longitude	nirk_Cluster57	0.0017	0.0007	0.028	-0.0467	0.5509	0.8476038	Mineral
Longitude	nirk_Cluster58	0.0102	-0.0029	0.0689	-0.0028	0.3447	0.8052395	Mineral
Longitude	nirk_Cluster59	0.007	-0.003	0.161	0.0965	0.1382	0.6793983	Mineral
Longitude	nirk_Cluster6	0.1713	-0.0295	0.2218	0.1619	0.0764	0.5703289	Mineral
Longitude	nirk_Cluster60	0.0006	-0.0003	0.0253	-0.0497	0.5712	0.8476038	Mineral
Longitude	nirk_Cluster62	0.1789	0.0144	0.0155	-0.0602	0.658	0.8775051	Mineral
Longitude	nirk_Cluster63	0.1586	-0.0134	0.0521	-0.0209	0.4135	0.8052395	Mineral
Longitude	nirk_Cluster64	0.0043	-0.0012	0.0519	-0.021	0.4142	0.8052395	Mineral
Longitude	nirk_Cluster65	0.0531	-0.0064	0.0639	-0.0082	0.3635	0.8052395	Mineral
Longitude	nirk_Cluster66	0.0095	0.0019	0.0557	-0.0169	0.3969	0.8052395	Mineral
Longitude	nirk_Cluster67	0.0232	-0.0164	0.5925	0.5612	0.0008	0.029133	Mineral
Longitude	nirk_Cluster68	0.0158	-0.0024	0.0391	-0.0348	0.4797	0.8227048	Mineral
Longitude	nirk_Cluster69	0.0362	-0.0081	0.1584	0.0937	0.1418	0.6828758	Mineral
Longitude	nirk_Cluster7	0.096	-0.0256	0.3676	0.319	0.0166	0.2289948	Mineral
Longitude	nirk_Cluster70	0.0892	0.0217	0.0608	-0.0115	0.3758	0.8052395	Mineral
Longitude	nirk_Cluster71	0.0071	-0.0005	0.0058	-0.0707	0.7878	0.9151759	Mineral
Longitude	nirk_Cluster72	0.0278	-0.0006	0.0017	-0.0751	0.8855	0.9526986	Mineral
Longitude	nirk_Cluster73	0.1947	0.0023	0.0007	-0.0762	0.9264	0.9673696	Mineral
Longitude	nirk_Cluster74	0.8012	0.1356	0.0946	0.0249	0.2649	0.8052395	Mineral
Longitude	nirk_Cluster75	0.0186	-0.005	0.0546	-0.0181	0.4019	0.8052395	Mineral
Longitude	nirk_Cluster76	0.1377	0.0023	0.002	-0.0747	0.8737	0.9506414	Mineral
Longitude	nirk_Cluster77	0.0113	-0.0161	0.648	0.6209	0.0003	0.017437	Mineral
Longitude	nirk_Cluster78	0.3126	-0.0047	0.0011	-0.0758	0.9075	0.9544285	Mineral
Longitude	nirk_Cluster79	0.0096	-0.0098	0.537	0.5013	0.0019	0.0556684	Mineral
Longitude	nirk_Cluster8	0.1182	-0.0052	0.0059	-0.0706	0.7862	0.9151759	Mineral
Longitude	nirk_Cluster80	0.3544	0.0149	0.0067	-0.0697	0.7719	0.9146383	Mineral
Longitude	nirk_Cluster82	0.0021	0.0011	0.0628	-0.0092	0.3675	0.8052395	Mineral
Longitude	nirk_Cluster83	0.0045	-0.0024	0.2241	0.1644	0.0747	0.5683671	Mineral
Longitude	nirk_Cluster84	0.0235	0.0054	0.046	-0.0273	0.4425	0.810923	Mineral
Longitude	nirk_Cluster85	0.0053	-0.0043	0.516	0.4788	0.0026	0.0685686	Mineral
Longitude	nirk_Cluster86	0.0352	-0.0017	0.0044	-0.0722	0.8153	0.9259972	Mineral
Longitude	nirk_Cluster87	0.0138	0.0025	0.0438	-0.0298	0.4544	0.810923	Mineral
Longitude	nirk_Cluster88	0.0035	-0.0007	0.0291	-0.0455	0.543	0.8471771	Mineral
Longitude	nirk_Cluster89	0.0087	0.0028	0.0803	0.0096	0.3061	0.8052395	Mineral
Longitude	nirk_Cluster9	0.061	0.0023	0.0035	-0.0731	0.8337	0.9333213	Mineral
Longitude	nirk_Cluster90	0.0087	0.0049	0.1918	0.1296	0.1026	0.6097173	Mineral
Longitude	nirk_Cluster91	0.044	0.0059	0.0511	-0.0219	0.418	0.8085024	Mineral
Longitude	nirk_Cluster92	0.009	0.0038	0.1198	0.0521	0.2063	0.7801734	Mineral
Longitude	nirk_Cluster93	0.0357	-0.003	0.014	-0.0619	0.6748	0.8808101	Mineral
Longitude	nirk_Cluster94	0.0222	-0.0037	0.0468	-0.0265	0.4386	0.810923	Mineral
Longitude	nirk_Cluster95	0.0566	0.0168	0.1677	0.1037	0.1295	0.6704217	Mineral
Longitude	nirk_Cluster96	0.0588	0.0087	0.0582	-0.0142	0.3863	0.8052395	Mineral
Longitude	nirk_Cluster97	0.0329	0.0148	0.3178	0.2653	0.0286	0.3126969	Mineral
Longitude	nirk_Cluster98	0.0225	-0.0029	0.0161	-0.0596	0.6522	0.8775051	Mineral
Longitude	nirk_Cluster99	0.0074	-0.0025	0.0841	0.0136	0.2944	0.8052395	Mineral
Longitude	nirs_Cluster0	0.1586	-0.0173	0.0762	0.0051	0.3194	0.8052395	Mineral

Longitude	nirs_Cluster1	0.0051	-0.0003	0.0014	-0.0754	0.8929	0.9526986	Mineral
Longitude	nirs_Cluster14	0.0142	0.007	0.0723	0.001	0.3325	0.8052395	Mineral
Longitude	nirs_Cluster2	0.0174	-0.0188	0.5183	0.4813	0.0025	0.0685686	Mineral
Longitude	nirs_Cluster26	0.0007	0.0004	0.0445	-0.029	0.4504	0.810923	Mineral
Longitude	nirs_Cluster28	0.0099	0.0044	0.0602	-0.0121	0.378	0.8052395	Mineral
Longitude	nirs_Cluster3	0.1914	-0.0256	0.2577	0.2006	0.0534	0.4847867	Mineral
Longitude	nirs_Cluster36	0.0018	-0.0023	0.1557	0.0908	0.1455	0.6828758	Mineral
Longitude	nirs_Cluster37	0.0092	0.0049	0.0455	-0.0279	0.4453	0.810923	Mineral
Longitude	nirs_Cluster4	0.0003	0.0002	0.0207	-0.0546	0.609	0.8683974	Mineral
Longitude	nirs_Cluster50	0.0024	0.0003	0.004	-0.0726	0.8233	0.9259972	Mineral
Longitude	nirs_Cluster9	0.0376	0.0082	0.0145	-0.0613	0.6687	0.8775051	Mineral
Longitude	norb_Cluster0	0.0848	0.044	0.5065	0.4685	0.0029	0.071828	Mineral
Longitude	norb_Cluster1	1.2746	0.6711	0.4004	0.3543	0.0113	0.1968634	Mineral
Longitude	norb_Cluster101	0.0037	-1.00E-04	0.0003	-0.0766	0.9536	0.9800449	Mineral
Longitude	norb_Cluster102	0.005	0.0029	0.2239	0.1642	0.0748	0.5683671	Mineral
Longitude	norb_Cluster103	0.0025	0.0012	0.0766	0.0055	0.3181	0.8052395	Mineral
Longitude	norb_Cluster104	0.0025	0.0004	0.0041	-0.0726	0.8215	0.9259972	Mineral
Longitude	norb_Cluster106	0.1468	0.0164	0.0345	-0.0398	0.5076	0.8379568	Mineral
Longitude	norb_Cluster107	0.0163	-0.0023	0.0188	-0.0567	0.6259	0.8775051	Mineral
Longitude	norb_Cluster108	0.0069	-0.0015	0.0523	-0.0206	0.4125	0.8052395	Mineral
Longitude	norb_Cluster109	0.0282	-0.0033	0.0202	-0.0552	0.6134	0.8683974	Mineral
Longitude	norb_Cluster11	0.0156	0.0017	0.0123	-0.0637	0.6942	0.8900961	Mineral
Longitude	norb_Cluster110	0.0128	-0.0042	0.1023	0.0332	0.2452	0.8052395	Mineral
Longitude	norb_Cluster112	0.0215	-0.0006	0.0016	-0.0752	0.8882	0.9526986	Mineral
Longitude	norb_Cluster113	0.0157	0.0019	0.0171	-0.0585	0.6426	0.8775051	Mineral
Longitude	norb_Cluster114	0.0035	0.0019	0.0645	-0.0074	0.3609	0.8052395	Mineral
Longitude	norb_Cluster115	0.0034	0.0009	0.0697	-0.0018	0.3416	0.8052395	Mineral
Longitude	norb_Cluster116	0.0083	0.0045	0.19	0.1277	0.1044	0.6097173	Mineral
Longitude	norb_Cluster117	0.0034	1.00E-04	0.0001	-0.0768	0.9699	0.9825468	Mineral
Longitude	norb_Cluster118	0.0044	1.00E-04	1.00E-04	-0.0768	0.9731	0.9825468	Mineral
Longitude	norb_Cluster119	0.0263	-0.0041	0.0259	-0.049	0.5665	0.8476038	Mineral
Longitude	norb_Cluster12	0.005	0.0011	0.0183	-0.0572	0.6305	0.8775051	Mineral
Longitude	norb_Cluster121	0.0023	0.0007	0.0183	-0.0572	0.6306	0.8775051	Mineral
Longitude	norb_Cluster122	0.0184	0.0029	0.0332	-0.0412	0.5156	0.8427221	Mineral
Longitude	norb_Cluster123	0.0071	-0.0001	0.0002	-0.0767	0.9612	0.9812024	Mineral
Longitude	norb_Cluster124	0.7452	0.3794	0.4004	0.3543	0.0113	0.1968634	Mineral
Longitude	norb_Cluster125	0.0052	1.00E-04	1.00E-04	-0.0768	0.9742	0.9825468	Mineral
Longitude	norb_Cluster126	0.011	0.0005	0.0022	-0.0746	0.8681	0.946683	Mineral
Longitude	norb_Cluster127	0.2041	0.075	0.2094	0.1486	0.0863	0.5854439	Mineral
Longitude	norb_Cluster128	0.0123	0.0048	0.17	0.1061	0.1267	0.6704217	Mineral
Longitude	norb_Cluster13	0.0051	0.0017	0.036	-0.0382	0.4984	0.8348441	Mineral
Longitude	norb_Cluster131	0.0209	0.0025	0.0198	-0.0556	0.6165	0.870249	Mineral
Longitude	norb_Cluster132	1.2771	0.1544	0.0531	-0.0197	0.4086	0.8052395	Mineral
Longitude	norb_Cluster133	0.0023	-0.0015	0.1742	0.1106	0.1217	0.6704217	Mineral
Longitude	norb_Cluster135	0.0703	0.0204	0.1644	0.1001	0.1337	0.6704217	Mineral
Longitude	norb_Cluster136	0.025	0.0029	0.0187	-0.0568	0.6273	0.8775051	Mineral
Longitude	norb_Cluster139	0.0391	0.001	0.0011	-0.0757	0.9051	0.9544285	Mineral
Longitude	norb_Cluster14	0.0037	0.0015	0.0973	0.0278	0.2578	0.8052395	Mineral
Longitude	norb_Cluster140	0.0485	0.0179	0.2891	0.2345	0.0387	0.3936495	Mineral
Longitude	norb_Cluster142	0.0018	0.0005	0.0152	-0.0606	0.6618	0.8775051	Mineral
Longitude	norb_Cluster144	0.0122	-0.0022	0.0439	-0.0296	0.4534	0.810923	Mineral
Longitude	norb_Cluster146	0.0418	0.0077	0.0794	0.0086	0.3089	0.8052395	Mineral
Longitude	norb_Cluster15	0.0502	0.0221	0.2532	0.1958	0.0559	0.4898286	Mineral
Longitude	norb_Cluster150	0.0199	0.0009	0.0051	-0.0714	0.7997	0.9233387	Mineral
Longitude	norb_Cluster155	0.1729	0.0803	0.392	0.3453	0.0125	0.2051786	Mineral

Longitude	norb_Cluster16	0.0165	0.0043	0.0519	-0.021	0.4141	0.8052395	Mineral
Longitude	norb_Cluster164	0.0242	0.0107	0.2007	0.1392	0.094	0.6030881	Mineral
Longitude	norb_Cluster165	0.0092	0.0044	0.1235	0.0561	0.1989	0.7720497	Mineral
Longitude	norb_Cluster166	0.0046	0.0018	0.0796	0.0088	0.3082	0.8052395	Mineral
Longitude	norb_Cluster169	0.0309	0.0116	0.1449	0.0792	0.1615	0.7165439	Mineral
Longitude	norb_Cluster17	0.0035	0.001	0.047	-0.0264	0.4379	0.810923	Mineral
Longitude	norb_Cluster170	0.0299	0.0153	0.2181	0.1579	0.0793	0.5703289	Mineral
Longitude	norb_Cluster171	0.0089	0.0012	0.0084	-0.0679	0.7455	0.9047477	Mineral
Longitude	norb_Cluster172	0.0179	0.0077	0.1327	0.0659	0.182	0.7595118	Mineral
Longitude	norb_Cluster175	0.0595	0.0257	0.1624	0.0979	0.1364	0.6763755	Mineral
Longitude	norb_Cluster176	0.0392	0.0139	0.1138	0.0456	0.2188	0.7973679	Mineral
Longitude	norb_Cluster177	0.0361	0.0129	0.1128	0.0445	0.2211	0.7973679	Mineral
Longitude	norb_Cluster179	0.003	-0.002	0.1646	0.1004	0.1335	0.6704217	Mineral
Longitude	norb_Cluster18	0.0047	0.0006	0.0147	-0.0611	0.667	0.8775051	Mineral
Longitude	norb_Cluster180	0.0133	-0.0038	0.1274	0.0603	0.1916	0.7705672	Mineral
Longitude	norb_Cluster181	0.0003	0.0002	0.0207	-0.0546	0.609	0.8683974	Mineral
Longitude	norb_Cluster182	0.0018	0.0008	0.0696	-0.002	0.3421	0.8052395	Mineral
Longitude	norb_Cluster184	0.0175	0.0058	0.1126	0.0443	0.2214	0.7973679	Mineral
Longitude	norb_Cluster186	0.0011	0.0008	0.0908	0.0208	0.2752	0.8052395	Mineral
Longitude	norb_Cluster19	0.0058	0.0008	0.0218	-0.0534	0.5995	0.8668932	Mineral
Longitude	norb_Cluster192	0.0221	0.0092	0.1599	0.0953	0.1398	0.6814324	Mineral
Longitude	norb_Cluster193	0.0022	0.0007	0.0425	-0.0312	0.4611	0.810923	Mineral
Longitude	norb_Cluster197	0.0398	0.0196	0.0462	-0.0272	0.442	0.810923	Mineral
Longitude	norb_Cluster2	0.0032	0.0011	0.0376	-0.0364	0.4884	0.8258412	Mineral
Longitude	norb_Cluster20	0.0665	0.0297	0.341	0.2903	0.0223	0.279579	Mineral
Longitude	norb_Cluster208	0.0015	0.0005	0.0233	-0.0519	0.5874	0.8599568	Mineral
Longitude	norb_Cluster21	0.0542	0.0235	0.2828	0.2277	0.0413	0.4130305	Mineral
Longitude	norb_Cluster211	0.0141	0.0045	0.0265	-0.0484	0.5622	0.8476038	Mineral
Longitude	norb_Cluster212	0.0252	0.0103	0.074	0.0027	0.3268	0.8052395	Mineral
Longitude	norb_Cluster214	0.0163	0.004	0.027	-0.0479	0.5588	0.8476038	Mineral
Longitude	norb_Cluster217	0.0186	0.0067	0.0567	-0.0159	0.3928	0.8052395	Mineral
Longitude	norb_Cluster219	0.0108	0.0049	0.0646	-0.0074	0.3608	0.8052395	Mineral
Longitude	norb_Cluster22	0.0028	-0.0007	0.0142	-0.0617	0.6726	0.8798649	Mineral
Longitude	norb_Cluster220	0.0248	0.0117	0.0709	-0.0006	0.3376	0.8052395	Mineral
Longitude	norb_Cluster221	0.0248	0.0097	0.0416	-0.0321	0.4659	0.810923	Mineral
Longitude	norb_Cluster222	0.0104	0.0053	0.0526	-0.0203	0.4109	0.8052395	Mineral
Longitude	norb_Cluster224	0.0238	0.0098	0.0669	-0.0049	0.3521	0.8052395	Mineral
Longitude	norb_Cluster225	0.0095	0.001	0.0074	-0.069	0.7607	0.9085432	Mineral
Longitude	norb_Cluster226	0.0049	0.0001	0.0003	-0.0766	0.9546	0.9800449	Mineral
Longitude	norb_Cluster228	0.0009	0.0001	0.0015	-0.0753	0.8913	0.9526986	Mineral
Longitude	norb_Cluster229	0.004	0.0007	0.0176	-0.0579	0.6371	0.8775051	Mineral
Longitude	norb_Cluster230	0.0019	-0.0004	0.0151	-0.0607	0.663	0.8775051	Mineral
Longitude	norb_Cluster231	0.0061	-0.0017	0.029	-0.0457	0.5442	0.8471771	Mineral
Longitude	norb_Cluster232	0.0044	-0.0007	0.0103	-0.0658	0.7186	0.8948981	Mineral
Longitude	norb_Cluster234	0.017	0.0028	0.0177	-0.0579	0.6365	0.8775051	Mineral
Longitude	norb_Cluster235	0.0012	0.00E+00	0.00E+00	-0.0769	0.9997	0.999699	Mineral
Longitude	norb_Cluster238	0.0135	-0.0047	0.0554	-0.0173	0.3984	0.8052395	Mineral
Longitude	norb_Cluster239	0.0109	-0.0014	0.006	-0.0705	0.7839	0.9151759	Mineral
Longitude	norb_Cluster24	0.3014	0.1338	0.3923	0.3456	0.0125	0.2051786	Mineral
Longitude	norb_Cluster244	0.0015	-0.0012	0.2036	0.1423	0.0914	0.6030881	Mineral
Longitude	norb_Cluster247	0.006	-0.0043	0.556	0.5218	0.0014	0.0464589	Mineral
Longitude	norb_Cluster25	0.1263	0.065	0.3701	0.3216	0.0161	0.2289948	Mineral
Longitude	norb_Cluster250	0.0146	-0.0013	0.0082	-0.068	0.7476	0.9047477	Mineral
Longitude	norb_Cluster251	0.0064	-0.0007	0.0076	-0.0688	0.7576	0.9084971	Mineral
Longitude	norb_Cluster26	0.1134	0.0608	0.4291	0.3852	0.008	0.1634781	Mineral

Longitude	norb_Cluster28	0.0355	0.0068	0.0537	-0.0191	0.4061	0.8052395	Mineral
Longitude	norb_Cluster29	0.2454	0.0695	0.376	0.328	0.0151	0.2289948	Mineral
Longitude	norb_Cluster31	0.0632	0.0279	0.3266	0.2748	0.026	0.3012963	Mineral
Longitude	norb_Cluster32	0.1401	0.079	0.3661	0.3174	0.0168	0.2289948	Mineral
Longitude	norb_Cluster33	0.434	0.2124	0.6408	0.6131	0.0003	0.017437	Mineral
Longitude	norb_Cluster35	0.0746	0.0356	0.3722	0.3239	0.0157	0.2289948	Mineral
Longitude	norb_Cluster36	1.1002	0.697	0.6174	0.588	0.0005	0.0220937	Mineral
Longitude	norb_Cluster37	0.7173	0.3999	0.7194	0.6978	1.00E-04	0.0095231	Mineral
Longitude	norb_Cluster38	0.0138	0.0033	0.1007	0.0315	0.2491	0.8052395	Mineral
Longitude	norb_Cluster39	0.0465	0.0132	0.1315	0.0647	0.1841	0.7595118	Mineral
Longitude	norb_Cluster40	0.2213	0.0833	0.2484	0.1906	0.0586	0.5014199	Mineral
Longitude	norb_Cluster42	0.2825	0.1761	0.6165	0.587	0.0005	0.0220937	Mineral
Longitude	norb_Cluster44	0.0249	-0.0062	0.0668	-0.005	0.3523	0.8052395	Mineral
Longitude	norb_Cluster46	0.2575	0.0868	0.1537	0.0886	0.1484	0.6892045	Mineral
Longitude	norb_Cluster47	0.6453	0.2955	0.4061	0.3604	0.0106	0.1956212	Mineral
Longitude	norb_Cluster5	0.0115	0.004	0.1731	0.1095	0.123	0.6704217	Mineral
Longitude	norb_Cluster51	0.0122	0.0024	0.0203	-0.0551	0.6129	0.8683974	Mineral
Longitude	norb_Cluster52	0.2809	0.1291	0.3299	0.2784	0.0251	0.2964303	Mineral
Longitude	norb_Cluster56	0.0178	0.0031	0.046	-0.0274	0.4426	0.810923	Mineral
Longitude	norb_Cluster57	0.004	0.0011	0.0757	0.0046	0.3209	0.8052395	Mineral
Longitude	norb_Cluster59	0.0322	-0.0172	0.2235	0.1638	0.0751	0.5683671	Mineral
Longitude	norb_Cluster6	0.6934	0.4268	0.6637	0.6378	0.0002	0.017437	Mineral
Longitude	norb_Cluster60	0.345	0.2247	0.3358	0.2847	0.0236	0.2838888	Mineral
Longitude	norb_Cluster61	0.5486	0.1187	0.084	0.0135	0.2948	0.8052395	Mineral
Longitude	norb_Cluster62	0.003	0.0003	0.006	-0.0704	0.7835	0.9151759	Mineral
Longitude	norb_Cluster64	0.0407	0.0032	0.0163	-0.0594	0.6501	0.8775051	Mineral
Longitude	norb_Cluster66	0.0016	0.0004	0.0133	-0.0626	0.6824	0.8862043	Mineral
Longitude	norb_Cluster67	0.0279	-0.0071	0.0963	0.0268	0.2603	0.8052395	Mineral
Longitude	norb_Cluster68	0.003	0.0004	0.0116	-0.0645	0.703	0.8900961	Mineral
Longitude	norb_Cluster69	0.0032	0.0006	0.0163	-0.0593	0.65	0.8775051	Mineral
Longitude	norb_Cluster7	0.0786	0.0339	0.2621	0.2053	0.0511	0.4708164	Mineral
Longitude	norb_Cluster70	0.1262	0.0181	0.0524	-0.0205	0.4119	0.8052395	Mineral
Longitude	norb_Cluster71	0.1512	0.0194	0.0506	-0.0224	0.4201	0.810051	Mineral
Longitude	norb_Cluster74	0.0387	0.0032	0.0167	-0.0589	0.6459	0.8775051	Mineral
Longitude	norb_Cluster75	0.1531	0.0204	0.0726	0.0013	0.3314	0.8052395	Mineral
Longitude	norb_Cluster76	0.1215	0.0727	0.2186	0.1585	0.0788	0.5703289	Mineral
Longitude	norb_Cluster77	0.0492	0.0039	0.0173	-0.0583	0.64	0.8775051	Mineral
Longitude	norb_Cluster8	0.0206	0.0058	0.0962	0.0267	0.2605	0.8052395	Mineral
Longitude	norb_Cluster80	0.0021	0.0007	0.0358	-0.0384	0.4995	0.8348441	Mineral
Longitude	norb_Cluster81	0.0013	0.0006	0.053	-0.0198	0.409	0.8052395	Mineral
Longitude	norb_Cluster83	0.011	0.001	0.0029	-0.0738	0.8478	0.9392862	Mineral
Longitude	norb_Cluster84	0.0093	-0.0001	0.0001	-0.0768	0.9697	0.9825468	Mineral
Longitude	norb_Cluster85	0.0025	0.0013	0.0861	0.0158	0.2885	0.8052395	Mineral
Longitude	norb_Cluster86	0.041	0.0202	0.0915	0.0216	0.2731	0.8052395	Mineral
Longitude	norb_Cluster88	0.0019	0.0007	0.0457	-0.0278	0.4445	0.810923	Mineral
Longitude	norb_Cluster90	0.0013	-0.0003	0.0061	-0.0703	0.7812	0.9151759	Mineral
Longitude	norb_Cluster91	0.7571	0.2233	0.1269	0.0598	0.1924	0.7705672	Mineral
Longitude	norb_Cluster92	0.0021	-0.0005	0.0085	-0.0677	0.7433	0.9047477	Mineral
Longitude	norb_Cluster93	0.0028	0.0017	0.0947	0.0251	0.2644	0.8052395	Mineral
Longitude	norb_Cluster94	0.002	-0.0013	0.1649	0.1007	0.1331	0.6704217	Mineral
Longitude	norb_Cluster96	0.0006	-0.0004	0.0555	-0.0171	0.3979	0.8052395	Mineral
Longitude	norb_Cluster97	0.0162	-0.008	0.2202	0.1602	0.0776	0.5703289	Mineral
Longitude	norb_Cluster98	0.0176	0.0086	0.1567	0.0919	0.1441	0.6828758	Mineral
Longitude	nosz_Cluster0	0.0057	-0.0015	0.0567	-0.0158	0.3926	0.8052395	Mineral
Longitude	nosz_Cluster10	0.0128	0.0039	0.0925	0.0227	0.2705	0.8052395	Mineral

Longitude	nosz_Cluster102	0.0057	-0.0021	0.2152	0.1548	0.0815	0.5716535	Mineral
Longitude	nosz_Cluster103	0.0496	0.0108	0.0818	0.0112	0.3014	0.8052395	Mineral
Longitude	nosz_Cluster105	0.0016	-0.0006	0.0374	-0.0366	0.4895	0.8258412	Mineral
Longitude	nosz_Cluster106	0.0088	0.0023	0.0531	-0.0198	0.4088	0.8052395	Mineral
Longitude	nosz_Cluster107	0.0003	0.0002	0.0207	-0.0546	0.609	0.8683974	Mineral
Longitude	nosz_Cluster108	0.0043	0.0014	0.0461	-0.0273	0.4424	0.810923	Mineral
Longitude	nosz_Cluster109	0.0416	0.0023	0.0107	-0.0654	0.7137	0.8942401	Mineral
Longitude	nosz_Cluster11	0.0104	-0.0022	0.0496	-0.0235	0.4247	0.810923	Mineral
Longitude	nosz_Cluster110	0.0004	0.0004	0.0643	-0.0077	0.3619	0.8052395	Mineral
Longitude	nosz_Cluster111	0.0078	0.001	0.0165	-0.0591	0.6481	0.8775051	Mineral
Longitude	nosz_Cluster116	0.0074	-0.0005	0.0056	-0.0709	0.7918	0.9163049	Mineral
Longitude	nosz_Cluster118	0.0064	0.0014	0.0273	-0.0475	0.5563	0.8476038	Mineral
Longitude	nosz_Cluster119	0.0019	0.0004	0.0147	-0.0611	0.6669	0.8775051	Mineral
Longitude	nosz_Cluster120	0.0083	-0.0008	0.0094	-0.0668	0.7315	0.896443	Mineral
Longitude	nosz_Cluster123	0.0136	0.0008	0.0029	-0.0738	0.8492	0.9392862	Mineral
Longitude	nosz_Cluster128	0.005	0.0025	0.1356	0.0691	0.1769	0.7564888	Mineral
Longitude	nosz_Cluster129	0.05	0.0163	0.4195	0.3749	0.009	0.1719146	Mineral
Longitude	nosz_Cluster13	0.0124	0.0008	0.004	-0.0726	0.8218	0.9259972	Mineral
Longitude	nosz_Cluster130	0.1138	0.0523	0.0535	-0.0193	0.4067	0.8052395	Mineral
Longitude	nosz_Cluster132	0.004	0.0004	0.0098	-0.0664	0.7261	0.896443	Mineral
Longitude	nosz_Cluster14	0.0149	0.001	0.0029	-0.0738	0.8482	0.9392862	Mineral
Longitude	nosz_Cluster16	0.008	0.0017	0.0221	-0.0531	0.5972	0.8656584	Mineral
Longitude	nosz_Cluster17	0.0427	0.0068	0.0416	-0.0321	0.4657	0.810923	Mineral
Longitude	nosz_Cluster18	0.0184	-0.0033	0.0226	-0.0526	0.5929	0.8632482	Mineral
Longitude	nosz_Cluster20	0.0117	0.0032	0.0426	-0.031	0.4605	0.810923	Mineral
Longitude	nosz_Cluster21	0.0117	0.003	0.042	-0.0317	0.4637	0.810923	Mineral
Longitude	nosz_Cluster22	0.0325	0.0102	0.049	-0.0242	0.4279	0.810923	Mineral
Longitude	nosz_Cluster23	0.019	0.0031	0.0213	-0.054	0.6041	0.8683974	Mineral
Longitude	nosz_Cluster24	0.0019	-0.0009	0.0613	-0.0109	0.3736	0.8052395	Mineral
Longitude	nosz_Cluster25	0.0026	-0.0007	0.0231	-0.0521	0.5889	0.8599568	Mineral
Longitude	nosz_Cluster26	0.0068	0.0019	0.0374	-0.0367	0.4899	0.8258412	Mineral
Longitude	nosz_Cluster27	0.007	-0.001	0.0158	-0.0599	0.6554	0.8775051	Mineral
Longitude	nosz_Cluster28	0.0358	0.0055	0.0291	-0.0456	0.5435	0.8471771	Mineral
Longitude	nosz_Cluster30	0.0341	-0.0004	0.0002	-0.0767	0.9559	0.9800449	Mineral
Longitude	nosz_Cluster32	0.016	-0.0015	0.0099	-0.0662	0.7238	0.896443	Mineral
Longitude	nosz_Cluster33	0.0187	0.0058	0.0712	-0.0002	0.3364	0.8052395	Mineral
Longitude	nosz_Cluster34	0.0387	0.0158	0.0988	0.0295	0.2538	0.8052395	Mineral
Longitude	nosz_Cluster35	0.0097	0.0003	0.0011	-0.0757	0.9067	0.9544285	Mineral
Longitude	nosz_Cluster36	0.0213	0.0061	0.087	0.0168	0.2857	0.8052395	Mineral
Longitude	nosz_Cluster37	0.0077	-0.0011	0.0152	-0.0605	0.6611	0.8775051	Mineral
Longitude	nosz_Cluster38	0.0144	-0.0023	0.013	-0.0629	0.6853	0.8867081	Mineral
Longitude	nosz_Cluster39	0.0653	-0.0041	0.0062	-0.0703	0.7807	0.9151759	Mineral
Longitude	nosz_Cluster40	0.0262	-0.0043	0.0256	-0.0493	0.5687	0.8476038	Mineral
Longitude	nosz_Cluster41	0.0258	-0.0026	0.0094	-0.0668	0.7309	0.896443	Mineral
Longitude	nosz_Cluster42	0.03	0.0052	0.0276	-0.0472	0.5542	0.8476038	Mineral
Longitude	nosz_Cluster43	0.001	0.0003	0.0117	-0.0643	0.7014	0.8900961	Mineral
Longitude	nosz_Cluster44	0.0068	0.002	0.0562	-0.0163	0.3947	0.8052395	Mineral
Longitude	nosz_Cluster45	0.0056	0.0008	0.0117	-0.0644	0.7015	0.8900961	Mineral
Longitude	nosz_Cluster46	0.0082	0.0005	0.004	-0.0726	0.8223	0.9259972	Mineral
Longitude	nosz_Cluster47	0.0012	0.0005	0.0629	-0.0092	0.3674	0.8052395	Mineral
Longitude	nosz_Cluster48	0.0028	0.0003	0.0045	-0.0721	0.8132	0.9259972	Mineral
Longitude	nosz_Cluster49	0.0012	-0.0005	0.0335	-0.0409	0.5141	0.8425891	Mineral
Longitude	nosz_Cluster5	0.004	0.0013	0.0408	-0.033	0.4706	0.8151622	Mineral
Longitude	nosz_Cluster51	0.003	0.0009	0.0466	-0.0267	0.4397	0.810923	Mineral
Longitude	nosz_Cluster53	0.0012	-0.0005	0.0258	-0.0492	0.5677	0.8476038	Mineral

Longitude	nosz_Cluster54	0.0037	0.0015	0.0686	-0.0031	0.3458	0.8052395	Mineral
Longitude	nosz_Cluster57	0.2232	0.072	0.144	0.0781	0.1631	0.7180151	Mineral
Longitude	nosz_Cluster58	0.0053	0.0017	0.0441	-0.0294	0.4524	0.810923	Mineral
Longitude	nosz_Cluster59	0.0008	0.0001	0.0015	-0.0753	0.8913	0.9526986	Mineral
Longitude	nosz_Cluster6	0.0034	0.0006	0.0119	-0.0641	0.6992	0.8900961	Mineral
Longitude	nosz_Cluster60	0.0099	-0.0023	0.0382	-0.0358	0.4853	0.8258412	Mineral
Longitude	nosz_Cluster61	0.0178	0.0031	0.0734	0.0021	0.3287	0.8052395	Mineral
Longitude	nosz_Cluster62	0.0493	0.0155	0.1143	0.0461	0.2179	0.7973679	Mineral
Longitude	nosz_Cluster64	0.0023	-0.0022	0.1288	0.0618	0.189	0.7705672	Mineral
Longitude	nosz_Cluster65	0.0083	0.0002	0.0005	-0.0764	0.9379	0.9742567	Mineral
Longitude	nosz_Cluster66	0.0428	0.0197	0.1988	0.1371	0.0958	0.6041774	Mineral
Longitude	nosz_Cluster67	0.0669	0.023	0.1125	0.0442	0.2216	0.7973679	Mineral
Longitude	nosz_Cluster69	0.008	0.0036	0.1767	0.1134	0.1187	0.6704217	Mineral
Longitude	nosz_Cluster70	0.0655	0.0217	0.0419	-0.0318	0.4642	0.810923	Mineral
Longitude	nosz_Cluster71	0.0101	0.0019	0.0178	-0.0577	0.6354	0.8775051	Mineral
Longitude	nosz_Cluster72	0.066	0.0251	0.1721	0.1084	0.1242	0.6704217	Mineral
Longitude	nosz_Cluster73	0.0031	0.00E+00	1.00E-04	-0.0768	0.9725	0.9825468	Mineral
Longitude	nosz_Cluster74	0.0017	0.0003	0.0113	-0.0648	0.7064	0.89055	Mineral
Longitude	nosz_Cluster75	0.132	0.0598	0.0425	-0.0311	0.4609	0.810923	Mineral
Longitude	nosz_Cluster77	0.0002	0.0002	0.0643	-0.0077	0.3619	0.8052395	Mineral
Longitude	nosz_Cluster78	0.0054	0.0018	0.0863	0.016	0.288	0.8052395	Mineral
Longitude	nosz_Cluster79	0.0401	-0.0016	0.0026	-0.0741	0.8559	0.9408632	Mineral
Longitude	nosz_Cluster8	0.0106	0.0007	0.0074	-0.0689	0.7601	0.9085432	Mineral
Longitude	nosz_Cluster80	0.0039	0.001	0.0354	-0.0388	0.5016	0.8360225	Mineral
Longitude	nosz_Cluster81	0.0099	0.0029	0.0302	-0.0444	0.5358	0.8471771	Mineral
Longitude	nosz_Cluster82	0.0023	-0.0038	0.2955	0.2413	0.0362	0.374873	Mineral
Longitude	nosz_Cluster83	0.0014	0.0002	0.0028	-0.0739	0.8501	0.9392862	Mineral
Longitude	nosz_Cluster85	0.0027	-0.0024	0.1953	0.1334	0.0991	0.6041774	Mineral
Longitude	nosz_Cluster86	0.0038	-0.002	0.1329	0.0662	0.1816	0.7595118	Mineral
Longitude	nosz_Cluster87	0.0034	-0.0012	0.0709	-0.0005	0.3373	0.8052395	Mineral
Longitude	nosz_Cluster88	0.0246	0.0059	0.1485	0.083	0.156	0.7082233	Mineral
Longitude	nosz_Cluster89	0.0607	0.023	0.1698	0.106	0.1269	0.6704217	Mineral
Longitude	nosz_Cluster9	0.0034	0.00E+00	0.00E+00	-0.0769	0.9944	0.999544	Mineral
Longitude	nosz_Cluster90	0.0139	0.0013	0.0171	-0.0585	0.6421	0.8775051	Mineral
Longitude	nosz_Cluster91	0.0232	0.0043	0.0274	-0.0474	0.5552	0.8476038	Mineral
Longitude	nosz_Cluster93	0.0488	0.0039	0.0087	-0.0675	0.7407	0.9047477	Mineral
Longitude	nosz_Cluster95	0.0094	0.003	0.1216	0.054	0.2028	0.7756759	Mineral
Longitude	nosz_Cluster96	0.0064	-0.0011	0.0348	-0.0394	0.5053	0.8374309	Mineral
Longitude	nosz_Cluster97	0.0026	0.0002	0.0026	-0.0741	0.8563	0.9408632	Mineral
Longitude	nosz_Cluster98	0.0035	-0.005	0.2423	0.1841	0.0623	0.5251319	Mineral
Longitude	nosz_Cluster99	0.0061	-0.0002	0.0012	-0.0756	0.901	0.9526986	Mineral
Longitude	nrfa_Cluster10	0.0978	0.0113	0.0116	-0.0645	0.703	0.8900961	Mineral
Longitude	nrfa_Cluster100	0.1249	0.0131	0.0068	-0.0696	0.7695	0.9146383	Mineral
Longitude	nrfa_Cluster104	0.1478	-0.02	0.0309	-0.0437	0.5312	0.8471771	Mineral
Longitude	nrfa_Cluster105	0.006	0.0004	0.0024	-0.0744	0.8631	0.9456804	Mineral
Longitude	nrfa_Cluster109	0.2753	-0.0878	0.1908	0.1285	0.1035	0.6097173	Mineral
Longitude	nrfa_Cluster116	0.3208	-0.0524	0.0932	0.0234	0.2686	0.8052395	Mineral
Longitude	nrfa_Cluster118	0.2298	0.0262	0.0069	-0.0695	0.7693	0.9146383	Mineral
Longitude	nrfa_Cluster125	0.1081	0.0105	0.0045	-0.0721	0.8129	0.9259972	Mineral
Longitude	nrfa_Cluster154	0.0837	0.0024	0.0005	-0.0764	0.9353	0.9732142	Mineral
Longitude	nrfa_Cluster164	0.0044	0.0008	0.0206	-0.0548	0.61	0.8683974	Mineral
Longitude	nrfa_Cluster172	0.0036	0.0016	0.0641	-0.0079	0.3624	0.8052395	Mineral
Longitude	nrfa_Cluster175	0.0053	0.0009	0.0078	-0.0686	0.7549	0.9071073	Mineral
Longitude	nrfa_Cluster20	0.045	-0.0147	0.0897	0.0196	0.2782	0.8052395	Mineral
Longitude	nrfa_Cluster26	0.1433	0.0211	0.0127	-0.0632	0.6888	0.8881936	Mineral

Longitude	nrfa_Cluster29	0.0059	0.0038	0.195	0.1331	0.0993	0.6041774	Mineral
Longitude	nrfa_Cluster34	0.0642	-0.0164	0.0578	-0.0147	0.3882	0.8052395	Mineral
Longitude	nrfa_Cluster38	0.0033	0.0006	0.0096	-0.0666	0.7286	0.896443	Mineral
Longitude	nrfa_Cluster61	0.0047	-0.002	0.0814	0.0107	0.3028	0.8052395	Mineral
Longitude	nrfa_Cluster62	0.0589	0.0058	0.0045	-0.0721	0.8131	0.9259972	Mineral
Longitude	nrfa_Cluster66	0.1282	-0.0156	0.0343	-0.0399	0.5085	0.8379568	Mineral
Longitude	nrfa_Cluster69	0.0035	0.0018	0.1088	0.0402	0.23	0.8027796	Mineral
Longitude	nrfa_Cluster71	0.0322	-0.0007	0.0002	-0.0767	0.9568	0.9800449	Mineral
Longitude	nrfa_Cluster72	0.0969	-0.0023	0.0004	-0.0764	0.9408	0.9755143	Mineral
Longitude	nrfa_Cluster73	0.0042	0.0015	0.0407	-0.0331	0.4711	0.8151622	Mineral
Longitude	nrfa_Cluster74	0.1692	0.0216	0.0126	-0.0634	0.691	0.8881936	Mineral
Longitude	nrfa_Cluster76	0.0922	0.0144	0.015	-0.0608	0.6641	0.8775051	Mineral
Longitude	nrfa_Cluster79	0.0138	0.006	0.0396	-0.0343	0.4771	0.8206676	Mineral
Longitude	nrfa_Cluster87	0.0207	0.0098	0.0757	0.0046	0.3211	0.8052395	Mineral
Longitude	nrfa_Cluster89	0.0023	0.0008	0.0288	-0.0459	0.5455	0.8471771	Mineral
Longitude	nrfa_Cluster92	0.0041	0.0018	0.1211	0.0535	0.2038	0.7756759	Mineral
Longitude	nrfa_Cluster93	0.0581	0.0332	0.1361	0.0697	0.1759	0.7564888	Mineral
Longitude	nrfa_Cluster94	0.0003	0.00E+00	0.0015	-0.0753	0.8913	0.9526986	Mineral
Longitude	nrfa_Cluster97	0.0225	0.0046	0.0466	-0.0267	0.4396	0.810923	Mineral
Longitude	nrfa_Cluster99	0.0184	0.0031	0.0252	-0.0498	0.5718	0.8476038	Mineral
Elevation	amoA_A_Cluster26	0.0053	-0.0046	0.2873	0.2324	0.0395	0.4083886	Mineral
Elevation	amoA_A_Cluster45	0.0071	-0.0037	0.1516	0.0864	0.1514	0.5617054	Mineral
Elevation	amoA_B_Cluster0	0.1272	-0.0062	0.0197	-0.0557	0.6174	0.8580172	Mineral
Elevation	amoA_B_Cluster1	0.0205	0.013	0.6653	0.6395	0.0002	0.01882	Mineral
Elevation	amoA_B_Cluster10	0.0539	-0.0026	0.0102	-0.0659	0.7203	0.9133673	Mineral
Elevation	amoA_B_Cluster11	0.0486	0.0216	0.5079	0.47	0.0029	0.0890102	Mineral
Elevation	amoA_B_Cluster12	0.093	0.0023	0.0038	-0.0728	0.8274	0.9587544	Mineral
Elevation	amoA_B_Cluster13	0.6417	-0.0479	0.0172	-0.0584	0.641	0.859467	Mineral
Elevation	amoA_B_Cluster14	0.0339	-0.0081	0.0842	0.0137	0.2942	0.7047594	Mineral
Elevation	amoA_B_Cluster15	0.0413	0.0082	0.0983	0.0289	0.2553	0.6653333	Mineral
Elevation	amoA_B_Cluster2	0.2143	0.0103	0.0076	-0.0687	0.7575	0.9330426	Mineral
Elevation	amoA_B_Cluster20	0.0078	0.0043	0.18	0.1169	0.115	0.5049196	Mineral
Elevation	amoA_B_Cluster23	0.0626	0.0211	0.431	0.3872	0.0079	0.1782256	Mineral
Elevation	amoA_B_Cluster3	0.0244	0.0132	0.4278	0.3837	0.0082	0.178518	Mineral
Elevation	amoA_B_Cluster4	0.0169	0.0019	0.0151	-0.0607	0.6631	0.8791714	Mineral
Elevation	amoA_B_Cluster5	0.1032	0.0045	0.0131	-0.0629	0.6851	0.8902919	Mineral
Elevation	amoA_B_Cluster6	0.0279	0.0076	0.1975	0.1358	0.097	0.4845739	Mineral
Elevation	amoA_B_Cluster7	0.0232	0.0125	0.3616	0.3125	0.0177	0.2905089	Mineral
Elevation	amoA_B_Cluster8	0.0268	0.0189	0.596	0.5649	0.0007	0.0400183	Mineral
Elevation	amoA_B_Cluster9	0.0693	0.0314	0.3714	0.3231	0.0159	0.2715642	Mineral
Elevation	nifh_Cluster0	0.0318	0.0045	0.0679	-0.0038	0.3481	0.7277367	Mineral
Elevation	nifh_Cluster10	0.0033	-0.0003	0.0045	-0.0721	0.8128	0.9567112	Mineral
Elevation	nifh_Cluster100	0.002	-0.001	0.0815	0.0108	0.3025	0.7081466	Mineral
Elevation	nifh_Cluster103	0.0433	0.0004	0.0003	-0.0766	0.9519	0.9767256	Mineral
Elevation	nifh_Cluster1034	0.0337	-0.0057	0.0299	-0.0447	0.5377	0.827899	Mineral
Elevation	nifh_Cluster105	0.0164	-0.0058	0.1127	0.0445	0.2212	0.6244214	Mineral
Elevation	nifh_Cluster108	0.0076	-0.0035	0.2075	0.1466	0.0879	0.4812414	Mineral
Elevation	nifh_Cluster1099	0.0478	0.0048	0.0058	-0.0706	0.7868	0.9456687	Mineral
Elevation	nifh_Cluster11	0.0025	-0.0011	0.105	0.0362	0.2387	0.6373128	Mineral
Elevation	nifh_Cluster1112	0.0037	0.0011	0.0395	-0.0344	0.4779	0.8078339	Mineral
Elevation	nifh_Cluster112	0.0098	-0.0017	0.0162	-0.0595	0.6517	0.8699577	Mineral
Elevation	nifh_Cluster113	0.0374	-0.0004	0.0005	-0.0764	0.9393	0.9756996	Mineral
Elevation	nifh_Cluster114	0.0304	0.0005	0.0013	-0.0755	0.898	0.9698259	Mineral
Elevation	nifh_Cluster1141	0.0296	-0.0009	0.0012	-0.0756	0.9024	0.9698259	Mineral
Elevation	nifh_Cluster115	0.0098	-0.0002	0.0004	-0.0765	0.9443	0.9767256	Mineral

Elevation	nifh_Cluster116	0.0883	0.038	0.6554	0.6289	0.0003	0.01882	Mineral
Elevation	nifh_Cluster1163	0.0051	0.0023	0.1935	0.1314	0.1009	0.4845739	Mineral
Elevation	nifh_Cluster1179	0.0037	-0.0017	0.0768	0.0057	0.3175	0.7214545	Mineral
Elevation	nifh_Cluster1182	0.0209	-0.0003	0.0008	-0.0761	0.9218	0.9705002	Mineral
Elevation	nifh_Cluster1183	0.0045	0.001	0.0585	-0.0139	0.3851	0.7519237	Mineral
Elevation	nifh_Cluster1197	0.0115	0.0003	0.001	-0.0759	0.9112	0.9700106	Mineral
Elevation	nifh_Cluster1199	0.0048	0.0019	0.1052	0.0364	0.2381	0.6373128	Mineral
Elevation	nifh_Cluster120	0.02	-0.0062	0.0368	-0.0373	0.4936	0.8147683	Mineral
Elevation	nifh_Cluster1203	0.0217	0.0052	0.0656	-0.0063	0.3568	0.7277367	Mineral
Elevation	nifh_Cluster1206	0.006	0.0038	0.4474	0.4049	0.0064	0.164144	Mineral
Elevation	nifh_Cluster1207	0.0078	-0.0014	0.0214	-0.0539	0.6029	0.8580172	Mineral
Elevation	nifh_Cluster1209	0.1617	0.0393	0.4053	0.3596	0.0107	0.217888	Mineral
Elevation	nifh_Cluster121	0.0123	-0.0069	0.1264	0.0592	0.1935	0.5963517	Mineral
Elevation	nifh_Cluster1211	0.0068	0.0055	0.6578	0.6315	0.0002	0.01882	Mineral
Elevation	nifh_Cluster1213	0.0032	-0.0001	0.0016	-0.0752	0.8877	0.9698259	Mineral
Elevation	nifh_Cluster122	0.0041	-0.0023	0.1633	0.099	0.1352	0.5352263	Mineral
Elevation	nifh_Cluster123	0.0092	-0.0041	0.0932	0.0234	0.2686	0.6684333	Mineral
Elevation	nifh_Cluster1236	0.031	-0.0151	0.1319	0.0652	0.1833	0.5963517	Mineral
Elevation	nifh_Cluster1238	0.0034	-0.0021	0.1522	0.0869	0.1506	0.5617054	Mineral
Elevation	nifh_Cluster124	0.0101	0.0006	0.0055	-0.0711	0.7937	0.947959	Mineral
Elevation	nifh_Cluster1261	0.0023	-0.0003	0.0048	-0.0718	0.8069	0.9559836	Mineral
Elevation	nifh_Cluster1262	0.008	-0.0035	0.1375	0.0711	0.1737	0.5963517	Mineral
Elevation	nifh_Cluster1266	0.0072	-0.0054	0.5249	0.4883	0.0023	0.0737938	Mineral
Elevation	nifh_Cluster1267	0.0388	-0.0022	0.0014	-0.0754	0.8939	0.9698259	Mineral
Elevation	nifh_Cluster127	0.0033	-0.0014	0.0525	-0.0204	0.4116	0.7519237	Mineral
Elevation	nifh_Cluster1278	0.2105	0.0488	0.1655	0.1013	0.1323	0.5352263	Mineral
Elevation	nifh_Cluster128	0.0043	-0.0019	0.138	0.0717	0.1727	0.5963517	Mineral
Elevation	nifh_Cluster1289	0.004	-0.0015	0.1088	0.0402	0.2299	0.6314975	Mineral
Elevation	nifh_Cluster129	0.0086	-0.0035	0.0791	0.0083	0.3098	0.7192459	Mineral
Elevation	nifh_Cluster1292	0.0106	0.0023	0.0446	-0.0289	0.45	0.7879023	Mineral
Elevation	nifh_Cluster130	0.0071	-0.0033	0.0605	-0.0118	0.377	0.7463535	Mineral
Elevation	nifh_Cluster1305	0.0048	0.00E+00	0.00E+00	-0.0769	0.9826	0.9927005	Mineral
Elevation	nifh_Cluster1306	0.0055	0.0004	0.0048	-0.0718	0.8064	0.9559836	Mineral
Elevation	nifh_Cluster131	0.0067	-0.003	0.0886	0.0184	0.2814	0.6804013	Mineral
Elevation	nifh_Cluster1319	0.0048	-0.0005	0.0068	-0.0696	0.7703	0.9428508	Mineral
Elevation	nifh_Cluster132	0.0064	-0.0028	0.0952	0.0256	0.2633	0.6666241	Mineral
Elevation	nifh_Cluster1320	0.0028	0.0013	0.0562	-0.0164	0.3951	0.7519237	Mineral
Elevation	nifh_Cluster1323	0.0022	0.0014	0.2158	0.1554	0.0811	0.4645405	Mineral
Elevation	nifh_Cluster1324	0.0235	-0.0145	0.3701	0.3216	0.0161	0.2715642	Mineral
Elevation	nifh_Cluster1336	0.0159	0.003	0.1134	0.0452	0.2197	0.6244214	Mineral
Elevation	nifh_Cluster1340	0.0478	0.0508	0.8204	0.8066	0.00E+00	0.0019821	Mineral
Elevation	nifh_Cluster1345	0.033	0.0027	0.0035	-0.0732	0.8341	0.9587544	Mineral
Elevation	nifh_Cluster1370	0.0022	-0.0002	0.0039	-0.0727	0.8249	0.9587544	Mineral
Elevation	nifh_Cluster1375	0.0017	-0.0001	0.0024	-0.0744	0.8636	0.9628369	Mineral
Elevation	nifh_Cluster139	0.0038	0.0024	0.1473	0.0817	0.1579	0.5703421	Mineral
Elevation	nifh_Cluster140	0.0051	-0.0004	0.0044	-0.0722	0.814	0.9567112	Mineral
Elevation	nifh_Cluster141	0.0808	-0.001	0.0013	-0.0755	0.8971	0.9698259	Mineral
Elevation	nifh_Cluster148	0.006	0.0041	0.1849	0.1222	0.1097	0.4901939	Mineral
Elevation	nifh_Cluster1480	0.0006	0.0002	0.0133	-0.0626	0.682	0.8882985	Mineral
Elevation	nifh_Cluster152	0.0602	-0.0111	0.0797	0.009	0.3079	0.7179156	Mineral
Elevation	nifh_Cluster156	0.0459	-0.0143	0.2021	0.1407	0.0927	0.4845739	Mineral
Elevation	nifh_Cluster1562	0.0293	-0.0026	0.0028	-0.0739	0.8508	0.9603863	Mineral
Elevation	nifh_Cluster158	0.019	0.0002	1.00E-04	-0.0768	0.9739	0.9857699	Mineral
Elevation	nifh_Cluster16	0.0023	-0.0014	0.097	0.0275	0.2585	0.6653333	Mineral
Elevation	nifh_Cluster166	0.0165	-0.0059	0.0524	-0.0204	0.4116	0.7519237	Mineral

Elevation	nifh_Cluster205	0.0121	-0.0004	0.002	-0.0747	0.8729	0.9668722	Mineral
Elevation	nifh_Cluster21	0.0022	-0.0003	0.0058	-0.0707	0.787	0.9456687	Mineral
Elevation	nifh_Cluster222	0.0069	-0.0024	0.0401	-0.0337	0.474	0.8078339	Mineral
Elevation	nifh_Cluster225	0.021	0.0023	0.0287	-0.046	0.5459	0.827899	Mineral
Elevation	nifh_Cluster230	0.0052	-0.0019	0.1164	0.0484	0.2133	0.6139196	Mineral
Elevation	nifh_Cluster231	0.0019	0.0006	0.031	-0.0435	0.53	0.8272871	Mineral
Elevation	nifh_Cluster236	0.0034	-0.0007	0.034	-0.0403	0.5107	0.8189687	Mineral
Elevation	nifh_Cluster237	0.007	-0.0042	0.3383	0.2874	0.023	0.3302876	Mineral
Elevation	nifh_Cluster243	0.0253	-0.014	0.2073	0.1463	0.0881	0.4812414	Mineral
Elevation	nifh_Cluster2432	0.0317	0.0009	0.0005	-0.0764	0.9384	0.9756996	Mineral
Elevation	nifh_Cluster2433	0.0028	0.0015	0.0578	-0.0147	0.3883	0.7519237	Mineral
Elevation	nifh_Cluster246	0.0068	-0.0016	0.0293	-0.0453	0.5417	0.827899	Mineral
Elevation	nifh_Cluster25	0.0195	-0.0036	0.0914	0.0215	0.2733	0.6727603	Mineral
Elevation	nifh_Cluster256	0.0023	-0.0008	0.0531	-0.0197	0.4085	0.7519237	Mineral
Elevation	nifh_Cluster264	0.0025	-0.001	0.0928	0.023	0.2696	0.6684333	Mineral
Elevation	nifh_Cluster265	0.0702	-0.0025	0.0081	-0.0682	0.7499	0.9269425	Mineral
Elevation	nifh_Cluster267	0.0071	0.0006	0.0068	-0.0696	0.7708	0.9428508	Mineral
Elevation	nifh_Cluster268	0.0109	-0.0017	0.0578	-0.0147	0.3882	0.7519237	Mineral
Elevation	nifh_Cluster269	0.0283	-0.0023	0.006	-0.0705	0.7838	0.9456687	Mineral
Elevation	nifh_Cluster272	0.0012	-0.0003	0.0182	-0.0573	0.6317	0.8580172	Mineral
Elevation	nifh_Cluster274	0.0014	-0.0006	0.0178	-0.0578	0.636	0.8580172	Mineral
Elevation	nifh_Cluster275	0.0051	-0.0013	0.0752	0.0041	0.3225	0.7214545	Mineral
Elevation	nifh_Cluster278	0.0056	0.0011	0.0257	-0.0493	0.5683	0.8445465	Mineral
Elevation	nifh_Cluster279	0.0058	0.0002	0.0007	-0.0761	0.9244	0.9705002	Mineral
Elevation	nifh_Cluster28	0.0017	0.00E+00	0.0001	-0.0768	0.9712	0.9857699	Mineral
Elevation	nifh_Cluster280	0.0034	0.0034	0.2338	0.1749	0.0678	0.450158	Mineral
Elevation	nifh_Cluster282	0.0052	0.0008	0.0236	-0.0515	0.5848	0.8526487	Mineral
Elevation	nifh_Cluster287	0.003	-0.0005	0.0176	-0.058	0.6372	0.8580172	Mineral
Elevation	nifh_Cluster290	0.0096	-0.0047	0.1439	0.0781	0.1631	0.5774817	Mineral
Elevation	nifh_Cluster297	0.004	-0.0027	0.1094	0.0409	0.2285	0.6314975	Mineral
Elevation	nifh_Cluster299	0.0025	-0.0013	0.1255	0.0583	0.1951	0.5963517	Mineral
Elevation	nifh_Cluster303	0.002	-0.0016	0.2718	0.2157	0.0463	0.4247495	Mineral
Elevation	nifh_Cluster304	0.0025	-0.0014	0.1344	0.0678	0.179	0.5963517	Mineral
Elevation	nifh_Cluster313	0.0129	-0.0058	0.0839	0.0134	0.295	0.7047594	Mineral
Elevation	nifh_Cluster314	0.0027	-0.001	0.0636	-0.0084	0.3645	0.7290933	Mineral
Elevation	nifh_Cluster317	0.0089	-0.0045	0.2516	0.1941	0.0568	0.4356571	Mineral
Elevation	nifh_Cluster32	0.0029	-0.0022	0.386	0.3388	0.0134	0.2635219	Mineral
Elevation	nifh_Cluster320	13.944	-1.1618	0.1927	0.1306	0.1016	0.4845739	Mineral
Elevation	nifh_Cluster334	0.0084	-0.0021	0.0218	-0.0534	0.5995	0.8580172	Mineral
Elevation	nifh_Cluster335	0.0059	-0.0017	0.0291	-0.0456	0.5433	0.827899	Mineral
Elevation	nifh_Cluster338	0.0151	0.0029	0.0567	-0.0158	0.3926	0.7519237	Mineral
Elevation	nifh_Cluster340	0.0041	0.0008	0.0278	-0.047	0.5527	0.8306675	Mineral
Elevation	nifh_Cluster341	0.0021	-0.0019	0.2644	0.2078	0.0499	0.4264253	Mineral
Elevation	nifh_Cluster35	0.0079	0.0048	0.5473	0.5125	0.0016	0.0561223	Mineral
Elevation	nifh_Cluster351	0.0035	-0.001	0.0338	-0.0406	0.5122	0.8189687	Mineral
Elevation	nifh_Cluster3574	0.1191	-0.0074	0.0137	-0.0622	0.6782	0.8875059	Mineral
Elevation	nifh_Cluster3691	0.0224	0.0162	0.4351	0.3917	0.0075	0.1760462	Mineral
Elevation	nifh_Cluster37	0.0374	-0.0105	0.0652	-0.0067	0.3583	0.7277367	Mineral
Elevation	nifh_Cluster374	0.0029	0.0041	0.788	0.7716	0.00E+00	0.0029679	Mineral
Elevation	nifh_Cluster38	0.0008	-0.0005	0.0724	0.001	0.3322	0.7259547	Mineral
Elevation	nifh_Cluster382	0.0023	-0.0012	0.0567	-0.0158	0.3926	0.7519237	Mineral
Elevation	nifh_Cluster384	0.0023	-0.0015	0.1677	0.1036	0.1296	0.5310941	Mineral
Elevation	nifh_Cluster386	0.0041	-0.0009	0.0151	-0.0607	0.6631	0.8791714	Mineral
Elevation	nifh_Cluster389	0.003	-0.0009	0.0197	-0.0557	0.6175	0.8580172	Mineral
Elevation	nifh_Cluster394	0.0027	-0.0009	0.0217	-0.0535	0.6002	0.8580172	Mineral

Elevation	nifh_Cluster40	0.0014	-0.0008	0.0938	0.024	0.267	0.6684333	Mineral
Elevation	nifh_Cluster43	0.0107	-0.0015	0.0295	-0.0452	0.5406	0.827899	Mineral
Elevation	nifh_Cluster434	0.0048	0.0011	0.0299	-0.0447	0.5378	0.827899	Mineral
Elevation	nifh_Cluster440	0.0171	0.0084	0.2064	0.1453	0.0889	0.4812414	Mineral
Elevation	nifh_Cluster462	0.0048	0.0004	0.0036	-0.073	0.8314	0.9587544	Mineral
Elevation	nifh_Cluster470	0.017	0.0086	0.3355	0.2843	0.0237	0.3323748	Mineral
Elevation	nifh_Cluster49	0.0223	-0.0128	0.1437	0.0778	0.1635	0.5774817	Mineral
Elevation	nifh_Cluster499	0.0028	-0.0012	0.0775	0.0065	0.3151	0.7214545	Mineral
Elevation	nifh_Cluster5	0.0263	0.0068	0.2355	0.1767	0.0667	0.450158	Mineral
Elevation	nifh_Cluster506	0.0058	0.0012	0.0323	-0.0422	0.5217	0.8204359	Mineral
Elevation	nifh_Cluster51	0.0031	-0.0012	0.0383	-0.0357	0.4848	0.8147683	Mineral
Elevation	nifh_Cluster52	0.0396	-0.0201	0.1718	0.1081	0.1245	0.5196658	Mineral
Elevation	nifh_Cluster525	0.0025	-0.0009	0.0289	-0.0458	0.5449	0.827899	Mineral
Elevation	nifh_Cluster531	0.0129	-0.007	0.1954	0.1335	0.099	0.4845739	Mineral
Elevation	nifh_Cluster539	0.0056	-0.0012	0.0093	-0.0669	0.733	0.9162075	Mineral
Elevation	nifh_Cluster578	0.0593	0.023	0.3834	0.3359	0.0138	0.2635219	Mineral
Elevation	nifh_Cluster58	0.2389	-0.0055	0.0003	-0.0766	0.95	0.9767256	Mineral
Elevation	nifh_Cluster60	0.011	-0.005	0.153	0.0879	0.1494	0.5617054	Mineral
Elevation	nifh_Cluster61	0.0017	-0.001	0.1467	0.081	0.1588	0.5703421	Mineral
Elevation	nifh_Cluster65	0.0085	-0.0023	0.0516	-0.0213	0.4155	0.7565641	Mineral
Elevation	nifh_Cluster686	0.0013	-0.0005	0.05	-0.0231	0.423	0.7608291	Mineral
Elevation	nifh_Cluster69	0.0092	-0.0068	0.2461	0.1881	0.06	0.4356571	Mineral
Elevation	nifh_Cluster70	0.0144	-0.0061	0.0664	-0.0054	0.3536	0.7277367	Mineral
Elevation	nifh_Cluster717	0.0024	0.00E+00	0.00E+00	-0.0769	0.9969	0.9995238	Mineral
Elevation	nifh_Cluster725	0.0035	-0.001	0.0642	-0.0078	0.3621	0.7277367	Mineral
Elevation	nifh_Cluster727	0.0038	0.0011	0.0541	-0.0186	0.404	0.7519237	Mineral
Elevation	nifh_Cluster73	0.0133	-0.006	0.1478	0.0822	0.1572	0.5703421	Mineral
Elevation	nifh_Cluster74	0.005	-0.0024	0.0976	0.0282	0.257	0.6653333	Mineral
Elevation	nifh_Cluster748	0.0104	-1.00E-04	0.0002	-0.0767	0.9611	0.982752	Mineral
Elevation	nifh_Cluster749	0.0055	1.00E-04	0.0001	-0.0768	0.9693	0.9857699	Mineral
Elevation	nifh_Cluster755	0.0118	0.0097	0.6275	0.5989	0.0004	0.0256231	Mineral
Elevation	nifh_Cluster76	0.0033	0.00E+00	1.00E-04	-0.0768	0.9741	0.9857699	Mineral
Elevation	nifh_Cluster79	0.002	-0.0011	0.1462	0.0806	0.1595	0.5703421	Mineral
Elevation	nifh_Cluster81	0.0038	-0.0014	0.0579	-0.0146	0.3876	0.7519237	Mineral
Elevation	nifh_Cluster82	0.0014	-0.001	0.1274	0.0602	0.1916	0.5963517	Mineral
Elevation	nifh_Cluster83	0.0055	0.0035	0.2466	0.1886	0.0597	0.4356571	Mineral
Elevation	nifh_Cluster86	0.0161	0.0096	0.4772	0.437	0.0043	0.1283185	Mineral
Elevation	nifh_Cluster868	0.0098	-0.0009	0.0094	-0.0668	0.7317	0.9162075	Mineral
Elevation	nifh_Cluster87	0.003	-0.0014	0.0752	0.0041	0.3226	0.7214545	Mineral
Elevation	nifh_Cluster88	0.0054	0.0063	0.6904	0.6666	0.0001	0.0146783	Mineral
Elevation	nifh_Cluster9	0.0077	-0.0006	0.0032	-0.0734	0.8405	0.9587544	Mineral
Elevation	nifh_Cluster92	0.0068	-0.0027	0.0586	-0.0139	0.3849	0.7519237	Mineral
Elevation	nifh_Cluster93	0.0035	-0.0002	0.0012	-0.0756	0.9024	0.9698259	Mineral
Elevation	nifh_Cluster94	0.005	-0.0025	0.0954	0.0259	0.2626	0.6666241	Mineral
Elevation	nifh_Cluster95	0.0149	-0.0036	0.0308	-0.0438	0.5315	0.8274741	Mineral
Elevation	nifh_Cluster96	0.0131	0.0081	0.3562	0.3066	0.0188	0.2933662	Mineral
Elevation	nifh_Cluster97	0.0125	-0.0008	0.0111	-0.065	0.7092	0.9095678	Mineral
Elevation	nifh_Cluster98	0.0049	0.004	0.5503	0.5158	0.0015	0.0561223	Mineral
Elevation	nifh_Cluster99	0.0035	-0.0016	0.0741	0.0028	0.3265	0.7214545	Mineral
Elevation	nirk_Cluster0	6.5686	-0.5581	0.3243	0.2723	0.0267	0.3501035	Mineral
Elevation	nirk_Cluster1	0.2105	-0.0525	0.1927	0.1306	0.1016	0.4845739	Mineral
Elevation	nirk_Cluster10	0.6649	0.2044	0.7589	0.7403	0.00E+00	0.0046366	Mineral
Elevation	nirk_Cluster101	0.004	-0.0012	0.0466	-0.0267	0.4396	0.7835229	Mineral
Elevation	nirk_Cluster102	0.055	-0.005	0.0184	-0.0571	0.6296	0.8580172	Mineral
Elevation	nirk_Cluster103	0.09	0.0018	0.0012	-0.0756	0.9009	0.9698259	Mineral

Elevation	nirk_Cluster104	0.0028	0.001	0.0468	-0.0265	0.4386	0.7835229	Mineral
Elevation	nirk_Cluster105	0.1172	-0.0114	0.0268	-0.048	0.5596	0.8337449	Mineral
Elevation	nirk_Cluster106	0.073	-0.0099	0.0242	-0.0508	0.5796	0.8526487	Mineral
Elevation	nirk_Cluster107	0.1123	-0.0049	0.0048	-0.0718	0.8065	0.9559836	Mineral
Elevation	nirk_Cluster108	0.0184	0.0015	0.008	-0.0683	0.751	0.9269425	Mineral
Elevation	nirk_Cluster109	0.0158	0.0012	0.0083	-0.068	0.7475	0.9265134	Mineral
Elevation	nirk_Cluster11	0.1509	0.002	0.004	-0.0726	0.8231	0.9587544	Mineral
Elevation	nirk_Cluster12	0.1052	-0.0298	0.4657	0.4246	0.0051	0.1357133	Mineral
Elevation	nirk_Cluster13	0.0418	-0.0055	0.1176	0.0498	0.2108	0.6125298	Mineral
Elevation	nirk_Cluster14	0.1084	0.0185	0.3109	0.2578	0.0308	0.3636288	Mineral
Elevation	nirk_Cluster15	0.4141	-0.0539	0.0513	-0.0217	0.4168	0.7567304	Mineral
Elevation	nirk_Cluster16	0.5816	-0.1024	0.0788	0.0079	0.3109	0.7192459	Mineral
Elevation	nirk_Cluster17	0.1044	-0.0033	0.0083	-0.068	0.7472	0.9265134	Mineral
Elevation	nirk_Cluster18	0.1461	-0.0455	0.2293	0.17	0.071	0.450158	Mineral
Elevation	nirk_Cluster19	0.2953	-0.0303	0.1345	0.068	0.1787	0.5963517	Mineral
Elevation	nirk_Cluster2	0.2004	0.0327	0.2302	0.171	0.0703	0.450158	Mineral
Elevation	nirk_Cluster20	0.1934	0.0189	0.0743	0.003	0.3258	0.7214545	Mineral
Elevation	nirk_Cluster21	0.1711	0.002	0.002	-0.0747	0.8735	0.9668722	Mineral
Elevation	nirk_Cluster22	0.1469	-0.0072	0.0135	-0.0624	0.6799	0.8875059	Mineral
Elevation	nirk_Cluster23	0.0729	-1.00E-04	0.00E+00	-0.0769	0.992	0.9995238	Mineral
Elevation	nirk_Cluster24	0.0753	0.0168	0.3471	0.2969	0.0208	0.3072427	Mineral
Elevation	nirk_Cluster25	0.5519	0.0114	0.0046	-0.072	0.8099	0.9567112	Mineral
Elevation	nirk_Cluster26	0.1119	-0.0047	0.019	-0.0564	0.6241	0.8580172	Mineral
Elevation	nirk_Cluster27	0.2066	0.0177	0.0413	-0.0325	0.4676	0.8078339	Mineral
Elevation	nirk_Cluster28	0.0808	0.0125	0.1245	0.0571	0.1971	0.5994753	Mineral
Elevation	nirk_Cluster29	0.162	0.0471	0.5644	0.5309	0.0012	0.0488745	Mineral
Elevation	nirk_Cluster3	0.3427	0.0539	0.3047	0.2513	0.0329	0.3802752	Mineral
Elevation	nirk_Cluster30	0.0712	-0.0054	0.0187	-0.0567	0.6267	0.8580172	Mineral
Elevation	nirk_Cluster31	0.1423	-0.0019	0.0019	-0.0749	0.8785	0.9687952	Mineral
Elevation	nirk_Cluster32	0.1568	-0.0481	0.1984	0.1368	0.0961	0.4845739	Mineral
Elevation	nirk_Cluster33	0.0812	-0.0157	0.0683	-0.0034	0.3469	0.7277367	Mineral
Elevation	nirk_Cluster34	0.0462	0.0009	0.001	-0.0759	0.9126	0.9700106	Mineral
Elevation	nirk_Cluster35	0.1282	-0.0042	0.0019	-0.0749	0.8771	0.9687952	Mineral
Elevation	nirk_Cluster36	0.0021	0.0003	0.0068	-0.0696	0.7704	0.9428508	Mineral
Elevation	nirk_Cluster39	0.0045	0.0052	0.266	0.2095	0.0491	0.4264253	Mineral
Elevation	nirk_Cluster4	0.1521	0.0067	0.0101	-0.066	0.7212	0.9133673	Mineral
Elevation	nirk_Cluster40	0.0544	0.0006	0.0006	-0.0762	0.9295	0.9723663	Mineral
Elevation	nirk_Cluster41	0.1797	-0.008	0.0236	-0.0516	0.585	0.8526487	Mineral
Elevation	nirk_Cluster44	0.0756	0.0059	0.018	-0.0575	0.6336	0.8580172	Mineral
Elevation	nirk_Cluster46	0.0193	0.0026	0.0175	-0.0581	0.6384	0.8580172	Mineral
Elevation	nirk_Cluster48	0.003	-0.0004	0.0099	-0.0663	0.7245	0.9133673	Mineral
Elevation	nirk_Cluster49	0.0072	-0.0052	0.2711	0.215	0.0466	0.4247495	Mineral
Elevation	nirk_Cluster5	0.2174	-0.0062	0.0096	-0.0666	0.7289	0.9162075	Mineral
Elevation	nirk_Cluster50	0.0014	-0.0001	0.0033	-0.0734	0.8387	0.9587544	Mineral
Elevation	nirk_Cluster52	0.0551	-0.0015	0.0029	-0.0738	0.8478	0.96013	Mineral
Elevation	nirk_Cluster53	0.0039	-0.0014	0.0399	-0.034	0.4756	0.8078339	Mineral
Elevation	nirk_Cluster54	0.0024	-0.0002	0.003	-0.0737	0.846	0.959863	Mineral
Elevation	nirk_Cluster55	0.0028	-0.0015	0.0694	-0.0022	0.3427	0.7277367	Mineral
Elevation	nirk_Cluster56	0.0009	-0.0008	0.1013	0.0322	0.2477	0.6523106	Mineral
Elevation	nirk_Cluster57	0.0017	-0.0013	0.1019	0.0328	0.2463	0.6515405	Mineral
Elevation	nirk_Cluster58	0.0102	0.0036	0.1063	0.0376	0.2356	0.6373128	Mineral
Elevation	nirk_Cluster59	0.007	0.0039	0.2716	0.2155	0.0464	0.4247495	Mineral
Elevation	nirk_Cluster6	0.1713	0.0416	0.4419	0.399	0.0069	0.1685793	Mineral
Elevation	nirk_Cluster60	0.0006	0.0004	0.0536	-0.0192	0.4066	0.7519237	Mineral
Elevation	nirk_Cluster62	0.1789	-0.0034	0.0009	-0.076	0.9158	0.9700106	Mineral

Elevation	nirk_Cluster63	0.1586	0.0084	0.0205	-0.0549	0.6108	0.8580172	Mineral
Elevation	nirk_Cluster64	0.0043	0.0014	0.0646	-0.0073	0.3606	0.7277367	Mineral
Elevation	nirk_Cluster65	0.0531	0.001	0.0015	-0.0754	0.8925	0.9698259	Mineral
Elevation	nirk_Cluster66	0.0095	-0.0038	0.2279	0.1685	0.0719	0.4514137	Mineral
Elevation	nirk_Cluster67	0.0232	0.0182	0.7293	0.7085	1.00E-04	0.0074978	Mineral
Elevation	nirk_Cluster68	0.0158	0.0012	0.0093	-0.0669	0.7323	0.9162075	Mineral
Elevation	nirk_Cluster69	0.0362	0.0031	0.0236	-0.0515	0.5844	0.8526487	Mineral
Elevation	nirk_Cluster7	0.096	0.0216	0.2609	0.2041	0.0517	0.4293393	Mineral
Elevation	nirk_Cluster70	0.0892	-0.0114	0.0169	-0.0588	0.6446	0.8623423	Mineral
Elevation	nirk_Cluster71	0.0071	0.0003	0.0018	-0.075	0.8811	0.9698259	Mineral
Elevation	nirk_Cluster72	0.0278	-0.0008	0.0027	-0.074	0.853	0.9604784	Mineral
Elevation	nirk_Cluster73	0.1947	-0.03	0.1128	0.0445	0.221	0.6244214	Mineral
Elevation	nirk_Cluster74	0.8012	-0.0892	0.0409	-0.0329	0.4697	0.8078339	Mineral
Elevation	nirk_Cluster75	0.0186	-0.0031	0.0212	-0.0541	0.6047	0.8580172	Mineral
Elevation	nirk_Cluster76	0.1377	-0.0039	0.0058	-0.0707	0.7871	0.9456687	Mineral
Elevation	nirk_Cluster77	0.0113	0.0158	0.627	0.5984	0.0004	0.0256231	Mineral
Elevation	nirk_Cluster78	0.3126	0.00E+00	0.00E+00	-0.0769	0.9995	0.9995238	Mineral
Elevation	nirk_Cluster79	0.0096	0.0102	0.5743	0.5415	0.0011	0.0488745	Mineral
Elevation	nirk_Cluster8	0.1182	-0.0025	0.0014	-0.0754	0.8957	0.9698259	Mineral
Elevation	nirk_Cluster80	0.3544	-0.075	0.1704	0.1066	0.1262	0.5207319	Mineral
Elevation	nirk_Cluster82	0.0021	-0.0009	0.0448	-0.0287	0.4491	0.7879023	Mineral
Elevation	nirk_Cluster83	0.0045	0.0023	0.1915	0.1293	0.1028	0.4845739	Mineral
Elevation	nirk_Cluster84	0.0235	-0.0026	0.0108	-0.0653	0.7122	0.9095678	Mineral
Elevation	nirk_Cluster85	0.0053	0.0031	0.2711	0.215	0.0466	0.4247495	Mineral
Elevation	nirk_Cluster86	0.0352	0.0012	0.0023	-0.0745	0.8656	0.9628369	Mineral
Elevation	nirk_Cluster87	0.0138	-0.0043	0.1293	0.0623	0.1881	0.5963517	Mineral
Elevation	nirk_Cluster88	0.0035	0.0005	0.0147	-0.0611	0.6665	0.8804912	Mineral
Elevation	nirk_Cluster89	0.0087	-0.0033	0.1187	0.0509	0.2085	0.609693	Mineral
Elevation	nirk_Cluster9	0.061	-0.0019	0.0025	-0.0742	0.8596	0.9628369	Mineral
Elevation	nirk_Cluster90	0.0087	-0.0022	0.0397	-0.0342	0.4764	0.8078339	Mineral
Elevation	nirk_Cluster91	0.044	-0.0071	0.074	0.0028	0.3266	0.7214545	Mineral
Elevation	nirk_Cluster92	0.009	-0.0027	0.062	-0.0102	0.3709	0.7392512	Mineral
Elevation	nirk_Cluster93	0.0357	-0.0007	0.0007	-0.0762	0.9265	0.9708902	Mineral
Elevation	nirk_Cluster94	0.0222	0.0031	0.033	-0.0413	0.5168	0.8204359	Mineral
Elevation	nirk_Cluster95	0.0566	-0.0086	0.0439	-0.0297	0.4537	0.7896247	Mineral
Elevation	nirk_Cluster96	0.0588	-0.0024	0.0045	-0.0721	0.8121	0.9567112	Mineral
Elevation	nirk_Cluster97	0.0329	-0.0106	0.1639	0.0996	0.1344	0.5352263	Mineral
Elevation	nirk_Cluster98	0.0225	0.0072	0.0969	0.0274	0.2587	0.6653333	Mineral
Elevation	nirk_Cluster99	0.0074	0.0022	0.0658	-0.006	0.356	0.7277367	Mineral
Elevation	nirs_Cluster0	0.1586	0.0352	0.3132	0.2604	0.0301	0.3618384	Mineral
Elevation	nirs_Cluster1	0.0051	0.0015	0.0318	-0.0426	0.5245	0.820896	Mineral
Elevation	nirs_Cluster14	0.0142	-0.0047	0.0329	-0.0415	0.5176	0.8204359	Mineral
Elevation	nirs_Cluster2	0.0174	0.0196	0.5657	0.5323	0.0012	0.0488745	Mineral
Elevation	nirs_Cluster26	0.0007	-0.0001	0.0067	-0.0697	0.7719	0.9428508	Mineral
Elevation	nirs_Cluster28	0.0099	-0.0044	0.0613	-0.0109	0.3736	0.7422167	Mineral
Elevation	nirs_Cluster3	0.1914	0.025	0.2452	0.1871	0.0605	0.4356571	Mineral
Elevation	nirs_Cluster36	0.0018	0.0024	0.1713	0.1076	0.1251	0.5196658	Mineral
Elevation	nirs_Cluster37	0.0092	-0.0025	0.0125	-0.0635	0.6916	0.8948522	Mineral
Elevation	nirs_Cluster4	0.0003	-1.00E-04	0.0031	-0.0736	0.8434	0.9587544	Mineral
Elevation	nirs_Cluster50	0.0024	-0.0002	0.0017	-0.0751	0.8847	0.9698259	Mineral
Elevation	nirs_Cluster9	0.0376	-0.0326	0.2318	0.1727	0.0692	0.450158	Mineral
Elevation	norb_Cluster0	0.0848	-0.0332	0.2897	0.235	0.0385	0.4054766	Mineral
Elevation	norb_Cluster1	1.2746	-0.4158	0.1537	0.0886	0.1483	0.5617054	Mineral
Elevation	norb_Cluster101	0.0037	-0.0021	0.1216	0.054	0.2028	0.6092107	Mineral
Elevation	norb_Cluster102	0.005	-0.0024	0.1582	0.0935	0.142	0.5512015	Mineral

Elevation	norb_Cluster103	0.0025	-0.0011	0.0659	-0.0059	0.3557	0.7277367	Mineral
Elevation	norb_Cluster104	0.0025	0.0004	0.0041	-0.0725	0.8209	0.9587544	Mineral
Elevation	norb_Cluster106	0.1468	-0.0337	0.1462	0.0806	0.1595	0.5703421	Mineral
Elevation	norb_Cluster107	0.0163	-0.0006	0.0015	-0.0753	0.8899	0.9698259	Mineral
Elevation	norb_Cluster108	0.0069	0.0014	0.0502	-0.0228	0.422	0.7608291	Mineral
Elevation	norb_Cluster109	0.0282	-0.0017	0.0056	-0.0709	0.7912	0.9468175	Mineral
Elevation	norb_Cluster11	0.0156	-0.0071	0.208	0.1471	0.0875	0.4812414	Mineral
Elevation	norb_Cluster110	0.0128	0.0018	0.0176	-0.0579	0.6372	0.8580172	Mineral
Elevation	norb_Cluster112	0.0215	-0.0032	0.0543	-0.0184	0.4032	0.7519237	Mineral
Elevation	norb_Cluster113	0.0157	-0.0052	0.1256	0.0584	0.1949	0.5963517	Mineral
Elevation	norb_Cluster114	0.0035	-0.0011	0.0227	-0.0525	0.5918	0.8580172	Mineral
Elevation	norb_Cluster115	0.0034	-0.0008	0.0531	-0.0197	0.4086	0.7519237	Mineral
Elevation	norb_Cluster116	0.0083	-0.003	0.0815	0.0109	0.3022	0.7081466	Mineral
Elevation	norb_Cluster117	0.0034	-0.0003	0.0024	-0.0743	0.861	0.9628369	Mineral
Elevation	norb_Cluster118	0.0044	-1.00E-04	0.0003	-0.0766	0.9545	0.9776803	Mineral
Elevation	norb_Cluster119	0.0263	-0.0034	0.0179	-0.0576	0.6345	0.8580172	Mineral
Elevation	norb_Cluster12	0.005	-0.0003	0.0011	-0.0758	0.9085	0.9700106	Mineral
Elevation	norb_Cluster121	0.0023	-0.002	0.1532	0.0881	0.149	0.5617054	Mineral
Elevation	norb_Cluster122	0.0184	-0.0034	0.046	-0.0273	0.4425	0.7835229	Mineral
Elevation	norb_Cluster123	0.0071	-0.0009	0.0106	-0.0655	0.7148	0.9108658	Mineral
Elevation	norb_Cluster124	0.7452	-0.2646	0.1948	0.1328	0.0996	0.4845739	Mineral
Elevation	norb_Cluster125	0.0052	-0.0018	0.0967	0.0272	0.2594	0.6653333	Mineral
Elevation	norb_Cluster126	0.011	-0.0033	0.0962	0.0267	0.2606	0.6655191	Mineral
Elevation	norb_Cluster127	0.2041	-0.0605	0.1361	0.0696	0.176	0.5963517	Mineral
Elevation	norb_Cluster128	0.0123	-0.0033	0.0828	0.0122	0.2984	0.707063	Mineral
Elevation	norb_Cluster13	0.0051	-0.0037	0.1716	0.1079	0.1247	0.5196658	Mineral
Elevation	norb_Cluster131	0.0209	-0.0081	0.2048	0.1436	0.0903	0.4844765	Mineral
Elevation	norb_Cluster132	1.2771	-0.1434	0.0458	-0.0276	0.4436	0.7835229	Mineral
Elevation	norb_Cluster133	0.0023	0.0019	0.2655	0.209	0.0493	0.4264253	Mineral
Elevation	norb_Cluster135	0.0703	-0.0134	0.0711	-0.0004	0.3368	0.7277367	Mineral
Elevation	norb_Cluster136	0.025	-0.0021	0.0101	-0.0661	0.7221	0.9133673	Mineral
Elevation	norb_Cluster139	0.0391	-0.0014	0.0023	-0.0745	0.8666	0.9628369	Mineral
Elevation	norb_Cluster14	0.0037	-0.0029	0.3719	0.3236	0.0158	0.2715642	Mineral
Elevation	norb_Cluster140	0.0485	-0.0174	0.2742	0.2183	0.0451	0.4247495	Mineral
Elevation	norb_Cluster142	0.0018	-0.0015	0.1325	0.0657	0.1823	0.5963517	Mineral
Elevation	norb_Cluster144	0.0122	0.002	0.0354	-0.0388	0.5022	0.818053	Mineral
Elevation	norb_Cluster146	0.0418	-0.0011	0.0015	-0.0753	0.8906	0.9698259	Mineral
Elevation	norb_Cluster15	0.0502	-0.015	0.1167	0.0487	0.2128	0.6139196	Mineral
Elevation	norb_Cluster150	0.0199	0.0014	0.0109	-0.0652	0.7108	0.9095678	Mineral
Elevation	norb_Cluster155	0.1729	-0.0561	0.1918	0.1296	0.1025	0.4845739	Mineral
Elevation	norb_Cluster16	0.0165	-0.0109	0.3261	0.2742	0.0262	0.3501035	Mineral
Elevation	norb_Cluster164	0.0242	-0.0152	0.4068	0.3611	0.0105	0.217888	Mineral
Elevation	norb_Cluster165	0.0092	-0.0044	0.1213	0.0537	0.2034	0.6092107	Mineral
Elevation	norb_Cluster166	0.0046	-0.0024	0.1343	0.0677	0.1791	0.5963517	Mineral
Elevation	norb_Cluster169	0.0309	-0.0172	0.3155	0.2629	0.0293	0.3618384	Mineral
Elevation	norb_Cluster17	0.0035	-0.0021	0.1892	0.1269	0.1051	0.4845739	Mineral
Elevation	norb_Cluster170	0.0299	-0.0146	0.1997	0.1381	0.095	0.4845739	Mineral
Elevation	norb_Cluster171	0.0089	-0.0058	0.1896	0.1273	0.1047	0.4845739	Mineral
Elevation	norb_Cluster172	0.0179	-0.0098	0.2165	0.1562	0.0805	0.4645405	Mineral
Elevation	norb_Cluster175	0.0595	-0.0331	0.2692	0.213	0.0475	0.4247495	Mineral
Elevation	norb_Cluster176	0.0392	-0.0246	0.3559	0.3064	0.0189	0.2933662	Mineral
Elevation	norb_Cluster177	0.0361	-0.0166	0.1866	0.124	0.1078	0.4893803	Mineral
Elevation	norb_Cluster179	0.003	0.0025	0.2478	0.19	0.059	0.4356571	Mineral
Elevation	norb_Cluster18	0.0047	-0.0019	0.1231	0.0556	0.1999	0.6046772	Mineral
Elevation	norb_Cluster180	0.0133	0.0018	0.0273	-0.0475	0.5561	0.8306793	Mineral

Elevation	norb_Cluster181	0.0003	-1.00E-04	0.0031	-0.0736	0.8434	0.9587544	Mineral
Elevation	norb_Cluster182	0.0018	-0.0013	0.1876	0.1251	0.1068	0.4883873	Mineral
Elevation	norb_Cluster184	0.0175	-0.0075	0.1897	0.1274	0.1046	0.4845739	Mineral
Elevation	norb_Cluster186	0.0011	-0.0005	0.0296	-0.0451	0.5399	0.827899	Mineral
Elevation	norb_Cluster19	0.0058	-0.002	0.1257	0.0585	0.1947	0.5963517	Mineral
Elevation	norb_Cluster192	0.0221	-0.013	0.3173	0.2648	0.0288	0.3618384	Mineral
Elevation	norb_Cluster193	0.0022	-0.0014	0.1394	0.0732	0.1704	0.5963517	Mineral
Elevation	norb_Cluster197	0.0398	-0.0121	0.0177	-0.0579	0.6364	0.8580172	Mineral
Elevation	norb_Cluster2	0.0032	-0.0025	0.1932	0.1311	0.1012	0.4845739	Mineral
Elevation	norb_Cluster20	0.0665	-0.0211	0.1726	0.1089	0.1236	0.5196658	Mineral
Elevation	norb_Cluster208	0.0015	-0.001	0.0909	0.021	0.2748	0.6727603	Mineral
Elevation	norb_Cluster21	0.0542	-0.0146	0.1088	0.0403	0.2298	0.6314975	Mineral
Elevation	norb_Cluster211	0.0141	-0.0009	0.0011	-0.0757	0.9056	0.9700106	Mineral
Elevation	norb_Cluster212	0.0252	-0.0134	0.1258	0.0586	0.1945	0.5963517	Mineral
Elevation	norb_Cluster214	0.0163	-0.0019	0.0061	-0.0704	0.7825	0.9456687	Mineral
Elevation	norb_Cluster217	0.0186	-0.0075	0.0695	-0.0021	0.3423	0.7277367	Mineral
Elevation	norb_Cluster219	0.0108	-0.0045	0.0528	-0.0201	0.4101	0.7519237	Mineral
Elevation	norb_Cluster22	0.0028	0.0012	0.0461	-0.0273	0.4424	0.7835229	Mineral
Elevation	norb_Cluster220	0.0248	-0.0084	0.0367	-0.0374	0.4939	0.8147683	Mineral
Elevation	norb_Cluster221	0.0248	-0.0078	0.0275	-0.0473	0.5547	0.8306675	Mineral
Elevation	norb_Cluster222	0.0104	-0.0033	0.0207	-0.0547	0.6093	0.8580172	Mineral
Elevation	norb_Cluster224	0.0238	-0.0117	0.0946	0.0249	0.2649	0.6678883	Mineral
Elevation	norb_Cluster225	0.0095	-0.0011	0.0084	-0.0678	0.7448	0.9265134	Mineral
Elevation	norb_Cluster226	0.0049	-0.0015	0.0392	-0.0347	0.4795	0.8083032	Mineral
Elevation	norb_Cluster228	0.0009	-0.0009	0.0661	-0.0058	0.355	0.7277367	Mineral
Elevation	norb_Cluster229	0.004	-0.0013	0.0551	-0.0176	0.3997	0.7519237	Mineral
Elevation	norb_Cluster230	0.0019	-0.0004	0.01	-0.0662	0.7232	0.9133673	Mineral
Elevation	norb_Cluster231	0.0061	0.0024	0.0573	-0.0153	0.3904	0.7519237	Mineral
Elevation	norb_Cluster232	0.0044	0.0012	0.0364	-0.0377	0.4958	0.8147683	Mineral
Elevation	norb_Cluster234	0.017	-0.0095	0.2087	0.1478	0.0869	0.4812414	Mineral
Elevation	norb_Cluster235	0.0012	0.00E+00	0.0003	-0.0766	0.9507	0.9767256	Mineral
Elevation	norb_Cluster238	0.0135	-0.0024	0.0145	-0.0613	0.6686	0.8804912	Mineral
Elevation	norb_Cluster239	0.0109	-0.0047	0.0673	-0.0045	0.3505	0.7277367	Mineral
Elevation	norb_Cluster24	0.3014	-0.0901	0.178	0.1147	0.1173	0.5087492	Mineral
Elevation	norb_Cluster244	0.0015	0.0006	0.0533	-0.0195	0.4077	0.7519237	Mineral
Elevation	norb_Cluster247	0.006	0.004	0.4704	0.4297	0.0048	0.1336576	Mineral
Elevation	norb_Cluster25	0.1263	-0.0444	0.1722	0.1086	0.124	0.5196658	Mineral
Elevation	norb_Cluster250	0.0146	-0.0003	0.0004	-0.0765	0.944	0.9767256	Mineral
Elevation	norb_Cluster251	0.0064	-0.0011	0.0176	-0.058	0.6376	0.8580172	Mineral
Elevation	norb_Cluster26	0.1134	-0.0436	0.2208	0.1609	0.0772	0.4576249	Mineral
Elevation	norb_Cluster28	0.0355	-0.0089	0.0929	0.0232	0.2692	0.6684333	Mineral
Elevation	norb_Cluster29	0.2454	-0.0693	0.3739	0.3257	0.0154	0.2715642	Mineral
Elevation	norb_Cluster31	0.0632	-0.014	0.0817	0.0111	0.3016	0.7081466	Mineral
Elevation	norb_Cluster32	0.1401	-0.0478	0.1341	0.0675	0.1795	0.5963517	Mineral
Elevation	norb_Cluster33	0.434	-0.1378	0.2696	0.2134	0.0473	0.4247495	Mineral
Elevation	norb_Cluster35	0.0746	-0.0249	0.1815	0.1186	0.1133	0.5025837	Mineral
Elevation	norb_Cluster36	1.1002	-0.4616	0.2708	0.2147	0.0468	0.4247495	Mineral
Elevation	norb_Cluster37	0.7173	-0.2782	0.3482	0.2981	0.0206	0.3072427	Mineral
Elevation	norb_Cluster38	0.0138	-0.002	0.0361	-0.038	0.4975	0.8152794	Mineral
Elevation	norb_Cluster39	0.0465	-0.0118	0.1056	0.0368	0.2373	0.6373128	Mineral
Elevation	norb_Cluster40	0.2213	-0.0576	0.1186	0.0508	0.2087	0.609693	Mineral
Elevation	norb_Cluster42	0.2825	-0.1123	0.2509	0.1933	0.0572	0.4356571	Mineral
Elevation	norb_Cluster44	0.0249	0.0008	0.001	-0.0758	0.9098	0.9700106	Mineral
Elevation	norb_Cluster46	0.2575	-0.0333	0.0226	-0.0526	0.5929	0.8580172	Mineral
Elevation	norb_Cluster47	0.6453	-0.1994	0.1849	0.1222	0.1096	0.4901939	Mineral

Elevation	norb_Cluster5	0.0115	-0.0025	0.0703	-0.0012	0.3395	0.7277367	Mineral
Elevation	norb_Cluster51	0.0122	-0.0068	0.1634	0.099	0.1351	0.5352263	Mineral
Elevation	norb_Cluster52	0.2809	-0.0781	0.1207	0.0531	0.2045	0.6092508	Mineral
Elevation	norb_Cluster56	0.0178	-0.0022	0.0235	-0.0516	0.5853	0.8526487	Mineral
Elevation	norb_Cluster57	0.004	-0.0009	0.0558	-0.0168	0.3964	0.7519237	Mineral
Elevation	norb_Cluster59	0.0322	0.0084	0.0527	-0.0201	0.4103	0.7519237	Mineral
Elevation	norb_Cluster6	0.6934	-0.2936	0.314	0.2612	0.0298	0.3618384	Mineral
Elevation	norb_Cluster60	0.345	-0.1278	0.1087	0.0401	0.2301	0.6314975	Mineral
Elevation	norb_Cluster61	0.5486	-0.2235	0.2978	0.2438	0.0354	0.3831179	Mineral
Elevation	norb_Cluster62	0.003	-0.0001	0.0007	-0.0761	0.9234	0.9705002	Mineral
Elevation	norb_Cluster64	0.0407	-0.0036	0.02	-0.0553	0.6148	0.8580172	Mineral
Elevation	norb_Cluster66	0.0016	0.0001	0.0015	-0.0753	0.8914	0.9698259	Mineral
Elevation	norb_Cluster67	0.0279	0.0044	0.0372	-0.0369	0.4911	0.8147683	Mineral
Elevation	norb_Cluster68	0.003	-0.0014	0.1141	0.0459	0.2183	0.6244214	Mineral
Elevation	norb_Cluster69	0.0032	0.0003	0.0032	-0.0734	0.8404	0.9587544	Mineral
Elevation	norb_Cluster7	0.0786	-0.024	0.1306	0.0637	0.1857	0.5963517	Mineral
Elevation	norb_Cluster70	0.1262	-0.0125	0.025	-0.0501	0.5739	0.8486922	Mineral
Elevation	norb_Cluster71	0.1512	-0.0314	0.1336	0.0669	0.1804	0.5963517	Mineral
Elevation	norb_Cluster74	0.0387	-0.0057	0.0544	-0.0184	0.4029	0.7519237	Mineral
Elevation	norb_Cluster75	0.1531	-0.0281	0.1378	0.0714	0.1732	0.5963517	Mineral
Elevation	norb_Cluster76	0.1215	-0.0379	0.0595	-0.0129	0.3811	0.7519237	Mineral
Elevation	norb_Cluster77	0.0492	-0.0005	0.0003	-0.0766	0.9488	0.9767256	Mineral
Elevation	norb_Cluster8	0.0206	-0.006	0.105	0.0361	0.2387	0.6373128	Mineral
Elevation	norb_Cluster80	0.0021	-0.0013	0.126	0.0588	0.1941	0.5963517	Mineral
Elevation	norb_Cluster81	0.0013	-0.0007	0.085	0.0146	0.2917	0.7024502	Mineral
Elevation	norb_Cluster83	0.011	-0.0072	0.1501	0.0848	0.1536	0.566325	Mineral
Elevation	norb_Cluster84	0.0093	0.0001	0.0001	-0.0768	0.9667	0.9850655	Mineral
Elevation	norb_Cluster85	0.0025	-0.001	0.0571	-0.0154	0.3909	0.7519237	Mineral
Elevation	norb_Cluster86	0.041	-0.015	0.0507	-0.0223	0.4198	0.7597299	Mineral
Elevation	norb_Cluster88	0.0019	-0.001	0.0831	0.0126	0.2974	0.707063	Mineral
Elevation	norb_Cluster90	0.0013	-0.0005	0.0184	-0.0571	0.6298	0.8580172	Mineral
Elevation	norb_Cluster91	0.7571	-0.3596	0.3291	0.2775	0.0254	0.3478932	Mineral
Elevation	norb_Cluster92	0.0021	0.0009	0.0359	-0.0383	0.499	0.8155962	Mineral
Elevation	norb_Cluster93	0.0028	-0.0012	0.0452	-0.0282	0.4467	0.7866982	Mineral
Elevation	norb_Cluster94	0.002	0.0011	0.1105	0.0421	0.226	0.6314975	Mineral
Elevation	norb_Cluster96	0.0006	1.00E-04	0.0032	-0.0735	0.8417	0.9587544	Mineral
Elevation	norb_Cluster97	0.0162	0.0087	0.2623	0.2055	0.051	0.4293393	Mineral
Elevation	norb_Cluster98	0.0176	-0.0068	0.0976	0.0282	0.2569	0.6653333	Mineral
Elevation	nosz_Cluster0	0.0057	0.0012	0.0343	-0.0399	0.5085	0.8189687	Mineral
Elevation	nosz_Cluster10	0.0128	-0.0039	0.091	0.0211	0.2744	0.6727603	Mineral
Elevation	nosz_Cluster102	0.0057	0.0014	0.0892	0.0191	0.2796	0.6804013	Mineral
Elevation	nosz_Cluster103	0.0496	-0.0197	0.2728	0.2168	0.0458	0.4247495	Mineral
Elevation	nosz_Cluster105	0.0016	0.0005	0.0344	-0.0399	0.5083	0.8189687	Mineral
Elevation	nosz_Cluster106	0.0088	-0.0055	0.2968	0.2427	0.0357	0.3831179	Mineral
Elevation	nosz_Cluster107	0.0003	-1.00E-04	0.0031	-0.0736	0.8434	0.9587544	Mineral
Elevation	nosz_Cluster108	0.0043	-0.0016	0.0641	-0.0079	0.3626	0.7277367	Mineral
Elevation	nosz_Cluster109	0.0416	-0.0055	0.0644	-0.0076	0.3615	0.7277367	Mineral
Elevation	nosz_Cluster11	0.0104	0.0026	0.0701	-0.0014	0.3402	0.7277367	Mineral
Elevation	nosz_Cluster110	0.0004	-0.0003	0.0321	-0.0423	0.5227	0.8204359	Mineral
Elevation	nosz_Cluster111	0.0078	-0.0033	0.1918	0.1296	0.1025	0.4845739	Mineral
Elevation	nosz_Cluster116	0.0074	0.0003	0.0028	-0.0739	0.8513	0.9603863	Mineral
Elevation	nosz_Cluster118	0.0064	-0.0009	0.0112	-0.0649	0.7075	0.909468	Mineral
Elevation	nosz_Cluster119	0.0019	-0.0016	0.2357	0.1769	0.0666	0.450158	Mineral
Elevation	nosz_Cluster120	0.0083	-0.0016	0.0321	-0.0423	0.5229	0.8204359	Mineral
Elevation	nosz_Cluster123	0.0136	-0.0057	0.1643	0.1	0.1339	0.5352263	Mineral

Elevation	nosz_Cluster128	0.005	-0.002	0.0886	0.0185	0.2813	0.6804013	Mineral
Elevation	nosz_Cluster129	0.05	-0.0191	0.5727	0.5398	0.0011	0.0488745	Mineral
Elevation	nosz_Cluster13	0.0124	0.002	0.0255	-0.0495	0.5698	0.8446831	Mineral
Elevation	nosz_Cluster130	0.1138	-0.0424	0.0352	-0.039	0.5033	0.818053	Mineral
Elevation	nosz_Cluster132	0.004	-0.0012	0.0737	0.0024	0.3277	0.7214545	Mineral
Elevation	nosz_Cluster14	0.0149	0.00E+00	0.00E+00	-0.0769	0.9966	0.9995238	Mineral
Elevation	nosz_Cluster16	0.008	0.00E+00	0.00E+00	-0.0769	0.9984	0.9995238	Mineral
Elevation	nosz_Cluster17	0.0427	-0.0133	0.1598	0.0951	0.1399	0.5502539	Mineral
Elevation	nosz_Cluster18	0.0184	0.0015	0.0048	-0.0718	0.8062	0.9559836	Mineral
Elevation	nosz_Cluster20	0.0117	-0.0041	0.0704	-0.0011	0.3393	0.7277367	Mineral
Elevation	nosz_Cluster21	0.0117	-0.0058	0.1583	0.0935	0.1419	0.5512015	Mineral
Elevation	nosz_Cluster22	0.0325	-0.005	0.0116	-0.0645	0.7028	0.9073623	Mineral
Elevation	nosz_Cluster23	0.019	-0.0075	0.1264	0.0593	0.1933	0.5963517	Mineral
Elevation	nosz_Cluster24	0.0019	0.0012	0.1197	0.052	0.2065	0.6092508	Mineral
Elevation	nosz_Cluster25	0.0026	0.0007	0.0283	-0.0465	0.5491	0.8285298	Mineral
Elevation	nosz_Cluster26	0.0068	-0.002	0.0411	-0.0327	0.4687	0.8078339	Mineral
Elevation	nosz_Cluster27	0.007	0.0002	0.0008	-0.076	0.9184	0.9705002	Mineral
Elevation	nosz_Cluster28	0.0358	-0.0056	0.0297	-0.0449	0.5389	0.827899	Mineral
Elevation	nosz_Cluster30	0.0341	-0.0006	0.0005	-0.0764	0.9379	0.9756996	Mineral
Elevation	nosz_Cluster32	0.016	0.0019	0.0156	-0.0601	0.6572	0.8753179	Mineral
Elevation	nosz_Cluster33	0.0187	-0.0045	0.0429	-0.0307	0.4589	0.7962739	Mineral
Elevation	nosz_Cluster34	0.0387	-0.0179	0.1276	0.0605	0.1911	0.5963517	Mineral
Elevation	nosz_Cluster35	0.0097	-0.0002	0.0006	-0.0763	0.9328	0.9740392	Mineral
Elevation	nosz_Cluster36	0.0213	-0.0055	0.0717	0.0002	0.3348	0.7277367	Mineral
Elevation	nosz_Cluster37	0.0077	0.0012	0.0189	-0.0565	0.6247	0.8580172	Mineral
Elevation	nosz_Cluster38	0.0144	-0.0024	0.0146	-0.0612	0.6681	0.8804912	Mineral
Elevation	nosz_Cluster39	0.0653	-0.0016	0.0009	-0.0759	0.9145	0.9700106	Mineral
Elevation	nosz_Cluster40	0.0262	0.0046	0.0289	-0.0458	0.5445	0.827899	Mineral
Elevation	nosz_Cluster41	0.0258	0.0021	0.0063	-0.0701	0.778	0.9456687	Mineral
Elevation	nosz_Cluster42	0.03	-0.0058	0.0344	-0.0399	0.508	0.8189687	Mineral
Elevation	nosz_Cluster43	0.001	-0.0007	0.0671	-0.0046	0.351	0.7277367	Mineral
Elevation	nosz_Cluster44	0.0068	-0.0021	0.0668	-0.005	0.3524	0.7277367	Mineral
Elevation	nosz_Cluster45	0.0056	-0.0006	0.0064	-0.0701	0.7774	0.9456687	Mineral
Elevation	nosz_Cluster46	0.0082	0.0003	0.0023	-0.0745	0.8664	0.9628369	Mineral
Elevation	nosz_Cluster47	0.0012	-0.0006	0.0767	0.0057	0.3176	0.7214545	Mineral
Elevation	nosz_Cluster48	0.0028	-0.0014	0.0726	0.0013	0.3313	0.7259547	Mineral
Elevation	nosz_Cluster49	0.0012	0.0007	0.0762	0.0051	0.3194	0.7214545	Mineral
Elevation	nosz_Cluster5	0.004	-0.0009	0.0201	-0.0552	0.614	0.8580172	Mineral
Elevation	nosz_Cluster51	0.003	-0.0006	0.0183	-0.0572	0.6306	0.8580172	Mineral
Elevation	nosz_Cluster53	0.0012	0.0007	0.0541	-0.0186	0.404	0.7519237	Mineral
Elevation	nosz_Cluster54	0.0037	-0.0023	0.1541	0.089	0.1478	0.5617054	Mineral
Elevation	nosz_Cluster57	0.2232	-0.0258	0.0184	-0.0571	0.6293	0.8580172	Mineral
Elevation	nosz_Cluster58	0.0053	-0.0039	0.2364	0.1777	0.0661	0.450158	Mineral
Elevation	nosz_Cluster59	0.0008	-0.0008	0.0661	-0.0058	0.355	0.7277367	Mineral
Elevation	nosz_Cluster6	0.0034	0.00E+00	0.00E+00	-0.0769	0.9977	0.9995238	Mineral
Elevation	nosz_Cluster60	0.0099	0.0017	0.02	-0.0554	0.6152	0.8580172	Mineral
Elevation	nosz_Cluster61	0.0178	-0.0051	0.1925	0.1304	0.1019	0.4845739	Mineral
Elevation	nosz_Cluster62	0.0493	-0.0055	0.0143	-0.0616	0.6715	0.8824016	Mineral
Elevation	nosz_Cluster64	0.0023	0.002	0.1055	0.0367	0.2376	0.6373128	Mineral
Elevation	nosz_Cluster65	0.0083	-0.0003	0.0008	-0.0761	0.9214	0.9705002	Mineral
Elevation	nosz_Cluster66	0.0428	-0.0084	0.0364	-0.0377	0.4958	0.8147683	Mineral
Elevation	nosz_Cluster67	0.0669	-0.0052	0.0057	-0.0708	0.7886	0.9456687	Mineral
Elevation	nosz_Cluster69	0.008	-0.0043	0.2522	0.1947	0.0564	0.4356571	Mineral
Elevation	nosz_Cluster70	0.0655	-0.0154	0.0212	-0.0541	0.6045	0.8580172	Mineral
Elevation	nosz_Cluster71	0.0101	-0.0017	0.0135	-0.0624	0.6797	0.8875059	Mineral

Elevation	nosz_Cluster72	0.066	-0.0121	0.0398	-0.034	0.4757	0.8078339	Mineral
Elevation	nosz_Cluster73	0.0031	-0.001	0.0396	-0.0342	0.4768	0.8078339	Mineral
Elevation	nosz_Cluster74	0.0017	-0.0016	0.2526	0.1951	0.0562	0.4356571	Mineral
Elevation	nosz_Cluster75	0.132	-0.0417	0.0207	-0.0546	0.6091	0.8580172	Mineral
Elevation	nosz_Cluster77	0.0002	-0.0002	0.0321	-0.0423	0.5227	0.8204359	Mineral
Elevation	nosz_Cluster78	0.0054	-0.0028	0.2203	0.1603	0.0776	0.4576249	Mineral
Elevation	nosz_Cluster79	0.0401	0.0029	0.0086	-0.0677	0.7428	0.9264867	Mineral
Elevation	nosz_Cluster8	0.0106	-0.0012	0.021	-0.0543	0.6064	0.8580172	Mineral
Elevation	nosz_Cluster80	0.0039	-0.0017	0.1031	0.0341	0.2432	0.6463547	Mineral
Elevation	nosz_Cluster81	0.0099	-0.0008	0.0026	-0.0742	0.8581	0.9628369	Mineral
Elevation	nosz_Cluster82	0.0023	0.0038	0.2976	0.2435	0.0354	0.3831179	Mineral
Elevation	nosz_Cluster83	0.0014	-0.0014	0.1262	0.059	0.1937	0.5963517	Mineral
Elevation	nosz_Cluster85	0.0027	0.0027	0.2543	0.1969	0.0553	0.4356571	Mineral
Elevation	nosz_Cluster86	0.0038	0.0023	0.1731	0.1095	0.1229	0.5196658	Mineral
Elevation	nosz_Cluster87	0.0034	0.0003	0.0037	-0.0729	0.8294	0.9587544	Mineral
Elevation	nosz_Cluster88	0.0246	-0.0084	0.2997	0.2458	0.0347	0.3831179	Mineral
Elevation	nosz_Cluster89	0.0607	-0.0094	0.0284	-0.0463	0.5481	0.8285298	Mineral
Elevation	nosz_Cluster9	0.0034	-0.0003	0.0035	-0.0732	0.8347	0.9587544	Mineral
Elevation	nosz_Cluster90	0.0139	-0.0011	0.0127	-0.0632	0.6891	0.8935876	Mineral
Elevation	nosz_Cluster91	0.0232	-0.0037	0.0204	-0.055	0.6118	0.8580172	Mineral
Elevation	nosz_Cluster93	0.0488	-0.0165	0.1529	0.0878	0.1495	0.5617054	Mineral
Elevation	nosz_Cluster95	0.0094	-0.0024	0.0745	0.0033	0.325	0.7214545	Mineral
Elevation	nosz_Cluster96	0.0064	-0.0008	0.0191	-0.0564	0.6237	0.8580172	Mineral
Elevation	nosz_Cluster97	0.0026	-0.0003	0.0039	-0.0727	0.8244	0.9587544	Mineral
Elevation	nosz_Cluster98	0.0035	0.0052	0.256	0.1988	0.0543	0.4356571	Mineral
Elevation	nosz_Cluster99	0.0061	-1.00E-04	0.0002	-0.0767	0.9637	0.9836687	Mineral
Elevation	nrfa_Cluster10	0.0978	-0.0164	0.0245	-0.0506	0.5778	0.8522328	Mineral
Elevation	nrfa_Cluster100	0.1249	-0.0754	0.2254	0.1658	0.0738	0.4533497	Mineral
Elevation	nrfa_Cluster104	0.1478	-0.019	0.0277	-0.0471	0.5535	0.8306675	Mineral
Elevation	nrfa_Cluster105	0.006	-0.0016	0.037	-0.0371	0.4925	0.8147683	Mineral
Elevation	nrfa_Cluster109	0.2753	0.0158	0.0062	-0.0703	0.7805	0.9456687	Mineral
Elevation	nrfa_Cluster116	0.3208	-0.0099	0.0033	-0.0734	0.8385	0.9587544	Mineral
Elevation	nrfa_Cluster118	0.2298	-0.1506	0.2262	0.1667	0.0731	0.4533497	Mineral
Elevation	nrfa_Cluster125	0.1081	-0.0735	0.2169	0.1566	0.0802	0.4645405	Mineral
Elevation	nrfa_Cluster154	0.0837	-0.0437	0.1795	0.1164	0.1155	0.5049196	Mineral
Elevation	nrfa_Cluster164	0.0044	-0.0011	0.0367	-0.0374	0.4941	0.8147683	Mineral
Elevation	nrfa_Cluster172	0.0036	-0.0017	0.0742	0.003	0.3261	0.7214545	Mineral
Elevation	nrfa_Cluster175	0.0053	-0.005	0.2337	0.1747	0.0679	0.450158	Mineral
Elevation	nrfa_Cluster20	0.045	-0.0008	0.0003	-0.0766	0.9511	0.9767256	Mineral
Elevation	nrfa_Cluster26	0.1433	-0.0915	0.2404	0.182	0.0635	0.450158	Mineral
Elevation	nrfa_Cluster29	0.0059	-0.0024	0.0778	0.0069	0.3139	0.7214545	Mineral
Elevation	nrfa_Cluster34	0.0642	-0.0073	0.0114	-0.0646	0.7046	0.9076527	Mineral
Elevation	nrfa_Cluster38	0.0033	-0.003	0.2237	0.164	0.075	0.4549639	Mineral
Elevation	nrfa_Cluster61	0.0047	-0.0002	0.001	-0.0758	0.9101	0.9700106	Mineral
Elevation	nrfa_Cluster62	0.0589	-0.0403	0.2135	0.153	0.0829	0.4705706	Mineral
Elevation	nrfa_Cluster66	0.1282	-0.0164	0.038	-0.036	0.4863	0.8147683	Mineral
Elevation	nrfa_Cluster69	0.0035	-0.001	0.0331	-0.0413	0.5165	0.8204359	Mineral
Elevation	nrfa_Cluster71	0.0322	-0.0153	0.1257	0.0584	0.1948	0.5963517	Mineral
Elevation	nrfa_Cluster72	0.0969	-0.0398	0.1343	0.0677	0.1791	0.5963517	Mineral
Elevation	nrfa_Cluster73	0.0042	-0.0026	0.12	0.0523	0.2058	0.6092508	Mineral
Elevation	nrfa_Cluster74	0.1692	-0.0964	0.2501	0.1924	0.0577	0.4356571	Mineral
Elevation	nrfa_Cluster76	0.0922	-0.0572	0.2356	0.1768	0.0666	0.450158	Mineral
Elevation	nrfa_Cluster79	0.0138	-0.0063	0.0441	-0.0295	0.4527	0.7896247	Mineral
Elevation	nrfa_Cluster87	0.0207	-0.0079	0.0489	-0.0243	0.4285	0.7684715	Mineral
Elevation	nrfa_Cluster89	0.0023	-0.0015	0.1108	0.0424	0.2254	0.6314975	Mineral

Elevation	nrfa_Cluster92	0.0041	-0.0023	0.2003	0.1388	0.0944	0.4845739	Mineral
Elevation	nrfa_Cluster93	0.0581	-0.0166	0.0339	-0.0405	0.5115	0.8189687	Mineral
Elevation	nrfa_Cluster94	0.0003	-0.0003	0.0661	-0.0058	0.355	0.7277367	Mineral
Elevation	nrfa_Cluster97	0.0225	-0.0102	0.2303	0.1711	0.0702	0.450158	Mineral
Elevation	nrfa_Cluster99	0.0184	-0.0093	0.2229	0.1631	0.0756	0.4549639	Mineral
DNA	amoA_A_Cluster26	0.0009	-0.0014	0.4277	0.2846	0.1589	0.5737231	Organic
DNA	amoA_A_Cluster45	0	0	#VALUE!	#VALUE!	#VALUE!	NA	Organic
DNA	amoA_B_Cluster0	0.0665	-0.0264	0.416	0.27	0.1667	0.5737231	Organic
DNA	amoA_B_Cluster1	0.0089	-0.0129	0.4836	0.3545	0.125	0.5737231	Organic
DNA	amoA_B_Cluster10	0.013	-0.0112	0.2658	0.0822	0.2952	0.5830102	Organic
DNA	amoA_B_Cluster11	0.0103	-0.0111	0.4022	0.2527	0.1763	0.5737231	Organic
DNA	amoA_B_Cluster12	0.0501	-0.0212	0.2709	0.0886	0.2898	0.5821367	Organic
DNA	amoA_B_Cluster13	0.2409	-0.2523	0.4359	0.2949	0.1535	0.5737231	Organic
DNA	amoA_B_Cluster14	0.0117	-0.0185	0.4661	0.3327	0.135	0.5737231	Organic
DNA	amoA_B_Cluster15	0.0082	-0.0101	0.4347	0.2933	0.1543	0.5737231	Organic
DNA	amoA_B_Cluster2	0.0666	-0.0874	0.5111	0.3889	0.1103	0.5737231	Organic
DNA	amoA_B_Cluster20	0.0007	-0.0012	0.4277	0.2846	0.1589	0.5737231	Organic
DNA	amoA_B_Cluster23	0.0211	-0.0146	0.3911	0.2388	0.1842	0.5737231	Organic
DNA	amoA_B_Cluster3	0.0028	-0.0021	0.2143	0.0179	0.3552	0.6227278	Organic
DNA	amoA_B_Cluster4	0.0017	-0.0027	0.4277	0.2846	0.1589	0.5737231	Organic
DNA	amoA_B_Cluster5	0.0667	-0.0394	0.416	0.27	0.1667	0.5737231	Organic
DNA	amoA_B_Cluster6	0.0116	-0.0072	0.4518	0.3147	0.1436	0.5737231	Organic
DNA	amoA_B_Cluster7	0.0047	0.0029	0.2765	0.0957	0.2839	0.5821367	Organic
DNA	amoA_B_Cluster8	0.0054	-0.0053	0.5192	0.399	0.1062	0.5737231	Organic
DNA	amoA_B_Cluster9	0.019	-0.0236	0.5188	0.3985	0.1064	0.5737231	Organic
DNA	nifh_Cluster0	0.0368	0.0089	0.5749	0.4686	0.0806	0.5737231	Organic
DNA	nifh_Cluster10	0.0093	0.0074	0.1622	-0.0472	0.4285	0.6670148	Organic
DNA	nifh_Cluster100	0.0019	0.0025	0.2893	0.1117	0.271	0.5737231	Organic
DNA	nifh_Cluster103	0.0259	-0.0162	0.5033	0.3791	0.1144	0.5737231	Organic
DNA	nifh_Cluster1034	0.0074	-0.0031	0.2024	0.003	0.3707	0.627386	Organic
DNA	nifh_Cluster105	0.0047	0.00E+00	1.00E-04	-0.2499	0.9854	0.9872826	Organic
DNA	nifh_Cluster108	0.0098	-0.0047	0.1598	-0.0502	0.4323	0.6689043	Organic
DNA	nifh_Cluster1099	0.1117	0.0395	0.5896	0.487	0.0746	0.5737231	Organic
DNA	nifh_Cluster11	0.001	0.0013	0.2893	0.1117	0.271	0.5737231	Organic
DNA	nifh_Cluster1112	0.0008	-0.0009	0.2408	0.051	0.323	0.5989858	Organic
DNA	nifh_Cluster112	0.0145	0.0002	0.001	-0.2488	0.9533	0.9771021	Organic
DNA	nifh_Cluster113	0.021	0.011	0.4373	0.2966	0.1526	0.5737231	Organic
DNA	nifh_Cluster114	0.0403	-0.007	0.3039	0.1299	0.2568	0.5737231	Organic
DNA	nifh_Cluster1141	0.0138	-0.0119	0.4308	0.2884	0.1569	0.5737231	Organic
DNA	nifh_Cluster115	0.0088	0.0061	0.6574	0.5717	0.0503	0.5737231	Organic
DNA	nifh_Cluster116	0.0503	-0.0112	0.0786	-0.1518	0.5906	0.7617846	Organic
DNA	nifh_Cluster1163	0.0028	-0.0017	0.2692	0.0865	0.2916	0.5821367	Organic
DNA	nifh_Cluster1179	0.0016	-1.00E-04	0.001	-0.2488	0.9535	0.9771021	Organic
DNA	nifh_Cluster1182	0.0191	0.0032	0.184	-0.02	0.396	0.6472114	Organic
DNA	nifh_Cluster1183	0.0119	0.0015	0.0479	-0.1901	0.6768	0.8182394	Organic
DNA	nifh_Cluster1197	0.0113	-0.0006	0.0049	-0.2439	0.8954	0.9576604	Organic
DNA	nifh_Cluster1199	0.0041	-0.0027	0.5297	0.4121	0.101	0.5737231	Organic
DNA	nifh_Cluster120	0.0019	0.0025	0.2893	0.1117	0.271	0.5737231	Organic
DNA	nifh_Cluster1203	0	0	#VALUE!	#VALUE!	#VALUE!	NA	Organic
DNA	nifh_Cluster1206	0	0	#VALUE!	#VALUE!	#VALUE!	NA	Organic
DNA	nifh_Cluster1207	0.0064	-0.0034	0.1058	-0.1178	0.5294	0.7324644	Organic
DNA	nifh_Cluster1209	0.1228	0.0068	0.1255	-0.0931	0.4908	0.7110912	Organic
DNA	nifh_Cluster121	0.0027	0.0016	0.1152	-0.106	0.5104	0.7195314	Organic
DNA	nifh_Cluster1211	0.0026	0.0007	0.054	-0.1824	0.6576	0.811809	Organic
DNA	nifh_Cluster1213	0.0012	0.0011	0.1439	-0.0701	0.4583	0.6927624	Organic

DNA	nifh_Cluster122	0	0	#VALUE!	#VALUE!	#VALUE!	NA	Organic
DNA	nifh_Cluster123	0.0105	0.0138	0.2893	0.1117	0.271	0.5737231	Organic
DNA	nifh_Cluster1236	0.0046	-0.0017	0.0515	-0.1856	0.6653	0.8149108	Organic
DNA	nifh_Cluster1238	0.0012	0.0009	0.1676	-0.0405	0.4202	0.6580549	Organic
DNA	nifh_Cluster124	0.0066	0.007	0.4183	0.2729	0.1651	0.5737231	Organic
DNA	nifh_Cluster1261	0.0012	-0.0005	0.0473	-0.1909	0.679	0.8182394	Organic
DNA	nifh_Cluster1262	0.0028	-0.0015	0.0972	-0.1285	0.5475	0.7407953	Organic
DNA	nifh_Cluster1266	0.0148	0.0094	0.4525	0.3156	0.1432	0.5737231	Organic
DNA	nifh_Cluster1267	0.0996	0.0159	0.0996	-0.1255	0.5424	0.7407953	Organic
DNA	nifh_Cluster127	0.0009	-0.0015	0.4277	0.2846	0.1589	0.5737231	Organic
DNA	nifh_Cluster1278	0.0346	-0.0281	0.3763	0.2204	0.1953	0.5737231	Organic
DNA	nifh_Cluster128	0.0019	0.0025	0.2893	0.1117	0.271	0.5737231	Organic
DNA	nifh_Cluster1289	0.0036	0.002	0.0995	-0.1256	0.5425	0.7407953	Organic
DNA	nifh_Cluster129	0.0057	0.0075	0.2893	0.1117	0.271	0.5737231	Organic
DNA	nifh_Cluster1292	0.0036	0.0013	0.0784	-0.152	0.5909	0.7617846	Organic
DNA	nifh_Cluster130	0.0029	0.0038	0.2893	0.1117	0.271	0.5737231	Organic
DNA	nifh_Cluster1305	0.0047	0.0043	0.556	0.4451	0.0888	0.5737231	Organic
DNA	nifh_Cluster1306	0.0036	-0.0044	0.5737	0.4671	0.0811	0.5737231	Organic
DNA	nifh_Cluster131	0.001	0.0013	0.2893	0.1117	0.271	0.5737231	Organic
DNA	nifh_Cluster1319	0.0009	0.0011	0.2893	0.1117	0.271	0.5737231	Organic
DNA	nifh_Cluster132	0.0064	0.0077	0.4723	0.3404	0.1314	0.5737231	Organic
DNA	nifh_Cluster1320	0.0017	0.0003	0.0151	-0.2312	0.8169	0.9148251	Organic
DNA	nifh_Cluster1323	0.0027	0.0008	0.0712	-0.161	0.6093	0.774766	Organic
DNA	nifh_Cluster1324	0.0463	0.0146	0.2275	0.0344	0.3388	0.6109232	Organic
DNA	nifh_Cluster1336	0.0138	0.0048	0.6704	0.588	0.0463	0.5737231	Organic
DNA	nifh_Cluster1340	0.0073	-0.0037	0.2631	0.0789	0.298	0.583864	Organic
DNA	nifh_Cluster1345	0.0863	0.0236	0.1927	-0.0091	0.3838	0.6379711	Organic
DNA	nifh_Cluster1370	0.0018	-0.0007	0.0647	-0.1691	0.6265	0.7839346	Organic
DNA	nifh_Cluster1375	0.0019	-0.0014	0.2093	0.0116	0.3616	0.6227278	Organic
DNA	nifh_Cluster139	0.0049	0.0042	0.469	0.3362	0.1334	0.5737231	Organic
DNA	nifh_Cluster140	0.0023	-0.001	0.1368	-0.079	0.4705	0.6964051	Organic
DNA	nifh_Cluster141	0.035	-0.0442	0.4536	0.317	0.1425	0.5737231	Organic
DNA	nifh_Cluster148	0.0009	-0.0014	0.4277	0.2846	0.1589	0.5737231	Organic
DNA	nifh_Cluster1480	0.0019	0.0008	0.0727	-0.1592	0.6055	0.7723363	Organic
DNA	nifh_Cluster152	0.0593	0.004	0.0957	-0.1304	0.5508	0.7407953	Organic
DNA	nifh_Cluster156	0.1158	0.0279	0.2788	0.0985	0.2816	0.5821367	Organic
DNA	nifh_Cluster1562	0.1168	0.0416	0.3977	0.2471	0.1795	0.5737231	Organic
DNA	nifh_Cluster158	0.0028	0.0018	0.1822	-0.0223	0.3986	0.6494668	Organic
DNA	nifh_Cluster16	0.001	0.0013	0.2893	0.1117	0.271	0.5737231	Organic
DNA	nifh_Cluster166	0.0191	0.0246	0.314	0.1425	0.2475	0.5737231	Organic
DNA	nifh_Cluster205	0.0124	0.0087	0.2146	0.0183	0.3548	0.6227278	Organic
DNA	nifh_Cluster21	0.0018	-0.0029	0.4277	0.2846	0.1589	0.5737231	Organic
DNA	nifh_Cluster222	0.0038	0.005	0.2893	0.1117	0.271	0.5737231	Organic
DNA	nifh_Cluster225	0.0136	0.0037	0.207	0.0088	0.3646	0.6227866	Organic
DNA	nifh_Cluster230	0.0017	-0.0005	0.0371	-0.2037	0.7147	0.8381411	Organic
DNA	nifh_Cluster231	0.0029	0.0033	0.4571	0.3214	0.1404	0.5737231	Organic
DNA	nifh_Cluster236	0.0007	-0.0012	0.4277	0.2846	0.1589	0.5737231	Organic
DNA	nifh_Cluster237	0.0029	0.0003	0.0087	-0.2391	0.8601	0.9381137	Organic
DNA	nifh_Cluster243	0.0532	0.0083	0.0687	-0.1642	0.6159	0.774766	Organic
DNA	nifh_Cluster2432	0.0734	0.0255	0.546	0.4324	0.0934	0.5737231	Organic
DNA	nifh_Cluster2433	0.004	0.0039	0.4706	0.3383	0.1324	0.5737231	Organic
DNA	nifh_Cluster246	0.0028	0.0001	0.0014	-0.2482	0.9434	0.9771021	Organic
DNA	nifh_Cluster25	0.0544	0.0193	0.5671	0.4588	0.0839	0.5737231	Organic
DNA	nifh_Cluster256	0.0042	-0.0005	0.0106	-0.2368	0.8463	0.9357969	Organic
DNA	nifh_Cluster264	0.004	0.0036	0.5324	0.4155	0.0997	0.5737231	Organic

DNA	nifh_Cluster265	0.0823	0.0167	0.099	-0.1262	0.5436	0.7407953	Organic
DNA	nifh_Cluster267	0.0206	0.002	0.0115	-0.2357	0.84	0.9342807	Organic
DNA	nifh_Cluster268	0.0115	0.0051	0.1518	-0.0603	0.4452	0.6848524	Organic
DNA	nifh_Cluster269	0.0097	-0.0005	0.0189	-0.2263	0.7949	0.901761	Organic
DNA	nifh_Cluster272	0.0114	0.0053	0.3108	0.1386	0.2504	0.5737231	Organic
DNA	nifh_Cluster274	0.0076	0.01	0.2893	0.1117	0.271	0.5737231	Organic
DNA	nifh_Cluster275	0	0	#VALUE!	#VALUE!	#VALUE!	NA	Organic
DNA	nifh_Cluster278	0.013	0.0039	0.1192	-0.1011	0.5028	0.7195314	Organic
DNA	nifh_Cluster279	0.0067	0.0004	0.0314	-0.2108	0.7371	0.8586362	Organic
DNA	nifh_Cluster28	0.0027	0.001	0.1222	-0.0973	0.497	0.7141478	Organic
DNA	nifh_Cluster280	0.0024	-0.0036	0.7635	0.7044	0.0229	0.5737231	Organic
DNA	nifh_Cluster282	0.0052	0.0025	0.1728	-0.0341	0.4124	0.6580549	Organic
DNA	nifh_Cluster287	0	0	#VALUE!	#VALUE!	#VALUE!	NA	Organic
DNA	nifh_Cluster290	0.0147	0.0049	0.5582	0.4477	0.0878	0.5737231	Organic
DNA	nifh_Cluster297	0	0	#VALUE!	#VALUE!	#VALUE!	NA	Organic
DNA	nifh_Cluster299	0.0028	0.0036	0.2893	0.1117	0.271	0.5737231	Organic
DNA	nifh_Cluster303	0.0021	0.0019	0.1439	-0.0701	0.4583	0.6927624	Organic
DNA	nifh_Cluster304	0.002	0.002	0.4348	0.2935	0.1542	0.5737231	Organic
DNA	nifh_Cluster313	0.0038	0.0045	0.4161	0.2701	0.1666	0.5737231	Organic
DNA	nifh_Cluster314	0.001	0.0008	0.0924	-0.1345	0.5581	0.7407953	Organic
DNA	nifh_Cluster317	0.0027	-0.0004	0.0192	-0.226	0.7936	0.901761	Organic
DNA	nifh_Cluster32	0.0043	-0.0018	0.0981	-0.1274	0.5456	0.7407953	Organic
DNA	nifh_Cluster320	9.7369	-1.1206	0.2703	0.0878	0.2905	0.5821367	Organic
DNA	nifh_Cluster334	0.001	0.0013	0.2893	0.1117	0.271	0.5737231	Organic
DNA	nifh_Cluster335	0.0114	0.015	0.2893	0.1117	0.271	0.5737231	Organic
DNA	nifh_Cluster338	0.0053	-0.0019	0.4201	0.2752	0.1639	0.5737231	Organic
DNA	nifh_Cluster340	0.0143	0.0188	0.2893	0.1117	0.271	0.5737231	Organic
DNA	nifh_Cluster341	0.0029	0.0013	0.0685	-0.1643	0.6163	0.774766	Organic
DNA	nifh_Cluster35	0.0076	-0.0058	0.3982	0.2477	0.1791	0.5737231	Organic
DNA	nifh_Cluster351	0	0	#VALUE!	#VALUE!	#VALUE!	NA	Organic
DNA	nifh_Cluster3574	0.085	-0.049	0.9163	0.8953	0.0027	0.5737231	Organic
DNA	nifh_Cluster3691	0.0049	-0.0055	0.4015	0.2519	0.1767	0.5737231	Organic
DNA	nifh_Cluster37	0.0214	0.0108	0.2046	0.0057	0.3678	0.6245626	Organic
DNA	nifh_Cluster374	0	0	#VALUE!	#VALUE!	#VALUE!	NA	Organic
DNA	nifh_Cluster38	0.001	0.0008	0.0924	-0.1345	0.5581	0.7407953	Organic
DNA	nifh_Cluster382	0	0	#VALUE!	#VALUE!	#VALUE!	NA	Organic
DNA	nifh_Cluster384	0.001	0.0013	0.2893	0.1117	0.271	0.5737231	Organic
DNA	nifh_Cluster386	0.0019	0.0025	0.2893	0.1117	0.271	0.5737231	Organic
DNA	nifh_Cluster389	0.0031	0.0031	0.7977	0.7471	0.0165	0.5737231	Organic
DNA	nifh_Cluster394	0	0	#VALUE!	#VALUE!	#VALUE!	NA	Organic
DNA	nifh_Cluster40	0.0007	-0.0012	0.4277	0.2846	0.1589	0.5737231	Organic
DNA	nifh_Cluster43	0.0111	0.0018	0.0441	-0.1949	0.6897	0.8235579	Organic
DNA	nifh_Cluster434	0	0	#VALUE!	#VALUE!	#VALUE!	NA	Organic
DNA	nifh_Cluster440	0.0039	-0.0063	0.4277	0.2846	0.1589	0.5737231	Organic
DNA	nifh_Cluster462	0	0	#VALUE!	#VALUE!	#VALUE!	NA	Organic
DNA	nifh_Cluster470	0.0107	0.0049	0.3203	0.1503	0.2418	0.5737231	Organic
DNA	nifh_Cluster49	0.019	0.0212	0.3447	0.1809	0.2205	0.5737231	Organic
DNA	nifh_Cluster499	0.0019	0.0011	0.1285	-0.0893	0.4853	0.7069263	Organic
DNA	nifh_Cluster5	0.0239	-0.0071	0.4447	0.3059	0.148	0.5737231	Organic
DNA	nifh_Cluster506	0.0022	0.0018	0.3645	0.2057	0.2044	0.5737231	Organic
DNA	nifh_Cluster51	0.002	0.002	0.4353	0.2941	0.1539	0.5737231	Organic
DNA	nifh_Cluster52	0.0187	0.0136	0.2625	0.0782	0.2987	0.583864	Organic
DNA	nifh_Cluster525	0.0054	-0.0087	0.4277	0.2846	0.1589	0.5737231	Organic
DNA	nifh_Cluster531	0.058	0.014	0.3072	0.134	0.2538	0.5737231	Organic
DNA	nifh_Cluster539	0.003	0.0028	0.3006	0.1257	0.26	0.5737231	Organic

DNA	nifh_Cluster578	0.0202	-0.0086	0.2656	0.082	0.2954	0.5830102	Organic
DNA	nifh_Cluster58	0.6425	0.1362	0.2008	0.001	0.3728	0.6289276	Organic
DNA	nifh_Cluster60	0.0039	0.0031	0.433	0.2912	0.1554	0.5737231	Organic
DNA	nifh_Cluster61	0.0046	-0.0005	0.0084	-0.2395	0.8629	0.9381137	Organic
DNA	nifh_Cluster65	0.0104	0.0077	0.1924	-0.0095	0.3842	0.6379711	Organic
DNA	nifh_Cluster686	0	0	#VALUE!	#VALUE!	#VALUE!	NA	Organic
DNA	nifh_Cluster69	0.0085	0.0022	0.0474	-0.1907	0.6784	0.8182394	Organic
DNA	nifh_Cluster70	0.0029	0.0038	0.2893	0.1117	0.271	0.5737231	Organic
DNA	nifh_Cluster717	0.0046	0.0037	0.9042	0.8802	0.0036	0.5737231	Organic
DNA	nifh_Cluster725	0.0118	0.0022	0.0688	-0.1641	0.6157	0.774766	Organic
DNA	nifh_Cluster727	0.0042	-0.0044	0.2921	0.1151	0.2682	0.5737231	Organic
DNA	nifh_Cluster73	0.0038	0.0025	0.2843	0.1054	0.276	0.582031	Organic
DNA	nifh_Cluster74	0.0048	0.0063	0.2893	0.1117	0.271	0.5737231	Organic
DNA	nifh_Cluster748	0.0023	0.0005	0.0239	-0.2201	0.77	0.8818511	Organic
DNA	nifh_Cluster749	0.0111	0.0033	0.2063	0.0078	0.3656	0.6227866	Organic
DNA	nifh_Cluster755	0.006	-0.0096	0.4277	0.2846	0.1589	0.5737231	Organic
DNA	nifh_Cluster76	0.0019	1.00E-04	0.0004	-0.2495	0.9715	0.9771021	Organic
DNA	nifh_Cluster79	0.0027	0.0009	0.0402	-0.1998	0.7034	0.8304343	Organic
DNA	nifh_Cluster81	0.0099	-0.0047	0.0883	-0.1396	0.5674	0.7457001	Organic
DNA	nifh_Cluster82	0.0003	-0.0003	0.2408	0.051	0.323	0.5989858	Organic
DNA	nifh_Cluster83	0.0105	0.0077	0.2606	0.0757	0.3008	0.5848711	Organic
DNA	nifh_Cluster86	0.007	-0.0042	0.2728	0.091	0.2878	0.5821367	Organic
DNA	nifh_Cluster868	0.01	-0.0022	0.106	-0.1175	0.5289	0.7324644	Organic
DNA	nifh_Cluster87	0.001	0.0013	0.2893	0.1117	0.271	0.5737231	Organic
DNA	nifh_Cluster88	0	0	#VALUE!	#VALUE!	#VALUE!	NA	Organic
DNA	nifh_Cluster9	0.0083	0.0024	0.1495	-0.0632	0.449	0.6886212	Organic
DNA	nifh_Cluster92	0.001	0.0013	0.2893	0.1117	0.271	0.5737231	Organic
DNA	nifh_Cluster93	0.0038	0.0036	0.2691	0.0864	0.2916	0.5821367	Organic
DNA	nifh_Cluster94	0.0038	0.005	0.2893	0.1117	0.271	0.5737231	Organic
DNA	nifh_Cluster95	0.005	0.0045	0.2524	0.0655	0.3098	0.5979672	Organic
DNA	nifh_Cluster96	0.0104	0.0001	0.0004	-0.2495	0.9689	0.9771021	Organic
DNA	nifh_Cluster97	0.0317	0.0162	0.2146	0.0182	0.3549	0.6227278	Organic
DNA	nifh_Cluster98	0.0084	-0.0006	0.0072	-0.241	0.873	0.9414421	Organic
DNA	nifh_Cluster99	0.0028	0.0032	0.4519	0.3149	0.1435	0.5737231	Organic
DNA	nirk_Cluster0	4.2317	-1.0751	0.7226	0.6532	0.032	0.5737231	Organic
DNA	nirk_Cluster1	0.0715	-0.0858	0.4032	0.254	0.1755	0.5737231	Organic
DNA	nirk_Cluster10	0.3374	-0.0456	0.1383	-0.0771	0.4679	0.6964051	Organic
DNA	nirk_Cluster101	0.001	0.0013	0.2893	0.1117	0.271	0.5737231	Organic
DNA	nirk_Cluster102	0.0115	0.0012	0.0088	-0.239	0.8597	0.9381137	Organic
DNA	nirk_Cluster103	0.0388	-0.0376	0.4003	0.2504	0.1776	0.5737231	Organic
DNA	nirk_Cluster104	0.0009	-0.0014	0.4277	0.2846	0.1589	0.5737231	Organic
DNA	nirk_Cluster105	0.0379	-0.0235	0.3986	0.2482	0.1788	0.5737231	Organic
DNA	nirk_Cluster106	0.0248	-0.018	0.6073	0.5091	0.0677	0.5737231	Organic
DNA	nirk_Cluster107	0.0313	-0.0158	0.3952	0.2439	0.1813	0.5737231	Organic
DNA	nirk_Cluster108	0.0068	0.0082	0.4875	0.3594	0.1229	0.5737231	Organic
DNA	nirk_Cluster109	0.0007	-0.0012	0.4277	0.2846	0.1589	0.5737231	Organic
DNA	nirk_Cluster11	0.1133	0.0043	0.0218	-0.2227	0.7799	0.8886753	Organic
DNA	nirk_Cluster12	0.0661	-0.0186	0.1976	-0.003	0.3771	0.632111	Organic
DNA	nirk_Cluster13	0.0382	-0.0014	0.0082	-0.2398	0.8648	0.9381137	Organic
DNA	nirk_Cluster14	0.06	-0.0255	0.1762	-0.0298	0.4074	0.6580549	Organic
DNA	nirk_Cluster15	0.1402	-0.0917	0.3153	0.1441	0.2463	0.5737231	Organic
DNA	nirk_Cluster16	0.2004	-0.1208	0.3511	0.1889	0.2152	0.5737231	Organic
DNA	nirk_Cluster17	0.0882	0.02	0.2297	0.0371	0.3361	0.6104132	Organic
DNA	nirk_Cluster18	0.0885	0.0257	0.0963	-0.1296	0.5494	0.7407953	Organic
DNA	nirk_Cluster19	0.2332	-0.0098	0.0165	-0.2294	0.8084	0.9131557	Organic

DNA	nirk_Cluster2	0.2103	-0.0485	0.391	0.2387	0.1843	0.5737231	Organic
DNA	nirk_Cluster20	0.1247	0.0741	0.351	0.1887	0.2153	0.5737231	Organic
DNA	nirk_Cluster21	0.1085	0.0109	0.0457	-0.1929	0.6842	0.8200131	Organic
DNA	nirk_Cluster22	0.0844	-0.0189	0.3474	0.1842	0.2183	0.5737231	Organic
DNA	nirk_Cluster23	0.0533	0.0039	0.0638	-0.1703	0.6292	0.7853776	Organic
DNA	nirk_Cluster24	0.0405	-0.0182	0.17	-0.0375	0.4166	0.6580549	Organic
DNA	nirk_Cluster25	0.2941	-0.1636	0.3804	0.2255	0.1921	0.5737231	Organic
DNA	nirk_Cluster26	0.0877	-0.0114	0.0653	-0.1684	0.625	0.7838889	Organic
DNA	nirk_Cluster27	0.0878	-0.0315	0.3277	0.1596	0.2351	0.5737231	Organic
DNA	nirk_Cluster28	0.0375	0.0087	0.1602	-0.0498	0.4317	0.6689043	Organic
DNA	nirk_Cluster29	0.1289	0.0042	0.0091	-0.2386	0.8572	0.9381137	Organic
DNA	nirk_Cluster3	0.2014	-0.0909	0.5958	0.4948	0.0721	0.5737231	Organic
DNA	nirk_Cluster30	0.0164	-0.0121	0.2979	0.1224	0.2626	0.5737231	Organic
DNA	nirk_Cluster31	0.1697	0.0221	0.4754	0.3443	0.1296	0.5737231	Organic
DNA	nirk_Cluster32	0.0468	-0.0304	0.2144	0.018	0.3551	0.6227278	Organic
DNA	nirk_Cluster33	0.0203	-0.0104	0.2793	0.0991	0.2811	0.5821367	Organic
DNA	nirk_Cluster34	0.0301	0.0114	0.225	0.0312	0.3419	0.6144893	Organic
DNA	nirk_Cluster35	0.032	-0.0179	0.3037	0.1296	0.2571	0.5737231	Organic
DNA	nirk_Cluster36	0.0009	-0.0014	0.4277	0.2846	0.1589	0.5737231	Organic
DNA	nirk_Cluster39	0.001	0.0013	0.2893	0.1117	0.271	0.5737231	Organic
DNA	nirk_Cluster4	0.0827	0.0028	0.008	-0.24	0.8664	0.9381137	Organic
DNA	nirk_Cluster40	0.0232	-0.0081	0.1767	-0.0292	0.4066	0.6580549	Organic
DNA	nirk_Cluster41	0.0976	-0.024	0.1412	-0.0735	0.4629	0.6956685	Organic
DNA	nirk_Cluster44	0.0451	-0.0542	0.4301	0.2876	0.1573	0.5737231	Organic
DNA	nirk_Cluster46	0.0055	-0.0073	0.5919	0.4899	0.0737	0.5737231	Organic
DNA	nirk_Cluster48	0.0028	-0.0022	0.2189	0.0236	0.3495	0.6227278	Organic
DNA	nirk_Cluster49	0.0019	-0.0002	0.0055	-0.2431	0.889	0.9527586	Organic
DNA	nirk_Cluster5	0.1328	0.0022	0.0028	-0.2465	0.9208	0.9728498	Organic
DNA	nirk_Cluster50	0.0019	1.00E-04	0.0004	-0.2495	0.9715	0.9771021	Organic
DNA	nirk_Cluster52	0.0205	-0.0099	0.2348	0.0434	0.3301	0.6057502	Organic
DNA	nirk_Cluster53	0.0037	-0.0003	0.0031	-0.2462	0.9171	0.9728498	Organic
DNA	nirk_Cluster54	0.001	0.0008	0.0924	-0.1345	0.5581	0.7407953	Organic
DNA	nirk_Cluster55	0.001	0.0013	0.2893	0.1117	0.271	0.5737231	Organic
DNA	nirk_Cluster56	0.0008	-0.0009	0.2408	0.051	0.323	0.5989858	Organic
DNA	nirk_Cluster57	0.0012	0.0011	0.1439	-0.0701	0.4583	0.6927624	Organic
DNA	nirk_Cluster58	0.0053	-0.0015	0.0548	-0.1816	0.6554	0.811809	Organic
DNA	nirk_Cluster59	0.0028	0.001	0.0478	-0.1902	0.6772	0.8182394	Organic
DNA	nirk_Cluster6	0.117	-0.0514	0.4503	0.3129	0.1445	0.5737231	Organic
DNA	nirk_Cluster60	0.0008	0.001	0.2893	0.1117	0.271	0.5737231	Organic
DNA	nirk_Cluster62	0.0486	-0.0468	0.3971	0.2463	0.1799	0.5737231	Organic
DNA	nirk_Cluster63	0.0526	-0.0693	0.4265	0.2831	0.1597	0.5737231	Organic
DNA	nirk_Cluster64	0.0045	0.0013	0.0894	-0.1383	0.565	0.7457001	Organic
DNA	nirk_Cluster65	0.0296	-0.0034	0.0113	-0.2359	0.8414	0.9342807	Organic
DNA	nirk_Cluster66	0	0	#VALUE!	#VALUE!	#VALUE!	NA	Organic
DNA	nirk_Cluster67	0.0028	-0.0017	0.1705	-0.0368	0.4158	0.6580549	Organic
DNA	nirk_Cluster68	0.0044	-0.007	0.4277	0.2846	0.1589	0.5737231	Organic
DNA	nirk_Cluster69	0.0164	0.0004	0.0007	-0.2492	0.9616	0.9771021	Organic
DNA	nirk_Cluster7	0.0414	-0.0094	0.0913	-0.1359	0.5606	0.7422508	Organic
DNA	nirk_Cluster70	0.0293	-0.0369	0.4999	0.3748	0.1162	0.5737231	Organic
DNA	nirk_Cluster71	0.0009	0.0008	0.1439	-0.0701	0.4583	0.6927624	Organic
DNA	nirk_Cluster72	0.0144	-0.0069	0.1446	-0.0693	0.4572	0.6927624	Organic
DNA	nirk_Cluster73	0.0187	-0.0282	0.6753	0.5941	0.0448	0.5737231	Organic
DNA	nirk_Cluster74	0.2364	-0.3359	0.4426	0.3033	0.1493	0.5737231	Organic
DNA	nirk_Cluster75	0.005	-0.0079	0.4277	0.2846	0.1589	0.5737231	Organic
DNA	nirk_Cluster76	0.025	-0.0225	0.4524	0.3155	0.1432	0.5737231	Organic

DNA	nirk_Cluster77	0	0	#VALUE!	#VALUE!	#VALUE!	NA	Organic
DNA	nirk_Cluster78	0.0612	-0.0562	0.4424	0.3029	0.1495	0.5737231	Organic
DNA	nirk_Cluster79	0	0	#VALUE!	#VALUE!	#VALUE!	NA	Organic
DNA	nirk_Cluster8	0.048	0.0009	0.0007	-0.2491	0.96	0.9771021	Organic
DNA	nirk_Cluster80	0.0466	-0.0388	0.4235	0.2794	0.1617	0.5737231	Organic
DNA	nirk_Cluster82	0	0	#VALUE!	#VALUE!	#VALUE!	NA	Organic
DNA	nirk_Cluster83	0.0027	0.0013	0.0786	-0.1517	0.5904	0.7617846	Organic
DNA	nirk_Cluster84	0.0138	0.006	0.0834	-0.1458	0.5789	0.7551385	Organic
DNA	nirk_Cluster85	0.0005	-0.0006	0.2408	0.051	0.323	0.5989858	Organic
DNA	nirk_Cluster86	0.0121	-0.0178	0.4705	0.3381	0.1325	0.5737231	Organic
DNA	nirk_Cluster87	0.0057	-0.0066	0.3253	0.1566	0.2372	0.5737231	Organic
DNA	nirk_Cluster88	0.0025	-0.0014	0.1291	-0.0886	0.4841	0.7069263	Organic
DNA	nirk_Cluster89	0.001	-0.0012	0.2408	0.051	0.323	0.5989858	Organic
DNA	nirk_Cluster9	0.0285	0.0111	0.1186	-0.1018	0.5039	0.7195314	Organic
DNA	nirk_Cluster90	0.0017	-0.0024	0.764	0.705	0.0228	0.5737231	Organic
DNA	nirk_Cluster91	0.0208	-0.0084	0.0883	-0.1397	0.5675	0.7457001	Organic
DNA	nirk_Cluster92	0.0025	-0.0011	0.0853	-0.1434	0.5745	0.753012	Organic
DNA	nirk_Cluster93	0.0185	0.0044	0.2473	0.0592	0.3155	0.5989858	Organic
DNA	nirk_Cluster94	0.0056	0.0021	0.0507	-0.1866	0.6678	0.8160415	Organic
DNA	nirk_Cluster95	0.0238	-0.0113	0.1038	-0.1202	0.5334	0.7360512	Organic
DNA	nirk_Cluster96	0.0181	-0.0097	0.2126	0.0158	0.3574	0.6227278	Organic
DNA	nirk_Cluster97	0.0248	-0.0115	0.4614	0.3268	0.1378	0.5737231	Organic
DNA	nirk_Cluster98	0.007	-0.008	0.5113	0.3892	0.1102	0.5737231	Organic
DNA	nirk_Cluster99	0.0027	-0.0042	0.5167	0.3959	0.1075	0.5737231	Organic
DNA	nirs_Cluster0	0.0968	-0.0341	0.2733	0.0916	0.2873	0.5821367	Organic
DNA	nirs_Cluster1	0.0013	-0.0016	0.2408	0.051	0.323	0.5989858	Organic
DNA	nirs_Cluster14	0.0019	0.0025	0.2893	0.1117	0.271	0.5737231	Organic
DNA	nirs_Cluster2	0.005	-0.0034	0.1989	-0.0014	0.3754	0.631293	Organic
DNA	nirs_Cluster26	0	0	#VALUE!	#VALUE!	#VALUE!	NA	Organic
DNA	nirs_Cluster28	0	0	#VALUE!	#VALUE!	#VALUE!	NA	Organic
DNA	nirs_Cluster3	0.1217	-0.0263	0.3107	0.1383	0.2505	0.5737231	Organic
DNA	nirs_Cluster36	0	0	#VALUE!	#VALUE!	#VALUE!	NA	Organic
DNA	nirs_Cluster37	0	0	#VALUE!	#VALUE!	#VALUE!	NA	Organic
DNA	nirs_Cluster4	0	0	#VALUE!	#VALUE!	#VALUE!	NA	Organic
DNA	nirs_Cluster50	0.001	0.0007	0.1423	-0.0721	0.461	0.6947724	Organic
DNA	nirs_Cluster9	0	0	#VALUE!	#VALUE!	#VALUE!	NA	Organic
DNA	norb_Cluster0	0.0592	-0.0371	0.4027	0.2533	0.1759	0.5737231	Organic
DNA	norb_Cluster1	0.6783	-0.4675	0.3544	0.193	0.2125	0.5737231	Organic
DNA	norb_Cluster101	0	0	#VALUE!	#VALUE!	#VALUE!	NA	Organic
DNA	norb_Cluster102	0.0009	-0.0014	0.4277	0.2846	0.1589	0.5737231	Organic
DNA	norb_Cluster103	0	0	#VALUE!	#VALUE!	#VALUE!	NA	Organic
DNA	norb_Cluster104	0.0018	-0.0002	0.0031	-0.2462	0.9171	0.9728498	Organic
DNA	norb_Cluster106	0.0705	0.0336	0.1159	-0.1051	0.5091	0.7195314	Organic
DNA	norb_Cluster107	0.0056	-0.0019	0.1137	-0.1079	0.5134	0.7217454	Organic
DNA	norb_Cluster108	0.0009	-0.0015	0.4277	0.2846	0.1589	0.5737231	Organic
DNA	norb_Cluster109	0.0091	-0.0013	0.0152	-0.231	0.8162	0.9148251	Organic
DNA	norb_Cluster11	0.0053	-0.003	0.1163	-0.1046	0.5083	0.7195314	Organic
DNA	norb_Cluster110	0.0027	-0.0026	0.7654	0.7067	0.0225	0.5737231	Organic
DNA	norb_Cluster112	0.006	0.0072	0.2699	0.0874	0.2908	0.5821367	Organic
DNA	norb_Cluster113	0.0076	0.01	0.2893	0.1117	0.271	0.5737231	Organic
DNA	norb_Cluster114	0	0	#VALUE!	#VALUE!	#VALUE!	NA	Organic
DNA	norb_Cluster115	0.0394	0.0476	0.266	0.0825	0.295	0.5830102	Organic
DNA	norb_Cluster116	0.0046	0.006	0.2893	0.1117	0.271	0.5737231	Organic
DNA	norb_Cluster117	0.001	0.0013	0.2893	0.1117	0.271	0.5737231	Organic
DNA	norb_Cluster118	0.0019	-0.0026	0.8003	0.7504	0.0161	0.5737231	Organic

DNA	norb_Cluster119	0.001	0.0013	0.2893	0.1117	0.271	0.5737231	Organic
DNA	norb_Cluster12	0.0039	-0.0054	0.7973	0.7467	0.0166	0.5737231	Organic
DNA	norb_Cluster121	0	0	#VALUE!	#VALUE!	#VALUE!	NA	Organic
DNA	norb_Cluster122	0.0044	0.0015	0.0811	-0.1486	0.5843	0.7602311	Organic
DNA	norb_Cluster123	0.001	0.0013	0.2893	0.1117	0.271	0.5737231	Organic
DNA	norb_Cluster124	0.524	0.0213	0.0026	-0.2468	0.9241	0.9744214	Organic
DNA	norb_Cluster125	0.001	0.0013	0.2893	0.1117	0.271	0.5737231	Organic
DNA	norb_Cluster126	0.0012	0.0009	0.1676	-0.0405	0.4202	0.6580549	Organic
DNA	norb_Cluster127	0.0969	-0.0178	0.0428	-0.1965	0.6941	0.8250549	Organic
DNA	norb_Cluster128	0.0095	-0.0052	0.2898	0.1122	0.2706	0.5737231	Organic
DNA	norb_Cluster13	0.003	-0.0026	0.2219	0.0274	0.3456	0.619024	Organic
DNA	norb_Cluster131	0.0121	0.0091	0.2372	0.0465	0.3272	0.604747	Organic
DNA	norb_Cluster132	0.4322	-0.0169	0.0017	-0.2479	0.9381	0.9771021	Organic
DNA	norb_Cluster133	0.0007	-0.0012	0.4277	0.2846	0.1589	0.5737231	Organic
DNA	norb_Cluster135	0.0227	0.0046	0.0292	-0.2135	0.7461	0.8608647	Organic
DNA	norb_Cluster136	0.012	-0.0037	0.1243	-0.0946	0.493	0.7123103	Organic
DNA	norb_Cluster139	0.0171	-0.0036	0.0523	-0.1846	0.663	0.8139072	Organic
DNA	norb_Cluster14	0	0	#VALUE!	#VALUE!	#VALUE!	NA	Organic
DNA	norb_Cluster140	0.0318	-0.0348	0.4866	0.3583	0.1233	0.5737231	Organic
DNA	norb_Cluster142	0.0009	-0.0015	0.4277	0.2846	0.1589	0.5737231	Organic
DNA	norb_Cluster144	0.0038	0.005	0.2893	0.1117	0.271	0.5737231	Organic
DNA	norb_Cluster146	0.0174	-0.0007	0.0007	-0.2491	0.9593	0.9771021	Organic
DNA	norb_Cluster15	0.0387	-0.0187	0.1709	-0.0364	0.4153	0.6580549	Organic
DNA	norb_Cluster150	0.0052	-0.0053	0.4202	0.2752	0.1639	0.5737231	Organic
DNA	norb_Cluster155	0.1387	-0.0013	1.00E-04	-0.2499	0.9851	0.9872826	Organic
DNA	norb_Cluster16	0.0045	-0.0009	0.0833	-0.1458	0.579	0.7551385	Organic
DNA	norb_Cluster164	0.0091	-0.009	0.2714	0.0893	0.2892	0.5821367	Organic
DNA	norb_Cluster165	0.0036	0.0026	0.139	-0.0763	0.4667	0.6964051	Organic
DNA	norb_Cluster166	0.0024	0.0019	0.1676	-0.0405	0.4202	0.6580549	Organic
DNA	norb_Cluster169	0.0062	-0.0017	0.0296	-0.213	0.7446	0.8608647	Organic
DNA	norb_Cluster17	0.0009	-0.0015	0.4277	0.2846	0.1589	0.5737231	Organic
DNA	norb_Cluster170	0.0104	0.0111	0.2323	0.0404	0.333	0.6088992	Organic
DNA	norb_Cluster171	0.0019	0.0025	0.2893	0.1117	0.271	0.5737231	Organic
DNA	norb_Cluster172	0.0043	-0.0013	0.0381	-0.2024	0.711	0.8375084	Organic
DNA	norb_Cluster175	0.0115	0.0007	0.0015	-0.2482	0.9426	0.9771021	Organic
DNA	norb_Cluster176	0.0065	0.0003	0.0012	-0.2485	0.9475	0.9771021	Organic
DNA	norb_Cluster177	0.0084	0.0088	0.231	0.0388	0.3346	0.6096559	Organic
DNA	norb_Cluster179	0	0	#VALUE!	#VALUE!	#VALUE!	NA	Organic
DNA	norb_Cluster18	0.0009	-0.0015	0.4277	0.2846	0.1589	0.5737231	Organic
DNA	norb_Cluster180	0.0051	-0.0023	0.1372	-0.0785	0.4698	0.6964051	Organic
DNA	norb_Cluster181	0.0019	0.0025	0.2893	0.1117	0.271	0.5737231	Organic
DNA	norb_Cluster182	0	0	#VALUE!	#VALUE!	#VALUE!	NA	Organic
DNA	norb_Cluster184	0.0073	0.0018	0.0221	-0.2224	0.7789	0.8886753	Organic
DNA	norb_Cluster186	0.001	0.0013	0.2893	0.1117	0.271	0.5737231	Organic
DNA	norb_Cluster19	0.0028	0.0011	0.0538	-0.1828	0.6584	0.811809	Organic
DNA	norb_Cluster192	0.0054	-0.0003	0.0015	-0.2482	0.9428	0.9771021	Organic
DNA	norb_Cluster193	0.0019	0.0025	0.2893	0.1117	0.271	0.5737231	Organic
DNA	norb_Cluster197	0.0065	0.0063	0.2117	0.0147	0.3585	0.6227278	Organic
DNA	norb_Cluster2	0.003	0.0035	0.5301	0.4126	0.1009	0.5737231	Organic
DNA	norb_Cluster20	0.0394	-0.0143	0.1636	-0.0455	0.4264	0.6656325	Organic
DNA	norb_Cluster208	0	0	#VALUE!	#VALUE!	#VALUE!	NA	Organic
DNA	norb_Cluster21	0.0329	-0.0299	0.3788	0.2235	0.1933	0.5737231	Organic
DNA	norb_Cluster211	0.001	0.0013	0.2893	0.1117	0.271	0.5737231	Organic
DNA	norb_Cluster212	0.0295	0.0388	0.2893	0.1117	0.271	0.5737231	Organic
DNA	norb_Cluster214	0.001	0.0013	0.2893	0.1117	0.271	0.5737231	Organic

DNA	norb_Cluster217	0.0057	0.0075	0.2893	0.1117	0.271	0.5737231	Organic
DNA	norb_Cluster219	0.0019	0.0025	0.2893	0.1117	0.271	0.5737231	Organic
DNA	norb_Cluster22	0.0017	-0.0027	0.4277	0.2846	0.1589	0.5737231	Organic
DNA	norb_Cluster220	0	0	#VALUE!	#VALUE!	#VALUE!	NA	Organic
DNA	norb_Cluster221	0	0	#VALUE!	#VALUE!	#VALUE!	NA	Organic
DNA	norb_Cluster222	0	0	#VALUE!	#VALUE!	#VALUE!	NA	Organic
DNA	norb_Cluster224	0.0086	0.0113	0.2893	0.1117	0.271	0.5737231	Organic
DNA	norb_Cluster225	0.0104	0.0137	0.2893	0.1117	0.271	0.5737231	Organic
DNA	norb_Cluster226	0.0029	0.0038	0.2893	0.1117	0.271	0.5737231	Organic
DNA	norb_Cluster228	0.0086	0.0113	0.2893	0.1117	0.271	0.5737231	Organic
DNA	norb_Cluster229	0.0019	0.0025	0.2893	0.1117	0.271	0.5737231	Organic
DNA	norb_Cluster230	0	0	#VALUE!	#VALUE!	#VALUE!	NA	Organic
DNA	norb_Cluster231	0	0	#VALUE!	#VALUE!	#VALUE!	NA	Organic
DNA	norb_Cluster232	0	0	#VALUE!	#VALUE!	#VALUE!	NA	Organic
DNA	norb_Cluster234	0	0	#VALUE!	#VALUE!	#VALUE!	NA	Organic
DNA	norb_Cluster235	0	0	#VALUE!	#VALUE!	#VALUE!	NA	Organic
DNA	norb_Cluster238	0.001	-0.0002	0.0059	-0.2427	0.8855	0.9527586	Organic
DNA	norb_Cluster239	0.001	-0.0012	0.2408	0.051	0.323	0.5989858	Organic
DNA	norb_Cluster24	0.1693	-0.1177	0.336	0.17	0.2279	0.5737231	Organic
DNA	norb_Cluster244	0	0	#VALUE!	#VALUE!	#VALUE!	NA	Organic
DNA	norb_Cluster247	0.0017	1.00E-04	0.0004	-0.2495	0.9708	0.9771021	Organic
DNA	norb_Cluster25	0.0743	-0.0415	0.291	0.1138	0.2693	0.5737231	Organic
DNA	norb_Cluster250	0.0038	0.005	0.2893	0.1117	0.271	0.5737231	Organic
DNA	norb_Cluster251	0.002	0.002	0.4353	0.2941	0.1539	0.5737231	Organic
DNA	norb_Cluster26	0.0426	-0.0473	0.4292	0.2865	0.1579	0.5737231	Organic
DNA	norb_Cluster28	0.0171	0.0174	0.3685	0.2107	0.2012	0.5737231	Organic
DNA	norb_Cluster29	0.1633	-0.0059	0.0035	-0.2457	0.9117	0.9728498	Organic
DNA	norb_Cluster31	0.0281	-0.028	0.4251	0.2814	0.1606	0.5737231	Organic
DNA	norb_Cluster32	0.0675	-0.0723	0.5028	0.3785	0.1146	0.5737231	Organic
DNA	norb_Cluster33	0.6461	0.0653	0.1687	-0.0391	0.4185	0.6580549	Organic
DNA	norb_Cluster35	0.0334	-0.0292	0.4775	0.3469	0.1285	0.5737231	Organic
DNA	norb_Cluster36	1.2204	-0.3724	0.3132	0.1415	0.2482	0.5737231	Organic
DNA	norb_Cluster37	0.8221	-0.2027	0.3696	0.212	0.2004	0.5737231	Organic
DNA	norb_Cluster38	0.0063	0.0002	0.0008	-0.249	0.9576	0.9771021	Organic
DNA	norb_Cluster39	0.0328	-0.0238	0.2491	0.0613	0.3136	0.5989858	Organic
DNA	norb_Cluster40	0.0977	-0.0288	0.073	-0.1588	0.6046	0.7723363	Organic
DNA	norb_Cluster42	0.2962	-0.0708	0.337	0.1712	0.2271	0.5737231	Organic
DNA	norb_Cluster44	0.0015	-0.0024	0.4277	0.2846	0.1589	0.5737231	Organic
DNA	norb_Cluster46	0.2508	0.1139	0.2981	0.1226	0.2624	0.5737231	Organic
DNA	norb_Cluster47	0.3292	-0.1401	0.2696	0.087	0.2911	0.5821367	Organic
DNA	norb_Cluster5	0.0063	-0.002	0.2115	0.0144	0.3588	0.6227278	Organic
DNA	norb_Cluster51	0.0016	-0.0014	0.295	0.1187	0.2654	0.5737231	Organic
DNA	norb_Cluster52	0.1345	-0.0799	0.3607	0.2008	0.2075	0.5737231	Organic
DNA	norb_Cluster56	0.0045	-0.0022	0.2091	0.0114	0.3619	0.6227278	Organic
DNA	norb_Cluster57	0.0019	0.0025	0.2893	0.1117	0.271	0.5737231	Organic
DNA	norb_Cluster59	0.002	-0.0003	0.0108	-0.2364	0.8443	0.9355699	Organic
DNA	norb_Cluster6	0.6681	-0.2203	0.3266	0.1583	0.2361	0.5737231	Organic
DNA	norb_Cluster60	0.2572	0.0688	0.0534	-0.1833	0.6597	0.811809	Organic
DNA	norb_Cluster61	0.3154	0.1652	0.1533	-0.0584	0.4427	0.6829897	Organic
DNA	norb_Cluster62	0.001	0.0013	0.2893	0.1117	0.271	0.5737231	Organic
DNA	norb_Cluster64	0.0123	0.0036	0.0411	-0.1987	0.7002	0.8285534	Organic
DNA	norb_Cluster66	0	0	#VALUE!	#VALUE!	#VALUE!	NA	Organic
DNA	norb_Cluster67	0.0065	-0.0028	0.1268	-0.0915	0.4885	0.7096715	Organic
DNA	norb_Cluster68	0.0044	0.0004	0.0143	-0.2321	0.8212	0.9177474	Organic
DNA	norb_Cluster69	0.0018	-0.0029	0.4277	0.2846	0.1589	0.5737231	Organic

DNA	norb_Cluster7	0.0518	-0.0082	0.0581	-0.1774	0.6455	0.8018621	Organic
DNA	norb_Cluster70	0.0526	0.0133	0.0295	-0.2132	0.745	0.8608647	Organic
DNA	norb_Cluster71	0.0753	0.0338	0.1173	-0.1033	0.5063	0.7195314	Organic
DNA	norb_Cluster74	0.0178	0.0023	0.0074	-0.2408	0.8717	0.9414421	Organic
DNA	norb_Cluster75	0.0691	0.0042	0.0029	-0.2464	0.9193	0.9728498	Organic
DNA	norb_Cluster76	0.0831	0.0236	0.0423	-0.1971	0.6959	0.8252395	Organic
DNA	norb_Cluster77	0.0432	0.0029	0.0082	-0.2397	0.8643	0.9381137	Organic
DNA	norb_Cluster8	0.0116	0.0057	0.107	-0.1162	0.5267	0.7324644	Organic
DNA	norb_Cluster80	0	0	#VALUE!	#VALUE!	#VALUE!	NA	Organic
DNA	norb_Cluster81	0.001	0.0013	0.2893	0.1117	0.271	0.5737231	Organic
DNA	norb_Cluster83	0	0	#VALUE!	#VALUE!	#VALUE!	NA	Organic
DNA	norb_Cluster84	0.0054	-0.0008	0.0243	-0.2196	0.7679	0.8818511	Organic
DNA	norb_Cluster85	0.0018	-0.0002	0.0031	-0.2462	0.9171	0.9728498	Organic
DNA	norb_Cluster86	0.0061	0.0034	0.1099	-0.1126	0.5209	0.7294198	Organic
DNA	norb_Cluster88	0.001	0.0013	0.2893	0.1117	0.271	0.5737231	Organic
DNA	norb_Cluster90	0	0	#VALUE!	#VALUE!	#VALUE!	NA	Organic
DNA	norb_Cluster91	0.3756	0.0664	0.0238	-0.2203	0.7706	0.8818511	Organic
DNA	norb_Cluster92	0	0	#VALUE!	#VALUE!	#VALUE!	NA	Organic
DNA	norb_Cluster93	0.001	0.0013	0.2893	0.1117	0.271	0.5737231	Organic
DNA	norb_Cluster94	0.0009	0.0007	0.0924	-0.1345	0.5581	0.7407953	Organic
DNA	norb_Cluster96	0.001	0.0013	0.2893	0.1117	0.271	0.5737231	Organic
DNA	norb_Cluster97	0.0012	-0.0018	0.6528	0.566	0.0518	0.5737231	Organic
DNA	norb_Cluster98	0.0022	0.0017	0.3391	0.1738	0.2253	0.5737231	Organic
DNA	nosz_Cluster0	0.0017	0.0003	0.0151	-0.2312	0.8169	0.9148251	Organic
DNA	nosz_Cluster10	0.0156	0.0179	0.4398	0.2998	0.151	0.5737231	Organic
DNA	nosz_Cluster102	0.001	0.0013	0.2893	0.1117	0.271	0.5737231	Organic
DNA	nosz_Cluster103	0.0355	-0.0005	0.0007	-0.2491	0.9603	0.9771021	Organic
DNA	nosz_Cluster105	0.0017	-0.0005	0.0371	-0.2037	0.7147	0.8381411	Organic
DNA	nosz_Cluster106	0.0036	-0.0026	0.3744	0.2179	0.1968	0.5737231	Organic
DNA	nosz_Cluster107	0	0	#VALUE!	#VALUE!	#VALUE!	NA	Organic
DNA	nosz_Cluster108	0.0033	0.0024	0.3148	0.1434	0.2467	0.5737231	Organic
DNA	nosz_Cluster109	0.0272	0.001	0.0018	-0.2478	0.9367	0.9771021	Organic
DNA	nosz_Cluster11	0.019	0.0251	0.2893	0.1117	0.271	0.5737231	Organic
DNA	nosz_Cluster110	0	0	#VALUE!	#VALUE!	#VALUE!	NA	Organic
DNA	nosz_Cluster111	0.0019	0.0014	0.0924	-0.1345	0.5581	0.7407953	Organic
DNA	nosz_Cluster116	0.0048	0.0063	0.2893	0.1117	0.271	0.5737231	Organic
DNA	nosz_Cluster118	0	0	#VALUE!	#VALUE!	#VALUE!	NA	Organic
DNA	nosz_Cluster119	0	0	#VALUE!	#VALUE!	#VALUE!	NA	Organic
DNA	nosz_Cluster120	0.0039	-0.0012	0.069	-0.1638	0.6152	0.774766	Organic
DNA	nosz_Cluster123	0	0	#VALUE!	#VALUE!	#VALUE!	NA	Organic
DNA	nosz_Cluster128	0.0038	0.005	0.2893	0.1117	0.271	0.5737231	Organic
DNA	nosz_Cluster129	0.0478	0.0086	0.1851	-0.0186	0.3944	0.6466501	Organic
DNA	nosz_Cluster13	0.0093	0.0045	0.0777	-0.1529	0.5928	0.7617846	Organic
DNA	nosz_Cluster130	0.0272	0.0105	0.3431	0.1788	0.2219	0.5737231	Organic
DNA	nosz_Cluster132	0.0031	0.0003	0.0094	-0.2383	0.8553	0.9381137	Organic
DNA	nosz_Cluster14	0.0467	0.0609	0.2991	0.1239	0.2614	0.5737231	Organic
DNA	nosz_Cluster16	0.0067	0.0088	0.2893	0.1117	0.271	0.5737231	Organic
DNA	nosz_Cluster17	0.0207	0.0082	0.1856	-0.018	0.3937	0.6466501	Organic
DNA	nosz_Cluster18	0.0105	0.0138	0.2893	0.1117	0.271	0.5737231	Organic
DNA	nosz_Cluster20	0.0026	-0.0001	0.0015	-0.2481	0.9418	0.9771021	Organic
DNA	nosz_Cluster21	0	0	#VALUE!	#VALUE!	#VALUE!	NA	Organic
DNA	nosz_Cluster22	0.0123	0.0104	0.2166	0.0207	0.3523	0.6227278	Organic
DNA	nosz_Cluster23	0.0017	1.00E-04	0.0004	-0.2495	0.9708	0.9771021	Organic
DNA	nosz_Cluster24	0	0	#VALUE!	#VALUE!	#VALUE!	NA	Organic
DNA	nosz_Cluster25	0.0038	0.005	0.2893	0.1117	0.271	0.5737231	Organic

DNA	nosz_Cluster26	0.0018	0.0018	0.403	0.2538	0.1757	0.5737231	Organic
DNA	nosz_Cluster27	0.001	0.0013	0.2893	0.1117	0.271	0.5737231	Organic
DNA	nosz_Cluster28	0.0167	-0.0021	0.0082	-0.2397	0.8642	0.9381137	Organic
DNA	nosz_Cluster30	0.0114	0.0139	0.379	0.2237	0.1932	0.5737231	Organic
DNA	nosz_Cluster32	0.0079	0.0092	0.3372	0.1715	0.2268	0.5737231	Organic
DNA	nosz_Cluster33	0.0162	0.0213	0.2893	0.1117	0.271	0.5737231	Organic
DNA	nosz_Cluster34	0.0353	0.0293	0.2078	0.0097	0.3636	0.6227866	Organic
DNA	nosz_Cluster35	0.0019	0.0025	0.2893	0.1117	0.271	0.5737231	Organic
DNA	nosz_Cluster36	0.0096	0.012	0.3406	0.1758	0.224	0.5737231	Organic
DNA	nosz_Cluster37	0.0019	0.0025	0.2893	0.1117	0.271	0.5737231	Organic
DNA	nosz_Cluster38	0.0087	0.0082	0.5381	0.4227	0.097	0.5737231	Organic
DNA	nosz_Cluster39	0.0246	0.0185	0.1702	-0.0373	0.4163	0.6580549	Organic
DNA	nosz_Cluster40	0.0106	0.0128	0.3866	0.2333	0.1875	0.5737231	Organic
DNA	nosz_Cluster41	0.0064	0.0032	0.1231	-0.0962	0.4954	0.7137479	Organic
DNA	nosz_Cluster42	0.0226	0.021	0.2769	0.0962	0.2835	0.5821367	Organic
DNA	nosz_Cluster43	0	0	#VALUE!	#VALUE!	#VALUE!	NA	Organic
DNA	nosz_Cluster44	0.0027	-0.0036	0.5483	0.4354	0.0923	0.5737231	Organic
DNA	nosz_Cluster45	0.0018	-1.00E-04	0.0006	-0.2492	0.9626	0.9771021	Organic
DNA	nosz_Cluster46	0.0038	0.0026	0.137	-0.0787	0.4702	0.6964051	Organic
DNA	nosz_Cluster47	0	0	#VALUE!	#VALUE!	#VALUE!	NA	Organic
DNA	nosz_Cluster48	0.0009	0.0007	0.0924	-0.1345	0.5581	0.7407953	Organic
DNA	nosz_Cluster49	0.002	0.0022	0.5173	0.3966	0.1072	0.5737231	Organic
DNA	nosz_Cluster5	0.0057	0.0075	0.2893	0.1117	0.271	0.5737231	Organic
DNA	nosz_Cluster51	0	0	#VALUE!	#VALUE!	#VALUE!	NA	Organic
DNA	nosz_Cluster53	0	0	#VALUE!	#VALUE!	#VALUE!	NA	Organic
DNA	nosz_Cluster54	0	0	#VALUE!	#VALUE!	#VALUE!	NA	Organic
DNA	nosz_Cluster57	0.0572	-0.0003	0.00E+00	-0.25	0.9938	0.9938486	Organic
DNA	nosz_Cluster58	0.0019	-0.0002	0.0055	-0.2431	0.889	0.9527586	Organic
DNA	nosz_Cluster59	0.001	0.0013	0.2893	0.1117	0.271	0.5737231	Organic
DNA	nosz_Cluster6	0.0027	0.0031	0.4556	0.3195	0.1413	0.5737231	Organic
DNA	nosz_Cluster60	0.0027	0.0019	0.4181	0.2726	0.1653	0.5737231	Organic
DNA	nosz_Cluster61	0.0179	-0.0068	0.228	0.035	0.3382	0.6109232	Organic
DNA	nosz_Cluster62	0.0212	-0.0009	0.0015	-0.2481	0.942	0.9771021	Organic
DNA	nosz_Cluster64	0	0	#VALUE!	#VALUE!	#VALUE!	NA	Organic
DNA	nosz_Cluster65	0.0019	0.0025	0.2893	0.1117	0.271	0.5737231	Organic
DNA	nosz_Cluster66	0.0065	-0.0072	0.5097	0.3872	0.111	0.5737231	Organic
DNA	nosz_Cluster67	0.0153	-0.0014	0.009	-0.2387	0.8578	0.9381137	Organic
DNA	nosz_Cluster69	0.0013	0.0004	0.0322	-0.2098	0.7339	0.8567605	Organic
DNA	nosz_Cluster70	0.0037	0.0023	0.1163	-0.1047	0.5084	0.7195314	Organic
DNA	nosz_Cluster71	0.0038	0.0043	0.5247	0.4058	0.1035	0.5737231	Organic
DNA	nosz_Cluster72	0.012	0.0037	0.103	-0.1213	0.5352	0.7366013	Organic
DNA	nosz_Cluster73	0.0029	0.0009	0.0331	-0.2087	0.7302	0.8544041	Organic
DNA	nosz_Cluster74	0.0017	1.00E-04	0.0004	-0.2495	0.9708	0.9771021	Organic
DNA	nosz_Cluster75	0.0085	0.0021	0.0454	-0.1932	0.6852	0.8200131	Organic
DNA	nosz_Cluster77	0	0	#VALUE!	#VALUE!	#VALUE!	NA	Organic
DNA	nosz_Cluster78	0.002	-0.0014	0.209	0.0113	0.362	0.6227278	Organic
DNA	nosz_Cluster79	0.0165	0.0061	0.1363	-0.0796	0.4714	0.6964051	Organic
DNA	nosz_Cluster8	0.0082	0.0059	0.1946	-0.0067	0.3812	0.6368998	Organic
DNA	nosz_Cluster80	0.006	0.0072	0.2699	0.0874	0.2908	0.5821367	Organic
DNA	nosz_Cluster81	0.0028	0.001	0.0478	-0.1902	0.6772	0.8182394	Organic
DNA	nosz_Cluster82	0	0	#VALUE!	#VALUE!	#VALUE!	NA	Organic
DNA	nosz_Cluster83	0.0015	-0.0008	0.1081	-0.1149	0.5246	0.7316454	Organic
DNA	nosz_Cluster85	0.001	0.0013	0.2893	0.1117	0.271	0.5737231	Organic
DNA	nosz_Cluster86	0	0	#VALUE!	#VALUE!	#VALUE!	NA	Organic
DNA	nosz_Cluster87	0.002	0.002	0.4353	0.2941	0.1539	0.5737231	Organic

DNA	nosz_Cluster88	0.0148	-0.0025	0.0465	-0.1919	0.6816	0.8194506	Organic
DNA	nosz_Cluster89	0.0114	-0.0096	0.2621	0.0776	0.2992	0.583864	Organic
DNA	nosz_Cluster9	0.0019	0.0011	0.1285	-0.0893	0.4853	0.7069263	Organic
DNA	nosz_Cluster90	0.0056	0.0014	0.043	-0.1962	0.6933	0.8250549	Organic
DNA	nosz_Cluster91	0.0082	0.008	0.2552	0.069	0.3067	0.5940168	Organic
DNA	nosz_Cluster93	0.0157	0.0003	0.0009	-0.2488	0.9539	0.9771021	Organic
DNA	nosz_Cluster95	0.001	0.0008	0.0924	-0.1345	0.5581	0.7407953	Organic
DNA	nosz_Cluster96	0.0072	0.0031	0.1347	-0.0817	0.4743	0.6987269	Organic
DNA	nosz_Cluster97	0	0	#VALUE!	#VALUE!	#VALUE!	NA	Organic
DNA	nosz_Cluster98	0	0	#VALUE!	#VALUE!	#VALUE!	NA	Organic
DNA	nosz_Cluster99	0.0042	0.0037	0.3912	0.239	0.1842	0.5737231	Organic
DNA	nrfa_Cluster10	0.0368	0.0393	0.2429	0.0537	0.3205	0.5989858	Organic
DNA	nrfa_Cluster100	0.0248	0.0115	0.1701	-0.0374	0.4165	0.6580549	Organic
DNA	nrfa_Cluster104	0.0308	-0.0077	0.0291	-0.2137	0.7467	0.8608647	Organic
DNA	nrfa_Cluster105	0.0067	0.0088	0.2893	0.1117	0.271	0.5737231	Organic
DNA	nrfa_Cluster109	0.1147	0.044	0.0596	-0.1755	0.6411	0.7982643	Organic
DNA	nrfa_Cluster116	0.1051	-0.0547	0.1702	-0.0373	0.4163	0.6580549	Organic
DNA	nrfa_Cluster118	0.0309	0.0183	0.1907	-0.0117	0.3866	0.6393507	Organic
DNA	nrfa_Cluster125	0.0128	0.0086	0.19	-0.0125	0.3875	0.6393507	Organic
DNA	nrfa_Cluster154	0.0212	0.019	0.295	0.1188	0.2654	0.5737231	Organic
DNA	nrfa_Cluster164	0.0048	0.0063	0.2893	0.1117	0.271	0.5737231	Organic
DNA	nrfa_Cluster172	0	0	#VALUE!	#VALUE!	#VALUE!	NA	Organic
DNA	nrfa_Cluster175	0.0022	0.0019	0.4169	0.2711	0.1661	0.5737231	Organic
DNA	nrfa_Cluster20	0.0101	0.0019	0.0169	-0.2289	0.8063	0.9127251	Organic
DNA	nrfa_Cluster26	0.0192	0.0073	0.0778	-0.1527	0.5924	0.7617846	Organic
DNA	nrfa_Cluster29	0.0019	0.0025	0.2893	0.1117	0.271	0.5737231	Organic
DNA	nrfa_Cluster34	0.01	-0.0034	0.07	-0.1626	0.6125	0.774766	Organic
DNA	nrfa_Cluster38	0.0028	0.001	0.0478	-0.1902	0.6772	0.8182394	Organic
DNA	nrfa_Cluster61	0.0009	-0.0015	0.4277	0.2846	0.1589	0.5737231	Organic
DNA	nrfa_Cluster62	0.0112	0.0043	0.0768	-0.154	0.595	0.76274	Organic
DNA	nrfa_Cluster66	0.058	0.0095	0.0123	-0.2346	0.8343	0.9303109	Organic
DNA	nrfa_Cluster69	0.0017	0.0003	0.0151	-0.2312	0.8169	0.9148251	Organic
DNA	nrfa_Cluster71	0.0139	0.0145	0.2361	0.0451	0.3285	0.6049414	Organic
DNA	nrfa_Cluster72	0.014	0.0046	0.1096	-0.113	0.5216	0.7294198	Organic
DNA	nrfa_Cluster73	0.0029	0.0038	0.2893	0.1117	0.271	0.5737231	Organic
DNA	nrfa_Cluster74	0.0428	0.0255	0.1325	-0.0844	0.4782	0.7025175	Organic
DNA	nrfa_Cluster76	0.0339	0.039	0.3629	0.2036	0.2057	0.5737231	Organic
DNA	nrfa_Cluster79	0.0456	0.0601	0.2893	0.1117	0.271	0.5737231	Organic
DNA	nrfa_Cluster87	0.0076	0.01	0.2893	0.1117	0.271	0.5737231	Organic
DNA	nrfa_Cluster89	0.0008	-0.0009	0.2408	0.051	0.323	0.5989858	Organic
DNA	nrfa_Cluster92	0.0048	0.0063	0.2893	0.1117	0.271	0.5737231	Organic
DNA	nrfa_Cluster93	0.0045	0.0009	0.0289	-0.2138	0.7473	0.8608647	Organic
DNA	nrfa_Cluster94	0	0	#VALUE!	#VALUE!	#VALUE!	NA	Organic
DNA	nrfa_Cluster97	0.0028	-0.0001	0.0011	-0.2486	0.9492	0.9771021	Organic
DNA	nrfa_Cluster99	0.0029	0.0033	0.4571	0.3214	0.1404	0.5737231	Organic
Water_content	amoA_A_Cluster26	0.0009	-0.0017	0.5977	0.4971	0.0714	0.3805486	Organic
Water_content	amoA_A_Cluster45	0	0	#VALUE!	#VALUE!	#VALUE!	NA	Organic
Water_content	amoA_B_Cluster0	0.0665	-0.0356	0.7517	0.6896	0.0254	0.3805486	Organic
Water_content	amoA_B_Cluster1	0.0089	-0.0147	0.6306	0.5382	0.0592	0.3805486	Organic
Water_content	amoA_B_Cluster10	0.013	-0.0163	0.5671	0.4588	0.0839	0.4035856	Organic
Water_content	amoA_B_Cluster11	0.0103	-0.0142	0.6578	0.5723	0.0502	0.3805486	Organic
Water_content	amoA_B_Cluster12	0.0501	-0.0309	0.5731	0.4664	0.0814	0.4031833	Organic
Water_content	amoA_B_Cluster13	0.2409	-0.3103	0.6595	0.5743	0.0497	0.3805486	Organic
Water_content	amoA_B_Cluster14	0.0117	-0.0215	0.6275	0.5344	0.0603	0.3805486	Organic
Water_content	amoA_B_Cluster15	0.0082	-0.0123	0.6381	0.5476	0.0567	0.3805486	Organic

Water_content	amoA_B_Cluster2	0.0666	-0.1022	0.6982	0.6228	0.0383	0.3805486	Organic
Water_content	amoA_B_Cluster20	0.0007	-0.0014	0.5977	0.4971	0.0714	0.3805486	Organic
Water_content	amoA_B_Cluster23	0.0211	-0.0178	0.5877	0.4846	0.0754	0.3863826	Organic
Water_content	amoA_B_Cluster3	0.0028	-0.0033	0.5121	0.3901	0.1098	0.4383994	Organic
Water_content	amoA_B_Cluster4	0.0017	-0.0032	0.5977	0.4971	0.0714	0.3805486	Organic
Water_content	amoA_B_Cluster5	0.0667	-0.0544	0.7938	0.7422	0.0172	0.3805486	Organic
Water_content	amoA_B_Cluster6	0.0116	-0.0098	0.8361	0.7951	0.0107	0.3805486	Organic
Water_content	amoA_B_Cluster7	0.0047	0.0016	0.0792	-0.151	0.5889	0.8719664	Organic
Water_content	amoA_B_Cluster8	0.0054	-0.0062	0.7009	0.6261	0.0376	0.3805486	Organic
Water_content	amoA_B_Cluster9	0.019	-0.0275	0.7069	0.6336	0.036	0.3805486	Organic
Water_content	nifh_Cluster0	0.0368	0.0094	0.6286	0.5358	0.0599	0.3805486	Organic
Water_content	nifh_Cluster10	0.0093	0.0016	0.0074	-0.2408	0.8716	0.9740331	Organic
Water_content	nifh_Cluster100	0.0019	0.0012	0.0642	-0.1697	0.6281	0.8719664	Organic
Water_content	nifh_Cluster103	0.0259	-0.0143	0.3956	0.2445	0.181	0.5770643	Organic
Water_content	nifh_Cluster1034	0.0074	-0.004	0.3321	0.1651	0.2313	0.6626931	Organic
Water_content	nifh_Cluster105	0.0047	-0.0012	0.0862	-0.1422	0.5721	0.8719664	Organic
Water_content	nifh_Cluster108	0.0098	-0.0069	0.3461	0.1826	0.2194	0.6517478	Organic
Water_content	nifh_Cluster1099	0.1117	0.0462	0.8053	0.7566	0.0153	0.3805486	Organic
Water_content	nifh_Cluster11	0.001	0.0006	0.0642	-0.1697	0.6281	0.8719664	Organic
Water_content	nifh_Cluster1112	0.0008	-0.0005	0.0702	-0.1622	0.6118	0.8719664	Organic
Water_content	nifh_Cluster112	0.0145	0.0025	0.1032	-0.121	0.5347	0.8719664	Organic
Water_content	nifh_Cluster113	0.021	0.007	0.1785	-0.0269	0.404	0.8719664	Organic
Water_content	nifh_Cluster114	0.0403	-0.0076	0.3644	0.2055	0.2045	0.6288238	Organic
Water_content	nifh_Cluster1141	0.0138	-0.0152	0.7107	0.6383	0.035	0.3805486	Organic
Water_content	nifh_Cluster115	0.0088	0.0044	0.3502	0.1877	0.216	0.6491637	Organic
Water_content	nifh_Cluster116	0.0503	-0.0208	0.274	0.0924	0.2866	0.7347126	Organic
Water_content	nifh_Cluster1163	0.0028	-0.0018	0.2917	0.1147	0.2686	0.7064556	Organic
Water_content	nifh_Cluster1179	0.0016	-0.0009	0.1278	-0.0902	0.4866	0.8719664	Organic
Water_content	nifh_Cluster1182	0.0191	0.0013	0.0296	-0.213	0.7445	0.8944593	Organic
Water_content	nifh_Cluster1183	0.0119	0.0004	0.0027	-0.2466	0.9218	0.9777342	Organic
Water_content	nifh_Cluster1197	0.0113	-0.003	0.1163	-0.1047	0.5084	0.8719664	Organic
Water_content	nifh_Cluster1199	0.0041	-0.003	0.6426	0.5532	0.0551	0.3805486	Organic
Water_content	nifh_Cluster120	0.0019	0.0012	0.0642	-0.1697	0.6281	0.8719664	Organic
Water_content	nifh_Cluster1203	0	0	#VALUE!	#VALUE!	#VALUE!	NA	Organic
Water_content	nifh_Cluster1206	0	0	#VALUE!	#VALUE!	#VALUE!	NA	Organic
Water_content	nifh_Cluster1207	0.0064	-0.0062	0.3548	0.1935	0.2122	0.6452468	Organic
Water_content	nifh_Cluster1209	0.1228	-0.0014	0.0056	-0.243	0.8883	0.9777342	Organic
Water_content	nifh_Cluster121	0.0027	0.0007	0.0214	-0.2233	0.7822	0.9193125	Organic
Water_content	nifh_Cluster1211	0.0026	-0.0008	0.0713	-0.1608	0.6089	0.8719664	Organic
Water_content	nifh_Cluster1213	0.0012	0.002	0.4623	0.3279	0.1372	0.4850007	Organic
Water_content	nifh_Cluster122	0	0	#VALUE!	#VALUE!	#VALUE!	NA	Organic
Water_content	nifh_Cluster123	0.0105	0.0065	0.0642	-0.1697	0.6281	0.8719664	Organic
Water_content	nifh_Cluster1236	0.0046	-0.0015	0.0411	-0.1986	0.7001	0.8916615	Organic
Water_content	nifh_Cluster1238	0.0012	0.0004	0.0336	-0.208	0.7281	0.8916615	Organic
Water_content	nifh_Cluster124	0.0066	0.0028	0.0674	-0.1658	0.6194	0.8719664	Organic
Water_content	nifh_Cluster1261	0.0012	0.00E+00	0.0002	-0.2497	0.9765	0.9830342	Organic
Water_content	nifh_Cluster1262	0.0028	-0.0011	0.0532	-0.1835	0.6602	0.8719664	Organic
Water_content	nifh_Cluster1266	0.0148	0.0114	0.6624	0.578	0.0487	0.3805486	Organic
Water_content	nifh_Cluster1267	0.0996	0.0316	0.3915	0.2393	0.184	0.5786787	Organic
Water_content	nifh_Cluster127	0.0009	-0.0017	0.5977	0.4971	0.0714	0.3805486	Organic
Water_content	nifh_Cluster1278	0.0346	-0.0316	0.475	0.3438	0.1299	0.4716244	Organic
Water_content	nifh_Cluster128	0.0019	0.0012	0.0642	-0.1697	0.6281	0.8719664	Organic
Water_content	nifh_Cluster1289	0.0036	-1.00E-04	0.0002	-0.2498	0.9801	0.9838481	Organic
Water_content	nifh_Cluster129	0.0057	0.0035	0.0642	-0.1697	0.6281	0.8719664	Organic
Water_content	nifh_Cluster1292	0.0036	-0.0007	0.022	-0.2225	0.7791	0.9176966	Organic

Water_content	nifh_Cluster130	0.0029	0.0018	0.0642	-0.1697	0.6281	0.8719664	Organic
Water_content	nifh_Cluster1305	0.0047	0.0039	0.4576	0.3221	0.1401	0.4883233	Organic
Water_content	nifh_Cluster1306	0.0036	-0.0046	0.6409	0.5511	0.0557	0.3805486	Organic
Water_content	nifh_Cluster131	0.001	0.0006	0.0642	-0.1697	0.6281	0.8719664	Organic
Water_content	nifh_Cluster1319	0.0009	0.0005	0.0642	-0.1697	0.6281	0.8719664	Organic
Water_content	nifh_Cluster132	0.0064	0.0058	0.2701	0.0876	0.2906	0.7414139	Organic
Water_content	nifh_Cluster1320	0.0017	1.00E-04	0.0012	-0.2485	0.9489	0.9817257	Organic
Water_content	nifh_Cluster1323	0.0027	-0.0007	0.0563	-0.1796	0.6507	0.8719664	Organic
Water_content	nifh_Cluster1324	0.0463	0.0167	0.296	0.1199	0.2645	0.7021797	Organic
Water_content	nifh_Cluster1336	0.0138	0.0044	0.5629	0.4537	0.0858	0.4040371	Organic
Water_content	nifh_Cluster1340	0.0073	-0.0014	0.0359	-0.2051	0.7192	0.8916615	Organic
Water_content	nifh_Cluster1345	0.0863	0.0239	0.1974	-0.0032	0.3773	0.8719664	Organic
Water_content	nifh_Cluster1370	0.0018	-0.0016	0.3086	0.1358	0.2524	0.6875221	Organic
Water_content	nifh_Cluster1375	0.0019	-0.0005	0.029	-0.2138	0.747	0.8944593	Organic
Water_content	nifh_Cluster139	0.0049	0.005	0.6709	0.5886	0.0461	0.3805486	Organic
Water_content	nifh_Cluster140	0.0023	-0.0015	0.344	0.18	0.2211	0.6517478	Organic
Water_content	nifh_Cluster141	0.035	-0.0519	0.6246	0.5308	0.0613	0.3805486	Organic
Water_content	nifh_Cluster148	0.0009	-0.0017	0.5977	0.4971	0.0714	0.3805486	Organic
Water_content	nifh_Cluster1480	0.0019	0.0018	0.3888	0.236	0.1859	0.5786787	Organic
Water_content	nifh_Cluster152	0.0593	0.0078	0.363	0.2038	0.2056	0.6288238	Organic
Water_content	nifh_Cluster156	0.1158	0.0396	0.561	0.4513	0.0866	0.4042903	Organic
Water_content	nifh_Cluster1562	0.1168	0.035	0.2817	0.1022	0.2786	0.7212615	Organic
Water_content	nifh_Cluster158	0.0028	0.001	0.0596	-0.1755	0.641	0.8719664	Organic
Water_content	nifh_Cluster16	0.001	0.0006	0.0642	-0.1697	0.6281	0.8719664	Organic
Water_content	nifh_Cluster166	0.0191	0.0113	0.0668	-0.1665	0.6209	0.8719664	Organic
Water_content	nifh_Cluster205	0.0124	0.0042	0.0511	-0.1861	0.6666	0.8721895	Organic
Water_content	nifh_Cluster21	0.0018	-0.0034	0.5977	0.4971	0.0714	0.3805486	Organic
Water_content	nifh_Cluster222	0.0038	0.0024	0.0642	-0.1697	0.6281	0.8719664	Organic
Water_content	nifh_Cluster225	0.0136	-0.0002	0.0005	-0.2493	0.9656	0.981822	Organic
Water_content	nifh_Cluster230	0.0017	-0.0013	0.2499	0.0623	0.3126	0.7786386	Organic
Water_content	nifh_Cluster231	0.0029	0.0013	0.0722	-0.1598	0.6067	0.8719664	Organic
Water_content	nifh_Cluster236	0.0007	-0.0014	0.5977	0.4971	0.0714	0.3805486	Organic
Water_content	nifh_Cluster237	0.0029	0.0002	0.0026	-0.2467	0.9236	0.9777342	Organic
Water_content	nifh_Cluster243	0.0532	0.0113	0.1261	-0.0924	0.4898	0.8719664	Organic
Water_content	nifh_Cluster2432	0.0734	0.0261	0.5723	0.4654	0.0817	0.4031833	Organic
Water_content	nifh_Cluster2433	0.004	0.0042	0.5484	0.4355	0.0922	0.4084099	Organic
Water_content	nifh_Cluster246	0.0028	-0.0001	0.0018	-0.2478	0.937	0.9817257	Organic
Water_content	nifh_Cluster25	0.0544	0.0202	0.621	0.5263	0.0626	0.3805486	Organic
Water_content	nifh_Cluster256	0.0042	-0.0012	0.0582	-0.1773	0.6452	0.8719664	Organic
Water_content	nifh_Cluster264	0.004	0.0027	0.2908	0.1134	0.2696	0.7064556	Organic
Water_content	nifh_Cluster265	0.0823	0.0369	0.4849	0.3561	0.1243	0.4610662	Organic
Water_content	nifh_Cluster267	0.0206	0.0062	0.1145	-0.1069	0.5118	0.8719664	Organic
Water_content	nifh_Cluster268	0.0115	0.0012	0.0085	-0.2394	0.8625	0.9679667	Organic
Water_content	nifh_Cluster269	0.0097	-0.0004	0.0127	-0.2342	0.8319	0.9417321	Organic
Water_content	nifh_Cluster272	0.0114	0.0079	0.6847	0.6059	0.0421	0.3805486	Organic
Water_content	nifh_Cluster274	0.0076	0.0047	0.0642	-0.1697	0.6281	0.8719664	Organic
Water_content	nifh_Cluster275	0	0	#VALUE!	#VALUE!	#VALUE!	NA	Organic
Water_content	nifh_Cluster278	0.013	0.0021	0.0328	-0.2091	0.7315	0.8916615	Organic
Water_content	nifh_Cluster279	0.0067	0.0008	0.1117	-0.1104	0.5174	0.8719664	Organic
Water_content	nifh_Cluster28	0.0027	0.0002	0.0045	-0.2444	0.8994	0.9777342	Organic
Water_content	nifh_Cluster280	0.0024	-0.0036	0.7807	0.7258	0.0196	0.3805486	Organic
Water_content	nifh_Cluster282	0.0052	0.0014	0.0537	-0.1829	0.6588	0.8719664	Organic
Water_content	nifh_Cluster287	0	0	#VALUE!	#VALUE!	#VALUE!	NA	Organic
Water_content	nifh_Cluster290	0.0147	0.0033	0.2557	0.0696	0.3062	0.773548	Organic
Water_content	nifh_Cluster297	0	0	#VALUE!	#VALUE!	#VALUE!	NA	Organic

Water_content	nifh_Cluster299	0.0028	0.0017	0.0642	-0.1697	0.6281	0.8719664	Organic
Water_content	nifh_Cluster303	0.0021	0.0034	0.4623	0.3279	0.1372	0.4850007	Organic
Water_content	nifh_Cluster304	0.002	0.0007	0.0536	-0.183	0.6589	0.8719664	Organic
Water_content	nifh_Cluster313	0.0038	0.0019	0.0738	-0.1577	0.6025	0.8719664	Organic
Water_content	nifh_Cluster314	0.001	0.0001	0.0021	-0.2474	0.931	0.9777342	Organic
Water_content	nifh_Cluster317	0.0027	0.0004	0.0203	-0.2246	0.7876	0.9193944	Organic
Water_content	nifh_Cluster32	0.0043	-0.0025	0.1914	-0.0108	0.3857	0.8719664	Organic
Water_content	nifh_Cluster320	9.7369	-1.5496	0.5168	0.396	0.1074	0.4327967	Organic
Water_content	nifh_Cluster334	0.001	0.0006	0.0642	-0.1697	0.6281	0.8719664	Organic
Water_content	nifh_Cluster335	0.0114	0.0071	0.0642	-0.1697	0.6281	0.8719664	Organic
Water_content	nifh_Cluster338	0.0053	-0.0027	0.8462	0.8077	0.0094	0.3805486	Organic
Water_content	nifh_Cluster340	0.0143	0.0089	0.0642	-0.1697	0.6281	0.8719664	Organic
Water_content	nifh_Cluster341	0.0029	0.0004	0.0053	-0.2433	0.8908	0.9777342	Organic
Water_content	nifh_Cluster35	0.0076	-0.0065	0.489	0.3613	0.122	0.4559184	Organic
Water_content	nifh_Cluster351	0	0	#VALUE!	#VALUE!	#VALUE!	NA	Organic
Water_content	nifh_Cluster3574	0.085	-0.0477	0.8667	0.8334	0.007	0.3805486	Organic
Water_content	nifh_Cluster3691	0.0049	-0.007	0.6533	0.5666	0.0516	0.3805486	Organic
Water_content	nifh_Cluster37	0.0214	0.0014	0.0036	-0.2454	0.9095	0.9777342	Organic
Water_content	nifh_Cluster374	0	0	#VALUE!	#VALUE!	#VALUE!	NA	Organic
Water_content	nifh_Cluster38	0.001	0.0001	0.0021	-0.2474	0.931	0.9777342	Organic
Water_content	nifh_Cluster382	0	0	#VALUE!	#VALUE!	#VALUE!	NA	Organic
Water_content	nifh_Cluster384	0.001	0.0006	0.0642	-0.1697	0.6281	0.8719664	Organic
Water_content	nifh_Cluster386	0.0019	0.0012	0.0642	-0.1697	0.6281	0.8719664	Organic
Water_content	nifh_Cluster389	0.0031	0.0027	0.5931	0.4913	0.0732	0.3828675	Organic
Water_content	nifh_Cluster394	0	0	#VALUE!	#VALUE!	#VALUE!	NA	Organic
Water_content	nifh_Cluster40	0.0007	-0.0014	0.5977	0.4971	0.0714	0.3805486	Organic
Water_content	nifh_Cluster43	0.0111	0.0047	0.3034	0.1292	0.2574	0.6974156	Organic
Water_content	nifh_Cluster434	0	0	#VALUE!	#VALUE!	#VALUE!	NA	Organic
Water_content	nifh_Cluster440	0.0039	-0.0074	0.5977	0.4971	0.0714	0.3805486	Organic
Water_content	nifh_Cluster462	0	0	#VALUE!	#VALUE!	#VALUE!	NA	Organic
Water_content	nifh_Cluster470	0.0107	0.0048	0.3013	0.1267	0.2593	0.6989961	Organic
Water_content	nifh_Cluster49	0.019	0.011	0.0921	-0.1349	0.5587	0.8719664	Organic
Water_content	nifh_Cluster499	0.0019	0.0007	0.0599	-0.1751	0.6402	0.8719664	Organic
Water_content	nifh_Cluster5	0.0239	-0.0069	0.4209	0.2761	0.1634	0.5471125	Organic
Water_content	nifh_Cluster506	0.0022	0.0023	0.5791	0.4739	0.0789	0.3965355	Organic
Water_content	nifh_Cluster51	0.002	0.0007	0.0537	-0.1828	0.6585	0.8719664	Organic
Water_content	nifh_Cluster52	0.0187	0.006	0.0513	-0.1859	0.6661	0.8721895	Organic
Water_content	nifh_Cluster525	0.0054	-0.0103	0.5977	0.4971	0.0714	0.3805486	Organic
Water_content	nifh_Cluster531	0.058	0.0187	0.5458	0.4323	0.0934	0.4084099	Organic
Water_content	nifh_Cluster539	0.003	0.0008	0.0265	-0.2169	0.7581	0.8987941	Organic
Water_content	nifh_Cluster578	0.0202	-0.0112	0.4479	0.3099	0.146	0.5023342	Organic
Water_content	nifh_Cluster58	0.6425	0.1369	0.2031	0.0039	0.3698	0.8719664	Organic
Water_content	nifh_Cluster60	0.0039	0.0014	0.0931	-0.1336	0.5565	0.8719664	Organic
Water_content	nifh_Cluster61	0.0046	-0.002	0.1378	-0.0777	0.4687	0.8719664	Organic
Water_content	nifh_Cluster65	0.0104	0.0013	0.0055	-0.2431	0.8888	0.9777342	Organic
Water_content	nifh_Cluster686	0	0	#VALUE!	#VALUE!	#VALUE!	NA	Organic
Water_content	nifh_Cluster69	0.0085	-0.0025	0.0656	-0.1681	0.6243	0.8719664	Organic
Water_content	nifh_Cluster70	0.0029	0.0018	0.0642	-0.1697	0.6281	0.8719664	Organic
Water_content	nifh_Cluster717	0.0046	0.003	0.5909	0.4886	0.0741	0.3835939	Organic
Water_content	nifh_Cluster725	0.0118	0.0017	0.0374	-0.2033	0.7137	0.8916615	Organic
Water_content	nifh_Cluster727	0.0042	-0.0059	0.5314	0.4142	0.1002	0.422233	Organic
Water_content	nifh_Cluster73	0.0038	0.0009	0.0398	-0.2002	0.7046	0.8916615	Organic
Water_content	nifh_Cluster74	0.0048	0.003	0.0642	-0.1697	0.6281	0.8719664	Organic
Water_content	nifh_Cluster748	0.0023	0.0017	0.3225	0.1531	0.2398	0.6706009	Organic
Water_content	nifh_Cluster749	0.0111	-0.0002	0.0009	-0.2488	0.9545	0.9817257	Organic

Water_content	nifh_Cluster755	0.006	-0.0113	0.5977	0.4971	0.0714	0.3805486	Organic
Water_content	nifh_Cluster76	0.0019	-1.00E-04	0.0003	-0.2496	0.9724	0.981822	Organic
Water_content	nifh_Cluster79	0.0027	-0.0006	0.0184	-0.227	0.798	0.9221226	Organic
Water_content	nifh_Cluster81	0.0099	-0.0092	0.331	0.1637	0.2322	0.6626931	Organic
Water_content	nifh_Cluster82	0.0003	-0.0002	0.0702	-0.1622	0.6118	0.8719664	Organic
Water_content	nifh_Cluster83	0.0105	0.0026	0.0307	-0.2116	0.7399	0.8916615	Organic
Water_content	nifh_Cluster86	0.007	-0.0058	0.5262	0.4078	0.1028	0.4265151	Organic
Water_content	nifh_Cluster868	0.01	-0.0023	0.1171	-0.1036	0.5068	0.8719664	Organic
Water_content	nifh_Cluster87	0.001	0.0006	0.0642	-0.1697	0.6281	0.8719664	Organic
Water_content	nifh_Cluster88	0	0	#VALUE!	#VALUE!	#VALUE!	NA	Organic
Water_content	nifh_Cluster9	0.0083	0.0024	0.1424	-0.0721	0.4609	0.8719664	Organic
Water_content	nifh_Cluster92	0.001	0.0006	0.0642	-0.1697	0.6281	0.8719664	Organic
Water_content	nifh_Cluster93	0.0038	0.0019	0.0761	-0.1548	0.5966	0.8719664	Organic
Water_content	nifh_Cluster94	0.0038	0.0024	0.0642	-0.1697	0.6281	0.8719664	Organic
Water_content	nifh_Cluster95	0.005	0.0023	0.0668	-0.1665	0.621	0.8719664	Organic
Water_content	nifh_Cluster96	0.0104	-0.0015	0.0456	-0.193	0.6846	0.8856156	Organic
Water_content	nifh_Cluster97	0.0317	0.0245	0.4926	0.3657	0.1201	0.4551504	Organic
Water_content	nifh_Cluster98	0.0084	-0.0015	0.0375	-0.2031	0.7131	0.8916615	Organic
Water_content	nifh_Cluster99	0.0028	0.0013	0.0729	-0.1589	0.6048	0.8719664	Organic
Water_content	nirk_Cluster0	4.2317	-1.2369	0.9565	0.9457	0.0007	0.37596	Organic
Water_content	nirk_Cluster1	0.0715	-0.1063	0.6189	0.5237	0.0634	0.3805486	Organic
Water_content	nirk_Cluster10	0.3374	-0.066	0.2902	0.1127	0.2702	0.7064556	Organic
Water_content	nirk_Cluster101	0.001	0.0006	0.0642	-0.1697	0.6281	0.8719664	Organic
Water_content	nirk_Cluster102	0.0115	-0.0019	0.021	-0.2238	0.7843	0.9193944	Organic
Water_content	nirk_Cluster103	0.0388	-0.0484	0.6661	0.5826	0.0476	0.3805486	Organic
Water_content	nirk_Cluster104	0.0009	-0.0017	0.5977	0.4971	0.0714	0.3805486	Organic
Water_content	nirk_Cluster105	0.0379	-0.0322	0.7464	0.683	0.0265	0.3805486	Organic
Water_content	nirk_Cluster106	0.0248	-0.021	0.8324	0.7905	0.0112	0.3805486	Organic
Water_content	nirk_Cluster107	0.0313	-0.0218	0.747	0.6838	0.0264	0.3805486	Organic
Water_content	nirk_Cluster108	0.0068	0.0064	0.2987	0.1233	0.2619	0.7021797	Organic
Water_content	nirk_Cluster109	0.0007	-0.0014	0.5977	0.4971	0.0714	0.3805486	Organic
Water_content	nirk_Cluster11	0.1133	0.0065	0.0499	-0.1876	0.6704	0.8721895	Organic
Water_content	nirk_Cluster12	0.0661	-0.0263	0.3977	0.2471	0.1795	0.5770643	Organic
Water_content	nirk_Cluster13	0.0382	-0.0053	0.1121	-0.1099	0.5166	0.8719664	Organic
Water_content	nirk_Cluster14	0.06	-0.0357	0.3468	0.1834	0.2188	0.6517478	Organic
Water_content	nirk_Cluster15	0.1402	-0.1304	0.6379	0.5473	0.0567	0.3805486	Organic
Water_content	nirk_Cluster16	0.2004	-0.1692	0.6895	0.6119	0.0407	0.3805486	Organic
Water_content	nirk_Cluster17	0.0882	0.0008	0.0004	-0.2495	0.97	0.981822	Organic
Water_content	nirk_Cluster18	0.0885	-0.0119	0.0205	-0.2244	0.7868	0.9193944	Organic
Water_content	nirk_Cluster19	0.2332	-0.0327	0.1848	-0.019	0.3949	0.8719664	Organic
Water_content	nirk_Cluster2	0.2103	-0.0438	0.3179	0.1474	0.2439	0.6784409	Organic
Water_content	nirk_Cluster20	0.1247	0.0247	0.039	-0.2012	0.7076	0.8916615	Organic
Water_content	nirk_Cluster21	0.1085	-0.0069	0.0183	-0.2271	0.7984	0.9221226	Organic
Water_content	nirk_Cluster22	0.0844	-0.026	0.6568	0.571	0.0505	0.3805486	Organic
Water_content	nirk_Cluster23	0.0533	-0.0009	0.0032	-0.246	0.9151	0.9777342	Organic
Water_content	nirk_Cluster24	0.0405	-0.0276	0.3917	0.2396	0.1838	0.5786787	Organic
Water_content	nirk_Cluster25	0.2941	-0.1958	0.5445	0.4306	0.0941	0.4084099	Organic
Water_content	nirk_Cluster26	0.0877	-0.0236	0.2803	0.1004	0.28	0.7214988	Organic
Water_content	nirk_Cluster27	0.0878	-0.0463	0.7078	0.6347	0.0358	0.3805486	Organic
Water_content	nirk_Cluster28	0.0375	0.0037	0.0283	-0.2146	0.75	0.8955526	Organic
Water_content	nirk_Cluster29	0.1289	-0.0038	0.0075	-0.2406	0.8705	0.9740331	Organic
Water_content	nirk_Cluster3	0.2014	-0.0951	0.6526	0.5657	0.0519	0.3805486	Organic
Water_content	nirk_Cluster30	0.0164	-0.0164	0.5474	0.4342	0.0927	0.4084099	Organic
Water_content	nirk_Cluster31	0.1697	0.0223	0.482	0.3525	0.1259	0.4637152	Organic
Water_content	nirk_Cluster32	0.0468	-0.0462	0.4936	0.367	0.1195	0.4551504	Organic

Water_content	nirk_Cluster33	0.0203	-0.0145	0.5407	0.4259	0.0958	0.4084099	Organic
Water_content	nirk_Cluster34	0.0301	0.0042	0.0307	-0.2116	0.7397	0.8916615	Organic
Water_content	nirk_Cluster35	0.032	-0.0211	0.4218	0.2772	0.1628	0.5471125	Organic
Water_content	nirk_Cluster36	0.0009	-0.0017	0.5977	0.4971	0.0714	0.3805486	Organic
Water_content	nirk_Cluster39	0.001	0.0006	0.0642	-0.1697	0.6281	0.8719664	Organic
Water_content	nirk_Cluster4	0.0827	-0.0043	0.0183	-0.2272	0.7985	0.9221226	Organic
Water_content	nirk_Cluster40	0.0232	-0.0112	0.3385	0.1731	0.2258	0.6560043	Organic
Water_content	nirk_Cluster41	0.0976	-0.0417	0.4271	0.2839	0.1593	0.5409333	Organic
Water_content	nirk_Cluster44	0.0451	-0.0639	0.5984	0.498	0.0711	0.3805486	Organic
Water_content	nirk_Cluster46	0.0055	-0.0079	0.6896	0.612	0.0407	0.3805486	Organic
Water_content	nirk_Cluster48	0.0028	-0.0034	0.5165	0.3956	0.1076	0.4327967	Organic
Water_content	nirk_Cluster49	0.0019	-0.0011	0.1565	-0.0544	0.4376	0.8719664	Organic
Water_content	nirk_Cluster5	0.1328	-0.0076	0.0325	-0.2093	0.7324	0.8916615	Organic
Water_content	nirk_Cluster50	0.0019	-1.00E-04	0.0003	-0.2496	0.9724	0.981822	Organic
Water_content	nirk_Cluster52	0.0205	-0.0152	0.5551	0.4439	0.0892	0.4084099	Organic
Water_content	nirk_Cluster53	0.0037	-0.0022	0.1437	-0.0704	0.4586	0.8719664	Organic
Water_content	nirk_Cluster54	0.001	0.0001	0.0021	-0.2474	0.931	0.9777342	Organic
Water_content	nirk_Cluster55	0.001	0.0006	0.0642	-0.1697	0.6281	0.8719664	Organic
Water_content	nirk_Cluster56	0.0008	-0.0005	0.0702	-0.1622	0.6118	0.8719664	Organic
Water_content	nirk_Cluster57	0.0012	0.002	0.4623	0.3279	0.1372	0.4850007	Organic
Water_content	nirk_Cluster58	0.0053	-0.0032	0.2506	0.0632	0.3118	0.7786386	Organic
Water_content	nirk_Cluster59	0.0028	-0.0006	0.0136	-0.233	0.8257	0.9388201	Organic
Water_content	nirk_Cluster6	0.117	-0.0619	0.6515	0.5644	0.0522	0.3805486	Organic
Water_content	nirk_Cluster60	0.0008	0.0005	0.0642	-0.1697	0.6281	0.8719664	Organic
Water_content	nirk_Cluster62	0.0486	-0.0611	0.6766	0.5957	0.0444	0.3805486	Organic
Water_content	nirk_Cluster63	0.0526	-0.0841	0.6284	0.5355	0.06	0.3805486	Organic
Water_content	nirk_Cluster64	0.0045	-0.0005	0.0129	-0.2338	0.83	0.9416749	Organic
Water_content	nirk_Cluster65	0.0296	-0.0133	0.1716	-0.0354	0.4141	0.8719664	Organic
Water_content	nirk_Cluster66	0	0	#VALUE!	#VALUE!	#VALUE!	NA	Organic
Water_content	nirk_Cluster67	0.0028	-0.0027	0.4328	0.2911	0.1555	0.5316305	Organic
Water_content	nirk_Cluster68	0.0044	-0.0083	0.5977	0.4971	0.0714	0.3805486	Organic
Water_content	nirk_Cluster69	0.0164	-0.0042	0.0573	-0.1784	0.6478	0.8719664	Organic
Water_content	nirk_Cluster7	0.0414	-0.0179	0.3344	0.1679	0.2293	0.662615	Organic
Water_content	nirk_Cluster70	0.0293	-0.0417	0.6401	0.5501	0.056	0.3805486	Organic
Water_content	nirk_Cluster71	0.0009	0.0015	0.4623	0.3279	0.1372	0.4850007	Organic
Water_content	nirk_Cluster72	0.0144	-0.0126	0.4903	0.3629	0.1213	0.4559184	Organic
Water_content	nirk_Cluster73	0.0187	-0.0298	0.7554	0.6943	0.0246	0.3805486	Organic
Water_content	nirk_Cluster74	0.2364	-0.3982	0.6221	0.5277	0.0622	0.3805486	Organic
Water_content	nirk_Cluster75	0.005	-0.0094	0.5977	0.4971	0.0714	0.3805486	Organic
Water_content	nirk_Cluster76	0.025	-0.0274	0.6719	0.5898	0.0458	0.3805486	Organic
Water_content	nirk_Cluster77	0	0	#VALUE!	#VALUE!	#VALUE!	NA	Organic
Water_content	nirk_Cluster78	0.0612	-0.0705	0.697	0.6213	0.0386	0.3805486	Organic
Water_content	nirk_Cluster79	0	0	#VALUE!	#VALUE!	#VALUE!	NA	Organic
Water_content	nirk_Cluster8	0.048	-0.0133	0.1522	-0.0597	0.4444	0.8719664	Organic
Water_content	nirk_Cluster80	0.0466	-0.0477	0.6405	0.5506	0.0558	0.3805486	Organic
Water_content	nirk_Cluster82	0	0	#VALUE!	#VALUE!	#VALUE!	NA	Organic
Water_content	nirk_Cluster83	0.0027	-0.0002	0.0027	-0.2467	0.9228	0.9777342	Organic
Water_content	nirk_Cluster84	0.0138	0.0004	0.0004	-0.2495	0.9701	0.981822	Organic
Water_content	nirk_Cluster85	0.0005	-0.0003	0.0702	-0.1622	0.6118	0.8719664	Organic
Water_content	nirk_Cluster86	0.0121	-0.0205	0.6231	0.5288	0.0619	0.3805486	Organic
Water_content	nirk_Cluster87	0.0057	-0.0086	0.5523	0.4403	0.0905	0.4084099	Organic
Water_content	nirk_Cluster88	0.0025	-0.0011	0.0781	-0.1524	0.5918	0.8719664	Organic
Water_content	nirk_Cluster89	0.001	-0.0006	0.0702	-0.1622	0.6118	0.8719664	Organic
Water_content	nirk_Cluster9	0.0285	0.0012	0.0013	-0.2484	0.9456	0.9817257	Organic
Water_content	nirk_Cluster90	0.0017	-0.0021	0.5674	0.4593	0.0838	0.4035856	Organic

Water_content	nirk_Cluster91	0.0208	-0.0161	0.3227	0.1534	0.2395	0.6706009	Organic
Water_content	nirk_Cluster92	0.0025	-0.0022	0.3266	0.1582	0.2361	0.6674677	Organic
Water_content	nirk_Cluster93	0.0185	0.0057	0.413	0.2662	0.1687	0.558554	Organic
Water_content	nirk_Cluster94	0.0056	-0.001	0.0121	-0.2349	0.8359	0.944231	Organic
Water_content	nirk_Cluster95	0.0238	-0.022	0.3961	0.2451	0.1806	0.5770643	Organic
Water_content	nirk_Cluster96	0.0181	-0.0134	0.4076	0.2596	0.1724	0.5671803	Organic
Water_content	nirk_Cluster97	0.0248	-0.0146	0.747	0.6838	0.0264	0.3805486	Organic
Water_content	nirk_Cluster98	0.007	-0.0093	0.6984	0.623	0.0383	0.3805486	Organic
Water_content	nirk_Cluster99	0.0027	-0.0047	0.6641	0.5801	0.0482	0.3805486	Organic
Water_content	nirs_Cluster0	0.0968	-0.0529	0.6583	0.5728	0.05	0.3805486	Organic
Water_content	nirs_Cluster1	0.0013	-0.0008	0.0702	-0.1622	0.6118	0.8719664	Organic
Water_content	nirs_Cluster14	0.0019	0.0012	0.0642	-0.1697	0.6281	0.8719664	Organic
Water_content	nirs_Cluster2	0.005	-0.0043	0.3099	0.1373	0.2513	0.6875221	Organic
Water_content	nirs_Cluster26	0	0	#VALUE!	#VALUE!	#VALUE!	NA	Organic
Water_content	nirs_Cluster28	0	0	#VALUE!	#VALUE!	#VALUE!	NA	Organic
Water_content	nirs_Cluster3	0.1217	-0.0348	0.5433	0.4291	0.0946	0.4084099	Organic
Water_content	nirs_Cluster36	0	0	#VALUE!	#VALUE!	#VALUE!	NA	Organic
Water_content	nirs_Cluster37	0	0	#VALUE!	#VALUE!	#VALUE!	NA	Organic
Water_content	nirs_Cluster4	0	0	#VALUE!	#VALUE!	#VALUE!	NA	Organic
Water_content	nirs_Cluster50	0.001	0.0003	0.0276	-0.2155	0.753	0.8970295	Organic
Water_content	nirs_Cluster9	0	0	#VALUE!	#VALUE!	#VALUE!	NA	Organic
Water_content	norb_Cluster0	0.0592	-0.0462	0.624	0.53	0.0616	0.3805486	Organic
Water_content	norb_Cluster1	0.6783	-0.6258	0.6349	0.5436	0.0577	0.3805486	Organic
Water_content	norb_Cluster101	0	0	#VALUE!	#VALUE!	#VALUE!	NA	Organic
Water_content	norb_Cluster102	0.0009	-0.0017	0.5977	0.4971	0.0714	0.3805486	Organic
Water_content	norb_Cluster103	0	0	#VALUE!	#VALUE!	#VALUE!	NA	Organic
Water_content	norb_Cluster104	0.0018	-0.0011	0.1437	-0.0704	0.4586	0.8719664	Organic
Water_content	norb_Cluster106	0.0705	-0.003	0.0009	-0.2488	0.954	0.9817257	Organic
Water_content	norb_Cluster107	0.0056	-0.0021	0.1417	-0.0729	0.462	0.8719664	Organic
Water_content	norb_Cluster108	0.0009	-0.0017	0.5977	0.4971	0.0714	0.3805486	Organic
Water_content	norb_Cluster109	0.0091	0.0007	0.0053	-0.2434	0.891	0.9777342	Organic
Water_content	norb_Cluster11	0.0053	-0.0053	0.3683	0.2103	0.2015	0.6234465	Organic
Water_content	norb_Cluster110	0.0027	-0.0021	0.5062	0.3828	0.1128	0.4437038	Organic
Water_content	norb_Cluster112	0.006	0.0034	0.0591	-0.1761	0.6424	0.8719664	Organic
Water_content	norb_Cluster113	0.0076	0.0047	0.0642	-0.1697	0.6281	0.8719664	Organic
Water_content	norb_Cluster114	0	0	#VALUE!	#VALUE!	#VALUE!	NA	Organic
Water_content	norb_Cluster115	0.0394	0.0207	0.0504	-0.187	0.669	0.8721895	Organic
Water_content	norb_Cluster116	0.0046	0.0028	0.0642	-0.1697	0.6281	0.8719664	Organic
Water_content	norb_Cluster117	0.001	0.0006	0.0642	-0.1697	0.6281	0.8719664	Organic
Water_content	norb_Cluster118	0.0019	-0.0023	0.6303	0.5379	0.0593	0.3805486	Organic
Water_content	norb_Cluster119	0.001	0.0006	0.0642	-0.1697	0.6281	0.8719664	Organic
Water_content	norb_Cluster12	0.0039	-0.0047	0.6244	0.5306	0.0614	0.3805486	Organic
Water_content	norb_Cluster121	0	0	#VALUE!	#VALUE!	#VALUE!	NA	Organic
Water_content	norb_Cluster122	0.0044	0.0017	0.104	-0.12	0.533	0.8719664	Organic
Water_content	norb_Cluster123	0.001	0.0006	0.0642	-0.1697	0.6281	0.8719664	Organic
Water_content	norb_Cluster124	0.524	-0.153	0.1325	-0.0844	0.4782	0.8719664	Organic
Water_content	norb_Cluster125	0.001	0.0006	0.0642	-0.1697	0.6281	0.8719664	Organic
Water_content	norb_Cluster126	0.0012	0.0004	0.0336	-0.208	0.7281	0.8916615	Organic
Water_content	norb_Cluster127	0.0969	-0.0494	0.3299	0.1624	0.2331	0.6626931	Organic
Water_content	norb_Cluster128	0.0095	-0.0079	0.6891	0.6113	0.0409	0.3805486	Organic
Water_content	norb_Cluster13	0.003	-0.0038	0.4778	0.3472	0.1283	0.4692273	Organic
Water_content	norb_Cluster131	0.0121	0.0034	0.0319	-0.2101	0.7349	0.8916615	Organic
Water_content	norb_Cluster132	0.4322	-0.1821	0.1985	-0.0019	0.3759	0.8719664	Organic
Water_content	norb_Cluster133	0.0007	-0.0014	0.5977	0.4971	0.0714	0.3805486	Organic
Water_content	norb_Cluster135	0.0227	-0.0064	0.0557	-0.1804	0.6527	0.8719664	Organic

Water_content	norb_Cluster136	0.012	-0.0074	0.5029	0.3786	0.1146	0.4472531	Organic
Water_content	norb_Cluster139	0.0171	-0.0099	0.4031	0.2539	0.1756	0.5740574	Organic
Water_content	norb_Cluster14	0	0	#VALUE!	#VALUE!	#VALUE!	NA	Organic
Water_content	norb_Cluster140	0.0318	-0.0411	0.676	0.595	0.0446	0.3805486	Organic
Water_content	norb_Cluster142	0.0009	-0.0017	0.5977	0.4971	0.0714	0.3805486	Organic
Water_content	norb_Cluster144	0.0038	0.0024	0.0642	-0.1697	0.6281	0.8719664	Organic
Water_content	norb_Cluster146	0.0174	-0.0093	0.1479	-0.0652	0.4516	0.8719664	Organic
Water_content	norb_Cluster15	0.0387	-0.0282	0.3897	0.2371	0.1852	0.5786787	Organic
Water_content	norb_Cluster150	0.0052	-0.0065	0.6446	0.5558	0.0544	0.3805486	Organic
Water_content	norb_Cluster155	0.1387	-0.0522	0.1649	-0.0438	0.4243	0.8719664	Organic
Water_content	norb_Cluster16	0.0045	-0.0022	0.4601	0.3251	0.1386	0.4864047	Organic
Water_content	norb_Cluster164	0.0091	-0.0124	0.517	0.3962	0.1073	0.4327967	Organic
Water_content	norb_Cluster165	0.0036	0.0004	0.0026	-0.2467	0.9234	0.9777342	Organic
Water_content	norb_Cluster166	0.0024	0.0008	0.0336	-0.208	0.7281	0.8916615	Organic
Water_content	norb_Cluster169	0.0062	-0.0047	0.2286	0.0357	0.3375	0.8326022	Organic
Water_content	norb_Cluster17	0.0009	-0.0017	0.5977	0.4971	0.0714	0.3805486	Organic
Water_content	norb_Cluster170	0.0104	0.0042	0.033	-0.2088	0.7306	0.8916615	Organic
Water_content	norb_Cluster171	0.0019	0.0012	0.0642	-0.1697	0.6281	0.8719664	Organic
Water_content	norb_Cluster172	0.0043	-0.0033	0.2468	0.0586	0.3161	0.7834195	Organic
Water_content	norb_Cluster175	0.0115	-0.0053	0.0865	-0.1418	0.5715	0.8719664	Organic
Water_content	norb_Cluster176	0.0065	-0.0028	0.0843	-0.1447	0.5768	0.8719664	Organic
Water_content	norb_Cluster177	0.0084	0.0033	0.0324	-0.2096	0.7331	0.8916615	Organic
Water_content	norb_Cluster179	0	0	#VALUE!	#VALUE!	#VALUE!	NA	Organic
Water_content	norb_Cluster18	0.0009	-0.0017	0.5977	0.4971	0.0714	0.3805486	Organic
Water_content	norb_Cluster180	0.0051	-0.0027	0.1939	-0.0076	0.3822	0.8719664	Organic
Water_content	norb_Cluster181	0.0019	0.0012	0.0642	-0.1697	0.6281	0.8719664	Organic
Water_content	norb_Cluster182	0	0	#VALUE!	#VALUE!	#VALUE!	NA	Organic
Water_content	norb_Cluster184	0.0073	-0.0022	0.0353	-0.2058	0.7214	0.8916615	Organic
Water_content	norb_Cluster186	0.001	0.0006	0.0642	-0.1697	0.6281	0.8719664	Organic
Water_content	norb_Cluster19	0.0028	-0.0005	0.0106	-0.2368	0.8462	0.9537546	Organic
Water_content	norb_Cluster192	0.0054	-0.0031	0.1324	-0.0845	0.4783	0.8719664	Organic
Water_content	norb_Cluster193	0.0019	0.0012	0.0642	-0.1697	0.6281	0.8719664	Organic
Water_content	norb_Cluster197	0.0065	0.0021	0.0239	-0.2201	0.77	0.9091021	Organic
Water_content	norb_Cluster2	0.003	0.0031	0.4196	0.2745	0.1642	0.5471125	Organic
Water_content	norb_Cluster20	0.0394	-0.0254	0.5172	0.3965	0.1072	0.4327967	Organic
Water_content	norb_Cluster208	0	0	#VALUE!	#VALUE!	#VALUE!	NA	Organic
Water_content	norb_Cluster21	0.0329	-0.0392	0.6529	0.5661	0.0518	0.3805486	Organic
Water_content	norb_Cluster211	0.001	0.0006	0.0642	-0.1697	0.6281	0.8719664	Organic
Water_content	norb_Cluster212	0.0295	0.0183	0.0642	-0.1697	0.6281	0.8719664	Organic
Water_content	norb_Cluster214	0.001	0.0006	0.0642	-0.1697	0.6281	0.8719664	Organic
Water_content	norb_Cluster217	0.0057	0.0035	0.0642	-0.1697	0.6281	0.8719664	Organic
Water_content	norb_Cluster219	0.0019	0.0012	0.0642	-0.1697	0.6281	0.8719664	Organic
Water_content	norb_Cluster22	0.0017	-0.0032	0.5977	0.4971	0.0714	0.3805486	Organic
Water_content	norb_Cluster220	0	0	#VALUE!	#VALUE!	#VALUE!	NA	Organic
Water_content	norb_Cluster221	0	0	#VALUE!	#VALUE!	#VALUE!	NA	Organic
Water_content	norb_Cluster222	0	0	#VALUE!	#VALUE!	#VALUE!	NA	Organic
Water_content	norb_Cluster224	0.0086	0.0053	0.0642	-0.1697	0.6281	0.8719664	Organic
Water_content	norb_Cluster225	0.0104	0.0064	0.0642	-0.1697	0.6281	0.8719664	Organic
Water_content	norb_Cluster226	0.0029	0.0018	0.0642	-0.1697	0.6281	0.8719664	Organic
Water_content	norb_Cluster228	0.0086	0.0053	0.0642	-0.1697	0.6281	0.8719664	Organic
Water_content	norb_Cluster229	0.0019	0.0012	0.0642	-0.1697	0.6281	0.8719664	Organic
Water_content	norb_Cluster230	0	0	#VALUE!	#VALUE!	#VALUE!	NA	Organic
Water_content	norb_Cluster231	0	0	#VALUE!	#VALUE!	#VALUE!	NA	Organic
Water_content	norb_Cluster232	0	0	#VALUE!	#VALUE!	#VALUE!	NA	Organic
Water_content	norb_Cluster234	0	0	#VALUE!	#VALUE!	#VALUE!	NA	Organic

Water_content	norb_Cluster235	0	0	#VALUE!	#VALUE!	#VALUE!	NA	Organic
Water_content	norb_Cluster238	0.001	0.0001	0.0034	-0.2457	0.9121	0.9777342	Organic
Water_content	norb_Cluster239	0.001	-0.0006	0.0702	-0.1622	0.6118	0.8719664	Organic
Water_content	norb_Cluster24	0.1693	-0.1568	0.596	0.495	0.072	0.3805486	Organic
Water_content	norb_Cluster244	0	0	#VALUE!	#VALUE!	#VALUE!	NA	Organic
Water_content	norb_Cluster247	0.0017	-0.0008	0.0971	-0.1286	0.5476	0.8719664	Organic
Water_content	norb_Cluster25	0.0743	-0.0624	0.6597	0.5747	0.0496	0.3805486	Organic
Water_content	norb_Cluster250	0.0038	0.0024	0.0642	-0.1697	0.6281	0.8719664	Organic
Water_content	norb_Cluster251	0.002	0.0007	0.0537	-0.1828	0.6585	0.8719664	Organic
Water_content	norb_Cluster26	0.0426	-0.056	0.6009	0.5011	0.0701	0.3805486	Organic
Water_content	norb_Cluster28	0.0171	0.0077	0.071	-0.1612	0.6097	0.8719664	Organic
Water_content	norb_Cluster29	0.1633	-0.0425	0.1806	-0.0242	0.4009	0.8719664	Organic
Water_content	norb_Cluster31	0.0281	-0.0351	0.6686	0.5857	0.0468	0.3805486	Organic
Water_content	norb_Cluster32	0.0675	-0.085	0.6943	0.6179	0.0394	0.3805486	Organic
Water_content	norb_Cluster33	0.6461	-0.0076	0.0023	-0.2472	0.9287	0.9777342	Organic
Water_content	norb_Cluster35	0.0334	-0.0337	0.6394	0.5492	0.0562	0.3805486	Organic
Water_content	norb_Cluster36	1.2204	-0.5423	0.6643	0.5804	0.0481	0.3805486	Organic
Water_content	norb_Cluster37	0.8221	-0.286	0.7357	0.6697	0.0289	0.3805486	Organic
Water_content	norb_Cluster38	0.0063	-0.0031	0.1978	-0.0027	0.3768	0.8719664	Organic
Water_content	norb_Cluster39	0.0328	-0.0351	0.5402	0.4252	0.0961	0.4084099	Organic
Water_content	norb_Cluster40	0.0977	-0.0624	0.3424	0.178	0.2225	0.6517478	Organic
Water_content	norb_Cluster42	0.2962	-0.1077	0.78	0.725	0.0197	0.3805486	Organic
Water_content	norb_Cluster44	0.0015	-0.0028	0.5977	0.4971	0.0714	0.3805486	Organic
Water_content	norb_Cluster46	0.2508	0.0373	0.032	-0.21	0.7345	0.8916615	Organic
Water_content	norb_Cluster47	0.3292	-0.2061	0.5837	0.4797	0.077	0.390774	Organic
Water_content	norb_Cluster5	0.0063	-0.0013	0.0907	-0.1367	0.562	0.8719664	Organic
Water_content	norb_Cluster51	0.0016	-0.0013	0.2666	0.0832	0.2944	0.7473357	Organic
Water_content	norb_Cluster52	0.1345	-0.1039	0.6094	0.5118	0.0669	0.3805486	Organic
Water_content	norb_Cluster56	0.0045	-0.0025	0.2844	0.1055	0.2759	0.7178389	Organic
Water_content	norb_Cluster57	0.0019	0.0012	0.0642	-0.1697	0.6281	0.8719664	Organic
Water_content	norb_Cluster59	0.002	-0.001	0.1556	-0.0555	0.439	0.8719664	Organic
Water_content	norb_Cluster6	0.6681	-0.3068	0.6334	0.5417	0.0583	0.3805486	Organic
Water_content	norb_Cluster60	0.2572	-0.0397	0.0177	-0.2278	0.8014	0.9232337	Organic
Water_content	norb_Cluster61	0.3154	0.012	0.0008	-0.249	0.9573	0.9817257	Organic
Water_content	norb_Cluster62	0.001	0.0006	0.0642	-0.1697	0.6281	0.8719664	Organic
Water_content	norb_Cluster64	0.0123	-0.0022	0.0146	-0.2317	0.8195	0.9388201	Organic
Water_content	norb_Cluster66	0	0	#VALUE!	#VALUE!	#VALUE!	NA	Organic
Water_content	norb_Cluster67	0.0065	-0.0056	0.493	0.3662	0.1199	0.4551504	Organic
Water_content	norb_Cluster68	0.0044	-0.0006	0.032	-0.21	0.7347	0.8916615	Organic
Water_content	norb_Cluster69	0.0018	-0.0034	0.5977	0.4971	0.0714	0.3805486	Organic
Water_content	norb_Cluster7	0.0518	-0.0146	0.1855	-0.0182	0.394	0.8719664	Organic
Water_content	norb_Cluster70	0.0526	-0.0125	0.0261	-0.2173	0.7596	0.8987941	Organic
Water_content	norb_Cluster71	0.0753	-0.0005	0.00E+00	-0.25	0.9923	0.9922852	Organic
Water_content	norb_Cluster74	0.0178	-0.0065	0.0605	-0.1744	0.6386	0.8719664	Organic
Water_content	norb_Cluster75	0.0691	-0.0228	0.0872	-0.1411	0.57	0.8719664	Organic
Water_content	norb_Cluster76	0.0831	-0.0155	0.0182	-0.2272	0.7987	0.9221226	Organic
Water_content	norb_Cluster77	0.0432	-0.0077	0.0575	-0.1782	0.6473	0.8719664	Organic
Water_content	norb_Cluster8	0.0116	0.0006	0.0011	-0.2487	0.9514	0.9817257	Organic
Water_content	norb_Cluster80	0	0	#VALUE!	#VALUE!	#VALUE!	NA	Organic
Water_content	norb_Cluster81	0.001	0.0006	0.0642	-0.1697	0.6281	0.8719664	Organic
Water_content	norb_Cluster83	0	0	#VALUE!	#VALUE!	#VALUE!	NA	Organic
Water_content	norb_Cluster84	0.0054	-0.0028	0.3098	0.1372	0.2513	0.6875221	Organic
Water_content	norb_Cluster85	0.0018	-0.0011	0.1437	-0.0704	0.4586	0.8719664	Organic
Water_content	norb_Cluster86	0.0061	0.0005	0.0023	-0.2471	0.928	0.9777342	Organic
Water_content	norb_Cluster88	0.001	0.0006	0.0642	-0.1697	0.6281	0.8719664	Organic

Water_content	norb_Cluster90	0	0	#VALUE!	#VALUE!	#VALUE!	NA	Organic
Water_content	norb_Cluster91	0.3756	-0.101	0.0551	-0.1812	0.6545	0.8719664	Organic
Water_content	norb_Cluster92	0	0	#VALUE!	#VALUE!	#VALUE!	NA	Organic
Water_content	norb_Cluster93	0.001	0.0006	0.0642	-0.1697	0.6281	0.8719664	Organic
Water_content	norb_Cluster94	0.0009	0.0001	0.0021	-0.2474	0.931	0.9777342	Organic
Water_content	norb_Cluster96	0.001	0.0006	0.0642	-0.1697	0.6281	0.8719664	Organic
Water_content	norb_Cluster97	0.0012	-0.0019	0.7451	0.6814	0.0268	0.3805486	Organic
Water_content	norb_Cluster98	0.0022	0.0005	0.0339	-0.2077	0.7271	0.8916615	Organic
Water_content	nosz_Cluster0	0.0017	1.00E-04	0.0012	-0.2485	0.9489	0.9817257	Organic
Water_content	nosz_Cluster10	0.0156	0.0127	0.2201	0.0252	0.3479	0.8501985	Organic
Water_content	nosz_Cluster102	0.001	0.0006	0.0642	-0.1697	0.6281	0.8719664	Organic
Water_content	nosz_Cluster103	0.0355	-0.0068	0.1088	-0.114	0.5232	0.8719664	Organic
Water_content	nosz_Cluster105	0.0017	-0.0013	0.2499	0.0623	0.3126	0.7786386	Organic
Water_content	nosz_Cluster106	0.0036	-0.0031	0.5667	0.4584	0.0841	0.4035856	Organic
Water_content	nosz_Cluster107	0	0	#VALUE!	#VALUE!	#VALUE!	NA	Organic
Water_content	nosz_Cluster108	0.0033	0.0009	0.0413	-0.1984	0.6994	0.8916615	Organic
Water_content	nosz_Cluster109	0.0272	-0.0074	0.0929	-0.1339	0.557	0.8719664	Organic
Water_content	nosz_Cluster11	0.019	0.0118	0.0642	-0.1697	0.6281	0.8719664	Organic
Water_content	nosz_Cluster110	0	0	#VALUE!	#VALUE!	#VALUE!	NA	Organic
Water_content	nosz_Cluster111	0.0019	0.0002	0.0021	-0.2474	0.931	0.9777342	Organic
Water_content	nosz_Cluster116	0.0048	0.003	0.0642	-0.1697	0.6281	0.8719664	Organic
Water_content	nosz_Cluster118	0	0	#VALUE!	#VALUE!	#VALUE!	NA	Organic
Water_content	nosz_Cluster119	0	0	#VALUE!	#VALUE!	#VALUE!	NA	Organic
Water_content	nosz_Cluster120	0.0039	0.001	0.0452	-0.1935	0.6858	0.8856156	Organic
Water_content	nosz_Cluster123	0	0	#VALUE!	#VALUE!	#VALUE!	NA	Organic
Water_content	nosz_Cluster128	0.0038	0.0024	0.0642	-0.1697	0.6281	0.8719664	Organic
Water_content	nosz_Cluster129	0.0478	0.0118	0.3516	0.1895	0.2148	0.6491637	Organic
Water_content	nosz_Cluster13	0.0093	-0.0009	0.0028	-0.2464	0.9201	0.9777342	Organic
Water_content	nosz_Cluster130	0.0272	0.0054	0.0911	-0.1362	0.5611	0.8719664	Organic
Water_content	nosz_Cluster132	0.0031	-0.0011	0.1532	-0.0585	0.4428	0.8719664	Organic
Water_content	nosz_Cluster14	0.0467	0.0284	0.0653	-0.1684	0.6251	0.8719664	Organic
Water_content	nosz_Cluster16	0.0067	0.0041	0.0642	-0.1697	0.6281	0.8719664	Organic
Water_content	nosz_Cluster17	0.0207	-0.0005	0.0008	-0.249	0.9573	0.9817257	Organic
Water_content	nosz_Cluster18	0.0105	0.0065	0.0642	-0.1697	0.6281	0.8719664	Organic
Water_content	nosz_Cluster20	0.0026	-0.0007	0.0619	-0.1726	0.6345	0.8719664	Organic
Water_content	nosz_Cluster21	0	0	#VALUE!	#VALUE!	#VALUE!	NA	Organic
Water_content	nosz_Cluster22	0.0123	0.0026	0.0137	-0.2329	0.8254	0.9388201	Organic
Water_content	nosz_Cluster23	0.0017	-0.0008	0.0971	-0.1286	0.5476	0.8719664	Organic
Water_content	nosz_Cluster24	0	0	#VALUE!	#VALUE!	#VALUE!	NA	Organic
Water_content	nosz_Cluster25	0.0038	0.0024	0.0642	-0.1697	0.6281	0.8719664	Organic
Water_content	nosz_Cluster26	0.0018	0.0006	0.0456	-0.193	0.6845	0.8856156	Organic
Water_content	nosz_Cluster27	0.001	0.0006	0.0642	-0.1697	0.6281	0.8719664	Organic
Water_content	nosz_Cluster28	0.0167	-0.0104	0.1951	-0.0062	0.3806	0.8719664	Organic
Water_content	nosz_Cluster30	0.0114	0.0061	0.0722	-0.1598	0.6067	0.8719664	Organic
Water_content	nosz_Cluster32	0.0079	0.0041	0.066	-0.1674	0.623	0.8719664	Organic
Water_content	nosz_Cluster33	0.0162	0.01	0.0642	-0.1697	0.6281	0.8719664	Organic
Water_content	nosz_Cluster34	0.0353	0.0115	0.0322	-0.2097	0.7336	0.8916615	Organic
Water_content	nosz_Cluster35	0.0019	0.0012	0.0642	-0.1697	0.6281	0.8719664	Organic
Water_content	nosz_Cluster36	0.0096	0.0054	0.0693	-0.1634	0.6143	0.8719664	Organic
Water_content	nosz_Cluster37	0.0019	0.0012	0.0642	-0.1697	0.6281	0.8719664	Organic
Water_content	nosz_Cluster38	0.0087	0.0078	0.4975	0.3718	0.1175	0.4550726	Organic
Water_content	nosz_Cluster39	0.0246	0.0035	0.0062	-0.2423	0.8826	0.9777342	Organic
Water_content	nosz_Cluster40	0.0106	0.0055	0.0726	-0.1592	0.6055	0.8719664	Organic
Water_content	nosz_Cluster41	0.0064	-0.0006	0.0046	-0.2442	0.8981	0.9777342	Organic
Water_content	nosz_Cluster42	0.0226	0.0071	0.0314	-0.2107	0.7369	0.8916615	Organic

Water_content	nosz_Cluster43	0	0	#VALUE!	#VALUE!	#VALUE!	NA	Organic
Water_content	nosz_Cluster44	0.0027	-0.0027	0.3123	0.1404	0.249	0.6875221	Organic
Water_content	nosz_Cluster45	0.0018	-0.0001	0.0016	-0.248	0.9394	0.9817257	Organic
Water_content	nosz_Cluster46	0.0038	0.0011	0.0264	-0.217	0.7585	0.8987941	Organic
Water_content	nosz_Cluster47	0	0	#VALUE!	#VALUE!	#VALUE!	NA	Organic
Water_content	nosz_Cluster48	0.0009	0.0001	0.0021	-0.2474	0.931	0.9777342	Organic
Water_content	nosz_Cluster49	0.002	0.0023	0.5633	0.4542	0.0856	0.4040371	Organic
Water_content	nosz_Cluster5	0.0057	0.0035	0.0642	-0.1697	0.6281	0.8719664	Organic
Water_content	nosz_Cluster51	0	0	#VALUE!	#VALUE!	#VALUE!	NA	Organic
Water_content	nosz_Cluster53	0	0	#VALUE!	#VALUE!	#VALUE!	NA	Organic
Water_content	nosz_Cluster54	0	0	#VALUE!	#VALUE!	#VALUE!	NA	Organic
Water_content	nosz_Cluster57	0.0572	-0.0277	0.17	-0.0376	0.4167	0.8719664	Organic
Water_content	nosz_Cluster58	0.0019	-0.0011	0.1565	-0.0544	0.4376	0.8719664	Organic
Water_content	nosz_Cluster59	0.001	0.0006	0.0642	-0.1697	0.6281	0.8719664	Organic
Water_content	nosz_Cluster6	0.0027	0.0012	0.0724	-0.1594	0.606	0.8719664	Organic
Water_content	nosz_Cluster60	0.0027	0.0022	0.55	0.4374	0.0915	0.4084099	Organic
Water_content	nosz_Cluster61	0.0179	-0.0083	0.3417	0.1771	0.2231	0.6517478	Organic
Water_content	nosz_Cluster62	0.0212	-0.0092	0.1592	-0.051	0.4333	0.8719664	Organic
Water_content	nosz_Cluster64	0	0	#VALUE!	#VALUE!	#VALUE!	NA	Organic
Water_content	nosz_Cluster65	0.0019	0.0012	0.0642	-0.1697	0.6281	0.8719664	Organic
Water_content	nosz_Cluster66	0.0065	-0.0084	0.699	0.6237	0.0381	0.3805486	Organic
Water_content	nosz_Cluster67	0.0153	-0.006	0.1704	-0.037	0.416	0.8719664	Organic
Water_content	nosz_Cluster69	0.0013	-1.00E-04	0.0006	-0.2493	0.9648	0.981822	Organic
Water_content	nosz_Cluster70	0.0037	0.0001	0.0002	-0.2497	0.9774	0.9830342	Organic
Water_content	nosz_Cluster71	0.0038	0.0044	0.5475	0.4344	0.0926	0.4084099	Organic
Water_content	nosz_Cluster72	0.012	-0.0016	0.019	-0.2263	0.7948	0.9221226	Organic
Water_content	nosz_Cluster73	0.0029	0.0009	0.0318	-0.2102	0.7352	0.8916615	Organic
Water_content	nosz_Cluster74	0.0017	-0.0008	0.0971	-0.1286	0.5476	0.8719664	Organic
Water_content	nosz_Cluster75	0.0085	-0.0026	0.0664	-0.1671	0.6221	0.8719664	Organic
Water_content	nosz_Cluster77	0	0	#VALUE!	#VALUE!	#VALUE!	NA	Organic
Water_content	nosz_Cluster78	0.002	-0.0005	0.0289	-0.2139	0.7474	0.8944593	Organic
Water_content	nosz_Cluster79	0.0165	-0.0001	1.00E-04	-0.2499	0.9877	0.9896103	Organic
Water_content	nosz_Cluster8	0.0082	0.0008	0.0038	-0.2453	0.9082	0.9777342	Organic
Water_content	nosz_Cluster80	0.006	0.0034	0.0591	-0.1761	0.6424	0.8719664	Organic
Water_content	nosz_Cluster81	0.0028	-0.0006	0.0136	-0.233	0.8257	0.9388201	Organic
Water_content	nosz_Cluster82	0	0	#VALUE!	#VALUE!	#VALUE!	NA	Organic
Water_content	nosz_Cluster83	0.0015	-0.0002	0.0066	-0.2418	0.8787	0.9777342	Organic
Water_content	nosz_Cluster85	0.001	0.0006	0.0642	-0.1697	0.6281	0.8719664	Organic
Water_content	nosz_Cluster86	0	0	#VALUE!	#VALUE!	#VALUE!	NA	Organic
Water_content	nosz_Cluster87	0.002	0.0007	0.0537	-0.1828	0.6585	0.8719664	Organic
Water_content	nosz_Cluster88	0.0148	-0.0047	0.1694	-0.0382	0.4175	0.8719664	Organic
Water_content	nosz_Cluster89	0.0114	-0.0133	0.5082	0.3853	0.1118	0.4429717	Organic
Water_content	nosz_Cluster9	0.0019	0.0007	0.0599	-0.1751	0.6402	0.8719664	Organic
Water_content	nosz_Cluster90	0.0056	-0.0016	0.0558	-0.1803	0.6523	0.8719664	Organic
Water_content	nosz_Cluster91	0.0082	0.0039	0.0623	-0.1721	0.6334	0.8719664	Organic
Water_content	nosz_Cluster93	0.0157	-0.0025	0.0543	-0.1822	0.6569	0.8719664	Organic
Water_content	nosz_Cluster95	0.001	0.0001	0.0021	-0.2474	0.931	0.9777342	Organic
Water_content	nosz_Cluster96	0.0072	0.0053	0.3972	0.2465	0.1798	0.5770643	Organic
Water_content	nosz_Cluster97	0	0	#VALUE!	#VALUE!	#VALUE!	NA	Organic
Water_content	nosz_Cluster98	0	0	#VALUE!	#VALUE!	#VALUE!	NA	Organic
Water_content	nosz_Cluster99	0.0042	0.0012	0.044	-0.1951	0.6901	0.8890182	Organic
Water_content	nrfa_Cluster10	0.0368	0.0157	0.0389	-0.2014	0.708	0.8916615	Organic
Water_content	nrfa_Cluster100	0.0248	0.0037	0.0173	-0.2283	0.8037	0.9238215	Organic
Water_content	nrfa_Cluster104	0.0308	-0.0213	0.2245	0.0306	0.3425	0.840861	Organic
Water_content	nrfa_Cluster105	0.0067	0.0041	0.0642	-0.1697	0.6281	0.8719664	Organic

Water_content	nrfa_Cluster109	0.1147	-0.0171	0.009	-0.2388	0.8581	0.9651824	Organic
Water_content	nrfa_Cluster116	0.1051	-0.0894	0.4544	0.318	0.142	0.4918744	Organic
Water_content	nrfa_Cluster118	0.0309	0.0025	0.0034	-0.2457	0.9121	0.9777342	Organic
Water_content	nrfa_Cluster125	0.0128	0.0038	0.0365	-0.2043	0.7168	0.8916615	Organic
Water_content	nrfa_Cluster154	0.0212	0.0064	0.0337	-0.2079	0.7278	0.8916615	Organic
Water_content	nrfa_Cluster164	0.0048	0.003	0.0642	-0.1697	0.6281	0.8719664	Organic
Water_content	nrfa_Cluster172	0	0	#VALUE!	#VALUE!	#VALUE!	NA	Organic
Water_content	nrfa_Cluster175	0.0022	0.0021	0.53	0.4125	0.1009	0.422233	Organic
Water_content	nrfa_Cluster20	0.0101	-0.0028	0.0388	-0.2014	0.7082	0.8916615	Organic
Water_content	nrfa_Cluster26	0.0192	-0.0019	0.0051	-0.2437	0.8933	0.9777342	Organic
Water_content	nrfa_Cluster29	0.0019	0.0012	0.0642	-0.1697	0.6281	0.8719664	Organic
Water_content	nrfa_Cluster34	0.01	-0.0071	0.2959	0.1199	0.2645	0.7021797	Organic
Water_content	nrfa_Cluster38	0.0028	-0.0006	0.0136	-0.233	0.8257	0.9388201	Organic
Water_content	nrfa_Cluster61	0.0009	-0.0017	0.5977	0.4971	0.0714	0.3805486	Organic
Water_content	nrfa_Cluster62	0.0112	-0.0004	0.0007	-0.2491	0.9607	0.981822	Organic
Water_content	nrfa_Cluster66	0.058	-0.0191	0.0502	-0.1872	0.6695	0.8721895	Organic
Water_content	nrfa_Cluster69	0.0017	1.00E-04	0.0012	-0.2485	0.9489	0.9817257	Organic
Water_content	nrfa_Cluster71	0.0139	0.0067	0.0505	-0.1869	0.6687	0.8721895	Organic
Water_content	nrfa_Cluster72	0.014	-0.0007	0.0029	-0.2464	0.9198	0.9777342	Organic
Water_content	nrfa_Cluster73	0.0029	0.0018	0.0642	-0.1697	0.6281	0.8719664	Organic
Water_content	nrfa_Cluster74	0.0428	0.0041	0.0034	-0.2458	0.913	0.9777342	Organic
Water_content	nrfa_Cluster76	0.0339	0.0195	0.0907	-0.1366	0.5618	0.8719664	Organic
Water_content	nrfa_Cluster79	0.0456	0.0283	0.0642	-0.1697	0.6281	0.8719664	Organic
Water_content	nrfa_Cluster87	0.0076	0.0047	0.0642	-0.1697	0.6281	0.8719664	Organic
Water_content	nrfa_Cluster89	0.0008	-0.0005	0.0702	-0.1622	0.6118	0.8719664	Organic
Water_content	nrfa_Cluster92	0.0048	0.003	0.0642	-0.1697	0.6281	0.8719664	Organic
Water_content	nrfa_Cluster93	0.0045	-0.0002	0.0011	-0.2486	0.9495	0.9817257	Organic
Water_content	nrfa_Cluster94	0	0	#VALUE!	#VALUE!	#VALUE!	NA	Organic
Water_content	nrfa_Cluster97	0.0028	1.00E-04	0.0005	-0.2494	0.9666	0.981822	Organic
Water_content	nrfa_Cluster99	0.0029	0.0013	0.0722	-0.1598	0.6067	0.8719664	Organic
Bulk_density	amoA_A_Cluster26	0.0009	0.001	0.2138	0.0172	0.3559	0.7561219	Organic
Bulk_density	amoA_A_Cluster45	0	0	#VALUE!	#VALUE!	#VALUE!	NA	Organic
Bulk_density	amoA_B_Cluster0	0.0665	0.0172	0.1769	-0.0288	0.4062	0.7654958	Organic
Bulk_density	amoA_B_Cluster1	0.0089	0.0097	0.2768	0.096	0.2836	0.7561219	Organic
Bulk_density	amoA_B_Cluster10	0.013	0.0066	0.0931	-0.1336	0.5564	0.8106192	Organic
Bulk_density	amoA_B_Cluster11	0.0103	0.0076	0.188	-0.015	0.3903	0.7561219	Organic
Bulk_density	amoA_B_Cluster12	0.0501	0.0118	0.0845	-0.1444	0.5763	0.8164993	Organic
Bulk_density	amoA_B_Cluster13	0.2409	0.179	0.2193	0.0241	0.3489	0.7561219	Organic
Bulk_density	amoA_B_Cluster14	0.0117	0.0134	0.2433	0.0541	0.3201	0.7561219	Organic
Bulk_density	amoA_B_Cluster15	0.0082	0.0074	0.2354	0.0443	0.3293	0.7561219	Organic
Bulk_density	amoA_B_Cluster2	0.0666	0.0639	0.273	0.0913	0.2875	0.7561219	Organic
Bulk_density	amoA_B_Cluster20	0.0007	0.0008	0.2138	0.0172	0.3559	0.7561219	Organic
Bulk_density	amoA_B_Cluster23	0.0211	0.0093	0.1582	-0.0523	0.4349	0.7715644	Organic
Bulk_density	amoA_B_Cluster3	0.0028	0.001	0.0432	-0.196	0.6926	0.866022	Organic
Bulk_density	amoA_B_Cluster4	0.0017	0.0019	0.2138	0.0172	0.3559	0.7561219	Organic
Bulk_density	amoA_B_Cluster5	0.0667	0.0267	0.1911	-0.0111	0.386	0.7561219	Organic
Bulk_density	amoA_B_Cluster6	0.0116	0.0053	0.2461	0.0577	0.3169	0.7561219	Organic
Bulk_density	amoA_B_Cluster7	0.0047	-0.0018	0.1065	-0.1169	0.5279	0.7954793	Organic
Bulk_density	amoA_B_Cluster8	0.0054	0.0043	0.3455	0.1819	0.2198	0.7561219	Organic
Bulk_density	amoA_B_Cluster9	0.019	0.0176	0.2902	0.1128	0.2701	0.7561219	Organic
Bulk_density	nifh_Cluster0	0.0368	-0.0082	0.478	0.3475	0.1282	0.7561219	Organic
Bulk_density	nifh_Cluster10	0.0093	-0.0062	0.1154	-0.1057	0.51	0.7954793	Organic
Bulk_density	nifh_Cluster100	0.0019	-0.002	0.1885	-0.0143	0.3896	0.7561219	Organic
Bulk_density	nifh_Cluster103	0.0259	0.0123	0.2908	0.1135	0.2695	0.7561219	Organic
Bulk_density	nifh_Cluster1034	0.0074	0.0033	0.2246	0.0308	0.3423	0.7561219	Organic

Bulk_density	nifh_Cluster105	0.0047	-0.001	0.0641	-0.1698	0.6283	0.8460558	Organic
Bulk_density	nifh_Cluster108	0.0098	0.0031	0.0706	-0.1617	0.6108	0.8354169	Organic
Bulk_density	nifh_Cluster1099	0.1117	-0.0325	0.398	0.2475	0.1792	0.7561219	Organic
Bulk_density	nifh_Cluster11	0.001	-0.001	0.1885	-0.0143	0.3896	0.7561219	Organic
Bulk_density	nifh_Cluster1112	0.0008	0.0011	0.3238	0.1547	0.2386	0.7561219	Organic
Bulk_density	nifh_Cluster112	0.0145	0.0018	0.0524	-0.1845	0.6626	0.866022	Organic
Bulk_density	nifh_Cluster113	0.021	-0.0138	0.6838	0.6047	0.0423	0.7561219	Organic
Bulk_density	nifh_Cluster114	0.0403	0.0067	0.2828	0.1035	0.2775	0.7561219	Organic
Bulk_density	nifh_Cluster1141	0.0138	0.0075	0.173	-0.0337	0.4121	0.7677153	Organic
Bulk_density	nifh_Cluster115	0.0088	-0.0065	0.7526	0.6907	0.0252	0.7561219	Organic
Bulk_density	nifh_Cluster116	0.0503	0.0044	0.012	-0.2351	0.8367	0.9280387	Organic
Bulk_density	nifh_Cluster1163	0.0028	0.0018	0.2981	0.1226	0.2624	0.7561219	Organic
Bulk_density	nifh_Cluster1179	0.0016	-1.00E-04	0.0005	-0.2493	0.9651	0.9915282	Organic
Bulk_density	nifh_Cluster1182	0.0191	-0.0017	0.0486	-0.1892	0.6746	0.866022	Organic
Bulk_density	nifh_Cluster1183	0.0119	-0.0008	0.0147	-0.2317	0.8192	0.9254138	Organic
Bulk_density	nifh_Cluster1197	0.0113	-0.0016	0.0324	-0.2095	0.733	0.8853241	Organic
Bulk_density	nifh_Cluster1199	0.0041	0.0028	0.5576	0.447	0.0881	0.7561219	Organic
Bulk_density	nifh_Cluster120	0.0019	-0.002	0.1885	-0.0143	0.3896	0.7561219	Organic
Bulk_density	nifh_Cluster1203	0	0	#VALUE!	#VALUE!	#VALUE!	NA	Organic
Bulk_density	nifh_Cluster1206	0	0	#VALUE!	#VALUE!	#VALUE!	NA	Organic
Bulk_density	nifh_Cluster1207	0.0064	0.0021	0.0401	-0.1999	0.7037	0.866022	Organic
Bulk_density	nifh_Cluster1209	0.1228	-0.0074	0.147	-0.0662	0.453	0.7715644	Organic
Bulk_density	nifh_Cluster121	0.0027	-0.001	0.0417	-0.1979	0.6979	0.866022	Organic
Bulk_density	nifh_Cluster1211	0.0026	-0.0011	0.154	-0.0575	0.4415	0.7715644	Organic
Bulk_density	nifh_Cluster1213	0.0012	-0.0013	0.2002	0.0003	0.3736	0.7561219	Organic
Bulk_density	nifh_Cluster122	0	0	#VALUE!	#VALUE!	#VALUE!	NA	Organic
Bulk_density	nifh_Cluster123	0.0105	-0.0111	0.1885	-0.0143	0.3896	0.7561219	Organic
Bulk_density	nifh_Cluster1236	0.0046	0.0015	0.0423	-0.1971	0.6957	0.866022	Organic
Bulk_density	nifh_Cluster1238	0.0012	-0.0006	0.0794	-0.1507	0.5884	0.8185082	Organic
Bulk_density	nifh_Cluster124	0.0066	-0.0064	0.356	0.195	0.2112	0.7561219	Organic
Bulk_density	nifh_Cluster1261	0.0012	0.001	0.2063	0.0079	0.3655	0.7561219	Organic
Bulk_density	nifh_Cluster1262	0.0028	0.0015	0.1004	-0.1245	0.5406	0.7995575	Organic
Bulk_density	nifh_Cluster1266	0.0148	-0.0105	0.5648	0.456	0.0849	0.7561219	Organic
Bulk_density	nifh_Cluster1267	0.0996	-0.0065	0.0164	-0.2295	0.8087	0.9254138	Organic
Bulk_density	nifh_Cluster127	0.0009	0.001	0.2138	0.0172	0.3559	0.7561219	Organic
Bulk_density	nifh_Cluster1278	0.0346	0.0178	0.1514	-0.0608	0.4458	0.7715644	Organic
Bulk_density	nifh_Cluster128	0.0019	-0.002	0.1885	-0.0143	0.3896	0.7561219	Organic
Bulk_density	nifh_Cluster1289	0.0036	-0.0018	0.077	-0.1538	0.5945	0.8213178	Organic
Bulk_density	nifh_Cluster129	0.0057	-0.0061	0.1885	-0.0143	0.3896	0.7561219	Organic
Bulk_density	nifh_Cluster1292	0.0036	-0.0021	0.2029	0.0037	0.37	0.7561219	Organic
Bulk_density	nifh_Cluster130	0.0029	-0.003	0.1885	-0.0143	0.3896	0.7561219	Organic
Bulk_density	nifh_Cluster1305	0.0047	-0.0051	0.7749	0.7186	0.0206	0.7561219	Organic
Bulk_density	nifh_Cluster1306	0.0036	0.0038	0.4271	0.2839	0.1592	0.7561219	Organic
Bulk_density	nifh_Cluster131	0.001	-0.001	0.1885	-0.0143	0.3896	0.7561219	Organic
Bulk_density	nifh_Cluster1319	0.0009	-0.0009	0.1885	-0.0143	0.3896	0.7561219	Organic
Bulk_density	nifh_Cluster132	0.0064	-0.0068	0.3721	0.2151	0.1985	0.7561219	Organic
Bulk_density	nifh_Cluster1320	0.0017	1.00E-04	0.0005	-0.2494	0.9669	0.9915282	Organic
Bulk_density	nifh_Cluster1323	0.0027	-0.0012	0.1704	-0.037	0.416	0.7677153	Organic
Bulk_density	nifh_Cluster1324	0.0463	-0.0067	0.0474	-0.1908	0.6786	0.866022	Organic
Bulk_density	nifh_Cluster1336	0.0138	-0.0045	0.5796	0.4745	0.0787	0.7561219	Organic
Bulk_density	nifh_Cluster1340	0.0073	0.0045	0.3806	0.2258	0.192	0.7561219	Organic
Bulk_density	nifh_Cluster1345	0.0863	-0.0088	0.0265	-0.2169	0.758	0.8941622	Organic
Bulk_density	nifh_Cluster1370	0.0018	0.00E+00	0.00E+00	-0.25	0.9988	0.9993728	Organic
Bulk_density	nifh_Cluster1375	0.0019	0.002	0.4665	0.3331	0.1348	0.7561219	Organic
Bulk_density	nifh_Cluster139	0.0049	-0.0047	0.5842	0.4803	0.0768	0.7561219	Organic

Bulk_density	nifh_Cluster140	0.0023	0.0009	0.1071	-0.1162	0.5267	0.7954793	Organic
Bulk_density	nifh_Cluster141	0.035	0.0323	0.2419	0.0524	0.3217	0.7561219	Organic
Bulk_density	nifh_Cluster148	0.0009	0.001	0.2138	0.0172	0.3559	0.7561219	Organic
Bulk_density	nifh_Cluster1480	0.0019	-0.0005	0.0312	-0.211	0.7378	0.8870847	Organic
Bulk_density	nifh_Cluster152	0.0593	-0.0014	0.0122	-0.2347	0.8347	0.9280387	Organic
Bulk_density	nifh_Cluster156	0.1158	-0.0166	0.0987	-0.1266	0.5442	0.7995575	Organic
Bulk_density	nifh_Cluster1562	0.1168	-0.0277	0.1765	-0.0294	0.4069	0.7654958	Organic
[truncated: 435,909 more chars]
